# Murine scald models characterize the role of neutrophils and neutrophil extracellular traps in severe burns

**DOI:** 10.3389/fimmu.2023.1113948

**Published:** 2023-02-07

**Authors:** Julia Elrod, Moritz Lenz, Antonia Kiwit, Lina Armbrust, Lavinia Schönfeld, Konrad Reinshagen, Laia Pagerols Raluy, Christoph Mohr, Ceren Saygi, Malik Alawi, Holger Rohde, Martin Herrmann, Michael Boettcher

**Affiliations:** ^1^Department of Pediatric Surgery, University Medical Center Mannheim, Heidelberg University, Mannheim, Germany; ^2^Department of Pediatric Surgery, University Medical Center Hamburg-Eppendorf, Hamburg, Germany; ^3^Bioinformatics Core, University Medical Center Hamburg-Eppendorf, Hamburg, Germany; ^4^Institute of Medical Microbiology, Virology and Hygiene, University Medical Center Hamburg-Eppendorf, Hamburg, Germany; ^5^Department of Medicine 3, Friedrich Alexander University Erlangen-Nuremberg and Universitaetsklinikum Erlangen, Erlangen, Germany; ^6^Deutsches Zentrum Immuntherapie DZI, Friedrich Alexander University Erlangen-Nuremberg and Universitaetsklinikum Erlangen, Erlangen, Germany

**Keywords:** wound healing, burn, scald, extracellular DNA, sepsis, neutrophil extracellular traps (NETs), bacterial translocation

## Abstract

**Introduction:**

Severe burns cause unique pathophysiological alterations especially on the immune system. A murine scald model was optimized as a basis for the understanding of immunological reactions in response to heat induced injury. The understanding of the roles of neutrophil extracellular traps (NETs) and DNases will support the development of new surgical or pharmacological strategies for the therapy of severe burns.

**Methods:**

We studied C57BL/6 mice (n=30) and employed four scalding protocols with varying exposure times to hot water. An additional scald group with a shorter observational time was generated to reduce mortality and study the very early phase of pathophysiology. At 24h or 72h, blood was drawn and tissue (wound, liver, lung, spleen) was analyzed for the presence of NETs, oxidative stress, apoptosis, bacterial translocation, and extracellular matrix re-organization. In addition, we analyzed the transcriptome from lung and liver tissues.

**Results:**

Exposure to hot water for 7s led to significant systemic and local effects and caused considerable late mortality. Therefore, we used an observation time of 24h in this groups. To study later phases of burns (72h) an exposure time of 6s is optimal. Both conditions led to significant disorganization of collagen, increased oxidative stress, NET formation (by immunodetection of H3cit, NE, MPO), apoptosis (cC3) and alterations of the levels of DNase1 and DNase1L3. Transcriptome analysis revealed remarkable alterations in genes involved in acute phase signaling, cell cohesion, extracellular matrix organization, and immune response.

**Conclusion:**

We identified two scald models that allow the analysis of early (24h) or late (72h) severe burn effects, thereby generating reproducible and standardized scald injuries. The study elucidated the important involvement of neutrophil activity and the role of NETs in burns. Extensive transcriptome analysis characterized the acute phase and tissue remodeling pathways involved in the process of healing and may serve as crucial basis for future in-depth studies.

## Introduction

As the number of thermal injuries has been decreasing worldwide for several decades, according to data from the Global Burden of Disease 2017 study, approximately 9 million burns occurred in 2017 (1). A relevant proportion of these arise in low- and middle-income countries (2, 3). Despite improvements in intensive care and surgical management, mortality of severe burns remains high (4). This is partly due to our limited understanding of the disease. The pathophysiology of thermal injury, including the systemic effects on the immune system is complex and depends on several factors, such as the size of the burn, indicated as the total body surface area (TBSA), the depth of the defect, and the extent of the inflammatory response (3, 5, 6). An inflammatory process is triggered immediately after the insult (3). This reaction can be beneficial to healing. On the other hand, it may lead to systemic inflammation which ultimately impairs wound healing (7). Thermal injury-induced immunosuppression is an important feature of the immunological dysfunction in burns. Its understanding is crucial, as approximately 3/4 of all burn related deaths can be attributed to infection (8).

Neutrophils represent the first line of innate immune defense against infectious pathogens. Amongst other effector functions, they fight a variety of microorganisms by the formation of neutrophil extracellular traps (NETs) (9). Importantly, anti-NET therapy, either on a pharmacological or a genetical basis can improve primary and secondary wound healing in mice (10). NETs consist of chromatin, decorated with cytotoxic proteins, such as neutrophil elastase (NE) and myeloperoxidase (MPO) (9).

Despite the development of modern techniques to replace animal models, they unfortunately remain indispensable up to now. They are required to study immunological reactions in response to heat induced injury and to develop new surgical or pharmacological strategies, such as immunomodulation. In the past, several models of heat-induced injury have been developed. Amongst others, the immersion into hot water in specifically constructed harnesses, allows the creation of scald injuries with reproducibly uniform depth and scope (11). Yet, a literature review revealed that authors use varying protocols to achieve third degree burns (12–15). The most common animals used for scald experiments are mice which are cost- and time-efficient (16). Mouse-based models enable answering detailed pathophysiological questions, since many transgenic mouse lines are available. Further species are rats, pigs, or rabbits (11). A review by Abdullahi et al. illustrated that the experimental protocols for full thickness scalds in mice vary regarding exposure time, temperature and TBSA depending on the experimental hypothesis (16). The TBSA ranges from 2.5 (17) to 35% (18), the length of exposure from 6 (19) to 45 s (20), and the water temperature from 54 (17) to 100°C (21–25). The use of a heat-resistant form with a defined area of exposed skin is an established method to induce a sharply defined, reproducible wound.

The aim of this paper is (I) to optimize the scald model in terms of exposure and observational time, (II) to elucidate the role of neutrophils and NETs in burns, (III) to characterize molecular and biochemical aspects in this process, and (IV) to perform extensive transcriptome analyses, as basis for future mechanistic studies in mice.

## Materials and methods

### Animal procedures

This study was performed with approval by the Authority for Justice and Consumer Protection Hamburg (N065/2020) in accordance with the German Animal Welfare Act. 30 eight-week-old male mice (C57BL/6) were included in the experiments. Animals were kept under constant standard conditions. The mice were randomly divided into five groups receiving following treatments:

1) Sham – no thermal injury (n=5)
2) 4 s scald at 98°C (n=5)
3) 6 s scald at 98°C (n=5)
4) 7 s scald at 98°C (n=13)
5) 10 s scald at 98°C (n=2)

Two days prior to intervention, tramadol was added to the drinking water (10 mg/ml) to ensure periinterventional analgesia.

Anesthesia was induced with 5% isoflurane (Baxter, Unterschleissheim, Germany) by facemask. Buprenorphine (0.1 mg/kg bodyweight) was applied for analgesia and 0.5 ml NaCl were applied i.p. for fluid resuscitation. The dorsum was shaved, treated with depilatory cream and 1 ml of NaCl was injected s.c. along the spinal column for protection of the spinal cord. To reduce the discrepancy between murine and human peripheric neutrophil count (26–28), animals were treated with recombinant G-CSF i.p. (250 µg/kg bodyweight – Granulocyte 34, Chugai Pharmaceuticals, Chuo, Japan) at 0 and 48 h. Anesthetized animals were then placed on their back in a plastic, heat-resistant device, with an opening of 2.5 cm x 5 cm, corresponding to 20-25% TBSA, based on the formula by Cheung et al. (29). Immersion in a 98°C hot water bath was performed for 4, 6, 7 or 10 s. Immediately afterwards, the exposed skin was cooled in a water bath (20°C). Sham group was subjected to all procedural steps, except scalding. Afterwards, all mice were closely monitored and kept in heated retainers. Mice were housed individually to avoid interindividual manipulation of the wounds, which were not covered by a dressing. An i.v. or i.p. fluid resuscitation was not performed due to concerns of the ethical committee; however, the mice were allowed to drink and eat *ad libitum* during the entire study period. After 24 h or 72 h, depending on the groups, mice were anaesthetized using isoflurane. Blood was collected *via* cardiac puncture. Finally, cervical dislocation was performed.

### Sample preparation

Tissue samples were harvested under sterile conditions. Samples of lung, liver and burnt skin were punched out (3 mm diameter) and stored on ice in 1 ml phosphate buffered saline (PBS; Gibco, Carlsbad, CA, USA) for microbiological analysis. Samples of liver, spleen and lung were aliquoted, flash frozen in liquid nitrogen and stored at -80°C. Additionally, lung and liver tissue and burnt skin was fixed in 4% paraformaldehyde (Morphisto, Offenbach am Main, Germany) for 24 h for histological evaluation. After embedding in paraffin, the samples were cut into 3 µm thick sections for staining. Blood samples were collected in EDTA tubes and centrifuged (2000 g, 21°C, 10 min.) within no more than one hour after collection. Plasma was stored at -80°C.

### Bacterial translocation

Samples were homogenized in PBS (agitation speed 5500 min^-1^, 3x30 s) using the Precellys Lysing Kit (Bertin Industries, Île-de-France, France). The resulting solution was diluted 1:5 and 1:100 with PBS. 50 µl of each sample were transferred on columbia agar + 5% sheep blood (COS), columbia agar CNA + 5% sheep blood agar plates (Biomerieux, Marcy l’Etoile, France) and pseudomonas isolation agar (BD, Heidelberg, Germany). After 48 hours of incubation at 37°C, the number of colony forming units (CFU) was determined and multiplied by the respective dilution factors. Species were identified using MALDY-TOF-mass spectrometer (Bruker Daltonik MALDI Biotyper, Bruker, Billerica, MA, USA).

### Transcriptome sequencing and statistics

Tissue samples (≥ 30 mg) of lung and liver of sham and 7 s scald (24 h) mice were collected, rinsed with RNase-free water, and flash frozen. RNA isolation and transcriptome analysis were outsourced to a commercial supplier (Beijing Genomics Institute, Shenzhen, China) (30). Briefly, mRNA molecules were purified using oligo(dT)-attached magnetic beads and incubated with fragmentation reagents. Afterward, mRNA fragments were converted into cDNA molecules, and amplified using polymerase chain reaction (PCR). Finally, DNA nanoball sequencing platform (DNBSEQ) was used for library sequencing. Sequence reads were processed with fastp 0.20.1 to remove sequences of sequencing adapters and low quality (Phred quality score < 15) sequences from the 3’-end of the sequence reads (31). Thereafter, reads were aligned to the mouse reference assembly (GRCm39.104) using STAR 2.7.9a (32). Differential expression was assessed with DESeq2 (33) and a gene was considered differentially expressed if the corresponding false discovery rate (FDR) was ≤ 0.1 and the absolute log2-transformed fold change (|log2FC|) was ≥ 1. Deviating data processing was performed for the analysis including the sham group since replicates were not available. Here the read counts were normalized with DESeq2 too, but only the 60% of protein coding genes with the highest sum of normalized counts across samples in the comparison were further investigated. The log2FC was than calculated between sham and each replicate individually and only the value of lowest fold change, and thus the most conservative estimate, was reported. The detection of overrepresented pathways was performed using clusterProfiler 4.05 (34) in combination with the Molecular Signatures Database (MSigDB) (35) and Gene Ontology (36). Top 5 up- and downregulated genes were tabularized with further information, like protein name and cellular function (37). All sequence data presented in this study have been submitted to the European Nucleotide Archive (ENA) and they are publicly available under accession PRJEB57842.

### Glutathione peroxidase assay

GPx activity, a marker for antioxidative capacity (38), was measured in liver and lung tissue samples using a kit (Cayman-Chemical, Ann Arbor, MI, USA). 10 mg of each sample were homogenized in 500 µl lysis buffer using the TissueRuptor (Qiagen, Venlo, The Netherlands).

### Malondialdehyde assay

To examine lipid peroxidation, MDA concentration (nmol/mg) in liver, lung and spleen was measured using the MDA assay kit (Sigma-Aldrich, St. Louis, MO, USA) as described previously (38). 10 mg of each sample were homogenized in 300 µl lysis buffer master mix using the TissueRuptor (Qiagen, Venlo, The Netherlands).

### Lipopolysaccharide ELISA

A mouse LPS ELISA kit (Biomatik, Kitchener, Canada) was performed with liver samples, according to the official protocol.

### Nucleosome ELISA

A cell death detection ELISA kit (Roche, Basel, Switzerland) was used to measure plasma concentration of nucleosome complexes. Statistical analysis was carried out by interpolation of a standard curve using a four-parameter logistic curve fit (4-PL), as described previously (39). For visualization a nondimensional scale was used.

### Deoxyribonuclease 1 ELISA

The concentration of DNase1 (ng/ml), which is involved in degradation of NETs (9), was assessed by means of ELISA (MyBioSource, San Diego, CA, USA).

### Neutrophil elastase ELISA

NE, a marker for neutrophil activation (40), was quantified using the ELANE ELISA kit (Boster Biological Technology, Pleasanton, CA, USA).

### Circulating free DNA assay

CfDNA was quantified on a fluorescence-based assay (39, 41). After serial dilution of a DNA standard, diluted samples (1:20) and standard curve specimens (range 0-2000 ng/ml) were placed on a 96-well microtiter plate in quadruples. Two wells each were incubated with a Sytox Orange solution. The remaining two wells each, were incubated with a dilution buffer (0.1% BSA, 2 mM EDTA in PBS) as a blank value. After 5 min. of incubation, fluorescence measurement (Ex: 544 nm, Em: 570 nm) was carried out.

### Histological staining (H&E, MG)

For hematoxylin and eosin staining (H&E) and Masson Goldner Trichrome staining (MG) standardized staining protocols were used. Samples were analyzed and photographed using light microscopy at 20x and 40x magnification.

### Picro sirius red staining and analysis of collagen composition and orientation

After a deparaffinization and rehydration process, skin tissue sections were stained using the picro sirius red stain kit (AB150681, Abcam, Cambridge, UK). Light microscopy with polarization filters (Olympus BX60 Microscope, Olympus, Shinjuku, Japan) was used to visualize collagen fibers in the connective tissue of the skin. Polarized pictures were taken and exported to ImageJ 1.53e (National Institutes of Health, Bethesda, MD, USA) for further analysis. Analysis was performed using the validated OrientationJ plug-in (Biomedical Imaging Group, École Polytechnique Fédérale de Lausanne, Lausanne, Switzerland) (42). Orientation of collagen fibers was analyzed based on their deviation from a horizontal line (-90° to +90°). The composition of collagen fibers was scored semiquantitatively (0 = very little amount to 5 = maximum) by an independent, blinded investigator by means of polarized pictures.

### Immunohistochemical staining (cC3)

IHC staining for cleaved caspase 3 (cC3) was performed using a fully automated staining machine (Ventana BenchMark XT, Roche, Basel, Switzerland). Sections were placed in cell conditioning solution (Roche, Basel, Switzerland) for one hour. An anti-mouse-cC3-antibody (AF835, R&D Systems, Minneapolis, MN, USA) was applied at a concentration of 0.67 µg/ml. The UltraView universal DAB detection kit (Roche, Basel, Switzerland) was used.

### Immunofluorescence staining (H3cit, MPO)

After deparaffinization and rehydration, an immunofluorescence double staining for myeloperoxidase (MPO) and citrullinated histone 3 (H3cit) was performed to visualize NETs. MaxBlock Autofluorescence Reducing Reagent (Max Vision Biosciences, Bothell, WA, USA) was applied. Next, slides were treated with Dako Target Retrieval Solution Citrate pH6 (Agilent, Santa Clara, CA, USA) for 10 min. at 98°C, followed by 40 min. of cooling. A donkey block solution (BioGenex, Fremont, CA, USA) was used to reduce unspecific signals. A primary recombinant anti-mouse-H3cit-antibody (AB219406, Abcam, Cambridge, UK) and a goat-anti-mouse-MPO-antibody (AF3667, R&D Systems, Minneapolis, MN, USA) were diluted in buffer (DCS, Hamburg, Germany) to a concentration of 5.27 µg/ml and 10 µg/ml, respectively. Slices were incubated for 12 hours at 4°C. A goat-IgG-antibody (AB108C, R&D Systems, Minneapolis, MN, USA) and a recombinant rabbit-IgG-antibody (AB172730, Abcam, Cambridge, UK) served as isotype controls. Next, Cy3 AffiniPure donkey-anti-goat-IgG (705-165-14, Jackson ImmunoResearch, Ely, UK) and Alexa Fluor 647 AffiniPured Donkey-anti-rabbit-IgG (711-605-152, Jackson ImmunoResearch, Ely, UK) were applied for 30 min. DNA was counterstained for 5 min. using a 4’,6-diamidino-2-phenylindole (DAPI) solution (Invitrogen, Grand Island, NY, USA). Slides were mounted with Fluoromount-G (Southern Biotech, Birmingham, AL, USA).

### Immunofluorescence staining (DNase1, DNase1L3)

A separate staining for DNase1 and DNase1L3 was performed using the protocol described above. As primary antibodies a DNase-1-polyclonal-antibody (BS-7651R, Bioss, Woburn, MA, USA) and a DNase-1L3-polyclonal-antibody (BS-7653R, Bioss, Woburn, MA, USA) were used in a concentration of 0.01 µg/ml. Rabbit-IgG (AB37415, Abcam, Cambridge, UK) served as isotype control. As secondary antibody, a donkey-anti-rabbit-IgG Alexa Fluor 647 (AB150075, Abcam, Cambridge, UK) was used.

### Semiquantitative evaluation of immunofluorescence and immunohistochemical staining (H3cit/MPO, DNase1, DNase1L3, cC3)

Immunofluorescence samples were analyzed using a fluorescence microscope (Zeiss Observer Z1, Carl Zeiss Microscopy, Jena, Germany), immunohistochemical staining using a light microscope. Dyes were scored semiquantitatively by an independent, blinded observer.

### Statistics

A pre-power study calculation was performed using G*Power 3.1 (43), deducted from previous trials regarding inflammation and NET formation. Data was analyzed using GraphPad Prism 9.4.1 (GraphPad, San Diego, CA, USA). Unpaired *t*-tests were used for comparisons of two groups. Three groups were compared by ordinary one-way ANOVA with Dunnett’s correction. The level of significance was set at 0.05. Differences of survival curves were analyzed using log-rank (Mantel-Cox) test.

## Results

### Determination of exposure time of scald

**Figure 1:**
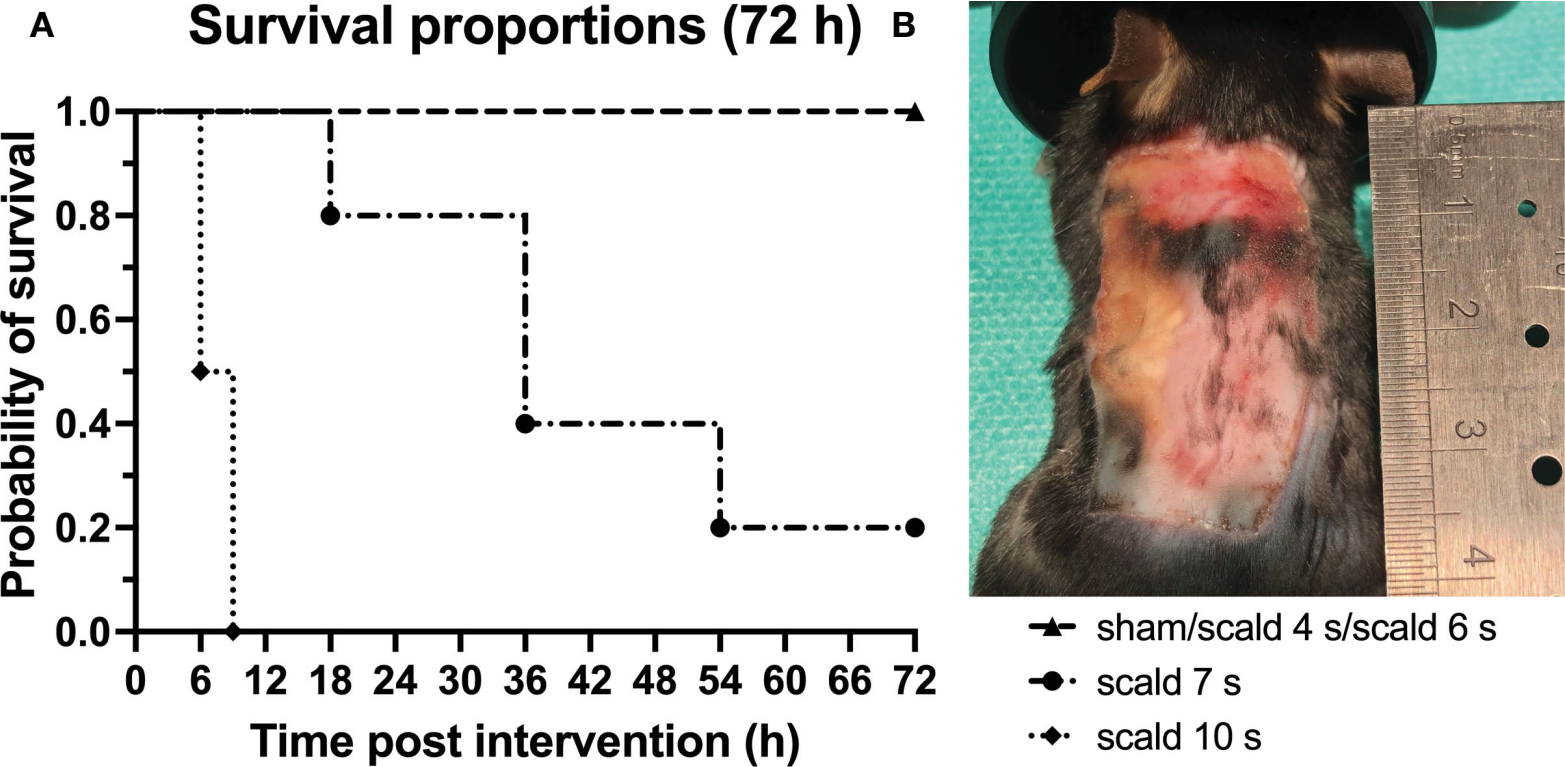
Scalding for 7 s or more significantly decreased the survival rates of mice. **(A)**: The probability of survival for all study groups up to 72 h after interventions is depicted. Log-rank (Mantel-Cox) test confirms a significant difference between survival curves (p = 0.0095). Note the sharp increase in mortality when mice were scalded for 7 s or more. **(B)**: Macroscopic finding of the wound site of the surviving 7 s scald mouse 72 h after intervention. Note the sharply bounded wound area.

Survival: Probability of survival after 72 h as a function of exposure time to hot water is demonstrated in **Figure 1**. 5/5 animals (100%) reached the final endpoint in each of the 4 s, 6 s scald and sham group. In the 7 s scald group only 1/5 mice (20%) reached the final time point of 72 h. In the 10 s scald group 2/2 mice (100%) died within 9 h. According to the 3R principles, this condition was discontinued.

Histopathology: Histological staining (**Figure S1**), revealed an exposure-time-dependent gradual destruction of epidermis and dermis and an infiltration of the dermal white adipose tissue (dWAT) by leucocytes and erythrocytes at 72 h. Full thickness scald, was reliably observed only at 6 s scald group. Macroscopically, a sharply delimited wound field was achieved (**Figure 1B**).

Apoptotic activity: Scald induced upregulation of cC3 was observed in the infiltrate of the dermis and dWAT as shown by IHC (**Figure S2**). Semiquantitative scoring revealed a significant upregulation in the 4 s group (p = 0.0022) and the 6 s group (p = 0.0136), compared to sham.

**Figure 2:**
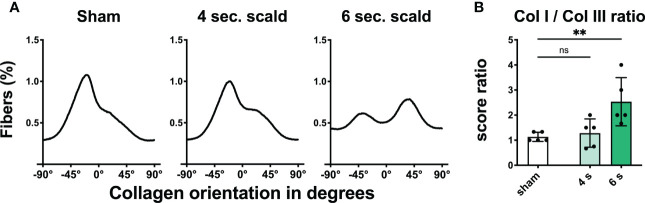
Scalding for 6 s induces reorganization and changes in composition of the dermal collagen matrix. The fraction of fibers **(A)** is displayed as a function of orientation in degrees for sham, 4 s, and 6 s scald. The graphs represent the mean of all individuals of each group. The ratio of the semiquantitative scores **(B)** is illustrated to visualize the changes in collagen composition after thermal injury. Results are provided as mean ± SD. For comparison, one-way AVONA with Dunnett’s correction was performed. The levels of significance: ns, not significant; **p < 0.01.

Dermal collagen distribution and orientation: Shrinking of the epidermis and dermis with densely packed collagen fibers was observed in polarized PSR stain (**Figure 2**, **S3**). The collagen I to collagen III ratio was significantly altered at 6 s scald compared to sham (p = 0.0098); moreover, fibers lost their parallel orientation.

Systemic markers of neutrophil activation: Level of cfDNA in plasma was not significantly altered in the 4 s (p = 0.9783) and 6 s scald model (p = 0.8231) compared to sham mice, whereas DNase1 was significantly elevated in 4 s (p = 0.0001) and 6 s scald (p = 0.0153). NE and nucleosome were significantly elevated in 6 s scald (NE: p = 0.0042; nucleosome: p = 0.0217), but not in 4 s scald (NE: p = 0.8822; nucleosome: p = 0.9767), see **Figure S4**.

Tissue oxidative stress: MDA was significantly elevated in the lung (p = 0.0114) and liver (p = 0.0212) in the 6 s scald model, but not in the spleen, nor at 4 s in any organ. GPx, a maker of oxidative capacity, was reduced in livers of 4 s scald at 72 h (p = 0.0185) only (**Figure S5**).

**Figure 3:**
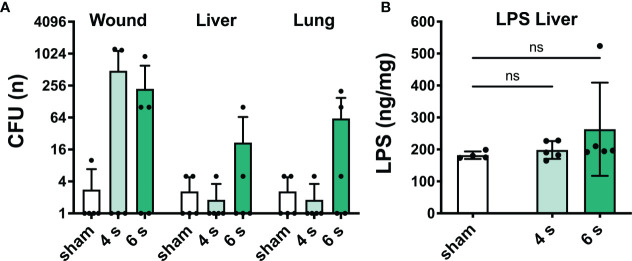
6 s scald causes systemic bacterial translocation. CFU were determined **(A)** in wound, liver, and lung samples. Results are provided as mean ± SD on a log_2_ scale. LPS level in the liver **(B)** was quantified by means of ELISA. Note, translocation of viable bacteria dramatically increased in 6 s scald when compared with 4 s scald. Results are provided as mean ± SD and one-way AVONA with Dunnett’s correction was performed. The level of significance: ns, not significant.

Bacterial translocation and lipopolysaccharide level: At 72 h, bacterial translocation to liver and lung was only observed in 3/5 mice of the 6 s scald group. Wound contamination was found in 2/5 (4 s) and 3/5 (6 s) mice, respectively. LPS level in the liver did not change after scalding (**Figure 3**).

**Figure 4:**
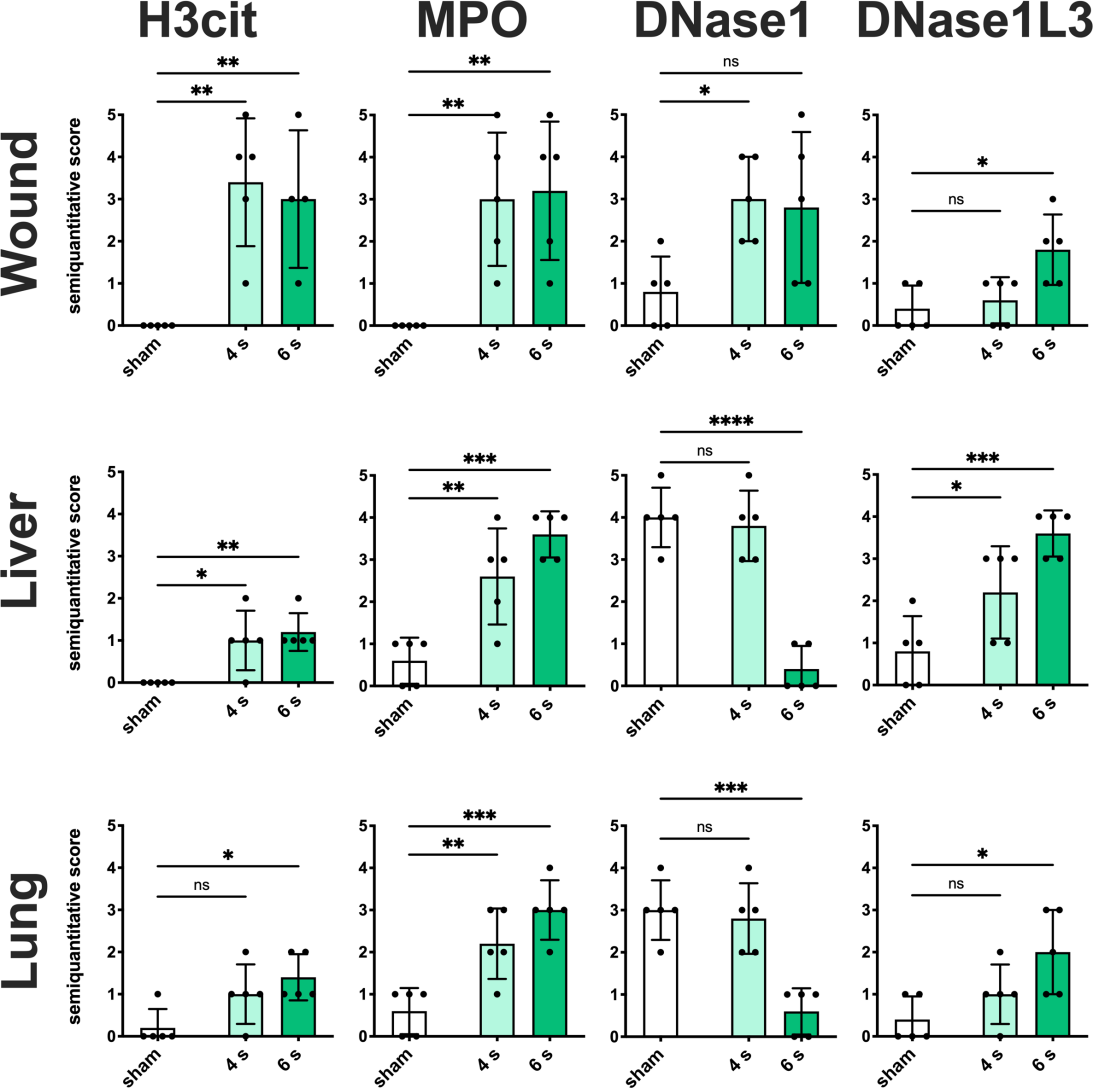
6 s scald leads to a significant NET release, an upregulation of DNase1L3, and downregulation of DNase1. Semiquantitative scores were used to estimate the amount of H3cit, MPO, DNase1, and DNase1L3. Results are provided as mean ± SD and one-way AVONA with Dunnett’s correction was performed. The levels of significance: ns, not significant; *p < 0.05; **p < 0.01; ***p < 0.001; ****p < 0.0001.

Immunofluorescence staining: Elevated expression of H3cit and MPO was observed in the wound (**Figure 4**, **S6A**), especially in the dermis and dWAT, in 4 s (H3cit: p = 0.0024; MPO: p = 0.0068) and 6 s scald (H3cit: p = 0.0081; MPO: p = 0.0044) groups, whereas upregulation of DNase1L3 was only detected in 6 s scald (p = 0.0105) and finally DNase1 was only significantly upregulated in the 4 s scald group (p = 0.0337).

Regarding the liver (**Figure 4**, **S6B**), only the 6 s scald led to a significant upregulation of H3cit (p = 0.0120), whereas MPO was elevated in both intervention times (4 s: p = 0.0071; 6 s: p = 0.0003). DNase1L3 was significantly altered in 6 s scald compared to sham (P = 0.0125) and finally DNase1 expression was reduced after 6 s scald (p = 0.0003).

Regarding lung tissue (**Figure 4**, **S6C**), H3cit was significantly elevated in 4 s (p = 0.0123) and 6 s scald (p = 0.0038), so was MPO (4 s: p = 0.0035; 6 s: p = 0.0001) and DNase1L3 (4 s: p = 0.0431; 6 s: p = 0.0005). The latter was mainly located next to the endothelium of the pulmonal vessels. DNase1 signal was significantly lower in 6 s scald group compared to sham (p < 0.0001), but not in 4 s.

Internal organ damage after 7 s of exposure: Autopsy of mice, which needed to be sacrificed prematurely due to high distress, revealed macroscopically visual injuries of kidney and liver in 2/5 mice and histologic necrotic destruction of the renal and hepatic tissue (**Figure S7–8**).

### In-depth analysis of alterations after 7 s of exposure

Survival: Due to the high late mortality (80%) of 7 s scald mice, a second cohort with an exposure time of 7 s, however with a shorter study observation time of 24 h, was added for further analysis. All mice (8/8) survived the observation time.

Histopathology: Again, a clear destruction and shrinking of the epidermis and dermis and leukocyte infiltration of the dermis and dWAT was observed (**Figure S9**).

**Figure 5:**
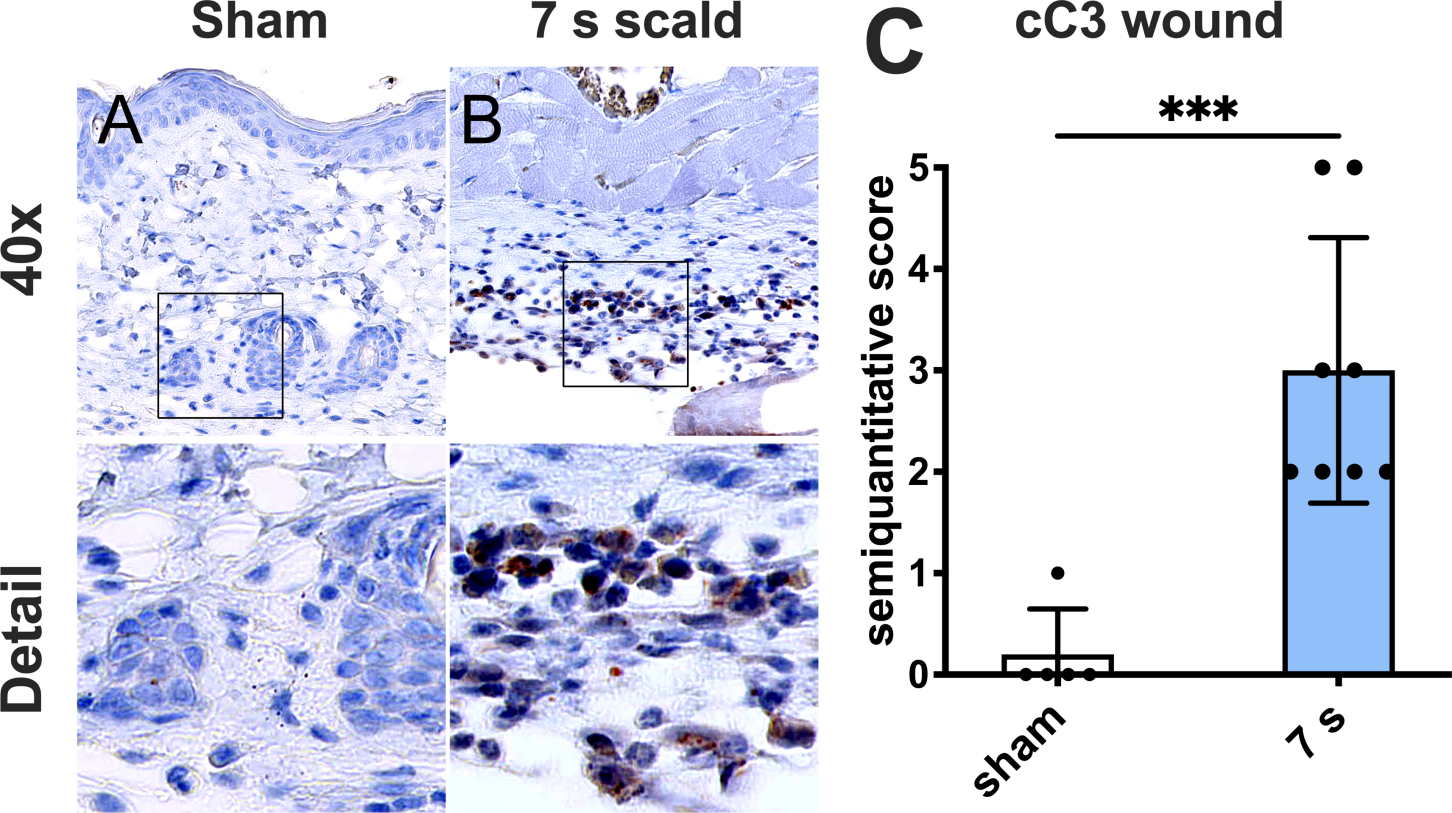
A 7 s scald injury induces dermal apoptosis after 24 h. Representative images of IHC staining for cC3 are shown for sham **(A)** and 7 s scald **(B)**. Semiquantitative score **(C)** revealed a significant upregulation of cC3 in the subcutaneous connective tissue in 7 s scald compared to sham. Results are provided as mean ± SD. For comparison, an unpaired *t*-test was performed. The level of significance: ***p < 0.001.

Apoptotic activity: cC3 was significantly upregulated compared to sham (p = 0.0008). Positive cells were mainly located in the subcutaneous connective tissue (**Figure 5**).

**Figure 6:**
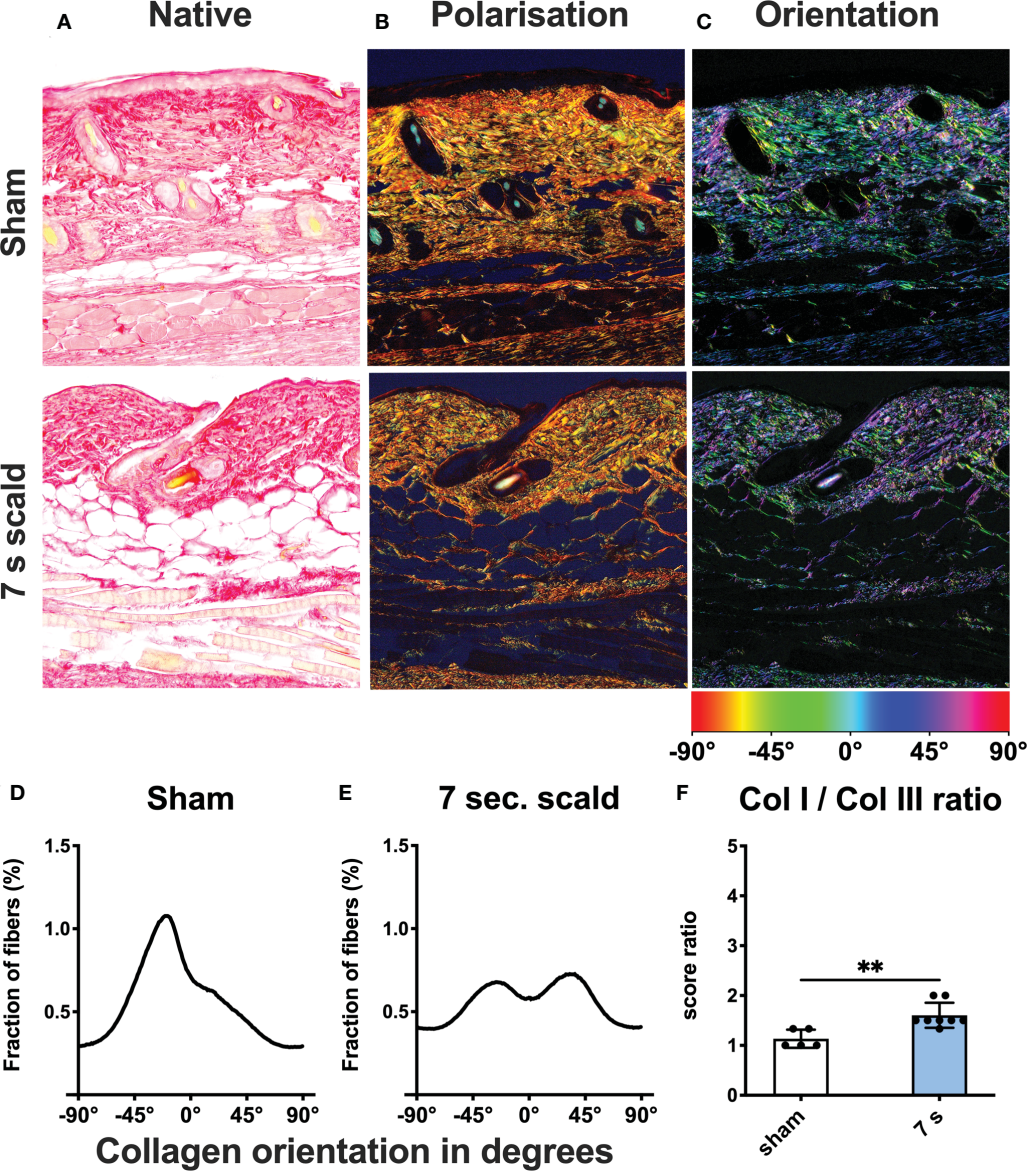
Dermal collagen composition and orientation after 24 h. Representative images of native **(A)** and polarized **(B)** picro sirius red stains of sham and 7 s scald are shown. Green represents collagen III and red represents collagen I fibers. The ratio of the semiquantitative scores **(F)** is illustrated to visualize the changes in collagen composition after thermal injury. Results are provided as mean ± SD. For comparison, an unpaired *t*-test was performed. The level of significance is: **p < 0.01. Distribution of fibers is visualized using a color-based scale **(C)**. The fraction of fibers is displayed as a function of orientation in degree for sham **(D)** and 7 s scald **(E)**. The graph represents the mean of all individuals of each group.

Dermal collagen distribution and orientation: The ratio of collagen I to collagen III changed significantly in 7 s scald group (p = 0.0041) and fibers lost their parallel orientation (**Figure 6**).

**Figure 7:**
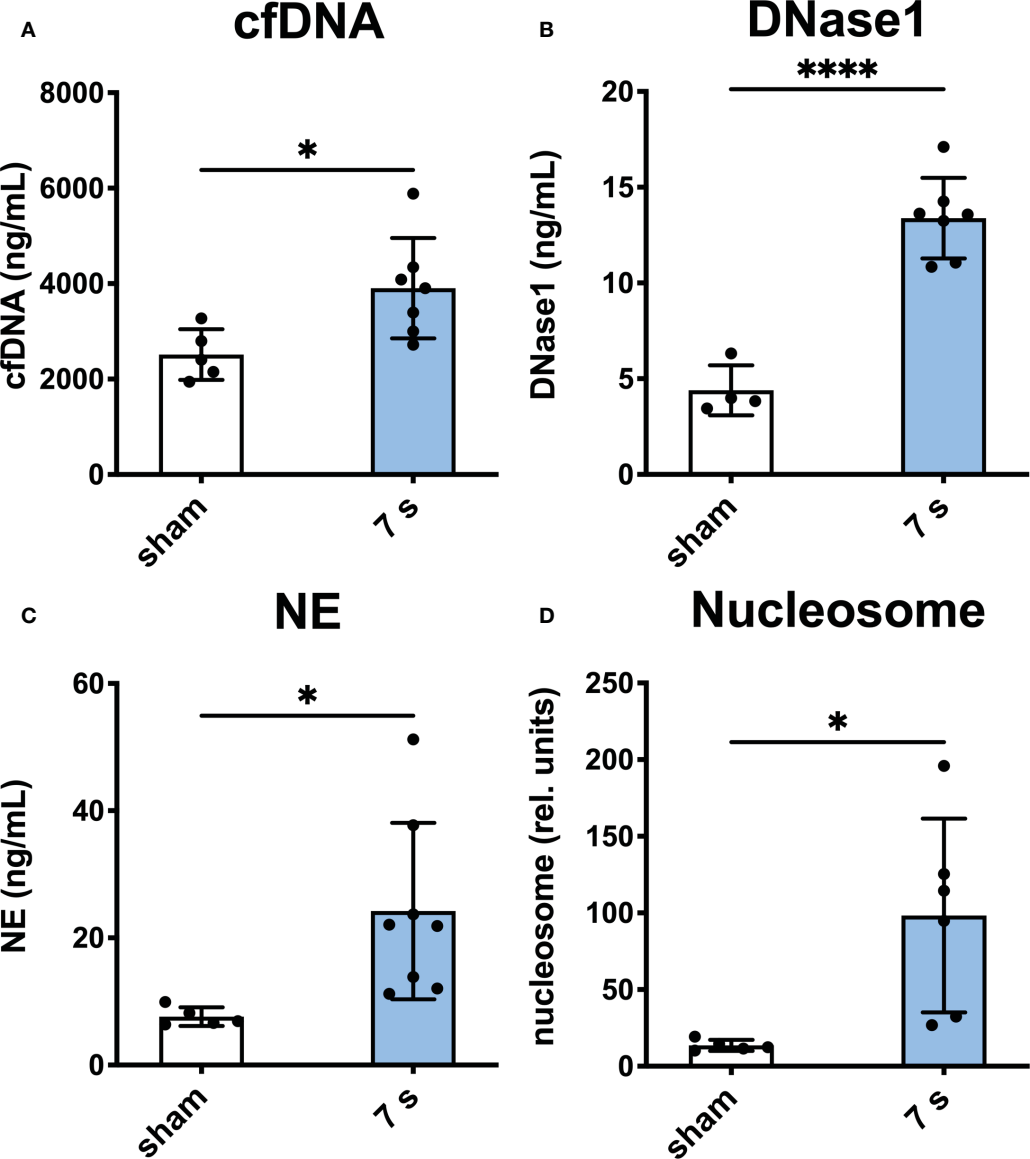
7 s scald leads to an upregulation of markers of neutrophil activation in plasma after 24 h. CfDNA assay **(A)**, DNase1 ELISA **(B)**, NE ELISA **(C)**, and nucleosome ELISA **(D)** are shown. Results are provided as mean ± SD. For comparison, an unpaired t-test was performed. The levels of significance: *p < 0.05; ****p < 0.0001.

Systemic markers of neutrophil activation: Levels of cfDNA (p = 0.0224), DNase1 (p < 0.0001), NE (p = 0.0236), and nucleosome (p = 0.0158) were significantly elevated compared to sham (**Figure 7**).

**Figure 8:**
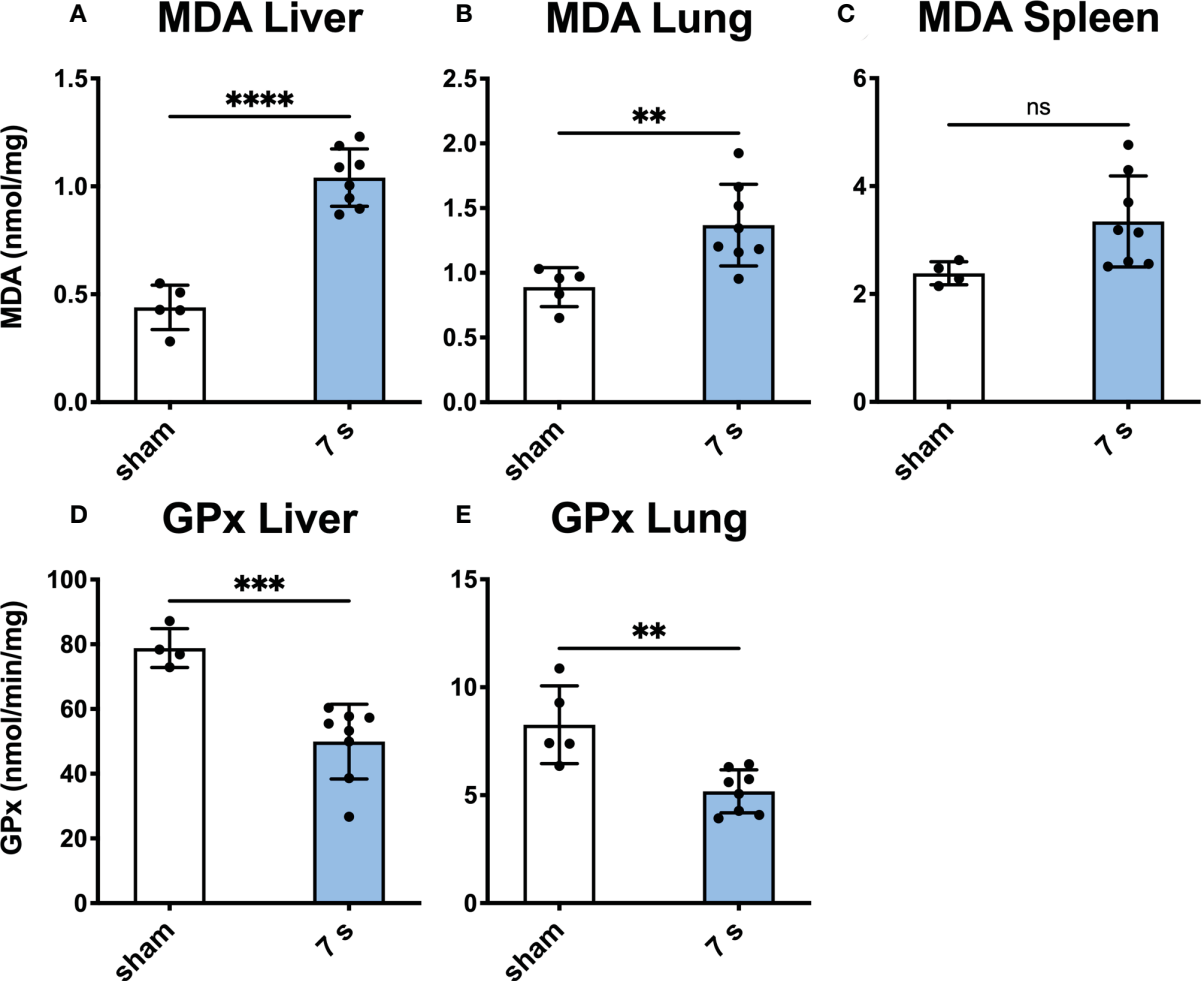
Scalding for 7 s induces oxidative stress in liver and lung after 24 h. MDA, a marker for oxidative stress reaction, was measured in the liver **(A)**, lung **(B)**, and spleen **(C)**. To measure changes in oxidative capacity, GPx was assayed in the liver **(D)** and lung **(E)**. In liver and lung, 7 s scald induces oxidative stress and reduces the oxidative capacity. Results are provided as mean ± SD. For comparison, an unpaired *t*-test was performed. The levels of significance: ns, not significant; **p < 0.01; ***p < 0.001; ****p < 0.0001.

Tissue oxidative stress: Oxidative stress was significantly elevated in the liver and lung as indicated by the increase in MDA (liver: p < 0.0001; lung: p = 0.0094) and decrease in GPx (liver: p = 0.0009; lung: p = 0.0020). GPx cannot be quantified in the spleen due to the presence of erythrocytes (**Figure 8**).

**Figure 9:**
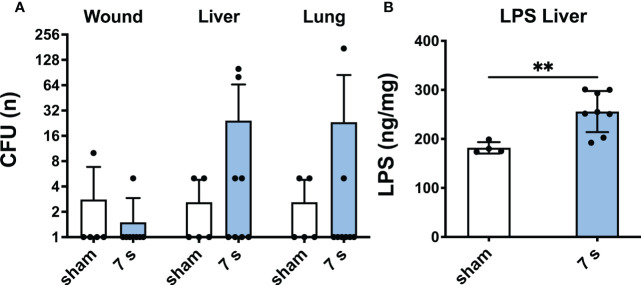
7 s scald induces bacterial translocation to liver and lung and an increase of LPS in the liver after 24 h. CFU **(A)** were examined in wound, liver, and lung samples. Results are provided as mean ± SD on a log_2_ scale. LPS level in the liver **(B)** was quantified by means of ELISA. Results are provided as mean ± SD. For comparison, an unpaired *t*-test was performed. The level of significance: **p < 0.01.

Bacterial translocation and lipopolysaccharide: Relevant translocation of bacteria was observed in the liver in 2/8 and the lung in 1/8 animals, respectively (**Figure 9**). *Enterococcus gallinarum*, a species of murine intestinal flora (44), was identified in two liver samples and one wound sample. All other detected species were considered as contamination. LPS was significantly elevated in the liver (p = 0.0069), but technically not quantifiable in other organs.

**Figure 10:**
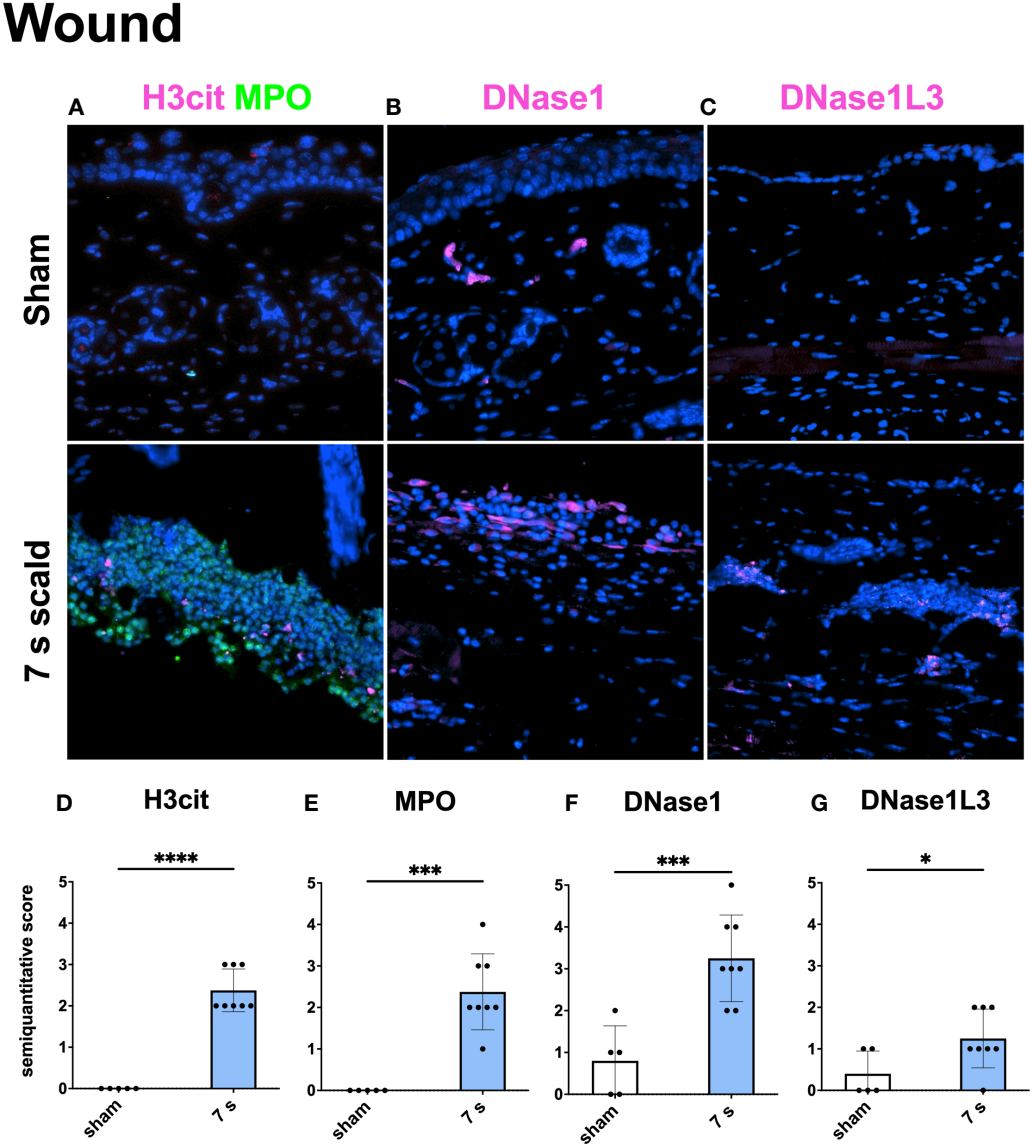
Immunofluorescence staining of the wound reveals a significant NET release and the upregulation of two DNases. Representative merge images of immunofluorescence staining of the wound for H3cit/MPO **(A)**, DNase1 **(B)**, and DNase1L3 **(C)** are displayed. DNA counterstain was performed with DAPI (blue). Magnification level 40x. Semiquantitative scores were used to estimate the amount of H3cit **(D)**, MPO **(E)**, DNase1 **(F)**, and DNase1L3 **(G)**. Results are provided as mean ± SD. For comparison, an unpaired *t*-test was performed. The levels of significance: *p < 0.05; ***p < 0.001; ****p < 0.0001.

**Figure 11:**
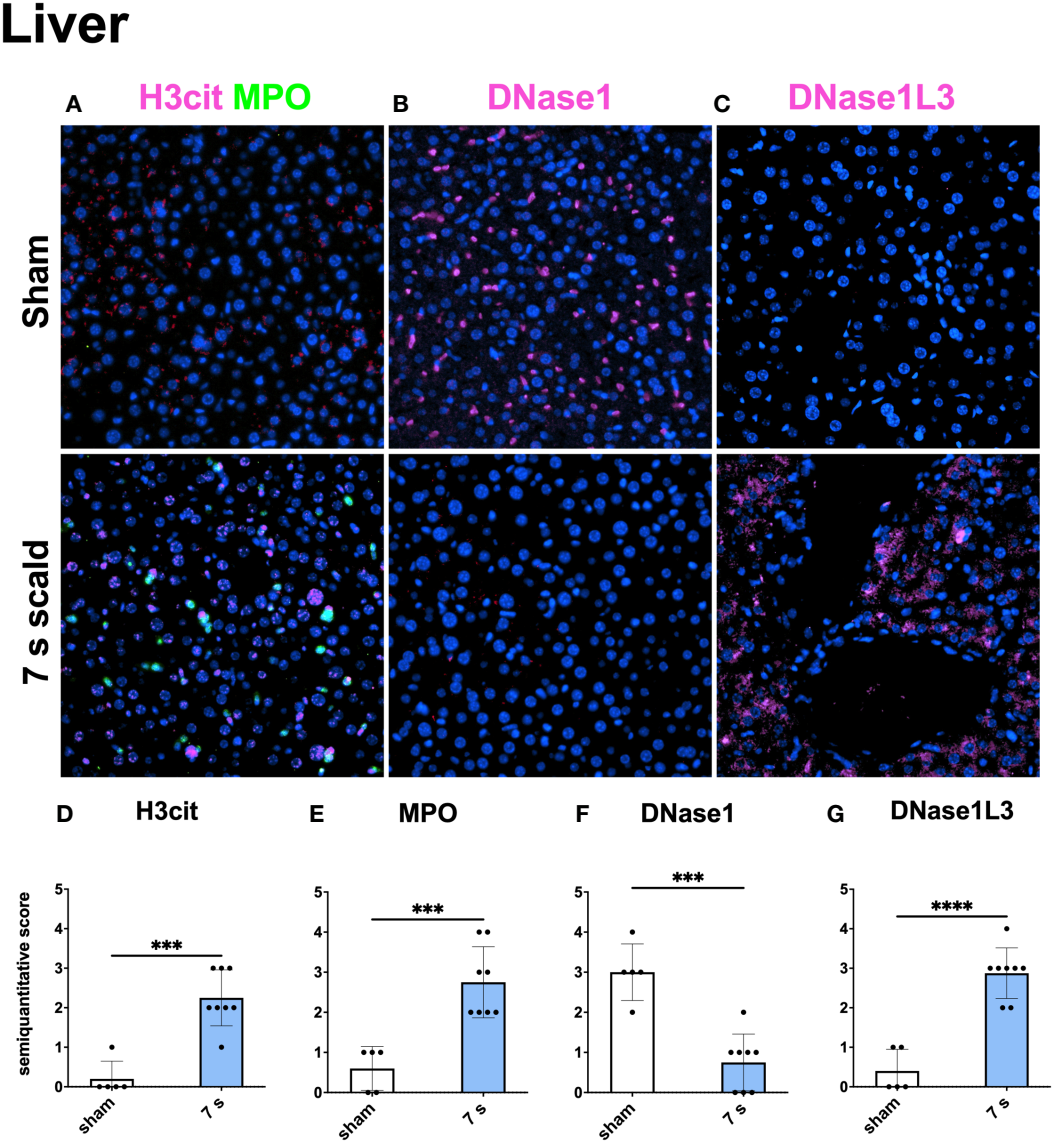
7 s scald leads to an upregulation of NETosis and DNase1L3, and a downregulation of DNase1 in the immunofluorescence staining of the Liver. Representative merge images of immunofluorescence staining of the liver for H3cit/MPO **(A)**, DNase1 **(B)**, and DNase1L3 **(C)** are displayed. DNA counterstain was performed with DAPI (blue). Magnification level 40x. Semiquantitative scores were used to estimate the amount of H3cit **(D)**, MPO **(E)**, DNase1 **(F)**, and DNase1L3 **(G)**. Results are provided as mean ± SD. For comparison, an unpaired *t*-test was performed. The levels of significance: ***p < 0.001; ****p < 0.0001.

**Figure 12:**
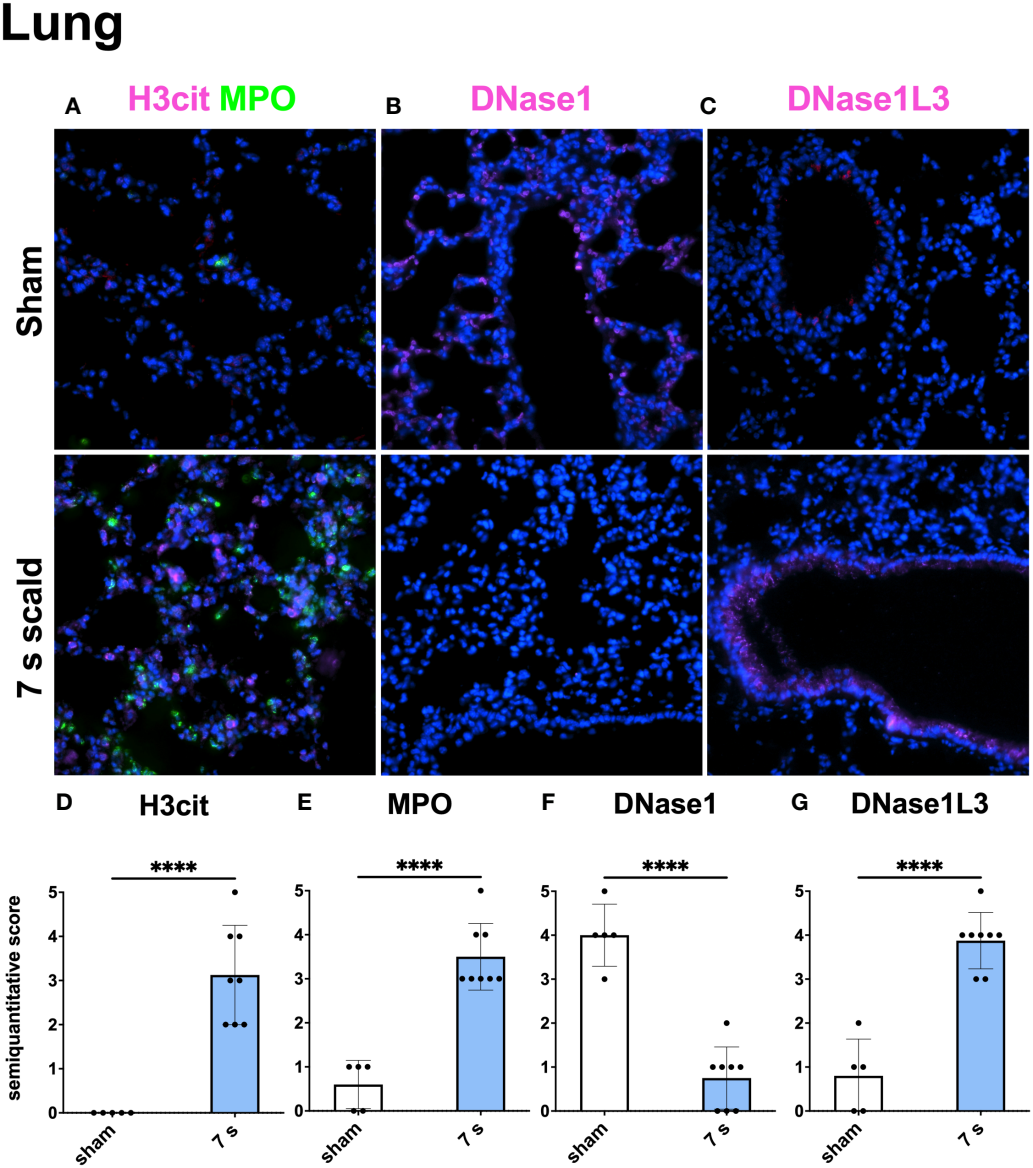
The immunofluorescence staining of the lung shows a massive NET formation and a down- or upregulation of DNase1 and DNase1L3, respectively. Representative merge images of immunofluorescence staining of the lung for H3cit/MPO **(A)**, DNase1 **(B)**, and DNase1L3 **(C)** are displayed. DNA counterstain was performed with DAPI (blue). Magnification level 40x. Note the inverse reaction of DNase1 and DNase1L3. Semiquantitative scores were used to estimate the amount of H3cit **(D)**, MPO **(E)**, DNase1 **(F)**, and DNase1L3 **(G)**. Results are provided as mean ± SD. For comparison, an unpaired *t*-test was performed. The level of significance: ****p < 0.0001.

Immunofluorescence staining: H3cit (p < 0.0001) and MPO (p = 0.0001) were significantly upregulated in wounds (**Figure 10**). DNase1L3 was upregulated (p = 0.0435), mainly located in the dermis and dWAT. DNase1 was significantly elevated (p = 0.0010) and located mainly in the dermal immune infiltrate. In the liver and lung (**Figures 11**, **12**), upregulation was found for H3cit (liver: p = 0.0001; lung: p < 0.0001), MPO (liver: p = 0.0005; lung: p < 0.0001), and DNase1L3 (liver: p < 0.0001; lung: p < 0.0001). In contrast, DNase1 was significantly downregulated in the liver (p = 0.0002) and lung (p < 0.0001).

Transcriptome sequencing: In liver samples, mRNA molecules coding for 8755 different proteins were investigated in the scald (7 s) and the control group. 1382 (16%) of them were significantly up- or downregulated (|log2FC| ≥ 1). In lung samples, 162/9554 detected genes (2%) were significantly up- or downregulated. Top 5 up- or downregulated genes are displayed for liver (**Table S1**) and lung (**Table S2**). The full table is attached in the **Supplemental Materials (Table S3)**. Subsequently, an overrepresentation analysis was performed to identify GO terms (36) and molecular signature database pathways (MSigDB) (35) overrepresented in the differentially expressed genes. In liver tissue, mainly GO terms for metabolic processes, including lipid metabolic process (GO:0006629), cellular amino acid metabolic process (GO:0006520), and carbohydrate metabolic process (GO:0005975) were altered. Moreover, reduction of reactive oxygen species (ROS) and other cellular stress reactions, like peroxisome (GO:0005777), cell death (GO:0008219), and oxidoreductase activity (GO:0006629), were altered. A total of 80 MSigDB pathways were altered in liver samples, including fat, amino acid, and carbohydrate metabolism. Furthermore, other pathways, such as those involving activation of caspases (M26906), detoxification of ROS (M27244), and cell death signaling (M1983902) indicated relevant tissue damage. In the lung, morphological and tissue architectural changes were found. Furthermore, for example extracellular space (GO:0005615), cell adhesion (GO:0007155), and extracellular matrix (GO:0031012) were found to be significantly altered. For MSigDB, mainly immunity related terms were observed to be altered. For example, chemokine receptor binding (M625), chemokine signaling pathway (M39400), and interleukin-10 signaling (M27605) were upregulated. Full GO terms and MSigDB lists of lung and liver can be found in the **Supplemental Material (Table S4-S7)**.

## Discussion

The effects occurring within the first hours after burn injuries are complex. Wound healing includes overlapping phases. The brief hemostatic phase is followed by inflammatory, proliferative, and finally remodeling phases (45). Unfortunately, animal models are still essential to study these complex pathological conditions, although much energy is being invested in exploring alternative methods. Animal models should be replaced, reduced, and refined whenever possible (3R principle) (46). The basis for a successful experiment is the selection of a suitable model. Large-scale scalding models are considered more reliable than contact burn models (47) and serve as a platform for the characterization of the immunological, microbiological, and pathohistological processes. Nevertheless, mice are not “little humans” and exhibit histological and immunological characteristics that differ from the human organism; this restricts the one-to-one translation of the results (16).

Submersion for 10 s considerably injured livers and kidneys and caused 100% mortality within 9 h. It was not used in further experiments. This contrasts with the experience of previous trials (48, 49). Possible explanations include the use of younger mice, with a thinner layer of subcutaneous fat in our experiments (around 20 g), leading to less isolation from heat. This emphasizes the need for standardization of time, age, and weight of mice in scald injury models. Exposure for 6 s induced systemic responses after 72 h, with a survival rate of 100%. This indicates a low systemic effect when investigating burn induced sepsis. Exposure for 7 s caused delayed mortality between 24 and 72 h. Consequently, we already evaluated the 7 s scald experiments after 24 h. Scalding for 6 s and 7 s damaged the integrity of the dermal collagen fibers. This included the loss of the mainly parallel orientation. Cross-linked collagen fibers provide the structural and mechanical basis of human and murine skin (50). An irreversible denaturation of the native triple helical structure triggers a shrinkage of the dermal skin layer (51, 52). Loss of amino acid bonding randomizes the orientation of the collagen fibers (53). These denaturized collagen fibers reportedly provide the stimulus for fibroblasts that initiates the healing of burns (54). Leukocytes infiltrated the space between dermal white adipose tissue (dWAT) and panniculus carnosus (PC). In this infiltration, enhanced cC3 levels, an early marker for apoptosis (55), are displayed. It rises within the first hours after thermal injury, especially in deeper skin layers. Apoptosis is an active process that requires functional metabolism and the maintenance of intact membranes. Only cells in deeper skin layers, which do not immediately necrotize after the thermal stimulus, are able to initiate apoptosis (56). The depth of the injury increases with the exposure-time. Thus, apoptosis after 7 s scalding occurred in deeper skin layers compared to shorter treatment times.

In addition to local effects, systemic reactions emerged especially 24 h after 7 s scalding. Markers of neutrophil activation and NET formation were significantly elevated in plasma (cfDNA, NE, nucleosome) and in wounds, lungs, and livers (H3cit, MPO). A previous trial using a rat burn model suggested that NET release after thermal injury correlates with the total body surface area (TBSA) (57).

The increase of cfDNA in injured patients reportedly served as an early predictor of sepsis and mortality (58–60). Even in burn patients, an early increase of cfDNA level is associated with death (61). The pro-thrombotic NETs link innate immunity to the hemostatic system (62–66). Burns release intact chromatin, which is extremely thrombogenic (57) and contributes to organ disfunction in burn patients (60, 67). In the current study, DNase1 protein, was upregulated in the plasma of all scald groups. This may be due to the high levels of actin, which is released by injured cells and inhibits the activity of DNase1 (60, 68). Whether it circulates as an active nucleolytic enzyme or as an inactive complex with g-actin is elusive and requires further studies. The enzyme is mainly expressed in the intestinal tract and thus the systemic increase may be explained by an increased permeability of the intestinal tract after severe trauma (69, 70). In contrast, tissue bound DNase1, was significantly reduced in lung and liver samples after thermal injury. From the current experiments it is unclear, whether the tissue bound DNase1 is synthesized locally or implanted from circulation. Yet, NET-induced vascular occlusion and consecutive organ dysfunction favors the hypothesis of locally impaired synthesis. DNase1L3 is a second enzyme able to degrade NETs and other kinds of extracellular chromatin (66). We detected this enzyme in wound, liver, and lung samples after thermal injury. This nuclease is secreted by macrophages into inflamed tissue and balances the immune responses of neutrophils (71).

Why does scalding of the skin lead to damage to the liver and lungs? NETs have been identified as a cause of tissue damage in murine sepsis (72). In our large area scald model, neutrophils may play a similar role as reported for other forms of sepsis. 24 h after a 7 s scald, neutrophil activation, NETs (via H3cit/MPO) and oxidative stress (via MDA) were high in lung and liver. This may be the result of an increased neutrophil sequestration after thermal injury. In our model, activated neutrophils may cause oxidative stress which subsequently induces tissue damage, reduces organ function, and eventually multiple organ failure (73).

The transcriptome of lung tissue confirms this explanation, as gene expressions involved in cell cohesion, extracellular matrix organization, and immune response are altered. Beside upregulation of several acute phase proteins, we observed a remarkable upregulation of metallothionein in the liver. These proteins are crucial for the anti-inflammatory response and the prevention of organ damage after thermal injury in mice (74). In addition, hepatic gene expression changes are compatible with the known hypermetabolic state after thermal injury (75). The early phase is characterized through an impaired energy metabolism, including increased gluconeogenesis and protein turnover which results in a hyperglycemia (76). Similar changes regarding inflammation and metabolism were observed in humans as well (3). However, results concerning the regulation of individual genes cannot be transferred to humans one-to-one: As shown previously, inflammation and host responses to injuries tend to differ significantly between humans and mice (77). This accounts also for genomic responses after burns. As shown by Seok et al., gene changes of murine burns correlate poorly with gene changes in human burns (Pearson correlation R^2^ = 0.08). Interestingly, genomic responses to burns, mechanical trauma, and endotoxemia correlate much more strongly in humans (R^2^ = 0.47-0.91) than in mice (R^2^ = 0.00-0.13) (78). To draw conclusions from mouse findings in case of burns, one must bear in mind that the changes of gene expression over time differs significantly: while the alterations of gene expression in both humans and mice occurs within the first few hours, the recovery time was dramatically longer and lasted up to several months in humans (78). Since NETs are the main targets in this study, we stimulated neutrophil bone marrow release with G-CSF to minimize the differences in the peripheral blood neutrophil counts of murine and human blood; the former has a strong preponderance of lymphocytes (75-90% lymphocytes, 10-25% neutrophils), whereas the latter is rich in neutrophils (50-70% neutrophils, 30-50% lymphocytes). As reported by Mestas et al., there are many other differences in the innate and adaptive immune systems of humans and mice. This applies to the toll receptors, some NK-inhibitory receptor families, components of the T-cell signaling pathway or chemokine (receptor) expression, just to mention a few (27). For these reasons, it is important to keep in mind that immune responses to murine burns might not be similar in humans. Despite the limitation regarding the transferability of results obtained in murine models, the importance of these models in relation to metabolic or immune responses and to various types of traumata, including burns, cannot be denied.

Of note, in this study, no wound dressings were used since the ethics committee did not approve the use of tie-over-dressing. An overview of the literature shows that many murine scald models are conducted without wound dressing, even those analyzing late phases of wound healing (10, 14, 48, 79, 80). Nonetheless, one should keep in mind, that the omission of wound dressings can potentially affect the validity of the presented models in terms of analysis of later healing phases, including scar formation, which might be influenced by wound dressing. As shown in a histological study, the use of a wound dressing can positively influence wound healing parameters such as wound closure time and inflammatory response, possibly by influencing the wounds micromilieu (81).

Patients with large-scale burn injuries often develop bacterial translocation and consecutive systemic infections (82). The acute shift of the blood flow to essential organs after thermal injury causes ischemia in the gastrointestinal tract. This results in an increased permeability of the intestinal mucosa and a transfer of intestinal pathogens into the bloodstream (83). These settle in large organs like lung, liver, brain and in lymph nodes (84). In our experiment, we only observed a moderate bacterial translocation to lung and liver after 6 and 7 s scald in a few mice. Possibly, the 24 h observation time was too short for the detection of reliable bacterial translocation in 7 s scald. Nevertheless, the observed rise of LPS levels in the liver may indicate an incipient bacterial translocation. This assumption is supported by the data of the single survivor of the 7 s 72 h group (not shown). Here, an increased bacterial load (CFU n = 10280) was observed which might explain the high mortality rate in this group. Besides the possibility of endogenous bacterial translocation from the intestine, potential exogenous wound contamination should also be considered, inter alia by lack of a wound dressing. However, in a comparable murine scald model, systemic spread of local wound contamination tended to begin at the earliest after three days (85). In patients with severe thermal injuries, sepsis often occurs after a few days or weeks, despite intensive medical treatment at a high standard (86).

In addition to oxidative stress and bacterial translocation, insufficient blood flow in the capillary bed due to non-canonical micro-thromboembolism and vascular occlusion may serve as a further mechanism that precipitates distant pathologies including multi organ failure in burns. Indeed, in fatal COVID-19-related sepsis vascular occlusions can be detected in most autopsy samples from lung, liver, and kidney (87). Interestingly, enormous difference between mice exposed to hot water for 6 and 7 s regarding mortality rates was observed in this study, which might be explainable by a related mechanism: After surface scalding, organ failure occurs despite the organs not being exposed to high temperatures. This organ damage does not occur immediately. Having this in mind, we argue that the tissue-toxic effect somehow spreads from the skin to the internal organs, most likely *via* a hematogenous route. Candidate culprits are intravascular NETs that have been reported to be able to occlude vessels with various diameters and consequently cause failure of distant organs (87, 88). Perhaps, initiating a sufficient amount of NETs capable of initiating multiple organ damage and death depends on a significant volume of injured subdermal tissue.

This study has several limitations, for instance the limited number of organs included for analysis, the limited number of experimental settings such as water temperature and exposure time, and the limited number of timepoints of evaluation. The relatively small sample sizes were necessary to comply with the specifications for stressful animal experiments. Furthermore, regarding for example transcriptome analysis, it was unfortunately not possible to add animals with other types of traumata for legal reasons; this limits the possibility to understand the specificity of the alterations found in this trial. Further studies based on the results described above are needed to provide mechanistic findings. Moreover, the lack of measurement of blood values which are typically altered in sepsis, such as blood count, coagulation, or liver enzymes, restrict the informative value. The omission of wound dressings could affect the validity of the presented model in terms of analysis of later healing phases, including scar formation, and might favor wound contamination and development of sepsis.

In conclusion, the current study characterized two murine scald models. For trials focusing on the very early phase of burn-induced sepsis and the later stages of burn pathophysiology exposure times of 7 s and 6 s scald are advisable, respectively. The characterization of these two models, which induce a reliable, standardized thermal injury, shows an involvement of neutrophil activity, oxidative stress, bacterial translocation, and vascular occlusions. The models are suitable to standardize thermal injury in mice and enable further investigations. The extensive transcriptome analysis conducted in this study, constitutes the basis for further in-depth analysis of the mechanisms of specific pathways involved in the early phase of burn induced pathologies.

## Data availability statement

The data of the transcriptome analysis presented in the study are deposited in the European Nucleotide Archive repository, accession number PRJEB57842. You can acces them directly via this link: https://www.ebi.ac.uk/ena/browser/view/PRJEB57842.

## Ethics statement

The animal study was reviewed and approved by Authority for Justice and Consumer Protection Hamburg.

## Author contributions

JE: designed study, acquisition, analyzed data, and interpretated the data, drafted the manuscript, and approved the final revision. ML: designed study, acquisition, analyzed data, and interpretated the data, drafted the manuscript, and approved the final revision. AK: acquisition, analyzed and interpreted the data, and approved the final revision. LA: acquisition, analyzed and interpreted the data, and approved the final revision. LS: analyzed data, and approved the final revision. KR: analyzed data, and approved the final revision. LPR: analyzed data, and approved the final revision. CM: analyzed data, interpreted the data, and approved the final revision. CS: analyzed data, and approved the final revision. MA: analyzed data, and approved the final revision. HR: analyzed data, interpreted the data, and approved the final revision. MH: analyzed and interpreted the data, drafted the manuscript, and approved the final revision. MB: designed study, analyzed and interpreted the data, drafted the manuscript, and approved the final revision. All authors contributed to the article and approved the submitted version.
