# Supplementary material for: Murine scald models characterize the role of neutrophils and neutrophil extracellular traps in severe burns
Source: Front Immunol. 2023 Feb 7;14:1113948. doi: 10.3389/fimmu.2023.1113948 (PMC9941538; doi:10.3389/fimmu.2023.1113948)
Supplement: Supplementary file 1 [file DataSheet_1.pdf]

Supplemental material

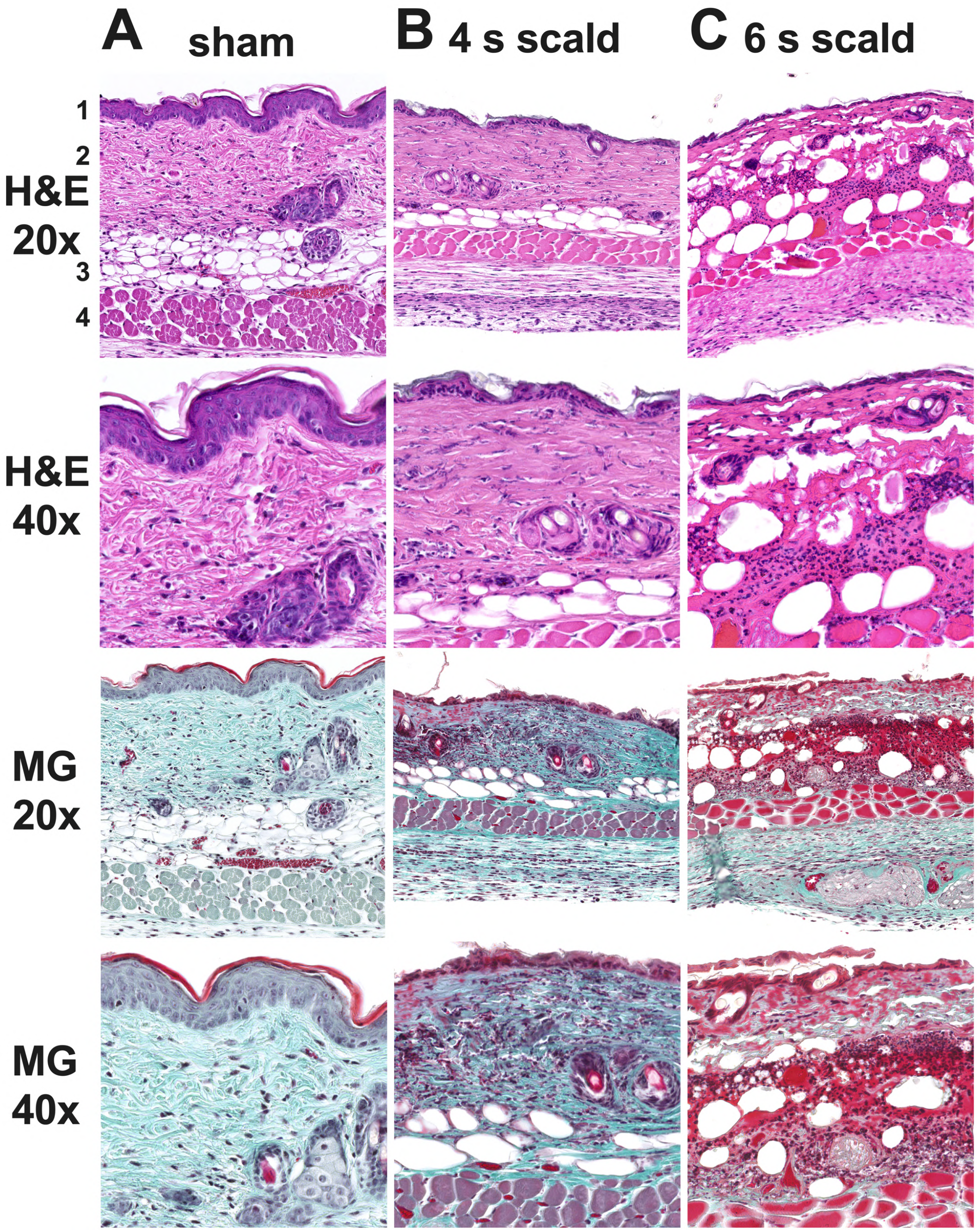

Figure S1: **Depth of the wounds 72 h after intervention.** Representative images of hematoxylin and eosin (HE) and Masson Goldner Trichrome (MG) staining of sham (A) compared to 4 s scald (B) and 6 s scald (C) are depicted. We observed a gradual destruction of the skin layers after 4 s and 6 s scald. For orientation, murine skin layers are indicated exemplary (1: Epidermis, 2: dermis, 3: dermal white adipose tissue (dWAT), 4: panniculus carnosus).

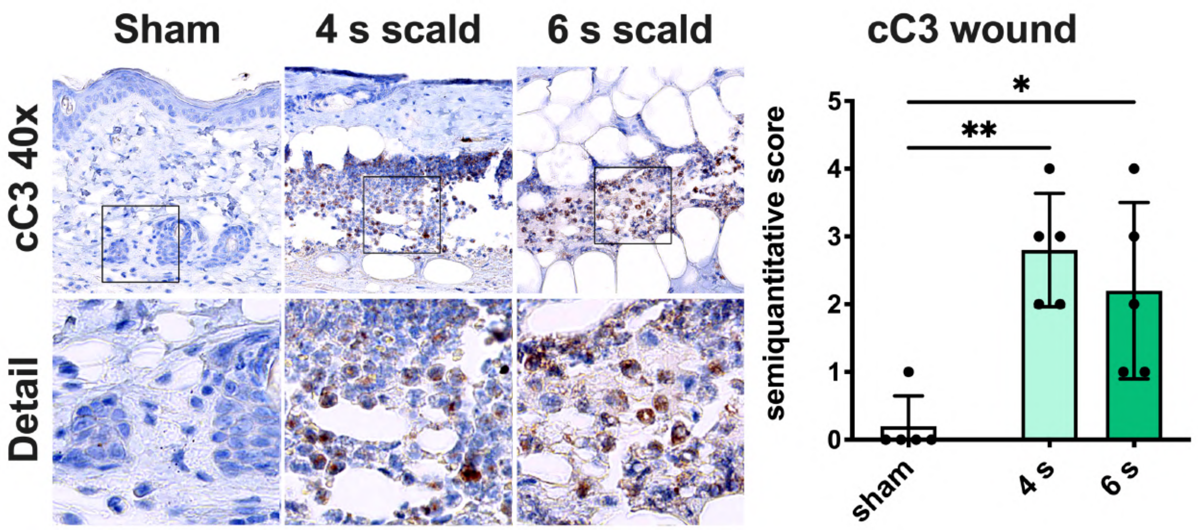

Fig. S2: **Scalding for 4 and 6 s leads to increased dermal apoptosis after 72 h.** Representative images of IHC staining for cleaved caspase 3 (cC3) are shown for sham, 4 s, and 6 s scald. Semiquantitative scoring reveals a significant increase of cC3 in the dermis and the dermal white adipose tissue (dWAT) in 4 s and 6 s scald protocols compared to sham. Results are provided as mean ± SD. For comparison, one-way AVONA with Dunnett's correction was performed. The levels of significance: * $p \leq 0.05$; ** $p < 0.01$.

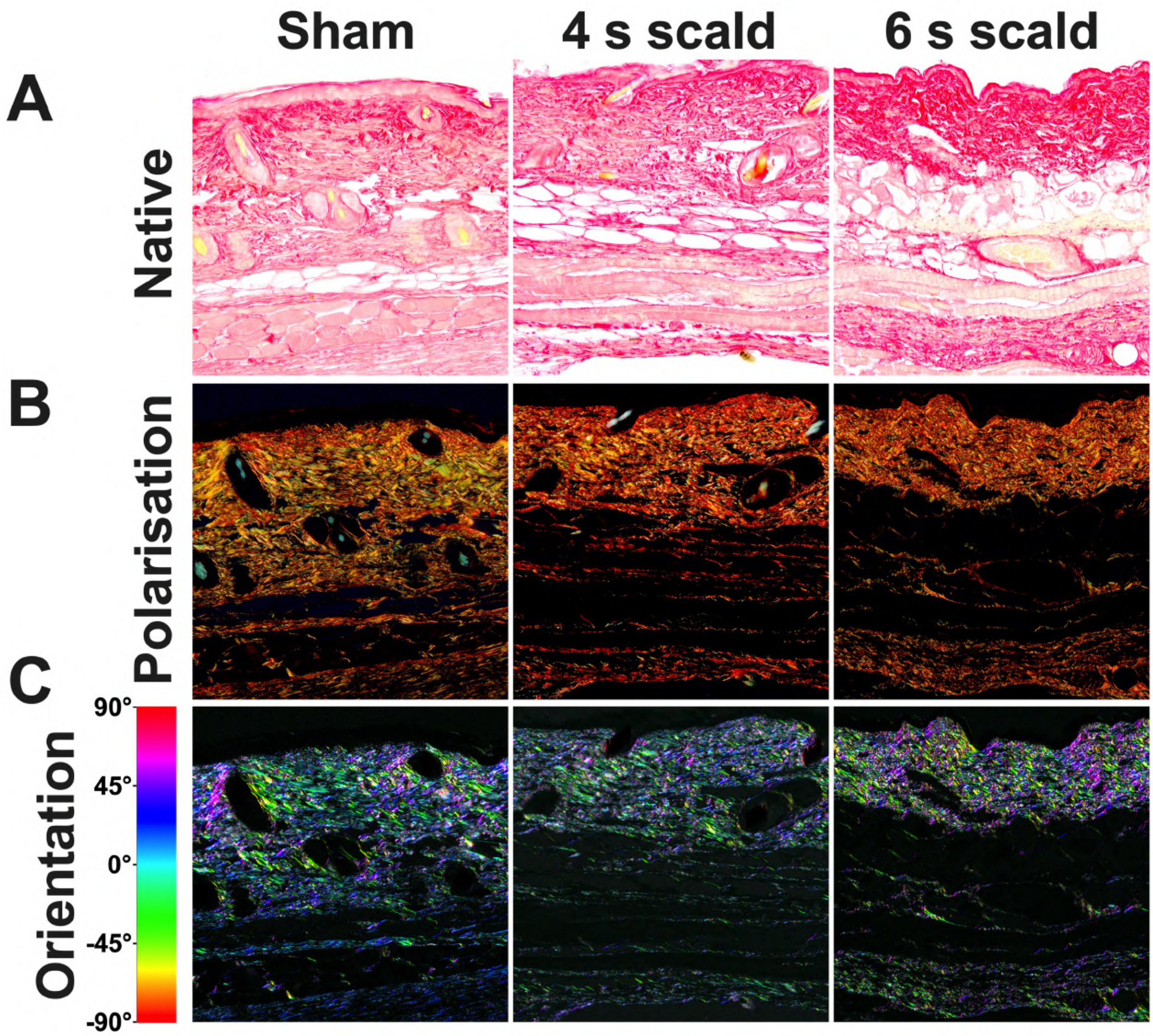

Fig. S3: **Scalding induces shrinking and reorganization of dermal collagen matrix.** Representative images of native (A) and polarized (B) picro sirius red stains of sham, 4 s and 6 s scald are shown. Green represents collagen III and red represents collagen I fibers. Orientation of fibers is visualized using a color-based scale (C). Scalding for 6 s induces a reorganization of the dermal collagen fibers, which lose their mainly parallel formation.

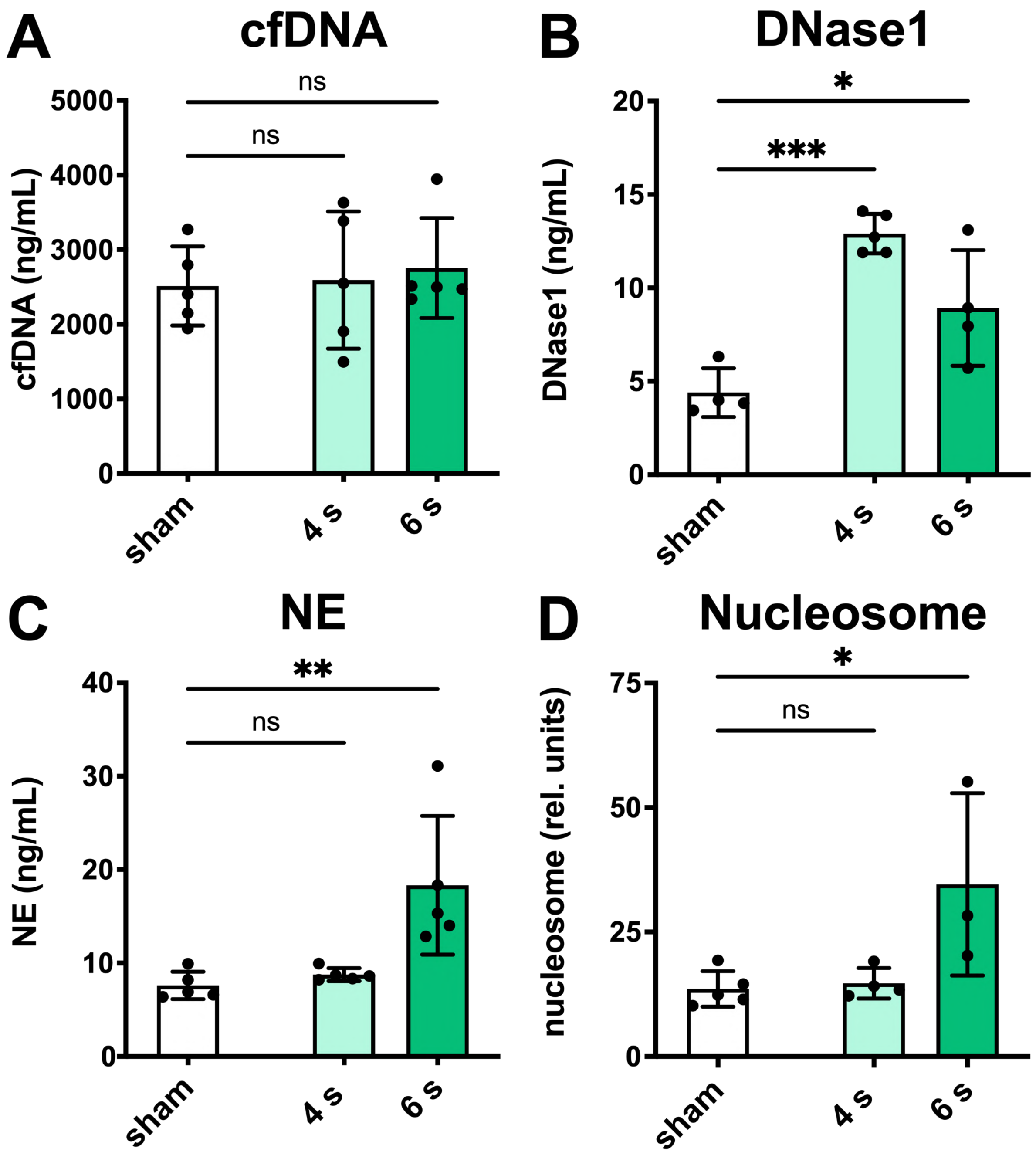

Fig. S4: **Plasma levels of markers of NETosis rise after 6 s scald.** CfDNA assay (A), DNase1 ELISA (B), NE ELISA (C), and nucleosome ELISA (D) are depicted. Only 6 s scald leads to a significant upregulation of NETosis markers in the circulation. Results are provided as mean ± SD. For comparison, one-way AVONA with Dunnett's correction was performed. The levels of significance: ns – not significant; * $p < 0.05$; ** $p < 0.01$; *** $p < 0.001$.

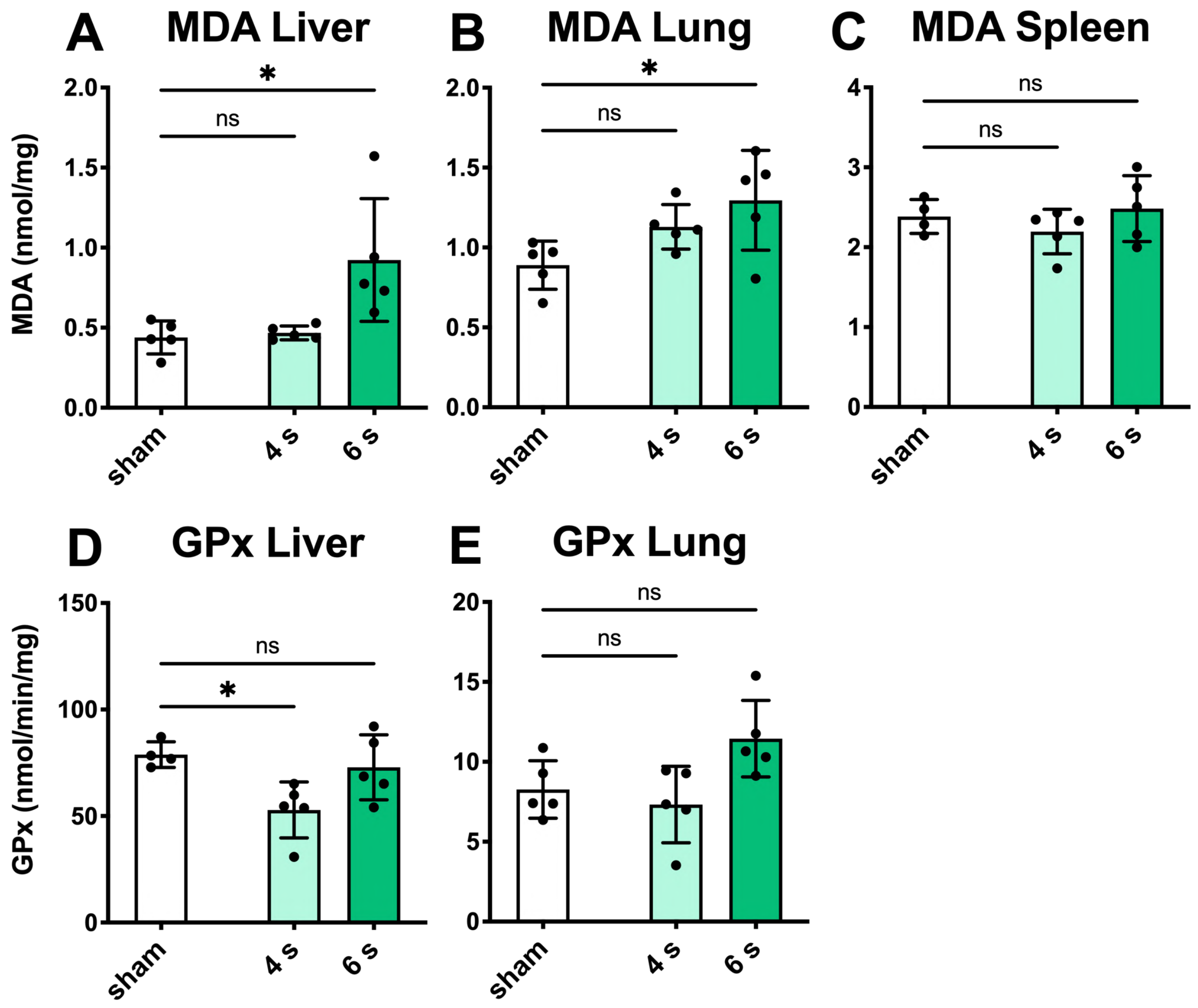

Fig. S5: **6 s scald induces oxidative stress in the liver and lung after 72 h.** MDA, a marker for oxidative stress reaction, was measured in the liver (A), lung (B), and spleen (C). To measure changes in oxidative capacity, GPx was assayed in the liver (D) and lung (E). 6 s but not 4 s scald induces oxidative stress in the liver and lung. Oxidative capacity was not altered. Results are provided as mean ± SD. For comparison, one-way AVONA with Dunnett's correction was performed. The levels of significance: ns – not significant; * $p < 0.05$.

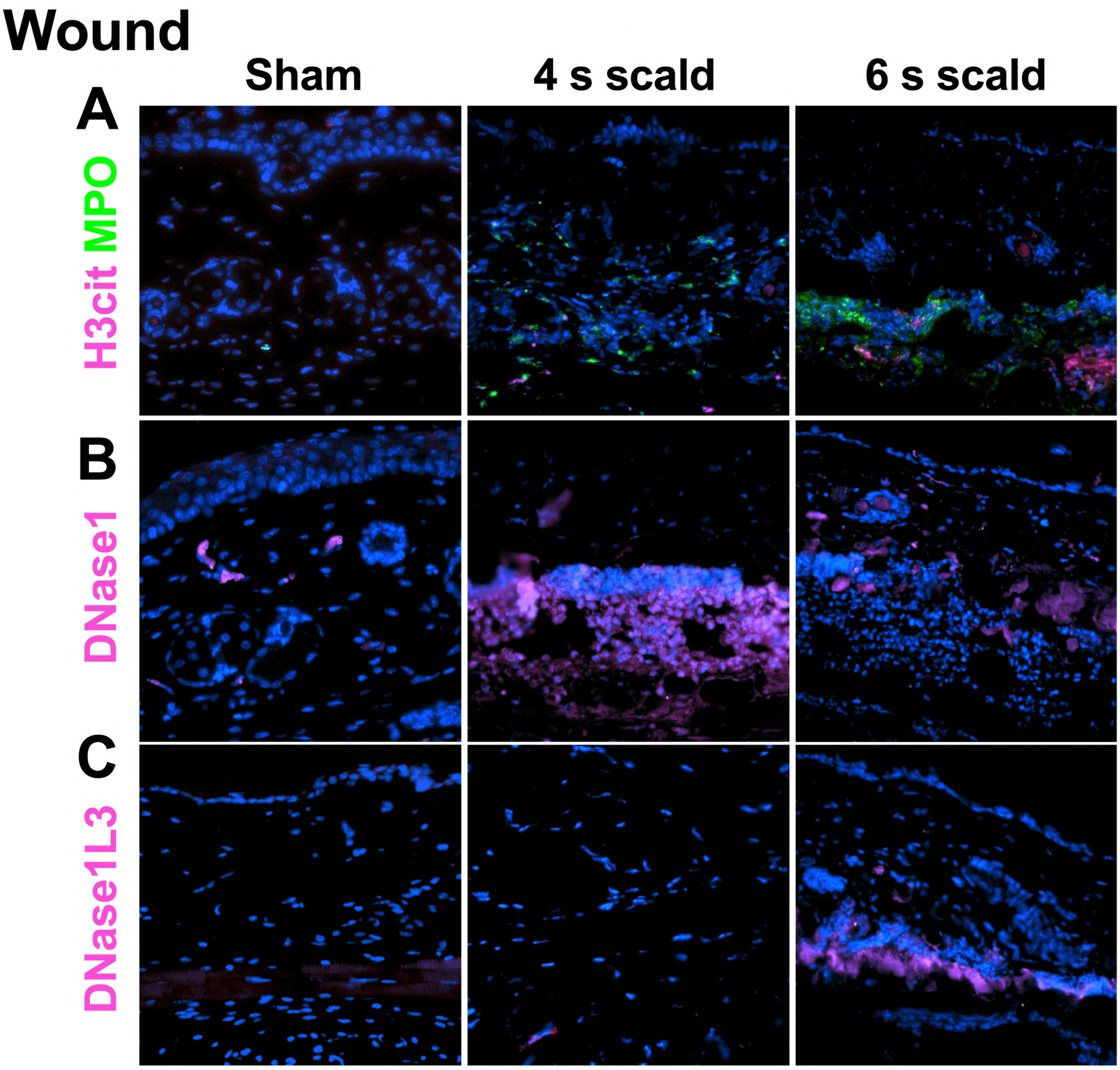

Fig. S6A: **NETs and NET-degrading nucleases increased in scalding wounds.** Representative merge images of immunofluorescence staining of the wound for H3cit/MPO (A), DNase1 (B), and DNase1L3 (C) are displayed. DNA counterstain was performed with DAPI (blue). Magnification level 40x. Note that we have seen significant NET formation even with 4 s scalds.

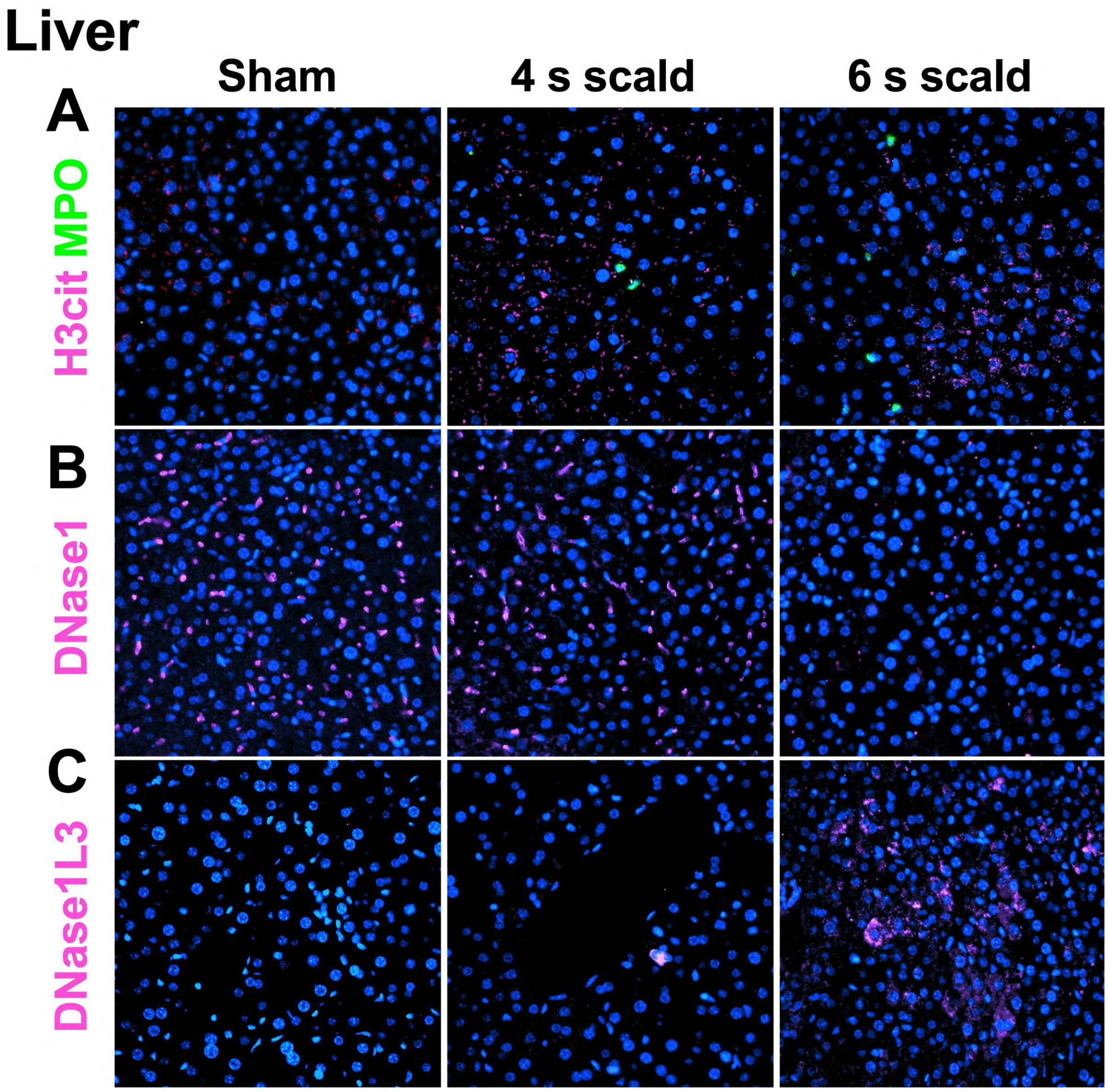

Fig. S6B. **Significant NET formation, decreased DNase1 and increased DNase1L3 in the liver after 6 s scald.** Representative merge images of immunofluorescence staining of the liver for H3cit/MPO (A), DNase1 (B), and DNase1L3 (C) are displayed. DNA counterstain was performed with DAPI (blue). Magnification level 40x. Note that in the group of 6 s scalds DNase1 behaves inversely to the other parameters.

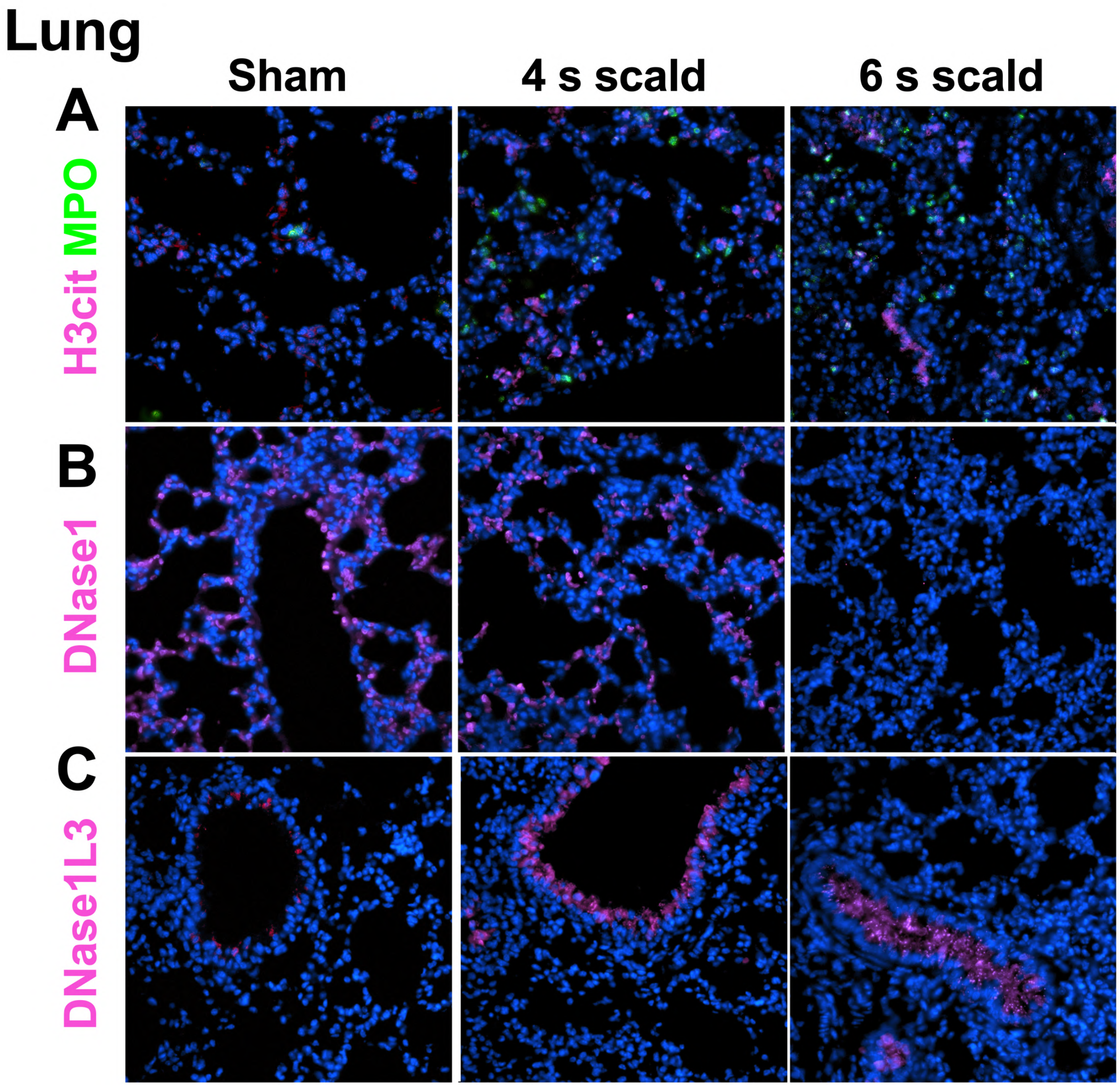

Fig. S6C: **Scalding for 4 s and 6 s induces NET formation and DNase1L3 upregulation.** Representative merge images of immunofluorescence staining of the lung for H3cit/MPO (A), DNase 1 (B), and DNase 1L3 (C) are displayed. DNA counterstain was performed with DAPI (blue). Magnification level 40x. Note that in the group of 6 s scalds DNase1 behaves inversely to the other parameters.

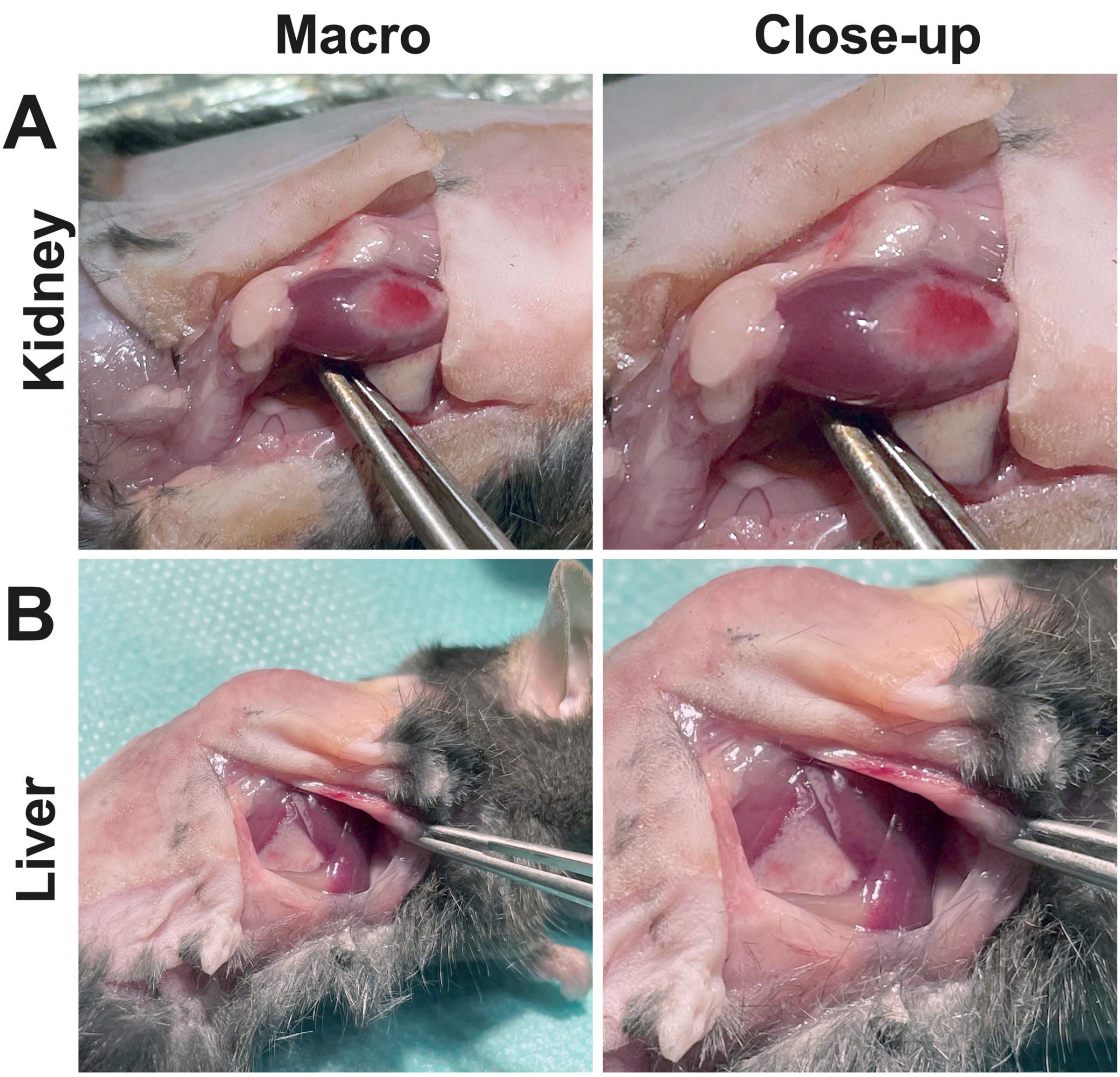

Fig. S7: **7 s scalding causes damage of kidneys and liver.** Representative macroscopic images of the kidney (A) and liver (B) of two mice from 7 s scald group, which had to be sacrificed, are displayed.

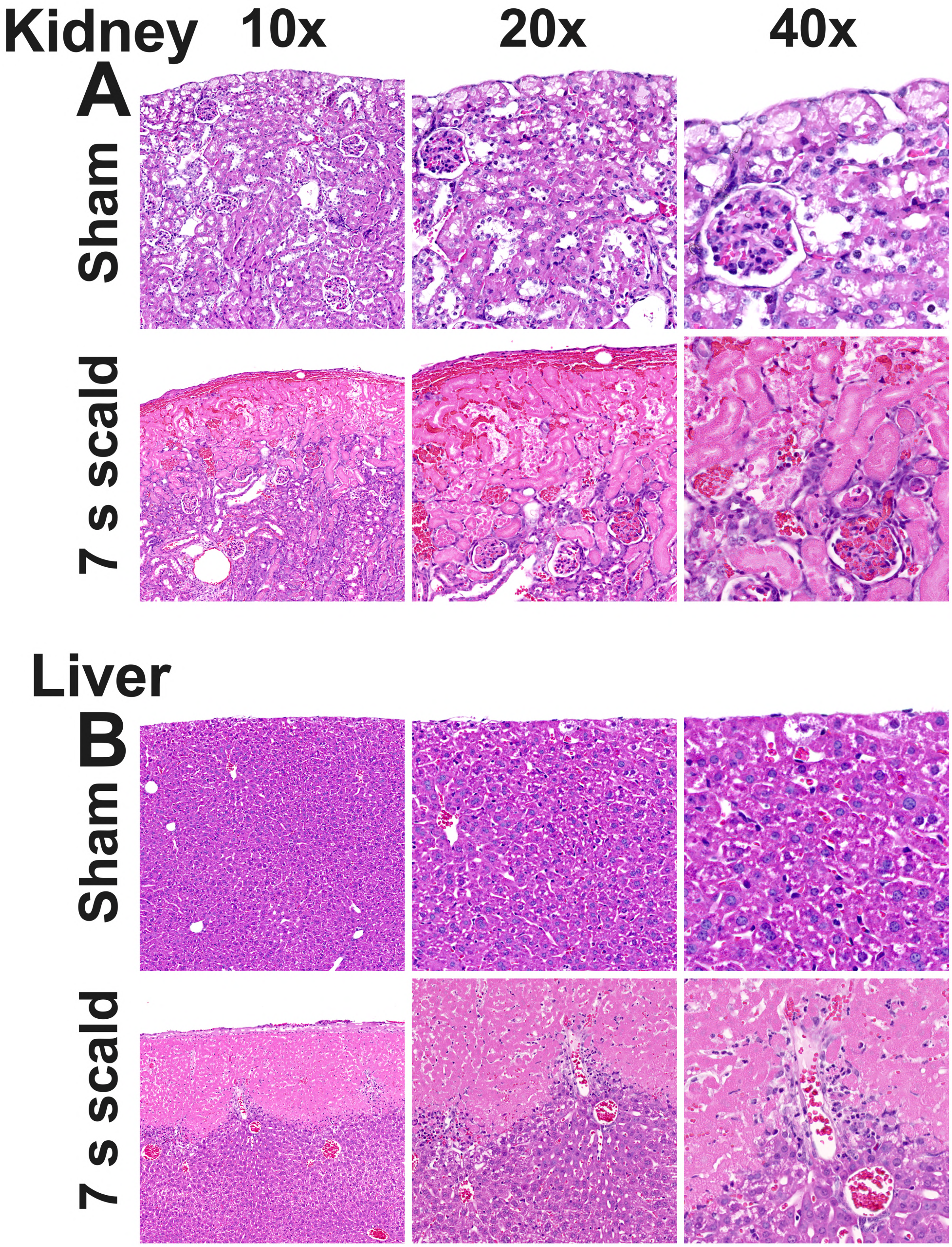

Fig. S8: **7 s scald causes necrotic tissue damage in kidney and liver.** Representative histological images of the kidney (A) and liver (B) of two mice from 7 s scald group, which had to be sacrificed, are displayed in comparison to sham. 10x, 20x, and 40x magnifications are shown. Note the sharply bounded necrotic area.

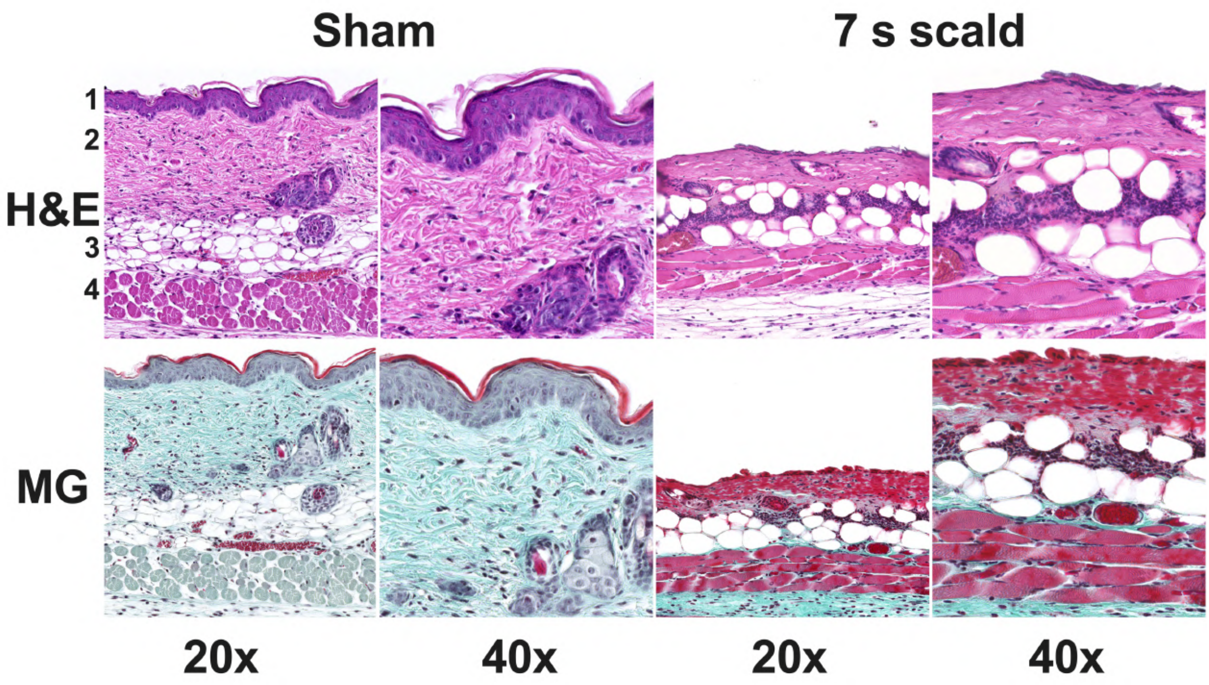

Fig. S9: **Histopathological evaluation reveals a destruction of the epidermal and dermal skin layers and a distinct leucocyte infiltration after 24 h.** Representative images in two magnifications of hematoxylin and eosin (H&E) and Masson Goldner Trichrome (MG) of sham group compared to 7 s scald are shown. For orientation, the main murine skin layers are displayed in one image (1: epidermis, 2: dermis, 3: dermal white adipose tissue, 4: panniculus carnosus). Note the complete destruction of the epidermis and the shrinking of the dermis.

Gene	Protein name	Function	Log2FC
		Liver top 5 upregulated	
Saa2	Serum amyloid A2	Acute phase protein	11,21697143
Saa1	Serum amyloid A1	Acute phase protein	9,652682200
Lcn2	Lipocalin-2	Neutrophil innate immunity	8,566388467
Mt2	Metallothionein-2	Heavy metals binding	7,845776444
Mt1	Metallothionein-1	Heavy metals binding	6,264934479
		Liver top 5 downregulated	
Ugt1a9	UDP-Glucuronosyltransferase 1-9	Glucuronidation pathway	-6,127202235
Atp2b2	ATPase Plasma Membrane Ca^{2+} Transporting 2	Intracellular calcium homeostasis	-5,310555817
Ugt1a5	UDP-Glucuronosyltransferase 1-5	Glucuronidation pathway	-5,302704396
Ushbp1	USH1 Protein Network Component Harmonin Binding Protein 1	PDZ domain binding activity	-4,999354129
A1bg	Alpha-1-B Glycoprotein	Innate Immunity	-4,998026812

Tab. S1: **Top 5 up- and downregulated coding mRNA molecules in liver.** Shown are the detected genes, the protein names, the functions and the corresponding log2FC values. Functions of proteins were taken out of GeneCard database provided by the Weizmann Institute of Science.

Gene	Protein name	Function	Log2FC
		Lung top 5 upregulated	
Pla1a	Phospholipase A1 Member A	Hydrolyzes fatty acids	3,36476881
Apod	Apolipoprotein D	Component of high-density lipoprotein	2,94194633
Ahsg	Alpha 2-HS Glycoprotein	Endocytoses promotion/ Reduces ectopic calcification	2,846083195
Serpina1b	Alpha-1-Antitrypsin	Protease Inhibitor	2,310397035
Ttr	Transthyretin	Thyroid hormone binding	2,207143074
		Lung top 5 downregulated	
Spon2	Spondin 2	Lipopolysaccharide binding activity	-2,814544263
Wisp2	WNT1-inducible-signaling pathway protein 2	Connective tissue growth factor	-2,718376276
Jchain	Joining Chain of Multimeric IgA/IgM	IgA binding activity	-2,637237949
Muc5b	Mucin 5B	Component of mucus secretions	-2,553768354
Dio2	Iodothyronine Deiodinase 2	T4 to T3 conversion	-2,531324546

Tab. S2: **Top 5 up- and downregulated coding mRNA molecules in lung.** Shown are the detected genes, the protein names, the functions and the corresponding log2FC values. Functions of proteins were taken out of GeneCard database provided by the Weizmann Institute of Science.

Table S3

Ensembl id	gene name	01ShamLiverA	03Ctrl1LiverA	05Ctrl2LiverA	07Ctrl3LiverA	09Ctrl4LiverA	log2FC	|log2FC| >= 1	abs log2FC
ENSMUSG00000022097	Sftpc	0	267,5427844	2216,456409	966,0338667	482,7572767	1023,41977	True	1023,41977
ENSMUSG00000056370	Sftpb	0	98,43555276	910,5347346	463,3363831	208,7599034	1021,977251	True	1021,977251
ENSMUSG00000024653	Scgb1a1	0	1795,817841	9016,407299	5696,113545	4640,993603	1019,522713	True	1019,522713
ENSMUSG00000070564	Ntn5	0	222,1109908	2,641783563	5,623014358	2263,740203	1016,757656	True	1016,757656
ENSMUSG00000039057	Myo16	0	323,0705321	0,880594521	458,8379716	174,8364191	1015,172694	True	1015,172694
ENSMUSG00000086503	Xist	0,772838763	6889,226699	9583,51017	4013,707649	6479,385503	12,34248046	True	12,34248046
ENSMUSG00000057465	Saa2	66,4641336	323822,6807	158209,3728	277892,7434	180257,6529	11,21697143	True	11,21697143
ENSMUSG00000074115	Saa1	104,333233	167611,7685	83978,77708	98467,97823	116533,6923	9,6526822	True	9,6526822
ENSMUSG00000026822	Lcn2	108,1974268	402158,4508	41016,33159	111479,6335	510203,985	8,566388467	True	8,566388467
ENSMUSG00000031762	Mt2	88,87645772	84029,88821	20445,64359	23647,02458	139895,2303	7,845776444	True	7,845776444
ENSMUSG00000031765	Mt1	215,6220148	86436,51128	16581,59483	26263,97546	136305,8647	6,264934479	True	6,264934479
ENSMUSG00000090175	Ugt1a9	550,2611991	1,261994266	2,641783563	7,872220101	6,523746983	-6,127202235	True	6,127202235
ENSMUSG00000025150	Cbr2	2,318516288	146,3913349	752,9083153	583,6688904	220,502648	5,980484488	True	5,980484488
ENSMUSG00000087579	Hectd2os	1069,608848	12,61994266	10,56713425	0	16,96174215	-5,978655136	True	5,978655136
ENSMUSG00000015452	Ager	6,182710102	312,974578	297,640948	319,3872155	293,5686142	5,569314564	True	5,569314564
ENSMUSG00000061540	Orm2	207,8936272	9825,887356	11844,8769	10310,35913	11428,29996	5,562670261	True	5,562670261
ENSMUSG00000030302	Atp2b2	103,5603942	5,047977065	372,4914823	40,48570338	2,609498793	-5,310555817	True	5,310555817
ENSMUSG00000089943	Ugt1a5	2265,963253	13,88193693	23,77605206	43,85951199	57,40897345	-5,302704396	True	5,302704396
ENSMUSG00000034911	Ushbp1	208,666466	75,71965597	485,207581	677,0109287	6,523746983	-4,999354129	True	4,999354129
ENSMUSG00000022347	A1bg	14689,34636	10,09595413	459,6703399	7,872220101	204,8456553	-4,998026812	True	4,998026812
ENSMUSG00000072664	Ugt3a1	444,3822886	1,261994266	14,97010685	2,249205743	7,828496379	-4,891644893	True	4,891644893
ENSMUSG00000029417	Cxcl9	196,3010458	44,16979931	46,67150961	6,74761723	1017,704529	-4,862545912	True	4,862545912
ENSMUSG00000064246	Chil1	6,955548865	323,0705321	191,9696055	978,4044983	260,9498793	4,786569735	True	4,786569735
ENSMUSG00000039648	Kyat1	3070,488405	316,7605608	9468,152288	2802,510356	122,6464433	-4,645890853	True	4,645890853
ENSMUSG00000036083	Slc17a3	1284,458024	922,5178085	2594,231458	2397,653322	56,10422405	-4,516906539	True	4,516906539
ENSMUSG00000004098	Col5a3	208,666466	10,09595413	627,8638934	429,598297	9,133245776	-4,513927302	True	4,513927302
ENSMUSG00000027762	Sucnr1	1020,147167	2,523988532	44,91032056	8,996822973	3,91424819	-4,505586461	True	4,505586461
ENSMUSG00000026475	Rgs16	617,4981715	1052,503218	27,29843015	150,6967848	1907,543618	-4,499544971	True	4,499544971
ENSMUSG00000044734	Serpinb1a	542,5328115	8,833959863	20,25367398	7,872220101	24,79023853	-4,451866334	True	4,451866334
ENSMUSG00000038550	Ciart	6,955548865	244,8268876	149,7010685	152,9459905	307,9208576	4,427776346	True	4,427776346
ENSMUSG00000046160	Olig1	47,91600329	137,557375	25,5372411	2,249205743	563,6517393	-4,413020036	True	4,413020036
ENSMUSG00000089960	Ugt1a1	3711,944578	50,47977065	119,7608548	79,84680388	174,8364191	-4,40809753	True	4,40809753
ENSMUSG00000031489	Adrb3	109,7431043	10,09595413	657,8041071	93,34203834	5,218997586	-4,394213746	True	4,394213746
ENSMUSG00000029272	Sult1e1	1,545677526	1124,436891	31,70140275	332,88245	373,1583274	4,358235413	True	4,358235413
ENSMUSG00000087516	Tbx3os1	131,3825897	7,571965597	487,8493646	23,6166603	6,523746983	-4,331929468	True	4,331929468
ENSMUSG00000041653	Pnpla3	391,8292527	21,45390252	17,61189042	0	19,57124095	-4,190913102	True	4,190913102
ENSMUSG00000034837	Gnat1	28,59503422	3062,860084	486,96877	633,1514167	514,0712622	4,089992716	True	4,089992716
ENSMUSG00000075551	Cyp3a41a	3153,182152	7,571965597	80,1341014	7,872220101	185,2744143	-4,089072944	True	4,089072944
ENSMUSG00000029352	Crybb3	3,091355051	4505,31953	51,07448221	1376,513915	2985,266619	4,046291406	True	4,046291406
ENSMUSG00000002265	Peg3	234,9429839	55,52774771	1598,279055	5128,189095	14,35224336	-4,032962522	True	4,032962522
ENSMUSG00000018339	Gpx3	133,701106	3609,303601	2151,292414	6836,460857	2332,891921	4,008120332	True	4,008120332
ENSMUSG00000021789	Sftpa1	4,637032577	74,4576617	338,148296	203,5531198	120,0369445	4,005146543	True	4,005146543
ENSMUSG00000003355	Fkbp11	59,50858474	1514,393119	1232,832329	950,2894265	1353,025124	3,997197265	True	3,997197265
ENSMUSG00000025185	Loxl4	289,8145361	10,09595413	18,49248494	6,74761723	5,218997586	-3,970118951	True	3,970118951
ENSMUSG00000030170	Wnt5b	100,4690392	117,3654668	265,9395453	188,9332824	6,523746983	-3,944906345	True	3,944906345
ENSMUSG00000020429	Igfbp1	2830,908388	7863,486272	198,1337672	9763,802131	7735,859172	-3,836718383	True	3,836718383
ENSMUSG00000021091	Serpina3n	4796,237362	210067,7796	67585,62948	98893,07812	224449,5149	3,81674156	True	3,81674156
ENSMUSG00000037411	Serpine1	2,318516288	1282,186174	31,70140275	751,2347182	1097,294242	3,773272912	True	3,773272912
ENSMUSG00000032329	Hmg20a	225,6689187	194,347117	416,5212084	459,9625745	16,96174215	-3,733851468	True	3,733851468
ENSMUSG00000004187	Kifc2	204,0294334	25,23988532	588,2371399	408,2308424	15,65699276	-3,703898245	True	3,703898245
ENSMUSG00000061322	Dnaic1	17,00245278	6,309971331	752,9083153	7,872220101	1,304749397	-3,703898245	True	3,703898245
ENSMUSG00000024036	Slc37a1	19,32096097	827,8682386	245,6858713	558,9276272	807,6398764	3,668575533	True	3,668575533
ENSMUSG00000031722	Hp	18556,63153	373646,2143	231022,2114	239174,9157	302348,2729	3,638024808	True	3,638024808
ENSMUSG00000036995	Asap3	129,8369122	140,0813635	442,939044	447,5919429	10,43799517	-3,63678405	True	3,63678405
ENSMUSG00000030659	Nucb2	35,55058309	946,4956996	442,0584495	526,3141439	581,9182308	3,636292009	True	3,636292009
ENSMUSG00000025127	Gcgr	3009,434142	325,5945207	7225,278044	4862,782817	242,6833878	-3,632344984	True	3,632344984
ENSMUSG00000040473	Cfap69	3,091355051	453,0559415	36,10437536	1034,634642	131,779689	3,545862416	True	3,545862416
ENSMUSG00000066361	Serpina3c	0,772838763	455,5799301	8,805945209	209,1761341	319,6636021	3,510238506	True	3,510238506
ENSMUSG00000020037	Rfx4	178,5257542	3,785982798	15,85070138	15,7444402	11,74274457	-3,493513629	True	3,493513629
ENSMUSG00000037243	Zfp692	142,2023324	74,4576617	619,0579482	410,4800481	13,04749397	-3,446100488	True	3,446100488
ENSMUSG00000096688	Mup17	645,3203669	439,1740046	235,1187371	59,6039522	12256,81583	-3,436535658	True	3,436535658
ENSMUSG00000031904	Slc7a6	1,545677526	280,1627271	16,7312959	196,8055025	610,6227176	3,436237925	True	3,436237925
ENSMUSG00000001155	Ftcd	5245,256683	4575,991209	3577,855538	15646,59975	491,8905225	-3,414604211	True	3,414604211
ENSMUSG00000062515	Fabp4	42,50613195	813,9863017	516,0283892	452,0903544	1148,179469	3,410868252	True	3,410868252
ENSMUSG00000038233	Fam198a	377,918155	2,523988532	38,74615892	1,124602872	1,304749397	-3,285948625	True	3,285948625
ENSMUSG00000090145	Ugt1a6b	367,8712511	1,261994266	37,8655644	12,37063159	9,133245776	-3,280242597	True	3,280242597
ENSMUSG00000040964	Arhgef10l	350,8687983	344,5244347	345,1930522	845,7013595	36,5329831	-3,263660192	True	3,263660192
ENSMUSG00000037095	Lrg1	3718,900127	81894,59391	37712,34095	35255,17542	96223,96324	3,24488715	True	3,24488715
ENSMUSG00000011263	Exoc3l2	115,9258144	65,62370184	127,6862055	298,019761	13,04749397	-3,151357222	True	3,151357222
ENSMUSG00000040350	Trim7	276,6762771	673,9049381	336,387107	1391,133752	31,31398552	-3,143319903	True	3,143319903
ENSMUSG00000040663	Clcf1	20,09380783	540,1335459	174,3577151	517,3173209	208,7599034	3,117227313	True	3,117227313
ENSMUSG00000053113	Socs3	108,9702656	4926,825615	904,3705729	2231,212097	5062,427658	3,052979525	True	3,052979525
ENSMUSG00000015533	Itga2	0,772838763	355,8823831	6,164161646	290,1475409	225,7216456	2,995665333	True	2,995665333
ENSMUSG00000059060	Rad51b	135,2467835	69,40968464	382,1780221	268,7800863	16,96174215	-2,995238021	True	2,995238021
ENSMUSG00000031150	Ccdc120	14,68393649	800,1043647	116,2384768	605,0363449	310,5303564	2,984777017	True	2,984777017
ENSMUSG00000021285	Ppp1r13b	193,9825295	261,2328131	225,4321973	328,3840385	24,79023853	-2,968082667	True	2,968082667
ENSMUSG00000078674	Mup18	131,3825897	104,7455241	63,4028055	16,86904307	982,4762956	-2,961324066	True	2,961324066
ENSMUSG00000032271	Nnmt	499,2538408	13649,72998	3829,705571	4198,14252	11369,58624	2,93938805	True	2,93938805
ENSMUSG00000076617	Ighm	785,204183	42,90780505	103,0295589	33,73808615	49,58047707	-2,930009556	True	2,930009556
ENSMUSG00000044258	Ctla2a	4,637032577	339,4764576	35,22378083	380,1157706	263,5593781	2,925276006	True	2,925276006
ENSMUSG00000005803	Sqor	2694,888766	235,9929278	354,8795919	220,4221628	258,3403805	-2,924824209	True	2,924824209
ENSMUSG00000052726	Kcnt2	10,81974268	932,6137627	81,89529044	1420,373427	319,6636021	2,9201143	True	2,9201143
ENSMUSG00000078651	Aoc2	6,955548865	256,184836	51,95507673	141,6999618	503,6332671	2,901028459	True	2,901028459
ENSMUSG00000040026	Saa3	30,91355051	36416,10654	230,7157645	23730,24519	32467,38398	2,899805318	True	2,899805318
ENSMUSG00000061878	Sphk1	1,545677526	217,0630138	11,44772877	143,9491676	373,1583274	2,888750129	True	2,888750129
ENSMUSG00000028445	Enho	317,6367315	42,90780505	1309,444053	192,307091	46,97097827	-2,888065748	True	2,888065748
ENSMUSG00000041828	Abca8a	1355,55919	11,3579484	188,4472275	57,35474645	35,22823371	-2,846655635	True	2,846655635
ENSMUSG00000019232	Etnppl	1683,242825	3,785982798	235,1187371	6,74761723	114,8179469	-2,839781896	True	2,839781896
ENSMUSG00000040829	Zmynd15	112,0616206	36,59783372	239,5217097	172,0642394	15,65699276	-2,839413216	True	2,839413216
ENSMUSG00000022111	Uchl3	213,3034985	302,8786239	148,820474	30,36427753	397,9485659	-2,81246068	True	2,81246068
ENSMUSG00000032113	Chek1	5,40987134	95,91156423	37,8655644	121,4571101	429,2625515	2,807220244	True	2,807220244
ENSMUSG00000034867	Ankrd27	360,1428635	411,4101308	711,5203729	1806,112212	52,18997586	-2,786724677	True	2,786724677
ENSMUSG00000067279	Ppp1r3c	1388,791257	3,785982798	203,4173343	12,37063159	6,523746983	-2,771315241	True	2,771315241
ENSMUSG00000017167	Cntnap1	27,0493567	267,5427844	186,6860384	184,4348709	481,4525273	2,769445262	True	2,769445262
ENSMUSG00000031725	Ces1f	5718,234006	503,5357122	747,6247482	19,11824882	838,953862	-2,768906283	True	2,768906283
ENSMUSG00000023800	Tiam2	234,9429839	16,40592546	33,46259179	34,86268902	20,87599034	-2,752554929	True	2,752554929
ENSMUSG00000040146	Rgl3	656,1401096	499,7497294	1137,728121	1193,203647	97,85620474	-2,74526868	True	2,74526868
ENSMUSG00000072849	Serpina1e	3681,803866	729,4326858	48395,71368	552,18001	2974,828624	-2,737202209	True	2,737202209
ENSMUSG00000010051	Hyal1	417,3329319	413,9341193	796,0574469	976,1552926	62,62797103	-2,736319723	True	2,736319723
ENSMUSG00000049303	Syt12	6,955548865	542,6575344	45,79091508	405,9816367	487,9762743	2,718825128	True	2,718825128
ENSMUSG00000030254	Rad18	11,59258144	147,6533291	1,761189042	69,72537804	464,4907852	-2,718580184	True	2,718580184
ENSMUSG00000044505	Lingo4	16,22961402	639,8310929	106,551937	194,5562968	465,7955346	2,714856226	True	2,714856226
ENSMUSG00000045790	Ccdc149	15,45677526	227,1589679	101,2683699	467,8347946	246,5976359	2,711872367	True	2,711872367
ENSMUSG00000028957	Per3	68,00981113	249,8748647	829,5200386	573,5474645	10,43799517	-2,703898245	True	2,703898245
ENSMUSG00000009092	Derl3	78,05671504	552,7534886	504,5806605	10396,95355	520,5950092	2,692490257	True	2,692490257
ENSMUSG00000032177	Pde4a	92,74065154	54,26575344	309,9692713	182,1856652	14,35224336	-2,691925604	True	2,691925604
ENSMUSG00000024981	Acsl5	6115,47313	778,6504622	948,400299	738,8640867	669,3364404	-2,688896096	True	2,688896096
ENSMUSG00000030806	Stx1b	138,3381385	246,0888819	22,01486302	223,7959715	437,0910478	-2,651649176	True	2,651649176
ENSMUSG00000021213	Akr1c13	971,4583248	49,21777638	154,9846357	74,22378953	71,76121681	-2,648026904	True	2,648026904

ENSMUSG00000021594	Srd5a1	1109,023625	7,571965597	177,8800932	6,74761723	13,04749397	-2,640313127	True	2,640313127
ENSMUSG00000020893	Per1	83,46658638	3216,823384	516,9089837	2831,750031	5324,682287	2,630639602	True	2,630639602
ENSMUSG00000032816	Igdcc4	2,318516288	93,38757569	85,41766852	795,0942302	14,35224336	2,630002491	True	2,630002491
ENSMUSG00000053964	Lgals4	132,9282672	1761,743996	821,594688	2803,634959	1490,023811	2,627778928	True	2,627778928
ENSMUSG00000019577	Pdk4	292,1330523	2102,482447	47,55210413	501,5728807	6359,348559	-2,619044511	True	2,619044511
ENSMUSG00000030762	Aqp8	3483,184304	148,9153234	374,2526714	22,49205743	579,3087321	-2,588002495	True	2,588002495
ENSMUSG00000038188	Scarf1	194,7553682	121,1514495	733,5352359	316,0134069	32,61873491	-2,577890361	True	2,577890361
ENSMUSG00000027931	Npr1	194,7553682	99,69754702	420,0435864	610,6593593	32,61873491	-2,577890361	True	2,577890361
ENSMUSG00000049858	Suox	1843,993288	74,4576617	309,9692713	60,72855507	90,02770836	-2,572636298	True	2,572636298
ENSMUSG00000056234	Ncoa4	660,7771422	56,78974198	112,7160987	112,4602872	79,58971319	-2,551470198	True	2,551470198
ENSMUSG00000028194	Ddah1	2378,797712	70,6716789	412,9988303	43,85951199	198,3219083	-2,526022992	True	2,526022992
ENSMUSG00000038895	Zfp653	72,6468437	1451,293406	409,4764522	1938,815351	1088,160997	2,494808466	True	2,494808466
ENSMUSG00000034035	Ccdc17	72,6468437	56,78974198	225,4321973	160,8182106	13,04749397	-2,477127384	True	2,477127384
ENSMUSG00000006638	Abhd1	86,55794143	222,1109908	323,1781892	421,7260769	15,65699276	-2,466859048	True	2,466859048
ENSMUSG00000037405	Icam1	186,2541418	1110,554954	1013,564294	2589,960413	1364,767869	2,444093193	True	2,444093193
ENSMUSG00000024827	Gldc	2709,572702	239,7789106	494,0135262	498,1990721	118,7321951	-2,443271118	True	2,443271118
ENSMUSG00000039196	Orm1	11012,95237	126379,8918	59575,74171	94628,58403	101870,9186	2,435523727	True	2,435523727
ENSMUSG00000029591	Ung	39,4147769	382,3842626	213,103874	240,6650145	1107,732238	2,434748307	True	2,434748307
ENSMUSG00000002769	Gnmt	23141,88391	1394,503664	4345,73396	1363,01868	3081,818075	-2,412834552	True	2,412834552
ENSMUSG00000029596	Sdsl	94,28632906	6940,968464	500,1776878	6193,188014	4022,542389	2,407320193	True	2,407320193
ENSMUSG00000054708	Ankrd24	165,3874952	75,71965597	331,9841344	229,4189858	31,31398552	-2,400971112	True	2,400971112
ENSMUSG00000030895	Hpx	35872,08401	291306,1365	241027,5263	188342,8659	223576,6376	2,39242792	True	2,39242792
ENSMUSG00000031963	Bmper	7,728387628	546,4435172	40,50734796	443,0935314	245,2928865	2,389944273	True	2,389944273
ENSMUSG00000002222	Rmnd5a	1714,929215	2588,35024	376,8944549	328,3840385	6150,588655	-2,384693119	True	2,384693119
ENSMUSG00000020431	Adcy1	58,73574597	903,5878946	11,44772877	518,4419238	1277,349659	-2,359177384	True	2,359177384
ENSMUSG00000019590	Cyb561	37,86909938	397,5281938	193,7307946	232,7927944	388,8153202	2,354960281	True	2,354960281
ENSMUSG00000049404	Rarres1	252,7182754	175,417203	339,9094851	49,48252635	457,9670382	-2,352538932	True	2,352538932
ENSMUSG00000032690	Oas2	132,9282672	100,9595413	150,5816631	275,5277035	26,09498793	-2,348803287	True	2,348803287
ENSMUSG00000076441	Ass1	51914,67105	3321,568908	10235,15012	2598,957236	8574,813034	-2,342610039	True	2,342610039
ENSMUSG00000009566	Fpgs	2837,091098	1968,711055	4133,510681	5146,182741	559,7374911	-2,341590188	True	2,341590188
ENSMUSG00000022304	Dpys	5040,454411	10,09595413	995,0718086	110,2110814	86,11346017	-2,340681257	True	2,340681257
ENSMUSG00000037573	Tob1	2421,303844	160,2732718	478,1628248	132,7031389	444,9195442	-2,340210255	True	2,340210255
ENSMUSG00000001918	Slc1a5	55,64439092	319,2845493	516,9089837	432,9721056	280,5211202	2,333801221	True	2,333801221
ENSMUSG00000034471	Caskin2	182,389948	1205,204524	1102,50434	1557,574977	918,5435751	2,332321942	True	2,332321942
ENSMUSG00000034591	Slc41a2	128,2912346	1799,603824	644,5951893	671,3879144	1859,26789	2,328970818	True	2,328970818
ENSMUSG00000004038	Gstm3	1934,415423	47,95578211	386,5809947	5,623014358	210,0646528	-2,323055035	True	2,323055035
ENSMUSG00000024906	Mus81	135,2467835	95,91156423	265,0589508	188,9332824	27,39973733	-2,303360316	True	2,303360316
ENSMUSG00000069668	Sult3a1	3457,680625	49,21777638	702,7144276	38,23649764	254,4261323	-2,298794193	True	2,298794193
ENSMUSG00000026417	Pigr	27249,52194	2580,778274	5556,551427	1533,958317	3862,058214	-2,293969236	True	2,293969236
ENSMUSG00000071551	Akr1c19	745,0165673	7,571965597	153,2234466	28,11507179	13,04749397	-2,281635429	True	2,281635429
ENSMUSG00000068114	Ccdc134	71,87400494	975,5215677	348,7154303	3139,891218	1299,530399	2,27850822	True	2,27850822
ENSMUSG00000058258	Idi1	605,90559	27,76387386	125,044422	11,24602872	5,218997586	-2,276652313	True	2,276652313
ENSMUSG00000029380	Cxcl1	131,3825897	2670,379867	1390,458748	635,4006225	2638,20328	2,273892396	True	2,273892396
ENSMUSG00000040721	Zfhx2	245,7627266	397,5281938	712,4009674	750,1101154	50,88522646	-2,271947363	True	2,271947363
ENSMUSG00000040270	Bach2	93,5134903	249,8748647	19,37307946	91,0928326	61,32322164	-2,271121207	True	2,271121207
ENSMUSG00000026077	Npas2	275,9034383	20,19190826	3,522378083	2,249205743	57,40897345	-2,264815272	True	2,264815272
ENSMUSG00000002109	Ddb2	87,3307802	112,3174897	174,3577151	548,8062014	18,26649155	-2,257290667	True	2,257290667
ENSMUSG00000079045	Prox1os	129,8369122	229,6829564	243,9246823	221,5467657	27,39973733	-2,244466627	True	2,244466627
ENSMUSG00000052921	Arhgef15	98,15052288	99,69754702	176,1189042	200,1793111	20,87599034	-2,233151314	True	2,233151314
ENSMUSG00000042350	Arel1	386,4193814	239,7789106	334,6259179	964,9092638	83,50396138	-2,210250911	True	2,210250911
ENSMUSG00000015357	Clpx	3031,846466	540,1335459	656,9235126	602,7871392	494,5000213	-2,206399388	True	2,206399388
ENSMUSG00000022671	Mzt2	129,0640734	148,9153234	136,4921507	28,11507179	288,3496166	-2,198671828	True	2,198671828
ENSMUSG00000004730	Adgre1	161,5233014	10,09595413	35,22378083	30,36427753	18,26649155	-2,197120626	True	2,197120626
ENSMUSG00000036782	Klhl13	165,3874952	8,833959863	36,10437536	14,61983733	20,87599034	-2,19560457	True	2,19560457
ENSMUSG00000047638	Nr1h4	2082,800466	1519,441096	4165,212084	3504,262548	456,6622888	-2,189325073	True	2,189325073
ENSMUSG00000034254	Agpat1	178,5257542	270,066773	789,0126907	483,5792348	39,1424819	-2,189325073	True	2,189325073
ENSMUSG00000003541	Ier3	17,00245278	2812,985219	77,49231784	969,4076753	2846,963183	2,188310411	True	2,188310411
ENSMUSG00000044244	Il20rb	4,637032577	302,8786239	21,1342685	346,3776845	326,1873491	2,188310411	True	2,188310411
ENSMUSG00000057880	Abat	5664,908131	132,5093979	1243,399463	229,4189858	276,6068721	-2,187762698	True	2,187762698
ENSMUSG00000021453	Gadd45g	115,1529757	5826,627527	523,9537399	2931,839686	4836,706013	2,185887749	True	2,185887749
ENSMUSG00000004568	Arhgef18	438,1995785	590,6133166	477,2822303	926,6727662	96,55145534	-2,182218186	True	2,182218186
ENSMUSG00000019139	Isyna1	58,73574597	709,2407776	1769,114392	2753,02783	266,1688769	2,180031202	True	2,180031202
ENSMUSG00000006732	Mettl1	194,7553682	984,3555276	1084,011855	1810,610623	882,010592	2,179132886	True	2,179132886
ENSMUSG00000055866	Per2	128,2912346	1482,843263	1408,070639	1268,552039	580,6134814	2,178155468	True	2,178155468
ENSMUSG00000027870	Hao2	445,9279661	1,261994266	98,62658634	0	10,43799517	-2,176762175	True	2,176762175
ENSMUSG00000060860	Ube2s	116,6986532	165,3212489	122,4026384	25,86586605	748,9261536	-2,173666509	True	2,173666509
ENSMUSG00000004100	Ppan	132,1554284	807,6763303	594,4013016	1089,740183	832,430115	2,169201588	True	2,169201588
ENSMUSG00000028837	Psmb2	889,537416	1118,12692	982,7434853	197,9301054	1603,537008	-2,168064225	True	2,168064225
ENSMUSG00000063849	Ppcdc	181,6171093	235,9929278	506,3418495	888,4362686	40,44723129	-2,166787263	True	2,166787263
ENSMUSG00000024391	Apom	2473,85688	243,5648934	551,2521701	367,745139	412,3008093	-2,165977703	True	2,165977703
ENSMUSG00000052656	Rnf103	1695,608246	1446,245429	442,939044	377,8665649	6526,356481	-2,165854113	True	2,165854113
ENSMUSG00000032207	Lipc	4075,951635	870,7760436	619,0579482	399,2340194	912,0198282	-2,159999837	True	2,159999837
ENSMUSG00000031613	Hpgd	2374,160679	98,43555276	534,5208742	250,7864404	70,45646741	-2,151099383	True	2,151099383
ENSMUSG00000018849	Wwc1	1092,794011	1405,861612	1219,623411	1512,590862	246,5976359	-2,147790607	True	2,147790607
ENSMUSG00000024190	Dusp1	891,0830935	1082,79108	201,6561453	608,4101535	1847,525145	-2,143662598	True	2,143662598
ENSMUSG00000063929	Cyp4a32	258,1281468	488,391781	176,1189042	657,8926799	58,71372284	-2,136317823	True	2,136317823
ENSMUSG00000016382	Pls3	1275,956797	252,3988532	290,5961919	157,444402	237,4643902	-2,134491779	True	2,134491779
ENSMUSG00000028186	Uox	15807,64405	552,7534886	3603,392779	779,34979	1109,036987	-2,133194544	True	2,133194544
ENSMUSG00000037583	Nr0b2	956,7743883	87,07760436	175,2383097	218,1729571	2,609498793	-2,132706483	True	2,132706483
ENSMUSG00000023990	Tfeb	91,19497401	74,4576617	213,9844686	154,0705934	20,87599034	-2,127109676	True	2,127109676
ENSMUSG00000030802	Bckdk	238,0343389	305,4026124	331,1035398	413,8538568	54,79947465	-2,118935745	True	2,118935745
ENSMUSG00000044254	Pcsk9	706,3746292	630,9971331	1953,158647	594,9149191	163,0936746	-2,114732697	True	2,114732697
ENSMUSG00000026097	Ormdl1	209,4393047	34,07384519	48,43269865	12,37063159	19,57124095	-2,112478916	True	2,112478916
ENSMUSG00000025429	Pstpip2	709,4659843	44,16979931	165,5517699	25,86586605	45,66622888	-2,09945108	True	2,09945108
ENSMUSG00000028211	Trp53inp1	735,7425022	964,1636193	171,7159316	774,8513785	3066,161082	-2,099177038	True	2,099177038
ENSMUSG00000021809	Nudt13	206,3479497	136,2953807	334,6259179	411,604651	48,27572767	-2,095709193	True	2,095709193
ENSMUSG00000063480	Snu13	320,7280866	408,8861422	265,9395453	75,3483924	861,1346017	-2,089702061	True	2,089702061
ENSMUSG00000039738	Slx4	46,37032577	1453,817395	197,2531727	1763,377303	588,4419778	2,088774738	True	2,088774738
ENSMUSG00000052520	Cyp2j5	9945,662039	169,1072317	2347,664993	178,8118566	872,8773463	-2,082840855	True	2,082840855
ENSMUSG00000021069	Pygl	6069,875643	459,3659129	1433,60788	509,4451008	508,8522646	-2,082016487	True	2,082016487
ENSMUSG00000032009	Sesn3	483,7970655	45,43179358	114,4772877	31,48888041	62,62797103	-2,079340623	True	2,079340623
ENSMUSG00000041959	S100a10	1449,07268	2258,969736	1620,293918	343,0038758	5880,50553	-2,078833173	True	2,078833173
ENSMUSG00000035948	Acss3	912,7225789	2,523988532	216,6262521	56,23014358	36,5329831	-2,074968333	True	2,074968333
ENSMUSG00000055652	Klhl25	159,2047851	138,8193693	271,2231124	224,9205743	37,8377325	-2,072986159	True	2,072986159
ENSMUSG00000001983	Taco1	177,7529154	11,3579484	42,268537	21,36745456	11,74274457	-2,072217139	True	2,072217139
ENSMUSG00000030786	Itgam	12,3654202	319,2845493	51,95507673	273,2784978	255,7308817	2,070953461	True	2,070953461
ENSMUSG00000052974	Cyp2f2	18078,24434	624,6871617	4315,793747	795,0942302	1187,321951	-2,066556753	True	2,066556753
ENSMUSG00000039096	Rsad1	71,10116618	4919,253649	297,640948	2726,037361	1201,674194	2,065627892	True	2,065627892
ENSMUSG00000020620	Abca8b	1443,662809	59,31373051	346,0736467	143,9491676	71,76121681	-2,060582826	True	2,060582826
ENSMUSG00000040563	Plppr2	244,217049	94,64956996	1788,487472	778,2251872	58,71372284	-2,056394279	True	2,056394279
ENSMUSG00000032418	Me1	3915,974011	699,1448234	537,1626577	444,2181343	942,0290643	-2,055527713	True	2,055527713
ENSMUSG00000037686	Aspg	2735,84922	6944,754447	4230,376078	18692,02433	658,8984452	-2,053860694	True	2,053860694
ENSMUSG00000020917	Acly	5901,396793	397,5281938	1424,801935	308,1411868	429,2625515	-2,050295084	True	2,050295084
ENSMUSG00000027199	Gatm	159,9776239	12,61994266	38,74615892	17,99364595	1,304749397	-2,045744928	True	2,045744928
ENSMUSG00000042659	Arrdc4	454,4291925	2714,549666	110,0743151	187,8086796	5682,183622	-2,045577649	True	2,045577649
ENSMUSG00000064373	Selenop	118276,0171	10970,51616	28691,53068	4464,6734	20462,38479	-2,043460725	True	2,043460725
ENSMUSG00000025507	Pidd1	27,82219546	136,2953807	114,4772877	376,741962	230,9406432	2,040753223	True	2,040753223
ENSMUSG00000050103	Agmo	1125,253239	68,14769037	273,864896	80,97140676	189,1886625	-2,038713459	True	2,038713459
ENSMUSG00000003865	Gys1	64,14561731	44,16979931	187,5666329	360,9975218	15,65699276	-2,034543557	True	2,034543557
ENSMUSG00000029195	Klb	411,9230606	29,02586812	101,2683699	33,73808615	86,11346017	-2,02419126	True	2,02419126
ENSMUSG00000023960	Enpp5	254,263953	13,88193693	62,52221098	41,61030625	52,18997586	-2,023886243	True	2,023886243
ENSMUSG00000057933	Gsta2	493,0711307	47,95578211	31,70140275	1,124602872	121,3416939	-2,022720429	True	2,022720429
ENSMUSG00000070044	Fam149a	453,6563538	109,7935012	111,8355041	74,22378953	108,2941999	-2,020221595	True	2,020221595

ENSMUSG00000028028	Alpk1	30,91355051	808,9383246	125,044422	2513,487418	335,3205949	2,016129436 True	2,016129436
ENSMUSG00000024665	Fads2	7113,207973	478,2958269	1758,547258	492,5760578	611,927467	-2,016116239 True	2,016116239
ENSMUSG00000090298	Sult3a2	179,298593	0	44,91032056	10,12142584	2,609498793	-1,997245242 True	1,997245242
ENSMUSG00000031010	Usp9x	669,2783686	795,0563877	168,1935535	490,326852	2214,159726	-1,992483975 True	1,992483975
ENSMUSG00000049152	Ugt3a2	3041,89337	217,0630138	692,1472934	31,48888041	764,5831464	-1,992224278 True	1,992224278
ENSMUSG00000055027	Smyd1	88,10361896	60,57572477	166,4323644	127,0801245	22,18073974	-1,9898938 True	1,9898938
ENSMUSG00000023908	Pkmyt1	27,0493567	191,8231285	107,4325315	885,06246	122,6464433	1,989764732 True	1,989764732
ENSMUSG00000031832	Taf1c	35,55058309	209,4910482	154,9846357	316,0134069	140,9129348	1,986858919 True	1,986858919
ENSMUSG00000032591	Mst1	2235,822541	1837,463651	5405,969764	7539,337651	567,5659875	-1,977945644 True	1,977945644
ENSMUSG00000001891	Ugp2	3471,591723	623,4251675	881,4751154	447,5919429	845,4776089	-1,977605542 True	1,977605542
ENSMUSG00000027820	Mme	1222,630923	85,8156101	69,56696715	24,74126318	310,5303564	-1,977182748 True	1,977182748
ENSMUSG00000062040	Zfp27	87,3307802	74,4576617	95,10420825	248,5372346	22,18073974	-1,977182748 True	1,977182748
ENSMUSG00000016495	Plgrkt	158,4319464	131,2474037	262,4171672	296,8951581	40,44723129	-1,969750416 True	1,969750416
ENSMUSG00000069456	Rdh16	1841,674772	205,7050654	470,2374741	254,160249	396,6438165	-1,969556893 True	1,969556893
ENSMUSG00000034584	Exph5	245,7627266	26,50187959	43,14913152	62,97776081	36,5329831	-1,964351759 True	1,964351759
ENSMUSG00000049728	Zfp668	34,77774433	151,4393119	135,6115562	231,6681916	144,827183	1,963243856 True	1,963243856
ENSMUSG00000026368	F13b	2861,821939	155,2252947	737,057614	430,7228998	395,3390671	-1,95708461 True	1,95708461
ENSMUSG00000057103	Nat8f1	2631,515987	97,17355849	678,0577811	34,86268902	327,4920985	-1,956414036 True	1,956414036
ENSMUSG00000048410	Zfp407	102,0147167	196,8711055	26,41783563	42,73490912	200,9314071	-1,949193112 True	1,949193112
ENSMUSG00000055912	Tmem150a	2593,646888	1930,851227	6027,669495	6113,34121	671,9459392	-1,948565005 True	1,948565005
ENSMUSG00000053286	Tmt1l	376,3724775	585,5653395	562,6998988	1701,524145	97,85620474	-1,943425898 True	1,943425898
ENSMUSG00000032724	Abtb2	436,653901	31,54985665	113,5966932	46,10871774	43,05673008	-1,942569391 True	1,942569391
ENSMUSG00000045996	Polr2k	103,5603942	118,627461	81,01469592	26,99046892	194,4076601	-1,939950415 True	1,939950415
ENSMUSG00000022565	Plec	593,5401698	9370,307426	2277,217431	5663,500061	13750,75389	1,93985447 True	1,93985447
ENSMUSG00000055491	Pprc1	78,05671504	1114,340937	299,4021371	608,4101535	1328,234886	1,939489865 True	1,939489865
ENSMUSG00000032369	Plscr1	219,4862086	581,7793567	57,23864386	125,9555216	2455,538364	-1,939068895 True	1,939068895
ENSMUSG00000061959	Ces1e	2289,148415	243,5648934	597,0430851	182,1856652	384,901072	-1,938904051 True	1,938904051
ENSMUSG00000026489	Coq8a	8215,276049	92,12558143	2146,008847	513,9435123	233,550142	-1,936653029 True	1,936653029
ENSMUSG00000057074	Ces1g	4952,350792	714,2887546	1110,429691	109,0864785	1294,311401	-1,935928749 True	1,935928749
ENSMUSG00000036503	Rnf13	2245,096606	369,76432	589,1177345	384,6141821	561,0422405	-1,930149636 True	1,930149636
ENSMUSG00000047496	Rnf152	295,9972462	44,16979931	78,37291236	44,98411486	58,71372284	-1,91715674 True	1,91715674
ENSMUSG00000060961	Slc4a4	870,2164469	1207,728513	230,7157645	600,5379334	2413,786384	-1,915257699 True	1,915257699
ENSMUSG00000020642	Rnf144a	132,1554284	147,6533291	35,22378083	98,9650527	370,5488286	-1,907614009 True	1,907614009
ENSMUSG00000068523	Gng5	1083,519945	1229,182415	1514,622576	289,022938	1747,059442	-1,90646981 True	1,90646981
ENSMUSG00000055116	Amtl	504,6637121	63,09971331	134,7309617	91,0928326	101,7704529	-1,905240928 True	1,905240928
ENSMUSG00000021127	Zfp36l1	7767,802405	1405,861612	1972,531727	666,8895029	2077,161039	-1,902893432 True	1,902893432
ENSMUSG00000041096	Tspyl2	217,1676923	2743,575535	811,9081482	11906,1706	10103,97933	1,902507032 True	1,902507032
ENSMUSG00000011752	Pgam1	364,0070573	71,93367317	88,94004661	26,99046892	97,85620474	-1,895231186 True	1,895231186
ENSMUSG00000022676	Snai2	159,9776239	17,66791973	41,38794248	17,99364595	43,05673008	-1,893559465 True	1,893559465
ENSMUSG00000027907	S100a11	132,1554284	1779,411915	489,6105536	601,6625363	3164,017287	1,889398969 True	1,889398969
ENSMUSG00000018599	Mief2	197,8467233	133,7713922	432,3719097	428,4736941	53,49472526	-1,886914622 True	1,886914622
ENSMUSG00000063232	Serpina11	286,723181	1405,861612	1460,90631	1880,336001	1058,151761	1,883816101 True	1,883816101
ENSMUSG00000072623	Zfp9	26,27651794	113,579484	96,86539729	386,8633878	662,8126934	1,882207284 True	1,882207284
ENSMUSG00000050965	Prkca	302,1799563	78,2436445	73,96993975	39,36110051	82,19921198	-1,878211501 True	1,878211501
ENSMUSG00000022554	Hgh1	37,09626061	136,2953807	136,4921507	220,4221628	172,2269203	1,877390995 True	1,877390995
ENSMUSG00000056973	Ces1d	9889,244809	843,0121698	2693,738639	104,5880671	1756,192688	-1,876250474 True	1,876250474
ENSMUSG00000057054	Inca1	325,3651191	759,7205482	492,2523372	2000,668509	88,72295896	-1,874680209 True	1,874680209
ENSMUSG00000049382	Krt8	2250,506477	2845,79707	2124,874579	614,0331679	6480,690252	-1,873861225 True	1,873861225
ENSMUSG00000020805	Slc13a5	14,68393649	1010,857407	53,71626577	393,6110051	896,3628354	1,871120235 True	1,871120235
ENSMUSG00000049971	Glt1d1	734,9696634	453,0559415	1455,622743	1025,637819	200,9314071	-1,870981617 True	1,870981617
ENSMUSG00000036093	Arl5a	1033,285426	170,3692259	197,2531727	143,9491676	283,130619	-1,867699144 True	1,867699144
ENSMUSG00000027187	Cat	42804,44772	1458,865372	11748,8921	1629,549561	3466,719147	-1,865235992 True	1,865235992
ENSMUSG00000043987	Cep164	190,1183356	143,8673463	211,342685	398,1094166	52,18997586	-1,865053037 True	1,865053037
ENSMUSG00000067928	Zfp760	214,8491761	30,28786239	58,9998329	20,24285169	19,57124095	-1,864541471 True	1,864541471
ENSMUSG00000058921	Slc10a5	522,4390037	13,88193693	143,5369069	14,61983733	3,91424819	-1,863840871 True	1,863840871
ENSMUSG00000025192	Entpd7	20,09380783	136,2953807	73,08934523	97,84044983	323,5778503	1,862910125 True	1,862910125
ENSMUSG00000044937	Ttc41	56,41722968	628,4731445	276,5066795	964,9092638	204,8456553	1,860329563 True	1,860329563
ENSMUSG00000044749	Abca6	1524,810879	230,9449507	406,8346686	420,601474	176,1411685	-1,858104506 True	1,858104506
ENSMUSG00000074743	Thbd	30,91355051	265,0187959	111,8355041	374,4927562	347,0633395	1,855067003 True	1,855067003
ENSMUSG00000038286	Bphl	1500,080039	59,31373051	414,7600193	128,2047274	110,9036987	-1,854690742 True	1,854690742
ENSMUSG00000040712	Camta2	501,5723571	5986,900799	1924,099028	13759,51613	1812,296912	1,853289589 True	1,853289589
ENSMUSG00000038400	Pmepa1	103,5603942	290,2586812	81,89529044	494,8252635	28,70448672	-1,851124199 True	1,851124199
ENSMUSG00000020376	Rnf130	878,7176733	143,8673463	228,0739809	136,0769475	243,9881371	-1,848588706 True	1,848588706
ENSMUSG00000035561	Aldh1b1	1273,638281	10,09595413	353,9989974	13,49523446	15,65699276	-1,847138425 True	1,847138425
ENSMUSG00000020017	Hal	6823,393437	678,9529152	1896,800598	570,1736559	236,1596408	-1,846921382 True	1,846921382
ENSMUSG00000053560	Ier2	595,8586861	1166,082702	166,4323644	446,46734	730,659662	-1,840034214 True	1,840034214
ENSMUSG00000028024	Enpep	1679,378632	288,9966869	470,2374741	140,575359	361,4155828	-1,836466116 True	1,836466116
ENSMUSG00000022270	Fam134b	1121,389045	2130,246321	314,3722439	1655,415427	5345,558278	-1,834741129 True	1,834741129
ENSMUSG00000062181	Ces3b	5534,29838	564,111437	1552,48814	600,5379334	1182,102953	-1,833818181 True	1,833818181
ENSMUSG00000026072	Il1r1	255,8096305	7873,582226	911,4153291	9364,568112	10820,28675	1,833038057 True	1,833038057
ENSMUSG00000052957	Gas1	357,0515084	2,523988532	100,3877754	7,872220101	9,133245776	-1,830548616 True	1,830548616
ENSMUSG00000045991	Onecut2	1677,060115	1832,415674	3358,587503	3596,479983	472,3192815	-1,828100067 True	1,828100067
ENSMUSG00000001227	Sema6b	61,82710102	2091,124499	219,2680357	1213,446498	859,8298523	1,826384249 True	1,826384249
ENSMUSG00000025172	Ankrd2	13,91109773	524,9896147	49,31329317	145,0737704	473,6240309	1,825740332 True	1,825740332
ENSMUSG00000034157	Cipc	364,779896	401,3141766	740,579992	1268,552039	103,0752023	-1,823328928 True	1,823328928
ENSMUSG00000020182	Ddc	1432,070227	320,5465436	405,0734796	190,0578853	99,16095413	-1,821846704 True	1,821846704
ENSMUSG00000025316	Banp	193,2096907	123,6754381	358,40197	391,3617993	54,79947465	-1,817933489 True	1,817933489
ENSMUSG00000027176	Cstf3	188,5726581	172,8932145	291,4767864	339,6300672	53,49472526	-1,81765196 True	1,81765196
ENSMUSG00000042680	Garem1	253,4911142	687,786875	72,20875071	256,4094547	585,832479	-1,811689589 True	1,811689589
ENSMUSG00000022885	St6gal1	1307,643187	241,0409048	213,9844686	374,4927562	297,4828624	-1,803959212 True	1,803959212
ENSMUSG00000015647	Lama5	60,2814235	237,254922	210,4620905	804,0910532	263,5593781	1,803775 True	1,803775
ENSMUSG00000045094	Arhgef37	381,0095101	17,66791973	109,1937206	19,11824882	11,74274457	-1,802937115 True	1,802937115
ENSMUSG00000023764	Sfi1	154,5677526	256,184836	437,6554769	557,8030243	44,36147948	-1,800859975 True	1,800859975
ENSMUSG00000040891	Foxa3	217,9405311	540,1335459	402,431696	512,8189095	62,62797103	-1,799055478 True	1,799055478
ENSMUSG00000030161	Gabarapl1	2809,268903	427,8160562	678,9383756	312,6395983	807,6398764	-1,798410676 True	1,798410676
ENSMUSG00000025402	Nab2	208,666466	357,1443773	347,8348357	172,0642394	60,01847224	-1,797720268 True	1,797720268
ENSMUSG00000024694	Keg1	1738,887216	15,14393119	500,1776878	10,12142584	31,31398552	-1,797651755 True	1,797651755
ENSMUSG00000053303	Slc22a26	4003,304791	198,1330998	1154,459417	80,97140676	964,209804	-1,793974001 True	1,793974001
ENSMUSG00000026986	Hnmt	308,3626664	25,23988532	88,94004661	14,61983733	48,27572767	-1,793723042 True	1,793723042
ENSMUSG00000001082	Mfsd10	68,78264989	373,5503028	278,2678686	238,4158088	417,5198069	1,793363298 True	1,793363298
ENSMUSG00000035885	Cox8a	2827,817033	2617,376108	4257,674508	816,4616848	3662,431556	-1,792231688 True	1,792231688
ENSMUSG00000028633	Ctps	20,09380783	189,2991399	69,56696715	255,2848519	177,4459179	1,791651441 True	1,791651441
ENSMUSG00000035780	Ugt2a3	3830,188908	519,9416376	714,1621564	93,34203834	1106,427488	-1,791506644 True	1,791506644
ENSMUSG00000030826	Bcat2	415,0144156	119,8894553	825,9976606	887,3116657	148,7414312	-1,791456676 True	1,791456676
ENSMUSG00000027559	Car3	43534,00751	3109,553872	4772,822303	76,47299527	12580,39368	-1,790965764 True	1,790965764
ENSMUSG00000021228	Acot3	1922,822842	307,9266009	378,655644	301,3935696	555,8232429	-1,790527777 True	1,790527777
ENSMUSG00000000711	Rab5b	1296,823444	156,487289	375,1332659	205,8023255	88,72295896	-1,789506969 True	1,789506969
ENSMUSG00000023921	Mut	1908,138905	205,7050654	553,0133591	183,3102681	328,7968479	-1,786779961 True	1,786779961
ENSMUSG00000020733	Slc9a3r1	1871,042645	1937,161199	2451,575146	543,183187	3967,742915	-1,784331711 True	1,784331711
ENSMUSG00000038745	Nlrp6	3111,448859	918,7318257	13124,38074	7285,177402	904,1913318	-1,782886542 True	1,782886542
ENSMUSG00000040415	Dtx3	79,60239257	276,3767443	273,864896	462,2117802	294,8733636	1,782580654 True	1,782580654
ENSMUSG00000031924	Cyb5b	7328,057149	1196,370564	2131,03874	593,7903162	944,6385631	-1,781873934 True	1,781873934
ENSMUSG00000026687	Aldh9a1	5478,65399	6920,776555	2906,842513	1593,562269	13188,4069	-1,781566097 True	1,781566097
ENSMUSG00000020051	Pah	14387,93925	148,9153234	4187,226947	273,2784978	1769,240182	-1,780792954 True	1,780792954
ENSMUSG00000020653	Klf11	142,2023324	164,0592546	41,38794248	73,09918666	446,2242936	-1,780662693 True	1,780662693
ENSMUSG00000009772	Nuak2	134,4739447	119,8894553	295,879759	289,022938	39,1424819	-1,780519527 True	1,780519527
ENSMUSG00000030382	Slc27a5	10564,70589	1778,149921	2745,693716	629,7776081	3077,903826	-1,779232546 True	1,779232546
ENSMUSG00000059248	Sept9	1586,63798	318,0225551	462,3121234	211,4253399	301,3971106	-1,779033888 True	1,779033888
ENSMUSG00000031628	Casp3	468,3402903	128,7234151	136,4921507	98,9650527	135,6939372	-1,778739167 True	1,778739167
ENSMUSG00000041351	Rap1gap	107,424588	64,36170757	316,133433	260,9078662	31,31398552	-1,778445199 True	1,778445199
ENSMUSG00000028179	Cth	5038,908733	51,74176491	1468,831661	175,438048	600,1847224	-1,778442264 True	1,778442264
ENSMUSG00000074272	Ceacam1	3613,021216	912,4218544	1053,191047	588,1673019	799,8113801	-1,778438564 True	1,778438564
ENSMUSG00000029465	Arpc3	891,0830935	1072,695126	793,4156633	259,7832633	1684,431471	-1,778251482 True	1,778251482

ENSMUSG00000069206	Zfp874a	369,4169286	64,36170757	105,6713425	107,9618757	31,31398552	-1,774728033	True	1,774728033
ENSMUSG00000026821	Ralgds	21,63948536	223,3729851	73,96993975	170,9396365	465,7955346	1,773272912	True	1,773272912
ENSMUSG00000024873	Cnih2	2,318516288	302,8786239	7,925350688	52,85633497	794,5923825	1,773272912	True	1,773272912
ENSMUSG00000028167	Bdh2	171,5702053	8,833959863	50,19388769	3,373808615	10,43799517	-1,773215441	True	1,773215441
ENSMUSG00000027709	Mccc1	764,3375364	73,19566744	223,6710083	97,84044983	84,80871077	-1,772831613	True	1,772831613
ENSMUSG00000037344	Slc12a9	209,4393047	155,2252947	821,594688	1186,45603	61,32322164	-1,772026816	True	1,772026816
ENSMUSG00000004798	Ulk2	967,594131	181,7271743	283,5514357	257,5340576	195,7124095	-1,770791557	True	1,770791557
ENSMUSG00000028199	Cryz	881,0361896	128,7234151	243,0440878	114,7094929	258,3403805	-1,769928116	True	1,769928116
ENSMUSG00000071350	Setdb2	149,93072	29,02586812	44,02972604	29,23967466	28,70448672	-1,767746241	True	1,767746241
ENSMUSG00000074794	Arrdc3	1367,151771	73,19566744	401,5511015	147,3229762	207,455154	-1,767517907	True	1,767517907
ENSMUSG00000027569	Mrgbp	111,2887818	542,6575344	376,8944549	693,8799718	446,2242936	1,759852396	True	1,759852396
ENSMUSG00000044037	Als2cl	641,4561731	6680,997645	2169,784899	4510,782118	6077,522689	1,758129426	True	1,758129426
ENSMUSG00000030562	Nox4	202,4837559	20,19190826	59,88042742	28,11507179	39,1424819	-1,757649749	True	1,757649749
ENSMUSG00000046949	Nqo2	1738,887216	100,9595413	514,2672002	35,98729189	206,1504046	-1,757574316	True	1,757574316
ENSMUSG00000022615	Tymp	758,1548263	25,23988532	224,5516028	127,0801245	82,19921198	-1,755445478	True	1,755445478
ENSMUSG00000052595	A1cf	1902,729034	17,66791973	563,5804933	104,5880671	48,27572767	-1,75537654	True	1,75537654
ENSMUSG00000036751	Cox6b1	3255,196869	4203,702901	4690,927013	964,9092638	5340,33928	-1,754279609	True	1,754279609
ENSMUSG00000055301	Adh7	28,59503422	442,9599874	95,98480277	353,1253017	197,0171589	1,74704137	True	1,74704137
ENSMUSG00000038393	Txnip	1189,398856	1553,514942	354,8795919	1430,494853	8893,171887	-1,744831076	True	1,744831076
ENSMUSG00000019872	Smpdl3a	680,0981113	113,579484	108,3131261	132,7031389	203,5409059	-1,740424121	True	1,740424121
ENSMUSG00000022146	Osmr	61,05426226	1024,739344	203,4173343	1713,894776	572,7849851	1,736278705	True	1,736278705
ENSMUSG00000038656	Cyp3a16	512,3920997	0	44,91032056	1,124602872	153,9604288	-1,734688638	True	1,734688638
ENSMUSG00000030207	Fam234b	290,5873748	122,4134438	635,7892441	349,7514931	87,41820957	-1,732966288	True	1,732966288
ENSMUSG00000042082	Arsb	613,6339777	59,31373051	184,9248494	58,47934932	117,4274457	-1,730439268	True	1,730439268
ENSMUSG00000031767	Nudt7	3024,890918	242,3028991	912,2959236	286,7737323	159,1794264	-1,729309342	True	1,729309342
ENSMUSG00000025991	Cps1	37364,43567	1511,869131	11271,60987	5644,381813	9249,368472	-1,72897215	True	1,72897215
ENSMUSG00000002831	Plin4	40,18761567	17252,72361	132,9697726	30465,49179	3393,65318	1,726275433	True	1,726275433
ENSMUSG00000044005	Gls2	5641,722968	642,3550815	1707,472776	1282,047274	572,7849851	-1,724273249	True	1,724273249
ENSMUSG00000026107	Nabp1	112,0616206	129,9854094	171,7159316	155,1951963	33,92348431	-1,723935999	True	1,723935999
ENSMUSG00000021577	Sdha	5045,091444	986,8795161	1527,831494	766,9791584	1170,360209	-1,723394983	True	1,723394983
ENSMUSG00000021957	Tkt	2305,378029	604,4952535	698,311455	221,5467657	687,602932	-1,723060795	True	1,723060795
ENSMUSG00000029016	Clcn6	150,7035587	85,8156101	271,2231124	269,9046892	45,66622888	-1,722513924	True	1,722513924
ENSMUSG00000036368	Rmdn2	962,1842597	193,0851227	235,9993316	292,3967466	155,2651782	-1,71838594	True	1,71838594
ENSMUSG00000043059	Zfp513	139,8838161	1120,650908	471,1180687	1361,894078	457,9670382	1,711014706	True	1,711014706
ENSMUSG00000027397	Slc20a1	224,89608	3235,753298	736,1770194	8661,691317	6570,717961	1,710794201	True	1,710794201
ENSMUSG00000035711	Dok3	47,91600329	431,602039	156,7458247	349,7514931	463,1860358	1,709847532	True	1,709847532
ENSMUSG00000022560	Slc52a2	106,6517493	232,206945	154,9846357	294,6459524	32,61873491	-1,709134894	True	1,709134894
ENSMUSG00000003032	Klf4	11,59258144	288,9966869	37,8655644	105,7126699	395,3390671	1,70768457	True	1,70768457
ENSMUSG00000036390	Gadd45a	88,10361896	297,8306468	175,2383097	26,99046892	882,010592	1,706751239	True	1,706751239
ENSMUSG00000014418	Hps5	221,0318862	137,557375	351,3572138	356,4991103	67,84696862	-1,703898245	True	1,703898245
ENSMUSG00000054630	Ugt2b5	9450,272392	464,4138899	2909,484297	739,9886895	859,8298523	-1,699592454	True	1,699592454
ENSMUSG00000071528	Usmg5	1568,862688	2931,61268	2588,947891	483,5792348	3334,939457	-1,697894888	True	1,697894888
ENSMUSG00000025511	Tspan4	384,8737039	3287,495063	1246,921842	1736,386834	2763,459222	1,695914029	True	1,695914029
ENSMUSG00000086370	Ftx	40,96045443	280,1627271	133,8503672	132,7031389	138,303436	1,695898869	True	1,695898869
ENSMUSG00000052031	Tagap1	111,2887818	887,1819691	360,163159	746,7363068	712,3931705	1,694342443	True	1,694342443
ENSMUSG00000023805	Synj2	28,59503422	195,6091113	92,46242469	109,0864785	473,6240309	1,693102563	True	1,693102563
ENSMUSG00000024292	Cyp4f14	5368,138046	174,1552087	1661,681861	42,73490912	1041,190018	-1,691777576	True	1,691777576
ENSMUSG00000015854	Cd5l	1487,714618	56,78974198	331,1035398	461,0871774	203,5409059	-1,689986357	True	1,689986357
ENSMUSG00000025190	Got1	3031,846466	4268,064608	940,4749483	4556,890836	9120,198282	-1,688735277	True	1,688735277
ENSMUSG00000021947	Cryl1	295,2244074	11,3579484	91,58183017	28,11507179	16,96174215	-1,688678699	True	1,688678699
ENSMUSG00000033107	Rnf125	1256,635828	111,0554954	77,49231784	97,84044983	390,1200696	-1,687576495	True	1,687576495
ENSMUSG00000039952	Dag1	1309,961703	315,4985665	407,7152632	205,8023255	174,8364191	-1,683890763	True	1,683890763
ENSMUSG00000057322	Rpl38	1617,551531	7182,009369	5870,923671	5194,540664	5693,926366	1,683184509	True	1,683184509
ENSMUSG00000000303	Cdh1	463,7032577	23,97789106	144,4175014	64,10236368	61,32322164	-1,682956275	True	1,682956275
ENSMUSG00000089678	Agxt2	1083,519945	665,0709783	1359,63794	2930,715083	337,9300937	-1,680928973	True	1,680928973
ENSMUSG00000041075	Fzd7	312,9996989	45,43179358	97,74599182	23,6166603	70,45646741	-1,67905182	True	1,67905182
ENSMUSG00000018417	Myo1b	2592,874049	595,6612936	811,0275537	648,8958569	480,1477779	-1,676729294	True	1,676729294
ENSMUSG00000031805	Jak3	173,8887216	916,2078372	603,2072468	998,64735	555,8232429	1,676461802	True	1,676461802
ENSMUSG00000001288	Rarg	129,0640734	44,16979931	297,640948	202,4285169	40,44723129	-1,673974609	True	1,673974609
ENSMUSG00000040913	Fbxw4	129,0640734	61,83771904	217,5068467	197,9301054	40,44723129	-1,673974609	True	1,673974609
ENSMUSG00000030278	Cidec	3,864193814	1211,514495	12,32832329	869,3180198	3359,729696	1,673737239	True	1,673737239
ENSMUSG00000039929	Urb1	34,77774433	257,4468303	110,9549096	210,300737	283,130619	1,673737239	True	1,673737239
ENSMUSG00000019960	Dusp6	816,8905723	256,184836	184,0442549	96,71584696	250,5118841	-1,67295775	True	1,67295775
ENSMUSG00000025702	March8	914,2682564	199,395094	287,0738138	170,9396365	217,8931492	-1,671195793	True	1,671195793
ENSMUSG00000030257	Srgap3	255,0367917	20,19190826	80,1341014	5,623014358	37,8377325	-1,670217163	True	1,670217163
ENSMUSG00000031068	Glrx3	875,6263183	201,9190826	260,6559782	224,9205743	275,3021227	-1,669299584	True	1,669299584
ENSMUSG00000025934	Gsta3	21147,95991	604,4952535	6655,533389	220,4221628	2266,349702	-1,6678923	True	1,6678923
ENSMUSG00000039031	Arhgap18	197,8467233	55,52774771	62,52221098	29,23967466	27,39973733	-1,661942469	True	1,661942469
ENSMUSG00000042613	Pbxip1	317,6367315	295,3066583	560,0581153	741,1132924	100,4657035	-1,66067467	True	1,66067467
ENSMUSG00000033526	Ppip5k1	74,19252123	275,11475	234,2381425	985,1521155	464,4907852	1,658630346	True	1,658630346
ENSMUSG00000022094	Slc39a14	1920,504326	13197,93604	6062,012682	15203,50622	35369,14664	1,658311657	True	1,658311657
ENSMUSG00000068762	Gstm6	1538,721977	275,11475	364,5661316	21,36745456	487,9762743	-1,656849673	True	1,656849673
ENSMUSG00000028367	Txn1	2104,439951	2247,611788	2264,889108	668,0141057	3408,005424	-1,655485872	True	1,655485872
ENSMUSG00000092021	Gbp11	353,9601534	508,5836893	704,4756167	112,4602872	284,4353684	-1,654171324	True	1,654171324
ENSMUSG00000060862	Zbtb40	131,3825897	95,91156423	190,2084165	294,6459524	41,75198069	-1,653857563	True	1,653857563
ENSMUSG00000039114	Nrn1	5062,093896	106,0075184	1609,726784	205,8023255	465,7955346	-1,652918425	True	1,652918425
ENSMUSG00000041939	Mvk	378,6909938	106,0075184	1190,563792	96,71584696	14,35224336	1,652551909	True	1,652551909
ENSMUSG00000030378	Sult2a8	6146,386681	633,5211216	1108,668502	56,23014358	1955,819345	-1,651965414	True	1,651965414
ENSMUSG00000022672	Prkdc	110,5159431	128,7234151	158,5070138	210,300737	35,22823371	-1,649450461	True	1,649450461
ENSMUSG00000042102	Dmgdh	7352,787989	949,0196881	2344,142615	428,4736941	1681,821972	-1,649231043	True	1,649231043
ENSMUSG00000047721	Bola2	195,528207	874,5620264	1110,429691	664,6402971	613,2322164	1,649056745	True	1,649056745
ENSMUSG00000035673	Sbno2	442,0637723	6795,839123	1386,055776	4755,945544	9874,343433	1,6486589	True	1,6486589
ENSMUSG00000034647	Ankrd12	117,4714919	684,0008922	368,0885097	1432,744058	592,356226	1,64774203	True	1,64774203
ENSMUSG00000079014	Serpina3i	3,091355051	2226,157885	9,686539729	1825,230461	1145,56997	1,64774203	True	1,64774203
ENSMUSG00000039168	Dap	3693,396447	4105,267348	3311,915993	1179,708412	6262,797103	-1,646517815	True	1,646517815
ENSMUSG00000032946	Rasgrp2	195,528207	131,2474037	285,3126248	468,9593975	62,62797103	-1,642497701	True	1,642497701
ENSMUSG00000002083	Bbc3	357,0515084	18,92991399	114,4772877	20,24285169	41,75198069	-1,641070817	True	1,641070817
ENSMUSG00000049339	Retreg2	1592,82069	333,1664863	510,7448221	497,0744693	263,5593781	-1,640909288	True	1,640909288
ENSMUSG00000026691	Fmo3	7969,513322	17,66791973	2555,4853	217,0483542	170,9221709	-1,640894333	True	1,640894333
ENSMUSG00000025465	Echs1	2374,933518	488,391781	761,7142605	522,9403353	491,8905225	-1,640565318	True	1,640565318
ENSMUSG00000015671	Psma2	672,3697236	1084,053075	537,1626577	215,9237514	1298,22565	-1,638732816	True	1,638732816
ENSMUSG00000024042	Sik1	229,5331126	1268,304237	859,4602524	3265,846739	712,3931705	1,633971393	True	1,633971393
ENSMUSG00000069874	Irgm2	1060,334783	142,6053521	341,6706741	86,59442111	58,71372284	-1,633841513	True	1,633841513
ENSMUSG00000021638	Ocln	322,2737641	34,07384519	103,9101535	19,11824882	40,44723129	-1,632950113	True	1,632950113
ENSMUSG00000054263	Lifr	4817,876847	213,277031	1555,129924	454,3395601	641,9367031	-1,631362401	True	1,631362401
ENSMUSG00000028978	Nos3	30,14071175	849,3221411	93,34301921	1252,807599	444,9195442	1,630828647	True	1,630828647
ENSMUSG00000031095	Cul4b	299,86144	406,3621537	96,86539729	314,8888041	640,6319537	-1,630242717	True	1,630242717
ENSMUSG00000027313	Chac1	57,19006845	548,9675058	18,49248494	219,29756	733,2691608	-1,628825531	True	1,628825531
ENSMUSG00000000171	Sdhd	1570,408366	312,974578	508,1030385	169,8150336	469,7097827	-1,627946767	True	1,627946767
ENSMUSG00000039616	Mocos	918,905289	232,206945	297,640948	96,71584696	194,4076601	-1,626343152	True	1,626343152
ENSMUSG00000022205	Sub1	568,0364907	670,1189553	482,5657974	184,4348709	1724,878702	-1,622872161	True	1,622872161
ENSMUSG00000028518	Prkaa2	666,1870135	104,7455241	216,6262521	86,59442111	163,0936746	-1,620719142	True	1,620719142
ENSMUSG00000076609	Igkc	364,779896	44,16979931	118,8802603	22,49205743	71,76121681	-1,617517041	True	1,617517041
ENSMUSG00000024955	Esrra	231,8516288	177,9411915	502,8194714	389,1125936	75,675465	-1,615304322	True	1,615304322
ENSMUSG00000045211	Nudt18	99,6962004	118,627461	199,0143617	730,9918666	32,61873491	-1,611837692	True	1,611837692
ENSMUSG00000062070	Pgk1	1820,808125	528,7755975	596,1624906	265,4062777	524,5092574	-1,610801389	True	1,610801389
ENSMUSG00000020458	Rtn4	1083,519945	228,4209622	328,4617563	233,9173973	354,8918359	-1,61027442	True	1,61027442
ENSMUSG00000004815	Dgkq	414,2415769	507,321695	567,983466	1291,044097	135,6939372	-1,610116099	True	1,610116099
ENSMUSG00000004263	Atn1	61,82710102	949,0196881	188,4472275	681,5093402	408,3865611	1,607849303	True	1,607849303
ENSMUSG00000027184	Caprin1	3516,416371	508,5836893	1154,459417	819,8354934	609,3179682	-1,606888448	True	1,606888448
ENSMUSG00000037253	Mex3c	150,7035587	150,1773177	61,64161646	49,48252635	394,0343177	-1,606722421	True	1,606722421
ENSMUSG00000073834	Mup11	23,95800165	224,6349794	59,88042742	7,872220101	1553,956531	-1,605665114	True	1,605665114
ENSMUSG00000043467	Zbtb37	119,0171695	109,7935012	127,6862055	218,1729571	39,1424819	-1,604362572	True	1,604362572

ENSMUSG00000057388	Mrpl18	484,5699043	4989,925328	1473,234633	3221,987227	3174,455282	1,604210509	True	1,604210509
ENSMUSG00000022110	Sucla2	1328,509833	273,8527558	318,7752165	200,1793111	437,0910478	-1,603803171	True	1,603803171
ENSMUSG00000014873	Surf2	186,2541418	261,2328131	457,0285563	272,1538949	61,32322164	-1,602767111	True	1,602767111
ENSMUSG00000006932	Ctnnb1	2207,227507	726,9086973	648,1175674	502,6974836	273,9973733	-1,602389269	True	1,602389269
ENSMUSG00000044783	Hjurp	1429,751711	471,9858555	2296,59051	4669,351123	712,3931705	-1,598949102	True	1,598949102
ENSMUSG00000004558	Ndrg2	22246,16379	1356,643836	7345,919493	1508,092451	1792,725671	-1,598541584	True	1,598541584
ENSMUSG00000052085	Dock8	469,113129	26,50187959	154,9846357	33,73808615	28,70448672	-1,597810676	True	1,597810676
ENSMUSG00000006494	Pdk1	1608,277465	242,3028991	531,8790906	301,3935696	281,8258696	-1,596346099	True	1,596346099
ENSMUSG00000029217	Tec	332,320668	392,4802168	110,0743151	419,4768711	671,9459392	-1,594098154	True	1,594098154
ENSMUSG00000021660	Btf3	1398,065322	1404,599618	1190,563792	463,3363831	2760,849723	-1,593299891	True	1,593299891
ENSMUSG00000066687	Zbtb16	146,0665262	885,9199748	48,43269865	708,4998091	705,8694235	-1,592572299	True	1,592572299
ENSMUSG00000061898	Rbak	34,77774433	323,0705321	104,790748	431,8475027	180,0554167	1,591275078	True	1,591275078
ENSMUSG00000071757	Zhx2	233,3973064	70,6716789	77,49231784	41,61030625	43,05673008	-1,590662709	True	1,590662709
ENSMUSG00000041920	Slc16a6	116,6986532	1312,474037	351,3572138	472,3332061	1296,9209	1,590150608	True	1,590150608
ENSMUSG00000027285	Haus2	142,2023324	128,7234151	81,89529044	47,23332061	314,4446046	-1,590068259	True	1,590068259
ENSMUSG00000030787	Lyve1	71,10116618	2889,966869	213,9844686	4356,711525	1981,914333	1,589560959	True	1,589560959
ENSMUSG00000039270	Megf9	384,1008651	95,91156423	127,6862055	69,72537804	96,55145534	-1,58888254	True	1,58888254
ENSMUSG00000022821	Hgd	7293,279405	473,2478498	2425,15731	1149,344135	600,1847224	-1,588489332	True	1,588489332
ENSMUSG00000040857	Erf	307,5898276	407,624148	115,3578822	102,3388613	708,4789223	-1,587653706	True	1,587653706
ENSMUSG00000023249	Parp3	728,0141146	826,6062443	1398,384099	2036,655801	242,6833878	-1,584891065	True	1,584891065
ENSMUSG00000040370	Etfrf1	652,2759158	150,1773177	217,5068467	160,8182106	126,5606915	-1,584421546	True	1,584421546
ENSMUSG00000070002	Ell	551,8068766	2513,892578	1653,75651	2128,873236	1880,14388	1,583511495	True	1,583511495
ENSMUSG00000021360	Gcnt2	287,4960198	878,3480092	95,98480277	143,9491676	1461,319324	-1,582664075	True	1,582664075
ENSMUSG00000002032	Tmem25	664,641336	11,3579484	1990,143617	178,8118566	6,523746983	-1,582224619	True	1,582224619
ENSMUSG00000053897	Slc39a8	527,0760362	150,1773177	176,1189042	109,0864785	152,6556794	-1,581461328	True	1,581461328
ENSMUSG00000026102	Inpp1	152,2492363	121,1514495	155,8652302	318,2626127	50,88522646	-1,581116227	True	1,581116227
ENSMUSG00000024601	Isoc1	1165,440854	113,579484	390,1033727	145,0737704	177,4459179	-1,578947413	True	1,578947413
ENSMUSG00000042642	Flad1	553,3525542	508,5836893	900,8481948	856,9473882	185,2744143	-1,578535285	True	1,578535285
ENSMUSG00000020282	Rhbdf1	74,19252123	604,4952535	221,0292247	705,1260005	454,05279	1,574891465	True	1,574891465
ENSMUSG00000010651	Acaa1b	8626,42627	1322,569991	2897,155974	2516,861227	2267,654451	-1,574125651	True	1,574125651
ENSMUSG00000026542	Apcs	2167,81273	6889,226699	13278,48478	6450,722072	8221,225947	1,573220528	True	1,573220528
ENSMUSG00000059851	Kmt5c	357,0515084	334,4284805	704,4756167	822,0846992	120,0369445	-1,572653712	True	1,572653712
ENSMUSG00000033715	Akr1c14	3120,722924	13,88193693	1049,668669	60,72855507	62,62797103	-1,571946264	True	1,571946264
ENSMUSG00000026024	Als2	1101,295237	242,3028991	370,7302933	321,6364213	236,1596408	-1,57075937	True	1,57075937
ENSMUSG00000027513	Pck1	14476,8157	4339,998281	4875,851862	3535,751428	1720,964454	-1,570018105	True	1,570018105
ENSMUSG00000029250	Polr2b	499,2538408	118,627461	113,5966932	94,46664122	168,3126721	-1,568629726	True	1,568629726
ENSMUSG00000015090	Ptgds	206,3479497	10,09595413	69,56696715	0	20,87599034	-1,568604772	True	1,568604772
ENSMUSG00000071076	Jund	930,4978704	1042,407264	595,2818961	313,7642012	2518,166335	-1,568322091	True	1,568322091
ENSMUSG00000028249	Sdcbp	639,9104956	633,5211216	283,5514357	215,9237514	1745,754693	-1,567348182	True	1,567348182
ENSMUSG00000020130	Tbc1d15	547,9426828	581,7793567	184,9248494	789,4712159	1505,680804	-1,567085888	True	1,567085888
ENSMUSG00000069805	Fbp1	10914,02901	3690,071234	3528,542245	2692,299275	3394,95793	-1,564463211	True	1,564463211
ENSMUSG00000036461	Elf1	389,5107365	124,9374323	95,10420825	47,23332061	131,779689	-1,563535068	True	1,563535068
ENSMUSG00000020611	Gna13	312,2268602	348,3104175	105,6713425	129,3293302	763,278397	-1,563010476	True	1,563010476
ENSMUSG00000002778	Kdelr1	1561,134301	2158,010195	1821,069469	528,5633497	3424,967166	-1,562446357	True	1,562446357
ENSMUSG00000047822	Angptl8	1588,183658	213,277031	4673,315122	185,5594738	169,6174215	1,557068566	True	1,557068566
ENSMUSG00000073565	Prr16	831,5745088	88,33959863	282,6708412	244,0388231	97,85620474	-1,556722464	True	1,556722464
ENSMUSG00000025269	Apex2	44,05180948	155,2252947	129,4473946	147,3229762	161,7889252	1,555092742	True	1,555092742
ENSMUSG00000028525	Pde4b	432,0168684	605,7572477	147,059285	418,3522682	1399,996102	-1,554689769	True	1,554689769
ENSMUSG00000024369	Nelfe	340,8218944	371,0263142	373,3720768	985,1521155	116,1226963	-1,553368041	True	1,553368041
ENSMUSG00000061207	Stk19	103,5603942	2863,46499	303,8051097	1252,807599	3340,158455	1,552673773	True	1,552673773
ENSMUSG00000045975	C2cd2	2275,237318	179,2031858	775,8037729	386,8633878	131,779689	-1,552253336	True	1,552253336
ENSMUSG00000010025	Aldh3a2	5388,231854	415,1961136	1839,561954	657,8926799	527,1187562	-1,550449666	True	1,550449666
ENSMUSG00000090877	Hspa1b	26,27651794	40,38381652	151,4622576	8,996822973	683,6886838	-1,546286567	True	1,546286567
ENSMUSG00000040147	Maob	4234,383581	258,7088246	1450,339176	140,575359	668,031691	-1,545761637	True	1,545761637
ENSMUSG00000018427	Ypel2	456,7477088	88,33959863	65,16399454	26,99046892	156,5699276	-1,544590351	True	1,544590351
ENSMUSG00000041237	Pklr	4714,316453	1077,743103	1618,532729	353,1253017	701,9551753	-1,542362065	True	1,542362065
ENSMUSG00000037887	Dusp8	6,955548865	228,4209622	20,25367398	89,96822973	366,6345804	1,541947366	True	1,541947366
ENSMUSG00000039989	Cbx4	307,5898276	157,7492833	558,2969262	430,7228998	105,6847011	-1,541241244	True	1,541241244
ENSMUSG00000022844	Pdia5	877,1719958	461,8899014	1825,472442	1070,621934	301,3971106	-1,541194168	True	1,541194168
ENSMUSG00000042541	Sem1	421,9699645	518,6796434	613,774381	145,0737704	654,984197	-1,540353611	True	1,540353611
ENSMUSG00000025971	Maip1	271,2664057	230,9449507	185,8054439	93,34203834	622,3654621	-1,539111515	True	1,539111515
ENSMUSG00000056209	Npm3	143,7480099	217,0630138	165,5517699	49,48252635	198,3219083	-1,538550918	True	1,538550918
ENSMUSG00000029389	Ddx55	87,3307802	253,6608475	278,2678686	374,4927562	354,8918359	1,538338723	True	1,538338723
ENSMUSG00000072082	Ccnf	147,6122037	68,14769037	109,1937206	399,2340194	50,88522646	-1,536493236	True	1,536493236
ENSMUSG00000041235	Chd7	168,4788503	1456,341383	488,7299591	3789,911677	1530,471042	1,536470048	True	1,536470048
ENSMUSG00000024414	Mrpl27	329,229313	392,4802168	475,5210413	113,58489	486,6715249	-1,535321863	True	1,535321863
ENSMUSG00000051615	Rap2a	231,8516288	70,6716789	80,1341014	59,6039522	39,1424819	-1,532713639	True	1,532713639
ENSMUSG00000032420	Nt5e	947,5003232	243,5648934	327,5811618	154,0705934	315,749354	-1,532274041	True	1,532274041
ENSMUSG00000101609	Kcnq1ot1	136,792461	1350,333865	1271,578488	6721,751364	395,3390671	1,531101807	True	1,531101807
ENSMUSG00000031173	Otc	8331,974702	1328,879962	2372,321639	203,5531198	2883,496166	-1,530839355	True	1,530839355
ENSMUSG00000022000	Zc3h13	208,666466	2672,903856	602,3266523	2331,301753	1303,444647	1,529347329	True	1,529347329
ENSMUSG00000013033	Adgrl1	86,55794143	65,62370184	312,6110549	307,016584	30,00923612	-1,528259593	True	1,528259593
ENSMUSG00000060591	Ifitm2	1442,117131	8656,018671	4634,568963	4158,781419	11834,07703	1,527972513	True	1,527972513
ENSMUSG00000001323	Srr	2432,123587	118,627461	843,609551	348,6268902	76,98021439	-1,527569206	True	1,527569206
ENSMUSG00000021335	Slc17a1	248,0812429	320,5465436	197,2531727	391,3617993	86,11346017	-1,526501995	True	1,526501995
ENSMUSG00000029263	Pigg	180,0714317	25,23988532	62,52221098	35,98729189	54,79947465	-1,526128614	True	1,526128614
ENSMUSG00000027287	Snap23	401,1033179	78,2436445	126,805611	139,4507561	118,7321951	-1,524218144	True	1,524218144
ENSMUSG00000039853	Trim14	303,7256338	11,3579484	105,6713425	38,23649764	26,09498793	-1,523184495	True	1,523184495
ENSMUSG00000035031	C8a	2619,150567	474,5098441	7524,680181	1005,394967	720,2216669	1,522531266	True	1,522531266
ENSMUSG00000040127	Sdr9c7	1344,739447	3,785982798	468,4762851	16,86904307	2,609498793	-1,521278744	True	1,521278744
ENSMUSG00000058818	Pirb	142,2023324	180,4651801	109,1937206	345,2530816	49,58047707	-1,520101069	True	1,520101069
ENSMUSG00000030861	Acadsb	1782,166187	237,254922	622,5803262	248,5372346	323,5778503	-1,517299981	True	1,517299981
ENSMUSG00000038894	Irs2	607,4512676	1532,061039	212,2232795	390,2371965	2010,61882	-1,517185755	True	1,517185755
ENSMUSG00000032715	Trib3	78,05671504	159,0112775	27,29843015	300,2689667	181,3601661	-1,515704761	True	1,515704761
ENSMUSG00000059714	Flot1	290,5873748	1755,434024	830,4006332	1150,468738	1291,701903	1,51483552	True	1,51483552
ENSMUSG00000011382	Dhdh	3004,024271	176,6791973	1051,429858	255,2848519	93,94195655	-1,51454386	True	1,51454386
ENSMUSG00000028550	Atg4c	168,4788503	6,309971331	58,9998329	19,11824882	5,218997586	-1,513784723	True	1,513784723
ENSMUSG00000015806	Qdpr	4706,588065	377,3362856	1651,114727	435,2213113	785,4591367	-1,511241221	True	1,511241221
ENSMUSG00000030498	Gas2	376,3724775	97,17355849	132,0891781	77,59759814	95,24670595	-1,51064886	True	1,51064886
ENSMUSG00000109198	D7Bwg0826e	1,545677526	407,624148	4,402972604	40,48570338	982,4762956	1,510238506	True	1,510238506
ENSMUSG00000032010	Usp2	33,2320668	94,64956996	482,5657974	110,2110814	207,455154	1,51001993	True	1,51001993
ENSMUSG00000060733	Ipmk	813,7992172	195,6091113	204,2979288	168,6904307	285,7401178	-1,509969288	True	1,509969288
ENSMUSG00000024863	Mbl2	7950,965192	637,3071044	2792,365226	132,7031389	2772,592468	-1,509642354	True	1,509642354
ENSMUSG00000028494	Plin2	5039,681572	26012,22581	1769,994987	13997,93194	32720,50537	-1,509587307	True	1,509587307
ENSMUSG00000034936	Arl4d	415,7872544	68,14769037	146,1786905	140,575359	48,27572767	-1,50811252	True	1,50811252
ENSMUSG00000030465	Psd3	555,6710705	109,7935012	195,4919836	50,60712922	126,5606915	-1,507121683	True	1,507121683
ENSMUSG00000078974	Sec61g	495,389647	1828,629692	1605,323812	1408,002795	1534,38529	1,507014575	True	1,507014575
ENSMUSG00000020483	Dynll2	1461,4381	272,5907615	514,2672002	202,4285169	331,4063467	-1,506798678	True	1,506798678
ENSMUSG00000033909	Usp36	186,2541418	832,9162157	529,237307	2480,873935	1136,436724	1,506642256	True	1,506642256
ENSMUSG00000028517	Plpp3	2402,755714	685,2628865	847,1319291	625,2791966	571,4802357	-1,504031406	True	1,504031406
ENSMUSG00000032311	Nrg4	71,10116618	1497,987194	201,6561453	1880,336001	532,3377538	1,503952243	True	1,503952243
ENSMUSG00000050270	Tmem220	229,5331126	10,09595413	81,01469592	7,872220101	28,70448672	-1,502446753	True	1,502446753
ENSMUSG00000054387	Mdm4	622,9080428	314,2365723	1446,816798	1716,143982	220,502648	-1,498223219	True	1,498223219
ENSMUSG00000038084	Opa1	1193,26305	422,7680792	361,0437536	319,3872155	373,1583274	-1,496973758	True	1,496973758
ENSMUSG00000000751	Rpa1	305,2713113	95,91156423	108,3131261	106,8372728	106,9894505	-1,494883926	True	1,494883926
ENSMUSG00000020593	Lpin1	1084,292784	2426,814974	384,8198056	8033,038312	2460,757362	-1,494499413	True	1,494499413
ENSMUSG00000000708	Kat2b	969,9126473	297,8306468	344,3124577	314,8888041	156,5699276	-1,49413644	True	1,49413644
ENSMUSG00000003500	Impdh1	40,96045443	171,6312202	115,3578822	167,5658279	386,2058214	1,493812958	True	1,493812958
ENSMUSG00000035828	Pim3	441,2909336	2698,143741	1242,518869	2458,381877	2235,035716	1,493465749	True	1,493465749
ENSMUSG00000022307	Oxr1	691,6906927	152,7013062	245,6858713	154,0705934	177,4459179	-1,493312149	True	1,493312149
ENSMUSG00000008384	Sertad1	39,4147769	392,4802168	110,9549096	192,307091	227,026395	1,493164993	True	1,493164993
ENSMUSG00000031959	Wdr59	157,6591076	181,7271743	168,1935535	405,9816367	56,10422405	-1,490627214	True	1,490627214
ENSMUSG00000001755	Ppat	1509,354104	383,6462569	537,1626577	220,4221628	233,550142	-1,49050039	True	1,49050039

ENSMUSG00000005362	Crbn	271,2664057	53,00375918	53,71626577	95,59124409	96,55145534	-1,490340481	True	1,490340481
ENSMUSG00000021906	Oxnad1	333,8663455	37,85982798	118,8802603	65,22696655	24,79023853	-1,489761494	True	1,489761494
ENSMUSG00000040482	Dxo	324,5922804	1313,736031	911,4153291	1229,190939	1333,453883	1,489479946	True	1,489479946
ENSMUSG00000054619	Mettl7a1	2809,268903	117,3654668	1001,23597	149,5721819	233,550142	-1,488412699	True	1,488412699
ENSMUSG00000024084	Qpct	193,9825295	17,66791973	32,58199727	19,11824882	69,15171802	-1,488089726	True	1,488089726
ENSMUSG00000037370	Enpp1	609,7697839	108,5315069	217,5068467	190,0578853	69,15171802	-1,487203847	True	1,487203847
ENSMUSG00000021338	Carmil1	229,5331126	417,7201021	81,89529044	223,7959715	219,1978986	-1,486849898	True	1,486849898
ENSMUSG00000001065	Zfp276	255,8096305	798,8423705	728,2516687	931,1711777	715,0026693	1,482878049	True	1,482878049
ENSMUSG00000001123	Lgals9	7878,318348	1492,939217	2818,783061	877,1902399	1987,133331	-1,482815265	True	1,482815265
ENSMUSG00000057315	Arhgap24	147,6122037	26,50187959	52,83567125	37,11189476	15,65699276	-1,482227821	True	1,482227821
ENSMUSG00000052752	Traf7	234,9429839	1641,85454	656,042918	2670,93182	1972,781088	1,481479513	True	1,481479513
ENSMUSG00000026678	Rgs5	648,411722	104,7455241	143,5369069	87,71902399	232,2453926	-1,481260196	True	1,481260196
ENSMUSG00000054469	Lclat1	604,3599125	109,7935012	216,6262521	125,9555216	109,5989493	-1,480199881	True	1,480199881
ENSMUSG00000078440	Dohh	46,37032577	182,9891686	156,7458247	129,3293302	241,3786384	1,47977573	True	1,47977573
ENSMUSG00000044206	Vsig4	350,8687983	26,50187959	125,9250165	61,85315794	73,0659662	-1,478366739	True	1,478366739
ENSMUSG00000030788	Rnf141	647,6388832	106,0075184	232,4769535	76,47299527	130,4749397	-1,478101903	True	1,478101903
ENSMUSG00000019923	Zwint	723,377082	1056,289201	350,4766193	259,7832633	1876,229632	-1,477439404	True	1,477439404
ENSMUSG00000029269	Sult1b1	472,9773228	49,21777638	169,9547425	41,61030625	69,15171802	-1,476620394	True	1,476620394
ENSMUSG00000022108	Itm2b	14908,83257	2576,992291	5360,178848	1261,804422	4841,92501	-1,475814249	True	1,475814249
ENSMUSG00000025538	Sumf2	94,28632906	82,0296273	159,3876083	148,4475791	33,92348431	-1,474764246	True	1,474764246
ENSMUSG00000031447	Lamp1	7422,343478	1645,640523	1664,323644	1024,513216	2672,126764	-1,473886315	True	1,473886315
ENSMUSG00000036309	Skp1a	1617,551531	285,2107041	582,9535728	205,8023255	551,9089947	-1,472358778	True	1,472358778
ENSMUSG00000027605	Acss2	8274,784633	90,86358716	2984,334831	251,9110432	163,0936746	-1,471312354	True	1,471312354
ENSMUSG00000027710	Acad9	372,5082837	76,98165023	87,17885756	70,84998091	134,3891878	-1,470855436	True	1,470855436
ENSMUSG00000021238	Aldh6a1	7004,237707	820,296273	1653,75651	2526,982653	1478,281066	-1,470812286	True	1,470812286
ENSMUSG00000050627	Gpd1l	529,3945525	25,23988532	191,089011	55,10554071	31,31398552	-1,470098534	True	1,470098534
ENSMUSG00000036078	Sigmar1	3171,730283	301,6166296	1145,653472	250,7864404	114,8179469	-1,469099358	True	1,469099358
ENSMUSG00000026471	Mr1	158,4319464	11,3579484	57,23864386	34,86268902	13,04749397	-1,468801875	True	1,468801875
ENSMUSG00000058761	Rnf169	465,2489352	713,0267604	710,6397783	1924,195513	168,3126721	-1,466859048	True	1,466859048
ENSMUSG00000060183	Cxcl11	108,9702656	26,50187959	301,1633261	67,4761723	16,96174215	1,466611574	True	1,466611574
ENSMUSG00000014039	Prdm15	56,41722968	280,1627271	155,8652302	459,9625745	200,9314071	1,466091403	True	1,466091403
ENSMUSG00000055782	Abcd2	836,2115414	68,14769037	302,9245152	156,3197992	31,31398552	-1,464909618	True	1,464909618
ENSMUSG00000031310	Zmym3	258,9009855	126,1994266	460,5509344	570,1736559	93,94195655	-1,462558911	True	1,462558911
ENSMUSG00000032782	Cntrob	132,9282672	65,62370184	250,9694384	185,5594738	48,27572767	-1,461278016	True	1,461278016
ENSMUSG00000032328	Tmem30a	2336,29158	591,8753108	849,7737126	598,2887277	581,9182308	-1,45906972	True	1,45906972
ENSMUSG00000029068	Ccnl2	1507,808426	10383,68882	4144,95841	13532,34635	7647,136213	1,458904486	True	1,458904486
ENSMUSG00000003053	Cyp2c29	64242,22216	1041,14527	23380,66512	233,9173973	3554,137356	-1,458205825	True	1,458205825
ENSMUSG00000035000	Dpp4	2147,718922	352,0964003	781,9679345	488,0776463	298,7876118	-1,457623841	True	1,457623841
ENSMUSG00000047090	Tmem198b	78,82955381	58,05173624	127,6862055	260,9078662	28,70448672	-1,45746035	True	1,45746035
ENSMUSG00000023905	Tnfrsf12a	41,73329319	2479,818733	114,4772877	441,9689285	1638,765242	1,455790722	True	1,455790722
ENSMUSG00000040028	Elavl1	418,8786094	378,5982798	425,3271536	152,9459905	750,230903	-1,453509926	True	1,453509926
ENSMUSG00000028013	Ppa2	583,4932659	151,4393119	213,103874	138,3261532	123,9511927	-1,453159186	True	1,453159186
ENSMUSG00000005615	Pcyt1a	935,9077418	136,2953807	246,5664658	101,2142584	341,8443419	-1,453026775	True	1,453026775
ENSMUSG00000020988	L2hgdh	534,8044239	47,95578211	195,4919836	56,23014358	105,6847011	-1,45190195	True	1,45190195
ENSMUSG00000039395	Mreg	588,9031373	59,31373051	170,835337	21,36745456	215,2836504	-1,4517916	True	1,4517916
ENSMUSG00000027487	Cdk5rap1	139,1109773	199,395094	157,6264192	151,8213877	50,88522646	-1,450917504	True	1,450917504
ENSMUSG00000020455	Trim11	286,723181	1102,982989	782,848529	1415,875015	898,9723342	1,449074644	True	1,449074644
ENSMUSG00000030236	Slco1b2	10512,15285	42,90780505	3857,004001	418,3522682	225,7216456	-1,446505611	True	1,446505611
ENSMUSG00000026918	Brd3	615,1796552	596,9232879	1069,922343	1243,810776	225,7216456	-1,44646302	True	1,44646302
ENSMUSG00000037710	Cisd1	1303,006154	170,3692259	478,1628248	182,1856652	230,9406432	-1,446270022	True	1,446270022
ENSMUSG00000037580	Gch1	1492,351651	470,7238613	547,729792	245,163426	416,2150575	-1,446051267	True	1,446051267
ENSMUSG00000025348	Itga7	91,96781277	493,4397581	302,0439207	1225,81713	250,5118841	1,445678111	True	1,445678111
ENSMUSG00000005981	Trap1	2708,027025	696,6208349	995,0718086	594,9149191	784,1543873	-1,444369591	True	1,444369591
ENSMUSG00000058793	Cds2	1390,336934	242,3028991	511,6254166	262,0324691	217,8931492	-1,442274706	True	1,442274706
ENSMUSG00000038838	Vars2	340,0490556	384,9082512	393,6257508	770,3529671	125,2559421	-1,44086384	True	1,44086384
ENSMUSG00000020196	Cabin1	250,3997591	1342,761899	678,0577811	2138,994662	2104,560777	1,437175044	True	1,437175044
ENSMUSG00000032349	Elovl5	8523,638715	1046,193247	1836,920171	927,7973691	3148,360294	-1,436868794	True	1,436868794
ENSMUSG00000038037	Socs1	6,182710102	1321,307997	16,7312959	49,48252635	1127,303479	1,436237925	True	1,436237925
ENSMUSG00000049470	Aff4	726,468437	667,5949668	268,5813289	535,3109669	1698,783714	-1,435541103	True	1,435541103
ENSMUSG00000003873	Bax	122,1085245	424,0300734	346,9542412	395,8602108	330,1015973	1,4347462	True	1,4347462
ENSMUSG00000038412	Higd1a	294,4515686	340,7384519	334,6259179	109,0864785	662,8126934	-1,432558071	True	1,432558071
ENSMUSG00000001173	Ocrl	126,7455571	122,4134438	176,9994987	440,8443257	46,97097827	-1,43209363	True	1,43209363
ENSMUSG00000031886	Ces2e	2789,947934	704,1928005	551,2521701	256,4094547	1034,666271	-1,431072693	True	1,431072693
ENSMUSG00000028553	Angptl3	14377,89234	1649,426506	5332,880418	273,2784978	4621,422362	-1,430865323	True	1,430865323
ENSMUSG00000024781	Lipa	5194,249325	861,9420838	1927,621406	873,8164312	712,3931705	-1,430093538	True	1,430093538
ENSMUSG00000037353	Letmd1	393,3749303	427,8160562	539,8044413	663,5156943	146,1319324	-1,428633551	True	1,428633551
ENSMUSG00000040713	Creg1	7056,790743	1313,736031	2623,291078	981,7783069	2150,227005	-1,427634334	True	1,427634334
ENSMUSG00000062980	Cped1	649,1845608	1007,071424	538,0432522	836,7045365	241,3786384	-1,427330684	True	1,427330684
ENSMUSG00000028862	Map3k6	19,32096907	1943,47117	51,95507673	1340,526623	1899,715121	1,427097271	True	1,427097271
ENSMUSG00000046229	Scand1	227,987435	1404,599618	612,8937865	630,902211	1077,723002	1,426682763	True	1,426682763
ENSMUSG00000040451	Sgms1	191,6640132	1383,145716	71,32815619	417,2276654	2649,946024	-1,426035896	True	1,426035896
ENSMUSG00000025002	Cyp2c55	241,125694	3,785982798	89,82064113	6,74761723	13,04749397	-1,424666466	True	1,424666466
ENSMUSG00000033965	Slc16a2	2578,962951	396,2661996	960,7286223	390,2371965	520,5950092	-1,424590174	True	1,424590174
ENSMUSG00000036292	Gramd1c	693,2363702	388,694234	1839,561954	1009,893379	258,3403805	-1,424074182	True	1,424074182
ENSMUSG00000027589	Pcmtd2	552,5797154	109,7935012	206,0591179	177,6872537	90,02770836	-1,423124301	True	1,423124301
ENSMUSG00000029207	Apbb2	87,3307802	99,69754702	128,5668	169,8150336	32,61873491	-1,420789399	True	1,420789399
ENSMUSG00000027761	Aadac	6297,863078	1171,130679	2353,829154	578,045876	1920,591112	-1,419852782	True	1,419852782
ENSMUSG00000057666	Gapdh	12859,26417	34807,06385	19508,69102	4811,051085	34685,45796	-1,418384068	True	1,418384068
ENSMUSG00000090258	Churc1	49,46168082	169,1072317	132,0891781	148,4475791	136,9986866	1,417129102	True	1,417129102
ENSMUSG00000040848	Sft2d2	663,0956585	224,6349794	248,3276549	178,8118566	233,550142	-1,416972074	True	1,416972074
ENSMUSG00000027349	Fam98b	183,1627868	150,1773177	138,2533398	68,60077517	377,0725756	-1,416829637	True	1,416829637
ENSMUSG00000035686	Thrsp	5596,89832	2097,43447	1664,323644	136,0769475	1657,031734	-1,416001801	True	1,416001801
ENSMUSG00000028228	Cpne3	448,2464824	66,88569611	168,1935535	56,23014358	73,0659662	-1,414169851	True	1,414169851
ENSMUSG00000030560	Ctsc	2633,834504	989,4035047	641,9534057	539,8093784	812,858874	-1,412533782	True	1,412533782
ENSMUSG00000033308	Dpyd	5360,409659	646,1410643	1867,740979	554,4292157	2014,533068	-1,411897773	True	1,411897773
ENSMUSG00000021109	Hif1a	515,4834548	933,8757569	193,7307946	600,5379334	2010,61882	-1,411872826	True	1,411872826
ENSMUSG00000063382	Bcl9l	867,8979306	1733,980122	1329,697726	2266,074786	326,1873491	-1,411824555	True	1,411824555
ENSMUSG00000024854	Pold4	94,28632906	398,7901881	376,0138604	250,7864404	255,7308817	1,411338837	True	1,411338837
ENSMUSG00000023070	Rgn	15585,83933	3250,89723	5725,625575	137,2015503	5862,239039	-1,410712148	True	1,410712148
ENSMUSG00000026398	Nr5a2	1010,873102	256,184836	380,416833	347,5022873	182,6649155	-1,409948912	True	1,409948912
ENSMUSG00000029265	Dr1	244,9898878	205,7050654	132,9697726	92,21743547	593,6609754	-1,409610751	True	1,409610751
ENSMUSG00000031604	Msmo1	3359,530102	1699,906276	8923,06428	1189,829838	550,6042453	1,409279776	True	1,409279776
ENSMUSG00000021040	Slirp	232,6244676	251,136859	485,207581	87,71902399	276,6068721	-1,407041184	True	1,407041184
ENSMUSG00000058230	Arhgap35	1559,588623	345,7864289	589,1177345	314,8888041	401,8628141	-1,404537647	True	1,404537647
ENSMUSG00000078675	Mup16	391,056414	111,0554954	54,59686029	12,37063159	1034,666271	1,403716854	True	1,403716854
ENSMUSG00000059824	Dbp	48,68884206	128,7234151	474,6404467	283,3999236	177,4459179	1,402611412	True	1,402611412
ENSMUSG00000025396	Hsd17b6	3291,520291	184,2511629	1245,160652	55,10554071	328,7968479	-1,402422198	True	1,402422198
ENSMUSG00000019362	D8Ertd738e	669,2783686	2014,142849	1804,338173	1767,875714	2143,703258	1,401338562	True	1,401338562
ENSMUSG00000042249	Grk3	37,09626061	102,2215356	193,7307946	610,6593593	97,85620474	1,399389563	True	1,399389563
ENSMUSG00000078652	Psme3	850,1226391	911,1598602	575,0282221	322,7610242	1671,383977	-1,397204607	True	1,397204607
ENSMUSG00000029385	Ccng2	245,7627266	65,62370184	36,98496988	93,34203834	70,45646741	-1,39666725	True	1,39666725
ENSMUSG00000000056	Narf	512,3920997	65,62370184	194,6113891	80,97140676	44,36147948	-1,396652089	True	1,396652089
ENSMUSG00000028195	Cyr61	206,3479497	26,50187959	78,37291236	12,37063159	66,54221922	-1,396652089	True	1,396652089
ENSMUSG00000050174	Nudt6	116,6986532	123,6754381	156,7458247	143,9491676	44,36147948	-1,395408525	True	1,395408525
ENSMUSG00000024948	Map4k2	267,4022119	273,8527558	585,5953564	717,4966321	101,7704529	-1,393692636	True	1,393692636
ENSMUSG00000024425	Ndfip1	2558,096305	641,0930872	973,9375401	517,3173209	615,8417152	-1,39316942	True	1,39316942
ENSMUSG00000022820	Ndufb4	510,8464222	628,4731445	950,161488	194,5562968	610,6227176	-1,392701959	True	1,392701959
ENSMUSG00000022003	Slc25a30	228,7602738	690,3108636	87,17885756	378,9911677	198,3219083	-1,391786334	True	1,391786334
ENSMUSG00000027601	Mtfr1	802,9794746	199,395094	306,4468933	173,1888422	250,5118841	-1,389726032	True	1,389726032
ENSMUSG00000001119	Col6a1	119,0171695	45,43179358	257,1336001	141,6999618	53,49472526	-1,389395545	True	1,389395545
ENSMUSG00000030060	Hmces	62,59993979	408,8861422	163,7905809	360,9975218	216,5883998	1,38761922	True	1,38761922
ENSMUSG00000021665	Hexb	303,7256338	56,78974198	116,2384768	46,10871774	87,41820957	-1,385680972	True	1,385680972
ENSMUSG00000001473	Tubb6	47,91600329	774,8644794	125,044422	173,1888422	2469,890608	1,38386122	True	1,38386122

ENSMUSG00000027405	Nop56	233,3973064	782,436445	608,4908139	623,0299909	621,0607127	1,382447572	True	1,382447572
ENSMUSG00000029580	Actb	7611,688975	44953,49775	19838,03337	31180,73922	28447,45109	1,381980495	True	1,381980495
ENSMUSG00000015451	C4a	48,68884206	172,8932145	126,805611	134,9523446	392,7295683	1,380955489	True	1,380955489
ENSMUSG00000038473	Nos1ap	27,0493567	432,8640333	70,44756167	499,323675	540,1662502	1,380955489	True	1,380955489
ENSMUSG00000022019	Tdrd3	139,1109773	242,3028991	141,7757179	173,1888422	53,49472526	-1,378767719	True	1,378767719
ENSMUSG00000059908	Mug1	24642,73679	2213,537943	9480,480612	7746,26458	2773,897217	-1,378130385	True	1,378130385
ENSMUSG00000030088	Aldh1l1	16793,01348	4268,064608	6463,563783	2602,331045	5740,897345	-1,377459402	True	1,377459402
ENSMUSG00000030533	Unc45a	111,2887818	499,7497294	288,8350028	1023,388613	827,2111174	1,375937415	True	1,375937415
ENSMUSG00000047109	Cldn14	156,1134301	1303,640077	404,1928851	563,4260387	1831,868153	1,372449272	True	1,372449272
ENSMUSG00000041324	Inhba	238,8071777	109,7935012	618,1773536	192,307091	31,31398552	1,372174604	True	1,372174604
ENSMUSG00000042043	Tbca	479,9328717	667,5949668	368,0885097	185,5594738	781,5448885	-1,37095097	True	1,37095097
ENSMUSG00000021131	Erh	110,5159431	172,8932145	111,8355041	42,73490912	324,8825997	-1,370767549	True	1,370767549
ENSMUSG00000035459	Stab2	536,3501014	759,7205482	426,2077481	1811,735226	207,455154	-1,370375524	True	1,370375524
ENSMUSG00000007827	Ankrd26	74,19252123	214,5390252	72,20875071	383,4895792	28,70448672	-1,369997509	True	1,369997509
ENSMUSG00000014778	Fhod1	121,3356858	127,4614209	253,611222	241,7896174	46,97097827	-1,369162374	True	1,369162374
ENSMUSG00000033538	Casp4	23,18516288	208,2290539	59,88042742	232,7927944	152,6556794	1,368882657	True	1,368882657
ENSMUSG00000017286	Glod4	429,6983521	99,69754702	166,4323644	70,8498091	92,63720715	-1,368388237	True	1,368388237
ENSMUSG00000027792	Bche	2556,550627	336,9524691	862,1020359	256,4094547	990,304792	-1,368254071	True	1,368254071
ENSMUSG00000062762	Ei24	2440,624813	740,7906342	945,7585154	915,4267375	925,0673221	-1,367706767	True	1,367706767
ENSMUSG00000053175	Bcl3	297,5429237	1543,418987	1742,696557	766,9791584	1132,522476	1,366089568	True	1,366089568
ENSMUSG00000068206	Pick1	73,41968247	253,6608475	191,9696055	431,8475027	189,1886625	1,365586855	True	1,365586855
ENSMUSG00000021135	Slc10a1	16674,76915	297,8306468	6477,653295	441,9689285	776,3258909	-1,36412363	True	1,36412363
ENSMUSG00000028998	Tomm7	416,5600932	477,0338326	651,6399454	161,9428135	621,0607127	-1,363040187	True	1,363040187
ENSMUSG00000000340	Dbt	1046,423685	152,7013062	406,8346686	215,9237514	250,5118841	-1,362952571	True	1,362952571
ENSMUSG00000036856	Wnt4	54,0987134	296,5686525	139,1339343	214,7991485	182,6649155	1,362808143	True	1,362808143
ENSMUSG00000032215	Rsl24d1	89,64929649	100,9595413	73,08934523	34,86268902	233,550142	-1,36260841	True	1,36260841
ENSMUSG00000029273	Sult1d1	1871,815484	696,6208349	728,2516687	321,6364213	679,7744356	-1,361929219	True	1,361929219
ENSMUSG00000031134	Rbmx	259,6738243	782,436445	204,2979288	101,2142584	499,7190189	-1,359288052	True	1,359288052
ENSMUSG00000045409	Trim39	206,3479497	1660,784454	529,237307	1775,747934	2018,447316	1,35883566	True	1,35883566
ENSMUSG00000036278	Macrod1	768,2017302	235,9929278	268,5813289	38,23649764	300,0923612	-1,356078617	True	1,356078617
ENSMUSG00000017831	Rab5a	546,3970053	367,2403314	145,2980959	114,7094929	1398,691353	1,356056159	True	1,356056159
ENSMUSG00000026981	Il1rn	27,0493567	1023,47735	10,56713425	850,1997709	2246,778461	-1,356010105	True	1,356010105
ENSMUSG00000009549	Srp14	319,182409	310,4505895	303,8051097	124,8309187	647,1557007	-1,354405831	True	1,354405831
ENSMUSG00000022361	Zhx1	400,3304791	111,0554954	88,94004661	112,4602872	156,5699276	-1,354384319	True	1,354384319
ENSMUSG00000035399	Oser1	125,9727183	104,7455241	49,31329317	120,3325073	272,6926239	-1,353062821	True	1,353062821
ENSMUSG00000027828	Ssr3	3841,008651	6137,078116	3861,406974	1505,843245	14974,60882	-1,350913617	True	1,350913617
ENSMUSG00000051977	Prdm9	119,7900082	88,33959863	256,2530056	236,166603	46,97097827	-1,350666031	True	1,350666031
ENSMUSG00000040471	Ggt6	598,9500412	678,9529152	500,1776878	235,0420002	1073,808753	-1,349517094	True	1,349517094
ENSMUSG00000031822	Gse1	209,4393047	760,9825425	594,4013016	953,6632351	82,19921198	-1,349335745	True	1,349335745
ENSMUSG00000024978	Gpam	3609,929861	633,5211216	1416,876584	1251,682996	636,7177055	-1,349256708	True	1,349256708
ENSMUSG00000028952	Zbtb48	141,4294936	511,1076778	442,939044	635,4006225	360,1108334	1,348357992	True	1,348357992
ENSMUSG00000040841	Six5	146,0665262	106,0075184	269,4619234	311,5149954	57,40897345	-1,347277432	True	1,347277432
ENSMUSG00000025993	Slc40a1	1146,892724	121,1514495	450,8643947	199,0547083	49,58047707	-1,346964965	True	1,346964965
ENSMUSG00000038121	Fam210a	908,8583851	291,5206755	194,6113891	274,4031007	357,5013346	-1,346106889	True	1,346106889
ENSMUSG00000028082	Sh3d19	957,5472271	328,1185092	376,8944549	196,8055025	178,7506673	-1,345183074	True	1,345183074
ENSMUSG00000022766	Serpind1	8760,127376	116,1034725	3448,408144	221,5467657	397,9485659	-1,34502131	True	1,34502131
ENSMUSG00000037622	Wdtc1	1525,583718	580,5173624	600,5654632	289,022938	434,481549	-1,344967934	True	1,344967934
ENSMUSG00000040855	Reps2	333,0935068	54,26575344	131,2085836	91,0928326	104,3799517	-1,344065127	True	1,344065127
ENSMUSG00000035342	Lzts2	162,2961402	89,6015929	319,6558111	245,163426	63,93272043	-1,3440023	True	1,3440023
ENSMUSG00000051041	Olfml1	310,6811826	6,309971331	122,4026384	22,49205743	23,48548914	-1,343800207	True	1,343800207
ENSMUSG00000029545	Acads	1436,70726	239,7789106	566,2222769	364,3713304	268,7783757	-1,343325716	True	1,343325716
ENSMUSG00000014361	Mertk	466,7946127	286,4726984	501,9388769	1062,749714	183,9696649	-1,343320014	True	1,343320014
ENSMUSG00000035878	Hykk	966,8212923	133,7713922	381,2974275	109,0864785	69,15171802	-1,342332448	True	1,342332448
ENSMUSG00000087370	Tmem170b	273,584922	440,4359989	177,8800932	107,9618757	501,0237683	-1,341466775	True	1,341466775
ENSMUSG00000071177	Serpina1d	74786,83424	12465,97936	29544,82677	6013,251555	24286,60527	-1,339878775	True	1,339878775
ENSMUSG00000032295	Man2c1	374,8268	1543,418987	948,400299	2032,157389	1247,340423	1,339272009	True	1,339272009
ENSMUSG00000034793	G6pc3	84,23942515	499,7497294	213,103874	796,2188331	296,178113	1,338989324	True	1,338989324
ENSMUSG00000021193	Pitrm1	603,5870737	203,1810768	238,6411152	202,4285169	167,0079228	-1,338719285	True	1,338719285
ENSMUSG00000014177	Tvp23b	207,8936272	60,57572477	58,9998329	42,73490912	82,19921198	-1,338649066	True	1,338649066
ENSMUSG00000057894	Zfp329	85,78510267	103,4835298	112,7160987	245,163426	33,92348431	-1,338442775	True	1,338442775
ENSMUSG00000054417	Cyp3a44	4548,156119	34,07384519	359,2825645	6,74761723	1799,249418	-1,337886585	True	1,337886585
ENSMUSG00000084128	Esrp2	1783,711865	2569,420326	3647,422505	4344,340893	705,8694235	-1,337409352	True	1,337409352
ENSMUSG00000056666	Retsat	4521,106762	1264,518255	1744,457746	1789,243169	859,8298523	-1,337326515	True	1,337326515
ENSMUSG00000022791	Tnk2	368,6440899	411,4101308	352,2378083	689,3815603	146,1319324	-1,334957161	True	1,334957161
ENSMUSG00000015092	Edf1	1506,262749	2823,081173	3048,618231	597,1641248	2529,90908	-1,33477405	True	1,33477405
ENSMUSG00000028088	Fmo5	7134,074619	180,4651801	2829,350196	805,2156561	323,5778503	-1,334255552	True	1,334255552
ENSMUSG00000071645	Tut1	145,2936874	554,0154828	366,3273207	835,5799336	686,2981826	1,334161278	True	1,334161278
ENSMUSG00000013275	Slc41a1	65,69129484	530,0375918	165,5517699	380,1157706	463,1860358	1,333508327	True	1,333508327
ENSMUSG00000026853	Crat	860,9423818	807,6763303	1119,235636	2995,94205	341,8443419	-1,332577143	True	1,332577143
ENSMUSG00000070348	Ccnd1	1005,46323	79,50563877	383,0586166	67,4761723	399,2533153	-1,332484031	True	1,332484031
ENSMUSG00000028914	Casp9	261,2195018	54,26575344	103,9101535	44,98411486	45,66622888	-1,329925976	True	1,329925976
ENSMUSG00000028527	Ak4	325,3651191	78,2436445	129,4473946	50,60712922	73,0659662	-1,329693667	True	1,329693667
ENSMUSG00000039234	Sec24d	1263,591377	906,1118831	502,8194714	580,2950818	3751,154515	-1,329417574	True	1,329417574
ENSMUSG00000052560	Cpne8	34,00490556	1484,105257	85,41766852	1184,206824	1294,311401	1,328791635	True	1,328791635
ENSMUSG00000029247	Paics	2877,278714	705,4547948	1145,653472	535,3109669	699,3456765	-1,328534243	True	1,328534243
ENSMUSG00000040536	Necab1	678,5524337	49,21777638	270,3425179	84,34521537	49,58047707	-1,327671873	True	1,327671873
ENSMUSG00000026579	F5	7806,444343	1841,249634	3110,259848	1554,201169	2375,948651	-1,327630468	True	1,327630468
ENSMUSG00000021715	Cwc27	108,1974268	225,8969736	43,14913152	188,9332824	292,2638648	-1,326262761	True	1,326262761
ENSMUSG00000035284	Vps13c	247,3084041	71,93367317	98,62658634	62,97776081	57,40897345	-1,326262761	True	1,326262761
ENSMUSG00000002625	Akap8l	868,6707694	753,4105769	1108,668502	2199,723217	347,0633395	-1,323610512	True	1,323610512
ENSMUSG00000006941	Eif1b	149,1578812	140,0813635	169,074148	59,6039522	272,6926239	-1,323360308	True	1,323360308
ENSMUSG00000093803	Ppp2r3d	245,7627266	1002,023447	614,6549756	967,1584696	1193,845698	1,322510682	True	1,322510682
ENSMUSG00000026123	Plekhb2	173,8887276	44,16979931	69,56696715	53,98093784	56,10422405	-1,321690032	True	1,321690032
ENSMUSG00000039145	Camk1d	2035,657301	177,9411915	814,5499318	203,5531198	236,1596408	-1,321419664	True	1,321419664
ENSMUSG00000027248	Pdia3	8663,522531	15108,59535	9120,317453	3467,150653	25236,46283	-1,321203209	True	1,321203209
ENSMUSG00000045092	S1pr1	959,0929046	219,5870023	383,9392111	149,5721819	292,2638648	-1,320792664	True	1,320792664
ENSMUSG00000001665	Gstt3	1717,247731	227,1589679	687,7443208	96,71584696	339,2348431	-1,320153952	True	1,320153952
ENSMUSG00000057691	Zfp746	187,7998194	528,7755975	468,4762851	881,6886513	1098,598992	1,318780343	True	1,318780343
ENSMUSG00000044562	Rasip1	57,19006845	566,6354255	142,6563124	761,3561441	241,3786384	1,318707049	True	1,318707049
ENSMUSG00000017802	Retreg3	902,675675	252,3988532	361,9243481	322,7610242	236,1596408	-1,318519564	True	1,318519564
ENSMUSG00000033021	Gmppa	721,0585657	2391,479134	1798,174012	2887,980174	3760,287761	1,318344291	True	1,318344291
ENSMUSG00000031349	Nsdhl	2080,481949	848,0601468	5186,701728	525,189541	273,9973733	1,317899635	True	1,317899635
ENSMUSG00000018293	Pfn1	2394,254487	3620,66155	3287,259346	960,4108524	3462,804898	-1,317852895	True	1,317852895
ENSMUSG00000027439	Gzf1	782,8856667	90,86358716	314,3722439	155,1951963	62,62797103	-1,316327783	True	1,316327783
ENSMUSG00000006373	Pgrmc1	14209,41349	2997,236382	4663,628582	1231,440144	5710,888109	-1,315059983	True	1,315059983
ENSMUSG00000037709	Fam13a	604,3599125	31,54985665	212,2232795	242,9142203	49,58047707	-1,314961021	True	1,314961021
ENSMUSG00000026390	Marco	105,8789105	519,9416376	263,2977617	1078,494154	430,5673008	1,314280003	True	1,314280003
ENSMUSG00000035021	Baz1a	102,0147167	851,8461296	253,611222	1109,983034	533,6425032	1,313841293	True	1,313841293
ENSMUSG00000032018	Sc5d	13704,74978	605,7572477	5516,924673	522,9403353	382,2915732	-1,3127398	True	1,3127398
ENSMUSG00000034731	Dgkh	21,63948536	129,9854094	53,71626577	323,885627	193,1029107	1,311692827	True	1,311692827
ENSMUSG00000022559	Fbxl6	174,6615604	1062,599172	463,192718	1243,810776	433,1767996	1,310393842	True	1,310393842
ENSMUSG00000025470	Zfp511	128,2912346	122,4134438	172,5965261	51,73173209	169,6174215	-1,310301201	True	1,310301201
ENSMUSG00000032314	Etfa	2697,980121	489,6537753	1088,414828	325,0102299	598,879973	-1,309651202	True	1,309651202
ENSMUSG00000038641	Akr1d1	3915,974011	95,91156423	1579,78657	94,46664122	305,3113588	-1,309641527	True	1,309641527
ENSMUSG00000032393	Dpp8	527,0760362	769,8165023	479,0434193	1016,640996	212,6741516	-1,309366402	True	1,309366402
ENSMUSG00000025004	Cyp2c40	5538,162574	532,5615803	1202,011521	24,74126318	2235,035716	-1,309109519	True	1,309109519
ENSMUSG00000046532	Ar	357,8243472	106,0075184	144,4175014	95,59124409	41,75198069	-1,309005967	True	1,309005967
ENSMUSG00000029186	Pi4k2b	401,1033179	416,4581078	221,0292247	161,9428135	1313,882642	-1,308489453	True	1,308489453
ENSMUSG00000033862	Cdk10	216,3948536	382,3842626	422,68537	897,4330916	87,41820957	-1,307660453	True	1,307660453
ENSMUSG00000020321	Mdh1	5750,693234	1259,470278	1988,382428	660,1418856	2323,758675	-1,307275629	True	1,307275629
ENSMUSG00000032557	Uba5	502,3451958	1556,03893	1241,638274	3218,613419	2428,138627	1,305493951	True	1,305493951
ENSMUSG00000036892	Prodh2	2644,654246	388,694234	6530,488967	266,5308806	540,1662502	1,304111895	True	1,304111895
ENSMUSG00000040511	Pvr	78,82955381	572,9453968	194,6113891	536,4355698	943,3338137	1,303787629	True	1,303787629

ENSMUSG00000020692	Nle1	37,09626061	198,1330998	91,58183017	188,9332824	283,130619	1,303787629	True	1,303787629
ENSMUSG00000024049	Myom1	9,274065154	670,1189553	22,89545754	2018,662155	551,9089947	1,303787629	True	1,303787629
ENSMUSG00000006412	Pfdn2	305,2713113	301,6166296	314,3722439	123,7063159	585,832479	-1,303172855	True	1,303172855
ENSMUSG00000033105	Lss	1814,625415	753,4105769	4477,823139	755,7331297	152,6556794	1,303125777	True	1,303125777
ENSMUSG00000038611	Phrf1	257,355308	909,8978659	634,028055	1354,021857	1321,711139	1,300785141	True	1,300785141
ENSMUSG00000089862	Umad1	146,8393649	45,43179358	39,62675344	59,6039522	27,39973733	-1,300758879	True	1,300758879
ENSMUSG00000063856	Gpx1	21421,54483	4227,680792	8707,378622	2532,605667	7150,026693	-1,298762103	True	1,298762103
ENSMUSG00000022587	Ly6e	10125,73347	1668,35642	4117,65998	2588,83581	3113,13206	-1,298129807	True	1,298129807
ENSMUSG00000041506	Rrp9	73,41968247	426,554062	180,5218768	256,4094547	427,9578021	1,297934903	True	1,297934903
ENSMUSG00000035936	Aldh5a1	846,2584453	164,0592546	344,3124577	76,47299527	167,0079228	-1,297379946	True	1,297379946
ENSMUSG00000035722	Abca7	78,82955381	374,812297	193,7307946	677,0109287	255,7308817	1,297244783	True	1,297244783
ENSMUSG00000003848	Nob1	86,55794143	292,7826697	212,2232795	213,6745456	271,3878745	1,293844826	True	1,293844826
ENSMUSG00000008682	Rpl10	5818,703045	8603,014912	8225,633419	2375,161265	9946,10465	-1,29267215	True	1,29267215
ENSMUSG00000033434	Gtpbp6	132,1554284	482,0818097	346,0736467	601,6625363	323,5778503	1,291877169	True	1,291877169
ENSMUSG00000034723	Tmx4	122,8813633	206,9670596	50,19388769	101,2142584	225,7216456	-1,29168253	True	1,29168253
ENSMUSG00000037098	Rab11fip3	265,0836956	254,9228418	408,5958577	819,8354934	108,2941999	-1,291491962	True	1,291491962
ENSMUSG00000017778	Cox7c	2215,728733	3698,905194	3463,378251	905,3053117	4252,178283	-1,291304942	True	1,291304942
ENSMUSG00000034789	Rab24	341,5947332	358,4063716	538,9238468	456,5887659	139,6081854	-1,2909022	True	1,2909022
ENSMUSG00000023034	Nr4a1	71,10116618	107,2695126	29,05961919	68,60077517	323,5778503	-1,290857425	True	1,290857425
ENSMUSG00000046808	Atp10d	26,27651794	93,38757569	64,28340002	422,8506797	153,9604288	1,290672129	True	1,290672129
ENSMUSG00000032500	Dclk3	210,9849822	2,523988532	86,29826304	3,373808615	14,35224336	-1,289736885	True	1,289736885
ENSMUSG00000032540	Abhd5	384,8737039	1091,62504	303,8051097	157,444402	562,3469899	-1,289542641	True	1,289542641
ENSMUSG00000034201	Gas2l1	255,0367917	752,1485826	435,8942878	727,6180579	104,3799517	-1,288860746	True	1,288860746
ENSMUSG00000000168	Dlat	870,2164469	224,6349794	302,0439207	239,5404117	356,1965852	-1,288700598	True	1,288700598
ENSMUSG00000025534	Gusb	266,6293732	68,14769037	109,1937206	100,0896556	84,80871077	-1,28794583	True	1,28794583
ENSMUSG00000063410	Stk24	244,9898878	295,3066583	100,3877754	119,2079044	656,2889464	-1,287138605	True	1,287138605
ENSMUSG00000057400	Ces1c	47496,35185	11698,68685	15884,16397	2203,097026	19464,2515	-1,286989839	True	1,286989839
ENSMUSG00000054422	Fabp1	42915,7365	3985,377892	17589,87555	1364,143283	6812,096599	-1,286761481	True	1,286761481
ENSMUSG00000031432	Prps1	353,1873146	126,1994266	100,3877754	74,22378953	144,827183	-1,286101116	True	1,286101116
ENSMUSG00000067203	H2-K2	438,1995785	145,1293406	1068,161154	312,6395983	146,1319324	1,285469322	True	1,285469322
ENSMUSG00000059326	Csf2ra	111,2887818	345,7864289	271,2231124	416,1030625	273,9973733	1,285171951	True	1,285171951
ENSMUSG00000055737	Ghr	6678,872588	1357,90583	2741,290743	997,5227471	1110,341736	-1,284749243	True	1,284749243
ENSMUSG00000019054	Fis1	558,7624255	1759,220007	1360,518535	1373,140106	1801,858917	1,283849697	True	1,283849697
ENSMUSG00000041220	Elovl6	3035,71066	306,6646067	1246,921842	231,6681916	191,7981613	-1,283663253	True	1,283663253
ENSMUSG00000073987	Ggh	180,0714317	50,47977065	73,96993975	41,61030625	65,23746983	-1,283558311	True	1,283558311
ENSMUSG00000029649	Pomp	1275,183959	1644,378529	1566,577653	524,0649382	1945,38135	-1,28288789	True	1,28288789
ENSMUSG00000002319	Ipo4	194,7553682	661,2849955	473,7598522	664,6402971	1298,22565	1,28249285	True	1,28249285
ENSMUSG00000025351	Cd63	69,55548865	1337,713922	169,074148	260,9078662	1817,515909	1,281419816	True	1,281419816
ENSMUSG00000038522	Mfsd4b1	647,6388832	123,6754381	249,2082494	266,5308806	50,88522646	-1,28088691	True	1,28088691
ENSMUSG00000029781	Fkbp9	359,3700247	70,6716789	147,9398795	136,0769475	96,55145534	-1,280459072	True	1,280459072
ENSMUSG00000030815	Phkg2	452,1106762	2034,334757	1098,101368	3071,290442	2476,414355	1,280263347	True	1,280263347
ENSMUSG00000049090	Zadh2	974,5496799	316,7605608	401,5511015	250,7864404	131,779689	-1,279152135	True	1,279152135
ENSMUSG00000061130	Ppm1b	1059,561944	378,5982798	436,7748823	308,1411868	391,424819	-1,278506134	True	1,278506134
ENSMUSG00000040410	Fbxl4	287,4960198	118,627461	88,05945209	80,97140676	52,18997586	-1,277103965	True	1,277103965
ENSMUSG00000072946	Ptgr2	687,0536601	47,95578211	283,5514357	141,6999618	35,22823371	-1,276812319	True	1,276812319
ENSMUSG00000021210	Akr1c6	12512,25957	200,6570883	5167,328648	32,61348328	1248,645172	-1,275851798	True	1,275851798
ENSMUSG00000031782	Coq9	1108,250786	276,3767443	457,9091508	327,2594356	204,8456553	-1,275151084	True	1,275151084
ENSMUSG00000007670	Khsrp	581,9475884	522,4656262	530,1179016	240,6650145	1153,398467	-1,273862794	True	1,273862794
ENSMUSG00000025377	Tepsin	139,8838161	338,2144633	457,9091508	625,2791966	365,329831	1,273709297	True	1,273709297
ENSMUSG00000044042	Fmn1	189,3454969	16,40592546	78,37291236	38,23649764	9,133245776	-1,272594097	True	1,272594097
ENSMUSG00000039428	Tmem135	1341,648092	372,2883085	555,6551427	348,6268902	408,3865611	-1,271744626	True	1,271744626
ENSMUSG00000053801	Grwd1	64,91845608	258,7088246	156,7458247	202,4285169	294,8733636	1,27172642	True	1,27172642
ENSMUSG00000074733	Zfp950	478,3871942	376,0742913	1071,683532	780,4743929	198,3219083	-1,270334713	True	1,270334713
ENSMUSG00000073436	Eme2	64,91845608	398,7901881	252,7306275	761,3561441	156,5699276	1,270106546	True	1,270106546
ENSMUSG00000044279	Crb3	220,2590474	288,9966869	413,8794248	286,7737323	91,33245776	-1,270001719	True	1,270001719
ENSMUSG00000051586	Mical3	272,8120833	1240,540364	671,8936194	1207,823484	657,5936958	1,269288923	True	1,269288923
ENSMUSG00000030400	Ercc2	164,6146565	397,5281938	396,2675344	574,6720674	408,3865611	1,267381982	True	1,267381982
ENSMUSG00000036438	Calm2	982,2780675	937,6617397	865,624414	408,2308424	2056,285049	-1,266746303	True	1,266746303
ENSMUSG00000035572	Dcaf10	220,2590474	196,8711055	91,58183017	164,1920193	588,4419778	-1,26606798	True	1,26606798
ENSMUSG00000041028	Ghitm	2854,093551	695,3588406	901,7287894	377,8665649	1187,321951	-1,265321439	True	1,265321439
ENSMUSG00000030259	Rassf8	451,3378375	71,93367317	176,1189042	65,22696655	187,8839131	-1,264366184	True	1,264366184
ENSMUSG00000035150	Eif2s3x	247,3084041	51,74176491	25,5372411	47,23332061	103,0752023	-1,262613974	True	1,262613974
ENSMUSG00000050912	Tmem123	494,6168082	97,17355849	190,2084165	100,0896556	206,1504046	-1,262613974	True	1,262613974
ENSMUSG00000075590	Nrbp2	1064,971815	6111,838231	2554,604705	17799,08965	2869,143923	1,262284819	True	1,262284819
ENSMUSG00000032528	Vipr1	481,4785492	92,12558143	200,7755508	195,6808997	113,5131975	-1,261887928	True	1,261887928
ENSMUSG00000010663	Fads1	9081,628302	1005,80943	3787,437034	1093,119991	1987,133331	-1,261729086	True	1,261729086
ENSMUSG00000042570	Mier2	112,0616206	474,5098441	268,5813289	579,1704789	353,5870865	1,261066754	True	1,261066754
ENSMUSG00000026866	Kynu	2257,462026	441,6979931	942,2361373	326,1348328	82,19921198	-1,26054115	True	1,26054115
ENSMUSG00000021211	Akr1c12	857,8510267	204,4430711	358,40197	120,3325073	225,7216456	-1,259148566	True	1,259148566
ENSMUSG00000037965	Zc3h7a	608,9969451	2896,276841	1457,383932	8242,214446	5656,088634	1,258874094	True	1,258874094
ENSMUSG00000025607	Copg2	339,2762169	59,31373051	141,7757179	84,34521537	43,05673008	-1,25884984	True	1,25884984
ENSMUSG00000026238	Ptma	2548,049401	3089,361964	1722,442883	1064,998919	5666,526629	-1,258541282	True	1,258541282
ENSMUSG00000053617	Sh3pxd2a	768,974569	136,2953807	321,4170001	136,0769475	146,1319324	-1,25848965	True	1,25848965
ENSMUSG00000038217	Tlcd2	2044,158528	109,7935012	855,0572798	94,46664122	109,5989493	-1,25741411	True	1,25741411
ENSMUSG00000036764	Dnajc12	87,3307802	90,86358716	448,2226111	66,35156943	36,5329831	-1,257290667	True	1,257290667
ENSMUSG00000024222	Fkbp5	738,8338572	2305,663524	309,0886768	2032,157389	926,3720715	-1,257229176	True	1,257229176
ENSMUSG00000034664	Itga2b	31,68638927	302,8786239	75,73112879	240,6650145	169,6174215	1,257023161	True	1,257023161
ENSMUSG00000046798	Cldn12	881,8090284	287,7346927	368,9691042	246,2880289	301,3971106	-1,256966231	True	1,256966231
ENSMUSG00000042063	Zfp386	183,9356255	254,9228418	233,357548	240,6650145	76,98021439	-1,256641341	True	1,256641341
ENSMUSG00000028339	Col15a1	280,5404709	234,7309335	376,8944549	558,9276272	117,4274457	-1,256439268	True	1,256439268
ENSMUSG00000020609	Apob	57697,05068	17784,0232	18893,15544	11442,83422	24158,73983	-1,255952372	True	1,255952372
ENSMUSG00000036281	Snapc4	169,2516891	1007,071424	404,1928851	1185,331427	1192,540948	1,255873695	True	1,255873695
ENSMUSG00000027774	Gfm1	1030,194071	323,0705321	431,4913152	183,3102681	378,377325	-1,255512715	True	1,255512715
ENSMUSG00000019762	Iyd	1131,435949	127,4614209	474,6404467	77,59759814	216,5883998	-1,253247964	True	1,253247964
ENSMUSG00000016942	Tmprss6	5532,752703	725,646703	2322,127751	636,5252253	1049,018515	-1,252550097	True	1,252550097
ENSMUSG00000025497	Cdhr5	833,1201863	1020,953361	1139,48931	883,9378571	349,6728383	-1,252518899	True	1,252518899
ENSMUSG00000022623	Shank3	77,28387628	542,6575344	230,7157645	773,7267757	183,9696649	1,251228536	True	1,251228536
ENSMUSG00000040269	Mrps28	109,7431043	186,7751514	218,3874412	46,10871774	249,2071347	-1,251018841	True	1,251018841
ENSMUSG00000062906	Hdac10	183,1627868	227,1589679	309,0886768	317,1380098	76,98021439	-1,250566826	True	1,250566826
ENSMUSG00000052539	Magi3	483,7970655	170,3692259	203,4173343	150,6967848	53,49472526	-1,249959394	True	1,249959394
ENSMUSG00000041272	Tox	136,0196223	218,325008	57,23864386	139,4507561	150,0461806	-1,248753394	True	1,248753394
ENSMUSG00000018459	Slc13a3	365,5527348	208,2290539	948,400299	500,4482779	153,9604288	-1,247519951	True	1,247519951
ENSMUSG00000021364	Elovl2	6798,662596	406,3621537	2863,693382	421,7260769	662,8126934	-1,247373943	True	1,247373943
ENSMUSG00000047153	Khnyn	901,1299974	325,5945207	287,0738138	173,1888422	379,6820744	-1,246943357	True	1,246943357
ENSMUSG00000024997	Prdx3	1300,687638	336,9524691	548,6103865	175,438048	456,6622888	-1,245420697	True	1,245420697
ENSMUSG00000018425	Dhx40	488,4340981	397,5281938	206,0591179	292,3967466	2040,628056	-1,245105617	True	1,245105617
ENSMUSG00000022025	Cnmd	166,9331728	0	70,44756167	4,498411486	2,609498793	-1,244648996	True	1,244648996
ENSMUSG00000041025	Iffo2	114,3801369	446,7459702	317,894622	271,0292921	617,1464645	1,244612245	True	1,244612245
ENSMUSG00000033327	Tnxb	148,3850425	254,9228418	383,9392111	439,7197228	62,62797103	-1,244466627	True	1,244466627
ENSMUSG00000041660	Bbox1	1775,210638	129,9854094	749,3859372	170,9396365	170,9221709	-1,244209408	True	1,244209408
ENSMUSG00000031176	Dynlt3	746,5622449	238,5169163	307,3274878	161,9428135	315,749354	-1,241482768	True	1,241482768
ENSMUSG00000034684	Sema3f	73,41968247	357,1443773	184,0442549	915,4267375	173,5316697	1,2409602	True	1,2409602
ENSMUSG00000039737	Prkrip1	53,32587463	128,7234151	134,7309617	125,9555216	227,026395	1,24000674	True	1,24000674
ENSMUSG00000025207	Sema4g	8749,307634	787,4844221	20665,79222	9029,436456	969,4288016	1,240003909	True	1,240003909
ENSMUSG00000031706	Rfx1	159,9776239	782,436445	377,7750494	644,3974454	1240,816676	1,239657291	True	1,239657291
ENSMUSG00000072949	Acot1	371,7354449	56,78974198	73,96993975	157,444402	143,5224336	-1,239433792	True	1,239433792
ENSMUSG00000021048	Mthfd1	3043,439048	194,347117	1289,190379	337,3808615	78,28496379	-1,239237146	True	1,239237146
ENSMUSG00000064127	Med14	212,5306598	85,8156101	88,05945209	51,73173209	90,02770836	-1,239229978	True	1,239229978
ENSMUSG00000097195	Snhg5	33,2320668	338,2144633	78,37291236	194,5562968	654,984197	1,237779088	True	1,237779088
ENSMUSG00000027775	Mfsd1	830,0288313	213,277031	327,5811618	172,0642394	352,2823371	-1,236429308	True	1,236429308
ENSMUSG00000053128	Rnf26	32,45922804	102,2215356	125,9250165	76,47299527	264,8641275	1,236321693	True	1,236321693
ENSMUSG00000027999	Pla2g12a	126,7455571	892,2299462	298,5215426	340,7546701	1491,32856	1,23589987	True	1,23589987
ENSMUSG00000003810	Mast2	331,5478292	2409,147054	780,2067455	1905,077265	3036,151846	1,234639462	True	1,234639462

ENSMUSG00000068856	Sf3b4	156,1134301	223,3729851	199,0143617	66,35156943	550,6042453	-1,234392159	True	1,234392159
ENSMUSG00000042903	Foxo4	304,4984725	65,62370184	129,4473946	88,84362686	70,45646741	-1,234069063	True	1,234069063
ENSMUSG00000022863	Btg3	12,3654202	145,1293406	29,05961919	91,0928326	521,8997586	1,232704531	True	1,232704531
ENSMUSG00000031561	Tenm3	195,528207	71,93367317	70,44756167	83,2206125	58,71372284	-1,232363935	True	1,232363935
ENSMUSG00000062647	Rpl7a	4663,309095	5068,168973	6484,698052	1984,924068	5507,347203	-1,232270239	True	1,232270239
ENSMUSG00000030727	Rabep2	165,3874952	175,417203	216,6262521	264,2816748	70,45646741	-1,231046111	True	1,231046111
ENSMUSG00000020474	Polm	142,9751711	802,6283533	335,5065124	854,6981824	623,6702115	1,230576138	True	1,230576138
ENSMUSG00000021242	Npc2	1034,831103	241,0409048	441,1778549	236,166603	409,6913105	-1,229963041	True	1,229963041
ENSMUSG00000036655	Colec11	480,7057105	58,05173624	205,1785234	98,9650527	136,9986866	-1,228274214	True	1,228274214
ENSMUSG00000030814	Bcl7c	262,7651794	196,8711055	556,5357372	212,5499427	112,2084481	-1,227592808	True	1,227592808
ENSMUSG00000000686	Abhd15	253,4911142	65,62370184	108,3131261	106,8372728	19,57124095	-1,226727088	True	1,226727088
ENSMUSG00000058446	Znrf2	748,1079224	315,4985665	294,11857	170,9396365	319,6636021	-1,226691925	True	1,226691925
ENSMUSG00000054455	Vapb	891,0830935	253,6608475	381,2974275	175,438048	210,0646528	-1,224643172	True	1,224643172
ENSMUSG00000001942	Siae	604,3599125	31,54985665	253,611222	258,6586605	33,92348431	-1,224358472	True	1,224358472
ENSMUSG00000022822	Abcc5	109,7431043	71,93367317	223,6710083	199,0547083	46,97097827	-1,224288745	True	1,224288745
ENSMUSG00000024026	Glo1	4062,813376	1124,436891	1740,054773	725,3688522	848,0871077	-1,223346377	True	1,223346377
ENSMUSG00000007739	Cct4	1098,976321	326,8565149	471,1180687	389,1125936	409,6913105	-1,222000257	True	1,222000257
ENSMUSG00000027778	Ift80	97,37768411	87,07760436	118,8802603	195,6808997	41,75198069	-1,22174655	True	1,22174655
ENSMUSG00000052557	Gan	85,78510267	662,5469897	199,8949562	522,9403353	1082,941999	1,220443032	True	1,220443032
ENSMUSG00000020368	Canx	4518,015407	1398,289647	1939,069135	1401,255178	1268,216413	-1,220324949	True	1,220324949
ENSMUSG00000068742	Cry2	396,4662853	273,8527558	199,8949562	184,4348709	923,7625727	1,220323908	True	1,220323908
ENSMUSG00000047492	Inhbe	279,7676321	2406,623066	651,6399454	3286,089591	1503,071305	1,219845985	True	1,219845985
ENSMUSG00000008200	Fnbp4	418,8786094	3122,173814	974,8181346	3981,094166	2473,804856	1,218600877	True	1,218600877
ENSMUSG00000042453	Reln	752,744955	234,7309335	189,327822	179,9364595	323,5778503	-1,218048279	True	1,218048279
ENSMUSG00000032179	Bmp5	198,619562	45,43179358	85,41766852	16,86904307	45,66622888	-1,217401296	True	1,217401296
ENSMUSG00000034055	Phka1	77,28387628	572,9453968	179,6412823	808,5894647	442,3100454	1,216879564	True	1,216879564
ENSMUSG00000070704	Ugt2b36	7976,468871	764,7685253	3435,199226	224,9205743	1711,831208	-1,215356445	True	1,215356445
ENSMUSG00000024560	Cxxc1	491,5254531	3009,856325	1141,250499	2481,998538	4078,646613	1,215277459	True	1,215277459
ENSMUSG00000028444	Cntfr	40,96045443	191,8231285	95,10420825	272,1538949	106,9894505	1,215277459	True	1,215277459
ENSMUSG00000021890	Eaf1	407,286028	418,9820964	218,3874412	175,438048	1033,361522	-1,215080658	True	1,215080658
ENSMUSG00000031555	Adam9	857,0781879	244,8268876	190,2084165	161,9428135	369,2440792	-1,214852034	True	1,214852034
ENSMUSG00000016534	Lamp2	10680,6317	3637,067475	4402,972604	2846,369868	4601,851121	-1,214710762	True	1,214710762
ENSMUSG00000048307	Ankrd46	551,8068766	175,417203	237,7605206	119,2079044	169,6174215	-1,214654256	True	1,214654256
ENSMUSG00000028359	Orm3	236,4886614	955,3296595	957,2062442	548,8062014	1824,039656	1,214525769	True	1,214525769
ENSMUSG00000029169	Dhx15	758,1548263	699,1448234	326,7005672	652,2696655	1520,033047	-1,214523538	True	1,214523538
ENSMUSG00000025486	Sirt3	965,2756147	668,8569611	785,4903126	2239,084317	127,8654409	1,213896018	True	1,213896018
ENSMUSG00000029776	Hibadh	5395,960242	682,738898	2326,530724	421,7260769	1534,38529	-1,213699479	True	1,213699479
ENSMUSG00000018796	Acsl1	14957,52142	2515,154572	4037,525878	6451,846674	4010,799645	-1,21308707	True	1,21308707
ENSMUSG00000003812	Dnase2a	241,8985328	339,4764576	430,6107207	638,7744311	104,3799517	-1,212557379	True	1,212557379
ENSMUSG00000036197	Gxylt1	140,6566548	157,7492833	60,76102194	103,4634642	271,3878745	-1,210959772	True	1,210959772
ENSMUSG00000028011	Tdo2	9475,776071	3505,820071	3599,870401	4093,554453	3812,477737	-1,210890019	True	1,210890019
ENSMUSG00000002105	Slc39a13	170,7973666	648,6650528	425,3271536	1320,283771	395,3390671	1,210804797	True	1,210804797
ENSMUSG00000036989	Trim3	197,0738845	1207,728513	456,1479618	1536,207523	750,230903	1,210765262	True	1,210765262
ENSMUSG00000022200	Golph3	790,6140543	678,9529152	351,3572138	341,879273	1825,344406	-1,209486642	True	1,209486642
ENSMUSG00000040560	Wdr7	211,757821	79,50563877	91,58183017	73,09918666	52,18997586	-1,209281953	True	1,209281953
ENSMUSG00000033623	Pcgf3	472,2044841	148,9153234	204,2979288	152,9459905	156,5699276	-1,208737163	True	1,208737163
ENSMUSG00000043183	Simc1	76,51103752	176,6791973	191,9696055	204,6777226	212,6741516	1,207392391	True	1,207392391
ENSMUSG00000038695	Josd2	872,5349632	273,8527558	2013,919669	291,2721437	108,2941999	1,206721291	True	1,206721291
ENSMUSG00000062234	Gak	861,7152205	918,7318257	373,3720768	815,3370819	1559,175529	-1,206597129	True	1,206597129
ENSMUSG00000001285	Myg1	185,4813031	512,3696721	460,5509344	563,4260387	427,9578021	1,206194782	True	1,206194782
ENSMUSG00000021963	Sap18	609,7697839	841,7501755	660,4458906	264,2816748	1193,845698	-1,206188269	True	1,206188269
ENSMUSG00000005312	Ubqln1	2320,834805	590,6133166	858,5796578	588,1673019	1005,961785	-1,206068336	True	1,206068336
ENSMUSG00000023991	Foxp4	421,1971257	2262,755719	971,2957565	2234,585906	2035,409059	1,205415067	True	1,205415067
ENSMUSG00000075703	Selenoi	710,238823	100,9595413	308,2080823	212,5499427	113,5131975	-1,204399528	True	1,204399528
ENSMUSG00000037742	Eef1a1	59712,61417	17985,94228	18548,84299	12806,9775	25931,89426	-1,203308135	True	1,203308135
ENSMUSG00000071072	Ptges3	1120,616206	1421,005544	1032,937373	486,9530434	1976,695336	-1,202437696	True	1,202437696
ENSMUSG00000041698	Slco1a1	762,0190201	108,5315069	57,23864386	10,12142584	331,4063467	-1,201225776	True	1,201225776
ENSMUSG00000023963	Cyp39a1	489,9797756	54,26575344	213,103874	67,4761723	131,779689	-1,201165382	True	1,201165382
ENSMUSG00000026773	Pfkfb3	43,27897072	1793,293852	99,50718086	284,5245265	2442,49087	1,201134452	True	1,201134452
ENSMUSG00000061607	Mdc1	209,4393047	218,325008	148,820474	91,0928326	471,0145321	-1,201122764	True	1,201122764
ENSMUSG00000030237	Slco1a4	1439,798615	111,0554954	626,9832988	209,1761341	591,0514766	-1,199368117	True	1,199368117
ENSMUSG00000062054	Iah1	1667,78605	388,694234	726,4904797	193,4316939	580,6134814	-1,198918429	True	1,198918429
ENSMUSG00000038591	Colec10	424,2884808	23,97789106	184,9248494	24,74126318	100,4657035	-1,19810641	True	1,19810641
ENSMUSG00000052684	Jun	365,5527348	596,9232879	159,3876083	168,6904307	1497,852307	-1,197540075	True	1,197540075
ENSMUSG00000034435	Tmem30b	579,6290721	109,7935012	252,7306275	103,4634642	118,7321951	-1,197529447	True	1,197529447
ENSMUSG00000001518	Itfg2	125,9727183	430,3400448	288,8350028	355,3745074	606,7084694	1,197134262	True	1,197134262
ENSMUSG00000020873	Slc35b1	1017,828651	3624,447532	2332,694886	4207,139343	6599,422448	1,196502909	True	1,196502909
ENSMUSG00000041215	Yeats2	182,389948	171,6312202	204,2979288	362,1221247	79,58971319	-1,196372339	True	1,196372339
ENSMUSG00000021958	Pinx1	69,55548865	259,9708188	159,3876083	175,438048	250,5118841	1,196303202	True	1,196303202
ENSMUSG00000038982	Bloc1s5	300,6342787	58,05173624	131,2085836	70,84998091	46,97097827	-1,196147413	True	1,196147413
ENSMUSG00000064289	Tank	264,3108569	429,0780505	115,3578822	177,6872537	938,1148161	-1,196119102	True	1,196119102
ENSMUSG00000002679	Med6	108,1974268	108,5315069	119,7608548	47,23332061	281,8258696	-1,19578932	True	1,19578932
ENSMUSG00000024346	Pfdn1	332,320668	384,9082512	549,490981	145,0737704	328,7968479	-1,19578932	True	1,19578932
ENSMUSG00000004665	Cnn2	86,55794143	94,64956996	153,2234466	151,8213877	37,8377325	-1,193840554	True	1,193840554
ENSMUSG00000026667	Uhmk1	432,0168684	79,50563877	94,22361373	60,72855507	189,1886625	-1,19126201	True	1,19126201
ENSMUSG00000060224	Pyroxd2	419,6514482	268,8047787	1031,176184	1160,590164	183,9696649	-1,189723662	True	1,189723662
ENSMUSG00000039976	Tbc1d16	295,2244074	74,4576617	129,4473946	104,5880671	52,18997586	-1,189446072	True	1,189446072
ENSMUSG00000003808	Farsa	192,4368519	556,5394714	438,5360714	508,320498	703,2599247	1,188310411	True	1,188310411
ENSMUSG00000039154	Shd	0,772838763	164,0592546	1,761189042	40,48570338	568,8707369	1,188310411	True	1,188310411
ENSMUSG00000047446	Arl4a	381,0095101	878,3480092	167,312959	891,8100772	1112,951235	-1,187277817	True	1,187277817
ENSMUSG00000051790	Nlgn2	51,00735834	194,347117	139,1339343	356,4991103	116,1226963	1,186872685	True	1,186872685
ENSMUSG00000062604	Srpk2	320,7280866	300,3546353	423,5659645	765,8545556	140,9129348	-1,186546651	True	1,186546651
ENSMUSG00000020232	Hmg20b	467,5674515	1749,124053	1063,758181	1842,099504	1067,285006	1,185923819	True	1,185923819
ENSMUSG00000034543	Morc2a	226,4417575	1179,964639	515,1477947	1271,925848	832,430115	1,185846371	True	1,185846371
ENSMUSG00000032359	Ctsh	4182,603384	1132,008857	1839,561954	1300,04092	1719,659705	-1,185038936	True	1,185038936
ENSMUSG00000021905	Dph3	272,0392445	1167,344696	618,1773536	942,4172064	917,2388257	1,184206013	True	1,184206013
ENSMUSG00000024276	Zfp397	343,9132494	148,9153234	151,4622576	74,22378953	114,8179469	-1,18308636	True	1,18308636
ENSMUSG00000024220	Zfp523	302,1799563	316,7605608	454,3867728	540,9339812	133,0844384	-1,183066082	True	1,183066082
ENSMUSG00000042625	Safb2	616,7253327	2194,608029	1400,145288	4139,66317	2681,26001	1,182876525	True	1,182876525
ENSMUSG00000028959	Fastk	555,6710705	3315,258937	1261,011354	2981,322213	2696,917003	1,182278228	True	1,182278228
ENSMUSG00000027173	Depdc7	485,342743	88,33959863	213,9844686	107,9618757	33,92348431	-1,181497834	True	1,181497834
ENSMUSG00000016194	Hsd11b1	8285,604376	1934,63721	2057,068801	612,908565	3653,29831	-1,181407374	True	1,181407374
ENSMUSG00000052102	Gnpda1	399,5576404	87,07760436	139,1339343	176,5626508	87,41820957	-1,178223447	True	1,178223447
ENSMUSG00000078866	Zfp970	180,0714317	538,8715516	294,11857	495,9498664	79,58971319	-1,177915434	True	1,177915434
ENSMUSG00000050234	Gja4	40,18761567	90,86358716	101,2683699	190,0578853	208,7599034	1,176951277	True	1,176951277
ENSMUSG00000008140	Emc10	1075,018719	283,9487099	475,5210413	260,9078662	306,6161082	-1,176780696	True	1,176780696
ENSMUSG00000026895	Ndufa8	996,1891653	1906,873336	1987,501834	440,8443257	1705,307461	-1,17615043	True	1,17615043
ENSMUSG00000022656	Nectin3	713,3301781	162,7972603	316,133433	110,2110814	240,073889	-1,174036392	True	1,174036392
ENSMUSG00000032306	Mpi	153,022075	147,6533291	262,4171672	287,8983351	67,84696862	-1,173383529	True	1,173383529
ENSMUSG00000045917	Tmem268	104,333233	614,5912076	235,1187371	817,5862877	326,1873491	1,172190746	True	1,172190746
ENSMUSG00000073678	Pgap1	220,2590474	25,23988532	97,74599182	29,23967466	20,87599034	-1,172091831	True	1,172091831
ENSMUSG00000058392	Rrp1b	66,4641336	458,1039186	149,7010685	287,8983351	1067,285006	1,171436593	True	1,171436593
ENSMUSG00000032479	Map4	662,3228197	203,1810768	294,11857	289,022938	136,9986866	-1,17113669	True	1,17113669
ENSMUSG00000032513	Gorasp1	315,3182152	140,0813635	604,9684358	801,8418475	148,7414312	-1,170543482	True	1,170543482
ENSMUSG00000021998	Lcp1	1377,198675	425,2920677	504,5806605	247,4126318	611,927467	-1,170304136	True	1,170304136
ENSMUSG00000038271	Iffo1	119,7900082	641,0930872	269,4619234	1069,497331	344,4538407	1,169573849	True	1,169573849
ENSMUSG00000049516	Spty2d1	193,9825295	193,0851227	86,29826304	160,8182106	549,2994959	-1,168523298	True	1,168523298
ENSMUSG00000023073	Slc10a2	244,9898878	180,4651801	398,0287234	109,0864785	414,9103081	-1,167249914	True	1,167249914
ENSMUSG00000023025	Larp4	472,2044841	401,3141766	302,0439207	210,300737	1094,684744	-1,166957836	True	1,166957836
ENSMUSG00000058135	Gstm1	42847,72669	11091,66761	10333,7767	1816,233638	19084,56942	-1,166812026	True	1,166812026
ENSMUSG00000043162	Pyurf	276,6762771	22,71589679	123,2832329	34,86268902	16,96174215	-1,166222349	True	1,166222349
ENSMUSG00000057497	Fam136a	628,3179142	162,7972603	280,0290576	111,3356843	70,45646741	-1,165918175	True	1,165918175
ENSMUSG00000062232	Rapgef2	227,2145963	205,7050654	101,2683699	184,4348709	426,6530527	-1,165871883	True	1,165871883

ENSMUSG00000005481	Ddx39	94,28632906	352,0964003	211,342685	281,1507179	427,9578021	1,164463669	True	1,164463669
ENSMUSG00000020849	Ywhae	2613,740696	3177,701562	2883,066461	1166,213178	3676,783799	-1,164284491	True	1,164284491
ENSMUSG00000005897	Nr2c1	242,6713715	78,2436445	108,3131261	80,97140676	65,23746983	-1,163795832	True	1,163795832
ENSMUSG00000027360	Hdc	22,41232412	283,9487099	50,19388769	527,4387468	189,1886625	1,16321943	True	1,16321943
ENSMUSG00000038279	Nop2	153,022075	555,2774771	342,5512686	537,5601726	566,2612381	1,162580136	True	1,162580136
ENSMUSG00000062410	Hsd3b3	2878,051553	21,45390252	1285,668	30,36427753	91,33245776	-1,162574292	True	1,162574292
ENSMUSG00000026112	Coa5	971,4583248	340,7384519	434,1330988	334,0070529	266,1688769	-1,162014687	True	1,162014687
ENSMUSG00000024163	Mapk8ip3	466,021774	1042,407264	1786,726283	4114,921907	1723,573953	1,161449774	True	1,161449774
ENSMUSG00000058454	Dhcr7	1607,504627	195,6091113	3594,586834	346,3776845	306,6161082	1,161003065	True	1,161003065
ENSMUSG00000026181	Ppm1f	251,9454367	111,0554954	112,7160987	84,34521537	95,24670595	-1,160417743	True	1,160417743
ENSMUSG00000039457	Ppl	227,2145963	206,9670596	394,5063453	328,3840385	101,7704529	-1,158736753	True	1,158736753
ENSMUSG00000013663	Pten	1469,939327	533,8235746	553,0133591	368,8697419	658,8984452	-1,15762858	True	1,15762858
ENSMUSG00000030747	Dgat2	6264,631011	1559,824913	2808,215927	1885,959016	2197,197984	-1,157575666	True	1,157575666
ENSMUSG00000027351	Spred1	188,5726581	275,11475	84,537074	156,3197992	371,853578	-1,157464425	True	1,157464425
ENSMUSG00000038024	Dennd4c	235,7158227	82,0296273	66,04458906	68,60077517	105,6847011	-1,157282056	True	1,157282056
ENSMUSG00000041261	Car8	1333,146866	151,4393119	597,9236797	211,4253399	399,2533153	-1,156802471	True	1,156802471
ENSMUSG00000030827	Fgf21	17,77529154	612,0672191	39,62675344	300,2689667	883,3153414	1,156601552	True	1,156601552
ENSMUSG00000035776	Cd99l2	392,6020915	142,6053521	176,1189042	112,4602872	159,1794264	-1,156518086	True	1,156518086
ENSMUSG00000070284	Gmppb	171,5702053	76,98165023	276,5066795	305,8919811	79,58971319	-1,156212535	True	1,156212535
ENSMUSG00000031842	Pde4c	294,4515686	51,74176491	656,042918	294,6459524	9,133245776	1,155759839	True	1,155759839
ENSMUSG00000057722	Lepr	85,78510267	4756,456389	191,089011	5157,428769	4112,570098	1,155445777	True	1,155445777
ENSMUSG00000034640	Tiparp	142,9751711	989,4035047	64,28340002	383,4895792	2634,289032	-1,15324649	True	1,15324649
ENSMUSG00000053646	Plxnb1	582,7204272	1320,046002	1397,503505	2766,523064	262,2546287	-1,151835649	True	1,151835649
ENSMUSG00000021497	Txndc15	872,5349632	295,3066583	392,7451563	352,0006988	260,9498793	-1,15161946	True	1,15161946
ENSMUSG00000023176	Cpn2	5021,906281	400,0521824	2262,247324	417,2276654	271,3878745	-1,150478442	True	1,150478442
ENSMUSG00000045672	Col27a1	376,3724775	430,3400448	2314,202401	1432,744058	169,6174215	-1,149876776	True	1,149876776
ENSMUSG00000000204	Slfn4	14,68393649	111,0554954	32,58199727	212,5499427	294,8733636	1,149836264	True	1,149836264
ENSMUSG00000021779	Thrb	526,3031975	114,8414782	195,4919836	237,2912059	70,45646741	-1,149235525	True	1,149235525
ENSMUSG00000030881	Arfip2	209,4393047	508,5836893	630,5056769	1575,568623	464,4907852	1,149117763	True	1,149117763
ENSMUSG00000079659	Tmem243	703,2832742	839,226187	756,4306934	317,1380098	964,209804	-1,148995108	True	1,148995108
ENSMUSG00000039166	Akap7	89,64929649	69,40968464	95,98480277	206,9269284	40,44723129	-1,148251312	True	1,148251312
ENSMUSG00000018042	Cyb5r3	10193,74328	2383,907169	4601,986966	1104,36002	3302,320723	-1,147355123	True	1,147355123
ENSMUSG00000028691	Prdx1	12039,28225	3436,410387	5014,985796	959,2862495	5435,585986	-1,147241905	True	1,147241905
ENSMUSG00000037601	Nme1	131,3825897	301,6166296	495,7747152	346,3776845	290,9591154	1,147042337	True	1,147042337
ENSMUSG00000040548	Tex2	1840,901933	450,531953	831,2812277	668,0141057	273,9973733	-1,147004237	True	1,147004237
ENSMUSG00000030935	Acsm3	939,7719356	155,2252947	424,4465591	367,745139	121,3416939	-1,146727766	True	1,146727766
ENSMUSG00000024241	Sos1	494,6168082	2222,371903	223,6710083	802,9664503	1937,552854	-1,144932997	True	1,144932997
ENSMUSG00000006057	Atp5g1	2633,061665	4737,526475	4926,04575	1190,954441	5149,845868	-1,144623084	True	1,144623084
ENSMUSG00000022698	Naa50	611,3154614	576,7313796	372,4914823	276,6523064	1582,661018	-1,143843105	True	1,143843105
ENSMUSG00000035840	Lysmd3	410,377383	237,254922	185,8054439	214,7991485	1419,567343	-1,143158451	True	1,143158451
ENSMUSG00000030131	Mug2	8012,019454	1158,510736	2231,426516	3627,968864	1619,194001	-1,14300385	True	1,14300385
ENSMUSG00000091811	Inafm1	61,82710102	667,5949668	136,4921507	539,8093784	1035,971021	1,142506722	True	1,142506722
ENSMUSG00000020776	Fbf1	132,1554284	2458,36483	291,4767864	3275,968165	1573,527772	1,141145303	True	1,141145303
ENSMUSG00000046079	Lrrc8d	644,5475282	156,487289	146,1786905	78,72220101	292,2638648	-1,141015278	True	1,141015278
ENSMUSG00000001670	Tat	18858,81149	36562,49788	8551,453392	53770,6371	35140,8155	-1,140997214	True	1,140997214
ENSMUSG00000025812	Pard3	812,2535397	230,9449507	368,9691042	142,8245647	273,9973733	-1,138430109	True	1,138430109
ENSMUSG00000065126	Snord104	61,05426226	223,3729851	213,103874	157,444402	134,3891878	1,138253152	True	1,138253152
ENSMUSG00000029017	Pmpcb	1335,465382	382,3842626	606,7296249	409,3554453	525,8140068	-1,138216918	True	1,138216918
ENSMUSG00000066278	Vps37b	341,5947332	363,4543486	530,9984961	155,1951963	426,6530527	-1,138201828	True	1,138201828
ENSMUSG00000048787	Dcun1d3	100,4690392	155,2252947	121,5220439	121,4571101	45,66622888	-1,137551423	True	1,137551423
ENSMUSG00000032320	Rcn2	86,55794143	136,2953807	61,64161646	39,36110051	213,978901	-1,136895631	True	1,136895631
ENSMUSG00000009376	Met	923,5423215	228,4209622	420,0435864	282,2753208	253,1213829	-1,136639036	True	1,136639036
ENSMUSG00000025197	Cyp2c23	8226,095791	2019,190826	3741,646119	290,1475409	3434,100412	-1,136534755	True	1,136534755
ENSMUSG00000030096	Slc6a6	2204,136152	358,4063716	1002,997159	302,5181725	337,9300937	-1,135895823	True	1,135895823
ENSMUSG00000024959	Bad	194,7553682	262,4948074	331,1035398	219,29756	88,72295896	-1,134283709	True	1,134283709
ENSMUSG00000061838	Suclg2	2558,869144	696,6208349	1165,907146	535,3109669	871,5725969	-1,134053477	True	1,134053477
ENSMUSG00000018995	Nars2	185,4813031	35,33583945	84,537074	59,6039522	18,26649155	-1,133617684	True	1,133617684
ENSMUSG00000025508	Rplp2	1098,976721	3286,233069	3660,631423	2411,148557	3287,968479	1,133559715	True	1,133559715
ENSMUSG00000025479	Cyp2e1	91005,62851	20434,21116	41480,4049	11648,63654	26652,11592	-1,133525799	True	1,133525799
ENSMUSG00000024483	Ankhd1	386,4193814	401,3141766	641,0728112	1592,437666	176,1411685	-1,133435314	True	1,133435314
ENSMUSG00000042729	Wdr74	227,2145963	691,5728578	498,4164988	664,6402971	884,6200908	1,133296309	True	1,133296309
ENSMUSG00000026715	Serpinc1	40432,60555	7661,56719	18435,24629	3858,512453	12187,66411	-1,133052486	True	1,133052486
ENSMUSG00000026500	Cox20	389,5107365	299,0926411	857,6990633	177,6872537	241,3786384	-1,132322901	True	1,132322901
ENSMUSG00000036835	Psenen	507,7550672	700,4068177	862,1020359	231,6681916	523,204508	-1,132072759	True	1,132072759
ENSMUSG00000034930	Rtkn	151,4763975	114,8414782	405,0734796	223,7959715	69,15171802	-1,131256016	True	1,131256016
ENSMUSG00000053644	Aldh7a1	4647,852319	657,4990127	2123,11339	412,7292539	994,2190401	-1,130382806	True	1,130382806
ENSMUSG00000057228	Aadat	1058,016266	191,8231285	483,446392	308,1411868	253,1213829	-1,129933981	True	1,129933981
ENSMUSG00000031570	Plpp5	534,8044239	2139,080281	1170,310118	1379,887723	2493,376097	1,129807573	True	1,129807573
ENSMUSG00000048439	Nupl2	68,00981113	271,3287672	148,820474	314,8888041	290,9591154	1,129758229	True	1,129758229
ENSMUSG00000027901	Dennd2d	185,4813031	119,8894553	218,3874412	375,6173591	84,80871077	-1,128989409	True	1,128989409
ENSMUSG00000045039	Megf8	156,8862688	296,5686525	281,7902467	741,1132924	71,76121681	-1,12844283	True	1,12844283
ENSMUSG00000025484	Bet1l	121,3356858	378,5982798	265,0589508	998,64735	754,1451512	1,127309339	True	1,127309339
ENSMUSG00000028919	Arhgef19	1122,934722	56,78974198	2451,575146	340,7546701	13,04749397	1,12643492	True	1,12643492
ENSMUSG00000025816	Sec61a2	101,2418779	412,672125	221,0292247	1043,631465	409,6913105	1,126430964	True	1,126430964
ENSMUSG00000062376	Borcs7	108,1974268	676,4289266	235,9993316	452,0903544	441,005296	1,125116585	True	1,125116585
ENSMUSG00000046727	Cystm1	54,0987134	180,4651801	117,9996658	150,6967848	263,5593781	1,125116585	True	1,125116585
ENSMUSG00000040033	Stat2	1251,225957	2728,431603	4238,301429	3848,391027	3297,101725	1,124729531	True	1,124729531
ENSMUSG00000022974	Paxbp1	325,3651191	709,2407776	908,7735455	2647,31516	850,6966065	1,124215891	True	1,124215891
ENSMUSG00000061360	Phf5a	204,0294334	609,5432305	444,700233	464,460986	664,1174428	1,12405587	True	1,12405587
ENSMUSG00000027249	F2	25355,29413	6390,738964	11645,86254	5312,623966	7837,629625	-1,122469515	True	1,122469515
ENSMUSG00000030539	Sema4b	88,10361896	295,3066583	220,1486302	281,1507179	191,7981613	1,122315704	True	1,122315704
ENSMUSG00000025743	Sdc3	981,5052288	176,6791973	450,8643947	212,5499427	249,2071347	-1,12230237	True	1,12230237
ENSMUSG00000021340	Gpld1	3134,634022	555,2774771	1440,652636	552,18001	904,1913318	-1,121574493	True	1,121574493
ENSMUSG00000036402	Gng12	753,5177937	1010,857407	367,2079152	346,3776845	1780,982926	-1,121295594	True	1,121295594
ENSMUSG00000020521	Rnft1	448,2464824	119,8894553	206,0591179	113,58489	174,8364191	-1,121233959	True	1,121233959
ENSMUSG00000024899	Papss2	4575,978315	324,3325264	2103,74031	648,8958569	451,4432912	-1,121123589	True	1,121123589
ENSMUSG00000006456	Rbm14	119,7900082	201,9190826	107,4325315	55,10554071	405,7770623	-1,120238287	True	1,120238287
ENSMUSG00000071637	Cebpd	50,23451958	940,1857283	109,1937206	564,5506416	1517,423548	1,120138909	True	1,120138909
ENSMUSG00000031666	Rbl2	496,1624857	117,3654668	197,2531727	228,2943829	113,5131975	-1,119917296	True	1,119917296
ENSMUSG00000003348	Mob3a	102,0147167	102,2215356	177,8800932	101,2142584	46,97097827	-1,118935745	True	1,118935745
ENSMUSG00000021376	Tpmt	445,9279661	233,4689392	967,7733784	195,6808997	96,55145534	1,117858574	True	1,117858574
ENSMUSG00000043252	Tmem64	588,1302985	247,3508762	240,4023042	271,0292921	267,4736263	-1,117687033	True	1,117687033
ENSMUSG00000038508	Gdf15	288,2688585	420,2440906	132,9697726	491,4514549	384,901072	-1,11631667	True	1,11631667
ENSMUSG00000028003	Lrat	251,9454367	78,2436445	116,2384768	60,72855507	74,3707156	-1,116023624	True	1,116023624
ENSMUSG00000030956	Fam53b	207,8936272	18,92991399	95,98480277	47,23332061	28,70448672	-1,114967626	True	1,114967626
ENSMUSG00000028433	Ubap2	348,550282	984,3555276	754,6695044	1170,711589	1236,902428	1,114478182	True	1,114478182
ENSMUSG00000068587	Mgam	1466,075133	150,1773177	677,1771865	194,5562968	217,8931492	-1,114353764	True	1,114353764
ENSMUSG00000092008	Cyp2c69	18504,0785	604,4952535	2079,083664	42,73490912	8547,413297	-1,114283503	True	1,114283503
ENSMUSG00000048668	Rhno1	84,23942515	343,2624404	182,2830658	364,3713304	202,2361565	1,113613044	True	1,113613044
ENSMUSG00000036103	Colec12	711,0116618	118,627461	292,3573809	124,8309187	328,7968479	-1,112676754	True	1,112676754
ENSMUSG00000035473	Galm	1886,49942	98,43555276	874,4303592	62,97776081	319,6636021	-1,109296261	True	1,109296261
ENSMUSG00000071226	Cecr2	258,9009855	232,206945	358,40197	346,3776845	120,0369445	-1,108921956	True	1,108921956
ENSMUSG00000021877	Arf4	1646,146565	2180,726092	1333,220105	763,6053498	5369,043767	-1,108193676	True	1,108193676
ENSMUSG00000032621	Srek1	449,79216	3711,525137	969,5345675	3118,523763	3072,684829	1,108033822	True	1,108033822
ENSMUSG00000032515	Csmp1	71,10116618	473,2478498	153,2234466	393,6110051	517,9855104	1,107691951	True	1,107691951
ENSMUSG00000028893	Sesn2	146,0665262	389,9562282	67,80577811	584,7934932	540,1662502	-1,107145472	True	1,107145472
ENSMUSG00000055730	Ces2a	3948,433239	1294,806117	1824,591847	728,7426608	1833,172902	-1,106937432	True	1,106937432
ENSMUSG00000021786	Oxsm	430,4711909	129,9854094	199,8949562	89,96822973	109,5989493	-1,106674619	True	1,106674619
ENSMUSG00000071176	Arhgef10	34,77774433	215,8010195	74,85053427	182,1856652	297,4828624	1,105848251	True	1,105848251
ENSMUSG00000006517	Mvd	925,8608378	205,7050654	1991,904806	735,490278	112,2084481	1,105281433	True	1,105281433
ENSMUSG00000000753	Serpinf1	13142,12316	2955,590571	4619,598856	719,7458378	6108,836674	-1,105228793	True	1,105228793
ENSMUSG00000025492	Ifitm3	5230,572747	8423,811726	8288,15563	2431,391408	11520,93717	-1,105186771	True	1,105186771
ENSMUSG00000025026	Add3	223,3504025	56,78974198	103,9101535	82,09600963	33,92348431	-1,103972222	True	1,103972222

ENSMUSG00000085241	Snhg3	34,00490556	504,7977065	73,08934523	158,5690049	327,4920985	1,103918224	True	1,103918224
ENSMUSG00000075000	Nrbf2	164,6146565	129,9854094	76,61172331	125,9555216	328,7968479	-1,103455713	True	1,103455713
ENSMUSG00000053559	Smagp	485,342743	225,8969736	213,9844686	142,8245647	155,2651782	-1,103338978	True	1,103338978
ENSMUSG00000025027	Xpnpep1	304,4984725	318,0225551	141,7757179	316,0134069	525,8140068	-1,10282453	True	1,10282453
ENSMUSG00000027890	Gstm4	1231,132149	45,43179358	573,2670331	61,85315794	71,76121681	-1,102706407	True	1,102706407
ENSMUSG00000024513	Mbd2	676,2339175	872,0380379	479,0434193	314,8888041	1193,845698	-1,102679915	True	1,102679915
ENSMUSG00000020715	Em1	831,5745088	136,2953807	387,4615892	77,59759814	153,9604288	-1,101792238	True	1,101792238
ENSMUSG00000039956	Mrap	979,9595512	14332,46888	2102,859716	2654,062777	25403,47075	1,101558502	True	1,101558502
ENSMUSG00000062960	Kdr	387,1922202	20,19190826	180,5218768	154,0705934	16,96174215	-1,100876282	True	1,100876282
ENSMUSG00000021576	Pdcd6	484,5699043	468,1998727	298,5215426	226,0451772	1120,779732	-1,100093667	True	1,100093667
ENSMUSG00000051359	Ncald	293,6787299	26,50187959	83,65647948	32,61348328	136,9986866	-1,100076717	True	1,100076717
ENSMUSG00000007476	Lrrc8a	78,05671504	331,904492	167,312959	199,0547083	455,3575394	1,099954537	True	1,099954537
ENSMUSG00000068329	Htra2	226,4417575	181,7271743	429,7301262	481,3300291	105,6847011	-1,099373478	True	1,099373478
ENSMUSG00000034566	Atp5h	2050,341238	1995,212935	3536,467596	957,0370437	1860,572639	-1,099217364	True	1,099217364
ENSMUSG00000020827	Mink1	1193,26305	1124,436891	1658,159483	2731,660375	557,1279923	-1,098831404	True	1,098831404
ENSMUSG00000025940	Tmem70	472,2044841	182,9891686	196,3725782	147,3229762	220,502648	-1,09861576	True	1,09861576
ENSMUSG00000022223	Sdr39u1	72,6468437	109,7935012	208,7009014	132,7031389	33,92348431	-1,09861576	True	1,09861576
ENSMUSG00000032902	Slc16a1	708,6931455	1028,525327	331,1035398	337,3808615	1710,526459	-1,097878661	True	1,097878661
ENSMUSG00000091264	Smim13	252,7182754	64,36170757	95,98480277	118,0833015	58,71372284	-1,097725033	True	1,097725033
ENSMUSG00000023393	Slc17a9	169,2516891	362,1923544	765,2366386	2593,334222	558,4327417	1,097585861	True	1,097585861
ENSMUSG00000075054	Yae1d1	163,8418177	34,07384519	76,61172331	34,86268902	44,36147948	-1,096666547	True	1,096666547
ENSMUSG00000053279	Aldh1a1	12565,58544	527,5136032	5876,207238	593,7903162	1570,918273	-1,096520708	True	1,096520708
ENSMUSG00000026135	Zfp142	105,1060717	224,6349794	334,6259179	723,1196465	442,3100454	1,095736584	True	1,095736584
ENSMUSG00000019487	Trip10	142,9751711	514,8936606	305,5662987	669,1387086	772,4116427	1,095720803	True	1,095720803
ENSMUSG00000006526	Tmem110	149,1578812	1785,721887	318,7752165	435,2213113	1749,668941	1,095699261	True	1,095699261
ENSMUSG00000001632	Brpf1	204,8022721	1188,798599	437,6554769	1758,878891	1681,821972	1,095563903	True	1,095563903
ENSMUSG00000041650	Pcca	870,9892857	119,8894553	407,7152632	151,8213877	186,5791637	-1,095093006	True	1,095093006
ENSMUSG00000028456	Unc13b	183,9356255	145,1293406	273,864896	522,9403353	86,11346017	-1,094890271	True	1,094890271
ENSMUSG00000022765	Snap29	387,1922202	177,9411915	181,4024713	89,96822973	143,5224336	-1,093855855	True	1,093855855
ENSMUSG00000074211	Sdhaf1	175,4343992	199,395094	542,4462248	286,7737323	82,19921198	-1,093735191	True	1,093735191
ENSMUSG00000052738	Suclg1	1623,734241	402,5761709	760,833666	359,8729189	525,8140068	-1,093662533	True	1,093662533
ENSMUSG00000006958	Chrd	99,6962004	333,1664863	328,4617563	770,3529671	212,6741516	1,093034272	True	1,093034272
ENSMUSG00000020806	Rhbdf2	156,8862688	1255,684295	334,6259179	1204,449676	746,3166548	1,092830102	True	1,092830102
ENSMUSG00000063919	Srrm4	54,0987134	672,6429439	115,3578822	421,7260769	879,4010932	1,092450396	True	1,092450396
ENSMUSG00000006442	Srm	346,2317657	738,2666457	1163,265362	887,3116657	2433,357624	1,092403886	True	1,092403886
ENSMUSG00000090213	Tmem189	142,9751711	3420,004461	304,6857042	1535,08292	5098,960642	1,091557178	True	1,091557178
ENSMUSG00000030513	Pcsk6	1276,729636	502,2737179	392,7451563	599,4133306	541,4709996	-1,090829974	True	1,090829974
ENSMUSG00000046711	Hmga1	100,4690392	75,71965597	29,94021371	52,85633497	213,978901	1,090717565	True	1,090717565
ENSMUSG00000027243	Harbi1	189,3454969	44,16979931	88,94004661	83,2206125	30,00923612	-1,090116045	True	1,090116045
ENSMUSG00000049755	Zfp672	149,93072	129,9854094	211,342685	204,6777226	70,45646741	-1,089491967	True	1,089491967
ENSMUSG00000031379	Pir	234,1701451	40,38381652	110,0743151	39,36110051	60,01847224	-1,089079287	True	1,089079287
ENSMUSG00000049791	Fzd4	380,2366713	147,6533291	148,820474	115,8340958	178,7506673	-1,088949049	True	1,088949049
ENSMUSG00000069744	Psmb3	987,6879389	2134,032304	1688,980291	464,460986	2188,064738	-1,088497875	True	1,088497875
ENSMUSG00000010608	Rbm25	1428,978782	5427,837339	3038,051097	11950,03011	7683,669196	1,088161549	True	1,088161549
ENSMUSG00000067931	Zfp948	80,37523133	342,0004461	170,835337	242,9142203	185,2744143	1,087783535	True	1,087783535
ENSMUSG00000078348	Sf3b5	181,6171093	307,9266009	253,611222	85,46981824	374,4630768	-1,087413159	True	1,087413159
ENSMUSG00000019979	Apaf1	35,55058309	180,4651801	16,7312959	151,8213877	716,3074187	-1,087324031	True	1,087324031
ENSMUSG00000037251	Pomk	127,5183959	100,9595413	150,5816631	287,8983351	60,01847224	-1,087226885	True	1,087226885
ENSMUSG00000015755	Tab2	1449,845519	5401,335459	682,4607537	2136,745456	5470,81422	-1,087081201	True	1,087081201
ENSMUSG00000057037	Cfhr1	1971,511684	179,2031858	928,146625	160,8182106	353,5870865	-1,086877621	True	1,086877621
ENSMUSG00000054757	Akr1c20	1522,492363	8,833959863	716,80394	43,85951199	138,303436	-1,086784519	True	1,086784519
ENSMUSG00000018651	Tada2a	85,01226391	204,4430711	180,5218768	268,7800863	227,026395	1,086430797	True	1,086430797
ENSMUSG00000026869	Psmd5	469,113129	129,9854094	221,0292247	133,8277417	110,9036987	-1,085698741	True	1,085698741
ENSMUSG00000037797	Adh4	1534,084944	85,8156101	722,9681016	177,6872537	28,70448672	-1,085374469	True	1,085374469
ENSMUSG00000024732	Ccdc86	89,64929649	391,2182225	229,8351699	190,0578853	1041,190018	1,084074716	True	1,084074716
ENSMUSG00000032652	Crebl2	440,5180948	155,2252947	207,8203069	206,9269284	57,40897345	-1,083864648	True	1,083864648
ENSMUSG00000030538	Cib1	536,3501014	658,7610069	861,2214414	253,0356461	541,4709996	-1,083834387	True	1,083834387
ENSMUSG00000027495	Fam210b	1677,832954	280,1627271	791,6544742	474,5824118	181,3601661	-1,083656294	True	1,083656294
ENSMUSG00000000538	Tom1l2	431,2440296	489,6537753	361,9243481	916,5513404	203,5409059	-1,08318572	True	1,08318572
ENSMUSG00000042682	Selenok	609,7697839	959,1156423	870,9079811	287,8983351	2532,518579	-1,082705216	True	1,082705216
ENSMUSG00000034926	Dhcr24	9211,465214	685,2628865	4349,256339	1355,14646	236,1596408	-1,082661914	True	1,082661914
ENSMUSG00000008496	Pou2f2	64,14561731	334,4284805	135,6115562	675,8863258	277,9116215	1,080057521	True	1,080057521
ENSMUSG00000024002	Brd4	884,1275446	3147,4137	1868,621573	3133,1436	3860,753464	1,079648015	True	1,079648015
ENSMUSG00000061028	Clasrp	238,8071777	1017,167379	504,5806605	852,4489767	1084,246749	1,079238712	True	1,079238712
ENSMUSG00000006522	Itih3	13155,26142	40084,72388	46575,5248	88519,74123	27782,0289	1,078512044	True	1,078512044
ENSMUSG00000010097	Nxf1	756,6091488	1597,684741	2218,217598	4691,84318	2112,389273	1,078362634	True	1,078362634
ENSMUSG00000040383	Aqr	316,091054	552,7534886	149,7010685	385,738785	821,9921198	-1,078255685	True	1,078255685
ENSMUSG00000055172	C1ra	3606,065667	1314,998025	1708,35337	930,0465748	1658,336483	-1,077819244	True	1,077819244
ENSMUSG00000029771	Irf5	232,6244676	82,0296273	108,3131261	110,2110814	96,55145534	-1,077733558	True	1,077733558
ENSMUSG00000001089	Luzp1	224,89608	304,1406181	106,551937	168,6904307	327,4920985	-1,077701694	True	1,077701694
ENSMUSG00000032434	Cmtm6	711,7845005	275,11475	337,2677015	97,84044983	258,3403805	-1,077546353	True	1,077546353
ENSMUSG00000054889	Dsp	850,1226391	358,4063716	199,0143617	172,0642394	403,1675635	-1,076291407	True	1,076291407
ENSMUSG00000045466	Zfp956	61,05426226	128,7234151	143,5369069	472,3332061	173,5316697	1,076110588	True	1,076110588
ENSMUSG00000024431	Nr3c1	1213,356858	503,5357122	575,9088166	483,5792348	497,1095201	-1,075091607	True	1,075091607
ENSMUSG00000044197	Gpr146	772,065924	1461,38936	684,2219427	366,6205361	1152,093717	-1,074436439	True	1,074436439
ENSMUSG00000022788	Fgd4	274,3577608	54,26575344	130,3279891	69,72537804	56,10422405	-1,073911437	True	1,073911437
ENSMUSG00000038828	Tmem214	1337,783898	4938,183563	2814,380089	6375,373679	8342,567641	1,072972095	True	1,072972095
ENSMUSG00000040659	Efhd2	548,7155216	796,3183819	422,68537	260,9078662	1547,432784	-1,072517945	True	1,072517945
ENSMUSG00000033610	Pank1	2448,353201	151,4393119	1165,026551	707,3752062	131,779689	-1,071448862	True	1,071448862
ENSMUSG00000061751	Kalrn	403,4218342	159,0112775	191,9696055	104,5880671	56,10422405	-1,07141126	True	1,07141126
ENSMUSG00000020451	Limk2	145,2936874	157,7492833	213,103874	387,9879907	69,15171802	-1,071135024	True	1,071135024
ENSMUSG00000042485	Mustn1	74,19252123	816,5102902	155,8652302	325,0102299	583,2229802	1,070953461	True	1,070953461
ENSMUSG00000028709	Mob3c	202,4837559	1616,614655	425,3271536	957,0370437	1932,333856	1,070766789	True	1,070766789
ENSMUSG00000000346	Dazap2	2101,348596	1991,426952	818,9529044	898,5576944	4411,35771	1,069907238	True	1,069907238
ENSMUSG00000022234	Cct5	1297,596283	498,4877351	618,1773536	280,026115	588,4419778	-1,069748883	True	1,069748883
ENSMUSG00000026946	Nmi	257,355308	79,50563877	120,6414494	55,10554071	122,6464433	-1,069256142	True	1,069256142
ENSMUSG00000029478	Ncor2	485,342743	3972,75795	1017,967266	3100,530117	2082,380037	1,068615345	True	1,068615345
ENSMUSG00000048970	C1galt1c1	193,2096907	92,12558143	76,61172331	34,86268902	90,02770836	-1,068493732	True	1,068493732
ENSMUSG00000027195	Hsd17b12	1565,771333	398,7901881	746,7441537	242,9142203	610,6227176	-1,068187593	True	1,068187593
ENSMUSG00000027048	Abcb11	11544,66544	291,5206755	5508,999322	799,5926417	260,9498793	-1,067364174	True	1,067364174
ENSMUSG00000052681	Rap1b	645,3203669	681,4769037	308,2080823	449,8411486	1350,415625	-1,066110864	True	1,066110864
ENSMUSG00000015112	Slc25a13	2289,921254	364,7163429	1093,698395	440,8443257	311,8351058	-1,066083041	True	1,066083041
ENSMUSG00000022707	Gbe1	1540,267564	724,3847088	265,0589508	528,5633497	735,8786596	-1,065641269	True	1,065641269
ENSMUSG00000041763	Tpp2	632,182108	595,6612936	302,0439207	564,5506416	1212,112189	-1,065581855	True	1,065581855
ENSMUSG00000019558	Slc6a8	42,50613195	347,0484232	88,94004661	210,300737	318,3588527	1,065162181	True	1,065162181
ENSMUSG00000026811	St6galnac6	795,2510869	42,90780505	380,416833	100,0896556	33,92348431	-1,063829353	True	1,063829353
ENSMUSG00000020534	Shmt1	3442,22385	69,40968464	1646,711754	233,9173973	106,9894505	-1,063752877	True	1,063752877
ENSMUSG00000041132	N4bp2l1	726,468437	237,254922	347,8348357	259,7832633	69,15171802	-1,062497692	True	1,062497692
ENSMUSG00000024958	Gpr137	378,6909938	752,1485826	927,2660305	1262,929025	181,3601661	-1,062163493	True	1,062163493
ENSMUSG00000078812	Eif5a	3896,653042	4938,183563	4409,136766	1866,840767	7960,276068	-1,061636602	True	1,061636602
ENSMUSG00000022340	Sybu	85,01226391	217,0630138	308,2080823	690,5061632	177,4459179	1,061636501	True	1,061636501
ENSMUSG00000014313	Cox6c	2695,661605	4000,521824	3697,616393	1292,168699	4030,370886	-1,060844968	True	1,060844968
ENSMUSG00000035596	Mboat7	557,9895867	267,5427844	234,2381425	213,6745456	245,2928865	-1,060468578	True	1,060468578
ENSMUSG00000024785	Rcl1	1246,588924	1669,618414	597,9236797	1632,92337	2396,824641	-1,059952548	True	1,059952548
ENSMUSG00000028076	Cd1d1	3315,478292	1023,47735	1590,353705	606,1609478	1392,167606	-1,059869344	True	1,059869344
ENSMUSG00000028121	Bcar3	698,6462416	233,4689392	314,3722439	226,0451772	335,3205949	-1,05902104	True	1,05902104
ENSMUSG00000031834	Pik3r2	320,7280866	278,9007328	346,9542412	594,9149191	153,9604288	-1,058791104	True	1,058791104
ENSMUSG00000037600	Kdf1	105,8789105	196,8711055	141,7757179	146,1983733	50,88522646	-1,057096491	True	1,057096491
ENSMUSG00000031147	Magix	311,4540214	16,40592546	149,7010685	22,49205743	9,133245776	-1,056934681	True	1,056934681
ENSMUSG00000028712	Cyp4a31	53,32587463	186,7751514	784,6097181	380,1157706	110,9036987	1,056399853	True	1,056399853
ENSMUSG00000004032	Gstm5	180,0714317	179,2031858	295,879759	86,59442111	167,0079228	-1,05622333	True	1,05622333
ENSMUSG00000005682	Pan2	622,1352041	3372,048679	1293,593351	4103,675879	7018,247004	1,056084119	True	1,056084119
ENSMUSG00000020794	Ube2g1	556,4439092	586,8273338	324,0587837	267,6554834	1427,39584	-1,055859059	True	1,055859059

ENSMUSG00000024985	Tcf7l2	197,8467233	556,5394714	411,2376412	650,0204598	567,5659875	1,055589151	True	1,055589151
ENSMUSG00000006095	Tbcb	180,0714317	853,1081239	374,2526714	425,0998855	735,8786596	1,055443298	True	1,055443298
ENSMUSG00000001930	Vwf	51,00735834	106,0075184	144,4175014	413,8538568	143,5224336	1,055389297	True	1,055389297
ENSMUSG00000052428	Tmco1	1044,878007	175,417203	502,8194714	127,0801245	268,7783757	-1,05522209	True	1,05522209
ENSMUSG00000073700	Klhl21	181,6171093	51,74176491	79,25350688	40,48570338	87,41820957	-1,054894383	True	1,054894383
ENSMUSG00000052707	Tnrc6a	1164,668016	5195,630394	2418,112554	11248,27792	3289,273229	1,05396262	True	1,05396262
ENSMUSG00000052562	Slc22a30	2566,597531	41,64581078	1236,354707	29,23967466	195,7124095	-1,053764376	True	1,053764376
ENSMUSG00000054199	Gon4l	190,8911744	714,2887546	396,2675344	1196,577455	780,2401391	1,053724371	True	1,053724371
ENSMUSG00000039771	Polr2j	343,1404107	599,4472764	531,8790906	165,3166221	575,3944839	-1,053567247	True	1,053567247
ENSMUSG00000016487	Ppfibp1	316,091054	334,4284805	152,3428521	601,6625363	537,5567514	-1,053018394	True	1,053018394
ENSMUSG00000028180	Zranb2	570,3550069	275,11475	270,3425179	182,1856652	273,9973733	-1,051826687	True	1,051826687
ENSMUSG00000041684	Bivm	171,5702053	36,59783372	82,77588496	58,47934932	18,26649155	-1,051516603	True	1,051516603
ENSMUSG00000029311	Hsd17b11	2281,420028	624,6871617	1100,743151	777,1005843	840,2586114	-1,051454215	True	1,051454215
ENSMUSG00000032348	Gsta4	1005,46323	343,2624404	116,2384768	30,36427753	485,3667755	-1,050713062	True	1,050713062
ENSMUSG00000033917	Gde1	394,147769	1461,38936	816,3111208	1379,887723	2729,535737	1,050382503	True	1,050382503
ENSMUSG00000021785	Ngly1	399,5576404	193,0851227	170,835337	191,1824882	90,02770836	-1,049166634	True	1,049166634
ENSMUSG00000029642	Polr1d	453,6563538	437,9120103	330,2229453	219,29756	836,3443632	-1,048710105	True	1,048710105
ENSMUSG00000003135	Cnot11	250,3997591	229,6829564	153,2234466	78,72220101	517,9855104	1,048678567	True	1,048678567
ENSMUSG00000028713	Cyp4b1	164,6146565	122,4134438	264,1783563	318,2626127	79,58971319	-1,048438909	True	1,048438909
ENSMUSG00000041481	Serpina3g	30,91355051	119,8894553	77,49231784	329,5086414	63,93272043	1,048315122	True	1,048315122
ENSMUSG00000005150	Wdr83	148,3850425	123,6754381	339,9094851	182,1856652	71,76121681	-1,048069414	True	1,048069414
ENSMUSG00000028041	Adam15	66,4641336	177,9411915	137,3727453	363,2467275	186,5791637	1,047447876	True	1,047447876
ENSMUSG00000063694	Cycs	694,7820478	952,8056709	925,5048414	336,2562586	879,4010932	-1,046999357	True	1,046999357
ENSMUSG00000027162	Lin7c	355,5058309	408,8861422	251,850033	172,0642394	828,5158668	-1,046925934	True	1,046925934
ENSMUSG00000007029	Vars	364,0070573	1545,942976	752,0277208	1160,590164	923,7625727	1,046819421	True	1,046819421
ENSMUSG00000005442	Cic	595,0858474	1285,972157	1229,309951	1751,006671	1480,890565	1,046679002	True	1,046679002
ENSMUSG00000044864	Ankrd50	123,654202	167,8452374	59,88042742	85,46981824	292,2638648	-1,046154842	True	1,046154842
ENSMUSG00000027371	Fahd2a	161,5233014	78,2436445	73,96993975	44,98411486	66,54221922	-1,045696828	True	1,045696828
ENSMUSG00000003555	Cyp17a1	1453,709713	668,8569611	704,4756167	422,8506797	442,3100454	-1,045117532	True	1,045117532
ENSMUSG00000032239	Rp9	625,2265591	2004,046895	1289,190379	2339,173973	2103,256027	1,044014357	True	1,044014357
ENSMUSG00000028977	Casz1	16,22961402	127,4614209	33,46259179	93,34203834	561,0422405	1,043920502	True	1,043920502
ENSMUSG00000051989	Smim11	141,4294936	203,1810768	243,0440878	68,60077517	138,303436	-1,043786226	True	1,043786226
ENSMUSG00000001761	Smo	55,64439092	128,7234151	117,1190713	114,7094929	129,1701903	1,043676615	True	1,043676615
ENSMUSG00000042211	Fbxo38	355,5058309	311,7125837	172,5965261	468,9593975	566,2612381	-1,042469795	True	1,042469795
ENSMUSG00000021466	Ptch1	330,0021517	35,33583945	160,2682028	56,23014358	35,22823371	-1,041987208	True	1,041987208
ENSMUSG00000045294	Insig1	9393,855162	596,9232879	4562,360213	378,9911677	408,3865611	-1,041936993	True	1,041936993
ENSMUSG00000011148	Adssl1	276,6762771	64,36170757	126,805611	105,7126699	134,3891878	-1,041781877	True	1,041781877
ENSMUSG00000022016	Akap11	358,5971859	406,3621537	191,089011	174,3134451	678,4696862	-1,040680315	True	1,040680315
ENSMUSG00000022512	Cldn1	1391,109773	85,8156101	676,296592	89,96822973	76,98021439	-1,040508279	True	1,040508279
ENSMUSG00000031954	Cfdp1	557,216748	695,3588406	513,3866057	271,0292921	876,7915945	-1,039789839	True	1,039789839
ENSMUSG00000049940	Pgrmc2	1027,102716	402,5761709	431,4913152	258,6586605	499,7190189	-1,039391434	True	1,039391434
ENSMUSG00000039242	B3galnt2	222,5775637	27,76387386	108,3131261	24,74126318	19,57124095	-1,039100085	True	1,039100085
ENSMUSG00000027428	Rbbp9	323,8194416	124,9374323	157,6264192	80,97140676	92,63720715	-1,038680245	True	1,038680245
ENSMUSG00000033788	Dysf	54,87155216	172,8932145	112,7160987	291,2721437	167,0079228	1,038563292	True	1,038563292
ENSMUSG00000010122	Slc47a1	2419,758166	73,19566744	4969,194881	304,7673782	243,9881371	1,038149252	True	1,038149252
ENSMUSG00000006307	Kmt2b	398,0119628	1875,323479	817,1917154	2757,526241	3824,220481	1,037862784	True	1,037862784
ENSMUSG00000018446	C1qbp	664,641336	808,9383246	834,8036058	323,885627	1262,997416	-1,037091574	True	1,037091574
ENSMUSG00000037379	Spon2	149,93072	12,61994266	73,08934523	22,49205743	16,96174215	-1,036562999	True	1,036562999
ENSMUSG00000035941	Ibtk	664,641336	1279,662186	324,0587837	1971,428834	2198,502733	-1,036320482	True	1,036320482
ENSMUSG00000044393	Dsg2	2957,653945	519,9416376	1442,413825	633,1514167	1084,246749	-1,035968133	True	1,035968133
ENSMUSG00000027599	Armc1	417,3329319	156,487289	193,7307946	166,441225	203,5409059	-1,035880005	True	1,035880005
ENSMUSG00000031327	Chic1	201,7109171	98,43555276	97,74599182	55,10554071	80,89446258	-1,035037784	True	1,035037784
ENSMUSG00000034259	Exosc4	191,6640132	459,3659129	603,2072468	474,5824118	392,7295683	1,03495674	True	1,03495674
ENSMUSG00000068917	Clk2	377,918155	995,713476	774,0425838	1754,38048	960,2955558	1,034339112	True	1,034339112
ENSMUSG00000029270	Fam69a	151,4763975	11,3579484	73,96993975	29,23967466	20,87599034	-1,03408201	True	1,03408201
ENSMUSG00000018548	Trim37	135,2467835	44,16979931	66,04458906	46,10871774	31,31398552	-1,03408201	True	1,03408201
ENSMUSG00000022338	Eny2	554,8982317	1044,931252	551,2521701	271,0292921	1518,728298	-1,033774423	True	1,033774423
ENSMUSG00000020003	Pex7	785,9770218	212,0150367	383,9392111	183,3102681	285,7401178	-1,033609228	True	1,033609228
ENSMUSG00000021583	Erap1	1394,973967	252,3988532	681,5801591	256,4094547	540,1662502	-1,033282954	True	1,033282954
ENSMUSG00000026005	Rpe	385,6465426	124,9374323	188,4472275	65,22696655	135,6939372	-1,033118608	True	1,033118608
ENSMUSG00000040385	Ppp1ca	2095,165886	892,2299462	1024,131428	844,5767566	996,8285389	-1,032663605	True	1,032663605
ENSMUSG00000025813	Homer2	495,389647	73,19566744	242,1634932	50,60712922	46,97097827	-1,032582327	True	1,032582327
ENSMUSG00000026179	Pnkd	3105,266149	54,26575344	1518,144954	65,22696655	56,10422405	-1,032407378	True	1,032407378
ENSMUSG00000031549	Ido2	1291,413573	30,28786239	631,3862715	38,23649764	30,00923612	-1,032356298	True	1,032356298
ENSMUSG00000025428	Atp5a1	9472,684716	3811,222684	3362,109881	2822,753208	4633,165107	-1,031775272	True	1,031775272
ENSMUSG00000054715	Zscan22	82,69374762	78,2436445	176,9994987	190,0578853	40,44723129	-1,031737303	True	1,031737303
ENSMUSG00000025246	Tbl1x	609,7697839	191,8231285	298,5215426	185,5594738	211,3694022	-1,030429616	True	1,030429616
ENSMUSG00000040505	Abcg5	705,6017904	1696,120294	429,7301262	3216,364213	345,7585901	-1,029089127	True	1,029089127
ENSMUSG00000079037	Pmp	364,779896	73,19566744	178,7606877	31,48888041	142,2176842	-1,028996721	True	1,028996721
ENSMUSG00000022721	Trmt2a	162,2961402	492,1777638	331,1035398	536,4355698	472,3192815	1,028653745	True	1,028653745
ENSMUSG00000051234	Rnf7	286,723181	367,2403314	230,7157645	140,575359	499,7190189	-1,028314818	True	1,028314818
ENSMUSG00000032372	Plscr2	439,745256	42,90780505	215,7456576	102,3388613	75,675465	-1,027336492	True	1,027336492
ENSMUSG00000039063	Echdc3	1864,859935	69,40968464	914,9377072	121,4571101	140,9129348	-1,02732185	True	1,02732185
ENSMUSG00000028136	Snx27	377,918155	136,2953807	173,4771206	185,5594738	108,2941999	-1,026192166	True	1,026192166
ENSMUSG00000042677	Zc3h12a	36,32342185	244,8268876	73,96993975	133,8277417	765,8878958	1,026038982	True	1,026038982
ENSMUSG00000027829	Ccnl1	532,4859076	3643,377446	1084,011855	2481,998538	7508,832777	1,025565285	True	1,025565285
ENSMUSG00000032252	Glce	231,0787901	74,4576617	66,04458906	58,47934932	113,5131975	-1,025524805	True	1,025524805
ENSMUSG00000040940	Arhgef1	593,5401698	2203,441989	1208,175683	3746,052165	1914,067365	1,025412677	True	1,025412677
ENSMUSG00000030203	Dusp16	460,6119026	431,602039	226,3127919	748,9855125	568,8707369	-1,02523356	True	1,02523356
ENSMUSG00000036167	Pphln1	176,2072379	117,3654668	141,7757179	86,59442111	424,0435539	-1,024927199	True	1,024927199
ENSMUSG00000084883	Ccdc85c	265,0836956	17,66791973	130,3279891	35,98729189	35,22823371	-1,024300989	True	1,024300989
ENSMUSG00000079215	Zfp664	272,0392445	133,7713922	108,3131261	113,58489	71,76121681	-1,02404517	True	1,02404517
ENSMUSG00000040720	Virma	302,1799563	424,0300734	148,820474	309,2657897	588,4419778	-1,02183495	True	1,02183495
ENSMUSG00000043964	Orai3	171,5702053	54,26575344	84,537074	40,48570338	23,48548914	-1,021142954	True	1,021142954
ENSMUSG00000020467	Efemp1	174,6615604	46,69378785	67,80577811	26,99046892	86,11346017	-1,02025147	True	1,02025147
ENSMUSG00000023170	Gps2	407,286028	1561,086907	825,9976606	1238,187762	2084,989536	1,020095372	True	1,020095372
ENSMUSG00000021518	Ptdss1	587,3574597	273,8527558	269,4619234	240,6650145	289,654366	-1,019906369	True	1,019906369
ENSMUSG00000054226	Tprkb	2133,807824	772,3404909	1052,310452	571,2982588	696,7361777	-1,019869858	True	1,019869858
ENSMUSG00000024299	Adamts10	137,5652998	288,9966869	379,5362385	1060,500508	67,84696862	-1,01976034	True	1,01976034
ENSMUSG00000048047	Zbtb33	201,7109171	75,71965597	99,50718086	22,49205743	66,54221922	-1,019416623	True	1,019416623
ENSMUSG00000039943	Plcb4	30,91355051	175,417203	63,4028055	452,0903544	62,62797103	1,018567779	True	1,018567779
ENSMUSG00000030967	Zranb1	646,0932057	1383,145716	1307,682863	2622,573897	1642,67949	1,017198495	True	1,017198495
ENSMUSG00000021470	Ercc6l2	190,1183356	410,1481365	191,089011	396,9848137	93,94195655	-1,017056131	True	1,017056131
ENSMUSG00000031521	Aga	158,4319464	71,93367317	76,61172331	31,48888041	78,28496379	-1,017056131	True	1,017056131
ENSMUSG00000028016	Ints12	144,5208486	112,3174897	44,02972604	41,61030625	292,2638648	1,015993835	True	1,015993835
ENSMUSG00000009145	Dqx1	267,4022119	87,07760436	540,6850358	232,7927944	120,0369445	1,015777029	True	1,015777029
ENSMUSG00000043257	Pigv	121,3356858	212,0150367	190,2084165	173,1888422	60,01847224	-1,01552542	True	1,01552542
ENSMUSG00000006215	Zbtb17	112,8344594	350,834406	228,0739809	377,8665649	441,005296	1,01529414	True	1,01529414
ENSMUSG00000038578	Susd1	135,2467835	266,2807902	66,92518359	122,581713	136,9986866	-1,014973187	True	1,014973187
ENSMUSG00000015087	Rabl6	355,5058309	718,0747374	759,072477	1433,868661	1428,700589	1,014260785	True	1,014260785
ENSMUSG00000024805	Pcgf5	165,3874952	181,7271743	81,89529044	159,6936078	293,5686142	-1,013997764	True	1,013997764
ENSMUSG00000061825	Ces2c	256,5824693	116,1034725	88,94004661	23,6166603	517,9855104	1,013489139	True	1,013489139
ENSMUSG00000046562	Unc119b	337,7305393	343,2624404	167,312959	185,5594738	784,1543873	-1,01332345	True	1,01332345
ENSMUSG00000059278	Naa38	284,4046647	235,9929278	679,8189701	200,1793111	140,9129348	-1,013141081	True	1,013141081
ENSMUSG00000031578	Mak16	145,2936874	571,6834026	293,2379754	554,4292157	476,2335297	1,013099927	True	1,013099927
ENSMUSG00000038145	Snrk	847,031284	571,6834026	763,4754496	1709,396365	139,6081854	1,0129998	True	1,0129998
ENSMUSG00000032310	Cyp1a2	10352,94807	2635,044028	4027,839338	131,578536	5132,884126	-1,012200045	True	1,012200045
ENSMUSG00000043079	Synpo	134,4739447	1008,333419	271,2231124	1282,047274	621,0607127	1,012153456	True	1,012153456
ENSMUSG00000028069	Gpatch4	137,5652998	427,8160562	277,3872741	278,9015122	474,9287803	1,011784999	True	1,011784999
ENSMUSG00000026870	Cutal	788,2955381	68,14769037	390,9839673	50,60712922	30,00923612	-1,011627159	True	1,011627159
ENSMUSG00000040302	Rbm48	60,2814235	191,8231285	121,5220439	143,9491676	622,3654621	1,011432649	True	1,011432649
ENSMUSG00000033453	Adamts15	10,04690392	367,2403314	20,25367398	277,7769093	237,4643902	1,011432649	True	1,011432649
ENSMUSG00000022790	Igsf11	275,1305996	349,5724117	136,4921507	247,4126318	613,2322164	-1,011298614	True	1,011298614

ENSMUSG00000031901	Dus2	113,6072981	386,1702454	228,9545754	1002,021159	238,7691396	1,01100588	True	1,01100588
ENSMUSG00000020994	Pnn	734,1968247	947,7576939	657,8041071	364,3713304	1379,120112	-1,010757426	True	1,010757426
ENSMUSG00000090946	Ccdc71l	60,2814235	151,4393119	29,94021371	52,85633497	254,4261323	-1,009628966	True	1,009628966
ENSMUSG00000030685	Kctd13	46,37032577	251,136859	93,34301921	269,9046892	185,2744143	1,00934027	True	1,00934027
ENSMUSG00000035614	Togaram1	163,068979	137,557375	81,01469592	172,0642394	531,0330044	-1,009226821	True	1,009226821
ENSMUSG00000001909	Trmt1	377,1453162	896,015929	759,072477	1325,906786	1420,872093	1,009117133	True	1,009117133
ENSMUSG00000028755	Cda	188,5726581	837,9641927	379,5362385	476,8316176	934,2005679	1,009117133	True	1,009117133
ENSMUSG00000021910	Nisch	2252,824994	5248,634153	4533,300593	10786,06614	5224,216584	1,008826581	True	1,008826581
ENSMUSG00000036513	Commd2	132,9282672	87,07760436	81,01469592	48,35792348	267,4736263	1,008748718	True	1,008748718
ENSMUSG00000040918	Slc19a2	2072,753562	619,6391847	823,355877	1030,13623	460,576537	-1,008713459	True	1,008713459
ENSMUSG00000035212	Leprot	200,1652396	254,9228418	99,50718086	112,4602872	467,100284	-1,008318914	True	1,008318914
ENSMUSG00000005732	Ranbp1	92,74065154	121,1514495	106,551937	46,10871774	238,7691396	-1,008162317	True	1,008162317
ENSMUSG00000019857	Asf1a	92,74065154	103,4835298	81,01469592	46,10871774	182,6649155	-1,008162317	True	1,008162317
ENSMUSG00000038374	Rbm8a	395,6934466	584,3033452	507,222444	196,8055025	1026,837775	-1,007612614	True	1,007612614
ENSMUSG00000050846	Zfp623	147,6122037	421,5060849	296,7603535	581,4196846	794,5923825	1,007486364	True	1,007486364
ENSMUSG00000086054	Hnf1aos1	207,1207884	1,261994266	103,0295589	5,623014358	6,523746983	-1,00741406	True	1,00741406
ENSMUSG00000024953	Prdx5	1950,645037	2199,656006	2896,275379	970,5322782	2131,960514	-1,007103171	True	1,007103171
ENSMUSG00000068874	Selenbp1	4511,832697	570,4214083	2245,516028	849,0751681	476,2335297	-1,006666552	True	1,006666552
ENSMUSG00000061983	Rps12	1714,929215	4760,242372	4596,703399	3444,658596	4059,075373	1,006211974	True	1,006211974
ENSMUSG00000032733	Snx33	322,2737641	537,6095574	253,611222	347,5022873	160,4841758	-1,005855695	True	1,005855695
ENSMUSG00000017707	Serinc3	2579,73579	3608,041607	1284,787406	3868,633878	4246,959286	-1,00569366	True	1,00569366
ENSMUSG00000062785	Kcnc3	164,6146565	82,0296273	339,9094851	274,4031007	281,8258696	-1,004875813	True	1,004875813
ENSMUSG00000022253	Nadk2	1920,504326	263,7568016	958,0868387	336,2562586	233,550142	-1,003256884	True	1,003256884
ENSMUSG00000024965	Fermt3	70,32832742	219,5870023	140,8951233	287,8983351	195,7124095	1,002443866	True	1,002443866
ENSMUSG00000029640	Usp12	184,7084643	49,21777638	72,20875071	92,21743547	49,58047707	-1,002138529	True	1,002138529
ENSMUSG00000020123	Avpr1a	700,1919191	271,3287672	349,5960248	84,34521537	74,3707156	-1,002061632	True	1,002061632
ENSMUSG00000040213	Kyat3	4266,842809	1038,621281	2131,03874	1047,005273	1623,108249	-1,001612142	True	1,001612142
ENSMUSG00000020919	Stat5b	789,8412156	189,2991399	394,5063453	309,2657897	148,7414312	-1,001514148	True	1,001514148
ENSMUSG00000021248	Tmed10	1746,615604	2108,792419	1941,710918	872,6918284	3685,917045	-1,001017941	True	1,001017941
ENSMUSG00000031634	Ufsp2	481,4785492	117,3654668	235,1187371	240,6650145	120,0369445	-1,000445092	True	1,000445092
ENSMUSG00000074151	Nlrc5	280,5404709	638,5690987	723,8486961	809,7140676	561,0422405	0,999900485	False	0,999900485
ENSMUSG00000059498	Fcgr3	107,424588	200,6570883	53,71626577	137,2015503	176,1411685	-0,999893324	False	0,999893324
ENSMUSG00000031755	Bbs2	37,86909938	159,0112775	75,73112879	274,4031007	147,4366818	0,999865322	False	0,999865322
ENSMUSG00000020091	Eif4ebp2	3437,586817	647,4030585	1719,801099	930,0465748	340,5395925	-0,999154427	False	0,999154427
ENSMUSG00000053091	Lins1	50,23451958	169,1072317	100,3877754	218,1729571	126,5606915	0,998832612	False	0,998832612
ENSMUSG00000021250	Fos	174,6615604	40,38381652	15,85070138	23,6166603	87,41820957	-0,998556399	False	0,998556399
ENSMUSG00000035048	Anapc13	407,286028	301,6166296	813,6693373	182,1856652	206,1504046	0,998400301	False	0,998400301
ENSMUSG00000016756	Cmah	2164,721375	371,0263142	1084,011855	282,2753208	100,4657035	-0,99780081	False	0,99780081
ENSMUSG00000041548	Hspb8	1847,084693	2487,390699	2131,03874	925,5481633	3856,839216	-0,996870008	False	0,996870008
ENSMUSG00000035242	Oaz1	2284,511383	887,1819691	1144,772877	417,2276654	1062,066009	-0,996824235	False	0,996824235
ENSMUSG00000057649	Brd9	180,0714317	503,5357122	359,2825645	698,3783833	490,5857731	0,996549609	False	0,996549609
ENSMUSG00000026095	Asnsd1	93,5134903	217,0630138	219,2680357	202,4285169	186,5791637	0,996541473	False	0,996541473
ENSMUSG00000025464	Paox	765,8832139	49,21777638	383,9392111	46,10871774	24,79023853	-0,996246511	False	0,996246511
ENSMUSG00000000787	Ddx3x	1870,269806	1388,193693	869,1467921	1295,542508	3730,278525	0,996036946	False	0,996036946
ENSMUSG00000028565	Nfia	942,0904519	4602,493089	2072,919502	2867,737323	1878,839131	0,995904059	False	0,995904059
ENSMUSG00000062761	Zfp512	78,05671504	217,0630138	155,8652302	526,3141439	39,1424819	-0,995787514	False	0,995787514
ENSMUSG00000023206	Il15ra	519,3476486	1646,902517	1035,579157	1393,382958	1249,949922	0,995665333	False	0,995665333
ENSMUSG00000004535	Tax1bp1	1796,850124	1530,799045	1099,862557	744,487101	3582,841843	0,995634282	False	0,995634282
ENSMUSG00000027193	Api5	724,1499207	863,204078	664,8488632	363,2467275	1719,659705	-0,995338609	False	0,995338609
ENSMUSG00000040212	Emp3	56,41722968	162,7972603	139,1339343	112,4602872	164,398424	0,995207905	False	0,995207905
ENSMUSG00000039740	Alg2	201,7109171	191,8231285	296,7603535	101,2142584	337,9300937	-0,994876626	False	0,994876626
ENSMUSG00000049422	Chchd10	9427,087229	5521,224914	18785,72291	3084,785677	6513,308987	0,994752652	False	0,994752652
ENSMUSG00000022514	Il1rap	1544,131848	320,5465436	774,9231784	100,0896556	579,3087321	-0,994670743	False	0,994670743
ENSMUSG00000035847	Ids	201,7109171	36,59783372	101,2683699	43,85951199	43,05673008	-0,994105534	False	0,994105534
ENSMUSG00000005413	Hmox1	413,4687381	353,3583945	324,0587837	150,6967848	823,2968692	0,993634487	False	0,993634487
ENSMUSG00000022386	Trmu	76,51103752	222,1109908	152,3428521	222,6713686	204,8456553	0,993582019	False	0,993582019
ENSMUSG00000036181	Hist1h1c	639,9104956	46,69378785	321,4170001	116,9586986	180,0554167	-0,993423892	False	0,993423892
ENSMUSG00000034574	Daam1	641,4561731	114,8414782	322,2975946	188,9332824	71,76121681	-0,992957277	False	0,992957277
ENSMUSG00000041775	Mapk1ip1	310,6811826	767,2925138	618,1773536	1199,951264	711,0884211	0,99258594	False	0,99258594
ENSMUSG00000035967	Ints6l	78,82955381	306,6646067	156,7458247	642,1482397	290,9591154	0,9916185	False	0,9916185
ENSMUSG00000038500	Prr3	75,73819875	477,0338326	150,5816631	337,3808615	765,8878958	0,991453082	False	0,991453082
ENSMUSG00000060090	Rp2	178,5257542	66,88569611	89,82064113	69,72537804	40,44723129	-0,991013288	False	0,991013288
ENSMUSG00000021611	Tert	198,619562	22,71589679	394,5063453	95,59124409	24,79023853	0,990040784	False	0,990040784
ENSMUSG00000024737	Slc15a3	99,6962004	142,6053521	50,19388769	114,7094929	245,2928865	-0,99002683	False	0,99002683
ENSMUSG00000037563	Rps16	1997,788202	6296,089394	7113,442539	3967,598931	5089,827396	0,989862555	False	0,989862555
ENSMUSG00000086859	Snhg20	74,19252123	283,9487099	227,1933864	147,3229762	272,6926239	0,989636775	False	0,989636775
ENSMUSG00000073643	Wdfy1	132,1554284	965,4256136	262,4171672	1764,501906	519,2902598	0,989626417	False	0,989626417
ENSMUSG00000036775	Decr2	1813,079738	230,9449507	913,1765181	403,7324309	386,2058214	-0,989476708	False	0,989476708
ENSMUSG00000046598	Bdh1	2878,824391	227,1589679	1451,21977	338,5054644	186,5791637	-0,988213772	False	0,988213772
ENSMUSG00000025532	Crcp	298,3157624	2014,142849	591,759518	1053,752891	2681,26001	0,988170797	False	0,988170797
ENSMUSG00000024135	Srbd1	181,6117093	53,00375918	91,58183017	50,60712922	22,18073974	-0,987766817	False	0,987766817
ENSMUSG00000025821	Zfp282	59,50858474	118,627461	117,9996658	175,438048	131,779689	0,987613061	False	0,987613061
ENSMUSG00000071335	Mfsd4b3	188,5726581	12,61994266	95,10420825	51,73173209	3,91424819	-0,987539424	False	0,987539424
ENSMUSG00000034158	Lrrc58	479,9328717	488,391781	325,8199727	114,7094929	951,1623101	0,986858919	False	0,986858919
ENSMUSG00000018381	Abi3	74,96535999	71,93367317	146,1786905	194,5562968	37,8377325	-0,986398474	False	0,986398474
ENSMUSG00000066366	Serpina1a	39400,09297	18748,18682	78051,49536	11871,30791	28846,70441	0,986227239	False	0,986227239
ENSMUSG00000031353	Rbbp7	630,6364304	238,5169163	162,9099864	147,3229762	318,3588527	-0,986154631	False	0,986154631
ENSMUSG00000020171	Yeats4	227,2145963	405,1001594	162,0293918	114,7094929	640,6319537	-0,986070728	False	0,986070728
ENSMUSG00000034285	Nipsnap1	3849,509878	179,2031858	1944,352702	382,3649764	323,5778503	-0,985384828	False	0,985384828
ENSMUSG00000027533	Fabp5	1530,993589	773,6024851	465,8345015	113,58489	606,7084694	-0,984803907	False	0,984803907
ENSMUSG00000039756	Dnttip2	319,182409	256,184836	176,9994987	139,4507561	631,4987079	0,984398639	False	0,984398639
ENSMUSG00000024143	Rhoq	102,7875555	118,627461	51,95507673	169,8150336	155,2651782	-0,984328975	False	0,984328975
ENSMUSG00000015126	Tsr3	152,2492363	465,6758842	301,1633261	445,3427372	540,1662502	0,984111107	False	0,984111107
ENSMUSG00000037075	Rnf139	449,0193212	196,8711055	214,8650631	103,4634642	227,026395	-0,983917484	False	0,983917484
ENSMUSG00000026014	Raph1	1279,820991	1360,429819	1328,817132	2729,411169	647,1557007	-0,983757274	False	0,983757274
ENSMUSG00000021661	Ankra2	130,6097509	301,6166296	66,04458906	74,22378953	234,8548914	-0,983750334	False	0,983750334
ENSMUSG00000038705	Gmeb2	218,7133699	517,4176491	432,3719097	1048,129876	580,6134814	0,983231383	False	0,983231383
ENSMUSG00000032244	Fem1b	167,7060115	148,9153234	66,04458906	114,7094929	331,4063467	-0,982666828	False	0,982666828
ENSMUSG00000036104	Rab3gap1	360,1428635	384,9082512	182,2830658	483,5792348	503,6332671	-0,982388776	False	0,982388776
ENSMUSG00000045776	Lrtm1	159,2047851	27,76387386	314,3722439	128,2047274	10,43799517	0,981590148	False	0,981590148
ENSMUSG00000020752	Recql5	224,1232412	198,1330998	449,1032056	377,8665649	113,5131975	-0,981432221	False	0,981432221
ENSMUSG00000028240	Cyp7a1	2045,704205	6,309971331	4038,406473	5,623014358	14,35224336	0,981188571	False	0,981188571
ENSMUSG00000030315	Vgll4	346,2317657	124,9374323	123,2832329	175,438048	147,4366818	-0,980776429	False	0,980776429
ENSMUSG00000028641	P3h1	189,3454969	373,5503028	419,1629919	1315,78536	868,9630981	0,980281421	False	0,980281421
ENSMUSG00000029664	Tfpi2	1122,161884	363,4543486	568,8640605	363,2467275	550,6042453	-0,980124972	False	0,980124972
ENSMUSG00000021716	Srek1ip1	265,0836956	272,5907615	361,9243481	474,5824118	134,3891878	-0,980030866	False	0,980030866
ENSMUSG00000036246	Gmip	97,37768411	377,3362856	191,9696055	319,3872155	247,9023853	0,979214813	False	0,979214813
ENSMUSG00000024217	Snrpc	217,1676923	230,9449507	149,7010685	110,2110814	390,1200696	-0,978540202	False	0,978540202
ENSMUSG00000056917	Sipa1	239,5800165	690,3108636	471,9986632	643,2728426	645,8509513	0,978275197	False	0,978275197
ENSMUSG00000022414	Tab1	54,0987134	164,0592546	106,551937	255,2848519	227,026395	0,977890632	False	0,977890632
ENSMUSG00000028843	Sh3bgrl3	155,3405913	442,9599874	331,1035398	305,8919811	387,5105708	0,977587424	False	0,977587424
ENSMUSG00000030744	Rps3	2590,555533	3132,269769	3923,929185	1315,78536	3248,825997	-0,977337345	False	0,977337345
ENSMUSG00000054814	Usp46	128,2912346	36,59783372	65,16399454	43,85951199	43,05673008	-0,977275654	False	0,977275654
ENSMUSG00000043384	Gprasp1	143,7480099	75,71965597	250,9694384	363,2467275	73,0659662	-0,976270516	False	0,976270516
ENSMUSG00000029368	Alb	2100076,503	693860,8534	1067595,812	133907,5885	1014549,645	-0,976076333	False	0,976076333
ENSMUSG00000025732	Mcrip2	333,0935068	191,8231285	655,1623235	377,8665649	75,675465	0,975925163	False	0,975925163
ENSMUSG00000006356	Crip2	1092,794011	463,1518957	2148,650631	657,8926799	579,3087321	0,975409439	False	0,975409439
ENSMUSG00000053841	Txlna	306,8169888	608,2812363	603,2072468	1248,309187	1165,141211	0,975275392	False	0,975275392
ENSMUSG00000055436	Srsf11	1459,892423	2869,774961	3251,154971	9303,839557	4258,70203	0,975075546	False	0,975075546
ENSMUSG00000000420	Galnt1	462,1575802	188,0371457	235,1187371	185,5594738	230,9406432	-0,974995331	False	0,974995331
ENSMUSG00000030753	Thap12	394,9206078	135,0333865	138,2533398	110,2110814	200,9314071	-0,974859567	False	0,974859567
ENSMUSG00000027714	Exosc9	141,4294936	153,9633005	91,58183017	71,97458378	255,7308817	-0,974523564	False	0,974523564
ENSMUSG00000028282	Casp8ap2	65,69129484	162,7972603	33,46259179	84,34521537	210,0646528	-0,973153011	False	0,973153011

ENSMUSG00000042251	Pm20d1	2091,301692	502,2737179	1065,51937	492,5760578	414,9103081	-0,97284438	False	0,97284438
ENSMUSG00000021268	Meg3	13,91109773	160,2732718	27,29843015	103,4634642	431,8720502	0,97258172	False	0,97258172
ENSMUSG00000019254	Ppp1r12c	357,8243472	1261,994266	701,8338331	2140,119265	1583,965767	0,971877942	False	0,971877942
ENSMUSG00000029735	Tpk1	169,2516891	11,3579484	86,29826304	58,47934932	19,57124095	-0,971766804	False	0,971766804
ENSMUSG00000032548	Slco2a1	2193,316409	543,9195287	1119,235636	519,5665267	614,5369658	-0,970600148	False	0,970600148
ENSMUSG00000036270	Edc4	281,3133097	1616,614655	551,2521701	2164,860528	2819,563446	0,970534618	False	0,970534618
ENSMUSG00000021981	Cab39l	1100,522398	126,1994266	561,8193043	200,1793111	113,5131975	-0,970010406	False	0,970010406
ENSMUSG00000021420	Fars2	334,6391843	155,2252947	170,835337	93,34203834	120,0369445	-0,969999961	False	0,969999961
ENSMUSG00000022489	Pde1b	24,73084041	344,5244347	48,43269865	557,8030243	227,026395	0,969670125	False	0,969670125
ENSMUSG00000033249	Hsf4	112,8344594	220,8489966	289,7155974	222,6713686	224,4168962	0,968852547	False	0,968852547
ENSMUSG00000036904	Fzd8	293,6787299	55,52774771	119,7608548	38,23649764	150,0461806	-0,968832184	False	0,968832184
ENSMUSG00000044533	Rps2	8985,796295	11392,02224	12826,73979	4591,753525	13500,24201	-0,968601153	False	0,968601153
ENSMUSG00000067369	Trmt2b	375,5996387	89,6015929	191,9696055	86,59442111	58,71372284	-0,968317767	False	0,968317767
ENSMUSG00000026239	Pde6d	102,0147167	153,9633005	144,4175014	79,84680388	52,18997586	-0,966932651	False	0,966932651
ENSMUSG00000030224	Strap	874,8534795	947,7576939	619,0579482	447,5919429	1855,353642	-0,966857348	False	0,966857348
ENSMUSG00000022536	Glyr1	1197,900082	2310,711501	612,8937865	3124,146777	4304,368259	-0,966798593	False	0,966798593
ENSMUSG00000046364	Rpl27a	2586,691339	4164,581078	4445,241141	1323,65758	4832,791765	-0,966577956	False	0,966577956
ENSMUSG00000028085	Gatb	478,3871942	103,4835298	244,8052768	125,9555216	153,9604288	-0,966544115	False	0,966544115
ENSMUSG00000033068	Entpd6	124,4270408	59,31373051	243,0440878	155,1951963	39,1424819	0,96591799	False	0,96591799
ENSMUSG00000040583	Cyp2b13	3415,947332	61,83771904	1748,860718	300,2689667	140,9129348	-0,965870335	False	0,965870335
ENSMUSG00000041426	Hibch	949,0460007	90,86358716	486,0881755	224,9205743	39,1424819	-0,965259977	False	0,965259977
ENSMUSG00000070283	Ndufaf3	137,5652998	79,50563877	268,5813289	65,22696655	54,79947465	0,965242413	False	0,965242413
ENSMUSG00000029381	Shroom3	155,3405913	265,0187959	272,1037069	266,5308806	79,58971319	-0,96478098	False	0,96478098
ENSMUSG00000022898	Dscr3	272,0392445	94,64956996	114,4772877	139,4507561	95,24670595	-0,964059034	False	0,964059034
ENSMUSG00000025907	Rb1cc1	510,0735834	444,2219817	261,5365727	482,4546319	922,4578233	-0,963692682	False	0,963692682
ENSMUSG00000029095	Ablim2	61,05426226	76,98165023	209,581496	262,0324691	31,31398552	-0,963284874	False	0,963284874
ENSMUSG00000049553	Polr1a	253,4911142	845,5361583	494,0135262	1515,964671	734,5739102	0,962615367	False	0,962615367
ENSMUSG00000032388	Spg21	333,0935068	439,1740046	282,6708412	170,9396365	742,4024066	-0,962440271	False	0,962440271
ENSMUSG00000026526	Fh1	1064,9718051	1383,145716	697,4308605	546,5569956	1847,525145	-0,962371395	False	0,962371395
ENSMUSG00000013646	Sh3bp5l	199,3924008	65,62370184	94,22361373	102,3388613	43,05673008	-0,962256341	False	0,962256341
ENSMUSG00000021493	Pdlim7	95,83200659	832,9162157	186,6860384	869,3180198	457,9670382	0,962034556	False	0,962034556
ENSMUSG00000012017	Scarf2	127,5183959	306,6646067	248,3276549	522,9403353	541,4709996	0,96153955	False	0,96153955
ENSMUSG00000034297	Med13	299,86144	357,1443773	196,3725782	154,0705934	784,1543873	-0,960704485	False	0,960704485
ENSMUSG00000029405	G3bp2	860,169543	1361,691813	580,3117892	441,9689285	1906,238868	-0,960676101	False	0,960676101
ENSMUSG00000024664	Fads3	71,10116618	140,0813635	198,1337672	148,4475791	36,5329831	-0,960673661	False	0,960673661
ENSMUSG00000038886	Man2a2	563,3994581	386,1702454	512,5060111	1265,178231	289,654366	-0,959825765	False	0,959825765
ENSMUSG00000027546	Atp9a	545,6241665	517,4176491	460,5509344	1008,768776	280,5211202	-0,95979815	False	0,95979815
ENSMUSG00000059481	Plg	37348,20605	9334,971587	19202,24412	12167,07847	9468,56637	-0,959764022	False	0,959764022
ENSMUSG00000029198	Grpel1	1204,855631	792,5323991	610,252003	267,6554849	2342,025167	0,958896289	False	0,958896289
ENSMUSG00000072294	Klf12	231,0787901	10,09595413	118,8802603	14,61983733	2,609498793	-0,958875666	False	0,958875666
ENSMUSG00000024304	Cdh2	1364,060416	343,2624404	701,8338331	369,9943448	185,2744143	-0,958706143	False	0,958706143
ENSMUSG00000074071	Fam169b	370,1897674	623,4251675	389,2227782	564,5506416	190,4934119	-0,958523914	False	0,958523914
ENSMUSG00000056692	D17Wsu92e	3280,700548	1588,850781	1651,114727	936,7941921	1688,345719	-0,958393563	False	0,958393563
ENSMUSG00000032383	Ppib	1993,151169	5372,309591	6912,666989	3872,007687	6157,112402	0,958030682	False	0,958030682
ENSMUSG00000028766	Alpl	198,619562	1082,79108	457,9091508	385,738785	404,4723129	0,957616491	False	0,957616491
ENSMUSG00000042901	Aida	182,389948	182,9891686	217,5068467	269,9046892	93,94195655	-0,957184675	False	0,957184675
ENSMUSG00000046404	Yod1	138,3381385	421,5060849	268,5813289	768,1037613	600,1847224	0,957160067	False	0,957160067
ENSMUSG00000048490	Nrip1	268,1750507	214,5390252	129,4473946	123,7063159	520,5950092	0,956986457	False	0,956986457
ENSMUSG00000000563	Atp5f1	2945,288525	980,5695448	1517,264359	516,1927181	1479,585816	-0,956936495	False	0,956936495
ENSMUSG00000020454	Eif4enif1	408,8317055	1361,691813	793,4156633	1517,089274	2349,853663	0,956569795	False	0,956569795
ENSMUSG00000028969	Cdk5	331,5478292	362,1923544	766,1172331	856,9473882	170,9221709	-0,955877463	False	0,955877463
ENSMUSG00000042073	Abhd14b	3238,967255	165,3212489	1670,487806	236,166603	202,2361565	-0,955264431	False	0,955264431
ENSMUSG00000037197	Rbm17	489,9797756	524,9896147	252,7306275	314,8888041	1093,379994	-0,955121692	False	0,955121692
ENSMUSG00000029267	Mtf2	126,7455571	503,5357122	245,6858713	1006,51957	504,9380164	0,954879719	False	0,954879719
ENSMUSG00000039917	Rhbdd2	229,5331126	586,8273338	544,2074139	741,1132924	444,9195442	0,954842181	False	0,954842181
ENSMUSG00000031482	Slc25a15	2799,221989	834,1782099	1444,175014	1163,963972	588,4419778	-0,95478032	False	0,95478032
ENSMUSG00000024851	Pitpnm1	74,96535999	345,7864289	145,2980959	809,7140676	323,5778503	0,954719783	False	0,954719783
ENSMUSG00000026034	Clk1	902,675675	2465,936796	1747,980124	4761,568558	2548,175571	0,953409145	False	0,953409145
ENSMUSG00000041798	Gck	1376,425837	37,85982798	2664,67902	25,86586605	118,7321951	0,953034882	False	0,953034882
ENSMUSG00000006395	Hyi	772,8387628	880,8719978	885,878088	1251,682996	399,2533153	-0,952863069	False	0,952863069
ENSMUSG00000022905	Kpna1	555,6710705	752,1485826	287,0738138	295,7705552	1154,703216	-0,952809395	False	0,952809395
ENSMUSG00000028672	Hmgcl	1636,099661	446,7459702	845,37074	712,9982206	341,8443419	-0,952604547	False	0,952604547
ENSMUSG00000036371	Serbp1	2282,965705	2840,749093	2087,009014	1179,708412	3380,605686	-0,952478874	False	0,952478874
ENSMUSG00000075701	Selenos	635,273463	835,4402042	492,2523372	328,3840385	1479,585816	-0,95199375	False	0,95199375
ENSMUSG00000050199	Lgr4	636,8191405	180,4651801	329,3423508	187,8086796	243,9881371	-0,951295656	False	0,951295656
ENSMUSG00000075543	Urad	621,3623653	76,98165023	321,4170001	58,47934932	48,27572767	-0,950988626	False	0,950988626
ENSMUSG00000024969	Mark2	255,8096305	1039,883275	553,0133591	1057,126699	494,5000213	0,950900005	False	0,950900005
ENSMUSG00000028910	Mecr	239,5800165	208,2290539	235,1187371	446,46734	123,9511927	-0,950735424	False	0,950735424
ENSMUSG00000053317	Sec61b	755,83631	1399,551641	1904,725949	391,3617993	1975,390586	-0,949570885	False	0,949570885
ENSMUSG00000035697	Arhgap45	194,7553682	376,0742913	376,8944549	769,2283642	408,3865611	0,949354591	False	0,949354591
ENSMUSG00000016344	Ppdpf	1189,398856	851,8461296	2296,59051	864,8196083	403,1675635	0,949261049	False	0,949261049
ENSMUSG00000012296	Tjap1	111,2887818	535,0855688	214,8650631	346,3776845	362,7203322	0,949122747	False	0,949122747
ENSMUSG00000035836	Ugt2b1	4065,904731	517,4176491	1181,757847	53,98093784	2107,170275	-0,948269512	False	0,948269512
ENSMUSG00000068284	Usf3	489,2069369	146,3913349	253,611222	175,438048	103,0752023	-0,947826277	False	0,947826277
ENSMUSG00000043535	Setx	241,125694	288,9966869	125,044422	488,0776463	361,4155828	-0,947344688	False	0,947344688
ENSMUSG00000056493	Foxk1	309,9083439	261,2328131	89,82064113	147,3229762	597,5752236	0,947278735	False	0,947278735
ENSMUSG00000038594	Cep85l	293,6787299	126,1994266	176,1189042	149,5721819	566,2612381	0,947228997	False	0,947228997
ENSMUSG00000050144	Slc25a44	1770,573606	657,4990127	661,3264852	263,157072	918,5435751	-0,946796751	False	0,946796751
ENSMUSG00000049295	Zfp219	306,8169888	295,3066583	471,9986632	393,6110051	159,1794264	-0,946724486	False	0,946724486
ENSMUSG00000038526	Car14	632,9549467	17,66791973	328,4617563	39,36110051	60,01847224	-0,94637741	False	0,94637741
ENSMUSG00000033902	Mapkbp1	95,05916782	233,4689392	183,1636603	483,5792348	302,70186	0,946235624	False	0,946235624
ENSMUSG00000074093	Svip	225,6689187	22,71589679	117,1190713	20,24285169	22,18073974	-0,946231712	False	0,946231712
ENSMUSG00000056763	Cspp1	265,0836956	706,716789	510,7448221	994,1489385	559,7374911	0,946154735	False	0,946154735
ENSMUSG00000058806	Col13a1	42,50613195	334,4284805	81,89529044	502,6974836	210,0646528	0,946109509	False	0,946109509
ENSMUSG00000019505	Ubb	4225,109516	5902,347183	5955,460745	2192,9756	10655,88832	-0,946098977	False	0,946098977
ENSMUSG00000013858	Tmem259	1293,732089	4312,234407	2490,321305	4114,921907	2893,934161	0,944793004	False	0,944793004
ENSMUSG00000025232	Hexa	635,273463	189,2991399	281,7902467	187,8086796	330,1015973	-0,944467636	False	0,944467636
ENSMUSG00000047141	Zfp654	184,7084643	305,4026124	95,98480277	282,2753208	388,8153202	-0,944372072	False	0,944372072
ENSMUSG00000049232	Tigd2	176,2072379	324,3325264	91,58183017	291,2721437	159,1794264	-0,944139885	False	0,944139885
ENSMUSG00000028478	Clta	941,3176131	275,11475	349,5960248	172,0642394	489,2810237	-0,944018259	False	0,944018259
ENSMUSG00000020462	Cfap36	159,9776239	353,3583945	121,5220439	83,2206125	425,3483033	-0,942857317	False	0,942857317
ENSMUSG00000020694	Tlk2	338,5033781	667,5949668	176,1189042	592,6657133	1366,072618	-0,942620458	False	0,942620458
ENSMUSG00000035504	Reep6	19024,97182	2945,494617	36563,1651	15228,24748	2447,709868	0,942496646	False	0,942496646
ENSMUSG00000035890	Rnf126	401,8761567	772,3404909	1320,011187	1271,925848	812,858874	0,942486024	False	0,942486024
ENSMUSG00000039682	Lap3	3941,47769	829,1302329	2050,904639	352,0006988	751,5356524	-0,942476196	False	0,942476196
ENSMUSG00000040928	S100pbp	205,5751109	227,1589679	197,2531727	384,6141821	106,9894505	-0,942197058	False	0,942197058
ENSMUSG00000005674	Tomm40l	102,7875555	169,1072317	95,98480277	202,4285169	53,49472526	-0,942197058	False	0,942197058
ENSMUSG00000063730	Hsd3b2	438,1995785	5,047977065	228,0739809	7,872220101	7,828496379	-0,942086226	False	0,942086226
ENSMUSG00000022574	Naprt	1402,702354	411,4101308	2694,619234	968,2830725	247,9023853	0,941872516	False	0,941872516
ENSMUSG00000022521	Crebbp	255,8096305	962,9016251	491,3717426	757,9823355	1405,2151	0,941744316	False	0,941744316
ENSMUSG00000031309	Rps6ka3	136,792461	363,4543486	71,32815619	175,438048	251,8166335	-0,939445136	False	0,939445136
ENSMUSG00000002983	Relb	31,68638927	177,9411915	60,76102194	511,6943066	210,0646528	0,939282864	False	0,939282864
ENSMUSG00000035356	Nfkbiz	213,3034985	408,8861422	502,8194714	536,4355698	926,3720715	0,93879154	False	0,93879154
ENSMUSG00000094870	Zfp131	136,0196223	454,3179358	260,6559782	575,7966703	464,4907852	0,938332158	False	0,938332158
ENSMUSG00000046876	Atxn1	284,4046647	570,4214083	174,3577151	148,4475791	296,178113	-0,937991562	False	0,937991562
ENSMUSG00000038025	Phf2	355,5058309	396,2661996	214,8650631	185,5594738	797,2018813	-0,937991562	False	0,937991562
ENSMUSG00000040097	Flywch1	279,7676321	358,4063716	560,0581153	870,4426226	146,1319324	-0,936957592	False	0,936957592
ENSMUSG00000001415	Smg5	422,7428033	1055,027206	809,2663647	1469,855953	965,5145534	0,93683444	False	0,93683444
ENSMUSG00000014426	Map3k4	316,8638927	136,2953807	165,5517699	165,3166221	95,24670595	-0,936580836	False	0,936580836
ENSMUSG00000022637	Cblb	139,8838161	585,5653395	73,08934523	472,3332061	1426,09109	-0,936496044	False	0,936496044
ENSMUSG00000042404	Dennd4b	129,8369122	83,29162157	204,2979288	256,4094547	67,84696862	-0,936344331	False	0,936344331
ENSMUSG00000000440	Pparg	139,8838161	268,8047787	141,7757179	73,09918666	160,4841758	-0,9363018	False	0,9363018
ENSMUSG00000030410	Dmwd	344,6860882	974,2595735	659,5652961	1745,383657	1291,701903	0,93623242	False	0,93623242

ENSMUSG00000058355	Abce1	1133,754465	1351,595859	1107,787907	592,6657133	2549,480321	-0,93581773	False	0,93581773
ENSMUSG00000106864	Gtf3c2	378,6909938	928,8277799	833,9230113	1865,716164	724,1359151	0,935239395	False	0,935239395
ENSMUSG00000029647	Pan3	234,9429839	810,2003189	449,1032056	695,0045747	884,6200908	0,934736335	False	0,934736335
ENSMUSG00000032812	Arap1	790,6140543	877,086015	612,013192	1403,504384	413,6055587	-0,934718026	False	0,934718026
ENSMUSG00000033054	Npat	75,73819875	127,4614209	39,62675344	157,444402	206,1504046	-0,934546336	False	0,934546336
ENSMUSG00000019850	Tnfaip3	28,59503422	70,6716789	14,97010685	169,8150336	327,4920985	-0,933680113	False	0,933680113
ENSMUSG00000030754	Copb1	1185,534662	1091,62504	620,8191372	624,1545937	2503,814092	-0,933292909	False	0,933292909
ENSMUSG00000028059	Arhgef2	59,50858474	297,8306468	113,5966932	390,2371965	186,5791637	0,932751126	False	0,932751126
ENSMUSG00000005677	Nr1i3	5162,562936	739,52864	7497,381751	9853,770361	207,455154	0,932588386	False	0,932588386
ENSMUSG00000031392	Irak1	1112,887818	2979,568462	2540,515193	6235,922923	2124,132018	0,932565261	False	0,932565261
ENSMUSG00000014850	Msh3	166,160334	104,7455241	153,2234466	317,1380098	65,23746983	0,932534777	False	0,932534777
ENSMUSG00000039000	Ube3c	370,9626061	176,6791973	120,6414494	175,438048	194,4076601	-0,932188702	False	0,932188702
ENSMUSG00000032667	Pon2	699,4190803	314,2365723	302,9245152	366,6205361	353,5870865	-0,931869553	False	0,931869553
ENSMUSG00000024944	Arl2	101,2418779	403,8381652	205,1785234	413,8538568	193,1029107	0,931563737	False	0,931563737
ENSMUSG00000036707	Cab39	649,9573995	1116,864926	340,7900796	544,3077899	1594,403763	-0,931461823	False	0,931461823
ENSMUSG00000049115	Agtr1a	1224,1766	157,7492833	641,9534057	365,4959333	143,5224336	-0,931271204	False	0,931271204
ENSMUSG00000042408	Zmym6	159,2047851	258,7088246	217,5068467	259,7832633	83,50396138	-0,930967154	False	0,930967154
ENSMUSG00000028293	Slc35a1	224,89608	99,69754702	117,9996658	84,34521537	108,2941999	-0,930475741	False	0,930475741
ENSMUSG00000020189	Osbpl8	441,2909336	170,3692259	231,596359	178,8118566	185,2744143	-0,930117535	False	0,930117535
ENSMUSG00000002588	Pon1	13589,59681	2757,457472	7132,815619	414,9784596	6586,374954	-0,929959067	False	0,929959067
ENSMUSG00000055839	Elob	1149,21124	3508,34406	4270,883426	2186,227982	2434,662374	0,927799846	False	0,927799846
ENSMUSG00000003235	Eif2b5	384,1008651	201,9190826	171,7159316	164,1920193	156,5699276	-0,927707953	False	0,927707953
ENSMUSG00000021482	Aaed1	1163,895177	600,7092707	595,2818961	362,1221247	611,927467	-0,927528569	False	0,927528569
ENSMUSG00000032637	Atxn2l	1518,628169	2120,150367	1654,637105	798,4680388	4601,851121	-0,927462108	False	0,927462108
ENSMUSG00000027951	Adar	347,7774433	853,1081239	661,3264852	1240,436967	884,6200908	0,927198318	False	0,927198318
ENSMUSG00000032776	Mctp2	210,9849822	413,9341193	110,9549096	201,303914	581,9182308	-0,927166806	False	0,927166806
ENSMUSG00000063704	Mapk15	452,1106762	15,14393119	237,7605206	74,22378953	45,66622888	-0,927166806	False	0,927166806
ENSMUSG00000020260	Pofut2	240,3528552	1201,418541	457,0285563	714,1228235	1448,27183	0,92713037	False	0,92713037
ENSMUSG00000037722	Gnpnat1	867,1250919	275,11475	456,1479618	328,3840385	348,3680889	-0,926738262	False	0,926738262
ENSMUSG00000022300	Dcaf13	182,389948	241,0409048	95,98480277	277,7769093	399,2533153	-0,926148313	False	0,926148313
ENSMUSG00000037646	Vps13b	508,5279059	233,4689392	192,8502001	267,6554834	225,7216456	-0,925949736	False	0,925949736
ENSMUSG00000003444	Med29	145,2936874	152,7013062	263,2977617	76,47299527	174,8364191	-0,925949736	False	0,925949736
ENSMUSG00000057147	Dph6	143,7480099	214,5390252	161,1487973	247,4126318	75,675465	-0,925644443	False	0,925644443
ENSMUSG00000054321	Taf4b	23,18516288	364,7163429	44,02972604	385,738785	327,4920985	0,925276006	False	0,925276006
ENSMUSG00000029178	Klf3	603,5870737	919,99382	317,894622	757,9823355	2228,511969	-0,9250133	False	0,9250133
ENSMUSG00000036932	Aifm1	1300,687638	407,624148	685,1025372	353,1253017	433,1767996	-0,924882705	False	0,924882705
ENSMUSG00000031781	Ciapin1	215,6220148	454,3179358	568,8640605	409,3554453	451,4432912	0,924849603	False	0,924849603
ENSMUSG00000031381	Piga	71,87400494	37,85982798	95,10420825	164,1920193	169,6174215	-0,924802222	False	0,924802222
ENSMUSG00000061175	Fnip2	341,5947332	102,2215356	178,7606877	78,72220101	180,0554167	-0,923844729	False	0,923844729
ENSMUSG00000003849	Nqo1	155,3405913	54,26575344	81,89529044	73,09918666	80,89446258	-0,923582469	False	0,923582469
ENSMUSG00000041560	Nop53	1033,285426	1152,200765	545,0880084	879,4394456	1897,105623	-0,92267774	False	0,92267774
ENSMUSG00000032299	Commd4	257,355308	719,3367317	487,8493646	743,3624981	579,3087321	0,92267421	False	0,92267421
ENSMUSG00000031295	Phka2	385,6465426	203,1810768	364,5661316	730,9918666	63,93272043	0,922576177	False	0,922576177
ENSMUSG00000007050	Lsm2	149,1578862	195,6091113	176,9994987	78,72220101	251,8166335	-0,921997746	False	0,921997746
ENSMUSG00000025408	Ddit3	77,28387628	146,3913349	240,4023042	915,4267375	270,0831251	0,921590799	False	0,921590799
ENSMUSG00000017210	Med24	292,9058911	556,5394714	554,7745481	981,7783069	741,0976572	0,921464392	False	0,921464392
ENSMUSG00000041891	Lman1	3360,302941	1158,510736	1774,39796	846,8259623	1752,27844	-0,92126169	False	0,92126169
ENSMUSG00000036180	Gatad2a	489,2069369	1384,40771	926,3854359	1044,756068	1777,068678	0,921167711	False	0,921167711
ENSMUSG00000032050	Rdx	2654,70115	912,4218544	1401,906477	1109,983034	1261,692666	-0,921159352	False	0,921159352
ENSMUSG00000035401	Emsy	258,1281468	701,668812	488,7299591	1539,581331	571,4802357	0,92095008	False	0,92095008
ENSMUSG00000010047	Hyal2	469,113129	637,3071044	516,0283892	850,1997709	247,9023853	-0,920163725	False	0,920163725
ENSMUSG00000029062	Cdk11b	509,3007447	2447,006882	963,3704058	5159,677975	2796,077957	0,919572779	False	0,919572779
ENSMUSG00000042286	Stab1	562,6266193	1899,301371	982,7434853	4598,501142	297,4828624	-0,919371253	False	0,919371253
ENSMUSG00000020149	Rab1	1758,981024	1962,401084	1338,503672	930,0465748	4678,831336	-0,919365049	False	0,919365049
ENSMUSG00000019461	Plscr3	149,93072	547,7055115	283,5514357	755,7331297	317,0541034	0,919314447	False	0,919314447
ENSMUSG00000026189	Pecr	3185,64138	162,7972603	1684,577318	176,5626508	285,7401178	-0,91919722	False	0,91919722
ENSMUSG00000031918	Mtmr2	312,9996989	311,7125837	165,5517699	236,166603	609,3179682	-0,918878835	False	0,918878835
ENSMUSG00000030301	Ccdc91	302,952795	32,81185092	160,2682028	67,4761723	28,70448672	-0,918604793	False	0,918604793
ENSMUSG00000029022	Miip	239,5800165	808,9383246	685,9831317	1264,053628	452,7480406	0,91820082	False	0,91820082
ENSMUSG00000042167	Papd4	421,9699645	280,1627271	164,6711754	186,6840767	797,2018813	0,917804802	False	0,917804802
ENSMUSG00000014542	Clec4f	845,4856065	349,5724117	522,1925509	1596,936078	177,4459179	0,917454466	False	0,917454466
ENSMUSG00000026544	Dusp23	214,8491761	1188,798599	502,8194714	563,4260387	405,7770623	0,917363071	False	0,917363071
ENSMUSG00000063884	Ptcd3	352,4144758	142,6053521	186,6860384	178,8118566	106,9894505	-0,916659148	False	0,916659148
ENSMUSG00000031853	Map3k21	112,0616206	44,16979931	101,2683699	211,4253399	83,50396138	0,915856036	False	0,915856036
ENSMUSG00000003226	Ranbp2	722,6042432	943,9717111	383,0586166	977,2798954	3046,589841	-0,915640553	False	0,915640553
ENSMUSG00000025134	Alyref	125,1998796	140,0813635	90,70123565	28,11507179	236,1596408	0,915529257	False	0,915529257
ENSMUSG00000024104	Washc2	657,6857871	652,4510356	348,7154303	747,8609096	1129,912977	-0,91534829	False	0,91534829
ENSMUSG00000025016	Tm9sf3	1476,122037	490,9157695	624,3415153	580,2950818	782,8496379	-0,915004859	False	0,915004859
ENSMUSG00000026857	Ntmt1	54,87155216	145,1293406	110,9549096	103,4634642	161,7889252	0,914991111	False	0,914991111
ENSMUSG00000055660	Mettl4	257,355308	87,07760436	136,4921507	48,35792348	49,58047707	-0,91494355	False	0,91494355
ENSMUSG00000025409	Mbd6	635,273463	4350,094235	1634,383431	4039,330663	1197,759946	0,914889133	False	0,914889133
ENSMUSG00000028911	Srsf4	554,8982317	1644,378529	1046,146291	1941,064556	2280,701945	0,914789498	False	0,914789498
ENSMUSG00000029461	Fam168a	448,2464824	177,9411915	237,7605206	212,5499427	167,0079228	-0,914783082	False	0,914783082
ENSMUSG00000020432	Tcn2	2624,560438	1022,215356	1281,265028	1068,372728	1392,167606	-0,91474291	False	0,91474291
ENSMUSG00000021264	Yy1	818,4362498	885,9199748	434,1330988	503,8220865	1599,62276	-0,914732626	False	0,914732626
ENSMUSG00000035392	Dennd1a	472,2044841	782,436445	453,5061782	476,8316176	250,5118841	-0,914532696	False	0,914532696
ENSMUSG00000033940	Brk1	593,5401698	724,3847088	641,9534057	314,8888041	1028,142524	-0,914503209	False	0,914503209
ENSMUSG00000016757	Ttll12	146,0665262	50,47977065	77,49231784	47,23332061	22,18073974	-0,914500394	False	0,914500394
ENSMUSG00000031862	Atp13a1	643,0018507	2280,423639	1211,698061	2674,305629	3398,872178	0,914135448	False	0,914135448
ENSMUSG00000045180	Shroom2	246,5355653	219,5870023	112,7160987	464,460986	133,0844384	0,913761632	False	0,913761632
ENSMUSG00000045302	Preb	1680,15147	3957,614019	3164,856708	8651,569891	5031,113673	0,91354888	False	0,91354888
ENSMUSG00000022552	Sharpin	514,710616	1256,946289	969,5345675	1846,597915	1974,085837	0,913530798	False	0,913530798
ENSMUSG00000004934	Pias4	282,8589872	684,0008922	532,7596851	624,1545937	542,7757489	0,913401905	False	0,913401905
ENSMUSG00000067212	H2-T23	2004,743751	596,9232879	983,6240798	283,3999236	1064,675508	-0,913004049	False	0,913004049
ENSMUSG00000026638	Irf6	935,9077418	241,0409048	416,5212084	497,0744693	268,7783757	-0,912904316	False	0,912904316
ENSMUSG00000018821	Avpi1	342,3675719	302,8786239	644,5951893	163,0674164	118,7321951	0,912847361	False	0,912847361
ENSMUSG00000031483	Erlin2	454,4291925	238,5169163	206,9397124	132,7031389	241,3786384	-0,912757511	False	0,912757511
ENSMUSG00000022817	Itgb5	962,9570985	201,9190826	511,6254166	262,0324691	118,7321951	-0,912383588	False	0,912383588
ENSMUSG00000032725	Folr2	152,2492363	20,19190826	51,95507673	15,7444402	80,89446258	-0,912322136	False	0,912322136
ENSMUSG00000020777	Acox1	18768,38935	43900,99454	9972,732949	66273,97183	40717,31442	-0,912244025	False	0,912244025
ENSMUSG00000059456	Ptk2b	654,5944321	85,8156101	347,8348357	103,4634642	131,779689	-0,912198905	False	0,912198905
ENSMUSG00000023075	Akirin1	262,7651794	257,4468303	150,5816631	129,5555216	494,5000213	0,912196475	False	0,912196475
ENSMUSG00000096054	Syne1	427,3798358	84,55361583	227,1933864	104,5880671	35,22823371	-0,911598003	False	0,911598003
ENSMUSG00000050440	Hamp	29165,38923	86380,98353	54859,27746	57136,5735	124963,6782	0,911478293	False	0,911478293
ENSMUSG00000032002	Dcun1d5	284,4046647	222,1109908	184,0442549	121,4571101	534,9472526	0,911451517	False	0,911451517
ENSMUSG00000029500	Pgam5	143,7480099	175,417203	135,6115562	76,47299527	258,3403805	-0,910519695	False	0,910519695
ENSMUSG00000051285	Pcmtd1	1415,840613	194,347117	723,8486961	753,483924	86,11346017	-0,910010228	False	0,910010228
ENSMUSG00000021411	Pxdc1	1517,082491	287,7346927	807,5051756	155,1951963	424,0435539	-0,909756123	False	0,909756123
ENSMUSG00000067813	Xkr9	176,9800767	13,88193693	94,22361373	15,7444402	10,43799517	-0,90942639	False	0,90942639
ENSMUSG00000040557	Mettl27	484,5699043	127,4614209	258,0141946	253,0356461	116,1226963	-0,909254367	False	0,909254367
ENSMUSG00000019818	Cd164	4865,020012	1664,570437	1754,144286	598,2887277	2591,232301	-0,908807382	False	0,908807382
ENSMUSG00000019841	Rev3l	155,3405913	193,0851227	82,77588496	262,0324691	254,4261323	-0,908152428	False	0,908152428
ENSMUSG00000044791	Setd2	601,2685575	1061,337178	320,5364056	648,8958569	2134,570013	-0,907521293	False	0,907521293
ENSMUSG00000027076	Timm10	124,4270408	151,4393119	171,7159316	66,35156943	155,2651782	-0,907097554	False	0,907097554
ENSMUSG00000024583	Txnl1	1558,042946	1592,636764	818,0723099	566,7998473	2921,333899	0,906892263	False	0,906892263
ENSMUSG00000025937	Lactb2	6259,993979	906,1118831	3339,214423	570,1736559	759,3641488	-0,906652532	False	0,906652532
ENSMUSG00000024887	Asah2	353,1873146	50,47977065	188,4472275	116,9586986	78,28496379	-0,906272957	False	0,906272957
ENSMUSG00000031818	Cox4i1	2837,863937	3138,57974	3827,063788	1514,840068	3451,062154	-0,905639934	False	0,905639934
ENSMUSG00000065979	Cpped1	1159,258144	441,6979931	619,0579482	273,2784978	383,5963226	-0,905055495	False	0,905055495
ENSMUSG00000079184	Mphosph8	207,8936272	638,5690987	389,2227782	1295,542508	441,005296	0,904750608	False	0,904750608
ENSMUSG00000055538	Zcchc24	1504,717071	803,8903475	690,3861044	476,8316176	206,1504046	-0,904421612	False	0,904421612
ENSMUSG00000026020	Nop58	249,6269204	1292,282129	466,7150961	1086,366374	1878,839131	0,902768606	False	0,902768606
ENSMUSG00000053289	Ddx10	180,0714317	159,0112775	83,65647948	82,09600963	336,6253443	0,902574484	False	0,902574484

ENSMUSG00000036613	Eipr1	126,7455571	49,21777638	67,80577811	57,35474645	36,5329831	-0,902455053	False	0,902455053
ENSMUSG00000040181	Fmo1	5781,606785	200,6570883	3093,528552	1413,62581	1112,951235	-0,902217143	False	0,902217143
ENSMUSG00000023883	Phf10	292,9058911	430,3400448	156,7458247	285,6491294	356,1965852	-0,902010196	False	0,902010196
ENSMUSG00000024208	Uqcc2	187,0269806	170,3692259	384,8198056	100,0896556	120,0369445	-0,901953532	False	0,901953532
ENSMUSG00000034616	Ssh3	248,8540816	738,2666457	464,953907	688,2569574	549,2994959	0,901787653	False	0,901787653
ENSMUSG00000003154	Foxj2	152,2492363	150,1773177	91,58183017	78,72220101	284,4353684	0,901665879	False	0,901665879
ENSMUSG00000032076	Cadm1	424,2884808	162,7972603	227,1933864	184,4348709	155,2651782	-0,901124672	False	0,901124672
ENSMUSG00000025878	Uimc1	286,723181	2414,195031	535,4014687	2441,512834	1925,810109	0,900962548	False	0,900962548
ENSMUSG00000041308	Sntb2	77,28387628	118,627461	41,38794248	80,97140676	180,0554167	-0,900956927	False	0,900956927
ENSMUSG00000046718	Bst2	1296,823444	189,2991399	694,789077	39,36110051	525,8140068	-0,900335099	False	0,900335099
ENSMUSG00000028455	Stoml2	545,6241665	162,7972603	292,3573809	173,1888422	163,0936746	-0,900174531	False	0,900174531
ENSMUSG00000002475	Abhd3	779,0214729	170,3692259	360,163159	84,34521537	417,5198069	-0,899818455	False	0,899818455
ENSMUSG00000001366	Fbxo9	523,9846812	107,2695126	280,9096522	253,0356461	116,1226963	-0,899418438	False	0,899418438
ENSMUSG00000002320	Tm9sf1	429,6983521	420,2440906	176,1189042	293,5213495	801,1161295	0,898687146	False	0,898687146
ENSMUSG00000038811	Gngt2	74,19252123	160,2732718	140,0145288	182,1856652	138,303436	0,898491327	False	0,898491327
ENSMUSG00000026839	Upp2	3248,24132	190,5611342	1742,696557	161,9428135	271,3878745	-0,898337433	False	0,898337433
ENSMUSG00000002980	Bcam	59,50858474	161,5352661	227,1933864	458,8379716	110,9036987	0,898137769	False	0,898137769
ENSMUSG00000031845	Bco1	82,69374762	411,4101308	155,8652302	154,0705934	438,3957972	0,897741371	False	0,897741371
ENSMUSG00000014905	Dnajb9	1175,487758	630,9971331	767,8784222	1430,494853	3822,915732	-0,897554159	False	0,897554159
ENSMUSG00000064294	Aox3	3210,372221	41,64581078	1723,323477	80,97140676	103,0752023	-0,897547049	False	0,897547049
ENSMUSG00000039220	Ppp1r10	187,0269806	381,1222684	523,0731454	426,2244883	348,3680889	0,897366068	False	0,897366068
ENSMUSG00000029998	Pcyox1	1858,677225	453,0559415	946,6391099	461,0871774	998,1332883	-0,896971868	False	0,896971868
ENSMUSG00000090935	Synj2bp	812,2535397	402,5761709	389,2227782	436,3459142	197,0171589	-0,896457837	False	0,896457837
ENSMUSG00000005107	Slc2a9	489,9797756	45,43179358	263,2977617	65,22696655	18,26649155	-0,896026945	False	0,896026945
ENSMUSG00000025044	Msr1	169,2516891	97,17355849	173,4771206	314,8888041	50,88522646	0,895672232	False	0,895672232
ENSMUSG00000050730	Arhgap42	792,9325706	213,277031	384,8198056	426,2244883	242,6833878	-0,895584703	False	0,895584703
ENSMUSG00000056025	Clca3a1	98,92336164	262,4948074	250,9694384	1213,446498	183,9696649	0,895084726	False	0,895084726
ENSMUSG00000024066	Xdh	2340,155774	3902,086271	1258,36957	2786,765916	3597,194086	-0,895048878	False	0,895048878
ENSMUSG00000010517	Faf1	397,2391241	209,4910482	188,4472275	213,6745456	177,4459179	-0,894592666	False	0,894592666
ENSMUSG00000030087	Klf15	1612,141659	376,0742913	867,385603	399,2340194	272,6926239	-0,894233117	False	0,894233117
ENSMUSG00000059995	Atxn7l3	253,4911142	670,1189553	481,6852029	699,5029861	471,0145321	0,893836395	False	0,893836395
ENSMUSG00000030870	Ubfd1	384,8737039	378,5982798	297,640948	128,2047274	715,0026693	0,893563524	False	0,893563524
ENSMUSG00000089917	Uckl1	302,952795	586,8273338	348,7154303	673,6371201	163,0936746	-0,893392186	False	0,893392186
ENSMUSG00000035540	Gc	122060,6085	37850,99402	65726,69444	18322,02998	54731,62768	-0,893046353	False	0,893046353
ENSMUSG00000025795	Rassf3	343,9132494	150,1773177	110,0743151	98,9650527	185,2744143	-0,892381033	False	0,892381033
ENSMUSG00000002825	Qtrt1	47,91600329	266,2807902	88,94004661	130,4539331	377,0725756	0,892325584	False	0,892325584
ENSMUSG00000058240	Cryzl1	145,2936874	171,6312202	136,4921507	233,9173973	78,28496379	-0,892164883	False	0,892164883
ENSMUSG00000024750	Zfand5	751,1992774	873,3000322	405,0734796	638,7744311	1510,899801	-0,891012041	False	0,891012041
ENSMUSG00000028618	Tmem59	2204,136152	632,2591273	955,4450551	355,3745074	1188,6267	-0,890917649	False	0,890917649
ENSMUSG00000033813	Tcea1	373,2811224	453,0559415	383,9392111	201,303914	541,4709996	-0,890887327	False	0,890887327
ENSMUSG00000004843	Chmp2b	333,0935068	381,1222684	179,6412823	208,0515312	700,6504259	-0,890808306	False	0,890808306
ENSMUSG00000027261	Hao1	1219,539568	27,76387386	657,8041071	52,85633497	87,41820957	-0,890606646	False	0,890606646
ENSMUSG00000046761	Fam83h	261,2195018	685,2628865	382,1780221	1114,481446	140,9129348	-0,890458561	False	0,890458561
ENSMUSG00000021709	Erbin	755,0634713	527,5136032	361,0437536	444,2181343	1398,691353	0,889407812	False	0,889407812
ENSMUSG00000024045	Akap8	517,8019711	959,1156423	1114,832663	2105,256576	987,6952932	0,889304317	False	0,889304317
ENSMUSG00000038704	Aspdh	2261,32622	291,5206755	4188,107541	571,2982588	514,0712622	0,889129357	False	0,889129357
ENSMUSG00000038909	Kat7	340,8218944	345,7864289	184,0442549	357,6237132	855,9156041	-0,888965302	False	0,888965302
ENSMUSG00000073411	H2-D1	6606,998583	11744,11864	6460,041405	3569,489515	19048,03644	-0,888277271	False	0,888277271
ENSMUSG00000020541	Tom1l1	292,1330523	145,1293406	142,6563124	149,5721819	157,874677	-0,887845814	False	0,887845814
ENSMUSG00000025782	Taf3	90,42213525	368,5023257	167,312959	561,1768329	652,3746983	0,8878013	False	0,8878013
ENSMUSG00000057596	Trim30d	180,8442705	17,66791973	97,74599182	61,85315794	14,35224336	-0,887638442	False	0,887638442
ENSMUSG00000020926	Adam11	184,7084643	30,28786239	341,6706741	71,97458378	71,76121681	0,887356446	False	0,887356446
ENSMUSG00000023965	Fbxl17	197,0738845	88,33959863	106,551937	70,84998091	74,3707156	-0,887179788	False	0,887179788
ENSMUSG00000035900	Gramd4	182,389948	85,8156101	98,62658634	96,71584696	36,5329831	-0,886977716	False	0,886977716
ENSMUSG00000063785	Utp14a	167,7060115	586,8273338	309,9692713	878,3148427	1193,845698	0,886190798	False	0,886190798
ENSMUSG00000037824	Tspan14	398,7848016	215,8010195	206,9397124	202,4285169	194,4076601	-0,885908747	False	0,885908747
ENSMUSG00000026938	Fcna	585,8117822	295,3066583	317,0140275	226,0451772	288,3496166	-0,88589053	False	0,88589053
ENSMUSG00000020463	Ppp4r3b	466,021774	435,3880218	182,2830658	244,0388231	861,1346017	0,885841396	False	0,885841396
ENSMUSG00000031715	Smarca5	401,1033179	388,694234	214,8650631	248,5372346	741,0976572	0,885689764	False	0,885689764
ENSMUSG00000026788	Zbtb43	302,952795	188,0371457	179,6412823	192,307091	559,7374911	0,885657367	False	0,885657367
ENSMUSG00000073910	Mob3b	388,7378977	79,50563877	210,4620905	86,59442111	46,97097827	-0,88523737	False	0,88523737
ENSMUSG00000027104	Atf2	374,0539612	787,4844221	202,5367398	606,1609478	845,4776089	-0,885062775	False	0,885062775
ENSMUSG00000029166	Mapre3	791,3868931	1461,38936	1614,129757	2672,056423	1523,947295	0,884885532	False	0,884885532
ENSMUSG00000028127	Abcd3	5011,859377	2019,190826	1630,861053	2714,791332	2375,948651	-0,884504624	False	0,884504624
ENSMUSG00000025980	Hspd1	4188,786094	1201,418541	1343,787239	486,9530434	2268,959201	-0,884501546	False	0,884501546
ENSMUSG00000039662	Icmt	328,4564742	177,9411915	145,2980959	129,3293302	155,2651782	-0,884301685	False	0,884301685
ENSMUSG00000015094	Npdc1	62,59993979	102,2215356	155,8652302	193,4316939	33,92348431	-0,883876912	False	0,883876912
ENSMUSG00000025781	Atp5c1	3522,599081	1142,104811	1909,128921	432,9721056	1653,117485	-0,883725757	False	0,883725757
ENSMUSG00000001576	Ergic1	891,8559323	291,5206755	483,446392	301,3935696	225,7216456	-0,883454758	False	0,883454758
ENSMUSG00000048332	Lhfp	190,1183356	66,88569611	54,59686029	65,22696655	103,0752023	-0,883200384	False	0,883200384
ENSMUSG00000006471	Ndor1	88,87645772	209,4910482	163,7905809	228,2943829	352,2823371	0,881979172	False	0,881979172
ENSMUSG00000034403	Pja1	717,9672106	389,9562282	303,8051097	218,1729571	324,8825997	-0,880605764	False	0,880605764
ENSMUSG00000025766	D3Ertd751e	149,1578812	5,047977065	81,01469592	15,7444402	37,8377325	-0,88058467	False	0,88058467
ENSMUSG00000055835	Zfp1	94,28632906	334,4284805	173,4771206	509,4451008	523,204508	0,879624893	False	0,879624893
ENSMUSG00000035877	Zhx3	602,0413962	249,8748647	327,5811618	185,5594738	168,3126721	-0,878010296	False	0,878010296
ENSMUSG00000000568	Hnrnpd	539,4414564	767,2925138	415,6406138	293,5213495	1638,765242	-0,878000955	False	0,878000955
ENSMUSG00000032081	Apoc3	28761,9674	19613,91488	52841,83541	13612,19316	27371,03284	0,877518215	False	0,877518215
ENSMUSG00000019302	Atp6v0a1	929,7250317	6837,484934	1706,592181	3189,373744	2969,609626	0,87624234	False	0,87624234
ENSMUSG00000032312	Csk	680,0981113	783,6984393	1100,743151	872,6918284	370,5488286	-0,876079221	False	0,876079221
ENSMUSG00000031622	Sin3b	369,4169286	478,2958269	490,4911481	201,303914	416,2150575	-0,875874756	False	0,875874756
ENSMUSG00000034173	Zbed5	17,77529154	239,7789106	9,686539729	566,7998473	232,2453926	-0,875819926	False	0,875819926
ENSMUSG00000014763	Fam120b	342,3675719	371,0263142	361,0437536	621,905388	186,5791637	-0,875758179	False	0,875758179
ENSMUSG00000038344	Txlng	410,377383	177,9411915	752,9083153	389,1125936	101,7704529	0,87552297	False	0,87552297
ENSMUSG00000042216	Sgsm1	112,8344594	206,9670596	327,5811618	402,607828	240,073889	0,87519344	False	0,87519344
ENSMUSG00000042997	Nhlrc3	143,7480099	160,2732718	78,37291236	191,1824882	216,5883998	-0,875114969	False	0,875114969
ENSMUSG00000031877	Ces2g	833,1201863	200,6570883	454,3867728	159,6936078	279,2163709	-0,874603796	False	0,874603796
ENSMUSG00000075600	Zc3h3	71,10116618	195,6091113	130,3279891	139,4507561	356,1965852	0,874201821	False	0,874201821
ENSMUSG00000019832	Rab32	529,3945525	127,4614209	288,8350028	101,2142584	212,6741516	-0,874097762	False	0,874097762
ENSMUSG00000039438	Ttc36	6110,836097	1704,954254	11200,28171	1819,607446	1540,909037	0,874093328	False	0,874093328
ENSMUSG00000078622	Ccdc47	1079,655752	583,041351	589,1177345	356,4991103	225,7216456	-0,873943493	False	0,873943493
ENSMUSG00000071256	Zfp213	76,51103752	137,557375	128,5668	160,8182106	41,75198069	-0,873823247	False	0,873823247
ENSMUSG00000040549	Ckap5	201,7109171	79,50563877	110,0743151	61,85315794	95,24670595	-0,873811301	False	0,873811301
ENSMUSG00000035245	Eogt	74,96535999	201,9190826	137,3727453	222,6713686	498,4142695	0,873799788	False	0,873799788
ENSMUSG00000021065	Fut8	222,5775637	65,62370184	121,5220439	84,34521537	22,18073974	-0,873090133	False	0,873090133
ENSMUSG00000048249	Crebrf	399,5576404	123,6754381	204,2979288	218,1729571	108,2941999	-0,872931354	False	0,872931354
ENSMUSG00000034636	Zyg11b	520,8933261	223,3729851	130,3279891	182,1856652	284,4353684	-0,872887083	False	0,872887083
ENSMUSG00000046785	Epm2aip1	470,6588065	204,4430711	257,1336001	98,9650527	247,9023853	-0,872163448	False	0,872163448
ENSMUSG00000064030	Pym1	172,3430441	552,7534886	315,2528385	325,0102299	553,2137441	0,871226289	False	0,871226289
ENSMUSG00000043789	Vwce	625,2265591	1047,455241	1141,250499	2188,477188	341,8443419	-0,871039518	False	0,871039518
ENSMUSG00000028191	Bcl10	111,2887818	109,7935012	60,76102194	43,85951199	203,5409059	0,871010591	False	0,871010591
ENSMUSG00000030882	Dnhd1	18,54813031	159,0112775	127,6862055	466,7101917	33,92348431	0,871010591	False	0,871010591
ENSMUSG00000056952	Tatdn2	231,8516288	326,8565149	126,805611	188,9332824	446,2242936	-0,870583278	False	0,870583278
ENSMUSG00000005580	Adcy9	159,2047851	87,07760436	56,35804933	74,22378953	33,92348431	-0,870510076	False	0,870510076
ENSMUSG00000030245	Golt1b	40,96045443	98,43555276	74,85053427	76,47299527	361,4155828	0,869780893	False	0,869780893
ENSMUSG00000027575	Arfgap1	350,8687983	1043,669258	641,0728112	2723,788155	1312,577893	0,869556564	False	0,869556564
ENSMUSG00000041773	Enc1	78,82955381	358,4063716	43,14913152	231,6681916	943,3338137	-0,869405087	False	0,869405087
ENSMUSG00000025158	Rfng	284,4046647	113,579484	519,5507673	255,2848519	236,1596408	0,8693196	False	0,8693196
ENSMUSG00000033720	Sfxn5	358,5971859	63,09971331	196,3725782	52,85633497	36,5329831	-0,868770684	False	0,868770684
ENSMUSG00000026202	Tuba4a	4223,563839	1486,629246	2313,321806	1386,635341	913,3245776	-0,868494884	False	0,868494884
ENSMUSG00000029455	Aldh2	30714,15811	11606,56127	11464,46007	3991,215591	16823,43872	-0,868431216	False	0,868431216
ENSMUSG00000070473	Cldn3	1658,511985	306,6646067	3027,483963	358,748316	138,303436	0,86822988	False	0,86822988
ENSMUSG00000059923	Grb2	495,389647	367,2403314	420,924181	218,1729571	904,1913318	0,868064369	False	0,868064369
ENSMUSG00000029201	Ugdh	1733,477345	281,4247213	950,161488	206,9269284	605,40372	-0,867424344	False	0,867424344

ENSMUSG00000086040	Wipf3	119,0171695	243,5648934	79,25350688	156,3197992	65,23746983	-0,867396978	False	0,867396978
ENSMUSG00000078650	G6pc	4205,015708	2305,663524	1338,503672	1662,163044	1556,56603	-0,8669292	False	0,8669292
ENSMUSG00000033386	Frrs1	295,9972462	747,1006056	539,8044413	850,1997709	888,534339	0,866853093	False	0,866853093
ENSMUSG00000028393	Alad	1669,331728	442,9599874	3044,215259	842,3275508	281,8258696	0,866799703	False	0,866799703
ENSMUSG00000031605	Klhl2	207,1207884	324,3325264	113,5966932	269,9046892	319,6636021	-0,866551524	False	0,866551524
ENSMUSG00000079036	Alkbh1	96,60484535	232,206945	176,1189042	367,745139	592,356226	0,866382316	False	0,866382316
ENSMUSG00000031388	Naa10	123,654202	133,7713922	393,6257508	151,8213877	67,84696862	-0,865955004	False	0,865955004
ENSMUSG00000066441	Rdh11	2197,953441	371,0263142	1206,414494	194,5562968	189,1886625	-0,86543516	False	0,86543516
ENSMUSG00000025869	Nop16	273,584922	296,5686525	183,1636603	114,7094929	498,4142695	0,865356651	False	0,865356651
ENSMUSG00000027099	Mtx2	449,79216	464,4138899	300,2827316	134,9523446	819,382621	0,865278779	False	0,865278779
ENSMUSG00000058207	Serpina3k	95401,5354	195941,0157	211538,177	173763,5143	175710,6012	0,865040796	False	0,865040796
ENSMUSG00000019432	Ddx39b	744,2437286	2960,638548	1355,234968	3767,41962	3578,927595	0,86469594	False	0,86469594
ENSMUSG00000035047	Kri1	142,2023324	535,0855688	258,8947891	564,5506416	427,9578021	0,8644208	False	0,8644208
ENSMUSG00000022403	St13	2375,706357	754,6725712	1305,04108	499,323675	1094,684744	-0,864261306	False	0,864261306
ENSMUSG00000042423	Fbrs	477,6143554	1774,363938	869,1467921	1916,323293	1898,410372	0,863753658	False	0,863753658
ENSMUSG00000032047	Acat1	6120,883001	1539,633005	3363,87107	2688,925466	1243,426175	-0,863617381	False	0,863617381
ENSMUSG00000002731	Prkra	219,4862086	51,74176491	120,6414494	62,97776081	28,70448672	-0,863404625	False	0,863404625
ENSMUSG00000038467	Chmp4b	642,2290119	1557,300924	452,6255837	353,1253017	2816,953947	-0,862907644	False	0,862907644
ENSMUSG00000027253	Lrp4	168,4788503	157,7492833	389,2227782	362,1221247	92,63720715	-0,862903832	False	0,862903832
ENSMUSG00000028229	Rmdn1	155,3405913	58,05173624	85,41766852	71,97458378	39,1424819	-0,862828438	False	0,862828438
ENSMUSG00000048701	Ccdc6	329,229313	561,5874484	202,5367398	181,0610623	867,6583487	-0,862616468	False	0,862616468
ENSMUSG00000019710	Mrpl24	863,2608981	1076,481109	1229,309951	1558,69958	474,9287803	-0,862085457	False	0,862085457
ENSMUSG00000009555	Cdk9	389,5107365	1773,101944	707,9979948	2574,215973	1610,060755	0,862082179	False	0,862082179
ENSMUSG00000019433	Gipc1	194,7553682	357,1443773	353,9989974	376,741962	383,5963226	0,862082179	False	0,862082179
ENSMUSG00000025935	Tram1	2738,940575	3267,303155	4179,301596	1506,967848	5601,289159	-0,861969329	False	0,861969329
ENSMUSG00000030327	Necap1	464,4760964	278,9007328	243,9246823	170,9396365	844,1728595	0,861934094	False	0,861934094
ENSMUSG00000017418	Arl5b	386,4193814	131,2474037	146,1786905	94,46664122	212,6741516	-0,861522757	False	0,861522757
ENSMUSG00000018770	Atp5g3	2333,200225	2905,110801	2405,784231	1284,296479	3062,246834	-0,861331832	False	0,861331832
ENSMUSG00000017978	Cadps2	159,9776239	40,38381652	88,05945209	75,3483924	67,84696862	-0,861320356	False	0,861320356
ENSMUSG00000058655	Eif4b	2901,236716	1048,717235	1264,533732	978,4044983	1597,013261	-0,861291719	False	0,861291719
ENSMUSG00000035104	Eva1a	815,3448948	223,3729851	1481,159984	526,3141439	87,41820957	0,861245118	False	0,861245118
ENSMUSG00000038844	Kif16b	204,0294334	112,3174897	66,92518359	88,84362686	70,45646741	-0,861194694	False	0,861194694
ENSMUSG00000022848	Dirc2	285,1775035	306,6646067	117,1190713	147,3229762	517,9855104	0,861051564	False	0,861051564
ENSMUSG00000091144	Phf11c	91,96781277	343,2624404	279,1484631	584,7934932	167,0079228	0,86071561	False	0,86071561
ENSMUSG00000033793	Atp6v1h	490,7526144	461,8899014	284,4320302	292,3967466	891,1438378	0,86066236	False	0,86066236
ENSMUSG00000027433	Xrn2	396,4662853	451,7939473	218,3874412	467,8347946	541,4709996	-0,860308294	False	0,860308294
ENSMUSG00000034659	Tmem109	327,6836354	59,31373051	180,5218768	50,60712922	48,27572767	-0,860129944	False	0,860129944
ENSMUSG00000015083	C8g	3742,858128	1248,112329	2062,352368	494,8252635	1557,870779	-0,859849516	False	0,859849516
ENSMUSG00000026827	Gpd2	353,1873146	1414,695572	194,6113891	672,5125172	1208,197941	-0,859837384	False	0,859837384
ENSMUSG00000038733	Wdr26	1193,26305	1933,375216	657,8041071	1352,897255	2138,484261	-0,859182193	False	0,859182193
ENSMUSG00000027881	Prpf38b	747,3350836	1855,131571	1355,234968	2796,887342	1854,048892	0,858715848	False	0,858715848
ENSMUSG00000002658	Gtf2f1	287,4960198	984,3555276	521,3119563	620,7807851	1366,072618	0,858604966	False	0,858604966
ENSMUSG00000040268	Plekha1	85,01226391	288,9966869	154,1040411	444,2181343	164,398424	0,85816181	False	0,85816181
ENSMUSG00000040472	Rabggta	442,0637753	518,6796434	870,9079811	1099,861608	243,9881371	-0,857443504	False	0,857443504
ENSMUSG00000026113	Inpp4a	163,068979	353,3583945	221,0292247	431,8475027	90,02770836	-0,857041359	False	0,857041359
ENSMUSG00000071078	Nr2c2ap	129,8369122	345,7864289	235,1187371	307,016584	245,2928865	0,85668892	False	0,85668892
ENSMUSG00000041577	Prelp	1133,754465	61,83771904	626,1027043	268,7800863	127,8654409	-0,856636995	False	0,856636995
ENSMUSG00000010358	Ifi35	372,5082837	214,5390252	674,535403	233,9173973	275,3021227	0,856621657	False	0,856621657
ENSMUSG00000022210	Dhrs4	1388,791257	595,6612936	766,9978277	332,88245	457,9670382	-0,856535374	False	0,856535374
ENSMUSG00000027079	Clp1	101,2418779	397,5281938	185,8054439	183,3102681	722,8311657	0,856481427	False	0,856481427
ENSMUSG00000005125	Ndrg1	445,9279661	230,9449507	112,7160987	246,2880289	112,2084481	-0,856464175	False	0,856464175
ENSMUSG00000032808	Cyp2c38	2463,037137	95,91156423	1360,518535	52,85633497	378,377325	-0,85628177	False	0,85628177
ENSMUSG00000025241	Fyco1	688,5993377	155,2252947	380,416833	326,1348328	62,62797103	-0,856083708	False	0,856083708
ENSMUSG00000033701	Acbd6	411,1502218	541,3955402	411,2376412	227,1697801	819,382621	-0,855894678	False	0,855894678
ENSMUSG00000013736	Tmt1	154,5677526	306,6646067	85,41766852	166,441225	414,9103081	-0,855632936	False	0,855632936
ENSMUSG00000057375	Yipf1	315,3182152	93,38757569	174,3577151	118,0833015	60,01847224	-0,854758311	False	0,854758311
ENSMUSG00000079427	Mthfsl	193,2096907	312,974578	206,0591179	106,8372728	350,9775877	-0,854752402	False	0,854752402
ENSMUSG00000062646	Ganc	426,6069971	132,5093979	527,476118	771,4775699	39,1424819	0,854716584	False	0,854716584
ENSMUSG00000037772	Mrpl23	213,3034985	184,2511629	385,7004001	85,46981824	140,9129348	0,854573014	False	0,854573014
ENSMUSG00000035278	Plekhj1	228,7602738	310,4505895	386,5809947	228,2943829	126,5606915	-0,85400715	False	0,85400715
ENSMUSG00000030849	Fgfr2	581,1747496	218,325008	367,2079152	1050,379082	213,978901	0,853866162	False	0,853866162
ENSMUSG00000034761	Map4k5	189,3454969	160,2732718	104,790748	260,9078662	344,4538407	-0,853509764	False	0,853509764
ENSMUSG00000004771	Rab11a	387,9650589	503,5357122	379,5362385	214,7991485	558,4327417	-0,852938452	False	0,852938452
ENSMUSG00000037349	Nudt22	96,60484535	143,8673463	221,0292247	152,9459905	53,49472526	-0,852698907	False	0,852698907
ENSMUSG00000028291	Akirin2	207,1207884	203,1810768	188,4472275	114,7094929	350,9775877	-0,852487574	False	0,852487574
ENSMUSG00000036712	Cyld	255,8096305	79,50563877	122,4026384	141,6999618	114,8179469	-0,852231209	False	0,852231209
ENSMUSG00000006335	Tfpt	102,7875555	352,0964003	216,6262521	185,5594738	707,1741729	0,852216053	False	0,852216053
ENSMUSG00000062729	Ppox	504,6637121	300,3546353	649,8787564	910,928326	90,02770836	0,852015191	False	0,852015191
ENSMUSG00000021591	Glrx	252,7182754	517,4176491	140,0145288	156,3197992	991,6095413	-0,851953459	False	0,851953459
ENSMUSG00000075419	Dolk	141,4294936	47,95578211	78,37291236	69,72537804	23,48548914	-0,851655996	False	0,851655996
ENSMUSG00000030652	Coq7	139,1109773	434,1260276	250,9694384	260,9078662	474,9287803	0,851275424	False	0,851275424
ENSMUSG00000028394	Pole3	259,6738243	398,7901881	220,1486302	143,9491676	536,252002	-0,851141148	False	0,851141148
ENSMUSG00000034880	Mrpl34	400,3304791	138,8193693	722,0875071	150,6967848	91,33245776	0,850982223	False	0,850982223
ENSMUSG00000022892	App	941,3176131	424,0300734	490,4911481	446,46734	521,8997586	-0,850908855	False	0,850908855
ENSMUSG00000034820	Cpsf7	415,0144156	822,8202615	748,5053427	1542,95514	1090,770495	0,850851164	False	0,850851164
ENSMUSG00000064138	Fam172a	163,8418177	90,86358716	81,89529044	51,73173209	82,19921198	-0,850529459	False	0,850529459
ENSMUSG00000097336	Fendrr	91,19497401	218,325008	248,3276549	377,8665649	164,398424	0,850170247	False	0,850170247
ENSMUSG00000020564	Atxn7l1	247,3084041	112,3174897	108,3131261	137,2015503	100,4657035	-0,850014483	False	0,850014483
ENSMUSG00000021477	Ctsl	5397,505919	12154,26678	4501,599191	2994,817447	17928,56146	-0,849824856	False	0,849824856
ENSMUSG00000028706	Nsun4	197,8467233	175,417203	196,3725782	356,4991103	44,36147948	0,849515305	False	0,849515305
ENSMUSG00000030035	Wbp1	585,0389434	273,8527558	1054,071641	782,7235986	159,1794264	0,849368358	False	0,849368358
ENSMUSG00000027997	Casp6	230,3059513	80,76763303	97,74599182	51,73173209	127,8654409	-0,848925303	False	0,848925303
ENSMUSG00000054723	Vmac	277,4491158	49,21777638	140,0145288	154,0705934	48,27572767	-0,848631676	False	0,848631676
ENSMUSG00000037731	Themis2	91,96781277	137,557375	51,07448221	115,8340958	206,1504046	-0,848526357	False	0,848526357
ENSMUSG00000021548	Ccnh	307,5898276	104,7455241	170,835337	61,85315794	103,0752023	-0,848401367	False	0,848401367
ENSMUSG00000028307	Aldob	65011,19673	95614,99557	78054,13714	36107,6244	85147,94562	-0,848384706	False	0,848384706
ENSMUSG00000031133	Arhgef6	61,05426226	94,64956996	100,3877754	283,3999236	33,92348431	-0,847807657	False	0,847807657
ENSMUSG00000052446	Zfp961	125,1998796	65,62370184	69,56696715	62,97776081	53,49472526	-0,847758843	False	0,847758843
ENSMUSG00000005899	Smpd4	214,8491761	418,9820964	386,5809947	625,2791966	454,05279	0,847446468	False	0,847446468
ENSMUSG00000031158	Timm17b	592,7673311	554,0154828	931,6690031	329,5086414	559,7374911	-0,847149639	False	0,847149639
ENSMUSG00000027577	Chrna4	543,3056503	23,97789106	302,0439207	8,996822973	35,22823371	-0,847005702	False	0,847005702
ENSMUSG00000028889	Yrdc	113,6072981	522,4656262	204,2979288	218,1729571	1548,737534	0,846619062	False	0,846619062
ENSMUSG00000022427	Tomm22	485,342743	697,8828292	636,6698386	269,9046892	934,2005679	-0,846553879	False	0,846553879
ENSMUSG00000028247	Coq3	197,0738845	263,7568016	292,3573809	652,2696655	109,5989493	-0,846502641	False	0,846502641
ENSMUSG00000025173	Wdr45b	463,7032577	502,2737179	258,0141946	436,3459142	1135,131975	-0,845751425	False	0,845751425
ENSMUSG00000032350	Gclc	5117,738287	1052,503218	2101,979121	944,6664122	2848,267933	-0,845421507	False	0,845421507
ENSMUSG00000038175	Mylip	70,32832742	76,98165023	47,55210413	281,1507179	39,1424819	-0,845370671	False	0,845370671
ENSMUSG00000025034	Trim8	270,493567	798,8423705	567,983466	485,8284405	696,7361777	0,844852665	False	0,844852665
ENSMUSG00000048546	Tob2	329,229313	542,6575344	212,2232795	183,3102681	571,4802357	-0,844805192	False	0,844805192
ENSMUSG00000028268	Gbp3	140,6566548	150,1773177	78,37291236	114,7094929	394,0343177	-0,843750798	False	0,843750798
ENSMUSG00000032066	Bco2	567,2636519	66,88569611	316,133433	202,4285169	57,40897345	-0,843485808	False	0,843485808
ENSMUSG00000026617	Bpnt1	609,7697839	150,1773177	339,9094851	89,96822973	221,8073974	-0,843114041	False	0,843114041
ENSMUSG00000031583	Wrn	186,2541418	59,31373051	103,9101535	79,84680388	48,27572767	-0,841935876	False	0,841935876
ENSMUSG00000040463	Mybbp1a	843,1670902	3701,429183	1511,100198	1847,722518	7640,612466	0,841708863	False	0,841708863
ENSMUSG00000037857	Nufip2	225,6689187	398,7901881	125,9250165	160,8182106	357,5013346	-0,841642811	False	0,841642811
ENSMUSG00000020661	Dnmt3a	189,3454969	214,5390252	265,9395453	223,7959715	105,6847011	-0,841254563	False	0,841254563
ENSMUSG00000025894	Aasdhppt	419,6514482	126,1994266	234,2381425	140,575359	80,89446258	-0,841215541	False	0,841215541
ENSMUSG00000028655	Mfsd2a	245,7627266	3278,661103	440,2972604	5140,559726	4565,318138	0,841211741	False	0,841211741
ENSMUSG00000029406	Pitpnm2	789,8412156	639,8310929	1690,74148	1253,932202	441,005296	-0,840766671	False	0,840766671
ENSMUSG00000004364	Cul3	892,628771	1187,536604	498,4164988	822,0846992	2236,340466	-0,840708482	False	0,840708482
ENSMUSG00000038398	Upf3a	132,9282672	237,254922	117,1190713	74,22378953	262,2546287	-0,840694361	False	0,840694361
ENSMUSG00000003721	Insig2	2136,899179	760,9825425	269,4619234	1193,203647	1086,856247	-0,84067855	False	0,84067855
ENSMUSG00000030590	Fam98c	162,2961402	411,4101308	290,5961919	681,5093402	322,2731009	0,840387108	False	0,840387108

ENSMUSG00000035530	Eif1	2232,731186	2862,202996	2504,410817	1247,184585	4403,529213	-0,840134564	False	0,840134564
ENSMUSG00000089911	Mfsd14a	402,6489954	638,5690987	304,6857042	224,9205743	1049,018515	-0,840107098	False	0,840107098
ENSMUSG00000021241	Isca2	432,7897072	586,8273338	559,1775207	241,7896174	503,6332671	-0,839913893	False	0,839913893
ENSMUSG00000024516	Sec11c	257,355308	460,6279071	487,8493646	484,7038377	648,4604501	0,839840281	False	0,839840281
ENSMUSG00000053119	Chmp3	612,0883001	342,0004461	315,2528385	295,7705552	297,4828624	-0,839741585	False	0,839741585
ENSMUSG00000024331	Dsc2	696,3277253	376,0742913	236,8799261	389,1125936	134,3891878	-0,839578794	False	0,839578794
ENSMUSG00000046330	Rpl37a	1231,904988	3090,623958	3952,10821	2204,221628	2565,137314	0,839378299	False	0,839378299
ENSMUSG00000021770	Samd8	455,2020313	213,277031	142,6563124	199,0547083	254,4261323	-0,839260137	False	0,839260137
ENSMUSG00000062908	Acadm	3174,048799	829,1302329	1774,39796	1755,505083	761,9736476	-0,838994697	False	0,838994697
ENSMUSG00000051652	Lrrc3	1578,136754	31,54985665	882,3557099	85,46981824	27,39973733	-0,838789946	False	0,838789946
ENSMUSG00000031451	Gas6	1118,29769	5453,077224	1999,830157	2293,065255	2398,129391	0,838573196	False	0,838573196
ENSMUSG00000031024	St5	502,3451958	1414,695572	280,9096522	611,7839622	2587,318053	-0,838572883	False	0,838572883
ENSMUSG00000079426	Arpc4	244,9898878	89,6015929	80,1341014	25,86586605	136,9986866	-0,838560139	False	0,838560139
ENSMUSG00000057863	Rpl36	1232,677827	3105,767889	3341,856207	2204,221628	2415,091133	0,838473505	False	0,838473505
ENSMUSG00000011257	Pabpc4	410,377383	796,3183819	733,5352359	1721,766996	1555,261281	0,837915046	False	0,837915046
ENSMUSG00000004896	Rmad1	171,5702053	309,1885952	323,1781892	456,5887659	306,6161082	0,837634453	False	0,837634453
ENSMUSG00000053931	Cnn3	1694,835407	663,808984	835,6842003	283,3999236	948,5528113	-0,83734517	False	0,83734517
ENSMUSG00000019873	Reep3	1239,633376	1472,747309	693,9084824	779,34979	2456,843114	-0,837096196	False	0,837096196
ENSMUSG00000024620	Pdgfrb	146,8393649	141,3433578	122,4026384	287,8983351	82,19921198	-0,837042312	False	0,837042312
ENSMUSG00000027582	Zgpat	177,7529154	23,97789106	99,50718086	16,86904307	58,71372284	-0,837000677	False	0,837000677
ENSMUSG00000025959	Klf7	103,5603942	253,6608475	184,9248494	336,2562586	267,4736263	0,836466739	False	0,836466739
ENSMUSG00000018143	Mafk	136,792461	126,1994266	76,61172331	100,0896556	358,806084	-0,836351643	False	0,836351643
ENSMUSG00000002550	Uck1	493,8439694	215,8010195	186,6860384	173,1888422	276,6068721	-0,836218293	False	0,836218293
ENSMUSG00000032280	Tle3	139,1109773	633,5211216	248,3276549	501,5728807	574,0897345	0,836008667	False	0,836008667
ENSMUSG00000022383	Ppara	518,5748098	883,3959863	290,5961919	552,18001	1205,588442	-0,835536331	False	0,835536331
ENSMUSG00000038876	Rnf146	218,7133699	282,6867156	137,3727453	122,581713	789,3733849	-0,835297644	False	0,835297644
ENSMUSG00000037257	Aagab	404,9675117	217,0630138	218,3874412	78,72220101	227,026395	-0,834946133	False	0,834946133
ENSMUSG00000037541	Shank2	483,7970655	6,309971331	271,2231124	52,85633497	2,609498793	-0,834921895	False	0,834921895
ENSMUSG00000048371	Pdp2	545,6241665	613,3292133	436,7748823	305,8919811	1019,009279	-0,834885258	False	0,834885258
ENSMUSG00000030505	Prmt3	71,10116618	181,7271743	126,805611	155,1951963	174,8364191	0,834673457	False	0,834673457
ENSMUSG00000015120	Ube2i	736,515341	1335,189934	412,9988303	605,0363449	1557,870779	-0,83457788	False	0,83457788
ENSMUSG00000024858	Grk2	380,2366713	1036,097292	678,0577811	937,9187949	1145,56997	0,834510542	False	0,834510542
ENSMUSG00000029439	Sfswap	392,6020915	1468,961326	700,0726441	3358,064175	3499,337881	0,834436775	False	0,834436775
ENSMUSG00000029103	Lrpap1	602,814235	179,2031858	338,148296	246,2880289	206,1504046	-0,834057402	False	0,834057402
ENSMUSG00000033257	Ttll4	221,8047249	922,5178085	395,3869399	1109,983034	1146,87472	0,833975119	False	0,833975119
ENSMUSG00000022568	Scrib	289,0416973	1282,186174	515,1477947	1675,658279	1505,680804	0,833708766	False	0,833708766
ENSMUSG00000020359	Phykpl	267,4022119	224,6349794	379,5362385	391,3617993	150,0461806	-0,833604804	False	0,833604804
ENSMUSG00000053768	Chchd3	1444,435648	474,5098441	811,0275537	264,2816748	631,4987079	-0,832683096	False	0,832683096
ENSMUSG00000030737	Slco2b1	3265,243773	759,7205482	1833,397792	1449,613102	433,1767996	-0,832670861	False	0,832670861
ENSMUSG00000026692	Fmo4	383,3280264	526,251609	531,8790906	1865,716164	215,2836504	-0,832340723	False	0,832340723
ENSMUSG00000032212	Sltm	401,1033179	2164,320166	714,1621564	2517,98583	1253,86417	0,832277787	False	0,832277787
ENSMUSG00000038633	Degs1	1037,14962	482,0818097	552,1327646	582,5442875	502,3285177	-0,832184394	False	0,832184394
ENSMUSG00000033400	Agl	1053,379234	85,8156101	591,759518	151,8213877	66,54221922	-0,831942013	False	0,831942013
ENSMUSG00000054474	Thnsl2	667,7326911	153,9633005	375,1332659	203,5531198	118,7321951	-0,831867471	False	0,831867471
ENSMUSG00000036291	Ap5m1	240,3528552	135,0333865	126,805611	66,35156943	92,63720715	-0,83183779	False	0,83183779
ENSMUSG00000057982	Zfp809	473,7501616	759,7205482	455,2673673	877,1902399	266,1688769	-0,831784549	False	0,831784549
ENSMUSG00000042507	Elmsan1	214,0763373	162,7972603	152,3428521	151,8213877	380,9868238	-0,831615766	False	0,831615766
ENSMUSG00000028382	Ptbp3	1082,747107	513,6316663	429,7301262	608,4101535	562,3469899	-0,831580182	False	0,831580182
ENSMUSG00000034853	Acot11	114,3801369	16,40592546	125,044422	203,5531198	24,79023853	0,831568796	False	0,831568796
ENSMUSG00000027367	Stard7	1165,440854	426,554062	639,3116221	515,0681152	654,984197	-0,831343786	False	0,831343786
ENSMUSG00000038346	Zfp384	659,2314647	1351,595859	1172,951902	1517,089274	1333,453883	0,831286847	False	0,831286847
ENSMUSG00000029993	Nfu1	459,0662251	193,0851227	258,0141946	150,6967848	176,1411685	-0,831251855	False	0,831251855
ENSMUSG00000032435	Dync1li1	172,3430441	212,0150367	96,86539729	150,6967848	327,4920985	-0,831229775	False	0,831229775
ENSMUSG00000037326	Capn15	408,8317055	832,9162157	727,3710742	1287,670288	1221,245435	0,831184471	False	0,831184471
ENSMUSG00000032040	Dcps	282,0861484	535,0855688	216,6262521	158,5690049	388,8153202	-0,831025027	False	0,831025027
ENSMUSG00000019883	Echdc1	1569,635573	205,7050654	882,3557099	233,9173973	116,1226963	-0,83099732	False	0,83099732
ENSMUSG00000006599	Gtf2h1	350,8687983	485,8677925	197,2531727	481,3300291	716,3074187	-0,830883154	False	0,830883154
ENSMUSG00000062825	Actg1	697,8734028	1091,62504	579,4311947	392,4864022	970,733551	-0,830322677	False	0,830322677
ENSMUSG00000031480	Thsd1	70,32832742	349,5724117	125,044422	283,3999236	746,3166548	0,830262891	False	0,830262891
ENSMUSG00000053411	Cbx7	191,6640132	89,6015929	131,2085836	340,7546701	44,36147948	0,830153947	False	0,830153947
ENSMUSG00000072501	Phf20l1	387,9650589	928,8277799	689,5055098	1965,80582	962,9050546	0,829635355	False	0,829635355
ENSMUSG00000056211	R3hdm1	401,8761567	750,8865884	714,1621564	1072,87114	735,8786596	0,829500703	False	0,829500703
ENSMUSG00000071253	Slc25a16	730,3326308	191,8231285	205,1785234	104,5880671	410,9960599	-0,829429128	False	0,829429128
ENSMUSG00000024847	Aip	282,8589872	225,8969736	506,3418495	251,9110432	159,1794264	-0,829429128	False	0,829429128
ENSMUSG00000026192	Atic	319,182409	111,0554954	179,6412823	78,72220101	139,6081854	-0,829262218	False	0,829262218
ENSMUSG00000001627	Ifrd1	150,7035587	810,2003189	267,7007343	427,3490912	1446,967081	0,828907611	False	0,828907611
ENSMUSG00000037876	Jmjd1c	525,5303587	5580,538645	295,879759	3412,045112	2947,428887	-0,828763102	False	0,828763102
ENSMUSG00000029029	Wrap73	131,3825897	47,95578211	73,96993975	51,73173209	28,70448672	-0,828763102	False	0,828763102
ENSMUSG00000037703	Lzts3	461,3847414	35,33583945	259,7753837	89,96822973	24,79023853	-0,828705566	False	0,828705566
ENSMUSG00000019889	Ptprk	888,7645772	365,9783372	404,1928851	500,4482779	259,6451299	-0,828580346	False	0,828580346
ENSMUSG00000041459	Tardbp	1088,929817	546,4435172	584,7147618	363,2467275	613,2322164	-0,828405577	False	0,828405577
ENSMUSG00000029571	Tmem106b	2368,750808	899,8019118	722,9681016	549,9308042	1334,758633	-0,827547556	False	0,827547556
ENSMUSG00000037847	Nmrk1	326,9107967	184,2511629	838,3259839	668,0141057	186,5791637	-0,8272233	False	0,8272233
ENSMUSG00000037458	Azin1	843,939929	325,5945207	452,6255837	282,2753208	476,2335297	-0,825471114	False	0,825471114
ENSMUSG00000028760	Eif4g3	783,6585055	5036,619116	1388,697559	7374,021029	3062,246834	0,825435419	False	0,825435419
ENSMUSG00000096188	Cmtm4	460,6119026	259,9708188	173,4771206	133,8277417	246,5976359	-0,825201999	False	0,825201999
ENSMUSG00000027751	Supt20	454,4291925	1318,784008	826,8782551	1440,616279	805,0303776	0,824987708	False	0,824987708
ENSMUSG00000024228	Nudt12	761,2461814	212,0150367	429,7301262	172,0642394	181,3601661	-0,824932165	False	0,824932165
ENSMUSG00000039887	Alg14	170,0245278	58,05173624	95,98480277	55,10554071	84,80871077	-0,824864977	False	0,824864977
ENSMUSG00000018846	Pank3	730,3326308	169,1072317	353,1184029	96,71584696	412,3008093	-0,824856398	False	0,824856398
ENSMUSG00000042834	Nrep	260,4466631	3,785982798	147,059285	10,12142584	5,218997586	-0,824590077	False	0,824590077
ENSMUSG00000071266	Zfp946	263,5380181	85,8156101	148,820474	70,84998091	114,8179469	-0,824438081	False	0,824438081
ENSMUSG00000031090	Nadsyn1	214,8491761	157,7492833	380,416833	274,4031007	19,57124095	0,824256841	False	0,824256841
ENSMUSG00000028848	Gpn2	100,4690392	304,1406181	177,8800932	185,5594738	412,3008093	0,824154081	False	0,824154081
ENSMUSG00000004105	Angptl2	271,2664057	13,88193693	153,2234466	49,48252635	36,5329831	-0,824073313	False	0,824073313
ENSMUSG00000066568	Lsm14a	302,1799563	225,8969736	172,5965261	168,6904307	534,9472526	0,823988675	False	0,823988675
ENSMUSG00000062949	Atp11c	2272,145963	757,1965597	1283,906811	642,1482397	484,0620261	-0,823515024	False	0,823515024
ENSMUSG00000044252	Osbpl1a	1449,07268	364,7163429	818,9529044	573,5474645	345,7585901	-0,823277563	False	0,823277563
ENSMUSG00000029369	Afm	5133,195063	548,9675058	2901,558946	169,8150336	1137,741474	-0,823028846	False	0,823028846
ENSMUSG00000033898	Cfhr2	6926,953831	1590,112775	3916,003834	1564,322594	2640,812779	-0,822838881	False	0,822838881
ENSMUSG00000108414	Snhg1	51,78019711	328,1185092	91,58183017	170,9396365	270,0831251	0,822660939	False	0,822660939
ENSMUSG00000015340	Cybb	193,9825295	109,7935012	103,9101535	343,0038758	114,8179469	0,822298153	False	0,822298153
ENSMUSG00000031668	Eif2ak3	217,9405311	136,2953807	123,2832329	223,7959715	463,1860358	-0,821957924	False	0,821957924
ENSMUSG00000036553	Sh3tc1	82,69374762	234,7309335	146,1786905	670,2633115	177,4459179	0,821882856	False	0,821882856
ENSMUSG00000057522	Spop	1053,379234	360,9303601	553,8939536	596,039522	396,6438165	-0,821545023	False	0,821545023
ENSMUSG00000025227	Mfsd13a	113,6072981	20,19190826	200,7755508	116,9586986	7,828496379	0,821528081	False	0,821528081
ENSMUSG00000001248	Gramd1a	281,3133097	605,7572477	635,7892441	1246,059982	497,1095201	0,82138592	False	0,82138592
ENSMUSG00000036572	Upf3b	230,3059513	863,204078	406,8346686	1713,894776	448,8337924	0,820890932	False	0,820890932
ENSMUSG00000030447	Cyfip1	536,3501014	259,9708188	246,5664658	303,6427753	215,2836504	-0,820799981	False	0,820799981
ENSMUSG00000030614	Tmem126b	247,3084041	88,33959863	140,0145288	47,23332061	28,70448672	-0,820734728	False	0,820734728
ENSMUSG00000063511	Snrnp70	1585,092303	1528,275056	1595,637272	897,4330916	3049,19934	-0,820690566	False	0,820690566
ENSMUSG00000019647	Sema6a	92,74065154	27,76387386	163,7905809	122,581713	19,57124095	0,820578627	False	0,820578627
ENSMUSG00000045671	Spred2	129,0640734	426,554062	73,08934523	154,0705934	300,0923612	-0,82035445	False	0,82035445
ENSMUSG00000022948	Setd4	161,5233014	285,2107041	290,5961919	787,2220101	684,9934332	0,820285824	False	0,820285824
ENSMUSG00000006567	Atp7b	470,6588065	74,4576617	266,8201398	157,444402	18,26649155	-0,818814023	False	0,818814023
ENSMUSG00000038671	Arfrp1	197,8467233	243,5648934	264,1783563	221,5467657	112,2084481	-0,818201872	False	0,818201872
ENSMUSG00000021704	Mtx3	193,2096907	85,8156101	97,74599182	39,36110051	109,5989493	-0,817933489	False	0,817933489
ENSMUSG00000068747	Sort1	115,9258144	56,78974198	204,2979288	130,4539331	48,27572767	0,817472716	False	0,817472716
ENSMUSG00000031565	Fgfr1	57,96290721	377,3362856	102,1489644	482,4546319	350,9775877	0,817472716	False	0,817472716
ENSMUSG00000020918	Kat2a	370,1897674	336,9524691	734,4158304	748,9855125	210,0646528	-0,817431594	False	0,817431594
ENSMUSG00000021079	Timm9	166,9331728	410,1481365	294,11857	453,2149573	425,3483033	0,817127202	False	0,817127202
ENSMUSG00000042632	Pla2g6	441,2909336	1024,739344	544,2074139	2676,554834	250,5118841	-0,816851061	False	0,816851061
ENSMUSG00000002249	Tead3	253,4911142	978,0455563	446,4614221	1078,494154	831,1253656	0,816600344	False	0,816600344

ENSMUSG00000028073	Pear1	127,5183959	469,461867	224,5516028	1230,315542	486,6715249	0,816341634	False	0,816341634
ENSMUSG00000030695	Aldoa	1347,057964	3496,986111	3237,065459	2618,075485	2372,034403	0,816313003	False	0,816313003
ENSMUSG00000030225	Dera	750,4264387	147,6533291	426,2077481	97,84044983	176,1411685	-0,816153837	False	0,816153837
ENSMUSG00000022664	Slc35a5	177,7529154	100,9595413	85,41766852	58,47934932	45,66622888	-0,816095962	False	0,816095962
ENSMUSG00000079334	Nat6	1118,29769	1986,378975	2759,783228	4074,436204	635,4129561	-0,815537871	False	0,815537871
ENSMUSG00000031696	Vps35	1115,979173	413,9341193	425,3271536	368,8697419	634,1082067	-0,81550915	False	0,81550915
ENSMUSG00000058325	Dock1	180,0714317	87,07760436	81,01469592	102,3388613	87,41820957	-0,81521523	False	0,81521523
ENSMUSG00000026248	Mrpl44	227,2145963	75,71965597	81,89529044	35,98729189	129,1701903	-0,814782352	False	0,814782352
ENSMUSG00000027244	Atg13	788,2955381	649,9270471	448,2226111	1363,01868	1931,029107	-0,814521179	False	0,814521179
ENSMUSG00000015776	Med22	173,1158829	98,43555276	91,58183017	46,10871774	78,28496379	-0,814486709	False	0,814486709
ENSMUSG00000041720	Pi4ka	411,9230606	743,3146228	234,2381425	607,2855507	587,1372284	-0,814398876	False	0,814398876
ENSMUSG00000006445	Epha2	218,7133699	574,2073911	401,5511015	384,6141821	486,6715249	0,814370546	False	0,814370546
ENSMUSG00000041362	Shtn1	428,9255134	185,5131571	243,9246823	209,1761341	88,72295896	-0,814291384	False	0,814291384
ENSMUSG00000027566	Psma7	952,9101945	1444,983435	1069,041748	542,0585841	1360,853621	-0,813891474	False	0,813891474
ENSMUSG00000051495	Irf2bp2	843,1670902	1133,270851	275,626085	389,1125936	1482,195314	0,813845106	False	0,813845106
ENSMUSG00000045392	Olfr1033	145,2936874	26,50187959	255,372411	58,47934932	31,31398552	0,81363065	False	0,81363065
ENSMUSG00000025223	Ldb1	455,9748701	1633,02058	801,341014	1532,833714	1218,635936	0,813462002	False	0,813462002
ENSMUSG00000004056	Akt2	1418,931969	511,1076778	807,5051756	464,460986	532,3377538	-0,813262008	False	0,813262008
ENSMUSG00000021816	Ppp3cb	347,7774433	128,7234151	163,7905809	197,9301054	73,0659662	-0,813173298	False	0,813173298
ENSMUSG00000041000	Trim62	18,54813031	246,0888819	32,58199727	194,5562968	273,9973733	0,812801276	False	0,812801276
ENSMUSG00000005802	Slc30a4	124,4270408	174,1552087	104,790748	70,84998091	339,2348431	-0,81246068	False	0,81246068
ENSMUSG00000006276	Eps15l1	302,952795	571,6834026	531,8790906	815,3370819	610,6227176	0,812005307	False	0,812005307
ENSMUSG00000034826	Nup54	197,8467233	338,2144633	112,7160987	217,0483542	636,7177055	-0,811689589	False	0,811689589
ENSMUSG00000021765	Fst	30,91355051	286,4726984	17,61189042	300,2689667	661,507944	-0,811689589	False	0,811689589
ENSMUSG00000028743	Akr7a5	1585,865141	191,8231285	903,4899784	310,3903926	237,4643902	-0,811689589	False	0,811689589
ENSMUSG00000022663	Atg3	1372,561643	420,2440906	781,9679345	195,6808997	694,1266789	-0,811689589	False	0,811689589
ENSMUSG00000054843	Atml1	256,5824693	85,8156101	146,1786905	75,3483924	125,2559421	-0,811689589	False	0,811689589
ENSMUSG00000022237	Ankrd33b	315,3182152	70,6716789	179,6412823	49,48252635	140,9129348	-0,811689589	False	0,811689589
ENSMUSG00000027879	Sec22b	513,1649385	642,3550815	364,5661316	292,3967466	2639,508029	-0,811495344	False	0,811495344
ENSMUSG00000030551	Nr2f2	425,8341583	470,7238613	498,4164988	690,5061632	242,6833878	-0,811216324	False	0,811216324
ENSMUSG00000001034	Mapk7	48,68884206	278,9007328	85,41766852	219,29756	155,2651782	0,81094333	False	0,81094333
ENSMUSG00000032232	Cgnl1	1064,971815	182,9891686	553,0133591	607,2855507	224,4168962	-0,810368301	False	0,810368301
ENSMUSG00000087687	Pet100	268,1750507	259,9708188	461,4315289	152,9459905	225,7216456	-0,810152737	False	0,810152737
ENSMUSG00000045795	Whamm	497,7081632	1466,437337	872,6691702	1722,891599	1162,531712	0,81013478	False	0,81013478
ENSMUSG00000054604	Cggbp1	575,7648783	945,2337053	328,4617563	538,6847755	1071,199255	-0,809754384	False	0,809754384
ENSMUSG00000030602	Pak4	136,0196223	257,4468303	140,0145288	77,59759814	362,7203322	-0,809730887	False	0,809730887
ENSMUSG00000020889	Nr1d1	1973,057361	1125,698885	377,7750494	419,4768711	925,0673221	-0,809611929	False	0,809611929
ENSMUSG00000036478	Btg1	978,4138737	516,1556549	214,8650631	196,8055025	1714,440707	0,809221244	False	0,809221244
ENSMUSG00000035559	Mpv17l2	162,2961402	162,7972603	255,372411	211,4253399	92,63720715	-0,808965025	False	0,808965025
ENSMUSG00000021820	Camk2g	165,3874952	531,299586	289,7155974	482,4546319	379,6820744	0,808787199	False	0,808787199
ENSMUSG00000044709	Gemin7	143,7480099	146,3913349	185,8054439	82,09600963	177,4459179	-0,808157978	False	0,808157978
ENSMUSG00000042213	Zfand4	308,3626664	31,54985665	176,1189042	66,35156943	73,0659662	-0,808078335	False	0,808078335
ENSMUSG00000025574	Tk1	556,4439092	31,54985665	317,894622	74,22378953	46,97097827	-0,807687658	False	0,807687658
ENSMUSG00000025317	Car5a	1272,865442	45,43179358	727,3710742	4,498411486	86,11346017	-0,807316457	False	0,807316457
ENSMUSG00000025825	Iscu	1192,490211	1438,673463	1314,72762	681,5093402	1799,249418	-0,807172089	False	0,807172089
ENSMUSG00000022323	Rida	7974,150355	2529,036509	3467,781223	593,7903162	4557,489642	-0,807091431	False	0,807091431
ENSMUSG00000051306	Usp42	98,15052288	282,6867156	171,7159316	434,0967084	232,2453926	0,806956038	False	0,806956038
ENSMUSG00000027016	Zfp385b	454,4291925	69,40968464	259,7753837	39,36110051	44,36147948	-0,806790789	False	0,806790789
ENSMUSG00000063358	Mapk1	976,8681962	333,1664863	362,8049426	140,575359	558,4327417	-0,806780389	False	0,806780389
ENSMUSG00000043085	Tmem82	219,4862086	1042,407264	383,9392111	597,1641248	801,1161295	0,806747617	False	0,806747617
ENSMUSG00000018401	Mtmr4	661,549981	335,6904748	611,1325975	1157,216355	317,0541034	0,806736555	False	0,806736555
ENSMUSG00000021258	Ccnk	450,5649987	408,8861422	210,4620905	258,6586605	788,0686355	0,806586039	False	0,806586039
ENSMUSG00000037552	Plekhg2	107,424588	489,6537753	318,7752165	565,6752444	187,8839131	0,806517302	False	0,806517302
ENSMUSG00000037942	Crp	7500,400193	449,2699587	4288,495317	780,4743929	1398,691353	-0,806496029	False	0,806496029
ENSMUSG00000029561	Oasl2	134,4739447	82,0296273	116,2384768	235,0420002	45,66622888	0,805591911	False	0,805591911
ENSMUSG00000023074	Mospd1	109,7431045	90,86358716	69,56696715	28,11507179	191,7981613	0,805458599	False	0,805458599
ENSMUSG00000017747	Ghdc	254,263953	145,1293406	353,9989974	444,2181343	44,36147948	0,804941344	False	0,804941344
ENSMUSG00000040134	Rdh7	12617,36564	1563,610896	7224,397449	2361,66603	1706,612211	-0,804461554	False	0,804461554
ENSMUSG00000060261	Gtf2i	1202,537115	116,1034725	688,6249153	642,1482397	41,75198069	-0,804291136	False	0,804291136
ENSMUSG00000001143	Lman2l	259,6738243	217,0630138	277,3872741	548,8062014	148,7414312	-0,803894035	False	0,803894035
ENSMUSG00000032518	Rpsa	7332,694181	15615,91705	15853,34316	12801,35449	14935,46634	0,803881191	False	0,803881191
ENSMUSG00000075467	Dnlz	300,6342787	319,2845493	612,8937865	396,9848137	172,2269203	-0,803698852	False	0,803698852
ENSMUSG00000052914	Cyp2j6	1007,008908	329,3805035	549,490981	576,9212731	490,5857731	-0,803630079	False	0,803630079
ENSMUSG00000022377	Asap1	273,584922	305,4026124	156,7458247	391,3617993	395,3390671	-0,803561708	False	0,803561708
ENSMUSG00000047187	Rab2a	1238,087698	1139,580822	903,4899784	413,8538568	2160,665001	0,803361898	False	0,803361898
ENSMUSG00000038370	Pcp4l1	381,0095101	41,64581078	664,8488632	44,98411486	31,31398552	0,803199409	False	0,803199409
ENSMUSG00000047417	Rexo1	530,94023	2383,907169	926,3854359	1855,594738	2702,136	0,803063112	False	0,803063112
ENSMUSG00000063281	Zfp35	122,8813633	232,206945	70,44756167	119,2079044	203,5409059	-0,802644449	False	0,802644449
ENSMUSG00000037949	Ano10	122,8813633	314,2365723	147,059285	479,0808233	70,45646741	-0,80246208	False	0,80246208
ENSMUSG00000042747	Krtcap2	517,8019711	1350,333865	2070,277719	903,0561059	1068,589756	0,802415167	False	0,802415167
ENSMUSG00000030521	Mphosph10	97,37768411	297,8306468	172,5965261	169,8150336	263,5593781	0,80230109	False	0,80230109
ENSMUSG00000039783	Kmo	3809,322262	1322,569991	2184,755006	1304,539331	545,3852477	-0,802062834	False	0,802062834
ENSMUSG00000030101	Sumf1	272,8120833	156,487289	143,5369069	133,8277417	131,779689	-0,801862069	False	0,801862069
ENSMUSG00000030876	Mettl9	588,1302985	776,1264737	538,9238468	337,3808615	1230,378681	-0,801757679	False	0,801757679
ENSMUSG00000037936	Scarb1	2265,190414	802,6283533	988,9076469	859,1965939	1299,530399	-0,801641947	False	0,801641947
ENSMUSG00000004567	Mcoln1	655,3672709	283,9487099	376,0138604	142,8245647	257,0356311	-0,801517784	False	0,801517784
ENSMUSG00000021273	Fdft1	4540,427731	1641,85454	7913,022364	1734,137628	698,0409271	0,801400622	False	0,801400622
ENSMUSG00000024130	Abca3	1583,546625	668,8569611	795,1768523	908,6791203	468,4050333	-0,801316512	False	0,801316512
ENSMUSG00000026819	Slc25a25	805,2979908	2603,494171	462,3121234	2854,242088	2694,307504	-0,800655538	False	0,800655538
ENSMUSG00000038235	F11r	887,9917385	509,8456835	495,7747152	205,8023255	481,4525273	-0,800485606	False	0,800485606
ENSMUSG00000032872	Cyb5r4	343,1404107	159,0112775	143,5369069	155,1951963	197,0171589	-0,800477754	False	0,800477754
ENSMUSG00000022704	Qtrt2	55,64439092	123,6754381	96,86539729	156,3197992	136,9986866	0,799745123	False	0,799745123
ENSMUSG00000040151	Hs2st1	166,9331728	95,91156423	94,22361373	66,35156943	78,28496379	-0,799493995	False	0,799493995
ENSMUSG00000036052	Dnajb5	102,0147167	199,395094	246,5664658	363,2467275	177,4459179	0,798602095	False	0,798602095
ENSMUSG00000069495	Epc2	168,4788503	225,8969736	96,86539729	127,0801245	578,0039827	-0,7985142	False	0,7985142
ENSMUSG00000006050	Sra1	517,0291523	3615,613572	899,0870058	918,8005461	4160,845825	0,798215162	False	0,798215162
ENSMUSG00000025144	Cenpx	560,308103	410,1481365	1477,637606	430,7228998	322,2731009	-0,79793658	False	0,79793658
ENSMUSG00000061559	Wdr61	489,9797756	209,4910482	239,5217097	87,71902399	281,8258696	-0,797918155	False	0,797918155
ENSMUSG00000034450	Gulo	5203,52339	657,4990127	2993,140776	462,2117802	1367,377368	-0,797828692	False	0,797828692
ENSMUSG00000033467	Crlf2	99,6962004	294,044664	116,2384768	57,35474645	216,5883998	-0,797625639	False	0,797625639
ENSMUSG00000057137	Tmem140	469,8859678	494,7017523	244,8052768	175,438048	816,7731222	0,797624707	False	0,797624707
ENSMUSG00000051223	Bzw1	1493,12449	1977,545015	1194,08617	859,1965939	3389,738932	-0,797274276	False	0,797274276
ENSMUSG00000050079	Rspry1	256,5824693	147,6533291	132,9697726	131,578536	78,28496379	-0,797208718	False	0,797208718
ENSMUSG00000070811	Sult2a2	1707,200827	294,044664	917,5794907	62,97776081	982,4762956	-0,797138275	False	0,797138275
ENSMUSG00000038406	Scaf1	634,5006243	2169,368143	1102,50434	2419,020777	1659,641232	0,797090847	False	0,797090847
ENSMUSG00000039959	Hip1	102,0147167	118,627461	116,2384768	294,6459524	58,71372284	-0,79700765	False	0,79700765
ENSMUSG00000026810	Dpm2	239,5800165	599,4472764	486,96877	416,1030625	628,8892091	0,79643333	False	0,79643333
ENSMUSG00000039615	Stub1	516,2562936	1304,902071	1414,2348	1769,000317	896,3628354	0,79599537	False	0,79599537
ENSMUSG00000031311	Nono	1644,600887	651,1890413	816,3111208	522,9403353	947,2480619	-0,795923324	False	0,795923324
ENSMUSG00000020048	Hsp90b1	12091,06244	37936,80963	6967,263849	15163,02052	67068,03323	-0,795276916	False	0,795276916
ENSMUSG00000046753	Ccdc66	111,2887818	193,0851227	255,372411	298,019761	193,1029107	0,794928835	False	0,794928835
ENSMUSG00000038160	Atg5	636,0463018	228,4209622	241,2828987	110,2110814	366,6345804	-0,794788927	False	0,794788927
ENSMUSG00000020423	Btg2	310,6811826	538,8715516	110,9549096	201,303914	388,8153202	0,794506562	False	0,794506562
ENSMUSG00000019948	Actr6	126,7455571	47,95578211	73,08934523	67,4761723	49,58047707	-0,794202162	False	0,794202162
ENSMUSG00000056749	Nfil3	685,5079826	1089,101052	395,3869399	548,8062014	1779,678177	-0,793908248	False	0,793908248
ENSMUSG00000036594	H2-Aa	229,5331126	133,7713922	228,9545754	65,22696655	397,9485659	0,793879685	False	0,793879685
ENSMUSG00000032067	Pts	493,0711307	112,3174897	284,4320302	122,581713	88,72295896	-0,793711848	False	0,793711848
ENSMUSG00000005268	Prlr	7569,955682	1793,293852	4366,868229	802,9664503	3301,015973	-0,793685856	False	0,793685856
ENSMUSG00000027597	Ahcy	13059,42941	6326,377256	7160,994644	6116,715019	7533,623015	-0,793676117	False	0,793676117
ENSMUSG00000071866	Ppia	4923,755758	2139,080281	2840,797924	539,8093784	2810,4302	-0,793462988	False	0,793462988
ENSMUSG00000020894	Vamp2	452,1106762	497,2257409	334,6259179	260,9078662	795,8971319	-0,793135545	False	0,793135545
ENSMUSG00000039202	Abhd2	2844,046647	1116,864926	1641,428187	552,18001	1441,748083	-0,792993495	False	0,792993495
ENSMUSG00000046879	Irgm1	942,8632906	145,1293406	544,2074139	148,4475791	254,4261323	-0,792891993	False	0,792891993

ENSMUSG00000024131	Slc3a1	745,7894061	191,8231285	430,6107207	208,0515312	78,28496379	-0,792384066	False	0,792384066
ENSMUSG00000034639	Setmar	56,41722968	309,1885952	32,58199727	107,9618757	101,7704529	-0,792060782	False	0,792060782
ENSMUSG00000025962	Fastkd2	166,160334	53,00375918	95,98480277	66,35156943	62,62797103	-0,791698113	False	0,791698113
ENSMUSG00000041164	Zmiz2	581,1747496	1153,462759	1060,235803	1306,788537	1005,961785	0,791531571	False	0,791531571
ENSMUSG00000025647	Shisa5	546,3970053	204,4430711	309,0886768	285,6491294	315,749354	-0,791169794	False	0,791169794
ENSMUSG00000027690	Slc2a2	3288,428936	92,12558143	1900,322976	300,2689667	298,7876118	-0,791153856	False	0,791153856
ENSMUSG00000051786	Tubgcp6	107,424588	311,7125837	185,8054439	609,5347564	221,8073974	0,790468527	False	0,790468527
ENSMUSG00000022498	Txndc11	431,2440296	1421,005544	745,8635592	1831,978078	1912,762615	0,790407259	False	0,790407259
ENSMUSG00000024121	Atp6v0c	316,8638927	267,5427844	174,3577151	55,10554071	547,9947465	0,790298791	False	0,790298791
ENSMUSG00000063931	Pepd	473,7501616	180,4651801	229,8351699	103,4634642	273,9973733	-0,789964373	False	0,789964373
ENSMUSG00000032288	Imp3	320,7280866	296,5686525	335,5065124	185,5594738	507,5475152	-0,789469038	False	0,789469038
ENSMUSG00000032855	Pkd1	342,3675719	687,786875	591,759518	1041,382259	888,534339	0,789464946	False	0,789464946
ENSMUSG00000018604	Tbx3	370,9626061	129,9854094	641,0728112	226,0451772	105,6847011	0,789214456	False	0,789214456
ENSMUSG00000060904	Arl1	996,962004	2353,619306	1003,877754	576,9212731	3097,475067	-0,78916406	False	0,78916406
ENSMUSG00000016319	Slc25a5	4557,430184	2006,570883	2617,126916	841,202948	2638,20328	-0,788664824	False	0,788664824
ENSMUSG00000017858	Ift52	380,2366713	151,4393119	220,1486302	129,3293302	100,4657035	-0,788419809	False	0,788419809
ENSMUSG00000061778	Mospd2	472,9773228	164,0592546	273,864896	101,2142584	208,7599034	-0,788306661	False	0,788306661
ENSMUSG00000017715	Pgs1	124,4270408	797,5803762	214,8650631	680,3847373	690,2124308	0,788130871	False	0,788130871
ENSMUSG00000031216	Stard8	157,6591076	35,33583945	144,4175014	272,1538949	31,31398552	0,78761417	False	0,78761417
ENSMUSG00000062300	Nectin2	470,6588065	652,4510356	433,2525043	565,6752444	272,6926239	-0,787405913	False	0,787405913
ENSMUSG00000042473	Tbc1d8b	261,2195018	65,62370184	89,82064113	52,85633497	151,35093	-0,787365068	False	0,787365068
ENSMUSG00000056185	Snx32	105,1060717	20,19190826	181,4024713	100,0896556	2,609498793	0,787348097	False	0,787348097
ENSMUSG00000031157	Pqbp1	428,9255134	483,3438039	634,028055	248,5372346	480,1477779	-0,787265127	False	0,787265127
ENSMUSG00000026496	Parp1	490,7526144	95,91156423	284,4320302	190,0578853	83,50396138	-0,786912016	False	0,786912016
ENSMUSG00000035575	Utp6	463,7032577	210,7530424	211,342685	110,2110814	268,7783757	-0,78678479	False	0,78678479
ENSMUSG00000026430	Rassf5	180,0714317	83,29162157	100,3877754	48,35792348	104,3799517	-0,786724677	False	0,786724677
ENSMUSG00000024168	Tmem204	173,1158829	63,09971331	100,3877754	88,84362686	50,88522646	-0,786154497	False	0,786154497
ENSMUSG00000067144	Slc22a7	353,9601534	29,02586812	610,252003	0	37,8377325	0,785818165	False	0,785818165
ENSMUSG00000018861	Fdxr	276,6762771	508,5836893	791,6544742	1359,644872	160,4841758	-0,785767899	False	0,785767899
ENSMUSG00000022241	Tars	2490,086494	927,5657856	589,998329	738,8640867	1444,357582	-0,785767899	False	0,785767899
ENSMUSG00000032370	Lactb	596,6315249	137,557375	346,0736467	120,3325073	125,2559421	-0,785761124	False	0,785761124
ENSMUSG00000026074	Map4k4	251,9454367	644,87907	146,1786905	672,5125172	832,430115	-0,785378312	False	0,785378312
ENSMUSG00000018387	Shroom1	862,4880593	3064,122078	1486,443551	1942,189159	2433,357624	0,785288288	False	0,785288288
ENSMUSG00000005893	Nr2c2	330,7749905	324,3325264	191,9696055	475,7070147	519,2902598	-0,78497225	False	0,78497225
ENSMUSG00000001946	Esam	96,60484535	319,2845493	166,4323644	345,2530816	262,2546287	0,784768551	False	0,784768551
ENSMUSG00000029474	Rnf34	122,8813633	55,52774771	71,32815619	40,48570338	46,97097827	-0,784722541	False	0,784722541
ENSMUSG00000018415	Gid4	465,2489352	140,0813635	195,4919836	143,9491676	270,0831251	-0,784599346	False	0,784599346
ENSMUSG00000019312	Grb7	3609,929861	1259,470278	2095,81496	1482,226585	258,3403805	-0,78445946	False	0,78445946
ENSMUSG00000025757	Hspa4l	485,342743	90,86358716	281,7902467	191,1824882	129,1701903	-0,784382243	False	0,784382243
ENSMUSG00000032384	Csnk1g1	80,37523133	147,6533291	46,67150961	148,4475791	232,2453926	-0,784208852	False	0,784208852
ENSMUSG00000038608	Dock10	80,37523133	94,64956996	46,67150961	136,0769475	150,0461806	-0,784208852	False	0,784208852
ENSMUSG00000074264	Amy1	2802,313354	807,6763303	1627,338675	906,4299145	1073,808753	-0,784103757	False	0,784103757
ENSMUSG00000070953	Rabepk	96,60484535	141,3433578	143,5369069	143,9491676	56,10422405	-0,783986157	False	0,783986157
ENSMUSG00000062075	Lmnb2	78,05671504	209,4910482	211,342685	156,3197992	134,3891878	0,783822418	False	0,783822418
ENSMUSG00000042363	Lgalsl	498,481002	99,69754702	164,6711754	97,84044983	289,654366	-0,783206111	False	0,783206111
ENSMUSG00000033411	Ctdspl2	77,28387628	102,2215356	44,91032056	139,4507561	174,8364191	-0,783120436	False	0,783120436
ENSMUSG00000044408	Sptssa	260,4466631	146,3913349	144,4175014	105,7126699	151,35093	-0,783090413	False	0,783090413
ENSMUSG00000033554	Dph5	75,73819875	213,277031	130,3279891	211,4253399	156,5699276	0,783053933	False	0,783053933
ENSMUSG00000047084	Ngrn	192,4368519	69,40968464	111,8355041	52,85633497	69,15171802	-0,783006834	False	0,783006834
ENSMUSG00000025577	Cbx2	190,8911744	39,12182225	110,9549096	80,97140676	32,61873491	-0,782776897	False	0,782776897
ENSMUSG00000026313	Hdac4	81,92090886	307,9266009	140,8951233	308,1411868	267,4736263	0,782318052	False	0,782318052
ENSMUSG00000030660	Pik3c2a	337,7305393	425,2920677	196,3725782	412,7292539	728,0501633	-0,782279158	False	0,782279158
ENSMUSG00000022564	Grina	2538,775336	6495,484488	4365,987634	8560,477059	5456,461976	0,782175312	False	0,782175312
ENSMUSG00000020978	Klhdc2	373,2811224	347,0484232	246,5664658	247,4126318	641,9367031	0,7821685	False	0,7821685
ENSMUSG00000052533	Nup188	129,0640734	489,6537753	221,9098193	445,3427372	434,481549	0,781886042	False	0,781886042
ENSMUSG00000031808	Slc27a1	91,96781277	68,14769037	141,7757179	248,5372346	53,49472526	-0,781732386	False	0,781732386
ENSMUSG00000001847	Rac1	1826,990835	1851,345588	992,430025	981,7783069	3140,531797	0,781539479	False	0,781539479
ENSMUSG00000042650	Alkbh5	660,7771422	748,3625998	423,5659645	384,6141821	1435,224336	-0,780751821	False	0,780751821
ENSMUSG00000049969	Plekhf2	123,654202	141,3433578	92,46242469	71,97458378	191,7981613	-0,780751821	False	0,780751821
ENSMUSG00000033423	Eri3	452,1106762	1211,514495	776,6843674	1544,079743	969,4288016	0,780652442	False	0,780652442
ENSMUSG00000037475	Thoc2	505,4365509	1516,917108	868,2661976	3235,482462	872,8773463	0,780607422	False	0,780607422
ENSMUSG00000027189	Trim44	789,0683768	459,3659129	422,68537	278,9015122	390,1200696	-0,780506517	False	0,780506517
ENSMUSG00000037234	Hook3	952,9101945	403,8381652	554,7745481	548,8062014	482,7572767	-0,780438655	False	0,780438655
ENSMUSG00000036916	Zfp280c	106,6517493	241,0409048	183,1636603	301,3935696	257,0356311	0,780225673	False	0,780225673
ENSMUSG00000069919	Hba-a1	2514,044495	2015,404843	2946,469267	4153,158405	1463,928823	-0,780164773	False	0,780164773
ENSMUSG00000061947	Serpina10	2674,794958	2457,102836	5596,17818	1557,574977	3034,847096	-0,780126691	False	0,780126691
ENSMUSG00000092341	Malat1	10832,1081	23889,55146	18599,91747	31667,69226	20839,45736	0,779982178	False	0,779982178
ENSMUSG00000021270	Hsp90aa1	3388,897975	3469,222238	3835,869733	1973,67804	4999,799687	-0,779929538	False	0,779929538
ENSMUSG00000045160	Bola3	646,8660445	596,9232879	894,6840332	376,741962	734,5739102	-0,779890253	False	0,779890253
ENSMUSG00000027305	Ndufaf1	346,2317657	166,5832431	201,6561453	68,60077517	157,874677	-0,779840723	False	0,779840723
ENSMUSG00000042308	Setd1a	170,7973666	983,0935333	293,2379754	1356,271063	1110,341736	0,779786219	False	0,779786219
ENSMUSG00000023043	Krt18	2480,03959	7917,752026	5081,91098	4257,746472	9446,385631	0,779726896	False	0,779726896
ENSMUSG00000029613	Eif2ak1	689,3721764	590,6133166	365,4467262	401,4832252	1183,407703	0,779592214	False	0,779592214
ENSMUSG00000037001	Zfp39	136,0196223	37,85982798	79,25350688	60,72855507	33,92348431	-0,779268111	False	0,779268111
ENSMUSG00000020792	Exoc7	506,2093896	265,0187959	294,9991645	253,0356461	213,978901	-0,7790234	False	0,7790234
ENSMUSG00000036611	Eepd1	229,5331126	133,7713922	107,4325315	112,4602872	76,98021439	-0,778932672	False	0,778932672
ENSMUSG00000007594	Hapln4	353,1873146	697,8828292	605,8490303	1421,49803	1895,800873	0,778524811	False	0,778524811
ENSMUSG00000048874	Phf3	794,4782482	1019,691367	463,192718	724,2442493	2323,758675	-0,778395149	False	0,778395149
ENSMUSG00000032849	Abcc4	127,5183959	41,64581078	60,76102194	66,35156943	74,3707156	-0,777898827	False	0,777898827
ENSMUSG00000034245	Hdac11	910,4040626	26,50187959	530,9984961	64,10236368	41,75198069	-0,777799221	False	0,777799221
ENSMUSG00000032553	Srprb	418,1057707	937,6617397	716,80394	764,7299527	1574,832522	0,777710612	False	0,777710612
ENSMUSG00000020952	Scfd1	655,3672709	307,9266009	321,4170001	291,2721437	382,2915732	-0,777630227	False	0,777630227
ENSMUSG00000026718	Stam	251,1725979	205,7050654	115,3578822	229,4189858	430,5673008	0,77755968	False	0,77755968
ENSMUSG00000018659	Pnpo	2285,284222	1269,566232	3916,884429	1675,658279	848,0871077	0,777332956	False	0,777332956
ENSMUSG00000021051	Ppp2r5e	523,2118424	199,395094	221,9098193	166,441225	305,3113588	-0,777113931	False	0,777113931
ENSMUSG00000040699	Limd2	253,4911142	78,2436445	147,9398795	61,85315794	73,0659662	-0,77692417	False	0,77692417
ENSMUSG00000024608	Rps14	1670,104566	4594,921123	3653,586667	2860,989705	3995,142652	0,776575873	False	0,776575873
ENSMUSG00000026781	Acbd5	2208,000345	349,5724117	1289,190379	657,8926799	343,1490913	-0,776275071	False	0,776275071
ENSMUSG00000004069	Dnaja3	987,6879389	358,4063716	576,7894112	345,2530816	136,9986866	-0,776010613	False	0,776010613
ENSMUSG00000008226	Scrn3	768,2017302	359,6683658	421,8047755	448,7165458	332,7110961	-0,775680831	False	0,775680831
ENSMUSG00000051671	Coa6	169,2516891	53,00375918	289,7155974	46,10871774	49,58047707	0,775467125	False	0,775467125
ENSMUSG00000025742	Prps2	180,8442705	35,33583945	105,6713425	29,23967466	44,36147948	-0,775163713	False	0,775163713
ENSMUSG00000069631	Strada	58,73574597	113,579484	150,5816631	235,0420002	100,4657035	0,7743924	False	0,7743924
ENSMUSG00000061136	Prpf40a	755,83631	826,6062443	559,1775207	441,9689285	1293,006652	-0,774128878	False	0,774128878
ENSMUSG00000021902	Phf7	105,8789105	66,88569611	96,86539729	181,0610623	49,58047707	0,77406107	False	0,77406107
ENSMUSG00000021215	Net1	1078,110074	368,5023257	630,5056769	332,88245	306,6161082	-0,773923218	False	0,773923218
ENSMUSG00000031352	Hccs	121,3356858	95,91156423	59,88042742	65,22696655	207,455154	0,77379558	False	0,77379558
ENSMUSG00000032519	Slc25a38	312,2268602	252,3988532	607,6102194	927,7973691	182,6649155	-0,773395093	False	0,773395093
ENSMUSG00000026393	Nek7	717,9672106	1328,879962	420,0435864	453,2149573	1704,002712	-0,773378919	False	0,773378919
ENSMUSG00000020876	Snx11	63,37277855	181,7271743	108,3131261	185,5594738	147,4366818	0,773272912	False	0,773272912
ENSMUSG00000032834	Pwp2	92,74065154	235,9929278	158,5070138	258,6586605	285,7401178	0,773272912	False	0,773272912
ENSMUSG00000057363	Uxs1	133,701106	78,2436445	62,52221098	48,35792348	54,79947465	-0,772965923	False	0,772965923
ENSMUSG00000038722	Bud31	422,7428033	547,7055115	524,8343344	247,4126318	677,1649368	-0,772861035	False	0,772861035
ENSMUSG00000073434	Wdr90	64,91845608	195,6091113	142,6563124	485,8284405	110,9036987	0,772606887	False	0,772606887
ENSMUSG00000027883	Gpsm2	166,9331728	39,12182225	97,74599182	71,97458378	18,26649155	-0,772161224	False	0,772161224
ENSMUSG00000022218	Tgm1	398,7848016	811,4623131	437,6554769	638,7744311	233,550142	-0,771878105	False	0,771878105
ENSMUSG00000024639	Gnaq	255,8096305	686,5248808	436,7748823	501,5728807	583,2229802	0,771819315	False	0,771819315
ENSMUSG00000019822	Smpd2	229,5331126	497,2257409	391,8645618	612,908565	750,230903	0,771652816	False	0,771652816
ENSMUSG00000038696	Mapkap1	464,4760964	199,395094	272,1037069	119,2079044	164,398424	-0,771447741	False	0,771447741
ENSMUSG00000050029	Rap2c	558,7624255	482,0818097	372,4914823	269,9046892	953,7718088	0,771409132	False	0,771409132
ENSMUSG00000020671	Rab10	1129,117432	507,321695	558,2969262	269,9046892	661,507944	-0,771365153	False	0,771365153
ENSMUSG00000033618	Map3k13	299,0886012	36,59783372	175,2383097	46,10871774	2,609498793	-0,771254724	False	0,771254724
ENSMUSG00000040234	Tm7sf3	988,4607776	503,5357122	482,5657974	423,9752826	579,3087321	-0,770851309	False	0,770851309

ENSMUSG00000026709	Dars2	166,9331728	49,21777638	89,82064113	97,84044983	15,65699276	-0,770767732	False	0,770767732
ENSMUSG00000025583	Rptor	450,5649987	950,2816824	264,1783563	1122,353666	456,6622888	-0,770222971	False	0,770222971
ENSMUSG00000079057	Cyp4v3	3348,710359	904,8498888	1093,698395	629,7776081	1963,647842	-0,770069376	False	0,770069376
ENSMUSG00000034709	Ppp1r21	293,6787299	395,0042053	329,3423508	465,5855889	172,2269203	-0,769928116	False	0,769928116
ENSMUSG00000041355	Ssr2	1667,78605	2234,991845	2300,112888	978,4044983	3054,418337	-0,769431284	False	0,769431284
ENSMUSG00000017721	Pigt	780,5671504	368,5023257	397,1481289	326,1348328	457,9670382	-0,769278984	False	0,769278984
ENSMUSG00000074918	Inafm2	95,83200659	220,8489966	107,4325315	56,23014358	207,455154	-0,769163846	False	0,769163846
ENSMUSG00000019987	Arg1	16248,16215	20019,01504	10309,12006	6397,865737	27685,47744	0,76885286	False	0,76885286
ENSMUSG00000020413	Hus1	136,0196223	51,74176491	55,47745481	79,84680388	78,28496379	-0,768508225	False	0,768508225
ENSMUSG00000017405	Nek8	88,87645772	328,1185092	132,0891781	535,3109669	52,18997586	-0,768028583	False	0,768028583
ENSMUSG00000002413	Braf	274,3577608	378,5982798	161,1487973	305,8919811	657,5936958	-0,767664965	False	0,767664965
ENSMUSG00000000078	Klf6	199,3924008	885,9199748	117,1190713	383,4895792	1764,021184	-0,767634409	False	0,767634409
ENSMUSG00000066232	Ipo7	1041,786652	417,7201021	410,3570467	330,6332443	611,927467	-0,767627295	False	0,767627295
ENSMUSG00000024006	Stk38	832,3473475	561,5874484	863,863225	1416,999618	270,0831251	0,767581758	False	0,767581758
ENSMUSG00000002580	Mien1	377,1453162	377,3362856	666,6100523	221,5467657	347,0633395	-0,767509244	False	0,767509244
ENSMUSG00000006134	Crkl	373,2811224	256,184836	230,7157645	170,9396365	635,4129561	0,767431956	False	0,767431956
ENSMUSG00000026254	Eif4e2	598,9500417	940,1857283	464,0733125	352,0006988	1432,614837	-0,766857379	False	0,766857379
ENSMUSG00000033256	Shf	104,333233	164,0592546	164,6711754	140,575359	61,32322164	-0,766693372	False	0,766693372
ENSMUSG00000017718	Afmid	411,9230606	6,309971331	242,1634932	6,74761723	24,79023853	-0,766393503	False	0,766393503
ENSMUSG00000040325	Dcaf1	290,5873748	169,1072317	170,835337	160,8182106	62,62797103	-0,766365598	False	0,766365598
ENSMUSG00000090266	Mettl23	193,2096907	44,16979931	113,5966932	62,97776081	24,79023853	-0,766246618	False	0,766246618
ENSMUSG00000023832	Acat2	922,7694828	397,5281938	1568,338842	438,5951199	172,2269203	0,765195091	False	0,765195091
ENSMUSG00000026361	Cdc73	238,0343389	208,2290539	93,34301921	165,3166221	404,4723129	0,764871238	False	0,764871238
ENSMUSG00000002059	Rab34	44,05180948	492,1777638	74,85053427	322,7610242	182,6649155	0,764811333	False	0,764811333
ENSMUSG00000073684	Faap20	132,1554284	444,2219817	224,5516028	347,5022873	267,4736263	0,764811333	False	0,764811333
ENSMUSG00000022792	Yars2	168,4788503	56,78974198	82,77588496	24,74126318	99,16095413	-0,764723438	False	0,764723438
ENSMUSG00000041058	Wwp1	1064,971815	319,2845493	626,9832988	368,8697419	392,7295683	-0,76431633	False	0,76431633
ENSMUSG00000032387	Rbpms2	969,1398086	408,8861422	570,6252495	328,3840385	309,225607	-0,764161219	False	0,764161219
ENSMUSG00000059866	Tnip2	77,28387628	855,6321124	131,2085836	610,6593593	1145,56997	0,763622742	False	0,763622742
ENSMUSG00000032112	Trappc4	190,8911744	107,2695126	70,44756167	112,4602872	62,62797103	-0,763334767	False	0,763334767
ENSMUSG00000019494	Cops6	792,1597319	248,6128704	466,7150961	280,026115	212,6741516	-0,763249234	False	0,763249234
ENSMUSG00000070923	Klhl9	608,9969451	172,8932145	251,850033	79,84680388	358,806084	-0,763230638	False	0,763230638
ENSMUSG00000067851	Arfgef1	877,9448345	398,7901881	296,7603535	517,3173209	424,0435539	-0,763080794	False	0,763080794
ENSMUSG00000022401	Xpnpep3	248,0812429	82,0296273	146,1786905	109,0864785	62,62797103	-0,763079644	False	0,763079644
ENSMUSG00000031644	Nek1	149,1578812	59,31373051	124,1638274	253,0356461	122,6464433	0,762500428	False	0,762500428
ENSMUSG00000035517	Tdrd7	355,5058309	162,7972603	209,581496	159,6936078	139,6081854	-0,762361876	False	0,762361876
ENSMUSG00000027215	Cd82	1102,068076	336,9524691	649,8787564	238,4158088	575,3944839	-0,761970849	False	0,761970849
ENSMUSG00000040250	Ints13	261,2195018	306,6646067	175,2383097	154,0705934	576,6992333	-0,761671079	False	0,761671079
ENSMUSG00000034560	Washc4	253,4911142	97,17355849	117,1190713	149,5721819	146,1319324	-0,761093295	False	0,761093295
ENSMUSG00000025903	Lypla1	1817,71677	427,8160562	1072,564126	366,6205361	790,6781343	-0,761063516	False	0,761063516
ENSMUSG00000006673	Qrich1	457,5205476	386,1702454	323,1781892	238,4158088	775,0211415	0,760399128	False	0,760399128
ENSMUSG00000031807	Pgls	724,9227595	671,3809496	1286,548595	1418,124221	427,9578021	-0,760358735	False	0,760358735
ENSMUSG00000013787	Ehmt2	972,2311636	2931,61268	1840,442549	5155,179564	1646,593738	0,760113361	False	0,760113361
ENSMUSG00000003644	Rps6ka1	187,7998194	204,4430711	194,6113891	268,7800863	110,9036987	-0,759888194	False	0,759888194
ENSMUSG00000023272	Creld2	617,4981715	2025,500797	1045,265696	3329,949103	9400,719402	0,759362938	False	0,759362938
ENSMUSG00000053110	Yap1	629,0907529	552,7534886	434,1330988	378,9911677	1064,675508	0,759073731	False	0,759073731
ENSMUSG00000017057	Il13ra1	553,3525542	714,2887546	386,5809947	327,2594356	1324,320637	-0,75776416	False	0,75776416
ENSMUSG00000031584	Gsr	516,2562936	474,5098441	217,5068467	217,0483542	872,8773463	0,757691481	False	0,757691481
ENSMUSG00000033735	Spr	860,169543	228,4209622	508,9836331	233,9173973	180,0554167	-0,757001783	False	0,757001783
ENSMUSG00000009575	Cbx5	279,7676321	150,1773177	165,5517699	94,46664122	60,01847224	-0,756946624	False	0,756946624
ENSMUSG00000025279	Dnase1l3	757,3819875	20,19190826	448,2226111	57,35474645	73,0659662	-0,756805682	False	0,756805682
ENSMUSG00000024165	Jpt2	553,3525542	214,5390252	279,1484631	212,5499427	327,4920985	-0,75673885	False	0,75673885
ENSMUSG00000054312	Mrps21	336,1848618	324,3325264	567,983466	157,444402	270,0831251	0,756594171	False	0,756594171
ENSMUSG00000024937	Ehbp1l1	207,1207884	194,347117	212,2232795	402,607828	122,6464433	-0,755966966	False	0,755966966
ENSMUSG00000028849	Map7d1	537,1229401	1005,80943	907,0123565	1066,123522	942,0290643	0,755869866	False	0,755869866
ENSMUSG00000002210	Smg9	105,8789105	437,9120103	178,7606877	459,9625745	452,7480406	0,755614246	False	0,755614246
ENSMUSG00000001507	Itga3	141,4294936	386,1702454	355,7601864	495,9498664	238,7691396	0,755533373	False	0,755533373
ENSMUSG00000012640	Zfp715	149,1578812	384,9082512	401,5511015	1022,26401	251,8166335	0,755533373	False	0,755533373
ENSMUSG00000028582	Cc2d1b	183,9356255	530,0375918	376,0138604	918,8005461	310,5303564	0,755533373	False	0,755533373
ENSMUSG00000030019	Fbxl14	98,92336164	79,50563877	42,268537	14,61983733	167,0079228	0,755533373	False	0,755533373
ENSMUSG00000074165	Zfp788	61,05426226	109,7935012	107,4325315	159,6936078	103,0752023	0,755533373	False	0,755533373
ENSMUSG00000033099	Nol12	164,6146565	420,2440906	418,2823974	277,7769093	377,0725756	0,754833886	False	0,754833886
ENSMUSG00000071054	Safb	425,8341583	2113,840396	718,565129	1722,891599	3319,282465	0,754827245	False	0,754827245
ENSMUSG00000026663	Atf6	997,7348428	672,6429439	302,0439207	287,8983351	1683,126721	0,754415438	False	0,754415438
ENSMUSG00000037364	Srrt	871,7621244	3016,166296	1470,59285	3886,627524	3795,515994	0,754391446	False	0,754391446
ENSMUSG00000022371	Col14a1	591,9944923	185,5131571	173,4771206	55,10554071	350,9775877	-0,754204846	False	0,754204846
ENSMUSG00000022711	Pmm2	817,663411	1991,426952	1692,502669	2302,062078	1379,120112	0,754169122	False	0,754169122
ENSMUSG00000064225	Paqr9	5444,649084	1457,603377	3228,259513	831,0815221	1167,75071	-0,754082509	False	0,754082509
ENSMUSG00000049521	Cdc42ep1	349,3231208	1168,60669	589,1177345	673,6371201	914,629327	0,75399385	False	0,75399385
ENSMUSG00000026389	Steap3	510,8464222	20,19190826	302,9245152	31,48888041	45,66622888	-0,753931295	False	0,753931295
ENSMUSG00000052751	Repin1	792,9325706	161,5352661	470,2374741	121,4571101	165,7031734	-0,753808673	False	0,753808673
ENSMUSG00000035811	Ugt2b35	1928,232713	106,0075184	1143,892283	293,5213495	144,827183	-0,753327973	False	0,753327973
ENSMUSG00000060216	Arrb2	327,6836354	310,4505895	479,0434193	495,9498664	194,4076601	-0,753218561	False	0,753218561
ENSMUSG00000029840	Mtpn	1558,815785	526,251609	501,9388769	215,9237514	925,0673221	-0,752820178	False	0,752820178
ENSMUSG00000017291	Taok1	458,2933863	663,808984	272,1037069	383,4895792	803,7256283	-0,752114855	False	0,752114855
ENSMUSG00000016018	Skiv2l2	300,6342787	208,2290539	109,1937206	278,9015122	506,2427658	0,75181987	False	0,75181987
ENSMUSG00000054477	Kcnn2	455,2020313	45,43179358	270,3425179	167,5658279	32,61873491	-0,751718567	False	0,751718567
ENSMUSG00000031645	F11	808,3893459	1190,060593	692,1472934	2639,44294	480,1477779	-0,751571807	False	0,751571807
ENSMUSG00000024078	Ttc27	125,1998796	59,31373051	62,52221098	35,98729189	74,3707156	-0,751426616	False	0,751426616
ENSMUSG00000029456	Acad10	379,4638325	68,14769037	225,4321973	185,5594738	61,32322164	-0,751268803	False	0,751268803
ENSMUSG00000028840	Zfp593	64,14561731	150,1773177	125,044422	107,9618757	114,8179469	0,751099344	False	0,751099344
ENSMUSG00000058317	Ube2e2	214,8491761	225,8969736	127,6862055	265,4062777	348,3680889	-0,750721571	False	0,750721571
ENSMUSG00000005505	Kbtbd4	164,6146565	70,6716789	96,86539729	97,84044983	67,84696862	-0,75058985	False	0,75058985
ENSMUSG00000038991	Txndc5	2492,40501	2264,017713	2578,380757	1482,226585	4006,885397	-0,749772516	False	0,749772516
ENSMUSG00000025967	Eef1b2	1261,272861	1698,644282	1404,548261	750,1101154	1449,57658	-0,749706116	False	0,749706116
ENSMUSG00000040688	Tbl3	141,4294936	335,6904748	237,7605206	377,8665649	288,3496166	0,74942617	False	0,74942617
ENSMUSG00000053329	D10Jhu81e	944,4089681	103,4835298	561,8193043	138,3261532	133,0844384	-0,749305545	False	0,749305545
ENSMUSG00000029260	Ugt2b34	7994,244162	754,6725712	4757,852196	190,0578853	2451,624116	-0,748651182	False	0,748651182
ENSMUSG00000028266	Lmo4	211,757821	1155,986748	355,7601864	2063,646269	1486,109563	0,748489811	False	0,748489811
ENSMUSG00000020124	Usp15	548,7155216	691,5728578	326,7005672	558,9276272	784,1543873	-0,748089426	False	0,748089426
ENSMUSG00000022193	Psmb5	336,1848618	691,5728578	549,490981	200,1793111	675,8601874	-0,747961885	False	0,747961885
ENSMUSG00000029554	Mad1l1	146,8393649	826,6062443	618,1773536	503,8220865	246,5976359	0,747920189	False	0,747920189
ENSMUSG00000075700	Selenot	1590,502174	743,3146228	866,5050085	409,3554453	947,2480619	-0,747668156	False	0,747668156
ENSMUSG00000034932	Mrpl54	275,1305996	605,7572477	841,8483619	480,2054262	461,8812864	0,747405492	False	0,747405492
ENSMUSG00000035521	Gnptg	311,4540214	304,1406181	341,6706741	185,5594738	726,7454139	-0,74713754	False	0,74713754
ENSMUSG00000026504	Sdccag8	91,96781277	283,9487099	56,35804933	124,8309187	54,79947465	-0,746966967	False	0,746966967
ENSMUSG00000009739	Pou6f1	78,82955381	253,6608475	119,7608548	413,8538568	46,97097827	-0,746966967	False	0,746966967
ENSMUSG00000021259	Cyp46a1	78,82955381	54,26575344	71,32815619	309,2657897	46,97097827	-0,746966967	False	0,746966967
ENSMUSG00000036822	Topors	350,0959595	464,4138899	208,7009014	280,026115	718,9169175	-0,74631358	False	0,74631358
ENSMUSG00000034674	Tdg	118,2443307	94,64956996	75,73112879	97,84044983	198,3219083	0,746073044	False	0,746073044
ENSMUSG00000020570	Sypl	647,6388832	267,5427844	276,5066795	264,2816748	386,2058214	-0,745819695	False	0,745819695
ENSMUSG00000059316	Slc27a4	413,4687381	673,9049381	246,5664658	524,0649382	250,5118841	-0,745801653	False	0,745801653
ENSMUSG00000025791	Pgm2	2810,041742	899,8019118	1675,771373	1246,059982	1201,674194	-0,745766227	False	0,745766227
ENSMUSG00000038375	Trp53inp2	2024,06472	530,0375918	1207,295088	775,9759814	481,4525273	-0,745477077	False	0,745477077
ENSMUSG00000031939	Taf1d	175,4343992	1369,263779	294,11857	1304,539331	2018,447316	0,745466217	False	0,745466217
ENSMUSG00000027207	Galk2	169,2516891	100,9595413	58,9998329	53,98093784	82,19921198	-0,745392971	False	0,745392971
ENSMUSG00000047648	Fbxo30	109,7431043	126,1994266	73,96993975	43,85951199	183,9696649	0,745337606	False	0,745337606
ENSMUSG00000018166	Erbb3	2194,862086	612,0672191	1310,324647	872,6918284	157,874677	-0,744205992	False	0,744205992
ENSMUSG00000060807	Serpina6	7513,538452	803,8903475	4485,748489	89,96822973	2244,168962	-0,744143763	False	0,744143763
ENSMUSG00000032249	Anp32a	2578,190113	220,8489966	1539,279222	152,9459905	211,3694022	-0,744103693	False	0,744103693
ENSMUSG00000059447	Hadhb	1412,749258	628,4731445	843,609551	727,6180579	785,4591367	-0,743858098	False	0,743858098
ENSMUSG00000070003	Ssbp4	92,74065154	198,1330998	290,5961919	214,7991485	155,2651782	0,743460541	False	0,743460541
ENSMUSG00000073838	Tufm	1099,749559	165,3212489	656,9235126	320,5118184	237,4643902	-0,743377715	False	0,743377715

ENSMUSG00000027435	Cd93	62,59993979	585,5653395	104,790748	321,6364213	506,2427658	0,743278172	False	0,743278172
ENSMUSG00000015468	Notch4	50,23451958	137,557375	57,23864386	327,7610242	30,00923612	-0,743272484	False	0,743272484
ENSMUSG00000027859	Ngf	87,3307802	306,6646067	146,1786905	267,6554834	202,2361565	0,74317088	False	0,74317088
ENSMUSG00000070661	Rnf186	142,9751711	27,76387386	85,41766852	40,48570338	2,609498793	-0,743158207	False	0,743158207
ENSMUSG00000034263	Ints14	180,8442705	140,0813635	122,4026384	87,71902399	302,70186	0,743149649	False	0,743149649
ENSMUSG00000020132	Rab21	380,2366713	182,9891686	227,1933864	158,5690049	114,8179469	-0,742976839	False	0,742976839
ENSMUSG00000003549	Ercc1	61,82710102	138,8193693	110,0743151	103,4634642	161,7889252	0,742810136	False	0,742810136
ENSMUSG00000006386	Tek	123,654202	84,55361583	131,2085836	206,9269284	53,49472526	0,742810136	False	0,742810136
ENSMUSG00000027823	Gmps	579,6290721	603,2332592	363,6855371	346,3776845	1008,571284	-0,74278397	False	0,74278397
ENSMUSG00000040174	Alkbh3	153,7949138	427,8160562	257,1336001	284,5245265	566,2612381	0,74151035	False	0,74151035
ENSMUSG00000003477	Inmt	10200,69883	1166,082702	6103,400624	215,9237514	2047,151803	-0,740982795	False	0,740982795
ENSMUSG00000015243	Abca1	1676,287277	8206,748713	1002,997159	8073,524015	12451,22349	-0,740951895	False	0,740951895
ENSMUSG00000032294	Pkm	150,7035587	431,602039	251,850033	600,5379334	422,7388045	0,740851434	False	0,740851434
ENSMUSG00000054051	Ercc6	165,3874952	276,3767443	381,2974275	475,7070147	344,4538407	0,740786067	False	0,740786067
ENSMUSG00000038646	Fam103a1	217,1676923	156,487289	133,8503672	66,35156943	362,7203322	0,740048126	False	0,740048126
ENSMUSG00000038506	Dcun1d2	188,5726581	84,55361583	233,357548	314,8888041	37,8377325	0,739721954	False	0,739721954
ENSMUSG00000037062	Sh3glb1	962,9570985	1330,141957	576,7894112	966,0338667	1865,791637	-0,739426845	False	0,739426845
ENSMUSG00000064254	Ethe1	961,4114209	25,23988532	575,9088166	59,6039522	28,70448672	-0,739313533	False	0,739313533
ENSMUSG00000025862	Stag2	647,6388832	371,0263142	296,7603535	387,9879907	345,7585901	-0,739177608	False	0,739177608
ENSMUSG00000008035	Mid1ip1	1171,623564	397,5281938	630,5056769	65,22696655	701,9551753	-0,739058302	False	0,739058302
ENSMUSG00000021598	Med10	170,7973666	391,2182225	218,3874412	102,3388613	656,2889464	-0,738931645	False	0,738931645
ENSMUSG00000020224	Llph	105,1060717	128,7234151	130,3279891	62,97776081	195,7124095	-0,738931645	False	0,738931645
ENSMUSG00000039886	Tmem120a	447,4736437	2537,870469	746,7441537	3991,215591	3556,746855	0,738811328	False	0,738811328
ENSMUSG00000018171	Vmp1	1292,95295	9824,625362	2157,456576	6476,587938	4194,76931	0,738654715	False	0,738654715
ENSMUSG00000022109	Med4	119,0171695	143,8673463	71,32815619	84,34521537	236,1596408	-0,738626126	False	0,738626126
ENSMUSG00000043323	Fbrsl1	346,2317657	955,3296595	577,6700057	1006,51957	1450,881329	0,738507494	False	0,738507494
ENSMUSG00000036644	Tbc1d9b	947,5003232	263,7568016	479,0434193	567,9244502	297,4828624	-0,738427412	False	0,738427412
ENSMUSG00000031523	Dlc1	799,1152807	166,5832431	479,0434193	250,7864404	331,4063467	-0,738247218	False	0,738247218
ENSMUSG00000040952	Rps19	957,5472271	2845,79707	3052,140609	1596,936078	2026,275813	0,737891017	False	0,737891017
ENSMUSG00000020027	Socs2	1440,571454	4650,448871	863,863225	3326,575294	5889,638776	-0,737766407	False	0,737766407
ENSMUSG00000026208	Des	95,05916782	63,09971331	158,5070138	93,34203834	48,27572767	0,737649002	False	0,737649002
ENSMUSG00000026626	Ppp2r5a	2656,246828	882,133992	1592,995488	1554,201169	619,7559633	-0,737647032	False	0,737647032
ENSMUSG00000032261	Sh3bgrl2	439,745256	263,7568016	207,8203069	259,7832633	220,502648	-0,737459716	False	0,737459716
ENSMUSG00000028936	Rpl22	1571,181205	2134,032304	2116,949228	942,4172064	1838,3919	-0,737411792	False	0,737411792
ENSMUSG00000031754	Nudt21	189,3454969	191,8231285	113,5966932	142,8245647	319,6636021	-0,737100272	False	0,737100272
ENSMUSG00000029229	Chic2	159,9776239	728,1706916	95,98480277	316,0134069	1509,595052	-0,736992221	False	0,736992221
ENSMUSG00000029167	Ppargc1a	163,068979	66,88569611	90,70123565	97,84044983	26,09498793	-0,736979418	False	0,736979418
ENSMUSG00000031594	Fgl1	4930,711307	12173,19669	24019,09615	8217,473183	11938,45698	0,736899058	False	0,736899058
ENSMUSG00000037071	Scd1	116153,8109	30658,8887	7513,232452	6021,123775	69737,55049	-0,73602878	False	0,73602878
ENSMUSG00000027412	Lpin3	60,2814235	304,1406181	100,3877754	725,3688522	319,6636021	0,735798207	False	0,735798207
ENSMUSG00000031917	Nip7	80,37523133	253,6608475	133,8503672	139,4507561	485,3667755	0,735798207	False	0,735798207
ENSMUSG00000029433	Diablo	30,14071175	137,557375	50,19388769	156,3197992	335,3205949	0,735798207	False	0,735798207
ENSMUSG00000021144	Mta1	538,6686177	1000,761453	773,1619893	838,9537422	323,5778503	-0,735285162	False	0,735285162
ENSMUSG00000040356	Skiv2l	889,537416	2189,560052	1526,950899	1479,977379	2726,926239	0,73444793	False	0,73444793
ENSMUSG00000045934	Mtmr11	102,0147167	156,487289	416,5212084	418,3522682	61,32322164	-0,734271894	False	0,734271894
ENSMUSG00000022556	Hsf1	312,9996989	745,8386113	622,5803262	520,6911296	942,0290643	0,73426656	False	0,73426656
ENSMUSG00000031897	Psmb10	364,779896	277,6387386	619,0579482	219,29756	473,6240309	-0,734136461	False	0,734136461
ENSMUSG00000031600	Vps37a	713,3301781	249,8748647	428,8495317	394,7356079	221,8073974	-0,734098464	False	0,734098464
ENSMUSG00000024357	Sil1	741,1523735	348,3104175	445,5808276	276,6523064	334,0158455	-0,734083019	False	0,734083019
ENSMUSG00000068922	Msto1	112,8344594	219,5870023	138,2533398	98,9650527	67,84696862	-0,733851468	False	0,733851468
ENSMUSG00000047921	Trappc9	200,1652396	143,8673463	178,7606877	332,88245	78,28496379	0,733821352	False	0,733821352
ENSMUSG00000001105	Ift20	639,1376568	2102,482447	1064,638776	1062,749714	1234,292929	0,733603274	False	0,733603274
ENSMUSG00000014599	Csf1	205,5751109	123,6754381	756,4306934	255,2848519	173,5316697	-0,733106597	False	0,733106597
ENSMUSG00000050552	Lamtor4	321,5009253	326,8565149	617,2967591	193,4316939	227,026395	-0,732998689	False	0,732998689
ENSMUSG00000005483	Dnajb1	294,4515686	210,7530424	287,9544083	83,2206125	489,2810237	0,732632971	False	0,732632971
ENSMUSG00000000555	Itga5	533,2587463	885,9199748	1234,593518	1029,011628	958,9908064	0,732340663	False	0,732340663
ENSMUSG00000016831	Tox4	433,5625459	271,3287672	167,312959	160,8182106	720,2216669	0,732200869	False	0,732200869
ENSMUSG00000021102	Glrx5	676,2339175	644,87907	626,1027043	407,1062395	1578,74677	-0,732117045	False	0,732117045
ENSMUSG00000037894	H2afz	420,424287	172,8932145	134,7309617	192,307091	253,1213829	-0,732016626	False	0,732016626
ENSMUSG00000020385	Clk4	593,5401698	985,6175219	1048,788074	2448,260452	1422,176842	0,731682231	False	0,731682231
ENSMUSG00000039159	Ube2h	1167,759371	469,461867	458,7897454	446,46734	703,2599247	-0,731613109	False	0,731613109
ENSMUSG00000044876	Zfp444	306,0441501	123,6754381	508,1030385	374,4927562	32,61873491	0,7313813	False	0,7313813
ENSMUSG00000075040	Zfp408	109,7431043	259,9708188	231,596359	182,1856652	302,70186	0,731279158	False	0,731279158
ENSMUSG00000005804	Bloc1s6	164,6146565	93,38757569	80,1341014	44,98411486	99,16095413	-0,731248734	False	0,731248734
ENSMUSG00000038267	Slc22a23	1003,917553	309,1885952	604,9684358	473,457809	127,8654409	-0,730709015	False	0,730709015
ENSMUSG00000027257	Pacsin3	578,0833946	576,7313796	564,4610879	1086,366374	348,3680889	-0,730665155	False	0,730665155
ENSMUSG00000026273	Mterf4	215,6220148	129,9854094	118,8802603	68,60077517	118,7321951	-0,730154791	False	0,730154791
ENSMUSG00000027109	Sp3	574,9920395	434,1260276	250,0888439	236,166603	953,7718088	0,730102158	False	0,730102158
ENSMUSG00000036450	Hif1an	264,3108569	257,4468303	132,9697726	130,4539331	438,3957972	0,729998281	False	0,729998281
ENSMUSG00000046351	Zfp322a	172,3430441	54,26575344	103,9101535	77,59759814	88,72295896	-0,729946439	False	0,729946439
ENSMUSG00000028405	Aco1	3022,572401	641,0930872	1822,830658	691,630766	691,5171802	-0,729596357	False	0,729596357
ENSMUSG00000025085	Ablim1	592,7673311	687,786875	643,7145947	802,9664503	357,5013346	-0,729517311	False	0,729517311
ENSMUSG00000007891	Ctsd	2468,447008	3264,779166	2051,785234	1488,974202	3916,857688	-0,729284916	False	0,729284916
ENSMUSG00000026864	Hspa5	14142,94936	21393,3268	8531,199718	26819,52928	131867,1073	-0,729262467	False	0,729262467
ENSMUSG00000040253	Gbp7	420,424287	176,6791973	253,611222	167,5658279	195,7124095	-0,729227428	False	0,729227428
ENSMUSG00000043090	Zfp866	144,5208486	59,31373051	87,17885756	41,61030625	54,79947465	-0,729227428	False	0,729227428
ENSMUSG00000035623	Rsf1	170,7973666	175,417203	103,0295589	166,441225	258,3403805	-0,729227428	False	0,729227428
ENSMUSG00000044950	Pwwp2a	127,5183959	259,9708188	281,7902467	335,1316557	211,3694022	0,729061162	False	0,729061162
ENSMUSG00000029165	Agbl5	144,5208486	113,579484	160,2682028	239,5404117	31,31398552	0,728991435	False	0,728991435
ENSMUSG00000035762	Tmem161b	91,96781277	692,8348521	152,3428521	378,9911677	527,1187562	0,728120876	False	0,728120876
ENSMUSG00000084786	Ubl5	653,8215933	962,9016251	1139,48931	394,7356079	975,9525486	-0,728010358	False	0,728010358
ENSMUSG00000037791	Phf12	472,2044841	1556,03893	781,9679345	1572,194815	1536,994789	0,727697708	False	0,727697708
ENSMUSG00000024844	Banf1	346,2317657	604,4952535	811,0275537	209,1761341	1298,22565	-0,727019837	False	0,727019837
ENSMUSG00000022681	Ntan1	268,1750507	90,86358716	162,0293918	106,8372728	97,85620474	-0,726919485	False	0,726919485
ENSMUSG00000047797	Gjb1	13798,26327	3125,959797	8338,349518	3710,064873	1565,699276	-0,726652941	False	0,726652941
ENSMUSG00000021509	Slc25a48	164,6146565	36,59783372	99,50718086	59,6039522	14,35224336	-0,726220246	False	0,726220246
ENSMUSG00000026035	Ppil3	119,7900082	99,69754702	198,1337672	91,0928326	76,98021439	0,725967197	False	0,725967197
ENSMUSG00000021097	Clmn	1399,610999	237,254922	846,2513345	267,6554834	219,1978986	-0,725867799	False	0,725867799
ENSMUSG00000032475	Nck1	217,9405311	95,91156423	101,2683699	69,72537804	131,779689	-0,725806496	False	0,725806496
ENSMUSG00000001134	Uxt	226,4417575	561,5874484	441,1778549	374,4927562	417,5198069	0,725797787	False	0,725797787
ENSMUSG00000005034	Prkacb	726,468437	371,0263142	439,4166659	235,0420002	319,6636021	-0,72531053	False	0,72531053
ENSMUSG00000047454	Gphn	988,4607776	98,43555276	597,9236797	543,183187	106,9894505	-0,725222373	False	0,725222373
ENSMUSG00000035049	Rrp12	54,87155216	309,1885952	90,70123565	246,2880289	350,9775877	0,725063819	False	0,725063819
ENSMUSG00000020707	Rnf135	147,6122037	94,64956996	243,9246823	148,4475791	14,35224336	0,724623749	False	0,724623749
ENSMUSG00000026356	Dars	990,7792939	373,5503028	599,6848687	326,1348328	357,5013346	-0,724359147	False	0,724359147
ENSMUSG00000093930	Hmgcs1	8049,888553	2049,478688	13298,73845	2571,966767	721,5264163	0,72424868	False	0,72424868
ENSMUSG00000031996	Aplp2	4381,995785	1506,821154	2374,082828	1679,032087	2652,555523	-0,724205147	False	0,724205147
ENSMUSG00000039834	Zfp335	284,4046647	633,5211216	474,6404467	1122,353666	469,7097827	0,723824513	False	0,723824513
ENSMUSG00000055799	Tcf7l1	141,4294936	288,9966869	242,1634932	353,1253017	233,550142	0,723649312	False	0,723649312
ENSMUSG00000018572	Phf23	187,7998194	511,1076778	309,9692713	570,1736559	472,3192815	0,722929526	False	0,722929526
ENSMUSG00000024678	Ms4a4d	97,37768411	133,7713922	58,9998329	151,8213877	79,58971319	-0,722880322	False	0,722880322
ENSMUSG00000028106	Rprd2	292,1330523	321,8085379	176,9994987	229,4189858	477,5382791	-0,722880322	False	0,722880322
ENSMUSG00000022914	Brwd1	268,9478895	677,6909209	443,8196385	1081,867962	876,7915945	0,722646839	False	0,722646839
ENSMUSG00000062115	Rai1	186,2541418	644,87907	307,3274878	557,8030243	374,4630768	0,722504301	False	0,722504301
ENSMUSG00000021993	Mipep	495,389647	238,5169163	256,2530056	300,2689667	152,6556794	-0,722308341	False	0,722308341
ENSMUSG00000031770	Herpud1	2858,730584	4307,18643	1733,010017	12268,29273	27686,78219	-0,722094668	False	0,722094668
ENSMUSG00000027540	Ptpn1	398,7848016	518,6796434	398,9093179	241,7896174	745,0119054	-0,721858131	False	0,721858131
ENSMUSG00000022678	Nde1	183,1627868	111,0554954	191,089011	345,2530816	369,2440792	-0,721845636	False	0,721845636
ENSMUSG00000020283	Pex13	938,226258	427,8160562	389,2227782	232,7927944	568,8707369	-0,721835008	False	0,721835008
ENSMUSG00000000915	Hip1r	300,6342787	1101,720994	495,7747152	1078,494154	1711,831208	0,721675178	False	0,721675178
ENSMUSG00000020029	Nudt4	2338,610096	3706,47716	1915,293083	1418,124221	5221,607085	-0,721667438	False	0,721667438
ENSMUSG00000009535	Rnmt	130,6097509	146,3913349	55,47745481	97,84044983	215,2836504	0,720976151	False	0,720976151
ENSMUSG00000032702	Kank1	843,1670902	162,7972603	481,6852029	511,6943066	200,9314071	-0,720536377	False	0,720536377

ENSMUSG00000021395	Spin1	591,9944923	1020,953361	359,2825645	392,4864022	868,9630981	-0,720464829	False	0,720464829
ENSMUSG00000070407	Hs3st3b1	519,3476486	170,3692259	315,2528385	134,9523446	197,0171589	-0,720191234	False	0,720191234
ENSMUSG00000030199	Etv6	251,9454367	348,3104175	230,7157645	152,9459905	472,3192815	-0,720089039	False	0,720089039
ENSMUSG00000006998	Psmd2	1653,874952	1877,847468	1422,160151	1004,270364	2484,242851	-0,719702442	False	0,719702442
ENSMUSG00000030079	Ruvbl1	195,528207	111,0554954	113,5966932	65,22696655	118,7321951	-0,719665561	False	0,719665561
ENSMUSG00000020018	Snrpf	98,15052288	229,6829564	206,9397124	59,6039522	377,0725756	-0,719587958	False	0,719587958
ENSMUSG00000024039	Cbs	7670,424721	2097,43447	4658,345015	3245,603887	1959,733594	-0,719488969	False	0,719488969
ENSMUSG00000000159	Igsf5	161,5233014	641,0930872	265,9395453	762,480747	452,7480406	0,719356019	False	0,719356019
ENSMUSG00000029070	Mxra8	105,8789105	56,78974198	99,50718086	174,3134451	61,32322164	0,719268597	False	0,719268597
ENSMUSG00000018476	Kdm6b	300,6342787	806,4143361	586,4759509	494,8252635	737,183409	0,718909643	False	0,718909643
ENSMUSG00000005054	Cstb	277,4491158	254,9228418	213,103874	121,4571101	456,6622888	0,718904451	False	0,718904451
ENSMUSG00000028086	Fbxw7	76,51103752	193,0851227	125,9250165	263,157072	237,4643902	0,718825128	False	0,718825128
ENSMUSG00000027739	Rab33b	90,42213525	275,11475	148,820474	257,5340576	438,3957972	0,718825128	False	0,718825128
ENSMUSG00000031400	G6pdx	220,2590474	113,579484	113,5966932	133,8277417	93,94195655	-0,718824071	False	0,718824071
ENSMUSG00000028879	Stx12	231,8516288	383,6462569	140,8951233	169,8150336	562,3469899	-0,718580184	False	0,718580184
ENSMUSG00000045107	Saysd1	154,5677526	194,347117	306,4468933	320,5118184	93,94195655	-0,718397815	False	0,718397815
ENSMUSG00000028164	Manba	154,5677526	142,6053521	135,6115562	269,9046892	93,94195655	-0,718397815	False	0,718397815
ENSMUSG00000022265	Ank	485,342743	235,9929278	294,9991645	94,46664122	126,5606915	-0,718293052	False	0,718293052
ENSMUSG00000017485	Top2b	673,9154012	330,6424977	241,2828987	337,3808615	409,6913105	-0,718030203	False	0,718030203
ENSMUSG00000035649	Zcchc7	425,0613195	966,6876079	699,1920496	1542,95514	1094,684744	0,7180178	False	0,7180178
ENSMUSG00000023938	Aars2	240,3528552	177,9411915	329,3423508	392,4864022	146,1319324	-0,717882475	False	0,717882475
ENSMUSG00000020402	Vdac1	1860,995741	695,3588406	1131,563959	302,5181725	872,8773463	-0,717756621	False	0,717756621
ENSMUSG00000020097	Sgpl1	1319,235768	636,0451101	802,2216085	742,2378953	798,5066307	-0,717629688	False	0,717629688
ENSMUSG00000062901	Klhl24	617,4981715	219,5870023	88,94004661	237,2912059	375,7678262	-0,716593318	False	0,716593318
ENSMUSG00000022174	Dad1	1234,223504	2113,840396	2568,694217	751,2347182	1799,249418	-0,716268031	False	0,716268031
ENSMUSG00000041781	Cpsf2	215,6220148	131,2474037	99,50718086	124,8309187	62,62797103	-0,7162156	False	0,7162156
ENSMUSG00000057897	Camk2b	112,8344594	157,7492833	68,68637263	190,0578853	318,3588527	-0,716111929	False	0,716111929
ENSMUSG00000026563	Tada1	173,8887216	107,2695126	140,0145288	285,6491294	139,6081854	0,71607977	False	0,71607977
ENSMUSG00000023830	Igf2r	360,1428635	64,36170757	219,2680357	148,4475791	87,41820957	-0,715873801	False	0,715873801
ENSMUSG00000093904	Tomm20	427,3798358	379,8602741	243,0440878	187,8086796	701,9551753	0,715860066	False	0,715860066
ENSMUSG00000027357	Crls1	1022,465683	369,76432	622,5803262	281,1507179	561,0422405	-0,71572053	False	0,71572053
ENSMUSG00000004455	Ppp1cc	397,2391241	466,9378785	241,2828987	172,0642394	652,3746983	0,715693109	False	0,715693109
ENSMUSG00000038298	Pdzk1	1098,976721	63,09971331	669,2518359	150,6967848	70,45646741	-0,71553973	False	0,71553973
ENSMUSG00000028763	Hspg2	291,3602136	194,347117	358,40197	490,326852	177,4459179	-0,715424499	False	0,715424499
ENSMUSG00000030800	Prss8	124,4270408	16,40592546	204,2979288	103,4634642	16,96174215	0,715374528	False	0,715374528
ENSMUSG00000028542	Slc6a9	499,2538408	304,1406181	564,4610879	2208,72004	392,7295683	-0,715035024	False	0,715035024
ENSMUSG00000090115	Usp49	64,91845608	112,3174897	106,551937	112,4602872	182,6649155	0,714856226	False	0,714856226
ENSMUSG00000057143	Trim12c	190,1183356	98,43555276	90,70123565	115,8340958	62,62797103	-0,714837704	False	0,714837704
ENSMUSG00000042348	Arl15	111,2887818	162,7972603	67,80577811	92,21743547	541,4709996	-0,714828049	False	0,714828049
ENSMUSG00000026701	Prdx6	6431,564184	1903,087353	3919,526212	1053,752891	3269,701988	-0,714490376	False	0,714490376
ENSMUSG00000059586	Nsmce2	90,42213525	306,6646067	103,0295589	55,10554071	348,3680889	-0,714478601	False	0,714478601
ENSMUSG00000052298	Cdc42se2	132,9282672	136,2953807	81,01469592	84,34521537	287,0448672	-0,714392387	False	0,714392387
ENSMUSG00000022105	Rb1	132,9282672	68,14769037	81,01469592	67,4761723	58,71372284	-0,714392387	False	0,714392387
ENSMUSG00000022515	Anks3	173,8887216	473,2478498	285,3126248	644,3974454	306,6161082	0,714379223	False	0,714379223
ENSMUSG00000056999	Ide	1156,939628	570,4214083	670,1324304	705,1260005	656,2889464	-0,714360599	False	0,714360599
ENSMUSG00000038902	Pogz	217,9405311	426,554062	365,4467262	513,9435123	357,5013346	0,714014104	False	0,714014104
ENSMUSG00000022668	Gtpbp8	217,9405311	107,2695126	132,9697726	84,34521537	56,10422405	-0,712836202	False	0,712836202
ENSMUSG00000024182	Axin1	238,0343389	206,9670596	118,8802603	113,58489	390,1200696	0,712748507	False	0,712748507
ENSMUSG00000025939	Ube2w	192,4368519	80,76763303	117,1190713	59,6039522	117,4274457	-0,712615463	False	0,712615463
ENSMUSG00000026896	Ifih1	598,9500412	271,3287672	324,0587837	365,4959333	168,3126721	-0,712580318	False	0,712580318
ENSMUSG00000028772	Zcchc17	228,7602738	228,4209622	346,0736467	285,6491294	139,6081854	-0,712453006	False	0,712453006
ENSMUSG00000024354	Slc23a1	1672,423083	1684,762345	2740,410149	1803,863006	281,8258696	0,712451973	False	0,712451973
ENSMUSG00000031596	Slc7a2	2940,651492	1725,146162	922,8630579	1879,211398	4818,439521	0,712430182	False	0,712430182
ENSMUSG00000000976	Heatr6	231,8516288	379,8602741	477,2822303	624,1545937	397,9485659	0,71226698	False	0,71226698
ENSMUSG00000028419	Chmp5	574,9920395	215,8010195	205,1785234	147,3229762	350,9775877	-0,712163075	False	0,712163075
ENSMUSG00000038538	Ubn2	316,091054	790,0084106	517,7895783	1563,197992	722,8311657	0,712025723	False	0,712025723
ENSMUSG00000024474	Ik	1090,475494	986,8795161	665,7294578	1973,67804	1851,439394	-0,711949437	False	0,711949437
ENSMUSG00000029670	Ing3	129,0640734	103,4835298	81,89529044	56,23014358	211,3694022	0,711679084	False	0,711679084
ENSMUSG00000052305	Hbb-bs	1530,22075	200,6570883	625,2221098	934,5449863	268,7783757	-0,711403774	False	0,711403774
ENSMUSG00000026888	Grb14	686,2808214	143,8673463	419,1629919	139,4507561	190,4934119	-0,711287692	False	0,711287692
ENSMUSG00000000916	Nsun5	129,8369222	318,0225551	239,5217097	212,5499427	315,749354	0,711101276	False	0,711101276
ENSMUSG00000022994	Adcy6	198,619562	396,2661996	396,2675344	1033,510039	121,3416939	-0,710932365	False	0,710932365
ENSMUSG00000019731	Slc35e1	559,5352643	222,1109908	264,1783563	282,2753208	341,8443419	-0,710889512	False	0,710889512
ENSMUSG00000027198	Ext2	404,9675117	176,6791973	247,4470604	113,58489	204,8456553	-0,71068627	False	0,71068627
ENSMUSG00000060038	Dhps	102,7875555	194,347117	168,1935535	182,1856652	272,6926239	0,710456804	False	0,710456804
ENSMUSG00000092260	Zfp963	30,14071175	138,8193693	49,31329317	280,026115	117,4274457	0,710263115	False	0,710263115
ENSMUSG00000002372	Ranbp3	379,4638325	925,0417971	620,8191372	1117,855254	1063,370758	0,710210644	False	0,710210644
ENSMUSG00000004748	Mtfp1	465,2489352	23,97789106	284,4320302	34,86268902	13,04749397	-0,709918911	False	0,709918911
ENSMUSG00000074884	Serf2	983,0509063	815,2482959	1607,965595	431,8475027	858,5251029	0,709898506	False	0,709898506
ENSMUSG00000063659	Zbtb18	232,6244676	102,2215356	380,416833	139,4507561	79,58971319	0,709578237	False	0,709578237
ENSMUSG00000043207	Zmpste24	755,83631	1243,064352	670,1324304	462,2117802	2104,560777	-0,709519797	False	0,709519797
ENSMUSG00000026914	Psmd14	547,1698441	841,7501755	334,6259179	372,2435505	1380,424862	-0,70943953	False	0,70943953
ENSMUSG00000022100	Xpo7	838,5300576	458,1039186	393,6257508	512,8189095	447,529043	-0,709413039	False	0,709413039
ENSMUSG00000031386	Hcfc1	605,90559	601,9712649	370,7302933	535,3109669	1381,729611	-0,70872301	False	0,70872301
ENSMUSG00000025580	Eif4a3	452,1106762	455,5799301	384,8198056	276,6523064	854,6108547	-0,708602035	False	0,708602035
ENSMUSG00000051695	Pcbp1	2469,992686	1884,157439	2189,157979	949,1648236	4034,285134	0,707806284	False	0,707806284
ENSMUSG00000025393	Atp5b	13705,52262	7473,530044	8392,065784	5170,924004	7259,625642	-0,70765945	False	0,70765945
ENSMUSG00000095440	Fignl2	149,1578812	186,7751514	220,1486302	224,9205743	91,33245776	-0,707640647	False	0,707640647
ENSMUSG00000003233	Dvl3	242,6713715	396,2661996	440,2972604	467,8347946	487,9762743	0,707465994	False	0,707465994
ENSMUSG00000055137	Sugct	315,3182152	141,3433578	130,3279891	25,86586605	193,1029107	-0,707438603	False	0,707438603
ENSMUSG00000036026	Tmem63b	1325,418478	499,7497294	811,9081482	646,6466512	243,9881371	-0,707059509	False	0,707059509
ENSMUSG00000040464	Gtpbp10	162,2961402	133,7713922	116,2384768	41,61030625	264,8641275	0,706623773	False	0,706623773
ENSMUSG00000030541	Idh2	1705,65515	253,6608475	1045,265696	556,6784215	397,9485659	-0,706456283	False	0,706456283
ENSMUSG00000033706	Smyd5	218,7133699	458,1039186	356,6407809	542,0585841	537,5567514	0,705430266	False	0,705430266
ENSMUSG00000024507	Hsd17b4	4215,835451	1672,142403	2585,425513	1960,182805	2146,312757	-0,705416817	False	0,705416817
ENSMUSG00000073421	H2-Ab1	293,6787299	194,347117	301,1633261	163,0674164	478,8430285	0,705314018	False	0,705314018
ENSMUSG00000026473	Glul	9985,849654	3923,540173	9555,331146	1301,165522	16279,35822	0,705086735	False	0,705086735
ENSMUSG00000024360	Etf1	996,1891653	1530,799045	611,1325975	988,5259242	2326,368174	-0,704934284	False	0,704934284
ENSMUSG00000021922	Itih4	34281,58184	125867,5221	55871,96116	77929,35599	92270,57257	0,704690781	False	0,704690781
ENSMUSG00000046062	Ppp1r15b	1020,920006	972,9975792	916,6988962	381,2403735	1663,555481	0,704400154	False	0,704400154
ENSMUSG00000030545	Pex11a	728,7869533	716,8127432	447,3420166	1178,583809	895,058086	-0,704118863	False	0,704118863
ENSMUSG00000040209	Zfp704	199,3924008	121,1514495	122,4026384	118,0833015	82,19921198	-0,703975771	False	0,703975771
ENSMUSG00000019826	Zbtb24	153,022075	209,4910482	154,1040411	211,4253399	93,94195655	-0,703898245	False	0,703898245
ENSMUSG00000017404	Rpl19	1115,206335	2049,478688	1959,322809	684,8831488	2128,046266	-0,703380892	False	0,703380892
ENSMUSG00000005417	Mprip	1540,267654	3242,06327	3823,54141	10545,40113	2507,72834	0,703199999	False	0,703199999
ENSMUSG00000028601	Echdc2	1671,650244	1833,677669	4481,345517	6247,168952	1026,837775	-0,703064751	False	0,703064751
ENSMUSG00000009633	G0s2	883,3547059	542,6575344	1954,919836	1116,730652	855,9156041	-0,702950845	False	0,702950845
ENSMUSG00000000776	Polr3d	91,96781227	268,8047787	149,7010685	170,9396365	391,424819	0,702883584	False	0,702883584
ENSMUSG00000055044	Pdlim1	619,043849	400,0521824	251,850033	200,1793111	1007,266534	0,702331978	False	0,702331978
ENSMUSG00000031681	Smad1	201,7109171	328,1185092	108,3131261	131,578536	169,6174215	0,70192781	False	0,70192781
ENSMUSG00000022814	Umps	557,216748	108,5315069	342,5512686	67,4761723	95,24670595	-0,701918693	False	0,701918693
ENSMUSG00000036833	Pnpla7	1234,996343	1703,692259	1682,816129	1276,424259	759,3641488	-0,701642977	False	0,701642977
ENSMUSG00000003585	Sec14l2	8335,838896	1477,795286	5125,940706	1397,881369	1210,80744	-0,701510605	False	0,701510605
ENSMUSG00000024712	Rfk	671,5968849	546,4435172	453,5061782	249,6618375	1092,075245	0,701404819	False	0,701404819
ENSMUSG00000021819	Zswim8	831,5745088	4028,285698	1351,71259	4215,011563	6198,864383	0,700870989	False	0,700870989
ENSMUSG00000009647	Mcu	396,4662853	204,4430711	243,9246823	125,9555216	112,2084481	-0,700762438	False	0,700762438
ENSMUSG00000006024	Napa	1341,648092	3585,32571	2180,352034	3270,345151	4985,447444	0,700554778	False	0,700554778
ENSMUSG00000071180	Smim15	296,7700849	133,7713922	130,3279891	64,10236368	182,6649155	-0,700146111	False	0,700146111
ENSMUSG00000074896	Ifit3	307,5898276	89,6015929	189,327822	59,6039522	177,4459179	-0,70012136	False	0,70012136
ENSMUSG00000032606	Nicn1	336,1848618	30,28786239	206,9397124	167,5658279	14,35224336	-0,700044233	False	0,700044233
ENSMUSG00000014496	Ankrd28	442,8366111	272,5907615	227,1933864	268,7800863	203,5409059	-0,700037833	False	0,700037833
ENSMUSG00000059486	Kbtbd2	140,6566548	116,1034725	131,2085836	86,59442111	260,9498793	-0,699831825	False	0,699831825
ENSMUSG00000025066	Sfr1	672,3697236	581,7793567	284,4320302	232,7927944	1092,075245	0,699745595	False	0,699745595

ENSMUSG00000024319	Vps52	418,1057707	1066,385155	678,9383756	1238,187762	1165,141211	0,699412677	False	0,699412677
ENSMUSG00000044768	D1Ertd622e	114,3801369	190,5611342	70,44756167	85,46981824	181,3601661	-0,699214859	False	0,699214859
ENSMUSG00000035472	Slc25a21	171,5702053	54,26575344	105,6713425	77,59759814	35,22823371	-0,699214859	False	0,699214859
ENSMUSG00000032526	Ss18l2	282,0861484	214,5390252	457,9091508	191,1824882	213,978901	0,698925571	False	0,698925571
ENSMUSG00000030082	Sec61a1	2327,017515	4143,127176	3579,616727	1433,868661	5799,611067	-0,698569187	False	0,698569187
ENSMUSG00000028238	Atp6v0d2	229,5331126	27,76387386	372,4914823	7,872220101	288,3496166	0,698505144	False	0,698505144
ENSMUSG00000002107	Celf2	127,5183959	538,8715516	206,9397124	629,7776081	383,5963226	0,698505144	False	0,698505144
ENSMUSG00000058291	Zfp68	217,1676923	194,347117	152,3428521	133,8277417	416,2150575	-0,698432283	False	0,698432283
ENSMUSG00000030527	Crtc3	198,619562	552,7534886	322,2975946	569,049053	628,8892091	0,6983857	False	0,6983857
ENSMUSG00000054484	Tmem62	167,7060115	540,1335459	272,1037069	645,5220483	403,1675635	0,698222207	False	0,698222207
ENSMUSG00000027466	Rbck1	581,9475884	681,4769037	910,5347346	1063,874317	358,806084	-0,697684873	False	0,697684873
ENSMUSG00000004319	Clcn3	458,2933863	162,7972603	282,6708412	241,7896174	260,9498793	-0,697148396	False	0,697148396
ENSMUSG00000063052	Lrrc40	160,7504627	83,29162157	73,96993975	62,97776081	99,16095413	-0,696978832	False	0,696978832
ENSMUSG00000035933	Cog5	345,458927	78,2436445	213,103874	134,9523446	46,97097827	-0,696957373	False	0,696957373
ENSMUSG00000041570	Camsap2	158,4319464	180,4651801	97,74599182	125,9555216	240,073889	-0,696753822	False	0,696753822
ENSMUSG00000031788	Kifc3	278,2219546	181,7271743	450,8643947	338,5054644	53,49472526	0,696457315	False	0,696457315
ENSMUSG00000039367	Sec24c	1363,287578	1259,470278	769,6396112	602,7871392	2208,940728	0,696264786	False	0,696264786
ENSMUSG00000028273	Pdlim5	703,2832742	172,8932145	434,1330988	272,1538949	140,9129348	-0,695968487	False	0,695968487
ENSMUSG00000067995	Gtf2f2	102,0147167	127,4614209	89,82064113	62,97776081	294,8733636	-0,695862923	False	0,695862923
ENSMUSG00000038692	Hoxb4	37,86909938	106,0075184	128,5668	410,4800481	61,32322164	0,695412381	False	0,695412381
ENSMUSG00000031153	Gripap1	348,550282	742,0526285	525,714929	585,9180961	215,2836504	-0,695128036	False	0,695128036
ENSMUSG00000060002	Chpt1	1459,892423	776,1264737	901,7287894	811,9632733	677,1649368	-0,695096575	False	0,695096575
ENSMUSG00000026365	Cfh	12912,59005	10918,77439	14099,19887	19583,83441	7975,933061	-0,695053203	False	0,695053203
ENSMUSG00000029033	Acap3	56,41722968	210,7530424	164,6711754	411,604651	91,33245776	0,694991831	False	0,694991831
ENSMUSG00000038781	Stap2	669,2783686	1898,039376	1658,159483	1466,482145	413,6055587	-0,694350811	False	0,694350811
ENSMUSG00000033009	Ogfod1	182,389948	53,00375918	112,7160987	87,71902399	69,15171802	-0,694332638	False	0,694332638
ENSMUSG00000041303	Gtf3c3	173,1158829	55,52774771	88,05945209	70,84998091	106,9894505	-0,694269544	False	0,694269544
ENSMUSG00000039047	Pigk	418,8786094	204,4430711	258,8947891	120,3325073	225,7216456	-0,694166285	False	0,694166285
ENSMUSG00000003435	Supt5	889,537416	2127,722333	1438,891447	4492,788472	3559,356354	0,693830561	False	0,693830561
ENSMUSG00000027358	Bmp2	244,9898878	83,29162157	151,4622576	138,3261532	67,84696862	-0,693763864	False	0,693763864
ENSMUSG00000063268	Parp10	239,5800165	427,8160562	471,9986632	506,0712922	387,5105708	0,693728089	False	0,693728089
ENSMUSG00000027303	Ptpra	517,0291323	307,9266009	207,8203069	274,4031007	319,6636021	-0,693691088	False	0,693691088
ENSMUSG00000040483	Xaf1	146,8393649	103,4835298	132,0891781	42,73490912	237,4643902	0,693472405	False	0,693472405
ENSMUSG00000021712	Trim23	128,2912346	74,4576617	63,4028055	74,22378953	207,455154	0,693376897	False	0,693376897
ENSMUSG00000067847	Romo1	314,5453765	615,8532019	759,072477	194,5562968	613,2322164	-0,693080482	False	0,693080482
ENSMUSG00000024451	Arap3	236,4886614	575,4693854	516,0283892	1785,86936	382,2915732	0,692902385	False	0,692902385
ENSMUSG00000041417	Pik3r1	490,7526144	1442,459446	366,3273207	303,6427753	1294,311401	-0,69262091	False	0,69262091
ENSMUSG00000066829	Zfp810	75,73819875	227,1589679	122,4026384	317,1380098	223,1121468	0,69254164	False	0,69254164
ENSMUSG00000042444	Mindy2	434,3353847	156,487289	137,3727453	119,2079044	268,7783757	-0,69239242	False	0,69239242
ENSMUSG00000040599	Mis12	179,298593	104,7455241	110,9549096	60,72855507	86,11346017	-0,69239066	False	0,69239066
ENSMUSG00000061518	Cox5b	1997,015363	2942,970629	4083,316793	1235,938556	2690,393256	-0,692238409	False	0,692238409
ENSMUSG00000033088	Triobp	333,0935068	503,5357122	382,1780221	268,7800863	206,1504046	-0,692229938	False	0,692229938
ENSMUSG00000042303	Sgsm3	94,28632906	294,044664	152,3428521	372,2435505	264,8641275	0,692201301	False	0,692201301
ENSMUSG00000003458	Ncstn	653,0487546	633,5211216	404,1928851	463,3363831	1186,017201	-0,692146776	False	0,692146776
ENSMUSG00000032083	Apoa1	109847,4376	99067,81189	203869,0793	67990,11581	115016,2688	-0,692104284	False	0,692104284
ENSMUSG00000074247	Dda1	207,1207884	499,7497294	334,6259179	345,2530816	487,9762743	0,692076829	False	0,692076829
ENSMUSG00000057530	Ece1	3092,900729	550,2295	1914,412488	554,4292157	923,7625727	-0,692058816	False	0,692058816
ENSMUSG00000040722	Scamp5	92,74065154	119,8894553	145,2980959	249,6618375	57,40897345	-0,691925604	False	0,691925604
ENSMUSG00000032038	St3gal4	867,8979306	480,8198154	1401,906477	885,06246	349,6728383	0,691792819	False	0,691792819
ENSMUSG00000000759	Tubgcp3	281,3133097	737,0046514	454,3867728	1058,251302	562,3469899	0,691743027	False	0,691743027
ENSMUSG00000026960	Arl6ip6	108,9702656	148,9153234	89,82064113	67,4761723	206,1504046	-0,691484482	False	0,691484482
ENSMUSG00000028967	Errfi1	7138,711652	9748,905706	4420,584495	12059,11659	14076,94124	-0,691426593	False	0,691426593
ENSMUSG00000026640	Plxna2	432,0168684	267,5427844	210,4620905	160,8182106	180,0554167	-0,691318024	False	0,691318024
ENSMUSG00000028621	Cyb5rl	120,562847	27,76387386	194,6113891	154,0705934	13,04749397	0,690810752	False	0,690810752
ENSMUSG00000027981	Rnpc3	244,217049	159,0112775	52,83567125	120,3325073	394,0343177	0,690157364	False	0,690157364
ENSMUSG00000027286	Lrrc57	125,1998796	161,5352661	137,3727453	77,59759814	233,550142	-0,690149272	False	0,690149272
ENSMUSG00000033808	Tmem87a	170,7973666	363,4543486	318,7752165	275,5277035	473,6240309	0,689911654	False	0,689911654
ENSMUSG00000025555	Farp1	256,5824693	914,9458429	413,8794248	977,2798954	918,5435751	0,689787927	False	0,689787927
ENSMUSG00000006262	Mob1b	381,0095101	304,1406181	123,2832329	196,8055025	614,5369658	0,689672786	False	0,689672786
ENSMUSG00000015013	Trappc2l	78,82955381	167,8452374	153,2234466	127,0801245	147,4366818	0,688929895	False	0,688929895
ENSMUSG00000021025	Nfkbia	471,4316453	2207,227971	759,9530715	2767,647667	3559,356354	0,688861728	False	0,688861728
ENSMUSG00000001052	Sec24b	312,2268602	470,7238613	193,7307946	427,3490912	420,1293057	-0,688541358	False	0,688541358
ENSMUSG00000066072	Cyp4a10	8078,483588	5012,641225	12138,99547	7099,617929	9533,80384	-0,688513532	False	0,688513532
ENSMUSG00000023068	Nus1	611,3154614	413,9341193	346,9542412	239,5404117	985,0857944	0,688332323	False	0,688332323
ENSMUSG00000037110	Ralgapa2	587,3574597	1789,507869	364,5661316	1012,142584	1393,472355	-0,688058239	False	0,688058239
ENSMUSG00000006736	Tspan31	3225,828996	1809,699778	5196,388268	1012,142584	2592,537051	0,687839269	False	0,687839269
ENSMUSG00000002365	Snx9	748,1079224	464,4138899	399,7899125	222,6713686	424,0435539	-0,687835287	False	0,687835287
ENSMUSG00000002763	Pex6	932,0435479	591,8753108	870,0273866	1501,344834	225,7216456	0,687786109	False	0,687786109
ENSMUSG00000028552	Eps15	554,8982317	344,5244347	187,5666329	303,6427753	131,779689	-0,687616899	False	0,687616899
ENSMUSG00000011831	Evi5	1548,768881	545,181523	961,6092168	789,4712159	409,6913105	-0,687599241	False	0,687599241
ENSMUSG00000026796	Fam129b	109,7431043	68,14769037	179,6412823	113,58489	206,1504046	-0,687393623	False	0,687393623
ENSMUSG00000022426	Josd1	418,1057707	159,0112775	214,8650631	88,84362686	259,6451299	-0,68732679	False	0,68732679
ENSMUSG00000026234	Ncl	1969,966006	2671,641861	1035,579157	868,1934169	3171,845783	0,687151893	False	0,687151893
ENSMUSG00000052299	Ltn1	343,9132494	169,1072317	191,089011	213,6745456	198,3219083	-0,686629643	False	0,686629643
ENSMUSG00000039016	Timm8b	370,9626061	395,0042053	597,0430851	129,3293302	328,7968479	0,686561279	False	0,686561279
ENSMUSG00000025047	Pdcd11	215,6220148	595,6612936	346,9542412	810,8386704	618,4512139	0,686240919	False	0,686240919
ENSMUSG00000051984	Sec31b	155,3405913	498,4877351	265,0589508	809,7140676	96,55145534	-0,686064952	False	0,686064952
ENSMUSG00000028683	Eif2b3	91,96781277	295,3066583	147,9398795	163,0674164	300,0923612	0,685810071	False	0,685810071
ENSMUSG00000042015	Wdr41	214,0763373	212,0150367	264,1783563	304,7673782	133,0844384	-0,685783451	False	0,685783451
ENSMUSG00000032459	Mrps22	258,1281468	106,0075184	160,2682028	29,23967466	160,4841758	-0,685656414	False	0,685656414
ENSMUSG00000032265	Fam46a	187,7998194	570,4214083	302,0439207	528,5633497	585,832479	0,685562674	False	0,685562674
ENSMUSG00000037958	Nsrp1	191,6640132	458,1039186	308,2080823	834,4553307	780,2401391	0,685325213	False	0,685325213
ENSMUSG00000001783	Rtcb	625,2265591	306,6646067	222,7904138	240,6650145	388,8153202	-0,685293999	False	0,685293999
ENSMUSG00000055371	Stam2	202,4837559	257,4468303	125,9250165	139,4507561	371,853578	-0,685241253	False	0,685241253
ENSMUSG00000025925	Terf1	142,9751711	256,184836	88,94004661	138,3261532	243,9881371	-0,684859566	False	0,684859566
ENSMUSG00000023806	Rsph3b	125,9727183	74,4576617	78,37291236	38,23649764	58,71372284	-0,684684312	False	0,684684312
ENSMUSG00000026159	Agfg1	226,4417575	257,4468303	140,8951233	235,0420002	555,8232429	-0,684518348	False	0,684518348
ENSMUSG00000075470	Deaf1	526,3031975	194,347117	96,86539729	101,2142584	327,4920985	-0,684434061	False	0,684434061
ENSMUSG00000066150	Slc31a1	2918,239168	644,87907	1816,666497	428,4736941	593,6609754	-0,683804532	False	0,683804532
ENSMUSG00000031357	Syap1	618,2710102	281,4247213	336,387107	321,6364213	384,901072	-0,683751672	False	0,683751672
ENSMUSG00000037826	Ppm1k	557,216748	148,9153234	346,9542412	326,1348328	53,49472526	-0,683493218	False	0,683493218
ENSMUSG00000031631	Cfap97	166,160334	103,4835298	81,01469592	73,09918666	90,02770836	-0,683174851	False	0,683174851
ENSMUSG00000042079	Hnrnpf	900,3571587	805,1523418	513,3866057	271,0292921	1445,662331	0,6831613	False	0,6831613
ENSMUSG00000022751	Nit2	2028,701752	147,6533291	1263,653137	137,2015503	211,3694022	-0,682956275	False	0,682956275
ENSMUSG00000062691	Cebpzos	122,8813633	103,4835298	197,2531727	37,11189476	61,32322164	0,682782378	False	0,682782378
ENSMUSG00000028896	Rcc1	54,87155216	179,2031858	88,05945209	103,4634642	207,455154	0,682419482	False	0,682419482
ENSMUSG00000037706	Cd81	3014,071175	1357,90583	1878,308113	825,4585078	1778,373427	-0,682279747	False	0,682279747
ENSMUSG00000038324	Trpc4ap	1524,03804	786,2224278	625,2221098	845,7013595	949,8575607	-0,682115823	False	0,682115823
ENSMUSG00000029999	Tgfa	244,9898878	152,7013062	101,2683699	93,34203834	120,0369445	-0,682009799	False	0,682009799
ENSMUSG00000021124	Vti1b	394,147769	177,9411915	245,6858713	124,8309187	161,7889252	-0,681921714	False	0,681921714
ENSMUSG00000005397	Nid1	204,8022721	776,1264737	127,6862055	269,9046892	970,733551	-0,681629048	False	0,681629048
ENSMUSG00000109511	Nup62	122,8813633	165,3212489	76,61172331	112,4602872	300,0923612	-0,681629048	False	0,681629048
ENSMUSG00000061544	Zfp229	122,8813633	208,2290539	76,61172331	127,0801245	105,6847011	-0,681629048	False	0,681629048
ENSMUSG00000030058	Copg1	1160,803822	1891,729405	1861,576817	2467,3787	2950,038386	0,681400975	False	0,681400975
ENSMUSG00000031835	Mbtps1	809,1621847	797,5803762	504,5806605	921,0497519	1103,817989	-0,681343987	False	0,681343987
ENSMUSG00000021687	Scamp1	292,1330523	364,7163429	213,9844686	182,1856652	656,2889464	-0,681216147	False	0,681216147
ENSMUSG00000046556	Zfp319	91,96781277	95,91156423	86,29826304	57,35474645	131,779689	-0,681216147	False	0,681216147
ENSMUSG00000002477	Snrpd1	173,1158829	363,4543486	266,8201398	107,9618757	739,7929078	-0,681216147	False	0,681216147
ENSMUSG00000062997	Rpl35	1912,775938	2950,542594	4551,793078	1193,203647	3041,370843	-0,680827595	False	0,680827595
ENSMUSG00000024091	Vapa	769,7474078	1033,573304	516,9089837	480,2054262	2061,504046	-0,680733398	False	0,680733398
ENSMUSG00000022779	Top3b	467,5674515	865,7280666	749,3859372	1526,086097	1034,666271	0,680534401	False	0,680534401
ENSMUSG00000028955	Vamp3	166,9331728	175,417203	119,7608548	103,4634642	267,4736263	0,680125971	False	0,680125971
ENSMUSG00000032096	Arcn1	2211,0917	1722,622173	1165,026551	1197,702058	3542,394612	0,679966074	False	0,679966074

ENSMUSG00000005078	Jkamp	261,2195018	230,9449507	418,2823974	316,0134069	61,32322164	0,679214678	False	0,679214678
ENSMUSG00000009281	Rarres2	5907,579503	2891,228864	3530,303434	1017,765599	3689,831293	-0,679012285	False	0,679012285
ENSMUSG00000038564	Ift172	187,0269806	601,9712649	299,4021371	400,3586223	1513,5093	0,67883811	False	0,67883811
ENSMUSG00000020592	Sdc1	2363,340937	1476,533291	1007,400132	672,5125172	1223,854934	-0,678613883	False	0,678613883
ENSMUSG00000024137	E4f1	133,701106	416,4581078	213,9844686	303,6427753	290,9591154	0,678494687	False	0,678494687
ENSMUSG00000015733	Capza2	623,6808816	638,5690987	368,0885097	390,2371965	998,1332883	0,678424447	False	0,678424447
ENSMUSG00000017868	Sgk2	660,7771422	70,6716789	412,9988303	401,4832252	39,1424819	-0,678026086	False	0,678026086
ENSMUSG00000039519	Cyp7b1	571,9006845	76,98165023	204,2979288	24,74126318	357,5013346	-0,677816004	False	0,677816004
ENSMUSG00000019916	P4ha1	136,0196223	79,50563877	217,5068467	138,3261532	56,10422405	0,677246024	False	0,677246024
ENSMUSG00000030588	Yif1b	375,5996387	109,7935012	600,5654632	111,3356843	172,2269203	0,677125837	False	0,677125837
ENSMUSG00000024949	Sf1	1517,85533	2426,814974	2473,590009	4551,267821	6408,929036	0,677029827	False	0,677029827
ENSMUSG00000035051	Dhx57	235,7158227	379,8602741	354,8795919	1031,260833	147,4366818	-0,676953097	False	0,676953097
ENSMUSG00000033295	Ptprf	2210,318862	718,0747374	1382,533398	916,5513404	382,2915732	-0,676940177	False	0,676940177
ENSMUSG00000027010	Slc25a12	173,1158829	184,2511629	210,4620905	271,0292921	108,2941999	-0,676782118	False	0,676782118
ENSMUSG00000025025	Mxi1	354,7329921	116,1034725	221,9098193	148,4475791	180,0554167	-0,676760009	False	0,676760009
ENSMUSG00000017144	Rnd3	159,9776239	248,6128704	125,044422	100,0896556	294,8733636	-0,676577252	False	0,676577252
ENSMUSG00000030159	Clec1b	89,64929649	150,1773177	77,49231784	210,300737	56,10422405	-0,676182867	False	0,676182867
ENSMUSG00000038542	Pcid2	138,3381385	502,2737179	221,0292247	555,5538186	1569,613524	0,676038188	False	0,676038188
ENSMUSG00000039703	Nploc4	658,4586259	594,3992994	412,1182358	815,3370819	942,0290643	-0,676034489	False	0,676034489
ENSMUSG00000048833	Slc39a9	689,3721764	350,834406	431,4913152	296,8951581	293,5686142	-0,67595155	False	0,67595155
ENSMUSG00000034321	Exosc1	134,4739447	468,1998727	265,0589508	214,7991485	584,5277296	0,675661607	False	0,675661607
ENSMUSG00000040785	Ttc3	302,1799563	263,7568016	249,2082494	594,9149191	189,1886625	-0,675582334	False	0,675582334
ENSMUSG00000020737	Hn1	127,5183959	161,5352661	190,2084165	79,84680388	144,827183	-0,67539882	False	0,67539882
ENSMUSG00000025612	Bach1	295,2244074	330,6424977	184,9248494	244,0388231	541,4709996	-0,674872899	False	0,674872899
ENSMUSG00000035181	Heatr5a	235,7158227	147,6533291	162,0293918	482,4546319	271,3878745	-0,674834719	False	0,674834719
ENSMUSG00000054408	Spcs3	875,6263183	513,6316663	548,6103865	292,3967466	525,8140068	-0,674533381	False	0,674533381
ENSMUSG00000041762	Gpr155	432,7897072	37,85982798	271,2231124	30,36427753	27,39973733	-0,674186065	False	0,674186065
ENSMUSG00000020227	Irak3	30,91355051	193,0851227	49,31329317	475,7070147	791,9828837	0,673737239	False	0,673737239
ENSMUSG00000063160	Numbl	19,32096907	336,9524691	30,82080823	100,0896556	330,1015973	0,673737239	False	0,673737239
ENSMUSG00000032290	Ptpn9	603,5870737	225,8969736	265,0589508	161,9428135	378,377325	-0,673736275	False	0,673736275
ENSMUSG00000048039	Isg20l2	139,8838161	164,0592546	136,4921507	87,71902399	310,5303564	-0,673267394	False	0,673267394
ENSMUSG00000022558	Mroh1	418,1057707	1251,898312	666,6100523	3152,261849	1992,352328	0,672975118	False	0,672975118
ENSMUSG00000038074	Fkbp14	116,6986532	73,19566744	196,3725782	395,8602108	150,0461806	-0,67295775	False	0,67295775
ENSMUSG00000063972	Nr6a1	228,7602738	133,7713922	143,5369069	124,8309187	103,0752023	-0,6724148	False	0,6724148
ENSMUSG00000071652	Ints5	114,3801369	339,4764576	182,2830658	191,1824882	367,9393298	0,672344003	False	0,672344003
ENSMUSG00000025817	Nudt5	174,6615604	184,2511629	239,5217097	380,1157706	109,5989493	-0,672328166	False	0,672328166
ENSMUSG00000026087	Mrpl30	573,446362	651,1890413	818,9529044	359,8729189	678,4696862	-0,672171007	False	0,672171007
ENSMUSG00000032959	Pebp1	964,502776	468,1998727	560,0581153	170,9396365	605,40372	-0,671887851	False	0,671887851
ENSMUSG00000024614	Tmx3	265,0836956	146,3913349	166,4323644	115,8340958	134,3891878	-0,671511931	False	0,671511931
ENSMUSG00000023089	Ndufa5	547,9426828	834,1782099	892,9228441	344,1284787	832,430115	-0,6710777	False	0,6710777
ENSMUSG00000062929	Cfl2	694,009209	185,5131571	435,8942878	224,9205743	335,3205949	-0,670976508	False	0,670976508
ENSMUSG00000021417	Eci2	1823,89948	2864,726984	1145,653472	2032,157389	4443,976445	-0,670855486	False	0,670855486
ENSMUSG00000028851	Nudc	678,5524337	909,8978659	640,1922167	426,2244883	1035,971021	-0,670846817	False	0,670846817
ENSMUSG00000020156	Mum1	132,9282672	167,8452374	295,879759	235,0420002	83,50396138	-0,670731382	False	0,670731382
ENSMUSG00000027957	Slc35a3	608,2241063	275,11475	382,1780221	217,0483542	298,7876118	-0,670358182	False	0,670358182
ENSMUSG00000047649	Cd3eap	67,23697236	227,1589679	132,0891781	157,444402	106,9894505	0,670141882	False	0,670141882
ENSMUSG00000037486	Asxl2	314,5453765	247,3508762	392,7451563	500,4482779	83,50396138	0,669952816	False	0,669952816
ENSMUSG00000038388	Mpp6	779,0214729	489,6537753	424,4465591	352,0006988	434,481549	-0,669901087	False	0,669901087
ENSMUSG00000020427	Igfbp3	289,8145361	340,7384519	264,1783563	182,1856652	537,5567514	-0,669720508	False	0,669720508
ENSMUSG00000024921	Smarca2	1287,549379	643,6170757	1151,817633	2047,901829	285,7401178	0,669518796	False	0,669518796
ENSMUSG00000022257	Laptm4b	302,952795	109,7935012	128,5668	68,60077517	190,4934119	-0,669351912	False	0,669351912
ENSMUSG00000029569	Tmem168	182,389948	217,0630138	142,6563124	114,7094929	394,0343177	-0,669041433	False	0,669041433
ENSMUSG00000033004	Mycbp2	365,5527348	750,8865884	581,1923838	1384,386135	653,6794477	0,668936252	False	0,668936252
ENSMUSG00000027562	Car2	155,3405913	18,92991399	97,74599182	14,61983733	61,32322164	-0,668325413	False	0,668325413
ENSMUSG00000028982	Slc25a33	88,10361896	152,7013062	140,0145288	311,5149954	177,4459179	0,668303352	False	0,668303352
ENSMUSG00000028757	Ddost	1418,15193	1161,034725	2253,441379	766,9791584	1226,464433	0,668110496	False	0,668110496
ENSMUSG00000017119	Nbr1	2466,901331	1153,462759	1497,010685	1291,044097	1552,651782	-0,667965705	False	0,667965705
ENSMUSG00000069678	Pcgf1	57,19006845	90,86358716	168,1935535	122,581713	108,2941999	0,66793763	False	0,66793763
ENSMUSG00000026213	Stk11ip	109,7431043	320,5465436	174,3577151	482,4546319	441,005296	0,667919912	False	0,667919912
ENSMUSG00000005069	Pex5	802,9794746	436,6500161	505,461255	434,0967084	425,3483033	-0,667762601	False	0,667762601
ENSMUSG00000031723	Txnl4b	198,619562	74,4576617	125,044422	48,35792348	26,09498793	-0,667567018	False	0,667567018
ENSMUSG00000001247	Lsr	4442,277209	1545,942976	2796,768198	385,738785	1333,453883	-0,667538738	False	0,667538738
ENSMUSG00000028381	Ugcg	325,3651191	137,557375	168,1935535	68,60077517	204,8456553	-0,667522301	False	0,667522301
ENSMUSG00000036151	Tm6sf2	1010,873102	844,274164	1605,323812	777,1005843	262,2546287	0,667262432	False	0,667262432
ENSMUSG00000058076	Sdhc	2126,852275	720,598726	1339,384266	314,8888041	1075,113503	-0,667149906	False	0,667149906
ENSMUSG00000020326	Ccng1	596,6315249	233,4689392	207,8203069	173,1888422	375,7678262	-0,666998663	False	0,666998663
ENSMUSG00000048756	Foxo3	461,3847414	243,5648934	290,5961919	177,6872537	227,026395	-0,666954496	False	0,666954496
ENSMUSG00000015747	Vps45	124,4270408	66,88569611	78,37291236	57,35474645	37,8377325	-0,666873036	False	0,666873036
ENSMUSG00000034083	Ccdc174	112,0616206	200,6570883	177,8800932	230,5435887	311,8351058	0,666612804	False	0,666612804
ENSMUSG00000025188	Hps1	131,3825897	51,74176491	82,77588496	69,72537804	75,675465	-0,666491673	False	0,666491673
ENSMUSG00000019877	Serinc1	2496,269204	1885,419434	1507,57782	853,5735796	3961,219168	0,666170999	False	0,666170999
ENSMUSG00000020457	Drg1	462,1575802	604,4952535	493,1329317	291,2721437	910,7150788	-0,666017112	False	0,666017112
ENSMUSG00000027712	Anxa5	885,6732222	764,7685253	558,2969262	592,6657133	2545,566073	-0,665741887	False	0,665741887
ENSMUSG00000028986	Klhl7	190,8911744	186,7751514	145,2980959	120,3325073	474,9287803	-0,665723971	False	0,665723971
ENSMUSG00000021585	Cast	595,0858474	1090,363046	375,1332659	909,8037231	2215,464475	-0,665694604	False	0,665694604
ENSMUSG00000026991	Pkp4	1320,008607	471,9858555	832,1618222	584,7934932	330,1015973	-0,665611329	False	0,665611329
ENSMUSG00000034308	Sdr42e1	2614,513535	550,2295	1648,472943	793,9696274	264,8641275	-0,665412331	False	0,665412331
ENSMUSG00000034459	Ifit1	262,7651794	37,85982798	161,1487973	41,61030625	165,7031734	-0,665172876	False	0,665172876
ENSMUSG00000029592	Usp30	209,4393047	302,8786239	132,0891781	235,0420002	480,1477779	-0,66501994	False	0,66501994
ENSMUSG00000036860	Mrpl55	221,0318862	314,2365723	384,8198056	139,4507561	350,9775877	-0,664498752	False	0,664498752
ENSMUSG00000052392	Acot4	1473,030682	755,9345654	929,907814	698,3783833	722,8311657	-0,663627873	False	0,663627873
ENSMUSG00000037434	Slc30a1	325,3651191	376,0742913	221,0292247	95,59124409	515,3760116	0,663565793	False	0,663565793
ENSMUSG00000028693	Nasp	108,9702656	485,8677925	172,5965261	222,6713686	510,157014	0,663468903	False	0,663468903
ENSMUSG00000036442	Thap11	178,5257542	92,12558143	112,7160987	75,3483924	109,5989493	-0,66343863	False	0,66343863
ENSMUSG00000030284	Creld1	602,814235	276,3767443	954,5644606	711,8736177	227,026395	0,663129139	False	0,663129139
ENSMUSG00000030064	Frmd4b	1327,736994	612,0672191	825,9976606	627,5284024	838,953862	-0,662306021	False	0,662306021
ENSMUSG00000021794	Glud1	14608,97113	3483,104175	9233,914146	2776,64449	2944,819388	-0,661840353	False	0,661840353
ENSMUSG00000064068	Mtx1	435,8810622	489,6537753	547,729792	275,5277035	707,1741729	-0,661737139	False	0,661737139
ENSMUSG00000047909	Ankrd16	208,666466	468,1998727	392,7451563	850,1997709	330,1015973	0,661711351	False	0,661711351
ENSMUSG00000028270	Gbp2	94,28632906	292,7826697	77,49231784	59,6039522	1482,195314	-0,661640609	False	0,661640609
ENSMUSG00000035585	Tsen34	246,5355653	73,19566744	155,8652302	39,36110051	86,11346017	-0,661496652	False	0,661496652
ENSMUSG00000039809	Gabbr2	788,2955381	2394,003123	498,4164988	979,5291012	2043,237555	-0,661384783	False	0,661384783
ENSMUSG00000022332	Khdrbs3	267,4022159	97,17355849	169,074148	68,60077517	93,94195655	-0,661355316	False	0,661355316
ENSMUSG00000042410	Agps	140,6566548	132,5093979	88,94004661	119,2079044	307,9208576	-0,661272746	False	0,661272746
ENSMUSG00000027884	Clcc1	637,5919793	348,3104175	329,3423508	277,7769093	403,1675635	-0,661253908	False	0,661253908
ENSMUSG00000038150	Ormdl3	1048,742201	1039,883275	473,7598522	368,8697419	1658,336483	0,661076683	False	0,661076683
ENSMUSG00000046675	Tmem251	62,59993979	191,8231285	101,2683699	98,9650527	279,2163709	0,66075789	False	0,66075789
ENSMUSG00000029162	Khk	6197,394039	18958,93986	5885,893777	3920,36561	16430,70915	-0,660673494	False	0,660673494
ENSMUSG00000013878	Rnf170	225,6689187	88,33959863	124,1638274	142,8245647	49,58047707	-0,659963598	False	0,659963598
ENSMUSG00000042476	Abcb4	2476,948235	974,2595735	1644,06997	3913,617993	636,7177055	0,659939224	False	0,659939224
ENSMUSG00000027956	Tmem144	125,1998796	61,83771904	79,25350688	59,6039522	58,71372284	-0,659686495	False	0,659686495
ENSMUSG00000038351	Sgsm2	208,666466	15,14393119	132,0891781	78,72220101	20,87599034	-0,659686495	False	0,659686495
ENSMUSG00000032380	Dapk2	166,9331728	34,07384519	105,6713425	32,61348328	27,39973733	-0,659686495	False	0,659686495
ENSMUSG00000025078	Nhlrc2	238,0343389	374,812297	176,9994987	150,6967848	639,3272043	-0,659521076	False	0,659521076
ENSMUSG00000022130	Tgds	315,3182152	127,4614209	141,7757179	102,3388613	199,6266577	-0,659504126	False	0,659504126
ENSMUSG00000036896	C1qc	692,4635315	206,9670596	349,5960248	213,6745456	438,3957972	-0,659504126	False	0,659504126
ENSMUSG00000031508	Ankrd10	98,92336164	118,627461	291,4767864	332,88245	62,62797103	-0,659504126	False	0,659504126
ENSMUSG00000066151	Fkbp15	519,3476486	580,5173624	485,207581	1027,887025	328,7968479	-0,659504126	False	0,659504126
ENSMUSG00000087260	Lamtor5	202,4837559	306,6646067	301,1633261	128,2047274	403,1675635	-0,659356713	False	0,659356713
ENSMUSG00000037022	Mmaa	334,6391843	128,7234151	528,3567125	272,1538949	49,58047707	0,658905887	False	0,658905887
ENSMUSG00000047866	Lonp2	3676,393995	1751,648041	1178,235469	1038,008451	2328,977673	-0,658594578	False	0,658594578
ENSMUSG00000051098	Mblac2	272,8120833	55,52774771	430,6107207	86,59442111	9,133245776	0,658476693	False	0,658476693
ENSMUSG00000038425	Poli	124,4270408	82,0296273	196,3725782	182,1856652	22,18073974	0,658293433	False	0,658293433

ENSMUSG00000024371	C2	258,9009855	164,0592546	448,2226111	392,4864022	320,9683515	-0,658183478	False	0,658183478
ENSMUSG00000026219	Trip12	1196,354405	1126,96088	758,1918825	1846,597915	1201,674194	-0,658009917	False	0,658009917
ENSMUSG00000043421	Hilpda	20,09380783	1361,691813	31,70140275	483,5792348	1757,497437	0,657795695	False	0,657795695
ENSMUSG00000024378	Stard4	1451,391197	455,5799301	920,2212743	211,4253399	388,8153202	-0,657383709	False	0,657383709
ENSMUSG00000060429	Sntb1	1094,339688	580,5173624	693,9084824	396,9848137	187,8839131	-0,657243319	False	0,657243319
ENSMUSG00000027593	Raly	535,5772626	993,1894875	387,4615892	339,6300672	1341,28238	-0,657130528	False	0,657130528
ENSMUSG00000015850	Adamtsl4	70,32832742	117,3654668	201,6561453	213,6745456	110,9036987	0,657129669	False	0,657129669
ENSMUSG00000040565	Btaf1	241,8985328	641,0930872	381,2974275	755,7331297	778,9353897	0,656514779	False	0,656514779
ENSMUSG00000024422	Dhx16	510,0735834	827,8682386	803,9827975	908,6791203	1019,009279	0,656459247	False	0,656459247
ENSMUSG00000026721	Rabgap1l	476,8415166	218,325008	291,4767864	159,6936078	302,70186	-0,655612311	False	0,655612311
ENSMUSG00000004865	Srpk1	323,0466029	874,5620264	549,490981	988,5259242	508,8522646	0,655504555	False	0,655504555
ENSMUSG00000048495	Tyw5	91,96781277	106,0075184	50,19388769	40,48570338	144,827183	0,655131476	False	0,655131476
ENSMUSG00000078817	Nlrp12	388,7378977	1693,596305	612,013192	1406,878192	1423,481592	0,654764989	False	0,654764989
ENSMUSG00000020744	Slc25a19	166,160334	109,7935012	261,5365727	215,9237514	63,93272043	0,654436683	False	0,654436683
ENSMUSG00000026547	Tagln2	192,4368519	302,8786239	391,8645618	311,5149954	390,1200696	0,654354658	False	0,654354658
ENSMUSG00000015745	Plekho1	91,19497401	155,2252947	198,1337672	287,8983351	143,5224336	0,654250037	False	0,654250037
ENSMUSG00000032867	Fbxw8	629,8635917	784,9604335	579,4311947	400,3586223	1360,853621	-0,653746544	False	0,653746544
ENSMUSG00000023861	Mpc1	1720,339086	682,738898	1093,698395	278,9015122	805,0303776	-0,653478008	False	0,653478008
ENSMUSG00000037993	Dhx38	246,5355653	407,624148	156,7458247	374,4927562	795,8971319	-0,653368771	False	0,653368771
ENSMUSG00000024769	Cdc42bpg	164,6146565	82,0296273	258,8947891	188,9332824	28,70448672	0,653273136	False	0,653273136
ENSMUSG00000061111	Mcrip1	238,0343389	306,6646067	251,850033	691,630766	151,35093	-0,653272172	False	0,653272172
ENSMUSG00000024990	Rbp4	32893,56342	14101,52393	51726,12216	5824,318272	18120,35962	0,653087732	False	0,653087732
ENSMUSG00000019261	Map1s	101,2418779	176,6791973	167,312959	183,3102681	159,1794264	0,652847709	False	0,652847709
ENSMUSG00000031683	Lsm6	238,0343389	138,8193693	374,2526714	98,9650527	146,1319324	0,652842902	False	0,652842902
ENSMUSG00000020381	Mrnip	192,4368519	30,28786239	122,4026384	60,72855507	18,26649155	-0,652750448	False	0,652750448
ENSMUSG00000018377	Vezf1	235,7158227	196,8711055	143,5369069	178,8118566	370,5488286	0,65261506	False	0,65261506
ENSMUSG00000063894	Zkscan8	152,2492363	66,88569611	96,86539729	16,86904307	48,27572767	-0,652381695	False	0,652381695
ENSMUSG00000001785	Pwp1	178,5257542	203,1810768	140,0145288	113,58489	345,7585901	-0,652361284	False	0,652361284
ENSMUSG00000015750	Aph1a	507,7550672	323,0705321	275,626085	194,5562968	213,978901	-0,652283566	False	0,652283566
ENSMUSG00000058407	Txndc9	493,0711307	593,1373051	499,2970933	313,7642012	681,079185	-0,652115028	False	0,652115028
ENSMUSG00000024811	Tnks2	479,9328717	395,0042053	305,5662987	374,4927562	754,1451512	0,652009597	False	0,652009597
ENSMUSG00000031730	Dhodh	95,83200659	41,64581078	150,5816631	94,46664122	49,58047707	0,651966616	False	0,651966616
ENSMUSG00000023460	Rab12	312,2268602	248,6128704	227,1933864	255,2848519	490,5857731	0,651910742	False	0,651910742
ENSMUSG00000039199	Zdhhc1	47,14316453	113,579484	128,5668	278,9015122	30,00923612	-0,651642008	False	0,651642008
ENSMUSG00000042558	Adprhl2	130,6097509	359,6683658	205,1785234	305,8919811	485,3667755	0,65161712	False	0,65161712
ENSMUSG00000026116	Tmem131	546,3970053	632,2591273	347,8348357	706,2506034	658,8984452	-0,65154715	False	0,65154715
ENSMUSG00000027796	Smad9	173,1158829	8,833959863	58,11923838	110,2110814	14,35224336	-0,651468804	False	0,651468804
ENSMUSG00000090841	Myl6	1359,423384	1587,588787	1784,084499	865,9442111	1339,97763	-0,650648858	False	0,650648858
ENSMUSG00000035329	Fbxo33	122,8813633	215,8010195	192,8502001	245,163246	302,70186	0,650214516	False	0,650214516
ENSMUSG00000039323	Igfbp2	5049,728476	2444,482894	3217,692379	233,9173973	3047,89459	-0,650179409	False	0,650179409
ENSMUSG00000039826	Trub2	91,19497401	147,6533291	58,11923838	205,8023255	108,2941999	-0,649938519	False	0,649938519
ENSMUSG00000029009	Mthfr	309,1355051	502,2737179	367,2079152	1518,213877	197,0171589	-0,649918077	False	0,649918077
ENSMUSG00000021767	Kat6b	257,355308	164,0592546	245,6858713	413,8538568	241,3786384	-0,64954456	False	0,64954456
ENSMUSG00000074254	Cyp2a4	8020,52068	2404,099077	2511,455573	954,787838	5113,312885	-0,64943759	False	0,64943759
ENSMUSG00000041642	Kif21b	64,91845608	82,0296273	41,38794248	194,5562968	126,5606915	-0,64941816	False	0,64941816
ENSMUSG00000030681	Mvp	476,8415166	1712,526219	922,8630579	747,8609096	1848,829895	0,649260126	False	0,649260126
ENSMUSG00000036585	Fgf1	2197,180603	1095,411023	1401,025883	1224,692527	403,1675635	-0,649169852	False	0,649169852
ENSMUSG00000030451	Herc2	428,1526746	259,9708188	404,1928851	671,3879144	82,19921198	0,64902123	False	0,64902123
ENSMUSG00000032398	Snapc5	193,9825295	215,8010195	269,4619234	123,7063159	167,0079228	-0,649007566	False	0,649007566
ENSMUSG00000024998	Plce1	139,1109773	132,5093979	155,8652302	654,5188713	88,72295896	-0,648856882	False	0,648856882
ENSMUSG00000041445	Mmm2	139,1109773	99,69754702	268,5813289	275,5277035	88,72295896	-0,648856882	False	0,648856882
ENSMUSG00000044676	Zfp612	140,6566548	524,9896147	458,7897454	1257,30601	220,502648	0,648618169	False	0,648618169
ENSMUSG00000078566	Bnip3	1821,580964	1788,245875	1505,816631	850,1997709	2854,79168	0,648194353	False	0,648194353
ENSMUSG00000026766	Mmadhc	469,113129	637,3071044	299,4021371	519,5665267	1024,228276	-0,647851359	False	0,647851359
ENSMUSG00000003184	Irf3	1067,290331	1009,595413	1672,248995	1400,130575	168,3126721	0,647836997	False	0,647836997
ENSMUSG00000026455	Klhl12	152,2492363	97,17355849	103,9101535	283,3999236	240,073889	-0,647799285	False	0,647799285
ENSMUSG00000042210	Abhd14a	166,9331728	54,26575344	261,5365727	112,4602872	28,70448672	0,64774203	False	0,64774203
ENSMUSG00000024986	Hhex	490,7526144	191,8231285	768,7590167	349,7514931	109,5989493	0,647535473	False	0,647535473
ENSMUSG00000034748	Sirt6	74,96535999	199,395094	182,2830658	151,8213877	117,4274457	0,647473627	False	0,647473627
ENSMUSG00000006800	Sulf2	195,528207	47,95578211	114,4772877	124,8309187	92,63720715	-0,647401434	False	0,647401434
ENSMUSG00000059734	Ndufs8	1177,806275	834,1782099	1844,845521	400,3586223	478,8430285	0,647397752	False	0,647397752
ENSMUSG00000072625	Gdf2	308,3626664	196,8711055	179,6412823	132,7031389	140,9129348	-0,647376723	False	0,647376723
ENSMUSG00000060475	Wtap	249,6269204	844,274164	390,9839673	395,8602108	1017,704529	0,647335923	False	0,647335923
ENSMUSG00000021224	Numb	539,4414564	410,1481365	742,3411811	873,8164312	344,4538407	-0,647155734	False	0,647155734
ENSMUSG00000021428	Riok1	113,6072981	371,0263142	177,8800932	680,3847373	617,1464645	0,646849549	False	0,646849549
ENSMUSG00000044712	Slc38a6	142,9751711	117,3654668	280,0290576	290,1475409	91,33245776	-0,64656507	False	0,64656507
ENSMUSG00000024188	Luc7l	392,6020915	614,5912076	636,6698386	1141,471915	1223,854934	0,646559269	False	0,646559269
ENSMUSG00000038967	Pdk2	1433,615905	41,64581078	915,8183017	165,3166221	108,2941999	-0,646525247	False	0,646525247
ENSMUSG00000040740	Slc25a34	95,05916782	135,0333865	91,58183017	60,72855507	129,1701903	-0,646450729	False	0,646450729
ENSMUSG00000069844	Sco1	136,792461	46,69378785	72,20875071	33,73808615	87,41820957	-0,645982986	False	0,645982986
ENSMUSG00000037885	Stk35	126,7455571	137,557375	70,44756167	32,61348328	198,3219083	-0,645908882	False	0,645908882
ENSMUSG00000040706	Agmat	2360,249582	215,8010195	1508,458414	250,7864404	412,3008093	-0,645864499	False	0,645864499
ENSMUSG00000040532	Abhd11	483,7970655	551,4914943	974,8181346	1324,782183	309,225607	-0,645742225	False	0,645742225
ENSMUSG00000046811	Gltpd2	727,2412758	133,7713922	1137,728121	200,1793111	178,7506673	0,645649853	False	0,645649853
ENSMUSG00000014355	Anapc1	487,6612593	463,1518957	311,7304604	542,0585841	716,3074187	-0,645580234	False	0,645580234
ENSMUSG00000029614	Rpl6	2974,656398	3479,318192	4082,436199	1901,703456	3625,898573	-0,645430737	False	0,645430737
ENSMUSG00000020329	Polrmt	294,4515686	743,3146228	468,4762851	542,0585841	460,576537	0,645410559	False	0,645410559
ENSMUSG00000027406	Idh3b	2595,965404	1346,547882	1647,592349	518,4419238	1659,641232	-0,645399752	False	0,645399752
ENSMUSG00000028822	Tmem50a	809,1621847	405,1001594	508,1030385	517,3173209	444,9195442	-0,645379402	False	0,645379402
ENSMUSG00000009863	Sdhb	2523,318561	1118,12692	1613,249162	485,8284405	1150,788968	-0,645353077	False	0,645353077
ENSMUSG00000002102	Psmc3	1141,482853	1612,828672	1062,877587	729,8672637	1745,754693	-0,645203168	False	0,645203168
ENSMUSG00000037355	Uvssa	296,7700849	1302,378083	464,0733125	1947,812174	906,8008306	0,645007062	False	0,645007062
ENSMUSG00000034453	Polr3b	176,2072379	206,9670596	112,7160987	289,022938	211,3694022	-0,644579603	False	0,644579603
ENSMUSG00000005774	Rfx5	364,779896	32,81185092	233,357548	110,2110814	44,36147948	-0,644484089	False	0,644484089
ENSMUSG00000037363	Letm2	73,41968247	116,1034725	113,5966932	145,0737704	46,97097827	-0,644397234	False	0,644397234
ENSMUSG00000045594	Glb1	188,5726581	47,95578211	120,6414494	33,73808615	49,58047707	-0,644394843	False	0,644394843
ENSMUSG00000020608	Smc6	313,7725377	528,7755975	200,7755508	326,1348328	362,7203322	-0,644135492	False	0,644135492
ENSMUSG00000033159	Cnppd1	595,8586861	710,5027718	381,2974275	1441,740881	707,1741729	-0,644053424	False	0,644053424
ENSMUSG00000038776	Ephx1	6207,440943	795,0563877	3972,361884	646,6466512	1067,285006	-0,644001572	False	0,644001572
ENSMUSG00000027573	Gid8	507,7550672	165,3212489	324,9393782	86,59442111	159,1794264	-0,643962143	False	0,643962143
ENSMUSG00000022337	Emc2	407,286028	198,1330998	260,6559782	160,8182106	208,7599034	-0,643895375	False	0,643895375
ENSMUSG00000033166	Dis3	125,1998796	261,2328131	80,1341014	166,441225	416,2150575	-0,643744951	False	0,643744951
ENSMUSG00000059895	Ptp4a3	140,6566548	100,9595413	384,8198056	258,6586605	90,02770836	-0,64373681	False	0,64373681
ENSMUSG00000015966	Il17rb	116,6986532	16,40592546	182,2830658	116,9586986	13,04749397	0,64339263	False	0,64339263
ENSMUSG00000019944	Rhobtb1	636,8191405	781,1744507	407,7152632	1199,951264	478,8430285	-0,643321733	False	0,643321733
ENSMUSG00000036810	Cnep1r1	251,1725979	251,136859	268,5813289	160,8182106	373,1583274	-0,643248297	False	0,643248297
ENSMUSG00000002332	Dhrs1	1990,059814	234,7309335	1274,220272	263,157072	301,3971106	-0,643197099	False	0,643197099
ENSMUSG00000004035	Gstm7	1122,161884	82,0296273	718,565129	158,5690049	182,6649155	-0,643089985	False	0,643089985
ENSMUSG00000020717	Pecam1	156,8862688	136,2953807	154,9846357	268,7800863	100,4657035	-0,643016003	False	0,643016003
ENSMUSG00000043998	Mgat2	694,7820478	147,6533291	298,5215426	244,0388231	444,9195442	-0,643016003	False	0,643016003
ENSMUSG00000049922	Slc35c1	267,4022119	184,2511629	224,5516028	206,9269284	417,5198069	0,64283324	False	0,64283324
ENSMUSG00000022052	Ppp2r2a	236,4886614	436,6500161	151,4622576	352,0006988	887,2295896	-0,642812677	False	0,642812677
ENSMUSG00000004096	Cwc15	728,0141146	808,9383246	519,5507673	196,8055025	1136,436724	0,642479032	False	0,642479032
ENSMUSG00000037366	Pafah2	1376,425837	933,8757569	1585,070138	2147,991485	281,8258696	0,642061396	False	0,642061396
ENSMUSG00000001482	Def8	480,7057105	400,0521824	513,3866057	750,1101154	238,7691396	0,641948455	False	0,641948455
ENSMUSG00000025504	Eps8l2	1296,823444	292,7826697	2023,606209	1302,290125	143,5224336	0,641946494	False	0,641946494
ENSMUSG00000047824	Pygo2	171,5702053	392,4802168	267,7007343	273,2784978	498,4142695	0,641822058	False	0,641822058
ENSMUSG00000090958	Lrrc32	234,1701451	711,7647661	474,6404467	761,3561441	365,329831	0,641642407	False	0,641642407
ENSMUSG00000003581	Rnf215	144,5208486	436,6500161	225,4321973	322,7610242	417,5198069	0,641415951	False	0,641415951
ENSMUSG00000019470	Xab2	224,1232412	588,089328	349,5960248	665,7649	891,1438378	0,641396518	False	0,641396518
ENSMUSG00000034724	Cnot6l	547,1698441	340,7384519	212,2232795	350,8760959	320,9683515	-0,641027057	False	0,641027057
ENSMUSG00000007815	Rhoa	2415,121134	1135,79484	966,0121894	668,0141057	1548,737534	-0,641002882	False	0,641002882
ENSMUSG00000001100	Poldip2	1188,626017	615,8532019	762,5948551	453,2149573	375,7678262	-0,640306162	False	0,640306162

ENSMUSG00000060450	Rnf14	785,204183	344,5244347	367,2079152	503,8220865	251,8166335	-0,64015349	False	0,64015349
ENSMUSG00000055917	Zfp277	198,619562	127,4614209	102,1489644	120,3325073	96,55145534	-0,639947072	False	0,639947072
ENSMUSG00000034902	Pip5k1c	557,9895867	334,4284805	619,0579482	869,3180198	310,5303564	0,639645851	False	0,639645851
ENSMUSG00000028945	Rheb	525,5303587	760,9825425	594,4013016	337,3808615	1238,207177	-0,639395971	False	0,639395971
ENSMUSG00000021112	Mpp5	152,2492363	68,14769037	97,74599182	51,73173209	67,84696862	-0,639325542	False	0,639325542
ENSMUSG00000051124	Gimap9	150,7035587	234,7309335	162,9099864	156,3197992	48,27572767	0,639294491	False	0,639294491
ENSMUSG00000034361	Cpne2	115,9258144	369,76432	180,5218768	369,9943448	267,4736263	0,63897182	False	0,63897182
ENSMUSG00000014791	Elmo3	399,5576404	424,0300734	450,8643947	621,905388	74,3707156	0,638291474	False	0,638291474
ENSMUSG00000025314	Ptprj	663,8684972	426,554062	195,4919836	151,8213877	322,2731009	-0,638168891	False	0,638168891
ENSMUSG00000037523	Mavs	1377,198675	176,6791973	884,9974935	525,189541	41,75198069	-0,637991424	False	0,637991424
ENSMUSG00000030671	Pde3b	1065,744654	224,6349794	685,1025372	545,4323927	318,3588527	-0,637469985	False	0,637469985
ENSMUSG00000029135	Fosl2	97,37768411	485,8677925	151,4622576	395,8602108	664,1174428	0,637295243	False	0,637295243
ENSMUSG00000030834	Abcc6	2345,565645	318,0225551	3647,422505	3114,025352	348,3680889	0,636941448	False	0,636941448
ENSMUSG00000007812	Zfp655	371,7354449	300,3546353	172,5965261	247,4126318	578,0039827	0,636803178	False	0,636803178
ENSMUSG00000035493	Tgfbi	599,7228799	261,2328131	300,2827316	385,738785	255,7308817	-0,636671802	False	0,636671802
ENSMUSG00000036501	Fam13b	239,5800165	309,1885952	154,1040411	314,8888041	331,4063467	-0,636602882	False	0,636602882
ENSMUSG00000059325	Hopx	323,0466029	160,2732718	207,8203069	121,4571101	143,5224336	-0,636405671	False	0,636405671
ENSMUSG00000002768	Mea1	620,5895265	752,1485826	1053,191047	399,2340194	481,4525273	-0,636404689	False	0,636404689
ENSMUSG00000023092	Fhl1	125,1998796	64,36170757	194,6113891	166,441225	58,71372284	0,636362968	False	0,636362968
ENSMUSG00000025184	R3hcc1l	133,701106	449,2699587	207,8203069	400,3586223	438,3957972	0,636325233	False	0,636325233
ENSMUSG00000028833	Ncdn	150,7035587	816,5102902	234,2381425	975,0306897	1093,379994	0,636262533	False	0,636262533
ENSMUSG00000021094	Dhrs7	424,2884808	129,9854094	272,9843015	53,98093784	123,9511927	-0,636227522	False	0,636227522
ENSMUSG00000060376	Bckdha	2661,656699	1070,171138	1713,636938	1386,635341	1323,015888	-0,635263021	False	0,635263021
ENSMUSG00000021185	Dglucy	748,1079224	66,88569611	481,6852029	228,2943829	82,19921198	-0,635155803	False	0,635155803
ENSMUSG00000027007	Ssfa2	578,8562333	517,4176491	265,0589508	347,5022873	898,9723342	0,635071637	False	0,635071637
ENSMUSG00000075704	Txnrd2	471,4316453	251,136859	617,2967591	732,1164694	148,7414312	0,635024575	False	0,635024575
ENSMUSG00000041841	Rpl37	1278,275314	3067,908061	2172,426683	823,209302	2199,807483	-0,634867407	False	0,634867407
ENSMUSG00000046032	Snx12	133,701106	85,8156101	66,04458906	23,6166603	86,11346017	-0,634700735	False	0,634700735
ENSMUSG00000020430	Pes1	414,2415769	572,9453968	266,8201398	727,6180579	617,1464645	-0,634604796	False	0,634604796
ENSMUSG00000042670	Immp1l	236,4886614	94,64956996	152,3428521	67,4761723	133,0844384	-0,634449204	False	0,634449204
ENSMUSG00000035357	Pdzrn3	53,32587463	331,904492	82,77588496	427,3490912	634,1082067	0,634374806	False	0,634374806
ENSMUSG00000019478	Rab4a	311,4540214	868,2520551	483,446392	659,0172828	854,6108547	0,634336722	False	0,634336722
ENSMUSG00000025135	Anapc11	311,4540214	174,1552087	483,446392	176,5626508	193,1029107	0,634336722	False	0,634336722
ENSMUSG00000052369	Tmem106c	232,6244676	164,0592546	361,0437536	200,1793111	95,24670595	0,634170834	False	0,634170834
ENSMUSG00000033253	Szt2	382,5551876	1000,761453	744,9829646	2396,528719	246,5976359	-0,633508918	False	0,633508918
ENSMUSG00000029415	Sdad1	128,2912346	396,2661996	199,0143617	358,748316	562,3469899	0,633449942	False	0,633449942
ENSMUSG00000023829	Slc22a1	4794,691684	1409,647595	3090,886768	732,1164694	1816,21116	-0,633417244	False	0,633417244
ENSMUSG00000025264	Tsr2	129,0640734	170,3692259	151,4622576	83,2206125	322,2731009	-0,633074652	False	0,633074652
ENSMUSG00000037692	Ahdc1	276,6762771	429,0780505	197,2531727	181,0610623	95,24670595	0,633041153	False	0,633041153
ENSMUSG00000035505	Cox18	142,9751711	328,1185092	169,9547425	92,21743547	407,0818117	-0,632653182	False	0,632653182
ENSMUSG00000047098	Rnf31	232,6244676	379,8602741	325,8199727	774,8513785	150,0461806	-0,632596253	False	0,632596253
ENSMUSG00000071337	Tia1	163,8418177	182,9891686	326,7005672	490,326852	105,6847011	-0,632537078	False	0,632537078
ENSMUSG00000030057	Cnbp	3244,377126	3037,620199	3652,706073	2092,885944	6508,08999	-0,632447837	False	0,632447837
ENSMUSG00000041769	Ppp2r2d	356,2786697	1101,720994	229,8351699	976,1552926	2014,533068	-0,632406533	False	0,632406533
ENSMUSG00000030166	Rad52	85,01226391	235,9929278	158,5070138	336,2562586	131,779689	0,632385142	False	0,632385142
ENSMUSG00000031431	Tsc22d3	548,7155216	196,8711055	353,9989974	156,3197992	240,073889	-0,632313112	False	0,632313112
ENSMUSG00000024780	Cdc37l1	577,3105558	320,5465436	372,4914823	301,3935696	280,5211202	-0,632140168	False	0,632140168
ENSMUSG00000068246	Apol9b	1821,580964	80,76763303	2823,186034	118,0833015	322,2731009	0,632133078	False	0,632133078
ENSMUSG00000037499	Nenf	96,60484535	71,93367317	149,7010685	39,36110051	69,15171802	0,631917063	False	0,631917063
ENSMUSG00000029370	Rassf6	313,7725377	75,71965597	202,5367398	133,8277417	96,55145534	-0,631535455	False	0,631535455
ENSMUSG00000025236	Adpgk	271,2664057	420,2440906	518,6701728	1157,216355	531,0330044	0,631517141	False	0,631517141
ENSMUSG00000029815	Malsu1	167,7060115	387,4322397	282,6708412	259,7832633	382,2915732	0,631374083	False	0,631374083
ENSMUSG00000035845	Alg12	193,9825295	333,1664863	479,0434193	918,8005461	125,2559421	-0,63104768	False	0,63104768
ENSMUSG00000033728	Lrrc14	90,42213525	223,3729851	140,0145288	204,6777226	163,0936746	0,630828647	False	0,630828647
ENSMUSG00000038080	Kdm1b	335,4120231	100,9595413	216,6262521	124,8309187	49,58047707	-0,630726316	False	0,630726316
ENSMUSG00000051451	Crebzf	328,4564742	90,86358716	212,2232795	93,34203834	43,05673008	-0,630119283	False	0,630119283
ENSMUSG00000060601	Nr1h2	744,2437286	2599,708188	1151,817633	2419,020777	4021,23764	0,630065249	False	0,630065249
ENSMUSG00000059005	Hnrnpa3	1842,447611	1137,056834	1190,563792	808,5894647	1077,723002	-0,629978673	False	0,629978673
ENSMUSG00000028920	Fbxo42	132,9282672	205,7050654	242,1634932	341,879273	217,8931492	0,629929394	False	0,629929394
ENSMUSG00000069633	Pex11g	523,2118424	129,9854094	338,148296	190,0578853	74,3707156	-0,629739112	False	0,629739112
ENSMUSG00000031389	Arhgap4	196,3010458	109,7935012	287,0738138	303,6427753	54,79947465	0,629307185	False	0,629307185
ENSMUSG00000017386	Traf4	508,5279059	347,0484232	786,3709071	398,1094166	88,72295896	0,628883003	False	0,628883003
ENSMUSG00000041679	Lrrc29	85,78510267	239,7789106	55,47745481	92,21743547	116,1226963	-0,628825531	False	0,628825531
ENSMUSG00000047067	Dusp28	115,9258144	179,2031858	116,2384768	85,46981824	69,15171802	0,628394423	False	0,628394423
ENSMUSG00000074797	Itpa	238,0343389	271,3287672	212,2232795	98,9650527	367,9393298	0,628298185	False	0,628298185
ENSMUSG00000053580	Tanc2	91,19497401	259,9708188	58,9998329	218,1729571	182,6649155	-0,628243448	False	0,628243448
ENSMUSG00000021752	Kctd6	78,05671504	285,2107041	120,6414494	194,5562968	524,5092574	0,628131012	False	0,628131012
ENSMUSG00000015290	Ubl4a	373,2811224	1067,647149	774,0425838	576,9212731	862,4393511	0,628111911	False	0,628111911
ENSMUSG00000026730	Pter	1491,578812	66,88569611	965,1315949	97,84044983	45,66622888	-0,628042638	False	0,628042638
ENSMUSG00000009907	Vps4b	639,1376568	384,9082512	222,7904138	421,7260769	987,6952932	0,627939344	False	0,627939344
ENSMUSG00000045503	Sys1	249,6269204	694,0968464	399,7899125	385,738785	798,5066307	0,627850686	False	0,627850686
ENSMUSG00000061689	Dlgap4	318,4095703	1376,835744	564,4610879	1984,924068	491,8905225	0,627453559	False	0,627453559
ENSMUSG00000004929	Thop1	102,7875555	157,7492833	176,1189042	341,879273	66,54221922	-0,62732372	False	0,62732372
ENSMUSG00000025283	Sat1	576,5377171	1326,355974	400,670507	373,3681534	3030,932848	-0,626816115	False	0,626816115
ENSMUSG00000025571	Tnrc6c	235,7158227	292,7826697	317,0140275	581,4196846	152,6556794	-0,62676734	False	0,62676734
ENSMUSG00000042742	Bmt2	112,8344594	155,2252947	73,08934523	174,3134451	227,026395	-0,626474716	False	0,626474716
ENSMUSG00000002897	Il17ra	159,2047851	537,6095574	245,6858713	254,160249	786,7638861	0,625931196	False	0,625931196
ENSMUSG00000024007	Ppil1	159,2047851	94,64956996	245,6858713	79,84680388	69,15171802	0,625931196	False	0,625931196
ENSMUSG00000052906	Ubxn8	289,8145361	453,0559415	335,5065124	187,8086796	448,8337924	-0,625866219	False	0,625866219
ENSMUSG00000020949	Fkbp3	286,723181	126,1994266	185,8054439	150,6967848	87,41820957	-0,625865777	False	0,625865777
ENSMUSG00000038009	Dnajc22	1105,93227	511,1076778	716,80394	242,9142203	176,1411685	-0,625612561	False	0,625612561
ENSMUSG00000072115	Ang	3468,500367	5988,162793	16131,61103	5382,349344	5350,777275	0,625436441	False	0,625436441
ENSMUSG00000037697	Ddhd1	366,3255736	465,6758842	570,6252495	556,6784215	237,4643902	-0,625415236	False	0,625415236
ENSMUSG00000021076	Actr10	411,1502218	591,8753108	357,5213755	266,5308806	797,2018813	-0,625362912	False	0,625362912
ENSMUSG00000038587	Akap12	44,82464824	628,4731445	29,05961919	98,9650527	544,0804983	-0,625276464	False	0,625276464
ENSMUSG00000022244	Amacr	1432,843066	825,3442501	929,0272195	653,3942684	735,8786596	-0,625087834	False	0,625087834
ENSMUSG00000037361	Sf3b6	285,9503422	292,7826697	200,7755508	123,7063159	441,005296	0,625031349	False	0,625031349
ENSMUSG00000041124	Msantd4	196,3010458	100,9595413	115,3578822	91,0928326	302,70186	0,624829682	False	0,624829682
ENSMUSG00000000088	Cox5a	1228,813633	596,9232879	796,9380414	167,5658279	782,8496379	-0,624726657	False	0,624726657
ENSMUSG00000033499	Larp4b	790,6140543	1510,607137	1386,93637	2607,954059	512,7665128	-0,624671554	False	0,624671554
ENSMUSG00000020153	Ndufs7	953,6830333	879,6100035	1649,353538	618,5315794	798,5066307	-0,624662596	False	0,624662596
ENSMUSG00000032097	Ddx6	703,2832742	589,3513223	456,1479618	861,4457997	990,304792	-0,624604036	False	0,624604036
ENSMUSG00000035367	Rmi1	157,6591076	88,33959863	243,0440878	168,6904307	73,0659662	0,624409526	False	0,624409526
ENSMUSG00000001604	Tcea3	1204,082792	75,71965597	781,08734	32,61348328	126,5606915	-0,624378812	False	0,624378812
ENSMUSG00000078964	Ces1b	1665,467534	448,0079645	1080,489477	102,3388613	597,5752236	-0,624242209	False	0,624242209
ENSMUSG00000040747	Cd53	121,3356858	151,4393119	83,65647948	78,72220101	412,3008093	-0,624161458	False	0,624161458
ENSMUSG00000024065	Ehd3	935,134903	60,57572477	606,7296249	184,4348709	134,3891878	-0,624120748	False	0,624120748
ENSMUSG00000036529	Sbf1	550,2611991	2541,656452	848,0125236	3248,977696	5890,943525	0,623968968	False	0,623968968
ENSMUSG00000030830	Itgal	170,7973666	122,4134438	155,8652302	263,157072	106,9894505	0,623638435	False	0,623638435
ENSMUSG00000021792	Fam213a	799,1152807	265,0187959	518,6701728	251,9110432	395,3390671	-0,623586235	False	0,623586235
ENSMUSG00000062169	Cnih4	188,5726581	261,2328131	122,4026384	122,581713	287,0448672	-0,623485853	False	0,623485853
ENSMUSG00000021943	Gdf10	96,60484535	70,6716789	148,820474	60,72855507	37,8377325	0,623405563	False	0,623405563
ENSMUSG00000014164	Klhl3	105,8789105	39,12182225	117,1190713	163,0674164	11,74274457	0,623053281	False	0,623053281
ENSMUSG00000043131	Mob1a	482,251388	176,6791973	164,6711754	103,4634642	313,1398552	-0,62297825	False	0,62297825
ENSMUSG00000049800	Sertad2	231,0787901	116,1034725	137,3727453	87,71902399	150,0461806	-0,62297825	False	0,62297825
ENSMUSG00000033916	Chmp2a	835,4387026	2234,991845	2040,337505	1286,545685	1740,535695	0,622896803	False	0,622896803
ENSMUSG00000001663	Gstt1	6482,571542	1982,592992	4210,122404	376,741962	3431,490913	-0,622704044	False	0,622704044
ENSMUSG00000029802	Abcg2	402,6489954	233,4689392	261,5365727	211,4253399	163,0936746	-0,62251003	False	0,62251003
ENSMUSG00000037295	Ldlrap1	353,1873146	225,8969736	205,1785234	229,4189858	200,9314071	-0,622448739	False	0,622448739
ENSMUSG00000060510	Zfp266	258,9009855	97,17355849	168,1935535	136,0769475	153,9604288	-0,622278046	False	0,622278046
ENSMUSG00000060600	Eno3	52,55303587	261,2328131	143,5369069	664,6402971	80,89446258	0,622266842	False	0,622266842
ENSMUSG00000040220	Gas8	44,05180948	228,4209622	67,80577811	191,1824882	194,4076601	0,622206938	False	0,622206938
ENSMUSG00000020821	Kif1c	2305,378029	806,4143361	1191,444387	1497,971025	559,7374911	-0,621993621	False	0,621993621

ENSMUSG00000004895	Prcc	285,9503422	275,11475	185,8054439	195,6808997	679,7744356	-0,62197186	False	0,62197186
ENSMUSG00000091867	Cyp2a22	1223,403762	793,7943934	1882,711086	701,7521919	908,10558	0,621911009	False	0,621911009
ENSMUSG00000036580	Spg20	564,1722968	232,206945	240,4023042	366,6205361	204,8456553	-0,621848225	False	0,621848225
ENSMUSG00000001305	Rrp15	79,60239257	90,86358716	66,92518359	51,73173209	199,6266577	-0,621762297	False	0,621762297
ENSMUSG00000045636	Mtus1	2150,810277	2420,505002	1475,876417	1397,881369	3533,261366	-0,621638338	False	0,621638338
ENSMUSG00000026434	Nucks1	404,9675117	1659,52246	671,0130249	623,0299909	1384,33911	0,621495439	False	0,621495439
ENSMUSG00000032301	Psma4	1826,990835	2622,424085	1873,024546	1187,580632	2505,118841	-0,621443926	False	0,621443926
ENSMUSG00000028187	Rpf1	330,7749905	396,2661996	176,9994987	229,4189858	508,8522646	0,621396701	False	0,621396701
ENSMUSG00000025347	Mettl7b	10498,24175	2811,723225	16147,46173	3570,614117	1120,779732	0,621159675	False	0,621159675
ENSMUSG00000019906	Lin7a	203,2565946	22,71589679	312,6110549	41,61030625	11,74274457	0,621066636	False	0,621066636
ENSMUSG00000034522	Zfp395	727,2412758	184,2511629	472,8792577	228,2943829	99,16095413	-0,620962223	False	0,620962223
ENSMUSG00000038527	C1rl	2502,451914	907,3738774	3848,198056	1670,035264	229,6358938	0,620840705	False	0,620840705
ENSMUSG00000032363	Adamts7	159,2047851	7,571965597	244,8052768	100,0896556	19,57124095	0,620750957	False	0,620750957
ENSMUSG00000044469	Tnfaip8l1	349,3231208	49,21777638	227,1933864	64,10236368	36,5329831	-0,620641296	False	0,620641296
ENSMUSG00000025234	Arih1	561,8537806	473,2478498	365,4467262	411,604651	1085,551498	-0,620533616	False	0,620533616
ENSMUSG00000057551	Zfp317	151,4763975	102,2215356	184,9248494	232,7927944	39,1424819	0,619953388	False	0,619953388
ENSMUSG00000035172	Plekhh3	117,4714919	111,0554954	180,5218768	129,3293302	43,05673008	0,619862997	False	0,619862997
ENSMUSG00000019947	Arid5b	747,3350836	1148,414782	599,6848687	431,8475027	649,7651995	0,61981665	False	0,61981665
ENSMUSG00000028145	Them4	193,2096907	80,76763303	156,7458247	296,8951581	19,57124095	0,619786109	False	0,619786109
ENSMUSG00000022965	Ifngr2	292,1330523	176,6791973	178,7606877	154,0705934	448,8337924	0,619555704	False	0,619555704
ENSMUSG00000020859	Spag9	977,6410349	797,5803762	501,9388769	598,2887277	1501,766555	0,619283822	False	0,619283822
ENSMUSG00000010609	Psen2	3670,984123	200,6570883	2389,93353	1044,756068	223,1121468	-0,619196381	False	0,619196381
ENSMUSG00000038422	Hdhd3	333,0935068	22,71589679	511,6254166	38,23649764	6,523746983	0,619160706	False	0,619160706
ENSMUSG00000039041	Adrm1	1271,319765	1807,175789	1122,758014	827,7077135	1600,92751	-0,619133638	False	0,619133638
ENSMUSG00000031453	Rasa3	156,8862688	152,7013062	102,1489644	272,1538949	134,3891878	-0,619044511	False	0,619044511
ENSMUSG00000029552	Tes	17,77529154	185,5131571	27,29843015	94,46664122	540,1662502	0,618944766	False	0,618944766
ENSMUSG00000031402	Mpp1	342,3675719	142,6053521	525,714929	211,4253399	100,4657035	0,618734644	False	0,618734644
ENSMUSG00000020358	Hnrnpab	1495,443006	2858,417013	1453,861554	973,9060868	4064,29437	-0,618718362	False	0,618718362
ENSMUSG00000026484	Rnf2	187,7998194	181,7271743	132,9697726	82,09600963	288,3496166	0,618623429	False	0,618623429
ENSMUSG00000032458	Copb2	1925,141358	2362,453266	1387,816965	1253,932202	3819,001484	-0,618505037	False	0,618505037
ENSMUSG00000041815	Poldip3	376,3724775	363,4543486	289,7155974	245,163426	677,1649368	-0,618417363	False	0,618417363
ENSMUSG00000047230	Cldn2	1594,366368	866,9900608	751,1471263	508,320498	1038,58052	-0,618370112	False	0,618370112
ENSMUSG00000059981	Taok2	721,0585657	1071,433132	1607,965595	1811,735226	469,7097827	-0,618346801	False	0,618346801
ENSMUSG00000020659	Cbll1	132,9282672	146,3913349	103,0295589	86,59442111	270,0831251	-0,61830194	False	0,61830194
ENSMUSG00000021361	Tmem14c	996,1891653	262,4948074	648,9981619	114,7094929	194,4076601	-0,618205328	False	0,618205328
ENSMUSG00000023348	Trip6	169,2516891	749,6245941	259,7753837	467,8347946	463,1860358	0,618094496	False	0,618094496
ENSMUSG00000034101	Ctnnd1	155,3405913	219,5870023	202,5367398	101,2142584	200,9314071	-0,61802232	False	0,61802232
ENSMUSG00000067787	Blcap	356,2786697	434,1260276	207,8203069	177,6872537	546,6899971	0,617716866	False	0,617716866
ENSMUSG00000013539	Tango2	268,1750507	256,184836	448,2226111	593,7903162	174,8364191	-0,617169289	False	0,617169289
ENSMUSG00000061461	Smim20	180,8442705	331,904492	277,3872741	278,9015122	336,6253443	0,61715371	False	0,61715371
ENSMUSG00000025209	Twnk	261,9923406	88,33959863	170,835337	123,7063159	65,23746983	-0,61691821	False	0,61691821
ENSMUSG00000031591	Asah1	527,848875	98,43555276	344,3124577	109,0864785	125,2559421	-0,61640656	False	0,61640656
ENSMUSG00000031848	Lsm4	221,8047249	618,3771904	339,9094851	447,5919429	424,0435539	0,615860522	False	0,615860522
ENSMUSG00000030102	Itpr1	531,7130688	1482,843263	490,4911481	1357,395666	347,0633395	-0,615448946	False	0,615448946
ENSMUSG00000029328	Hnrnpdl	916,5867727	1003,285442	613,774381	598,2887277	2728,230988	-0,615429585	False	0,615429585
ENSMUSG00000029859	Epha1	449,79216	689,0488693	509,8642276	148,4475791	268,7783757	0,615347792	False	0,615347792
ENSMUSG00000048218	Amigo2	139,1109773	687,786875	213,103874	311,5149954	574,0897345	0,615320552	False	0,615320552
ENSMUSG00000027332	Ivd	3072,806921	483,3438039	2005,994319	1214,571101	745,0119054	-0,615239599	False	0,615239599
ENSMUSG00000033287	Kctd17	47,14316453	176,6791973	72,20875071	232,7927944	199,6266577	0,615125078	False	0,615125078
ENSMUSG00000037443	Cep85	474,5230004	846,7981526	726,4904797	1111,107637	852,0013559	0,614465875	False	0,614465875
ENSMUSG00000032194	Kank2	731,8783084	145,1293406	478,1628248	70,84998091	110,9036987	-0,614101816	False	0,614101816
ENSMUSG00000002944	Cd36	724,1499207	473,2478498	243,9246823	340,7546701	341,8443419	-0,613692458	False	0,613692458
ENSMUSG00000029703	Lrwd1	81,14807009	201,9190826	124,1638274	292,3967466	193,1029107	0,613616246	False	0,613616246
ENSMUSG00000018474	Chd3	1993,924008	5267,564067	4410,897955	8175,862877	3050,504089	0,613437237	False	0,613437237
ENSMUSG00000069922	Ces3a	12703,92358	1843,773623	8304,006332	285,6491294	4207,816804	-0,613394691	False	0,613394691
ENSMUSG00000053754	Chd8	593,5401698	1414,695572	907,892951	2379,659676	1647,898488	0,613176528	False	0,613176528
ENSMUSG00000035632	Cnot3	427,3798358	1123,174497	653,4011345	1709,396365	1242,121425	0,612450118	False	0,612450118
ENSMUSG00000049164	Zfp518a	147,6122037	64,36170757	65,16399454	30,36427753	96,55145534	-0,612442089	False	0,612442089
ENSMUSG00000039759	Thap3	122,1085245	61,83771904	132,9697726	186,6840767	20,87599034	0,612434958	False	0,612434958
ENSMUSG00000052566	Hook2	209,4393047	444,2219817	384,8198056	852,4489767	136,9986866	-0,612370151	False	0,612370151
ENSMUSG00000106918	Mrpl33	115,1529757	137,557375	135,6115562	75,3483924	150,0461806	-0,611903056	False	0,611903056
ENSMUSG00000037720	Tmem33	1158,485305	395,0042053	551,2521701	239,5404117	758,0593994	-0,611856941	False	0,611856941
ENSMUSG00000043311	D17H6S53E	99,6962004	246,0888819	107,4325315	107,9618757	65,23746983	-0,611837692	False	0,611837692
ENSMUSG00000028861	Mrps15	459,8390639	320,5465436	702,7144276	242,9142203	294,8733636	0,611809489	False	0,611809489
ENSMUSG00000019471	Cdc37	1143,801369	478,2958269	748,5053427	439,7197228	537,5567514	-0,611752018	False	0,611752018
ENSMUSG00000042487	Leo1	237,2615002	99,69754702	113,5966932	58,47934932	155,2651782	-0,611743709	False	0,611743709
ENSMUSG00000029501	Ankle2	301,4071175	326,8565149	197,2531727	336,2562586	614,5369658	-0,61166498	False	0,61166498
ENSMUSG00000025858	Get4	167,7060115	690,3108636	256,2530056	571,2982588	999,4380377	0,611634522	False	0,611634522
ENSMUSG00000037416	Dmxl1	244,217049	166,5832431	170,835337	240,6650145	373,1583274	0,611623962	False	0,611623962
ENSMUSG00000049932	H2afx	108,1974268	165,3212489	250,9694384	175,438048	457,9670382	0,611605979	False	0,611605979
ENSMUSG00000024747	Aldh1a7	3219,646286	204,4430711	2108,143283	121,4571101	433,1767996	-0,610929276	False	0,610929276
ENSMUSG00000023022	Lima1	819,2090886	310,4505895	380,416833	536,4355698	142,2176842	-0,610826819	False	0,610826819
ENSMUSG00000026925	Inpp5e	231,8516288	220,8489966	353,9989974	193,4316939	80,89446258	0,610543412	False	0,610543412
ENSMUSG00000015337	Endog	477,6143554	401,3141766	729,1322633	217,0483542	341,8443419	0,610334341	False	0,610334341
ENSMUSG00000027206	Cops2	935,9077418	663,808984	427,0883426	455,464163	1428,700589	0,610265378	False	0,610265378
ENSMUSG00000049764	Zfp280b	121,3356858	79,50563877	125,9250165	116,9586986	170,9221709	-0,609874831	False	0,609874831
ENSMUSG00000059890	Ube4a	860,169543	445,4839759	486,0881755	290,1475409	563,6517393	-0,609817002	False	0,609817002
ENSMUSG00000032041	Tirap	487,6612593	1014,64339	744,1023701	862,5704025	887,2295896	0,609621748	False	0,609621748
ENSMUSG00000031751	Amfr	2705,708509	914,9458429	1773,517365	864,8196083	1012,485532	-0,609392967	False	0,609392967
ENSMUSG00000027423	Snx5	608,9969451	389,9562282	361,0437536	396,9848137	399,2533153	-0,609130604	False	0,609130604
ENSMUSG00000014551	Mrps25	146,0665262	92,12558143	222,7904138	59,6039522	62,62797103	0,609061562	False	0,609061562
ENSMUSG00000032570	Atp2c1	471,4316453	709,2407776	309,0886768	942,4172064	669,3364404	-0,609027801	False	0,609027801
ENSMUSG00000025810	Nrp1	442,8366111	2732,217586	675,4159975	982,9029098	4513,128163	0,60900185	False	0,60900185
ENSMUSG00000022684	Bfar	350,8687983	716,8127432	554,7745481	2277,320815	534,9472526	0,608464985	False	0,608464985
ENSMUSG00000038416	Cdc16	260,4466631	280,1627271	170,835337	404,8570338	512,7665128	-0,608381528	False	0,608381528
ENSMUSG00000002329	Mdp1	135,2467835	112,3174897	266,8201398	150,6967848	88,72295896	-0,608214897	False	0,608214897
ENSMUSG00000021179	Nrde2	127,5183959	191,8231285	83,65647948	220,4221628	429,2625515	-0,608156195	False	0,608156195
ENSMUSG00000070972	Dnajc25	322,2737641	437,9120103	362,8049426	211,4253399	711,0884211	-0,608138447	False	0,608138447
ENSMUSG00000027359	Slc27a2	9270,20096	3018,690285	6082,266356	4495,037678	4181,721816	-0,607991618	False	0,607991618
ENSMUSG00000032897	Nfyc	315,3182152	403,8381652	415,6406138	206,9269284	696,7361777	-0,607687112	False	0,607687112
ENSMUSG00000038859	Baiap2l1	472,2044841	504,7977065	309,9692713	480,2054262	908,10558	-0,60728654	False	0,60728654
ENSMUSG00000027011	Ube2e3	316,091054	316,7605608	184,9248494	184,4348709	481,4525273	0,607053346	False	0,607053346
ENSMUSG00000029404	Arl6ip4	231,8516288	523,7276204	434,1330988	353,1253017	383,5963226	0,606978333	False	0,606978333
ENSMUSG00000048445	Ccdc57	80,37523133	218,325008	122,4026384	223,7959715	326,1873491	0,606811766	False	0,606811766
ENSMUSG00000016541	Atxn10	662,3228197	291,5206755	435,0136933	366,6205361	147,4366818	-0,606473751	False	0,606473751
ENSMUSG00000044018	Mrpl50	636,8191405	383,6462569	418,2823974	152,9459905	369,2440792	-0,606406413	False	0,606406413
ENSMUSG00000020877	Scrn2	483,0242268	167,8452374	735,2964249	215,9237514	123,9511927	0,606230419	False	0,606230419
ENSMUSG00000090150	Acad11	3819,369166	533,8235746	1538,398628	2508,989007	473,6240309	-0,606228223	False	0,606228223
ENSMUSG00000028447	Dctn3	318,4095703	524,9896147	428,8495317	209,1761341	602,7942212	-0,606165442	False	0,606165442
ENSMUSG00000027613	Eif6	520,1204874	3358,166742	791,6544742	2268,323992	5650,869636	0,606025022	False	0,606025022
ENSMUSG00000023055	Calcoco1	554,8982317	501,0117237	268,5813289	316,0134069	844,1728595	0,605315241	False	0,605315241
ENSMUSG00000030909	Anks4b	1070,381686	623,4251675	703,5950222	218,1729571	187,8839131	-0,605308157	False	0,605308157
ENSMUSG00000032030	Cul5	221,0318862	262,4948074	145,2980959	174,3134451	588,4419778	-0,605238711	False	0,605238711
ENSMUSG00000025613	Cct8	1357,877706	1121,912903	1102,50434	675,8863258	2065,418295	0,605080437	False	0,605080437
ENSMUSG00000066154	Mup3	58813,80269	13131,05034	13440,51417	2729,411169	38666,24837	-0,605079981	False	0,605079981
ENSMUSG00000025006	Sorbs1	234,1701451	306,6646067	196,3725782	423,9752826	153,9604288	-0,604997561	False	0,604997561
ENSMUSG00000015890	Amdhd1	3303,112872	1230,444409	1528,712088	2172,732748	250,5118841	-0,604315539	False	0,604315539
ENSMUSG00000062397	Zfp706	983,823745	304,1406181	647,2369728	204,6777226	459,2717876	-0,604105853	False	0,604105853
ENSMUSG00000034771	Tle2	370,1897674	76,98165023	562,6998988	325,0102299	76,98021439	0,604100687	False	0,604100687
ENSMUSG00000028383	Hsdl2	1005,46323	531,299586	468,4762851	453,2149573	661,507944	-0,604029937	False	0,604029937
ENSMUSG00000029920	Smarcad1	164,6146565	228,4209622	108,3131261	203,5531198	147,4366818	-0,603884704	False	0,603884704
ENSMUSG00000079012	Serpina3m	6457,067863	9439,717111	4248,868563	12588,80454	5596,070162	-0,603800475	False	0,603800475
ENSMUSG00000014550	Rbsn	131,3825897	76,98165023	81,89529044	97,84044983	199,6266577	0,60353028	False	0,60353028

ENSMUSG00000028035	Dnajb4	367,8712511	99,69754702	242,1634932	112,4602872	93,94195655	-0,603219544	False	0,603219544
ENSMUSG00000021028	Mbip	64,91845608	112,3174897	69,56696715	42,73490912	258,3403805	-0,603213635	False	0,603213635
ENSMUSG00000028671	Gale	368,6440899	256,184836	1093,698395	1265,178231	242,6833878	-0,603153272	False	0,603153272
ENSMUSG00000021559	Dapk1	2050,341238	427,8160562	1349,9514	733,2410723	152,6556794	-0,602956567	False	0,602956567
ENSMUSG00000028223	Decr1	1846,311804	766,0305195	942,2361373	1215,695704	416,2150575	-0,602864056	False	0,602864056
ENSMUSG00000030287	Itpr2	498,481002	181,7271743	560,9387098	756,8577326	285,7401178	0,602483619	False	0,602483619
ENSMUSG00000030029	Lrig1	267,4022119	109,7935012	176,1189042	123,7063159	66,54221922	-0,602461627	False	0,602461627
ENSMUSG00000070576	Mn1	136,0196223	89,6015929	62,52221098	24,74126318	54,79947465	-0,602218505	False	0,602218505
ENSMUSG00000034744	Nagk	179,298593	89,6015929	158,5070138	272,1538949	18,26649155	0,602058517	False	0,602058517
ENSMUSG00000067071	Hes6	731,1054696	107,2695126	481,6852029	121,4571101	105,6847011	-0,601988939	False	0,601988939
ENSMUSG00000032555	Topbp1	113,6072981	212,0150367	74,85053427	247,4126318	305,3113588	-0,601970997	False	0,601970997
ENSMUSG00000029447	Cct6a	1887,272259	1569,920867	1468,831661	978,4044983	2863,924925	0,601691113	False	0,601691113
ENSMUSG00000011960	Ccnt1	231,0787901	418,9820964	350,4766193	910,928326	482,7572767	0,600933358	False	0,600933358
ENSMUSG00000030816	Rnf40	437,4267397	2011,61886	663,0876742	2272,822404	2715,183494	0,600158223	False	0,600158223
ENSMUSG00000013160	Atp6v0d1	886,4460609	937,6617397	943,9973264	584,7934932	1093,379994	-0,600105589	False	0,600105589
ENSMUSG00000040225	Prrc2c	703,2832742	665,0709783	476,4016358	543,183187	1065,980257	0,600002906	False	0,600002906
ENSMUSG00000063334	Krr1	279,7676321	198,1330998	208,7009014	184,4348709	424,0435539	0,599983394	False	0,599983394
ENSMUSG00000035151	Elmod2	189,3454969	124,9374323	132,9697726	159,6936078	388,8153202	-0,599815325	False	0,599815325
ENSMUSG00000011877	Git1	236,4886614	1105,506977	358,40197	827,7077135	1065,980257	0,599807553	False	0,599807553
ENSMUSG00000048782	Insc	154,5677526	975,5215677	234,2381425	747,8609096	1766,630683	0,599736657	False	0,599736657
ENSMUSG00000032193	Ldlr	2071,207884	979,3075505	3138,438872	897,4330916	391,424819	0,599574748	False	0,599574748
ENSMUSG00000032594	Ip6k1	479,9328717	316,7605608	670,1324304	1029,011628	348,3680889	-0,59943991	False	0,59943991
ENSMUSG00000050052	Tdrp	112,0616206	169,1072317	73,96993975	106,8372728	280,5211202	-0,599281256	False	0,599281256
ENSMUSG00000018906	P4ha2	68,00981113	111,0554954	103,0295589	106,8372728	161,7889252	0,599243512	False	0,599243512
ENSMUSG00000057672	Pkn1	428,9255134	755,9345654	669,2518359	1427,121044	649,7651995	0,599191344	False	0,599191344
ENSMUSG00000002741	Ykt6	608,2241063	560,3254542	401,5511015	430,7228998	1045,104267	-0,5990194	False	0,5990194
ENSMUSG00000034917	Tjp3	680,0981113	22,71589679	449,1032056	97,84044983	40,44723129	-0,598695865	False	0,598695865
ENSMUSG00000049686	Orai1	210,2121435	138,8193693	134,7309617	89,96822973	52,18997586	-0,598637134	False	0,598637134
ENSMUSG00000053774	Ubxn7	298,3157624	106,0075184	83,65647948	88,84362686	197,0171589	-0,598518925	False	0,598518925
ENSMUSG00000037674	Rfx7	336,1848618	521,2036319	513,3866057	548,8062014	508,8522646	0,597992096	False	0,597992096
ENSMUSG00000025872	Thoc3	291,3602136	296,5686525	211,342685	149,5721819	441,005296	0,597992096	False	0,597992096
ENSMUSG00000024730	Ms4a8a	38,64193814	172,8932145	25,5372411	630,902211	30,00923612	-0,597564783	False	0,597564783
ENSMUSG00000042271	Nxt2	434,3353847	146,3913349	103,0295589	65,22696655	287,0448672	-0,597533234	False	0,597533234
ENSMUSG00000032000	Birc3	294,4515686	411,4101308	194,6113891	545,4323927	387,5105708	-0,597434217	False	0,597434217
ENSMUSG00000018340	Anxa6	2272,145963	323,0705321	1502,294253	1098,737006	275,3021227	-0,596888097	False	0,596888097
ENSMUSG00000021711	Trappc13	274,3577608	141,3433578	181,4024713	94,46664122	99,16095413	-0,596864276	False	0,596864276
ENSMUSG00000045817	Zfp36l2	534,0315851	807,6763303	110,0743151	266,5308806	557,1279923	0,596852188	False	0,596852188
ENSMUSG00000012429	Mplkip	149,1578812	50,47977065	98,62658634	41,61030625	11,74274457	-0,596791704	False	0,596791704
ENSMUSG00000029291	Rufy3	764,3375364	206,9670596	505,461255	255,2848519	120,0369445	-0,596609373	False	0,596609373
ENSMUSG00000021012	Zc3h14	950,5916782	593,1373051	628,7444879	497,0744693	605,40372	-0,596351925	False	0,596351925
ENSMUSG00000036199	Ndufa13	1538,721977	1641,85454	2507,933195	1017,765599	1871,010635	-0,596327249	False	0,596327249
ENSMUSG00000039218	Srrm2	4977,854471	4299,614465	3204,483461	3207,36739	7525,794519	0,596319846	False	0,596319846
ENSMUSG00000057068	Fam47e	198,619562	6,309971331	300,2827316	3,373808615	9,133245776	0,596313791	False	0,596313791
ENSMUSG00000030741	Spns1	234,1701451	636,0451101	353,9989974	719,7458378	1069,894505	0,596188119	False	0,596188119
ENSMUSG00000001844	Zdhhc4	258,1281468	566,6354255	390,1033727	459,9625745	684,9934332	0,595769007	False	0,595769007
ENSMUSG00000075254	Heg1	125,1998796	307,9266009	201,6561453	411,604651	189,1886625	0,59559246	False	0,59559246
ENSMUSG00000020961	Ston2	153,022075	66,88569611	101,2683699	48,35792348	66,54221922	-0,595556158	False	0,595556158
ENSMUSG00000025439	Clns1a	309,1355051	200,6570883	239,5217097	105,7126699	467,100284	0,595492961	False	0,595492961
ENSMUSG00000024096	Ralbp1	390,2835752	766,0305195	579,4311947	1047,005273	258,3403805	-0,595249584	False	0,595249584
ENSMUSG00000095304	Plac9	135,2467835	116,1034725	204,2979288	138,3261532	67,84696862	0,595080295	False	0,595080295
ENSMUSG00000025225	Nfkb2	88,10361896	182,9891686	187,5666329	336,2562586	133,0844384	0,595068701	False	0,595068701
ENSMUSG00000058318	Phf21a	315,3182152	454,3179358	470,2374741	818,7108905	208,7599034	-0,594963874	False	0,594963874
ENSMUSG00000078813	Leng1	166,160334	88,33959863	250,9694384	53,98093784	87,41820957	0,594935671	False	0,594935671
ENSMUSG00000019368	Sec14l4	2670,930764	482,0818097	2466,545253	4033,9505	310,5303564	0,594850802	False	0,594850802
ENSMUSG00000029657	Hsph1	933,5892255	79,50563877	618,1773536	158,5690049	249,2071347	-0,594767108	False	0,594767108
ENSMUSG00000079507	H2-Q1	179,298593	65,62370184	88,94004661	40,48570338	118,7321951	-0,594652982	False	0,594652982
ENSMUSG00000060470	Adgrg3	64,14561731	638,5690987	96,86539729	1088,61558	161,7889252	0,594630694	False	0,594630694
ENSMUSG00000008301	Phax	241,125694	277,6387386	223,6710083	159,6936078	361,4155828	-0,594478825	False	0,594478825
ENSMUSG00000024866	Acy3	1668,558889	99,69754702	1105,146124	112,4602872	106,9894505	-0,594365468	False	0,594365468
ENSMUSG00000030509	Asb7	95,05916782	162,7972603	75,73112879	62,97776081	190,4934119	-0,593983309	False	0,593983309
ENSMUSG00000019539	Rcn3	112,0616206	46,69378785	169,074148	58,47934932	67,84696862	0,593363822	False	0,593363822
ENSMUSG00000022809	Nr1i2	986,1422613	1121,912903	1200,250332	1501,344834	653,6794477	-0,593212447	False	0,593212447
ENSMUSG00000030921	Trim30a	139,1109773	36,59783372	81,89529044	92,21743547	30,00923612	-0,593124817	False	0,593124817
ENSMUSG00000027259	Adal	170,0245278	34,07384519	112,7160987	67,4761723	28,70448672	-0,593049302	False	0,593049302
ENSMUSG00000009905	Kdsr	169,2516891	138,8193693	90,70123565	255,2848519	176,1411685	0,592937702	False	0,592937702
ENSMUSG00000002043	Trappc6a	155,3405913	95,91156423	234,2381425	133,8277417	84,80871077	0,592541156	False	0,592541156
ENSMUSG00000029192	Tbc1d14	197,8467233	177,9411915	131,2085836	301,3935696	212,6741516	-0,592521068	False	0,592521068
ENSMUSG00000027429	Sec23b	904,2213525	665,0709783	599,6848687	682,6339431	1841,001398	-0,592471415	False	0,592471415
ENSMUSG00000053477	Tcf4	252,7182754	478,2958269	452,6255837	881,6886513	380,9868238	0,592211107	False	0,592211107
ENSMUSG00000026670	Uap1	408,8317055	374,812297	271,2231124	344,1284787	1129,912977	-0,59202696	False	0,59202696
ENSMUSG00000020720	Psmd12	871,7621244	918,7318257	730,0128578	337,3808615	1313,882642	0,591829989	False	0,591829989
ENSMUSG00000036792	Mbd5	112,0616206	359,6683658	98,62658634	818,7108905	74,3707156	-0,591485703	False	0,591485703
ENSMUSG00000030779	Rbbp6	683,1894663	3283,70908	1029,414995	2128,873236	8231,663943	0,591467066	False	0,591467066
ENSMUSG00000030279	C2cd5	89,64929649	135,0333865	147,9398795	522,9403353	289,654366	0,590951985	False	0,590951985
ENSMUSG00000021277	Traf3	140,6566548	93,38757569	171,7159316	187,8086796	134,3891878	-0,590875281	False	0,590875281
ENSMUSG00000032079	Apoa5	15102,8151	20006,3951	10029,091	19054,14645	17015,23688	-0,590626636	False	0,590626636
ENSMUSG00000063229	Ldha	6671,144201	21109,37809	10045,82229	19399,39954	23655,10656	0,590589528	False	0,590589528
ENSMUSG00000036333	Kidins220	682,4166276	403,8381652	416,5212084	453,2149573	324,8825997	-0,590457325	False	0,590457325
ENSMUSG00000074785	Plxnc1	186,2541418	40,38381652	83,65647948	123,7063159	23,48548914	-0,590353348	False	0,590353348
ENSMUSG00000000673	Haao	9771,773317	1760,482001	6490,862213	3273,718959	1299,530399	-0,590210265	False	0,590210265
ENSMUSG00000037805	Rpl10a	1075,791558	2070,932591	1619,413324	1688,02891	2373,339152	0,59007268	False	0,59007268
ENSMUSG00000030884	Uqcrc2	3208,826543	1761,743996	1954,039242	642,1482397	2131,960514	-0,589865087	False	0,589865087
ENSMUSG00000021840	Mapk1ip1l	427,3798358	326,8565149	219,2680357	122,581713	643,2414525	0,589841539	False	0,589841539
ENSMUSG00000000881	Dlg3	311,4540214	59,31373051	206,9397124	163,0674164	65,23746983	-0,589808671	False	0,589808671
ENSMUSG00000055447	Cd47	704,8289517	1185,012616	1090,176017	1252,807599	1060,761259	0,589754901	False	0,589754901
ENSMUSG00000024896	Minpp1	243,4442103	159,0112775	133,8503672	116,9586986	161,7889252	-0,589478335	False	0,589478335
ENSMUSG00000011658	Fuz	161,5233014	16,40592546	243,0440878	55,10554071	18,26649155	0,589475736	False	0,589475736
ENSMUSG00000027809	Etfdh	3101,401955	1453,817395	674,535403	2061,397064	1996,266577	-0,589298096	False	0,589298096
ENSMUSG00000034663	Bmp2k	83,46658638	246,0888819	55,47745481	160,8182106	460,576537	-0,589297167	False	0,589297167
ENSMUSG00000039967	Zfp292	465,2489352	243,5648934	227,1933864	309,2657897	267,4736263	-0,589155594	False	0,589155594
ENSMUSG00000021754	Map3k1	156,8862688	181,7271743	235,9993316	148,4475791	19,57124095	0,589063685	False	0,589063685
ENSMUSG00000060373	Hnrnpc	1552,633074	922,5178085	1461,786905	891,8100772	2335,50142	0,589015397	False	0,589015397
ENSMUSG00000005354	Txn2	2449,898878	1952,30513	3684,407475	1250,558393	2544,261323	0,588710423	False	0,588710423
ENSMUSG00000036099	Vezt	229,5331126	320,5465436	225,4321973	293,5213495	152,6556794	-0,588421028	False	0,588421028
ENSMUSG00000020936	Nmt1	1122,161884	2243,825805	1687,219102	1937,690748	2970,914376	0,588366519	False	0,588366519
ENSMUSG00000036966	Spryd3	241,8985328	424,0300734	363,6855371	566,7998473	600,1847224	0,588289536	False	0,588289536
ENSMUSG00000027998	Plrg1	313,7725377	1288,496146	208,7009014	356,4991103	1332,149134	-0,588282257	False	0,588282257
ENSMUSG00000021000	Mia2	4552,020313	2241,301817	1986,621239	1797,115389	3028,323349	-0,587987738	False	0,587987738
ENSMUSG00000063253	Scoc	119,7900082	102,2215356	112,7160987	58,47934932	180,0554167	0,587933425	False	0,587933425
ENSMUSG00000040413	Timd2	2126,852275	596,9232879	1415,115395	412,7292539	327,4920985	-0,58780013	False	0,58780013
ENSMUSG00000030232	Aebp2	183,9356255	324,3325264	122,4026384	134,9523446	459,2717876	-0,587566279	False	0,587566279
ENSMUSG00000056076	Eif3b	1168,532209	3283,70908	1755,905475	4888,648683	4147,798332	0,587517682	False	0,587517682
ENSMUSG00000024892	Pcx	5342,634367	2440,696911	3555,840675	2460,631083	2437,271873	-0,587360604	False	0,587360604
ENSMUSG00000043140	Tmem186	109,7431043	160,2732718	162,9099864	106,8372728	73,0659662	-0,586858824	False	0,586858824
ENSMUSG00000024975	Pdcd4	1328,509833	755,9345654	415,6406138	545,4323927	884,6200908	-0,586678993	False	0,586678993
ENSMUSG00000047394	Odf3b	238,0343389	27,76387386	158,5070138	87,71902399	18,26649155	-0,586623033	False	0,586623033
ENSMUSG00000040660	Cyp2b9	7432,390382	378,5982798	4947,180018	4949,377238	523,204508	-0,586579273	False	0,586579273
ENSMUSG00000000093	Tbx2	82,69374762	742,0526285	124,1638274	638,7744311	482,7572767	0,586394777	False	0,586394777
ENSMUSG00000028412	Slc44a1	816,8905723	360,9303601	430,6107207	293,5213495	544,0804983	-0,586322715	False	0,586322715
ENSMUSG00000032064	Dixdc1	466,021774	58,05173624	277,3872741	310,3903926	32,61873491	-0,586313461	False	0,586313461
ENSMUSG00000010067	Rassf1	65,69129484	537,6095574	98,62658634	377,8665649	752,8404018	0,586274397	False	0,586274397
ENSMUSG00000032735	Ablim3	414,2415769	268,8047787	298,5215426	621,905388	93,94195655	-0,586222754	False	0,586222754
ENSMUSG00000000148	Brat1	142,9751711	199,395094	227,1933864	255,2848519	95,24670595	-0,586023528	False	0,586023528

ENSMUSG00000038604	Ripor1	160,7504627	329,3805035	241,2828987	404,8570338	320,9683515	0,585902776	False	0,585902776
ENSMUSG00000031488	Rab11fip1	125,1998796	50,47977065	76,61172331	41,61030625	187,8839131	0,585608372	False	0,585608372
ENSMUSG00000024242	Map4k3	418,8786094	336,9524691	417,4018029	680,3847373	279,2163709	-0,585148682	False	0,585148682
ENSMUSG00000027006	Dnajc10	246,5355653	437,9120103	369,8496988	566,7998473	413,6055587	0,585143315	False	0,585143315
ENSMUSG00000047735	Samd9l	353,9601534	132,5093979	235,9993316	159,6936078	46,97097827	-0,584804186	False	0,584804186
ENSMUSG00000026142	Rhbdd1	278,2219546	555,2774771	214,8650631	185,5594738	675,8601874	-0,584354608	False	0,584354608
ENSMUSG00000039795	Zfand1	275,9034383	56,78974198	184,0442549	114,7094929	46,97097827	-0,584110721	False	0,584110721
ENSMUSG00000035202	Lars2	651,503077	3313,996943	976,5793236	3078,03806	2086,294285	0,583965241	False	0,583965241
ENSMUSG00000028673	Fuca1	885,6732222	471,9858555	590,8789235	389,1125936	386,2058214	-0,583911961	False	0,583911961
ENSMUSG00000005320	Fgfr4	688,5993377	2009,094872	1032,056778	1163,963972	3661,126807	0,583785644	False	0,583785644
ENSMUSG00000030987	Stim1	490,7526144	294,044664	330,2229453	735,490278	190,4934119	0,583710318	False	0,583710318
ENSMUSG00000042737	Dpm3	195,528207	237,254922	533,6402796	151,8213877	130,4749397	-0,583604012	False	0,583604012
ENSMUSG00000058799	Nap1l1	613,6339777	773,6024851	409,4764522	596,039522	678,4696862	-0,58359788	False	0,58359788
ENSMUSG00000022149	C9	3577,470633	719,3367317	2387,291746	385,738785	1877,534382	-0,583565038	False	0,583565038
ENSMUSG00000018774	Cd68	205,5751109	285,2107041	205,1785234	137,2015503	551,9089947	-0,583368824	False	0,583368824
ENSMUSG00000016481	Cr1l	564,9451356	315,4985665	273,864896	159,6936078	377,0725756	-0,58326854	False	0,58326854
ENSMUSG00000044786	Zfp36	4780,780587	1737,766104	1452,980959	537,5601726	3191,417024	-0,583049056	False	0,583049056
ENSMUSG00000048897	Zfp710	191,6640132	157,7492833	287,0738138	97,84044983	112,2084481	0,582842255	False	0,582842255
ENSMUSG00000033487	Fndc3a	1007,008908	436,6500161	665,7294578	1508,092451	805,0303776	0,582648428	False	0,582648428
ENSMUSG00000026491	Ahctf1	1176,260597	502,2737179	407,7152632	529,6879525	785,4591367	-0,582599594	False	0,582599594
ENSMUSG00000015176	Nolc1	255,8096305	668,8569611	383,0586166	565,6752444	1123,38923	0,582494595	False	0,582494595
ENSMUSG00000074063	Osgin1	4268,388487	2031,810768	6390,474438	2628,196911	1339,97763	0,582231554	False	0,582231554
ENSMUSG00000021208	Ifi27l2b	107,424588	199,395094	532,7596851	191,1824882	71,76121681	-0,582047986	False	0,582047986
ENSMUSG00000018927	Ccl6	304,4984725	365,9783372	203,4173343	620,7807851	334,0158455	-0,581992367	False	0,581992367
ENSMUSG00000016520	Lnx2	237,2615002	475,7718383	158,5070138	206,9269284	906,8008306	-0,581931338	False	0,581931338
ENSMUSG00000034641	Cd300ld	147,6122037	29,02586812	98,62658634	62,97776081	30,00923612	-0,581763495	False	0,581763495
ENSMUSG00000002345	Borcs8	153,022075	111,0554954	228,9545754	79,84680388	54,79947465	0,581321604	False	0,581321604
ENSMUSG00000034801	Sos2	196,3010458	321,8085379	131,2085836	267,6554834	644,5462019	-0,581205755	False	0,581205755
ENSMUSG00000026270	Capn10	418,8786094	218,325008	280,0290576	199,0547083	92,63720715	-0,580955675	False	0,580955675
ENSMUSG00000030431	Tmem238	285,9503422	305,4026124	508,9836331	191,1824882	249,2071347	-0,58081425	False	0,58081425
ENSMUSG00000051557	Pusl1	78,05671504	126,1994266	178,7606877	142,8245647	52,18997586	-0,580750015	False	0,580750015
ENSMUSG00000026942	Traf2	123,654202	271,3287672	184,9248494	356,4991103	240,073889	0,580627834	False	0,580627834
ENSMUSG00000033285	Wdr3	164,6146565	194,347117	110,0743151	215,9237514	309,225607	-0,580614924	False	0,580614924
ENSMUSG00000001829	Clpb	506,2093896	603,2332592	638,4310276	338,5054644	460,576537	-0,580555145	False	0,580555145
ENSMUSG00000036138	Acaa1a	1147,665563	528,7755975	1512,861387	1716,143982	426,6530527	0,580468306	False	0,580468306
ENSMUSG00000026977	March7	449,0193212	440,4359989	300,2827316	407,1062395	1274,74016	-0,580456013	False	0,580456013
ENSMUSG00000034108	Ccs	1910,457422	634,7831159	2856,648626	825,4585078	606,7084694	0,580405488	False	0,580405488
ENSMUSG00000003762	Coq8b	170,7973666	3806,174707	255,372411	2235,710509	2301,577935	0,580316942	False	0,580316942
ENSMUSG00000035011	Zbtb7a	571,9006845	1121,912903	855,0572798	999,7719529	874,1820957	0,580256436	False	0,580256436
ENSMUSG00000022106	Rcbtb2	513,1649385	343,2624404	307,3274878	263,157072	285,7401178	-0,580110596	False	0,580110596
ENSMUSG00000008435	Rdh13	59,50858474	391,2182225	88,94004661	745,6117039	176,1411685	0,579735353	False	0,579735353
ENSMUSG00000022575	Gsdmd	571,1278457	193,0851227	686,8637263	853,5735796	183,9696649	0,579701796	False	0,579701796
ENSMUSG00000068882	Ssb	792,1597319	879,6100035	530,1179016	596,039522	1574,832522	-0,579478106	False	0,579478106
ENSMUSG00000060550	H2-Q7	261,2195018	102,2215356	120,6414494	73,09918666	174,8364191	-0,579256873	False	0,579256873
ENSMUSG00000033533	Acsm1	6127,065712	971,7355849	4100,928684	1038,008451	1720,964454	-0,579245669	False	0,579245669
ENSMUSG00000024622	Hmgxb3	149,1578812	387,4322397	222,7904138	571,2982588	293,5686142	0,578846949	False	0,578846949
ENSMUSG00000029648	Flt1	114,3801369	347,0484232	170,835337	642,1482397	198,3219083	0,578769888	False	0,578769888
ENSMUSG00000024829	Mrpl21	180,8442705	171,6312202	140,8951233	80,97140676	270,0831251	0,578655611	False	0,578655611
ENSMUSG00000057110	Cntrl	293,6787299	1188,798599	438,5360714	987,4013213	1577,44202	0,578456735	False	0,578456735
ENSMUSG00000038507	Parp12	457,5205476	106,0075184	306,4468933	179,9364595	161,7889252	-0,578199458	False	0,578199458
ENSMUSG00000024759	Atl3	1428,978872	363,4543486	957,2062442	522,9403353	177,4459179	-0,578082873	False	0,578082873
ENSMUSG00000066900	Suds3	1007,008908	460,6279071	493,1329317	468,9593975	674,555438	-0,578067525	False	0,578067525
ENSMUSG00000009630	Ppp2cb	311,4540214	778,6504622	208,7009014	377,8665649	1312,577893	-0,577582368	False	0,577582368
ENSMUSG00000036646	Man1b1	425,8341583	280,1627271	369,8496988	635,4006225	221,8073974	0,577374823	False	0,577374823
ENSMUSG00000019256	Ahr	669,2783686	479,5578211	998,5941867	499,323675	403,1675635	0,577292122	False	0,577292122
ENSMUSG00000002733	Plekha3	223,3504025	210,7530424	149,7010685	173,1888422	396,6438165	-0,577224335	False	0,577224335
ENSMUSG00000024695	Zfp91	810,7078622	793,7943934	510,7448221	516,1927181	1209,502691	0,577159939	False	0,577159939
ENSMUSG00000024446	Rpp21	153,022075	691,5728578	271,2231124	228,2943829	2011,923569	0,577155571	False	0,577155571
ENSMUSG00000042148	Cox10	214,8491761	478,2958269	338,148296	320,5118184	536,252002	0,577053311	False	0,577053311
ENSMUSG00000023007	Prpf40b	237,2615002	598,1852821	658,6847016	1578,942432	159,1794264	-0,575824135	False	0,575824135
ENSMUSG00000015869	Prpsap1	541,7599727	311,7125837	807,5051756	730,9918666	290,9591154	0,575817701	False	0,575817701
ENSMUSG00000029427	Zcchc8	282,8589872	421,5060849	460,5509344	581,4196846	447,529043	0,575470448	False	0,575470448
ENSMUSG00000030432	Rpl28	3642,389089	9417,001214	9787,808099	5427,333458	8564,375039	0,575358509	False	0,575358509
ENSMUSG00000029030	Tprgl	654,5944321	276,3767443	439,4166659	313,7642012	294,8733636	-0,575011743	False	0,575011743
ENSMUSG00000024713	Pcsk5	299,0886012	1297,330106	200,7755508	380,1157706	746,3166548	-0,574989331	False	0,574989331
ENSMUSG00000070427	Il18bp	204,0294334	321,8085379	449,1032056	277,7769093	136,9986866	-0,574615229	False	0,574615229
ENSMUSG00000032561	Acpp	300,6342787	201,9190826	63,4028055	140,575359	105,6847011	-0,574232256	False	0,574232256
ENSMUSG00000031790	Mmp15	440,5180948	10,09595413	295,879759	34,86268902	19,57124095	-0,574190275	False	0,574190275
ENSMUSG00000042700	Sipa1l1	500,7995183	937,6617397	336,387107	817,5862877	859,8298523	-0,574110763	False	0,574110763
ENSMUSG00000042831	Alkbh6	85,01226391	215,8010195	140,8951233	133,8277417	126,5606915	0,574086502	False	0,574086502
ENSMUSG00000006731	B4galnt1	1691,744052	1706,216248	2115,188039	2654,062777	1136,436724	-0,573993957	False	0,573993957
ENSMUSG00000089832	Shkbp1	130,6097509	367,2403314	282,6708412	320,5118184	194,4076601	0,573822457	False	0,573822457
ENSMUSG00000021027	Ralgapa1	130,6097509	291,5206755	202,5367398	472,3332061	194,4076601	0,573822457	False	0,573822457
ENSMUSG00000060613	Cyp2c70	11920,26508	3134,793757	3898,391944	617,4069765	8008,551796	-0,573803033	False	0,573803033
ENSMUSG00000032330	Cox7a2	1069,608848	1287,234151	1802,576984	718,621235	1461,319324	-0,573779832	False	0,573779832
ENSMUSG00000015575	Atp6v0e	371,7354449	344,5244347	298,5215426	136,0769475	553,2137441	0,573560744	False	0,573560744
ENSMUSG00000026803	Ttf1	171,5702053	126,1994266	178,7606877	255,2848519	43,05673008	0,573308895	False	0,573308895
ENSMUSG00000042354	Gnl3	224,89608	913,6838487	334,6259179	708,4998091	1670,079228	0,573290677	False	0,573290677
ENSMUSG00000039191	Rbpj	163,068979	73,19566744	80,1341014	77,59759814	109,5989493	-0,573248393	False	0,573248393
ENSMUSG00000028221	Tmem55a	214,8491761	75,71965597	144,4175014	35,98729189	96,55145534	-0,573078657	False	0,573078657
ENSMUSG00000048175	Asb8	201,7109171	243,5648934	135,6115562	227,1697801	318,3588527	-0,572809045	False	0,572809045
ENSMUSG00000036057	Ptpn23	387,1922202	2028,024786	575,9088166	1261,804422	5008,932933	0,572790444	False	0,572790444
ENSMUSG00000026150	Mff	411,1502218	569,159414	276,5066795	359,8729189	784,1543873	-0,572351275	False	0,572351275
ENSMUSG00000006273	Atp6v1b2	522,4390037	575,4693854	351,3572138	598,2887277	1244,730924	-0,572324089	False	0,572324089
ENSMUSG00000022890	Atp5j	1962,237619	1792,031858	2917,409648	690,5061632	1891,886625	0,57218822	False	0,57218822
ENSMUSG00000044340	Phlpp1	292,9058911	135,0333865	148,820474	145,0737704	197,0171589	-0,572115926	False	0,572115926
ENSMUSG00000028334	Nans	251,1725979	989,4035047	538,0432522	373,3681534	1192,540948	0,571919798	False	0,571919798
ENSMUSG00000030298	Sec13	493,0711307	981,8315391	757,3112879	331,7578471	1409,129348	-0,571665195	False	0,571665195
ENSMUSG00000002504	Slc9a3r2	326,9107967	222,1109908	451,7449892	485,8284405	60,01847224	0,571549923	False	0,571549923
ENSMUSG00000020109	Dnajb12	868,6707694	432,8640333	584,7147618	272,1538949	434,481549	-0,571076478	False	0,571076478
ENSMUSG00000038648	Creb3l2	476,0686779	550,2295	320,5364056	374,4927562	1019,009279	-0,570681489	False	0,570681489
ENSMUSG00000040447	Spns2	670,0512074	118,627461	995,0718086	264,2816748	39,1424819	0,570529285	False	0,570529285
ENSMUSG00000000823	Zfp512b	408,8317055	498,4877351	448,2226111	843,4521537	275,3021227	-0,57049135	False	0,57049135
ENSMUSG00000029196	Tada2b	238,8071777	300,3546353	253,611222	160,8182106	374,4630768	-0,570415417	False	0,570415417
ENSMUSG00000067942	Zfp160	113,6072981	55,52774771	98,62658634	168,6904307	14,35224336	0,57032262	False	0,57032262
ENSMUSG00000031438	Rnf128	1315,371574	359,6683658	885,878088	357,6237132	688,9076814	-0,570290321	False	0,570290321
ENSMUSG00000039275	Foxk2	217,9405311	368,5023257	440,2972604	645,5220483	323,5778503	0,570178331	False	0,570178331
ENSMUSG00000032267	Usp28	85,01226391	126,1994266	135,6115562	156,3197992	134,3891878	0,56996247	False	0,56996247
ENSMUSG00000027284	Cdan1	84,23942515	247,3508762	125,044422	186,6840767	260,9498793	0,569873206	False	0,569873206
ENSMUSG00000025968	Ndufs1	1296,823444	707,9787833	747,6247482	873,8164312	786,7638861	-0,569579938	False	0,569579938
ENSMUSG00000031197	Vbp1	121,3356858	78,2436445	62,52221098	35,98729189	180,0554167	0,569437081	False	0,569437081
ENSMUSG00000031796	Cfap20	342,3675719	357,1443773	230,7157645	455,464163	468,4050333	-0,569429476	False	0,569429476
ENSMUSG00000030942	Thumpd1	172,3430441	122,4134438	98,62658634	47,23332061	255,7308817	0,569343317	False	0,569343317
ENSMUSG00000034617	Mtrr	74,19252123	235,9929278	110,0743151	262,0324691	198,3219083	0,569132195	False	0,569132195
ENSMUSG00000023232	Serinc2	115,1529757	90,86358716	170,835337	131,578536	52,18997586	0,569054733	False	0,569054733
ENSMUSG00000022708	Zbtb20	1630,68979	5041,667093	3803,287736	4444,430549	2419,005381	0,568931618	False	0,568931618
ENSMUSG00000031958	Ldhd	1933,642585	1303,640077	3118,185198	3804,531515	1493,938059	-0,568775541	False	0,568775541
ENSMUSG00000007833	Aldh16a1	737,2881797	614,5912076	1072,564126	861,4457997	497,1095201	-0,568664895	False	0,568664895
ENSMUSG00000018932	Map2k3	603,5870737	527,5136032	615,5355701	407,1062395	919,8483245	-0,568156577	False	0,568156577
ENSMUSG00000020393	Kremen1	152,2492363	135,0333865	116,2384768	82,09600963	225,7216456	0,568109781	False	0,568109781
ENSMUSG00000021549	Rasa1	365,5527348	601,9712649	246,5664658	462,2117802	660,2031946	-0,568102945	False	0,568102945
ENSMUSG00000041718	Alg13	71,87400494	199,395094	106,551937	238,4158088	301,3971106	0,568014838	False	0,568014838
ENSMUSG00000041757	Plekha6	1009,327424	344,5244347	1496,130091	960,4108524	95,24670595	0,567841367	False	0,567841367

ENSMUSG00000056153	Socs6	170,0245278	358,4063716	117,1190713	114,7094929	731,9644114	-0,567758097	False	0,567758097
ENSMUSG00000055148	Klf2	118,2443307	388,694234	175,2383097	383,4895792	776,3258909	0,567547189	False	0,567547189
ENSMUSG00000037656	Slc20a2	863,2608981	473,2478498	474,6404467	582,5442875	323,5778503	-0,567428908	False	0,567428908
ENSMUSG00000040396	Abhd13	159,9776239	194,347117	109,1937206	107,9618757	374,4630768	-0,567348182	False	0,567348182
ENSMUSG00000040843	Tiprl	263,5380181	285,2107041	177,8800932	200,1793111	497,1095201	-0,567106035	False	0,567106035
ENSMUSG00000020422	Tns3	476,0686779	171,6312202	321,4170001	136,0769475	161,7889252	-0,566723476	False	0,566723476
ENSMUSG00000040612	Ildr2	987,6879389	422,7680792	457,0285563	666,8895029	223,1121468	-0,566607552	False	0,566607552
ENSMUSG00000031328	Flna	412,6958993	921,2558143	611,1325975	1724,016202	938,1148161	0,566406332	False	0,566406332
ENSMUSG00000031221	Igbp1	656,9129484	286,4726984	228,0739809	185,5594738	443,6147948	-0,566394722	False	0,566394722
ENSMUSG00000030339	Ltbr	915,8139339	2281,685633	1356,115562	1439,491676	1509,595052	0,566353703	False	0,566353703
ENSMUSG00000002279	Lmf1	728,7869533	492,1777638	1606,204406	854,6981824	508,8522646	-0,566317653	False	0,566317653
ENSMUSG00000002845	Tmem39a	188,5726581	338,2144633	279,1484631	719,7458378	1364,767869	0,565912104	False	0,565912104
ENSMUSG00000035027	Map2k2	1371,788804	2520,202549	2030,650965	2236,835112	2292,44469	0,5658839	False	0,5658839
ENSMUSG00000038299	Wdr36	319,9552478	249,8748647	145,2980959	157,444402	473,6240309	0,565872154	False	0,565872154
ENSMUSG00000040771	Oard1	220,2590474	141,3433578	148,820474	55,10554071	117,4274457	-0,565628261	False	0,565628261
ENSMUSG00000020612	Prkar1a	1403,475193	1342,761899	833,0424167	666,8895029	2077,161039	0,565609506	False	0,565609506
ENSMUSG00000042289	Hsd3b7	132,9282672	44,16979931	89,82064113	7,872220101	52,18997586	-0,565529001	False	0,565529001
ENSMUSG00000030872	Gga2	227,987435	93,38757569	113,5966932	154,0705934	130,4749397	-0,565362787	False	0,565362787
ENSMUSG00000016619	Nup50	364,779896	642,3550815	246,5664658	328,3840385	1034,666271	-0,565049621	False	0,565049621
ENSMUSG00000057778	Cyb5d2	182,389948	55,52774771	123,2832329	44,98411486	40,44723129	-0,565049621	False	0,565049621
ENSMUSG00000020553	Pctp	1635,326822	276,3767443	1035,579157	1105,484623	547,9947465	-0,564900031	False	0,564900031
ENSMUSG00000050390	C77080	938,9990968	634,7831159	322,2975946	191,1824882	510,157014	-0,564860015	False	0,564860015
ENSMUSG00000079418	Atg4a	115,9258144	129,9854094	78,37291236	172,0642394	100,4657035	-0,564774848	False	0,564774848
ENSMUSG00000033220	Rac2	153,022075	170,3692259	133,8503672	103,4634642	229,6358938	-0,56461839	False	0,56461839
ENSMUSG00000025764	Jade1	378,6909938	706,716789	560,0581153	673,6371201	803,7256283	0,564555428	False	0,564555428
ENSMUSG00000037461	Ints7	193,2096907	331,904492	383,9392111	485,8284405	285,7401178	0,564536148	False	0,564536148
ENSMUSG00000003380	Rabac1	430,4711909	1008,333419	759,9530715	636,5252253	1385,643859	0,564301	False	0,564301
ENSMUSG00000001056	Nhp2	191,6640132	528,7755975	420,924181	283,3999236	862,4393511	0,564259887	False	0,564259887
ENSMUSG00000015748	Prpf3	187,0269806	372,2883085	276,5066795	423,9752826	514,0712622	0,564067923	False	0,564067923
ENSMUSG00000072258	Taf1a	85,78510267	164,0592546	126,805611	233,9173973	309,225607	0,563819546	False	0,563819546
ENSMUSG00000058503	Fam133b	163,8418177	738,2666457	242,1634932	949,1648236	1042,494768	0,563677765	False	0,563677765
ENSMUSG00000047793	Sned1	504,6637121	170,3692259	745,8635592	484,7038377	113,5131975	0,563589389	False	0,563589389
ENSMUSG00000046447	Camk2n1	1079,655752	389,9562282	1595,637272	565,6752444	204,8456553	0,563561346	False	0,563561346
ENSMUSG00000025139	Tollip	638,3648181	478,2958269	305,5662987	251,9110432	943,3338137	0,563387239	False	0,563387239
ENSMUSG00000069089	Cdk7	127,5183959	148,9153234	86,29826304	308,1411868	206,1504046	-0,563301959	False	0,563301959
ENSMUSG00000008393	Carhsp1	1220,312406	1474,009303	655,1623235	449,8411486	1803,163666	0,563279818	False	0,563279818
ENSMUSG00000025921	Rdh10	415,0144156	194,347117	192,8502001	67,4761723	613,2322164	0,563272042	False	0,563272042
ENSMUSG00000073664	Nbeal1	203,2565946	137,557375	123,2832329	132,7031389	101,7704529	-0,563268671	False	0,563268671
ENSMUSG00000022433	Csnk1e	119,7900082	406,3621537	176,9994987	482,4546319	390,1200696	0,563237697	False	0,563237697
ENSMUSG00000009995	Taz	453,6563538	217,0630138	474,6404467	670,2633115	44,36147948	0,563128102	False	0,563128102
ENSMUSG00000031700	Gpt2	2930,604589	2320,807455	1899,442381	4329,721056	2172,407745	0,563075756	False	0,563075756
ENSMUSG00000058569	Tmed9	687,8264989	1171,130679	643,7145947	465,5855889	2439,881371	-0,562998294	False	0,562998294
ENSMUSG00000004768	Rab23	163,8418177	90,86358716	98,62658634	69,72537804	110,9036987	-0,562996145	False	0,562996145
ENSMUSG00000040327	Cul9	98,92336164	381,1222684	197,2531727	464,460986	146,1319324	0,562888295	False	0,562888295
ENSMUSG00000048154	Kmt2d	621,3623653	1415,957567	1149,17585	917,6759432	2064,113545	0,562549925	False	0,562549925
ENSMUSG00000038736	Nudcd1	217,1676923	234,7309335	147,059285	241,7896174	357,5013346	-0,562411616	False	0,562411616
ENSMUSG00000041881	Ndufa7	1084,292784	1505,55916	1877,427518	734,3656752	1552,651782	-0,562183838	False	0,562183838
ENSMUSG00000018900	Slc22a5	200,1652396	666,3329725	135,6115562	1549,702757	558,4327417	-0,561711336	False	0,561711336
ENSMUSG00000009030	Pdcl	264,3108569	132,5093979	140,0145288	147,3229762	390,1200696	0,561682532	False	0,561682532
ENSMUSG00000002379	Ndufa11	731,8783084	1073,95712	1368,443885	495,9498664	865,0488499	-0,561409496	False	0,561409496
ENSMUSG00000046805	Mpeg1	501,5723571	671,3809496	339,9094851	427,3490912	811,5541246	-0,561307219	False	0,561307219
ENSMUSG00000029684	Wasl	1081,974268	1222,872444	881,4751154	733,2410723	1801,858917	-0,561306683	False	0,561306683
ENSMUSG00000068086	Cyp2d9	288,2688585	1291,020134	2019,203236	2596,708031	425,3483033	0,561229707	False	0,561229707
ENSMUSG00000026199	Ankzf1	400,3304791	401,3141766	458,7897454	1400,130575	271,3878745	-0,560835196	False	0,560835196
ENSMUSG00000025159	Mms19	385,6465426	893,4919404	568,8640605	1301,165522	1060,761259	0,560804761	False	0,560804761
ENSMUSG00000035069	Oma1	215,6220148	45,43179358	146,1786905	67,4761723	18,26649155	-0,560771469	False	0,560771469
ENSMUSG00000028557	Rnf11	995,4163265	1181,226633	522,1925509	612,908565	1467,843071	0,560325781	False	0,560325781
ENSMUSG00000003437	Paf1	357,8243472	469,461867	332,8647289	443,0935314	242,6833878	-0,560176199	False	0,560176199
ENSMUSG00000020248	Nfyb	437,4267397	644,87907	255,372411	358,748316	387,5105708	0,559987232	False	0,559987232
ENSMUSG00000038957	Edc3	198,619562	93,38757569	134,7309617	94,46664122	58,71372284	-0,559926295	False	0,559926295
ENSMUSG00000031119	Gpc4	370,1897674	251,136859	161,1487973	141,6999618	189,1886625	-0,559791231	False	0,559791231
ENSMUSG00000022540	Rogdi	238,0343389	350,834406	761,7142605	1059,375905	460,576537	0,559620526	False	0,559620526
ENSMUSG00000026281	Dtymk	157,6591076	200,6570883	287,9544083	136,0769475	106,9894505	-0,559339964	False	0,559339964
ENSMUSG00000020576	Nbas	355,5058309	686,5248808	241,2828987	459,9625745	1406,519849	-0,559147557	False	0,559147557
ENSMUSG00000085385	Snhg17	230,3059513	349,5724117	471,1180687	410,4800481	339,2348431	0,558732666	False	0,558732666
ENSMUSG00000030374	Stm4	411,9230606	699,1448234	606,7296249	679,2601345	707,1741729	0,558678861	False	0,558678861
ENSMUSG00000026820	Ptges2	189,3454969	734,4806629	128,5668	1032,385436	451,4432912	-0,558502969	False	0,558502969
ENSMUSG00000029617	Ccz1	418,8786094	426,554062	284,4320302	305,8919811	679,7744356	-0,558448275	False	0,558448275
ENSMUSG00000021510	Zfp729a	156,1134301	106,0075184	76,61172331	76,47299527	69,15171802	-0,558428066	False	0,558428066
ENSMUSG00000028007	Snx7	248,0812429	169,1072317	82,77588496	65,22696655	365,329831	0,558386903	False	0,558386903
ENSMUSG00000091537	Tma7	521,6661649	781,1744507	874,4303592	354,2499046	808,9446258	-0,558359399	False	0,558359399
ENSMUSG00000004637	Wwox	116,6986532	54,26575344	79,25350688	55,10554071	61,32322164	-0,558241232	False	0,558241232
ENSMUSG00000068036	Afdn	656,1401096	2847,059064	966,0121894	4316,225821	2656,469771	0,558037478	False	0,558037478
ENSMUSG00000040725	Hnrnpul1	1357,877706	1113,078943	997,7135921	782,7235986	1998,876075	0,557835479	False	0,557835479
ENSMUSG00000015305	Sash1	462,9304189	388,694234	222,7904138	374,4927562	681,079185	0,557027177	False	0,557027177
ENSMUSG00000041702	Btbd7	224,1232412	213,277031	152,3428521	314,8888041	306,6161082	-0,556970451	False	0,556970451
ENSMUSG00000042292	Mkl1	122,1085245	528,7755975	179,6412823	806,340259	446,2242936	0,556955005	False	0,556955005
ENSMUSG00000028979	Masp2	4332,534104	1890,467411	6373,743142	4174,525859	1432,614837	0,556929775	False	0,556929775
ENSMUSG00000024052	Lpin2	2364,113775	357,1443773	1256,608381	3477,272079	503,6332671	0,556656487	False	0,556656487
ENSMUSG00000025544	Tm9sf2	1842,447611	1241,802358	948,400299	549,9308042	2709,964497	0,556650354	False	0,556650354
ENSMUSG00000038482	Tfdp1	170,7973666	328,1185092	255,372411	265,4062777	116,1226963	-0,556635755	False	0,556635755
ENSMUSG00000025630	Hprt	401,8761567	391,2182225	300,2827316	235,0420002	591,0514766	0,5565328	False	0,5565328
ENSMUSG00000020289	Nprl3	228,7602738	535,0855688	336,387107	936,7941921	555,8232429	0,556285874	False	0,556285874
ENSMUSG00000023027	Atf1	257,355308	203,1810768	125,9250165	111,3356843	378,377325	0,556064096	False	0,556064096
ENSMUSG00000020131	Pcsk4	209,4393047	583,041351	634,9086495	1287,670288	307,9208576	0,556027381	False	0,556027381
ENSMUSG00000014470	Rnf166	244,9898878	654,9750241	360,163159	538,6847755	781,5448885	0,555928414	False	0,555928414
ENSMUSG00000040390	Map3k10	137,5652998	224,6349794	243,9246823	273,2784978	202,2361565	0,555924347	False	0,555924347
ENSMUSG00000035227	Spcs2	1782,166187	2825,605162	2432,202067	1212,321896	3101,389316	-0,555859061	False	0,555859061
ENSMUSG00000060073	Psma3	1296,050605	1361,691813	1277,74265	881,6886513	1941,467102	-0,555780855	False	0,555780855
ENSMUSG00000027552	E2f5	77,28387628	152,7013062	113,5966932	118,0833015	273,9973733	0,555681477	False	0,555681477
ENSMUSG00000005656	Snx6	218,7133699	222,1109908	148,820474	242,9142203	455,3575394	-0,555468395	False	0,555468395
ENSMUSG00000028080	Lrba	278,2219546	137,557375	189,327822	79,84680388	116,1226963	-0,555349835	False	0,555349835
ENSMUSG00000078517	Emc1	277,4491158	182,9891686	407,7152632	106,8372728	210,0646528	0,555338761	False	0,555338761
ENSMUSG00000020023	Tmcc3	174,6615604	502,2737179	118,8802603	659,0172828	523,204508	-0,555052954	False	0,555052954
ENSMUSG00000030555	Ttc23	587,3574597	186,7751514	399,7899125	294,6459524	113,5131975	-0,55499671	False	0,55499671
ENSMUSG00000036087	Slain2	469,8859678	408,8861422	369,8496988	386,8633878	690,2124308	0,554729772	False	0,554729772
ENSMUSG00000019718	L3hypdh	245,7627266	116,1034725	167,312959	84,34521537	63,93272043	-0,554716936	False	0,554716936
ENSMUSG00000000441	Raf1	1121,389045	1110,554954	763,4754496	870,4426226	1961,038343	-0,554633209	False	0,554633209
ENSMUSG00000032498	Mlh1	248,8540816	170,3692259	210,4620905	365,4959333	125,2559421	0,554555304	False	0,554555304
ENSMUSG00000024613	Tcof1	144,5208486	325,5945207	212,2232795	349,7514931	322,2731009	0,554305288	False	0,554305288
ENSMUSG00000035305	Ror1	67,23697236	345,7864289	45,79091508	163,0674164	92,63720715	-0,554193366	False	0,554193366
ENSMUSG00000098557	Kctd12	180,0714317	97,17355849	60,76102194	82,09600963	122,6464433	-0,55406392	False	0,55406392
ENSMUSG00000025809	Itgb1	1670,104566	723,1227145	715,0427509	520,6911296	1137,741474	-0,553765659	False	0,553765659
ENSMUSG00000016637	Ift27	136,792461	68,14769037	200,7755508	119,2079044	46,97097827	0,553594875	False	0,553594875
ENSMUSG00000026860	Sh3glb2	418,8786094	186,7751514	614,6549756	381,2403735	84,80871077	0,553244596	False	0,553244596
ENSMUSG00000026492	Tfb2m	312,9996989	223,3729851	185,8054439	150,6967848	459,2717876	-0,553186894	False	0,553186894
ENSMUSG00000030748	Il4ra	496,1624857	856,8941067	338,148296	1000,896556	949,8575607	-0,553156575	False	0,553156575
ENSMUSG00000049482	Ctu2	93,5134903	239,7789106	205,1785234	137,2015503	186,5791637	0,553050375	False	0,553050375
ENSMUSG00000047126	Cltc	2011,6993	1322,569991	991,5494305	1297,791714	2951,343135	0,552956991	False	0,552956991
ENSMUSG00000051177	Plcb1	146,8393649	29,02586812	85,41766852	100,0896556	24,79023853	-0,552945903	False	0,552945903
ENSMUSG00000002455	Prpf6	496,1624857	338,2144633	232,4769535	326,1348328	323,5778503	-0,552874303	False	0,552874303
ENSMUSG00000031007	Atp6ap2	318,4095703	410,1481365	280,0290576	217,0483542	801,1161295	-0,552867216	False	0,552867216
ENSMUSG00000060935	Tmem263	67,23697236	126,1994266	98,62658634	100,0896556	240,073889	0,552721838	False	0,552721838

ENSMUSG00000042032	Mat2b	282,0861484	195,6091113	121,5220439	133,8277417	413,6055587	0,55211975	False	0,55211975
ENSMUSG00000079179	Rab10os	140,6566548	321,8085379	95,98480277	105,7126699	742,4024066	-0,551299904	False	0,551299904
ENSMUSG00000047881	Rell1	148,3850425	614,5912076	101,2683699	195,6808997	1349,110876	-0,551162038	False	0,551162038
ENSMUSG00000028150	Rorc	904,9941912	3316,520931	1353,473779	1325,906786	2283,311444	0,550998917	False	0,550998917
ENSMUSG00000024436	Mrps18b	364,0070573	273,8527558	282,6708412	248,5372346	743,707156	-0,550504416	False	0,550504416
ENSMUSG00000030168	Adipor2	8726,122471	5766,051802	2807,335332	5161,927181	5958,790494	-0,550321198	False	0,550321198
ENSMUSG00000021748	Pdhb	1365,606094	2262,755719	932,5495976	1330,405197	4742,764056	-0,550289039	False	0,550289039
ENSMUSG00000028603	Scp2	19889,7784	7718,356932	13583,17048	6208,932454	9905,657418	-0,55020694	False	0,55020694
ENSMUSG00000073489	Ifi204	123,654202	94,64956996	44,91032056	181,0610623	58,71372284	0,550165058	False	0,550165058
ENSMUSG00000059273	Zc3h4	377,918155	560,3254542	618,1773536	665,7649	553,2137441	0,549763173	False	0,549763173
ENSMUSG00000058952	Cfi	9928,659586	2624,948074	6783,219594	2449,385054	3625,898573	-0,549628762	False	0,549628762
ENSMUSG00000022555	Dgat1	191,6640132	586,8273338	288,8350028	657,8926799	280,5211202	0,549529912	False	0,549529912
ENSMUSG00000001289	Pfdn5	482,251388	527,5136032	622,5803262	329,5086414	456,6622888	-0,54946909	False	0,54946909
ENSMUSG00000079111	Kdelr2	1333,146866	3136,055751	2137,202902	910,928326	4176,502818	-0,549426274	False	0,549426274
ENSMUSG00000052488	Cherp	630,6364304	1441,197452	922,8630579	1487,849599	2344,634666	0,549308071	False	0,549308071
ENSMUSG00000041044	Lrit1	224,1232412	0	153,2234466	10,12142584	2,609498793	-0,548655183	False	0,548655183
ENSMUSG00000039990	Edrf1	216,3948536	152,7013062	147,9398795	337,3808615	275,3021227	-0,548655183	False	0,548655183
ENSMUSG00000054414	Slc30a7	247,3084041	469,461867	169,074148	219,29756	1049,018515	-0,548655183	False	0,548655183
ENSMUSG00000041849	Card6	305,2713113	150,1773177	95,98480277	79,84680388	208,7599034	-0,548247375	False	0,548247375
ENSMUSG00000030340	Scnn1a	1001,599037	123,6754381	1464,428688	210,300737	139,6081854	0,548032862	False	0,548032862
ENSMUSG00000014402	Tsg101	574,2192008	834,1782099	392,7451563	805,2156561	808,9446258	-0,548008089	False	0,548008089
ENSMUSG00000015837	Sqstm1	5606,945224	3411,170501	3230,020703	2019,786757	8196,435709	0,547781702	False	0,547781702
ENSMUSG00000010110	Stx5a	874,8534795	1278,400192	1333,220105	4255,497266	2365,510656	0,54722621	False	0,54722621
ENSMUSG00000024588	Fech	2277,555834	855,6321124	1064,638776	1558,69958	865,0488499	-0,547143529	False	0,547143529
ENSMUSG00000018765	Fxr2	495,389647	1294,806117	723,8486961	1583,440843	1046,409016	0,547124448	False	0,547124448
ENSMUSG00000015943	Bola1	171,5702053	225,8969736	530,1179016	145,0737704	117,4274457	-0,547029397	False	0,547029397
ENSMUSG00000059743	Fdps	257,355308	46,69378785	376,0138604	127,0801245	52,18997586	0,547024304	False	0,547024304
ENSMUSG00000035078	Mtmr9	234,1701451	160,2732718	141,7757179	67,4761723	101,7704529	-0,547023303	False	0,547023303
ENSMUSG00000048799	Cep120	203,2565946	184,2511629	139,1339343	336,2562586	307,9208576	-0,54682783	False	0,54682783
ENSMUSG00000064262	Gimap8	261,2195018	55,52774771	162,0293918	178,8118566	10,43799517	-0,546820207	False	0,546820207
ENSMUSG00000004947	Dtx2	60,2814235	257,4468303	88,05945209	178,8118566	236,1596408	0,546764382	False	0,546764382
ENSMUSG00000021461	Fancc	60,2814235	225,8969736	88,05945209	111,3356843	116,1226963	0,546764382	False	0,546764382
ENSMUSG00000047284	Neurl4	279,7676321	537,6095574	408,5958577	624,1545937	435,7862984	0,546445519	False	0,546445519
ENSMUSG00000068250	Amn1	125,9727183	213,277031	209,581496	341,879273	183,9696649	0,546356571	False	0,546356571
ENSMUSG00000020528	Prpsap2	156,8862688	68,14769037	107,4325315	48,35792348	71,76121681	-0,546288168	False	0,546288168
ENSMUSG00000022336	Eif3e	1024,011361	894,7539347	450,8643947	458,8379716	1495,242808	0,546148058	False	0,546148058
ENSMUSG00000041439	Mfsd6	141,4294936	61,83771904	96,86539729	58,47934932	66,54221922	-0,546029713	False	0,546029713
ENSMUSG00000047459	Dynlrb1	1554,951591	2622,424085	2472,709415	1064,998919	2784,335212	-0,5460177	False	0,5460177
ENSMUSG00000038280	Ostm1	133,701106	87,07760436	91,58183017	91,0928326	73,0659662	-0,545878098	False	0,545878098
ENSMUSG00000019810	Fuca2	116,6986532	170,3692259	66,04458906	69,72537804	157,874677	0,545876852	False	0,545876852
ENSMUSG00000037235	Mxd4	1372,561643	940,1857283	1084,89245	1883,70981	1765,325933	-0,545853257	False	0,545853257
ENSMUSG00000040331	Nsmce4a	377,1453162	132,5093979	160,2682028	143,9491676	258,3403805	-0,545847344	False	0,545847344
ENSMUSG00000061032	Rrp1	794,4782482	1379,359733	1159,742984	1541,830537	1379,120112	0,545725492	False	0,545725492
ENSMUSG00000031337	Mtm1	125,9727183	123,6754381	86,29826304	302,5181725	95,24670595	-0,545707899	False	0,545707899
ENSMUSG00000057963	Itpk1	472,9773228	124,9374323	324,0587837	105,7126699	176,1411685	-0,545515475	False	0,545515475
ENSMUSG00000024778	Fas	282,8589872	177,9411915	280,9096522	412,7292539	198,3219083	0,545112689	False	0,545112689
ENSMUSG00000038383	Pigu	226,4417575	50,47977065	127,6862055	155,1951963	19,57124095	-0,545056123	False	0,545056123
ENSMUSG00000024644	Cndp2	574,2192008	1231,706404	862,1020359	837,8291394	1837,08715	0,545054489	False	0,545054489
ENSMUSG00000024818	Slc25a45	399,5576404	237,254922	582,9535728	203,5531198	63,93272043	0,544977348	False	0,544977348
ENSMUSG00000042198	Chchd7	179,298593	204,4430711	261,5365727	112,4602872	134,3891878	0,544648537	False	0,544648537
ENSMUSG00000060675	Pla2g16	129,8369122	102,2215356	189,327822	65,22696655	133,0844384	0,544185838	False	0,544185838
ENSMUSG00000036110	Slc17a2	3638,524895	1882,895445	2110,785066	2495,493772	1132,522476	-0,544028378	False	0,544028378
ENSMUSG00000061232	H2-K1	13338,42421	10207,00962	7979,066953	7549,459077	19445,98501	0,543884077	False	0,543884077
ENSMUSG00000021244	Ylpm1	306,8169888	604,4952535	210,4620905	663,5156943	641,9367031	-0,543817978	False	0,543817978
ENSMUSG00000050229	Pigm	147,6122037	251,136859	101,2683699	168,6904307	232,2453926	-0,543628366	False	0,543628366
ENSMUSG00000066440	Zfyve26	427,3798358	440,4359989	293,2379754	897,4330916	512,7665128	-0,543446892	False	0,543446892
ENSMUSG00000018286	Psmb6	1251,998796	2040,644728	2285,142782	859,1965939	1521,337796	-0,543172995	False	0,543172995
ENSMUSG00000063457	Rps15	3106,811826	5429,099333	7239,367556	4793,057439	4524,870907	0,54244177	False	0,54244177
ENSMUSG00000036432	Siah2	248,0812429	170,3692259	254,4918165	179,9364595	551,9089947	-0,542147896	False	0,542147896
ENSMUSG00000022956	Atp5o	425,8341583	387,4322397	619,9385427	289,022938	375,7678262	0,541833521	False	0,541833521
ENSMUSG00000036214	Znrd1as	206,3479497	31,54985665	141,7757179	93,34203834	13,04749397	-0,541468642	False	0,541468642
ENSMUSG00000056962	Jmjd6	220,2590474	445,4839759	376,8944549	320,5118184	456,6622888	0,541176274	False	0,541176274
ENSMUSG00000025278	Flnb	820,7547661	652,4510356	829,5200386	1194,32825	296,178113	0,541176274	False	0,541176274
ENSMUSG00000039678	Tbc1d13	445,9279661	169,1072317	306,4468933	212,5499427	170,9221709	-0,541173601	False	0,541173601
ENSMUSG00000029076	Sdf4	1439,025776	805,1523418	862,1020359	563,4260387	989,0000426	-0,541049946	False	0,541049946
ENSMUSG00000032185	Carm1	469,8859678	1900,563365	793,4156633	1621,677341	683,6886838	0,541028861	False	0,541028861
ENSMUSG00000024383	Map3k2	223,3504025	177,9411915	120,6414494	102,3388613	324,8825997	0,540609623	False	0,540609623
ENSMUSG00000032235	Ice2	170,7973666	171,6312202	167,312959	285,6491294	117,4274457	-0,54051609	False	0,54051609
ENSMUSG00000033272	Slc35a4	262,7651794	1111,816948	382,1780221	735,490278	2237,645215	0,540470708	False	0,540470708
ENSMUSG00000004460	Dnajb11	1245,043247	2598,446194	1810,502335	1974,802643	4676,221837	0,540194182	False	0,540194182
ENSMUSG00000025270	Alas2	216,3948536	88,33959863	148,820474	114,7094929	54,79947465	-0,540093169	False	0,540093169
ENSMUSG00000049299	Trappc1	493,8439694	694,0968464	972,176351	339,6300672	354,8918359	-0,540091107	False	0,540091107
ENSMUSG00000040466	Blvrb	2007,062267	1644,378529	3323,363722	2314,43271	1380,424862	-0,539973013	False	0,539973013
ENSMUSG00000028019	Pdgfc	119,7900082	174,1552087	17,61189042	34,86268902	87,41820957	0,539866045	False	0,539866045
ENSMUSG00000040774	Cept1	1090,475494	448,0079645	676,296592	750,1101154	358,806084	-0,539783047	False	0,539783047
ENSMUSG00000021024	Psma6	984,5965838	1108,030966	885,878088	420,601474	1431,310088	0,539731621	False	0,539731621
ENSMUSG00000032741	Tpcn1	1079,655752	235,9929278	1569,219436	589,2919047	87,41820957	0,539475727	False	0,539475727
ENSMUSG00000032579	Hemk1	182,389948	102,2215356	265,0589508	215,9237514	27,39973733	0,539287039	False	0,539287039
ENSMUSG00000016940	Kctd2	284,4046647	268,8047787	273,864896	416,1030625	195,7124095	-0,539209892	False	0,539209892
ENSMUSG00000003161	Sri	501,5723571	601,9712649	345,1930522	474,5824118	657,5936958	-0,539054413	False	0,539054413
ENSMUSG00000028622	Mrpl37	504,6637121	480,8198154	353,1184029	362,1221247	733,2691608	0,539020512	False	0,539020512
ENSMUSG00000022463	Srebf2	1013,964457	858,156101	1473,234633	536,4355698	687,602932	0,538980137	False	0,538980137
ENSMUSG00000039217	Il18	243,4442103	47,95578211	126,8055611	167,5658279	67,84696862	-0,538863224	False	0,538863224
ENSMUSG00000036678	Aaas	123,654202	301,6166296	179,6412823	318,2626127	210,0646528	0,538807658	False	0,538807658
ENSMUSG00000055681	Cope	1401,929516	2819,295191	3815,616059	2036,655801	2768,678219	0,538788365	False	0,538788365
ENSMUSG00000025823	Pdia4	4263,751454	5029,047151	3767,18336	2935,213495	11184,31183	-0,538657899	False	0,538657899
ENSMUSG00000028868	Wasf2	326,1379579	479,5578211	224,5516028	366,6205361	775,0211415	-0,53843534	False	0,53843534
ENSMUSG00000024012	Mtch1	1148,438402	416,4581078	790,7738797	544,3077899	426,6530527	-0,538336354	False	0,538336354
ENSMUSG00000023973	Cnpy3	762,0190201	1140,842817	1526,950899	1106,609226	1565,699276	0,538246943	False	0,538246943
ENSMUSG00000025350	Rdh5	176,2072379	238,5169163	722,9681016	159,6936078	121,3416939	-0,53819783	False	0,53819783
ENSMUSG00000040374	Pex2	414,2415769	194,347117	285,3126248	185,5594738	250,5118841	-0,537928776	False	0,537928776
ENSMUSG00000029050	Ski	793,7054094	1843,773623	748,5053427	1221,318719	546,6899971	-0,537880659	False	0,537880659
ENSMUSG00000054517	Trim65	170,0245278	39,12182225	117,1190713	34,86268902	54,79947465	-0,537766867	False	0,537766867
ENSMUSG00000020361	Hspa4	1632,235467	1525,751068	897,3258168	644,3974454	2369,424904	0,537687741	False	0,537687741
ENSMUSG00000052253	Zfp622	189,3454969	204,4430711	169,9547425	130,4539331	354,8918359	-0,53748067	False	0,53748067
ENSMUSG00000025531	Chm	264,3108569	143,8673463	104,790748	131,578536	383,5963226	0,537353203	False	0,537353203
ENSMUSG00000022629	Kif21a	619,8166878	759,7205482	427,0883426	716,3720292	1380,424862	-0,537307078	False	0,537307078
ENSMUSG00000024054	Smchd1	162,2961402	172,8932145	111,8355041	264,2816748	335,3205949	-0,53725042	False	0,53725042
ENSMUSG00000037773	Pced1a	313,7725377	196,8711055	455,2673673	446,46734	103,0752023	0,536994964	False	0,536994964
ENSMUSG00000025964	Adam23	183,9356255	174,1552087	266,8201398	199,0547083	79,58971319	0,536666632	False	0,536666632
ENSMUSG00000024294	Mib1	531,7130688	199,395094	234,2381425	124,8309187	366,6345804	-0,536305061	False	0,536305061
ENSMUSG00000014504	Srp19	204,0294334	469,461867	295,879759	308,1411868	771,1068933	0,536233715	False	0,536233715
ENSMUSG00000051403	Ppp1r37	996,962004	851,8461296	743,2217756	349,7514931	1445,662331	0,536120189	False	0,536120189
ENSMUSG00000007880	Arid1a	615,1796552	2323,331444	892,0422496	1879,211398	1680,517223	0,53610425	False	0,53610425
ENSMUSG00000038039	Gcc2	311,4540214	181,7271743	162,0293918	214,7991485	164,398424	-0,536030926	False	0,536030926
ENSMUSG00000038766	Gabpb2	338,5033781	233,4689392	175,2383097	114,7094929	203,5409059	-0,535939605	False	0,535939605
ENSMUSG00000024563	Smad2	253,4911142	90,86358716	114,4772877	97,84044983	174,8364191	-0,535929441	False	0,535929441
ENSMUSG00000033106	Slc7a6os	215,6220148	453,0559415	312,6110549	325,0102299	497,1095201	0,535864314	False	0,535864314
ENSMUSG00000038976	Ppp1r9b	136,792461	201,9190826	200,7755508	289,022938	198,3219083	0,535855337	False	0,535855337
ENSMUSG00000052605	Isoc2b	201,7109171	13,88193693	139,1339343	4,498411486	28,70448672	-0,535814837	False	0,535814837
ENSMUSG00000054827	Cyp2c50	25513,72608	1838,725646	17598,6815	132,7031389	4971,095201	-0,535806263	False	0,535806263
ENSMUSG00000024218	Taf11	119,0171695	114,8414782	140,0145288	82,09600963	157,874677	-0,535785707	False	0,535785707
ENSMUSG00000042462	Dctpp1	232,6244676	167,8452374	580,3117892	176,5626508	160,4841758	-0,535571798	False	0,535571798

ENSMUSG00000021895	Arhgef3	282,0861484	358,4063716	194,6113891	264,2816748	313,1398552	-0,535539683	False	0,535539683
ENSMUSG00000048772	Tmem53	411,9230606	73,19566744	597,0430851	219,29756	22,18073974	0,535460152	False	0,535460152
ENSMUSG00000034707	Gns	1925,914197	1688,548328	1328,817132	2245,831935	2785,639962	-0,535400851	False	0,535400851
ENSMUSG00000029599	Ddx54	472,2044841	811,4623131	325,8199727	599,4133306	1039,885269	-0,535336698	False	0,535336698
ENSMUSG00000027509	Rae1	248,8540816	329,3805035	171,7159316	249,6618375	431,8720502	-0,535276153	False	0,535276153
ENSMUSG00000041992	Rapgef5	79,60239257	212,0150367	115,3578822	578,045876	134,3891878	0,535232886	False	0,535232886
ENSMUSG00000026621	Marc1	4669,491805	2163,058172	2311,560617	6766,735479	511,4617634	0,535194447	False	0,535194447
ENSMUSG00000030701	Plekhb1	738,8338572	177,9411915	509,8642276	219,29756	212,6741516	-0,535136859	False	0,535136859
ENSMUSG00000043885	Slc36a4	71,10116618	222,1109908	103,0295589	322,7610242	247,9023853	0,535113175	False	0,535113175
ENSMUSG00000026791	Slc2a8	204,8022721	141,3433578	206,9397124	304,7673782	262,2546287	-0,535027633	False	0,535027633
ENSMUSG00000061589	Dot1l	392,6020915	1399,551641	568,8640605	1391,133752	2525,994832	0,535016079	False	0,535016079
ENSMUSG00000028467	Gba2	172,3430441	133,7713922	156,7458247	249,6618375	83,50396138	0,534692241	False	0,534692241
ENSMUSG00000040651	Fam208a	394,147769	189,2991399	272,1037069	251,9110432	56,10422405	-0,534579998	False	0,534579998
ENSMUSG00000074994	Qser1	279,7676321	405,1001594	143,5369069	217,0483542	285,7401178	0,534049594	False	0,534049594
ENSMUSG00000001750	Tcirg1	668,5055298	967,9496021	1822,830658	3007,188079	1075,113503	0,533992439	False	0,533992439
ENSMUSG00000023008	Fmnl3	57,19006845	98,43555276	82,77588496	247,4126318	105,6847011	0,533445897	False	0,533445897
ENSMUSG00000027900	Dram2	940,5447743	412,672125	649,8787564	502,6974836	503,6332671	-0,533326035	False	0,533326035
ENSMUSG00000025733	Rhot2	183,1627868	179,2031858	227,1933864	217,0483542	126,5606915	-0,533297034	False	0,533297034
ENSMUSG00000021681	Aggf1	281,3133097	112,3174897	89,82064113	146,1983733	194,4076601	-0,533092747	False	0,533092747
ENSMUSG00000067288	Rps28	1064,198976	2388,955146	3342,736801	1539,581331	2034,104309	0,532770161	False	0,532770161
ENSMUSG00000019989	Enpp3	520,8933261	41,64581078	360,163159	166,441225	79,58971319	-0,532337337	False	0,532337337
ENSMUSG00000022214	Dcaf11	3725,082837	432,8640333	2575,738974	1951,185982	416,2150575	-0,53228611	False	0,53228611
ENSMUSG00000022517	Mgrn1	786,7498605	724,3847088	754,6695044	1198,826661	544,0804983	-0,532084899	False	0,532084899
ENSMUSG00000027801	Tm4sf4	624,4537203	692,8348521	588,2371399	272,1538949	902,8865824	0,531950118	False	0,531950118
ENSMUSG00000062110	Scfd2	123,654202	85,8156101	84,537074	62,97776081	178,7506673	0,531637361	False	0,531637361
ENSMUSG00000058586	Serhl	281,3133097	143,8673463	194,6113891	181,0610623	136,9986866	-0,531581669	False	0,531581669
ENSMUSG00000027282	Mtch2	1961,46478	686,5248808	1356,996157	575,7966703	1088,160997	-0,531514796	False	0,531514796
ENSMUSG00000040249	Lrp1	8049,888553	12661,58847	11634,41481	14121,63826	14606,66949	0,531357932	False	0,531357932
ENSMUSG00000026510	Trp53bp2	295,2244074	239,7789106	203,4173343	276,6523064	426,6530527	0,531251371	False	0,531251371
ENSMUSG00000039483	Asb6	95,05916782	275,11475	137,3727453	260,9078662	388,8153202	0,531198125	False	0,531198125
ENSMUSG00000003809	Gcdh	5898,305438	1827,367697	4081,555604	2625,947705	1786,201924	-0,531181421	False	0,531181421
ENSMUSG00000022177	Haus4	124,4270408	61,83771904	58,9998329	53,98093784	86,11346017	-0,530989386	False	0,530989386
ENSMUSG00000022752	Tomm70a	1160,030983	705,4547948	562,6998988	802,9664503	729,3549126	-0,530751723	False	0,530751723
ENSMUSG00000069833	Ahnak	142,2023324	98,43555276	273,864896	157,444402	125,2559421	-0,530693743	False	0,530693743
ENSMUSG00000059409	Ppp2r5d	553,3525542	225,8969736	383,0586166	265,4062777	242,6833878	-0,530633775	False	0,530633775
ENSMUSG00000028917	Plekhm2	132,9282672	371,0263142	191,9696055	377,8665649	532,3377538	0,530229981	False	0,530229981
ENSMUSG00000029191	Rfc1	240,3528552	142,6053521	166,4323644	119,2079044	139,6081854	-0,530217935	False	0,530217935
ENSMUSG00000037465	Klf10	144,5208486	1486,629246	208,7009014	594,9149191	2763,459222	0,5301592	False	0,5301592
ENSMUSG00000004070	Hmox2	545,6241665	740,7906342	582,0729783	377,8665649	818,0778716	-0,530030676	False	0,530030676
ENSMUSG00000049287	Iba57	118,2443307	66,88569611	81,89529044	48,35792348	57,40897345	-0,52991862	False	0,52991862
ENSMUSG00000013629	Cad	159,9776239	230,9449507	319,6558111	882,8132542	383,5963226	0,529678875	False	0,529678875
ENSMUSG00000036452	Arhgap26	227,2145963	80,76763303	88,05945209	157,444402	37,8377325	-0,529213054	False	0,529213054
ENSMUSG00000078672	Mup20	568,0364907	310,4505895	393,6257508	47,23332061	250,5118841	-0,529159007	False	0,529159007
ENSMUSG00000025204	Ndufb8	690,1450152	478,2958269	445,5808276	213,6745456	473,6240309	-0,528996333	False	0,528996333
ENSMUSG00000025521	Tmem192	132,9282672	122,4134438	63,4028055	96,71584696	191,7981613	0,528940963	False	0,528940963
ENSMUSG00000031347	Cetn2	142,2023324	55,52774771	205,1785234	39,36110051	48,27572767	-0,5289346	False	0,5289346
ENSMUSG00000040883	Tmem205	5192,703647	2089,862505	7492,098183	1212,321896	2472,500106	0,528883914	False	0,528883914
ENSMUSG00000059708	Akap17b	107,424588	74,4576617	161,1487973	428,4736941	78,28496379	-0,528832029	False	0,528832029
ENSMUSG00000027288	Zfp106	864,8065756	599,4472764	477,2822303	422,8506797	558,4327417	-0,528744624	False	0,528744624
ENSMUSG00000017765	Slc12a4	191,6640132	1138,318828	276,5066795	1129,101283	823,2968692	0,52873485	False	0,52873485
ENSMUSG00000032462	Pik3cb	164,6146565	129,9854094	87,17885756	103,4634642	237,4643902	0,528618393	False	0,528618393
ENSMUSG00000041037	Irgq	245,7627266	170,3692259	158,5070138	120,3325073	144,827183	-0,528601364	False	0,528601364
ENSMUSG00000031556	Tm2d2	623,6808816	194,347117	432,3719097	158,5690049	253,1213829	-0,528535238	False	0,528535238
ENSMUSG00000038014	Fam120a	2352,521194	1094,149029	1441,533231	1277,548862	1630,936746	-0,528506891	False	0,528506891
ENSMUSG00000049624	Slc17a5	289,8145361	124,9374323	177,8800932	160,8182106	200,9314071	-0,528426872	False	0,528426872
ENSMUSG00000043411	Usp48	622,1352041	974,2595735	897,3258168	2543,851696	1055,542262	0,528403775	False	0,528403775
ENSMUSG00000004951	Hspb1	413,4687381	137,557375	474,6404467	74,22378953	596,2704742	0,528188647	False	0,528188647
ENSMUSG00000028962	Slc4a2	508,5279059	1274,614209	904,3705729	1499,095628	733,2691608	0,52801592	False	0,52801592
ENSMUSG00000020537	Drg2	602,814235	1066,385155	869,1467921	895,1838858	1603,537008	0,527886372	False	0,527886372
ENSMUSG00000019189	Rnf145	166,9331728	194,347117	146,1786905	115,8340958	223,1121468	-0,527210701	False	0,527210701
ENSMUSG00000040997	Abhd4	622,9080428	559,0634599	435,8942878	298,019761	897,6675848	0,527162099	False	0,527162099
ENSMUSG00000033751	Gadd45gip1	651,503077	846,7981526	1362,279724	452,0903544	611,927467	-0,527160855	False	0,527160855
ENSMUSG00000029063	Nadk	3262,925257	914,9458429	2264,889108	1175,210001	465,7955346	-0,526725525	False	0,526725525
ENSMUSG00000020064	Herc4	584,2661047	487,1297867	339,0288905	535,3109669	841,5633607	0,526446299	False	0,526446299
ENSMUSG00000022010	Tsc22d1	388,7378977	466,9378785	405,9540741	269,9046892	478,8430285	-0,52634772	False	0,52634772
ENSMUSG00000024999	Noc3l	74,96535999	121,1514495	112,7160987	107,9618757	164,398424	0,526225933	False	0,526225933
ENSMUSG00000038212	Mfsd14b	300,6342787	82,0296273	98,62658634	74,22378953	208,7599034	-0,526164877	False	0,526164877
ENSMUSG00000057193	Slc44a2	157,6591076	296,5686525	234,2381425	674,761723	227,026395	0,526051527	False	0,526051527
ENSMUSG00000030499	Kctd15	78,82955381	182,9891686	145,2980959	387,9879907	113,5131975	0,526051527	False	0,526051527
ENSMUSG00000030516	Tjp1	646,0932057	333,1664863	410,3570467	448,7165458	344,4538407	-0,525937921	False	0,525937921
ENSMUSG00000038250	Usp38	225,6689187	292,7826697	156,7458247	277,7769093	390,1200696	-0,525780717	False	0,525780717
ENSMUSG00000026740	Dnajc1	432,0168684	392,4802168	552,1327646	502,6974836	300,0923612	-0,525681049	False	0,525681049
ENSMUSG00000032423	Syncrip	411,1502218	594,3992994	519,5507673	285,6491294	712,3931705	-0,525421474	False	0,525421474
ENSMUSG00000073131	Vma21	441,2909336	219,5870023	279,1484631	109,0864785	306,6161082	-0,525296616	False	0,525296616
ENSMUSG00000057788	Ddx49	238,0343389	594,3992994	342,5512686	542,0585841	814,1636234	0,525150216	False	0,525150216
ENSMUSG00000044813	Shb	449,79216	2036,858746	647,2369728	933,4203834	1246,035674	0,525035508	False	0,525035508
ENSMUSG00000087141	Plcxd2	864,0337368	161,5352661	600,5654632	305,8919811	178,7506673	-0,524766132	False	0,524766132
ENSMUSG00000029534	St7	66,4641336	135,0333865	96,86539729	95,59124409	183,9696649	0,524302456	False	0,524302456
ENSMUSG00000059883	Irak4	132,9282672	133,7713922	99,50718086	191,1824882	82,19921198	0,524302456	False	0,524302456
ENSMUSG00000020115	Tbk1	347,0046045	932,6137627	241,2828987	585,9180961	1607,451257	-0,52422914	False	0,52422914
ENSMUSG00000020679	Hnf1b	383,3280264	819,0342787	551,2521701	773,7267757	654,984197	0,524132948	False	0,524132948
ENSMUSG00000033022	Cdo1	32431,40584	13285,01364	12933,29173	13135,36154	22556,50757	-0,523847851	False	0,523847851
ENSMUSG00000039048	Foxred1	327,6836354	603,2332592	471,1180687	746,7363068	473,6240309	0,523785038	False	0,523785038
ENSMUSG00000021806	Nid2	202,4837559	138,8193693	140,8951233	71,97458378	70,45646741	-0,523184495	False	0,523184495
ENSMUSG00000029464	Gpn3	114,3801369	66,88569611	58,9998329	48,35792348	79,58971319	-0,523182655	False	0,523182655
ENSMUSG00000036241	Ube2r2	2287,602738	1153,462759	1481,159984	826,5831106	1591,794264	-0,523182655	False	0,523182655
ENSMUSG00000062328	Rpl17	4119,230606	7811,744507	7314,21809	2866,61272	7613,212729	-0,523027885	False	0,523027885
ENSMUSG00000058056	Palld	1102,068076	709,2407776	766,9978277	546,5569956	671,9459392	-0,522918946	False	0,522918946
ENSMUSG00000054021	Sirt5	142,9751711	39,12182225	99,50718086	34,86268902	24,79023853	-0,522892087	False	0,522892087
ENSMUSG00000024018	Ccdc167	163,068979	212,0150367	376,0138604	295,7705552	113,5131975	-0,52262232	False	0,52262232
ENSMUSG00000064120	Mocs1	1360,196223	1958,615101	1148,295255	946,9156179	1530,471042	-0,522507016	False	0,522507016
ENSMUSG00000026170	Cyp27a1	9471,139038	1557,300924	6596,533556	2475,25092	2569,051562	-0,521829846	False	0,521829846
ENSMUSG00000030852	Tacc2	291,3602136	6026,022621	418,2823974	4216,136166	5212,473839	0,521673401	False	0,521673401
ENSMUSG00000035247	Hectd1	1362,514739	1625,448615	949,2808935	2547,225504	2373,339152	-0,521364885	False	0,521364885
ENSMUSG00000030281	Il17rc	401,8761567	176,6791973	576,7894112	472,3332061	203,5409059	0,521293695	False	0,521293695
ENSMUSG00000021408	Ripk1	308,3626664	1041,14527	214,8650631	581,4196846	1506,985553	-0,521197187	False	0,521197187
ENSMUSG00000027346	Gpcpd1	682,4166276	1583,802804	475,5210413	5202,412884	1876,229632	-0,521143619	False	0,521143619
ENSMUSG00000028452	Vcp	4167,919448	4560,847278	2497,366061	2681,053246	5980,971234	0,521052383	False	0,521052383
ENSMUSG00000022389	Tef	567,2636519	912,4218544	2209,411653	2025,409772	395,3390671	-0,520928896	False	0,520928896
ENSMUSG00000025138	Sirt7	602,814235	527,5136032	1274,220272	669,1387086	420,1293057	-0,520880062	False	0,520880062
ENSMUSG00000045658	Pid1	1650,010759	1364,215802	1343,787239	2367,289045	1184,712452	0,520760438	False	0,520760438
ENSMUSG00000039354	Smarcal1	183,9356255	102,2215356	89,82064113	128,2047274	44,36147948	-0,520751475	False	0,520751475
ENSMUSG00000027411	Vps16	298,3157624	1899,301371	427,9689371	2182,854174	1987,133331	0,520665878	False	0,520665878
ENSMUSG00000027130	Slc12a6	163,8418177	119,8894553	161,1487973	235,0420002	65,23746983	0,520614952	False	0,520614952
ENSMUSG00000024908	Ppp6r3	774,3844403	848,0601468	539,8044413	865,9442111	1273,435411	-0,520613118	False	0,520613118
ENSMUSG00000034021	Pds5b	264,3108569	184,2511629	151,4622576	174,3134451	122,6464433	-0,520561962	False	0,520561962
ENSMUSG00000029462	Vps29	380,2366713	358,4063716	307,3274878	239,5404117	545,3852477	0,520378	False	0,520378
ENSMUSG00000070733	Fryl	226,4417575	220,8489966	293,2379754	401,4832252	157,874677	-0,520360244	False	0,520360244
ENSMUSG00000033396	Spg11	214,8491761	166,5832431	233,357548	308,1411868	118,7321951	0,520267285	False	0,520267285
ENSMUSG00000022131	Gpr180	362,4613798	442,9599874	252,7306275	381,2403735	558,4327417	-0,520226774	False	0,520226774
ENSMUSG00000029594	Rbm19	111,2887818	122,4134438	80,1341014	77,59759814	197,0171589	-0,52022427	False	0,52022427
ENSMUSG00000052151	Plpp2	210,2121435	391,2182225	379,5362385	301,3935696	503,6332671	0,519802624	False	0,519802624
ENSMUSG00000032802	Srxn1	598,9500412	585,5653395	536,2820632	344,1284787	858,5251029	0,519424647	False	0,519424647

ENSMUSG00000015994	Fnta	326,9107967	411,4101308	228,0739809	381,2403735	318,3588527	-0,519395154	False	0,519395154
ENSMUSG00000038437	Mllt6	269,7207282	524,9896147	386,5809947	561,1768329	576,6992333	0,519304315	False	0,519304315
ENSMUSG00000007656	Arpp19	662,3228197	888,4439634	924,6242469	462,2117802	767,1926451	-0,518980536	False	0,518980536
ENSMUSG00000009687	Fxyd5	153,022075	315,4985665	219,2680357	248,5372346	374,4630768	0,518955723	False	0,518955723
ENSMUSG00000028771	Ptpn12	291,3602136	535,0855688	203,4173343	372,2435505	502,3285177	-0,518361261	False	0,518361261
ENSMUSG00000022946	Dopey2	664,641336	1696,120294	951,922677	1196,577455	1424,786341	0,518268369	False	0,518268369
ENSMUSG00000049106	Dcaf5	397,2391241	868,2520551	277,3872741	686,0077517	1075,113503	-0,518106119	False	0,518106119
ENSMUSG00000028465	Tln1	1564,225656	2240,039822	2376,724612	4456,80118	2652,555523	0,518075729	False	0,518075729
ENSMUSG00000029227	Fip1l1	600,4957187	567,8974198	331,1035398	391,3617993	859,8298523	0,51789724	False	0,51789724
ENSMUSG00000081534	Slc48a1	732,6511471	490,9157695	791,6544742	409,3554453	1049,018515	0,517841815	False	0,517841815
ENSMUSG00000051413	Plagl2	96,60484535	95,91156423	115,3578822	67,4761723	238,7691396	-0,517717415	False	0,517717415
ENSMUSG00000026879	Gsn	328,4564742	170,3692259	470,2374741	259,7832633	241,3786384	0,517687312	False	0,517687312
ENSMUSG00000014444	Piezo1	96,60484535	196,8711055	142,6563124	290,1475409	138,303436	0,517669543	False	0,517669543
ENSMUSG00000042138	Msantd2	112,0616206	487,1297867	317,0140275	552,18001	78,28496379	-0,517485121	False	0,517485121
ENSMUSG00000025017	Pik3ap1	751,1992774	574,2073911	524,8343344	1599,185283	1437,833835	-0,517333572	False	0,517333572
ENSMUSG00000049577	Zfpm1	809,1621847	1250,636318	565,3416824	1228,066336	1089,465746	-0,517305828	False	0,517305828
ENSMUSG00000036905	C1qb	856,3053492	422,7680792	584,7147618	598,2887277	510,157014	-0,517283456	False	0,517283456
ENSMUSG00000020774	Aspa	163,8418177	20,19190826	114,4772877	41,61030625	19,57124095	-0,51724223	False	0,51724223
ENSMUSG00000042745	Id1	66,4641336	956,5916537	95,10420825	540,9339812	330,1015973	0,516933159	False	0,516933159
ENSMUSG00000027272	Ubr1	204,0294334	142,6053521	151,4622576	287,8983351	262,2546287	-0,516749163	False	0,516749163
ENSMUSG00000027263	Tubgcp4	459,8390639	378,5982798	440,2972604	657,8926799	174,8364191	0,516723231	False	0,516723231
ENSMUSG00000056608	Chd9	293,6787299	147,6533291	420,0435864	228,2943829	54,79947465	0,516300259	False	0,516300259
ENSMUSG00000015889	Lta4h	382,5551876	223,3729851	240,4023042	181,0610623	267,4736263	-0,516271242	False	0,516271242
ENSMUSG00000020198	Ap3d1	1038,695297	1532,061039	1485,562957	3013,935696	1981,914333	0,516237247	False	0,516237247
ENSMUSG00000038697	Taf5l	136,0196223	175,417203	95,10420825	104,5880671	206,1504046	-0,516233705	False	0,516233705
ENSMUSG00000040236	Trappc5	270,493567	769,8165023	560,9387098	386,8633878	594,9657248	0,516229917	False	0,516229917
ENSMUSG00000061315	Naca	2298,422481	2479,818733	1693,383264	791,7204216	3286,66373	0,515979848	False	0,515979848
ENSMUSG00000078578	Ube2d3	3091,355051	4957,113477	2161,859549	2501,116786	7396,624329	-0,515966564	False	0,515966564
ENSMUSG00000090306	Adh6-ps1	197,0738845	16,40592546	281,7902467	233,9173973	1,304749397	0,515885069	False	0,515885069
ENSMUSG00000033446	Lpar6	324,5922804	477,0338326	415,6406138	452,0903544	227,026395	-0,515768649	False	0,515768649
ENSMUSG00000068391	Chrac1	139,8838161	252,3988532	134,7309617	97,84044983	314,4446046	-0,515726117	False	0,515726117
ENSMUSG00000024194	Cuta	712,5573393	887,1819691	1783,203905	1136,973503	498,4142695	-0,515660739	False	0,515660739
ENSMUSG00000070934	Rraga	387,9650589	151,4393119	121,5220439	59,6039522	271,3878745	-0,515570463	False	0,515570463
ENSMUSG00000075415	Fnbp1	306,8169888	116,1034725	438,5360714	156,3197992	88,72295896	0,515317146	False	0,515317146
ENSMUSG00000029090	Adgra3	632,182108	225,8969736	289,7155974	196,8055025	442,3100454	-0,515282197	False	0,515282197
ENSMUSG00000029547	Ints1	347,0046045	495,9637466	664,8488632	724,2442493	503,6332671	0,515279862	False	0,515279862
ENSMUSG00000047843	Bri3	558,7624255	459,3659129	312,6110549	161,9428135	798,5066307	0,515069379	False	0,515069379
ENSMUSG00000039361	Picalm	1448,299841	2420,505002	1013,564294	1786,993963	4487,033175	-0,514922708	False	0,514922708
ENSMUSG00000005566	Trim28	729,5597921	1042,407264	1244,280058	1197,702058	1169,055459	0,514820916	False	0,514820916
ENSMUSG00000002748	Baz1b	561,0809418	811,4623131	1113,952069	1817,358241	392,7295683	-0,514672688	False	0,514672688
ENSMUSG00000039531	Zufsp	100,4690392	271,3287672	143,5369069	371,1189476	264,8641275	0,514670753	False	0,514670753
ENSMUSG00000030662	Ipo5	517,0291323	643,6170757	361,9243481	823,209302	1238,207177	-0,514557406	False	0,514557406
ENSMUSG00000020628	Trappc12	285,9503422	121,1514495	151,4622576	200,1793111	185,2744143	-0,514471755	False	0,514471755
ENSMUSG00000039831	Arhgap29	1425,114679	294,044664	997,7135921	434,0967084	310,5303564	-0,514380383	False	0,514380383
ENSMUSG00000030105	Arl8b	655,3672709	637,3071044	477,2822303	458,8379716	1770,544931	-0,514318838	False	0,514318838
ENSMUSG00000052395	Rft1	202,4837559	191,8231285	141,7757179	217,0483542	340,5395925	-0,514195712	False	0,514195712
ENSMUSG00000020409	Slu7	381,0095101	371,0263142	266,8201398	609,5347564	578,0039827	-0,513959442	False	0,513959442
ENSMUSG00000020644	Id2	2284,511383	2286,73361	1442,413825	1285,421082	3261,873491	0,513815199	False	0,513815199
ENSMUSG00000057367	Birc2	250,3997591	859,4180952	357,5213755	1261,804422	985,0857944	0,513796326	False	0,513796326
ENSMUSG00000054252	Fgfr3	506,9822284	118,627461	723,8486961	705,1260005	92,63720715	0,51375299	False	0,51375299
ENSMUSG00000027854	Sike1	188,5726581	109,7935012	132,0891781	122,581713	91,33245776	-0,513608236	False	0,513608236
ENSMUSG00000029475	Kdm2b	94,28632906	122,4134438	66,04458906	186,6840767	91,33245776	-0,513608236	False	0,513608236
ENSMUSG00000084957	Bbip1	163,068979	326,8565149	289,7155974	232,7927944	264,8641275	0,513564043	False	0,513564043
ENSMUSG00000021374	Nup153	315,3182152	248,6128704	169,9547425	263,157072	450,1385418	0,513560583	False	0,513560583
ENSMUSG00000024384	Iws1	315,3182152	261,2328131	152,3428521	169,8150336	450,1385418	0,513560583	False	0,513560583
ENSMUSG00000024807	Syvn1	2199,499119	3139,841734	4834,46392	8607,710379	12807,42008	0,513516818	False	0,513516818
ENSMUSG00000026209	Dnpep	561,8537806	336,9524691	370,7302933	393,6110051	341,8443419	-0,513424168	False	0,513424168
ENSMUSG00000021944	Gata4	250,3997591	199,395094	348,7154303	175,438048	622,3654621	-0,51327151	False	0,51327151
ENSMUSG00000023087	Noct	392,6020915	275,11475	677,1771865	516,1927181	360,1108334	-0,513034368	False	0,513034368
ENSMUSG00000048076	Arf1	2670,930764	2363,71526	2009,516697	924,4235605	3811,172987	0,512892514	False	0,512892514
ENSMUSG00000022562	Oplah	1393,428289	4398,050017	1987,501834	7875,59391	4335,682245	0,512317433	False	0,512317433
ENSMUSG00000027763	Mbnl1	1030,96691	1047,455241	1203,77271	1494,597216	722,8311657	-0,512267412	False	0,512267412
ENSMUSG00000038369	Ncoa6	510,0735834	572,9453968	457,9091508	357,6237132	764,5831464	-0,512262985	False	0,512262985
ENSMUSG00000029924	Slc37a3	282,8589872	344,5244347	289,7155974	419,4768711	198,3219083	-0,512238952	False	0,512238952
ENSMUSG00000058216	Gstp3	383,3280264	435,3880218	619,0579482	350,8760959	268,7783757	-0,51216241	False	0,51216241
ENSMUSG00000030272	Camk1	193,9825295	140,0813635	195,4919836	276,6523064	84,80871077	0,512147226	False	0,512147226
ENSMUSG00000033542	Arhgef5	442,0637723	781,1744507	309,9692713	558,9276272	745,0119054	-0,512129307	False	0,512129307
ENSMUSG00000042810	Krba1	251,9454367	591,8753108	359,2825645	1081,867962	364,0250816	0,512007599	False	0,512007599
ENSMUSG00000023723	Mrps23	173,1158829	169,1072317	124,1638274	121,4571101	284,4353684	-0,511291146	False	0,511291146
ENSMUSG00000021189	Atxn3	187,7998194	102,2215356	84,537074	50,60712922	131,779689	-0,511067648	False	0,511067648
ENSMUSG00000053012	Krcc1	780,5671504	629,7351388	593,5207071	1112,23224	118,7321951	0,510863407	False	0,510863407
ENSMUSG00000020766	Galk1	466,021774	323,0705321	663,9682687	546,5569956	181,3601661	0,510716933	False	0,510716933
ENSMUSG00000061292	Cyp3a59	529,3945525	29,02586812	371,6108878	136,0769475	70,45646741	-0,510550578	False	0,510550578
ENSMUSG00000024576	Csnk1a1	1256,635828	1004,547436	687,7443208	629,7776081	1790,116172	0,510486597	False	0,510486597
ENSMUSG00000035764	Fbxo45	88,10361896	108,5315069	78,37291236	61,85315794	130,4749397	-0,510354026	False	0,510354026
ENSMUSG00000026933	Camsap1	92,74065154	354,6203888	132,0891781	520,6911296	161,7889252	0,510238506	False	0,510238506
ENSMUSG00000079480	Pin4	92,74065154	117,3654668	132,0891781	50,60712922	66,54221922	0,510238506	False	0,510238506
ENSMUSG00000036813	Entpd8	1673,195921	283,9487099	2382,888773	1064,998919	86,11346017	0,510105226	False	0,510105226
ENSMUSG00000020163	Uqcr11	1899,637679	1600,208729	2705,186368	674,761723	1522,642546	0,510003712	False	0,510003712
ENSMUSG00000032199	Polr2m	754,2906325	315,4985665	335,5065124	318,2626127	529,728255	-0,509868047	False	0,509868047
ENSMUSG00000034892	Rps29	969,9126473	2675,427844	3533,825812	1381,012326	1988,43808	0,509799471	False	0,509799471
ENSMUSG00000003814	Calr	13069,47632	33808,82639	19866,21239	9179,008638	61340,18338	-0,509791083	False	0,509791083
ENSMUSG00000022452	Smdt1	782,8856667	742,0526285	1395,742316	549,9308042	635,4129561	-0,509551529	False	0,509551529
ENSMUSG00000021413	Prpf4b	833,1201863	800,1043647	648,1175674	546,5569956	1186,017201	0,509528395	False	0,509528395
ENSMUSG00000026426	Arl8a	179,298593	210,7530424	168,1935535	125,9555216	639,3272043	-0,509449799	False	0,509449799
ENSMUSG00000023951	Vegfa	3555,831148	927,5657856	5061,657306	1079,618757	554,5184935	0,509423017	False	0,509423017
ENSMUSG00000027185	Nat10	260,4466631	1490,415228	370,7302933	597,1641248	1955,819345	0,509382053	False	0,509382053
ENSMUSG00000051910	Sox6	199,3924008	140,0813635	97,74599182	125,9555216	43,05673008	-0,509345395	False	0,509345395
ENSMUSG00000032580	Rbm5	798,342442	1439,935458	1135,966932	2905,97382	1324,320637	0,508841223	False	0,508841223
ENSMUSG00000037316	Bag4	432,0168684	153,9633005	134,7309617	303,6427753	151,35093	-0,508712601	False	0,508712601
ENSMUSG00000015478	Rnf5	1016,282973	297,8306468	1445,936203	254,160249	193,1029107	0,508701739	False	0,508701739
ENSMUSG00000022008	Gpalpp1	275,1305996	242,3028991	102,1489644	130,4539331	391,424819	0,508618633	False	0,508618633
ENSMUSG00000036053	Fmnl2	155,3405913	299,0926411	109,1937206	398,1094166	118,7321951	-0,508544969	False	0,508544969
ENSMUSG00000003038	Hmgn2	230,3059513	1664,570437	327,5811618	409,3554453	1774,459179	0,508300702	False	0,508300702
ENSMUSG00000027108	Ola1	450,5649987	167,8452374	317,0140275	98,9650527	161,7889252	-0,507188566	False	0,507188566
ENSMUSG00000034269	Setd5	488,4340981	321,8085379	500,1776878	380,1157706	694,1266789	0,507035061	False	0,507035061
ENSMUSG00000037503	Fam168b	481,4785492	300,3546353	150,5816631	129,3293302	683,6886838	0,505868022	False	0,505868022
ENSMUSG00000036372	Tmem258	287,4960198	1031,049315	1118,355041	408,2308424	1110,341736	0,505843201	False	0,505843201
ENSMUSG00000030711	Sult1a1	9293,386123	3033,834216	4630,165991	704,0013976	6545,927722	-0,505606678	False	0,505606678
ENSMUSG00000037242	Clic4	816,8905723	416,4581078	441,1778549	329,5086414	575,3944839	-0,505591443	False	0,505591443
ENSMUSG00000025405	Inhbc	911,9497401	381,1222684	1294,473946	348,6268902	273,9973733	0,505339707	False	0,505339707
ENSMUSG00000058715	Fcer1g	273,584922	720,598726	388,3421837	500,4482779	1232,98818	0,505339707	False	0,505339707
ENSMUSG00000025198	Erlin1	564,9451356	203,1810768	398,0287234	116,9586986	155,2651782	-0,505238222	False	0,505238222
ENSMUSG00000031627	Irf2	544,8513278	248,6128704	383,9392111	219,29756	120,0369445	-0,504984711	False	0,504984711
ENSMUSG00000020267	Hint1	1391,882612	1333,927939	1975,17351	814,2124791	1195,150447	0,504941851	False	0,504941851
ENSMUSG00000022142	Nup155	208,666466	340,7384519	147,059285	334,0070529	322,2731009	-0,504800893	False	0,504800893
ENSMUSG00000021690	Jmy	208,666466	233,4689392	147,059285	255,2848519	390,1200696	-0,504800893	False	0,504800893
ENSMUSG00000026987	Baz2b	245,7627266	526,251609	348,7154303	872,6918284	474,9287803	0,504784076	False	0,504784076
ENSMUSG00000066026	Dhrs3	3604,51999	762,2445367	2540,515193	673,6371201	490,5857731	-0,504686058	False	0,504686058
ENSMUSG00000000743	Chmp1a	530,94023	649,9270471	374,2526714	480,2054262	735,8786596	-0,504536846	False	0,504536846
ENSMUSG00000031246	Sh3bgrl	1260,500022	326,8565149	888,5198715	418,3522682	575,3944839	-0,504520196	False	0,504520196
ENSMUSG00000025885	Myo5b	440,5180948	624,6871617	280,0290576	332,88245	399,2533153	0,5039326	False	0,5039326
ENSMUSG00000025362	Rps26	3693,396447	5574,228674	8439,617888	2604,580251	5279,016058	-0,50389724	False	0,50389724

ENSMUSG00000038970	Lmtk2	139,8838161	148,9153234	125,9250165	82,09600963	198,3219083	0,503615	False	0,503615
ENSMUSG00000030304	Ergic2	670,8240461	1248,112329	951,0420825	1700,399542	1371,291616	0,503574776	False	0,503574776
ENSMUSG00000057278	Snrpg	102,0147167	193,0851227	156,7458247	71,97458378	348,3680889	-0,503217845	False	0,503217845
ENSMUSG00000039474	Wfs1	187,7998194	132,5093979	167,312959	280,026115	604,0989706	-0,503100992	False	0,503100992
ENSMUSG00000038335	Tsr1	123,654202	362,1923544	175,2383097	396,9848137	463,1860358	0,503006937	False	0,503006937
ENSMUSG00000049957	Ccdc137	105,8789105	161,5352661	194,6113891	213,6745456	150,0461806	0,502991341	False	0,502991341
ENSMUSG00000060317	Acnat2	1778,301993	262,4948074	1254,847192	931,1711777	189,1886625	-0,502988653	False	0,502988653
ENSMUSG00000018672	Copz2	480,7057105	387,4322397	1065,51937	544,3077899	339,2348431	-0,502869584	False	0,502869584
ENSMUSG00000020134	Peli1	278,9947934	205,7050654	195,4919836	229,4189858	395,3390671	0,50285233	False	0,50285233
ENSMUSG00000032977	Fam207a	393,3749303	249,8748647	557,4163317	309,2657897	253,1213829	0,502850255	False	0,502850255
ENSMUSG00000036880	Acaa2	7895,320801	2811,723225	5572,402128	1781,370949	2717,792993	-0,502698514	False	0,502698514
ENSMUSG00000011305	Plin5	394,147769	545,181523	278,2678686	565,6752444	284,4353684	-0,502262277	False	0,502262277
ENSMUSG00000022261	Sdc2	3670,211285	964,1636193	2591,589675	600,5379334	713,6979199	-0,502025803	False	0,502025803
ENSMUSG00000024312	Wdr46	258,9009855	466,9378785	398,0287234	366,6205361	371,853578	0,501887143	False	0,501887143
ENSMUSG00000061536	Sec22c	118,2443307	35,33583945	79,25350688	32,61348328	83,50396138	-0,50185447	False	0,50185447
ENSMUSG00000019791	Hint3	217,9405311	191,8231285	365,4467262	182,1856652	153,9604288	-0,50137493	False	0,50137493
ENSMUSG00000036687	Tmem184a	270,493567	35,33583945	191,089011	48,35792348	27,39973733	-0,501349468	False	0,501349468
ENSMUSG00000016128	Stard13	469,8859678	66,88569611	331,9841344	163,0674164	120,0369445	-0,501196389	False	0,501196389
ENSMUSG00000042349	Ikbke	562,6266193	796,3183819	1739,174179	3495,265725	986,3905438	0,501167549	False	0,501167549
ENSMUSG00000061479	Snrpa	668,5055298	629,7351388	844,4901455	472,3332061	734,5739102	-0,50113453	False	0,50113453
ENSMUSG00000003316	Glg1	488,4340981	865,7280666	691,2666989	789,4712159	925,0673221	0,501078507	False	0,501078507
ENSMUSG00000071856	Mcc	398,7848016	201,9190826	281,7902467	195,6808997	104,3799517	-0,500988749	False	0,500988749
ENSMUSG00000038546	Ranbp9	366,3255736	613,3292133	258,8947891	520,6911296	1345,196628	-0,500760493	False	0,500760493
ENSMUSG00000006782	Cnp	244,217049	16,40592546	172,5965261	38,23649764	18,26649155	-0,500760493	False	0,500760493
ENSMUSG00000029817	Tra2a	295,2244074	417,7201021	600,5654632	708,4998091	761,9736476	0,500724573	False	0,500724573
ENSMUSG00000025481	Urah	4443,050047	2217,323926	3140,200061	1771,249523	2195,893234	-0,500693916	False	0,500693916
ENSMUSG00000000739	Sult5a1	411,1502218	17,66791973	290,5961919	62,97776081	63,93272043	-0,50064981	False	0,50064981
ENSMUSG00000114797	Nupl1	108,1974268	109,7935012	95,98480277	76,47299527	216,5883998	-0,500643901	False	0,500643901
ENSMUSG00000063354	Slc39a4	1102,068076	490,9157695	436,7748823	462,2117802	778,9353897	-0,500637772	False	0,500637772
ENSMUSG00000029512	Ulk1	867,1250919	2478,556739	612,8937865	2461,755686	2092,818032	-0,500603053	False	0,500603053
ENSMUSG00000027639	Samhd1	372,5082837	180,4651801	263,2977617	241,7896174	191,7981613	-0,500577251	False	0,500577251
ENSMUSG00000002250	Ppard	629,0907529	295,3066583	444,700233	405,9816367	210,0646528	-0,500434995	False	0,500434995
ENSMUSG00000022973	Synj1	171,5702053	291,5206755	253,611222	412,7292539	242,6833878	0,500276318	False	0,500276318
ENSMUSG00000020708	Psmc5	682,4166276	960,3776365	907,892951	482,4546319	823,2968692	-0,500259516	False	0,500259516
ENSMUSG00000026399	Cd55	150,7035587	32,81185092	106,551937	59,6039522	40,44723129	-0,500156665	False	0,500156665
ENSMUSG00000051232	Tmem199	82,69374762	102,2215356	106,551937	58,47934932	194,4076601	-0,499850994	False	0,499850994
ENSMUSG00000025003	Cyp2c39	179,298593	15,14393119	126,805611	35,98729189	35,22823371	-0,499745582	False	0,499745582
ENSMUSG00000023913	Pla2g7	41,73329319	165,3212489	58,9998329	179,9364595	169,6174215	0,4995121	False	0,4995121
ENSMUSG00000028923	Necap2	144,5208486	246,0888819	204,2979288	363,2467275	305,3113588	0,499396947	False	0,499396947
ENSMUSG00000026048	Ercc5	183,1627868	405,1001594	258,8947891	666,8895029	271,3878745	0,499239507	False	0,499239507
ENSMUSG00000044600	Smim7	308,3626664	422,7680792	291,4767864	218,1729571	549,2994959	-0,49915582	False	0,49915582
ENSMUSG00000054716	Zfp771	186,2541418	204,4430711	201,6561453	275,5277035	131,779689	-0,49914448	False	0,49914448
ENSMUSG00000034957	Cebpa	4133,914542	943,9717111	2925,334998	360,9975218	1650,507987	-0,498906719	False	0,498906719
ENSMUSG00000031333	Abcb7	513,9377773	232,206945	363,6855371	148,4475791	298,7876118	-0,498902148	False	0,498902148
ENSMUSG00000031714	Gab1	175,4343992	87,07760436	124,1638274	66,35156943	54,79947465	-0,498686724	False	0,498686724
ENSMUSG00000031371	Haus7	113,6072981	206,9670596	201,6561453	191,1824882	160,4841758	0,498375534	False	0,498375534
ENSMUSG00000018537	Pcgf2	75,73819875	412,672125	168,1935535	471,2086032	106,9894505	0,498375534	False	0,498375534
ENSMUSG00000042712	Tceal9	171,5702053	200,6570883	215,7456576	121,4571101	271,3878745	-0,49835209	False	0,49835209
ENSMUSG00000025745	Hadha	5282,352944	2370,025232	3606,915157	3740,429151	1929,724357	-0,497976895	False	0,497976895
ENSMUSG00000021846	Peli2	251,1725979	175,417203	177,8800932	136,0769475	152,6556794	-0,497774014	False	0,497774014
ENSMUSG00000022280	Rnf19a	308,3626664	368,5023257	218,3874412	593,7903162	691,5171802	-0,497738214	False	0,497738214
ENSMUSG00000092607	Scnm1	197,0738845	566,6354255	278,2678686	488,0776463	456,6622888	0,497737723	False	0,497737723
ENSMUSG00000048924	Ccdc125	85,01226391	286,4726984	132,0891781	354,2499046	120,0369445	0,497735616	False	0,497735616
ENSMUSG00000028437	Ubap1	479,9328717	493,4397581	339,9094851	483,5792348	729,3549126	-0,49768201	False	0,49768201
ENSMUSG00000028643	Svbp	87,3307802	94,64956996	117,1190713	61,85315794	136,9986866	-0,497642975	False	0,497642975
ENSMUSG00000030879	Mrpl17	476,8415166	822,8202615	815,4305263	692,7553689	673,2506886	0,497633949	False	0,497633949
ENSMUSG00000019988	Nedd1	171,5702053	174,1552087	121,5220439	211,4253399	191,7981613	-0,497580998	False	0,497580998
ENSMUSG00000033543	Gtf2a2	316,8638927	1765,529978	447,3420166	2008,540729	1663,555481	0,497514999	False	0,497514999
ENSMUSG00000096210	H1f0	2784,538062	6368,023067	2154,814793	1972,553437	4742,764056	-0,49737363	False	0,49737363
ENSMUSG00000021327	Zkscan3	874,0806407	648,6650528	1233,712924	998,64735	305,3113588	0,497168438	False	0,497168438
ENSMUSG00000039157	Fam102a	255,8096305	55,52774771	361,0437536	265,4062777	39,1424819	0,497103104	False	0,497103104
ENSMUSG00000030204	Ddx47	391,056414	340,7384519	301,1633261	293,5213495	551,9089947	0,497053652	False	0,497053652
ENSMUSG00000035354	Uvrag	325,3651191	275,11475	290,5961919	230,5435887	545,3852477	-0,497020049	False	0,497020049
ENSMUSG00000074405	Zfp865	230,3059513	636,0451101	390,9839673	325,0102299	430,5673008	0,496933436	False	0,496933436
ENSMUSG00000015214	Mtmr1	281,3133097	194,347117	180,5218768	396,9848137	273,9973733	0,496906007	False	0,496906007
ENSMUSG00000066415	Msl2	351,6416371	201,9190826	228,0739809	146,1983733	249,2071347	-0,496760534	False	0,496760534
ENSMUSG00000042055	Wdr11	432,0168684	334,4284805	367,2079152	609,5347564	354,8918359	0,496620843	False	0,496620843
ENSMUSG00000033831	Fgb	63345,72919	253351,6589	89368,01554	96904,78024	301547,1568	0,496511234	False	0,496511234
ENSMUSG00000046836	Brox	237,2615002	241,0409048	168,1935535	283,3999236	289,654366	-0,496355606	False	0,496355606
ENSMUSG00000030036	Mogs	663,0956585	273,8527558	385,7004001	470,0840003	264,8641275	-0,496298431	False	0,496298431
ENSMUSG00000031231	Cox7b	2007,835106	1785,721887	2831,991979	412,7292539	1920,591112	0,496176387	False	0,496176387
ENSMUSG00000032198	Dock6	487,6612593	468,1998727	1091,937206	975,0306897	345,7585901	-0,496114272	False	0,496114272
ENSMUSG00000019715	Gle1	423,515642	774,8644794	300,2827316	1671,159867	1928,419608	-0,496093743	False	0,496093743
ENSMUSG00000017756	Slc12a7	1337,783898	965,4256136	2580,141946	1977,051848	948,5528113	-0,496045082	False	0,496045082
ENSMUSG00000031365	Zfp275	131,3825897	199,395094	204,2979288	202,4285169	185,2744143	0,495889557	False	0,495889557
ENSMUSG00000021366	Hivep1	244,217049	263,7568016	239,5217097	173,1888422	495,8047707	-0,495817933	False	0,495817933
ENSMUSG00000036639	Nudt1	151,4763975	34,07384519	107,4325315	47,23332061	19,57124095	-0,495662095	False	0,495662095
ENSMUSG00000037992	Rara	314,5453765	306,6646067	594,4013016	264,2816748	223,1121468	-0,495499096	False	0,495499096
ENSMUSG00000034248	Slc25a37	605,1327513	499,7497294	1193,205576	943,5418093	429,2625515	-0,49539135	False	0,49539135
ENSMUSG00000086290	Snhg12	74,96535999	224,6349794	105,6713425	230,5435887	236,1596408	0,495288165	False	0,495288165
ENSMUSG00000038593	Tctn1	74,96535999	180,4651801	105,6713425	281,1507179	148,7414312	0,495288165	False	0,495288165
ENSMUSG00000020173	Cobl	208,666466	820,296273	294,11857	558,9276272	353,5870865	0,495199107	False	0,495199107
ENSMUSG00000070436	Serpinh1	208,666466	106,0075184	294,11857	102,3388613	131,779689	0,495199107	False	0,495199107
ENSMUSG00000019188	H13	2292,23977	5848,081429	3230,901297	3516,63318	7867,638861	0,495178718	False	0,495178718
ENSMUSG00000030494	Rhpn2	266,6293732	26,50187959	147,059285	93,34203834	189,1886625	-0,495010088	False	0,495010088
ENSMUSG00000020650	Bcap29	129,0640734	186,7751514	91,58183017	103,4634642	202,2361565	-0,494954163	False	0,494954163
ENSMUSG00000020519	Sap30l	220,2590474	324,3325264	222,7904138	156,3197992	264,8641275	-0,494700762	False	0,494700762
ENSMUSG00000028107	Tars2	303,7256338	449,2699587	508,1030385	746,7363068	427,9578021	0,494699876	False	0,494699876
ENSMUSG00000031577	Tti2	158,4319464	167,8452374	169,074148	112,4602872	289,654366	-0,494447635	False	0,494447635
ENSMUSG00000040423	Rc3h1	200,9380783	222,1109908	142,6563124	211,4253399	358,806084	-0,494207399	False	0,494207399
ENSMUSG00000044792	Isca1	522,4390037	379,8602741	277,3872741	307,016584	735,8786596	0,494205289	False	0,494205289
ENSMUSG00000006527	Sfmbt1	260,4466631	355,8823831	184,9248494	373,3681534	310,5303564	-0,494048852	False	0,494048852
ENSMUSG00000060152	Pop5	193,2096907	133,7713922	272,1037069	145,0737704	91,33245776	0,493989155	False	0,493989155
ENSMUSG00000003269	Cyth2	245,7627266	691,5728578	346,0736467	440,8443257	850,6966065	0,493812958	False	0,493812958
ENSMUSG00000018428	Akap1	1289,095056	634,7831159	1493,488307	1815,109035	146,1319324	0,493697564	False	0,493697564
ENSMUSG00000034620	Tmem5	195,528207	212,0150367	136,4921507	103,4634642	275,3021227	0,493638987	False	0,493638987
ENSMUSG00000017774	Myo1c	492,2982919	489,6537753	526,5955235	672,5125172	349,6728383	-0,493526999	False	0,493526999
ENSMUSG00000024122	Pdpk1	292,9058911	199,395094	119,7608548	208,0515312	99,16095413	-0,493496303	False	0,493496303
ENSMUSG00000021484	Lman2	3006,342787	1159,772731	2072,919502	984,0275127	2135,874762	-0,49318246	False	0,49318246
ENSMUSG00000039968	Rsbn1l	157,6591076	330,6424977	221,9098193	533,0617611	485,3667755	0,493164993	False	0,493164993
ENSMUSG00000032599	Ip6k2	556,4439092	348,3104175	242,1634932	376,741962	395,3390671	-0,49314574	False	0,49314574
ENSMUSG00000033885	Pxk	483,0242268	343,2624404	462,3121234	641,0236368	550,6042453	-0,492783544	False	0,492783544
ENSMUSG00000046959	Slc26a1	4098,363959	567,8974198	5767,013517	807,4648618	238,7691396	0,492776294	False	0,492776294
ENSMUSG00000022781	Pak2	340,8218944	479,5578211	189,327822	375,6173591	90,02770836	0,492686757	False	0,492686757
ENSMUSG00000016409	Nkap	106,6517493	180,4651801	150,5816631	202,4285169	150,0461806	0,492498967	False	0,492498967
ENSMUSG00000028277	Ube2j1	471,4316453	702,9308062	508,1030385	335,1316557	1530,471042	-0,492320638	False	0,492320638
ENSMUSG00000021646	Mccc2	962,1842597	113,579484	684,2219427	332,88245	232,2453926	-0,491848827	False	0,491848827
ENSMUSG00000032560	Dnajc13	291,3602136	200,6570883	168,1935535	253,0356461	409,6913105	0,491733409	False	0,491733409
ENSMUSG00000068663	Clec16a	274,3577608	285,2107041	193,7307946	385,738785	208,7599034	0,491565826	False	0,491565826
ENSMUSG00000058638	Zfp110	429,6983521	300,3546353	250,9694384	264,2816748	604,0989706	0,491460684	False	0,491460684
ENSMUSG00000037533	Rapgef6	207,8936272	203,1810768	115,3578822	131,578536	292,2638648	0,491425933	False	0,491425933
ENSMUSG00000044080	S100a1	1802,259995	1586,326793	2533,470437	907,5545174	1567,004025	0,491307844	False	0,491307844
ENSMUSG00000029136	Rbks	165,3874952	61,83771904	232,4769535	77,59759814	43,05673008	0,491237544	False	0,491237544

ENSMUSG00000015291	Gdi1	1174,714919	1140,842817	1651,114727	845,7013595	748,9261536	0,491129683	False	0,491129683
ENSMUSG00000044026	Slc35g1	214,0763373	326,8565149	152,3428521	283,3999236	277,9116215	-0,490803527	False	0,490803527
ENSMUSG00000053799	Exoc6	258,1281468	270,066773	151,4622576	146,1983733	362,7203322	0,490770153	False	0,490770153
ENSMUSG00000024339	Tap2	325,3651191	126,1994266	231,596359	136,0769475	189,1886625	-0,490447022	False	0,490447022
ENSMUSG00000024038	Ndufv3	1022,465683	612,0672191	1436,249664	384,6141821	598,879973	0,490254132	False	0,490254132
ENSMUSG00000018326	Ywhab	1973,057361	3478,056197	1948,755675	1404,628987	4232,607042	-0,490243786	False	0,490243786
ENSMUSG00000005882	Uqcc1	384,8737039	193,0851227	247,4470604	160,8182106	273,9973733	-0,490223041	False	0,490223041
ENSMUSG00000069769	Msi2	193,2096907	174,1552087	109,1937206	98,9650527	271,3878745	0,490188807	False	0,490188807
ENSMUSG00000020755	Sap30bp	189,3454969	477,0338326	265,9395453	411,604651	803,7256283	0,490077212	False	0,490077212
ENSMUSG00000002804	Nudt14	113,6072981	99,69754702	272,9843015	152,9459905	80,89446258	-0,489942661	False	0,489942661
ENSMUSG00000046982	Tshz1	257,355308	1060,075184	446,4614221	711,8736177	361,4155828	0,489897172	False	0,489897172
ENSMUSG00000027035	Cers6	340,0490556	378,5982798	126,805611	109,0864785	477,5382791	0,489873498	False	0,489873498
ENSMUSG00000031974	Abcb10	327,6836354	58,05173624	233,357548	77,59759814	32,61873491	-0,489761494	False	0,489761494
ENSMUSG00000026104	Stat1	229,5331126	135,0333865	322,2975946	138,3261532	110,9036987	0,489691129	False	0,489691129
ENSMUSG00000020527	Myo19	156,1134301	372,2883085	314,3722439	381,2403735	219,1978986	0,489639313	False	0,489639313
ENSMUSG00000008730	Hipk1	679,3252725	600,7092707	360,163159	585,9180961	953,7718088	0,489541614	False	0,489541614
ENSMUSG00000038552	Fndc4	622,9080428	874,5620264	542,4462248	328,3840385	684,9934332	0,489541508	False	0,489541508
ENSMUSG00000046962	Zbtb21	193,2096907	537,6095574	271,2231124	394,7356079	953,7718088	0,489312667	False	0,489312667
ENSMUSG00000060679	Mrps9	206,3479497	253,6608475	153,2234466	110,2110814	289,654366	0,489253308	False	0,489253308
ENSMUSG00000027465	Tbc1d20	370,9626061	528,7755975	291,4767864	264,2816748	768,4973945	-0,489197375	False	0,489197375
ENSMUSG00000003199	Mpnd	986,9151001	537,6095574	1385,175181	608,4101535	438,3957972	0,489070557	False	0,489070557
ENSMUSG00000017390	Aldoc	136,792461	30,28786239	191,9696055	25,86586605	32,61873491	0,488889186	False	0,488889186
ENSMUSG00000079084	Ccdc82	156,8862688	530,0375918	220,1486302	1337,152814	336,6253443	0,488758779	False	0,488758779
ENSMUSG00000036737	Oxsr1	334,6391843	238,5169163	186,6860384	228,2943829	159,1794264	-0,488514797	False	0,488514797
ENSMUSG00000023118	Sympk	836,2115414	970,4735907	596,1624906	781,5989958	1082,941999	-0,488162349	False	0,488162349
ENSMUSG00000034781	Gna11	683,1894663	494,7017523	958,0868387	741,1132924	323,5778503	0,487870693	False	0,487870693
ENSMUSG00000052459	Atp6v1a	428,1526746	244,8268876	227,1933864	188,9332824	305,3113588	-0,487844073	False	0,487844073
ENSMUSG00000025477	Inpp5a	227,2145963	275,11475	162,0293918	284,5245265	375,7678262	-0,487799977	False	0,487799977
ENSMUSG00000031703	Itfg1	991,5521327	605,7572477	576,7894112	473,457809	707,1741729	-0,487623041	False	0,487623041
ENSMUSG00000003228	Grk5	119,7900082	58,05173624	52,83567125	85,46981824	57,40897345	-0,487020617	False	0,487020617
ENSMUSG00000037286	Stag1	370,1897674	721,8607202	264,1783563	419,4768711	1056,847011	-0,486752744	False	0,486752744
ENSMUSG00000060636	Rpl35a	1252,771635	1644,378529	1968,128754	894,0592829	1471,757319	-0,486681051	False	0,486681051
ENSMUSG00000037795	N4bp2	372,5082837	161,5352661	151,4622576	244,0388231	521,8997586	0,486500227	False	0,486500227
ENSMUSG00000026131	Dst	784,4313442	1650,6885	1098,981962	1518,213877	1724,878702	0,486448618	False	0,486448618
ENSMUSG00000033174	Mgll	3248,24132	5114,862761	5602,342342	4229,6314	2318,539678	-0,486442402	False	0,486442402
ENSMUSG00000029004	Kmt2e	1058,016266	2126,460338	1535,756844	2562,969944	1482,195314	0,486373761	False	0,486373761
ENSMUSG00000046756	Mrps7	658,4586259	680,2149094	809,2663647	470,0840003	681,079185	-0,486174214	False	0,486174214
ENSMUSG00000022272	Myo10	330,7749905	185,5131571	208,7009014	154,0705934	236,1596408	-0,486087726	False	0,486087726
ENSMUSG00000057346	Apol9a	1224,1766	70,6716789	1714,517532	84,34521537	221,8073974	0,48599096	False	0,48599096
ENSMUSG00000039201	Tbc1d25	68,00981113	169,1072317	173,4771206	185,5594738	95,24670595	0,485926313	False	0,485926313
ENSMUSG00000032870	Smap2	683,1894663	681,4769037	487,8493646	711,8736177	1015,09503	-0,485849982	False	0,485849982
ENSMUSG00000002812	Flii	710,238823	874,5620264	507,222444	760,2315412	1020,314028	-0,485685638	False	0,485685638
ENSMUSG00000029864	Gstk1	1595,139206	555,2774771	1139,48931	287,8983351	726,7454139	-0,485294942	False	0,485294942
ENSMUSG00000028700	Pomgnt1	233,3973064	466,9378785	326,7005672	547,6815985	591,0514766	0,485181049	False	0,485181049
ENSMUSG00000000194	Gpr107	240,3528552	249,8748647	171,7159316	295,7705552	315,749354	-0,485130045	False	0,485130045
ENSMUSG00000031059	Ndufb11	2146,946083	2017,928832	2874,260516	1533,958317	2175,017244	-0,485026681	False	0,485026681
ENSMUSG00000006728	Cdk4	470,6588065	379,8602741	658,6847016	272,1538949	225,7216456	0,484906453	False	0,484906453
ENSMUSG00000022364	Tbc1d31	130,6097509	53,00375918	73,96993975	93,34203834	54,79947465	-0,484663731	False	0,484663731
ENSMUSG00000020307	Cdc34	742,6980511	772,3404909	1039,101535	340,7546701	818,0778716	0,484488935	False	0,484488935
ENSMUSG00000057130	Txnl4a	88,10361896	63,09971331	189,327822	62,97776081	84,80871077	-0,484358818	False	0,484358818
ENSMUSG00000039671	Zmynd8	614,4068164	265,0187959	859,4602524	734,3656752	143,5224336	0,484236699	False	0,484236699
ENSMUSG00000017428	Psmd11	1534,857783	1511,869131	1098,981962	971,6568811	2146,312757	0,483755334	False	0,483755334
ENSMUSG00000022051	Bnip3l	1007,008908	1763,00599	720,3263181	1295,542508	1807,077914	-0,483353924	False	0,483353924
ENSMUSG00000026874	Hc	9607,931499	2565,634343	13431,70823	7504,474962	1495,242808	0,483345026	False	0,483345026
ENSMUSG00000020074	Ccar1	667,7326911	2382,645174	933,4301921	4307,228998	2659,07927	0,483271459	False	0,483271459
ENSMUSG00000022811	Zfp148	411,1502218	275,11475	294,11857	227,1697801	281,8258696	-0,483267732	False	0,483267732
ENSMUSG00000025266	Gnl3l	139,8838161	235,9929278	125,9250165	100,0896556	183,9696649	-0,482936182	False	0,482936182
ENSMUSG00000013622	Atraid	845,4856065	465,6758842	604,9684358	603,9117421	444,9195442	-0,482920323	False	0,482920323
ENSMUSG00000038759	Nup205	193,2096907	426,554062	138,2533398	273,2784978	812,858874	-0,482853124	False	0,482853124
ENSMUSG00000035851	Ythdc1	361,688541	819,0342787	505,461255	966,0338667	1722,269203	0,482852618	False	0,482852618
ENSMUSG00000059291	Rpl11	2156,220148	3936,160116	4395,927848	1542,95514	3612,851079	-0,482808366	False	0,482808366
ENSMUSG00000054792	Klhl18	122,8813633	249,8748647	171,7159316	208,0515312	234,8548914	0,48275777	False	0,48275777
ENSMUSG00000024187	Fam234a	700,9647579	630,9971331	750,2665318	979,5291012	435,7862984	0,482746442	False	0,482746442
ENSMUSG00000044066	Cep68	187,0269806	50,47977065	133,8503672	66,35156943	9,133245776	-0,482625312	False	0,482625312
ENSMUSG00000036216	Leap2	674,6882399	942,7097168	646,3563783	292,3967466	673,2506886	0,482592583	False	0,482592583
ENSMUSG00000030091	Nup210	203,2565946	283,9487099	369,8496988	494,8252635	301,3971106	0,482328196	False	0,482328196
ENSMUSG00000036534	Slc38a7	437,4267397	417,7201021	630,5056769	743,3624981	313,1398552	-0,482234274	False	0,482234274
ENSMUSG00000036570	Fxyd1	5442,330568	1530,799045	7602,172499	1369,766298	2732,145236	0,482187173	False	0,482187173
ENSMUSG00000003166	Dgcr2	488,4340981	251,136859	313,4916494	265,4062777	349,6728383	-0,482158185	False	0,482158185
ENSMUSG00000047514	Tspyl1	269,7207282	179,2031858	132,0891781	154,0705934	193,1029107	-0,482096487	False	0,482096487
ENSMUSG00000052812	Atad2b	201,7109171	194,347117	144,4175014	213,6745456	366,6345804	-0,482043581	False	0,482043581
ENSMUSG00000031913	Vps4a	215,6220148	575,4693854	301,1633261	396,9848137	862,4393511	0,482041614	False	0,482041614
ENSMUSG00000035476	Tab3	114,3801369	169,1072317	81,89529044	134,9523446	298,7876118	-0,481984143	False	0,481984143
ENSMUSG00000055319	Sec23ip	249,6269204	177,9411915	140,0145288	348,6268902	198,3219083	0,481910325	False	0,481910325
ENSMUSG00000042111	Ccdc115	147,6122037	214,5390252	155,8652302	105,7126699	395,3390671	-0,481663702	False	0,481663702
ENSMUSG00000052155	Acvr2a	297,5429237	509,8456835	213,103874	343,0038758	639,3272043	-0,481540987	False	0,481540987
ENSMUSG00000032583	Mon1a	299,0886012	512,3696721	479,9240139	834,4553307	417,5198069	0,481271712	False	0,481271712
ENSMUSG00000028470	Hint2	602,814235	639,8310929	1364,040913	490,326852	431,8720502	-0,481109534	False	0,481109534
ENSMUSG00000030795	Fus	959,0929046	898,5399175	668,3712413	340,7546701	1338,672881	0,481060989	False	0,481060989
ENSMUSG00000022185	Acin1	1116,752012	2009,094872	1558,652302	2766,523064	2188,064738	0,480990279	False	0,480990279
ENSMUSG00000030760	Acer3	255,8096305	372,2883085	190,2084165	183,3102681	1122,084481	-0,480782978	False	0,480782978
ENSMUSG00000078201	Tmem203	103,5603942	133,7713922	129,4473946	74,22378953	122,6464433	-0,480518797	False	0,480518797
ENSMUSG00000034290	Nek9	531,7130688	381,1222684	517,7895783	832,206125	490,5857731	-0,480394021	False	0,480394021
ENSMUSG00000004285	Atp6v1f	731,1054696	1465,175343	1262,772543	524,0649382	1790,116172	-0,480333954	False	0,480333954
ENSMUSG00000015488	Cacfd1	272,8120833	617,1151961	380,416833	554,4292157	816,7731222	0,47967354	False	0,47967354
ENSMUSG00000097328	Tnfsf12	108,1974268	170,3692259	257,1336001	77,59759814	133,0844384	-0,479582286	False	0,479582286
ENSMUSG00000029298	Gbp9	58,73574597	166,5832431	81,89529044	364,3713304	259,6451299	0,479541709	False	0,479541709
ENSMUSG00000038312	Edem2	798,342442	1113,078943	1383,413992	1946,687571	1539,604288	0,479476301	False	0,479476301
ENSMUSG00000026499	Acbd3	274,3577608	321,8085379	213,9844686	196,8055025	901,581833	-0,479287828	False	0,479287828
ENSMUSG00000040564	Apoc1	52811,93686	55128,95752	73618,58254	24683,90843	52020,35844	0,47920592	False	0,47920592
ENSMUSG00000022634	Yaf2	221,0318862	422,7680792	229,8351699	158,5690049	587,1372284	-0,47914371	False	0,47914371
ENSMUSG00000044857	Lemd2	252,7182754	691,5728578	352,2378083	567,9244502	735,8786596	0,479019776	False	0,479019776
ENSMUSG00000026972	Arrdc1	61,82710102	137,557375	97,74599182	262,0324691	44,36147948	-0,47893188	False	0,47893188
ENSMUSG00000078713	Tomm5	181,6171093	143,8673463	222,7904138	66,35156943	253,1213829	0,478929269	False	0,478929269
ENSMUSG00000020271	Fbxw11	340,8218944	261,2328131	147,059285	215,9237514	474,9287803	0,478693168	False	0,478693168
ENSMUSG00000024400	Wdr33	377,918155	873,3000322	271,2231124	683,7585459	1416,957845	-0,478593703	False	0,478593703
ENSMUSG00000032536	Trak1	861,7152205	1505,55916	1146,534066	1869,089973	618,4512139	-0,478551373	False	0,478551373
ENSMUSG00000048856	Slc25a47	2867,23181	12484,90927	2057,949395	6788,102933	24539,72665	-0,478451041	False	0,478451041
ENSMUSG00000071660	Ttc9c	246,5355653	121,1514495	176,9994987	83,2206125	58,71372284	-0,478050511	False	0,478050511
ENSMUSG00000054008	Ndst1	2240,459573	983,0935333	911,4153291	642,1482397	1608,756006	-0,47784916	False	0,47784916
ENSMUSG00000036160	Surf6	83,46658638	190,5611342	116,2384768	127,0801245	263,5593781	0,477817029	False	0,477817029
ENSMUSG00000053510	Nrd1	1340,875253	1577,492833	1319,130592	3873,13229	962,9050546	-0,477709568	False	0,477709568
ENSMUSG00000034422	Parp14	474,5230004	103,4835298	340,7900796	251,9110432	70,45646741	-0,477594678	False	0,477594678
ENSMUSG00000003617	Cp	26387,03388	57940,68075	36741,04519	78277,98288	70703,06505	0,477563479	False	0,477563479
ENSMUSG00000039982	Dtx4	284,4046647	169,1072317	204,2979288	143,9491676	194,4076601	-0,47727055	False	0,47727055
ENSMUSG00000030890	Ilk	818,4362498	758,4585539	929,0272195	1139,222709	364,0250816	0,477107859	False	0,477107859
ENSMUSG00000024095	Hnrnpll	343,1404107	620,9011789	246,5664658	572,4228617	572,7849851	-0,476822438	False	0,476822438
ENSMUSG00000025194	Abcc2	7523,585356	1687,286334	5406,850358	3560,492692	786,7638861	-0,476631912	False	0,476631912
ENSMUSG00000035949	Fbxw2	336,9577006	473,2478498	242,1634932	507,1958951	904,1913318	-0,476586105	False	0,476586105
ENSMUSG00000016528	Mapkapk2	487,6612593	863,204078	350,4766193	517,3173209	1556,56603	-0,476561163	False	0,476561163
ENSMUSG00000015839	Nfe2l2	1203,309954	647,4030585	799,5798249	786,0974073	865,0488499	-0,476154796	False	0,476154796
ENSMUSG00000028676	Srsf10	433,5625459	434,1260276	311,7304604	475,7070147	538,8615008	-0,475940999	False	0,475940999
ENSMUSG00000026333	Gin1	105,1060717	276,3767443	146,1786905	435,2213113	198,3219083	0,475887001	False	0,475887001

ENSMUSG00000036698	Ago2	253,4911142	560,3254542	182,2830658	413,8538568	660,2031946	-0,475754636	False	0,475754636
ENSMUSG00000022031	Elp3	261,2195018	196,8711055	139,1339343	363,2467275	285,7401178	0,475687193	False	0,475687193
ENSMUSG00000015002	Efr3a	624,4537203	747,1006056	449,1032056	659,0172828	729,3549126	-0,475547635	False	0,475547635
ENSMUSG00000028156	Eif4e	603,5870737	975,5215677	507,222444	434,0967084	1857,963141	-0,475545427	False	0,475545427
ENSMUSG00000024812	Tjp2	341,5947332	681,4769037	245,6858713	548,8062014	476,2335297	-0,475470836	False	0,475470836
ENSMUSG00000059439	Bcas3	277,4491158	251,136859	260,6559782	385,738785	204,8456553	0,475401007	False	0,475401007
ENSMUSG00000021670	Hmgcr	1692,516891	562,8494427	2352,94856	400,3586223	387,5105708	0,475299549	False	0,475299549
ENSMUSG00000056978	Hamp2	6214,396492	1071,433132	80,1341014	33,73808615	8638,745754	0,475207574	False	0,475207574
ENSMUSG00000079658	Eloc	343,9132494	648,6650528	496,6553098	247,4126318	790,6781343	-0,475125538	False	0,475125538
ENSMUSG00000001767	Cmkl1	166,160334	151,4393119	87,17885756	122,581713	230,9406432	0,474946074	False	0,474946074
ENSMUSG00000025176	Hoga1	1697,153923	2477,294744	2624,171672	1221,318719	2416,395882	-0,474677677	False	0,474677677
ENSMUSG00000031885	Cbfb	117,4714919	184,2511629	84,537074	141,6999618	276,6068721	-0,474654601	False	0,474654601
ENSMUSG00000027660	Skil	161,5233014	504,7977065	116,2384768	375,6173591	529,728255	-0,474654601	False	0,474654601
ENSMUSG00000025609	Mkln1	862,4880593	1561,086907	620,8191372	1461,983733	1625,717748	-0,474331453	False	0,474331453
ENSMUSG00000063952	Brpf3	479,9328717	985,6175219	1128,041581	2235,710509	666,7269416	0,474263396	False	0,474263396
ENSMUSG00000021275	Tecpr2	152,2492363	276,3767443	178,7606877	276,6523064	109,5989493	-0,474201023	False	0,474201023
ENSMUSG00000070520	Nsmce3	178,5257542	131,2474037	146,1786905	48,35792348	247,9023853	0,47363994	False	0,47363994
ENSMUSG00000042992	Borcs5	110,5159431	141,3433578	167,312959	121,4571101	79,58971319	-0,473600626	False	0,473600626
ENSMUSG00000048826	Dact2	283,6318259	23,97789106	204,2979288	86,59442111	7,828496379	-0,473344846	False	0,473344846
ENSMUSG00000042364	Snx18	243,4442103	215,8010195	169,074148	150,6967848	337,9300937	0,473133642	False	0,473133642
ENSMUSG00000028333	Anp32b	584,2661047	790,0084106	420,924181	569,049053	833,7348644	-0,473065205	False	0,473065205
ENSMUSG00000035173	Ccdc186	172,3430441	257,4468303	124,1638274	278,9015122	682,3839344	-0,473038136	False	0,473038136
ENSMUSG00000029575	Mmab	871,7621244	78,2436445	1209,936872	93,34203834	24,79023853	0,472925348	False	0,472925348
ENSMUSG00000009828	Ick	396,4662853	315,4985665	467,5956906	810,8386704	285,7401178	-0,472494583	False	0,472494583
ENSMUSG00000020253	Ppm1m	166,160334	73,19566744	204,2979288	230,5435887	20,87599034	0,472463524	False	0,472463524
ENSMUSG00000056131	Pgm3	149,1578812	339,4764576	271,2231124	206,9269284	503,6332671	0,472281193	False	0,472281193
ENSMUSG00000066798	Zbtb6	99,6962004	73,19566744	51,95507673	33,73808615	138,303436	0,472226572	False	0,472226572
ENSMUSG00000060739	Nsa2	915,8139339	557,8014656	473,7598522	383,4895792	660,2031946	-0,472144396	False	0,472144396
ENSMUSG00000032609	Klhdc8b	159,2047851	531,299586	496,6553098	1653,166221	114,8179469	-0,471535535	False	0,471535535
ENSMUSG00000020840	Blmh	334,6391843	190,5611342	201,6561453	161,9428135	241,3786384	-0,471308381	False	0,471308381
ENSMUSG00000026272	Agxt	4270,707003	748,3625998	1850,129088	607,2855507	3080,513325	-0,471304147	False	0,471304147
ENSMUSG00000028729	Ebna1bp2	271,2664057	434,1260276	250,9694384	195,6808997	682,3839344	-0,47120745	False	0,47120745
ENSMUSG00000032014	Oaf	2731,985027	551,4914943	1970,770538	808,5894647	331,4063467	-0,471189767	False	0,471189767
ENSMUSG00000022550	Adck5	363,2342185	83,29162157	261,5365727	262,0324691	65,23746983	-0,471154528	False	0,471154528
ENSMUSG00000028234	Rps20	1779,074832	3326,616886	3846,436867	2549,47471	2465,976359	0,471031774	False	0,471031774
ENSMUSG00000051154	Commd3	819,2090886	440,4359989	545,0880084	405,9816367	591,0514766	-0,470947936	False	0,470947936
ENSMUSG00000057541	Pus7	166,160334	119,8894553	197,2531727	206,9269284	217,8931492	-0,470871247	False	0,470871247
ENSMUSG00000038127	Ccdc50	670,8240461	435,3880218	341,6706741	236,166603	484,0620261	-0,470742483	False	0,470742483
ENSMUSG00000022314	Rad21	638,3648181	402,5761709	244,8052768	240,6650145	884,6200908	0,470676865	False	0,470676865
ENSMUSG00000024594	Prrc1	440,5180948	638,5690987	317,894622	408,2308424	1080,3325	-0,470652671	False	0,470652671
ENSMUSG00000036019	Tmtc2	69,55548865	234,7309335	50,19388769	60,72855507	92,63720715	-0,470652671	False	0,470652671
ENSMUSG00000017999	Ddx27	301,4071175	506,0597007	217,5068467	480,2054262	688,9076814	-0,470652671	False	0,470652671
ENSMUSG00000014077	Chp1	4096,045443	1831,15368	2157,456576	1000,896556	2956,562132	-0,470311125	False	0,470311125
ENSMUSG00000028820	Sfpq	492,2982919	1140,842817	420,924181	355,3745074	1803,163666	-0,47019254	False	0,47019254
ENSMUSG00000022723	Crybg3	189,3454969	126,1994266	87,17885756	113,58489	262,2546287	0,469947125	False	0,469947125
ENSMUSG00000032412	Atp1b3	445,1551274	526,251609	321,4170001	341,879273	785,4591367	-0,469861936	False	0,469861936
ENSMUSG00000050931	Sgms2	518,5748098	70,6716789	163,7905809	83,2206125	374,4630768	-0,469728656	False	0,469728656
ENSMUSG00000062203	Gspt1	1096,658204	713,0267604	586,4759509	764,7299527	791,9828837	-0,469572795	False	0,469572795
ENSMUSG00000052056	Zfp217	144,5208486	103,4835298	103,0295589	82,09600963	104,3799517	-0,469432992	False	0,469432992
ENSMUSG00000039246	Lyplal1	236,4886614	27,76387386	170,835337	65,22696655	24,79023853	-0,469164589	False	0,469164589
ENSMUSG00000032175	Tyk2	370,1897674	494,7017523	515,1477947	1060,500508	267,4736263	-0,468868373	False	0,468868373
ENSMUSG00000035765	Dym	422,7428033	156,487289	305,5662987	212,5499427	109,5989493	-0,468294759	False	0,468294759
ENSMUSG00000037025	Foxa2	490,7526144	535,0855688	678,9383756	488,0776463	157,874677	0,46828468	False	0,46828468
ENSMUSG00000030123	Plxnd1	344,6860882	214,5390252	433,2525043	476,8316176	67,84696862	0,468196829	False	0,468196829
ENSMUSG00000041241	Mul1	354,7329921	219,5870023	211,342685	242,9142203	490,5857731	0,467771881	False	0,467771881
ENSMUSG00000053641	Dennd4a	401,8761567	328,1185092	290,5961919	494,8252635	781,5448885	-0,467735187	False	0,467735187
ENSMUSG00000026409	Pfkfb2	57,96290721	784,9604335	80,1341014	1008,768776	172,2269203	0,467286361	False	0,467286361
ENSMUSG00000032386	Trip4	406,5131892	294,044664	479,0434193	407,1062395	550,6042453	-0,467266851	False	0,467266851
ENSMUSG00000079197	Psme2	931,2707092	605,7572477	1287,429189	301,3935696	583,2229802	0,467220576	False	0,467220576
ENSMUSG00000003662	Ciao1	429,6983521	254,9228418	310,8498659	157,444402	178,7506673	-0,467106288	False	0,467106288
ENSMUSG00000115338	Pnp	840,0757352	624,6871617	700,9532386	283,3999236	1161,226963	0,467058674	False	0,467058674
ENSMUSG00000027180	Fbxo3	1558,042946	383,6462569	1127,160987	594,9149191	425,3483033	-0,467041417	False	0,467041417
ENSMUSG00000039105	Atp6v1g1	547,1698441	1527,013062	757,3112879	395,8602108	1628,327247	-0,466997657	False	0,466997657
ENSMUSG00000056201	Cfl1	3153,954991	5668,878243	4416,181522	2281,819227	9195,873747	-0,466977571	False	0,466977571
ENSMUSG00000039997	Ifi203	214,0763373	82,0296273	295,879759	239,5404117	46,97097827	0,466885668	False	0,466885668
ENSMUSG00000024457	Trim26	740,3795348	490,9157695	1023,250833	837,8291394	534,9472526	0,466822919	False	0,466822919
ENSMUSG00000073889	Il11ra1	497,7081632	126,1994266	360,163159	191,1824882	91,33245776	-0,466649434	False	0,466649434
ENSMUSG00000063171	Rps4l	200,1652396	203,1810768	145,2980959	20,24285169	276,6068721	0,46664554	False	0,46664554
ENSMUSG00000028138	Adh5	3685,66788	1222,872444	2667,320804	663,5156943	1672,688726	-0,466534793	False	0,466534793
ENSMUSG00000029179	Zcchc4	98,15052288	350,834406	135,6115562	609,5347564	318,3588527	0,466412265	False	0,466412265
ENSMUSG00000033184	Tmed7	1330,82835	918,7318257	646,3563783	443,0935314	1838,3919	0,466119842	False	0,466119842
ENSMUSG00000036391	Sec24a	932,0435479	691,5728578	746,7441537	674,761723	2235,035716	-0,466019227	False	0,466019227
ENSMUSG00000019039	Dalrd3	484,5699043	350,834406	984,5046743	481,3300291	405,7770623	-0,465914566	False	0,465914566
ENSMUSG00000032673	Prorsd1	163,8418177	118,6217461	115,3578822	61,85315794	82,19921198	-0,465865609	False	0,465865609
ENSMUSG00000070644	Etnk2	2455,308749	3390,978593	2575,738974	636,5252253	1895,800873	0,465797225	False	0,465797225
ENSMUSG00000071711	Mpst	1017,828651	89,6015929	737,057614	64,10236368	147,4366818	-0,465645406	False	0,465645406
ENSMUSG00000022178	Ajuba	156,8862688	56,78974198	216,6262521	156,3197992	19,57124095	0,465489	False	0,465489
ENSMUSG00000020829	Slc46a1	572,6735232	37,85982798	414,7600193	49,48252635	54,79947465	-0,465436071	False	0,465436071
ENSMUSG00000021932	Rnaseh2b	60,2814235	142,6053521	111,8355041	83,2206125	134,3891878	0,465227421	False	0,465227421
ENSMUSG00000028410	Dnaja1	2079,709111	502,2737179	1506,697225	449,8411486	437,0910478	-0,46499222	False	0,46499222
ENSMUSG00000030220	Arhgdib	181,6171093	59,31373051	60,76102194	131,578536	84,80871077	-0,464975953	False	0,464975953
ENSMUSG00000021254	Gpatch2l	142,2023324	193,0851227	103,0295589	463,3363831	110,9036987	-0,464886825	False	0,464886825
ENSMUSG00000063428	Ddo	309,9083439	107,2695126	224,5516028	48,35792348	71,76121681	-0,464794578	False	0,464794578
ENSMUSG00000001910	Nacc1	588,1302985	962,9016251	764,3560441	426,2244883	918,5435751	-0,464522331	False	0,464522331
ENSMUSG00000029780	Nt5c3	262,7651794	161,5352661	176,9994987	170,9396365	190,4934119	-0,464033004	False	0,464033004
ENSMUSG00000026743	Mllt10	385,6465426	320,5465436	499,2970933	531,9371583	165,7031734	0,463976642	False	0,463976642
ENSMUSG00000034177	Rnf43	357,0515084	113,579484	258,8947891	249,6618375	93,94195655	-0,463766285	False	0,463766285
ENSMUSG00000059552	Trp53	401,1033179	235,9929278	553,0133591	269,9046892	84,80871077	0,463340432	False	0,463340432
ENSMUSG00000053565	Eif3k	502,3451958	641,0930872	744,9829646	364,3713304	600,1847224	-0,463269631	False	0,463269631
ENSMUSG00000034429	Zfp707	704,8289517	617,1151961	745,8635592	971,6568811	482,7572767	0,463173762	False	0,463173762
ENSMUSG00000090733	Rps27	1988,514137	6140,864099	4143,197221	2740,657198	4271,749524	0,462831068	False	0,462831068
ENSMUSG00000036120	Rfxank	501,5723571	1288,496146	691,2666989	1154,967149	2912,200653	0,462784587	False	0,462784587
ENSMUSG00000022677	Fopnl	170,7973666	99,6954702	122,4026384	80,97140676	123,9511927	-0,462513578	False	0,462513578
ENSMUSG00000042496	Prdm10	166,160334	47,95578211	228,9545754	86,59442111	49,58047707	0,462485375	False	0,462485375
ENSMUSG00000022235	Cmbl	2088,210337	329,3805035	1515,50317	459,9625745	454,05279	-0,462470166	False	0,462470166
ENSMUSG00000010554	Mettl16	159,9776239	98,43555276	108,3131261	70,84998091	116,1226963	-0,462220153	False	0,462220153
ENSMUSG00000020072	Pbld2	2280,647189	18,92991399	1655,517699	66,35156943	79,58971319	-0,462160848	False	0,462160848
ENSMUSG00000030654	Arl6ip1	1367,151771	721,8607202	992,430025	278,9015122	614,5369658	-0,462136121	False	0,462136121
ENSMUSG00000003949	Hlf	479,1600329	267,5427844	347,8348357	130,4539331	176,1411685	-0,462105151	False	0,462105151
ENSMUSG00000038742	Angptl6	183,1627868	35,33583945	132,9697726	60,72855507	50,88522646	-0,462028098	False	0,462028098
ENSMUSG00000028149	Rap1gds1	366,3255736	204,4430711	265,9395453	233,9173973	200,9314071	-0,462028098	False	0,462028098
ENSMUSG00000031967	Afg3l1	711,0116618	424,0300734	484,3269865	516,1927181	332,7110961	-0,461963433	False	0,461963433
ENSMUSG00000018076	Med13l	174,6615604	312,974578	126,805611	209,1761341	386,2058214	-0,46194355	False	0,46194355
ENSMUSG00000035206	Sppl2b	229,5331126	84,55361583	316,133433	255,2848519	18,26649155	0,461831324	False	0,461831324
ENSMUSG00000025355	Mmp19	1120,616206	182,9891686	813,6693373	166,441225	140,9129348	-0,461777732	False	0,461777732
ENSMUSG00000062116	Zfp954	78,82955381	147,6533291	57,23864386	128,2047274	105,6847011	-0,461747118	False	0,461747118
ENSMUSG00000056313	Tcim	281,3133097	1133,270851	204,2979288	227,1697801	370,5488286	-0,461503234	False	0,461503234
ENSMUSG00000028572	Hook1	675,4610787	248,6128704	254,4918165	311,5149954	490,5857731	-0,461367245	False	0,461367245
ENSMUSG00000038013	Wipf2	204,0294334	790,0084106	280,9096522	569,049053	501,0237683	0,461328906	False	0,461328906
ENSMUSG00000028402	Mpdz	317,6367315	136,2953807	230,7157645	214,7991485	219,1978986	-0,461261171	False	0,461261171
ENSMUSG00000030530	Furin	2060,388142	2120,150367	1802,576984	1000,896556	2836,525188	0,461208533	False	0,461208533
ENSMUSG00000020790	Ankfy1	494,6168082	548,9675058	359,2825645	516,1927181	853,3061053	-0,461192342	False	0,461192342
ENSMUSG00000019977	Hbs1l	741,9252123	1202,680536	538,9238468	1569,945609	1591,794264	-0,461192342	False	0,461192342

ENSMUSG00000011158	Brf1	150,7035587	416,4581078	360,163159	1096,4878	207,455154	0,461086015	False	0,461086015
ENSMUSG00000024900	Cpt1a	2637,698697	4906,633707	1916,173677	7010,774302	4318,720502	-0,461051446	False	0,461051446
ENSMUSG00000034974	Dapk3	316,091054	434,1260276	533,6402796	616,2823736	229,6358938	-0,460992041	False	0,460992041
ENSMUSG00000026568	Mpc2	1976,921555	763,506531	1436,249664	474,5824118	1112,951235	-0,460949071	False	0,460949071
ENSMUSG00000034807	Colgalt1	856,3053492	258,7088246	710,6397783	1178,583809	268,7783757	0,46085711	False	0,46085711
ENSMUSG00000031987	Egln1	364,779896	124,9374323	265,0589508	84,34521537	190,4934119	-0,460712961	False	0,460712961
ENSMUSG00000035248	Zcchc6	1077,337235	970,4735907	782,848529	1260,679819	1319,10164	-0,460664826	False	0,460664826
ENSMUSG00000035569	Ankrd11	852,4411554	2405,361071	1172,951902	2840,746854	5919,648012	0,460471703	False	0,460471703
ENSMUSG00000049658	Bdp1	154,5677526	151,4393119	102,1489644	151,8213877	212,6741516	0,460405338	False	0,460405338
ENSMUSG00000000902	Smarcb1	283,6318259	420,2440906	432,3719097	367,745139	206,1504046	-0,460322131	False	0,460322131
ENSMUSG00000021336	Slc17a4	1161,57666	844,274164	218,3874412	371,1189476	123,9511927	-0,460300899	False	0,460300899
ENSMUSG00000023572	Ccndbp1	207,1207884	247,3508762	150,5816631	227,1697801	450,1385418	-0,459926264	False	0,459926264
ENSMUSG00000003970	Rpl8	2507,088947	5013,903219	5520,447051	3448,032404	3975,571411	0,459760152	False	0,459760152
ENSMUSG00000037174	Elf2	271,2664057	355,8823831	197,2531727	289,022938	417,5198069	-0,459661887	False	0,459661887
ENSMUSG00000021936	Mapk8	262,7651794	352,0964003	191,089011	313,7642012	618,4512139	-0,459529292	False	0,459529292
ENSMUSG00000073481	Marc2	3354,120231	488,391781	2439,246823	419,4768711	617,1464645	-0,459498655	False	0,459498655
ENSMUSG00000024231	Cul2	416,5600932	405,1001594	208,7009014	263,157072	572,7849851	0,45946904	False	0,45946904
ENSMUSG00000044627	Swi5	1309,188864	1048,717235	1799,935201	323,885627	1107,732238	0,459271733	False	0,459271733
ENSMUSG00000038387	Rras	466,021774	1039,883275	753,7889099	810,8386704	640,6319537	0,459098396	False	0,459098396
ENSMUSG00000029725	Ppp1r35	132,9282672	213,277031	187,5666329	96,71584696	118,7321951	-0,458823726	False	0,458823726
ENSMUSG00000020843	Timm22	85,01226391	148,9153234	104,790748	61,85315794	112,2084481	-0,458823726	False	0,458823726
ENSMUSG00000061650	Med9	92,74065154	147,6533291	121,5220439	67,4761723	151,35093	-0,458823726	False	0,458823726
ENSMUSG00000078453	Abracl	98,92336164	185,5131571	85,41766852	71,97458378	194,4076601	-0,458823726	False	0,458823726
ENSMUSG00000014606	Slc25a11	853,2139941	241,0409048	620,8191372	346,3776845	173,5316697	-0,458734598	False	0,458734598
ENSMUSG00000060681	Slc9a6	156,1134301	254,9228418	113,5966932	322,7610242	425,3483033	-0,458673816	False	0,458673816
ENSMUSG00000028756	Pink1	2680,204829	1326,355974	1950,516864	1476,60357	952,4670595	-0,458486788	False	0,458486788
ENSMUSG00000024539	Ptpn2	135,2467835	489,6537753	185,8054439	213,6745456	1418,262594	0,458198488	False	0,458198488
ENSMUSG00000034951	Cog7	256,5824693	186,7751514	113,5966932	147,3229762	110,9036987	-0,458120072	False	0,458120072
ENSMUSG00000022807	Osbpl11	222,5775567	533,8235746	162,0293918	533,0617611	670,6411898	-0,458052634	False	0,458052634
ENSMUSG00000012848	Rps5	2579,73579	6211,535778	6051,445547	3543,623648	5083,303649	0,458002074	False	0,458002074
ENSMUSG00000031060	Rbm10	481,4785492	661,2849955	830,4006332	2042,278815	968,1240522	0,457800642	False	0,457800642
ENSMUSG00000024072	Yipf4	326,1379579	131,2474037	121,5220439	78,72220101	237,4643902	-0,457771175	False	0,457771175
ENSMUSG00000112129	Pbld1	3243,604287	25,23988532	2361,754505	527,4387468	43,05673008	-0,457738815	False	0,457738815
ENSMUSG00000017453	Pipox	5351,135594	579,2553682	3896,630755	422,8506797	1149,484218	-0,457617858	False	0,457617858
ENSMUSG00000035597	Prpf39	377,1453162	682,738898	517,7895783	934,5449863	1063,370758	0,457245419	False	0,457245419
ENSMUSG00000031953	Tmem170	169,2516891	102,2215356	123,2832329	71,97458378	87,41820957	-0,457193631	False	0,457193631
ENSMUSG00000018322	Tomm34	132,1554284	1393,24167	181,4024713	723,1196465	1974,085837	0,456958424	False	0,456958424
ENSMUSG00000095567	Noc2l	464,4760964	678,9529152	637,5504331	764,7299527	1026,837775	0,456935118	False	0,456935118
ENSMUSG00000031021	Tmem9b	259,6738243	166,5832431	148,820474	74,22378953	189,1886625	-0,45687496	False	0,45687496
ENSMUSG00000036781	Rps27l	928,9521929	790,0084106	1106,907313	242,9142203	1274,74016	0,456526944	False	0,456526944
ENSMUSG00000020098	Pcbd1	3674,848317	865,7280666	5042,284226	411,604651	1101,208491	0,456392741	False	0,456392741
ENSMUSG00000068566	Myadm	425,0613195	418,9820964	485,207581	184,4348709	583,2229802	0,456376586	False	0,456376586
ENSMUSG00000094483	Purb	496,1624857	1513,131125	680,6995646	781,5989958	892,4485872	0,456205528	False	0,456205528
ENSMUSG00000061079	Zfp143	124,4270408	285,2107041	90,70123565	339,6300672	187,8839131	-0,45610594	False	0,45610594
ENSMUSG00000021481	Zfp346	49,46168082	94,64956996	76,61172331	266,5308806	67,84696862	0,455973091	False	0,455973091
ENSMUSG00000079477	Rab7	3148,54512	2621,162091	2168,904305	1477,728173	4318,720502	0,45591861	False	0,45591861
ENSMUSG00000022685	Parn	278,9947934	296,5686525	203,4173343	560,0522301	550,6042453	-0,455795574	False	0,455795574
ENSMUSG00000040963	Asgr2	3922,156721	639,8310929	2860,171004	575,7966703	1030,752023	-0,455545778	False	0,455545778
ENSMUSG00000024181	Mrpl28	309,1355051	151,4393119	225,4321973	164,1920193	142,2176842	-0,455545778	False	0,455545778
ENSMUSG00000053898	Ech1	3293,838807	2068,408602	4516,569297	1515,964671	1838,3919	0,45545739	False	0,45545739
ENSMUSG00000068735	Trp53i11	91,19497401	347,0484232	125,044422	457,7133687	225,7216456	0,455414481	False	0,455414481
ENSMUSG00000002718	Cse1l	420,424287	159,0112775	204,2979288	141,6999618	306,6161082	-0,455412521	False	0,455412521
ENSMUSG00000061665	Cd2ap	709,4659843	699,1448234	566,2222769	972,781484	494,5000213	0,455382253	False	0,455382253
ENSMUSG00000054302	Eapp	242,6713715	198,1330998	176,1189042	85,46981824	332,7110961	0,455266061	False	0,455266061
ENSMUSG00000002984	Tomm40	360,9157022	777,3884679	678,9383756	494,8252635	657,5936958	0,455257248	False	0,455257248
ENSMUSG00000024098	Twsg1	699,4190803	417,7201021	470,2374741	269,9046892	510,157014	-0,455215811	False	0,455215811
ENSMUSG00000021771	Vdac2	914,2682564	1274,614209	1066,399965	666,8895029	1740,535695	-0,455169789	False	0,455169789
ENSMUSG00000031299	Pdha1	1138,391498	830,3922271	495,7747152	468,9593975	825,906368	-0,455131947	False	0,455131947
ENSMUSG00000003234	Abcf3	597,4043636	523,7276204	656,042918	792,8450245	435,7862984	-0,455086938	False	0,455086938
ENSMUSG00000023010	Tmbim6	17304,63274	7816,792484	8401,752323	3166,881686	12623,45041	-0,455052022	False	0,455052022
ENSMUSG00000062270	Morf4l1	1645,373726	734,4806629	355,7601864	441,9689285	1200,369445	-0,45493681	False	0,45493681
ENSMUSG00000028992	Nmnat1	264,3108569	135,0333865	192,8502001	68,60077517	69,15171802	-0,454755044	False	0,454755044
ENSMUSG00000020841	Cpd	272,8120833	343,2624404	252,7306275	199,0547083	639,3272043	-0,454742549	False	0,454742549
ENSMUSG00000027787	Nmd3	266,6293732	287,7346927	221,9098193	194,5562968	550,6042453	-0,45464805	False	0,45464805
ENSMUSG00000028292	Rars2	159,2047851	103,4835298	99,50718086	218,1729571	41,75198069	0,454588589	False	0,454588589
ENSMUSG00000001280	Sp1	405,7403505	109,7935012	159,3876083	49,48252635	555,8232429	0,454069381	False	0,454069381
ENSMUSG00000039285	Azi2	645,3203669	805,1523418	471,1180687	723,1196465	1257,778418	-0,453926895	False	0,453926895
ENSMUSG00000024566	Atp9b	378,6909938	1057,551195	518,6701728	1558,69958	1032,056773	0,453796296	False	0,453796296
ENSMUSG00000041961	Znrf3	119,7900082	164,0592546	85,41766852	56,23014358	75,675465	0,453709402	False	0,453709402
ENSMUSG00000022471	Xrcc6	189,3454969	29,02586812	138,2533398	69,72537804	26,09498793	-0,453706779	False	0,453706779
ENSMUSG00000025491	Ifitm1	196,3010458	268,8047787	318,7752165	430,7228998	356,1965852	0,453490927	False	0,453490927
ENSMUSG00000028703	Lrrc41	445,9279661	413,9341193	317,0140275	173,1888422	610,6227176	0,453470584	False	0,453470584
ENSMUSG00000015568	Lpl	180,0714317	365,9783372	246,5664658	425,0998855	403,1675635	0,453407284	False	0,453407284
ENSMUSG00000004565	Pnpla6	560,308103	1755,434024	766,9978277	1618,303532	1274,74016	0,453002135	False	0,453002135
ENSMUSG00000027984	Hadh	4160,963899	559,0634599	3039,812286	443,0935314	951,1623101	-0,452935534	False	0,452935534
ENSMUSG00000067336	Bmpr2	367,8712511	268,8047787	221,0292247	204,6777226	251,8166335	-0,452642149	False	0,452642149
ENSMUSG00000037669	Ldah	467,5674515	233,4689392	341,6706741	340,7546701	101,7704529	-0,452568079	False	0,452568079
ENSMUSG00000045441	Gprin3	175,4343992	76,98165023	99,50718086	128,2047274	43,05673008	-0,452482199	False	0,452482199
ENSMUSG00000039050	Osbpl2	407,286028	379,8602741	297,640948	642,1482397	349,6728383	-0,452469304	False	0,452469304
ENSMUSG00000003200	Sh3gl1	272,0392445	503,5357122	450,8643947	372,2435505	561,0422405	0,452432063	False	0,452432063
ENSMUSG00000024151	Msh2	110,5159431	80,76763303	32,58199727	68,60077517	76,98021439	-0,452405343	False	0,452405343
ENSMUSG00000017188	Coa3	412,6958993	217,0630138	564,4610879	278,9015122	103,0752023	0,451795026	False	0,451795026
ENSMUSG00000040105	Plpp6	299,86144	215,8010195	219,2680357	112,4602872	187,8839131	-0,451600499	False	0,451600499
ENSMUSG00000045282	Tmem86b	961,4114209	545,181523	1314,72762	227,1697801	501,0237683	0,451538091	False	0,451538091
ENSMUSG00000053158	Fes	112,0616206	369,76432	153,2234466	304,7673782	705,8694235	0,451344817	False	0,451344817
ENSMUSG00000042197	Zfp451	146,8393649	618,3771904	200,7755508	432,9721056	422,7388045	0,451344817	False	0,451344817
ENSMUSG00000054766	Set	1111,342141	691,5728578	716,80394	552,18001	812,858874	-0,451226234	False	0,451226234
ENSMUSG00000061759	Armt1	146,0665262	61,83771904	96,86539729	106,8372728	63,93272043	-0,451210542	False	0,451210542
ENSMUSG00000020315	Sptbn1	984,5965838	1073,95712	1099,862557	1082,992565	720,2216669	-0,451091732	False	0,451091732
ENSMUSG00000009563	Tor2a	365,5527348	612,0672191	529,237307	684,8831488	267,4736263	-0,450682901	False	0,450682901
ENSMUSG00000028617	Lrrc42	168,4788503	111,0554954	123,2832329	96,71584696	99,16095413	-0,450590896	False	0,450590896
ENSMUSG00000039167	Adgrl4	132,1554284	29,02586812	74,85053427	96,71584696	49,58047707	-0,450411486	False	0,450411486
ENSMUSG00000024393	Prrc2a	1836,2649	2648,925965	2812,6189	3229,859447	2509,033089	0,4503573	False	0,4503573
ENSMUSG00000022020	Naa16	136,0196223	265,0187959	185,8054439	407,1062395	319,6636021	0,449977981	False	0,449977981
ENSMUSG00000026867	Gapvd1	528,6217138	1157,248742	722,0875071	1848,847121	785,4591367	0,449937996	False	0,449937996
ENSMUSG00000030986	Dhx32	436,653901	297,8306468	230,7157645	220,4221628	319,6636021	-0,449935745	False	0,449935745
ENSMUSG00000049657	Zbtb5	181,6171093	46,69378785	132,9697726	50,60712922	11,74274457	-0,449801796	False	0,449801796
ENSMUSG00000063362	Alg11	435,8810622	259,9708188	595,2818961	289,022938	241,3786384	0,449638495	False	0,449638495
ENSMUSG00000006362	Cbfa2t3	47,14316453	64,36170757	105,6713425	484,7038377	96,55145534	0,449153998	False	0,449153998
ENSMUSG00000031827	Cotl1	216,3948536	126,1994266	158,5070138	133,8277417	136,9986866	-0,449119509	False	0,449119509
ENSMUSG00000036975	Tmem177	140,6566548	71,93367317	103,0295589	57,35474645	61,32322164	-0,449119509	False	0,449119509
ENSMUSG00000043716	Rpl7	4240,566291	5093,408858	5007,94104	3107,277734	5138,103123	-0,448605739	False	0,448605739
ENSMUSG00000039046	Usp6nl	434,3353847	311,7125837	175,2383097	318,2626127	268,7783757	-0,448591803	False	0,448591803
ENSMUSG00000089876	Tmem102	125,1998796	147,6533291	170,835337	103,4634642	50,88522646	0,448373251	False	0,448373251
ENSMUSG00000032481	Smarcc1	325,3651191	238,5169163	227,1933864	145,0737704	144,827183	-0,447968005	False	0,447968005
ENSMUSG00000032757	Bet1	708,6931455	299,0926411	519,5507673	256,4094547	174,8364191	-0,447896368	False	0,447896368
ENSMUSG00000002602	Axl	292,9058911	88,33959863	197,2531727	214,7991485	106,9894505	-0,447448936	False	0,447448936
ENSMUSG00000020781	Tsen54	60,2814235	205,7050654	214,8650631	444,2181343	82,19921198	0,447411078	False	0,447411078
ENSMUSG00000021884	Hacl1	875,6263183	377,3362856	497,5359043	642,1482397	292,2638648	-0,447408936	False	0,447408936
ENSMUSG00000035769	Xylb	1855,585869	2732,217586	3170,140275	4942,629621	1360,853621	-0,447362873	False	0,447362873
ENSMUSG00000021483	Cdk20	112,0616206	223,3729851	206,9397124	467,8347946	82,19921198	-0,447095793	False	0,447095793
ENSMUSG00000058006	Mdn1	164,6146565	395,0042053	274,7454905	421,7260769	224,4168962	0,447088508	False	0,447088508
ENSMUSG00000028708	Mknk1	295,2244074	436,6500161	402,431696	553,3046128	785,4591367	0,446931938	False	0,446931938

ENSMUSG00000023367	Tmem176a	2180,17815	2103,744442	6781,458405	2420,14538	1599,62276	-0,446714314	False	0,446714314
ENSMUSG00000021114	Atp6v1d	408,8317055	427,8160562	332,8647289	268,7800863	557,1279923	0,446501721	False	0,446501721
ENSMUSG00000032366	Tpm1	591,9944923	928,8277799	1375,488642	1280,922671	434,481549	-0,446288842	False	0,446288842
ENSMUSG00000061950	Ppp4r1	230,3059513	681,4769037	169,074148	700,627589	542,7757489	-0,445895608	False	0,445895608
ENSMUSG00000038489	Polr2l	254,263953	328,1185092	668,3712413	186,6840767	480,1477779	-0,445728068	False	0,445728068
ENSMUSG00000050148	Ubqln2	278,9947934	68,14769037	77,49231784	132,7031389	204,8456553	-0,445700905	False	0,445700905
ENSMUSG00000056035	Cyp3a11	22098,55158	3868,012426	13169,29106	312,6395983	16225,8635	-0,445656557	False	0,445656557
ENSMUSG00000035824	Tk2	278,2219546	310,4505895	204,2979288	393,6110051	254,4261323	-0,44556169	False	0,44556169
ENSMUSG00000025240	Sacm1l	927,4065154	533,8235746	616,4161646	292,3967466	681,079185	-0,445379321	False	0,445379321
ENSMUSG00000025175	Fn3k	163,068979	7,571965597	119,7608548	3,373808615	6,523746983	-0,445325936	False	0,445325936
ENSMUSG00000019295	Tmem129	291,3602136	123,6754381	213,9844686	66,35156943	75,675465	-0,445297798	False	0,445297798
ENSMUSG00000028860	Sytl1	115,1529757	10,09595413	156,7458247	71,97458378	13,04749397	0,444875322	False	0,444875322
ENSMUSG00000080316	Spaca6	83,46658638	495,9637466	103,9101535	258,6586605	61,32322164	-0,444765277	False	0,444765277
ENSMUSG00000020280	Pus10	179,298593	88,33959863	117,1190713	244,0388231	157,874677	0,444746512	False	0,444746512
ENSMUSG00000031533	Mrps31	402,6489954	280,1627271	124,1638274	59,6039522	547,9947465	0,444639329	False	0,444639329
ENSMUSG00000028657	Ppt1	513,1649385	294,044664	296,7603535	238,4158088	377,0725756	-0,444580376	False	0,444580376
ENSMUSG00000005836	Gata6	163,068979	961,6396308	221,9098193	561,1768329	1613,975003	0,444491146	False	0,444491146
ENSMUSG00000004945	Tmem242	307,5898276	338,2144633	559,1775207	226,0451772	352,2823371	-0,444396655	False	0,444396655
ENSMUSG00000020946	Gosr2	721,0585657	759,7205482	492,2523372	546,5569956	981,1715462	0,444388954	False	0,444388954
ENSMUSG00000028329	Xpa	229,5331126	599,4472764	228,0739809	168,6904307	456,6622888	-0,444324156	False	0,444324156
ENSMUSG00000031878	Nae1	304,4984725	172,8932145	155,8652302	223,7959715	207,455154	-0,444250925	False	0,444250925
ENSMUSG00000026269	Rnpepl1	1123,707561	1230,444409	1211,698061	1339,40202	825,906368	-0,444216491	False	0,444216491
ENSMUSG00000047407	Tgif1	298,3157624	986,8795161	219,2680357	411,604651	1178,188705	-0,444144694	False	0,444144694
ENSMUSG00000026305	Lrrfip1	468,3402903	715,5507489	344,3124577	524,0649382	962,9050546	-0,443838775	False	0,443838775
ENSMUSG00000028796	Phc2	348,550282	479,5578211	256,2530056	272,1538949	551,9089947	-0,443797869	False	0,443797869
ENSMUSG00000034595	Ppp1r18	71,87400494	254,9228418	97,74599182	339,6300672	339,2348431	0,443567467	False	0,443567467
ENSMUSG00000022095	Fam160b2	143,7480099	230,9449507	195,4919836	222,6713686	242,6833878	0,443567467	False	0,443567467
ENSMUSG00000028798	Eif3i	803,7523133	526,251609	562,6998988	501,5728807	591,0514766	-0,4434672	False	0,4434672
ENSMUSG00000036376	Abt1	108,9702656	82,0296273	80,1341014	92,21743547	238,7691396	-0,443446301	False	0,443446301
ENSMUSG00000034610	Zcchc11	364,0070573	677,6909209	267,7007343	609,5347564	1112,951235	-0,443345325	False	0,443345325
ENSMUSG00000039233	Tbce	371,7354449	653,7130299	505,461255	1193,203647	833,7348644	0,443324254	False	0,443324254
ENSMUSG00000021814	Anxa7	1206,401309	1601,470724	980,1017017	887,3116657	1829,258654	-0,443197058	False	0,443197058
ENSMUSG00000052609	Plekhg3	560,308103	489,6537753	761,7142605	599,4133306	93,94195655	0,443029549	False	0,443029549
ENSMUSG00000029060	Mib2	276,6762771	321,8085379	509,8642276	878,3148427	203,5409059	-0,442880185	False	0,442880185
ENSMUSG00000033075	Senp1	108,1974268	148,9153234	147,059285	215,9237514	186,5791637	0,442731687	False	0,442731687
ENSMUSG00000039130	Zc3hc1	122,8813633	76,98165023	82,77588496	86,59442111	167,0079228	0,442650418	False	0,442650418
ENSMUSG00000024055	Cyp4f13	970,6854861	720,598726	985,3852688	1319,159168	554,5184935	0,442542824	False	0,442542824
ENSMUSG00000028414	Fktn	203,2565946	74,4576617	137,3727453	149,5721819	19,57124095	-0,442460279	False	0,442460279
ENSMUSG00000028343	Erp44	1271,319765	956,5916537	1209,936872	416,1030625	1727,488201	0,442348911	False	0,442348911
ENSMUSG00000033186	Mzt1	162,2961402	89,6015929	55,47745481	35,98729189	220,502648	0,442167292	False	0,442167292
ENSMUSG00000058709	Egln2	672,3697236	889,7059576	494,8941207	869,3180198	1817,515909	-0,442134859	False	0,442134859
ENSMUSG00000006014	Prg4	1390,336934	2746,099523	1941,710918	1023,388613	6402,405289	-0,442080462	False	0,442080462
ENSMUSG00000027889	Ampd2	783,6585055	1356,643836	1064,638776	2553,973121	2345,939415	0,442067004	False	0,442067004
ENSMUSG00000062078	Qk	724,1499207	547,7055115	305,5662987	371,1189476	983,781045	0,442048849	False	0,442048849
ENSMUSG00000026283	Ing5	87,3307802	111,0554954	64,28340002	74,22378953	167,0079228	-0,442043992	False	0,442043992
ENSMUSG00000089872	Rps6kc1	137,5652998	171,6312202	101,2683699	164,1920193	200,9314071	-0,441932969	False	0,441932969
ENSMUSG00000085396	Firre	767,4288915	697,8828292	1812,263524	3118,523763	564,9564887	-0,44189332	False	0,44189332
ENSMUSG00000026927	Sdccag3	450,5649987	845,5361583	612,013192	616,2823736	918,5435751	0,441827506	False	0,441827506
ENSMUSG00000037440	Vnn1	464,4760964	177,9411915	442,939044	630,902211	147,4366818	0,441812054	False	0,441812054
ENSMUSG00000031838	Ifi30	386,4193814	338,2144633	391,8645618	284,5245265	541,4709996	-0,441614436	False	0,441614436
ENSMUSG00000034300	Fam53c	151,4763975	205,7050654	125,044422	49,48252635	190,4934119	0,441484304	False	0,441484304
ENSMUSG00000040586	Ofd1	56,41722968	295,3066583	76,61172331	287,8983351	429,2625515	0,441429348	False	0,441429348
ENSMUSG00000004356	Utp20	190,1183356	234,7309335	140,0145288	152,9459905	472,3192815	-0,441321139	False	0,441321139
ENSMUSG00000037851	Iars	430,4711909	251,136859	244,8052768	217,0483542	317,0541034	-0,441187641	False	0,441187641
ENSMUSG00000050947	Amigo1	139,8838161	31,54985665	103,0295589	86,59442111	11,74274457	-0,441170756	False	0,441170756
ENSMUSG00000034194	R3hcc1	125,1998796	191,8231285	187,5666329	92,21743547	199,6266577	-0,441121724	False	0,441121724
ENSMUSG00000063077	Kif1b	1595,912045	672,6429439	631,3862715	748,9855125	1175,579206	-0,441009397	False	0,441009397
ENSMUSG00000038172	Ttc39b	582,7204272	379,8602741	284,4320302	190,0578853	429,2625515	-0,440943566	False	0,440943566
ENSMUSG00000039599	Fam149b	424,2884808	195,6091113	312,6110549	278,9015122	281,8258696	-0,440676713	False	0,440676713
ENSMUSG00000058587	Tmod3	315,3182152	301,6166296	190,2084165	323,885627	427,9578021	0,440660036	False	0,440660036
ENSMUSG00000003068	Stk11	1149,21124	1913,183307	1559,532896	2069,269284	2344,634666	0,440469976	False	0,440469976
ENSMUSG00000050244	Heatr1	202,4837559	636,0451101	274,7454905	490,326852	673,2506886	0,440289629	False	0,440289629
ENSMUSG00000019929	Dcn	3126,905634	5417,741385	2304,515861	3946,231477	4229,997543	-0,440271984	False	0,440271984
ENSMUSG00000022201	Zfr	1000,053359	1542,156993	737,057614	1320,283771	2428,138627	-0,440227678	False	0,440227678
ENSMUSG00000033228	Scaf11	768,2017302	777,3884679	566,2222769	573,5474645	1197,759946	-0,440116703	False	0,440116703
ENSMUSG00000021737	Psmd6	648,411722	705,4547948	567,983466	477,9562204	974,6477992	-0,440031695	False	0,440031695
ENSMUSG00000033326	Kdm4a	377,1453162	1164,820708	511,6254166	529,6879525	751,5356524	0,439967427	False	0,439967427
ENSMUSG00000026614	Slc30a10	1022,465683	3551,251865	753,7889099	1349,523446	4749,287803	-0,439819949	False	0,439819949
ENSMUSG00000021500	Ddx46	531,7130688	974,2595735	721,2069126	1221,318719	1072,504004	0,439765298	False	0,439765298
ENSMUSG00000028607	Cpt2	1913,548777	1410,90959	1183,519036	787,2220101	674,555438	-0,439625132	False	0,439625132
ENSMUSG00000053460	Ggcx	743,4708898	172,8932145	1008,280726	448,7165458	512,7665128	0,439549211	False	0,439549211
ENSMUSG00000030216	Wbp11	462,1575802	724,3847088	340,7900796	455,464163	1473,062069	-0,439501507	False	0,439501507
ENSMUSG00000055435	Maf	574,9920395	984,3555276	1141,250499	2976,823801	424,0435539	-0,43932953	False	0,43932953
ENSMUSG00000061410	Zcchc14	360,9157022	396,2661996	369,8496988	490,326852	266,1688769	-0,439320025	False	0,439320025
ENSMUSG00000000439	Mkm2	226,4417575	99,69754702	139,1339343	87,71902399	167,0079228	-0,439223481	False	0,439223481
ENSMUSG00000017801	Mlx	422,7428033	408,8861422	593,5207071	483,5792348	311,8351058	-0,438996842	False	0,438996842
ENSMUSG00000021286	Zfyve21	401,1033179	66,88569611	295,879759	150,6967848	106,9894505	-0,438962894	False	0,438962894
ENSMUSG00000026576	Atp1b1	670,8240461	208,2290539	494,8941207	163,0674164	334,0158455	-0,438814501	False	0,438814501
ENSMUSG00000039231	Suv39h1	94,28632906	150,1773177	69,56696715	98,9650527	215,2836504	-0,438646178	False	0,438646178
ENSMUSG00000039529	Atp8b1	312,9996989	230,9449507	153,2234466	89,96822973	53,49472526	-0,438612266	False	0,438612266
ENSMUSG00000038618	Rassf7	348,550282	165,3212489	381,2974275	472,3332061	44,36147948	0,438438169	False	0,438438169
ENSMUSG00000035437	Rabgap1	245,7627266	297,8306468	190,2084165	572,4228617	181,3601661	-0,438408509	False	0,438408509
ENSMUSG00000057421	Las1l	434,3353847	446,7459702	320,5364056	774,8513785	1045,104267	-0,438321269	False	0,438321269
ENSMUSG00000034675	Dbn1	28,59503422	440,4359989	38,74615892	289,022938	405,7770623	0,438288664	False	0,438288664
ENSMUSG00000028403	Zdhhc21	141,4294936	92,12558143	51,07448221	17,99364595	104,3799517	-0,43823837	False	0,43823837
ENSMUSG00000034845	Plvap	1016,282973	979,3075505	1395,742316	750,1101154	1603,537008	-0,438127859	False	0,438127859
ENSMUSG00000002289	Angptl4	533,2587463	4765,290349	393,6257508	5258,643028	4468,766683	-0,438011119	False	0,438011119
ENSMUSG00000017548	Suz12	274,3577608	256,184836	202,5367398	224,9205743	527,1187562	-0,437874752	False	0,437874752
ENSMUSG00000003119	Cdk12	261,2195018	353,3583945	192,8502001	454,3395601	487,9762743	-0,437781965	False	0,437781965
ENSMUSG00000021982	Cdadc1	591,9944923	336,9524691	306,4468933	250,7864404	437,0910478	-0,437649923	False	0,437649923
ENSMUSG00000042589	Cux2	1015,510134	825,3442501	1238,996491	1375,389312	332,7110961	0,437635403	False	0,437635403
ENSMUSG00000031012	Cask	208,666466	118,627461	141,7757179	154,0705934	117,4274457	-0,43760724	False	0,43760724
ENSMUSG00000022400	Rbx1	469,113129	874,5620264	780,2067455	346,3776845	923,7625727	-0,437589891	False	0,437589891
ENSMUSG00000027478	Dnmt3b	88,87645772	65,62370184	156,7458247	93,34203834	73,0659662	-0,437584339	False	0,437584339
ENSMUSG00000029394	Cdk2ap1	133,701106	344,5244347	235,9993316	181,0610623	341,8443419	0,437464925	False	0,437464925
ENSMUSG00000020823	Sec14l1	159,9776239	94,64956996	216,6262521	119,2079044	36,5329831	0,437337959	False	0,437337959
ENSMUSG00000074882	Cyp2c68	7699,019755	928,8277799	5685,998821	307,016584	2160,665001	-0,437260973	False	0,437260973
ENSMUSG00000020986	Sec23a	890,3102547	448,0079645	355,7601864	256,4094547	657,5936958	-0,437111705	False	0,437111705
ENSMUSG00000002981	Clptm1	1403,475193	1467,699332	1537,518033	1036,883848	1649,203237	-0,436749272	False	0,436749272
ENSMUSG00000026924	Sec16a	1350,149319	1005,80943	1120,116231	1827,479666	1381,729611	0,436736384	False	0,436736384
ENSMUSG00000028645	Slc2a1	197,8467233	219,5870023	257,1336001	146,1983733	199,6266577	-0,436455913	False	0,436455913
ENSMUSG00000028233	Tgs1	146,0665262	174,1552087	118,8802603	107,9618757	229,6358938	-0,436103649	False	0,436103649
ENSMUSG00000052926	Rnaseh2a	142,9751711	189,2991399	251,850033	219,29756	105,6847011	-0,435998084	False	0,435998084
ENSMUSG00000039148	Sart1	400,3304791	772,3404909	541,5656303	861,4457997	751,5356524	0,435944724	False	0,435944724
ENSMUSG00000056698	Elmod3	363,2342185	1692,334311	491,3717426	1939,939954	1805,773165	0,435914777	False	0,435914777
ENSMUSG00000033629	Hacd3	1478,440553	2255,183754	1999,830157	2415,646968	4634,469856	0,435801244	False	0,435801244
ENSMUSG00000033860	Fgg	61099,85975	159355,802	45178,90189	61970,11664	242764,2822	-0,435519863	False	0,435519863
ENSMUSG00000031931	Ankrd49	147,6122037	497,2257409	213,9844686	475,7070147	199,6266577	0,435492388	False	0,435492388
ENSMUSG00000041225	Arhgap12	331,5478292	128,7234151	221,9098193	245,163426	87,41820957	-0,435473238	False	0,435473238
ENSMUSG00000031529	Tnks	302,952795	116,1034725	125,9250165	68,60077517	409,6913105	0,435444278	False	0,435444278
ENSMUSG00000036257	Pnpla8	1415,067775	790,0084106	398,9093179	782,7235986	1046,409016	-0,435424276	False	0,435424276
ENSMUSG00000028745	Capzb	1357,877706	2155,486207	1409,831828	1004,270364	2262,435454	-0,435205837	False	0,435205837
ENSMUSG00000073702	Rpl31	1657,739146	3678,713286	4063,063119	2241,333523	2969,609626	0,435140337	False	0,435140337

ENSMUSG00000021039	Snw1	440,5180948	441,6979931	325,8199727	563,4260387	677,1649368	-0,435126237	False	0,435126237
ENSMUSG00000022853	Ehhadh	4848,790398	879,6100035	915,8183017	3586,358558	2180,236242	-0,435105157	False	0,435105157
ENSMUSG00000000567	Sox9	103,5603942	660,0230012	140,0145288	278,9015122	855,9156041	0,435104176	False	0,435104176
ENSMUSG00000036499	Eea1	517,8019711	278,9007328	383,0586166	276,6523064	190,4934119	-0,434835283	False	0,434835283
ENSMUSG00000029014	Dnajc2	249,6269204	562,8494427	342,5512686	337,3808615	407,0818117	0,43460461	False	0,43460461
ENSMUSG00000055980	Irs1	286,723181	143,8673463	250,0888439	53,98093784	387,5105708	0,434577117	False	0,434577117
ENSMUSG00000021539	Lect2	2284,511383	648,6650528	3087,36439	179,9364595	1020,314028	0,434490138	False	0,434490138
ENSMUSG00000022391	Rangap1	391,056414	660,0230012	528,3567125	537,5601726	1505,680804	0,434135527	False	0,434135527
ENSMUSG00000025384	Faap100	81,14807009	151,4393119	213,103874	200,1793111	109,5989493	0,433605278	False	0,433605278
ENSMUSG00000021607	Mrpl36	251,1725979	104,7455241	339,0288905	93,34203834	76,98021439	0,432729139	False	0,432729139
ENSMUSG00000030707	Coro1a	100,4690392	384,9082512	135,6115562	347,5022873	324,8825997	0,432729139	False	0,432729139
ENSMUSG00000045948	Mrps12	360,1428635	147,6533291	266,8201398	201,303914	161,7889252	-0,43270175	False	0,43270175
ENSMUSG00000019863	Qrsl1	53,32587463	162,7972603	83,65647948	71,97458378	193,1029107	0,432651818	False	0,432651818
ENSMUSG00000037679	Inf2	783,6585055	790,0084106	1327,055943	1014,39179	580,6134814	-0,432647038	False	0,432647038
ENSMUSG00000029482	Aacs	1044,105169	275,11475	1408,951233	201,303914	101,7704529	0,432354642	False	0,432354642
ENSMUSG00000005609	Ctr9	332,320668	198,1330998	229,8351699	246,2880289	221,8073974	-0,432229516	False	0,432229516
ENSMUSG00000047989	Ino80c	174,6615604	401,3141766	129,4473946	161,9428135	472,3192815	-0,432196206	False	0,432196206
ENSMUSG00000097571	Jpx	63,37277855	321,8085379	252,7306275	459,9625745	46,97097827	-0,43209363	False	0,43209363
ENSMUSG00000059013	Sh2d3c	158,4319464	246,0888819	272,9843015	782,7235986	117,4274457	-0,43209363	False	0,43209363
ENSMUSG00000071654	Uqcc3	158,4319464	146,3913349	293,2379754	145,0737704	117,4274457	-0,43209363	False	0,43209363
ENSMUSG00000058444	Map2k5	243,4442103	271,3287672	152,3428521	328,3840385	199,6266577	0,431792815	False	0,431792815
ENSMUSG00000046573	Lyrm4	162,2961402	336,9524691	188,4472275	120,3325073	352,2823371	-0,431602257	False	0,431602257
ENSMUSG00000033417	Cacul1	597,4043636	257,4468303	442,939044	395,8602108	310,5303564	-0,431599603	False	0,431599603
ENSMUSG00000029714	Gigyf1	454,4291925	1381,883721	612,8937865	889,5608715	1259,083168	0,431581562	False	0,431581562
ENSMUSG00000069729	Arid1b	371,7354449	599,4472764	275,626085	571,2982588	567,5659875	-0,431563825	False	0,431563825
ENSMUSG00000025366	Esyt1	206,3479497	426,554062	278,2678686	932,2957806	615,8417152	0,431395228	False	0,431395228
ENSMUSG00000017421	Zfp207	1381,062869	1756,696018	1024,131428	1826,355064	2237,645215	-0,431378126	False	0,431378126
ENSMUSG00000051483	Cbr1	1208,719825	748,3625998	1629,980458	295,7705552	998,1332883	0,431374794	False	0,431374794
ENSMUSG00000018481	Appbp2	359,3700247	492,1777638	299,4021371	266,5308806	802,4208789	-0,431167383	False	0,431167383
ENSMUSG00000032180	Tmed1	92,74065154	97,17355849	125,044422	84,34521537	28,70448672	0,431166935	False	0,431166935
ENSMUSG00000024456	Diaph1	965,2756147	599,4472764	715,9233455	706,2506034	531,0330044	-0,431135808	False	0,431135808
ENSMUSG00000025911	Adhfe1	1725,748957	979,3075505	1229,309951	1153,842546	1279,959158	-0,431124836	False	0,431124836
ENSMUSG00000060098	Prmt7	156,1134301	304,1406181	210,4620905	520,6911296	237,4643902	0,430965737	False	0,430965737
ENSMUSG00000022706	Mrpl40	345,458297	209,4910482	256,2530056	91,0928326	240,073889	-0,430945267	False	0,430945267
ENSMUSG00000031271	Serpina7	476,8415166	990,6654989	642,8340002	877,1902399	2986,571369	0,430936386	False	0,430936386
ENSMUSG00000022450	Ndufa6	1993,151169	990,6654989	2686,693883	687,1323546	913,3245776	0,430780818	False	0,430780818
ENSMUSG00000072825	Cep170b	471,4316453	729,4326858	359,2825645	349,7514931	645,8509513	-0,430718388	False	0,430718388
ENSMUSG00000021892	Sh3bp5	994,6434877	233,4689392	737,9382085	328,3840385	193,1029107	-0,430679493	False	0,430679493
ENSMUSG00000022744	Cldnd1	166,160334	258,7088246	123,2832329	143,9491676	377,0725756	-0,430599421	False	0,430599421
ENSMUSG00000002342	Tmem161a	261,9923406	311,7125837	412,1182358	495,9498664	194,4076601	-0,430439569	False	0,430439569
ENSMUSG00000074925	Ptar1	117,4714919	282,6867156	87,17885756	104,5880671	701,9551753	-0,430260482	False	0,430260482
ENSMUSG00000016921	Srsf6	1580,45527	1823,581715	2227,904138	1172,960795	2797,382706	-0,430185411	False	0,430185411
ENSMUSG00000020850	Prpf8	3375,759716	1443,72144	2405,784231	1242,686173	2506,423591	-0,429581964	False	0,429581964
ENSMUSG00000040018	Cox15	394,9206078	272,5907615	293,2379754	192,307091	260,9498793	-0,429490703	False	0,429490703
ENSMUSG00000001435	Col18a1	12431,1115	2233,729851	9231,272362	7429,12657	2443,79562	-0,429353882	False	0,429353882
ENSMUSG00000033594	Spata2l	433,5625459	70,6716789	583,8341673	123,7063159	32,61873491	0,429318511	False	0,429318511
ENSMUSG00000035266	Helq	51,00735834	181,7271743	68,68637263	195,6808997	123,9511927	0,429318511	False	0,429318511
ENSMUSG00000002814	Top3a	85,01226391	652,4510356	114,4772877	417,2276654	619,7559633	0,429318511	False	0,429318511
ENSMUSG00000045664	Cdc42ep2	64,91845608	111,0554954	100,3877754	155,1951963	87,41820957	0,429305141	False	0,429305141
ENSMUSG00000051469	Zfp24	259,6738243	246,0888819	192,8502001	322,7610242	298,7876118	-0,429219952	False	0,429219952
ENSMUSG00000002028	Kmt2a	442,8366111	1096,673017	678,9383756	1535,08292	596,2704742	0,429192399	False	0,429192399
ENSMUSG00000018209	Stk4	301,4071175	329,3805035	181,4024713	185,5594738	405,7770623	0,42897383	False	0,42897383
ENSMUSG00000044134	Fam109a	101,2418779	136,2953807	192,8502001	253,0356461	164,398424	0,428930495	False	0,428930495
ENSMUSG00000034064	Poglut1	144,5208486	107,2695126	95,98480277	194,5562968	148,7414312	0,428910042	False	0,428910042
ENSMUSG00000009013	Dynll1	289,0416973	868,2520551	2314,202401	389,1125936	713,6979199	0,428910042	False	0,428910042
ENSMUSG00000019143	Hars2	289,0416973	176,6791973	237,7605206	389,1125936	229,6358938	0,428910042	False	0,428910042
ENSMUSG00000034940	Synrg	212,5306598	398,7901881	206,9397124	323,885627	157,874677	-0,428891198	False	0,428891198
ENSMUSG00000028443	Nudt2	149,1578812	50,47977065	200,7755508	77,59759814	30,00923612	0,428743388	False	0,428743388
ENSMUSG00000040017	Saa4	3137,725377	3191,583499	4223,331322	2218,841466	1787,506673	0,428662343	False	0,428662343
ENSMUSG00000001175	Calm1	2742,804769	3632,019498	2817,021872	2037,780403	3743,326019	-0,428653345	False	0,428653345
ENSMUSG00000033294	Noc4l	115,1529757	470,7238613	154,9846357	322,7610242	969,4288016	0,42857351	False	0,42857351
ENSMUSG00000004677	Myo9b	358,5971859	1031,049315	482,5657974	2008,540729	868,9630981	0,428361499	False	0,428361499
ENSMUSG00000032469	Dbr1	88,87645772	138,8193693	66,04458906	101,2142584	236,1596408	-0,428360949	False	0,428360949
ENSMUSG00000090623	Cfhr3	142,2023324	20,19190826	105,6713425	26,99046892	22,18073974	-0,428360949	False	0,428360949
ENSMUSG00000030738	Eif3c	1699,472439	2211,013954	1300,638107	1262,929025	2082,380037	-0,428313402	False	0,428313402
ENSMUSG00000026317	Cln8	289,0416973	265,0187959	216,6262521	214,7991485	615,8417152	-0,428289357	False	0,428289357
ENSMUSG00000058594	Fbxo18	289,0416973	97,17355849	154,1040411	214,7991485	23,48548914	-0,428289357	False	0,428289357
ENSMUSG00000038462	Uqcrfs1	1731,158829	996,9754702	1286,548595	571,2982588	1139,046223	-0,428232143	False	0,428232143
ENSMUSG00000030127	Cops7a	1000,053359	475,7718383	743,2217756	439,7197228	424,0435539	-0,428212302	False	0,428212302
ENSMUSG00000034247	Plekhm1	308,3626664	252,3988532	235,1187371	256,4094547	414,9103081	0,428171392	False	0,428171392
ENSMUSG00000030062	Rpn1	2347,884161	1943,47117	2176,829656	1114,481446	3158,798289	0,428014583	False	0,428014583
ENSMUSG00000026245	Farsb	414,2415769	307,9266009	258,8947891	256,4094547	249,2071347	-0,427885859	False	0,427885859
ENSMUSG00000079164	Tlr5	119,7900082	45,43179358	161,1487973	98,9650527	23,48548914	0,427885844	False	0,427885844
ENSMUSG00000042046	Dstyk	144,5208486	210,7530424	107,4325315	210,300737	186,5791637	-0,427846711	False	0,427846711
ENSMUSG00000057236	Rbbp4	751,1992774	306,6646067	373,3720768	169,8150336	558,4327417	-0,427812144	False	0,427812144
ENSMUSG00000087385	Frg2f1	84,23942515	83,29162157	130,3279891	129,3293302	62,62797103	-0,427688451	False	0,427688451
ENSMUSG00000004610	Etfb	323,0466029	690,3108636	1150,937039	1014,39179	434,481549	0,427552608	False	0,427552608
ENSMUSG00000025815	Dhtkd1	802,9794746	61,83771904	597,0430851	111,3356843	74,3707156	-0,427528064	False	0,427528064
ENSMUSG00000038990	Cables2	408,8317055	407,624148	586,4759509	547,6815985	304,0066094	-0,427404394	False	0,427404394
ENSMUSG00000020922	Lsm12	394,9206078	386,1702454	450,8643947	141,6999618	531,0330044	0,427238877	False	0,427238877
ENSMUSG00000063524	Eno1	4261,432938	2592,136223	3169,259681	1011,017982	2464,67161	-0,427192754	False	0,427192754
ENSMUSG00000025950	Idh1	5613,127934	1312,474037	4174,898623	1184,206824	1058,151761	-0,427063776	False	0,427063776
ENSMUSG00000037526	Atg14	208,666466	148,9153234	158,5070138	212,5499427	280,5211202	0,426910626	False	0,426910626
ENSMUSG00000021666	Gfm2	551,8068766	138,8193693	340,7900796	410,4800481	71,76121681	-0,426851336	False	0,426851336
ENSMUSG00000017446	C1qtnf1	93,5134903	181,7271743	69,56696715	467,8347946	79,58971319	-0,426772078	False	0,426772078
ENSMUSG00000079104	Prps1l3	227,987435	88,33959863	61,64161646	33,73808615	169,6174215	-0,426669958	False	0,426669958
ENSMUSG00000029038	Ssu72	461,3847414	343,2624404	223,6710083	204,6777226	259,6451299	-0,426658286	False	0,426658286
ENSMUSG00000037960	Card19	111,2887818	225,8969736	261,5365727	149,5721819	382,2915732	0,426533708	False	0,426533708
ENSMUSG00000029213	Commd8	189,3454969	294,044664	140,8951233	213,6745456	266,1688769	-0,426399433	False	0,426399433
ENSMUSG00000020532	Acaca	1503,944232	2641,353999	1119,235636	2004,042317	1309,968394	-0,426237268	False	0,426237268
ENSMUSG00000025137	Pcyt2	8383,754899	13255,98777	14891,73394	20456,52623	11265,20629	0,426205288	False	0,426205288
ENSMUSG00000027630	Tbl1xr1	289,8145361	778,6504622	215,7456576	274,4031007	1389,558107	-0,425798435	False	0,425798435
ENSMUSG00000027506	Tpd52	190,1183556	454,3179358	255,372411	325,0102299	545,3852477	0,425704996	False	0,425704996
ENSMUSG00000038366	Lasp1	2159,311503	470,7238613	1607,965595	398,1094166	273,9973733	-0,425334844	False	0,425334844
ENSMUSG00000034575	Papd7	272,8120833	112,3174897	366,3273207	213,6745456	104,3799517	0,425225756	False	0,425225756
ENSMUSG00000020053	Igf1	11918,7194	18201,7433	13495,99163	8876,490466	15497,81333	-0,425167944	False	0,425167944
ENSMUSG00000055707	Klhl26	159,9776239	109,7935012	139,1339343	214,7991485	109,5989493	0,425118145	False	0,425118145
ENSMUSG00000027649	Ctnnbl1	180,8442705	697,8828292	134,7309617	1240,436967	669,3364404	-0,424666466	False	0,424666466
ENSMUSG00000031902	Nfatc3	441,2909336	222,1109908	222,7904138	209,1761341	328,7968479	-0,424533639	False	0,424533639
ENSMUSG00000015522	Arnt	486,1155818	312,974578	437,6554769	272,1538949	652,3746983	0,424401451	False	0,424401451
ENSMUSG00000026395	Ptprc	120,562847	87,07760436	95,10420825	107,9618757	161,7889252	0,424327465	False	0,424327465
ENSMUSG00000040648	Ppip5k2	415,0144156	169,1072317	293,2379754	309,2657897	130,4749397	-0,424314195	False	0,424314195
ENSMUSG00000020716	Nf1	291,3602136	726,9086973	390,9839673	494,8252635	707,1741729	-0,424305564	False	0,424305564
ENSMUSG00000046139	Patl1	285,9503422	381,1222684	213,103874	235,0420002	791,9828837	-0,424207812	False	0,424207812
ENSMUSG00000026749	Nek6	709,4659843	2038,12074	951,922677	1573,319417	1677,907724	0,424110876	False	0,424110876
ENSMUSG00000026511	Srp9	633,7277855	1356,643836	908,7735455	472,3332061	2092,818032	-0,424058307	False	0,424058307
ENSMUSG00000047867	Gimap6	142,9751711	258,7088246	224,5516028	338,5054644	191,7981613	0,423824258	False	0,423824258
ENSMUSG00000030452	Nipa2	292,9058911	329,3805035	218,3874412	291,2721437	420,1293057	-0,423547316	False	0,423547316
ENSMUSG00000050708	Ftl1	22518,20303	7459,648107	6960,219093	2695,673083	16790,81998	-0,423419018	False	0,423419018
ENSMUSG00000019689	Fmc1	214,0763373	109,7935012	287,0738138	37,11189476	88,72295896	0,4232964	False	0,4232964
ENSMUSG00000027936	Crtc2	568,0364907	1277,138197	761,7142605	1657,664633	1212,112189	0,423266294	False	0,423266294
ENSMUSG00000017009	Sdc4	3403,581911	7921,538009	4800,120733	2538,228681	17451,02318	-0,423231776	False	0,423231776
ENSMUSG00000036898	Zfp157	95,05916782	127,4614209	169,074148	249,6618375	271,3878745	0,423162971	False	0,423162971

ENSMUSG00000008333	Snrpb2	183,9356255	253,6608475	157,6264192	137,2015503	618,4512139	-0,422904151	False	0,422904151
ENSMUSG00000005951	Shpk	764,3375364	371,0263142	436,7748823	570,1736559	242,6833878	-0,422808499	False	0,422808499
ENSMUSG00000039318	Rab3gap2	324,5922804	381,1222684	242,1634932	581,4196846	430,5673008	-0,422647298	False	0,422647298
ENSMUSG00000032051	Fdx1	673,9154012	278,9007328	502,8194714	101,2142584	444,9195442	-0,422526978	False	0,422526978
ENSMUSG00000034022	Cpsf1	657,6857871	940,1857283	881,4751154	895,1838858	1855,353642	0,422521349	False	0,422521349
ENSMUSG00000017221	Psmd3	1371,788804	1259,470278	808,3857701	672,5125172	1838,3919	0,42238596	False	0,42238596
ENSMUSG00000068798	Rap1a	711,0116618	453,0559415	419,1629919	233,9173973	952,4670595	0,421795976	False	0,421795976
ENSMUSG00000028066	Pmf1	493,0711307	278,9007328	368,0885097	158,5690049	255,7308817	-0,42174307	False	0,42174307
ENSMUSG00000032316	Clk3	756,6091488	1013,381396	1225,787573	2597,832633	1722,269203	0,421557122	False	0,421557122
ENSMUSG00000022476	Polr3h	87,3307802	194,347117	133,8503672	116,9586986	118,7321951	0,42143703	False	0,42143703
ENSMUSG00000017299	Dnttip1	159,2047851	248,6128704	118,8802603	143,9491676	364,0250816	-0,421374519	False	0,421374519
ENSMUSG00000029763	Exoc4	323,0466029	117,3654668	241,2828987	201,303914	60,01847224	-0,421016638	False	0,421016638
ENSMUSG00000028454	Pigo	185,4813031	584,3033452	248,3276549	468,9593975	455,3575394	0,420971168	False	0,420971168
ENSMUSG00000002496	Tsc2	745,7894061	657,4990127	1001,23597	1276,424259	557,1279923	-0,420759499	False	0,420759499
ENSMUSG00000054072	Iigp1	6796,34408	5079,526921	2420,754338	2273,947006	1568,308775	-0,420064755	False	0,420064755
ENSMUSG00000022299	Slc25a32	254,263953	133,7713922	106,551937	190,0578853	183,9696649	-0,419888063	False	0,419888063
ENSMUSG00000058833	Rex1bd	367,0984123	580,5173624	715,0427509	274,4031007	375,7678262	-0,419870091	False	0,419870091
ENSMUSG00000045319	Proser2	339,2762169	87,07760436	253,611222	228,2943829	31,31398552	-0,419841717	False	0,419841717
ENSMUSG00000026553	Copa	3391,98933	8445,265629	4537,703566	9498,395854	13749,44914	0,419830734	False	0,419830734
ENSMUSG00000043019	Edem3	446,7008049	281,4247213	251,850033	309,2657897	597,5752236	0,419811479	False	0,419811479
ENSMUSG00000005371	Fbxo11	364,0070573	837,9641927	272,1037069	829,9569193	734,5739102	-0,41980981	False	0,41980981
ENSMUSG00000058624	Gda	388,7378977	177,9411915	290,5961919	206,9269284	250,5118841	-0,419781964	False	0,419781964
ENSMUSG00000038619	Ensa	317,6367315	229,6829564	184,9248494	114,7094929	237,4643902	-0,41966657	False	0,41966657
ENSMUSG00000029823	Luc7l2	1888,817936	2526,512521	3274,050429	4195,893314	4265,225777	0,419663683	False	0,419663683
ENSMUSG00000007659	Bcl2l1	415,7872544	1673,404397	310,8498659	335,1316557	1333,453883	-0,419627578	False	0,419627578
ENSMUSG00000032396	Dis3l	279,7676321	131,2474037	174,3577151	209,1761341	155,2651782	-0,419510802	False	0,419510802
ENSMUSG00000031029	Eif3f	1030,194071	928,8277799	693,0278879	551,0554071	1377,815363	0,419466427	False	0,419466427
ENSMUSG00000001774	Chordc1	677,779595	242,3028991	906,131762	680,3847373	270,0831251	0,418904646	False	0,418904646
ENSMUSG00000006378	Gcat	236,4886614	108,5315069	316,133433	68,60077517	104,3799517	0,418762602	False	0,418762602
ENSMUSG00000078908	Mon1b	153,022075	111,0554954	114,4772877	82,09600963	62,62797103	-0,418678396	False	0,418678396
ENSMUSG00000039206	Daglb	204,8022721	97,17355849	153,2234466	57,35474645	110,9036987	-0,418594642	False	0,418594642
ENSMUSG00000022961	Son	2425,940876	10820,33884	3242,349026	20019,05572	11257,37779	0,418495009	False	0,418495009
ENSMUSG00000026000	Lancl1	210,9849822	292,7826697	212,2232795	490,326852	157,874677	-0,41836053	False	0,41836053
ENSMUSG00000022742	Cpox	1460,665262	330,6424977	875,3109537	1093,113991	332,7110961	-0,418181741	False	0,418181741
ENSMUSG00000034377	Tulp4	217,1676923	598,1852821	291,4767864	290,1475409	481,4525273	0,41797721	False	0,41797721
ENSMUSG00000022557	Bop1	246,5355653	329,3805035	173,4771206	242,9142203	202,2361565	0,417959377	False	0,417959377
ENSMUSG00000034075	Zdhhc5	894,1744486	531,299586	430,6107207	426,2244883	669,3364404	-0,41782476	False	0,41782476
ENSMUSG00000015942	Gtf2ird2	81,92090886	200,6570883	121,5220439	366,6205361	61,32322164	-0,41779823	False	0,41779823
ENSMUSG00000026728	Vim	321,5009253	314,2365723	379,5362385	240,6650145	404,4723129	-0,417796457	False	0,417796457
ENSMUSG00000025722	Wdr73	134,4739447	666,3329725	179,6412823	560,0522301	651,0699489	0,417792257	False	0,417792257
ENSMUSG00000075486	Commd6	319,9552478	138,8193693	239,5217097	79,84680388	146,1319324	-0,417713705	False	0,417713705
ENSMUSG00000054499	Dedd2	282,8589872	557,8014656	377,7750494	589,2919047	1115,560734	0,417444411	False	0,417444411
ENSMUSG00000027395	Polr1b	166,160334	230,9449507	221,9098193	450,9657515	290,9591154	0,417397485	False	0,417397485
ENSMUSG00000030407	Qpctl	207,1207884	417,7201021	417,4018029	283,3999236	276,6068721	0,417364637	False	0,417364637
ENSMUSG00000061353	Cxcl12	5613,127934	604,4952535	4203,077648	527,4387468	780,2401391	-0,417358832	False	0,417358832
ENSMUSG00000055670	Zzef1	518,5748098	442,9599874	388,3421837	1132,475092	738,4881584	-0,417223699	False	0,417223699
ENSMUSG00000039523	Cep104	110,5159431	128,7234151	82,77588496	94,46664122	250,5118841	-0,416972074	False	0,416972074
ENSMUSG00000032026	Rexo2	887,2188997	896,015929	1184,399631	475,7070147	570,1754863	0,416793942	False	0,416793942
ENSMUSG00000024213	Nudt3	154,5677526	357,1443773	221,0292247	115,8340958	544,0804983	-0,416179388	False	0,416179388
ENSMUSG00000002108	Nr1h3	1442,117131	1288,496146	1924,099028	1285,421082	427,9578021	0,415994705	False	0,415994705
ENSMUSG00000066839	Ecsit	511,619261	484,6057982	682,4607537	290,1475409	319,6636021	0,415675505	False	0,415675505
ENSMUSG00000015341	Golga7	877,1719958	413,9341193	539,8044413	434,0967084	657,5936958	-0,415663285	False	0,415663285
ENSMUSG00000035666	Gtf3c4	221,0318862	73,19566744	108,3131261	80,97140676	165,7031734	-0,415653277	False	0,415653277
ENSMUSG00000028248	Pnisr	1094,339688	781,1744507	620,8191372	1459,734527	1058,151761	0,415645392	False	0,415645392
ENSMUSG00000004633	Chn2	603,5870737	219,5870023	452,6255837	211,4253399	176,1411685	-0,415243778	False	0,415243778
ENSMUSG00000025958	Creb1	193,9825295	141,3433578	170,835337	258,6586605	80,89446258	0,415122771	False	0,415122771
ENSMUSG00000022204	Ngdn	181,6171093	299,0926411	242,1634932	317,1380098	623,6702115	0,415081273	False	0,415081273
ENSMUSG00000005779	Psmb4	928,1793541	1327,617968	1240,75768	696,1291775	1289,092404	-0,415048562	False	0,415048562
ENSMUSG00000022836	Mylk	1412,749258	185,5131571	1883,59168	809,7140676	101,7704529	0,414980823	False	0,414980823
ENSMUSG00000071646	Mta2	695,5548865	658,7610069	553,0133591	521,8157324	1165,141211	-0,414623922	False	0,414623922
ENSMUSG00000019866	Crybg1	171,5702053	128,7234151	146,1786905	223,7959715	412,3008093	-0,41452453	False	0,41452453
ENSMUSG00000028647	Mycbp	251,1725979	129,9854094	188,4472275	57,35474645	110,9036987	-0,41451851	False	0,41451851
ENSMUSG00000031065	Cdk16	587,3574597	1089,101052	782,848529	1122,353666	949,8575607	0,414494412	False	0,414494412
ENSMUSG00000016554	Eif3d	965,2756147	1192,584581	750,2665318	724,2442493	1371,291616	-0,414464609	False	0,414464609
ENSMUSG00000026854	Usp20	97,37768411	164,0592546	160,2682028	401,4832252	73,0659662	-0,414391628	False	0,414391628
ENSMUSG00000021595	Nsun2	869,4436082	466,9378785	381,2974275	385,738785	652,3746983	-0,414391628	False	0,414391628
ENSMUSG00000030595	Nfkbib	367,0984123	1244,326346	517,7895783	489,2022492	2887,410414	0,414264162	False	0,414264162
ENSMUSG00000036606	Plxnb2	2456,081588	1130,746862	1843,084332	1649,792413	493,1952719	-0,414236402	False	0,414236402
ENSMUSG00000001768	Rin2	220,2590474	107,2695126	148,820474	165,3166221	50,88522646	-0,41396949	False	0,41396949
ENSMUSG00000038095	Sbno1	530,1673913	998,2374645	706,2368057	887,3116657	1086,856247	0,413704072	False	0,413704072
ENSMUSG00000038193	Hand2	109,7431043	248,6128704	146,1786905	274,4031007	257,0356311	0,413602723	False	0,413602723
ENSMUSG00000075602	Ly6a	264,3108569	119,8894553	345,1930522	352,0006988	181,3601661	0,413342606	False	0,413342606
ENSMUSG00000004996	Mri1	217,1676923	230,9449507	343,4318631	402,607828	163,0936746	-0,413108662	False	0,413108662
ENSMUSG00000048351	Coa7	182,389948	225,8969736	311,7304604	190,0578853	136,9986866	-0,412864159	False	0,412864159
ENSMUSG00000024642	Tle4	135,2467835	76,98165023	58,11923838	67,4761723	180,0554167	0,412846718	False	0,412846718
ENSMUSG00000000826	Dnajc5	728,0141146	1111,816948	546,8491975	987,4013213	1985,828581	-0,41282338	False	0,41282338
ENSMUSG00000020459	Mtif2	426,6069971	435,3880218	427,0883426	567,9244502	198,3219083	0,412791395	False	0,412791395
ENSMUSG00000019897	Ccdc59	238,0343389	344,5244347	260,6559782	178,8118566	724,1359151	-0,412727311	False	0,412727311
ENSMUSG00000021240	Abcd4	161,5233014	259,9708188	234,2381425	823,209302	121,3416939	-0,412666948	False	0,412666948
ENSMUSG00000001036	Epn2	120,562847	203,1810768	184,9248494	418,3522682	160,4841758	0,41264566	False	0,41264566
ENSMUSG00000036779	Papd5	498,481002	395,0042053	332,8647289	663,5156943	314,4446046	0,412592068	False	0,412592068
ENSMUSG00000052962	Mrpl35	266,6293732	237,254922	219,2680357	91,0928326	354,8918359	0,412543663	False	0,412543663
ENSMUSG00000056941	Commd7	353,9601534	176,6791973	265,9395453	93,34203834	108,2941999	-0,412488637	False	0,412488637
ENSMUSG00000030761	Myo7a	267,4022119	547,7055115	508,1030385	1356,271063	200,9314071	-0,412308314	False	0,412308314
ENSMUSG00000028522	Mier1	358,5971859	548,9675058	269,4619234	462,2117802	713,6979199	-0,412282741	False	0,412282741
ENSMUSG00000022969	Il10rb	119,7900082	145,1293406	163,7905809	136,0769475	90,02770836	-0,412066575	False	0,412066575
ENSMUSG00000032527	Pccb	2480,812429	1703,692259	1755,02488	1048,129876	1864,486888	-0,412034008	False	0,412034008
ENSMUSG00000059208	Hnrnpm	799,8881195	1236,754381	601,4460577	910,928326	1392,167606	-0,411362873	False	0,411362873
ENSMUSG00000020523	Fam114a2	537,8957789	548,9675058	666,6100523	1140,347312	404,4723129	-0,411285717	False	0,411285717
ENSMUSG00000035198	Tubg1	449,0193212	210,7530424	597,0430851	122,581713	114,8179469	0,411057521	False	0,411057521
ENSMUSG00000029681	Bcl7b	152,2492363	287,7346927	216,6262521	202,4285169	438,3957972	0,410977551	False	0,410977551
ENSMUSG00000033055	Ankrd54	163,068979	141,3433578	213,9844686	194,5562968	122,6464433	-0,410976964	False	0,410976964
ENSMUSG00000010601	Apol7a	2273,69164	25,23988532	1710,11456	249,6618375	16,96174215	-0,410943634	False	0,410943634
ENSMUSG00000032624	Eml4	159,9776239	300,3546353	178,7606877	120,3325073	296,178113	-0,410843697	False	0,410843697
ENSMUSG00000038861	Pi4kb	454,4291925	295,3066583	242,1634932	218,1729571	341,8443419	-0,41071597	False	0,41071597
ENSMUSG00000028398	Dmac1	185,4813031	159,0112775	246,5664658	97,84044983	153,9604288	0,410702833	False	0,410702833
ENSMUSG00000073563	Csnk1g3	407,286028	541,3955402	306,4468933	372,2435505	271,3878745	0,41064068	False	0,41064068
ENSMUSG00000045078	Rnf216	282,0861484	442,9599874	212,2232795	376,741962	340,5395925	-0,410552906	False	0,410552906
ENSMUSG00000032382	Snx1	295,2244074	222,1109908	218,3874412	208,0515312	182,6649155	-0,410531215	False	0,410531215
ENSMUSG00000030556	Lrrc28	213,3034985	555,2774771	207,8203069	256,4094547	160,4841758	-0,410476578	False	0,410476578
ENSMUSG00000039001	Rps21	886,4460609	1934,63721	2839,036735	1584,565446	1178,188705	0,410465875	False	0,410465875
ENSMUSG00000025372	Baiap2	1027,102716	730,6946801	1364,921507	876,065637	575,3944839	0,410237522	False	0,410237522
ENSMUSG00000031858	Mau2	398,7848016	397,5281938	464,953907	644,3974454	300,0923612	-0,410203831	False	0,410203831
ENSMUSG00000014668	Chfr	209,4393047	300,3546353	157,6264192	348,6268902	210,0646528	-0,410022853	False	0,410022853
ENSMUSG00000021843	Ktn1	293,6787299	417,7201021	221,0292247	502,6974836	441,005296	-0,410001643	False	0,410001643
ENSMUSG00000022420	Dnal4	89,64929649	113,579484	103,9101535	67,4761723	120,0369445	-0,409914125	False	0,409914125
ENSMUSG00000020709	Adap2	555,6710705	738,2666457	635,7892441	389,1125936	247,9023853	0,409910848	False	0,409910848
ENSMUSG00000030847	Bag3	553,3525542	455,5799301	416,5212084	483,5792348	1786,201924	-0,409808993	False	0,409808993
ENSMUSG00000025437	Usp33	553,3525542	498,4877351	416,5212084	709,624412	1381,729611	-0,409808993	False	0,409808993
ENSMUSG00000025780	Itih5	188,5726581	146,3913349	92,46242469	82,09600963	250,5118841	0,409758536	False	0,409758536
ENSMUSG00000015656	Hspa8	17870,35071	13070,47461	23739,94769	4615,370185	19105,44541	0,409748808	False	0,409748808
ENSMUSG00000020372	Rack1	3390,443652	4881,393821	5165,567459	5168,674798	4503,994917	0,409731131	False	0,409731131
ENSMUSG00000047719	Ubiad1	163,068979	138,8193693	157,6264192	104,5880671	216,5883998	0,409473616	False	0,409473616

ENSMUSG00000061024	Rrs1	97,37768411	189,2991399	153,2234466	129,3293302	289,654366	0,409386402	False	0,409386402
ENSMUSG00000030718	Ppme1	429,6983521	928,8277799	570,6252495	626,4037995	1636,155743	0,409219342	False	0,409219342
ENSMUSG00000027519	Rab22a	374,8268	663,808984	285,3126248	282,2753208	1103,817989	-0,409121109	False	0,409121109
ENSMUSG00000109336	Samd4b	401,8761567	1529,537051	533,6402796	959,2862495	2465,976359	0,409116582	False	0,409116582
ENSMUSG00000010607	Pigyl	230,3059513	94,64956996	173,4771206	86,59442111	63,93272043	-0,40880629	False	0,40880629
ENSMUSG00000026193	Fn1	18317,82436	20591,96044	13798,03555	25016,79088	14939,38059	-0,40878528	False	0,40878528
ENSMUSG00000025860	Xiap	1172,396403	495,9637466	883,2363044	378,9911677	366,6345804	-0,408589068	False	0,408589068
ENSMUSG00000057177	Gsk3a	868,6707694	1395,765658	705,3562112	654,5188713	2391,605644	-0,408374703	False	0,408374703
ENSMUSG00000038803	Ost4	516,2562936	300,3546353	685,1025372	131,578536	379,6820744	0,408232464	False	0,408232464
ENSMUSG00000015133	Lrrk1	193,9825295	318,0225551	146,1786905	386,8633878	227,026395	-0,408193711	False	0,408193711
ENSMUSG00000072889	Nfxl1	146,0665262	180,4651801	110,0743151	212,5499427	883,3153414	-0,408147728	False	0,408147728
ENSMUSG00000048264	Dip2c	173,1158829	100,9595413	95,10420825	87,71902399	130,4749397	-0,407965359	False	0,407965359
ENSMUSG00000003948	Mmd	1536,40346	342,0004461	1157,981795	597,1641248	280,5211202	-0,407944546	False	0,407944546
ENSMUSG00000025485	Ric8a	230,3059513	1207,728513	305,5662987	1801,6138	1360,853621	0,407933743	False	0,407933743
ENSMUSG00000043702	Pde12	107,424588	156,487289	123,2832329	80,97140676	450,1385418	-0,407839797	False	0,407839797
ENSMUSG00000029128	Rab28	151,4763975	175,417203	65,16399454	136,0769475	200,9314071	0,40761007	False	0,40761007
ENSMUSG00000007836	Hnrnpa0	341,5947332	482,0818097	326,7005672	257,5340576	952,4670595	-0,407522497	False	0,407522497
ENSMUSG00000074227	Spint2	791,3868931	709,2407776	1049,668669	362,1221247	439,7005466	0,407478932	False	0,407478932
ENSMUSG00000070738	Dgkd	207,8936272	206,9670596	156,7458247	237,2912059	302,70186	-0,40741852	False	0,40741852
ENSMUSG00000029131	Dnajb6	544,078489	295,3066583	280,0290576	182,1856652	721,5264163	0,407237425	False	0,407237425
ENSMUSG00000057572	Zbtb8os	264,3108569	249,8748647	350,4766193	195,6808997	173,5316697	0,407082517	False	0,407082517
ENSMUSG00000023977	Ubr2	1204,855631	1260,732272	908,7735455	2928,465878	1200,369445	-0,406867546	False	0,406867546
ENSMUSG00000032998	Foxj3	109,7431043	150,1773177	82,77588496	125,9555216	387,5105708	-0,406847856	False	0,406847856
ENSMUSG00000030965	Abraxas2	359,5576404	198,1330998	141,7757179	301,3935696	268,7783757	-0,406755005	False	0,406755005
ENSMUSG00000029458	Brap	1018,601489	768,5545081	798,6992304	1556,450374	1032,056773	-0,406370243	False	0,406370243
ENSMUSG00000037278	Tmem97	1136,84582	663,808984	1506,697225	556,6784215	545,3852477	0,406352925	False	0,406352925
ENSMUSG00000026965	Anapc2	519,3476486	498,4877351	391,8645618	901,931503	788,0686355	-0,406345486	False	0,406345486
ENSMUSG00000001054	Rmnd5b	246,5355653	523,7276204	326,7005672	409,3554453	506,2427658	0,406173174	False	0,406173174
ENSMUSG00000066621	Tecpr1	262,7651794	330,6424977	559,1775207	897,4330916	198,3219083	-0,40593005	False	0,40593005
ENSMUSG00000039765	Cc2d2a	66,4641336	162,7972603	88,05945209	213,6745456	187,8839131	0,405901846	False	0,405901846
ENSMUSG00000047714	Ppp1r2	366,3255736	517,4176491	276,5066795	404,8570338	631,4987079	-0,405812089	False	0,405812089
ENSMUSG00000032497	Lrrfip2	484,5699043	1501,773177	641,9534057	1216,820307	2035,409059	0,405763783	False	0,405763783
ENSMUSG00000015095	Fbxw5	513,1649385	1108,030966	679,8189701	1363,01868	1398,691353	0,405728017	False	0,405728017
ENSMUSG00000026872	Zeb2	185,4813031	53,00375918	140,0145288	109,0864785	105,6847011	-0,405697229	False	0,405697229
ENSMUSG00000031813	Mvb12a	592,7673311	565,3734312	904,3705729	704,0013976	447,529043	-0,405484628	False	0,405484628
ENSMUSG00000020349	Ppp2ca	1456,801068	1985,116981	1169,429524	1099,861608	4025,151888	-0,405481879	False	0,405481879
ENSMUSG00000032596	Uba7	258,1281468	123,6754381	280,0290576	341,879273	93,94195655	0,405399495	False	0,405399495
ENSMUSG00000022761	Lztr1	336,9577006	493,4397581	401,5511015	977,2798954	254,4261323	-0,405320638	False	0,405320638
ENSMUSG00000019763	Rmnd1	489,9797756	280,1627271	625,2221098	648,8958569	143,5224336	0,405264753	False	0,405264753
ENSMUSG00000025132	Arhgdia	1519,401008	3979,067921	2012,15848	3359,188778	6287,587342	0,405241255	False	0,405241255
ENSMUSG00000032220	Myo1e	612,0883001	441,6979931	398,0287234	462,2117802	204,8456553	-0,405185762	False	0,405185762
ENSMUSG00000073490	Ifi207	85,78510267	232,206945	113,5966932	601,6625363	121,3416939	0,4051218	False	0,4051218
ENSMUSG00000027634	Ndrg3	268,1750507	99,69754702	202,5367398	160,8182106	80,89446258	-0,40499139	False	0,40499139
ENSMUSG00000039018	Mtg1	162,2961402	85,8156101	108,3131261	122,581713	37,8377325	-0,404884919	False	0,404884919
ENSMUSG00000038000	Acd	214,8491761	426,554062	288,8350028	569,049053	284,4353684	0,404776625	False	0,404776625
ENSMUSG00000009894	Snap47	792,9325706	193,0851227	1049,668669	250,7864404	95,24670595	0,404663916	False	0,404663916
ENSMUSG00000021116	Eif2s1	583,4932659	684,0008922	530,9984961	336,2562586	772,4116427	0,404653905	False	0,404653905
ENSMUSG00000021368	Tbc1d7	157,6591076	98,43555276	208,7009014	42,73490912	74,3707156	0,404628318	False	0,404628318
ENSMUSG00000036617	Etl4	210,9849822	60,57572477	159,3876083	94,46664122	31,31398552	-0,404600842	False	0,404600842
ENSMUSG00000031575	Ash2l	250,3997591	151,4393119	140,0145288	163,0674164	189,1886625	-0,40440754	False	0,40440754
ENSMUSG00000029597	Sds	1841,674772	812,7243074	1775,278554	2185,10338	2437,271873	0,404248877	False	0,404248877
ENSMUSG00000046179	E2f8	210,9849822	109,7935012	279,1484631	34,86268902	122,6464433	0,403892301	False	0,403892301
ENSMUSG00000020738	Sumo2	132,1554284	78,2436445	90,70123565	33,73808615	174,8364191	0,403770049	False	0,403770049
ENSMUSG00000029445	Hpd	36359,74527	4687,046704	27489,51916	5837,813507	9677,326274	-0,403460421	False	0,403460421
ENSMUSG00000022771	Ppil2	763,5646976	666,3329725	492,2523372	1009,893379	549,2994959	0,403380678	False	0,403380678
ENSMUSG00000028530	Jak1	1377,198675	1211,514495	1120,996825	1349,523446	1821,430158	0,403334978	False	0,403334978
ENSMUSG00000021772	Nkiras1	271,2664057	463,1518957	389,2227782	358,748316	438,3957972	0,403261668	False	0,403261668
ENSMUSG00000028859	Csf3r	92,74065154	215,8010195	125,9250165	404,8570338	122,6464433	0,403231629	False	0,403231629
ENSMUSG00000018909	Arrb1	169,2516891	124,9374323	206,0591179	223,7959715	58,71372284	0,403013835	False	0,403013835
ENSMUSG00000023932	Cdc5l	289,8145361	172,8932145	152,3428521	181,0610623	219,1978986	-0,402895989	False	0,402895989
ENSMUSG00000037570	Mcrs1	77,28387628	177,9411915	102,1489644	157,444402	341,8443419	0,402435217	False	0,402435217
ENSMUSG00000029833	Trim24	1238,087698	707,9787833	1253,086003	1636,297178	647,1557007	0,40232128	False	0,40232128
ENSMUSG00000033478	Fam160b1	643,7746894	487,1297867	590,8789235	891,8100772	768,4973945	-0,402249654	False	0,402249654
ENSMUSG00000078619	Smarcd2	901,9028362	224,6349794	682,4607537	268,7800863	281,8258696	-0,402225934	False	0,402225934
ENSMUSG00000078853	Igtp	711,0116618	343,2624404	538,0432522	77,59759814	146,1319324	-0,40215107	False	0,40215107
ENSMUSG00000045624	Esf1	183,9356255	507,321695	243,0440878	513,9435123	752,8404018	0,402017105	False	0,402017105
ENSMUSG00000040813	Tex264	948,273162	392,4802168	717,6845345	386,8633878	609,3179682	-0,401952873	False	0,401952873
ENSMUSG00000026027	Stradb	428,1526746	76,98165023	324,0587837	139,4507561	40,44723129	-0,401869799	False	0,401869799
ENSMUSG00000058301	Upf1	689,3721764	1130,746862	1303,279891	521,8157324	986,3905438	-0,40174263	False	0,40174263
ENSMUSG00000036023	Parp2	159,9776239	484,6057982	211,342685	574,6720674	443,6147948	0,401714049	False	0,401714049
ENSMUSG00000021486	Prelid1	2181,723287	1804,651801	2882,185867	1620,552738	1775,763929	0,401694885	False	0,401694885
ENSMUSG00000040782	Cop1	546,3970053	360,9303601	284,4320302	295,7705552	413,6055587	-0,401694001	False	0,401694001
ENSMUSG00000025271	Pfkfb1	602,0413962	224,6349794	700,9532386	795,0942302	125,2559421	0,401263161	False	0,401263161
ENSMUSG00000047879	Usp14	475,2958391	355,8823831	347,8348357	309,2657897	627,5844597	0,400983857	False	0,400983857
ENSMUSG00000029249	Rest	131,3825897	290,2586812	99,50718086	137,2015503	347,0633395	-0,400901562	False	0,400901562
ENSMUSG00000042699	Dhx9	641,4561731	352,0964003	198,1337672	145,0737704	846,7823583	0,400640515	False	0,400640515
ENSMUSG00000002227	Mov10	253,4911142	129,9854094	334,6259179	191,1824882	168,3126721	0,400614015	False	0,400614015
ENSMUSG00000052997	Uba2	292,9058911	381,1222684	221,9098193	338,5054644	576,6992333	-0,400463703	False	0,400463703
ENSMUSG00000040040	Ift88	78,05671504	267,5427844	103,0295589	263,157072	174,8364191	0,400463648	False	0,400463648
ENSMUSG00000039770	Ypel5	442,8366111	593,1373051	335,5065124	378,9911677	811,5541246	-0,40043373	False	0,40043373
ENSMUSG00000053581	Zfand2a	130,6097509	155,2252947	152,3428521	98,9650527	413,6055587	-0,400271543	False	0,400271543
ENSMUSG00000032125	Robo4	151,4763975	487,1297867	199,8949562	700,627589	302,70186	0,400149055	False	0,400149055
ENSMUSG00000033102	Cdc14b	706,3746292	213,277031	405,9540741	535,3109669	147,4366818	-0,400056317	False	0,400056317
ENSMUSG00000056268	Dennd1b	168,4788503	325,5945207	127,6862055	548,8062014	339,2348431	-0,399964823	False	0,399964823
ENSMUSG00000030930	Chst15	170,7973666	364,7163429	129,4473946	401,4832252	230,9406432	-0,399919803	False	0,399919803
ENSMUSG00000040525	Cblc	198,619562	156,487289	208,7009014	262,0324691	61,32322164	0,39973787	False	0,39973787
ENSMUSG00000030670	Cyp2r1	191,6640132	50,47977065	145,2980959	51,73173209	14,35224336	-0,399563685	False	0,399563685
ENSMUSG00000021731	Mrps30	314,5453765	198,1330998	152,3428521	83,2206125	414,9103081	0,399531344	False	0,399531344
ENSMUSG00000024830	Rps6kb2	371,7354449	596,9232879	779,326151	1358,520269	281,8258696	-0,399472208	False	0,399472208
ENSMUSG00000035498	Cdcp1	140,6566548	185,5131571	129,4473946	110,2110814	93,94195655	0,399343699	False	0,399343699
ENSMUSG00000040296	Ddx58	559,5352643	364,7163429	737,9382085	558,9276272	117,4274457	0,399270958	False	0,399270958
ENSMUSG00000073418	C4b	12583,36074	16595,2246	21965,54973	25567,84629	26036,27421	0,39925087	False	0,39925087
ENSMUSG00000031969	Acad8	805,2979908	417,7201021	428,8495317	610,6593593	467,100284	-0,3991549	False	0,3991549
ENSMUSG00000034785	Dio1	1470,712166	177,9411915	1451,21977	1938,815351	223,1121468	0,398660487	False	0,398660487
ENSMUSG00000002846	Timmdc1	369,4169286	135,0333865	486,96877	160,8182106	60,01847224	0,398579274	False	0,398579274
ENSMUSG00000028850	Gpatch3	39,4147769	167,8452374	51,95507673	231,6681916	105,6847011	0,398528119	False	0,398528119
ENSMUSG00000041936	Agrn	481,4785492	402,5761709	862,9826304	1494,597216	365,329831	-0,398271963	False	0,398271963
ENSMUSG00000028954	Nub1	510,8464222	427,8160562	305,5662987	470,0840003	673,2506886	0,398254167	False	0,398254167
ENSMUSG00000041740	Rnf10	1946,008005	2650,187959	1501,413658	1476,60357	4149,103081	-0,398235092	False	0,398235092
ENSMUSG00000022545	Ercc4	128,2912346	268,8047787	169,074148	295,7705552	240,073889	0,398233481	False	0,398233481
ENSMUSG00000048429	Timm29	380,2366713	297,8306468	185,8054439	159,6936078	501,0237683	0,397981369	False	0,397981369
ENSMUSG00000021281	Tnfaip2	697,8734028	1481,581268	1100,743151	2793,513533	529,728255	-0,397712887	False	0,397712887
ENSMUSG00000037254	Itih2	22015,85784	31010,9851	20507,2852	16712,72328	49070,32005	-0,397596225	False	0,397596225
ENSMUSG00000034424	Gcsh	973,0040024	229,6829564	738,818803	244,0388231	330,1015973	-0,397225156	False	0,397225156
ENSMUSG00000033760	Rbm4b	163,8418177	233,4689392	215,7456576	238,4158088	251,8166335	0,397027896	False	0,397027896
ENSMUSG00000028797	Tmem234	918,1324502	2000,260912	1269,817299	1208,948087	1591,794264	0,396978098	False	0,396978098
ENSMUSG00000070047	Fat1	332,320668	252,3988532	173,4771206	141,6999618	206,1504046	-0,396870666	False	0,396870666
ENSMUSG00000024386	Proc	6409,924699	4574,729215	8439,617888	4188,021094	3829,439479	0,396870272	False	0,396870272
ENSMUSG00000047260	Emc6	809,9350234	1495,463205	1090,176017	615,1577708	1741,840444	-0,396849704	False	0,396849704
ENSMUSG00000021555	Naa35	386,4193814	233,4689392	258,0141946	293,5213495	224,4168962	-0,396702014	False	0,396702014
ENSMUSG00000060743	H3f3a	1296,823444	617,1151961	710,6397783	418,3522682	985,0857944	-0,396660793	False	0,396660793
ENSMUSG00000053886	Sh2d4a	164,6146565	17,66791973	125,044422	49,48252635	14,35224336	-0,396652089	False	0,396652089
ENSMUSG00000024742	Fen1	34,77774433	102,2215356	26,41783563	55,10554071	377,0725756	-0,396652089	False	0,396652089

ENSMUSG00000024260	Sap130	577,3105558	448,0079645	438,5360714	1169,586986	452,7480406	-0,396652089	False	0,396652089
ENSMUSG00000005886	Ncoa2	598,9500412	571,6834026	356,6407809	788,346613	529,728255	0,396394408	False	0,396394408
ENSMUSG00000032932	Hspa13	575,7648783	658,7610069	452,6255837	437,4705171	1232,98818	-0,396293996	False	0,396293996
ENSMUSG00000021033	Gstz1	12526,94351	4157,009113	9518,346176	6540,690301	2463,366861	-0,396251619	False	0,396251619
ENSMUSG00000018999	Slc35b4	404,1946729	153,9633005	458,7897454	531,9371583	193,1029107	0,396205511	False	0,396205511
ENSMUSG00000031485	Plpbp	1051,060717	1306,164065	798,6992304	1185,331427	1454,795577	-0,396121784	False	0,396121784
ENSMUSG00000037815	Ctnna1	1667,78605	1576,230838	1199,369737	1262,929025	2194,588485	0,396016214	False	0,396016214
ENSMUSG00000026153	Fam135a	335,4120231	254,9228418	427,9689371	490,326852	290,9591154	-0,395873756	False	0,395873756
ENSMUSG00000025162	Csnk1d	654,5944321	1332,665945	497,5359043	918,8005461	1735,316697	-0,395800691	False	0,395800691
ENSMUSG00000001127	Araf	1124,4804	1720,098185	1479,398795	2483,123141	1510,899801	0,395752491	False	0,395752491
ENSMUSG00000001348	Acp5	1718,02057	559,0634599	726,4904797	371,1189476	1306,054146	-0,395532601	False	0,395532601
ENSMUSG00000028821	Syf2	427,3798358	614,5912076	324,9393782	405,9816367	748,9261536	-0,395348253	False	0,395348253
ENSMUSG00000025525	Apool	292,1330523	222,1109908	195,4919836	110,2110814	182,6649155	-0,395344812	False	0,395344812
ENSMUSG00000037266	Rsrp1	4419,092046	2322,06945	3360,348692	1549,702757	1581,356269	-0,395139037	False	0,395139037
ENSMUSG00000020572	Nampt	952,9101945	2193,346035	724,7292907	1829,728872	2332,891921	-0,394898053	False	0,394898053
ENSMUSG00000038181	Chpf2	261,2195018	416,4581078	343,4318631	597,1641248	387,5105708	0,394761289	False	0,394761289
ENSMUSG00000056054	S100a8	70,32832742	538,8715516	92,46242469	477,9562204	708,4789223	0,394761289	False	0,394761289
ENSMUSG00000028409	Smu1	506,2093896	256,184836	251,850033	174,3134451	665,4221922	0,394535714	False	0,394535714
ENSMUSG00000008668	Rps18	2623,014761	4603,755083	5472,014353	1996,170097	3636,336568	-0,393991266	False	0,393991266
ENSMUSG00000027770	Dhx36	416,5600932	965,4256136	317,0140275	1069,497331	1366,072618	-0,393977955	False	0,393977955
ENSMUSG00000018585	Atox1	852,4411554	1958,615101	2579,261352	1120,10446	2032,79956	0,393961131	False	0,393961131
ENSMUSG00000022488	Nckap1l	103,5603942	117,3654668	54,59686029	136,0769475	104,3799517	0,393950321	False	0,393950321
ENSMUSG00000021929	Kpna3	510,8464222	235,9929278	203,4173343	251,9110432	388,8153202	-0,393804568	False	0,393804568
ENSMUSG00000054277	Arfgap3	176,9800767	291,5206755	232,4769535	448,7165458	1094,684744	0,393500743	False	0,393500743
ENSMUSG00000051510	Mafg	1294,504928	985,6175219	1563,055275	1585,690049	1414,348346	-0,393300648	False	0,393300648
ENSMUSG00000048755	Mcat	277,4491158	196,8711055	169,9547425	364,3713304	118,7321951	0,393186243	False	0,393186243
ENSMUSG00000022665	Ccdc80	162,2961402	79,50563877	213,103874	143,9491676	113,5131975	0,392928131	False	0,392928131
ENSMUSG00000021945	Zmym2	295,9972462	318,0225551	225,4321973	403,7324309	280,5211202	-0,392890171	False	0,392890171
ENSMUSG00000045410	Akr1e1	720,2857269	51,74176491	548,6103865	192,307091	40,44723129	-0,39278738	False	0,39278738
ENSMUSG00000006390	Elovl1	822,3004436	832,9162157	1079,608883	795,0942302	358,806084	0,39277124	False	0,39277124
ENSMUSG00000055013	Agap1	106,6517493	343,2624404	140,0145288	330,6332443	287,0448672	0,39266891	False	0,39266891
ENSMUSG00000021054	Sgpp1	712,5573393	326,8565149	385,7004001	204,6777226	542,7757489	-0,392649849	False	0,392649849
ENSMUSG00000009073	Nf2	415,0144156	179,2031858	316,133433	133,8277417	219,1978986	-0,392627833	False	0,392627833
ENSMUSG00000034024	Cct2	1179,351952	1633,02058	955,4450551	898,5576944	2586,013304	-0,39231128	False	0,39231128
ENSMUSG00000019998	Stx7	249,6269204	234,7309335	190,2084165	210,300737	380,9868238	-0,392192441	False	0,392192441
ENSMUSG00000003546	Klc4	700,9647579	526,251609	591,759518	919,925149	311,8351058	0,392174567	False	0,392174567
ENSMUSG00000026374	Tsn	463,7032577	912,4218544	608,4908139	896,3084887	1303,444647	0,392033621	False	0,392033621
ENSMUSG00000071713	Csf2rb	116,6986532	198,1330998	88,94004661	245,163426	121,3416939	-0,391882845	False	0,391882845
ENSMUSG00000028327	Stra6l	4107,638024	4199,916918	3626,288237	5389,096961	1994,961827	0,391734491	False	0,391734491
ENSMUSG00000026342	Slc35f5	384,8737039	391,2182225	164,6711754	403,7324309	504,9380164	0,391721197	False	0,391721197
ENSMUSG00000026482	Rgl1	194,7553682	93,38757569	147,059285	148,4475791	113,5131975	-0,39170953	False	0,39170953
ENSMUSG00000037070	Rbmxl1	109,7431043	148,9153234	83,65647948	95,59124409	220,502648	-0,3915811	False	0,3915811
ENSMUSG00000042772	Smg7	444,3822886	585,5653395	582,9535728	721,9950436	797,2018813	0,391579672	False	0,391579672
ENSMUSG00000049354	Dcaf7	566,4908131	268,8047787	328,4617563	188,9332824	431,8720502	-0,391448608	False	0,391448608
ENSMUSG00000042390	Gatad2b	319,9552478	598,1852821	243,9246823	320,5118184	1202,978944	-0,39143438	False	0,39143438
ENSMUSG00000034947	Tmem106a	191,6640132	171,6312202	219,2680357	289,022938	146,1319324	-0,391308015	False	0,391308015
ENSMUSG00000033016	Nfatc1	160,7504627	248,6128704	125,9250165	122,581713	229,6358938	-0,391079119	False	0,391079119
ENSMUSG00000055320	Tead1	196,3010458	657,4990127	149,7010685	572,4228617	1133,827226	-0,390983339	False	0,390983339
ENSMUSG00000020594	Pum2	684,7351438	845,5361583	522,1925509	788,346613	825,906368	-0,390964183	False	0,390964183
ENSMUSG00000027163	Commd9	207,8936272	445,4839759	233,357548	158,5690049	630,1939585	-0,390734736	False	0,390734736
ENSMUSG00000052934	Fbxo31	272,0392445	1482,843263	356,6407809	2344,796987	2460,757362	0,39065689	False	0,39065689
ENSMUSG00000022889	Mrpl39	542,5328115	295,3066583	413,8794248	191,1824882	310,5303564	-0,390499862	False	0,390499862
ENSMUSG00000024325	Ring1	267,4022119	204,4430711	350,4766193	164,1920193	117,4274457	0,390306804	False	0,390306804
ENSMUSG00000019838	Slc16a10	1314,598736	468,1998727	1002,997159	504,9466894	241,3786384	-0,390304983	False	0,390304983
ENSMUSG00000024661	Fth1	16286,03125	31662,17414	13145,51501	12426,86173	49192,9665	-0,39017307	False	0,39017307
ENSMUSG00000027425	Kat14	256,5824693	214,5390252	206,0591179	336,2562586	250,5118841	0,390138517	False	0,390138517
ENSMUSG00000019802	Sec63	2388,844616	1720,098185	1822,830658	1428,245647	1791,420921	-0,390132475	False	0,390132475
ENSMUSG00000049555	Tmie	587,3574597	241,0409048	448,2226111	129,3293302	176,1411685	-0,390023351	False	0,390023351
ENSMUSG00000029675	Eln	62,59993979	82,0296273	116,2384768	177,6872537	88,72295896	0,389983804	False	0,389983804
ENSMUSG00000029716	Tfr2	5383,594822	7472,26805	7443,665485	6537,316493	4108,65585	-0,389903342	False	0,389903342
ENSMUSG00000020444	Guk1	258,1281468	213,277031	338,148296	281,1507179	173,5316697	0,38956862	False	0,38956862
ENSMUSG00000037907	Ankrd13b	44,82464824	148,9153234	132,9697726	176,5626508	58,71372284	0,389405474	False	0,389405474
ENSMUSG00000021494	Ddx41	330,7749905	680,2149094	433,2525043	1145,970326	902,8865824	0,38935793	False	0,38935793
ENSMUSG00000005161	Prdx2	1251,225957	513,6316663	1638,786403	500,4482779	485,3667755	0,389285481	False	0,389285481
ENSMUSG00000021111	Papola	1251,998796	812,7243074	860,3408469	955,9124409	266,1688769	-0,389282792	False	0,389282792
ENSMUSG00000016933	Plcg1	609,7697839	429,0780505	467,5956906	798,4680388	349,6728383	0,388969999	False	0,388969999
ENSMUSG00000038502	Ptov1	416,5600932	554,0154828	694,789077	873,8164312	545,3852477	0,388751043	False	0,388751043
ENSMUSG00000002728	Naa20	181,6171093	410,1481365	237,7605206	242,9142203	639,3272043	0,388609062	False	0,388609062
ENSMUSG00000040112	Mrps35	344,6860882	194,347117	263,2977617	112,4602872	242,6833878	-0,388587814	False	0,388587814
ENSMUSG00000032035	Ets1	161,5233014	150,1773177	108,3131261	211,4253399	97,85620474	0,388405994	False	0,388405994
ENSMUSG00000024911	Fibp	265,0836956	450,531953	346,9542412	446,46734	506,2427658	0,388297465	False	0,388297465
ENSMUSG00000020412	Ascc2	436,653901	1105,506977	571,505844	1306,788537	703,2599247	0,388278022	False	0,388278022
ENSMUSG00000041654	Slc39a11	249,6269204	121,1514495	326,7005672	116,9586986	122,6464433	0,388195433	False	0,388195433
ENSMUSG00000043284	Tmem11	307,5898276	417,7201021	256,2530056	235,0420002	1146,87472	-0,388089214	False	0,388089214
ENSMUSG00000039117	Taf4	197,8467233	321,8085379	258,8947891	285,6491294	349,6728383	0,387982756	False	0,387982756
ENSMUSG00000024082	Ndufaf7	190,1183356	121,1514495	145,2980959	79,84680388	74,3707156	-0,38788188	False	0,38788188
ENSMUSG00000032308	Ulk3	124,4270408	41,64581078	95,10420825	21,36745456	91,33245776	-0,387718965	False	0,387718965
ENSMUSG00000030591	Psmd8	1488,487457	2074,718574	1137,728121	1476,60357	3143,141296	-0,387691222	False	0,387691222
ENSMUSG00000029486	Mrpl1	392,6020915	204,4430711	198,1337672	137,2015503	300,0923612	-0,387661263	False	0,387661263
ENSMUSG00000039988	Ankrd13c	350,0959595	263,7568016	251,850033	146,1983733	457,9670382	0,387493353	False	0,387493353
ENSMUSG00000038871	Bpgm	137,5652998	233,4689392	228,9545754	179,9364595	284,4353684	0,387370938	False	0,387370938
ENSMUSG00000031445	Proz	7580,002586	610,8052248	9914,61371	1485,600393	1009,876033	0,387358223	False	0,387358223
ENSMUSG00000036990	Otud4	292,1330523	223,3729851	341,6706741	334,0070529	377,0725756	-0,38717088	False	0,38717088
ENSMUSG00000027177	Hipk3	761,2461814	2314,497484	582,0729783	742,2378953	3255,349744	-0,387163041	False	0,387163041
ENSMUSG00000092274	Neat1	5904,488148	4948,279518	7721,052759	3825,898969	2840,439436	0,386985571	False	0,386985571
ENSMUSG00000042248	Cyp2c37	22258,52921	2202,179994	17021,89209	195,6808997	8013,770793	-0,386966855	False	0,386966855
ENSMUSG00000042429	Adora1	78,82955381	1491,677223	177,8800932	2036,655801	103,0752023	0,386888779	False	0,386888779
ENSMUSG00000046707	Csnk2a2	385,6465426	748,3625998	294,9991645	391,3617993	1046,409016	-0,386568309	False	0,386568309
ENSMUSG00000052040	Klf13	418,1057707	576,7313796	586,4759509	546,5569956	683,6886838	0,386503994	False	0,386503994
ENSMUSG00000033114	Slc35d2	602,0413962	217,0630138	460,5509344	154,0705934	71,76121681	-0,38650197	False	0,38650197
ENSMUSG00000034168	Irf2bpl	282,0861484	215,8010195	107,4325315	56,23014358	100,4657035	-0,386434145	False	0,386434145
ENSMUSG00000070493	Chchd2	2064,252335	1702,430265	2389,052935	1040,257656	2698,221752	0,386389583	False	0,386389583
ENSMUSG00000040550	Otud6b	354,7329921	199,395094	160,2682028	115,8340958	271,3878745	-0,386377252	False	0,386377252
ENSMUSG00000046782	Ttc6	26,27651794	88,33959863	34,34318631	345,2530816	126,5606915	0,386249789	False	0,386249789
ENSMUSG00000015937	H2afy	778,2486341	1427,315515	1017,086672	1174,085398	1428,700589	0,38613958	False	0,38613958
ENSMUSG00000003382	Etv3	155,3405913	209,4910482	118,8802603	312,6395983	273,9973733	-0,385925683	False	0,385925683
ENSMUSG00000022329	Stk3	204,8022721	365,9783372	156,7458247	232,7927944	370,5488286	-0,385804707	False	0,385804707
ENSMUSG00000032615	Nt5m	88,87645772	128,7234151	167,312959	130,4539331	116,1226963	0,385776753	False	0,385776753
ENSMUSG00000036561	Ppp6r2	459,8390639	330,6424977	287,9544083	352,0006988	327,4920985	-0,385550737	False	0,385550737
ENSMUSG00000022404	Slc25a17	492,2982919	336,9524691	376,8944549	349,7514931	164,398424	-0,385372165	False	0,385372165
ENSMUSG00000039100	March6	1584,319464	940,1857283	708,8785893	814,2124791	2069,332543	0,385302234	False	0,385302234
ENSMUSG00000045969	Ing1	146,0665262	117,3654668	111,8355041	179,9364595	177,4459179	-0,385247326	False	0,385247326
ENSMUSG00000025899	Alkbh8	122,8813633	87,07760436	89,82064113	118,0833015	160,4841758	0,385164923	False	0,385164923
ENSMUSG00000059022	Kcp	105,8789105	44,16979931	138,2533398	89,96822973	33,92348431	0,384899077	False	0,384899077
ENSMUSG00000048186	Bend7	98,92336164	80,76763303	51,95507673	83,2206125	129,1701903	0,384889993	False	0,384889993
ENSMUSG00000005373	Mlxipl	8617,152205	7862,224278	11249,595	5389,096961	4629,250859	0,384589992	False	0,384589992
ENSMUSG00000024754	Tmem2	91,96781277	200,6570883	70,44756167	253,0356461	130,4749397	-0,384579257	False	0,384579257
ENSMUSG00000029438	Bcl7a	153,7949138	111,0554954	200,7755508	109,0864785	53,49472526	0,384575805	False	0,384575805
ENSMUSG00000109865	Hspa14	136,792461	185,5131571	104,790748	155,1951963	313,1398552	-0,384477375	False	0,384477375
ENSMUSG00000039067	Psmd7	910,4040626	1159,772731	697,4308605	748,9855125	1453,490828	-0,384456792	False	0,384456792
ENSMUSG00000044788	Fads6	1589,729335	181,7271743	1217,862222	708,4998091	90,02770836	-0,384430226	False	0,384430226
ENSMUSG00000028029	Aimp1	248,0812429	493,4397581	251,850033	190,0578853	491,8905225	-0,384373776	False	0,384373776
ENSMUSG00000037916	Ndufv1	1160,803822	749,6245941	889,4004661	625,2791966	703,2599247	-0,384219109	False	0,384219109

ENSMUSG00000062981	Mrpl42	648,411722	316,7605608	846,2513345	191,1824882	301,3971106	0,384176032	False	0,384176032
ENSMUSG00000032468	Armc8	172,3430441	64,36170757	132,0891781	93,34203834	90,02770836	-0,383770798	False	0,383770798
ENSMUSG00000039509	Nup133	127,5183959	141,3433578	97,74599182	217,0483542	194,4076601	-0,383595937	False	0,383595937
ENSMUSG00000034738	Nostrin	272,8120833	355,8823831	152,3428521	246,2880289	284,4353684	0,383492973	False	0,383492973
ENSMUSG00000026662	Sephs1	370,9626061	418,9820964	284,4320302	350,8760959	550,6042453	-0,38318983	False	0,38318983
ENSMUSG00000034430	Zxdc	111,2887818	145,1293406	76,61172331	66,35156943	125,2559421	0,383031043	False	0,383031043
ENSMUSG00000060407	Cyp2a12	12154,43522	5525,010897	9321,973598	3471,649065	5297,28255	-0,382775526	False	0,382775526
ENSMUSG00000033352	Map2k4	439,745256	484,6057982	337,2677015	417,2276654	597,5752236	-0,382773849	False	0,382773849
ENSMUSG00000031875	Cmtm3	78,05671504	189,2991399	110,9549096	233,9173973	101,7704529	0,382724109	False	0,382724109
ENSMUSG00000024426	Atat1	513,1649385	142,6053521	393,6257508	309,2657897	99,16095413	-0,382597999	False	0,382597999
ENSMUSG00000029101	Rgs12	105,1060717	97,17355849	74,85053427	48,35792348	136,9986866	0,38231605	False	0,38231605
ENSMUSG00000027655	Dhx35	84,23942515	109,7935012	116,2384768	161,9428135	180,0554167	0,382225165	False	0,382225165
ENSMUSG00000028032	Papss1	170,0245278	201,9190826	212,2232795	130,4539331	379,6820744	-0,382202444	False	0,382202444
ENSMUSG00000009470	Tnpo1	687,8264989	570,4214083	300,2827316	345,2530816	896,3628354	0,382038136	False	0,382038136
ENSMUSG00000026174	Cnot9	221,0318862	143,8673463	122,4026384	86,59442111	169,6174215	-0,381970151	False	0,381970151
ENSMUSG00000014353	Tmem87b	279,7676321	1644,378529	364,5661316	1656,54003	1979,304835	0,381951482	False	0,381951482
ENSMUSG00000022843	Clcn2	538,6686177	113,579484	701,8338331	178,8118566	146,1319324	0,381731479	False	0,381731479
ENSMUSG00000030409	Dmpk	497,7081632	667,5949668	1087,534233	1231,440144	648,4604501	0,381718537	False	0,381718537
ENSMUSG00000071415	Rpl23	2664,748054	4692,094681	3869,332325	2045,652623	3950,781173	-0,381437957	False	0,381437957
ENSMUSG00000044442	N6amt1	125,9727183	304,1406181	140,8951233	96,71584696	185,2744143	-0,381287125	False	0,381287125
ENSMUSG00000046352	Gjb2	4119,230606	3332,926857	4083,316793	1540,705934	5365,129518	0,381238105	False	0,381238105
ENSMUSG00000061374	Fiz1	251,1725979	283,9487099	192,8502001	271,0292921	371,853578	-0,381198437	False	0,381198437
ENSMUSG00000052296	Ppp6r1	1524,810879	1702,430265	849,7737126	1175,210001	1985,828581	0,381110776	False	0,381110776
ENSMUSG00000039701	Usp53	106,6517493	271,3287672	81,89529044	186,6840767	140,9129348	-0,381055234	False	0,381055234
ENSMUSG00000028416	Bag1	1004,690392	675,1669324	767,8784222	771,4775699	708,4789223	-0,381054867	False	0,381054867
ENSMUSG00000038685	Rtel1	162,2961402	296,5686525	211,342685	459,9625745	263,5593781	0,380955489	False	0,380955489
ENSMUSG00000061458	Nol10	59,50858474	205,7050654	77,49231784	174,3134451	379,6820744	0,380955489	False	0,380955489
ENSMUSG00000022952	Runx1	10,81974268	1362,953807	14,08951233	230,5435887	1983,219083	0,380955489	False	0,380955489
ENSMUSG00000026849	Tor1a	462,9304189	387,4322397	376,8944549	304,7673782	602,7942212	0,380870222	False	0,380870222
ENSMUSG00000026816	Gtf3c5	145,2936874	109,7935012	129,4473946	121,4571101	189,1886625	0,380853612	False	0,380853612
ENSMUSG00000022679	Mpv17l	244,217049	22,71589679	187,5666329	62,97776081	56,10422405	-0,380760717	False	0,380760717
ENSMUSG00000031985	Gnpat	625,2265591	813,9863017	354,8795919	590,4165076	495,8047707	0,380625452	False	0,380625452
ENSMUSG00000003402	Prkcsh	1127,571755	1033,573304	994,191214	796,2188331	1467,843071	0,380478491	False	0,380478491
ENSMUSG00000027580	Helz2	2145,400406	1648,164512	3262,6027	3936,110051	2027,580562	-0,380386677	False	0,380386677
ENSMUSG00000029467	Atp2a2	2697,980121	3855,392483	2072,919502	2165,985131	4916,295726	-0,380215625	False	0,380215625
ENSMUSG00000019768	Esr1	638,3648181	180,4651801	490,4911481	176,5626508	118,7321951	-0,380154043	False	0,380154043
ENSMUSG00000022544	Eef2kmt	120,562847	334,4284805	338,148296	537,5601726	92,63720715	-0,380121726	False	0,380121726
ENSMUSG00000052423	B4galt3	163,068979	273,8527558	212,2232795	414,9784596	330,1015973	0,380100559	False	0,380100559
ENSMUSG00000070305	Mpzl3	78,82955381	60,57572477	79,25350688	146,1983733	105,6847011	-0,379996847	False	0,379996847
ENSMUSG00000030751	Psma1	1187,08034	844,274164	912,2959236	468,9593975	808,9446258	-0,379843802	False	0,379843802
ENSMUSG00000069601	Ank3	250,3997591	47,95578211	325,8199727	203,5531198	39,1424819	0,379841869	False	0,379841869
ENSMUSG00000040170	Fmo2	1123,707561	131,2474037	678,9383756	863,6950054	88,72295896	-0,379672779	False	0,379672779
ENSMUSG00000028741	Mrto4	191,6640132	233,4689392	296,7603535	147,3229762	369,2440792	-0,379597034	False	0,379597034
ENSMUSG00000038521	C1s1	6948,593316	1110,554954	5342,566958	4988,738339	1371,291616	-0,379187862	False	0,379187862
ENSMUSG00000067274	Rplp0	5263,804813	7929,109974	7588,082986	4047,445735	7919,828837	-0,379094256	False	0,379094256
ENSMUSG00000016024	Lbp	1663,921856	2602,232177	2598,634431	2163,735925	3319,282465	0,378936754	False	0,378936754
ENSMUSG00000021302	Ggps1	154,5677526	199,395094	118,8802603	179,9364595	276,6068721	-0,378730181	False	0,378730181
ENSMUSG00000037204	Atg101	306,8169888	180,4651801	235,9993316	150,6967848	215,2836504	-0,378595595	False	0,378595595
ENSMUSG00000046794	Ppp1r3b	3459,226302	194,347117	2661,156642	68,60077517	227,026395	-0,378395964	False	0,378395964
ENSMUSG00000034349	Smc4	121,3356858	431,602039	93,34301921	517,3173209	532,3377538	-0,378389883	False	0,378389883
ENSMUSG00000054545	Ugt1a6a	240,3528552	13,88193693	184,9248494	23,6166603	2,609498793	-0,378214841	False	0,378214841
ENSMUSG00000035184	Fam124a	119,0171695	143,8673463	91,58183017	313,7642012	180,0554167	-0,378036411	False	0,378036411
ENSMUSG00000032285	Dnaja4	93,5134903	61,83771904	121,5220439	88,84362686	88,72295896	0,377971631	False	0,377971631
ENSMUSG00000049091	Sephs2	9393,855162	1944,733164	7228,800422	1431,619456	1662,250731	-0,37796109	False	0,37796109
ENSMUSG00000020102	Slc16a7	1180,89763	230,9449507	908,7735455	280,026115	540,1662502	-0,377891161	False	0,377891161
ENSMUSG00000069835	Sat2	176,2072379	95,91156423	228,9545754	112,4602872	37,8377325	0,37778821	False	0,37778821
ENSMUSG00000042460	C1galt1	702,5104354	465,6758842	540,6850358	310,3903926	328,7968479	-0,377731227	False	0,377731227
ENSMUSG00000027312	Atrn	1002,371875	574,2073911	452,6255837	771,4775699	519,2902598	-0,377721724	False	0,377721724
ENSMUSG00000073424	Cyp4f15	2423,62236	325,5945207	3148,125412	1998,419303	123,9511927	0,377328094	False	0,377328094
ENSMUSG00000021693	Kif2a	114,3801369	175,417203	88,05945209	190,0578853	172,2269203	-0,377286765	False	0,377286765
ENSMUSG00000027650	Tti1	228,7602738	334,4284805	253,611222	310,3903926	176,1411685	-0,377104395	False	0,377104395
ENSMUSG00000037933	Bicd2	326,1379579	108,5315069	423,5659645	114,7094929	86,11346017	0,377104306	False	0,377104306
ENSMUSG00000034602	Mon2	874,8534795	1063,861166	673,6548085	1794,866183	1834,477651	-0,377031894	False	0,377031894
ENSMUSG00000008090	Fgfrl1	391,8292527	330,6424977	224,5516028	195,6808997	508,8522646	0,37702175	False	0,37702175
ENSMUSG00000021959	Lats2	88,10361896	94,64956996	100,3877754	116,9586986	67,84696862	-0,376916923	False	0,376916923
ENSMUSG00000033845	Mrpl15	1037,14962	270,066773	798,6992304	296,8951581	287,0448672	-0,376899804	False	0,376899804
ENSMUSG00000001156	Mxd1	83,46658638	80,76763303	64,28340002	78,72220101	254,4261323	-0,376752532	False	0,376752532
ENSMUSG00000079316	Rab9	942,8632906	1224,134438	561,8193043	615,1577708	1166,44596	0,376641499	False	0,376641499
ENSMUSG00000033781	Asb13	775,9301179	257,4468303	1007,400132	610,6593593	105,6847011	0,376638194	False	0,376638194
ENSMUSG00000020122	Egfr	3067,39705	3255,945207	2362,635099	3237,731667	4049,942127	-0,376618096	False	0,376618096
ENSMUSG00000031375	Bgn	2322,380482	1234,230392	1425,682529	1476,60357	1788,811423	-0,376603047	False	0,376603047
ENSMUSG00000041975	Mettl8	164,6146565	2,523988532	126,805611	84,34521537	3,91424819	-0,376474207	False	0,376474207
ENSMUSG00000020641	Rsad2	163,8418177	88,33959863	52,83567125	79,84680388	212,6741516	0,376341073	False	0,376341073
ENSMUSG00000045268	Zfp691	115,1529757	118,627461	214,8650631	157,444402	88,72295896	-0,376172306	False	0,376172306
ENSMUSG00000030714	Sgf29	283,6318259	340,7384519	368,0885097	192,307091	146,1319324	0,376033291	False	0,376033291
ENSMUSG00000035125	Gcfc2	83,46658638	234,7309335	108,3131261	452,0903544	357,5013346	0,375937415	False	0,375937415
ENSMUSG00000090000	Ier3ip1	121,3356858	217,0630138	213,103874	157,444402	324,8825997	0,375838542	False	0,375838542
ENSMUSG00000031532	Saraf	1342,420931	935,1377512	785,4903126	394,7356079	1741,840444	0,375775361	False	0,375775361
ENSMUSG00000003868	Ruvbl2	128,2912346	172,8932145	166,4323644	176,5626508	242,6833878	0,375513404	False	0,375513404
ENSMUSG00000041629	Fam104a	285,9503422	695,3588406	248,3276549	220,4221628	840,2586114	-0,375495342	False	0,375495342
ENSMUSG00000002608	Ccdc97	357,0515084	1338,975916	463,192718	684,8831488	2224,597721	0,375480359	False	0,375480359
ENSMUSG00000031684	Slc10a7	99,6962004	83,29162157	110,9549096	129,3293302	39,1424819	0,37543907	False	0,37543907
ENSMUSG00000051579	Tceal8	170,7973666	492,1777638	329,3423508	221,5467657	772,4116427	0,375325534	False	0,375325534
ENSMUSG00000014956	Ppp1cb	864,8065756	331,904492	300,2827316	238,4158088	666,7269416	-0,375281467	False	0,375281467
ENSMUSG00000032109	Nlrx1	129,0640734	109,7935012	99,50718086	197,9301054	112,2084481	-0,375214919	False	0,375214919
ENSMUSG00000032525	Nktr	938,9990968	1414,695572	1217,862222	2632,695322	4573,146635	0,375155254	False	0,375155254
ENSMUSG00000061859	Patj	95,05916782	179,2031858	123,2832329	218,1729571	136,9986866	0,375078923	False	0,375078923
ENSMUSG00000073725	Lmbrd1	733,4239859	638,5690987	519,5507673	596,039522	951,1623101	0,3750441	False	0,3750441
ENSMUSG00000007603	Dus3l	184,7084643	873,3000322	239,5217097	394,7356079	1957,124095	0,374906445	False	0,374906445
ENSMUSG00000020088	Sar1a	789,8412156	762,2445367	576,7894112	617,4069765	1024,228276	0,374902736	False	0,374902736
ENSMUSG00000020741	Cluh	4060,49486	2684,261804	3540,870568	5265,390645	3417,138669	0,374885008	False	0,374885008
ENSMUSG00000025423	Pias2	327,6836354	484,6057982	252,7306275	320,5118184	588,4419778	-0,374703117	False	0,374703117
ENSMUSG00000039254	Pomt1	285,9503422	85,8156101	370,7302933	178,8118566	44,36147948	0,374605374	False	0,374605374
ENSMUSG00000020225	Tmbim4	552,5797154	537,6095574	495,7747152	307,016584	716,3074187	0,374396281	False	0,374396281
ENSMUSG00000032727	Mier3	146,0665262	422,7680792	189,327822	377,8665649	568,8707369	0,374260837	False	0,374260837
ENSMUSG00000026348	Acmsd	303,7256338	18,92991399	393,6257508	46,10871774	74,3707156	0,37405593	False	0,37405593
ENSMUSG00000031545	Gpat4	1144,574208	946,4956996	848,0125236	1483,351188	978,5620474	0,374049198	False	0,374049198
ENSMUSG00000025337	Sbds	548,7155216	492,1777638	368,9691042	192,307091	711,0884211	0,373970578	False	0,373970578
ENSMUSG00000020782	Llgl2	585,0389434	641,0930872	512,5060111	757,9823355	272,6926239	0,373631566	False	0,373631566
ENSMUSG00000010307	Tmem86a	88,87645772	174,1552087	103,0295589	68,60077517	153,9604288	-0,373576439	False	0,373576439
ENSMUSG00000041236	Vps41	520,8933261	395,0042053	386,5809947	674,761723	302,70186	0,373390184	False	0,373390184
ENSMUSG00000011837	Snapc2	164,6146565	220,8489966	174,3577151	127,0801245	724,1359151	-0,373354383	False	0,373354383
ENSMUSG00000026790	Odf2	146,8393649	781,1744507	190,2084165	495,9498664	745,0119054	0,373342305	False	0,373342305
ENSMUSG00000021606	Ndufs6	1092,021172	1482,843263	1703,069803	1984,924068	1414,348346	0,373136664	False	0,373136664
ENSMUSG00000022887	Masp1	2942,19717	8195,390764	2271,933864	5101,198626	8911,438378	-0,372973093	False	0,372973093
ENSMUSG00000020925	Ccdc43	136,0196223	137,557375	127,6862055	94,46664122	176,1411685	0,372917352	False	0,372917352
ENSMUSG00000024610	Cd74	500,7995183	203,1810768	353,9989974	240,6650145	648,4604501	0,372785412	False	0,372785412
ENSMUSG00000061244	Exoc5	249,6269204	615,8532019	323,1781892	569,049053	1064,675508	0,37255631	False	0,37255631
ENSMUSG00000017466	Timp2	249,6269204	82,0296273	192,8502001	112,4602872	97,85620474	-0,372292884	False	0,372292884
ENSMUSG00000021713	Ppwd1	115,9258144	97,17355849	61,64161646	76,47299527	150,0461806	0,372204734	False	0,372204734
ENSMUSG00000017713	Tha1	69,55548865	185,5131571	186,6860384	228,2943829	90,02770836	0,372204734	False	0,372204734
ENSMUSG00000029625	Cpsf4	216,3948536	450,531953	280,0290576	292,3967466	498,4142695	0,37191035	False	0,37191035
ENSMUSG00000041390	Mdfic	244,9898878	283,9487099	189,327822	329,5086414	357,5013346	-0,371835769	False	0,371835769

ENSMUSG00000039298	Cdk5rap2	88,87645772	271,3287672	68,68637263	132,7031389	237,4643902	-0,371777421	False	0,371777421
ENSMUSG00000020235	Fzr1	182,389948	641,0930872	235,9993316	542,0585841	536,252002	0,371756552	False	0,371756552
ENSMUSG00000022884	Eif4a2	2728,120833	1134,532845	1637,905809	503,8220865	2108,475025	-0,371707612	False	0,371707612
ENSMUSG00000029098	Acox3	452,883515	324,3325264	339,0288905	585,9180961	307,9208576	0,371558982	False	0,371558982
ENSMUSG00000002205	Vrk3	261,9923406	413,9341193	202,5367398	353,1253017	296,178113	-0,371341001	False	0,371341001
ENSMUSG00000075227	Znhit2	155,3405913	267,5427844	357,5213755	231,6681916	200,9314071	0,371268223	False	0,371268223
ENSMUSG00000022346	Myc	83,46658638	235,9929278	123,2832329	107,9618757	304,0066094	0,371251273	False	0,371251273
ENSMUSG00000041477	Dcp1b	69,55548865	112,3174897	107,4325315	89,96822973	116,1226963	0,371251273	False	0,371251273
ENSMUSG00000050240	Hic2	64,91845608	188,0371457	50,19388769	161,9428135	349,6728383	-0,371116997	False	0,371116997
ENSMUSG00000041977	Arhgef11	487,6612593	627,2111503	527,476118	627,5284024	377,0725756	-0,371037139	False	0,371037139
ENSMUSG00000028218	Fam92a	184,7084643	377,3362856	180,5218768	142,8245647	531,0330044	-0,371005847	False	0,371005847
ENSMUSG00000115987	Vps28	689,3721764	628,4731445	1263,653137	533,0617611	541,4709996	-0,370980377	False	0,370980377
ENSMUSG00000041736	Tspo	424,2884808	328,1185092	663,9682687	347,5022873	365,329831	-0,370828532	False	0,370828532
ENSMUSG00000040433	Zbtb38	249,6269204	193,0851227	117,1190713	159,6936078	144,827183	-0,370536518	False	0,370536518
ENSMUSG00000020056	Washc3	205,5751109	159,0112775	141,7757179	77,59759814	56,10422405	-0,370536518	False	0,370536518
ENSMUSG00000028688	Toe1	80,37523133	191,8231285	103,9101535	202,4285169	276,6068721	0,370513743	False	0,370513743
ENSMUSG00000071477	Zfp777	132,1554284	1220,348455	170,835337	1064,998919	883,3153414	0,370370739	False	0,370370739
ENSMUSG00000032253	Phip	183,9356255	556,5394714	237,7605206	807,4646618	562,3469899	0,370308245	False	0,370308245
ENSMUSG00000029003	Mad2l2	441,2909336	570,4214083	385,7004001	439,7197228	354,8918359	0,370298021	False	0,370298021
ENSMUSG00000025969	Nrp2	416,5600932	665,0709783	326,7005672	693,8799718	322,2731009	-0,370240858	False	0,370240858
ENSMUSG00000029710	Ephb4	230,3059513	82,0296273	297,640948	210,300737	74,3707156	0,370021327	False	0,370021327
ENSMUSG00000040687	Madd	226,4417575	64,36170757	175,2383097	64,10236368	40,44723129	-0,369821823	False	0,369821823
ENSMUSG00000019804	Snx3	1041,013813	599,4472764	805,7439866	239,5404117	777,6306403	-0,369595791	False	0,369595791
ENSMUSG00000090523	Gypc	262,7651794	156,487289	203,4173343	43,85951199	168,3126721	-0,369331483	False	0,369331483
ENSMUSG00000036885	Arhgef26	342,3675719	873,3000322	265,0589508	608,4101535	1516,118799	-0,3692328	False	0,3692328
ENSMUSG00000025785	Exosc7	190,8911744	140,0813635	246,5664658	94,46664122	159,1794264	0,369226197	False	0,369226197
ENSMUSG00000004085	Map3k20	128,2912346	82,0296273	72,20875071	64,10236368	165,7031734	0,369178629	False	0,369178629
ENSMUSG00000054733	Msra	2595,192565	1273,352215	1386,055776	473,457809	2009,314071	-0,369138506	False	0,369138506
ENSMUSG00000064210	Ano6	141,4294936	301,6166296	183,1636603	609,5347564	182,6649155	0,369116552	False	0,369116552
ENSMUSG00000020629	Adi1	2240,459573	270,066773	1734,771206	352,0006988	317,0541034	-0,369049292	False	0,369049292
ENSMUSG00000109901	Chmp1b	414,2415769	310,4505895	182,2830658	150,6967848	534,9472526	0,368924282	False	0,368924282
ENSMUSG00000039637	Coro7	330,7749905	530,0375918	427,0883426	1256,181408	638,0224549	0,368684362	False	0,368684362
ENSMUSG00000029189	Sel1l3	149,93072	198,1330998	255,372411	763,6053498	116,1226963	-0,368646038	False	0,368646038
ENSMUSG00000032046	Abhd12	492,2982919	166,5832431	381,2974275	169,8150336	220,502648	-0,368615936	False	0,368615936
ENSMUSG00000026519	Tmem63a	215,6220148	184,2511629	191,089011	575,7966703	167,0079228	-0,368587939	False	0,368587939
ENSMUSG00000027667	Zfp639	180,0714317	278,9007328	232,4769535	456,5887659	253,1213829	0,368518386	False	0,368518386
ENSMUSG00000025156	Gps1	452,883515	350,834406	322,2975946	209,1761341	347,0633395	-0,368349788	False	0,368349788
ENSMUSG00000021037	Ahsa1	799,1152807	358,4063716	619,0579482	97,84044983	497,1095201	-0,36832918	False	0,36832918
ENSMUSG00000032238	Rora	717,1943719	856,8941067	555,6551427	765,8545556	773,7163921	-0,368174389	False	0,368174389
ENSMUSG00000017754	Pltp	3806,230907	2949,2806	2831,111385	1856,719341	2576,880058	-0,367999996	False	0,367999996
ENSMUSG00000027940	Tpm3	862,4880593	710,5027718	618,1773536	701,7521919	1112,951235	0,367813993	False	0,367813993
ENSMUSG00000020375	Rufy1	360,1428635	230,9449507	279,1484631	257,5340576	185,2744143	-0,367536703	False	0,367536703
ENSMUSG00000074238	Ap1ar	307,5898276	456,8419243	240,4023042	238,4158088	301,3971106	-0,367527892	False	0,367527892
ENSMUSG00000030067	Foxp1	258,9009855	492,1777638	453,5061782	1022,26401	334,0158455	0,367516088	False	0,367516088
ENSMUSG00000036565	Ttyh3	122,1085245	94,64956996	129,4473946	151,8213877	125,2559421	-0,367496064	False	0,367496064
ENSMUSG00000045103	Dmd	319,182409	177,9411915	247,4470604	214,7991485	165,7031734	-0,36726124	False	0,36726124
ENSMUSG00000022024	Sugt1	376,3724775	455,5799301	308,2080823	167,5658279	485,3667755	0,366914222	False	0,366914222
ENSMUSG00000033910	Gucy1a1	117,4714919	56,78974198	73,08934523	91,0928326	75,675465	-0,366901236	False	0,366901236
ENSMUSG00000024160	Spsb3	153,7949138	320,5465436	332,8647289	399,2340194	198,3219083	0,366836266	False	0,366836266
ENSMUSG00000041278	Ttc1	187,7998194	521,2036319	242,1634932	336,2562586	734,5739102	0,366785716	False	0,366785716
ENSMUSG00000006699	Cdc42	2087,437498	1633,02058	1271,578488	831,0815221	2691,698005	0,366783555	False	0,366783555
ENSMUSG00000057134	Ado	196,3010458	241,0409048	180,5218768	85,46981824	253,1213829	0,366761529	False	0,366761529
ENSMUSG00000031568	Rwdd4a	175,4343992	325,5945207	176,1189042	136,0769475	388,8153202	-0,366508976	False	0,366508976
ENSMUSG00000022610	Mapk12	57,19006845	228,4209622	192,8502001	504,9466894	44,36147948	-0,366457151	False	0,366457151
ENSMUSG00000015790	Surf1	382,5551876	161,5352661	296,7603535	121,4571101	150,0461806	-0,366369522	False	0,366369522
ENSMUSG00000028669	Pithd1	221,8047249	148,9153234	98,62658634	172,0642394	135,6939372	-0,36634281	False	0,36634281
ENSMUSG00000078427	Sarnp	258,1281468	156,487289	143,5369069	129,3293302	332,7110961	0,366182518	False	0,366182518
ENSMUSG00000038102	Trappc11	360,9157022	670,1189553	280,0290576	467,8347946	1182,102953	-0,366085373	False	0,366085373
ENSMUSG00000034152	Exoc3	411,9230606	261,2328131	319,6558111	268,7800863	307,9208576	-0,365855573	False	0,365855573
ENSMUSG00000031360	Ctps2	129,8369122	263,7568016	167,312959	366,6205361	304,0066094	0,365848597	False	0,365848597
ENSMUSG00000020496	Rnf187	1180,124791	410,1481365	915,8183017	477,9562204	499,7190189	-0,365806122	False	0,365806122
ENSMUSG00000020477	Mrps24	507,7550672	352,0964003	654,281729	258,6586605	430,5673008	0,365779252	False	0,365779252
ENSMUSG00000034501	Pcnx4	81,14807009	102,2215356	101,2683699	62,97776081	114,8179469	-0,365714321	False	0,365714321
ENSMUSG00000000301	Pemt	4861,155818	2794,055305	6263,668827	1550,82736	1444,357582	0,365708558	False	0,365708558
ENSMUSG00000028426	Rad23b	3232,784545	1201,418541	1465,309283	818,7108905	2509,033089	-0,365645862	False	0,365645862
ENSMUSG00000022354	Ndufb9	1560,361462	1448,769418	2010,397291	1354,021857	1209,502691	0,36560036	False	0,36560036
ENSMUSG00000038520	Tbc1d17	530,94023	624,6871617	412,1182358	932,2957806	675,8601874	-0,365491158	False	0,365491158
ENSMUSG00000032485	Scap	1848,630321	827,8682386	1069,922343	1434,993264	884,6200908	-0,365412786	False	0,365412786
ENSMUSG00000039262	Prrc2b	682,4166276	784,9604335	618,1773536	1136,973503	529,728255	-0,365400337	False	0,365400337
ENSMUSG00000051147	Nat2	193,9825295	249,8748647	148,820474	136,0769475	88,72295896	0,36527906	False	0,36527906
ENSMUSG00000038855	Itpkb	142,2023324	129,9854094	183,1636603	104,5880671	126,5606915	0,365188173	False	0,365188173
ENSMUSG00000001467	Cyp51	3565,105213	815,2482959	4591,419832	811,9632733	377,0725756	0,364995702	False	0,364995702
ENSMUSG00000008859	Rala	452,883515	583,041351	483,446392	224,9205743	380,9868238	0,364458181	False	0,364458181
ENSMUSG00000050002	Idnk	411,1502218	275,11475	529,237307	272,1538949	211,3694022	0,364249157	False	0,364249157
ENSMUSG00000063406	Tmed5	905,76703	9977,326668	1165,907146	4614,245582	13692,04017	0,364240964	False	0,364240964
ENSMUSG00000021178	Psmc1	1219,539568	1461,38936	723,8486961	715,2474263	1569,613524	0,36407281	False	0,36407281
ENSMUSG00000025730	Rab40c	146,0665262	218,325008	196,3725782	344,1284787	113,5131975	-0,363765555	False	0,363765555
ENSMUSG00000036282	Naa30	146,0665262	98,43555276	107,4325315	71,97458378	113,5131975	-0,363765555	False	0,363765555
ENSMUSG00000031986	Sprtn	89,64929649	215,8010195	115,3578822	143,9491676	349,6728383	0,363752418	False	0,363752418
ENSMUSG00000001018	Snapin	283,6318259	307,9266009	450,8643947	220,4221628	233,550142	-0,363750134	False	0,363750134
ENSMUSG00000058558	Rpl5	2017,88201	1553,514942	1568,338842	970,5322782	1362,15837	-0,36360453	False	0,36360453
ENSMUSG00000027620	Rbm39	1730,38599	8298,874294	2226,142949	10633,12015	13300,61535	0,363452347	False	0,363452347
ENSMUSG00000074622	Mafb	404,1946729	314,2365723	164,6711754	173,1888422	180,0554167	-0,36319921	False	0,36319921
ENSMUSG00000019055	Plod1	677,779595	310,4505895	871,7885756	510,5697037	276,6068721	0,363162094	False	0,363162094
ENSMUSG00000032898	Fbxo21	936,6805805	119,8894553	728,2516687	371,1189476	241,3786384	-0,363120053	False	0,363120053
ENSMUSG00000026669	Mcm10	262,7651794	464,4138899	204,2979288	516,1927181	460,576537	-0,36309953	False	0,36309953
ENSMUSG00000008305	Tle1	540,2142952	1100,459	420,0435864	591,5411105	3705,488286	-0,362992778	False	0,362992778
ENSMUSG00000023072	Cep89	78,05671504	108,5315069	100,3877754	201,303914	165,7031734	0,362988943	False	0,362988943
ENSMUSG00000028668	Eloa	550,2611991	280,1627271	427,9689371	237,2912059	366,6345804	-0,362610516	False	0,362610516
ENSMUSG00000030733	Sh2b1	662,3228197	1291,020134	851,5349017	1316,909963	2721,707241	0,362531097	False	0,362531097
ENSMUSG00000000049	Apoh	23189,79992	8870,557697	18040,73995	6656,524397	11981,51371	-0,36223186	False	0,36223186
ENSMUSG00000075324	Fign	119,7900082	267,5427844	185,8054439	229,4189858	153,9604288	0,362052017	False	0,362052017
ENSMUSG00000039850	Endov	88,87645772	112,3174897	175,2383097	284,5245265	69,15171802	-0,362036223	False	0,362036223
ENSMUSG00000042406	Atf4	2215,728733	3520,964003	2270,172675	1724,016202	7427,938314	-0,362007933	False	0,362007933
ENSMUSG00000070372	Capza1	171,5702053	113,579484	97,74599182	146,1983733	220,502648	0,361996943	False	0,361996943
ENSMUSG00000060477	Irak2	308,3626664	682,738898	396,2675344	416,1030625	583,2229802	0,361846666	False	0,361846666
ENSMUSG00000054770	Kctd18	102,7875555	145,1293406	132,0891781	255,2848519	202,2361565	0,361846666	False	0,361846666
ENSMUSG00000032086	Bace1	396,4662853	135,0333865	367,2079152	509,4451008	24,79023853	0,361728499	False	0,361728499
ENSMUSG00000022528	Hes1	284,4046647	501,0117237	365,4467262	521,8157324	571,4802357	0,361715982	False	0,361715982
ENSMUSG00000024725	Ostf1	435,8810622	232,206945	292,3573809	163,0674164	339,2348431	-0,361650166	False	0,361650166
ENSMUSG00000033147	Slc22a15	199,3924008	256,184836	169,9547425	235,0420002	135,6939372	0,361574656	False	0,361574656
ENSMUSG00000045176	Borcs6	99,6962004	99,69754702	122,4026384	77,59759814	112,2084481	-0,361526524	False	0,361526524
ENSMUSG00000022181	C6	258,1281468	171,6312202	94,22361373	149,5721819	200,9314071	-0,361384379	False	0,361384379
ENSMUSG00000003847	Nfat5	770,5202465	508,5836893	623,4609208	989,650527	460,576537	0,361086293	False	0,361086293
ENSMUSG00000064145	Arih2	417,3329319	540,1335459	324,9393782	464,460986	546,6899971	-0,36102818	False	0,36102818
ENSMUSG00000074476	Spc24	139,1109773	11,3579484	108,3131261	4,498411486	28,70448672	-0,36102818	False	0,36102818
ENSMUSG00000019880	Rspo3	91,19497401	161,5352661	117,1190713	398,1094166	165,7031734	0,360949797	False	0,360949797
ENSMUSG00000022141	Nipbl	539,4414564	700,4068177	420,0435864	915,4267375	895,058086	-0,360927359	False	0,360927359
ENSMUSG00000020903	Stx8	180,8442705	220,8489966	170,835337	77,59759814	232,2453926	0,360902085	False	0,360902085
ENSMUSG00000025200	Cwf19l1	336,9577006	164,0592546	262,4171672	166,441225	150,0461806	-0,360705393	False	0,360705393
ENSMUSG00000057531	Dtnbp1	351,6416371	821,5582673	273,864896	391,3617993	730,659662	-0,360641554	False	0,360641554
ENSMUSG00000029669	Tspan12	1614,460175	805,1523418	1257,488976	1124,602872	895,058086	-0,360506102	False	0,360506102
ENSMUSG00000065990	Aurkaip1	799,8881195	1245,588341	1204,653305	623,0299909	1062,066009	-0,360496612	False	0,360496612

ENSMUSG00000001128	Cfp	427,3798358	855,6321124	548,6103865	954,787838	1030,752023	0,360263094	False	0,360263094
ENSMUSG00000024309	Pfdn6	349,3231208	653,7130299	723,8486961	272,1538949	439,7005466	-0,360139451	False	0,360139451
ENSMUSG00000030228	Pik3c2g	70,32832742	126,1994266	99,50718086	248,5372346	54,79947465	-0,359943844	False	0,359943844
ENSMUSG00000040462	Os9	4227,428033	3231,967316	5425,342843	2948,708729	4506,604416	0,359934114	False	0,359934114
ENSMUSG00000045980	Tmem104	171,5702053	109,7935012	220,1486302	84,34521537	82,19921198	0,35967883	False	0,35967883
ENSMUSG00000037926	Ssh2	257,355308	812,7243074	330,2229453	963,784661	528,4235056	0,35967883	False	0,35967883
ENSMUSG00000027852	Nras	257,355308	787,4844221	330,2229453	624,1545937	889,8390884	0,35967883	False	0,35967883
ENSMUSG00000019082	Slc25a22	2184,042344	7966,969802	2802,051765	9868,390198	7645,831464	0,359482781	False	0,359482781
ENSMUSG00000055053	Nfic	1865,632773	873,3000322	2393,455908	1275,299656	498,4142695	0,359430191	False	0,359430191
ENSMUSG00000000184	Ccnd2	135,2467835	59,31373051	173,4771206	116,9586986	39,1424819	0,359151119	False	0,359151119
ENSMUSG00000037013	Ss18	425,0613195	206,9670596	259,7753837	305,8919811	331,4063467	-0,359069748	False	0,359069748
ENSMUSG00000004591	Pkn2	523,9846812	437,9120103	285,3126248	364,3713304	671,9459392	0,358820532	False	0,358820532
ENSMUSG00000053253	Ndfip2	324,5922804	132,5093979	172,5965261	109,0864785	253,1213829	-0,358799302	False	0,358799302
ENSMUSG00000024014	Pim1	234,1701451	591,8753108	300,2827316	439,7197228	808,9446258	0,358764357	False	0,358764357
ENSMUSG00000040188	Scamp2	266,6293732	477,0338326	343,4318631	341,879273	698,0409271	0,358651236	False	0,358651236
ENSMUSG00000038007	Acer2	122,1085245	526,251609	188,4472275	675,8863258	156,5699276	0,358643221	False	0,358643221
ENSMUSG00000033096	Apmap	376,3724775	228,4209622	254,4918165	176,5626508	293,5686142	-0,358463398	False	0,358463398
ENSMUSG00000017309	Cd300lg	215,6220148	41,64581078	168,1935535	86,59442111	28,70448672	-0,358382072	False	0,358382072
ENSMUSG00000030403	Vasp	186,2541418	97,17355849	145,2980959	105,7126699	126,5606915	-0,358256711	False	0,358256711
ENSMUSG00000059772	Slx1b	148,3850425	47,95578211	190,2084165	62,97776081	10,43799517	0,358235413	False	0,358235413
ENSMUSG00000029314	Gpat3	49,46168082	152,7013062	63,4028055	143,9491676	220,502648	0,358235413	False	0,358235413
ENSMUSG00000038415	Foxq1	227,987435	151,4393119	177,8800932	177,6872537	80,89446258	-0,35804925	False	0,35804925
ENSMUSG00000029310	Nudt9	512,3920997	253,6608475	399,7899125	184,4348709	253,1213829	-0,358006161	False	0,358006161
ENSMUSG00000004264	Phb2	2636,925859	2372,54922	2413,709582	1503,594039	3379,300937	0,357867824	False	0,357867824
ENSMUSG00000032042	Srpr	2492,40501	2180,726092	1430,085502	1730,763819	3194,026523	0,357837771	False	0,357837771
ENSMUSG00000071644	Eef1g	1800,714317	1566,134884	3299,58767	1477,728173	1405,2151	-0,357778332	False	0,357778332
ENSMUSG00000032353	Tmed3	493,8439694	841,7501755	916,6988962	1170,711589	632,8034573	0,357702189	False	0,357702189
ENSMUSG00000003438	Timm50	297,5429237	232,206945	214,8650631	167,5658279	194,4076601	-0,357686686	False	0,357686686
ENSMUSG00000030946	Lhpp	808,3893459	1212,77649	2007,755508	630,902211	1077,723002	-0,357633901	False	0,357633901
ENSMUSG00000002409	Dyrk1b	286,723181	200,6570883	155,8652302	223,7959715	109,5989493	-0,357474482	False	0,357474482
ENSMUSG00000029430	Ran	826,9374762	521,2036319	369,8496988	228,2943829	1059,45651	0,357474209	False	0,357474209
ENSMUSG00000042688	Mapk6	377,918155	1153,462759	294,9991645	456,5887659	4082,560862	-0,357362958	False	0,357362958
ENSMUSG00000034037	Fgd5	71,87400494	112,3174897	98,62658634	199,0547083	56,10422405	-0,357360683	False	0,357360683
ENSMUSG00000020706	Ftsj3	398,0119628	786,2224278	509,8642276	1130,225886	763,278397	0,357301327	False	0,357301327
ENSMUSG00000041130	Zfp598	1038,695297	3886,94234	1330,578321	3125,27138	2338,110919	0,357280934	False	0,357280934
ENSMUSG00000055923	Aasdh	223,3504025	35,33583945	174,3577151	121,4571101	23,48548914	-0,357258651	False	0,357258651
ENSMUSG00000015971	Actr8	328,4564742	184,2511629	143,5369069	256,4094547	138,303436	-0,357252742	False	0,357252742
ENSMUSG00000038690	Atp5j2	1699,472439	1291,020134	2176,829656	500,4482779	1436,529086	0,35714155	False	0,35714155
ENSMUSG00000091337	Eid1	112,0616206	31,54985665	143,5369069	64,10236368	35,22823371	0,357129476	False	0,357129476
ENSMUSG00000033819	Ppp1r16a	316,091054	295,3066583	339,9094851	404,8570338	181,3601661	0,357072338	False	0,357072338
ENSMUSG00000039357	Fut11	89,64929649	119,8894553	257,1336001	166,441225	114,8179469	0,356983997	False	0,356983997
ENSMUSG00000024683	Mrpl16	425,0613195	331,904492	323,1781892	154,0705934	284,4353684	-0,356902825	False	0,356902825
ENSMUSG00000023079	Gtf2ird1	191,6640132	473,2478498	149,7010685	444,2181343	481,4525273	-0,356494963	False	0,356494963
ENSMUSG00000038446	Cdc40	268,9478895	114,8414782	103,9101535	143,9491676	210,0646528	-0,356493245	False	0,356493245
ENSMUSG00000029304	Spp1	588,1302985	459,3659129	1331,458916	548,8062014	533,6425032	-0,356492009	False	0,356492009
ENSMUSG00000029422	Rsrc2	481,4785492	376,0742913	494,8941207	628,6530052	457,9670382	-0,356453839	False	0,356453839
ENSMUSG00000028961	Pgd	695,5548865	1177,44065	543,3268194	718,621235	1304,749397	-0,356344101	False	0,356344101
ENSMUSG00000037112	Sik2	125,1998796	160,2732718	52,83567125	131,578536	147,4366818	0,356300678	False	0,356300678
ENSMUSG00000022089	Bin3	176,9800767	272,5907615	138,2533398	160,8182106	394,0343177	-0,356272628	False	0,356272628
ENSMUSG00000025280	Polr3a	232,6244676	348,3104175	297,640948	529,6879525	307,9208576	0,355570171	False	0,355570171
ENSMUSG00000018841	Rad51d	187,0269806	58,05173624	146,1786905	142,8245647	32,61873491	-0,355513395	False	0,355513395
ENSMUSG00000039958	Etfbkmt	166,9331728	619,6391847	243,9246823	547,6815985	130,4749397	-0,355497939	False	0,355497939
ENSMUSG00000048758	Rpl29	2786,856579	4944,493535	6518,160643	3564,991103	4586,194129	0,355259718	False	0,355259718
ENSMUSG00000004451	Ralb	145,2936874	127,4614209	117,1190713	113,58489	279,2163709	-0,355201095	False	0,355201095
ENSMUSG00000050567	Maml1	226,4417575	118,627461	110,0743151	124,8309187	289,654366	0,355192385	False	0,355192385
ENSMUSG00000024079	Eif2ak2	231,8516288	296,5686525	197,2531727	201,303914	178,7506673	0,35516425	False	0,35516425
ENSMUSG00000000290	Itgb2	105,8789105	189,2991399	82,77588496	177,6872537	400,5580647	-0,35513282	False	0,35513282
ENSMUSG00000064215	Ifi27	660,0043034	243,5648934	516,0283892	239,5404117	320,9683515	-0,355024994	False	0,355024994
ENSMUSG00000024427	Spry4	127,5183959	109,7935012	25,5372411	32,61348328	163,0936746	0,354995444	False	0,354995444
ENSMUSG00000028744	Pqlc2	178,5257542	299,0926411	265,0589508	312,6395983	228,3311444	0,354995444	False	0,354995444
ENSMUSG00000031504	Rab20	113,6072981	235,9929278	110,0743151	88,84362686	301,3971106	-0,354715322	False	0,354715322
ENSMUSG00000042675	Ypel3	569,5821682	271,3287672	728,2516687	695,0045747	200,9314071	0,354533121	False	0,354533121
ENSMUSG00000014859	E2f4	330,7749905	588,089328	426,2077481	422,8506797	751,5356524	0,35429814	False	0,35429814
ENSMUSG00000015143	Actn1	348,550282	714,2887546	382,1780221	562,3014358	272,6926239	-0,354091118	False	0,354091118
ENSMUSG00000032333	Stoml1	63,37277855	141,3433578	117,1190713	254,160249	49,58047707	-0,354091118	False	0,354091118
ENSMUSG00000003039	Fam32a	503,1180346	210,7530424	393,6257508	186,6840767	348,3680889	-0,354072301	False	0,354072301
ENSMUSG00000048109	Rbm15	91,96781277	275,11475	81,89529044	71,97458378	550,6042453	-0,353641489	False	0,353641489
ENSMUSG00000028245	Nsmaf	229,5331126	342,0004461	179,6412823	609,5347564	266,1688769	-0,353583367	False	0,353583367
ENSMUSG00000020257	Wdr82	394,9206078	249,8748647	504,5806605	385,738785	323,5778503	0,353522259	False	0,353522259
ENSMUSG00000020075	Ddx21	340,8218944	581,7793567	266,8201398	289,022938	1054,237512	-0,353150451	False	0,353150451
ENSMUSG00000070056	Mfhas1	351,6416371	121,1514495	235,1187371	124,8309187	275,3021227	-0,353090173	False	0,353090173
ENSMUSG00000000600	Krit1	261,9923406	653,7130299	334,6259179	574,6720674	751,5356524	0,353024557	False	0,353024557
ENSMUSG00000024942	Capn1	288,2688585	225,8969736	394,5063453	387,9879907	225,7216456	-0,352870219	False	0,352870219
ENSMUSG00000021893	Capn7	454,4291925	347,0484232	307,3274878	580,2950818	427,9578021	0,352731185	False	0,352731185
ENSMUSG00000043445	Pgp	214,8491761	220,8489966	339,0288905	250,7864404	168,3126721	-0,352180444	False	0,352180444
ENSMUSG00000026648	Dclre1c	88,10361896	64,36170757	75,73112879	112,4602872	99,16095413	0,35214245	False	0,35214245
ENSMUSG00000045896	Paip2b	161,5233014	136,2953807	58,11923838	49,48252635	206,1504046	0,351954989	False	0,351954989
ENSMUSG00000044033	Ccdc141	315,3182152	117,3654668	402,431696	235,0420002	58,71372284	0,351935424	False	0,351935424
ENSMUSG00000033065	Pfkm	150,7035587	106,0075184	114,4772877	118,0833015	92,63720715	-0,351908522	False	0,351908522
ENSMUSG00000041845	Rhod	296,7700849	304,1406181	378,655644	317,1380098	147,4366818	0,35154076	False	0,35154076
ENSMUSG00000020439	Smtn	133,701106	131,2474037	104,790748	254,160249	109,5989493	-0,351500053	False	0,351500053
ENSMUSG00000044700	Tmem201	309,9083439	249,8748647	395,3869399	182,1856652	202,2361565	0,35142362	False	0,35142362
ENSMUSG00000062526	Mppe1	108,1974268	88,33959863	117,1190713	147,3229762	84,80871077	-0,351381831	False	0,351381831
ENSMUSG00000026436	Elk4	303,7256338	1072,695126	387,4615892	428,4736941	1197,759946	0,351284623	False	0,351284623
ENSMUSG00000005907	Pex1	422,7428033	484,6057982	387,4615892	1048,129876	331,4063467	-0,351178963	False	0,351178963
ENSMUSG00000034998	Foxn2	163,068979	100,9595413	84,537074	61,85315794	127,8654409	-0,350855971	False	0,350855971
ENSMUSG00000020044	Timp3	298,3157624	190,5611342	380,416833	274,4031007	331,4063467	0,350740876	False	0,350740876
ENSMUSG00000036986	Pml	512,3920997	340,7384519	629,6250824	653,3942684	174,8364191	0,350705568	False	0,350705568
ENSMUSG00000029616	Erp29	412,6958993	526,251609	605,8490303	634,2760196	737,183409	0,350673636	False	0,350673636
ENSMUSG00000056724	Nbeal2	234,9429839	184,2511629	398,9093179	611,7839622	210,0646528	-0,350636961	False	0,350636961
ENSMUSG00000039263	Npepl1	956,7743883	1350,333865	1246,921842	1996,170097	1219,940686	0,350560329	False	0,350560329
ENSMUSG00000039463	Slc9a8	415,7872544	580,5173624	530,1179016	1486,724996	665,4221922	0,350467726	False	0,350467726
ENSMUSG00000048310	Pskh1	435,8810622	167,8452374	277,3872741	341,879273	75,675465	-0,350447565	False	0,350447565
ENSMUSG00000098923	Tmem185b	88,87645772	106,0075184	83,65647948	69,72537804	143,5224336	-0,350117466	False	0,350117466
ENSMUSG00000062421	Arf2	94,28632906	256,184836	73,96993975	114,7094929	320,9683515	-0,350109503	False	0,350109503
ENSMUSG00000061787	Rps17	1767,482251	3081,789998	3807,690708	1386,635341	2621,241538	-0,350107292	False	0,350107292
ENSMUSG00000068394	Cep152	78,82955381	135,0333865	176,1189042	250,7864404	100,4657035	0,349894572	False	0,349894572
ENSMUSG00000040414	Slc25a28	346,2317657	2264,017713	441,1778549	3051,047591	4207,816804	0,349622282	False	0,349622282
ENSMUSG00000045210	Vcpip1	447,4736437	342,0004461	257,1336001	218,1729571	570,1754863	0,349603305	False	0,349603305
ENSMUSG00000020817	Rabep1	608,9969451	377,3362856	387,4615892	477,9562204	360,1108334	-0,349556514	False	0,349556514
ENSMUSG00000020982	Nemf	438,1995785	1065,123161	558,2969262	2303,186681	1758,802186	0,349444517	False	0,349444517
ENSMUSG00000029407	Uso1	1601,321917	1877,847468	1009,161321	2040,029609	1166,44596	0,349326727	False	0,349326727
ENSMUSG00000027993	Trim2	330,7749905	286,4726984	377,7750494	682,6339431	259,6451299	-0,349308993	False	0,349308993
ENSMUSG00000028468	Rgp1	509,3007447	319,2845493	399,7899125	247,4126318	189,1886625	-0,349275756	False	0,349275756
ENSMUSG00000048217	Nags	962,9570985	893,4919404	1226,668168	472,3332061	574,0897345	0,349201601	False	0,349201601
ENSMUSG00000071014	Ndufb6	478,3871942	479,5578211	609,3714084	134,9523446	489,2810237	0,34914304	False	0,34914304
ENSMUSG00000004936	Map2k1	472,2044841	458,1039186	307,3274878	247,4126318	601,4894718	0,349127744	False	0,349127744
ENSMUSG00000033124	Atg9a	408,8317055	196,8711055	154,1040411	116,9586986	320,9683515	-0,349076034	False	0,349076034
ENSMUSG00000025289	Prdx4	1001,599037	418,9820964	786,3709071	299,1443639	515,3760116	-0,349023226	False	0,349023226
ENSMUSG00000032558	Nphp3	115,9258144	147,6533291	299,4021371	739,9886895	163,0936746	0,349012023	False	0,349012023
ENSMUSG00000039501	Znfx1	483,7970655	379,8602741	503,7000659	693,8799788	422,7388045	-0,348933176	False	0,348933176
ENSMUSG00000020687	Cdc27	232,6244676	147,6533291	170,835337	166,441225	182,6649155	-0,348803287	False	0,348803287
ENSMUSG00000027274	Mkks	136,792461	106,0075184	107,4325315	68,60077517	43,05673008	-0,348557801	False	0,348557801

ENSMUSG00000005514	Por	4309,348941	6616,635937	10987,17784	10815,30582	5486,471212	0,348408612	False	0,348408612
ENSMUSG00000041203	Trir	582,7204272	822,8202615	766,9978277	457,7133687	738,4881584	-0,348359455	False	0,348359455
ENSMUSG00000026634	Angel2	333,8663455	506,0597007	510,7448221	996,3981443	262,2546287	-0,348302438	False	0,348302438
ENSMUSG00000029629	Phf14	214,0763373	257,4468303	168,1935535	458,8379716	193,1029107	-0,348002927	False	0,348002927
ENSMUSG00000089682	Bcl2l2	217,1676923	276,3767443	124,1638274	118,0833015	174,8364191	0,347826733	False	0,347826733
ENSMUSG00000054027	Nt5dc3	115,9258144	65,62370184	88,94004661	91,0928326	75,675465	-0,347792413	False	0,347792413
ENSMUSG00000037103	Dcaf15	59,50858474	281,4247213	75,73112879	95,59124409	439,7005466	0,347788625	False	0,347788625
ENSMUSG00000038241	Cep250	147,6122037	82,0296273	157,6264192	187,8086796	35,22823371	0,347451739	False	0,347451739
ENSMUSG00000057229	Dmac2	589,675976	445,4839759	595,2818961	750,1101154	512,7665128	0,347179979	False	0,347179979
ENSMUSG00000040506	Ambra1	353,9601534	296,5686525	278,2678686	664,6402971	347,0633395	-0,347112629	False	0,347112629
ENSMUSG00000072568	Fam84b	517,0291323	360,9303601	228,9545754	131,578536	657,5936958	0,346950896	False	0,346950896
ENSMUSG00000015711	Prune1	241,125694	306,6646067	211,342685	205,8023255	157,874677	0,346876279	False	0,346876279
ENSMUSG00000016503	Gtf3a	153,022075	59,31373051	194,6113891	75,3483924	44,36147948	0,346856351	False	0,346856351
ENSMUSG00000028882	Ppp1r8	124,4270408	129,9854094	100,3877754	97,84044983	254,4261323	-0,346797108	False	0,346797108
ENSMUSG00000031527	Eri1	119,0171695	82,0296273	81,01469592	70,84998091	151,35093	0,346727828	False	0,346727828
ENSMUSG00000016833	Mrps18c	153,022075	262,4948074	188,4472275	120,3325073	254,4261323	-0,346713359	False	0,346713359
ENSMUSG00000019868	Vta1	153,022075	223,3729851	123,2832329	120,3325073	264,8641275	-0,346713359	False	0,346713359
ENSMUSG00000018974	Sart3	139,8838161	318,0225551	177,8800932	383,4895792	534,9472526	0,346676007	False	0,346676007
ENSMUSG00000032939	Nup93	53,32587463	888,4439634	67,80577811	267,6554834	2122,827268	0,346572495	False	0,346572495
ENSMUSG00000034216	Vps18	207,1207884	334,4284805	162,9099864	389,1125936	525,8140068	-0,346397319	False	0,346397319
ENSMUSG00000041057	Wdr43	247,3084041	408,8861422	314,3722439	475,7070147	572,7849851	0,34616258	False	0,34616258
ENSMUSG00000018547	Pip4k2b	429,6983521	75,71965597	338,148296	133,8277417	26,09498793	-0,345668161	False	0,345668161
ENSMUSG00000021996	Esd	2516,363012	1042,407264	1784,965094	341,879273	1980,609584	-0,345395536	False	0,345395536
ENSMUSG00000002428	Hltf	159,9776239	243,5648934	125,9250165	303,6427753	480,1477779	-0,345305209	False	0,345305209
ENSMUSG00000031358	Msl3	344,6860882	271,3287672	177,8800932	240,6650145	181,3601661	-0,345241056	False	0,345241056
ENSMUSG00000037003	Tns2	928,1793541	3968,971967	1179,116063	4482,667046	3937,733679	0,345230221	False	0,345230221
ENSMUSG00000039512	Uhrf1bp1	237,2615002	167,8452374	140,0145288	301,3935696	105,6847011	0,345170619	False	0,345170619
ENSMUSG00000026558	Uck2	180,0714317	1065,123161	141,7757179	149,5721819	931,5910691	-0,344958855	False	0,344958855
ENSMUSG00000054428	Atpif1	579,6290721	246,0888819	736,1770194	220,4221628	292,2638648	0,344922758	False	0,344922758
ENSMUSG00000032598	Nckipsd	84,23942515	209,4910482	193,7307946	356,4991103	106,9894505	0,344901053	False	0,344901053
ENSMUSG00000045205	Dpy19l4	183,9356255	74,4576617	121,5220439	110,2110814	144,827183	-0,344868524	False	0,344868524
ENSMUSG00000051768	Xrcc1	207,1207884	176,6791973	222,7904138	366,6205361	163,0936746	-0,344771533	False	0,344771533
ENSMUSG00000022263	Trio	216,3948536	359,6683658	274,7454905	312,6395983	294,8733636	0,344429613	False	0,344429613
ENSMUSG00000004642	Slbp	96,60484535	83,29162157	56,35804933	74,22378953	122,6464433	0,34433794	False	0,34433794
ENSMUSG00000052593	Adam17	196,3010458	177,9411915	129,4473946	165,3166221	249,2071347	0,344277514	False	0,344277514
ENSMUSG00000029577	Ube3b	1017,055812	783,6984393	1182,638442	1291,044097	600,1847224	0,344139427	False	0,344139427
ENSMUSG00000042628	Zfyve1	915,0410952	382,3842626	594,4013016	720,8704407	254,4261323	-0,344096545	False	0,344096545
ENSMUSG00000039908	Slc26a11	95,05916782	179,2031858	120,6414494	347,5022873	304,0066094	0,343827989	False	0,343827989
ENSMUSG00000052160	Pld4	169,2516891	99,69754702	130,3279891	214,7991485	70,45646741	0,343818043	False	0,343818043
ENSMUSG00000049076	Acap2	227,2145963	166,5832431	170,835337	181,0610623	288,3496166	0,343763588	False	0,343763588
ENSMUSG00000032501	Trib1	403,4218342	564,111437	317,894622	757,9823355	1518,728298	-0,343740558	False	0,343740558
ENSMUSG00000020472	Zkscan17	248,0812429	340,7384519	195,4919836	373,3681534	591,0514766	-0,343703209	False	0,343703209
ENSMUSG00000037369	Kdm6a	225,6689187	750,8865884	177,8800932	597,1641248	3273,616236	-0,343302665	False	0,343302665
ENSMUSG00000038812	Trmt112	265,8565344	253,6608475	337,2677015	138,3261532	334,0158455	0,343246239	False	0,343246239
ENSMUSG00000041119	Pde9a	481,4785492	61,83771904	379,5362385	209,1761341	273,9973733	-0,343233883	False	0,343233883
ENSMUSG00000029202	Pds5a	591,9944923	396,2661996	440,2972604	466,7101917	434,481549	-0,343056781	False	0,343056781
ENSMUSG00000038418	Egr1	3378,851071	1663,308443	151,4622576	158,5690049	2664,298268	-0,342777162	False	0,342777162
ENSMUSG00000032307	Ube2q2	258,1281468	93,38757569	114,4772877	49,48252635	203,5409059	-0,3427687	False	0,3427687
ENSMUSG00000042608	Stk40	735,7425022	535,0855688	673,6548085	624,1545937	932,8958185	0,342515042	False	0,342515042
ENSMUSG00000018372	Cep95	105,8789105	157,7492833	199,0143617	231,6681916	83,50396138	-0,34249871	False	0,34249871
ENSMUSG00000024758	Rtn3	1363,287578	1075,219115	1058,474614	872,6918284	662,8126934	-0,342459231	False	0,342459231
ENSMUSG00000035105	Egln3	200,1652396	61,83771904	127,6862055	38,23649764	157,874677	-0,342411677	False	0,342411677
ENSMUSG00000032058	Ppp2r1b	180,8442705	229,6829564	142,6563124	214,7991485	254,4261323	-0,342204305	False	0,342204305
ENSMUSG00000027381	Bcl2l11	70,32832742	720,598726	55,47745481	321,6364213	165,7031734	-0,342204305	False	0,342204305
ENSMUSG00000042797	Aqp11	432,0168684	34,07384519	340,7900796	25,86586605	35,22823371	-0,342204305	False	0,342204305
ENSMUSG00000036985	Zdhhc9	1522,492363	652,4510356	1201,130926	428,4736941	486,6715249	-0,342041574	False	0,342041574
ENSMUSG00000028630	Dyrk2	603,5870737	371,0263142	260,6559782	383,4895792	476,2335297	-0,341892711	False	0,341892711
ENSMUSG00000037029	Zfp146	200,1652396	253,6608475	140,0145288	79,84680388	176,1411685	0,341709397	False	0,341709397
ENSMUSG00000034345	Gtf2h5	347,0046045	752,1485826	634,028055	439,7197228	784,1543873	0,341629437	False	0,341629437
ENSMUSG00000025340	Rabgef1	178,5257542	195,6091113	140,8951233	185,5594738	431,8720502	-0,341510535	False	0,341510535
ENSMUSG00000030990	Pgap2	178,5257542	169,1072317	321,4170001	203,5531198	140,9129348	-0,341328166	False	0,341328166
ENSMUSG00000031503	Col4a2	221,0318862	864,4660723	280,0290576	718,621235	790,6781343	0,34132203	False	0,34132203
ENSMUSG00000002996	Hbp1	1117,524851	1100,459	371,6108878	1051,503685	1415,653095	0,341160864	False	0,341160864
ENSMUSG00000046814	Gchfr	1064,198976	368,5023257	840,0871729	147,3229762	463,1860358	-0,341156977	False	0,341156977
ENSMUSG00000062006	Rpl34	1411,203581	3041,406181	2577,500163	1114,481446	2933,076643	-0,340553528	False	0,340553528
ENSMUSG00000070699	Sars2	151,4763975	214,5390252	255,372411	317,1380098	191,7981613	0,340495874	False	0,340495874
ENSMUSG00000003378	Grik5	173,1158829	389,9562282	1020,60905	2017,537552	219,1978986	0,340495874	False	0,340495874
ENSMUSG00000031072	Oraov1	144,5208486	182,9891686	117,1190713	75,3483924	150,0461806	0,340480624	False	0,340480624
ENSMUSG00000035770	Dync1li2	216,3948536	129,9854094	131,2085836	107,9618757	170,9221709	-0,340326642	False	0,340326642
ENSMUSG00000053838	Nudcd3	261,9923406	127,4614209	206,9397124	188,9332824	136,9986866	-0,340314105	False	0,340314105
ENSMUSG00000074922	Fam122a	132,1554284	277,6387386	167,312959	248,5372346	242,6833878	0,340313505	False	0,340313505
ENSMUSG00000003420	Fcgrt	1731,158829	922,5178085	1367,563291	1212,321896	712,3931705	-0,340130491	False	0,340130491
ENSMUSG00000041890	Git2	320,7280866	581,7793567	405,9540741	557,8030243	494,5000213	0,339965826	False	0,339965826
ENSMUSG00000026675	Hsd17b7	915,0410952	521,2036319	722,9681016	176,5626508	442,3100454	-0,339904542	False	0,339904542
ENSMUSG00000037287	Tbcel	1087,384139	800,1043647	641,0728112	859,1965939	474,9287803	-0,339801511	False	0,339801511
ENSMUSG00000031657	Heatr3	287,4960198	189,2991399	179,6412823	227,1697801	157,874677	-0,339771054	False	0,339771054
ENSMUSG00000025417	Pip4k2c	297,5429237	435,3880218	235,1187371	337,3808615	636,7177055	-0,339708293	False	0,339708293
ENSMUSG00000033685	Ucp2	518,5748098	656,2370184	1319,130592	1107,733829	704,5646741	0,339664851	False	0,339664851
ENSMUSG00000041187	Prkd2	145,2936874	205,7050654	162,9099864	326,1348328	114,8179469	-0,33962386	False	0,33962386
ENSMUSG00000073639	Rab18	845,4856065	718,0747374	508,9836331	319,3872155	1069,894505	0,33961645	False	0,33961645
ENSMUSG00000025239	Limd1	416,5600932	617,1151961	614,6549756	890,6854743	527,1187562	0,339603393	False	0,339603393
ENSMUSG00000049878	Rlf	171,5702053	461,8899014	135,6115562	445,3427372	837,6491126	-0,339318914	False	0,339318914
ENSMUSG00000032478	Nme6	298,3157624	377,3362856	471,9986632	430,7228998	437,0910478	0,339010631	False	0,339010631
ENSMUSG00000018541	Cwc25	188,5726581	238,5169163	241,2828987	341,879273	477,5382791	0,338971081	False	0,338971081
ENSMUSG00000059183	Mtfmt	147,6122037	331,904492	186,6860384	242,9142203	219,1978986	0,338802038	False	0,338802038
ENSMUSG00000040734	Ppp1r13l	61,05426226	148,9153234	104,790748	222,6713686	48,27572767	-0,338794009	False	0,338794009
ENSMUSG00000029397	Rchy1	275,9034383	434,1260276	238,6411152	218,1729571	601,4894718	-0,338691147	False	0,338691147
ENSMUSG00000034445	Cyb561a3	204,0294334	170,3692259	258,0141946	254,160249	63,93272043	0,338673146	False	0,338673146
ENSMUSG00000035960	Apex1	382,5551876	590,6133166	380,416833	302,5181725	983,781045	-0,338646078	False	0,338646078
ENSMUSG00000012535	Tnpo3	523,2118424	519,9416376	445,5808276	464,460986	661,507944	0,338363287	False	0,338363287
ENSMUSG00000063049	Ing2	121,3356858	167,8452374	95,98480277	121,4571101	100,4657035	-0,338126013	False	0,338126013
ENSMUSG00000022228	Zscan26	1118,29769	884,6579806	2404,023042	3089,284088	1270,825912	-0,338112579	False	0,338112579
ENSMUSG00000022477	Aco2	2442,17049	1932,113221	2099,337338	2711,417523	3144,446046	-0,337984282	False	0,337984282
ENSMUSG00000032869	Psmf1	370,9626061	591,8753108	521,3119563	293,5213495	1076,418252	-0,337808325	False	0,337808325
ENSMUSG00000027067	Ssrp1	663,8684972	707,9787833	329,3423508	591,5411105	838,953862	0,337693979	False	0,337693979
ENSMUSG00000005374	Tbl2	218,7133699	276,3767443	365,4467262	517,3173209	657,5936958	0,337594811	False	0,337594811
ENSMUSG00000001424	Snd1	1511,67262	1400,813635	1910,009516	927,7973691	1426,09109	0,337434094	False	0,337434094
ENSMUSG00000030880	Polr3e	129,0640734	180,4651801	102,1489644	125,9555216	290,9591154	-0,337412886	False	0,337412886
ENSMUSG00000029416	Slc15a4	258,1281468	128,7234151	204,2979288	132,7031389	136,9986866	-0,337412886	False	0,337412886
ENSMUSG00000022760	Thap7	149,1578812	229,6829564	188,4472275	293,5213495	283,130619	0,33732036	False	0,33732036
ENSMUSG00000028680	Plk3	575,7648783	4457,363748	727,3710742	3925,988625	1231,68343	0,337211767	False	0,337211767
ENSMUSG00000026688	Mgst3	440,5180948	186,7751514	348,7154303	19,11824882	228,3311444	-0,337151078	False	0,337151078
ENSMUSG00000022718	Dgcr8	110,5159431	367,2403314	147,9398795	611,7839622	139,6081854	0,337129023	False	0,337129023
ENSMUSG00000027475	Kif3b	122,8813633	155,2252947	43,14913152	57,35474645	131,779689	0,337097544	False	0,337097544
ENSMUSG00000041354	Rgl2	217,9405311	501,0117237	538,0432522	1142,596518	275,3021227	0,337081209	False	0,337081209
ENSMUSG00000078515	Ddi2	1531,766428	1212,77649	1349,9514	1641,920193	2249,38796	-0,336882632	False	0,336882632
ENSMUSG00000020634	Ubxn2a	378,6909938	478,2958269	292,3573809	244,0388231	356,1965852	0,336882093	False	0,336882093
ENSMUSG00000000782	Tcf7	98,92336164	124,9374323	57,23864386	29,23967466	99,16095413	0,336822614	False	0,336822614
ENSMUSG00000029502	Golga3	291,3602136	244,8268876	210,4620905	319,3872155	367,9393298	0,336664012	False	0,336664012
ENSMUSG00000022472	Desi1	562,6266193	710,5027718	582,0729783	298,019761	377,0725756	0,336662466	False	0,336662466
ENSMUSG00000066979	Bub3	344,6860882	326,8565149	272,9843015	292,3967466	521,8997586	-0,336465083	False	0,336465083
ENSMUSG00000021226	Acot2	114,3801369	658,7610069	144,4175014	1422,622633	865,0488499	0,33640905	False	0,33640905
ENSMUSG00000021375	Kif13a	187,7998194	133,7713922	147,9398795	132,7031389	148,7414312	-0,336389116	False	0,336389116

ENSMUSG00000028184	Adgrl2	801,433797	634,7831159	386,5809947	378,9911677	163,0936746	-0,336319595	False	0,336319595
ENSMUSG00000040167	Ikzf5	81,14807009	87,07760436	64,28340002	97,84044983	243,9881371	-0,336110547	False	0,336110547
ENSMUSG00000031511	Arhgef7	472,9773228	623,4251675	597,0430851	608,4101535	1041,190018	0,336064032	False	0,336064032
ENSMUSG00000027665	Pik3ca	413,4687381	384,9082512	327,5811618	687,1323546	447,529043	-0,335925859	False	0,335925859
ENSMUSG00000027363	Usp8	731,1054696	499,7497294	474,6404467	479,0808233	579,3087321	-0,335747134	False	0,335747134
ENSMUSG00000026421	Csrp1	149,93072	114,8414782	128,5668	134,9523446	189,1886625	0,335529621	False	0,335529621
ENSMUSG00000019814	Ltv1	181,6171093	310,4505895	160,2682028	143,9491676	331,4063467	-0,335340672	False	0,335340672
ENSMUSG00000020745	Pafah1b1	865,5794143	771,0784966	383,9392111	369,9943448	1092,075245	0,335334169	False	0,335334169
ENSMUSG00000038563	Efl1	143,7480099	164,0592546	95,98480277	102,3388613	181,3601661	0,335315635	False	0,335315635
ENSMUSG00000028646	Rragc	444,3822886	519,9416376	352,2378083	410,4800481	992,9142907	-0,335251545	False	0,335251545
ENSMUSG00000022750	Klhl22	191,6640132	143,8673463	117,9996658	241,7896174	86,11346017	0,335172814	False	0,335172814
ENSMUSG00000026639	Lamb3	43,27897072	402,5761709	54,59686029	431,8475027	1710,526459	0,3351518	False	0,3351518
ENSMUSG00000020346	Mgat1	880,2633508	687,786875	1110,429691	646,6466512	634,1082067	0,33511094	False	0,33511094
ENSMUSG00000031667	Aktip	132,1554284	41,64581078	104,790748	49,48252635	20,87599034	-0,33472434	False	0,33472434
ENSMUSG00000029466	Anapc7	218,7133699	406,3621537	173,4771206	336,2562586	661,507944	-0,334296012	False	0,334296012
ENSMUSG00000021371	Mcur1	476,0686779	403,8381652	309,9692713	289,022938	600,1847224	0,334236883	False	0,334236883
ENSMUSG00000027164	Traf6	226,4417575	471,9858555	179,6412823	290,1475409	848,0871077	-0,334021101	False	0,334021101
ENSMUSG00000039879	Heca	457,5205476	335,6904748	99,50718086	146,1983733	576,6992333	0,333982567	False	0,333982567
ENSMUSG00000041528	Rnf123	1027,875555	1942,209176	1512,861387	2579,838987	1295,616151	0,33397275	False	0,33397275
ENSMUSG00000047368	Abhd17b	147,6122037	251,136859	117,1190713	185,5594738	594,9657248	-0,333835981	False	0,333835981
ENSMUSG00000023057	Fabp2	728,7869533	545,181523	918,4600853	204,6777226	870,2678475	0,333719893	False	0,333719893
ENSMUSG00000009640	Dmap1	160,7504627	311,7125837	202,5367398	278,9015122	334,0158455	0,333360744	False	0,333360744
ENSMUSG00000021832	Psmc6	1087,384139	1217,824467	769,6396112	592,6657133	1369,986866	0,333300373	False	0,333300373
ENSMUSG00000028863	Meaf6	124,4270408	136,2953807	156,7458247	59,6039522	104,3799517	0,333126964	False	0,333126964
ENSMUSG00000015970	Chdh	2384,980422	63,09971331	1893,27822	354,2499046	36,5329831	-0,333090991	False	0,333090991
ENSMUSG00000026003	Acadl	2408,938424	4626,47098	3034,528719	3336,69672	5284,235056	0,333074958	False	0,333074958
ENSMUSG00000025982	Sf3b1	1303,778993	1654,474483	1642,308781	3765,170414	3415,83392	0,333026068	False	0,333026068
ENSMUSG00000020105	Lrig3	129,8369122	95,91156423	94,22361373	67,4761723	103,0752023	-0,333003301	False	0,333003301
ENSMUSG00000030894	Tpp1	1442,88997	1038,621281	1585,070138	1817,358241	999,4380377	0,332881545	False	0,332881545
ENSMUSG00000005547	Cyp2a5	6372,055599	3430,100415	2743,932527	260,9078662	5059,81816	-0,332673317	False	0,332673317
ENSMUSG00000030094	Xpc	261,9923406	175,417203	110,9549096	208,0515312	129,1701903	-0,332583728	False	0,332583728
ENSMUSG00000024070	Prkd3	2573,55308	1071,433132	2043,859883	793,9696274	918,5435751	-0,332465243	False	0,332465243
ENSMUSG00000031783	Polr2c	219,4862086	357,1443773	222,7904138	174,3134451	557,1279923	-0,33244644	False	0,33244644
ENSMUSG00000033943	Mga	326,9107967	222,1109908	298,5215426	411,604651	186,5791637	0,33236226	False	0,33236226
ENSMUSG00000034118	Tpst1	288,2688585	108,5315069	228,9545754	136,0769475	207,455154	-0,332353596	False	0,332353596
ENSMUSG00000027677	Ttc14	482,251388	176,6791973	383,0586166	254,160249	127,8654409	-0,332220217	False	0,332220217
ENSMUSG00000046230	Vps13a	170,0245278	320,5465436	213,9844686	522,9403353	495,8047707	0,331763201	False	0,331763201
ENSMUSG00000026565	Pou2f1	117,4714919	325,5945207	93,34301921	291,2721437	343,1490913	-0,331696648	False	0,331696648
ENSMUSG00000009614	Sardh	10837,51797	2007,832877	8613,095008	1078,494154	3972,961912	-0,331430735	False	0,331430735
ENSMUSG00000030649	Anapc15	188,5726581	237,254922	365,4467262	239,5404117	242,6833878	0,331317508	False	0,331317508
ENSMUSG00000025731	Mettl26	1075,791558	378,5982798	855,0572798	97,84044983	425,3483033	-0,331305599	False	0,331305599
ENSMUSG00000039197	Adk	6977,961189	5074,478944	5252,746317	3534,626826	5546,489685	-0,33123058	False	0,33123058
ENSMUSG00000044477	Zfand3	1000,826198	972,9975792	705,3562112	423,9752826	1259,083168	0,331182123	False	0,331182123
ENSMUSG00000022906	Parp9	782,112828	294,044664	621,6997317	441,9689285	292,2638648	-0,33115879	False	0,33115879
ENSMUSG00000031921	Terf2	170,7973666	340,7384519	214,8650631	363,2467275	390,1200696	0,33114519	False	0,33114519
ENSMUSG00000029203	Ube2k	631,4092692	926,3037913	501,9388769	780,4743929	1366,072618	-0,331063748	False	0,331063748
ENSMUSG00000034187	Nsf	241,8985328	135,0333865	144,4175014	192,307091	138,303436	-0,330990058	False	0,330990058
ENSMUSG00000024271	Elp2	453,6563538	899,8019118	570,6252495	834,4553307	1336,063382	0,330943721	False	0,330943721
ENSMUSG00000042178	Armc5	140,6566548	253,6608475	111,8355041	208,0515312	420,1293057	-0,330799542	False	0,330799542
ENSMUSG00000009418	Nav1	119,7900082	247,3508762	243,0440878	669,1387086	95,24670595	-0,330766473	False	0,330766473
ENSMUSG00000027875	Hmgcs2	38036,80539	13314,03951	30248,42179	13368,15433	12890,92404	-0,330536211	False	0,330536211
ENSMUSG00000020010	Vnn3	1210,265503	677,6909209	962,4898113	910,928326	802,4208789	-0,3304804	False	0,3304804
ENSMUSG00000023852	Chd1	386,4193814	634,7831159	307,3274878	563,4260387	1229,073932	-0,330390647	False	0,330390647
ENSMUSG00000035311	Gnptab	156,1134301	165,3212489	124,1638274	208,0515312	135,6939372	-0,330349719	False	0,330349719
ENSMUSG00000025371	Chmp6	181,6171093	209,4910482	102,1489644	105,7126699	228,3311444	0,330227538	False	0,330227538
ENSMUSG00000013495	Tmem175	181,6171093	83,29162157	215,7456576	228,2943829	133,0844384	0,329995245	False	0,329995245
ENSMUSG00000024308	Tapbp	835,4387026	815,2482959	883,2363044	664,6402971	1420,872093	-0,329960213	False	0,329960213
ENSMUSG00000064037	Gpn1	157,6591076	107,2695126	198,1337672	136,0769475	117,4274457	0,329666261	False	0,329666261
ENSMUSG00000022607	Ptk2	285,9503422	750,8865884	359,2825645	700,627589	952,4670595	0,329354293	False	0,329354293
ENSMUSG00000018920	Cxcl16	142,9751711	74,4576617	179,6412823	142,8245647	63,93272043	0,329354293	False	0,329354293
ENSMUSG00000004626	Stxbp2	557,216748	1301,116088	700,0726441	1266,302833	912,0198282	0,329266012	False	0,329266012
ENSMUSG00000004931	Apba3	492,2982919	687,786875	1069,922343	690,5061632	618,4512139	0,32912706	False	0,32912706
ENSMUSG00000064302	Clasp1	213,3034985	281,4247213	188,4472275	169,8150336	263,5593781	-0,328943443	False	0,328943443
ENSMUSG00000031641	Cbr4	305,2713113	161,5352661	243,0440878	173,1888422	129,1701903	-0,328873975	False	0,328873975
ENSMUSG00000056612	Ppp1r14b	156,8862688	292,7826697	431,4913152	327,2594356	197,0171589	0,328602195	False	0,328602195
ENSMUSG00000011306	Sugp1	280,5404709	464,4138899	389,2227782	736,6148809	352,2823371	0,328523232	False	0,328523232
ENSMUSG00000022570	Tsta3	287,4960198	297,8306468	228,9545754	444,2181343	246,5976359	-0,328480587	False	0,328480587
ENSMUSG00000041733	Coq5	359,3700247	256,184836	286,1932193	213,6745456	202,2361565	-0,328480587	False	0,328480587
ENSMUSG00000031879	Fam96b	114,3801369	129,9854094	197,2531727	91,0928326	157,874677	-0,328427088	False	0,328427088
ENSMUSG00000002820	Atg4d	273,584922	249,8748647	460,5509344	325,0102299	217,8931492	-0,328367884	False	0,328367884
ENSMUSG00000041571	Selenow	1336,238221	406,3621537	1677,532562	475,7070147	849,3918571	0,32816354	False	0,32816354
ENSMUSG00000036825	Ssx2ip	87,3307802	98,43555276	69,56696715	106,8372728	133,0844384	-0,328087803	False	0,328087803
ENSMUSG00000027502	Rtf2	649,9573995	1253,160306	517,7895783	3614,473629	1242,121425	-0,327979234	False	0,327979234
ENSMUSG00000028538	St3gal3	760,4733426	223,3729851	605,8490303	329,5086414	161,7889252	-0,327939339	False	0,327939339
ENSMUSG00000032077	Bud13	102,7875555	230,9449507	81,89529044	195,6808997	135,6939372	-0,327813213	False	0,327813213
ENSMUSG00000050854	Tmem125	106,6517493	429,0780505	133,8503672	413,8538568	156,5699276	0,327713468	False	0,327713468
ENSMUSG00000066037	Hnrnpr	393,3749303	345,7864289	313,4916494	625,2791966	433,1767996	-0,327478004	False	0,327478004
ENSMUSG00000020309	Chac2	282,8589872	16,40592546	225,4321973	25,86586605	11,74274457	-0,327389427	False	0,327389427
ENSMUSG00000003526	Prodh	2990,886012	656,2370184	2383,769368	1098,737006	444,9195442	-0,327328267	False	0,327328267
ENSMUSG00000046324	Ermp1	324,5922804	258,7088246	271,2231124	378,9911677	430,5673008	-0,327299424	False	0,327299424
ENSMUSG00000050628	Ubald2	164,6146565	179,2031858	131,2085836	142,8245647	635,4129561	-0,327230688	False	0,327230688
ENSMUSG00000060032	H2afj	418,1057707	1138,318828	1455,622743	2009,665332	524,5092574	0,327100281	False	0,327100281
ENSMUSG00000016256	Ctsz	2227,321314	2243,825805	3238,826648	1775,747934	2716,488244	-0,32688289	False	0,32688289
ENSMUSG00000025579	Gaa	1503,171394	488,391781	1198,489143	905,3053117	648,4604501	-0,326792677	False	0,326792677
ENSMUSG00000020460	Rps27a	1527,902234	4201,178912	3485,393114	1916,323293	3367,558192	0,326788739	False	0,326788739
ENSMUSG00000075705	Msrb1	5501,839152	1646,902517	6900,338665	1718,393188	1195,150447	0,326753208	False	0,326753208
ENSMUSG00000024527	Afg3l2	811,4807009	579,2553682	677,1771865	508,320498	1017,704529	0,326690074	False	0,326690074
ENSMUSG00000017132	Cyth1	543,3056503	873,3000322	433,2525043	1197,702058	986,3905438	-0,326555962	False	0,326555962
ENSMUSG00000041598	Cdc42ep4	506,2093896	734,4806629	650,7593509	403,7324309	541,4709996	-0,326334788	False	0,326334788
ENSMUSG00000025049	Taf5	46,37032577	203,1810768	58,11923838	121,4571101	241,3786384	0,325813935	False	0,325813935
ENSMUSG00000025646	Atrip	100,4690392	141,3433578	125,9250165	150,6967848	264,8641275	0,325813935	False	0,325813935
ENSMUSG00000031161	Hdac6	310,6811826	432,8640333	551,2521701	675,8863258	247,9023853	-0,32566271	False	0,32566271
ENSMUSG00000031546	Gins4	137,5652998	109,7935012	104,790748	71,97458378	70,45646741	-0,325323941	False	0,325323941
ENSMUSG00000020170	Frs2	344,6860882	275,11475	307,3274878	650,0204598	418,8245563	-0,325249581	False	0,325249581
ENSMUSG00000041133	Smc1a	728,0141146	612,0672191	417,4018029	503,8220865	912,0198282	0,325098769	False	0,325098769
ENSMUSG00000022865	Cxadr	993,870649	473,2478498	793,4156633	513,9435123	309,225607	-0,32498122	False	0,32498122
ENSMUSG00000048222	Mfap1b	61,05426226	128,7234151	87,17885756	76,47299527	309,225607	0,324858367	False	0,324858367
ENSMUSG00000027014	Cwc22	164,6146565	179,2031858	71,32815619	86,59442111	206,1504046	0,324604501	False	0,324604501
ENSMUSG00000032299	Rpl4	7528,995227	6929,610515	5862,117725	4190,2703	9428,119139	0,324512645	False	0,324512645
ENSMUSG00000021003	Galc	90,42213525	97,17355849	72,20875071	149,5721819	84,80871077	-0,324502304	False	0,324502304
ENSMUSG00000027086	Fastkd1	227,2145963	208,2290539	192,8502001	284,5245265	92,63720715	0,324497504	False	0,324497504
ENSMUSG00000041915	Ammecr1l	702,5104354	485,8677925	526,5955235	458,8379716	561,0422405	-0,324410261	False	0,324410261
ENSMUSG00000041852	Tcf20	311,4540214	389,9562282	211,342685	190,0578853	33,92348431	0,324292993	False	0,324292993
ENSMUSG00000029053	Prkcz	323,8194416	94,64956996	210,4620905	258,6586605	65,23746983	-0,324140108	False	0,324140108
ENSMUSG00000001419	Mef2d	540,2142952	956,5916537	676,296592	1152,717943	1064,675508	0,324124267	False	0,324124267
ENSMUSG00000020290	Xpo1	408,8317055	306,6646067	504,5806605	511,6943066	249,2071347	0,323775097	False	0,323775097
ENSMUSG00000003531	Dgcr6	534,8044239	398,7901881	669,2518359	661,2664885	275,3021227	0,323537792	False	0,323537792
ENSMUSG00000049580	Tsku	352,4144758	2209,75196	3386,766527	2577,589782	441,005296	0,323522795	False	0,323522795
ENSMUSG00000018449	Rpain	237,2615002	469,461867	387,4615892	296,8951581	306,6161082	0,323475548	False	0,323475548
ENSMUSG00000020034	Tcp11l2	258,9009855	167,8452374	44,91032056	206,9269284	130,4749397	-0,323279055	False	0,323279055
ENSMUSG00000015542	Nat9	228,7602738	94,64956996	286,1932193	194,5562968	53,49472526	0,323152954	False	0,323152954
ENSMUSG00000026939	Tmem141	199,3924008	87,07760436	159,3876083	127,0801245	57,40897345	-0,323070957	False	0,323070957
ENSMUSG00000006005	Tpr	948,273162	676,4289266	496,6553098	643,2728426	758,0593994	-0,322991807	False	0,322991807

ENSMUSG00000045576	St7l	248,0812429	161,5352661	177,8800932	130,4539331	198,3219083	-0,3229686	False	0,3229686
ENSMUSG00000042557	Sin3a	373,2811224	466,9378785	242,1634932	398,1094166	259,6451299	0,322968076	False	0,322968076
ENSMUSG00000022831	Hcls1	95,83200659	98,43555276	76,61172331	176,5626508	150,0461806	-0,322942403	False	0,322942403
ENSMUSG00000029175	Slc35f6	244,9898878	555,2774771	306,4468933	386,8633878	574,0897345	0,322914877	False	0,322914877
ENSMUSG00000028869	Gnl2	450,5649987	1877,847468	563,5804933	855,8227853	4124,312842	0,322886433	False	0,322886433
ENSMUSG00000066149	Cdc26	265,8565344	176,6791973	165,5517699	212,5499427	180,0554167	-0,322846056	False	0,322846056
ENSMUSG00000004789	Dlst	1935,188262	2550,490412	1547,204573	3541,374443	3445,843156	-0,32280996	False	0,32280996
ENSMUSG00000002985	Apoe	344030,7209	212570,3142	275092,4447	93157,60347	233809,7871	-0,322620882	False	0,322620882
ENSMUSG00000034586	Hid1	31,68638927	478,2958269	39,62675344	736,6148809	1780,982926	0,322611503	False	0,322611503
ENSMUSG00000022394	L3mbtl2	168,4788503	210,7530424	134,7309617	192,307091	364,0250816	-0,322486071	False	0,322486071
ENSMUSG00000045639	Zfp629	174,6615604	312,974578	218,3874412	318,2626127	290,9591154	0,322327759	False	0,322327759
ENSMUSG00000037643	Prkci	197,0738845	69,40968464	157,6264192	139,4507561	106,9894505	-0,322227248	False	0,322227248
ENSMUSG00000041135	Ripk2	55,64439092	152,7013062	69,56696715	168,6904307	181,3601661	0,322166158	False	0,322166158
ENSMUSG00000029695	Aass	3245,922804	2293,043582	1669,607212	2352,669207	4057,770623	0,322058626	False	0,322058626
ENSMUSG00000003299	Mrpl4	707,147468	1336,451928	1183,519036	565,6752444	1440,443334	-0,322037069	False	0,322037069
ENSMUSG00000025466	Fuom	214,8491761	191,8231285	268,5813289	120,3325073	180,0554167	0,322034771	False	0,322034771
ENSMUSG00000020674	Pxdn	120,562847	26,50187959	64,28340002	150,6967848	33,92348431	0,321863246	False	0,321863246
ENSMUSG00000025494	Sigirr	223,3504025	23,97789106	279,1484631	62,97776081	24,79023853	0,321723759	False	0,321723759
ENSMUSG00000018068	Ints2	77,28387628	61,83771904	158,5070138	61,85315794	159,1794264	-0,321680352	False	0,321680352
ENSMUSG00000022091	Sorbs3	608,2241063	773,6024851	1402,787072	2607,954059	486,6715249	-0,321654632	False	0,321654632
ENSMUSG00000017679	Ttpal	148,3850425	388,694234	324,0587837	482,4546319	118,7321951	-0,321634487	False	0,321634487
ENSMUSG00000032913	Lrig2	95,83200659	362,1923544	119,7608548	210,300737	375,7678262	0,321576942	False	0,321576942
ENSMUSG00000024668	Sdhaf2	332,320668	251,136859	265,9395453	137,2015503	189,1886625	-0,321477699	False	0,321477699
ENSMUSG00000000384	Tbrg4	408,0588668	740,7906342	509,8642276	672,5125172	1017,704529	0,32133583	False	0,32133583
ENSMUSG00000051390	Zbtb22	292,9058911	365,9783372	197,2531727	233,9173973	249,2071347	0,321321046	False	0,321321046
ENSMUSG00000005982	Naa60	1155,39395	339,4764576	1443,29442	734,3656752	285,7401178	0,320980781	False	0,320980781
ENSMUSG00000041264	Uspl1	272,8120833	441,6979931	340,7900796	829,9569193	413,6055587	0,320975794	False	0,320975794
ENSMUSG00000037426	Depdc5	146,0665262	74,4576617	75,73112879	116,9586986	56,10422405	-0,320626432	False	0,320626432
ENSMUSG00000027708	Dcun1d1	571,9006845	304,1406181	455,2673673	389,1125936	457,9670382	-0,320520867	False	0,320520867
ENSMUSG00000008892	Vdac3	762,7918589	1018,429373	966,8927839	952,5386323	1290,397153	0,320488159	False	0,320488159
ENSMUSG00000034729	Mrps10	391,8292527	386,1702454	334,6259179	215,9237514	489,2810237	0,320438222	False	0,320438222
ENSMUSG00000042426	Dhx29	204,8022721	157,7492833	120,6414494	203,5531198	255,7308817	0,320394668	False	0,320394668
ENSMUSG00000031988	Vps26b	285,1775035	228,4209622	225,4321973	142,8245647	207,455154	-0,320165125	False	0,320165125
ENSMUSG00000015474	Ppt2	299,0886012	235,9929278	373,3720768	154,0705934	206,1504046	0,32004111	False	0,32004111
ENSMUSG00000004462	Tbccd1	374,8268	112,3174897	264,1783563	300,2689667	198,3219083	-0,319968731	False	0,319968731
ENSMUSG00000025792	Slc25a10	2007,062267	347,0484232	1607,965595	712,9982206	340,5395925	-0,319848837	False	0,319848837
ENSMUSG00000068039	Tcp1	1432,070227	1052,503218	1147,414661	1038,008451	1125,998729	-0,319715386	False	0,319715386
ENSMUSG00000011179	Odc1	770,5202465	561,5874484	427,9689371	467,8347946	961,6003052	0,319604488	False	0,319604488
ENSMUSG00000022216	Psme1	950,5916782	340,7384519	761,7142605	302,5181725	384,901072	-0,319575866	False	0,319575866
ENSMUSG00000037376	Trmt6	178,5257542	388,694234	222,7904138	486,9530434	537,5567514	0,319554945	False	0,319554945
ENSMUSG00000022464	Slc38a4	10213,83709	3499,5101	4191,629919	2851,992882	8184,692964	-0,319524751	False	0,319524751
ENSMUSG00000044952	Kctd21	140,6566548	6,309971331	112,7160987	11,24602872	11,74274457	-0,319484229	False	0,319484229
ENSMUSG00000029729	Zkscan1	411,1502218	247,3508762	279,1484631	329,5086414	302,70186	-0,319349307	False	0,319349307
ENSMUSG00000040274	Cdk6	187,0269806	214,5390252	233,357548	133,8277417	152,6556794	0,319295724	False	0,319295724
ENSMUSG00000037531	Mrpl47	138,3381385	155,2252947	172,5965261	89,96822973	114,8179469	0,319204478	False	0,319204478
ENSMUSG00000021621	Zcchc9	132,9282672	230,9449507	106,551937	127,0801245	465,7955346	-0,319091106	False	0,319091106
ENSMUSG00000039640	Mrpl12	1063,426138	415,1961136	852,4154962	464,460986	404,4723129	-0,319091106	False	0,319091106
ENSMUSG00000045095	Magi1	391,056414	323,0705321	487,8493646	371,1189476	306,6161082	0,319059003	False	0,319059003
ENSMUSG00000015804	Med28	138,3381385	92,12558143	108,3131261	61,85315794	110,9036987	-0,318891468	False	0,318891468
ENSMUSG00000017664	Slc35c2	277,4491158	407,624148	346,0736467	686,0077517	422,7388045	0,31885588	False	0,31885588
ENSMUSG00000000326	Comt	12462,02505	2252,659765	9991,225434	1301,165522	1619,194001	-0,31880498	False	0,31880498
ENSMUSG00000093674	Rpl41	2939,878654	3666,093343	4150,241977	5347,486655	4464,852435	0,318486911	False	0,318486911
ENSMUSG00000034673	Pbx2	229,5331126	241,0409048	286,1932193	282,2753208	93,94195655	0,318287198	False	0,318287198
ENSMUSG00000029163	Emilin1	168,4788503	229,6829564	221,0292247	312,6395983	210,0646528	0,318265927	False	0,318265927
ENSMUSG00000028330	Ncbp1	276,6762771	508,5836893	221,9098193	545,4323927	665,4221922	-0,318225442	False	0,318225442
ENSMUSG00000028901	Gmeb1	106,6517493	233,4689392	132,9697726	253,0356461	238,7691396	0,318190694	False	0,318190694
ENSMUSG00000002803	Btbd6	170,7973666	147,6533291	226,3127919	192,307091	136,9986866	-0,318123669	False	0,318123669
ENSMUSG00000074207	Adh1	54437,98961	4316,02039	43671,32407	3061,169017	10251,41601	-0,317927516	False	0,317927516
ENSMUSG00000038515	Grtp1	218,7133699	272,5907615	252,7306275	197,9301054	69,15171802	0,317695253	False	0,317695253
ENSMUSG00000031617	Tmem184c	333,8663455	541,3955402	717,6845345	416,1030625	455,3575394	0,317670233	False	0,317670233
ENSMUSG00000017837	Nkiras2	375,5996387	290,2586812	256,2530056	149,5721819	301,3971106	-0,317530089	False	0,317530089
ENSMUSG00000028790	Khdrbs1	355,5058309	469,461867	285,3126248	285,6491294	618,4512139	-0,317329637	False	0,317329637
ENSMUSG00000058022	Adtrp	2735,84922	3408,646513	3333,930856	1341,651226	2447,709868	0,317210274	False	0,317210274
ENSMUSG00000039840	Epg5	306,8169888	160,2732718	201,6561453	246,2880289	84,80871077	-0,317031863	False	0,317031863
ENSMUSG00000052139	Babam2	329,229313	383,6462569	368,0885097	264,2816748	438,3957972	-0,317016399	False	0,317016399
ENSMUSG00000024654	Asrgl1	170,0245278	39,12182225	136,4921507	68,60077517	39,1424819	-0,316924897	False	0,316924897
ENSMUSG00000028345	Tex10	102,0147167	153,9633005	81,89529044	213,6745456	409,6913105	-0,316924897	False	0,316924897
ENSMUSG00000039515	Ptpa	704,8289517	1137,056834	877,9527373	906,4299145	955,0765582	0,316870092	False	0,316870092
ENSMUSG00000028049	Scamp3	564,1722968	2086,076522	702,7144276	1679,032087	2599,060798	0,316802694	False	0,316802694
ENSMUSG00000030138	Bms1	324,5922804	470,7238613	260,6559782	576,9212731	713,6979199	-0,316481741	False	0,316481741
ENSMUSG00000021610	Clptm1l	1217,221051	2423,028991	1515,50317	1813,984432	2429,443376	0,31620568	False	0,31620568
ENSMUSG00000013662	Atad1	691,6906927	329,3805035	356,6407809	175,438048	861,1346017	0,316111715	False	0,316111715
ENSMUSG00000020069	Hnrnph3	208,666466	176,6791973	259,7753837	196,8055025	168,3126721	0,316065959	False	0,316065959
ENSMUSG00000038252	Ncapd2	41,73329319	131,2474037	51,95507673	168,6904307	181,3601661	0,316065959	False	0,316065959
ENSMUSG00000026664	Phyh	17949,9531	9289,539793	14419,73528	7176,090924	10207,05453	-0,315935395	False	0,315935395
ENSMUSG00000026279	Thap4	193,2096907	155,2252947	140,0145288	121,4571101	142,2176842	-0,315803785	False	0,315803785
ENSMUSG00000019795	Pcmt1	421,9699645	490,9157695	339,0288905	522,9403353	887,2295896	-0,315732094	False	0,315732094
ENSMUSG00000040435	Ppp1r15a	241,125694	471,9858555	193,7307946	339,6300672	1577,44202	-0,315732094	False	0,315732094
ENSMUSG00000029208	Guf1	211,757821	155,2252947	172,5965261	190,0578853	263,5593781	0,315712773	False	0,315712773
ENSMUSG00000023186	Vwa5a	401,1033179	108,5315069	322,2975946	223,7959715	120,0369445	-0,315580479	False	0,315580479
ENSMUSG00000021519	Mterf3	243,4442103	509,8456835	302,9245152	421,7260769	417,5198069	0,315367148	False	0,315367148
ENSMUSG00000033319	Fem1c	208,666466	166,5832431	109,1937206	88,84362686	259,6451299	0,315342397	False	0,315342397
ENSMUSG00000020397	Med7	125,9727183	314,2365723	156,7458247	192,307091	467,100284	0,315315688	False	0,315315688
ENSMUSG00000034858	Fam214a	1673,96876	344,5244347	1345,548428	301,3935696	125,2559421	-0,315078288	False	0,315078288
ENSMUSG00000068011	Mkrn2os	244,217049	143,8673463	303,8051097	112,4602872	105,6847011	0,314982215	False	0,314982215
ENSMUSG00000034390	Cmip	308,3626664	473,2478498	390,1033727	413,8538568	383,5963226	0,314960782	False	0,314960782
ENSMUSG00000026047	Kdelc1	58,73574597	150,1773177	114,4772877	147,3229762	73,0659662	0,314960782	False	0,314960782
ENSMUSG00000023106	Denr	452,1106762	487,1297867	402,431696	281,1507179	562,3469899	0,314784618	False	0,314784618
ENSMUSG00000063576	Klhdc3	616,7253327	1395,765658	766,9978277	889,5608715	1132,522476	0,314594384	False	0,314594384
ENSMUSG00000019935	Slc17a8	135,2467835	5,047977065	168,1935535	26,99046892	10,43799517	0,314528128	False	0,314528128
ENSMUSG00000031540	Kat6a	346,2317657	258,7088246	258,8947891	250,7864404	430,5673008	0,314500665	False	0,314500665
ENSMUSG00000019579	Mydgf	763,5646976	877,086015	1011,803104	614,0331679	1406,519849	-0,314433816	False	0,314433816
ENSMUSG00000026341	Actr3	844,7127677	648,6650528	455,2673673	531,9371583	1050,323264	0,314300661	False	0,314300661
ENSMUSG00000025068	Gsto1	1860,995741	951,5436767	1073,444721	371,1189476	2313,32068	0,313890517	False	0,313890517
ENSMUSG00000029718	Pcolce	110,5159431	63,09971331	137,3727453	34,86268902	65,23746983	0,313841293	False	0,313841293
ENSMUSG00000051346	Spryd4	371,7354449	299,0926411	241,2828987	159,6936078	251,8166335	-0,313683841	False	0,313683841
ENSMUSG00000040297	Suco	163,068979	256,184836	131,2085836	431,8475027	1711,831208	-0,313620257	False	0,313620257
ENSMUSG00000078695	Cisd3	694,4442583	1041,14527	2678,768532	1325,906786	558,4327417	-0,313571275	False	0,313571275
ENSMUSG00000025255	Zfhx4	344,6860882	114,8414782	277,3872741	201,303914	70,45646741	-0,31338147	False	0,31338147
ENSMUSG00000042790	Rnf214	275,1305996	186,7751514	257,1336001	341,879273	41,75198069	0,313370357	False	0,313370357
ENSMUSG00000034714	Ttyh2	803,7523133	469,461867	998,5941867	368,8697419	207,455154	0,313147523	False	0,313147523
ENSMUSG00000035620	Ric8b	155,3405913	177,9411915	125,044422	196,8055025	289,654366	-0,31299416	False	0,31299416
ENSMUSG00000074656	Eif2s2	1166,213693	2390,21714	1448,577987	1586,814652	2336,806169	0,312805189	False	0,312805189
ENSMUSG00000073468	Sft2d1	231,8516288	133,7713922	186,6860384	62,97776081	170,9221709	-0,312587825	False	0,312587825
ENSMUSG00000026584	Scyl3	353,1873146	743,3146228	284,4320302	849,0751681	554,5184935	-0,312349589	False	0,312349589
ENSMUSG00000024382	Ercc3	200,1652396	266,2807902	264,1783563	248,5372346	287,0448672	0,312270546	False	0,312270546
ENSMUSG00000029084	Cd38	190,1183356	391,2182225	235,9993316	372,2435505	356,1965852	0,311885096	False	0,311885096
ENSMUSG00000001569	Nom1	197,8467233	323,0705321	159,3876083	232,7927944	391,424819	-0,311843702	False	0,311843702
ENSMUSG00000041712	Ubr7	195,528207	148,9153234	123,2832329	115,8340958	242,6833878	0,31169861	False	0,31169861
ENSMUSG00000071454	Dtnb	262,7651794	171,6312202	318,7752165	326,1348328	159,1794264	0,311694428	False	0,311694428
ENSMUSG00000041879	Ipo9	356,2786697	326,8565149	287,0738138	393,6110051	420,1293057	-0,311584375	False	0,311584375
ENSMUSG00000048280	Zfp738	119,0171695	95,91156423	74,85053427	79,84680388	61,32322164	-0,311393033	False	0,311393033
ENSMUSG00000038774	Ascc3	359,3700247	108,5315069	101,2683699	193,4316939	289,654366	-0,311137666	False	0,311137666

ENSMUSG00000032231	Anxa2	130,6097509	98,43555276	162,0293918	96,71584696	135,6939372	0,310992931	False	0,310992931
ENSMUSG00000030615	Tmem126a	383,3280264	402,5761709	475,5210413	169,8150336	439,7005466	0,310929698	False	0,310929698
ENSMUSG00000052144	Ppp4r2	265,0836956	227,1589679	159,3876083	230,5435887	328,7968479	0,310748531	False	0,310748531
ENSMUSG00000047767	Atg16l2	75,73819875	266,2807902	139,1339343	784,9728044	93,94195655	0,310748531	False	0,310748531
ENSMUSG00000026117	Zap70	174,6615604	50,47977065	216,6262521	39,36110051	70,45646741	0,310645954	False	0,310645954
ENSMUSG00000050856	Atp5k	691,6906927	446,7459702	857,6990633	319,3872155	538,8615008	0,310344501	False	0,310344501
ENSMUSG00000028189	Ctbs	128,2912346	126,1994266	144,4175014	103,4634642	180,0554167	-0,310301201	False	0,310301201
ENSMUSG00000022369	Mtbp	26,27651794	335,6904748	32,58199727	244,0388231	628,8892091	0,310300936	False	0,310300936
ENSMUSG00000020818	Mfsd11	121,3356858	166,5832431	235,9993316	512,8189095	97,85620474	-0,310268685	False	0,310268685
ENSMUSG00000026201	Stk16	830,80167	584,3033452	989,7882414	1030,13623	700,6504259	0,310259118	False	0,310259118
ENSMUSG00000004393	Ddx56	306,8169888	535,0855688	380,416833	815,3370819	767,1926451	0,310202716	False	0,310202716
ENSMUSG00000030706	Mrpl48	291,3602136	330,6424977	341,6706741	235,0420002	356,1965852	-0,309885307	False	0,309885307
ENSMUSG00000022774	Ncbp2	230,3059513	66,88569611	185,8054439	40,48570338	79,58971319	-0,30975892	False	0,30975892
ENSMUSG00000013846	St3gal1	2077,390594	1964,925072	2574,858379	780,4743929	1095,989493	0,309720585	False	0,309720585
ENSMUSG00000004655	Aqp1	794,4782482	261,2328131	464,953907	641,0236368	304,0066094	-0,309630166	False	0,309630166
ENSMUSG00000067367	Lyar	149,93072	374,812297	185,8054439	191,1824882	772,4116427	0,309496758	False	0,309496758
ENSMUSG00000034908	Sidt2	2264,417575	3633,281492	1827,233631	5188,91765	5066,341907	-0,309478917	False	0,309478917
ENSMUSG00000079562	Maea	444,3822886	453,0559415	267,7007343	352,0006988	550,6042453	0,309214416	False	0,309214416
ENSMUSG00000018040	Rrp7a	296,7700849	382,3842626	239,5217097	273,2784978	454,05279	-0,309189248	False	0,309189248
ENSMUSG00000025358	Cdk2	148,3850425	179,2031858	119,7608548	166,441225	197,0171589	-0,309189248	False	0,309189248
ENSMUSG00000027488	Snta1	238,8071777	138,8193693	295,879759	159,6936078	159,1794264	0,309164806	False	0,309164806
ENSMUSG00000020265	Sumo3	743,4708898	604,4952535	511,6254166	365,4959333	921,1530739	0,309164663	False	0,309164663
ENSMUSG00000034203	Chchd4	95,83200659	99,69754702	72,20875071	68,60077517	118,7321951	0,309131703	False	0,309131703
ENSMUSG00000042626	Shc1	811,4807009	844,274164	655,1623235	797,343436	1527,861543	-0,30870439	False	0,30870439
ENSMUSG00000042770	Hebp1	2459,172943	140,0813635	1985,740645	399,2340194	296,178113	-0,308495991	False	0,308495991
ENSMUSG00000036941	Elac1	151,4763975	124,9374323	187,5666329	168,6904307	106,9894505	0,308310187	False	0,308310187
ENSMUSG00000029499	Pxmp2	1467,620811	215,8010195	1185,280225	394,7356079	276,6068721	-0,308251084	False	0,308251084
ENSMUSG00000066036	Ubr4	1784,484703	1441,197452	1758,547258	2789,015122	1827,953905	-0,308239528	False	0,308239528
ENSMUSG00000021751	Acox2	2649,291279	3279,923098	1289,190379	2552,848519	2008,009321	0,308055518	False	0,308055518
ENSMUSG00000023004	Tuba1b	164,6146565	46,69378785	132,9697726	24,74126318	36,5329831	-0,30799447	False	0,30799447
ENSMUSG00000041747	Utp15	107,424588	194,347117	132,9697726	179,9364595	335,3205949	0,307774078	False	0,307774078
ENSMUSG00000028609	Magoh	238,0343389	641,0930872	339,9094851	192,307091	1328,234886	-0,307757751	False	0,307757751
ENSMUSG00000030265	Kras	469,113129	526,251609	310,8498659	317,1380098	580,6134814	0,307642193	False	0,307642193
ENSMUSG00000002017	Fam98a	321,5009253	199,395094	259,7753837	140,575359	249,2071347	-0,307558163	False	0,307558163
ENSMUSG00000053907	Mat2a	917,3596114	1002,023447	355,7601864	617,4069765	1135,131975	0,307300744	False	0,307300744
ENSMUSG00000042133	Ppig	683,1894663	1889,205416	845,37074	1511,466259	1946,6861	0,307298448	False	0,307298448
ENSMUSG00000001786	Fbxo7	265,8565344	271,3287672	214,8650631	325,0102299	387,5105708	-0,307217006	False	0,307217006
ENSMUSG00000022139	Mbnl2	712,5573393	632,2591273	575,9088166	1072,87114	1049,018515	-0,307165704	False	0,307165704
ENSMUSG00000041697	Cox6a1	3225,828996	2566,896337	3918,645618	629,7776081	3991,228404	0,30716288	False	0,30716288
ENSMUSG00000027210	Meis2	90,42213525	136,2953807	73,08934523	238,4158088	83,50396138	-0,307014877	False	0,307014877
ENSMUSG00000032860	P2ry2	114,3801369	749,6245941	92,46242469	314,8888041	919,8483245	-0,306897437	False	0,306897437
ENSMUSG00000063787	Chchd1	252,7182754	140,0813635	204,2979288	105,7126699	165,7031734	-0,306855419	False	0,306855419
ENSMUSG00000035726	Supt16	285,1775035	410,1481365	280,0290576	230,5435887	510,157014	-0,306820632	False	0,306820632
ENSMUSG00000021645	Smn1	83,46658638	140,0813635	91,58183017	67,4761723	429,2625515	-0,306820632	False	0,306820632
ENSMUSG00000029470	P2rx4	678,5524337	986,8795161	548,6103865	1095,363197	892,4485872	-0,306678365	False	0,306678365
ENSMUSG00000024370	Cdc23	183,9356255	124,9374323	129,4473946	69,72537804	148,7414312	-0,306394376	False	0,306394376
ENSMUSG00000026158	Ogfrl1	234,9429839	206,9670596	284,4320302	190,0578853	280,5211202	-0,305871803	False	0,305871803
ENSMUSG00000007041	Clic1	290,5873748	193,0851227	235,1187371	105,7126699	156,5699276	-0,305582509	False	0,305582509
ENSMUSG00000040859	Bsdc1	697,8734028	513,6316663	367,2079152	459,9625745	862,4393511	0,305457657	False	0,305457657
ENSMUSG00000028030	Tbck	210,2121435	209,4910482	259,7753837	149,5721819	104,3799517	0,305418714	False	0,305418714
ENSMUSG00000038069	Cdkn2aip	187,0269806	113,579484	52,83567125	56,23014358	151,35093	-0,305348869	False	0,305348869
ENSMUSG00000027782	Kpna4	556,4439092	526,251609	243,9246823	210,300737	687,602932	0,305339428	False	0,305339428
ENSMUSG00000035671	Zswim4	136,0196223	278,9007328	110,0743151	309,2657897	390,1200696	-0,305336923	False	0,305336923
ENSMUSG00000022529	Zfp263	149,93072	164,0592546	203,4173343	300,2689667	121,3416939	-0,305220658	False	0,305220658
ENSMUSG00000027508	Pag1	149,93072	59,31373051	104,790748	98,9650527	121,3416939	-0,305220658	False	0,305220658
ENSMUSG00000039205	Ciz1	530,1673913	714,2887546	811,9081482	769,2283642	654,984197	0,305012161	False	0,305012161
ENSMUSG00000019996	Map7	453,6563538	978,0455563	367,2079152	1349,523446	2107,170275	-0,305002708	False	0,305002708
ENSMUSG00000020261	Slc36a1	571,1278457	258,7088246	462,3121234	185,5594738	243,9881371	-0,30494653	False	0,30494653
ENSMUSG00000029635	Cdk8	452,1106762	365,9783372	396,2675344	820,9600963	485,3667755	-0,30491773	False	0,30491773
ENSMUSG00000032409	Atr	95,05916782	199,395094	133,8503672	327,2594356	117,4274457	0,304871964	False	0,304871964
ENSMUSG00000020387	Jade2	152,2492363	402,5761709	123,2832329	453,2149573	245,2928865	-0,304458391	False	0,304458391
ENSMUSG00000018379	Srsf1	968,3669698	618,3771904	688,6249153	245,163426	784,1543873	-0,304416145	False	0,304416145
ENSMUSG00000027806	Tsc22d2	187,0269806	389,9562282	151,4622576	407,1062395	683,6886838	-0,304288071	False	0,304288071
ENSMUSG00000023944	Hsp90ab1	16111,36969	6798,363112	13047,76902	3557,118883	8033,342034	-0,304276001	False	0,304276001
ENSMUSG00000024259	Slc25a46	533,2587463	291,5206755	184,0442549	236,166603	431,8720502	-0,304231772	False	0,304231772
ENSMUSG00000041567	Serpina12	491,5254531	104,7455241	302,0439207	398,1094166	112,2084481	-0,304101131	False	0,304101131
ENSMUSG00000022122	Ednrb	128,2912346	387,4322397	103,9101535	245,163426	433,1767996	-0,304085971	False	0,304085971
ENSMUSG00000027962	Vcam1	133,701106	55,52774771	103,0295589	55,10554071	108,2941999	-0,304055423	False	0,304055423
ENSMUSG00000048706	Lurap1l	291,3602136	936,3997455	235,9993316	242,9142203	1163,836462	-0,304021111	False	0,304021111
ENSMUSG00000024317	Rnf138	149,93072	188,0371457	133,8503672	121,4571101	287,0448672	-0,303849066	False	0,303849066
ENSMUSG00000039286	Fndc3b	343,1404107	1686,02434	423,5659645	1501,344834	1914,067365	0,303787629	False	0,303787629
ENSMUSG00000025949	Pikfyve	235,7158227	169,1072317	194,6113891	196,8055025	290,9591154	0,303767841	False	0,303767841
ENSMUSG00000042121	Ssh1	183,9356255	288,9966869	254,4918165	341,879273	227,026395	0,303659106	False	0,303659106
ENSMUSG00000015961	Adss	605,90559	490,9157695	541,5656303	625,2791966	791,9828837	-0,303617505	False	0,303617505
ENSMUSG00000042042	Csgalnact2	104,333233	184,2511629	84,537074	151,8213877	318,3588527	-0,303542685	False	0,303542685
ENSMUSG00000051721	Wdcp	129,0640734	84,55361583	103,9101535	104,5880671	43,05673008	-0,303369207	False	0,303369207
ENSMUSG00000027695	Pld1	193,2096907	248,6128704	209,581496	327,2594356	156,5699276	-0,303360316	False	0,303360316
ENSMUSG00000026177	Slc11a1	144,5208486	186,7751514	117,1190713	287,8983351	194,4076601	-0,303301613	False	0,303301613
ENSMUSG00000022999	Lmbr1l	167,7060115	354,6203888	206,9397124	457,7133687	296,178113	0,303276125	False	0,303276125
ENSMUSG00000062480	Acat3	2796,903483	2212,275949	2266,650297	1007,644173	2182,84574	-0,303268643	False	0,303268643
ENSMUSG00000024248	Cox7a2l	1220,312406	739,52864	377,7750494	691,630766	989,0000426	-0,303208045	False	0,303208045
ENSMUSG00000027822	Slc33a1	926,6336766	492,1777638	751,1471263	495,9498664	263,5593781	-0,302903601	False	0,302903601
ENSMUSG00000050541	Adra1b	1683,242825	45,43179358	2076,44188	60,72855507	24,79023853	0,302870176	False	0,302870176
ENSMUSG00000032612	Usp4	1059,561944	629,7351388	761,7142605	859,1965939	451,4432912	-0,302407753	False	0,302407753
ENSMUSG00000010406	Mrpl52	521,6661649	1120,650908	1405,428855	643,2728426	684,9934332	0,302303919	False	0,302303919
ENSMUSG00000014226	Cacybp	605,90559	157,7492833	491,3717426	110,2110814	217,8931492	-0,302278121	False	0,302278121
ENSMUSG00000037262	Kin	112,8344594	210,7530424	139,1339343	148,4475791	168,3126721	0,302266601	False	0,302266601
ENSMUSG00000025245	Lztfl1	157,6591076	97,17355849	83,65647948	87,71902399	127,8654409	-0,302182125	False	0,302182125
ENSMUSG00000005621	Zfp592	479,9328717	654,9750241	591,759518	989,650527	761,9736476	0,302178376	False	0,302178376
ENSMUSG00000038342	Mlxip	141,4294936	262,4948074	174,3577151	638,7744311	289,654366	0,301967193	False	0,301967193
ENSMUSG00000047675	Rps8	2582,827145	3398,550559	4361,584662	2095,13515	3494,118884	-0,301907785	False	0,301907785
ENSMUSG00000002015	Bcap31	1981,558588	954,0676652	1353,473779	407,1062395	1607,451257	-0,301860633	False	0,301860633
ENSMUSG00000035696	Rnf38	157,6591076	194,347117	214,8650631	266,5308806	251,8166335	0,301827193	False	0,301827193
ENSMUSG00000029767	Calu	1343,19377	945,2337053	820,7140934	589,2919047	1655,726984	0,30179736	False	0,30179736
ENSMUSG00000025794	Rpl14	1839,356255	3353,118765	3842,914489	1492,348011	2674,736263	-0,301616928	False	0,301616928
ENSMUSG00000042978	Sbk1	499,2538408	93,38757569	405,0734796	88,84362686	136,9986866	-0,301589892	False	0,301589892
ENSMUSG00000050043	Tmx2	378,6909938	143,8673463	466,7150961	115,8340958	97,85620474	0,301521022	False	0,301521022
ENSMUSG00000050335	Lgals3	75,73819875	122,4134438	93,34301921	148,4475791	238,7691396	0,301521022	False	0,301521022
ENSMUSG00000026643	Nmt2	398,7848016	339,4764576	362,8049426	491,4514549	331,4063467	0,301438488	False	0,301438488
ENSMUSG00000053062	Jam2	139,8838161	171,6312202	162,0293918	432,9721056	113,5131975	-0,301369018	False	0,301369018
ENSMUSG00000022682	Rrn3	190,1183356	495,9637466	234,2381425	309,2657897	412,3008093	0,301078342	False	0,301078342
ENSMUSG00000026698	Pigc	129,0640734	159,0112775	150,5816631	116,9586986	39,1424819	0,301041625	False	0,301041625
ENSMUSG00000020530	Ggnbp2	1037,922458	2859,679007	1278,623244	4226,257592	2707,354998	0,30089256	False	0,30089256
ENSMUSG00000029319	Coq2	298,3157624	218,325008	242,1634932	236,166603	187,8839131	-0,300858818	False	0,300858818
ENSMUSG00000062963	Ufc1	704,8289517	1710,002231	1239,877085	868,1934169	1460,014575	0,300743297	False	0,300743297
ENSMUSG00000090035	Galnt4	138,3381385	112,3174897	106,551937	47,23332061	99,16095413	-0,300616352	False	0,300616352
ENSMUSG00000031922	Cep57	139,8838161	152,7013062	113,5966932	231,6681916	260,9498793	-0,30030822	False	0,30030822
ENSMUSG00000031976	Urb2	72,6468437	156,487289	58,9998329	74,22378953	190,4934119	-0,30018925	False	0,30018925
ENSMUSG00000041471	Fam35a	307,5898276	378,5982798	191,089011	338,5054644	56,10422405	0,299660064	False	0,299660064
ENSMUSG00000020440	Arf5	1012,418779	1178,702645	1139,48931	340,7546701	1246,035674	0,2995392	False	0,2995392
ENSMUSG00000036097	Slf2	178,5257542	116,1034725	113,5966932	145,0737704	144,827183	-0,299345512	False	0,299345512
ENSMUSG00000028132	Tmem56	2135,353502	988,1415104	882,3557099	1735,262231	1332,149134	-0,299321226	False	0,299321226
ENSMUSG00000031950	Gabarapl2	173,8887216	124,9374323	125,044422	71,97458378	213,978901	0,299304187	False	0,299304187

ENSMUSG00000024287	Thoc1	251,9454367	454,3179358	309,9692713	580,2950818	829,8206162	0,299013876	False	0,299013876
ENSMUSG00000031168	Ebp	3567,423729	1079,005098	2900,678352	375,6173591	1385,643859	-0,298492258	False	0,298492258
ENSMUSG00000028690	Mmachc	180,8442705	75,71965597	147,059285	98,9650527	31,31398552	-0,298350016	False	0,298350016
ENSMUSG00000032092	Mpzl2	553,3525542	32,81185092	449,9838002	80,97140676	49,58047707	-0,298325885	False	0,298325885
ENSMUSG00000020810	Cygb	193,9825295	157,7492833	282,6708412	212,5499427	176,1411685	-0,298293275	False	0,298293275
ENSMUSG00000028944	Prkag2	585,8117822	123,6754381	476,4016358	325,0102299	60,01847224	-0,298258843	False	0,298258843
ENSMUSG00000058672	Tubb2a	2051,114076	1670,880408	769,6396112	543,183187	2522,080583	0,29820664	False	0,29820664
ENSMUSG00000032178	Ilf3	207,8936272	376,0742913	169,074148	204,6777226	298,7876118	-0,29818945	False	0,29818945
ENSMUSG00000032217	Rnf111	282,0861484	329,3805035	284,4320302	229,4189858	435,7862984	-0,298151037	False	0,298151037
ENSMUSG00000039660	Spout1	159,2047851	223,3729851	208,7009014	316,0134069	195,7124095	0,297851537	False	0,297851537
ENSMUSG00000024817	Uhrf2	353,9601534	460,6279071	287,9544083	508,320498	373,1583274	-0,297746551	False	0,297746551
ENSMUSG00000025178	Pi4k2a	923,5423215	665,0709783	940,4749483	818,7108905	1135,131975	0,297610061	False	0,297610061
ENSMUSG00000023104	Rfc2	256,5824693	323,0705321	259,7753837	473,457809	208,7599034	-0,297577963	False	0,297577963
ENSMUSG00000021537	Cetn3	192,4368519	141,3433578	117,9996658	79,84680388	156,5699276	-0,297577963	False	0,297577963
ENSMUSG00000026172	Bcs1l	245,7627266	78,2436445	302,0439207	110,2110814	32,61873491	0,297492222	False	0,297492222
ENSMUSG00000003031	Cdkn1b	299,86144	152,7013062	169,9547425	136,0769475	243,9881371	-0,297485009	False	0,297485009
ENSMUSG00000032905	Atg12	323,8194416	199,395094	134,7309617	146,1983733	397,9485659	0,297392372	False	0,297392372
ENSMUSG00000030629	Zfand6	1022,465683	402,5761709	697,4308605	543,183187	1256,473669	0,297328015	False	0,297328015
ENSMUSG00000034120	Srsf2	1839,356255	1520,703091	2236,710083	1496,846422	3321,891964	-0,297274728	False	0,297274728
ENSMUSG00000037613	Tnfrsf23	10,81974268	263,7568016	8,805945209	24,74126318	945,9433125	-0,297116416	False	0,297116416
ENSMUSG00000036721	Zscan12	97,37768411	126,1994266	79,25350688	165,3166221	121,3416939	-0,297116416	False	0,297116416
ENSMUSG00000030619	Eed	164,6146565	194,347117	85,41766852	172,0642394	202,2361565	0,296948158	False	0,296948158
ENSMUSG00000023456	Tpi1	3858,783943	1003,285442	3141,080656	1247,184585	516,680761	-0,29688528	False	0,29688528
ENSMUSG00000063550	Nup98	247,3084041	731,9566744	303,8051097	520,6911296	1401,300852	0,296834868	False	0,296834868
ENSMUSG00000030213	Atf7ip	435,8810622	540,1335459	354,8795919	598,2887277	495,8047707	-0,296604913	False	0,296604913
ENSMUSG00000020211	Sf3a2	302,1799563	918,7318257	576,7894112	371,1189476	1274,74016	0,296473691	False	0,296473691
ENSMUSG00000006519	Cyba	93,5134903	114,8414782	152,3428521	165,3166221	127,8654409	0,296397397	False	0,296397397
ENSMUSG00000056770	Setd3	1184,761823	695,3588406	745,8635592	964,9092638	542,7757489	-0,29613187	False	0,29613187
ENSMUSG00000041995	Zbed3	93,5134903	60,57572477	88,94004661	60,72855507	114,8179469	0,296101755	False	0,296101755
ENSMUSG00000060121	Gemin2	98,92336164	78,2436445	92,46242469	121,4571101	65,23746983	0,296063777	False	0,296063777
ENSMUSG00000027750	Postn	176,2072379	78,2436445	64,28340002	24,74126318	143,5224336	-0,295996927	False	0,295996927
ENSMUSG00000059689	Zfp637	192,4368519	84,55361583	156,7458247	80,97140676	70,45646741	-0,29595809	False	0,29595809
ENSMUSG00000026589	Sec16b	1956,054909	1331,403951	2401,381258	2385,282691	755,4499006	0,295917604	False	0,295917604
ENSMUSG00000022868	Ahsg	195882,1671	51153,67558	240465,707	38712,20465	79062,59443	0,295845102	False	0,295845102
ENSMUSG00000025218	Poll	110,5159431	109,7935012	126,805611	177,6872537	90,02770836	-0,295813507	False	0,295813507
ENSMUSG00000032336	Nptn	474,5230004	205,7050654	386,5809947	183,3102681	255,7308817	-0,295707304	False	0,295707304
ENSMUSG00000038116	Phf20	132,9282672	146,3913349	149,7010685	474,5824118	108,2941999	-0,29569195	False	0,29569195
ENSMUSG00000067225	Cyp2c54	10287,25677	1118,12692	8381,49865	38,23649764	2582,099056	-0,295578188	False	0,295578188
ENSMUSG00000040043	Rbms2	143,7480099	59,31373051	117,1190713	55,10554071	32,61873491	-0,295565964	False	0,295565964
ENSMUSG00000030805	Stx4a	391,056414	326,8565149	479,9240139	343,0038758	117,4274457	0,295429256	False	0,295429256
ENSMUSG00000070697	Utp3	410,377383	376,0742913	234,2381425	185,5594738	503,6332671	0,29542236	False	0,29542236
ENSMUSG00000040681	Hmgn1	626,7722366	299,0926411	510,7448221	375,6173591	227,026395	-0,295338603	False	0,295338603
ENSMUSG00000038880	Mrps34	367,0984123	384,9082512	653,4011345	299,1443639	375,7678262	-0,295324993	False	0,295324993
ENSMUSG00000074749	Kiz	251,1725979	170,3692259	103,9101535	204,6777226	78,28496379	-0,295324993	False	0,295324993
ENSMUSG00000071359	Tbpl1	60,2814235	218,325008	73,96993975	160,8182106	240,073889	0,295225615	False	0,295225615
ENSMUSG00000021147	Wdr37	120,562847	184,2511629	147,9398795	332,88245	266,1688769	0,295225615	False	0,295225615
ENSMUSG00000091780	Sco2	120,562847	152,7013062	147,9398795	310,3903926	217,8931492	0,295225615	False	0,295225615
ENSMUSG00000029759	Pon3	933,5892255	267,5427844	760,833666	291,2721437	399,2533153	-0,295206826	False	0,295206826
ENSMUSG00000027933	Ints3	418,8786094	210,7530424	363,6855371	513,9435123	86,11346017	0,295077588	False	0,295077588
ENSMUSG00000074578	Zfas1	78,82955381	238,5169163	113,5966932	96,71584696	151,35093	0,295015687	False	0,295015687
ENSMUSG00000073982	Rhog	251,1725979	557,8014656	360,163159	308,1411868	610,6227176	0,294912449	False	0,294912449
ENSMUSG00000068290	Ddrgk1	1512,445459	909,8978659	1232,832329	761,3561441	1137,741474	-0,294906517	False	0,294906517
ENSMUSG00000030583	Sipa1l3	181,6171093	71,93367317	222,7904138	97,84044983	127,8654409	0,294787039	False	0,294787039
ENSMUSG00000024493	Lars	279,7676321	728,1706916	228,0739809	718,621235	275,3021227	-0,294727188	False	0,294727188
ENSMUSG00000061273	Mmgt1	288,2688585	188,0371457	176,9994987	119,2079044	353,5870865	0,294650594	False	0,294650594
ENSMUSG00000009927	Rps25	1765,163734	3793,554764	4514,808108	1439,491676	3756,373513	-0,294242566	False	0,294242566
ENSMUSG00000029176	Anapc4	225,6689187	357,1443773	184,0442549	419,4768711	455,3575394	-0,294155015	False	0,294155015
ENSMUSG00000022614	Lmf2	434,3353847	532,5615803	630,5056769	772,6021728	606,7084694	0,294138862	False	0,294138862
ENSMUSG00000067219	Nipal1	728,0141146	446,7459702	555,6551427	593,7903162	276,6068721	-0,294012856	False	0,294012856
ENSMUSG00000043154	Ppp2r3a	366,3255736	382,3842626	370,7302933	778,2251872	298,7876118	-0,294006088	False	0,294006088
ENSMUSG00000031839	Hsbp1	1098,203882	1068,909143	1346,429022	437,4705171	1269,521163	0,293992263	False	0,293992263
ENSMUSG00000021279	Cdc42bpb	1082,747107	1090,363046	1356,115562	2549,47471	883,3153414	-0,293695844	False	0,293695844
ENSMUSG00000039219	Arid4b	306,0441501	531,299586	375,1332659	874,9410341	791,9828837	0,293663411	False	0,293663411
ENSMUSG00000003527	Dgcr14	153,022075	401,3141766	187,5666329	500,4482779	369,2440792	0,293663411	False	0,293663411
ENSMUSG00000035024	Ncapd3	161,5233014	214,5390252	235,1187371	357,6237132	131,779689	-0,293614276	False	0,293614276
ENSMUSG00000026029	Casp8	265,8565344	1072,695126	325,8199727	407,1062395	1869,705885	0,293427117	False	0,293427117
ENSMUSG00000026944	Abca2	1197,127244	478,2958269	1467,070472	623,0299909	383,5963226	0,293361668	False	0,293361668
ENSMUSG00000022992	Kansl2	146,0665262	146,3913349	128,5668	119,2079044	208,7599034	-0,293145695	False	0,293145695
ENSMUSG00000022812	Gsk3b	403,4218342	1132,008857	329,3423508	470,0840003	1830,563403	-0,292701125	False	0,292701125
ENSMUSG00000037966	Ninj1	2020,200526	795,0563877	1649,353538	573,5474645	876,7915945	-0,29259783	False	0,29259783
ENSMUSG00000018761	Mpdu1	589,675976	460,6279071	348,7154303	305,8919811	481,4525273	-0,292528868	False	0,292528868
ENSMUSG00000022710	Usp7	927,4065154	901,063906	757,3112879	1514,840068	1399,996102	-0,29231543	False	0,29231543
ENSMUSG00000018669	Cdk5rap3	738,0610185	737,0046514	1076,086504	1634,047972	602,7942212	-0,292074508	False	0,292074508
ENSMUSG00000034518	Hmgxb4	119,7900082	40,38381652	88,94004661	97,84044983	19,57124095	-0,292004635	False	0,292004635
ENSMUSG00000022538	Lsg1	256,5824693	335,6904748	209,581496	417,2276654	537,5567514	-0,291911257	False	0,291911257
ENSMUSG00000024862	Klc2	132,1554284	307,9266009	330,2229453	582,5442875	161,7889252	0,291877169	False	0,291877169
ENSMUSG00000039844	Rapgef1	548,7155216	519,9416376	448,2226111	671,3879144	812,858874	-0,291842957	False	0,291842957
ENSMUSG00000037017	Zscan21	215,6220148	272,5907615	232,4769535	289,022938	176,1411685	-0,291772342	False	0,291772342
ENSMUSG00000028434	Epb41l4b	1463,756617	383,6462569	1195,847359	669,1387086	259,6451299	-0,29164244	False	0,29164244
ENSMUSG00000020689	Itgb3	41,73329319	191,8231285	51,07448221	241,7896174	74,3707156	0,291403904	False	0,291403904
ENSMUSG00000028207	Asph	505,4365509	153,9633005	412,9988303	229,4189858	163,0936746	-0,291392302	False	0,291392302
ENSMUSG00000063802	Hspbp1	200,9380783	444,2219817	314,3722439	164,1920193	691,5171802	-0,29136698	False	0,29136698
ENSMUSG00000005681	Apoa2	101407,2654	80522,80615	124093,3799	34476,95024	91610,36938	0,291265133	False	0,291265133
ENSMUSG00000031590	Frg1	252,7182754	146,3913349	128,5668	44,98411486	309,225607	0,291129797	False	0,291129797
ENSMUSG00000006589	Aprt	297,5429237	405,1001594	1103,384935	518,4419238	364,0250816	0,290940049	False	0,290940049
ENSMUSG00000002871	Tpra1	341,5947332	613,3292133	758,1918825	1510,341657	279,2163709	-0,2909022	False	0,2909022
ENSMUSG00000037211	Spry1	71,10116618	131,2474037	58,11923838	166,441225	169,6174215	-0,290857425	False	0,290857425
ENSMUSG00000033732	Sf3b3	410,377383	1368,001784	501,9388769	1554,201169	1539,604288	0,29056047	False	0,29056047
ENSMUSG00000025133	Ints4	325,3651191	466,9378785	415,6406138	615,1577708	397,9485659	0,290522383	False	0,290522383
ENSMUSG00000024902	Mrpl11	325,3651191	281,4247213	285,3126248	133,8277417	397,9485659	0,290522383	False	0,290522383
ENSMUSG00000024193	Phf1	482,251388	627,2111503	802,2216085	860,3211968	589,7467272	0,290310117	False	0,290310117
ENSMUSG00000024037	Wdr4	175,4343992	214,5390252	450,8643947	430,7228998	361,4155828	0,290308443	False	0,290308443
ENSMUSG00000023341	Mx2	111,2887818	286,4726984	287,0738138	136,0769475	471,0145321	0,29011451	False	0,29011451
ENSMUSG00000060938	Rpl26	2133,034985	4208,750878	4471,658977	2607,954059	3440,624159	0,290010827	False	0,290010827
ENSMUSG00000015605	Srf	169,2516891	312,974578	234,2381425	206,9269284	348,3680889	0,289951171	False	0,289951171
ENSMUSG00000022962	Gart	402,6489954	292,7826697	492,2523372	345,2530816	213,978901	0,289875322	False	0,289875322
ENSMUSG00000090272	Mndal	227,2145963	102,2215356	218,3874412	277,7769093	151,35093	0,289871161	False	0,289871161
ENSMUSG00000006717	Acot13	636,0463018	159,0112775	777,5649619	141,6999618	130,4749397	0,289831419	False	0,289831419
ENSMUSG00000045973	Slc25a51	604,3599125	4598,707106	738,818803	5239,524779	6577,241708	0,289812614	False	0,289812614
ENSMUSG00000030605	Mfge8	138,3381385	169,1072317	235,9993316	312,6395983	238,7691396	0,289739407	False	0,289739407
ENSMUSG00000042569	Dhrs7b	180,8442705	90,86358716	147,9398795	131,578536	44,36147948	-0,289736885	False	0,289736885
ENSMUSG00000040613	Apobec1	335,4120231	119,8894553	241,2828987	274,4031007	168,3126721	-0,289637621	False	0,289637621
ENSMUSG00000033335	Dnm2	871,7621244	2257,707742	1065,51937	2712,542126	3411,919672	0,289550391	False	0,289550391
ENSMUSG00000039768	Dnajc11	425,8341583	137,557375	520,4313618	454,3395601	74,3707156	0,289416223	False	0,289416223
ENSMUSG00000056091	St3gal5	2218,047249	2710,763684	963,3704058	2295,314461	2523,385333	0,28940925	False	0,28940925
ENSMUSG00000063488	Zkscan7	230,3059513	40,38381652	188,4472275	95,59124409	14,35224336	-0,289391123	False	0,289391123
ENSMUSG00000029064	Gnb1	850,8954778	772,3404909	810,1469592	501,5728807	1039,885269	0,289370534	False	0,289370534
ENSMUSG00000058440	Nrf1	106,6517493	312,974578	130,3279891	191,1824882	309,225607	0,28923932	False	0,28923932
ENSMUSG00000021876	Rnase4	14020,068	2705,715707	17131,08581	6829,713239	3400,176927	0,289123249	False	0,289123249
ENSMUSG00000006301	Tmbim1	563,3994581	415,1961136	442,939044	461,0871774	383,5963226	-0,28911863	False	0,28911863
ENSMUSG00000024120	Lrpprc	994,6434877	411,4101308	414,7600193	157,444402	814,1636234	-0,288860746	False	0,288860746
ENSMUSG00000041870	Ankrd13a	537,8957789	439,1740046	440,2972604	387,9879907	362,7203322	-0,2888488	False	0,2888488
ENSMUSG00000026377	Nifk	248,8540816	142,6053521	165,5517699	107,9618757	304,0066094	0,28880264	False	0,28880264

ENSMUSG00000025369	Smarcc2	1820,035286	1118,12692	1273,339677	634,2760196	1490,023811	-0,288631036	False	0,288631036
ENSMUSG00000022445	Cyp2d26	19082,93473	8277,420392	15624,38858	12397,62206	8530,451554	-0,288483323	False	0,288483323
ENSMUSG00000028560	Usp1	254,263953	199,395094	121,5220439	266,5308806	310,5303564	0,288407363	False	0,288407363
ENSMUSG00000029147	Ppm1g	443,6094498	629,7351388	399,7899125	363,2467275	651,0699489	-0,288340298	False	0,288340298
ENSMUSG00000024982	Zdhhc6	772,065924	632,2591273	399,7899125	535,3109669	501,0237683	-0,288208081	False	0,288208081
ENSMUSG00000085795	Zfp703	98,92336164	106,0075184	81,01469592	85,46981824	185,2744143	-0,288127633	False	0,288127633
ENSMUSG00000041444	Arhgap32	171,5702053	209,4910482	85,41766852	123,7063159	178,7506673	0,288089559	False	0,288089559
ENSMUSG00000041515	Irf8	86,55794143	181,7271743	124,1638274	218,1729571	105,6847011	0,288028454	False	0,288028454
ENSMUSG00000020015	Cdk17	168,4788503	205,7050654	251,850033	323,885627	313,1398552	0,288009823	False	0,288009823
ENSMUSG00000046070	Igfals	2581,281468	164,0592546	2114,307445	182,1856652	101,7704529	-0,287902288	False	0,287902288
ENSMUSG00000023994	Nfya	140,6566548	73,19566744	171,7159316	75,3483924	69,15171802	0,287846085	False	0,287846085
ENSMUSG00000070319	Eif3g	714,1030168	1301,116088	871,7885756	968,2830725	2180,236242	0,287846085	False	0,287846085
ENSMUSG00000002147	Stat6	940,5447743	954,0676652	1008,280726	1148,219532	352,2823371	0,287829973	False	0,287829973
ENSMUSG00000071649	B3gat3	640,6833344	482,0818097	524,8343344	382,3649764	228,3311444	-0,28774936	False	0,28774936
ENSMUSG00000051864	Tbc1d22a	210,2121435	162,7972603	93,34301921	85,46981824	172,2269203	-0,287535349	False	0,287535349
ENSMUSG00000041297	Cdk13	462,1575802	687,786875	378,655644	698,3783833	1223,854934	-0,287498413	False	0,287498413
ENSMUSG00000045427	Hnrnph2	530,94023	866,9900608	435,0136933	456,5887659	1414,348346	-0,287488646	False	0,287488646
ENSMUSG00000045216	Hs6st1	1783,711865	845,5361583	2176,829656	834,4553307	1123,38923	0,287345931	False	0,287345931
ENSMUSG00000028163	Nfkb1	338,5033781	495,9637466	277,3872741	557,8030243	502,3285177	-0,28726863	False	0,28726863
ENSMUSG00000062014	Gmfb	287,4960198	350,834406	182,2830658	233,9173973	337,9300937	0,287248256	False	0,287248256
ENSMUSG00000007613	Tgfbr1	217,9405311	742,0526285	265,9395453	531,9371583	1011,180782	0,287163798	False	0,287163798
ENSMUSG00000032216	Nedd4	2821,634323	1958,615101	2312,441212	1743,134451	1518,728298	-0,287114341	False	0,287114341
ENSMUSG00000034160	Ogt	1310,734542	435,3880218	1074,325315	995,2735414	433,1767996	-0,286944611	False	0,286944611
ENSMUSG00000034621	Gpatch8	327,6836354	436,6500161	399,7899125	660,1418856	431,8720502	0,286938444	False	0,286938444
ENSMUSG00000033955	Tnks1bp1	681,6437888	1966,187067	737,9382085	558,9276272	3505,861628	-0,28635653	False	0,28635653
ENSMUSG00000056629	Fkbp2	1407,339387	2550,490412	3178,065626	1716,143982	2439,881371	0,286200314	False	0,286200314
ENSMUSG00000015932	Dstn	1978,467233	1378,097739	926,3854359	584,7934932	2412,481634	0,286134788	False	0,286134788
ENSMUSG00000035478	Mbd3	595,0858474	355,8823831	725,6098852	378,9911677	212,6741516	0,286096303	False	0,286096303
ENSMUSG00000040407	Akap9	421,9699645	595,6612936	346,0736467	934,5449863	848,0871077	-0,286061227	False	0,286061227
ENSMUSG00000033628	Pik3c3	217,9405311	95,91156423	59,88042742	119,2079044	178,7506673	-0,285985896	False	0,285985896
ENSMUSG00000035139	Secisbp2	265,0836956	543,9195287	323,1781892	710,7490149	964,209804	0,285881898	False	0,285881898
ENSMUSG00000028779	Pef1	561,0809418	684,0008922	707,1174002	693,8799718	874,1820957	0,285789297	False	0,285789297
ENSMUSG00000023078	Cxcl13	96,60484535	193,0851227	79,25350688	854,6981824	172,2269203	-0,285620777	False	0,285620777
ENSMUSG00000003344	Btbd2	354,7329921	169,1072317	432,3719097	392,4864022	99,16095413	0,285539282	False	0,285539282
ENSMUSG00000038170	Pde4dip	816,8905723	567,8974198	502,8194714	670,2633115	561,0422405	-0,285414867	False	0,285414867
ENSMUSG00000012117	Dhdds	425,0613195	383,6462569	449,1032056	336,2562586	517,9855104	0,285240762	False	0,285240762
ENSMUSG00000054404	Slfn5	156,8862688	66,88569611	125,9250165	191,1824882	48,27572767	0,285231293	False	0,285231293
ENSMUSG00000020205	Phlda1	1012,418779	474,5098441	1233,712924	287,8983351	345,7585901	0,285200555	False	0,285200555
ENSMUSG00000055762	Eef1d	948,273162	1337,713922	1349,9514	778,2251872	1119,474982	-0,285115032	False	0,285115032
ENSMUSG00000022220	Adcy4	55,64439092	127,4614209	125,044422	348,6268902	45,66622888	-0,285108611	False	0,285108611
ENSMUSG00000079614	Seh1l	473,7501616	297,8306468	201,6561453	165,3166221	388,8153202	-0,28504137	False	0,28504137
ENSMUSG00000033454	Zbtb1	91,19497401	262,4948074	74,85053427	290,1475409	121,3416939	-0,284941702	False	0,284941702
ENSMUSG00000022837	Iqcb1	91,19497401	176,6791973	74,85053427	103,4634642	236,1596408	-0,284941702	False	0,284941702
ENSMUSG00000026856	Dolpp1	268,1750507	151,4393119	326,7005672	219,29756	138,303436	0,284793935	False	0,284793935
ENSMUSG00000037808	Fam76b	143,7480099	147,6533291	117,9996658	139,4507561	361,4155828	-0,284759209	False	0,284759209
ENSMUSG00000028185	Dnase2b	478,3871942	88,33959863	392,7451563	145,0737704	148,7414312	-0,284585288	False	0,284585288
ENSMUSG00000037470	Uggt1	1186,307501	1444,983435	1544,56279	2912,721437	2082,380037	0,284574937	False	0,284574937
ENSMUSG00000042302	Ehbp1	186,2541418	94,64956996	140,8951233	152,9459905	71,76121681	-0,284250221	False	0,284250221
ENSMUSG00000006304	Arpc2	1221,858084	781,1744507	657,8041071	598,2887277	1003,352286	-0,284248491	False	0,284248491
ENSMUSG00000024381	Bin1	189,3454969	315,4985665	305,5662987	230,5435887	396,6438165	0,284018435	False	0,284018435
ENSMUSG00000028089	Chd1l	114,3801369	153,9633005	196,3725782	256,4094547	93,94195655	-0,283994991	False	0,283994991
ENSMUSG00000024462	Gabbr1	57,19006845	109,7935012	115,3578822	257,5340576	46,97097827	-0,283994991	False	0,283994991
ENSMUSG00000018567	Gabarap	3112,221698	4617,63702	4682,121067	2556,222327	5498,213957	-0,283931514	False	0,283931514
ENSMUSG00000025260	Hsd17b10	1331,601188	576,7313796	1621,174513	519,5665267	648,4604501	0,283877336	False	0,283877336
ENSMUSG00000026309	Ilkap	396,4662853	574,2073911	482,5657974	1117,855254	575,3944839	0,283527479	False	0,283527479
ENSMUSG00000083282	Ctsf	275,1305996	87,07760436	99,50718086	226,0451772	96,55145534	-0,283505465	False	0,283505465
ENSMUSG00000013150	Gfod2	115,9258144	199,395094	123,2832329	133,8277417	95,24670595	-0,283460758	False	0,283460758
ENSMUSG00000028653	Trit1	113,6072981	141,3433578	93,34301921	200,1793111	147,4366818	-0,283441479	False	0,283441479
ENSMUSG00000000149	Gna12	736,515341	391,2182225	417,4018029	249,6618375	896,3628354	0,283367258	False	0,283367258
ENSMUSG00000018733	Pex12	160,7504627	135,0333865	132,0891781	190,0578853	189,1886625	-0,283310616	False	0,283310616
ENSMUSG00000044465	Fam160a2	204,8022721	453,0559415	249,2082494	353,1253017	360,1108334	0,283120105	False	0,283120105
ENSMUSG00000073609	D2hgdh	398,7848016	103,4835298	485,207581	391,3617993	45,66622888	0,282991664	False	0,282991664
ENSMUSG00000034928	Rnf44	684,7351438	562,8494427	603,2072468	826,5831106	884,6200908	-0,282796995	False	0,282796995
ENSMUSG00000031871	Cdh5	277,4491158	323,0705321	228,0739809	269,9046892	304,0066094	-0,282721335	False	0,282721335
ENSMUSG00000059263	Usp47	975,3225187	1022,215356	619,9385427	1186,45603	1049,018515	0,282707363	False	0,282707363
ENSMUSG00000025157	Zdhhc16	176,9800767	349,5724117	260,6559782	360,9975218	215,2836504	0,282651799	False	0,282651799
ENSMUSG00000016427	Ndufa1	410,377383	668,8569611	770,5202057	337,3808615	600,1847224	-0,282573086	False	0,282573086
ENSMUSG00000024764	Naa40	125,9727183	210,7530424	153,2234466	215,9237514	267,4736263	0,282525753	False	0,282525753
ENSMUSG00000035637	Grhpr	1366,378933	1828,629692	1661,681861	2245,831935	1971,476338	0,28228656	False	0,28228656
ENSMUSG00000037818	Abhd18	385,6465426	140,0813635	286,1932193	317,1380098	75,675465	-0,282168379	False	0,282168379
ENSMUSG00000035041	Creb3l3	3052,713151	6134,554128	2510,574979	7767,632034	6848,629582	-0,282074203	False	0,282074203
ENSMUSG00000024359	Hspa9	5337,997335	3218,085379	2168,904305	2171,608145	4390,481719	-0,281919346	False	0,281919346
ENSMUSG00000053347	Zfp943	212,5306598	137,557375	121,5220439	97,84044983	174,8364191	-0,281665245	False	0,281665245
ENSMUSG00000032114	Slc37a4	1131,435949	393,742211	930,7884085	566,7998473	139,6081854	-0,281629766	False	0,281629766
ENSMUSG00000042207	Kdm5b	197,0738845	277,6387386	239,5217097	301,3935696	279,2163709	0,281419816	False	0,281419816
ENSMUSG00000052214	Opa3	1054,924911	1215,300478	1282,145622	485,8284405	871,5725969	0,281419816	False	0,281419816
ENSMUSG00000032714	Syde1	91,96781277	107,2695126	145,2980959	230,5435887	75,675465	-0,281303395	False	0,281303395
ENSMUSG00000030610	Det1	98,15052288	80,76763303	180,5218768	106,8372728	106,9894505	-0,281218693	False	0,281218693
ENSMUSG00000024293	Esco1	111,2887818	137,557375	91,58183017	92,21743547	155,2651782	-0,281174872	False	0,281174872
ENSMUSG00000020246	Hcfc2	140,6566548	155,2252947	86,29826304	89,96822973	170,9221709	0,281161735	False	0,281161735
ENSMUSG00000036955	Kif1bp	191,6640132	157,7492833	95,98480277	122,581713	83,50396138	-0,280946032	False	0,280946032
ENSMUSG00000034994	Eef2	20416,85444	12313,27805	8587,557767	8174,738274	16805,17223	-0,280855283	False	0,280855283
ENSMUSG00000029426	Scarb2	1631,462628	932,6137627	642,8340002	518,4419238	1981,914333	0,280728664	False	0,280728664
ENSMUSG00000071172	Srsf3	590,4488148	531,299586	486,0881755	493,7006606	1350,415625	-0,28059396	False	0,28059396
ENSMUSG00000040123	Zmym5	385,6465426	694,0968464	531,8790906	937,9187949	468,4050333	0,280477402	False	0,280477402
ENSMUSG00000026843	Fubp3	220,2590474	175,417203	108,3131261	84,34521537	181,3601661	-0,280343663	False	0,280343663
ENSMUSG00000020009	Ifngr1	176,2072379	146,3913349	129,4473946	179,9364595	213,978901	0,280195364	False	0,280195364
ENSMUSG00000035944	Ttc38	1081,201629	686,5248808	1312,966431	1286,545685	227,026395	0,280194706	False	0,280194706
ENSMUSG00000032120	C2cd2l	505,4365509	230,9449507	613,774381	391,3617993	241,3786384	0,280178432	False	0,280178432
ENSMUSG00000020482	Ccdc117	158,4319464	53,00375918	83,65647948	51,73173209	130,4749397	-0,280090537	False	0,280090537
ENSMUSG00000040524	Zfp609	210,9849822	256,184836	135,6115562	232,7927944	157,874677	0,28004477	False	0,28004477
ENSMUSG00000018593	Sparc	872,5349632	694,0968464	1059,355209	683,7585459	636,7177055	0,279901568	False	0,279901568
ENSMUSG00000042502	Cd2bp2	259,6738243	363,4543486	315,2528385	410,4800481	487,9762743	0,279808766	False	0,279808766
ENSMUSG00000021775	Nr1d2	521,6661649	682,738898	429,7301262	797,343436	720,2216669	-0,279695943	False	0,279695943
ENSMUSG00000027800	Tm4sf1	164,6146565	45,43179358	135,6115562	92,21743547	36,5329831	-0,279612668	False	0,279612668
ENSMUSG00000025195	Dnmbp	530,1673913	1085,315069	436,7748823	1322,532977	2434,662374	-0,279558044	False	0,279558044
ENSMUSG00000031513	Leprotl1	236,4886614	217,0630138	171,7159316	119,2079044	287,0448672	0,279505244	False	0,279505244
ENSMUSG00000037447	Arid5a	35,55058309	1357,90583	43,14913152	461,0871774	1837,08715	0,279458299	False	0,279458299
ENSMUSG00000002058	Unc119	71,10116618	121,1514495	86,29826304	282,2753208	114,8179469	0,279458299	False	0,279458299
ENSMUSG00000026083	Eif5b	1450,618358	2527,774515	1894,158814	3692,071228	1760,106936	0,278995071	False	0,278995071
ENSMUSG00000029598	Plbd2	813,0263785	570,4214083	696,550266	986,2767184	947,2480619	0,278690318	False	0,278690318
ENSMUSG00000029106	Add1	608,2241063	280,1627271	596,1624906	737,7394838	117,4274457	0,278508453	False	0,278508453
ENSMUSG00000037239	Spred3	23,95800165	198,1330998	29,05961919	290,1475409	186,5791637	0,27850822	False	0,27850822
ENSMUSG00000027618	Nfs1	400,3304791	238,5169163	237,7605206	176,5626508	330,1015973	-0,27828134	False	0,27828134
ENSMUSG00000042605	Atxn2	1332,374027	1087,839057	1008,280726	1098,737006	417,5198069	-0,278153033	False	0,278153033
ENSMUSG00000060519	Tor3a	103,5603942	249,8748647	85,41766852	316,0134069	356,1965852	-0,277865937	False	0,277865937
ENSMUSG00000026032	Ndufb3	326,1379579	233,4689392	395,3869399	105,7126699	193,1029107	0,277782857	False	0,277782857
ENSMUSG00000025854	Fam20c	660,7771422	281,4247213	545,0880084	330,6332443	157,874677	-0,277674599	False	0,277674599
ENSMUSG00000059518	Znhit1	713,3301781	1060,075184	1379,891614	639,899034	588,4419778	-0,277669841	False	0,277669841
ENSMUSG00000053470	Kdm3a	305,2713113	673,9049381	251,850033	918,8005461	898,9723342	-0,277527095	False	0,277527095
ENSMUSG00000026275	Ppp1r7	342,3675719	381,1222684	276,5066795	131,578536	414,9103081	0,27725344	False	0,27725344
ENSMUSG00000066735	Vkorc1l1	398,0119628	249,8748647	328,4617563	274,4031007	167,0079228	-0,27708639	False	0,27708639
ENSMUSG00000028057	Rit1	165,3874952	294,044664	136,4921507	166,441225	348,3680889	-0,27703217	False	0,27703217

ENSMUSG00000036427	Gpi1	2950,698396	2602,232177	3575,213755	2254,828758	2659,07927	0,276973036	False	0,276973036
ENSMUSG00000027222	Pex16	673,1425624	883,3959863	555,6551427	627,5284024	1430,005339	-0,276722302	False	0,276722302
ENSMUSG00000036620	Mgat4b	454,4291925	325,5945207	375,1332659	327,2594356	294,8733636	-0,276652313	False	0,276652313
ENSMUSG00000061887	Ssbp3	1018,601489	449,2699587	1233,712924	602,7871392	335,3205949	0,276416997	False	0,276416997
ENSMUSG00000029701	Rbm28	350,0959595	424,0300734	666,6100523	779,34979	477,5382791	0,276416177	False	0,276416177
ENSMUSG00000023150	Ivns1abp	999,2805203	1210,252501	1314,72762	6541,814904	1347,806127	0,276346439	False	0,276346439
ENSMUSG00000027080	Med19	178,5257542	162,7972603	281,7902467	235,0420002	147,4366818	-0,276036706	False	0,276036706
ENSMUSG00000009291	Pttg1ip	3109,130343	1755,434024	1909,128921	1718,393188	2567,746812	-0,276008144	False	0,276008144
ENSMUSG00000005846	Rsl1d1	571,9006845	715,5507489	495,7747152	472,3332061	1324,320637	-0,275959668	False	0,275959668
ENSMUSG00000022940	Pigp	153,022075	219,5870023	361,0437536	318,2626127	185,2744143	0,275923873	False	0,275923873
ENSMUSG00000008604	Ubqln4	824,6189599	942,7097168	826,8782551	1431,619456	681,079185	-0,275905091	False	0,275905091
ENSMUSG00000037935	Smarce1	387,9650589	374,812297	280,9096522	277,7769093	469,7097827	0,275842916	False	0,275842916
ENSMUSG00000025981	Coq10b	91,96781277	190,5611342	135,6115562	111,3356843	240,073889	0,275715131	False	0,275715131
ENSMUSG00000008429	Herpud2	516,2562936	407,624148	245,6858713	254,160249	624,9749609	0,275710926	False	0,275710926
ENSMUSG00000023019	Gpd1	4760,686779	1770,577955	3932,73513	2883,481763	1222,550185	-0,275636688	False	0,275636688
ENSMUSG00000047731	Wbp1l	3342,527649	2761,243454	1465,309283	2647,31516	1898,410372	-0,275621401	False	0,275621401
ENSMUSG00000022136	Dnajc3	3298,47584	8427,597709	3992,615558	4240,877429	8193,82621	0,275534626	False	0,275534626
ENSMUSG00000032607	Amt	837,7572189	11,3579484	692,1472934	67,4761723	40,44723129	-0,275453128	False	0,275453128
ENSMUSG00000033697	Arhgap39	65,69129484	79,50563877	108,3131261	201,303914	199,6266577	0,275354981	False	0,275354981
ENSMUSG00000049504	Proser1	136,792461	378,5982798	165,5517699	240,6650145	1098,598992	0,275293713	False	0,275293713
ENSMUSG00000045128	Rpl18a	5091,461769	11553,55751	8570,826471	6161,699134	11835,38178	0,275248323	False	0,275248323
ENSMUSG00000042203	Tbc1d22b	126,7455571	104,7455241	176,9994987	128,2047274	186,5791637	-0,275046579	False	0,275046579
ENSMUSG00000063200	Nol7	548,7155216	210,7530424	453,5061782	247,4126318	225,7216456	-0,274936181	False	0,274936181
ENSMUSG00000049305	Ccdc71	237,2615002	222,1109908	160,2682028	130,4539331	287,0448672	0,274798241	False	0,274798241
ENSMUSG00000096546	Smlr1	274,3577608	331,904492	291,4767864	119,2079044	129,1701903	0,274709769	False	0,274709769
ENSMUSG00000039828	Wdr70	118,2443307	135,0333865	97,74599182	136,0769475	238,7691396	-0,274661565	False	0,274661565
ENSMUSG00000001687	Ubl3	692,4635315	627,2111503	443,8196385	423,9752826	837,6491126	0,274607938	False	0,274607938
ENSMUSG00000032842	Abcc10	225,6689187	776,1264737	884,9974935	2383,033485	186,5791637	-0,274419849	False	0,274419849
ENSMUSG00000039735	Fnbp1l	238,0343389	188,0371457	154,9846357	196,8055025	131,779689	-0,274399154	False	0,274399154
ENSMUSG00000053819	Camk2d	87,3307802	172,8932145	72,20875071	118,0833015	186,5791637	-0,274316546	False	0,274316546
ENSMUSG00000026111	Unc50	212,5306598	135,0333865	86,29826304	69,72537804	257,0356311	0,274297384	False	0,274297384
ENSMUSG00000038058	Nod1	189,3454969	228,4209622	199,8949562	338,5054644	156,5699276	-0,27421397	False	0,27421397
ENSMUSG00000020456	Ogdh	2626,106116	1309,950048	2171,546088	1791,492374	1025,533026	-0,274202641	False	0,274202641
ENSMUSG00000020357	Flt4	278,9947934	747,1006056	230,7157645	1292,168699	344,4538407	-0,274121614	False	0,274121614
ENSMUSG00000004500	Zfp324	109,7431043	21,45390252	114,4772877	132,7031389	15,65699276	0,274072204	False	0,274072204
ENSMUSG00000020089	Ppa1	1564,225656	482,0818097	1293,593351	313,7642012	656,2889464	-0,274064483	False	0,274064483
ENSMUSG00000029432	Nipsnap2	200,1652396	217,0630138	165,5517699	314,8888041	528,4235056	-0,273909025	False	0,273909025
ENSMUSG00000036040	Adamtsl2	152,2492363	598,1852821	125,9250165	818,7108905	204,8456553	-0,273870071	False	0,273870071
ENSMUSG00000033379	Atp6v0b	536,3501014	970,4735907	879,7139263	1175,210001	648,4604501	0,273843562	False	0,273843562
ENSMUSG00000031729	Ist1	643,0018507	1003,285442	531,8790906	1056,002096	2103,256027	-0,273724567	False	0,273724567
ENSMUSG00000004788	Eif2b2	217,1676923	485,8677925	179,6412823	319,3872155	825,906368	-0,273690567	False	0,273690567
ENSMUSG00000024127	Prepl	304,4984725	153,9633005	222,7904138	251,9110432	153,9604288	-0,273520623	False	0,273520623
ENSMUSG00000020770	Unk	231,8516288	191,8231285	346,9542412	409,3554453	197,0171589	-0,273425183	False	0,273425183
ENSMUSG00000024454	Hdac3	520,1204874	430,3400448	475,5210413	617,4069765	609,3179682	-0,273368772	False	0,273368772
ENSMUSG00000045954	Cavin2	127,5183959	23,97789106	154,1040411	96,71584696	27,39973733	0,273199309	False	0,273199309
ENSMUSG00000057342	Sphk2	1209,492664	619,6391847	737,057614	1000,896556	775,0211415	-0,273109142	False	0,273109142
ENSMUSG00000031661	Nkd1	146,8393649	49,21777638	121,5220439	61,85315794	74,3707156	-0,27302074	False	0,27302074
ENSMUSG00000013465	Nelfb	375,5996387	355,8823831	310,8498659	547,6815985	482,7572767	-0,272977719	False	0,272977719
ENSMUSG00000066258	Trim12a	214,0763373	208,2290539	151,4622576	258,6586605	66,54221922	0,272924159	False	0,272924159
ENSMUSG00000019969	Psen1	564,9451356	931,3517684	467,5956906	641,0236368	2143,703258	-0,272849134	False	0,272849134
ENSMUSG00000020258	Glyctk	2900,463877	1505,55916	3503,885598	2976,823801	644,5462019	0,27267202	False	0,27267202
ENSMUSG00000025498	Irf7	675,4610787	305,4026124	559,1775207	449,8411486	464,4907852	-0,272566276	False	0,272566276
ENSMUSG00000054612	Mgmt	296,7700849	78,2436445	245,6858713	65,22696655	60,01847224	-0,272530778	False	0,272530778
ENSMUSG00000024845	Tmem134	627,5450754	713,0267604	850,6543071	519,5665267	666,7269416	-0,272410601	False	0,272410601
ENSMUSG00000002660	Clpp	588,1302985	825,3442501	1099,862557	486,9530434	767,1926451	-0,272353154	False	0,272353154
ENSMUSG00000033161	Atp1a1	2618,377728	2168,106149	1136,847526	922,1743547	2104,560777	-0,272237844	False	0,272237844
ENSMUSG00000038615	Nfe2l1	1354,786351	527,5136032	1121,87742	690,5061632	538,8615008	-0,272150307	False	0,272150307
ENSMUSG00000022503	Nubp1	217,9405311	546,4435172	339,0288905	263,157072	281,8258696	0,271989642	False	0,271989642
ENSMUSG00000044456	Rin3	474,5230004	572,9453968	313,4916494	339,6300672	424,0435539	0,271919636	False	0,271919636
ENSMUSG00000008540	Mgst1	30340,10415	23204,28857	25170,03319	8509,86993	36632,14406	0,271884106	False	0,271884106
ENSMUSG00000031701	Dnaja2	1128,344594	1081,529086	1015,325483	934,5449863	1838,3919	-0,271871713	False	0,271871713
ENSMUSG00000047631	Apof	8149,584754	2696,881747	9838,882582	1459,734527	2652,555523	0,271767924	False	0,271767924
ENSMUSG00000023045	Soat2	754,2906325	4102,743359	910,5347346	6942,173527	2202,416981	0,271593544	False	0,271593544
ENSMUSG00000004667	Polr2e	250,3997591	257,4468303	339,9094851	264,2816748	207,455154	-0,271433674	False	0,271433674
ENSMUSG00000024856	Cdk2ap2	673,1425624	1020,953361	1219,623411	557,8030243	1788,811423	-0,271156324	False	0,271156324
ENSMUSG00000066148	Prpf4	110,5159431	131,2474037	91,58183017	120,3325073	174,8364191	-0,271121207	False	0,271121207
ENSMUSG00000041777	Cir1	195,528207	223,3729851	162,0293918	608,4101535	370,5488286	-0,271121207	False	0,271121207
ENSMUSG00000069208	Zfp825	222,5775637	213,277031	268,5813289	188,9332824	82,19921198	0,271050842	False	0,271050842
ENSMUSG00000028792	Ak2	2466,901331	1350,333865	1872,143951	391,3617993	2044,542304	-0,270922099	False	0,270922099
ENSMUSG00000020601	Trib2	26,27651794	612,0672191	31,70140275	221,5467657	383,5963226	0,270772572	False	0,270772572
ENSMUSG00000051329	Nup160	92,74065154	152,7013062	111,8355041	193,4316939	206,1504046	0,270104503	False	0,270104503
ENSMUSG00000049439	Cyp20a1	132,1554284	114,8414782	155,8652302	157,444402	109,5989493	-0,270001719	False	0,270001719
ENSMUSG00000027523	Gnas	7781,713503	15181,79102	12511,48695	6454,09588	16111,04555	-0,269872856	False	0,269872856
ENSMUSG00000030824	Nucb1	3105,266149	1962,401084	2575,738974	1852,22093	2523,385333	-0,269730528	False	0,269730528
ENSMUSG00000041168	Lonp1	1532,539267	1043,669258	689,5055098	1185,331427	1847,525145	0,269669961	False	0,269669961
ENSMUSG00000022769	Sdf2l1	496,1624857	710,5027718	779,326151	411,604651	1895,800873	-0,269553374	False	0,269553374
ENSMUSG00000010057	Nprl2	200,1652396	121,1514495	241,2828987	137,2015503	13,04749397	0,269534207	False	0,269534207
ENSMUSG00000012519	Mlkl	94,28632906	78,2436445	125,044422	96,71584696	112,2084481	-0,269075033	False	0,269075033
ENSMUSG00000034066	Farp2	267,4022119	402,5761709	221,9098193	398,1094166	370,5488286	-0,269037893	False	0,269037893
ENSMUSG00000027784	Ppm1l	238,8071777	287,7346927	141,7757179	100,0896556	167,0079228	0,26889298	False	0,26889298
ENSMUSG00000007872	Id3	646,8660445	1135,79484	856,8184688	779,34979	1063,370758	0,268804004	False	0,268804004
ENSMUSG00000024943	Smc5	282,8589872	427,8160562	340,7900796	691,630766	514,0712622	0,268800329	False	0,268800329
ENSMUSG00000033047	Eif3l	1156,939628	892,2299462	661,3264852	905,3053117	960,2955558	-0,268763177	False	0,268763177
ENSMUSG00000026304	Rab17	242,6713715	95,91156423	292,3573809	95,59124409	140,9129348	0,268729094	False	0,268729094
ENSMUSG00000022951	Rcan1	203,2565946	844,274164	244,8052768	339,6300672	587,1372284	0,268332495	False	0,268332495
ENSMUSG00000030269	Mtmr14	319,182409	265,0187959	294,11857	737,7394838	559,7374911	-0,26828646	False	0,26828646
ENSMUSG00000053436	Mapk14	928,9521929	1294,806117	771,4008003	1764,501906	838,953862	-0,26812371	False	0,26812371
ENSMUSG00000036867	Smad6	108,9702656	80,76763303	131,2085836	112,4602872	62,62797103	0,267927579	False	0,267927579
ENSMUSG00000010755	Cars	432,0168684	522,4656262	399,7899125	861,4457997	358,806084	-0,267883291	False	0,267883291
ENSMUSG00000019806	Aig1	190,8911744	270,066773	205,1785234	158,5690049	386,2058214	-0,267639605	False	0,267639605
ENSMUSG00000066551	Hmgb1	1195,581566	1013,381396	921,9824633	864,8196083	1439,138584	-0,267492967	False	0,267492967
ENSMUSG00000037740	Mrps26	219,4862086	275,11475	264,1783563	282,2753208	296,178113	0,267381982	False	0,267381982
ENSMUSG00000029390	Tmed2	844,7127677	1736,50411	701,8338331	779,34979	3109,217812	-0,26733136	False	0,26733136
ENSMUSG00000024067	Dpy30	214,0763373	97,17355849	177,8800932	73,09918666	148,7414312	-0,267220272	False	0,267220272
ENSMUSG00000029524	Sirt4	139,8838161	71,93367317	116,2384768	100,0896556	14,35224336	-0,267141356	False	0,267141356
ENSMUSG00000025217	Btrc	220,2590474	167,8452374	265,0589508	147,3229762	80,89446258	0,267111979	False	0,267111979
ENSMUSG00000042202	Slc35e2	688,5993377	177,9411915	572,3864386	391,3617993	121,3416939	-0,266675302	False	0,266675302
ENSMUSG00000028070	Naxe	745,7894061	334,4284805	619,9385427	177,6872537	254,4261323	-0,266643102	False	0,266643102
ENSMUSG00000051391	Ywhag	768,2017302	638,5690987	606,7296249	338,5054644	558,4327417	-0,266642473	False	0,266642473
ENSMUSG00000045983	Eif4g1	4279,981068	3808,698695	1553,368735	2085,013724	5148,541119	0,266559276	False	0,266559276
ENSMUSG00000047963	Stbd1	416,5600932	180,4651801	501,0582824	80,97140676	39,1424819	0,266453791	False	0,266453791
ENSMUSG00000053332	Gas5	517,0291323	962,9016251	863,863225	621,905388	701,9551753	0,266449544	False	0,266449544
ENSMUSG00000022148	Fyb	118,2443307	95,91156423	77,49231784	116,9586986	142,2176842	0,266329855	False	0,266329855
ENSMUSG00000004849	Ap1s1	333,8663455	239,7789106	401,5511015	195,6808997	170,9221709	0,266312923	False	0,266312923
ENSMUSG00000048100	Taf13	97,37768411	73,19566744	117,1190713	64,10236368	69,15171802	0,266312923	False	0,266312923
ENSMUSG00000041268	Dmxl2	257,355308	329,3805035	330,2229453	757,9823355	213,978901	-0,266292989	False	0,266292989
ENSMUSG00000046185	Zfp84	81,14807009	111,0554954	76,61172331	67,4761723	116,1226963	-0,266178648	False	0,266178648
ENSMUSG00000020614	Fam20a	697,8734028	1731,456133	580,3117892	1345,025034	845,4776089	-0,266137111	False	0,266137111
ENSMUSG00000085208	Brip1os	306,0441501	146,3913349	254,4918165	78,72220101	43,05673008	-0,266120526	False	0,266120526
ENSMUSG00000034190	Chmp7	376,3724775	312,974578	276,5066795	296,8951581	203,5409059	-0,266115658	False	0,266115658
ENSMUSG00000024050	Wiz	316,091054	468,1998727	409,4764522	380,1157706	643,2414525	0,266098678	False	0,266098678
ENSMUSG00000002014	Ssr4	1077,337235	1809,699778	3433,438037	1295,542508	1396,081854	0,26608643	False	0,26608643
ENSMUSG00000033557	Fam20b	260,4466631	167,8452374	125,044422	142,8245647	216,5883998	-0,266031977	False	0,266031977

ENSMUSG00000005299	Letm1	826,9374762	627,2111503	687,7443208	447,5919429	430,5673008	-0,265905932	False	0,265905932
ENSMUSG00000022757	Tfg	1280,59383	2366,239249	1570,980625	1539,581331	5543,880186	0,265725118	False	0,265725118
ENSMUSG00000021022	Ppp2r3c	98,15052288	195,6091113	117,9996658	192,307091	233,550142	0,265714915	False	0,265714915
ENSMUSG00000044221	Grsf1	1005,46323	417,7201021	368,9691042	303,6427753	836,3443632	-0,265691327	False	0,265691327
ENSMUSG00000054823	Nsd3	862,4880593	734,4806629	708,8785893	1036,883848	292,2638648	0,265677903	False	0,265677903
ENSMUSG00000026950	Neb	280,5404709	102,2215356	233,357548	136,0769475	105,6847011	-0,265666777	False	0,265666777
ENSMUSG00000037373	Ctbp1	583,4932659	702,9308062	622,5803262	620,7807851	485,3667755	-0,26564065	False	0,26564065
ENSMUSG00000046027	Stard5	1113,660657	364,7163429	926,3854359	729,8672637	232,2453926	-0,26562522	False	0,26562522
ENSMUSG00000041997	Tlk1	588,1302985	426,554062	312,6110549	444,2181343	489,2810237	-0,265472485	False	0,265472485
ENSMUSG00000026596	Abl2	97,37768411	181,7271743	81,01469592	111,3356843	238,7691396	-0,265407556	False	0,265407556
ENSMUSG00000028062	Lamtor2	706,3746292	190,5611342	848,8931181	114,7094929	228,3311444	0,265149393	False	0,265149393
ENSMUSG00000056919	Cep162	85,78510267	133,7713922	116,2384768	257,5340576	103,0752023	0,264898255	False	0,264898255
ENSMUSG00000063558	Aox1	1373,334482	424,0300734	1143,011688	1103,235417	163,0936746	-0,264842887	False	0,264842887
ENSMUSG00000004040	Stat3	1306,097509	4270,588597	1569,219436	2700,171495	6660,745669	0,264784502	False	0,264784502
ENSMUSG00000002233	Rhoc	142,2023324	437,9120103	170,835337	249,6618375	424,0435539	0,264661297	False	0,264661297
ENSMUSG00000034432	Cops8	298,3157624	324,3325264	248,3276549	269,9046892	775,0211415	-0,264595274	False	0,264595274
ENSMUSG00000030423	Pop4	209,4393047	429,0780505	174,3577151	271,0292921	679,7744356	-0,26448201	False	0,26448201
ENSMUSG00000037348	Paqr7	309,9083439	25,23988532	258,0141946	53,98093784	14,35224336	-0,264391161	False	0,264391161
ENSMUSG00000021392	Nol8	157,6591076	315,4985665	189,327822	350,8760959	392,7295683	0,264077919	False	0,264077919
ENSMUSG00000078429	Ctdsp2	731,8783084	793,7943934	694,789077	609,5347564	1192,540948	-0,2638953	False	0,2638953
ENSMUSG00000025512	Chid1	359,3700247	21,45390252	431,4913152	52,85633497	43,05673008	0,263861444	False	0,263861444
ENSMUSG00000006281	Tep1	244,217049	141,3433578	203,4173343	191,1824882	116,1226963	-0,263721295	False	0,263721295
ENSMUSG00000021650	Ptcd2	419,6514482	237,254922	349,5960248	231,6681916	255,7308817	-0,263502779	False	0,263502779
ENSMUSG00000033653	Vps8	116,6986532	140,0813635	89,82064113	123,7063159	91,33245776	0,263477121	False	0,263477121
ENSMUSG00000021756	Il6st	1843,220449	932,6137627	1535,756844	1409,127398	1389,558107	-0,263278815	False	0,263278815
ENSMUSG00000068921	Dap3	436,653901	1031,049315	523,9537399	1174,085398	1056,847011	0,262949212	False	0,262949212
ENSMUSG00000021635	Rad17	135,2467835	224,6349794	112,7160987	237,2912059	438,3957972	-0,2629007	False	0,2629007
ENSMUSG00000045193	Cirbp	215,6220148	258,7088246	110,0743151	256,4094547	229,6358938	0,262824782	False	0,262824782
ENSMUSG00000023892	Zfp51	79,60239257	121,1514495	73,08934523	66,35156943	144,827183	-0,262681203	False	0,262681203
ENSMUSG00000004980	Hnrnpa2b1	3082,080986	1861,441543	2500,007845	856,9473882	2569,051562	-0,262668925	False	0,262668925
ENSMUSG00000024479	Mal2	616,7253327	387,4322397	472,8792577	240,6650145	514,0712622	-0,262659744	False	0,262659744
ENSMUSG00000039480	Nt5dc1	269,7207282	138,8193693	111,8355041	74,22378953	323,5778503	0,262646457	False	0,262646457
ENSMUSG00000030980	Knop1	436,653901	492,1777638	515,1477947	815,3370819	364,0250816	-0,262452372	False	0,262452372
ENSMUSG00000031586	Rbpms	586,584621	2583,302263	703,5950222	2000,668509	3380,605686	0,262406029	False	0,262406029
ENSMUSG00000071655	Ubxn1	1788,348897	1977,545015	989,7882414	1094,238594	2145,008008	0,262354808	False	0,262354808
ENSMUSG00000030471	Zdhhc13	73,41968247	556,5394714	88,05945209	299,1443639	503,6332671	0,262310993	False	0,262310993
ENSMUSG00000015843	Rxrg	132,1554284	23,97789106	158,5070138	59,6039522	79,58971319	0,262310993	False	0,262310993
ENSMUSG00000044068	Zrsr1	250,3997591	181,7271743	300,2827316	170,9396365	130,4749397	0,262088337	False	0,262088337
ENSMUSG00000027133	Nop10	648,411722	622,1631732	777,5649619	231,6681916	730,659662	0,262053039	False	0,262053039
ENSMUSG00000079563	Pglyrp2	1507,035587	426,554062	1806,979957	421,7260769	649,7651995	0,261867018	False	0,261867018
ENSMUSG00000046034	Otulin	432,0168684	360,9303601	340,7900796	254,160249	517,9855104	0,261824097	False	0,261824097
ENSMUSG00000027835	Pdcd10	242,6713715	159,0112775	94,22361373	131,578536	290,9591154	0,261812524	False	0,261812524
ENSMUSG00000027678	Ncoa3	342,3675719	277,6387386	356,6407809	410,4800481	292,2638648	0,26176604	False	0,26176604
ENSMUSG00000028670	Lypla2	881,8090284	1568,658873	1792,890444	735,490278	1007,266534	-0,261759976	False	0,261759976
ENSMUSG00000054079	Utp18	121,3356858	135,0333865	124,1638274	101,2142584	435,7862984	-0,261591378	False	0,261591378
ENSMUSG00000089715	Cbx6	65,69129484	71,93367317	148,820474	184,4348709	54,79947465	-0,26154014	False	0,26154014
ENSMUSG00000074336	Apoc4	6554,445547	7771,360691	22537,93617	5467,819162	6504,175742	-0,261508216	False	0,261508216
ENSMUSG00000016559	H3f3b	1725,748957	3317,782926	1439,772042	1545,204346	12174,61662	-0,261382204	False	0,261382204
ENSMUSG00000018707	Dync1h1	1089,702656	1581,278815	1305,921674	2122,125619	2327,672923	0,261133847	False	0,261133847
ENSMUSG00000027333	Smox	44,82464824	281,4247213	53,71626577	101,2142584	296,178113	0,261066754	False	0,261066754
ENSMUSG00000057554	Lgals8	1643,05521	879,6100035	1371,085669	1310,162345	867,6583487	-0,261062241	False	0,261062241
ENSMUSG00000030359	Pzp	85078,72804	50279,11356	71010,26157	47099,49287	48151,77648	-0,260770944	False	0,260770944
ENSMUSG00000056342	Usp34	578,0833946	1072,695126	482,5657974	1300,04092	1146,87472	-0,260551966	False	0,260551966
ENSMUSG00000029656	C8b	2490,086494	521,2036319	2982,573642	574,6720674	589,7467272	0,260361904	False	0,260361904
ENSMUSG00000037798	Mat1a	37719,16866	62699,66112	31490,94066	62595,39583	42037,72081	-0,260361026	False	0,260361026
ENSMUSG00000020486	Sept4	226,4417575	46,69378785	271,2231124	195,6808997	31,31398552	0,260340098	False	0,260340098
ENSMUSG00000026915	Strbp	448,2464824	638,5690987	374,2526714	609,5347564	417,5198069	-0,260279648	False	0,260279648
ENSMUSG00000041079	Rwdd2b	89,64929649	88,33959863	74,85053427	104,5880671	93,94195655	-0,260279648	False	0,260279648
ENSMUSG00000031441	Atp11a	247,3084041	398,7901881	634,9086495	614,0331679	296,178113	0,260153766	False	0,260153766
ENSMUSG00000000001	Gnai3	1018,601489	398,7901881	371,6108878	183,3102681	850,6966065	-0,259873128	False	0,259873128
ENSMUSG00000024236	Svil	535,5772626	464,4138899	447,3420166	557,8030243	904,1913318	-0,259716444	False	0,259716444
ENSMUSG00000020700	Map3k3	228,7602738	235,9929278	191,089011	366,6205361	301,3971106	-0,259591722	False	0,259591722
ENSMUSG00000038503	Mesd	267,4022119	223,3729851	356,6407809	323,885627	305,3113588	-0,259556684	False	0,259556684
ENSMUSG00000030557	Mef2a	449,0193212	502,2737179	375,1332659	1131,350489	579,3087321	-0,259374322	False	0,259374322
ENSMUSG00000041926	Rnpep	583,4932659	684,0008922	531,8790906	698,3783833	220,502648	0,259292898	False	0,259292898
ENSMUSG00000039007	Cpq	991,5521327	368,5023257	480,8046084	78,72220101	828,5158668	-0,2591593	False	0,2591593
ENSMUSG00000018501	Ncor1	1765,936573	2145,390252	1551,607546	3653,83473	1475,671567	-0,259061863	False	0,259061863
ENSMUSG00000065954	Tacc1	554,8982317	1388,193693	663,9682687	1506,967848	1056,847011	0,258891091	False	0,258891091
ENSMUSG00000024109	Nrxn1	98,15052288	82,0296273	91,58183017	128,2047274	105,6847011	-0,25885088	False	0,25885088
ENSMUSG00000030342	Cd9	265,0836956	482,0818097	229,8351699	221,5467657	1195,150447	-0,258836672	False	0,258836672
ENSMUSG00000019210	Atp6v1e1	629,0907529	315,4985665	314,3722439	141,6999618	525,8140068	-0,258715583	False	0,258715583
ENSMUSG00000021868	Ppif	494,6168082	275,11475	591,759518	174,3134451	173,5316697	0,258699739	False	0,258699739
ENSMUSG00000075229	Ccdc58	231,8516288	166,5832431	277,3872741	64,10236368	140,9129348	0,258699739	False	0,258699739
ENSMUSG00000044098	Rsbn1	108,1974268	324,3325264	129,4473946	545,4323927	461,8812864	0,258699739	False	0,258699739
ENSMUSG00000028999	Rint1	248,0812429	474,5098441	296,7603535	591,5411105	661,507944	0,258485705	False	0,258485705
ENSMUSG00000036112	Metap2	699,4190803	1116,864926	584,7147618	684,8831488	1670,079228	-0,258424139	False	0,258424139
ENSMUSG00000034135	Sik3	725,6955983	1654,474483	606,7296249	1428,245647	2248,08321	-0,258310764	False	0,258310764
ENSMUSG00000020522	Mfap3	226,4417575	484,6057982	189,327822	289,022938	476,2335297	-0,258253593	False	0,258253593
ENSMUSG00000068732	Tmem167b	327,6836354	150,1773177	85,41766852	110,2110814	273,9973733	-0,258141564	False	0,258141564
ENSMUSG00000047036	Zfp445	647,6388832	792,5323991	541,5656303	679,2601345	681,079185	-0,258053422	False	0,258053422
ENSMUSG00000017493	Igfbp4	12116,56612	3340,498822	10132,12056	3125,27138	5988,79973	-0,258044744	False	0,258044744
ENSMUSG00000073988	Ttpa	5935,401698	5369,785602	2555,4853	2741,781801	7097,836717	0,258033714	False	0,258033714
ENSMUSG00000037119	Fam91a1	915,8139339	395,0042053	386,5809947	697,2537804	765,8878958	-0,257921277	False	0,257921277
ENSMUSG00000032508	Myd88	203,2565946	753,4105769	243,0440878	625,2791966	628,8892091	0,257915879	False	0,257915879
ENSMUSG00000022724	Riox2	229,5331126	132,5093979	191,9696055	154,0705934	91,33245776	-0,257824385	False	0,257824385
ENSMUSG00000009079	Ewsr1	1191,717372	2357,405289	1509,339009	1424,871838	2983,96187	0,257790034	False	0,257790034
ENSMUSG00000038784	Cnot4	204,0294334	441,6979931	243,9246823	347,5022873	651,0699489	0,257658458	False	0,257658458
ENSMUSG00000042520	Ubap2l	1098,976721	1087,839057	919,3406798	1321,408374	1642,67949	-0,257489342	False	0,257489342
ENSMUSG00000057335	Cep170	110,5159431	406,3621537	92,46242469	195,6808997	651,0699489	-0,257315408	False	0,257315408
ENSMUSG00000000532	Acvr1b	126,7455571	1015,905384	151,4622576	534,186364	786,7638861	0,257023161	False	0,257023161
ENSMUSG00000028788	Ptp4a2	1781,393348	2101,220453	1490,846524	1689,153513	2746,49748	-0,256874366	False	0,256874366
ENSMUSG00000023026	Dip2b	87,3307802	90,86358716	73,08934523	75,3483924	176,1411685	-0,25682912	False	0,25682912
ENSMUSG00000040850	Psme4	1242,724731	603,2332592	487,8493646	664,6402971	1484,804813	0,256766524	False	0,256766524
ENSMUSG00000021701	Plk2	163,068979	90,86358716	136,4921507	85,46981824	126,5606915	-0,256664372	False	0,256664372
ENSMUSG00000030979	Uros	350,8687983	32,81185092	419,1629919	150,6967848	15,65699276	0,256579687	False	0,256579687
ENSMUSG00000033355	Rtp4	302,952795	68,14769037	253,611222	177,6872537	58,71372284	-0,256474431	False	0,256474431
ENSMUSG00000027247	Arhgap1	242,6713715	203,1810768	270,3425179	414,9784596	240,073889	-0,256237877	False	0,256237877
ENSMUSG00000016257	Prelid3b	327,6836354	482,0818097	415,6406138	274,4031007	476,2335297	-0,256006843	False	0,256006843
ENSMUSG00000026036	Nif3l1	190,1183356	180,4651801	167,312959	122,581713	227,026395	-0,255962364	False	0,255962364
ENSMUSG00000068876	Cgn	585,8117822	89,6015929	574,1476276	699,5029861	24,79023853	0,255893006	False	0,255893006
ENSMUSG00000055723	Rras2	154,5677526	78,2436445	129,4473946	103,4634642	84,80871077	-0,255873434	False	0,255873434
ENSMUSG00000034903	Cobll1	421,9699645	212,0150367	339,0288905	503,8220865	185,2744143	0,255774056	False	0,255774056
ENSMUSG00000061477	Rps7	2172,449762	3486,890157	3590,183862	1819,607446	3408,005424	-0,255695572	False	0,255695572
ENSMUSG00000008976	Gabpa	619,8166878	266,2807902	228,0739809	227,1697801	739,7929078	0,255279872	False	0,255279872
ENSMUSG00000022551	Cyc1	1901,183356	1232,968398	1592,995488	1196,577455	1307,358895	-0,255155496	False	0,255155496
ENSMUSG00000023791	Pigx	146,0665262	122,4134438	99,50718086	110,2110814	108,2941999	-0,254863588	False	0,254863588
ENSMUSG00000022191	Drosha	180,0714317	387,4322397	214,8650631	242,9142203	268,7783757	0,254861604	False	0,254861604
ENSMUSG00000028140	Mrpl9	325,3651191	141,3433578	126,805611	84,34521537	272,6926239	-0,254783918	False	0,254783918
ENSMUSG00000019851	Perp	1408,885065	728,1706916	1180,877252	634,2760196	859,8298523	-0,254694913	False	0,254694913
ENSMUSG00000027074	Slc43a3	444,3822886	1386,931698	372,4914823	2638,318337	1452,186078	-0,254593881	False	0,254593881
ENSMUSG00000023915	Tnfrsf21	110,5159431	79,50563877	73,96993975	24,74126318	92,63720715	-0,254590844	False	0,254590844
ENSMUSG00000037519	Ppfia1	430,4711909	851,8461296	513,3866057	880,5640485	777,6306403	0,254128967	False	0,254128967
ENSMUSG00000024191	Bnip1	265,0836956	331,904492	316,133433	399,2340194	521,8997586	0,254085679	False	0,254085679

ENSMUSG00000043279	Trim56	343,1404107	287,7346927	263,2977617	255,2848519	86,11346017	-0,254059858	False	0,254059858
ENSMUSG00000009293	Ube2g2	275,1305996	397,5281938	230,7157645	455,464163	606,7084694	-0,254000018	False	0,254000018
ENSMUSG00000037053	Azgp1	24101,74966	9184,794269	28739,96338	11340,49536	10394,93844	0,253920341	False	0,253920341
ENSMUSG00000015672	Mrpl32	360,9157022	270,066773	267,7007343	89,96822973	302,70186	-0,253764371	False	0,253764371
ENSMUSG00000025040	Fundc1	136,792461	104,7455241	90,70123565	73,09918666	163,0936746	0,253712108	False	0,253712108
ENSMUSG00000024782	Ak3	6274,677915	3778,410833	5264,194046	2322,30493	4004,275898	-0,253328736	False	0,253328736
ENSMUSG00000057637	Prdm2	392,6020915	329,3805035	323,1781892	166,441225	266,1688769	-0,253312696	False	0,253312696
ENSMUSG00000001313	Rnd2	381,0095101	432,8640333	508,9836331	850,1997709	319,6636021	-0,253272524	False	0,253272524
ENSMUSG00000042726	Trafd1	763,5646976	403,8381652	628,7444879	429,598297	640,6319537	-0,253254644	False	0,253254644
ENSMUSG00000026767	Mindy3	284,4046647	176,6791973	238,6411152	173,1888422	229,6358938	-0,253102503	False	0,253102503
ENSMUSG00000046826	Fam187b	52,55303587	159,0112775	147,059285	195,6808997	62,62797103	0,253033033	False	0,253033033
ENSMUSG00000023707	Ogfod2	196,3010458	258,7088246	243,0440878	233,9173973	262,2546287	0,252931306	False	0,252931306
ENSMUSG00000069727	Zfp975	98,15052288	69,40968464	105,6713425	116,9586986	27,39973733	0,252931306	False	0,252931306
ENSMUSG00000027673	Ndufb5	844,7127677	545,181523	708,8785893	168,6904307	572,7849851	-0,252922301	False	0,252922301
ENSMUSG00000027746	Ufm1	604,3599125	911,1598602	603,2072468	507,1958951	1563,089777	-0,2528649	False	0,2528649
ENSMUSG00000060147	Serpinb6a	143,7480099	129,9854094	120,6414494	226,0451772	139,6081854	-0,252816317	False	0,252816317
ENSMUSG00000026798	Coq4	135,2467835	179,2031858	154,9846357	122,581713	113,5131975	-0,252734243	False	0,252734243
ENSMUSG00000090112	Shprh	226,4417575	78,2436445	87,17885756	190,0578853	76,98021439	-0,252701144	False	0,252701144
ENSMUSG00000034343	Ube2f	553,3525542	661,2849955	651,6399454	464,460986	992,9142907	-0,252641531	False	0,252641531
ENSMUSG00000040263	Klhdc4	136,0196223	169,1072317	162,0293918	195,6808997	181,3601661	0,252440749	False	0,252440749
ENSMUSG00000016253	Nelfcd	341,5947332	389,9562282	371,6108878	286,7737323	378,377325	-0,252372848	False	0,252372848
ENSMUSG00000020464	Pnpt1	391,056414	328,1185092	367,2079152	330,6332443	465,7955346	0,252320063	False	0,252320063
ENSMUSG00000032080	Apoa4	4186,467578	8796,100035	34689,25996	4986,489133	33031,03572	0,252290954	False	0,252290954
ENSMUSG00000039477	Tnrc18	430,4711909	644,87907	538,0432522	493,7006606	361,4155828	-0,252257978	False	0,252257978
ENSMUSG00000001440	Kpnb1	1527,902234	1819,795732	1291,832162	858,071991	1416,957845	0,252224287	False	0,252224287
ENSMUSG00000029650	Slc46a3	443,6094498	63,09971331	528,3567125	62,97776081	35,22823371	0,252222175	False	0,252222175
ENSMUSG00000055401	Fbxo6	581,1747496	1535,847022	862,1020359	756,8577326	487,9762743	-0,252161019	False	0,252161019
ENSMUSG00000063888	Rpl7l1	619,8166878	379,8602741	520,4313618	283,3999236	302,70186	-0,252133695	False	0,252133695
ENSMUSG00000036887	C1qa	575,7648783	378,5982798	483,446392	476,8316176	382,2915732	-0,252123865	False	0,252123865
ENSMUSG00000032745	Gpbp1	440,5180948	273,8527558	296,7603535	251,9110432	524,5092574	0,251766955	False	0,251766955
ENSMUSG00000051166	Eml5	68,78264989	516,1556549	81,89529044	743,3624981	420,1293057	0,251735791	False	0,251735791
ENSMUSG00000048486	Fitm2	377,1453162	316,7605608	292,3573809	308,1411868	242,6833878	-0,25172779	False	0,25172779
ENSMUSG00000028101	Pias3	135,2467835	109,7935012	113,5966932	98,9650527	58,71372284	-0,251673445	False	0,251673445
ENSMUSG00000021326	Trim27	238,0343389	363,4543486	292,3573809	283,3999236	558,4327417	0,251669657	False	0,251669657
ENSMUSG00000046865	Fbl	210,2121435	348,3104175	294,11857	176,5626508	421,4340551	-0,251665818	False	0,251665818
ENSMUSG00000034252	Senp6	588,1302985	941,4477225	494,0135262	931,1711777	1445,662331	-0,251585272	False	0,251585272
ENSMUSG00000022350	Washc5	415,0144156	344,5244347	187,5666329	348,6268902	301,3971106	-0,251477598	False	0,251477598
ENSMUSG00000038403	Hfe2	1717,247731	204,4430711	2043,859883	978,4044983	27,39973733	0,251198117	False	0,251198117
ENSMUSG00000025151	Maged1	1053,379234	1080,267092	1021,489644	885,06246	1762,716435	-0,251173747	False	0,251173747
ENSMUSG00000033392	Clasp2	138,3381385	127,4614209	116,2384768	294,6459524	221,8073974	-0,251111247	False	0,251111247
ENSMUSG00000034480	Diaph2	199,3924008	189,2991399	88,94004661	237,2912059	106,9894505	0,251048208	False	0,251048208
ENSMUSG00000029560	Snx8	139,1109773	184,2511629	165,5517699	204,6777226	193,1029107	0,251046167	False	0,251046167
ENSMUSG00000035798	Zdhhc17	156,1134301	78,2436445	131,2085836	124,8309187	13,04749397	-0,250732551	False	0,250732551
ENSMUSG00000073557	Ppp1r12b	122,8813633	382,3842626	146,1786905	210,300737	238,7691396	0,250466887	False	0,250466887
ENSMUSG00000038023	Atp6v0a2	351,6416371	495,9637466	418,2823974	901,931503	649,7651995	0,250371379	False	0,250371379
ENSMUSG00000000339	Rtca	79,60239257	148,9153234	66,92518359	111,3356843	200,9314071	-0,250262602	False	0,250262602
ENSMUSG00000040938	Slc16a11	191,6640132	122,4134438	161,1487973	56,23014358	113,5131975	-0,250186061	False	0,250186061
ENSMUSG00000026229	Psmd1	1190,171695	1499,249188	1036,459751	1000,896556	1873,620133	-0,249876835	False	0,249876835
ENSMUSG00000039852	Rere	421,9699645	829,1302329	354,8795919	558,9276272	775,0211415	-0,249810701	False	0,249810701
ENSMUSG00000062352	Itgb1bp1	124,4270408	116,1034725	147,9398795	77,59759814	117,4274457	0,249710956	False	0,249710956
ENSMUSG00000002617	Zfp40	54,87155216	266,2807902	104,790748	845,7013595	65,23746983	0,249642443	False	0,249642443
ENSMUSG00000035133	Arhgap5	895,7201261	753,4105769	507,222444	567,9244502	254,4261323	-0,249611736	False	0,249611736
ENSMUSG00000032309	Fbxo22	721,8314045	858,156101	899,0870058	1786,993963	1002,047537	0,24957819	False	0,24957819
ENSMUSG00000090862	Rps13	1148,438402	2021,714814	1867,740979	966,0338667	1941,467102	-0,249527805	False	0,249527805
ENSMUSG00000001416	Cct3	887,2188997	672,6429439	714,1621564	430,7228998	746,3166548	-0,249502217	False	0,249502217
ENSMUSG00000022747	St3gal6	232,6244676	109,7935012	79,25350688	195,6808997	80,89446258	-0,249499907	False	0,249499907
ENSMUSG00000015757	Ppil4	218,7133699	259,9708188	289,7155974	580,2950818	327,4920985	0,249308278	False	0,249308278
ENSMUSG00000054920	Klhl5	469,113129	248,6128704	350,4766193	394,7356079	80,89446258	-0,249049212	False	0,249049212
ENSMUSG00000000738	Spg7	384,8737039	132,5093979	261,5365727	323,885627	164,398424	-0,248900656	False	0,248900656
ENSMUSG00000074305	Peak1	181,6171093	215,8010195	131,2085836	152,9459905	148,7414312	0,248801562	False	0,248801562
ENSMUSG00000027131	Emc4	527,0760362	426,554062	568,8640605	182,1856652	626,2797103	0,24879604	False	0,24879604
ENSMUSG00000033014	Trim33	284,4046647	199,395094	154,9846357	323,885627	337,9300937	0,248779705	False	0,248779705
ENSMUSG00000017615	Tnfaip1	526,3031975	1081,529086	625,2221098	1044,756068	692,8219295	0,248474638	False	0,248474638
ENSMUSG00000001924	Uba1	3020,253885	1765,529978	1747,099529	1387,759944	2542,956574	-0,248163004	False	0,248163004
ENSMUSG00000033713	Foxn3	419,6514482	947,7576939	677,1771865	804,0910532	498,4142695	0,248153813	False	0,248153813
ENSMUSG00000079509	Zfx	197,8467233	166,5832431	204,2979288	194,5562968	361,4155828	-0,248139887	False	0,248139887
ENSMUSG00000003429	Rps11	1769,800767	3897,038294	4646,897287	2101,882767	3479,76664	0,248095245	False	0,248095245
ENSMUSG00000045438	Cox19	201,7109171	246,0888819	282,6708412	239,5404117	416,2150575	0,247979898	False	0,247979898
ENSMUSG00000037149	Ddx1	775,9301179	1166,082702	653,4011345	828,8323164	1497,852307	-0,247957766	False	0,247957766
ENSMUSG00000000934	Top1mt	327,6836354	190,5611342	307,3274878	389,1125936	56,10422405	0,247884047	False	0,247884047
ENSMUSG00000021738	Atxn7	339,2762169	492,1777638	292,3573809	536,4355698	285,7401178	-0,247756697	False	0,247756697
ENSMUSG00000047228	A2ml1	486,8884206	812,7243074	1741,815962	1152,717943	578,0039827	0,247488243	False	0,247488243
ENSMUSG00000029722	Agfg2	613,6339777	108,5315069	516,9089837	275,5277035	26,09498793	-0,247468093	False	0,247468093
ENSMUSG00000044934	Zfp367	138,3381385	150,1773177	88,05945209	164,1920193	125,2559421	0,247185056	False	0,247185056
ENSMUSG00000058099	Nfam1	91,96781277	166,5832431	77,49231784	172,0642394	203,5409059	-0,247075733	False	0,247075733
ENSMUSG00000028397	Kdm4c	106,6517493	118,627461	77,49231784	101,2142584	126,5606915	0,246921759	False	0,246921759
ENSMUSG00000041645	Ddx24	466,021774	644,87907	553,0133591	557,8030243	1229,073932	0,246916968	False	0,246916968
ENSMUSG00000031776	Arl2bp	74,19252123	338,2144633	62,52221098	147,3229762	584,5277296	-0,24690497	False	0,24690497
ENSMUSG00000018858	Mrpl58	444,3822886	643,6170757	676,296592	374,4927562	589,7467272	-0,246863504	False	0,246863504
ENSMUSG00000042524	Sun2	706,3746292	5113,600766	595,2818961	1892,706633	4128,227091	-0,246860507	False	0,246860507
ENSMUSG00000036054	Sugp2	242,6713715	223,3729851	287,9544083	186,6840767	103,0752023	0,246836488	False	0,246836488
ENSMUSG00000042396	Rbm7	478,3871942	314,2365723	201,6561453	419,4768711	567,5659875	0,246609365	False	0,246609365
ENSMUSG00000001445	Mrpl10	644,5475282	533,8235746	543,3268194	413,8538568	446,2242936	-0,246466483	False	0,246466483
ENSMUSG00000029238	Clock	701,7375966	384,9082512	373,3720768	591,5411105	447,529043	-0,246453224	False	0,246453224
ENSMUSG00000079435	Rpl36a	890,3102547	1486,629246	1723,323477	1056,002096	1289,092404	0,246232621	False	0,246232621
ENSMUSG00000007837	Prrg2	317,6367315	239,7789106	313,4916494	376,741962	181,3601661	0,246198976	False	0,246198976
ENSMUSG00000078671	Chd2	484,5699043	1317,522014	408,5958577	1069,497331	1553,956531	-0,246030226	False	0,246030226
ENSMUSG00000009646	Pla2g12b	564,9451356	469,461867	476,4016358	247,4126318	457,9670382	-0,245932401	False	0,245932401
ENSMUSG00000057841	Rpl32	2050,341238	4140,603187	4150,241977	2431,391408	3235,778503	0,245918122	False	0,245918122
ENSMUSG00000036686	Cc2d1a	231,0787901	471,9858555	323,1781892	502,6974836	273,9973733	0,245777217	False	0,245777217
ENSMUSG00000005871	Apc	451,3378375	535,0855688	319,6558111	416,1030625	300,0923612	0,24556189	False	0,24556189
ENSMUSG00000002885	Adgre5	267,4022119	542,6575344	317,0140275	1060,500508	696,7361777	0,24553528	False	0,24553528
ENSMUSG00000021044	Adck1	179,298593	195,6091113	120,6414494	212,5499427	90,02770836	0,245437703	False	0,245437703
ENSMUSG00000024926	Kat5	220,2590474	410,1481365	185,8054439	224,9205743	450,1385418	-0,245408509	False	0,245408509
ENSMUSG00000029102	Hgfac	4558,203023	2505,058618	5403,32798	2832,874634	1540,909037	0,245383072	False	0,245383072
ENSMUSG00000008845	Cd163	255,8096305	215,8010195	106,551937	176,5626508	108,2941999	-0,245368898	False	0,245368898
ENSMUSG00000004661	Arid3b	80,37523133	98,43555276	67,80577811	101,2142584	195,7124095	-0,245342766	False	0,245342766
ENSMUSG00000021495	Fam193b	1177,806275	2107,530424	3251,154971	5330,617611	1396,081854	0,245281267	False	0,245281267
ENSMUSG00000040824	Snrpd2	233,3973064	437,9120103	675,4159975	292,3967466	276,6068721	0,245049088	False	0,245049088
ENSMUSG00000022871	Fetub	10296,53084	15395,06805	20929,97057	8688,681786	18089,04563	-0,24494912	False	0,24494912
ENSMUSG00000020186	Csrp2	231,8516288	103,4835298	274,7454905	154,0705934	142,2176842	0,24489394	False	0,24489394
ENSMUSG00000024413	Npc1	1764,390895	599,4472764	1489,085335	1448,488499	559,7374911	-0,244743788	False	0,244743788
ENSMUSG00000040363	Bcor	256,5824693	259,9708188	160,2682028	121,4571101	304,0066094	0,244680086	False	0,244680086
ENSMUSG00000026259	Ngef	387,9650589	80,76763303	459,6703399	455,464163	48,27572767	0,244672854	False	0,244672854
ENSMUSG00000028854	Slc9a1	176,2072379	328,1185092	271,2231124	386,8633878	208,7599034	0,244571454	False	0,244571454
ENSMUSG00000024537	Psmg2	132,9282672	151,4393119	201,6561453	275,5277035	112,2084481	-0,244466627	False	0,244466627
ENSMUSG00000047907	Tshz2	174,6615604	210,7530424	297,640948	833,3307279	147,4366818	-0,244466627	False	0,244466627
ENSMUSG00000043683	Fem1a	342,3675719	354,6203888	361,0437536	289,022938	387,5105708	-0,244362065	False	0,244362065
ENSMUSG00000030579	Tyrobp	149,1578812	176,6791973	126,805611	122,581713	151,35093	0,244291974	False	0,244291974
ENSMUSG00000004562	Arhgef40	132,9282672	68,14769037	102,1489644	157,444402	86,11346017	0,244194537	False	0,244194537
ENSMUSG00000022425	Enpp2	360,1428635	426,554062	401,5511015	113,58489	284,4353684	0,244159286	False	0,244159286
ENSMUSG00000028158	Mttp	3555,058309	2611,066137	2877,782894	1564,322594	4210,426303	0,24409309	False	0,24409309
ENSMUSG00000027698	Nceh1	535,5772626	526,251609	212,2232795	634,2760196	534,9472526	0,244016085	False	0,244016085

ENSMUSG00000042535	Gtpbp1	238,8071777	232,206945	201,6561453	294,6459524	420,1293057	-0,243948828	False	0,243948828
ENSMUSG00000054364	Rhob	314,5453765	591,8753108	372,4914823	431,8475027	729,3549126	0,24393928	False	0,24393928
ENSMUSG00000030083	Abtb1	328,4564742	437,9120103	277,3872741	834,4553307	467,100284	-0,243800601	False	0,243800601
ENSMUSG00000020380	Rad50	157,6591076	131,2474037	138,2533398	186,6840767	50,88522646	0,243790364	False	0,243790364
ENSMUSG00000060459	Kng2	4433,775982	3079,266009	4581,733292	5249,646205	4451,804941	0,243684317	False	0,243684317
ENSMUSG00000024404	Riok3	711,0116618	1099,197006	600,5654632	933,4203834	1437,833835	-0,243551711	False	0,243551711
ENSMUSG00000008958	Vps72	292,9058911	538,8715516	401,5511015	247,4126318	356,1965852	-0,24351805	False	0,24351805
ENSMUSG00000021978	Extl3	217,9405311	547,7055115	258,0141946	283,3999236	515,3760116	0,243515913	False	0,243515913
ENSMUSG00000032078	Zpr1	496,9353245	13681,27984	588,2371399	4730,079678	34526,27853	0,243339776	False	0,243339776
ENSMUSG00000024870	Rab1b	1622,961402	787,4844221	1371,085669	481,3300291	765,8878958	-0,243309972	False	0,243309972
ENSMUSG00000026632	Tatdn3	103,5603942	63,09971331	95,98480277	122,581713	76,98021439	0,243271409	False	0,243271409
ENSMUSG00000036339	Tmem260	234,1701451	76,98165023	169,9547425	197,9301054	15,65699276	-0,24256609	False	0,24256609
ENSMUSG00000021311	Mtr	105,1060717	85,8156101	65,16399454	88,84362686	61,32322164	-0,242505819	False	0,242505819
ENSMUSG00000025326	Ube3a	547,9426828	702,9308062	559,1775207	1121,229063	463,1860358	-0,24243323	False	0,24243323
ENSMUSG00000050188	Lsm10	191,6640132	59,31373051	162,0293918	50,60712922	15,65699276	-0,242323943	False	0,242323943
ENSMUSG00000020561	Twistnb	284,4046647	798,8423705	336,387107	728,7426608	1024,228276	0,242177283	False	0,242177283
ENSMUSG00000074890	Lcmt2	129,8369122	109,7935012	65,16399454	86,59442111	83,50396138	-0,241907933	False	0,241907933
ENSMUSG00000021149	Gtpbp4	307,5898276	413,9341193	363,6855371	464,460986	558,4327417	0,241683762	False	0,241683762
ENSMUSG00000034210	Efcab14	238,0343389	281,4247213	172,5965261	169,8150336	271,3878745	0,241579353	False	0,241579353
ENSMUSG00000036748	Cuedc2	170,7973666	201,9190826	332,8647289	360,9975218	211,3694022	0,241491529	False	0,241491529
ENSMUSG00000037007	Zfp113	127,5183959	118,627461	101,2683699	150,6967848	40,44723129	0,240943251	False	0,240943251
ENSMUSG00000026335	Pam	306,8169888	256,184836	166,4323644	177,6872537	259,6451299	-0,240837203	False	0,240837203
ENSMUSG00000021807	Rtraf	909,6312238	748,3625998	766,9978277	607,2855507	769,8021439	-0,240794088	False	0,240794088
ENSMUSG00000026858	Miga2	625,9993979	104,7455241	739,6993975	593,7903162	28,70448672	0,240777831	False	0,240777831
ENSMUSG00000042506	Usp22	294,4515686	145,1293406	160,2682028	184,4348709	249,2071347	-0,240684986	False	0,240684986
ENSMUSG00000028715	Cyp4a14	8024,384874	1823,581715	8646,5576	9480,402208	2862,620176	0,240557463	False	0,240557463
ENSMUSG00000028431	Ikbkap	125,9727183	230,9449507	148,820474	452,0903544	331,4063467	0,240461693	False	0,240461693
ENSMUSG00000029911	Ssbp1	278,9947934	488,391781	361,9243481	236,166603	334,0158455	-0,240433235	False	0,240433235
ENSMUSG00000044350	Lacc1	273,584922	122,4134438	323,1781892	217,0482542	249,2071347	0,240341114	False	0,240341114
ENSMUSG00000030612	Mrpl46	210,9849822	185,5131571	166,4323644	84,34521537	249,2071347	0,24020506	False	0,24020506
ENSMUSG00000026260	Ndufa10	2160,857181	2227,41988	2428,679689	916,5513404	2552,08982	0,240075383	False	0,240075383
ENSMUSG00000018921	Pelp1	169,2516891	362,1923544	199,8949562	432,9721056	674,555438	0,240071839	False	0,240071839
ENSMUSG00000015363	Trabd	1535,630622	2862,202996	2088,770203	6544,06411	1813,601661	0,240026384	False	0,240026384
ENSMUSG00000063019	Manbal	105,8789105	297,8306468	125,044422	201,303914	495,8047707	0,240025448	False	0,240025448
ENSMUSG00000020828	Pld2	164,6146565	400,0521824	291,4767864	567,9244502	194,4076601	0,239992273	False	0,239992273
ENSMUSG00000027968	Larp7	148,3850425	204,4430711	175,2383097	204,6777226	215,2836504	0,239972531	False	0,239972531
ENSMUSG00000041346	Wrap53	94,28632906	184,2511629	161,1487973	111,3356843	221,8073974	0,239795557	False	0,239795557
ENSMUSG00000063760	Rnf217	290,5873748	246,0888819	122,4026384	132,7031389	157,874677	-0,239792544	False	0,239792544
ENSMUSG00000041483	Zfp281	213,3034985	653,7130299	251,850033	856,9473882	514,0712622	0,239657291	False	0,239657291
ENSMUSG00000021930	Spryd7	362,4613798	251,136859	249,2082494	176,5626508	427,9578021	0,239641265	False	0,239641265
ENSMUSG00000021468	Sptlc1	220,2590474	180,4651801	161,1487973	151,8213877	186,5791637	-0,239413399	False	0,239413399
ENSMUSG00000075232	Amd1	184,7084643	156,487289	143,5369069	147,3229762	153,9604288	-0,239204504	False	0,239204504
ENSMUSG00000050017	Pitpnb	991,5521327	1097,935012	974,8181346	468,9593975	1170,360209	0,239192093	False	0,239192093
ENSMUSG00000020739	Nup85	170,0245278	200,6570883	130,3279891	122,581713	139,6081854	0,238989236	False	0,238989236
ENSMUSG00000046667	Rbm12b1	79,60239257	224,6349794	120,6414494	221,5467657	93,94195655	0,238957847	False	0,238957847
ENSMUSG00000020802	Ube2o	147,6122037	174,1552087	205,1785234	432,9721056	224,4168962	0,238561623	False	0,238561623
ENSMUSG00000042298	Ttc19	126,7455571	367,2403314	107,4325315	471,2086032	326,1873491	-0,238504256	False	0,238504256
ENSMUSG00000047388	Atmin	128,2912346	85,8156101	60,76102194	77,59759814	151,35093	0,238474937	False	0,238474937
ENSMUSG00000022864	D16Ertd472e	65,69129484	230,9449507	77,49231784	161,9428135	292,2638648	0,238351094	False	0,238351094
ENSMUSG00000019518	Ap4m1	196,3010458	212,0150367	166,4323644	299,1443639	298,7876118	-0,238131851	False	0,238131851
ENSMUSG00000045665	Mfsd5	196,3010458	364,7163429	236,8799261	166,441225	364,0250816	-0,238055047	False	0,238055047
ENSMUSG00000026203	Dnajb2	824,6189599	280,1627271	699,1920496	285,6491294	297,4828624	-0,238038852	False	0,238038852
ENSMUSG00000026917	Wdr5	265,8565344	343,2624404	225,4321973	303,6427753	644,5462019	-0,237954343	False	0,237954343
ENSMUSG00000030750	Nsmce1	116,6986532	238,5169163	172,5965261	98,9650527	156,5699276	-0,237796846	False	0,237796846
ENSMUSG00000020721	Helz	265,8565344	508,5836893	313,4916494	576,9212731	701,9551753	0,237779088	False	0,237779088
ENSMUSG00000055128	Cgrrf1	330,7749905	229,6829564	272,1037069	199,0547083	280,5211202	-0,237740764	False	0,237740764
ENSMUSG00000020238	Ncln	932,8163867	1330,141957	1255,727787	1789,243169	1099,903741	0,237712233	False	0,237712233
ENSMUSG00000037049	Smpd1	695,5548865	326,8565149	589,998329	432,9721056	348,3680889	-0,237453495	False	0,237453495
ENSMUSG00000042118	Bhmt2	3065,851372	639,8310929	3613,959914	1761,128097	183,9696649	0,237292745	False	0,237292745
ENSMUSG00000032402	Smad3	181,6171093	89,6015929	154,1040411	84,34521537	43,05673008	-0,236995423	False	0,236995423
ENSMUSG00000019437	Tlcd1	881,0361896	299,0926411	747,6247482	698,3783833	125,2559421	-0,236886954	False	0,236886954
ENSMUSG00000040021	Lats1	367,0984123	455,5799301	353,9989974	311,5149954	964,209804	-0,236865263	False	0,236865263
ENSMUSG00000058835	Abi1	589,675976	451,7939473	371,6108878	500,4482779	448,8337924	-0,236701447	False	0,236701447
ENSMUSG00000057113	Npm1	1765,163734	1460,127366	807,5051756	627,5284024	2079,770538	0,236622352	False	0,236622352
ENSMUSG00000024298	Zfp871	564,1722968	256,184836	272,1037069	269,9046892	478,8430285	-0,236583028	False	0,236583028
ENSMUSG00000020883	Fbxl20	466,7946127	196,8711055	160,2682028	549,9308042	28,70448672	0,23646219	False	0,23646219
ENSMUSG00000036545	Adamts2	108,1974268	127,4614209	69,56696715	91,0928326	88,72295896	0,23639446	False	0,23639446
ENSMUSG00000024535	Snx24	154,5677526	185,5131571	131,2085836	143,9491676	228,3311444	-0,236377258	False	0,236377258
ENSMUSG00000058600	Rpl30	1635,326822	3441,458364	3403,497823	1926,444719	2283,311444	0,236361798	False	0,236361798
ENSMUSG00000026750	Psmb7	1089,702656	562,8494427	650,7593509	191,1824882	925,0673221	-0,236304257	False	0,236304257
ENSMUSG00000028811	Yars	401,1033179	450,531953	390,9839673	797,343436	340,5395925	-0,236151359	False	0,236151359
ENSMUSG00000027679	Dnajc19	598,1772024	440,4359989	704,4756167	318,2626127	421,4340551	0,235976845	False	0,235976845
ENSMUSG00000051043	Gprc5c	849,3498003	291,5206755	721,2069126	258,6586605	241,3786384	-0,235945618	False	0,235945618
ENSMUSG00000026349	Ccnt2	462,9304189	545,181523	779,326151	1131,350489	657,5936958	0,235941303	False	0,235941303
ENSMUSG00000046434	Hnrnpa1	625,9993979	537,6095574	485,207581	383,4895792	737,183409	0,235862333	False	0,235862333
ENSMUSG00000015668	Pdzd11	255,0367917	406,3621537	397,1481289	251,9110432	216,5883998	-0,23574941	False	0,23574941
ENSMUSG00000032264	Zw10	166,9331728	314,2365723	141,7757179	588,1673019	409,6913105	-0,235660213	False	0,235660213
ENSMUSG00000001855	Nup214	162,2961402	1027,263333	191,089011	493,7006606	3169,236284	0,235616126	False	0,235616126
ENSMUSG00000006611	Hfe	673,1425624	792,5323991	540,6850358	663,5156943	451,4432912	0,235557834	False	0,235557834
ENSMUSG00000019794	Katna1	191,6640132	162,7972603	118,8802603	85,46981824	103,0752023	-0,235503061	False	0,235503061
ENSMUSG00000047694	Yipf6	383,3280264	325,5945207	273,864896	178,8118566	285,7401178	-0,235503061	False	0,235503061
ENSMUSG00000010064	Slc38a3	18883,54233	5373,571585	16040,0292	10960,37959	3751,154515	-0,235452655	False	0,235452655
ENSMUSG00000016356	Col20a1	68,78264989	884,6579806	122,4026384	80,97140676	1193,845698	0,235367845	False	0,235367845
ENSMUSG00000020277	Pfkl	396,4662853	138,8193693	318,7752165	466,7101917	75,675465	0,235328785	False	0,235328785
ENSMUSG00000036114	Rpp25l	142,9751711	142,6053521	236,8799261	121,4571101	177,4459179	-0,235317684	False	0,235317684
ENSMUSG00000030397	Mark4	187,0269806	535,0855688	220,1486302	414,9784596	529,728255	0,235231459	False	0,235231459
ENSMUSG00000066640	Fbxl18	119,7900082	122,4134438	147,059285	110,2110814	101,7704529	-0,235188813	False	0,235188813
ENSMUSG00000051256	Jagn1	239,5800165	278,9007328	332,8647289	203,5531198	284,4353684	-0,235102244	False	0,235102244
ENSMUSG00000024589	Nedd4l	1011,645941	3748,12297	1190,563792	3405,297495	3304,930221	0,234940466	False	0,234940466
ENSMUSG00000034557	Zfyve9	237,2615002	199,395094	135,6115562	110,2110814	279,2163709	0,234905514	False	0,234905514
ENSMUSG00000074088	Snrnp40	88,87645772	167,8452374	113,5966932	104,5880671	241,3786384	0,234845035	False	0,234845035
ENSMUSG00000026837	Col5a1	165,3874952	66,88569611	194,6113891	152,9459905	48,27572767	0,234745984	False	0,234745984
ENSMUSG00000030108	Slc6a13	3431,404707	1318,784008	2916,529053	2442,637437	868,9630981	-0,234546592	False	0,234546592
ENSMUSG00000042709	Atpaf2	252,7182754	198,1330998	206,0591179	214,7991485	155,2651782	-0,234541723	False	0,234541723
ENSMUSG00000028577	Plaa	596,6315249	543,9195287	255,372411	494,8252635	701,9551753	0,234538698	False	0,234538698
ENSMUSG00000041115	Iqsec2	247,3084041	320,5465436	302,0439207	321,6364213	290,9591154	0,234505178	False	0,234505178
ENSMUSG00000027878	Notch2	559,5352643	475,7718383	457,9091508	311,5149954	356,1965852	-0,23395918	False	0,23395918
ENSMUSG00000039704	Lmbrd2	621,3623653	276,3767443	528,3567125	219,29756	396,6438165	-0,233922589	False	0,233922589
ENSMUSG00000025077	Dclre1a	208,666466	273,8527558	274,7454905	319,3872155	177,4459179	-0,233819383	False	0,233819383
ENSMUSG00000032590	Apeh	633,7277855	254,9228418	538,9238468	367,745139	272,6926239	-0,233781845	False	0,233781845
ENSMUSG00000050619	Zscan29	178,5257542	262,4948074	174,3577151	151,8213877	221,8073974	-0,23375717	False	0,23375717
ENSMUSG00000018102	Hist1h2bc	1738,887216	169,1072317	2044,740477	92,21743547	1110,341736	0,233753382	False	0,233753382
ENSMUSG00000068264	Ap5s1	390,2835752	1203,94253	331,9841344	653,3942684	1140,350973	-0,233408453	False	0,233408453
ENSMUSG00000025228	Actr1a	731,8783084	793,7943934	622,5803262	643,2728426	928,9815703	-0,233343799	False	0,233343799
ENSMUSG00000021474	Sfxn1	1251,225957	1682,238357	1064,638776	2398,777925	1295,616151	-0,23297833	False	0,23297833
ENSMUSG00000019158	Tmem160	139,8838161	203,1810768	420,924181	555,5538186	164,398424	0,23296741	False	0,23296741
ENSMUSG00000038459	Abhd17c	950,5916782	316,7605608	220,1486302	156,3197992	808,9446258	-0,232784822	False	0,232784822
ENSMUSG00000061288	Taok3	809,1621847	585,5653395	688,6249153	500,4482779	384,901072	-0,232710518	False	0,232710518
ENSMUSG00000030034	Ino80b	210,9849822	172,8932145	188,4472275	127,0801245	247,9023853	0,232631841	False	0,232631841
ENSMUSG00000002326	Gmpr2	189,3454969	97,17355849	161,1487973	109,0864785	62,62797103	-0,232627689	False	0,232627689
ENSMUSG00000064181	Rab3ip	272,8120833	320,5465436	257,1336001	195,6808997	170,9221709	0,232626307	False	0,232626307
ENSMUSG00000040760	Appl1	149,1578812	294,044664	175,2383097	290,1475409	254,4261323	0,232477995	False	0,232477995
ENSMUSG00000039115	Itga9	221,0318862	146,3913349	98,62658634	159,6936078	259,6451299	0,232286657	False	0,232286657

ENSMUSG00000055531	Cpsf6	441,2909336	281,4247213	274,7454905	245,163426	375,7678262	-0,231888561	False	0,231888561
ENSMUSG00000043940	Wdfy3	464,4760964	330,6424977	339,0288905	545,4323927	185,2744143	0,231796031	False	0,231796031
ENSMUSG00000002948	Map2k7	455,2020313	772,3404909	534,5208742	807,4648618	747,6214042	0,231739294	False	0,231739294
ENSMUSG00000035621	Midn	566,4908131	1477,795286	731,7740468	482,4546319	1492,63331	-0,231659276	False	0,231659276
ENSMUSG00000028134	Ptbp2	178,5257542	521,2036319	209,581496	646,6466512	1183,407703	0,231379133	False	0,231379133
ENSMUSG00000002910	Arrdc2	254,263953	524,9896147	427,0883426	890,6854743	216,5883998	-0,231370969	False	0,231370969
ENSMUSG00000026425	Srgap2	404,1946729	117,3654668	344,3124577	274,4031007	95,24670595	-0,231331928	False	0,231331928
ENSMUSG00000042548	Asxl1	346,2317657	1571,182861	294,9991645	1295,542508	1393,472355	-0,231027225	False	0,231027225
ENSMUSG00000024646	Cyb5a	14786,72405	5560,346737	17353,87622	1064,998919	8398,671865	0,230955481	False	0,230955481
ENSMUSG00000024286	Ccny	805,2979908	358,4063716	258,0141946	258,6586605	686,2981826	-0,2306872	False	0,2306872
ENSMUSG00000030726	Pold3	156,1134301	439,1740046	183,1636603	448,7165458	413,6055587	0,230538647	False	0,230538647
ENSMUSG00000026490	Cdc42bpa	395,6934466	641,0930872	337,2677015	692,7553689	617,1464645	-0,230489007	False	0,230489007
ENSMUSG00000020024	Cep83	104,333233	174,1552087	122,4026384	194,5562968	163,0936746	0,230435887	False	0,230435887
ENSMUSG00000021400	Wrnip1	710,238823	251,136859	324,9393782	157,444402	605,40372	-0,230406683	False	0,230406683
ENSMUSG00000033377	Palmd	511,619261	484,6057982	460,5509344	492,5760578	600,1847224	0,230336017	False	0,230336017
ENSMUSG00000015882	Lcorl	156,1134301	99,69754702	58,9998329	95,59124409	133,0844384	-0,230252768	False	0,230252768
ENSMUSG00000053536	Cstf2t	283,6318259	131,2474037	133,8503672	35,98729189	332,7110961	0,230250557	False	0,230250557
ENSMUSG00000008683	Rps15a	718,7400494	1484,105257	1522,547927	612,908565	1458,709825	-0,229798212	False	0,229798212
ENSMUSG00000004897	Hdgf	3437,586817	2146,652247	2931,49916	2018,662155	2433,357624	-0,229757506	False	0,229757506
ENSMUSG00000039069	Mtg2	212,5306598	145,1293406	249,2082494	219,29756	97,85620474	0,229680846	False	0,229680846
ENSMUSG00000047180	Neurl3	74,96535999	122,4134438	107,4325315	257,5340576	63,93272043	-0,229669625	False	0,229669625
ENSMUSG00000029634	Rnf6	373,2811224	220,8489966	178,7606877	75,3483924	318,3588527	-0,229608668	False	0,229608668
ENSMUSG00000029922	Mkrn1	264,3108569	340,7384519	225,4321973	265,4062777	537,5567514	-0,229542104	False	0,229542104
ENSMUSG00000020150	Gamt	2801,540515	763,506531	3284,617563	31,48888041	1259,083168	0,229505047	False	0,229505047
ENSMUSG00000040128	Pnrc1	2296,103964	2873,560944	1958,442214	3488,518108	3933,81943	-0,229481406	False	0,229481406
ENSMUSG00000025873	Faf2	804,5251521	556,5394714	545,0880084	491,4514549	686,2981826	-0,229301991	False	0,229301991
ENSMUSG00000021252	Erg28	993,0978102	374,812297	1164,145957	190,0578853	131,779689	0,229264229	False	0,229264229
ENSMUSG00000029578	Wipi2	792,1597319	672,6429439	655,1623235	675,8863258	654,984197	-0,229010739	False	0,229010739
ENSMUSG00000012405	Rpl15	1094,339688	875,8240207	1275,100866	378,9911677	1282,568657	0,228975429	False	0,228975429
ENSMUSG00000019951	Uhrf1bp1l	1097,431043	936,3997455	1038,22094	1737,511437	1240,816676	-0,228933844	False	0,228933844
ENSMUSG00000000605	Clcn4	920,4509665	943,9717111	998,5941867	1898,329647	785,4591367	-0,228804648	False	0,228804648
ENSMUSG00000020219	Timm13	366,3255736	446,7459702	711,5203729	485,8284405	429,2625515	0,228733898	False	0,228733898
ENSMUSG00000049606	Zfp644	193,9825295	287,7346927	165,5517699	303,6427753	451,4432912	-0,228644291	False	0,228644291
ENSMUSG00000051518	Rps19bp1	119,0171695	224,6349794	283,5514357	139,4507561	204,8456553	0,228586044	False	0,228586044
ENSMUSG00000006476	Nsmf	2088,210337	3305,162983	2869,857543	5991,8841	1782,287676	-0,228536818	False	0,228536818
ENSMUSG00000016028	Celsr1	415,7872544	11,3579484	354,8795919	119,2079044	7,828496379	-0,228515923	False	0,228515923
ENSMUSG00000035725	Prkx	82,69374762	208,2290539	96,86539729	181,0610623	296,178113	0,228203138	False	0,228203138
ENSMUSG00000030850	Ate1	249,6269204	176,6791973	145,2980959	292,3967466	169,6174215	0,228153733	False	0,228153733
ENSMUSG00000037221	Mospd3	540,987134	273,8527558	423,5659645	159,6936078	461,8812864	-0,228072189	False	0,228072189
ENSMUSG00000028800	Hdac1	270,493567	230,9449507	263,2977617	268,7800863	370,5488286	-0,22804528	False	0,22804528
ENSMUSG00000046873	Mbtps2	95,05916782	74,4576617	62,52221098	111,3356843	99,16095413	0,228018389	False	0,228018389
ENSMUSG00000021188	Trip11	360,1428635	748,3625998	421,8047755	744,487101	1453,490828	0,228006112	False	0,228006112
ENSMUSG00000035093	Secisbp2l	712,5573393	702,9308062	757,3112879	608,4101535	913,3245776	-0,227961882	False	0,227961882
ENSMUSG00000020964	Sel1l	2650,836966	4201,178912	3104,095686	10892,90341	8026,818287	0,227725092	False	0,227725092
ENSMUSG00000038683	Pak1ip1	364,779896	478,2958269	329,3423508	311,5149954	824,6016186	-0,227724609	False	0,227724609
ENSMUSG00000033705	Stard9	65,69129484	87,07760436	124,1638274	210,300737	56,10422405	-0,227592808	False	0,227592808
ENSMUSG00000032604	Qars	533,2587463	607,019242	459,6703399	455,464163	1024,228276	-0,22749818	False	0,22749818
ENSMUSG00000050890	Pdik1l	106,6517493	106,0075184	139,1339343	91,0928326	97,85620474	-0,22749818	False	0,22749818
ENSMUSG00000032115	Hyou1	3150,090797	6216,583755	4407,375577	3687,572816	15941,42813	0,227278125	False	0,227278125
ENSMUSG00000001525	Tubb5	1076,564397	1143,366805	1367,563291	1139,222709	919,8483245	-0,226966722	False	0,226966722
ENSMUSG00000026019	Wdr12	132,9282672	204,4430711	160,2682028	113,58489	170,9221709	-0,226876998	False	0,226876998
ENSMUSG00000027427	Polr3f	136,0196223	174,1552087	116,2384768	168,6904307	276,6068721	-0,226727088	False	0,226727088
ENSMUSG00000047539	Fbxo28	139,1109773	218,325008	118,8802603	205,8023255	240,073889	-0,226727088	False	0,226727088
ENSMUSG00000032484	Ngp	15,45677526	579,2553682	13,20891781	283,3999236	550,6042453	-0,226727088	False	0,226727088
ENSMUSG00000035834	Polr3g	200,9380783	45,43179358	171,7159316	59,6039522	24,79023853	-0,226727088	False	0,226727088
ENSMUSG00000054280	Prr14l	194,7553682	193,0851227	171,7159316	166,441225	268,7783757	-0,226650283	False	0,226650283
ENSMUSG00000074102	Rbm15b	143,7480099	480,8198154	168,1935535	282,2753208	3301,015973	0,226580428	False	0,226580428
ENSMUSG00000035495	Tstd2	201,7109171	323,0705321	235,9993316	278,9015122	307,9208576	0,226493605	False	0,226493605
ENSMUSG00000020167	Tcf3	232,6244676	224,6349794	272,1037069	146,1983733	163,0936746	0,226153762	False	0,226153762
ENSMUSG00000002881	Nab1	973,0040024	595,6612936	669,2518359	1138,098106	788,0686355	0,226107281	False	0,226107281
ENSMUSG00000030483	Cyp2b10	4599,936316	34,07384519	3932,73513	101,2142584	52,18997586	-0,226080864	False	0,226080864
ENSMUSG00000031166	Wdr13	350,0959595	133,7713922	409,4764522	335,1316557	105,6847011	0,226030077	False	0,226030077
ENSMUSG00000031955	Bcar1	612,8611389	455,5799301	716,80394	504,9466894	431,8720502	0,22601834	False	0,22601834
ENSMUSG00000047250	Ptgs1	169,2516891	107,2695126	140,0145288	197,9301054	54,79947465	0,225820833	False	0,225820833
ENSMUSG00000038244	Mical2	350,0959595	294,044664	299,4021371	174,3134451	290,9591154	-0,225665893	False	0,225665893
ENSMUSG00000015289	Lage3	177,7529154	87,07760436	207,8203069	80,97140676	27,39973733	0,22546341	False	0,22546341
ENSMUSG00000014547	Wdfy2	271,2664057	143,8673463	303,8051097	317,1380098	95,24670595	0,225400406	False	0,225400406
ENSMUSG00000070394	Tmem256	872,5349632	1317,522014	1765,592014	1020,014805	1041,190018	0,225305244	False	0,225305244
ENSMUSG00000074158	Zfp976	59,50858474	220,8489966	69,56696715	111,3356843	95,24670595	0,225304619	False	0,225304619
ENSMUSG00000074639	Rdh16f2	3182,550025	315,4985665	2722,798258	515,0681152	750,230903	-0,225093099	False	0,225093099
ENSMUSG00000028261	Ndufaf4	320,7280866	417,7201021	551,2521701	274,4031007	502,3285177	-0,225053914	False	0,225053914
ENSMUSG00000068580	Zfyve19	290,5873748	299,0926411	290,5961919	339,6300672	178,7506673	0,224992162	False	0,224992162
ENSMUSG00000032688	Malt1	198,619562	113,579484	169,9547425	163,0674164	127,8654409	-0,224857101	False	0,224857101
ENSMUSG00000022013	Dnajc15	361,688541	286,4726984	422,68537	125,9555216	323,5778503	0,224836287	False	0,224836287
ENSMUSG00000021999	Cpb2	5823,340078	7958,135842	5397,163818	4983,115324	7841,543873	-0,224798911	False	0,224798911
ENSMUSG00000031711	Zfp330	173,8887216	148,9153234	148,820474	193,4316939	366,6345804	-0,224591344	False	0,224591344
ENSMUSG00000025786	Zdhhc3	326,1379579	357,1443773	244,8052768	222,6713686	380,9868238	0,224258743	False	0,224258743
ENSMUSG00000030201	Lrp6	805,2979908	702,9308062	401,5511015	371,1189476	940,7243149	0,22424926	False	0,22424926
ENSMUSG00000007036	Abhd16a	461,3847414	291,5206755	538,9238468	364,3713304	263,5593781	0,224111133	False	0,224111133
ENSMUSG00000031903	Pla2g15	153,7949138	116,1034725	179,6412823	164,1920193	53,49472526	0,224111133	False	0,224111133
ENSMUSG00000050891	Tatdn1	108,1974268	156,487289	201,6561453	167,5658279	92,63720715	-0,224002524	False	0,224002524
ENSMUSG00000038068	Rnf144b	555,6710705	153,9633005	648,9981619	210,300737	193,1029107	0,22398326	False	0,22398326
ENSMUSG00000024283	Wac	717,1943719	392,4802168	259,7753837	337,3808615	837,6491126	0,223981865	False	0,223981865
ENSMUSG00000051934	Spats2	61,82710102	128,7234151	72,20875071	175,438048	177,4459179	0,223934321	False	0,223934321
ENSMUSG00000002010	Idh3g	1206,401309	803,8903475	1408,951233	1293,293302	708,4789223	0,223911779	False	0,223911779
ENSMUSG00000041438	Utp4	186,2541418	387,4322397	217,5068467	226,0451772	853,3061053	0,223788307	False	0,223788307
ENSMUSG00000015846	Rxra	4537,336376	4483,865628	3399,094851	3147,763438	5298,587299	0,223762144	False	0,223762144
ENSMUSG00000030084	Plxna1	124,4270408	100,9595413	145,2980959	107,9618757	120,0369445	0,223715747	False	0,223715747
ENSMUSG00000028878	Fam76a	161,5233014	204,4430711	154,1040411	138,3261532	241,3786384	-0,223668352	False	0,223668352
ENSMUSG00000090553	Snrpe	148,3850425	237,254922	195,4919836	127,0801245	339,2348431	-0,223607264	False	0,223607264
ENSMUSG00000020538	Srebf1	3648,571799	1373,049762	2479,754171	751,2347182	3124,874805	-0,223533454	False	0,223533454
ENSMUSG00000026848	Tor1b	314,5453765	367,2403314	541,5656303	647,7712541	408,3865611	0,223456353	False	0,223456353
ENSMUSG00000066043	Phactr4	560,308103	575,4693854	479,9240139	748,9855125	613,2322164	-0,223414354	False	0,223414354
ENSMUSG00000051674	Dcun1d4	340,0490556	229,6829564	151,4622576	396,9848137	360,1108334	0,223340934	False	0,223340934
ENSMUSG00000029622	Arpc1b	504,6637121	1747,862059	589,1177345	760,2315412	1486,109563	0,22323363	False	0,22323363
ENSMUSG00000020366	Mapk9	773,6116016	523,7276204	592,6401125	903,0561059	540,1662502	0,223206193	False	0,223206193
ENSMUSG00000027905	Ddx20	95,05916782	218,325008	110,9549096	194,5562968	326,1873491	0,22307583	False	0,22307583
ENSMUSG00000039910	Cited2	462,9304189	182,9891686	109,1937206	76,47299527	396,6438165	-0,222951306	False	0,222951306
ENSMUSG00000001151	Pcnt	127,5183959	420,2440906	148,820474	623,0299909	878,0963439	0,222867633	False	0,222867633
ENSMUSG00000032411	Tfdp2	204,0294334	141,3433578	120,6414494	130,4539331	174,8364191	-0,222771556	False	0,222771556
ENSMUSG00000038628	Polr3k	187,0269806	109,7935012	160,2682028	78,72220101	108,2941999	-0,222758186	False	0,222758186
ENSMUSG00000059796	Eif4a1	2107,531306	3655,997389	2385,530557	1806,112212	5594,765412	-0,222666533	False	0,222666533
ENSMUSG00000038712	Mindy1	1547,223203	2459,626825	1326,175348	2056,898652	1660,945982	-0,222409793	False	0,222409793
ENSMUSG00000024164	C3	144086,5133	109695,0656	121223,5223	123501,6382	79280,48758	-0,222405125	False	0,222405125
ENSMUSG00000030157	Clec2d	1064,198976	99,69754702	912,2959236	87,71902399	103,0752023	-0,222194145	False	0,222194145
ENSMUSG00000026509	Capn2	717,9672106	454,3179358	615,5355701	597,1641248	533,6425032	-0,22207573	False	0,22207573
ENSMUSG00000044224	Dnajc21	315,3182152	354,6203888	270,3425179	350,8760959	366,6345804	-0,222020085	False	0,222020085
ENSMUSG00000015950	Ncf1	78,05671504	129,9854094	66,92518359	172,0642394	83,50396138	-0,221973558	False	0,221973558
ENSMUSG00000031916	Cog8	295,9972462	381,1222684	345,1930522	449,8411486	457,9670382	0,221819674	False	0,221819674
ENSMUSG00000049659	Aftph	439,745256	297,8306468	245,6858713	241,7896174	512,7665128	0,221634033	False	0,221634033
ENSMUSG00000033382	Trappc8	579,6290721	318,0225551	298,5215426	309,2657897	675,8601874	0,221594875	False	0,221594875
ENSMUSG00000029610	Aimp2	159,9776239	225,8969736	182,2830658	137,2015503	199,6266577	-0,221573346	False	0,221573346
ENSMUSG00000008036	Ap2s1	653,0487546	1292,282129	1261,891948	761,3561441	995,5237895	0,221380767	False	0,221380767

ENSMUSG00000028953	Abcf2	412,6958993	869,5140494	353,9989974	561,1768329	872,8773463	-0,221333829	False	0,221333829
ENSMUSG00000025509	Pnpla2	867,8979306	4009,355784	1011,803104	3491,891916	6966,057028	0,221331282	False	0,221331282
ENSMUSG00000037210	Fam193a	380,2366713	699,1448234	401,5511015	771,4775699	326,1873491	-0,221196847	False	0,221196847
ENSMUSG00000024542	Cep192	65,69129484	114,8414782	56,35804933	176,5626508	156,5699276	-0,221080525	False	0,221080525
ENSMUSG00000027680	Fxr1	520,1204874	402,5761709	331,9841344	321,6364213	446,2242936	-0,221076807	False	0,221076807
ENSMUSG00000036615	Rfxap	91,19497401	78,2436445	85,41766852	95,59124409	135,6939372	-0,220980745	False	0,220980745
ENSMUSG00000024240	Epc1	202,4837559	344,5244347	235,9993316	293,5213495	424,0435539	0,2209766	False	0,2209766
ENSMUSG00000033565	Rbfox2	384,8737039	229,6829564	330,2229453	112,4602872	225,7216456	-0,220944735	False	0,220944735
ENSMUSG00000051375	Pcdh1	1170,077887	902,3259003	673,6548085	452,0903544	1363,463119	0,22067111	False	0,22067111
ENSMUSG00000030421	Uri1	306,8169888	511,1076778	357,5213755	1036,883848	624,9749609	0,220651131	False	0,220651131
ENSMUSG00000002325	Irf9	683,1894663	408,8861422	796,0574469	511,6943066	263,5593781	0,220586814	False	0,220586814
ENSMUSG00000037553	Zdhhc18	343,1404107	391,2182225	399,7899125	293,5213495	219,1978986	0,220443032	False	0,220443032
ENSMUSG00000058267	Mrps14	206,3479497	219,5870023	240,4023042	70,84998091	211,3694022	0,220371621	False	0,220371621
ENSMUSG00000059475	Zfp426	132,1554284	153,9633005	206,0591179	242,9142203	182,6649155	0,220350817	False	0,220350817
ENSMUSG00000038213	Tapbpl	258,9009855	301,6166296	228,9545754	155,1951963	219,1978986	0,220315517	False	0,220315517
ENSMUSG00000028991	Mtor	401,1033179	720,598726	344,3124577	661,2664885	661,507944	-0,22025552	False	0,22025552
ENSMUSG00000025041	Nt5c2	241,8985328	826,6062443	281,7902467	583,6688904	1633,546244	0,220219659	False	0,220219659
ENSMUSG00000025495	Ptdss2	136,792461	164,0592546	166,4323644	209,1761341	117,4274457	-0,220219081	False	0,220219081
ENSMUSG00000048878	Hexim1	480,7057105	412,672125	303,8051097	187,8086796	318,3588527	-0,220157951	False	0,220157951
ENSMUSG00000005469	Prkaca	697,8734028	417,7201021	812,7887428	221,5467657	386,2058214	0,219915072	False	0,219915072
ENSMUSG00000001506	Col1a1	89,64929649	76,98165023	209,581496	111,3356843	112,2084481	-0,219777664	False	0,219777664
ENSMUSG00000068749	Psma5	612,8611389	507,321695	457,9091508	186,6840767	713,6979199	0,21975334	False	0,21975334
ENSMUSG00000026755	Arpc5l	282,8589872	329,3805035	235,9993316	202,4285169	294,8733636	0,219672152	False	0,219672152
ENSMUSG00000029556	Hnf1a	738,8338572	739,52864	713,2815619	860,3211968	370,5488286	0,219625404	False	0,219625404
ENSMUSG00000000378	Ccm2	205,5751109	169,1072317	142,6563124	176,5626508	112,2084481	-0,219485412	False	0,219485412
ENSMUSG00000003778	Brd8	254,263953	497,2257409	218,3874412	601,6625363	786,7638861	-0,219437052	False	0,219437052
ENSMUSG00000062519	Zfp398	106,6517493	56,78974198	124,1638274	103,4634642	101,7704529	0,219337307	False	0,219337307
ENSMUSG00000076435	Acsf2	2801,540515	2270,327685	2406,664826	1222,443321	1510,899801	-0,219185124	False	0,219185124
ENSMUSG00000034889	Cactin	142,9751711	353,3583945	166,4323644	309,2657897	477,5382791	0,219171375	False	0,219171375
ENSMUSG00000032531	Amotl2	244,9898878	113,579484	210,4620905	203,5531198	52,18997586	-0,219161811	False	0,219161811
ENSMUSG00000078945	Naip2	128,2912346	82,0296273	41,38794248	110,2110814	44,36147948	-0,219153313	False	0,219153313
ENSMUSG00000030335	Mrpl51	513,1649385	802,6283533	975,6987291	440,8443257	567,5659875	-0,219153313	False	0,219153313
ENSMUSG00000044147	Arf6	238,8071777	193,0851227	72,20875071	68,60077517	277,9116215	0,218779966	False	0,218779966
ENSMUSG00000045482	Trrap	449,79216	614,5912076	386,5809947	666,8895029	738,4881584	-0,218487802	False	0,218487802
ENSMUSG00000068299	Nat8f4	109,7431043	37,85982798	127,6862055	80,97140676	41,75198069	0,218472382	False	0,218472382
ENSMUSG00000034484	Snx2	678,5524337	389,9562282	396,2675344	422,8506797	583,2229802	-0,218412735	False	0,218412735
ENSMUSG00000019861	Gopc	311,4540214	170,3692259	267,7007343	242,9142203	78,28496379	-0,218398104	False	0,218398104
ENSMUSG00000020743	Mif4gd	624,4537203	547,7055115	726,4904797	356,4991103	446,2242936	0,218349238	False	0,218349238
ENSMUSG00000026608	Kctd3	110,5159431	1308,688054	128,5668	1196,577455	1046,409016	0,218263633	False	0,218263633
ENSMUSG00000025171	Ubtd1	83,46658638	204,4430711	102,1489644	140,575359	71,76121681	-0,217994415	False	0,217994415
ENSMUSG00000030869	Ndufab1	1004,690392	805,1523418	1168,548929	284,5245265	806,335127	0,217967159	False	0,217967159
ENSMUSG00000042447	Mios	125,9727183	342,0004461	108,3131261	419,4768711	426,6530527	-0,217903238	False	0,217903238
ENSMUSG00000038215	Cep44	125,9727183	161,5352661	108,3131261	201,303914	164,398424	-0,217903238	False	0,217903238
ENSMUSG00000022035	Ccdc25	388,7378977	333,1664863	377,7750494	452,0903544	322,2731009	0,217813376	False	0,217813376
ENSMUSG00000030264	Thumpd3	187,7998194	321,8085379	218,3874412	553,3046128	452,7480406	0,217694218	False	0,217694218
ENSMUSG00000047945	Marcksl1	150,7035587	128,7234151	175,2383097	140,575359	140,9129348	0,217604718	False	0,217604718
ENSMUSG00000051439	Cd14	37,86909938	6287,255434	44,02972604	1518,213877	23052,31234	0,217456757	False	0,217456757
ENSMUSG00000063316	Rpl27	971,4583248	2476,03275	2092,292582	835,5799336	2190,674237	-0,21737426	False	0,21737426
ENSMUSG00000020993	Trappc6b	274,3577608	397,5281938	235,9993316	292,3967466	574,0897345	-0,217275613	False	0,217275613
ENSMUSG00000020865	Abcc3	3209,599382	585,5653395	2060,591179	2760,90005	921,1530739	-0,217254573	False	0,217254573
ENSMUSG00000024927	Rela	782,112828	1465,175343	672,7742139	1140,347312	2066,723044	-0,217254335	False	0,217254335
ENSMUSG00000005534	Insr	1367,92461	1049,979229	1324,414159	1590,18846	728,0501633	0,217209034	False	0,217209034
ENSMUSG00000024392	Bag6	1190,944533	1834,939663	1384,294587	1806,112212	2223,292972	0,217044767	False	0,217044767
ENSMUSG00000004221	Ikbkg	752,744955	624,6871617	662,2070797	874,9410341	246,5976359	0,217024657	False	0,217024657
ENSMUSG00000009733	Tfcp2	115,9258144	113,579484	134,7309617	131,578536	36,5329831	0,216879564	False	0,216879564
ENSMUSG00000032182	Yipf2	372,5082837	138,8193693	320,5364056	210,300737	71,76121681	-0,216784285	False	0,216784285
ENSMUSG00000028344	Invs	218,7133699	29,02586812	238,6411152	254,160249	39,1424819	0,216696994	False	0,216696994
ENSMUSG00000066406	Akap13	622,1352041	704,1928005	535,4014687	886,1870628	1082,941999	-0,216607048	False	0,216607048
ENSMUSG00000026672	Optn	1013,964457	1418,481555	872,6691702	2058,023255	1473,062069	-0,216500346	False	0,216500346
ENSMUSG00000029661	Col1a2	120,562847	140,0813635	202,5367398	143,9491676	160,4841758	0,216479641	False	0,216479641
ENSMUSG00000024983	Vti1a	157,6591076	135,0333865	95,10420825	115,8340958	135,6939372	-0,216452251	False	0,216452251
ENSMUSG00000059070	Rpl18	2086,66466	5131,268686	4908,433859	1795,990786	5105,484389	-0,21641882	False	0,21641882
ENSMUSG00000035469	Rcbtb1	874,8534795	547,7055115	487,8493646	537,5601726	1016,39978	0,216354648	False	0,216354648
ENSMUSG00000026753	Ppp6c	455,2020313	576,7313796	391,8645618	432,9721056	794,5923825	-0,216151887	False	0,216151887
ENSMUSG00000058013	Sept11	173,8887216	290,2586812	149,7010685	290,1475409	605,40372	-0,216079844	False	0,216079844
ENSMUSG00000047798	Cd300lf	34,77774433	295,3066583	29,94021371	205,8023255	99,16095413	-0,216079844	False	0,216079844
ENSMUSG00000037536	Fbxo34	184,7084643	214,5390252	126,805611	95,59124409	185,2744143	0,215990122	False	0,215990122
ENSMUSG00000051427	Ccdc157	201,7109171	49,21777638	234,2381425	152,9459905	56,10422405	0,21568685	False	0,21568685
ENSMUSG00000039128	Cdc123	427,3798358	567,8974198	368,0885097	441,9689285	420,1293057	-0,215466127	False	0,215466127
ENSMUSG00000033985	Tesk2	129,8369122	60,57572477	111,8355041	62,97776081	6,523746983	-0,215322325	False	0,215322325
ENSMUSG00000059119	Nap1l4	448,2464824	265,0187959	256,2530056	258,6586605	386,2058214	-0,214922351	False	0,214922351
ENSMUSG00000027808	Serp1	3109,130343	5749,645877	4482,226111	2678,80404	6231,483118	-0,21492205	False	0,21492205
ENSMUSG00000033809	Alg3	179,298593	262,4948074	466,7150961	208,0515312	357,5013346	0,21457674	False	0,21457674
ENSMUSG00000022453	Naga	392,6020915	318,0225551	324,9393782	455,464163	317,0541034	0,214269685	False	0,214269685
ENSMUSG00000034893	Cog3	177,7529154	200,6570883	153,2234466	329,5086414	431,8720502	-0,214236144	False	0,214236144
ENSMUSG00000060279	Ap2a1	513,1649385	1076,481109	595,2818961	1166,213178	891,1438378	0,214150416	False	0,214150416
ENSMUSG00000021686	Ap3b1	615,952494	306,6646067	350,4766193	256,4094547	531,0330044	-0,214017557	False	0,214017557
ENSMUSG00000049517	Rps23	1374,880159	3033,834216	2259,605541	1185,331427	2849,572682	-0,214015369	False	0,214015369
ENSMUSG00000026240	Cops7b	129,0640734	118,627461	149,7010685	98,9650527	101,7704529	0,213997055	False	0,213997055
ENSMUSG00000020691	Mettl2	142,9751711	116,1034725	123,2832329	104,5880671	112,2084481	-0,213788032	False	0,213788032
ENSMUSG00000024843	Chka	1217,221051	1892,991399	1411,593017	2938,587304	3514,994874	0,213733008	False	0,213733008
ENSMUSG00000031930	Wwp2	261,9923406	307,9266009	303,8051097	503,8220865	495,8047707	0,2136215	False	0,2136215
ENSMUSG00000071369	Map3k5	225,6689187	507,321695	194,6113891	602,7871392	293,5686142	-0,213611588	False	0,213611588
ENSMUSG00000017781	Pitpna	833,1201863	1505,55916	966,0121894	977,2798954	1037,27577	0,213516759	False	0,213516759
ENSMUSG00000022247	Brix1	258,1281468	360,9303601	226,3127919	222,6713686	803,7256283	-0,213171398	False	0,213171398
ENSMUSG00000013822	Elof1	410,377383	906,1118831	702,7144276	475,7070147	715,0026693	0,213122077	False	0,213122077
ENSMUSG00000029387	Gtf2h3	134,4739447	42,90780505	155,8652302	75,3483924	20,87599034	0,212972466	False	0,212972466
ENSMUSG00000041126	H2afv	241,8985328	73,19566744	208,7009014	71,97458378	133,0844384	-0,212965187	False	0,212965187
ENSMUSG00000008167	Fbxw9	244,9898878	251,136859	360,163159	601,6625363	211,3694022	-0,212955654	False	0,212955654
ENSMUSG00000026618	Iars2	698,6462416	474,5098441	376,0138604	602,7871392	403,1675635	-0,212913498	False	0,212913498
ENSMUSG00000028809	Srrm1	859,3967022	1167,344696	995,9524031	1791,492374	1677,907724	0,212752553	False	0,212752553
ENSMUSG00000017760	Ctsa	2084,346143	2415,457025	3201,841678	3633,591878	2432,052875	0,212701302	False	0,212701302
ENSMUSG00000034109	Golim4	1102,068076	494,7017523	951,0420825	748,9855125	644,5462019	-0,212632258	False	0,212632258
ENSMUSG00000020085	Aifm2	274,3577608	494,7017523	317,894622	565,6752444	395,3390671	0,212490224	False	0,212490224
ENSMUSG00000026383	Epb41l5	541,7599727	188,0371457	467,5956906	203,5531198	156,5699276	-0,212392172	False	0,212392172
ENSMUSG00000014498	Ankrd52	294,4515686	181,7271743	182,2830658	254,160249	183,9696649	-0,212291951	False	0,212291951
ENSMUSG00000019809	Pex3	411,1502218	283,9487099	186,6860384	184,4348709	354,8918359	-0,212286221	False	0,212286221
ENSMUSG00000025572	Tmc6	114,3801369	132,5093979	136,4921507	263,157072	150,0461806	0,212258146	False	0,212258146
ENSMUSG00000032601	Prkar2a	1266,682732	1106,768971	1467,070472	1256,181408	1095,989493	0,211882958	False	0,211882958
ENSMUSG00000035954	Dock4	378,6909938	266,2807902	267,7007343	438,5951199	78,28496379	0,211868649	False	0,211868649
ENSMUSG00000038866	Zcchc2	615,952494	212,0150367	531,8790906	287,8983351	187,8839131	-0,211720763	False	0,211720763
ENSMUSG00000038495	Otud7b	283,6318259	473,2478498	328,4617563	570,1736559	407,0818117	0,211705979	False	0,211705979
ENSMUSG00000041375	Ccdc9	236,4886614	994,4514817	273,864896	1510,341657	1214,721688	0,211693339	False	0,211693339
ENSMUSG00000033439	Tmtc13	95,05916782	145,1293406	110,0743151	141,6999618	160,4841758	0,211580191	False	0,211580191
ENSMUSG00000021236	Entpd5	2599,056759	559,0634599	2244,635434	898,5576944	879,4010932	-0,211506994	False	0,211506994
ENSMUSG00000074781	Ube2n	349,3231208	261,2328131	287,0738138	112,4602872	404,4723129	0,211478816	False	0,211478816
ENSMUSG00000045374	Wdr81	745,0165673	489,6537753	576,7894112	862,5704025	644,5462019	0,211369706	False	0,211369706
ENSMUSG00000029428	Stx2	192,4368519	53,00375918	222,7904138	199,0547083	14,35224336	0,211302054	False	0,211302054
ENSMUSG00000030045	Mrpl19	169,2516891	234,7309335	230,7157645	146,1983733	263,5593781	-0,211242972	False	0,211242972
ENSMUSG00000060803	Gstp1	2466,901331	676,4289266	2131,03874	783,8482015	1069,894505	-0,211143193	False	0,211143193
ENSMUSG00000043760	Pkhd1	123,654202	87,07760436	102,1489644	106,8372728	22,18073974	-0,210896212	False	0,210896212
ENSMUSG00000002968	Med25	634,5006243	974,2595735	866,5050085	734,3656752	1110,341736	0,210877044	False	0,210877044
ENSMUSG00000029992	Gfpt1	348,550282	386,1702454	301,1633261	468,9593975	716,3074187	-0,210820697	False	0,210820697

ENSMUSG00000024645	Timm21	300,6342787	169,1072317	236,8799261	259,7832633	116,1226963	-0,210701029	False	0,210701029
ENSMUSG00000025915	Sgk3	163,068979	94,64956996	100,3877754	89,96822973	140,9129348	-0,210678313	False	0,210678313
ENSMUSG00000026571	Dcaf6	662,3228197	348,3104175	198,1337672	572,4228617	199,6266577	-0,210453274	False	0,210453274
ENSMUSG00000046897	Zfp740	351,6416371	550,2295	406,8346686	1143,72112	614,5369658	0,210336718	False	0,210336718
ENSMUSG00000008348	Ubc	2407,392746	1066,385155	2080,844853	594,9149191	1159,922213	-0,210302123	False	0,210302123
ENSMUSG00000054737	Zfp182	96,60484535	204,4430711	136,4921507	260,9078662	83,50396138	-0,210250911	False	0,210250911
ENSMUSG00000042595	Fam199x	44,82464824	196,8711055	38,74615892	197,9301054	307,9208576	-0,210238965	False	0,210238965
ENSMUSG00000029185	Fam114a1	471,4316453	310,4505895	493,1329317	431,8475027	545,3852477	0,210227073	False	0,210227073
ENSMUSG00000034473	Sec22a	183,9356255	159,0112775	122,4026384	130,4539331	122,6464433	-0,210071846	False	0,210071846
ENSMUSG00000026185	Igfbp5	241,125694	151,4393119	164,6711754	278,9015122	50,88522646	0,209970366	False	0,209970366
ENSMUSG00000052911	Lamb2	63,37277855	82,0296273	160,2682028	200,1793111	54,79947465	-0,209701209	False	0,209701209
ENSMUSG00000034525	Ice1	207,1207884	1031,049315	239,5217097	968,2830725	1696,174215	0,209684062	False	0,209684062
ENSMUSG00000021703	Serinc5	377,1453162	146,3913349	118,8802603	80,97140676	326,1873491	-0,20941968	False	0,20941968
ENSMUSG00000040761	Spen	532,4859076	892,2299462	460,5509344	834,4553307	761,9736476	-0,209382625	False	0,209382625
ENSMUSG00000032582	Rbm6	423,515642	1079,005098	489,6105536	798,4680388	1405,2151	0,209219401	False	0,209219401
ENSMUSG00000025060	Slk	500,7995183	1097,935012	433,2525043	1340,526623	1852,744143	-0,209025086	False	0,209025086
ENSMUSG00000025616	Usp16	375,5996387	439,1740046	324,9393782	828,8323164	712,3931705	-0,209025086	False	0,209025086
ENSMUSG00000047888	Tnrc6b	369,4169286	372,2883085	319,6558111	804,0910532	529,728255	-0,208730659	False	0,208730659
ENSMUSG00000028461	Ccdc107	652,2759158	288,9966869	753,7889099	253,0356461	275,3021227	0,208678209	False	0,208678209
ENSMUSG00000054452	Aes	13843,08792	3710,263142	15995,11888	3228,734844	4800,17303	0,20846592	False	0,20846592
ENSMUSG00000027810	Eif2a	626,7722366	475,7718383	398,0287234	447,5919429	724,1359151	0,20831923	False	0,20831923
ENSMUSG00000025153	Fasn	16521,74707	6244,347629	8628,065115	3724,684711	19084,56942	0,208040387	False	0,208040387
ENSMUSG00000091586	Cyp4f17	225,6689187	747,1006056	260,6559782	861,4457997	597,5752236	0,207939218	False	0,207939218
ENSMUSG00000032012	Nectin1	760,4733426	878,3480092	595,2818961	146,1983733	212,6741516	0,207894984	False	0,207894984
ENSMUSG00000028495	Rps6	2182,496666	2147,914241	2111,665661	838,9537422	2520,775834	0,207888379	False	0,207888379
ENSMUSG00000026466	Tor1aip1	688,5993377	427,8160562	418,2823974	455,464163	596,2704742	-0,207697893	False	0,207697893
ENSMUSG00000048170	Mcmbp	367,0984123	624,6871617	317,894622	738,8640867	388,8153202	-0,207618265	False	0,207618265
ENSMUSG00000039623	Ap5z1	216,3948536	249,8748647	312,6110549	580,2950818	271,3878745	0,207539597	False	0,207539597
ENSMUSG00000028399	Ptprd	1794,531607	666,3329725	2072,038908	820,9600963	499,7190189	0,20744376	False	0,20744376
ENSMUSG00000019927	Ube2d1	177,7529154	65,62370184	139,1339343	44,98411486	153,9604288	-0,207313628	False	0,207313628
ENSMUSG00000028988	Ctnnbip1	132,1554284	65,62370184	114,4772877	56,23014358	57,40897345	-0,207174291	False	0,207174291
ENSMUSG00000038612	Mcl1	1183,988985	1465,175343	1059,355209	1025,637819	1778,373427	-0,207134293	False	0,207134293
ENSMUSG00000031167	Rbm3	299,0886012	1158,510736	507,222444	345,2530816	1466,538322	0,207081364	False	0,207081364
ENSMUSG00000057230	Aak1	234,9429839	326,8565149	285,3126248	389,1125936	203,5409059	-0,206991921	False	0,206991921
ENSMUSG00000027087	Itgav	197,8467233	128,7234151	108,3131261	71,97458378	228,3311444	0,206744485	False	0,206744485
ENSMUSG00000047123	Ticam1	319,9552478	239,7789106	159,3876083	192,307091	369,2440792	0,206704659	False	0,206704659
ENSMUSG00000020022	Ndufa12	731,8783084	1426,053521	1264,533732	634,2760196	1114,255985	-0,206492989	False	0,206492989
ENSMUSG00000003360	Ddx23	490,7526144	576,7313796	425,3271536	572,4228617	596,2704742	-0,206422991	False	0,206422991
ENSMUSG00000029020	Mfn2	1249,680293	523,7276204	892,9228441	1441,740881	511,4617634	0,206252858	False	0,206252858
ENSMUSG00000039089	L3mbtl3	92,74065154	323,0705321	179,6412823	441,9689285	106,9894505	0,206194782	False	0,206194782
ENSMUSG00000066892	Fbxl12	125,1998796	108,5315069	137,3727453	127,0801245	163,0936746	-0,206119254	False	0,206119254
ENSMUSG00000052337	Immt	1565,771333	1225,396432	771,4008003	1806,112212	1079,027751	0,206013993	False	0,206013993
ENSMUSG00000070808	Bicra	200,1652396	227,1589679	346,0736467	242,9142203	173,5316697	-0,205992479	False	0,205992479
ENSMUSG00000022875	Kng1	36856,6806	12831,9577	42513,34228	24759,25682	15949,25662	0,20598954	False	0,20598954
ENSMUSG00000001630	Stk38l	248,8540816	206,9670596	265,9395453	235,0420002	287,0448672	0,205976209	False	0,205976209
ENSMUSG00000042851	Zc3h6	35,55058309	330,6424977	30,82080823	572,4228617	213,978901	-0,205968528	False	0,205968528
ENSMUSG00000035529	Prdm4	71,10116618	232,206945	61,64161646	218,1729571	417,5198069	-0,205968528	False	0,205968528
ENSMUSG00000022913	Psmg1	265,0836956	630,9971331	229,8351699	269,9046892	1156,007965	-0,205848358	False	0,205848358
ENSMUSG00000020078	Vps26a	599,7228799	637,3071044	486,0881755	396,9848137	691,5171802	0,20546908	False	0,20546908
ENSMUSG00000036315	Znrd1	156,8862688	205,7050654	199,0143617	136,0769475	253,1213829	-0,205296406	False	0,205296406
ENSMUSG00000039745	Htatip2	588,9031373	141,3433578	678,9383756	182,1856652	103,0752023	0,205250274	False	0,205250274
ENSMUSG00000003464	Pex19	1198,672921	1005,80943	776,6843674	995,2735414	1381,729611	0,205037276	False	0,205037276
ENSMUSG00000014601	Strip1	265,8565344	559,0634599	306,4468933	644,3974454	579,3087321	0,204989152	False	0,204989152
ENSMUSG00000027804	Ppid	579,6290721	357,1443773	501,0582824	511,6943066	668,031691	0,204786588	False	0,204786588
ENSMUSG00000033931	Rbm34	116,6986532	196,8711055	101,2683699	151,8213877	233,550142	-0,204604277	False	0,204604277
ENSMUSG00000039936	Pik3cd	68,78264989	206,9670596	79,25350688	289,022938	136,9986866	0,204430077	False	0,204430077
ENSMUSG00000025050	Pcgf6	114,3801369	261,2328131	181,4024713	300,2689667	131,779689	0,20429149	False	0,20429149
ENSMUSG00000044308	Ubr3	1449,07268	917,4698315	685,9831317	626,4037995	1257,778418	-0,204252171	False	0,204252171
ENSMUSG00000016664	Pacsin2	736,515341	422,7680792	623,4609208	272,1538949	639,3272043	-0,204161092	False	0,204161092
ENSMUSG00000038302	Afg1l	129,8369122	13,88193693	112,7160987	59,6039522	10,43799517	-0,204007011	False	0,204007011
ENSMUSG00000000266	Mid2	97,37768411	84,55361583	84,537074	163,0674164	168,3126721	-0,204007011	False	0,204007011
ENSMUSG00000071659	Hnrnpul2	775,9301179	666,3329725	617,2967591	673,6371201	503,6332671	-0,203955087	False	0,203955087
ENSMUSG00000020783	Ncbp3	165,3874952	137,557375	73,96993975	155,1951963	190,4934119	0,203890946	False	0,203890946
ENSMUSG00000035754	Wdr18	640,6833344	420,2440906	737,9382085	400,3586223	497,1095201	0,203888554	False	0,203888554
ENSMUSG00000032966	Fkbp1a	745,0165673	1307,42606	925,5048414	858,071991	1850,134644	0,203826185	False	0,203826185
ENSMUSG00000021067	Sav1	216,3948536	477,0338326	249,2082494	381,2403735	1350,415625	0,203685637	False	0,203685637
ENSMUSG00000024429	Gnl1	207,8936272	667,5949668	180,5218768	542,0585841	1424,786341	-0,203671852	False	0,203671852
ENSMUSG00000026155	Smap1	506,9822284	1039,883275	583,8341673	1113,356843	2101,951278	0,203623467	False	0,203623467
ENSMUSG00000024515	Smad4	303,7256338	263,7568016	119,7608548	195,6808997	211,3694022	-0,203560376	False	0,203560376
ENSMUSG00000052837	Junb	802,2066358	4403,097995	923,7436524	1515,964671	3629,812821	0,203518646	False	0,203518646
ENSMUSG00000022184	Fbxo4	139,8838161	318,0225551	121,5220439	147,3229762	341,8443419	-0,203011019	False	0,203011019
ENSMUSG00000057406	Nsd2	185,4813031	333,1664863	161,1487973	372,2435505	354,8918359	-0,202880346	False	0,202880346
ENSMUSG00000066637	Ttc32	156,1134301	151,4393119	179,6412823	52,85633497	91,33245776	0,202524271	False	0,202524271
ENSMUSG00000038417	Fig4	208,666466	205,7050654	195,4919836	85,46981824	240,073889	0,202279732	False	0,202279732
ENSMUSG00000058756	Thra	238,0343389	88,33959863	206,9397124	128,2047274	54,79947465	-0,201959183	False	0,201959183
ENSMUSG00000020154	Ptprb	711,7845005	306,6646067	723,8486961	818,7108905	198,3219083	0,201913568	False	0,201913568
ENSMUSG00000020775	Mrpl38	954,4558721	1409,647595	1229,309951	1306,788537	829,8206162	-0,201878998	False	0,201878998
ENSMUSG00000042599	Kdm7a	163,068979	266,2807902	141,7757179	434,0967084	281,8258696	-0,201871899	False	0,201871899
ENSMUSG00000020111	Micu1	397,2391241	456,8419243	382,1780221	309,2657897	305,3113588	0,201687332	False	0,201687332
ENSMUSG00000046311	Zfp62	264,3108569	174,1552087	229,8351699	160,8182106	183,9696649	-0,201636107	False	0,201636107
ENSMUSG00000019373	Cops3	569,5821682	474,5098441	313,4916494	322,7610242	654,984197	0,201556118	False	0,201556118
ENSMUSG00000021557	Agtpbp1	130,6097509	60,57572477	100,3877754	113,58489	27,39973733	-0,201491679	False	0,201491679
ENSMUSG00000028514	Usp24	821,5276049	571,6834026	471,1180687	524,0649382	944,6385631	0,201453379	False	0,201453379
ENSMUSG00000038949	Cnst	254,263953	381,1222684	292,3573809	401,4832252	293,5686142	0,201406069	False	0,201406069
ENSMUSG00000023067	Cdkn1a	130,6097509	6827,38898	113,5966932	3193,872155	12730,43986	-0,20134177	False	0,20134177
ENSMUSG00000040731	Eif4h	1368,697449	1046,193247	776,6843674	446,46734	1573,527772	0,201199068	False	0,201199068
ENSMUSG00000031626	Sorbs2	1058,016266	415,1961136	1216,101033	1069,497331	83,50396138	0,200901284	False	0,200901284
ENSMUSG00000046679	C87436	170,0245278	235,9929278	147,9398795	299,1443639	204,8456553	-0,200731879	False	0,200731879
ENSMUSG00000022160	Mettl3	175,4343992	316,7605608	191,9696055	322,7610242	152,6556794	-0,200650395	False	0,200650395
ENSMUSG00000043733	Ptpn11	481,4785492	506,0597007	270,3425179	389,1125936	553,2137441	0,200365475	False	0,200365475
ENSMUSG00000068220	Lgals1	187,0269806	147,6533291	214,8650631	46,10871774	78,28496379	0,200184512	False	0,200184512
ENSMUSG00000005198	Polr2a	602,0413962	691,5728578	469,3568796	683,7585459	430,5673008	0,200018559	False	0,200018559
ENSMUSG00000018661	Cog1	275,1305996	342,0004461	239,5217097	253,0356461	327,4920985	-0,199960178	False	0,199960178
ENSMUSG00000068877	Selenbp2	487,6612593	0	560,0581153	12,37063159	61,32322164	0,199697172	False	0,199697172
ENSMUSG00000039087	Rreb1	530,94023	908,6358716	462,3121234	470,0840003	1261,692666	-0,199682265	False	0,199682265
ENSMUSG00000024081	Cebpz	464,4760964	401,3141766	236,8799261	311,5149954	404,4723129	-0,199563402	False	0,199563402
ENSMUSG00000026814	Eng	1099,749559	1169,868685	1814,905307	1767,875714	957,686057	-0,19955032	False	0,19955032
ENSMUSG00000044030	Irf2bp1	169,2516891	194,347117	158,5070138	70,84998091	106,9894505	0,199465475	False	0,199465475
ENSMUSG00000017264	Exosc10	390,2835752	613,3292133	339,9094851	522,9403353	776,3258909	-0,199372129	False	0,199372129
ENSMUSG00000020079	Supv3l1	200,1652396	223,3729851	174,3577151	190,0578853	397,9485659	-0,199141256	False	0,199141256
ENSMUSG00000022856	Tmem41a	77,28387628	140,0813635	93,34301921	113,58489	88,72295896	0,199140025	False	0,199140025
ENSMUSG00000003072	Atp5d	4164,055254	4529,297421	6391,355032	5402,592195	3627,203322	-0,199131595	False	0,199131595
ENSMUSG00000079641	Rpl39	779,7943117	1267,042243	1644,06997	679,2601345	1197,759946	-0,199129445	False	0,199129445
ENSMUSG00000026289	Atg16l1	392,6020915	342,0004461	431,4913152	515,0681152	523,204508	-0,199069651	False	0,199069651
ENSMUSG00000022505	Emp2	292,1330523	58,05173624	254,4918165	210,300737	41,75198069	-0,19900633	False	0,19900633
ENSMUSG00000028573	Fggy	1273,638281	353,3583945	1109,549096	501,5728807	259,6451299	-0,198982097	False	0,198982097
ENSMUSG00000048118	Arid4a	265,0836956	230,9449507	263,2977617	374,4927562	233,550142	-0,198898934	False	0,198898934
ENSMUSG00000052534	Pbx1	211,757821	156,487289	243,0440878	193,4316939	198,3219083	0,198802785	False	0,198802785
ENSMUSG00000022893	Adamts1	91,96781277	365,9783372	80,1341014	230,5435887	485,3667755	-0,198712712	False	0,198712712
ENSMUSG00000033545	Znrf1	472,9773228	1126,96088	951,0420825	1364,143283	542,7757489	0,198585249	False	0,198585249
ENSMUSG00000031314	Taf1	286,723181	249,8748647	321,4170001	480,2054262	292,2638648	-0,198452763	False	0,198452763
ENSMUSG00000039713	Plekhg5	85,78510267	98,43555276	160,2682028	197,9301054	103,0752023	0,198452346	False	0,198452346
ENSMUSG00000029632	Ndufa4	2301,513836	1568,658873	2005,994319	628,6530052	1873,620133	-0,198265596	False	0,198265596

ENSMUSG00000023845	Lnpep	219,4862086	170,3692259	91,58183017	107,9618757	251,8166335	0,198243291	False	0,198243291
ENSMUSG00000031819	Emc8	226,4417575	189,2991399	259,7753837	141,6999618	257,0356311	0,198124701	False	0,198124701
ENSMUSG00000009654	Oit3	269,7207282	36,59783372	235,1187371	197,9301054	20,87599034	-0,198076883	False	0,198076883
ENSMUSG00000022579	Gpihbp1	180,8442705	395,0042053	246,5664658	768,1037613	207,455154	0,198051609	False	0,198051609
ENSMUSG00000047412	Zbtb44	399,5576404	348,3104175	271,2231124	318,2626127	200,9314071	-0,19803002	False	0,19803002
ENSMUSG00000004394	Tmed4	615,1796552	942,7097168	536,2820632	855,8227853	917,2388257	-0,198015791	False	0,198015791
ENSMUSG00000035992	Fnip1	332,320668	393,742211	289,7155974	903,0561059	983,781045	-0,197938665	False	0,197938665
ENSMUSG00000024740	Ddb1	1959,919102	1455,079389	862,9826304	1857,843944	2248,08321	0,197901329	False	0,197901329
ENSMUSG00000025354	Dnajc14	455,9748701	397,5281938	317,894622	309,2657897	280,5211202	-0,197897132	False	0,197897132
ENSMUSG00000025224	Gbf1	1047,969362	1442,459446	1202,011521	2305,435887	2198,502733	0,197854184	False	0,197854184
ENSMUSG00000004880	Lbr	116,6986532	165,3212489	133,8503672	165,3166221	232,2453926	0,197833185	False	0,197833185
ENSMUSG00000024491	Rbm27	190,8911744	252,3988532	166,4323644	185,5594738	382,2915732	-0,197814396	False	0,197814396
ENSMUSG00000021235	Coq6	137,5652998	157,7492833	165,5517699	210,300737	287,0448672	0,197516848	False	0,197516848
ENSMUSG00000024841	Eif1ad	217,9405311	524,9896147	243,9246823	190,0578853	677,1649368	-0,197495642	False	0,197495642
ENSMUSG00000039960	Rhou	1283,685185	730,6946801	590,8789235	804,0910532	1119,474982	-0,197469147	False	0,197469147
ENSMUSG00000040029	Ipo8	513,1649385	300,3546353	270,3425179	235,0420002	447,529043	-0,197441292	False	0,197441292
ENSMUSG00000036591	Arhgap21	575,7648783	475,7718383	426,2077481	536,4355698	660,2031946	0,197430333	False	0,197430333
ENSMUSG00000026411	Tmem9	279,7676321	160,2732718	221,0292247	113,58489	243,9881371	-0,197418054	False	0,197418054
ENSMUSG00000021916	Glt8d1	136,0196223	118,627461	239,5217097	269,9046892	139,6081854	-0,197376773	False	0,197376773
ENSMUSG00000021918	Nek4	178,5257542	180,4651801	146,1786905	204,6777226	125,2559421	0,197221873	False	0,197221873
ENSMUSG00000005813	Metap1	300,6342787	208,2290539	198,1337672	232,7927944	262,2546287	-0,197041281	False	0,197041281
ENSMUSG00000028906	Epb41	1550,314558	4891,489776	1777,039743	5575,781037	3529,347118	0,19691498	False	0,19691498
ENSMUSG00000030655	Smg1	552,5797154	482,0818097	494,0135262	647,7712541	857,2203535	-0,19690461	False	0,19690461
ENSMUSG00000031671	Setd6	168,4788503	258,7088246	268,5813289	295,7705552	193,1029107	0,196802414	False	0,196802414
ENSMUSG00000046603	Tcaim	261,9923406	200,6570883	275,626085	300,2689667	106,9894505	0,196730743	False	0,196730743
ENSMUSG00000034327	Kctd9	122,1085245	103,4835298	106,551937	103,4634642	53,49472526	-0,1966071	False	0,1966071
ENSMUSG00000049323	Smcr8	255,0367917	201,9190826	182,2830658	89,96822973	292,2638648	0,196566081	False	0,196566081
ENSMUSG00000026623	Lpgat1	1875,679677	1636,806563	1525,18971	1608,182106	1309,968394	-0,196529635	False	0,196529635
ENSMUSG00000059434	Gckr	3954,615949	7262,777002	3451,049927	5282,259688	5637,822142	-0,196502249	False	0,196502249
ENSMUSG00000110185	Igip	181,6171093	40,38381652	158,5070138	112,4602872	10,43799517	-0,196353439	False	0,196353439
ENSMUSG00000040044	Orc3	336,9577006	436,6500161	294,11857	512,8189095	373,1583274	-0,196169621	False	0,196169621
ENSMUSG00000021629	Slc30a5	425,8341583	565,3734312	487,8493646	1504,718642	1625,717748	0,196144069	False	0,196144069
ENSMUSG00000030534	Vps33b	95,83200659	166,5832431	83,65647948	125,9555216	122,6464433	-0,196030291	False	0,196030291
ENSMUSG00000036820	Amdhd2	214,8491761	64,36170757	187,5666329	152,9459905	46,97097827	-0,195921041	False	0,195921041
ENSMUSG00000043067	Dpy19l1	300,6342787	522,4656262	344,3124577	671,3879144	568,8707369	0,195708864	False	0,195708864
ENSMUSG00000028568	Btf3l4	246,5355653	170,3692259	94,22361373	52,85633497	215,2836504	-0,195557026	False	0,195557026
ENSMUSG00000031948	Kars	708,6931455	637,3071044	522,1925509	362,1221247	811,5541246	0,19552622	False	0,19552622
ENSMUSG00000018565	Elp5	224,89608	400,0521824	325,8199727	257,5340576	476,2335297	0,19550472	False	0,19550472
ENSMUSG00000019734	Tmc4	88,87645772	116,1034725	172,5965261	226,0451772	101,7704529	0,195445541	False	0,195445541
ENSMUSG00000061286	Exosc5	253,4911142	290,2586812	440,2972604	459,9625745	300,0923612	0,19540404	False	0,19540404
ENSMUSG00000056492	Adgrf5	443,6094498	387,4322397	540,6850358	854,6981824	438,3957972	-0,195346087	False	0,195346087
ENSMUSG00000026878	Rab14	1871,815484	1274,614209	1146,534066	1138,098106	1634,850994	-0,195279077	False	0,195279077
ENSMUSG00000030846	Tial1	708,6931455	1261,994266	619,0579482	814,2124791	1897,105623	-0,195086633	False	0,195086633
ENSMUSG00000032171	Pin1	164,6146565	401,3141766	188,4472275	334,0070529	705,8694235	0,195067778	False	0,195067778
ENSMUSG00000071650	Ganab	2459,945782	2165,582161	2414,590176	4321,848836	2148,922256	-0,195013229	False	0,195013229
ENSMUSG00000026696	Vamp4	158,4319464	124,9374323	90,70123565	95,59124409	181,3601661	0,194994346	False	0,194994346
ENSMUSG00000027091	Zc3h15	462,1575802	583,041351	425,3271536	403,7324309	1028,142524	-0,194985366	False	0,194985366
ENSMUSG00000079015	Serpina1c	63248,35151	19020,77758	72388,39199	7055,758417	31033,4644	0,194730491	False	0,194730491
ENSMUSG00000033880	Lgals3bp	1396,519644	1289,75814	1336,742483	1220,194116	2830,001441	-0,194725189	False	0,194725189
ENSMUSG00000001098	Kctd10	173,8887216	362,1923544	199,0143617	200,1793111	452,7480406	0,194708183	False	0,194708183
ENSMUSG00000021774	Ube2e1	395,6934466	188,0371457	140,8951233	158,5690049	345,7585901	-0,194618077	False	0,194618077
ENSMUSG00000022037	Clu	19594,55399	11524,53164	14451,43668	6711,629938	17123,53108	-0,194472501	False	0,194472501
ENSMUSG00000034931	Dhx8	180,8442705	403,8381652	206,9397124	432,9721056	608,0132188	0,194462638	False	0,194462638
ENSMUSG00000040430	Pitpnc1	564,1722968	557,8014656	645,4757838	398,1094166	414,9103081	0,194227146	False	0,194227146
ENSMUSG00000027404	Snrpb	445,9279661	329,3805035	243,9246823	182,1856652	510,157014	0,194130662	False	0,194130662
ENSMUSG00000020288	Ahsa2	385,6465426	204,4430711	441,1778549	177,6872537	183,9696649	0,194081199	False	0,194081199
ENSMUSG00000069539	Scyl2	381,7823488	516,1556549	333,7453234	510,5697037	772,4116427	-0,194002782	False	0,194002782
ENSMUSG00000074491	Clec4g	986,1422613	42,90780505	862,1020359	313,7642012	56,10422405	-0,193937153	False	0,193937153
ENSMUSG00000036632	Alg5	302,1799563	148,9153234	264,1783563	131,578536	136,9986866	-0,193895695	False	0,193895695
ENSMUSG00000029630	Cyp3a25	7641,829687	1055,027206	6698,68252	8740,413518	1003,352286	0,193783432	False	0,193783432
ENSMUSG00000061731	Ext1	621,3623653	835,4402042	710,6397783	770,3529671	1243,426175	0,193683583	False	0,193683583
ENSMUSG00000033306	Lpp	697,100564	528,7755975	575,0282221	609,5347564	332,7110961	-0,193658308	False	0,193658308
ENSMUSG00000027651	Rprd1b	311,4540214	94,64956996	91,58183017	75,3483924	356,1965852	0,193654486	False	0,193654486
ENSMUSG00000039449	Prpf18	263,5380181	124,9374323	122,4026384	111,3356843	301,3971106	0,193654486	False	0,193654486
ENSMUSG00000034973	Dopey1	211,757821	1187,536604	242,1634932	778,2251872	979,8667968	0,193566137	False	0,193566137
ENSMUSG00000044617	Zbtb39	166,160334	358,4063716	145,2980959	151,8213877	651,0699489	-0,193560224	False	0,193560224
ENSMUSG00000057469	E2f6	166,160334	112,3174897	145,2980959	121,4571101	114,8179469	-0,193560224	False	0,193560224
ENSMUSG00000031245	Hmgn5	745,0165673	470,7238613	651,6399454	325,0102299	416,2150575	-0,193197464	False	0,193197464
ENSMUSG00000026171	Rnf25	235,7158227	388,694234	269,4619234	344,1284787	452,7480406	0,193032822	False	0,193032822
ENSMUSG00000024498	Tcerg1	243,4442103	574,2073911	278,2678686	520,6911296	868,9630981	0,192883141	False	0,192883141
ENSMUSG00000055240	Zfp101	125,9727183	69,40968464	67,80577811	110,2110814	19,57124095	-0,192842036	False	0,192842036
ENSMUSG00000039621	Prex1	86,55794143	100,9595413	75,73112879	122,581713	183,9696649	-0,192779756	False	0,192779756
ENSMUSG00000051853	Arf3	349,3231208	334,4284805	189,327822	141,6999618	399,2533153	0,192742253	False	0,192742253
ENSMUSG00000022855	Senp2	312,9996989	316,7605608	273,864896	430,7228998	340,5395925	-0,192696916	False	0,192696916
ENSMUSG00000021972	Hmbox1	236,4886614	403,8381652	233,357548	206,9269284	609,3179682	-0,192649612	False	0,192649612
ENSMUSG00000021192	Golga5	236,4886614	798,8423705	206,9397124	623,0299909	1202,978944	-0,192560485	False	0,192560485
ENSMUSG00000019173	Rab5c	1620,642886	990,6654989	1040,862724	393,6110051	1418,262594	-0,192441548	False	0,192441548
ENSMUSG00000023967	Mrps18a	506,9822284	352,0964003	255,372411	246,2880289	579,3087321	0,192397235	False	0,192397235
ENSMUSG00000036752	Tubb4b	1934,415423	2192,08404	1946,994486	1135,8489	2210,245478	0,192308957	False	0,192308957
ENSMUSG00000062031	Pgghg	502,3451958	153,9633005	360,163159	439,7197228	40,44723129	-0,192094836	False	0,192094836
ENSMUSG00000029471	Camkk2	301,4071175	239,7789106	344,3124577	326,1348328	61,32322164	0,192004895	False	0,192004895
ENSMUSG00000052384	Nrros	104,333233	100,9595413	117,9996658	124,8309187	91,33245776	-0,191999207	False	0,191999207
ENSMUSG00000068184	Ndufaf2	128,2912346	112,3174897	108,3131261	23,6166603	56,10422405	-0,191840006	False	0,191840006
ENSMUSG00000009035	Tmem184b	420,424287	141,3433578	368,0885097	258,6586605	117,4274457	-0,191793298	False	0,191793298
ENSMUSG00000031536	Polb	182,389948	160,2732718	191,089011	159,6936078	250,5118841	-0,191719655	False	0,191719655
ENSMUSG00000030888	Rrp8	106,6517493	93,38757569	66,04458906	91,0928326	87,41820957	-0,191605097	False	0,191605097
ENSMUSG00000079508	Apoo	240,3528552	113,579484	210,4620905	42,73490912	120,0369445	-0,191593551	False	0,191593551
ENSMUSG00000026031	Cflar	910,4040626	540,1335459	779,326151	552,18001	797,2018813	-0,191561881	False	0,191561881
ENSMUSG00000021072	Tmx1	412,6958993	316,7605608	250,9694384	217,0483542	361,4155828	-0,191420392	False	0,191420392
ENSMUSG00000034165	Ccnd3	493,0711307	820,296273	721,2069126	928,921972	431,8720502	-0,191191834	False	0,191191834
ENSMUSG00000001700	Gramd3	656,9129484	285,2107041	243,9246823	168,6904307	575,3944839	-0,191150812	False	0,191150812
ENSMUSG00000039810	Zc3h10	81,92090886	107,2695126	97,74599182	150,6967848	71,76121681	-0,191027368	False	0,191027368
ENSMUSG00000034544	Rsrc1	218,7133699	131,2474037	191,9696055	249,6618375	110,9036987	0,190933898	False	0,190933898
ENSMUSG00000040007	Bahd1	136,0196223	111,0554954	123,2832329	78,72220101	155,2651782	0,190919518	False	0,190919518
ENSMUSG00000044345	Marveld1	437,4267397	159,0112775	499,2970933	323,885627	57,40897345	0,190857093	False	0,190857093
ENSMUSG00000021810	Ecd	442,0637723	545,181523	504,5806605	808,5894647	521,8997586	0,190830403	False	0,190830403
ENSMUSG00000034320	Slc26a2	71,87400494	82,0296273	116,2384768	120,3325073	101,7704529	0,190674996	False	0,190674996
ENSMUSG00000035376	Hacd2	1352,467835	926,3037913	1453,861554	431,8475027	1543,518536	0,190628525	False	0,190628525
ENSMUSG00000056305	Usp39	278,9947934	156,487289	169,9547425	214,7991485	318,3588527	0,190415684	False	0,190415684
ENSMUSG00000028284	Map3k7	505,4365509	442,9599874	469,3568796	543,183187	698,0409271	-0,190353611	False	0,190353611
ENSMUSG00000050373	Snx21	200,1652396	54,26575344	108,3131261	175,438048	32,61873491	-0,190229794	False	0,190229794
ENSMUSG00000040723	Rcsd1	100,4690392	138,8193693	88,05945209	250,7864404	131,779689	-0,190201212	False	0,190201212
ENSMUSG00000032228	Tcf12	241,125694	448,0079645	211,342685	345,2530816	463,1860358	-0,190201212	False	0,190201212
ENSMUSG00000029730	Mcm7	170,7973666	324,3325264	149,7010685	493,7006606	233,550142	-0,190201212	False	0,190201212
ENSMUSG00000029066	Mrpl20	264,3108569	310,4505895	506,3418495	231,6681916	334,0158455	-0,190175713	False	0,190175713
ENSMUSG00000029994	Anxa4	632,9549467	267,5427844	722,0875071	431,8475027	219,1978986	0,190070869	False	0,190070869
ENSMUSG00000029119	Man2b2	1210,265503	665,0709783	1061,116398	463,3363831	464,4907852	-0,189740655	False	0,189740655
ENSMUSG00000063065	Mapk3	602,814235	339,4764576	470,2374741	528,5633497	223,1121468	-0,189637093	False	0,189637093
ENSMUSG00000021552	Gkap1	187,7998194	225,8969736	164,6711754	241,7896174	169,6174215	-0,189607632	False	0,189607632
ENSMUSG00000066306	Numa1	796,7967644	410,1481365	581,1923838	908,6791203	155,2651782	0,18955914	False	0,18955914
ENSMUSG00000041231	Ublcp1	335,4120231	662,5469897	294,11857	919,925149	364,0250816	-0,189536529	False	0,189536529
ENSMUSG00000034708	Grn	2036,43014	4034,595669	2322,127751	3787,662472	9094,103294	0,189405021	False	0,189405021
ENSMUSG00000004207	Psap	4827,923751	2198,394012	2455,978119	4234,129812	3175,760031	-0,189337391	False	0,189337391

ENSMUSG00000040701	Ap1g2	147,6122037	456,8419243	254,4918165	606,1609478	168,3126721	0,189331801	False	0,189331801
ENSMUSG00000046668	Cxxc5	664,641336	478,2958269	582,9535728	408,2308424	290,9591154	-0,189195031	False	0,189195031
ENSMUSG00000028053	Ash1l	484,5699043	393,742211	273,864896	425,0998855	199,6266577	-0,188902934	False	0,188902934
ENSMUSG00000036285	Noa1	124,4270408	37,85982798	109,1937206	20,24285169	33,92348431	-0,188410156	False	0,188410156
ENSMUSG00000034154	Ino80	248,0812429	282,6867156	137,3727453	154,0705934	268,7783757	0,188391429	False	0,188391429
ENSMUSG00000037035	Inhbb	9,274065154	425,2920677	10,56713425	616,2823736	249,2071347	0,188310411	False	0,188310411
ENSMUSG00000021504	B4galt7	92,74065154	78,2436445	105,6713425	84,34521537	69,15171802	0,188310411	False	0,188310411
ENSMUSG00000055805	Fmnl1	42,50613195	174,1552087	48,43269865	105,7126699	499,7190189	0,188310411	False	0,188310411
ENSMUSG00000071647	Eml3	278,9947934	517,4176491	317,894622	1184,206824	566,2612381	0,188310411	False	0,188310411
ENSMUSG00000029321	Slc10a6	0,772838763	432,8640333	0,880594521	158,5690049	1777,068678	0,188310411	False	0,188310411
ENSMUSG00000018287	Spag7	310,6811826	299,0926411	353,9989974	95,59124409	287,0448672	0,188310411	False	0,188310411
ENSMUSG00000035868	Zfp983	149,1578812	21,45390252	169,9547425	66,35156943	9,133245776	0,188310411	False	0,188310411
ENSMUSG00000038718	Pbx3	23,95800165	291,5206755	27,29843015	89,96822973	319,6636021	0,188310411	False	0,188310411
ENSMUSG00000032567	Aste1	54,0987134	157,7492833	61,64161646	125,9555216	331,4063467	0,188310411	False	0,188310411
ENSMUSG00000079363	Gbp4	60,2814235	126,1994266	68,68637263	317,1380098	75,675465	0,188310411	False	0,188310411
ENSMUSG00000074212	Dnajb14	308,3626664	205,7050654	351,3572138	217,0483542	193,1029107	0,188310411	False	0,188310411
ENSMUSG00000070871	Ccnyl1	40,96045443	143,8673463	46,67150961	107,9618757	268,7783757	0,188310411	False	0,188310411
ENSMUSG00000055850	Rnf181	1116,752012	448,0079645	1272,459083	645,5220483	262,2546287	0,188310411	False	0,188310411
ENSMUSG00000027514	Zbp1	359,3700247	222,1109908	409,4764522	93,34203834	208,7599034	0,188310411	False	0,188310411
ENSMUSG00000031370	Zrsr2	89,64929649	256,184836	102,1489644	154,0705934	241,3786384	0,188310411	False	0,188310411
ENSMUSG00000039238	Zfp750	132,1554284	97,17355849	150,5816631	125,9555216	33,92348431	0,188310411	False	0,188310411
ENSMUSG00000006019	Dhx34	125,9727183	161,5352661	260,6559782	321,6364213	143,5224336	0,188164932	False	0,188164932
ENSMUSG00000053453	Thoc7	333,0935068	426,554062	367,2079152	292,3967466	529,728255	-0,187999972	False	0,187999972
ENSMUSG00000025145	Lrrc45	125,1998796	142,6053521	247,4470604	247,4126318	163,0936746	0,187794954	False	0,187794954
ENSMUSG00000033307	Mif	1895,773485	3139,841734	3843,795084	1664,41225	4682,745584	-0,187773784	False	0,187773784
ENSMUSG00000029290	Zfp326	178,5257542	326,8565149	156,7458247	239,5404117	561,0422405	-0,187705199	False	0,187705199
ENSMUSG00000033634	Nat8f2	11455,78898	5093,408858	13046,88842	4353,337716	742,4024066	0,187628953	False	0,187628953
ENSMUSG00000041958	Pigs	265,8565344	233,4689392	324,0587837	290,1475409	267,4736263	-0,1874173	False	0,1874173
ENSMUSG00000020590	Snx13	364,0070573	248,6128704	144,4175014	284,5245265	319,6636021	-0,187411937	False	0,187411937
ENSMUSG00000028108	Ecm1	2865,686132	2164,320166	2094,053771	1466,482145	2516,861586	-0,187254727	False	0,187254727
ENSMUSG00000026737	Pip4k2a	102,0147167	89,6015929	101,2683699	218,1729571	130,4749397	-0,187181006	False	0,187181006
ENSMUSG00000029173	Sepsecs	823,0732824	164,0592546	936,9525702	556,6784215	161,7889252	0,186955132	False	0,186955132
ENSMUSG00000001552	Jup	755,0634713	859,4180952	857,6990633	431,8475027	778,9353897	0,18676223	False	0,18676223
ENSMUSG00000032575	Manf	2167,81273	3599,207647	3171,02087	2467,3787	9733,430498	0,186739029	False	0,186739029
ENSMUSG00000003283	Hck	68,78264989	94,64956996	88,05945209	160,8182106	78,28496379	0,186690538	False	0,186690538
ENSMUSG00000055204	Ankrd17	672,3697236	2390,21714	765,2366386	2087,26293	2895,238911	0,186651187	False	0,186651187
ENSMUSG00000024735	Prpf19	1211,81118	932,6137627	862,9826304	526,3141439	1379,120112	0,18658319	False	0,18658319
ENSMUSG00000023262	Acy1	618,2710102	232,206945	703,5950222	265,4062777	177,4459179	0,186505915	False	0,186505915
ENSMUSG00000038708	Golga4	834,6658638	803,8903475	733,5352359	1231,440144	1585,270517	-0,1863325	False	0,1863325
ENSMUSG00000069516	Lyz2	555,6710705	113,579484	632,266866	192,307091	187,8839131	0,186302485	False	0,186302485
ENSMUSG00000063870	Chd4	1095,885366	2874,822938	1246,921842	2961,079361	2702,136	0,186274144	False	0,186274144
ENSMUSG00000002395	Use1	426,6069971	824,0822558	707,9979948	638,7744311	485,3667755	0,186167728	False	0,186167728
ENSMUSG00000001962	Fam50a	243,4442103	142,6053521	213,9844686	206,9269284	134,3891878	-0,186085103	False	0,186085103
ENSMUSG00000063273	Naa15	486,1155818	1019,691367	553,0133591	1002,021159	1037,27577	0,186014953	False	0,186014953
ENSMUSG00000071281	Zfp65	110,5159431	97,17355849	62,52221098	64,10236368	63,93272043	-0,185618802	False	0,185618802
ENSMUSG00000007038	Neu1	491,5254531	569,159414	591,759518	558,9276272	606,7084694	0,185395361	False	0,185395361
ENSMUSG00000022983	Scaf4	364,0070573	618,3771904	427,0883426	413,8538568	758,0593994	0,185154981	False	0,185154981
ENSMUSG00000003604	Aven	132,1554284	78,2436445	116,2384768	65,22696655	100,4657035	-0,185147984	False	0,185147984
ENSMUSG00000004266	Ptpn6	381,0095101	242,3028991	286,1932193	335,1316557	147,4366818	-0,185099042	False	0,185099042
ENSMUSG00000011832	Evi5l	78,05671504	165,3212489	169,9547425	245,163426	88,72295896	0,184784732	False	0,184784732
ENSMUSG00000037275	Gemin5	118,2443307	164,0592546	189,327822	250,7864404	134,3891878	0,184646058	False	0,184646058
ENSMUSG00000039163	Cmc1	173,1158829	131,2474037	152,3428521	66,35156943	127,8654409	-0,184416283	False	0,184416283
ENSMUSG00000002221	Paxip1	268,1750507	400,0521824	235,9993316	407,1062395	495,8047707	-0,184392251	False	0,184392251
ENSMUSG00000023020	Cox14	282,8589872	232,206945	321,4170001	123,7063159	319,6636021	0,184363227	False	0,184363227
ENSMUSG00000026307	Scly	1956,827747	1039,883275	2223,501165	1702,648748	1274,74016	0,184316394	False	0,184316394
ENSMUSG00000027075	Slc43a1	119,0171695	104,7455241	96,86539729	64,10236368	87,41820957	-0,184281115	False	0,184281115
ENSMUSG00000033429	Mcee	534,0315851	143,8673463	606,7296249	75,3483924	253,1213829	0,184128684	False	0,184128684
ENSMUSG00000026880	Stom	860,169543	670,1189553	353,9989974	115,8340958	977,257298	0,184117404	False	0,184117404
ENSMUSG00000062867	Impdh2	346,2317657	482,0818097	361,0437536	304,7673782	365,329831	-0,184029606	False	0,184029606
ENSMUSG00000033020	Polr2f	264,3108569	663,808984	632,266866	300,2689667	671,9459392	0,184019691	False	0,184019691
ENSMUSG00000026761	Orc4	244,217049	126,1994266	277,3872741	250,7864404	255,7308817	0,183737682	False	0,183737682
ENSMUSG00000031774	Fam192a	294,4515686	334,4284805	221,9098193	307,016584	287,0448672	0,183667356	False	0,183667356
ENSMUSG00000054520	Sh3bp2	232,6244676	251,136859	264,1783563	127,0801245	234,8548914	0,183509425	False	0,183509425
ENSMUSG00000043923	Ccdc84	217,1676923	552,7534886	403,3122906	490,326852	246,5976359	0,183349477	False	0,183349477
ENSMUSG00000027952	Pmvk	986,9151001	119,8894553	869,1467921	367,745139	83,50396138	-0,183326124	False	0,183326124
ENSMUSG00000028636	Ppcs	215,6220148	244,8268876	155,8652302	232,7927944	131,779689	0,183257524	False	0,183257524
ENSMUSG00000038332	Sesn1	205,5751109	95,91156423	123,2832329	181,0610623	138,303436	-0,183189283	False	0,183189283
ENSMUSG00000027953	Slc50a1	142,2023324	148,9153234	287,9544083	229,4189858	125,2559421	-0,183066082	False	0,183066082
ENSMUSG00000042744	Hectd4	356,2786697	470,7238613	501,0582824	618,5315794	404,4723129	0,183034838	False	0,183034838
ENSMUSG00000025728	Pigq	542,5328115	242,3028991	412,9988303	477,9562204	151,35093	-0,182831915	False	0,182831915
ENSMUSG00000032554	Trf	240160,4184	180871,5422	203238,5736	152996,5977	211652,5329	-0,182300642	False	0,182300642
ENSMUSG00000022911	Arl13b	129,8369122	44,16979931	123,2832329	147,3229762	37,8377325	0,182281853	False	0,182281853
ENSMUSG00000031207	Msn	418,1057707	368,5023257	390,1033727	446,46734	524,5092574	-0,182194231	False	0,182194231
ENSMUSG00000033170	Card10	195,528207	668,8569611	398,9093179	1438,367073	221,8073974	0,181930735	False	0,181930735
ENSMUSG00000000740	Rpl13	2102,121435	3322,830903	3990,854369	1853,345532	3504,556879	-0,181714135	False	0,181714135
ENSMUSG00000024480	Ap3s1	381,7823488	325,5945207	202,5367398	177,6872537	336,6253443	-0,181606603	False	0,181606603
ENSMUSG00000027668	Mfn1	503,8908733	374,812297	338,148296	552,18001	571,4802357	0,181592279	False	0,181592279
ENSMUSG00000027012	Dync1i2	751,9721162	759,7205482	663,0876742	904,1807088	777,6306403	-0,181479529	False	0,181479529
ENSMUSG00000032123	Dpagt1	549,4883604	484,6057982	565,3416824	940,1680007	575,3944839	-0,181277255	False	0,181277255
ENSMUSG00000025403	Shmt2	3946,114723	1135,79484	4474,30076	1338,277417	1645,288989	0,181229338	False	0,181229338
ENSMUSG00000034312	Iqsec1	673,9154012	326,8565149	594,4013016	445,3427372	206,1504046	-0,181130221	False	0,181130221
ENSMUSG00000007589	Tinf2	153,7949138	432,8640333	174,3577151	316,0134069	315,749354	0,181042411	False	0,181042411
ENSMUSG00000022475	Hdac7	137,5652998	137,557375	262,4171672	286,7737323	121,3416939	-0,181041247	False	0,181041247
ENSMUSG00000019907	Ppp1r12a	454,4291925	517,4176491	515,1477947	941,2926035	700,6504259	0,180930881	False	0,180930881
ENSMUSG00000050445	Cyp8b1	3630,796508	16,40592546	4115,89879	102,3388613	260,9498793	0,180921432	False	0,180921432
ENSMUSG00000003575	Crtc1	162,2961402	241,0409048	264,1783563	448,7165458	183,9696649	0,180839208	False	0,180839208
ENSMUSG00000030275	Etnk1	720,2857269	218,325008	519,5507673	816,4616848	198,3219083	0,180815868	False	0,180815868
ENSMUSG00000046402	Rbp1	205,5751109	186,7751514	942,2361373	227,1697801	181,3601661	-0,18080799	False	0,18080799
ENSMUSG00000032788	Pdxk	442,8366111	129,9854094	501,9388769	121,4571101	441,005296	0,180737192	False	0,180737192
ENSMUSG00000029775	Klhdc10	534,0315851	518,6796434	524,8343344	471,2086032	681,079185	-0,180559192	False	0,180559192
ENSMUSG00000037410	Tbc1d2b	436,653901	252,3988532	447,3420166	494,8252635	135,6939372	0,180428931	False	0,180428931
ENSMUSG00000028483	Snapc3	136,0196223	188,0371457	133,8503672	301,3935696	120,0369445	-0,180336289	False	0,180336289
ENSMUSG00000038764	Ptpn3	391,056414	189,2991399	344,3124577	443,0935314	181,3601661	0,180234519	False	0,180234519
ENSMUSG00000000561	Wdr77	101,2418779	224,6349794	160,2682028	114,7094929	221,8073974	0,180178615	False	0,180178615
ENSMUSG00000004071	Cdip1	973,7768411	1620,400638	859,4602524	4762,693161	1013,790281	-0,180160269	False	0,180160269
ENSMUSG00000009569	Mkl2	416,5600932	200,6570883	306,4468933	367,745139	176,1411685	-0,179818363	False	0,179818363
ENSMUSG00000058254	Tspan7	252,7182754	103,4835298	286,1932193	209,1761341	100,4657035	0,179459494	False	0,179459494
ENSMUSG00000024180	Tmem8	278,2219546	453,0559415	245,6858713	391,3617993	277,9116215	-0,179421373	False	0,179421373
ENSMUSG00000037351	Actr1b	815,3448948	1190,060593	1044,385102	923,2989576	1307,358895	0,179387403	False	0,179387403
ENSMUSG00000034832	Tet3	166,9331728	311,7125837	175,2383097	251,9110432	147,4366818	-0,179175167	False	0,179175167
ENSMUSG00000028710	Atpaf1	116,6986532	76,98165023	69,56696715	49,48252635	103,0752023	-0,179090618	False	0,179090618
ENSMUSG00000030720	Cln3	238,8071777	318,0225551	270,3425179	373,3681534	388,8153202	0,178942229	False	0,178942229
ENSMUSG00000045435	Tmem60	108,1974268	152,7013062	152,3428521	95,59124409	125,2559421	-0,178715806	False	0,178715806
ENSMUSG00000028692	Akr1a1	4575,205476	4583,563175	4274,405804	2348,170796	5178,550355	0,17871176	False	0,17871176
ENSMUSG00000028980	H6pd	4454,642629	6947,278435	3936,257508	5387,972358	7126,541204	-0,178485093	False	0,178485093
ENSMUSG00000035199	Arl6ip5	363,2342185	185,5131571	187,5666329	52,85633497	320,9683515	-0,178469068	False	0,178469068
ENSMUSG00000025290	Rps24	3312,386937	4591,13514	5338,163985	2927,341275	5118,531883	-0,178280264	False	0,178280264
ENSMUSG00000057506	Bloc1s2	110,5159431	200,6570883	125,044422	152,9459905	304,0066094	0,178186194	False	0,178186194
ENSMUSG00000029034	Ints11	217,9405311	314,2365723	246,5664658	378,9911677	311,8351058	0,178042076	False	0,178042076
ENSMUSG00000055312	Them7	299,86144	41,64581078	265,0589508	32,61348328	41,75198069	-0,177982754	False	0,177982754
ENSMUSG00000022749	Tbc1d23	216,3948536	345,7864289	244,8052768	700,627589	533,6425032	0,177968467	False	0,177968467
ENSMUSG00000079487	Med12	424,2884808	1168,60669	479,9240139	1244,935379	1501,766555	0,177760492	False	0,177760492
ENSMUSG00000024338	Psmb8	386,4193814	205,7050654	341,6706741	164,1920193	317,0541034	-0,177561031	False	0,177561031

ENSMUSG00000020621	Rdh14	218,7133699	247,3508762	191,089011	128,2047274	143,5224336	0,177517595	False	0,177517595
ENSMUSG00000057069	Ero1lb	947,5003232	378,5982798	526,5955235	837,8291394	591,0514766	-0,177470374	False	0,177470374
ENSMUSG00000021288	Klc1	245,7627266	426,554062	357,5213755	560,0522301	277,9116215	0,177360038	False	0,177360038
ENSMUSG00000029110	Rnf4	1038,695297	280,1627271	225,4321973	253,0356461	918,5435751	-0,177352431	False	0,177352431
ENSMUSG00000021143	Pacs2	303,7256338	167,8452374	343,4318631	201,303914	109,5989493	0,177255223	False	0,177255223
ENSMUSG00000034462	Pkd2	221,0318862	318,0225551	195,4919836	593,7903162	243,9881371	-0,177145059	False	0,177145059
ENSMUSG00000052310	Slc39a1	966,8212923	2795,317299	1670,487806	1093,113991	2737,364234	0,177122704	False	0,177122704
ENSMUSG00000018433	Nol11	175,4343992	146,3913349	104,790748	107,9618757	198,3219083	0,176912399	False	0,176912399
ENSMUSG00000078920	Ifi47	425,0613195	55,52774771	376,0138604	157,444402	133,0844384	-0,176885137	False	0,176885137
ENSMUSG00000049739	Zfp646	195,528207	349,5724117	221,0292247	239,5404117	259,6451299	0,176860391	False	0,176860391
ENSMUSG00000029655	N4bp2l2	466,021774	310,4505895	211,342685	287,8983351	412,3008093	-0,176700071	False	0,176700071
ENSMUSG00000027291	Vps39	283,6318259	406,3621537	320,5364056	967,1584696	438,3957972	0,176468799	False	0,176468799
ENSMUSG00000019338	Zfp687	188,5726581	357,1443773	213,103874	372,2435505	263,5593781	0,176436311	False	0,176436311
ENSMUSG00000014932	Yes1	283,6318259	157,7492833	93,34301921	320,5118184	233,550142	0,176358131	False	0,176358131
ENSMUSG00000019578	Ubxn6	460,6119026	407,624148	258,8947891	343,0038758	305,3113588	-0,176312172	False	0,176312172
ENSMUSG00000027366	Sppl2a	2490,086494	1114,340937	1334,981294	941,2926035	2203,721731	-0,176253793	False	0,176253793
ENSMUSG00000021710	Nln	339,2762169	200,6570883	300,2827316	239,5404117	294,8733636	-0,176138789	False	0,176138789
ENSMUSG00000027752	Exosc8	92,74065154	126,1994266	124,1638274	82,09600963	139,6081854	-0,175889762	False	0,175889762
ENSMUSG00000001833	Sept7	809,1621847	455,5799301	294,9991645	151,8213877	716,3074187	-0,175850015	False	0,175850015
ENSMUSG00000021271	Zfp839	110,5159431	59,31373051	95,10420825	97,84044983	43,05673008	-0,175751567	False	0,175751567
ENSMUSG00000032633	Flcn	266,6293732	893,4919404	301,1633261	1067,248125	759,3641488	0,175710375	False	0,175710375
ENSMUSG00000024974	Smc3	427,3798358	304,1406181	234,2381425	282,2753208	378,377325	-0,175693207	False	0,175693207
ENSMUSG00000022466	Rpap3	112,0616206	107,2695126	62,52221098	56,23014358	126,5606915	0,175537125	False	0,175537125
ENSMUSG00000028651	Ppie	155,3405913	137,557375	141,7757179	204,6777226	217,8931492	-0,175401372	False	0,175401372
ENSMUSG00000038028	Tigar	94,28632906	114,8414782	172,5965261	190,0578853	83,50396138	-0,175203964	False	0,175203964
ENSMUSG00000022635	Zcrb1	330,7749905	742,0526285	509,8642276	373,3681534	780,2401391	0,174748719	False	0,174748719
ENSMUSG00000075595	Zfp652	401,1033179	263,7568016	222,7904138	206,9269284	452,7480406	0,174734497	False	0,174734497
ENSMUSG00000024387	Csnk2b	1568,862688	2992,188405	2785,320469	1390,009149	3389,738932	-0,17462471	False	0,17462471
ENSMUSG00000035413	Tmem98	244,217049	135,0333865	275,626085	152,9459905	69,15171802	0,17454851	False	0,17454851
ENSMUSG00000019878	Hsf2	324,5922804	169,1072317	366,3273207	186,6840767	130,4749397	0,174504612	False	0,174504612
ENSMUSG00000028960	Ube4b	643,0018507	725,646703	749,3859372	892,9346801	1035,971021	0,174444422	False	0,174444422
ENSMUSG00000066319	Rtp3	1198,672921	121,1514495	1352,593184	623,0299909	199,6266577	0,174289941	False	0,174289941
ENSMUSG00000067194	Eif1ax	479,9328717	297,8306468	326,7005672	155,1951963	425,3483033	-0,174187931	False	0,174187931
ENSMUSG00000028948	Nol9	158,4319464	334,4284805	178,7606877	358,748316	446,2242936	0,174166229	False	0,174166229
ENSMUSG00000041328	Pcf11	316,8638927	793,7943934	357,5213755	782,7235986	1197,759946	0,174166229	False	0,174166229
ENSMUSG00000019087	Atp6ap1	824,6189599	808,9383246	606,7296249	629,7776081	930,2863197	0,173947179	False	0,173947179
ENSMUSG00000026727	Rsu1	490,7526144	248,6128704	435,0136933	244,0388231	233,550142	-0,173935139	False	0,173935139
ENSMUSG00000032786	Alas1	3458,453464	5868,273338	11293,62473	3065,667428	7594,946237	-0,173925846	False	0,173925846
ENSMUSG00000029287	Tgfbr3	77,28387628	160,2732718	87,17885756	186,6840767	190,4934119	0,173810842	False	0,173810842
ENSMUSG00000020128	Vps54	538,6686177	398,7901881	316,133433	325,0102299	477,5382791	-0,173781634	False	0,173781634
ENSMUSG00000027531	Impa1	840,8485739	179,2031858	948,400299	325,0102299	78,28496379	0,173650105	False	0,173650105
ENSMUSG00000017686	Rhot1	438,9724173	427,8160562	389,2227782	516,1927181	583,2229802	-0,173534149	False	0,173534149
ENSMUSG00000051007	Gatd1	219,4862086	23,97789106	194,6113891	114,7094929	10,43799517	-0,173534149	False	0,173534149
ENSMUSG00000026043	Col3a1	298,3157624	80,76763303	336,387107	70,84998091	87,41820957	0,173282202	False	0,173282202
ENSMUSG00000024925	Rnaseh2c	296,7700849	179,2031858	334,6259179	73,09918666	167,0079228	0,173203519	False	0,173203519
ENSMUSG00000033712	Ccar2	294,4515686	479,5578211	331,9841344	624,1545937	477,5382791	0,173083937	False	0,173083937
ENSMUSG00000019842	Traf3ip2	73,41968247	142,6053521	82,77588496	119,2079044	227,026395	0,173043655	False	0,173043655
ENSMUSG00000032425	Zfp949	109,7431043	99,69754702	67,80577811	123,7063159	106,9894505	0,172788868	False	0,172788868
ENSMUSG00000029705	Cux1	349,3231208	393,742211	317,0140275	365,4959333	247,9023853	0,17268925	False	0,17268925
ENSMUSG00000029199	Lias	355,5058309	517,4176491	400,670507	642,1482397	482,7572767	0,172543095	False	0,172543095
ENSMUSG00000028675	Pnrc2	1006,236069	894,7539347	700,0726441	316,0134069	1133,827226	0,172232007	False	0,172232007
ENSMUSG00000025917	Cops5	671,5968849	552,7534886	422,68537	293,5213495	756,75465	0,172230096	False	0,172230096
ENSMUSG00000030109	Slc6a12	1384,927063	1039,883275	1560,413491	1172,960795	769,8021439	0,172118378	False	0,172118378
ENSMUSG00000002957	Ap2a2	1321,554284	559,0634599	925,5048414	1488,974202	520,5950092	0,172083071	False	0,172083071
ENSMUSG00000021824	Ap3m1	840,0757352	946,4956996	655,1623235	677,0109287	563,6517393	0,172076554	False	0,172076554
ENSMUSG00000031799	Tpm4	295,2244074	382,3842626	317,894622	262,0324691	557,1279923	-0,172066409	False	0,172066409
ENSMUSG00000045752	Tssc4	136,792461	208,2290539	154,1040411	245,163426	358,806084	0,171915973	False	0,171915973
ENSMUSG00000022325	Pop1	89,64929649	203,1810768	103,0295589	219,29756	79,58971319	-0,171710284	False	0,171710284
ENSMUSG00000078234	Klhdc7a	265,0836956	41,64581078	298,5215426	56,23014358	6,523746983	0,171387108	False	0,171387108
ENSMUSG00000040359	Ufl1	690,1450152	695,3588406	612,8937865	853,5735796	1156,007965	-0,171262458	False	0,171262458
ENSMUSG00000020571	Pdia6	3766,043291	3544,941894	3927,451563	3344,56894	6287,587342	-0,171229301	False	0,171229301
ENSMUSG00000006676	Usp19	904,2213525	880,8719978	803,102203	1024,513216	1722,269203	-0,171092389	False	0,171092389
ENSMUSG00000038256	Bcl9	124,4270408	140,0813635	272,9843015	387,9879907	208,7599034	0,170964982	False	0,170964982
ENSMUSG00000053334	Ficd	102,0147167	114,8414782	186,6860384	233,9173973	339,2348431	0,170866515	False	0,170866515
ENSMUSG00000036893	Ehmt1	295,2244074	589,3513223	421,8047755	741,1132924	262,2546287	-0,170843764	False	0,170843764
ENSMUSG00000014771	Pdcd2	129,8369853	118,627461	137,3727453	38,23649764	146,1319324	0,170570872	False	0,170570872
ENSMUSG00000033964	Zbtb41	136,792461	114,8414782	59,88042742	50,60712922	153,9604288	0,170570872	False	0,170570872
ENSMUSG00000057729	Prtn3	4,637032577	11,3579484	740,579992	5,623014358	5,218997586	0,170570872	False	0,170570872
ENSMUSG00000028224	Nbn	222,5775637	203,1810768	84,537074	121,4571101	250,5118841	0,170570872	False	0,170570872
ENSMUSG00000055943	Emc7	876,399157	714,2887546	604,0878413	246,2880289	986,3905438	0,170570872	False	0,170570872
ENSMUSG00000075256	Cerkl	51,00735834	128,7234151	102,1489644	364,3713304	57,40897345	0,170570872	False	0,170570872
ENSMUSG00000039753	Fbxl5	398,7848016	331,904492	257,1336001	383,4895792	448,8337924	0,170570872	False	0,170570872
ENSMUSG00000031169	Porcn	125,1998796	580,5173624	169,074148	253,0356461	140,9129348	0,170570872	False	0,170570872
ENSMUSG00000078612	Fyb2	171,5702053	242,3028991	344,3124577	891,8100772	193,1029107	0,170570872	False	0,170570872
ENSMUSG00000091512	Lamtor3	168,4788503	204,4430711	149,7010685	176,5626508	186,5791637	-0,170482977	False	0,170482977
ENSMUSG00000027474	Ccm2l	58,73574597	177,9411915	205,1785234	572,4228617	52,18997586	-0,170466045	False	0,170466045
ENSMUSG00000033703	Fuk	88,10361896	141,3433578	162,0293918	273,2784978	78,28496379	-0,170466045	False	0,170466045
ENSMUSG00000026712	Mrc1	464,4760964	281,4247213	245,6858713	412,7292539	199,6266577	-0,170408653	False	0,170408653
ENSMUSG00000028126	Pip5k1a	187,7998194	389,9562282	211,342685	263,157072	467,100284	0,170388503	False	0,170388503
ENSMUSG00000029518	Rab35	245,7627266	533,8235746	218,3874412	375,6173591	516,680761	-0,170376234	False	0,170376234
ENSMUSG00000033799	Fam208b	190,8911744	172,8932145	142,6563124	214,7991485	164,398424	0,170237871	False	0,170237871
ENSMUSG00000039308	Ndst2	386,4193814	1149,676776	343,4318631	761,3561441	1320,406389	-0,17014356	False	0,17014356
ENSMUSG00000025759	Mfsd8	142,9751711	73,19566744	99,50718086	127,0801245	62,62797103	-0,170026224	False	0,170026224
ENSMUSG00000022800	Fyttd1	333,8663455	389,9562282	296,7603535	327,2594356	658,8984452	-0,16997231	False	0,16997231
ENSMUSG00000031502	Col4a1	404,1946729	2197,132017	359,2825645	2010,789934	4076,037115	-0,169931383	False	0,169931383
ENSMUSG00000031320	Rps4x	2609,876502	2487,390699	2935,902133	1909,575676	2285,920943	0,169822336	False	0,169822336
ENSMUSG00000031887	Tradd	121,3356858	117,3654668	136,4921507	113,58489	93,94195655	0,169814068	False	0,169814068
ENSMUSG00000012428	Steap4	1107,477947	15069,47353	984,5046743	3487,393505	18289,97704	-0,16980801	False	0,16980801
ENSMUSG00000004270	Lpcat3	1338,556737	1047,455241	905,2511674	581,4196846	1189,93145	-0,169799827	False	0,169799827
ENSMUSG00000030522	Mtmr10	362,4613798	547,7055115	407,7152632	745,6117039	487,9762743	0,169734682	False	0,169734682
ENSMUSG00000021987	Mtmr6	254,263953	145,1293406	142,6563124	226,0451772	93,94195655	-0,169715808	False	0,169715808
ENSMUSG00000035692	Isg15	120,562847	34,07384519	135,6115562	15,7444402	110,9036987	0,169694733	False	0,169694733
ENSMUSG00000038762	Abcf1	841,6214127	2361,191272	946,6391099	2218,841466	3057,027836	0,169643117	False	0,169643117
ENSMUSG00000002603	Tgfb1	130,6097509	132,5093979	171,7159316	319,3872155	116,1226963	-0,169612632	False	0,169612632
ENSMUSG00000037089	Slc35b2	401,1033179	295,3066583	356,6407809	272,1538949	143,5224336	-0,169502219	False	0,169502219
ENSMUSG00000030868	Dctn5	488,4340981	697,8828292	626,1027043	994,1489385	549,2994959	0,169429048	False	0,169429048
ENSMUSG00000031826	Usp10	864,0337368	687,786875	890,2810606	971,6568811	497,1095201	0,169359304	False	0,169359304
ENSMUSG00000009681	Bcr	414,2415769	198,1330998	465,8345015	338,5054644	156,5699276	0,169345133	False	0,169345133
ENSMUSG00000001418	Glmp	932,0435479	1145,890794	1169,429524	828,8323164	1343,891878	-0,169317108	False	0,169317108
ENSMUSG00000028256	Odf2l	126,7455571	165,3212489	112,7160987	149,5721819	186,5791637	-0,169241593	False	0,169241593
ENSMUSG00000020180	Snrpd3	398,0119628	219,5870023	324,9393782	41,61030625	447,529043	0,169169517	False	0,169169517
ENSMUSG00000015536	Mocs2	1344,739447	737,0046514	1511,980792	338,5054644	320,9683515	0,169113145	False	0,169113145
ENSMUSG00000038780	Smurf1	183,1627868	348,3104175	162,9099864	183,3102681	472,3192815	-0,169051377	False	0,169051377
ENSMUSG00000035545	Leng8	1725,748957	1460,127366	1021,489644	394,7356079	1940,162353	0,168954769	False	0,168954769
ENSMUSG00000020605	Hs1bp3	200,9380783	59,31373051	178,7606877	136,0769475	46,97097827	-0,168721484	False	0,168721484
ENSMUSG00000005225	Plekha8	225,6689187	108,5315069	200,7755508	98,9650527	189,1886625	-0,168624133	False	0,168624133
ENSMUSG00000002699	Lcp2	107,424588	71,93367317	53,71626577	95,59124409	60,01847224	-0,168373862	False	0,168373862
ENSMUSG00000030968	Pdilt	139,1109773	15,14393119	138,2533398	156,3197992	7,828496379	0,168264251	False	0,168264251
ENSMUSG00000068328	Aup1	2386,5261	1530,799045	2681,410316	1466,482145	1748,364191	0,168079887	False	0,168079887
ENSMUSG00000029713	Gnb2	847,8041228	1407,123607	1154,459417	952,5386323	1269,521163	0,168046623	False	0,168046623
ENSMUSG00000026353	Ubxn4	2393,481648	2493,70067	2587,186702	1663,287647	2689,088506	0,168006495	False	0,168006495
ENSMUSG00000021096	Ppm1a	1263,591377	843,0121698	712,4009674	864,8196083	1124,69398	-0,167997488	False	0,167997488
ENSMUSG00000019970	Sgk1	109,7431043	431,602039	123,2832329	654,5188713	433,1767996	0,167846309	False	0,167846309

ENSMUSG00000033540	Idua	85,01226391	177,9411915	97,74599182	492,5760578	75,675465	-0,167845345	False	0,167845345
ENSMUSG00000018666	Cbx1	341,5947332	304,1406181	291,4767864	230,5435887	178,7506673	-0,167547229	False	0,167547229
ENSMUSG00000012114	Med15	430,4711909	1002,023447	483,446392	996,3981443	1278,654409	0,167439233	False	0,167439233
ENSMUSG00000031865	Dctn1	426,6069971	734,4806629	479,0434193	889,5608715	682,3839344	0,167248796	False	0,167248796
ENSMUSG00000062202	Btbd9	211,757821	410,1481365	237,7605206	516,1927181	289,654366	0,167093925	False	0,167093925
ENSMUSG00000029505	Ep400	541,7599727	1272,09022	482,5657974	1253,932202	2040,628056	-0,16692814	False	0,16692814
ENSMUSG00000022351	Sqle	1568,862688	819,0342787	1761,189042	470,0840003	284,4353684	0,166830684	False	0,166830684
ENSMUSG00000041084	Ostc	959,0929046	458,1039186	954,5644606	389,1125936	1076,418252	0,166496282	False	0,166496282
ENSMUSG00000096910	Zfp955b	139,1109773	51,74176491	44,91032056	35,98729189	123,9511927	-0,166464115	False	0,166464115
ENSMUSG00000021038	Vipas39	204,8022721	267,5427844	229,8351699	456,5887659	232,2453926	0,166367858	False	0,166367858
ENSMUSG00000068823	Csde1	2222,684282	1958,615101	1600,040244	1346,149637	1980,609584	-0,166358512	False	0,166358512
ENSMUSG00000036875	Dna2	180,8442705	188,0371457	161,1487973	170,9396365	245,2928865	-0,16635447	False	0,16635447
ENSMUSG00000037750	Fam222b	260,4466631	153,9633005	212,2232795	158,5690049	292,2638648	0,166283514	False	0,166283514
ENSMUSG00000003923	Tfam	401,1033179	141,3433578	166,4323644	142,8245647	357,5013346	-0,166025272	False	0,166025272
ENSMUSG00000020021	Fgd6	469,113129	320,5465436	304,6857042	526,3141439	232,2453926	0,165988288	False	0,165988288
ENSMUSG00000015377	Dennd6b	119,7900082	143,8673463	287,9544083	465,5855889	134,3891878	0,165909495	False	0,165909495
ENSMUSG00000066324	Impad1	525,5303587	1244,326346	468,4762851	546,5569956	2544,261323	-0,165798089	False	0,165798089
ENSMUSG00000020038	Cry1	184,7084643	239,7789106	164,6711754	186,6840767	517,9855104	-0,165661937	False	0,165661937
ENSMUSG00000048930	Tada3	220,2590474	108,5315069	196,3725782	150,6967848	82,19921198	-0,165607798	False	0,165607798
ENSMUSG00000055065	Ddx17	2666,293732	581,7793567	2990,498993	1276,424259	608,0132188	0,165550508	False	0,165550508
ENSMUSG00000038914	Dido1	714,1030168	517,4176491	407,7152632	474,5824118	636,7177055	-0,165478331	False	0,165478331
ENSMUSG00000061613	U2af1	472,9773228	642,3550815	458,7897454	421,7260769	1383,03436	-0,165464783	False	0,165464783
ENSMUSG00000033149	Phldb2	920,4509665	290,2586812	820,7140934	664,6402971	161,7889252	-0,165461142	False	0,165461142
ENSMUSG00000011958	Bnip2	485,342743	306,6646067	297,640948	544,3077899	245,2928865	0,165418763	False	0,165418763
ENSMUSG00000002280	Narfl	295,2244074	436,6500161	263,2977617	454,3395601	450,1385418	-0,165116742	False	0,165116742
ENSMUSG00000013593	Ndufs2	2024,06472	2269,065691	1654,637105	1684,655102	2151,531755	0,164842955	False	0,164842955
ENSMUSG00000024424	Ttc39c	711,0116618	396,2661996	577,6700057	634,2760196	337,9300937	-0,164762424	False	0,164762424
ENSMUSG00000038225	Primpol	161,5233014	94,64956996	178,7606877	181,0610623	60,01847224	0,16473402	False	0,16473402
ENSMUSG00000027361	Gabpb1	142,9751711	257,4468303	160,2682028	345,2530816	788,0686355	0,164723591	False	0,164723591
ENSMUSG00000020514	Mrpl22	331,5478292	218,325008	371,6108878	93,34203834	300,0923612	0,164575763	False	0,164575763
ENSMUSG00000047617	Paxx	1194,035889	223,3729851	1065,51937	301,3935696	123,9511927	-0,164289379	False	0,164289379
ENSMUSG00000026659	Dusp12	280,5404709	59,31373051	314,3722439	96,71584696	52,18997586	0,164264937	False	0,164264937
ENSMUSG00000076432	Ywhaq	608,2241063	343,2624404	314,3722439	257,5340576	542,7757489	-0,164246734	False	0,164246734
ENSMUSG00000052928	Ctif	313,7725377	681,4769037	280,0290576	653,3942684	855,9156041	-0,164142551	False	0,164142551
ENSMUSG00000036845	Lin37	172,3430441	352,0964003	318,7752165	372,2435505	193,1029107	0,164086839	False	0,164086839
ENSMUSG00000021796	Bmpr1a	427,3798358	323,0705321	265,9395453	232,7927944	478,8430285	0,164033956	False	0,164033956
ENSMUSG00000024833	Pola2	139,1109773	238,5169163	124,1638274	364,3713304	144,827183	-0,163991333	False	0,163991333
ENSMUSG00000048832	Vps37c	275,9034383	806,4143361	309,0886768	768,1037613	1778,373427	0,163857368	False	0,163857368
ENSMUSG00000028098	Rnf115	416,5600932	148,9153234	184,9248494	79,84680388	371,853578	-0,163789981	False	0,163789981
ENSMUSG00000025499	Hras	453,6563538	368,5023257	508,1030385	372,2435505	360,1108334	0,163521227	False	0,163521227
ENSMUSG00000048661	Lemd3	132,1554284	171,6312202	117,9996658	202,4285169	322,2731009	-0,163452913	False	0,163452913
ENSMUSG00000033953	Ppp3r1	347,7774433	156,487289	117,1190713	147,3229762	310,5303564	-0,163430055	False	0,163430055
ENSMUSG00000038510	Rpf2	102,0147167	210,7530424	123,2832329	91,0928326	241,3786384	-0,163367842	False	0,163367842
ENSMUSG00000035161	Ints6	246,5355653	512,3696721	220,1486302	294,6459524	382,2915732	-0,163317918	False	0,163317918
ENSMUSG00000072941	Sod3	851,6683166	314,2365723	953,6838661	214,7991485	913,3245776	0,16321943	False	0,16321943
ENSMUSG00000015759	Cnih1	772,8387628	422,7680792	601,4460577	240,6650145	690,2124308	-0,163126999	False	0,163126999
ENSMUSG00000026577	Blzf1	131,3825897	117,3654668	137,3727453	209,1761341	334,0158455	-0,162766131	False	0,162766131
ENSMUSG00000041229	Phf8	139,8838161	71,93367317	103,9101535	91,0928326	156,5699276	0,162578082	False	0,162578082
ENSMUSG00000027954	Efna1	1177,806275	1755,434024	1052,310452	1183,082221	2039,323307	-0,162541873	False	0,162541873
ENSMUSG00000010376	Nedd8	772,8387628	1407,123607	1401,906477	690,5061632	1176,883956	-0,162513165	False	0,162513165
ENSMUSG00000040818	Dennd6a	176,2072379	85,8156101	101,2683699	157,444402	69,15171802	-0,162430723	False	0,162430723
ENSMUSG00000030689	Ino80e	173,1158829	427,8160562	193,7307946	229,4189858	433,1767996	0,162315203	False	0,162315203
ENSMUSG00000024150	Mcfd2	2608,330824	1627,972603	2330,933697	1297,791714	1346,501377	-0,162218896	False	0,162218896
ENSMUSG00000022378	Fam49b	118,2443307	193,0851227	105,6713425	156,3197992	294,8733636	-0,162186836	False	0,162186836
ENSMUSG00000015461	Atf6b	425,0613195	195,6091113	475,5210413	375,6173591	193,1029107	0,1618382	False	0,1618382
ENSMUSG00000027865	Gdap2	527,0760362	700,4068177	509,8642276	471,2086032	832,430115	-0,161645221	False	0,161645221
ENSMUSG00000024140	Epas1	1891,136453	3570,181779	1690,74148	1852,22093	3538,480363	-0,161597379	False	0,161597379
ENSMUSG00000010054	Tusc2	547,1698441	892,2299462	612,013192	645,5220483	654,984197	0,161574029	False	0,161574029
ENSMUSG00000030313	Dennd5b	1428,206034	1130,746862	1597,398461	1324,782183	975,9525486	0,16152011	False	0,16152011
ENSMUSG00000025505	Tmem80	161,5233014	34,07384519	144,4175014	44,98411486	11,74274457	-0,161496716	False	0,161496716
ENSMUSG00000031505	Naxd	675,4610787	321,8085379	604,0878413	341,879273	228,3311444	-0,161114292	False	0,161114292
ENSMUSG00000074886	Grk6	206,3479497	408,8861422	230,7157645	628,6530052	390,1200696	0,161037481	False	0,161037481
ENSMUSG00000020114	Cand1	629,0907529	329,3805035	194,6113891	258,6586605	703,2599247	0,160789852	False	0,160789852
ENSMUSG00000044452	Zfp507	208,666466	193,0851227	186,6860384	257,5340576	292,2638648	-0,160584731	False	0,160584731
ENSMUSG00000046329	Slc25a23	9610,250015	940,1857283	8599,886091	2893,603189	489,2810237	-0,160256413	False	0,160256413
ENSMUSG00000026641	Usf1	200,1652396	256,184836	223,6710083	223,7959715	328,7968479	0,16018681	False	0,16018681
ENSMUSG00000001016	Ilf2	199,3924008	473,2478498	222,7904138	346,3776845	707,1741729	0,160076731	False	0,160076731
ENSMUSG00000006289	Osgep	230,3059513	142,6053521	184,0442549	86,59442111	206,1504046	-0,159854399	False	0,159854399
ENSMUSG00000020664	Dld	1299,914799	952,8056709	589,998329	526,3141439	1452,186078	0,15980926	False	0,15980926
ENSMUSG00000029171	Pgm1	78,82955381	227,1589679	88,05945209	101,2142584	314,4446046	0,159741259	False	0,159741259
ENSMUSG00000031601	Cnot7	353,9601534	533,8235746	395,3869399	618,5315794	838,953862	0,159678258	False	0,159678258
ENSMUSG00000059554	Ccdc28a	176,2072379	157,7492833	121,5220439	132,7031389	84,80871077	-0,159639736	False	0,159639736
ENSMUSG00000029528	Pxn	962,9570985	2147,914241	862,1020359	2125,499427	1242,121425	-0,159612892	False	0,159612892
ENSMUSG00000007415	Gatad1	692,4635315	543,9195287	619,9385427	396,9848137	396,6438165	-0,159612892	False	0,159612892
ENSMUSG00000025075	Habp2	3447,633721	1524,489073	3850,83984	1196,577455	1632,241495	0,159566612	False	0,159566612
ENSMUSG00000028974	Dffa	233,3973064	177,9411915	260,6559782	148,4475791	234,8548914	0,159359038	False	0,159359038
ENSMUSG00000015501	Hivep2	116,6986532	733,2186686	130,3279891	507,1958951	215,2836504	0,159359038	False	0,159359038
ENSMUSG00000022957	Itsn1	527,0760362	1412,171584	471,9986632	1223,567924	897,6675848	-0,159228327	False	0,159228327
ENSMUSG00000020705	Ddx42	424,2884808	497,2257409	473,7598522	840,0783451	678,4696862	0,159110435	False	0,159110435
ENSMUSG00000035704	Alg8	93,5134903	111,0554954	153,2234466	201,303914	104,3799517	0,158598231	False	0,158598231
ENSMUSG00000025737	Wdr24	232,6244676	311,7125837	373,3720768	403,7324309	259,6451299	0,158538317	False	0,158538317
ENSMUSG00000037542	Aldh8a1	8424,715353	2860,941001	7548,456233	3227,610242	243,9881371	-0,15844632	False	0,15844632
ENSMUSG00000017776	Crk	690,1450152	618,3771904	353,1184029	348,6268902	320,9683515	-0,158412432	False	0,158412432
ENSMUSG00000024621	Csf1r	560,308103	382,3842626	399,7899125	625,2791966	438,3957972	0,158280162	False	0,158280162
ENSMUSG00000038848	Ythdf1	376,3724775	458,1039186	337,2677015	429,598297	1430,005339	-0,158266969	False	0,158266969
ENSMUSG00000017670	Elmo2	148,3850425	228,4209622	132,9697726	383,4895792	254,4261323	-0,15824735	False	0,15824735
ENSMUSG00000074277	Phldb3	172,3430441	99,69754702	165,5517699	192,307091	53,49472526	0,158128889	False	0,158128889
ENSMUSG00000029313	Aff1	350,8687983	186,7751514	178,7606877	166,441225	314,4446046	-0,158125778	False	0,158125778
ENSMUSG00000063904	Dpp3	888,7645772	1236,754381	1099,862557	1680,15669	991,6095413	0,157970836	False	0,157970836
ENSMUSG00000092417	Gpank1	110,5159431	147,6533291	123,2832329	209,1761341	236,1596408	0,157722092	False	0,157722092
ENSMUSG00000068267	Cenpb	1318,462929	2407,88506	1932,024379	1181,957618	2762,154472	-0,157678703	False	0,157678703
ENSMUSG00000025142	Aspscr1	1384,927063	2067,146608	2315,082995	2458,381877	1544,823285	0,157631817	False	0,157631817
ENSMUSG00000031429	Psmd10	112,0616206	80,76763303	54,59686029	56,23014358	100,4657035	-0,157589176	False	0,157589176
ENSMUSG00000028274	Rngtt	136,792461	109,7935012	72,20875071	87,71902399	122,6464433	-0,157483325	False	0,157483325
ENSMUSG00000006529	Itih1	10250,16051	4196,130935	9190,765014	7294,174225	3010,056858	-0,157389644	False	0,157389644
ENSMUSG00000031753	Cog4	612,0883001	397,5281938	512,5060111	682,6339431	288,3496166	0,157372361	False	0,157372361
ENSMUSG00000071657	Bscl2	561,8537806	565,3734312	892,0422496	503,8220865	641,9367031	-0,157280357	False	0,157280357
ENSMUSG00000050323	Ndufaf6	119,7900082	60,57572477	107,4325315	47,23332061	71,76121681	-0,157076656	False	0,157076656
ENSMUSG00000028081	Rps3a1	2587,464178	3570,181779	3145,483628	2321,180327	3452,366903	-0,156680283	False	0,156680283
ENSMUSG00000016510	Mtif3	298,3157624	153,9633005	205,1785234	267,6554834	109,5989493	-0,156463	False	0,156463
ENSMUSG00000042105	Inpp5f	353,9601534	157,7492833	394,5063453	309,2657897	122,6464433	0,156461545	False	0,156461545
ENSMUSG00000026887	Mrrf	360,1428635	271,3287672	323,1781892	152,9459905	224,4168962	-0,15623948	False	0,15623948
ENSMUSG00000028521	Slc35d1	820,7547661	914,9458429	1344,667833	736,6148809	1316,492141	-0,15604068	False	0,15604068
ENSMUSG00000029073	Cptp	251,1725979	194,347117	225,4321973	120,3325073	185,2744143	-0,155985497	False	0,155985497
ENSMUSG00000030172	Erc1	304,4984725	292,7826697	212,2232795	255,2848519	339,2348431	0,155849367	False	0,155849367
ENSMUSG00000020899	Pfas	82,69374762	92,12558143	223,6710083	168,6904307	133,0844384	0,155823566	False	0,155823566
ENSMUSG00000021492	F12	6972,551318	7767,574708	5073,105035	6095,347564	7764,563659	0,155777565	False	0,155777565
ENSMUSG00000037260	Hgsnat	698,6462416	509,8456835	509,8642276	778,2251872	572,7849851	0,15562554	False	0,15562554
ENSMUSG00000040746	Rnf167	776,7029566	1119,388914	697,4308605	978,4044983	2142,398509	-0,155312755	False	0,155312755
ENSMUSG00000031844	Hsd17b2	3176,367315	1516,917108	2852,245653	853,5735796	1950,600348	-0,155279514	False	0,155279514
ENSMUSG00000006315	Tmem147	782,8856667	389,9562282	871,7885756	508,320498	230,9406432	0,155176668	False	0,155176668
ENSMUSG00000068479	Mfap1a	91,19497401	152,7013062	81,89529044	120,3325073	213,978901	-0,155173827	False	0,155173827
ENSMUSG00000018160	Med1	338,5033781	280,1627271	222,7904138	175,438048	304,0066094	-0,155067542	False	0,155067542

ENSMUSG00000027860	Vangl1	101,2418779	177,9411915	112,7160987	139,4507561	178,7506673	0,15488741	False	0,15488741
ENSMUSG00000040365	Trim41	404,9675117	863,204078	450,8643947	472,3332061	1045,104267	0,15488741	False	0,15488741
ENSMUSG00000021427	Ssr1	1637,645338	4048,477606	2782,678686	1470,980556	6512,004238	-0,154844773	False	0,154844773
ENSMUSG00000022407	Adsl	176,9800767	162,7972603	129,4473946	120,3325073	197,0171589	0,154734324	False	0,154734324
ENSMUSG00000020116	Pno1	367,8712511	801,366359	409,4764522	434,0967084	883,3153414	0,154579554	False	0,154579554
ENSMUSG00000003955	Fam162a	363,2342185	121,1514495	404,1928851	105,7126699	134,3891878	0,154143808	False	0,154143808
ENSMUSG00000027522	Stx16	455,9748701	507,321695	788,1320962	601,6625363	791,9828837	0,153946541	False	0,153946541
ENSMUSG00000036661	Dennd3	124,4270408	242,3028991	111,8355041	501,5728807	332,7110961	-0,15392178	False	0,15392178
ENSMUSG00000036980	Taf6	294,4515686	203,1810768	327,5811618	320,5118184	74,3707156	0,153822035	False	0,153822035
ENSMUSG00000030835	Nomo1	523,2118424	1312,474037	582,0729783	1331,5298	1673,993476	0,153804849	False	0,153804849
ENSMUSG00000041040	Fam117b	69,55548865	124,9374323	62,52221098	87,71902399	452,7480406	-0,153795565	False	0,153795565
ENSMUSG00000043091	Tuba1c	933,5892255	942,7097168	884,9974935	383,4895792	1038,58052	0,153753254	False	0,153753254
ENSMUSG00000023048	Prr13	762,0190201	589,3513223	627,8638934	411,604651	684,9934332	-0,153736851	False	0,153736851
ENSMUSG00000025024	Smndc1	200,1652396	296,5686525	203,4173343	179,9364595	589,7467272	-0,153703918	False	0,153703918
ENSMUSG00000036890	Gtdc1	84,23942515	137,557375	75,73112879	138,3261532	80,89446258	-0,153609159	False	0,153609159
ENSMUSG00000061313	Ddhd2	128,2912346	227,1589679	115,3578822	367,745139	197,0171589	-0,153306018	False	0,153306018
ENSMUSG00000027194	Ttc17	418,8786094	749,6245941	465,8345015	593,7903162	1141,655722	0,153285282	False	0,153285282
ENSMUSG00000030189	Ybx3	523,9846812	810,2003189	610,252003	471,2086032	1461,319324	-0,153158755	False	0,153158755
ENSMUSG00000022295	Atp6v1c1	321,5009253	301,6166296	321,4170001	291,2721437	357,5013346	0,153125738	False	0,153125738
ENSMUSG00000055862	Izumo4	245,7627266	11,3579484	221,0292247	22,49205743	11,74274457	-0,15302899	False	0,15302899
ENSMUSG00000028420	Tmem38b	507,7550672	248,6128704	265,9395453	123,7063159	456,6622888	-0,153005075	False	0,153005075
ENSMUSG00000029036	Atad3a	682,4166276	395,0042053	613,774381	438,5951199	358,806084	-0,15294437	False	0,15294437
ENSMUSG00000024142	Mlst8	94,28632906	90,86358716	106,551937	172,0642394	84,80871077	-0,152836151	False	0,152836151
ENSMUSG00000030357	Fkbp4	2857,184906	128,7234151	3176,304437	285,6491294	183,9696649	0,152754791	False	0,152754791
ENSMUSG00000034449	Dhrs11	367,0984123	215,8010195	330,2229453	290,1475409	212,6741516	-0,152726506	False	0,152726506
ENSMUSG00000009112	Bcl2l13	411,9230606	330,6424977	243,0440878	212,5499427	370,5488286	-0,15271123	False	0,15271123
ENSMUSG00000020647	Ncoa1	253,4911142	575,4693854	281,7902467	418,3522682	624,9749609	0,152686502	False	0,152686502
ENSMUSG00000015312	Gadd45b	55,64439092	141,3433578	324,0587837	61,85315794	358,806084	0,152610986	False	0,152610986
ENSMUSG00000034850	Tmem127	476,8415166	530,0375918	284,4320302	304,7673782	410,9960599	0,152584832	False	0,152584832
ENSMUSG00000030180	Kdm5a	252,7182754	719,3367317	280,9096522	912,0529289	932,8958185	0,1525762	False	0,1525762
ENSMUSG00000022339	Ebag9	242,6713715	218,325008	174,3577151	141,6999618	204,8456553	-0,152526527	False	0,152526527
ENSMUSG00000019428	Fkbp8	5209,7061	8364,497996	8051,275704	7240,193287	5790,477822	0,152480415	False	0,152480415
ENSMUSG00000031278	Acsl4	548,7155216	393,742211	409,4764522	493,7006606	349,6728383	-0,15242181	False	0,15242181
ENSMUSG00000021990	Spata13	378,6909938	340,7384519	286,1932193	308,1411868	146,1319324	-0,152356348	False	0,152356348
ENSMUSG00000039456	Morc3	153,7949138	199,395094	235,9993316	320,5118184	170,9221709	0,152331754	False	0,152331754
ENSMUSG00000035891	Cerk	77,28387628	292,7826697	69,56696715	365,4959333	130,4749397	-0,15176503	False	0,15176503
ENSMUSG00000003345	Csnk1g2	1148,438402	1818,533738	1033,817967	1449,613102	1471,757319	-0,151691296	False	0,151691296
ENSMUSG00000021639	Gtf2h2	139,1109773	71,93367317	102,1489644	98,9650527	125,2559421	-0,151357222	False	0,151357222
ENSMUSG00000026037	Orc2	61,05426226	160,2732718	67,80577811	118,0833015	271,3878745	0,151316204	False	0,151316204
ENSMUSG00000021891	Mettl6	122,1085245	448,0079645	135,6115562	354,2499046	643,2414525	0,151316204	False	0,151316204
ENSMUSG00000014748	Tex261	939,7719356	728,1706916	846,2513345	594,9149191	654,984197	-0,151224481	False	0,151224481
ENSMUSG00000032705	Exd2	152,2492363	103,4835298	169,074148	152,9459905	99,16095413	0,151221093	False	0,151221093
ENSMUSG00000020803	Txndc17	624,4537203	379,8602741	465,8345015	170,9396365	562,3469899	-0,15113405	False	0,15113405
ENSMUSG00000000134	Tfe3	464,4760964	999,4994588	418,2823974	464,460986	1966,257341	-0,151127066	False	0,151127066
ENSMUSG00000024069	Slc30a6	212,5306598	145,1293406	235,9993316	222,6713686	103,0752023	0,151111793	False	0,151111793
ENSMUSG00000032279	Idh3a	334,6391843	349,5724117	588,2371399	507,1958951	301,3971106	-0,1509408	False	0,1509408
ENSMUSG00000030842	Lamtor1	611,3154614	425,2920677	381,2974275	290,1475409	550,6042453	-0,150901323	False	0,150901323
ENSMUSG00000032754	Slc8b1	462,9304189	287,7346927	322,2975946	513,9435123	199,6266577	0,150814437	False	0,150814437
ENSMUSG00000028826	Tmem57	537,8957789	400,0521824	557,4163317	597,1641248	285,7401178	0,150800829	False	0,150800829
ENSMUSG00000038650	Rnh1	1085,065623	2482,342721	1454,742148	1204,449676	2716,488244	0,150591819	False	0,150591819
ENSMUSG00000025404	R3hdm2	1417,386291	1345,285888	1254,847192	1573,319417	1317,79689	0,150578598	False	0,150578598
ENSMUSG00000032578	Cish	380,2366713	757,1965597	342,5512686	558,9276272	371,853578	-0,150577749	False	0,150577749
ENSMUSG00000041341	Atg2b	347,0046045	227,1589679	168,1935535	312,6395983	139,6081854	-0,150454288	False	0,150454288
ENSMUSG00000031446	Cul4a	1118,29769	803,8903475	665,7294578	1007,644173	532,3377538	-0,15031801	False	0,15031801
ENSMUSG00000028639	Ybx1	4412,136497	2747,361517	2573,09719	2141,243868	3975,571411	-0,150315189	False	0,150315189
ENSMUSG00000028063	Lmna	515,4834548	630,9971331	857,6990633	504,9466894	464,4907852	-0,150276147	False	0,150276147
ENSMUSG00000031217	Efnb1	275,9034383	248,6128704	221,0292247	127,0801245	139,6081854	-0,150262451	False	0,150262451
ENSMUSG00000019864	Rtn4ip1	236,4886614	71,93367317	213,103874	128,2047274	71,76121681	-0,150214194	False	0,150214194
ENSMUSG00000032126	Hmbs	724,9227595	468,1998727	653,4011345	500,4482779	585,832479	-0,149858325	False	0,149858325
ENSMUSG00000023952	Gtpbp2	410,377383	1795,817841	455,2673673	3029,680136	1372,596365	0,149762831	False	0,149762831
ENSMUSG00000098234	Snhg6	87,3307802	296,5686525	161,1487973	78,72220101	414,9103081	-0,149719671	False	0,149719671
ENSMUSG00000020640	Itsn2	467,5674515	800,1043647	518,6701728	823,209302	652,3746983	0,149642903	False	0,149642903
ENSMUSG00000047409	Ctdspl	148,3850425	133,7713922	182,2830658	328,3840385	163,0936746	-0,149576052	False	0,149576052
ENSMUSG00000036256	Igfbp7	1464,529456	1119,388914	1140,369905	583,6688904	1320,406389	-0,149455185	False	0,149455185
ENSMUSG00000042719	Naa25	231,8516288	506,0597007	257,1336001	669,1387086	484,0620261	0,14931628	False	0,14931628
ENSMUSG00000019920	Lims1	263,5380181	204,4430711	148,820474	190,0578853	292,2638648	0,149260366	False	0,149260366
ENSMUSG00000033577	Myo6	659,2314647	321,8085379	531,8790906	730,9918666	257,0356311	0,149070251	False	0,149070251
ENSMUSG00000029344	Tpst2	363,2342185	248,6128704	327,5811618	325,0102299	221,8073974	-0,149047724	False	0,149047724
ENSMUSG00000031393	Mecp2	237,2615002	377,3362856	213,9844686	391,3617993	503,6332671	-0,14897193	False	0,14897193
ENSMUSG00000028042	Zbtb7b	1299,14196	1153,462759	1161,504173	1171,836192	795,8971319	-0,148788173	False	0,148788173
ENSMUSG00000019564	Arid3a	28,59503422	191,8231285	31,70140275	263,157072	225,7216456	0,148782047	False	0,148782047
ENSMUSG00000056204	Pgpep1	548,7155216	608,2812363	581,1923838	208,0515312	292,2638648	0,148680116	False	0,148680116
ENSMUSG00000043681	Fam25c	258,9009855	442,9599874	2227,904138	440,8443257	233,550142	-0,148668135	False	0,148668135
ENSMUSG00000102644	Thap6	101,2418779	82,0296273	69,56696715	61,85315794	91,33245776	-0,148606611	False	0,148606611
ENSMUSG00000039427	Alg1	230,3059513	320,5465436	273,864896	255,2848519	382,2915732	0,148556241	False	0,148556241
ENSMUSG00000061904	Slc25a3	3320,115325	4716,072573	3724,914823	3679,700596	6431,109775	0,148355029	False	0,148355029
ENSMUSG00000023904	Hcfc1r1	1102,840915	252,3988532	1222,265195	215,9237514	431,8720502	0,148332644	False	0,148332644
ENSMUSG00000070426	Rnf121	196,3010458	122,4134438	217,5068467	139,4507561	32,61873491	0,147992956	False	0,147992956
ENSMUSG00000068083	Cyp2d40	719,5128882	398,7901881	733,5352359	181,0610623	797,2018813	0,147924585	False	0,147924585
ENSMUSG00000024073	Birc6	1027,875555	771,0784966	690,3861044	927,7973691	699,3456765	-0,147783947	False	0,147783947
ENSMUSG00000022359	Wdyhv1	119,0171695	47,95578211	107,4325315	68,60077517	91,33245776	-0,147738792	False	0,147738792
ENSMUSG00000029826	Zc3hav1	781,3399892	465,6758842	705,3562112	230,5435887	640,6319537	-0,147598438	False	0,147598438
ENSMUSG00000000385	Tmprss2	95,05916782	85,8156101	149,7010685	271,0292921	129,1701903	-0,14758567	False	0,14758567
ENSMUSG00000061118	Dnajc30	499,2538408	349,5724117	553,0133591	209,1761341	454,05279	0,147540806	False	0,147540806
ENSMUSG00000037300	Ttc13	398,0119628	198,1330998	361,9243481	440,8443257	117,4274457	0,147457496	False	0,147457496
ENSMUSG00000033769	Exoc6b	110,5159431	378,5982798	122,4026384	152,9459905	362,7203322	0,147380147	False	0,147380147
ENSMUSG00000055239	Kcmf1	651,503077	1071,433132	588,2371399	592,6657133	1509,595052	-0,147374117	False	0,147374117
ENSMUSG00000028165	Cisd2	386,4193814	355,8823831	269,4619234	248,5372346	427,9578021	0,147301093	False	0,147301093
ENSMUSG00000041632	Mrps27	598,1772024	570,4214083	922,8630579	1039,133053	540,1662502	-0,147169425	False	0,147169425
ENSMUSG00000020362	Cnot6	435,8810622	885,9199748	393,6257508	934,5449863	855,9156041	-0,14710992	False	0,14710992
ENSMUSG00000024097	Srsf7	391,056414	678,9529152	565,3416824	432,9721056	973,3430498	0,146897335	False	0,146897335
ENSMUSG00000030512	Snrpa1	106,6517493	135,0333865	187,5666329	118,0833015	164,398424	0,146897335	False	0,146897335
ENSMUSG00000030647	Ndufc2	687,0536601	471,9858555	485,207581	69,72537804	760,6688982	0,146845838	False	0,146845838
ENSMUSG00000033863	Klf9	950,5916782	4525,511438	1052,310452	4056,442558	4541,832649	0,146662714	False	0,146662714
ENSMUSG00000001729	Akt1	1078,110074	1880,371457	1193,205576	1271,925848	1518,728298	0,146338141	False	0,146338141
ENSMUSG00000023923	Tbc1d5	214,0763373	94,64956996	129,4473946	193,4316939	24,79023853	-0,146301137	False	0,146301137
ENSMUSG00000047789	Slc38a9	164,6146565	577,9933739	244,8052768	1510,341657	148,7414312	-0,146286233	False	0,146286233
ENSMUSG00000026435	Slc45a3	635,273463	702,9308062	520,4313618	417,2276654	233,550142	0,146004928	False	0,146004928
ENSMUSG00000043881	Kbtbd7	114,3801369	59,31373051	43,14913152	17,99364595	126,5606915	0,14599285	False	0,14599285
ENSMUSG00000031256	Cstf2	228,7602738	135,0333865	134,7309617	80,97140676	253,1213829	0,14599285	False	0,14599285
ENSMUSG00000033589	Reep4	141,4294936	156,487289	147,059285	113,58489	109,5989493	0,145962466	False	0,145962466
ENSMUSG00000024099	Ndufv2	1571,181205	1437,411469	1738,293584	644,3974454	1311,273143	0,145822186	False	0,145822186
ENSMUSG00000035064	Eef2k	160,7504627	12,61994266	145,2980959	67,4761723	84,80871077	-0,145807093	False	0,145807093
ENSMUSG00000072676	Tmem254a	3496,322563	1031,049315	3867,571136	1463,108336	726,7454139	0,145589536	False	0,145589536
ENSMUSG00000028776	Tinagl1	316,8638927	464,4138899	350,4766193	683,7585459	434,481549	0,145454932	False	0,145454932
ENSMUSG00000070939	Tgfbrap1	666,1870135	430,3400448	602,3266523	502,6974836	515,3760116	-0,145381133	False	0,145381133
ENSMUSG00000066880	Zfp617	193,2096907	194,347117	158,5070138	213,6745456	116,1226963	0,145247598	False	0,145247598
ENSMUSG00000031691	Tnpo2	357,8243472	408,8861422	432,3719097	323,885627	323,5778503	-0,1451387	False	0,1451387
ENSMUSG00000038965	Ube2l3	1820,808125	1235,492387	1220,504006	543,183187	2013,228319	0,144931896	False	0,144931896
ENSMUSG00000027615	Hps3	108,1974268	113,579484	117,9996658	197,9301054	97,85620474	-0,144930953	False	0,144930953
ENSMUSG00000031833	Mast3	562,6266193	772,3404909	996,8329976	1224,692527	508,8522646	-0,144930953	False	0,144930953
ENSMUSG00000027341	Tmem230	173,1158829	128,7234151	141,7757179	100,0896556	156,5699276	-0,144930953	False	0,144930953
ENSMUSG00000035443	Thyn1	208,666466	179,2031858	230,7157645	57,35474645	180,0554167	0,144917816	False	0,144917816

ENSMUSG00000034854	Mfsd12	149,93072	50,47977065	135,6115562	32,61348328	54,79947465	-0,14481589	False	0,14481589
ENSMUSG00000026771	Spopl	107,424588	97,17355849	66,04458906	95,59124409	28,70448672	-0,144688538	False	0,144688538
ENSMUSG00000040521	Tsfm	103,5603942	71,93367317	114,4772877	30,36427753	73,0659662	0,144589034	False	0,144589034
ENSMUSG00000028857	Tmem222	234,9429839	288,9966869	280,9096522	212,5499427	533,6425032	-0,144508815	False	0,144508815
ENSMUSG00000004054	Map3k11	540,2142952	1712,526219	597,0430851	1248,309187	1664,86023	0,144303229	False	0,144303229
ENSMUSG00000020077	Srgn	183,9356255	919,99382	166,4323644	539,8093784	1936,248104	-0,144264928	False	0,144264928
ENSMUSG00000023971	Rrp36	153,022075	169,1072317	155,8652302	103,4634642	155,2651782	0,144198564	False	0,144198564
ENSMUSG00000069792	Wfdc17	275,1305996	147,6533291	122,4026384	60,72855507	304,0066094	0,143986087	False	0,143986087
ENSMUSG00000039230	Tbcd	269,7207282	268,8047787	261,5365727	298,019761	206,1504046	0,143941598	False	0,143941598
ENSMUSG00000021870	Slmap	717,9672106	395,0042053	394,5063453	428,4736941	793,2876331	0,1439261	False	0,1439261
ENSMUSG00000006288	Ttc5	108,9702656	151,4393119	98,62658634	172,0642394	169,6174215	-0,143886019	False	0,143886019
ENSMUSG00000024604	Rbm22	274,3577608	383,6462569	248,3276549	372,2435505	755,4499006	-0,143813451	False	0,143813451
ENSMUSG00000025237	Parp6	210,2121435	358,4063716	306,4468933	645,5220483	232,2453926	0,143803963	False	0,143803963
ENSMUSG00000039356	Exosc2	98,15052288	165,3212489	97,74599182	88,84362686	153,9604288	-0,143727664	False	0,143727664
ENSMUSG00000047248	C2cd3	147,6122037	106,0075184	69,56696715	163,0674164	65,23746983	0,143656536	False	0,143656536
ENSMUSG00000020137	Thap2	125,1998796	186,7751514	193,7307946	295,7705552	138,303436	0,143603825	False	0,143603825
ENSMUSG00000021134	Srsf5	3414,401654	1245,588341	2256,083162	1618,303532	3090,95132	-0,143581847	False	0,143581847
ENSMUSG00000026457	Adipor1	1423,569001	1101,720994	962,4898113	753,483924	1572,223023	0,143293458	False	0,143293458
ENSMUSG00000022636	Alcam	589,675976	651,1890413	357,5213755	407,1062395	287,0448672	0,143154003	False	0,143154003
ENSMUSG00000022789	Dnm1l	826,9374762	612,0672191	913,1765181	822,0846992	823,2968692	0,143115509	False	0,143115509
ENSMUSG00000029104	Htt	318,4095703	403,8381652	310,8498659	547,6815985	288,3496166	-0,143064595	False	0,143064595
ENSMUSG00000037487	Ubr5	1126,026077	2216,061931	1019,728455	2749,654021	4260,00678	-0,143055213	False	0,143055213
ENSMUSG00000042594	Sh2b3	350,0959595	440,4359989	413,8794248	603,9117421	317,0541034	-0,143021363	False	0,143021363
ENSMUSG00000062797	Hikeshi	180,8442705	12,61994266	163,7905809	47,23332061	22,18073974	-0,142895497	False	0,142895497
ENSMUSG00000062627	Mysm1	214,8491761	446,7459702	194,6113891	299,1443639	636,7177055	-0,142728102	False	0,142728102
ENSMUSG00000038485	Socs7	123,654202	256,184836	136,4921507	186,6840767	686,2981826	0,142506722	False	0,142506722
ENSMUSG00000026923	Notch1	321,5009253	244,8268876	259,7753837	291,2721437	232,2453926	-0,142455156	False	0,142455156
ENSMUSG00000033039	Micall1	163,8418177	156,487289	155,8652302	148,4475791	221,8073974	-0,142350061	False	0,142350061
ENSMUSG00000029148	Nrbp1	588,9031373	660,0230012	533,6402796	702,8767948	895,058086	-0,142162793	False	0,142162793
ENSMUSG00000015023	Ddx19a	274,3577608	227,1589679	123,2832329	125,9555216	302,70186	0,141839154	False	0,141839154
ENSMUSG00000050565	Tor1aip2	2069,662207	937,6617397	1512,861387	678,1355316	2283,311444	0,141732335	False	0,141732335
ENSMUSG00000096674	Mup15	387,1922202	18,92991399	27,29843015	1,124602872	350,9775877	-0,141671057	False	0,141671057
ENSMUSG00000051169	Rpusd3	170,0245278	257,4468303	187,5666329	323,885627	375,7678262	0,141660318	False	0,141660318
ENSMUSG00000035781	R3hdm4	412,6958993	242,3028991	455,2673673	218,1729571	266,1688769	0,14163495	False	0,14163495
ENSMUSG00000044433	Camsap3	360,9157022	1225,396432	398,0287234	736,6148809	1454,795577	0,141210634	False	0,141210634
ENSMUSG00000038664	Herc1	454,4291925	512,3696721	412,1182358	885,06246	622,3654621	-0,140997214	False	0,140997214
ENSMUSG00000032392	Parp16	340,8218944	74,4576617	309,0886768	110,2110814	53,49472526	-0,140997214	False	0,140997214
ENSMUSG00000024423	Impact	175,4343992	201,9190826	277,3872741	193,4316939	396,6438165	0,140892542	False	0,140892542
ENSMUSG00000027652	Ralgapb	238,8071777	352,0964003	237,7605206	530,8125554	216,5883998	-0,140890223	False	0,140890223
ENSMUSG00000022827	Rabl3	155,3405913	227,1589679	140,8951233	182,1856652	293,5686142	-0,140813185	False	0,140813185
ENSMUSG00000024132	Eci1	1564,998495	493,4397581	1419,518368	394,7356079	501,0237683	-0,140759753	False	0,140759753
ENSMUSG00000008855	Hdac5	674,6882399	259,9708188	612,013192	553,3046128	434,481549	-0,140658265	False	0,140658265
ENSMUSG00000043013	Onecut1	204,0294334	188,0371457	110,9549096	224,9205743	65,23746983	0,140638345	False	0,140638345
ENSMUSG00000032492	Pth1r	190,1183356	42,90780505	209,581496	148,4475791	19,57124095	0,140613669	False	0,140613669
ENSMUSG00000025353	Ormdl2	639,1376568	524,9896147	404,1928851	311,5149954	704,5646741	0,140605451	False	0,140605451
ENSMUSG00000001998	Ap4e1	76,51103752	69,40968464	102,1489644	179,9364595	96,55145534	-0,140530913	False	0,140530913
ENSMUSG00000026088	Mitd1	123,654202	136,2953807	114,4772877	83,2206125	27,39973733	0,140425401	False	0,140425401
ENSMUSG00000063801	Ap3s2	330,0021517	253,6608475	299,4021371	131,578536	290,9591154	-0,140390912	False	0,140390912
ENSMUSG00000067825	Pex26	236,4886614	75,71965597	260,6559782	129,3293302	33,92348431	0,140375934	False	0,140375934
ENSMUSG00000021928	Ebpl	1417,386291	157,7492833	1562,17468	209,1761341	289,654366	0,140322782	False	0,140322782
ENSMUSG00000031226	Pbdc1	105,1060717	223,3729851	190,2084165	115,8340958	206,1504046	0,14021396	False	0,14021396
ENSMUSG00000022119	Rbm26	254,263953	265,0187959	230,7157645	363,2467275	454,05279	-0,140210361	False	0,140210361
ENSMUSG00000029484	Anxa3	55,64439092	112,3174897	68,68637263	371,1189476	61,32322164	0,140197223	False	0,140197223
ENSMUSG00000053483	Usp21	275,1305996	440,4359989	358,40197	249,6618375	1109,036987	-0,14014129	False	0,14014129
ENSMUSG00000022516	Nudt16l1	187,7998194	116,1034725	206,9397124	94,46664122	74,3707156	0,140014854	False	0,140014854
ENSMUSG00000037395	Rcor3	143,7480099	69,40968464	116,2384768	130,4539331	28,70448672	-0,140001542	False	0,140001542
ENSMUSG00000036073	Galt	1337,01106	1034,835298	1202,011521	1473,229762	431,8720502	0,139971048	False	0,139971048
ENSMUSG00000028479	Gne	1731,158829	540,1335459	1907,367732	1303,414728	409,6913105	0,139844922	False	0,139844922
ENSMUSG00000038872	Zfhx3	143,7480099	311,7125837	381,2974275	383,4895792	130,4749397	-0,139769248	False	0,139769248
ENSMUSG00000052512	Nav2	959,0929046	870,7760436	1437,130258	2341,423179	1679,212473	-0,139368854	False	0,139368854
ENSMUSG00000031201	Brcc3	247,3084041	397,5281938	224,5516028	328,3840385	544,0804983	-0,139264247	False	0,139264247
ENSMUSG00000059495	Arhgef12	1271,319765	1468,961326	1154,459417	1320,283771	2298,968437	-0,139109487	False	0,139109487
ENSMUSG00000036850	Mrpl41	268,1750507	295,3066583	247,4470604	195,6808997	200,9314071	0,139038861	False	0,139038861
ENSMUSG00000030417	Pdcd5	326,9107967	798,8423705	517,7895783	296,8951581	704,5646741	-0,138943459	False	0,138943459
ENSMUSG00000022194	Pabpn1	264,3108569	251,136859	333,7453234	655,6434742	240,073889	-0,138757186	False	0,138757186
ENSMUSG00000027447	Cst3	1728,067474	1153,462759	1457,383932	696,1291775	1569,613524	-0,138750172	False	0,138750172
ENSMUSG00000024395	Lims2	1168,532209	1321,307997	2293,948727	2122,125619	1286,482905	0,138734785	False	0,138734785
ENSMUSG00000022982	Sod1	7876,77267	5459,387195	7154,830482	1563,197992	6149,283906	-0,138687053	False	0,138687053
ENSMUSG00000030282	Cmas	426,6069971	333,1664863	287,0738138	368,8697419	387,5105708	-0,138671963	False	0,138671963
ENSMUSG00000032381	Fam96a	597,4043636	542,6575344	533,6402796	194,5562968	465,7955346	-0,13866576	False	0,13866576
ENSMUSG00000024913	Lrp5	1003,917553	776,1264737	652,52054	757,9823355	912,0198282	-0,138503697	False	0,138503697
ENSMUSG00000032883	Acsl3	705,6017904	102,2215356	641,0728112	231,6681916	43,05673008	-0,138365998	False	0,138365998
ENSMUSG00000070985	Acnat1	411,9230606	36,59783372	374,2526714	112,4602872	1,304749397	-0,13836228	False	0,13836228
ENSMUSG00000024238	Zeb1	262,7651794	453,0559415	281,7902467	475,7070147	238,7691396	-0,138157725	False	0,138157725
ENSMUSG00000036854	Hspb6	333,0935068	427,8160562	529,237307	536,4355698	302,70186	-0,138029691	False	0,138029691
ENSMUSG00000019777	Hdac2	333,0935068	241,0409048	214,8650631	104,5880671	302,70186	-0,138029691	False	0,138029691
ENSMUSG00000014074	Rnf168	190,8911744	368,5023257	173,4771206	405,9816367	345,7585901	-0,138005001	False	0,138005001
ENSMUSG00000038195	Rilp	538,6686177	237,254922	592,6401125	322,7610242	73,0659662	0,13775826	False	0,13775826
ENSMUSG00000005881	Ergic3	1122,161884	721,8607202	1234,593518	575,7966703	623,6702115	0,137755307	False	0,137755307
ENSMUSG00000050310	Rictor	271,2664057	323,0705321	246,5664658	254,160249	383,5963226	-0,137733792	False	0,137733792
ENSMUSG00000035451	Foxa1	257,355308	99,69754702	198,1337672	118,0833015	283,130619	0,137706239	False	0,137706239
ENSMUSG00000055334	Snupn	89,64929649	85,8156101	98,62658634	86,59442111	71,76121681	0,137684338	False	0,137684338
ENSMUSG00000006705	Pknox1	193,2096907	123,6754381	170,835337	212,5499427	73,0659662	0,137634414	False	0,137634414
ENSMUSG00000038384	Setd1b	381,7823488	347,0484232	480,8046084	534,186364	365,329831	-0,137613429	False	0,137613429
ENSMUSG00000029440	Psmd9	291,3602136	384,9082512	339,9094851	320,5118184	601,4894718	0,13757367	False	0,13757367
ENSMUSG00000036552	Ermard	140,6566548	156,487289	235,9993316	383,4895792	127,8654409	-0,137551423	False	0,137551423
ENSMUSG00000002396	Ocel1	111,2887818	79,50563877	122,4026384	66,35156943	44,36147948	0,137326483	False	0,137326483
ENSMUSG00000032059	Alg9	252,7182754	230,9449507	206,9397124	86,59442111	277,9116215	0,137096168	False	0,137096168
ENSMUSG00000030120	Mlf2	2088,210337	1881,633451	2044,740477	1072,87114	2296,358938	0,137081127	False	0,137081127
ENSMUSG00000032422	Snx14	272,0392445	129,9854094	150,5816631	299,1443639	112,2084481	0,137027091	False	0,137027091
ENSMUSG00000061455	Stx17	421,1971257	463,1518957	220,1486302	285,6491294	241,3786384	0,136989827	False	0,136989827
ENSMUSG00000035203	Epn1	2801,540515	2679,213827	2872,499327	2433,640614	3080,513325	0,13695042	False	0,13695042
ENSMUSG00000030042	Pole4	197,8467233	349,5724117	253,611222	179,9364595	259,6451299	-0,136895631	False	0,136895631
ENSMUSG00000078572	Ndufaf8	150,7035587	199,395094	241,2828987	167,5658279	165,7031734	0,136887746	False	0,136887746
ENSMUSG00000002307	Daxx	187,7998194	309,1885952	170,835337	219,29756	322,2731009	-0,13658925	False	0,13658925
ENSMUSG00000002833	Hdgfl2	354,7329921	389,9562282	564,4610879	908,6791203	399,2533153	0,136578678	False	0,136578678
ENSMUSG00000055024	Ep300	486,8884206	661,2849955	442,939044	490,326852	895,058086	-0,136483017	False	0,136483017
ENSMUSG00000056216	Cebpg	602,0413962	547,7055115	475,5210413	443,0935314	194,4076601	-0,136462292	False	0,136462292
ENSMUSG00000005575	Ube2m	505,4365509	894,7539347	532,7596851	459,9625745	887,2295896	-0,136013518	False	0,136013518
ENSMUSG00000028902	Sf3a3	249,6269204	261,2328131	262,4171672	227,1697801	448,8337924	-0,136002598	False	0,136002598
ENSMUSG00000023175	Bsg	6204,349588	2954,328577	6817,56278	2826,127016	3785,077999	0,135976102	False	0,135976102
ENSMUSG00000041540	Sox5	212,5306598	80,76763303	140,8951233	193,4316939	67,84696862	-0,135846779	False	0,135846779
ENSMUSG00000044475	Ascc1	151,4763975	74,4576617	166,4323644	66,35156943	43,05673008	0,135842991	False	0,135842991
ENSMUSG00000039601	Rcan2	233,3973064	331,904492	259,7753837	256,4094547	300,0923612	0,135661549	False	0,135661549
ENSMUSG00000033191	Tie1	112,8344594	157,7492833	221,9098193	429,598297	123,9511927	0,135564423	False	0,135564423
ENSMUSG00000017639	Rab11fip4	78,82955381	119,8894553	172,5965261	309,2657897	71,76121681	-0,135532255	False	0,135532255
ENSMUSG00000010175	Prox1	1522,492363	1230,444409	1386,055776	809,7140676	664,1174428	-0,135449677	False	0,135449677
ENSMUSG00000022246	Rai14	550,2611991	921,2558143	922,8630579	1195,452853	501,0237683	-0,135237557	False	0,135237557
ENSMUSG00000031979	Cog2	200,1652396	1537,109016	182,2830658	733,2410723	1300,835148	-0,135010919	False	0,135010919
ENSMUSG00000026439	Rbbp5	305,2713113	248,6128704	280,0290576	335,1316557	234,8548914	0,134635952	False	0,134635952
ENSMUSG00000028973	Abcb8	329,229313	647,4030585	363,6855371	802,9664503	361,4155828	0,134565919	False	0,134565919
ENSMUSG00000031537	Ikbkb	779,0214729	1307,42606	855,0572798	1854,470135	1008,571284	0,134357973	False	0,134357973
ENSMUSG00000021823	Vcl	330,7749905	244,8268876	281,7902467	195,6808997	301,3971106	-0,134184572	False	0,134184572

ENSMUSG00000056310	Tyw1	159,2047851	63,09971331	92,46242469	145,0737704	32,61873491	-0,134096997	False	0,134096997
ENSMUSG00000031708	Tecr	4987,128536	2025,500797	5472,894947	1756,629685	2151,531755	0,134094777	False	0,134094777
ENSMUSG00000045659	Plekha7	92,74065154	146,3913349	141,7757179	192,307091	101,7704529	0,134044996	False	0,134044996
ENSMUSG00000000552	Zfp385a	438,9724173	233,4689392	481,6852029	161,9428135	298,7876118	0,133960315	False	0,133960315
ENSMUSG00000024234	Mtpap	191,6640132	314,2365723	210,4620905	210,300737	705,8694235	0,133874424	False	0,133874424
ENSMUSG00000030291	Med21	250,3997591	199,395094	274,7454905	77,59759814	268,7783757	0,133862627	False	0,133862627
ENSMUSG00000007892	Rplp1	4920,664403	7814,268496	9351,913811	4484,916252	7087,398722	-0,13377208	False	0,13377208
ENSMUSG00000053134	Supt7l	148,3850425	162,7972603	210,4620905	255,2848519	236,1596408	0,133730749	False	0,133730749
ENSMUSG00000023110	Prmt5	249,6269204	715,5507489	273,864896	621,905388	615,8417152	0,133690827	False	0,133690827
ENSMUSG00000005087	Cd44	77,28387628	80,76763303	70,44756167	83,2206125	200,9314071	-0,133617684	False	0,133617684
ENSMUSG00000020287	Mpg	103,5603942	261,2328131	113,5966932	362,1221247	114,8179469	0,133448476	False	0,133448476
ENSMUSG00000021707	Dhfr	476,0686779	214,5390252	522,1925509	115,8340958	176,1411685	0,133412165	False	0,133412165
ENSMUSG00000062248	Cks2	144,5208486	27,76387386	158,5070138	3,373808615	52,18997586	0,133269048	False	0,133269048
ENSMUSG00000059436	Max	244,217049	342,0004461	299,4021371	222,6713686	805,0303776	-0,133247854	False	0,133247854
ENSMUSG00000022620	Arsa	238,0343389	156,487289	169,9547425	217,0483542	100,4657035	-0,133153229	False	0,133153229
ENSMUSG00000041408	Wapl	754,2906325	493,4397581	314,3722439	441,9689285	827,2111174	0,133135066	False	0,133135066
ENSMUSG00000027200	Sema6d	263,5380181	288,9966869	326,7005672	543,183187	314,4446046	0,133041853	False	0,133041853
ENSMUSG00000026469	Xpr1	421,1971257	288,9966869	210,4620905	240,6650145	461,8812864	0,133026504	False	0,133026504
ENSMUSG00000026385	Dbi	9743,178283	5027,785156	8885,198715	2074,892298	4779,297039	-0,132988423	False	0,132988423
ENSMUSG00000038371	Sbf2	433,5625459	696,6208349	395,3869399	1163,963972	678,4696862	-0,132974915	False	0,132974915
ENSMUSG00000002103	Acp2	1103,613753	598,1852821	1109,549096	1210,07269	232,2453926	0,132858373	False	0,132858373
ENSMUSG00000026594	Ralgps2	219,4862086	145,1293406	152,3428521	200,1793111	125,2559421	-0,132837414	False	0,132837414
ENSMUSG00000031731	Ap1g1	1047,196524	760,9825425	546,8491975	730,9918666	1148,179469	0,13281595	False	0,13281595
ENSMUSG00000020400	Tnip1	122,8813633	254,9228418	134,7309617	275,5277035	473,6240309	0,132815299	False	0,132815299
ENSMUSG00000030126	Tmcc1	454,4291925	575,4693854	562,6998988	498,1990721	554,5184935	0,132666818	False	0,132666818
ENSMUSG00000031077	Fadd	142,9751711	89,6015929	156,7458247	106,8372728	22,18073974	0,132662382	False	0,132662382
ENSMUSG00000025076	Casp7	286,723181	45,43179358	261,5365727	62,97776081	61,32322164	-0,132645844	False	0,132645844
ENSMUSG00000035958	Tdp2	383,3280264	165,3212489	212,2232795	265,4062777	349,6728383	-0,132573747	False	0,132573747
ENSMUSG00000028413	B4galt1	629,8635917	892,2299462	1095,459584	574,6720674	821,9921198	-0,132300494	False	0,132300494
ENSMUSG00000033486	Catsper2	44,05180948	80,76763303	197,2531727	228,2943829	48,27572767	0,132096725	False	0,132096725
ENSMUSG00000022125	Cln5	371,7354449	224,6349794	240,4023042	191,1824882	339,2348431	-0,131991898	False	0,131991898
ENSMUSG00000000957	Mmp14	483,7970655	1042,407264	530,1179016	779,34979	558,4327417	0,131911241	False	0,131911241
ENSMUSG00000069917	Hba-a2	656,1401096	771,0784966	999,4747812	1572,194815	718,9169175	0,131821138	False	0,131821138
ENSMUSG00000028001	Fga	76538,08687	183639,0956	69859,32453	85618,26582	265015,4784	-0,131725147	False	0,131725147
ENSMUSG00000023495	Pcbp4	190,1183356	696,6208349	479,9240139	840,0783451	173,5316697	-0,131698697	False	0,131698697
ENSMUSG00000027002	Nckap1	1441,344293	1625,448615	1315,608214	1339,40202	2081,075287	-0,131685071	False	0,131685071
ENSMUSG00000049401	Ogfr	435,8810622	335,6904748	328,4617563	201,303914	477,5382791	0,131681859	False	0,131681859
ENSMUSG00000005610	Eif4g2	5708,959941	3447,768335	2670,843182	3380,556232	5212,473839	-0,131259701	False	0,131259701
ENSMUSG00000004043	Stat5a	238,0343389	204,4430711	260,6559782	160,8182106	131,779689	0,130977236	False	0,130977236
ENSMUSG00000039221	Rpl22l1	78,05671504	225,8969736	230,7157645	85,46981824	305,3113588	0,130892305	False	0,130892305
ENSMUSG00000005510	Ndufs3	872,5349632	570,4214083	796,9380414	237,2912059	717,6121681	-0,130745377	False	0,130745377
ENSMUSG00000001017	Chtop	624,4537203	487,1297867	493,1329317	439,7197228	683,6886838	0,130744892	False	0,130744892
ENSMUSG00000035382	Pcsk7	348,550282	401,3141766	353,1184029	606,1609478	318,3588527	-0,130712913	False	0,130712913
ENSMUSG00000049502	Dtx3l	658,4586259	200,6570883	601,4460577	272,1538949	167,0079228	-0,130657441	False	0,130657441
ENSMUSG00000089809	Rasgef1b	93,5134903	225,8969736	85,41766852	190,0578853	241,3786384	-0,130639984	False	0,130639984
ENSMUSG00000026694	Mettl13	98,92336164	148,9153234	188,4472275	275,5277035	108,2941999	0,130572805	False	0,130572805
ENSMUSG00000011096	Akt1s1	673,9154012	1202,680536	854,1766852	737,7394838	1123,38923	0,130543954	False	0,130543954
ENSMUSG00000038368	Focad	139,1109773	23,97789106	103,0295589	127,0801245	30,00923612	-0,13049786	False	0,13049786
ENSMUSG00000028064	Sema4a	1598,230561	1409,647595	1460,025716	1364,143283	755,4499006	-0,130481767	False	0,130481767
ENSMUSG00000025586	Cpeb1	85,78510267	141,3433578	78,37291236	181,0610623	82,19921198	-0,130372024	False	0,130372024
ENSMUSG00000044894	Uqcrq	3385,033781	5885,941257	8610,453225	3092,657897	4660,564844	-0,130322977	False	0,130322977
ENSMUSG00000034341	Wbp2	2103,667112	1922,017267	1887,994653	1152,717943	1851,439394	-0,130285131	False	0,130285131
ENSMUSG00000036555	Iqce	117,4714919	29,02586812	128,5668	83,2206125	23,48548914	0,130207457	False	0,130207457
ENSMUSG00000040006	Ginm1	365,5527348	859,4180952	425,3271536	334,0070529	1401,300852	-0,130200978	False	0,130200978
ENSMUSG00000039653	Baat	3727,401353	4078,765468	2501,769034	3230,98405	1705,307461	0,129962382	False	0,129962382
ENSMUSG00000018583	G3bp1	693,2363702	633,5211216	883,2363044	730,9918666	644,5462019	-0,129954627	False	0,129954627
ENSMUSG00000037152	Ndufc1	507,7550672	667,5949668	1108,668502	555,5538186	605,40372	0,129793946	False	0,129793946
ENSMUSG00000032679	Cd59a	1230,35931	560,3254542	1314,72762	1346,149637	300,0923612	0,129759091	False	0,129759091
ENSMUSG00000020935	Dcakd	373,2811224	540,1335459	549,490981	583,6688904	408,3865611	0,129672841	False	0,129672841
ENSMUSG00000001062	Vps9d1	289,8145361	463,1518957	374,2526714	605,0363449	317,0541034	0,129599091	False	0,129599091
ENSMUSG00000028099	Polr3c	340,0490556	965,4256136	310,8498659	453,2149573	998,1332883	-0,129524929	False	0,129524929
ENSMUSG00000028932	Psmc2	1042,559491	984,3555276	953,6838661	375,6173591	1140,350973	0,12934821	False	0,12934821
ENSMUSG00000025169	Ogfod3	87,3307802	94,64956996	132,9697726	79,84680388	87,41820957	-0,129255569	False	0,129255569
ENSMUSG00000017478	Zc3h18	751,1992774	813,9863017	686,8637263	859,1965939	1059,45651	-0,129171779	False	0,129171779
ENSMUSG00000029141	Slc4a1ap	173,8887216	159,0112775	118,8802603	152,9459905	135,6939372	-0,129035274	False	0,129035274
ENSMUSG00000017344	Vtn	34538,93715	97057,45502	74733,4152	31587,84546	87771,79665	-0,128854151	False	0,128854151
ENSMUSG00000040093	Bmf	162,2961402	116,1034725	28,17902467	148,4475791	67,84696862	-0,128675124	False	0,128675124
ENSMUSG00000041258	Zfp236	269,7207282	353,3583945	399,7899125	392,4864022	294,8733636	0,128629109	False	0,128629109
ENSMUSG00000063108	Zfp26	109,7431043	215,8010195	100,3877754	247,4126318	125,2559421	-0,128546694	False	0,128546694
ENSMUSG00000021266	Wars	285,1775035	494,7017523	311,7304604	618,5315794	835,0396138	0,128438955	False	0,128438955
ENSMUSG00000067889	Sptbn2	792,1597419	273,8527558	724,7292907	644,3974454	76,98021439	-0,128349163	False	0,128349163
ENSMUSG00000041966	Dcaf17	179,298593	164,0592546	120,6414494	134,9523446	69,15171802	-0,128147188	False	0,128147188
ENSMUSG00000005949	Ctns	207,8936272	590,6133166	227,1933864	292,3967466	674,555438	0,128075304	False	0,128075304
ENSMUSG00000056938	Acbd4	750,4264387	214,5390252	686,8637263	483,5792348	57,40897345	-0,12768676	False	0,12768676
ENSMUSG00000021243	Fcf1	261,2195018	860,6800895	285,3126248	509,4451008	1192,540948	0,127280978	False	0,127280978
ENSMUSG00000013701	Timm23	457,5205476	265,0187959	175,2383097	114,7094929	499,7190189	0,127280589	False	0,127280589
ENSMUSG00000005089	Slc1a2	1514,763975	618,3771904	1386,93637	292,3967466	1235,597678	-0,127191414	False	0,127191414
ENSMUSG00000024247	Pkdcc	209,4393047	151,4393119	186,6860384	145,0737704	191,7981613	-0,126943323	False	0,126943323
ENSMUSG00000057738	Sptan1	1283,685185	1824,843709	1175,593685	2054,649446	1446,967081	-0,12690192	False	0,12690192
ENSMUSG00000045827	Serpinb9	155,3405913	76,98165023	79,25350688	76,47299527	169,6174215	0,126849495	False	0,126849495
ENSMUSG00000022967	Ifnar1	461,3847414	292,7826697	310,8498659	457,7133687	503,6332671	0,126403289	False	0,126403289
ENSMUSG00000034456	Uroc1	7126,346232	376,0742913	6528,727778	997,5227471	528,4235056	-0,12636069	False	0,12636069
ENSMUSG00000006818	Sod2	2091,301692	9454,861042	2282,500998	3436,786376	11177,78808	0,12621429	False	0,12621429
ENSMUSG00000022772	Senp5	229,5331126	181,7271743	200,7755508	217,0483542	250,5118841	0,126176753	False	0,126176753
ENSMUSG00000034875	Nudt19	515,4834548	453,0559415	346,9542412	200,1793111	472,3192815	-0,126163691	False	0,126163691
ENSMUSG00000002661	Alkbh7	127,5183959	32,81185092	139,1339343	35,98729189	20,87599034	0,125768945	False	0,125768945
ENSMUSG00000015224	Cyp2j9	163,8418177	180,4651801	178,7606877	1556,450374	210,0646528	0,125725874	False	0,125725874
ENSMUSG00000028556	Dock7	315,3182152	116,1034725	209,581496	289,022938	112,2084481	-0,125624518	False	0,125624518
ENSMUSG00000049044	Rapgef4	3106,038988	1248,112329	3388,527716	1920,821705	1235,597678	0,125582633	False	0,125582633
ENSMUSG00000021969	Zdhhc20	181,6171093	497,2257409	198,1337672	504,9466894	661,507944	0,125574656	False	0,125574656
ENSMUSG00000035901	Dennd5a	438,9724173	484,6057982	402,431696	698,3783833	521,8997586	-0,125386353	False	0,125386353
ENSMUSG00000038582	Pptc7	166,160334	204,4430711	152,3428521	230,5435887	232,2453926	-0,125254211	False	0,125254211
ENSMUSG00000038072	Galnt11	162,2961402	66,88569611	148,820474	119,2079044	46,97097827	-0,12505567	False	0,12505567
ENSMUSG00000040351	Ankib1	299,0886012	224,6349794	186,6860384	326,1348328	254,4261323	0,124895608	False	0,124895608
ENSMUSG00000000127	Fer	95,05916782	102,2215356	87,17885756	97,84044983	133,0844384	-0,124847474	False	0,124847474
ENSMUSG00000027340	Slc23a2	275,9034383	273,8527558	272,1037069	253,0356461	750,230903	-0,124822798	False	0,124822798
ENSMUSG00000075706	Gpx4	2943,742848	2394,003123	3209,767029	1777,99714	2711,269246	0,124816938	False	0,124816938
ENSMUSG00000001751	Naglu	322,2737641	132,5093979	351,3572138	147,3229762	143,5224336	0,124651774	False	0,124651774
ENSMUSG00000025575	Cant1	340,0490556	542,6575344	370,7302933	715,2474263	611,927467	0,124627121	False	0,124627121
ENSMUSG00000007817	Zmiz1	665,4141748	1240,540364	813,6693373	1062,749714	725,4406645	0,124605019	False	0,124605019
ENSMUSG00000025381	Cnpy2	1037,14962	326,8565149	1130,683365	271,0292921	317,0541034	0,124570942	False	0,124570942
ENSMUSG00000025413	Ttc4	285,9503422	311,7125837	353,9989974	339,6300672	418,8245563	0,124451765	False	0,124451765
ENSMUSG00000012609	Ttll5	177,7529154	102,2215356	114,4772877	163,0674164	100,4657035	-0,124404687	False	0,124404687
ENSMUSG00000038517	Tbkbp1	196,3010458	256,184836	324,9393782	632,0268139	213,978901	0,124400691	False	0,124400691
ENSMUSG00000031665	Sall1	1068,836009	969,2115964	1165,026551	865,9442111	380,9868238	0,124322316	False	0,124322316
ENSMUSG00000026028	Trak2	231,0787901	439,1740046	251,850033	447,5919429	400,5580647	0,124180074	False	0,124180074
ENSMUSG00000014329	Bicc1	71,10116618	68,14769037	119,7608548	194,5562968	65,23746983	-0,124172393	False	0,124172393
ENSMUSG00000015189	Casd1	366,3255736	203,1810768	376,8944549	399,2340194	186,5791637	0,12410824	False	0,12410824
ENSMUSG00000030972	Acsm5	1787,576058	42,90780505	1947,87508	378,9911677	54,79947465	0,123896531	False	0,123896531
ENSMUSG00000054309	Cpsf3	470,6588065	484,6057982	325,8199727	512,8189095	407,0818117	0,123767871	False	0,123767871
ENSMUSG00000033938	Ndufb7	724,1499207	1222,872444	1768,233798	664,6402971	932,8958185	-0,123714643	False	0,123714643
ENSMUSG00000025533	Asl	6599,270196	9008,115072	7190,054263	13680,79393	8426,071603	0,123696171	False	0,123696171
ENSMUSG00000017950	Hnf4a	6808,7095	5583,062633	6249,579315	4750,32253	3010,056858	-0,123622301	False	0,123622301

ENSMUSG00000026280	Atg4b	361,688541	333,1664863	250,9694384	292,3967466	394,0343177	0,123573393	False	0,123573393
ENSMUSG00000024789	Jak2	203,2565946	123,6754381	127,6862055	150,6967848	186,5791637	-0,123514279	False	0,123514279
ENSMUSG00000010936	Vac14	381,0095101	581,7793567	422,68537	349,7514931	793,2876331	-0,123496792	False	0,123496792
ENSMUSG00000037640	Zfp60	132,1554284	117,3654668	100,3877754	143,9491676	20,87599034	0,123323759	False	0,123323759
ENSMUSG00000046822	Slc39a3	132,1554284	157,7492833	167,312959	123,7063159	121,3416939	-0,123160331	False	0,123160331
ENSMUSG00000024826	Dpf2	339,2762169	364,7163429	400,670507	311,5149954	605,40372	-0,123158689	False	0,123158689
ENSMUSG00000022124	Fbxl3	346,2317657	172,8932145	203,4173343	194,5562968	377,0725756	0,123104134	False	0,123104134
ENSMUSG00000038429	Usp5	679,3252725	648,6650528	505,461255	582,5442875	739,7929078	0,123018943	False	0,123018943
ENSMUSG00000049807	Arhgap23	183,1627868	291,5206755	168,1935535	265,4062777	173,5316697	-0,12300401	False	0,12300401
ENSMUSG00000109764	Klkb1	1759,753863	114,8414782	1916,173677	626,4037995	352,2823371	0,122854676	False	0,122854676
ENSMUSG00000039210	Gpatch2	201,7109171	219,5870023	238,6411152	353,1253017	285,7401178	0,122503493	False	0,122503493
ENSMUSG00000030095	Tmem43	132,1554284	143,8673463	178,7606877	338,5054644	257,0356311	0,122503493	False	0,122503493
ENSMUSG00000030244	Gys2	1351,694996	1241,802358	1569,219436	1650,917016	2999,618863	-0,122334075	False	0,122334075
ENSMUSG00000030682	Cdipt	481,4785492	786,2224278	564,4610879	524,0649382	934,2005679	0,122274066	False	0,122274066
ENSMUSG00000022285	Ywhaz	956,7743883	2901,324818	1385,175181	1041,382259	6695,973903	0,122249058	False	0,122249058
ENSMUSG00000000581	C1d	340,8218944	162,7972603	168,1935535	76,47299527	313,1398552	-0,122210877	False	0,122210877
ENSMUSG00000031823	Zdhhc7	252,7182754	232,206945	238,6411152	238,4158088	334,0158455	-0,122118875	False	0,122118875
ENSMUSG00000021136	Smoc1	1998,561041	3316,520931	2213,814625	2174,981954	3708,097785	0,122041794	False	0,122041794
ENSMUSG00000037730	Mynn	128,2912346	84,55361583	84,537074	69,72537804	139,6081854	0,121960928	False	0,121960928
ENSMUSG00000026197	Zfand2b	429,6983521	210,7530424	467,5956906	376,741962	106,9894505	0,121937389	False	0,121937389
ENSMUSG00000054855	Rnd1	313,7725377	58,05173624	72,20875071	37,11189476	288,3496166	-0,121899985	False	0,121899985
ENSMUSG00000025545	Clybl	567,2636519	164,0592546	521,3119563	195,6808997	138,303436	-0,121872476	False	0,121872476
ENSMUSG00000026958	Dpp7	240,3528552	238,5169163	261,5365727	220,4221628	177,4459179	0,121858762	False	0,121858762
ENSMUSG00000055900	Tmem69	264,3108569	358,4063716	273,864896	242,9142203	259,6451299	-0,121788738	False	0,121788738
ENSMUSG00000019738	Polr2i	336,9577006	421,5060849	872,6691702	385,738785	366,6345804	0,121775369	False	0,121775369
ENSMUSG00000022451	Twf1	651,503077	906,1118831	708,8785893	737,7394838	1460,014575	0,121766563	False	0,121766563
ENSMUSG00000022150	Dab2	330,7749905	540,1335459	584,7147618	771,4775699	304,0066094	-0,121747469	False	0,121747469
ENSMUSG00000042590	Ipo11	200,1652396	157,7492833	134,7309617	140,575359	183,9696649	-0,121723562	False	0,121723562
ENSMUSG00000000399	Ndufa9	1139,164336	880,8719978	1047,026885	371,1189476	776,3258909	-0,121677398	False	0,121677398
ENSMUSG00000007458	M6pr	1459,892423	773,6024851	748,5053427	410,4800481	1587,880016	0,12123984	False	0,12123984
ENSMUSG00000037343	Taf2	204,0294334	1861,441543	221,9098193	829,9569193	957,686057	0,121196215	False	0,121196215
ENSMUSG00000033237	Arid2	241,125694	589,3513223	353,9989974	766,9791584	262,2546287	0,121182846	False	0,121182846
ENSMUSG00000034235	Usp54	91,19497401	118,627461	109,1937206	205,8023255	99,16095413	0,120817837	False	0,120817837
ENSMUSG00000067656	Slc22a27	676,2339175	54,26575344	735,2964249	113,58489	142,2176842	0,120803592	False	0,120803592
ENSMUSG00000047139	Cd24a	67,23697236	61,83771904	78,37291236	105,7126699	187,8839131	-0,120767658	False	0,120767658
ENSMUSG00000027610	Gss	476,8415166	1100,459	804,8633921	518,4419238	1008,571284	0,120672536	False	0,120672536
ENSMUSG00000000154	Slc22a18	1178,579113	331,904492	1084,011855	164,1920193	377,0725756	-0,12066807	False	0,12066807
ENSMUSG00000022571	Pycrl	336,9577006	93,38757569	366,3273207	66,35156943	27,39973733	0,120565805	False	0,120565805
ENSMUSG00000017376	Nlk	120,562847	170,3692259	125,044422	154,0705934	110,9036987	-0,12047791	False	0,12047791
ENSMUSG00000040667	Nup88	470,6588065	610,8052248	433,2525043	432,9721056	1063,370758	-0,120407508	False	0,120407508
ENSMUSG00000015127	Unkl	134,4739447	94,64956996	146,1786905	65,22696655	69,15171802	0,120406347	False	0,120406347
ENSMUSG00000031078	Cttn	437,4267397	572,9453968	402,431696	981,7783069	567,5659875	-0,120297476	False	0,120297476
ENSMUSG00000026775	Yme1l1	1246,588924	654,9750241	569,744655	563,4260387	1146,87472	-0,120277995	False	0,120277995
ENSMUSG00000021140	Pcnx	375,5996387	311,7125837	230,7157645	408,2308424	379,6820744	0,120189509	False	0,120189509
ENSMUSG00000001666	Ddt	670,0512074	292,7826697	728,2516687	284,5245265	297,4828624	0,120165747	False	0,120165747
ENSMUSG00000029071	Dvl1	985,3694226	1780,67391	2640,902968	3530,128414	906,8008306	-0,119878991	False	0,119878991
ENSMUSG00000028684	Urod	683,1894663	340,7384519	742,3411811	336,2562586	216,5883998	0,119796673	False	0,119796673
ENSMUSG00000028458	Tesk1	365,5527348	927,5657856	397,1481289	728,7426608	1183,407703	0,119597661	False	0,119597661
ENSMUSG00000020471	Pold2	248,0812429	132,5093979	269,4619234	163,0674164	257,0356311	0,119268767	False	0,119268767
ENSMUSG00000015749	Anp32e	253,4911142	176,6791973	123,2832329	53,98093784	275,3021227	0,119080557	False	0,119080557
ENSMUSG00000000131	Xpo6	577,3105558	1563,610896	626,9832988	2083,889121	2712,573995	0,11907941	False	0,11907941
ENSMUSG00000034780	B3galt1	306,0441501	1263,25626	382,1780221	2235,710509	281,8258696	-0,118935745	False	0,118935745
ENSMUSG00000024835	Coro1b	459,0662251	320,5465436	203,4173343	114,7094929	422,7388045	-0,118935745	False	0,118935745
ENSMUSG00000025374	Nabp2	540,987134	715,5507489	575,0282221	498,1990721	604,0989706	-0,118871949	False	0,118871949
ENSMUSG00000021156	Zmynd11	878,7176733	906,1118831	809,2663647	924,4235605	878,0963439	-0,118785076	False	0,118785076
ENSMUSG00000079469	Pigb	197,0738845	170,3692259	213,9844686	173,1888422	76,98021439	0,118769478	False	0,118769478
ENSMUSG00000067365	Tmem128	149,1578812	363,4543486	137,3727453	145,0737704	418,8245563	-0,118744407	False	0,118744407
ENSMUSG00000048271	Rbm33	797,5696032	400,0521824	264,1783563	201,303914	734,5739102	-0,11870277	False	0,11870277
ENSMUSG00000020585	Laptm4a	2546,503723	1349,071871	1495,249496	777,1005843	2764,763971	0,118638496	False	0,118638496
ENSMUSG00000014856	Tmem208	252,7182754	338,2144633	362,8049426	232,7927944	263,5593781	-0,118483594	False	0,118483594
ENSMUSG00000030352	Tspan9	454,4291925	400,0521824	324,0587837	218,1729571	493,1952719	0,118103453	False	0,118103453
ENSMUSG00000039062	Anpep	2407,392746	575,4693854	2218,217598	656,768077	365,329831	-0,11807063	False	0,11807063
ENSMUSG00000005698	Ctcf	460,6119026	772,3404909	424,4465591	512,8189095	1200,369445	-0,117968773	False	0,117968773
ENSMUSG00000020063	Sirt1	129,8369122	167,8452374	140,8951233	164,1920193	276,6068721	0,117921083	False	0,117921083
ENSMUSG00000034371	Tkfc	4379,677269	15968,01345	9011,123732	4036,199706	10719,82104	-0,117827005	False	0,117827005
ENSMUSG00000069237	Fam8a1	1118,29769	772,3404909	931,6690031	304,7673782	1030,752023	-0,11760699	False	0,11760699
ENSMUSG00000030678	Maz	790,6140543	878,3480092	899,0870058	728,7426608	734,5739102	-0,117564152	False	0,117564152
ENSMUSG00000038975	Rabggtb	392,6020915	851,8461296	361,9243481	771,4775699	1248,645172	-0,117379692	False	0,117379692
ENSMUSG00000062683	Atp5g2	631,4092266	359,6683658	582,0729783	152,9459905	452,7480406	-0,117375395	False	0,117375395
ENSMUSG00000029346	Srrd	85,01226391	117,3654668	128,5668	92,21743547	125,2559421	0,117368565	False	0,117368565
ENSMUSG00000071178	Serpina1b	115867,8515	34916,85736	106817,8766	18946,18458	54158,8427	-0,117327224	False	0,117327224
ENSMUSG00000029726	Mepce	305,2713113	709,2407776	331,1035398	553,3046128	622,3654621	0,11719042	False	0,11719042
ENSMUSG00000020736	Nt5c	175,4343992	254,9228418	218,3874412	176,5626508	161,7889252	-0,116818804	False	0,116818804
ENSMUSG00000014301	Pam16	197,0738845	342,0004461	438,5360714	213,6745456	260,9498793	0,116678446	False	0,116678446
ENSMUSG00000043831	Lysmd4	118,2443307	93,38757569	126,805611	128,2047274	56,10422405	0,116678446	False	0,116678446
ENSMUSG00000045038	Prkce	207,1207884	291,5206755	224,5516028	353,1253017	407,0818117	0,116574658	False	0,116574658
ENSMUSG00000055932	Fto	546,3970053	348,3104175	398,9093179	426,2244883	592,356226	0,116515456	False	0,116515456
ENSMUSG00000028559	Osbpl9	1779,847671	1511,869131	1929,382595	1438,367073	1416,957845	0,116385485	False	0,116385485
ENSMUSG00000032480	Dhx30	269,7207282	373,5503028	292,3573809	455,464163	412,3008093	0,116266616	False	0,116266616
ENSMUSG00000008575	Nfib	1588,183658	1913,183307	3012,513856	2809,257973	1465,233572	-0,116247093	False	0,116247093
ENSMUSG00000042572	Ube2q1	637,5919793	790,0084106	588,2371399	589,2919047	904,1913318	-0,116235605	False	0,116235605
ENSMUSG00000034667	Xpot	902,675675	846,7981526	714,1621564	978,4044983	591,0514766	0,116223306	False	0,116223306
ENSMUSG00000002797	Ggct	126,7455571	54,26575344	137,3727453	30,36427753	62,62797103	0,116160626	False	0,116160626
ENSMUSG00000036323	Srp72	2007,835106	2280,423639	1852,770872	3572,863323	3468,023896	-0,115956315	False	0,115956315
ENSMUSG00000027498	Cstf1	315,3182152	463,1518957	341,6706741	372,2435505	482,7572767	0,115797912	False	0,115797912
ENSMUSG00000019782	Rwdd1	248,0812429	262,4948074	172,5965261	160,8182106	268,7783757	0,115604413	False	0,115604413
ENSMUSG00000019820	Utrn	388,7378977	214,5390252	246,5664658	350,8760959	358,806084	-0,115593408	False	0,115593408
ENSMUSG00000060166	Zdhhc8	141,4294936	267,5427844	153,2234466	241,7896174	251,8166335	0,115554069	False	0,115554069
ENSMUSG00000026799	Med27	103,5603942	190,5611342	142,6563124	95,59124409	340,5395925	-0,11552198	False	0,11552198
ENSMUSG00000001376	Vps50	134,4739447	268,8047787	124,1638274	345,2530816	362,7203322	-0,115081732	False	0,115081732
ENSMUSG00000036879	Phkb	344,6860882	111,0554954	258,8947891	318,2626127	43,05673008	-0,115065383	False	0,115065383
ENSMUSG00000031775	Pllp	207,8936272	155,2252947	191,9696055	182,1856652	37,8377325	-0,114967626	False	0,114967626
ENSMUSG00000040488	Ltbp4	669,2783686	184,2511629	724,7292907	382,3649764	271,3878745	0,114835817	False	0,114835817
ENSMUSG00000022799	Arhgap31	90,42213525	112,3174897	154,1040411	150,6967848	83,50396138	-0,114831346	False	0,114831346
ENSMUSG00000022217	Emc9	186,2541418	18,92991399	201,6561453	60,72855507	10,43799517	0,114624863	False	0,114624863
ENSMUSG00000024477	Pggt1b	334,6391843	329,3805035	309,0886768	356,4991103	362,7203322	-0,114585583	False	0,114585583
ENSMUSG00000031993	Snx19	377,1453162	364,7163429	373,3720768	455,464163	348,3680889	-0,114508033	False	0,114508033
ENSMUSG00000031232	Magt1	1493,897328	672,6429439	1550,726951	1617,178929	777,6306403	0,114398313	False	0,114398313
ENSMUSG00000034342	Cbl	131,3825897	119,8894553	110,0743151	89,96822973	142,2176842	0,114326762	False	0,114326762
ENSMUSG00000029787	Avl9	269,7207282	117,3654668	73,96993975	120,3325073	249,2071347	-0,114121025	False	0,114121025
ENSMUSG00000028423	Nfx1	539,4414564	647,4030585	583,8341673	1403,504384	819,382621	0,114092245	False	0,114092245
ENSMUSG00000024792	Zfpl1	203,2565946	228,4209622	231,596359	187,8086796	721,5264163	-0,114038422	False	0,114038422
ENSMUSG00000037236	Matr3	335,4120231	567,8974198	309,9692713	560,0522301	772,4116427	-0,113809202	False	0,113809202
ENSMUSG00000027227	Sord	16567,34456	1360,429819	17927,14326	1465,357542	2674,736263	0,113803225	False	0,113803225
ENSMUSG00000075376	Rc3h2	237,2615002	459,3659129	219,2680357	327,2594356	651,0699489	-0,113782502	False	0,113782502
ENSMUSG00000025857	Dnaaf5	114,3801369	180,4651801	145,2980959	105,7126699	375,7678262	-0,11368824	False	0,11368824
ENSMUSG00000054237	Fra10ac1	183,1627868	198,1330998	193,7307946	182,1856652	101,7704529	0,113343494	False	0,113343494
ENSMUSG00000073676	Hspe1	1551,087397	643,6170757	1409,831828	334,0070529	1433,919587	-0,113315857	False	0,113315857
ENSMUSG00000022801	Lrch3	167,7060115	235,9929278	181,4024713	219,29756	268,7783757	0,113259706	False	0,113259706
ENSMUSG00000028096	Gpr89	352,4144758	287,7346927	325,8199727	223,7959715	277,9116215	-0,113198142	False	0,113198142
ENSMUSG00000039473	Ubn1	411,1502218	532,5615803	444,700233	1043,631465	627,5844597	0,113167553	False	0,113167553
ENSMUSG00000020228	Helb	197,8467233	218,325008	213,9844686	332,88245	454,05279	0,113122915	False	0,113122915
ENSMUSG00000019297	Nop9	171,5702053	247,3508762	247,4470604	185,5594738	365,329831	0,113082622	False	0,113082622

ENSMUSG00000056167	Cnot10	307,5898276	291,5206755	284,4320302	440,8443257	290,9591154	-0,112923854	False	0,112923854
ENSMUSG00000094806	Cyp2d10	11820,56888	6364,237084	12781,82947	3823,649764	7665,402704	0,112794876	False	0,112794876
ENSMUSG00000037608	Bclaf1	677,0067562	1749,124053	626,1027043	1321,408374	4284,797018	-0,112770899	False	0,112770899
ENSMUSG00000024083	Pja2	556,4439092	311,7125837	227,1933864	601,6625363	313,1398552	0,112718259	False	0,112718259
ENSMUSG00000043190	Rfesd	119,0171695	63,09971331	110,0743151	89,96822973	13,04749397	-0,112691845	False	0,112691845
ENSMUSG00000040842	Szrd1	981,5052288	619,6391847	1061,116398	215,9237514	695,4314283	0,112515061	False	0,112515061
ENSMUSG00000060586	H2-Eb1	107,424588	35,33583945	95,10420825	39,36110051	116,1226963	0,112325731	False	0,112325731
ENSMUSG00000002064	Sdf2	253,4911142	219,5870023	239,5217097	143,9491676	273,9973733	0,112226886	False	0,112226886
ENSMUSG00000057101	Zfp180	135,2467835	176,6791973	146,1786905	304,7673782	221,8073974	0,112138731	False	0,112138731
ENSMUSG00000059182	Skap2	230,3059513	335,6904748	213,103874	215,9237514	258,3403805	-0,111994872	False	0,111994872
ENSMUSG00000010392	Gosr1	466,021774	297,8306468	308,2080823	179,9364595	503,6332671	0,111976219	False	0,111976219
ENSMUSG00000029761	Cald1	4069,768925	3740,551005	3285,498157	3766,295017	1527,861543	-0,111800871	False	0,111800871
ENSMUSG00000023018	Smarcd1	156,1134301	181,7271743	281,7902467	168,6904307	410,9960599	0,111783482	False	0,111783482
ENSMUSG00000036568	Bicral	282,0861484	88,33959863	292,3573809	304,7673782	99,16095413	0,111572662	False	0,111572662
ENSMUSG00000060012	Kif13b	660,0043034	535,0855688	643,7145947	712,9982206	219,1978986	0,111423045	False	0,111423045
ENSMUSG00000027342	Pcna	240,3528552	145,1293406	130,3279891	68,60077517	259,6451299	0,111387224	False	0,111387224
ENSMUSG00000030770	Parva	1134,527304	966,6876079	907,0123565	951,4140294	1050,323264	-0,111257907	False	0,111257907
ENSMUSG00000040820	Hlcs	197,8467233	76,98165023	183,1636603	138,3261532	75,675465	-0,111249871	False	0,111249871
ENSMUSG00000033416	Gucd1	2596,738243	1207,728513	1470,59285	2804,759562	1021,618777	0,111176507	False	0,111176507
ENSMUSG00000000278	Scpep1	762,7918589	633,5211216	706,2368057	537,5601726	691,5171802	-0,111137437	False	0,111137437
ENSMUSG00000073616	Cops9	297,5429237	493,4397581	501,0582824	275,5277035	463,1860358	-0,110900422	False	0,110900422
ENSMUSG00000009090	Ap1b1	571,9006845	533,8235746	611,1325975	529,6879525	738,4881584	-0,110621937	False	0,110621937
ENSMUSG00000028580	Pum1	587,3574597	368,5023257	376,8944549	398,1094166	544,0804983	-0,110418662	False	0,110418662
ENSMUSG00000045867	Cradd	321,5009253	297,8306468	221,0292247	131,578536	193,1029107	-0,110330675	False	0,110330675
ENSMUSG00000030536	Iqgap1	176,2072379	291,5206755	190,2084165	504,9466894	515,3760116	0,110307899	False	0,110307899
ENSMUSG00000013833	Med16	223,3504025	480,8198154	206,9397124	278,9015122	542,7757489	-0,110098325	False	0,110098325
ENSMUSG00000002949	Timm44	527,0760362	487,1297867	378,655644	409,3554453	568,8707369	0,110089769	False	0,110089769
ENSMUSG00000038167	Plekhg6	160,7504627	161,5352661	173,4771206	170,9396365	45,66622888	0,109922513	False	0,109922513
ENSMUSG00000030314	Atg7	442,0637723	477,0338326	460,5509344	287,8983351	366,6345804	0,109837081	False	0,109837081
ENSMUSG00000035237	Lcat	8774,811313	14515,45805	14597,61537	13974,31528	9468,56637	0,109777902	False	0,109777902
ENSMUSG00000027551	Zfp64	113,6072981	244,8268876	124,1638274	122,581713	386,2058214	0,109688254	False	0,109688254
ENSMUSG00000024966	Stip1	1908,138905	689,0488693	2058,82999	488,0776463	647,1557007	0,109658505	False	0,109658505
ENSMUSG00000031660	Brd7	433,5625459	354,6203888	273,864896	395,8602108	401,8628141	-0,109537047	False	0,109537047
ENSMUSG00000036819	Jmjd4	130,6097509	109,7935012	140,8951233	49,48252635	44,36147948	0,10935907	False	0,10935907
ENSMUSG00000024991	Eif3a	2046,477044	1461,38936	1327,936537	975,0306897	1897,105623	-0,10934248	False	0,10934248
ENSMUSG00000049550	Clip1	377,1453162	682,738898	406,8346686	699,5029861	597,5752236	0,109322115	False	0,109322115
ENSMUSG00000009621	Vav2	398,0119628	306,6646067	368,9691042	224,9205743	156,5699276	-0,109311777	False	0,109311777
ENSMUSG00000022219	Cideb	3015,616852	1284,710163	3252,91616	873,8164312	902,8865824	0,1092805	False	0,1092805
ENSMUSG00000031154	Otud5	550,2611991	1057,551195	593,5207071	1006,51957	1497,852307	0,109181762	False	0,109181762
ENSMUSG00000034757	Tmub2	333,8663455	243,5648934	283,5514357	244,0388231	360,1108334	0,109170328	False	0,109170328
ENSMUSG00000030643	Rab30	144,5208486	2542,918446	155,8652302	832,206125	14446,18532	0,109021502	False	0,109021502
ENSMUSG00000074746	Pdzd8	408,0588668	305,4026124	154,9846357	168,6904307	378,377325	-0,108951656	False	0,108951656
ENSMUSG00000050608	Minos1	629,8635917	1385,669704	1213,45925	679,2601345	1837,08715	0,108924765	False	0,108924765
ENSMUSG00000000804	Usp32	367,0984123	246,0888819	200,7755508	395,8602108	281,8258696	0,10882419	False	0,10882419
ENSMUSG00000028937	Acot7	211,757821	47,95578211	196,3725782	76,47299527	71,76121681	-0,108821772	False	0,108821772
ENSMUSG00000018167	Stard3	219,4862086	254,9228418	228,0739809	298,019761	203,5409059	-0,108811527	False	0,108811527
ENSMUSG00000038717	Atp5l	1056,470589	821,5582673	907,892951	383,4895792	979,8667968	-0,108595057	False	0,108595057
ENSMUSG00000021975	Ints9	156,1134301	141,3433578	114,4772877	139,4507561	168,3126721	0,108549146	False	0,108549146
ENSMUSG00000046201	Scaf8	220,2590474	325,5945207	204,2979288	353,1253017	673,2506886	-0,108526703	False	0,108526703
ENSMUSG00000023939	Mrpl14	646,0932057	80,76763303	696,550266	110,2110814	113,5131975	0,108485164	False	0,108485164
ENSMUSG00000044573	Acp1	925,8608378	989,4035047	709,7591838	400,3586223	998,1332883	0,108437118	False	0,108437118
ENSMUSG00000003423	Pih1d1	367,0984123	498,4877351	398,9093179	521,8157324	340,5395925	-0,108344333	False	0,108344333
ENSMUSG00000033596	Rfwd3	187,7998194	68,14769037	91,58183017	202,4285169	139,6081854	0,108216867	False	0,108216867
ENSMUSG00000025451	Paip1	561,8537806	1335,189934	521,3119563	759,1069383	1496,547558	-0,108047777	False	0,108047777
ENSMUSG00000031812	Map1lc3b	1053,379234	2145,390252	977,4599182	1172,960795	3778,554252	-0,107915474	False	0,107915474
ENSMUSG00000022009	Nufip1	99,6962004	252,3988532	107,4325315	192,307091	557,1279923	0,107820493	False	0,107820493
ENSMUSG00000004099	Dnmt1	142,2023324	511,1076778	153,2234466	323,885627	709,7836717	0,107691951	False	0,107691951
ENSMUSG00000061436	Hipk2	255,8096305	265,0187959	275,626085	185,5594738	169,6174215	0,107641851	False	0,107641851
ENSMUSG00000024914	Drap1	950,5916782	1336,451928	1024,131428	1069,497331	1085,551498	0,107503193	False	0,107503193
ENSMUSG00000028899	Taf12	198,619562	201,9190826	213,9844686	140,575359	199,6266577	0,107498366	False	0,107498366
ENSMUSG00000031490	Eif4ebp1	653,0487546	1402,07563	860,3408469	606,1609478	2974,828624	-0,107489794	False	0,107489794
ENSMUSG00000073888	Ccl27a	241,8985328	42,90780505	224,5516028	56,23014358	19,57124095	-0,107354999	False	0,107354999
ENSMUSG00000018677	Slc25a39	5093,780286	2410,409048	4728,792577	2077,141504	2321,149176	-0,107264871	False	0,107264871
ENSMUSG00000056536	Pign	187,7998194	686,5248808	174,3577151	416,1030625	477,5382791	-0,107145472	False	0,107145472
ENSMUSG00000040596	Pogk	108,9702656	117,3654668	65,16399454	75,3483924	91,33245776	0,107073453	False	0,107073453
ENSMUSG00000028567	Txndc12	480,7057105	403,8381652	446,4614221	259,7832633	335,3205949	-0,106618422	False	0,106618422
ENSMUSG00000029708	Gcc1	146,0665262	94,64956996	117,1190713	55,10554071	135,6939372	-0,106269333	False	0,106269333
ENSMUSG00000040446	Rprd1a	146,0665262	79,50563877	53,71626577	39,36110051	135,6939372	-0,106269333	False	0,106269333
ENSMUSG00000030056	Isy1	136,792461	129,9854094	133,8503672	127,0801245	219,1978986	-0,106250313	False	0,106250313
ENSMUSG00000044167	Foxo1	448,2464824	1422,267538	416,5212084	1300,04092	1800,554167	-0,105902305	False	0,105902305
ENSMUSG00000028439	Fam219a	156,1134301	155,2252947	195,4919836	145,0737704	147,4366818	-0,105807953	False	0,105807953
ENSMUSG00000021546	Hnrnpk	2146,173244	1144,628799	1386,055776	975,0306897	2309,406432	0,105755556	False	0,105755556
ENSMUSG00000005873	Reep5	201,7109171	319,2845493	363,6855371	217,0483542	441,005296	0,105727315	False	0,105727315
ENSMUSG00000056820	Tsnax	306,0441501	176,6791973	190,2084165	188,9332824	284,4353684	-0,105638922	False	0,105638922
ENSMUSG00000018736	Ndel1	364,779896	373,5503028	339,0288905	374,4927562	708,4789223	-0,105618002	False	0,105618002
ENSMUSG00000046792	Zfp787	226,4417575	294,044664	210,4620905	257,5340576	251,8166335	-0,105579635	False	0,105579635
ENSMUSG00000047264	Zfp358	174,6615604	156,487289	90,70123565	106,8372728	187,8839131	0,105279412	False	0,105279412
ENSMUSG00000018574	Acadvl	3612,248377	3431,36241	3436,960415	3150,012643	3885,543703	0,105219393	False	0,105219393
ENSMUSG00000023235	Ccl25	71,10116618	136,2953807	137,3727453	76,47299527	76,98021439	0,10507716	False	0,10507716
ENSMUSG00000029507	Pus1	104,333233	186,7751514	117,9996658	142,8245647	112,2084481	0,104982531	False	0,104982531
ENSMUSG00000038290	Smg6	352,4144758	271,3287672	270,3425179	378,9911677	266,1688769	0,104891041	False	0,104891041
ENSMUSG00000024976	Shoc2	468,3402903	377,3362856	180,5218768	134,9523446	503,6332671	0,104816427	False	0,104816427
ENSMUSG00000043962	Thrap3	840,8485739	1211,514495	781,9679345	1138,098106	2381,167649	-0,104736564	False	0,104736564
ENSMUSG00000020263	Appl2	370,1897674	1157,248742	398,0287234	2529,231858	982,4762956	0,104607528	False	0,104607528
ENSMUSG00000015165	Hnrnpl	1006,236069	2033,072763	1108,668502	1081,867962	2373,339152	0,104555625	False	0,104555625
ENSMUSG00000005683	Cs	2504,77043	1288,496146	1486,443551	628,6530052	2693,002754	0,104537323	False	0,104537323
ENSMUSG00000059588	Calcrl	159,9776239	51,74176491	148,820474	82,09600963	56,10422405	-0,10429711	False	0,10429711
ENSMUSG00000036908	Unc93b1	546,3970053	353,3583945	491,3717426	508,320498	249,2071347	-0,104211168	False	0,104211168
ENSMUSG00000027598	Itch	559,5352643	275,11475	289,7155974	375,6173591	520,5950092	-0,104067578	False	0,104067578
ENSMUSG00000027472	Pdrg1	136,0196223	93,38757569	146,1786905	89,96822973	87,41820957	0,103918224	False	0,103918224
ENSMUSG00000034032	Rpap1	180,8442705	194,347117	229,8351699	336,2562586	217,8931492	0,103887815	False	0,103887815
ENSMUSG00000045962	Wnk1	953,6830333	870,7760436	703,5950222	1024,513216	1004,657035	0,103356839	False	0,103356839
ENSMUSG00000031792	Usb1	129,8369122	201,9190826	145,2980959	139,4507561	417,5198069	0,103055162	False	0,103055162
ENSMUSG00000029231	Pdgfra	67,23697236	118,627461	72,20875071	134,9523446	116,1226963	0,10291892	False	0,10291892
ENSMUSG00000034175	Rhbdd3	174,6615604	177,9411915	187,5666329	128,2047274	126,5606915	0,102841069	False	0,102841069
ENSMUSG00000036528	Ppfibp2	513,1649385	203,1810768	476,4016358	551,0554071	254,4261323	0,102774782	False	0,102774782
ENSMUSG00000041671	Pyroxd1	147,6122037	328,1185092	158,5070138	277,7769093	504,9380164	0,10273468	False	0,10273468
ENSMUSG00000022462	Slc38a2	1123,707561	5148,936606	1206,414494	3956,352902	10638,92658	0,102459035	False	0,102459035
ENSMUSG00000020740	Gga3	179,298593	175,417203	340,7900796	502,6974836	167,0079228	-0,102447622	False	0,102447622
ENSMUSG00000096727	Psmb9	211,757821	21,45390252	197,2531727	13,49523446	49,58047707	-0,10236675	False	0,10236675
ENSMUSG00000024773	Atg2a	643,7746894	2468,460785	599,6848687	3406,422098	4012,104394	-0,102351286	False	0,102351286
ENSMUSG00000038910	Plcl2	275,9034383	335,6904748	349,5960248	854,6981824	257,0356311	-0,102195071	False	0,102195071
ENSMUSG00000028958	Tmub1	156,8862688	80,76763303	146,1786905	107,9618757	31,31398552	-0,101986074	False	0,101986074
ENSMUSG00000021900	Btd	1425,114679	1804,651801	1327,936537	1451,862307	2472,500106	-0,101891816	False	0,101891816
ENSMUSG00000042660	Wdr55	123,654202	243,5648934	223,6710083	132,7031389	142,2176842	0,101891229	False	0,101891229
ENSMUSG00000030983	Bccip	651,503077	699,1448234	561,8193043	350,8760959	536,252002	0,101819339	False	0,101819339
ENSMUSG00000003099	Ppp5c	596,6315249	474,5098441	640,1922167	540,9339812	424,0435539	0,101664928	False	0,101664928
ENSMUSG00000050213	Snip1	92,74065154	141,3433578	99,50718086	136,0769475	213,978901	0,101598778	False	0,101598778
ENSMUSG00000038658	Ric1	374,0539612	200,6570883	287,0738138	348,6268902	324,8825997	-0,101562558	False	0,101562558
ENSMUSG00000024947	Men1	369,4169286	179,2031858	344,3124577	231,6681916	83,50396138	-0,101531599	False	0,101531599
ENSMUSG00000030801	Kat8	214,0763373	253,6608475	258,8947891	381,2403735	229,6358938	0,101222826	False	0,101222826
ENSMUSG00000022215	Fitm1	136,0196223	32,81185092	126,805611	21,36745456	63,93272043	-0,101196206	False	0,101196206
ENSMUSG00000020392	Cdkn2aipnl	161,5233014	306,6646067	150,5816631	173,1888422	212,6741516	-0,101196206	False	0,101196206

ENSMUSG00000015335	Zdhhc12	205,5751109	420,2440906	386,5809947	221,5467657	220,502648	0,101130374	False	0,101130374
ENSMUSG00000022508	Bcl6	105,1060717	172,8932145	112,7160987	238,4158088	653,6794477	0,10084757	False	0,10084757
ENSMUSG00000010045	Tmem115	299,0886012	403,8381652	327,5811618	278,9015122	507,5475152	-0,100817171	False	0,100817171
ENSMUSG00000026615	Eprs	1483,850425	1673,404397	1591,234299	2421,269983	3025,713851	0,100800607	False	0,100800607
ENSMUSG00000048537	Phldb1	57,19006845	85,8156101	154,1040411	428,4736941	61,32322164	0,100668859	False	0,100668859
ENSMUSG00000020669	Sh3yl1	200,1652396	103,4835298	186,6860384	43,85951199	52,18997586	-0,100577422	False	0,100577422
ENSMUSG00000035969	Rusc2	392,6020915	1019,691367	420,924181	1047,005273	1163,836462	0,100492533	False	0,100492533
ENSMUSG00000055720	Ubl7	581,1747496	737,0046514	757,3112879	623,0299909	812,858874	0,100329589	False	0,100329589
ENSMUSG00000037822	Smim14	835,4387026	591,8753108	895,5646277	385,738785	584,5277296	0,100263567	False	0,100263567
ENSMUSG00000040520	Manea	316,091054	210,7530424	140,8951233	61,85315794	294,8733636	-0,100244697	False	0,100244697
ENSMUSG00000020680	Taf15	494,6168082	931,3517684	461,4315289	674,761723	953,7718088	-0,100194682	False	0,100194682
ENSMUSG00000025898	Cwf19l2	230,3059513	358,4063716	214,8650631	728,7426608	456,6622888	-0,100120772	False	0,100120772
ENSMUSG00000020495	Smg8	117,4714919	53,00375918	45,79091508	29,23967466	109,5989493	-0,100076717	False	0,100076717
ENSMUSG00000024962	Vegfb	418,1057707	156,487289	390,1033727	325,0102299	168,3126721	-0,100011484	False	0,100011484
ENSMUSG00000001472	Tcf25	2347,111323	3938,684105	2190,038573	3833,771189	3607,632081	-0,09992999	False	0,09992999
ENSMUSG00000054874	Pcnx3	438,1995785	408,8861422	488,7299591	457,7133687	421,4340551	-0,099888928	False	0,099888928
ENSMUSG00000032204	Aqp9	4434,548821	446,7459702	4137,913654	1375,389312	405,7770623	-0,099883789	False	0,099883789
ENSMUSG00000041143	Tmco4	142,9751711	223,3729851	153,2234466	182,1856652	249,2071347	0,099872447	False	0,099872447
ENSMUSG00000030630	Fah	8925,514872	3673,665309	8328,662978	3536,876031	3496,728383	-0,099850479	False	0,099850479
ENSMUSG00000030007	Cct7	994,6434877	637,3071044	928,146625	355,3745074	760,6688982	-0,099826775	False	0,099826775
ENSMUSG00000028890	Mtf1	199,3924008	353,3583945	228,0739809	213,6745456	388,8153202	0,099804627	False	0,099804627
ENSMUSG00000033530	Ttc7b	183,1627868	220,8489966	176,9994987	170,9396365	190,4934119	-0,099639461	False	0,099639461
ENSMUSG00000010095	Slc3a2	407,286028	972,9975792	387,4615892	380,1157706	1535,69004	-0,099603441	False	0,099603441
ENSMUSG00000036550	Cnot1	1150,756918	657,4990127	487,8493646	987,4013213	1232,98818	0,099575854	False	0,099575854
ENSMUSG00000022971	Ifnar2	521,6661649	246,0888819	346,9542412	486,9530434	267,4736263	-0,099344203	False	0,099344203
ENSMUSG00000011114	Tbrg1	786,7498605	2527,774515	734,4158304	1286,545685	6200,169132	-0,099307861	False	0,099307861
ENSMUSG00000056228	Cars2	217,9405311	233,4689392	357,5213755	1067,248125	1125,998729	0,099296102	False	0,099296102
ENSMUSG00000041957	Pkp2	693,2363702	1061,337178	678,0577811	1745,383657	647,1557007	-0,099234491	False	0,099234491
ENSMUSG00000078970	Wdr92	111,2887818	90,86358716	70,44756167	119,2079044	31,31398552	0,099171727	False	0,099171727
ENSMUSG00000014349	Ube2z	670,8240461	480,8198154	276,5066795	260,9078662	626,2797103	-0,099127264	False	0,099127264
ENSMUSG00000027255	Arfgap2	1482,304747	836,7021984	1587,711921	1190,954441	862,4393511	0,099107088	False	0,099107088
ENSMUSG00000002963	Pnkp	386,4193814	212,0150367	280,9096522	413,8538568	121,3416939	0,098953946	False	0,098953946
ENSMUSG00000004530	Coro1c	205,5751109	223,3729851	220,1486302	287,8983351	240,073889	0,098812261	False	0,098812261
ENSMUSG00000001763	Tspan33	149,93072	100,9595413	140,0145288	137,2015503	130,4749397	-0,098719476	False	0,098719476
ENSMUSG00000017830	Dhx58	218,7133699	39,12182225	204,2979288	82,09600963	44,36147948	-0,098366836	False	0,098366836
ENSMUSG00000024580	Grpel2	643,7746894	689,0488693	440,2972604	614,0331679	638,0224549	0,09805045	False	0,09805045
ENSMUSG00000040479	Dgkz	559,5352643	950,2816824	708,8785893	1038,008451	598,879973	0,09803783	False	0,09803783
ENSMUSG00000020448	Rnf185	458,2933863	1039,883275	490,4911481	834,4553307	1613,975003	0,097955634	False	0,097955634
ENSMUSG00000017288	Vps53	395,6934466	369,76432	291,4767864	244,0388231	263,5593781	-0,097777152	False	0,097777152
ENSMUSG00000079003	Samd1	109,7431043	395,0042053	349,5960248	425,0998855	117,4274457	0,09763935	False	0,09763935
ENSMUSG00000032340	Neo1	216,3948536	117,3654668	105,6713425	121,4571101	202,2361565	-0,097625238	False	0,097625238
ENSMUSG00000032440	Tgfbr2	167,7060115	268,8047787	156,7458247	161,9428135	602,7942212	-0,09750739	False	0,09750739
ENSMUSG00000023224	Serping1	14713,30437	7407,906342	13752,24463	6744,243421	9156,731265	-0,097454175	False	0,097454175
ENSMUSG00000092416	Zfp141	105,8789105	69,40968464	87,17885756	98,9650527	45,66622888	-0,09742419	False	0,09742419
ENSMUSG00000013698	Pea15a	318,4095703	182,9891686	297,640948	230,5435887	71,76121681	-0,09731068	False	0,09731068
ENSMUSG00000037339	Fam53a	265,0836956	458,1039186	283,5514357	309,2657897	422,7388045	0,097162523	False	0,097162523
ENSMUSG00000087382	Ctcflos	75,73819875	1229,182415	81,01469592	900,8069002	658,8984452	0,097162523	False	0,097162523
ENSMUSG00000022023	Wbp4	241,125694	403,8381652	225,4321973	334,0070529	379,6820744	-0,097091808	False	0,097091808
ENSMUSG00000079020	Slc45a4	124,4270408	78,2436445	62,52221098	49,48252635	133,0844384	0,097041837	False	0,097041837
ENSMUSG00000025487	Psmd13	512,3920997	378,5982798	313,4916494	237,2912059	547,9947465	0,096913831	False	0,096913831
ENSMUSG00000055817	Mta3	214,8491761	160,2732718	135,6115562	84,34521537	200,9314071	-0,096621159	False	0,096621159
ENSMUSG00000026932	Nacc2	376,3724775	198,1330998	402,431696	178,8118566	31,31398552	0,096582804	False	0,096582804
ENSMUSG00000022533	Atp13a3	1388,018418	667,5949668	767,8784222	576,9212731	1298,22565	-0,096485546	False	0,096485546
ENSMUSG00000042380	Smim12	107,424588	114,8414782	98,62658634	87,71902399	91,33245776	0,096319561	False	0,096319561
ENSMUSG00000002052	Supt6	724,9227595	1291,020134	774,9231784	1640,79559	2480,328603	0,096226012	False	0,096226012
ENSMUSG00000004846	Plod3	299,86144	454,3179358	388,3421837	335,1316557	280,5211202	-0,096186619	False	0,096186619
ENSMUSG00000041895	Wipi1	241,8985328	363,4543486	226,3127919	516,1927181	905,4960812	-0,096083886	False	0,096083886
ENSMUSG00000039108	Lsm14b	232,6244676	248,6128704	188,4472275	159,6936078	219,1978986	0,095898137	False	0,095898137
ENSMUSG00000028251	Tstd3	371,7354449	171,6312202	347,8348357	124,8309187	82,19921198	-0,095873829	False	0,095873829
ENSMUSG00000034243	Golgb1	1095,112527	1418,481555	1507,57782	7344,781355	1170,360209	0,095873505	False	0,095873505
ENSMUSG00000023156	Rpp14	112,0616206	179,2031858	119,7608548	152,9459905	159,1794264	0,095864163	False	0,095864163
ENSMUSG00000000142	Axin2	112,0616206	37,85982798	119,7608548	80,97140676	35,22823371	0,095864163	False	0,095864163
ENSMUSG00000001999	Blvra	138,3381385	176,6791973	129,4473946	137,2015503	148,7414312	-0,095833021	False	0,095833021
ENSMUSG00000024290	Rock1	472,9773228	705,4547948	505,461255	930,0465748	805,0303776	0,095829495	False	0,095829495
ENSMUSG00000038518	Jarid2	145,2936874	152,7013062	134,7309617	138,3261532	155,2651782	0,095762285	False	0,095762285
ENSMUSG00000043587	Pxylp1	136,792461	26,50187959	146,1786905	42,73490912	30,00923612	0,095744293	False	0,095744293
ENSMUSG00000027893	Ahcyl1	1336,238221	615,8532019	1250,44422	1108,858431	643,2414525	-0,095736528	False	0,095736528
ENSMUSG00000031399	Fam3a	224,89608	104,7455241	210,4620905	102,3388613	30,00923612	-0,095698124	False	0,095698124
ENSMUSG00000024799	Tm7sf2	2148,491761	957,853648	2295,709916	299,1443639	1474,366818	0,095616111	False	0,095616111
ENSMUSG00000004994	Ccdc130	61,05426226	148,9153234	156,7458247	203,5531198	65,23746983	0,095608815	False	0,095608815
ENSMUSG00000022175	Lrp10	627,5450754	1231,706404	587,3565454	687,1323546	973,3430498	-0,095482555	False	0,095482555
ENSMUSG00000027170	Eif3m	570,3550069	405,1001594	270,3425179	247,4126318	609,3179682	0,095335107	False	0,095335107
ENSMUSG00000028476	Reck	156,1134301	46,69378785	48,43269865	114,7094929	146,1319324	-0,095323188	False	0,095323188
ENSMUSG00000033671	Cep350	340,0490556	278,9007328	271,2231124	363,2467275	246,5976359	0,095206916	False	0,095206916
ENSMUSG00000026974	Zmynd19	86,55794143	185,5131571	92,46242469	110,2110814	489,2810237	0,095201007	False	0,095201007
ENSMUSG00000041921	Metap1d	687,0536601	1105,506977	1911,770705	2807,008768	643,2414525	-0,095062399	False	0,095062399
ENSMUSG00000010914	Pdhx	294,4515686	189,2991399	113,5966932	163,0674164	314,4446046	0,094775522	False	0,094775522
ENSMUSG00000046985	Tapt1	841,6214127	1985,116981	788,1320962	3319,827677	2082,380037	-0,094733955	False	0,094733955
ENSMUSG00000014769	Psmb1	1639,191016	1750,386047	1664,323644	940,1680007	1730,0977	0,094689161	False	0,094689161
ENSMUSG00000001380	Hars	416,5600932	570,4214083	390,1033727	392,4864022	707,1741729	-0,094668163	False	0,094668163
ENSMUSG00000039236	Isg20	80,37523133	85,8156101	128,5668	133,8277417	152,6556794	0,094489117	False	0,094489117
ENSMUSG00000021339	Mrs2	281,3133097	300,3546353	337,2677015	461,0871774	409,6913105	0,094489117	False	0,094489117
ENSMUSG00000038055	Dexi	294,4515686	107,2695126	314,3722439	169,8150336	32,61873491	0,094443488	False	0,094443488
ENSMUSG00000033157	Abhd10	67,23697236	126,1994266	106,551937	62,97776081	208,7599034	-0,094412299	False	0,094412299
ENSMUSG00000042323	Pbrm1	472,9773228	758,4585539	537,1626577	961,5354552	504,9380164	0,094335287	False	0,094335287
ENSMUSG00000028599	Tnfrsf1b	257,355308	776,1264737	274,7454905	355,3745074	838,953862	0,094334263	False	0,094334263
ENSMUSG00000066153	Mup21	119,7900082	39,12182225	61,64161646	10,12142584	112,2084481	-0,094326277	False	0,094326277
ENSMUSG00000033060	Lmo7	286,723181	185,5131571	268,5813289	157,444402	80,89446258	-0,094299533	False	0,094299533
ENSMUSG00000026222	Sp100	211,757821	146,3913349	160,2682028	226,0451772	121,3416939	0,094195883	False	0,094195883
ENSMUSG00000024077	Stm	172,3430441	159,0112775	129,4473946	92,21743547	183,9696649	0,094184826	False	0,094184826
ENSMUSG00000037072	Selenof	2103,667012	1499,249188	2245,516028	833,3307279	1933,638606	0,094140592	False	0,094140592
ENSMUSG00000069565	Dazap1	680,87095	757,1965597	1097,220773	742,2378953	726,7454139	0,094068682	False	0,094068682
ENSMUSG00000056050	Mia3	3606,838506	1903,087353	2131,919335	3105,028529	3379,300937	-0,094009996	False	0,094009996
ENSMUSG00000024561	Mbd1	341,5947332	1390,717681	364,5661316	962,6600581	1199,064695	0,093894809	False	0,093894809
ENSMUSG00000024603	Dctn4	394,9206078	393,742211	260,6559782	326,1348328	421,4340551	0,093744247	False	0,093744247
ENSMUSG00000028648	Ndufs5	377,1453162	229,6829564	402,431696	98,9650527	186,5791637	0,093623429	False	0,093623429
ENSMUSG00000027602	Map1lc3a	357,0515084	374,812297	334,6259179	429,598297	417,5198069	-0,093583022	False	0,093583022
ENSMUSG00000030688	Stard10	12309,00298	4405,621983	11536,66882	6068,357095	4366,99623	-0,093487199	False	0,093487199
ENSMUSG00000030435	U2af2	666,1870135	750,8865884	855,0572798	710,7490149	1037,27577	0,093412963	False	0,093412963
ENSMUSG00000052406	Rexo4	228,7602738	176,6791973	190,2084165	244,0388231	118,7321951	0,093274141	False	0,093274141
ENSMUSG00000052456	Asna1	422,7428033	673,9049381	673,6548085	450,9657515	835,0396138	0,093237678	False	0,093237678
ENSMUSG00000022546	Gpt	2777,582514	338,2144633	2603,917998	332,88245	448,8337924	-0,093145754	False	0,093145754
ENSMUSG00000039176	Polg	423,515642	737,0046514	451,7449892	946,9156179	715,0026693	0,093093344	False	0,093093344
ENSMUSG00000063015	Ccni	1993,924008	1708,740236	1102,50434	1549,702757	2126,741516	0,093034272	False	0,093034272
ENSMUSG00000029145	Eif2b4	435,1082235	777,3884679	464,0733125	607,2855507	692,8219295	0,092978451	False	0,092978451
ENSMUSG00000031378	Abcd1	540,987134	171,6312202	507,222444	382,3649764	76,98021439	-0,092975699	False	0,092975699
ENSMUSG00000035007	Rundc1	135,2467835	232,206945	126,805611	247,4126318	236,1596408	-0,092975699	False	0,092975699
ENSMUSG00000004446	Bid	149,93072	82,0296273	140,0145288	140,575359	32,61873491	-0,092952283	False	0,092952283
ENSMUSG00000024687	Osbp	988,4607776	565,3734312	653,4011345	659,0172828	1054,237512	0,092944307	False	0,092944307
ENSMUSG00000029125	Stx18	283,6318259	397,5281938	265,9395453	423,9752826	861,1346017	-0,092921102	False	0,092921102
ENSMUSG00000071074	Yipf3	891,8559323	706,716789	709,7591838	521,8157324	951,1623101	0,092880869	False	0,092880869
ENSMUSG00000036959	Bcorl1	112,8344594	64,36170757	76,61172331	120,3325073	40,44723129	0,092818702	False	0,092818702
ENSMUSG00000040738	Ints8	260,4466631	277,6387386	176,9994987	201,303914	254,4261323	0,092220926	False	0,092220926

ENSMUSG00000014767	Tbp	205,5751109	469,461867	192,8502001	780,4743929	880,7058426	-0,092184965	False	0,092184965
ENSMUSG00000026842	Abl1	379,4638325	725,646703	482,5657974	793,9696274	404,4723129	0,092078564	False	0,092078564
ENSMUSG00000039156	Stim2	328,4564742	300,3546353	308,2080823	295,7705552	268,7783757	-0,091797508	False	0,091797508
ENSMUSG00000026295	Spp2	748,1079224	551,4914943	796,9380414	281,1507179	739,7929078	0,091221156	False	0,091221156
ENSMUSG00000053870	Fpgt	179,298593	131,2474037	98,62658634	137,2015503	168,3126721	-0,091220367	False	0,091220367
ENSMUSG00000041685	Fcho2	510,0735834	278,9007328	471,9986632	356,4991103	478,8430285	-0,091152588	False	0,091152588
ENSMUSG00000059534	Uqcr10	958,3200659	1470,22332	1758,547258	899,6822973	1195,150447	-0,091091941	False	0,091091941
ENSMUSG00000002767	Mrpl2	379,4638325	326,8565149	404,1928851	194,5562968	304,0066094	0,09108154	False	0,09108154
ENSMUSG00000037902	Sirpa	219,4862086	142,6053521	206,0591179	204,6777226	75,675465	-0,091071989	False	0,091071989
ENSMUSG00000053929	Cyhr1	851,6683166	888,4439634	930,7884085	799,5926417	945,9433125	-0,091026485	False	0,091026485
ENSMUSG00000024831	Ighmbp2	71,10116618	157,7492833	75,73112879	227,1697801	216,5883998	0,09101321	False	0,09101321
ENSMUSG00000035674	Ndufa3	1536,40346	2714,549666	3403,497823	1636,297178	2460,757362	0,090877671	False	0,090877671
ENSMUSG00000028737	Aldh4a1	3927,566593	551,4914943	3688,810448	1192,079044	975,9525486	-0,09048008	False	0,09048008
ENSMUSG00000026554	Dcaf8	1541,040493	1164,820708	1038,22094	1447,363896	1343,891878	-0,090477082	False	0,090477082
ENSMUSG00000029146	Snx17	141,4294936	190,5611342	150,5816631	218,1729571	238,7691396	0,090463088	False	0,090463088
ENSMUSG00000029174	Tbc1d1	87,3307802	82,0296273	104,790748	139,4507561	110,9036987	-0,090345155	False	0,090345155
ENSMUSG00000030122	Ptms	11929,53914	4271,850591	11205,56528	2877,858748	2231,121468	-0,090322881	False	0,090322881
ENSMUSG00000024085	Man2a1	1829,309352	1446,245429	872,6691702	1535,08292	1718,354955	-0,090270987	False	0,090270987
ENSMUSG00000022437	Samm50	902,675675	848,0601468	843,609551	753,483924	611,927467	-0,090041142	False	0,090041142
ENSMUSG00000024254	Abcg8	843,1670902	413,9341193	152,3428521	897,4330916	187,8839131	0,089985824	False	0,089985824
ENSMUSG00000031592	Pcm1	199,3924008	978,0455563	212,2232795	2805,884165	558,4327417	0,089972492	False	0,089972492
ENSMUSG00000030691	Fchsd2	270,493567	94,64956996	220,1486302	287,8983351	109,5989493	0,089965163	False	0,089965163
ENSMUSG00000091896	Ube2d2a	480,7057105	856,8941067	511,6254166	798,4680388	1413,043596	0,089933995	False	0,089933995
ENSMUSG00000025474	Tubgcp2	140,6566548	201,9190826	149,7010685	197,9301054	227,026395	0,089906707	False	0,089906707
ENSMUSG00000031148	Gpkow	281,3133097	363,4543486	299,4021371	454,3395601	517,9855104	0,089906707	False	0,089906707
ENSMUSG00000021967	Mrpl57	290,5873748	983,0935333	751,1471263	309,2657897	733,2691608	0,089875231	False	0,089875231
ENSMUSG00000036636	Clcn7	445,1551274	1264,518255	418,2823974	928,9212794	1321,711139	-0,089830887	False	0,089830887
ENSMUSG00000035268	Pkig	206,3479497	219,5870023	192,8502001	134,9523446	185,2744143	0,089713558	False	0,089713558
ENSMUSG00000017843	Ppp2r5c	842,3942515	704,1928005	505,461255	474,5824118	896,3628354	0,089587242	False	0,089587242
ENSMUSG00000067235	H2-Q10	25795,81222	20772,42562	24246,28954	14147,50412	23293,69098	-0,089372887	False	0,089372887
ENSMUSG00000069895	Atxn1l	225,6689187	166,5832431	78,37291236	79,84680388	240,073889	0,08927077	False	0,08927077
ENSMUSG00000024277	Mapre2	244,217049	73,19566744	259,7753837	114,7094929	165,7031734	0,089100807	False	0,089100807
ENSMUSG00000033972	Zfp944	162,2961402	243,5648934	172,5965261	203,5531198	232,2453926	0,088774738	False	0,088774738
ENSMUSG00000033323	Ctdp1	104,333233	281,4247213	110,9549096	210,300737	563,6517393	0,088774738	False	0,088774738
ENSMUSG00000066363	Serpina3f	23,18516288	1768,053967	24,65664658	556,6784215	4243,045037	0,088774738	False	0,088774738
ENSMUSG00000019179	Mdh2	2039,521495	1308,688054	1917,934866	576,9212731	1465,233572	-0,088676985	False	0,088676985
ENSMUSG00000021514	Zfp369	60,2814235	238,5169163	81,89529044	64,10236368	176,1411685	0,08866407	False	0,08866407
ENSMUSG00000030704	Rab6a	745,0165673	1121,912903	758,1918825	700,627589	2075,85629	-0,088624709	False	0,088624709
ENSMUSG00000042446	Zmym4	139,8838161	206,9670596	150,5816631	251,9110432	148,7414312	0,0885775	False	0,0885775
ENSMUSG00000006850	Tmco6	181,6171093	97,17355849	170,835337	168,6904307	41,75198069	-0,088293693	False	0,088293693
ENSMUSG00000019984	Med23	161,5233014	310,4505895	171,7159316	326,1348328	374,4630768	0,088281593	False	0,088281593
ENSMUSG00000048920	Fkrp	161,5233014	232,206945	171,7159316	174,3134451	211,3694022	0,088281593	False	0,088281593
ENSMUSG00000002346	Slc25a42	1216,448213	579,2553682	1086,653639	1050,379082	1144,265221	-0,08825342	False	0,08825342
ENSMUSG00000031652	N4bp1	250,3997591	217,0630138	233,357548	235,0420002	266,1688769	0,088108712	False	0,088108712
ENSMUSG00000029480	Dhx37	166,9331728	234,7309335	187,5666329	222,6713686	177,4459179	0,088108712	False	0,088108712
ENSMUSG00000042050	Wdr60	84,23942515	95,91156423	79,25350688	137,2015503	103,0752023	-0,088020817	False	0,088020817
ENSMUSG00000027365	Trpm7	603,5870737	567,8974198	720,3263181	1369,766298	738,4881584	-0,087931553	False	0,087931553
ENSMUSG00000072704	Smim10l1	989,2336164	249,8748647	930,7884085	371,1189476	232,2453926	-0,087858022	False	0,087858022
ENSMUSG00000043909	Trp53bp1	160,7504627	228,4209622	170,835337	367,745139	180,0554167	0,087783535	False	0,087783535
ENSMUSG00000042284	Itga1	516,2562936	282,6867156	463,192718	485,8284405	183,9696649	-0,087640516	False	0,087640516
ENSMUSG00000027293	Ehd4	112,8344594	119,8894553	132,0891781	147,3229762	251,8166335	0,087497043	False	0,087497043
ENSMUSG00000029772	Ahcyl2	168,4788503	80,76763303	88,94004661	158,5690049	104,3799517	-0,087456698	False	0,087456698
ENSMUSG00000020888	Dvl2	114,3801369	66,88569611	121,5220439	104,5880671	41,75198069	0,087381503	False	0,087381503
ENSMUSG00000029782	Tmem209	176,9800767	166,5832431	142,6563124	122,581713	127,8654409	-0,087343675	False	0,087343675
ENSMUSG00000037905	Bri3bp	408,8317055	193,0851227	384,8198056	120,3325073	142,2176842	-0,087324031	False	0,087324031
ENSMUSG00000042010	Acacb	2541,093852	1790,769864	784,6097181	2392,030308	754,1451512	-0,087213991	False	0,087213991
ENSMUSG00000033365	Ipo13	185,4813031	335,6904748	206,0591179	367,745139	197,0171589	0,087047517	False	0,087047517
ENSMUSG00000060802	B2m	24958,82784	12929,13126	14923,43534	4246,500443	23499,84138	-0,086899163	False	0,086899163
ENSMUSG00000039686	Zer1	409,6045443	368,5023257	435,0136933	309,2657897	161,7889252	0,086829093	False	0,086829093
ENSMUSG00000029012	Orc5	220,2590474	186,7751514	120,6414494	233,9173973	216,5883998	0,086797883	False	0,086797883
ENSMUSG00000029550	Sppl3	227,2145963	367,2403314	241,2828987	265,4062777	733,2691608	0,086670149	False	0,086670149
ENSMUSG00000057133	Chd6	266,6293732	402,5761709	474,6404467	804,0910532	283,130619	0,086632054	False	0,086632054
ENSMUSG00000029860	Zyx	734,9696634	1865,227525	692,1472934	1064,998919	1527,861543	-0,086605617	False	0,086605617
ENSMUSG00000037134	Prmt9	151,4763975	208,2290539	142,6563124	266,5308806	249,2071347	-0,08654943	False	0,08654943
ENSMUSG00000028581	Laptm5	300,6342787	140,0813635	168,1935535	251,9110432	283,130619	-0,086541739	False	0,086541739
ENSMUSG00000027706	Sec62	2450,671717	2012,880854	2212,053436	1924,195513	2308,101682	-0,086470456	False	0,086470456
ENSMUSG00000049680	Urgcp	204,0294334	268,8047787	216,6262521	399,2340194	533,6425032	0,086430797	False	0,086430797
ENSMUSG00000032437	Stt3b	1415,840613	1824,843709	1503,174847	2176,106557	2642,117528	0,086353966	False	0,086353966
ENSMUSG00000032512	Wdr48	372,5082837	259,9708188	232,4769535	350,8760959	207,455154	-0,086310843	False	0,086310843
ENSMUSG00000006058	Snf8	414,2415769	619,6391847	587,3565454	439,7197228	537,5567514	0,086111881	False	0,086111881
ENSMUSG00000035441	Myo1d	309,1355051	328,1185092	129,4473946	227,1697801	176,1411685	0,085977617	False	0,085977617
ENSMUSG00000029536	Gatc	496,1624857	471,9858555	526,5955235	224,9205743	322,2731009	0,085882598	False	0,085882598
ENSMUSG00000044447	Dock5	242,6713715	164,0592546	209,581496	257,5340576	135,6939372	0,085759314	False	0,085759314
ENSMUSG00000034171	Faah	2185,588021	340,7384519	2318,605373	409,3554453	490,5857731	0,085235813	False	0,085235813
ENSMUSG00000058704	Memo1	277,4491158	206,9670596	261,5365727	199,0547083	243,9881371	-0,085210502	False	0,085210502
ENSMUSG00000020152	Actr2	950,5916787	591,8753108	567,983466	428,4736941	896,3628354	-0,084742938	False	0,084742938
ENSMUSG00000061808	Ttr	92229,03228	49063,81308	97803,23046	10958,13038	60143,72818	0,084661158	False	0,084661158
ENSMUSG00000006418	Rnf114	814,572056	613,3292133	651,6399454	598,2887277	863,7441005	0,084561628	False	0,084561628
ENSMUSG00000034487	Kdelc2	138,3381385	39,12182225	92,46242469	28,11507179	130,4749397	-0,084426214	False	0,084426214
ENSMUSG00000048796	Cyb561d1	88,87645772	106,0075184	94,22361373	127,0801245	117,4274457	0,084287347	False	0,084287347
ENSMUSG00000038806	Sde2	94,28632906	151,4393119	88,94004661	134,9523446	277,9116215	-0,084215443	False	0,084215443
ENSMUSG00000074064	Mlycd	826,1646374	596,9232879	711,5203729	779,34979	493,1952719	-0,084158321	False	0,084158321
ENSMUSG00000028563	Tm2d1	166,160334	97,17355849	62,52221098	59,6039522	176,1411685	0,084156121	False	0,084156121
ENSMUSG00000071533	Pcnp	558,7624255	350,834406	445,5808276	269,9046892	527,1187562	-0,084106981	False	0,084106981
ENSMUSG00000001674	Ddx18	215,6220148	286,4726984	203,4173343	362,1221247	383,5963226	-0,084061859	False	0,084061859
ENSMUSG00000010048	Ifrd2	409,6045443	434,1260276	432,3719097	363,2467275	310,5303564	0,083882199	False	0,083882199
ENSMUSG00000028898	Tmau1ap	139,1109773	225,8969736	188,4472275	250,7864404	147,4366818	0,083859239	False	0,083859239
ENSMUSG00000028759	Hp1bp3	1390,336934	725,646703	1014,444888	1473,229762	711,0884211	0,0835479	False	0,0835479
ENSMUSG00000047547	Cltb	670,8240461	1572,444856	1021,489644	633,1514167	1535,69004	-0,083383846	False	0,083383846
ENSMUSG00000020390	Ube2b	1242,724731	644,87907	823,355877	521,8157324	1172,969707	-0,083341012	False	0,083341012
ENSMUSG00000045538	Ddx28	142,9751711	742,0526285	151,4622576	590,4165076	707,1741729	0,083193706	False	0,083193706
ENSMUSG00000024048	Myl12a	215,6220148	468,1998727	264,1783563	203,5531198	589,7467272	-0,08309915	False	0,08309915
ENSMUSG00000020092	Pald1	117,4714919	162,7972603	116,2384768	283,3999236	110,9036987	-0,083003204	False	0,083003204
ENSMUSG00000056144	Trim34a	130,6097509	41,64581078	125,9250165	138,3261532	18,26649155	0,082811343	False	0,082811343
ENSMUSG00000039345	Mettl22	145,2936874	214,5390252	165,5517699	137,2015503	281,8258696	-0,08267524	False	0,08267524
ENSMUSG00000036188	Ankmy2	142,2023324	100,9595413	150,5816631	134,9523446	44,36147948	0,08260097	False	0,08260097
ENSMUSG00000027937	Jtb	533,2587463	738,2666457	871,7885756	635,4006225	503,6332671	-0,082462141	False	0,082462141
ENSMUSG00000059878	Zfp422	227,987435	185,5131571	151,4622576	73,09918666	241,3786384	0,082343689	False	0,082343689
ENSMUSG00000035168	Tanc1	185,4813031	387,4322397	196,3725782	479,0808233	250,5118841	0,082319716	False	0,082319716
ENSMUSG00000054894	Atp5s	78,82955381	74,4576617	146,1786905	114,7094929	86,11346017	-0,082316299	False	0,082316299
ENSMUSG00000001240	Ramp2	129,8369122	179,2031858	359,2825645	298,019761	122,6464433	-0,082195198	False	0,082195198
ENSMUSG00000037820	Tgm2	1957,600586	8703,974454	1849,248494	4299,356778	15268,17744	-0,082147338	False	0,082147338
ENSMUSG00000035772	Mrps2	791,3868931	507,321695	663,9682687	276,6523064	747,6214042	-0,082075298	False	0,082075298
ENSMUSG00000019843	Fyn	149,1578812	146,3913349	164,6711754	258,6586605	140,9129348	-0,082036162	False	0,082036162
ENSMUSG00000021973	Micu2	534,0315851	252,3988532	504,5806605	205,8023255	253,1213829	-0,08184016	False	0,08184016
ENSMUSG00000028842	Ago3	187,7998194	151,4393119	162,0293918	124,8309187	177,4459179	-0,081816289	False	0,081816289
ENSMUSG00000025089	Gfra1	1231,132149	2739,789552	1163,265362	2010,789934	2385,081897	-0,081805389	False	0,081805389
ENSMUSG00000036352	Ubac1	144,5208486	112,3174897	145,2980959	152,9459905	82,19921198	0,081744656	False	0,081744656
ENSMUSG00000033444	Specc1l	221,8047249	234,7309335	236,8799261	446,46734	236,1596408	0,081717878	False	0,081717878
ENSMUSG00000005501	Usp40	566,4908131	199,395094	348,7154303	535,3109669	67,84696862	-0,08167535	False	0,08167535
ENSMUSG00000054611	Kdm2a	878,7176733	830,3922271	907,892951	1330,405197	927,6768209	-0,081606771	False	0,081606771
ENSMUSG00000028484	Psip1	374,8268	339,4764576	296,7603535	354,2499046	242,6833878	-0,081456644	False	0,081456644
ENSMUSG00000039382	Wdr45	140,6566548	152,7013062	148,820474	199,0547083	200,9314071	0,081395207	False	0,081395207

ENSMUSG00000003208	Ccdc94	64,91845608	220,8489966	68,68637263	182,1856652	354,8918359	0,081395207	False	0,081395207
ENSMUSG00000028034	Fubp1	646,0932057	458,1039186	411,2376412	610,6593593	463,1860358	-0,08137447	False	0,08137447
ENSMUSG00000031229	Atrx	689,3721764	991,9274932	651,6399454	1609,306709	1607,451257	-0,081208028	False	0,081208028
ENSMUSG00000038845	Phb	874,8534795	382,3842626	925,5048414	310,3903926	396,6438165	0,081199122	False	0,081199122
ENSMUSG00000022797	Tfrc	192,4368519	224,6349794	486,96877	242,9142203	203,5409059	0,08093366	False	0,08093366
ENSMUSG00000041238	Rbbp8	72,6468437	145,1293406	68,68637263	158,5690049	131,779689	-0,080876221	False	0,080876221
ENSMUSG00000021190	Lgmn	758,9276651	637,3071044	272,9843015	429,598297	717,6121681	-0,080758033	False	0,080758033
ENSMUSG00000000275	Trim25	856,3053492	766,0305195	718,565129	809,7140676	549,2994959	-0,080712795	False	0,080712795
ENSMUSG00000003123	Lipe	247,3084041	199,395094	261,5365727	191,1824882	56,10422405	0,080701437	False	0,080701437
ENSMUSG00000022561	Gpaa1	244,217049	595,6612936	564,4610879	917,6759432	230,9406432	-0,080641825	False	0,080641825
ENSMUSG00000059474	Mbtd1	193,2096907	431,602039	204,2979288	729,8672637	313,1398552	0,080507122	False	0,080507122
ENSMUSG00000051065	Mb21d2	170,7973666	161,5352661	70,44756167	87,71902399	79,58971319	-0,080436565	False	0,080436565
ENSMUSG00000022808	Snx4	459,0662251	312,974578	182,2830658	195,6808997	485,3667755	0,080373063	False	0,080373063
ENSMUSG00000027422	Rrbp1	5928,446149	12795,35986	6268,071799	11401,22391	26829,56184	0,080367683	False	0,080367683
ENSMUSG00000032463	Faim	81,92090886	165,3212489	77,49231784	80,97140676	288,3496166	-0,080178425	False	0,080178425
ENSMUSG00000039630	Hnrnpu	3107,584665	2905,110801	2274,575647	2939,711906	2101,951278	-0,080118921	False	0,080118921
ENSMUSG00000032504	Pdcd6ip	1814,625415	2229,943868	1917,934866	3470,524462	2335,50142	0,079881957	False	0,079881957
ENSMUSG00000030766	Arhgap17	223,3504025	343,2624404	211,342685	513,9435123	367,9393298	-0,079724676	False	0,079724676
ENSMUSG00000038545	Cul7	121,3356858	410,1481365	142,6563124	552,18001	114,8179469	-0,079655757	False	0,079655757
ENSMUSG00000026655	Fam107b	626,7722366	593,1373051	450,8643947	111,3356843	273,9973733	-0,079575164	False	0,079575164
ENSMUSG00000014867	Surf4	4070,541764	7590,895511	5956,341339	4300,481381	11614,87913	0,079277338	False	0,079277338
ENSMUSG00000027671	Actl6a	210,2121435	364,7163429	199,0143617	408,2308424	529,728255	-0,078973467	False	0,078973467
ENSMUSG00000055553	Kxd1	648,411722	492,1777638	544,2074139	684,8831488	345,7585901	0,078947692	False	0,078947692
ENSMUSG00000021432	Slc35b3	159,9776239	381,1222684	151,4622576	197,9301054	782,8496379	-0,078911791	False	0,078911791
ENSMUSG00000033773	Rpap2	137,5652998	219,5870023	145,2980959	208,0515312	238,7691396	0,078899195	False	0,078899195
ENSMUSG00000023988	Bysl	191,6640132	354,6203888	350,4766193	202,4285169	490,5857731	0,07883306	False	0,07883306
ENSMUSG00000015653	Steap2	164,6146565	103,4835298	155,8652302	120,3325073	48,27572767	-0,078793659	False	0,078793659
ENSMUSG00000026004	Kansl1l	158,4319464	55,52774771	167,312959	103,4634642	40,44723129	0,07868592	False	0,07868592
ENSMUSG00000035757	Selenoo	1450,618358	964,1636193	1132,444554	1531,709111	417,5198069	0,078474328	False	0,078474328
ENSMUSG00000022701	Ccdc191	63,37277855	112,3174897	125,044422	357,6237132	60,01847224	-0,078456675	False	0,078456675
ENSMUSG00000020677	Ddx52	221,0318862	468,1998727	233,357548	463,3363831	1024,228276	0,078287624	False	0,078287624
ENSMUSG00000020962	Gtf2a1	242,6713715	256,184836	249,2082494	154,0705934	217,8931492	0,078181162	False	0,078181162
ENSMUSG00000025503	Taldo1	1251,225957	675,1669324	1320,891781	350,8760959	638,0224549	0,078169926	False	0,078169926
ENSMUSG00000002129	Sf3a1	354,7329921	371,0263142	288,8350028	310,3903926	374,4630768	0,078089956	False	0,078089956
ENSMUSG00000069020	Urm1	132,9282672	194,347117	125,9250165	150,6967848	339,2348431	-0,078083007	False	0,078083007
ENSMUSG00000030697	Ppp4c	377,1453162	571,6834026	409,4764522	398,1094166	1043,799517	0,078044487	False	0,078044487
ENSMUSG00000042328	Hps4	199,3924008	46,69378785	210,4620905	100,0896556	23,48548914	0,077949964	False	0,077949964
ENSMUSG00000030104	Edem1	2159,311503	1442,459446	1679,293751	1239,312365	2045,847054	-0,077873088	False	0,077873088
ENSMUSG00000026656	Fcgr2b	881,0361896	161,5352661	834,8036058	395,8602108	106,9894505	-0,077764449	False	0,077764449
ENSMUSG00000026516	Nvl	293,6787299	256,184836	278,2678686	276,6523064	229,6358938	-0,077764449	False	0,077764449
ENSMUSG00000025995	Wdr75	136,0196223	217,0630138	143,5369069	222,6713686	309,225607	0,077606947	False	0,077606947
ENSMUSG00000035629	Rubcn	334,6391843	114,8414782	299,4021371	353,1253017	45,66622888	0,077573809	False	0,077573809
ENSMUSG00000079523	Tmsb10	346,2317657	328,1185092	589,1177345	331,7578471	373,1583274	-0,077521115	False	0,077521115
ENSMUSG00000056121	Fez2	334,6391843	475,7718383	412,1182358	317,1380098	422,7388045	-0,077495588	False	0,077495588
ENSMUSG00000021831	Ero1l	188,5726581	83,29162157	97,74599182	94,46664122	178,7506673	-0,077171881	False	0,077171881
ENSMUSG00000025364	Pa2g4	825,3917987	1566,134884	1194,08617	870,4426226	1774,459179	0,076670099	False	0,076670099
ENSMUSG00000046671	Mtfr1l	874,8534795	866,9900608	626,1027043	682,6339431	922,4578233	0,076441535	False	0,076441535
ENSMUSG00000020747	Tmem94	725,6955983	127,4614209	637,5504331	688,2569574	87,41820957	-0,076417231	False	0,076417231
ENSMUSG00000030214	Plbd1	659,2314647	334,4284805	625,2221098	404,8570338	319,6636021	-0,076416306	False	0,076416306
ENSMUSG00000034163	Zfc3h1	561,8537806	543,9195287	222,7904138	359,8729189	592,356226	0,076270307	False	0,076270307
ENSMUSG00000032407	U2surp	544,078489	516,1556549	580,3117892	836,7045365	720,2216669	-0,076008592	False	0,076008592
ENSMUSG00000000838	Fmr1	280,5404709	137,557375	178,7606877	118,0833015	266,1688769	-0,075867023	False	0,075867023
ENSMUSG00000025558	Dock9	226,4417575	119,8894553	238,6411152	212,5499427	135,6939372	0,075702598	False	0,075702598
ENSMUSG00000025130	P4hb	17555,0325	29603,8615	29103,64891	18499,71724	33850,41834	0,075618553	False	0,075618553
ENSMUSG00000022353	Mtss1	1974,603039	3527,273974	2080,844853	3414,294318	3760,287761	0,07560675	False	0,07560675
ENSMUSG00000025208	Mrpl43	411,1502218	340,7384519	433,2525043	272,1538949	309,225607	0,075542481	False	0,075542481
ENSMUSG00000058239	Usf2	628,3179142	454,3179358	457,9091508	196,8055025	596,2704742	-0,075527814	False	0,075527814
ENSMUSG00000021620	Acot12	1682,469987	916,2078372	732,6546414	1596,936078	742,4024066	-0,075274203	False	0,075274203
ENSMUSG00000039914	Coq10a	291,3602136	406,3621537	301,1633261	520,6911296	276,6068721	-0,074966886	False	0,074966886
ENSMUSG00000028124	Gclm	2752,851673	989,4035047	2613,604538	813,0878762	1895,800873	-0,074886013	False	0,074886013
ENSMUSG00000025277	Abhd6	598,1772024	1326,355974	650,7593509	567,9244502	1059,45651	-0,074873904	False	0,074873904
ENSMUSG00000026288	Inpp5d	91,96781277	201,9190826	96,86539729	237,2912059	182,6649155	0,074852362	False	0,074852362
ENSMUSG00000068551	Zfp467	469,8859678	248,6128704	494,8941207	353,1253017	93,94195655	0,074809218	False	0,074809218
ENSMUSG00000026078	Pdcl3	241,8985328	229,6829564	182,2830658	159,6936078	163,0936746	-0,074758213	False	0,074758213
ENSMUSG00000003752	Itpkc	74,19252123	188,0371457	70,44756167	128,2047274	229,6358938	-0,074723994	False	0,074723994
ENSMUSG00000047371	Zfp768	329,229313	280,1627271	312,6110549	74,22378953	160,4841758	-0,074723994	False	0,074723994
ENSMUSG00000000282	Mnt	190,1183356	198,1330998	180,5218768	280,026115	246,5976359	-0,074723994	False	0,074723994
ENSMUSG00000027642	Rpn2	2265,963253	1370,525773	1806,979957	1834,227284	2386,386646	0,074703345	False	0,074703345
ENSMUSG00000012422	Tmem167	425,0613195	758,4585539	663,0876742	447,5919429	896,3628354	0,074513087	False	0,074513087
ENSMUSG00000049792	Bag5	112,0616206	167,8452374	117,9996658	137,2015503	276,6068721	0,074490512	False	0,074490512
ENSMUSG00000052397	Ezr	75,73819875	71,93367317	108,3131261	92,21743547	161,7889252	-0,074353836	False	0,074353836
ENSMUSG00000026812	Tsc1	466,7946127	344,5244347	270,3425179	491,4514549	473,6240309	0,074261005	False	0,074261005
ENSMUSG00000022867	Usp25	387,9650589	189,2991399	162,9099864	143,9491676	408,3865611	0,074008666	False	0,074008666
ENSMUSG00000025584	Pde8a	765,8832139	330,6424977	381,2974275	727,6180579	309,225607	-0,073943071	False	0,073943071
ENSMUSG00000038301	Snx10	265,0836956	788,7464163	251,850033	730,9918666	373,1583274	-0,073883018	False	0,073883018
ENSMUSG00000042312	S100a13	182,389948	109,7935012	191,9696055	94,46664122	83,50396138	0,073851687	False	0,073851687
ENSMUSG00000062580	Timm17a	262,7651794	388,694234	259,7753837	219,6618375	746,3166548	-0,073798795	False	0,073798795
ENSMUSG00000031171	Ftsj1	293,6787299	227,1589679	309,0886768	259,7832633	156,5699276	0,073782023	False	0,073782023
ENSMUSG00000037712	Fermt2	1874,906839	1044,931252	1781,442716	914,3021346	1265,606915	-0,073772819	False	0,073772819
ENSMUSG00000023044	Csad	4561,294378	459,3659129	3067,110716	4335,34407	306,6161082	-0,073296786	False	0,073296786
ENSMUSG00000020250	Txnrd1	1391,882612	846,7981526	926,3854359	881,6886513	1323,015888	-0,073207156	False	0,073207156
ENSMUSG00000039414	Heatr5b	171,5702053	180,4651801	142,6563124	133,8277417	65,23746983	0,072921464	False	0,072921464
ENSMUSG00000035297	Cops4	389,5107365	249,8748647	211,342685	245,163426	409,6913105	0,072874199	False	0,072874199
ENSMUSG00000030739	Myh14	261,2195018	42,90780505	274,7454905	169,8150336	27,39973733	0,072833194	False	0,072833194
ENSMUSG00000027122	Arl14ep	183,1627868	174,1552087	104,790748	173,1888422	170,9221709	-0,072752798	False	0,072752798
ENSMUSG00000021952	Xpo4	112,0616206	166,5832431	106,551937	260,9078662	380,9868238	-0,072735441	False	0,072735441
ENSMUSG00000013997	Nit1	1525,583718	660,0230012	1604,443217	1508,092451	232,2453926	0,072711381	False	0,072711381
ENSMUSG00000021357	Exoc2	107,424588	210,7530424	102,1489644	249,6618375	237,4643902	-0,072649666	False	0,072649666
ENSMUSG00000026754	Golga1	317,6367315	189,2991399	302,0439207	240,6650145	71,76121681	-0,072619406	False	0,072619406
ENSMUSG00000031138	F9	2925,194717	1350,333865	2781,798091	797,343436	2533,823328	-0,072514952	False	0,072514952
ENSMUSG00000017307	Acot8	180,0714317	254,9228418	189,327822	226,0451772	220,502648	0,072317116	False	0,072317116
ENSMUSG00000020255	D10Wsu102e	147,6122037	863,204078	169,9547425	155,1951963	1632,241495	0,072271903	False	0,072271903
ENSMUSG00000018965	Ywhah	428,1526746	362,1923544	288,8350028	245,163426	450,1385418	0,072243759	False	0,072243759
ENSMUSG00000031672	Got2	7605,506265	5947,778976	7234,083989	3356,939572	6330,644072	-0,072233937	False	0,072233937
ENSMUSG00000072582	Ptrh2	158,4319464	312,974578	206,0591179	150,6967848	250,5118841	-0,072214635	False	0,072214635
ENSMUSG00000077450	Rab11b	679,3252725	1491,677223	798,6992304	714,1228235	2878,277169	0,072069701	False	0,072069701
ENSMUSG00000045775	Slc16a5	578,8562333	161,5352661	608,4908139	437,4705171	22,18073974	0,072030403	False	0,072030403
ENSMUSG00000066152	Slc31a2	109,7431043	112,3174897	115,3578822	78,72220101	45,66622888	0,071986293	False	0,071986293
ENSMUSG00000018882	Mrpl45	472,9773228	557,8014656	449,9838002	480,2054262	558,4327417	-0,07189795	False	0,07189795
ENSMUSG00000021133	Susd6	487,6612593	418,9820964	512,5060111	396,9848137	497,1095201	0,071689559	False	0,071689559
ENSMUSG00000009378	Slc16a12	741,1523735	215,8010195	705,3562112	170,9396365	112,2084481	-0,071418161	False	0,071418161
ENSMUSG00000057561	Eif1a	783,6585055	1375,57375	745,8635592	1401,255178	2368,120155	-0,071313366	False	0,071313366
ENSMUSG00000039253	Fn3krp	119,0171695	51,74176491	125,044422	22,49205743	22,18073974	0,07127099	False	0,07127099
ENSMUSG00000026021	Sumo1	214,8491761	161,5352661	148,820474	130,4539331	225,7216456	0,071220528	False	0,071220528
ENSMUSG00000036435	Exoc1	168,4788503	349,5724117	176,9994987	383,4895792	508,8522646	0,071177778	False	0,071177778
ENSMUSG00000054405	Dnajc8	405,7403505	455,5799301	482,5657974	426,2244883	563,6517393	0,0710567	False	0,0710567
ENSMUSG00000045098	Kmt5b	333,8663455	357,1443773	317,894622	430,7228998	512,7665128	-0,070722064	False	0,070722064
ENSMUSG00000018412	Kansl1	381,0095101	566,6354255	362,8049426	491,4514549	756,75465	-0,070632898	False	0,070632898
ENSMUSG00000003813	Rad23a	785,204183	201,9190826	371,6108878	205,8023255	824,6016186	0,070629435	False	0,070629435
ENSMUSG00000015149	Sirt2	1093,566849	731,9566744	1148,295255	880,5640485	820,6873704	0,070452228	False	0,070452228
ENSMUSG00000022841	Ap2m1	1515,536814	696,6208349	1591,234299	1120,10446	649,7651995	0,070317382	False	0,070317382
ENSMUSG00000034412	Tbc1d10a	119,0171695	124,9374323	160,2682028	183,3102681	170,9221709	0,070036073	False	0,070036073
ENSMUSG00000062937	Mtap	268,9478895	210,7530424	256,2530056	74,22378953	237,4643902	-0,069757742	False	0,069757742

ENSMUSG00000019961	Tmpo	533,2587463	285,2107041	508,1030385	344,1284787	318,3588527	-0,069714632	False	0,069714632
ENSMUSG00000037400	Atp11b	428,9255134	218,325008	268,5813289	312,6395983	450,1385418	0,069641964	False	0,069641964
ENSMUSG00000040048	Ndufb10	887,2188997	297,8306468	930,7884085	204,6777226	259,6451299	0,069163146	False	0,069163146
ENSMUSG00000002546	Golga2	544,078489	684,0008922	518,6701728	1153,842546	578,0039827	-0,068997383	False	0,068997383
ENSMUSG00000006920	Ezh1	268,9478895	142,6053521	191,9696055	256,4094547	69,15171802	-0,068877207	False	0,068877207
ENSMUSG00000060538	Tmem219	340,8218944	229,6829564	324,9393782	309,2657897	142,2176842	-0,068847428	False	0,068847428
ENSMUSG00000013973	Dedd	280,5404709	336,9524691	459,6703399	401,4832252	267,4736263	-0,068812265	False	0,068812265
ENSMUSG00000039450	Dcxr	1316,144413	331,904492	1254,847192	175,438048	489,2810237	-0,068806104	False	0,068806104
ENSMUSG00000000374	Trappc10	260,4466631	429,0780505	248,3276549	480,2054262	709,7836717	-0,068743017	False	0,068743017
ENSMUSG00000035234	Abraxas1	83,46658638	215,8010195	118,8802603	302,5181725	79,58971319	-0,068616791	False	0,068616791
ENSMUSG00000040455	Usp45	202,4837559	169,1072317	146,1786905	139,4507561	193,1029107	-0,068436263	False	0,068436263
ENSMUSG00000039568	Ubald1	373,2811224	502,2737179	514,2672002	391,3617993	481,4525273	0,068240391	False	0,068240391
ENSMUSG00000034274	Thoc5	222,5775637	422,7680792	233,357548	437,4705171	344,4538407	0,068233959	False	0,068233959
ENSMUSG00000023243	Kcnk5	308,3626664	37,85982798	294,11857	78,72220101	172,2269203	-0,068230232	False	0,068230232
ENSMUSG00000028799	Zfp362	143,7480099	89,6015929	142,6563124	150,6967848	82,19921198	0,068106654	False	0,068106654
ENSMUSG00000017677	Wsb1	611,3154614	740,7906342	766,1172331	1697,025733	583,2229802	-0,06786949	False	0,06786949
ENSMUSG00000033488	Cryzl2	403,4218342	256,184836	371,6108878	422,8506797	174,8364191	0,067859129	False	0,067859129
ENSMUSG00000030249	Abcc9	480,7057105	382,3842626	294,11857	503,8220865	471,0145321	0,067760426	False	0,067760426
ENSMUSG00000021496	Pcbd2	190,1183356	25,23988532	181,4024713	30,36427553	75,675465	-0,067703567	False	0,067703567
ENSMUSG00000025196	Cpn1	5284,67146	2434,386939	5538,058942	2580,96359	1784,897174	0,067566624	False	0,067566624
ENSMUSG00000040943	Tet2	298,3157624	191,8231285	312,6110549	156,3197992	185,2744143	0,067528588	False	0,067528588
ENSMUSG00000039835	Nhsl1	115,9258144	75,71965597	114,4772877	121,4571101	90,02770836	0,067245086	False	0,067245086
ENSMUSG00000003746	Man1a	2784,538062	1243,064352	1308,563458	346,3776845	2657,774521	-0,067219298	False	0,067219298
ENSMUSG00000045252	Zfp574	176,2072379	186,7751514	168,1935535	187,8086796	354,8918359	-0,067150775	False	0,067150775
ENSMUSG00000029621	Arpc1a	519,3476486	432,8640333	468,4762851	332,88245	495,8047707	-0,066928441	False	0,066928441
ENSMUSG00000061979	Rcc1l	124,4270408	159,0112775	130,3279891	230,5435887	242,6833878	0,066846899	False	0,066846899
ENSMUSG00000067150	Xpo5	252,7182754	403,8381652	241,2828987	497,0744693	425,3483033	-0,066804331	False	0,066804331
ENSMUSG00000028964	Park7	1165,440854	781,1744507	1220,504006	560,0522301	675,8601874	0,06660124	False	0,06660124
ENSMUSG00000060657	Marf1	1121,389045	188,0371457	1070,802937	452,0903544	138,303436	-0,066593879	False	0,066593879
ENSMUSG00000063663	Brwd3	117,4714919	94,64956996	72,20875071	42,73490912	112,2084481	-0,066129386	False	0,066129386
ENSMUSG00000022390	Zc3h7b	435,1082235	270,066773	415,6406138	396,9848137	88,72295896	-0,066037651	False	0,066037651
ENSMUSG00000056116	H2-T22	1458,346745	420,2440906	1393,100532	368,8697419	495,8047707	-0,066034412	False	0,066034412
ENSMUSG00000041779	Tram2	124,4270408	270,066773	118,8802603	256,4094547	317,0541034	-0,06579087	False	0,06579087
ENSMUSG00000022412	Mief1	320,7280866	335,6904748	343,4318631	436,3459142	499,7190189	0,065780903	False	0,065780903
ENSMUSG00000063808	Gpatch1	225,6689187	262,4948074	244,8052768	422,8506797	236,1596408	0,065554701	False	0,065554701
ENSMUSG00000032263	Bckdhb	1082,747107	1317,522014	1782,32331	7261,560742	1034,666271	-0,065530812	False	0,065530812
ENSMUSG00000029504	Ddx51	95,83200659	119,8894553	91,58183017	116,9586986	112,2084481	-0,065446181	False	0,065446181
ENSMUSG00000063882	Uqcrh	1211,038341	1019,691367	1267,175516	644,3974454	1192,540948	0,065371824	False	0,065371824
ENSMUSG00000042305	Tmem183a	1351,694996	1167,344696	864,7438195	1385,510738	1414,348346	0,065367841	False	0,065367841
ENSMUSG00000032803	Cdv3	991,5521327	777,3884679	806,6245811	590,4165076	1037,27577	0,065038968	False	0,065038968
ENSMUSG00000020262	Adarb1	94,28632906	128,7234151	98,62658634	149,5721819	225,7216456	0,064927996	False	0,064927996
ENSMUSG00000018820	Zfyve27	225,6689187	288,9966869	215,7456576	384,6141821	348,3680889	-0,064876209	False	0,064876209
ENSMUSG00000036817	Sun1	244,9898878	135,0333865	256,2530056	247,4126318	157,874677	0,064846724	False	0,064846724
ENSMUSG00000039536	Stau1	1395,746806	966,6876079	991,5494305	750,1101154	1334,758633	-0,064458375	False	0,064458375
ENSMUSG00000022897	Dyrk1a	290,5873748	675,1669324	303,8051097	321,6364213	1286,482905	0,064174111	False	0,064174111
ENSMUSG00000037514	Pank2	248,0812429	194,347117	202,5367398	237,2912059	87,41820957	-0,064154024	False	0,064154024
ENSMUSG00000074457	S100a16	224,89608	214,5390252	235,1187371	133,8277417	146,1319324	0,064131	False	0,064131
ENSMUSG00000038539	Atf5	9172,823276	3666,093343	9589,674332	2529,231858	2201,112232	0,064115977	False	0,064115977
ENSMUSG00000024581	Napg	265,0836956	324,3325264	253,611222	331,7578471	885,9248402	-0,063829353	False	0,063829353
ENSMUSG00000001802	Lrp3	289,8145361	181,7271743	302,9245152	276,6523064	86,11346017	0,063828381	False	0,063828381
ENSMUSG00000030609	Aen	101,2418779	135,0333865	96,86539729	142,8245647	143,5224336	-0,063752877	False	0,063752877
ENSMUSG00000109324	Prmt1	139,8838161	318,0225551	187,5666329	146,1983733	281,8258696	0,0636982	False	0,0636982
ENSMUSG00000036676	Tmtc3	129,8369122	82,0296273	86,29826304	83,2206125	135,6939372	0,063655669	False	0,063655669
ENSMUSG00000025035	Arl3	130,6097509	46,69378785	136,4921507	42,73490912	37,8377325	0,06355538	False	0,06355538
ENSMUSG00000033658	Ddx19b	166,160334	159,0112775	68,68637263	67,4761723	140,9129348	-0,063446932	False	0,063446932
ENSMUSG00000063683	Glyat	3249,014159	1269,566232	3109,379253	2209,844643	374,4630768	-0,063375437	False	0,063375437
ENSMUSG00000083844	Ube2d-ps	176,9800767	155,2252947	184,9248494	130,4539331	127,8654409	0,063352141	False	0,063352141
ENSMUSG00000006392	Med8	474,5230004	487,1297867	287,0738138	237,2912059	495,8047707	0,063294136	False	0,063294136
ENSMUSG00000066357	Wdr6	173,8887216	112,3174897	166,4323644	137,2015503	76,98021439	-0,063228356	False	0,063228356
ENSMUSG00000020108	Ddit4	144,5208486	90,86358716	47,55210413	138,3261532	116,1226963	-0,06320368	False	0,06320368
ENSMUSG00000047554	Tmem41b	271,2664057	267,5427844	384,8198056	374,4927562	259,6451299	-0,063169227	False	0,063169227
ENSMUSG00000050312	Nsun3	156,8862688	150,1773177	90,70123565	57,35474645	143,5224336	-0,06305216	False	0,06305216
ENSMUSG00000038002	Cramp1l	201,7109171	193,0851227	252,7306275	224,9205743	366,6345804	-0,06305216	False	0,06305216
ENSMUSG00000029723	Tsc22d4	148,3850425	301,6166296	154,9846357	226,0451772	290,9591154	0,062779529	False	0,062779529
ENSMUSG00000030231	Plekha5	495,389647	517,4176491	597,0430851	815,3370819	636,7177055	0,062765547	False	0,062765547
ENSMUSG00000036918	Ttc7	537,8957789	201,9190826	508,1030385	515,0681152	127,8654409	-0,062563433	False	0,062563433
ENSMUSG00000034463	Scara3	74,96535999	131,2474037	135,6115562	181,0610623	78,28496379	0,062511127	False	0,062511127
ENSMUSG00000025036	Sfxn2	740,3795348	127,4614209	773,1619893	173,1888422	120,0369445	0,062505695	False	0,062505695
ENSMUSG00000022312	Eif3h	1833,946384	1428,577509	1493,488307	570,1736559	1756,192688	-0,062500317	False	0,062500317
ENSMUSG00000027455	Nsfl1c	933,5892255	1293,544123	980,1017017	894,0592829	2332,891921	-0,062417415	False	0,062417415
ENSMUSG00000035152	Ap2b1	723,377082	495,9637466	371,6108878	275,5277035	692,8219295	-0,062263296	False	0,062263296
ENSMUSG00000003528	Slc25a1	3609,157022	1867,751514	3768,063955	1654,290824	1474,366818	0,06216154	False	0,06216154
ENSMUSG00000024787	Snx15	140,6566548	76,98165023	134,7309617	64,10236368	37,8377325	-0,062096386	False	0,062096386
ENSMUSG00000029190	D5Ertd579e	326,9107967	371,0263142	318,7752165	325,0102299	313,1398552	-0,062089884	False	0,062089884
ENSMUSG00000024570	Rbfa	303,7256338	374,812297	361,9243481	317,1380098	317,0541034	0,061960375	False	0,061960375
ENSMUSG00000027628	Aar2	244,9898878	136,2953807	75,73112879	103,4634642	255,7308817	0,061904187	False	0,061904187
ENSMUSG00000029810	Tmem176b	2640,790052	2900,062824	7124,009674	3242,230079	2529,90908	-0,061884071	False	0,061884071
ENSMUSG00000043991	Pura	337,7305393	1231,706404	1273,339677	2657,436586	323,5778503	-0,061759786	False	0,061759786
ENSMUSG00000024963	Dnajc4	199,3924008	287,7346927	306,4468933	208,0515312	219,1978986	0,061330479	False	0,061330479
ENSMUSG00000020220	Vps13d	942,8632906	947,7576939	523,0731454	742,2378953	983,781045	0,061288654	False	0,061288654
ENSMUSG00000067873	Htatsf1	327,6836354	268,8047787	230,7157645	309,2657897	341,8443419	0,06103592	False	0,06103592
ENSMUSG00000021377	Dek	516,2562936	1119,388914	494,8941207	635,4006225	1390,862857	-0,060967561	False	0,060967561
ENSMUSG00000028847	Trappc3	406,5131892	411,4101308	343,4318631	201,303914	424,0435539	0,060910292	False	0,060910292
ENSMUSG00000010453	Kansl3	484,5699043	1173,654668	505,461255	1107,733829	1372,596365	0,060895705	False	0,060895705
ENSMUSG00000020107	Anapc16	411,1502218	1649,426506	428,8495317	599,4133306	1290,397153	0,060805938	False	0,060805938
ENSMUSG00000050212	Eva1b	62,59993979	102,2215356	138,2533398	238,4158088	60,01847224	-0,060754674	False	0,060754674
ENSMUSG00000074221	Zfp568	147,6122037	106,0075184	121,5220439	124,8309187	153,9604288	0,060747595	False	0,060747595
ENSMUSG00000068015	Lrch1	133,701106	122,4134438	110,0743151	128,2047274	58,71372284	-0,060561939	False	0,060561939
ENSMUSG00000087590	Epb41l4aos	45,59748701	112,3174897	47,55210413	119,2079044	263,5593781	0,060554864	False	0,060554864
ENSMUSG00000026926	Pmpca	801,433797	627,2111503	713,2815619	608,4101535	768,4973945	-0,060542982	False	0,060542982
ENSMUSG00000037331	Larp1	2991,658851	2869,774961	3652,706073	3413,169715	3611,54633	-0,060008059	False	0,060008059
ENSMUSG00000034528	Hsd17b13	4566,704249	1280,92418	1593,876083	4760,443956	2511,642588	0,059942764	False	0,059942764
ENSMUSG00000020696	Rffl	239,5800165	321,8085379	229,8351699	617,4069765	326,1873491	-0,059907997	False	0,059907997
ENSMUSG00000047213	Ythdf3	990,0064552	662,5469897	712,4009674	921,0497519	949,8575607	-0,059726747	False	0,059726747
ENSMUSG00000024219	Anks1	163,068979	369,76432	169,9547425	430,7228998	173,5316697	0,05966826	False	0,05966826
ENSMUSG00000024960	Plcb3	367,0984123	161,5352661	352,2378083	292,3967466	61,32322164	-0,059617102	False	0,059617102
ENSMUSG00000038224	Serpinf2	12913,36289	6678,473656	12390,8455	5150,681152	8324,30115	-0,05959012	False	0,05959012
ENSMUSG00000020516	Rps6kb1	438,1995785	607,019242	465,8345015	683,7585459	456,6622888	0,05953956	False	0,05953956
ENSMUSG00000055991	Zkscan5	153,7949138	335,6904748	160,2682028	383,4895792	187,8839131	0,059480431	False	0,059480431
ENSMUSG00000026836	Acvr1	142,9751711	141,3433578	195,4919836	137,2015503	140,9129348	-0,059467849	False	0,059467849
ENSMUSG00000041012	Cmtm8	751,1992774	567,8974198	709,7591838	720,8704407	675,8601874	-0,059455683	False	0,059455683
ENSMUSG00000007338	Mrpl49	406,5131892	118,627461	390,1033727	67,4761723	32,61873491	-0,059445689	False	0,059445689
ENSMUSG00000024146	Cript	421,9699645	750,8865884	506,3418495	439,7197228	1128,608228	0,059443931	False	0,059443931
ENSMUSG00000049960	Mrps16	333,0935068	132,5093979	319,6558111	91,0928326	157,874677	-0,05940791	False	0,05940791
ENSMUSG00000029538	Srsf9	117,4714919	88,33959863	122,4026384	26,99046892	79,58971319	0,059323971	False	0,059323971
ENSMUSG00000051716	Apon	5179,565388	3095,671935	4970,95607	1871,339178	1531,775792	-0,059307694	False	0,059307694
ENSMUSG00000007721	Ccdc124	375,5996387	709,2407776	796,0574469	391,3617993	733,2691608	0,059307267	False	0,059307267
ENSMUSG00000027374	Mrps5	577,3105558	408,8861422	601,4460577	349,7514931	358,806084	0,059087747	False	0,059087747
ENSMUSG00000112023	Lilr4b	108,1974268	453,0559415	112,7160987	488,0776463	514,0712622	0,059027394	False	0,059027394
ENSMUSG00000055302	Mrfap1	1001,599037	793,7943934	961,6092168	244,0388231	905,4960812	-0,058782451	False	0,058782451
ENSMUSG00000001964	Emd	196,3010458	204,4430711	174,3577151	147,3229762	147,4366818	0,05863131	False	0,05863131
ENSMUSG00000029798	Herc6	137,5652998	70,6716789	132,0891781	121,4571101	75,675465	-0,058604329	False	0,058604329
ENSMUSG00000070327	Rnf213	864,8065756	247,3508762	830,4006332	545,4323927	216,5883998	-0,058569949	False	0,058569949

ENSMUSG00000060036	Rpl3	2514,817334	2227,41988	2618,888105	1830,853475	2128,046266	0,058500808	False	0,058500808
ENSMUSG00000070643	Sox13	82,69374762	112,3174897	144,4175014	113,58489	86,11346017	0,058460506	False	0,058460506
ENSMUSG00000050697	Prkaa1	168,4788503	146,3913349	114,4772877	175,438048	46,97097827	0,058394168	False	0,058394168
ENSMUSG00000051550	Zfp579	173,8887216	295,3066583	420,924181	508,320498	167,0079228	-0,058247818	False	0,058247818
ENSMUSG00000002781	Tmem143	319,9552478	90,86358716	307,3274878	259,7832633	43,05673008	-0,05809332	False	0,05809332
ENSMUSG00000039461	Tcta	359,3700247	273,8527558	345,1930522	174,3134451	129,1701903	-0,058066651	False	0,058066651
ENSMUSG00000037957	Wdr20	193,9825295	201,9190826	170,835337	112,4602872	170,9221709	0,057850535	False	0,057850535
ENSMUSG00000073139	Tmem185a	281,3133097	292,7826697	268,5813289	253,0356461	254,4261323	0,057652349	False	0,057652349
ENSMUSG00000028411	Aptx	122,1085245	131,2474037	172,5965261	127,0801245	153,9604288	0,057574489	False	0,057574489
ENSMUSG00000003033	Ap1m1	488,4340981	1029,787321	469,3568796	880,5640485	760,6688982	-0,057478614	False	0,057478614
ENSMUSG00000018196	Glrx2	267,4022119	203,1810768	278,2678686	167,5658279	130,4749397	0,057462932	False	0,057462932
ENSMUSG00000039782	Cpeb2	367,8712511	138,8193693	147,059285	119,2079044	353,5870865	-0,057135349	False	0,057135349
ENSMUSG00000005102	Eif2ak4	138,3381385	446,7459702	132,9697726	355,3745074	545,3852477	-0,057100627	False	0,057100627
ENSMUSG00000025103	Btbd1	540,2142952	400,0521824	244,8052768	227,1697801	519,2902598	-0,056990652	False	0,056990652
ENSMUSG00000024777	Ppp2r5b	153,022075	208,2290539	280,9096522	293,5213495	159,1794264	0,056914091	False	0,056914091
ENSMUSG00000035929	H2-Q4	618,2710102	594,3992994	1054,071641	913,1775318	1334,758633	-0,056806946	False	0,056806946
ENSMUSG00000028793	Rnf19b	289,0416973	170,3692259	215,7456576	141,6999618	277,9116215	-0,056651466	False	0,056651466
ENSMUSG00000027088	Phospho2	195,528207	188,0371457	110,9549096	102,3388613	135,6939372	-0,05635906	False	0,05635906
ENSMUSG00000024269	Tpgs2	141,4294936	92,12558143	147,059285	46,10871774	117,4274457	0,056314866	False	0,056314866
ENSMUSG00000008475	Arpc5	1050,287879	874,5620264	549,490981	610,6593593	1092,075245	0,056287445	False	0,056287445
ENSMUSG00000021759	Plpp1	143,7480099	403,8381652	138,2533398	512,8189095	306,6161082	-0,056227651	False	0,056227651
ENSMUSG00000024587	Nars	1445,981325	1390,717681	1224,906979	1081,867962	1281,263907	-0,056219341	False	0,056219341
ENSMUSG00000005625	Psmd4	1139,164336	1850,083594	1275,100866	1184,206824	1851,439394	0,055945186	False	0,055945186
ENSMUSG00000024235	Map3k8	44,05180948	225,8969736	45,79091508	191,1824882	246,5976359	0,055860115	False	0,055860115
ENSMUSG00000020547	Bzw2	102,7875555	243,5648934	108,3131261	106,8372728	1135,131975	0,055749447	False	0,055749447
ENSMUSG00000074129	Rpl13a	102,7875555	131,2474037	239,5217097	106,8372728	146,1319324	0,055749447	False	0,055749447
ENSMUSG00000036957	Lrfn3	158,4319464	71,93367317	164,6711754	105,7126699	48,27572767	0,055724772	False	0,055724772
ENSMUSG00000006191	Cdkal1	141,4294936	172,8932145	206,9397124	136,0769475	176,1411685	-0,055660327	False	0,055660327
ENSMUSG00000022847	Thpo	514,710616	1452,5554	1188,802603	1282,047274	534,9472526	0,055635106	False	0,055635106
ENSMUSG00000032119	Hinfp	139,1109773	136,2953807	133,8503672	221,5467657	156,5699276	-0,055615172	False	0,055615172
ENSMUSG00000099083	Atf7	307,5898276	253,6608475	231,596359	128,2047274	319,6636021	0,055546692	False	0,055546692
ENSMUSG00000028161	Ppp3ca	597,4043636	661,2849955	620,8191372	652,2696655	1019,009279	0,055465255	False	0,055465255
ENSMUSG00000041360	Pum3	398,0119628	350,834406	237,7605206	327,2594356	413,6055587	0,055443781	False	0,055443781
ENSMUSG00000032028	Nxpe2	512,3920997	6,309971331	493,1329317	16,86904307	15,65699276	-0,055271632	False	0,055271632
ENSMUSG00000001158	Snrnp27	296,7700849	426,554062	408,5958577	285,6491294	431,8720502	-0,05510154	False	0,05510154
ENSMUSG00000040620	Dhx33	258,9009855	339,4764576	249,2082494	431,8475027	738,4881584	-0,055048631	False	0,055048631
ENSMUSG00000026922	Agpat2	3136,952538	2335,951387	3019,558612	1452,98691	1333,453883	-0,055026024	False	0,055026024
ENSMUSG00000029221	Slc30a9	547,9426828	383,6462569	454,3867728	527,4387468	238,7691396	-0,05502143	False	0,05502143
ENSMUSG00000044986	Tst	3834,825941	1520,703091	3983,809612	946,9156179	1141,655722	0,054987605	False	0,054987605
ENSMUSG00000020087	Tysnd1	507,7550672	132,5093979	527,476118	286,7737323	168,3126721	0,054973044	False	0,054973044
ENSMUSG00000022895	Ets2	218,7133699	1545,942976	227,1933864	734,3656752	4009,494895	0,054879424	False	0,054879424
ENSMUSG00000030611	Mrps11	148,3850425	116,1034725	154,1040411	46,10871774	122,6464433	0,054559022	False	0,054559022
ENSMUSG00000033216	Eefsec	609,7697839	374,812297	633,1474605	242,9142203	219,1978986	0,054276882	False	0,054276882
ENSMUSG00000029345	Tfip11	324,5922804	348,3104175	312,6110549	395,8602108	527,1187562	-0,054259892	False	0,054259892
ENSMUSG00000030822	Prr14	598,9500412	921,2558143	682,4607537	621,905388	972,0383004	0,054259444	False	0,054259444
ENSMUSG00000028102	Pex11b	164,6146565	119,8894553	135,6115562	121,4571101	170,9221709	0,054246754	False	0,054246754
ENSMUSG00000025410	Dctn2	1175,487758	2640,092005	1220,504006	2138,994662	1846,220396	0,054217516	False	0,054217516
ENSMUSG00000031820	Babam1	809,1621847	571,6834026	779,326151	730,9918666	429,2625515	-0,054201671	False	0,054201671
ENSMUSG00000003134	Tbc1d8	269,7207282	1056,289201	280,0290576	2257,077963	529,728255	0,05411014	False	0,05411014
ENSMUSG00000031156	Slc35a2	134,4739447	109,7935012	111,8355041	42,73490912	139,6081854	0,054056864	False	0,054056864
ENSMUSG00000060992	Copz1	2173,99544	1100,459	2094,053771	603,9117421	1611,365505	-0,054050426	False	0,054050426
ENSMUSG00000042508	Dmtf1	278,9947934	349,5724117	442,0584495	499,323675	268,7783757	-0,053821127	False	0,053821127
ENSMUSG00000107283	Mpv17	218,7133699	263,7568016	336,387107	257,5340576	227,026395	0,053818626	False	0,053818626
ENSMUSG00000018189	Uchl5	231,8516288	223,3729851	166,4323644	160,8182106	159,1794264	-0,053747146	False	0,053747146
ENSMUSG00000007411	Mark3	515,4834548	768,5545081	496,6553098	861,4457997	705,8694235	-0,053681187	False	0,053681187
ENSMUSG00000023286	Ube2j2	337,7305393	687,786875	350,4766193	394,7356079	1586,575266	0,053445562	False	0,053445562
ENSMUSG00000018008	Cyth4	145,2936874	242,3028991	140,0145288	381,2403735	281,8258696	-0,053395485	False	0,053395485
ENSMUSG00000005823	Gpr108	346,2317657	122,4134438	359,2825645	179,9364595	108,2941999	0,053380831	False	0,053380831
ENSMUSG00000010911	Apip	146,8393649	83,29162157	152,3428521	93,34203834	58,71372284	0,053083031	False	0,053083031
ENSMUSG00000039656	Rxrb	345,458927	548,9675058	358,40197	949,1648236	429,2625515	0,053064374	False	0,053064374
ENSMUSG00000038206	Fbxo8	705,6017904	461,8899014	454,3867728	327,2594356	731,9644114	0,052919284	False	0,052919284
ENSMUSG00000031563	Wwc2	188,5726581	195,6091113	125,9250165	179,9364595	76,98021439	0,052853062	False	0,052853062
ENSMUSG00000021636	Marveld2	163,8418177	131,2474037	169,9547425	130,4539331	58,71372284	0,052846994	False	0,052846994
ENSMUSG00000036473	Tbc1d24	272,0392445	318,0225551	309,9692713	806,340259	262,2546287	-0,052846554	False	0,052846554
ENSMUSG00000037896	Rcor1	272,0392445	186,7751514	130,3279891	132,7031389	262,2546287	-0,052846554	False	0,052846554
ENSMUSG00000024201	Kdm4b	150,7035587	257,4468303	145,2980959	391,3617993	168,3126721	-0,052697688	False	0,052697688
ENSMUSG00000024212	Mllt1	309,9083439	299,0926411	321,4170001	299,1443639	219,1978986	0,052604639	False	0,052604639
ENSMUSG00000026478	Lamc1	243,4442103	234,7309335	212,2232795	133,8277417	221,8073974	-0,052583213	False	0,052583213
ENSMUSG00000031516	Dctn6	277,4491158	287,7346927	331,1035398	327,2594356	403,1675635	0,052515974	False	0,052515974
ENSMUSG00000026277	Stk25	595,0858474	791,2704049	768,7590167	976,1552926	617,1464645	0,052515111	False	0,052515111
ENSMUSG00000037652	Phc3	334,6391843	397,5281938	368,0885097	322,7610242	653,6794477	-0,052140014	False	0,052140014
ENSMUSG00000074582	Arfgef2	949,0460007	692,8348521	627,8638934	915,4267375	499,7190189	-0,052033587	False	0,052033587
ENSMUSG00000020884	Asgr1	5026,543313	1842,511629	4848,553432	1839,850298	1616,584502	-0,052012236	False	0,052012236
ENSMUSG00000028093	Acp6	596,6315249	410,1481365	582,9535728	618,5315794	168,3126721	0,052007046	False	0,052007046
ENSMUSG00000025155	Dus1l	788,2955381	856,8941067	817,1917154	998,64735	1075,113503	0,05193797	False	0,05193797
ENSMUSG00000030978	Rrm1	113,6072981	82,0296273	48,43269865	29,23967466	109,5989493	-0,051821549	False	0,051821549
ENSMUSG00000074406	Zfp628	111,2887818	379,8602741	115,3578822	159,6936078	357,5013346	0,051808411	False	0,051808411
ENSMUSG00000015016	Acsf3	319,182409	307,9266009	201,6561453	211,4253399	260,9498793	-0,05179464	False	0,05179464
ENSMUSG00000001229	Dpp9	1146,119885	8041,427464	1187,922009	7174,966321	4714,05957	0,051682162	False	0,051682162
ENSMUSG00000020536	Llgl1	122,1085245	201,9190826	202,5367398	309,2657897	126,5606915	0,051665467	False	0,051665467
ENSMUSG00000003559	As3mt	480,7057105	451,7939473	368,0885097	498,1990721	250,5118841	0,051568393	False	0,051568393
ENSMUSG00000037621	Atoh8	388,7378977	490,9157695	375,1332659	1049,254479	438,3957972	-0,051394558	False	0,051394558
ENSMUSG00000059142	Zfp945	227,2145963	263,7568016	219,2680357	294,6459524	221,8073974	-0,051360001	False	0,051360001
ENSMUSG00000025332	Kdm5c	667,7326911	855,6321124	728,2516687	644,3974454	1098,598992	-0,051319899	False	0,051319899
ENSMUSG00000024889	Rce1	147,6122037	141,3433578	145,2980959	152,9459905	139,6081854	0,051210288	False	0,051210288
ENSMUSG00000056851	Pcbp2	2359,476743	4992,449317	2615,365727	2277,320815	6316,291829	-0,051129407	False	0,051129407
ENSMUSG00000021171	Esyt2	374,8268	204,4430711	388,3421837	197,9301054	340,5395925	0,05110432	False	0,05110432
ENSMUSG00000041765	Ubac2	391,8292527	309,1885952	405,9540741	227,1697801	288,3496166	0,051091415	False	0,051091415
ENSMUSG00000014554	Dguok	176,9800767	84,55361583	170,835337	122,581713	23,48548914	-0,050980535	False	0,050980535
ENSMUSG00000040354	Mars	677,779595	392,4802168	654,281729	499,323675	262,2546287	-0,050904221	False	0,050904221
ENSMUSG00000043061	Tmem18	161,5233014	116,1034725	167,312959	143,9491676	44,36147948	0,050806888	False	0,050806888
ENSMUSG00000030016	Zfp638	457,5205476	441,6979931	246,5664658	362,1221247	78,28496379	-0,05077626	False	0,05077626
ENSMUSG00000049775	Tmsb4x	1311,50738	569,159414	1266,294921	913,1775318	808,9446258	-0,050612478	False	0,050612478
ENSMUSG00000032116	Stt3a	1596,684884	1137,056834	1541,921006	876,065637	1439,138584	-0,050350759	False	0,050350759
ENSMUSG00000024853	Sf3b2	1309,188864	1065,123161	758,1918825	962,6600581	1264,302165	-0,050331931	False	0,050331931
ENSMUSG00000024487	Yipf5	677,779595	1336,451928	701,8338331	944,6664122	1338,672881	0,050313293	False	0,050313293
ENSMUSG00000027078	Ube2l6	1016,282973	227,1589679	1052,310452	224,9205743	401,8628141	0,05025823	False	0,05025823
ENSMUSG00000041638	Gcn1l1	456,7477088	613,3292133	472,8792577	818,7108905	884,6200908	0,050074369	False	0,050074369
ENSMUSG00000038056	Kmt2c	409,6045443	424,0300734	424,4465591	829,9569193	588,4419778	0,049934867	False	0,049934867
ENSMUSG00000038274	Fau	919,6781277	1282,186174	1677,532562	888,4362686	1818,820659	-0,049860741	False	0,049860741
ENSMUSG00000056429	Tgoln1	1685,561342	1617,876649	589,998329	1274,175054	1628,327247	-0,049838463	False	0,049838463
ENSMUSG00000036992	Nxt1	74,96535999	112,3174897	87,17885756	77,59759814	121,3416939	0,049787889	False	0,049787889
ENSMUSG00000033066	Gas7	133,701106	68,14769037	97,74599182	64,10236368	129,1701903	-0,049738234	False	0,049738234
ENSMUSG00000029623	Pdap1	952,1373558	1354,119848	1455,622743	919,925149	1025,533026	-0,049653233	False	0,049653233
ENSMUSG00000074698	Csnk2a1	868,6707694	803,8903475	666,6100523	603,9117421	898,9723342	0,049467226	False	0,049467226
ENSMUSG00000020604	Arsg	159,9776239	280,1627271	165,5517699	356,4991103	296,178113	-0,049412306	False	0,049412306
ENSMUSG00000024074	Crim1	122,1085245	51,74176491	117,9996658	74,22378953	103,0752023	-0,049381146	False	0,049381146
ENSMUSG00000019978	Epb41l2	183,1627868	342,0004461	176,9994987	484,7038377	206,1504046	-0,049381146	False	0,049381146
ENSMUSG00000023353	Agap3	330,7749905	493,4397581	372,4914823	643,2728426	319,6636021	-0,049295674	False	0,049295674
ENSMUSG00000033128	Gga1	479,1600329	758,4585539	653,4011345	1175,210001	495,8047707	0,049264576	False	0,049264576
ENSMUSG00000056537	Rlim	210,2121435	613,3292133	217,5068467	222,6713686	802,4208789	0,049214802	False	0,049214802
ENSMUSG00000024772	Ehd1	646,8660445	343,2624404	625,2221098	419,4768711	555,8232429	-0,049098187	False	0,049098187
ENSMUSG00000028048	Gba	349,3231208	436,6500161	421,8047755	544,3077899	361,4155828	0,049096577	False	0,049096577

ENSMUSG00000008690	Ncaph2	512,3920997	1007,071424	530,1179016	1121,229063	909,4103294	0,049065028	False	0,049065028
ENSMUSG00000056899	Immp2l	275,1305996	155,2252947	265,9395453	84,34521537	216,5883998	-0,04901828	False	0,04901828
ENSMUSG00000035325	Sec31a	2840,182453	3268,565149	2937,663322	2944,210318	6663,355168	0,04868545	False	0,04868545
ENSMUSG00000032456	Nmnat3	92,74065154	95,91156423	83,65647948	68,60077517	93,94195655	0,048502912	False	0,048502912
ENSMUSG00000029234	Tmem165	142,9751711	190,5611342	138,2533398	143,9491676	147,4366818	-0,0484503	False	0,0484503
ENSMUSG00000026176	Ctdsp1	2483,130945	1244,326346	2567,813623	1113,356843	773,7163921	0,04838015	False	0,04838015
ENSMUSG00000050777	Tmem37	935,9077418	181,7271743	967,7733784	174,3134451	88,72295896	0,048302933	False	0,048302933
ENSMUSG00000007610	Gtpbp3	124,4270408	98,43555276	115,3578822	120,3325073	69,15171802	-0,048273617	False	0,048273617
ENSMUSG00000032187	Smarca4	758,1548263	860,6800895	980,9822962	927,7973691	733,2691608	-0,048149633	False	0,048149633
ENSMUSG00000029513	Prkab1	575,7648783	1894,253393	595,2818961	953,6632351	1249,949922	0,048093232	False	0,048093232
ENSMUSG00000005378	Bud23	261,2195018	270,066773	519,5507673	303,6427753	284,4353684	0,048053544	False	0,048053544
ENSMUSG00000029338	Antxr2	388,7378977	239,7789106	376,0138604	88,84362686	273,9973733	-0,048011919	False	0,048011919
ENSMUSG00000031534	Smim19	238,0343389	246,0888819	167,312959	217,0483542	191,7981613	0,048009767	False	0,048009767
ENSMUSG00000035953	Tmem55b	428,9255134	319,2845493	383,9392111	369,9943448	414,9103081	-0,047927633	False	0,047927633
ENSMUSG00000020954	Strn3	211,757821	206,9670596	206,0591179	500,4482779	204,8456553	-0,047877961	False	0,047877961
ENSMUSG00000063450	Syne2	325,3651191	156,487289	280,9096522	336,2562586	60,01847224	0,047501526	False	0,047501526
ENSMUSG00000029672	Fam3c	165,3874952	121,1514495	158,5070138	140,575359	170,9221709	0,047489388	False	0,047489388
ENSMUSG00000019943	Atp2b1	481,4785492	668,8569611	497,5359043	1068,372728	703,2599247	0,047329116	False	0,047329116
ENSMUSG00000040888	Gfer	450,5649987	638,5690987	523,0731454	465,5855889	499,7190189	0,047311159	False	0,047311159
ENSMUSG00000092558	Med20	179,298593	232,206945	240,4023042	226,0451772	185,2744143	0,047299498	False	0,047299498
ENSMUSG00000054793	Cadm4	89,64929649	159,0112775	103,9101535	176,5626508	92,63720715	0,047299498	False	0,047299498
ENSMUSG00000020311	Erlec1	386,4193814	389,9562282	233,357548	348,6268902	399,2533153	0,047136931	False	0,047136931
ENSMUSG00000020515	Cnot8	455,9748701	212,0150367	471,1180687	380,1157706	135,6939372	0,047134348	False	0,047134348
ENSMUSG00000026921	Egfl7	173,8887216	400,0521824	427,0883426	689,3815603	168,3126721	-0,047020563	False	0,047020563
ENSMUSG00000015697	Setdb1	324,5922804	598,1852821	420,924181	479,0808233	335,3205949	0,046912405	False	0,046912405
ENSMUSG00000014195	Dnajc7	679,3252725	974,2595735	731,7740468	789,4712159	657,5936958	-0,046906058	False	0,046906058
ENSMUSG00000023942	Slc29a1	4119,230606	338,2144633	3988,212585	1463,108336	211,3694022	-0,046632582	False	0,046632582
ENSMUSG00000020300	Cpeb4	867,8979306	275,11475	215,7456576	110,2110814	896,3628354	0,04655745	False	0,04655745
ENSMUSG00000038533	Cbfa2t2	157,6591076	148,9153234	89,82064113	77,59759814	152,6556794	-0,046527249	False	0,046527249
ENSMUSG00000020484	Xbp1	3664,801413	6686,045622	3784,795251	3972,097343	9917,400163	0,046480231	False	0,046480231
ENSMUSG00000046020	Pofut1	154,5677526	54,26575344	149,7010685	113,58489	62,62797103	-0,046154842	False	0,046154842
ENSMUSG00000038535	Zfp280d	221,8047249	782,436445	228,9545754	506,0712922	995,5237895	0,045771298	False	0,045771298
ENSMUSG00000000355	Mcts1	282,0861484	214,5390252	211,342685	273,2784978	114,8179469	-0,045763876	False	0,045763876
ENSMUSG00000015714	Cers2	4918,345886	2334,689392	4764,896952	3385,054644	1902,32462	-0,045728183	False	0,045728183
ENSMUSG00000040481	Bptf	454,4291925	1285,972157	440,2972604	1436,117867	1804,468415	-0,045577649	False	0,045577649
ENSMUSG00000058997	Vwa8	2029,474591	2902,586812	2272,814458	13201,71311	2094,122781	0,045239754	False	0,045239754
ENSMUSG00000032410	Xrn1	253,4911142	263,7568016	261,5365727	307,016584	341,8443419	0,045077528	False	0,045077528
ENSMUSG00000025059	Gk	1048,742201	661,2849955	762,5948551	1016,640996	842,8681101	-0,044849769	False	0,044849769
ENSMUSG00000025037	Maoa	197,8467233	165,3212489	182,2830658	68,60077517	191,7981613	-0,044794282	False	0,044794282
ENSMUSG00000001138	Cnnm3	212,5306598	103,4835298	206,0591179	178,8118566	93,94195655	-0,044612677	False	0,044612677
ENSMUSG00000029434	Vps33a	318,4095703	324,3325264	202,5367398	328,3840385	186,5791637	0,044500306	False	0,044500306
ENSMUSG00000038900	Rpl12	2469,219847	5726,92998	6251,340504	2394,279514	5832,229802	-0,044463707	False	0,044463707
ENSMUSG00000028309	Rnf20	269,7207282	324,3325264	261,5365727	362,1221247	461,8812864	-0,044453694	False	0,044453694
ENSMUSG00000018509	Cenpv	350,0959595	254,9228418	361,0437536	131,578536	354,8918359	0,044423271	False	0,044423271
ENSMUSG00000059811	Atl2	959,0929046	649,9270471	420,924181	707,3752062	989,0000426	0,044300011	False	0,044300011
ENSMUSG00000029472	Anapc5	836,2115414	866,9900608	811,0275537	1377,638518	846,7823583	-0,044117026	False	0,044117026
ENSMUSG00000034681	Rnps1	333,0935068	482,0818097	343,4318631	546,5569956	945,9433125	0,044096666	False	0,044096666
ENSMUSG00000015247	Nipsnap3b	698,6462416	448,0079645	547,729792	190,0578853	720,2216669	0,043878868	False	0,043878868
ENSMUSG00000026705	Klhl20	150,7035587	132,5093979	136,4921507	146,1983733	88,72295896	-0,043786226	False	0,043786226
ENSMUSG00000046574	Prr12	192,4368519	277,6387386	243,9246823	186,6840767	221,8073974	-0,043786226	False	0,043786226
ENSMUSG00000026792	Lrsam1	206,3479497	61,83771904	182,2830658	200,1793111	103,0752023	-0,043786226	False	0,043786226
ENSMUSG00000060288	Ppih	81,14807009	103,4835298	79,25350688	78,72220101	143,5224336	-0,043786226	False	0,043786226
ENSMUSG00000024197	Plin3	556,4439092	272,5907615	431,4913152	573,5474645	195,7124095	0,043676615	False	0,043676615
ENSMUSG00000022912	Pros1	469,8859678	908,6358716	484,3269865	1986,048671	996,8285389	0,043670706	False	0,043670706
ENSMUSG00000022553	Maf1	234,9429839	417,7201021	242,1634932	727,6180579	918,5435751	0,043670706	False	0,043670706
ENSMUSG00000061981	Flot2	770,5202465	3119,649826	747,6247482	2414,522365	2852,182181	-0,043518539	False	0,043518539
ENSMUSG00000036622	Atp13a2	275,1305996	186,7751514	283,5514357	248,5372346	271,3878745	0,043493858	False	0,043493858
ENSMUSG00000001150	Mcm3ap	242,6713715	382,3842626	250,0888439	562,3014358	555,8232429	0,043436782	False	0,043436782
ENSMUSG00000027882	Stxbp3	311,4540214	286,4726984	145,2980959	166,441225	320,9683515	0,04341185	False	0,04341185
ENSMUSG00000029777	Gars	547,1698441	882,133992	530,9984961	578,045876	944,6385631	-0,043280947	False	0,043280947
ENSMUSG00000053094	Tmem248	816,1177335	1421,005544	840,9677674	1169,586986	3252,740246	0,043273215	False	0,043273215
ENSMUSG00000039458	Mtmr12	177,7529154	215,8010195	183,1636603	341,879273	283,130619	0,043260079	False	0,043260079
ENSMUSG00000028356	Ambp	32073,5815	26811,06818	33048,71237	10781,56773	28553,13579	0,043208602	False	0,043208602
ENSMUSG00000005506	Celf1	868,6707694	625,949156	575,9088166	814,2124791	895,058086	0,043171819	False	0,043171819
ENSMUSG00000024993	Fam45a	315,3182152	243,5648934	212,2232795	132,7031389	324,8825997	0,043109963	False	0,043109963
ENSMUSG00000016308	Ube2a	151,4765879	145,1293406	147,059285	51,73173209	144,827183	-0,04269514	False	0,04269514
ENSMUSG00000021782	Dlg5	80,37523133	109,7935012	82,77588496	100,0896556	86,11346017	0,042459545	False	0,042459545
ENSMUSG00000029001	Fbxo44	68,00981113	145,1293406	66,04458906	155,1951963	71,76121681	-0,042302517	False	0,042302517
ENSMUSG00000068739	Sars	610,5426226	781,1744507	664,8488632	628,6530052	794,5923825	0,042171904	False	0,042171904
ENSMUSG00000020549	Elac2	339,2762169	265,0187959	223,6710083	329,5086414	268,7783757	-0,042144001	False	0,042144001
ENSMUSG00000027944	Hax1	601,2685575	362,1923544	619,0579482	289,022938	168,3126721	0,042064945	False	0,042064945
ENSMUSG00000052833	Sae1	176,2072379	119,8894553	181,4024713	70,84998091	112,2084481	0,041920924	False	0,041920924
ENSMUSG00000020485	Supt4a	258,1281468	628,4731445	346,0736467	250,7864404	835,0396138	-0,041628118	False	0,041628118
ENSMUSG00000078676	Casc3	299,0886012	466,9378785	290,5961919	411,604651	919,8483245	-0,041557131	False	0,041557131
ENSMUSG00000020719	Ddx5	4477,827792	2390,21714	4608,151128	2010,789934	3909,029192	0,041388986	False	0,041388986
ENSMUSG00000019370	Calm3	857,0781879	392,4802168	477,2822303	209,1761341	882,010592	0,041369159	False	0,041369159
ENSMUSG00000003346	Abhd17a	640,6833344	435,3880218	622,5803262	264,2816748	341,8443419	-0,041351475	False	0,041351475
ENSMUSG00000018363	Smurf2	674,6882399	589,3513223	483,446392	655,6434742	574,0897345	-0,041309496	False	0,041309496
ENSMUSG00000058979	Hdhd5	211,757821	336,9524691	265,9395453	443,0935314	217,8931492	0,041205583	False	0,041205583
ENSMUSG00000031816	Mthfsd	197,8467233	160,2732718	198,1337672	203,5531198	36,5329831	0,041022161	False	0,041022161
ENSMUSG00000074748	Atxn7l3b	1394,201128	2248,873782	1499,652469	1355,14646	1578,74677	-0,040989918	False	0,040989918
ENSMUSG00000026339	Ccdc93	153,022075	124,9374323	103,9101535	70,84998091	148,7414312	-0,040933233	False	0,040933233
ENSMUSG00000022370	Mrpl13	410,377383	268,8047787	398,9093179	143,9491676	362,7203322	-0,040890399	False	0,040890399
ENSMUSG00000045287	Rtn4rl1	284,4046647	11,3579484	276,5066795	71,97458378	13,04749397	-0,040630796	False	0,040630796
ENSMUSG00000034088	Hdlbp	6305,591466	10512,41224	6485,578646	14320,69297	22307,30043	0,040603596	False	0,040603596
ENSMUSG00000026437	Cdk18	574,2192008	930,0897741	558,2969262	849,0751681	816,7731222	-0,040568959	False	0,040568959
ENSMUSG00000022877	Hrg	20120,85719	4596,183117	19565,04906	3611,099821	8565,679788	-0,04041304	False	0,04041304
ENSMUSG00000040945	Rcc2	237,2615002	355,8823831	299,4021371	626,4037995	243,9881371	0,040332988	False	0,040332988
ENSMUSG00000028436	Dcaf12	237,2615002	239,7789106	149,7010685	121,4571101	243,9881371	0,040332988	False	0,040332988
ENSMUSG00000039065	Fam173b	179,298593	80,76763303	174,3577151	104,5880671	24,79023853	-0,040313964	False	0,040313964
ENSMUSG00000020415	Pttg1	369,4169286	201,9190826	359,2825645	112,4602872	347,0633395	-0,040131055	False	0,040131055
ENSMUSG00000054808	Actn4	2417,43965	2954,328577	3137,558278	2891,353983	2485,5476	0,040083858	False	0,040083858
ENSMUSG00000033427	Upb1	4518,788246	808,9383246	4645,136098	1464,232939	1088,160997	0,039784914	False	0,039784914
ENSMUSG00000018442	Derl2	1422,023324	1282,186174	1383,413992	944,6664122	1259,083168	-0,039712174	False	0,039712174
ENSMUSG00000018750	Zbtb4	301,4071175	210,7530424	293,2379754	255,2848519	37,8377325	-0,039641535	False	0,039641535
ENSMUSG00000026470	Stx6	149,93072	210,7530424	154,1040411	264,2816748	287,0448672	0,039608681	False	0,039608681
ENSMUSG00000022770	Dlg1	648,411722	345,7864289	391,8645618	630,902211	562,3469899	-0,039493765	False	0,039493765
ENSMUSG00000001911	Nfix	1402,702354	641,0930872	1364,921507	663,5156943	566,2612381	-0,039390921	False	0,039390921
ENSMUSG00000017561	Crlf3	88,87645772	119,8894553	145,2980959	96,71584696	91,33245776	0,039326339	False	0,039326339
ENSMUSG00000040025	Ythdf2	254,263953	321,8085379	247,4470604	254,160249	544,0804983	-0,039207042	False	0,039207042
ENSMUSG00000007570	Fance	148,3850425	142,6053521	144,4175014	130,4539331	112,2084481	-0,039100085	False	0,039100085
ENSMUSG00000026970	Rbms1	381,7823488	1181,226633	371,6108878	1116,730652	1136,436724	-0,038957632	False	0,038957632
ENSMUSG00000052632	Asap2	199,3924008	146,3913349	95,98480277	78,72220101	204,8456553	0,038926867	False	0,038926867
ENSMUSG00000055296	Tmem245	571,9006845	172,8932145	339,9094851	556,6784215	168,3126721	-0,038920471	False	0,038920471
ENSMUSG00000067148	Polr1c	149,1578812	254,9228418	153,2234466	182,1856652	424,0435539	0,03879687	False	0,03879687
ENSMUSG00000025793	Hgs	902,675675	1288,496146	927,2660305	1114,481446	2096,73228	0,038775573	False	0,038775573
ENSMUSG00000079470	Utp14b	156,8862688	111,0554954	161,1487973	48,35792348	117,4274457	0,038674332	False	0,038674332
ENSMUSG00000030337	Vamp1	102,7875555	69,40968464	78,37291236	100,0896556	93,94195655	-0,03837273	False	0,03837273
ENSMUSG00000018848	Rars	505,4365509	919,99382	492,2523372	625,2791966	1409,129348	-0,038131941	False	0,038131941
ENSMUSG00000058690	Ccser2	476,8415166	309,1885952	489,6105536	242,9142203	366,6345804	0,038124805	False	0,038124805
ENSMUSG00000061306	Slc38a10	1078,882913	670,1189553	630,5056769	829,9569193	1107,732238	0,038070891	False	0,038070891
ENSMUSG00000036840	Siah1a	70,32832742	223,3729851	72,20875071	93,34203834	456,6622888	0,038067776	False	0,038067776
ENSMUSG00000034126	Pomt2	273,584922	141,3433578	188,4472275	266,5308806	113,5131975	-0,037686027	False	0,037686027

ENSMUSG00000025357	Dgka	193,2096907	300,3546353	258,8947891	800,7172446	198,3219083	0,037676602	False	0,037676602
ENSMUSG00000058396	Gpr182	474,5230004	128,7234151	462,3121234	202,4285169	54,79947465	-0,037610821	False	0,037610821
ENSMUSG00000048379	Socs4	183,9356255	179,2031858	162,9099864	154,0705934	164,398424	-0,03760465	False	0,03760465
ENSMUSG00000028894	Inpp5b	622,1352041	556,5394714	594,4013016	606,1609478	528,4235056	-0,037527236	False	0,037527236
ENSMUSG00000021911	Parg	135,2467835	68,14769037	56,35804933	40,48570338	131,779689	-0,037466256	False	0,037466256
ENSMUSG00000051236	Msrb3	112,0616206	71,93367317	109,1937206	95,59124409	44,36147948	-0,037402368	False	0,037402368
ENSMUSG00000041846	Ppp4r3a	862,4880593	649,9270471	845,37074	885,06246	842,8681101	0,037274788	False	0,037274788
ENSMUSG00000046861	Hectd3	862,4880593	397,5281938	884,9974935	612,908565	224,4168962	0,037168886	False	0,037168886
ENSMUSG00000000631	Myo18a	1051,833556	2128,984327	1953,158647	3823,649764	1079,027751	0,036825541	False	0,036825541
ENSMUSG00000026603	Smyd2	348,550282	647,4030585	357,5213755	612,908565	392,7295683	0,036662705	False	0,036662705
ENSMUSG00000025323	Sp4	109,7431043	20,19190826	66,92518359	85,46981824	106,9894505	-0,036661742	False	0,036661742
ENSMUSG00000061755	Bod1l	356,2786697	551,4914943	365,4467262	940,1680007	571,4802357	0,036654997	False	0,036654997
ENSMUSG00000054648	Zfp869	313,7725377	321,8085379	330,2229453	692,7553689	635,4129561	0,036483514	False	0,036483514
ENSMUSG00000029322	Plac8	85,01226391	126,1994266	87,17885756	101,2142584	105,6847011	0,036307318	False	0,036307318
ENSMUSG00000025736	Jmjd8	766,6560527	68,14769037	747,6247482	195,6808997	79,58971319	-0,036265155	False	0,036265155
ENSMUSG00000023143	Nagpa	190,8911744	106,0075184	187,5666329	100,0896556	195,7124095	0,035984832	False	0,035984832
ENSMUSG00000029388	Eif2b1	238,0343389	427,8160562	315,2528385	244,0388231	489,2810237	0,035940966	False	0,035940966
ENSMUSG00000003037	Rab8a	1098,203882	930,0897741	1001,23597	576,9212731	1071,199255	-0,035919054	False	0,035919054
ENSMUSG00000024528	Srfbp1	102,0147167	165,3212489	99,50718086	123,7063159	195,7124095	-0,035904746	False	0,035904746
ENSMUSG00000004268	Emg1	418,8786094	241,0409048	408,5958577	183,3102681	358,806084	-0,035857635	False	0,035857635
ENSMUSG00000078681	Tm2d3	254,263953	243,5648934	260,6559782	157,444402	247,9023853	0,035820003	False	0,035820003
ENSMUSG00000025651	Uqcrc1	3132,315506	2848,321059	3055,662987	1798,239992	2257,216456	-0,035744101	False	0,035744101
ENSMUSG00000031403	Dkc1	198,619562	219,5870023	217,5068467	203,5531198	204,8456553	0,035397612	False	0,035397612
ENSMUSG00000032763	Ilvbl	224,1232412	229,6829564	148,820474	191,1824882	126,5606915	0,035351544	False	0,035351544
ENSMUSG00000070544	Top1	880,2633508	925,0417971	930,7884085	901,931503	1334,758633	0,035082669	False	0,035082669
ENSMUSG00000030002	Dusp11	929,7250317	979,3075505	1790,248661	1085,241771	952,4670595	0,0348651	False	0,0348651
ENSMUSG00000020184	Mdm2	333,8663455	342,0004461	536,2820632	698,3783833	761,9736476	0,034727533	False	0,034727533
ENSMUSG00000026433	Rab29	153,022075	93,38757569	156,7458247	109,0864785	56,10422405	0,034687222	False	0,034687222
ENSMUSG00000041625	Ggact	940,5447743	355,8823831	963,3704058	266,5308806	213,978901	0,034593981	False	0,034593981
ENSMUSG00000030871	Ears2	159,2047851	112,3174897	154,1040411	163,0674164	43,05673008	0,034584837	False	0,034584837
ENSMUSG00000026096	Osgepl1	221,0318862	215,8010195	183,1636603	141,6999618	138,303436	-0,034552828	False	0,034552828
ENSMUSG00000016252	Atp5e	657,6857871	844,274164	1666,965428	642,1482397	698,0409271	-0,034492112	False	0,034492112
ENSMUSG00000028975	Pex14	835,4387026	855,6321124	735,2964249	554,4292157	844,1728595	0,034456649	False	0,034456649
ENSMUSG00000040865	Ino80d	187,7998194	315,4985665	213,103874	192,307091	391,424819	0,034216286	False	0,034216286
ENSMUSG00000028886	Eya3	216,3948536	389,9562282	211,342685	458,8379716	391,424819	-0,03408201	False	0,03408201
ENSMUSG00000006740	Kif5b	495,389647	1824,843709	507,222444	967,1584696	2339,415668	0,034054866	False	0,034054866
ENSMUSG00000001249	Hpn	3943,796207	2920,254732	4037,525878	3449,157007	1346,501377	0,033886503	False	0,033886503
ENSMUSG00000007564	Ppp2r1a	882,5818671	2031,810768	903,4899784	978,4044983	4943,695463	0,033778492	False	0,033778492
ENSMUSG00000079557	March2	557,9895867	386,1702454	545,0880084	360,9975218	254,4261323	-0,033749016	False	0,033749016
ENSMUSG00000021948	Prkcd	144,5208486	427,8160562	147,9398795	446,46734	540,1662502	0,033733374	False	0,033733374
ENSMUSG00000008373	Prpf31	136,792461	160,2732718	140,0145288	154,0705934	197,0171589	0,033587817	False	0,033587817
ENSMUSG00000020929	Eftud2	303,7256338	545,181523	310,8498659	400,3586223	675,8601874	0,033449282	False	0,033449282
ENSMUSG00000042185	Nfrkb	167,7060115	171,6312202	222,7904138	275,5277035	258,3403805	0,033377603	False	0,033377603
ENSMUSG00000029727	Cyp3a13	3023,34524	1364,215802	2954,394617	1908,451073	1145,56997	-0,033283193	False	0,033283193
ENSMUSG00000031467	Agpat5	115,9258144	118,627461	115,3578822	93,34203834	117,4274457	0,033236155	False	0,033236155
ENSMUSG00000056501	Cebpb	648,4112722	1862,703537	1379,891614	663,5156943	2176,321993	0,033220418	False	0,033220418
ENSMUSG00000032127	Vps11	391,056414	836,7021984	382,1780221	952,5386323	1253,86417	-0,033131931	False	0,033131931
ENSMUSG00000022098	Bmp1	2430,577909	3446,506341	3778,631089	5780,45876	2486,85235	0,033021475	False	0,033021475
ENSMUSG00000031145	Prickle3	132,1554284	305,4026124	254,4918165	473,457809	129,1701903	-0,032962522	False	0,032962522
ENSMUSG00000006169	Clint1	992,3249714	485,8677925	373,3720768	417,2276654	1015,09503	0,032730231	False	0,032730231
ENSMUSG00000028430	Nol6	250,3997591	331,904492	244,8052768	472,3332061	624,9749609	-0,032598519	False	0,032598519
ENSMUSG00000025510	Cd151	582,7204272	266,2807902	569,744655	309,2657897	197,0171589	-0,0324884	False	0,0324884
ENSMUSG00000030107	Usp18	114,3801369	50,47977065	111,8355041	52,85633497	61,32322164	-0,032458268	False	0,032458268
ENSMUSG00000030059	Tmf1	256,5824693	567,8974198	262,4171672	305,8919811	815,4683728	0,0324395	False	0,0324395
ENSMUSG00000025868	Higd2a	762,7918589	641,0930872	745,8635592	177,6872537	688,9076814	-0,032377704	False	0,032377704
ENSMUSG00000051748	Wfdc21	4279,20823	3045,192164	4184,585163	1788,118566	3698,964539	-0,032259274	False	0,032259274
ENSMUSG00000021476	Habp4	1151,529757	295,3066583	1177,354874	476,8316176	155,2651782	0,031997546	False	0,031997546
ENSMUSG00000054509	Parp4	142,9751711	672,6429439	146,1786905	299,1443639	497,1095201	0,031968382	False	0,031968382
ENSMUSG00000021669	Col4a3bp	545,6241665	355,8823831	294,9991645	557,8030243	418,8245563	0,031848211	False	0,031848211
ENSMUSG00000020923	Ubtf	630,6364304	719,3367317	644,5951893	665,7649	1119,474982	0,031584908	False	0,031584908
ENSMUSG00000026502	Desi2	289,8145361	244,8268876	194,6113891	143,9491676	296,178113	0,031335075	False	0,031335075
ENSMUSG00000021460	Auh	470,6588065	282,6867156	411,2376412	142,8245647	460,576537	-0,031240671	False	0,031240671
ENSMUSG00000027803	Wwtr1	132,1554284	212,0150367	152,3428521	129,3293302	348,3680889	-0,03118619	False	0,03118619
ENSMUSG00000028649	Macf1	914,2682564	1095,411023	1222,265195	1888,208221	934,2005679	0,031114792	False	0,031114792
ENSMUSG00000020610	Amz2	248,8540816	243,5648934	226,3127919	236,166603	133,0844384	-0,030993847	False	0,030993847
ENSMUSG00000025880	Smad7	89,64929649	93,38757569	91,58183017	122,581713	101,7704529	0,030769134	False	0,030769134
ENSMUSG00000021578	Ccdc127	314,5453765	247,3508762	210,4620905	209,1761341	307,9208576	-0,030708562	False	0,030708562
ENSMUSG00000086784	Isoc2a	1059,561944	388,694234	1037,340346	426,2244883	494,5000213	-0,030578621	False	0,030578621
ENSMUSG00000028015	Ctso	782,8856667	368,5023257	353,1184029	799,5926417	148,7414312	0,030463565	False	0,030463565
ENSMUSG00000031935	Med17	126,7455571	256,184836	129,4473946	309,2657897	245,2928865	0,030430752	False	0,030430752
ENSMUSG00000000959	Oxa1l	888,7645772	769,8165023	763,4754496	634,2760196	870,2678475	-0,030341821	False	0,030341821
ENSMUSG00000053293	Pom121	197,8467233	463,1518957	193,7307946	345,2530816	948,5528113	-0,030329875	False	0,030329875
ENSMUSG00000046688	Tifa	245,7627266	7319,566744	250,9694384	2597,832633	13637,24069	0,030245565	False	0,030245565
ENSMUSG00000024335	Brd2	1211,038341	1898,039376	1186,16082	1541,830537	2334,19667	-0,029944918	False	0,029944918
ENSMUSG00000030103	Bhlhe40	787,5226993	4457,363748	803,9827975	1494,597216	6299,330086	0,029843125	False	0,029843125
ENSMUSG00000023980	Taf8	170,7973666	343,2624404	174,3577151	366,6205361	639,3272043	0,029764472	False	0,029764472
ENSMUSG00000027947	Il6ra	362,4613798	1119,388914	557,4163317	369,9943448	1268,216413	0,029675936	False	0,029675936
ENSMUSG00000028995	Fam126a	165,3874952	93,38757569	162,0293918	138,3261532	28,70448672	-0,029594619	False	0,029594619
ENSMUSG00000028252	Ccnc	197,0738845	126,1994266	140,8951233	71,97458378	193,1029107	-0,029366698	False	0,029366698
ENSMUSG00000000085	Scmh1	154,5677526	371,0263142	151,4622576	237,2912059	529,728255	-0,029281024	False	0,029281024
ENSMUSG00000027201	Myef2	115,9258144	199,395094	113,5966932	282,2753208	155,2651782	-0,029281024	False	0,029281024
ENSMUSG00000032534	Cep63	118,2443307	208,2290539	120,6414494	341,879273	224,4168962	0,028954652	False	0,028954652
ENSMUSG00000029233	Srd5a3	118,2443307	63,09971331	120,6414494	29,23967466	45,66622888	0,028954652	False	0,028954652
ENSMUSG00000020580	Rock2	359,3700247	442,9599874	352,2378083	510,5697037	394,0343177	-0,028920305	False	0,028920305
ENSMUSG00000001794	Capns1	1927,459874	2594,660211	3075,916661	2095,13515	1889,277126	-0,02886649	False	0,02886649
ENSMUSG00000030934	Oat	5397,505919	2436,910928	2869,857543	1215,695704	5290,758803	-0,028818271	False	0,028818271
ENSMUSG00000037938	Chchd5	184,7084643	251,136859	257,1336001	181,0610623	202,2361565	-0,028773655	False	0,028773655
ENSMUSG00000043241	Upf2	243,4442103	665,0709783	238,6411152	888,4362686	681,079185	-0,028748566	False	0,028748566
ENSMUSG00000078798	Sult2a1	1339,329576	243,5648934	1312,966431	134,9523446	648,4604501	-0,028680986	False	0,028680986
ENSMUSG00000038831	Ralgps1	132,9282672	15,14393119	130,3279891	48,35792348	14,35224336	-0,028500978	False	0,028500978
ENSMUSG00000039934	Gsap	427,3798358	59,31373051	435,8942878	267,6554834	91,33245776	0,028459456	False	0,028459456
ENSMUSG00000037321	Tap1	360,1428635	206,9670596	353,1184029	327,2594356	300,0923612	-0,028417307	False	0,028417307
ENSMUSG00000026187	Xrcc5	322,2737641	287,7346927	251,850033	316,0134069	147,4366818	-0,028300979	False	0,028300979
ENSMUSG00000003308	Keap1	693,2363702	729,4326858	751,1471263	725,3688522	679,7744356	-0,028291239	False	0,028291239
ENSMUSG00000000682	Cd52	127,5183959	71,93367317	125,044422	101,2142584	92,63720715	-0,028264683	False	0,028264683
ENSMUSG00000043866	Taf10	772,065924	1173,654668	1644,950565	787,2220101	885,9248402	0,028046518	False	0,028046518
ENSMUSG00000033767	Tmem131l	404,1946729	619,6391847	412,1182358	536,4355698	2370,729653	0,028007995	False	0,028007995
ENSMUSG00000039377	Hlx	88,10361896	111,0554954	89,82064113	109,0864785	110,9036987	0,027845739	False	0,027845739
ENSMUSG00000027236	Eif3j1	517,0291323	723,1227145	507,222444	908,6791203	698,0409271	-0,027626988	False	0,027626988
ENSMUSG00000044502	Bod1	204,8022721	126,1994266	124,1638274	61,85315794	208,7599034	0,027612919	False	0,027612919
ENSMUSG00000032571	Pik3r4	261,9923406	184,2511629	130,3279891	229,4189858	257,0356311	-0,02755627	False	0,02755627
ENSMUSG00000006021	Kptn	197,8467233	99,69754702	201,6561453	158,5690049	43,05673008	0,027514199	False	0,027514199
ENSMUSG00000026405	C4bp	5680,364907	1885,419434	5789,02838	2620,324691	1182,102953	0,027337618	False	0,027337618
ENSMUSG00000026127	Imp4	336,9577006	542,6575344	438,5360714	330,6332443	802,4208789	-0,027335706	False	0,027335706
ENSMUSG00000008763	Man1a2	429,6983521	437,9120103	290,5961919	283,3999236	339,2348431	0,027316774	False	0,027316774
ENSMUSG00000062624	Cyp2c67	1915,867293	320,5465436	1880,069302	98,9650527	631,4987079	-0,02721179	False	0,02721179
ENSMUSG00000006333	Rps9	2003,198073	2671,641861	2859,290409	2041,154212	2913,505402	0,027080104	False	0,027080104
ENSMUSG00000021457	Syk	80,37523133	179,2031858	81,89529044	169,8150336	177,4459179	0,027029504	False	0,027029504
ENSMUSG00000006127	Inpp5k	211,757821	376,0742913	215,7456576	435,2213113	658,8984452	0,026916267	False	0,026916267
ENSMUSG00000026082	Rev1	132,9282672	171,6312202	242,1634932	476,8316176	130,4749397	-0,026875192	False	0,026875192
ENSMUSG00000032376	Usp3	407,286028	601,9712649	399,7899125	688,2569574	758,0593994	-0,026800253	False	0,026800253
ENSMUSG00000005103	Wdr1	408,8317055	401,3141766	229,8351699	278,9015122	347,0633395	-0,026774963	False	0,026774963
ENSMUSG00000048376	F2r	116,6986532	129,9854094	118,8802603	170,9396365	168,3126721	0,026721269	False	0,026721269

ENSMUSG00000036202	Rif1	138,3381385	142,6053521	141,7757179	214,7991485	140,9129348	0,026605098	False	0,026605098
ENSMUSG00000020956	Dtd2	156,1134301	159,0112775	137,3727453	104,5880671	84,80871077	0,026534435	False	0,026534435
ENSMUSG00000024169	Ift140	125,9727183	41,64581078	119,7608548	123,7063159	15,65699276	-0,026192166	False	0,026192166
ENSMUSG00000025979	Mob4	262,7651794	267,5427844	299,4021371	356,4991103	490,5857731	0,025995512	False	0,025995512
ENSMUSG00000020328	Nudcd2	205,5751109	201,9190826	179,6412823	140,575359	182,6649155	-0,025888347	False	0,025888347
ENSMUSG00000006906	Stambp	264,3108569	243,5648934	198,1337672	164,1920193	259,6451299	-0,025694521	False	0,025694521
ENSMUSG00000061740	Cyp2d22	3675,621156	1285,972157	3739,88493	2480,873935	1180,798204	0,025005805	False	0,025005805
ENSMUSG00000025762	Larp1b	1456,801068	990,6654989	1431,846691	1224,692527	674,555438	-0,024926855	False	0,024926855
ENSMUSG00000052748	Swt1	176,9800767	238,5169163	262,4171672	536,4355698	180,0554167	0,024854042	False	0,024854042
ENSMUSG00000034211	Mrps17	436,653901	673,9049381	564,4610879	444,2181343	678,4696862	0,02477806	False	0,02477806
ENSMUSG00000003421	Nosip	244,217049	315,4985665	292,3573809	485,8284405	240,073889	-0,024685419	False	0,024685419
ENSMUSG00000069255	Dusp22	173,8887216	123,6754381	150,5816631	170,9396365	78,28496379	-0,024677403	False	0,024677403
ENSMUSG00000020869	Lrrc59	1181,670468	4719,858555	1283,026217	1161,714766	10773,31577	-0,024571878	False	0,024571878
ENSMUSG00000031479	Vps36	312,9996989	295,3066583	235,1187371	129,3293302	318,3588527	0,024492613	False	0,024492613
ENSMUSG00000020697	Lig3	400,3304791	266,2807902	357,5213755	393,6110051	263,5593781	-0,024420901	False	0,024420901
ENSMUSG00000056260	Lrif1	208,666466	951,5436767	212,2232795	715,2474263	811,5541246	0,024384151	False	0,024384151
ENSMUSG00000029364	Wsb2	769,7474078	1386,931698	782,848529	1154,967149	1704,002712	0,024348088	False	0,024348088
ENSMUSG00000025220	Mgea5	581,1747496	2213,537943	571,505844	1253,932202	3640,250816	-0,024203772	False	0,024203772
ENSMUSG00000032405	Pias1	299,0886012	343,2624404	294,11857	417,2276584	647,1557007	-0,024175052	False	0,024175052
ENSMUSG00000028907	Utp11	310,6811826	262,4948074	305,5662987	199,0547083	151,35093	-0,023949427	False	0,023949427
ENSMUSG00000028104	Polr3gl	202,4837559	75,71965597	176,9994987	205,8023255	26,09498793	0,023453111	False	0,023453111
ENSMUSG00000036398	Ppp1r11	405,7403505	793,7943934	687,7443208	463,3363831	399,2533153	-0,023252397	False	0,023252397
ENSMUSG00000020476	Dbnl	348,550282	200,6570883	270,3425179	343,0038758	126,5606915	-0,023141916	False	0,023141916
ENSMUSG00000022419	Deptor	2110,622661	638,5690987	2077,322475	1212,321896	309,225607	-0,022943484	False	0,022943484
ENSMUSG00000037780	Mbl1	1898,092001	543,9195287	1928,502001	625,2791966	537,5567514	0,02293072	False	0,02293072
ENSMUSG00000040562	Gstm2	2235,049702	1352,857853	1902,084165	358,748316	2199,807483	-0,022929643	False	0,022929643
ENSMUSG00000022641	Bbx	113,6072981	229,6829564	111,8355041	146,1983733	300,0923612	-0,022677247	False	0,022677247
ENSMUSG00000021733	Slc4a7	96,60484535	129,9854094	95,10420825	158,5690049	243,9881371	-0,022586371	False	0,022586371
ENSMUSG00000032602	Slc25a20	990,0064552	978,0455563	1039,982129	1509,217054	974,6477992	-0,022556954	False	0,022556954
ENSMUSG00000058486	Wdr91	238,8071777	389,9562282	235,1187371	597,1641248	250,5118841	-0,022456685	False	0,022456685
ENSMUSG00000034354	Mtmr3	534,0315851	345,7864289	302,0439207	448,7165458	525,8140068	-0,022372499	False	0,022372499
ENSMUSG00000003363	Pld3	384,1008651	195,6091113	390,1033727	268,7800863	146,1319324	0,022371258	False	0,022371258
ENSMUSG00000028322	Exosc3	102,7875555	74,4576617	88,94004661	34,86268902	104,3799517	0,022179033	False	0,022179033
ENSMUSG00000006299	Aamp	1383,381385	1216,562473	1362,279724	845,7013595	1127,303479	-0,022175979	False	0,022175979
ENSMUSG00000026889	Rbm18	149,1578812	213,277031	151,4622576	378,9911677	326,1873491	0,022118129	False	0,022118129
ENSMUSG00000074909	Ranbp6	148,3850425	70,6716789	99,50718086	50,60712922	146,1319324	-0,022074205	False	0,022074205
ENSMUSG00000068040	Tm9sf4	659,2314647	415,1961136	492,2523372	264,2816748	669,3364404	0,021946457	False	0,021946457
ENSMUSG00000040652	Oaz2	298,3157624	302,8786239	215,7456576	140,575359	272,6926239	0,021899552	False	0,021899552
ENSMUSG00000028466	Creb3	347,7774433	451,7939473	342,5512686	398,1094166	669,3364404	-0,021844435	False	0,021844435
ENSMUSG00000031980	Agt	12067,87728	58265,01327	18096,2174	11887,05235	105404,18	-0,021780918	False	0,021780918
ENSMUSG00000029686	Cul1	844,7127677	1723,884168	832,1618222	1025,637819	2342,025167	-0,021596755	False	0,021596755
ENSMUSG00000024118	Tedc2	753,5177937	129,9854094	742,3411811	364,3713304	78,28496379	-0,021559176	False	0,021559176
ENSMUSG00000023307	March5	908,0855463	2017,928832	894,6840332	1355,14646	3096,170318	-0,021449943	False	0,021449943
ENSMUSG00000046519	Golph3l	129,8369122	49,21777638	65,16399454	71,97458378	131,779689	0,021427433	False	0,021427433
ENSMUSG00000005687	Bcas2	217,1676923	124,9374323	129,4473946	65,22696655	213,978901	-0,021340942	False	0,021340942
ENSMUSG00000025147	Mob2	190,8911744	347,0484232	193,7307946	256,4094547	681,079185	0,021302893	False	0,021302893
ENSMUSG00000033918	Parl	302,952795	417,7201021	298,5215426	309,2657897	491,8905225	-0,02125797	False	0,02125797
ENSMUSG00000033991	Ttc37	196,3010458	103,4835298	115,3578822	193,4316939	172,2269203	-0,021243658	False	0,021243658
ENSMUSG00000063889	Crem	244,9898878	248,6128704	158,5070138	183,3102681	112,2084481	0,021178783	False	0,021178783
ENSMUSG00000021540	Smad5	321,5009253	215,8010195	192,8502001	128,2047274	326,1873491	0,02087794	False	0,02087794
ENSMUSG00000021282	Eif5	3102,947633	4688,308699	3147,244818	7141,228235	5657,393383	0,020450058	False	0,020450058
ENSMUSG00000040242	Fgfr1op2	745,7894061	623,4251675	756,4306934	466,7101917	678,4696862	0,0204396	False	0,0204396
ENSMUSG00000014294	Ndufa2	483,0242268	729,4326858	1173,832496	768,1037613	476,2335297	-0,020426353	False	0,020426353
ENSMUSG00000028232	Tmem68	70,32832742	93,38757569	71,32815619	101,2142584	174,8364191	0,020365774	False	0,020365774
ENSMUSG00000030341	Tnfrsf1a	920,4509665	1834,939663	1070,802937	907,5545174	1829,258654	-0,02035656	False	0,02035656
ENSMUSG00000003131	Pafah1b2	255,0367917	546,4435172	289,7155974	258,6586605	825,906368	0,020344111	False	0,020344111
ENSMUSG00000024767	Otub1	491,5254531	1168,60669	803,102203	484,7038377	1709,221709	-0,020162622	False	0,020162622
ENSMUSG00000030674	Qprt	2075,072078	675,1669324	2046,501666	413,8538568	300,0923612	-0,020001608	False	0,020001608
ENSMUSG00000019854	Reps1	280,5404709	1260,732272	284,4320302	874,9410341	2261,130704	0,019875028	False	0,019875028
ENSMUSG00000055322	Tns1	1441,344293	3838,986558	2165,381927	6307,897507	1461,319324	0,019856475	False	0,019856475
ENSMUSG00000026223	Itm2c	343,9132494	157,7492833	105,6713425	121,4571101	339,2348431	-0,01976034	False	0,01976034
ENSMUSG00000028677	Rnf220	293,6787299	572,9453968	289,7155974	458,8379716	930,2863197	-0,019601423	False	0,019601423
ENSMUSG00000002524	Puf60	707,147468	1855,131571	716,80394	1312,411551	3732,888023	0,019567462	False	0,019567462
ENSMUSG00000024824	Rad9a	67,23697236	68,14769037	156,7458247	145,0737704	71,76121681	0,01941	False	0,01941
ENSMUSG00000039804	Ncoa5	178,5257542	247,3508762	264,1783563	294,6459524	176,1411685	-0,019400071	False	0,019400071
ENSMUSG00000026739	Bmi1	288,2688585	417,7201021	284,4320302	405,9816367	515,3760116	-0,019331054	False	0,019331054
ENSMUSG00000009741	Ubp1	768,974569	1865,227525	779,326151	2387,531896	1737,926196	0,019291341	False	0,019291341
ENSMUSG00000026427	Eif2d	455,9748701	267,5427844	449,9838002	269,9046892	144,827183	-0,019081252	False	0,019081252
ENSMUSG00000052915	Msl1	656,1401096	2995,974388	664,8488632	1644,169398	1681,821972	0,019022502	False	0,019022502
ENSMUSG00000040697	Dnajc16	150,7035587	152,7013062	141,7757179	123,7063159	84,80871077	0,018998918	False	0,018998918
ENSMUSG00000059363	Fxn	251,9454367	257,4468303	268,5813289	255,2848519	260,9498793	0,018996607	False	0,018996607
ENSMUSG00000024816	Frmd8	150,7035587	235,9929278	210,4620905	285,6491294	148,7414312	-0,018906926	False	0,018906926
ENSMUSG00000004937	Sgta	1029,421232	1265,780249	1016,206077	1035,759245	1017,704529	-0,018640447	False	0,018640447
ENSMUSG00000043251	Exoc3l	65,69129484	227,1589679	123,2832329	549,9308042	66,54221922	0,018567779	False	0,018567779
ENSMUSG00000033318	Gstt2	1071,927364	227,1589679	1085,773044	389,1125936	129,1701903	0,018515423	False	0,018515423
ENSMUSG00000054435	Gimap4	194,7553682	46,69378785	197,2531727	79,84680388	14,35224336	0,01838541	False	0,01838541
ENSMUSG00000026883	Dab2ip	180,8442705	94,64956996	183,1636603	177,6872537	45,66622888	0,01838541	False	0,01838541
ENSMUSG00000036983	Tfb1m	125,1998796	35,33583945	126,805611	87,71902399	27,39973733	0,01838541	False	0,01838541
ENSMUSG00000028874	Fgr	46,37032577	141,3433578	45,79091508	212,5499427	156,5699276	-0,018140466	False	0,018140466
ENSMUSG00000056071	S100a9	104,333233	535,0855688	103,0295589	402,607828	862,4393511	-0,018140466	False	0,018140466
ENSMUSG00000021962	Dcp1a	149,93072	261,2328131	162,9099864	151,8213877	481,4525273	0,018079029	False	0,018079029
ENSMUSG00000070730	Rmdn3	739,606696	355,8823831	686,8637263	303,6427753	748,9261536	0,018065185	False	0,018065185
ENSMUSG00000040459	Arglu1	513,1649385	863,204078	519,5507673	1162,839369	1032,056773	0,017842124	False	0,017842124
ENSMUSG00000032737	Inppl1	364,779896	495,9637466	520,4313618	632,0268139	369,2440792	0,017548567	False	0,017548567
ENSMUSG00000021866	Anxa11	585,0389434	418,9820964	441,1778549	524,0649382	578,0039827	-0,017453228	False	0,017453228
ENSMUSG00000029111	Nelfa	183,9356255	181,7271743	220,1486302	202,4285169	371,853578	-0,017426768	False	0,017426768
ENSMUSG00000058546	Rpl23a	1777,529154	1178,702645	1799,054606	381,2403735	1595,708512	0,017365754	False	0,017365754
ENSMUSG00000032342	Mto1	228,7602738	117,3654668	122,4026384	226,0451772	58,71372284	-0,0172254	False	0,0172254
ENSMUSG00000071379	Hpcal1	170,0245278	66,88569611	85,41766852	172,0642394	113,5131975	0,017204404	False	0,017204404
ENSMUSG00000036492	Rnf39	117,4714919	116,1034725	73,08934523	100,0896556	71,76121681	-0,016899563	False	0,016899563
ENSMUSG00000031639	Tlr3	128,2912346	45,43179358	126,805611	57,35474645	61,32322164	-0,016804019	False	0,016804019
ENSMUSG00000069662	Marcks	172,3430441	244,8268876	174,3577151	409,3554453	276,6068721	0,016767132	False	0,016767132
ENSMUSG00000044763	Tmt10c	194,7553682	159,0112775	63,4028055	57,35474645	197,0171589	0,016658189	False	0,016658189
ENSMUSG00000025724	Sec11a	547,1698441	691,5728578	622,5803262	540,9339812	1496,547558	-0,016536192	False	0,016536192
ENSMUSG00000018362	Kpna2	311,4540214	199,395094	95,98480277	132,7031389	307,9208576	-0,016459606	False	0,016459606
ENSMUSG00000024924	Vldlr	161,5233014	155,2252947	55,47745481	159,6936078	147,4366818	-0,016435738	False	0,016435738
ENSMUSG00000048911	Rnf24	146,0665262	360,9303601	144,4175014	199,0547083	369,2440792	-0,016380008	False	0,016380008
ENSMUSG00000030061	Uba3	491,5254531	336,9524691	359,2825645	365,4959333	497,1095201	0,016297605	False	0,016297605
ENSMUSG00000036940	Kdm1a	134,4739447	478,2958269	132,9697726	518,4419238	341,8443419	-0,016228345	False	0,016228345
ENSMUSG00000038451	Spsb2	139,1109773	137,557375	132,0891781	55,10554071	33,92348431	-0,016202778	False	0,016202778
ENSMUSG00000033430	Terf2ip	156,1134301	126,1994266	126,805611	112,4602872	157,874677	0,016185128	False	0,016185128
ENSMUSG00000070780	Rbm47	649,1845608	479,5578211	641,9534057	244,0388231	456,6622888	-0,016160102	False	0,016160102
ENSMUSG00000001441	Npepps	421,1971257	713,0267604	416,5212084	648,8958569	923,7625727	-0,016105635	False	0,016105635
ENSMUSG00000034563	Ccpg1	109,7431043	108,5315069	163,7905809	177,6872537	195,7124095	-0,016016371	False	0,016016371
ENSMUSG00000005142	Man2b1	1132,981626	791,2704049	856,8184688	825,4585078	1145,56997	0,015941115	False	0,015941115
ENSMUSG00000038773	Kdm3b	428,1526746	432,8640333	462,3121234	966,0338667	716,3074187	0,015788594	False	0,015788594
ENSMUSG00000022899	Slc15a2	80,37523133	79,50563877	207,8203069	471,2086032	100,4657035	-0,015693801	False	0,015693801
ENSMUSG00000054693	Adam10	308,3626664	311,7125837	188,4472275	181,0610623	104,3799517	0,015588289	False	0,015588289
ENSMUSG00000037999	Arap2	194,7553682	196,8711055	109,1937206	188,9332824	163,0936746	0,015588289	False	0,015588289
ENSMUSG00000040661	Rad54l2	299,0886012	541,3955402	295,879759	373,3681534	837,6491126	-0,015561922	False	0,015561922
ENSMUSG00000034993	Vat1	523,2118424	528,7755975	522,1925509	470,0840003	410,9960599	0,015260404	False	0,015260404
ENSMUSG00000022365	Derl1	1497,761522	1577,492833	1482,040579	1556,450374	3021,799602	-0,015222983	False	0,015222983
ENSMUSG00000060703	Cd302	3186,414219	2361,191272	3008,991478	1341,651226	3220,121511	0,015181306	False	0,015181306

ENSMUSG00000021009	Ptpn21	293,6787299	566,6354255	296,7603535	601,6625363	447,529043	0,015059584	False	0,015059584
ENSMUSG00000027479	Mapre1	375,5996387	601,9712649	379,5362385	402,607828	857,2203535	0,015041967	False	0,015041967
ENSMUSG00000006498	Ptbp1	1901,956195	2804,151259	1921,457245	2531,481064	5641,736391	0,014716855	False	0,014716855
ENSMUSG00000028619	Tceanc2	123,654202	195,6091113	122,4026384	128,2047274	212,6741516	-0,014676611	False	0,014676611
ENSMUSG00000001524	Gtf2h4	182,389948	184,2511629	178,7606877	181,0610623	117,4274457	0,014647504	False	0,014647504
ENSMUSG00000020166	Cnot2	442,8366111	371,0263142	309,9692713	408,2308424	438,3957972	-0,014540533	False	0,014540533
ENSMUSG00000029152	Ociad1	1446,754164	1219,086461	1093,698395	461,0871774	1461,319324	0,014451671	False	0,014451671
ENSMUSG00000022601	Zbtb11	112,0616206	174,1552087	110,9549096	259,7832633	345,7585901	-0,014318755	False	0,014318755
ENSMUSG00000023030	Slc11a2	435,8810622	431,602039	512,5060111	637,6498282	1063,370758	-0,014232844	False	0,014232844
ENSMUSG00000027304	Rtf1	436,653901	941,4477225	432,3719097	954,787838	1139,046223	-0,014217432	False	0,014217432
ENSMUSG00000041530	Ago1	330,7749905	118,627461	256,2530056	334,0070529	67,84696862	0,014028409	False	0,014028409
ENSMUSG00000001542	Ell2	807,6165071	748,3625998	360,163159	580,2950818	799,8113801	-0,01401059	False	0,01401059
ENSMUSG00000041453	Rpl21	3105,266149	6116,886208	4614,315289	3135,392806	4240,435539	0,013929272	False	0,013929272
ENSMUSG00000020864	Ankrd40	777,4757954	458,1039186	683,3413482	784,9728044	135,6939372	0,013844911	False	0,013844911
ENSMUSG00000034042	Gpbp1l1	690,1450152	413,9341193	295,879759	223,7959715	696,7361777	0,01371294	False	0,01371294
ENSMUSG00000049421	Zfp260	289,8145361	170,3692259	287,0738138	241,7896174	92,63720715	-0,01370822	False	0,01370822
ENSMUSG00000019790	Stxbp5	207,1207884	546,4435172	205,1785234	489,2022492	555,8232429	-0,013592634	False	0,013592634
ENSMUSG00000022438	Parvb	85,01226391	85,8156101	115,3578822	199,0547083	131,779689	0,013569122	False	0,013569122
ENSMUSG00000024875	Yif1a	622,9080428	373,5503028	628,7444879	229,4189858	365,329831	0,013454647	False	0,013454647
ENSMUSG00000024101	Washc1	280,5404709	316,7605608	302,9245152	394,7356079	283,130619	0,013258868	False	0,013258868
ENSMUSG00000049327	Kmt5a	425,8341583	262,4948074	429,7301262	210,300737	405,7770623	0,01313924	False	0,01313924
ENSMUSG00000005370	Msh6	125,9727183	89,6015929	60,76102194	124,8309187	82,19921198	-0,013136014	False	0,013136014
ENSMUSG00000063235	Ptpmt1	574,2192008	1208,990507	700,0726441	569,049053	1544,823285	-0,013048551	False	0,013048551
ENSMUSG00000046722	Cdc42se1	224,1232412	222,1109908	183,1636603	112,4602872	156,5699276	-0,013011477	False	0,013011477
ENSMUSG00000031443	F7	1667,78605	386,1702454	1682,816129	702,8767948	404,4723129	0,012943325	False	0,012943325
ENSMUSG00000021917	Spcs1	656,9129484	499,7497294	461,4315289	358,748316	662,8126934	0,012899029	False	0,012899029
ENSMUSG00000020832	Eral1	202,4837559	227,1589679	204,2979288	204,6777226	502,3285177	0,012868405	False	0,012868405
ENSMUSG00000032562	Gnai2	2124,533759	3469,222238	2751,857878	2143,493073	3047,89459	0,012817487	False	0,012817487
ENSMUSG00000053333	Dis3l2	147,6122037	148,9153234	114,4772877	106,8372728	58,71372284	0,012680215	False	0,012680215
ENSMUSG00000003731	Kpna6	421,1971257	239,7789106	219,2680357	134,9523446	417,5198069	-0,012650952	False	0,012650952
ENSMUSG00000018559	Ctdnep1	441,2909336	774,8644794	458,7897454	437,4705171	885,9248402	-0,012544316	False	0,012544316
ENSMUSG00000069135	Fgfr1op	136,792461	288,9966869	135,6115562	349,7514931	390,1200696	-0,012508598	False	0,012508598
ENSMUSG00000048578	Mlec	2111,3955	1289,75814	2093,173176	949,1648236	1556,56603	-0,012505169	False	0,012505169
ENSMUSG00000028469	Npr2	1091,248333	647,4030585	819,8334989	1081,867962	399,2533153	-0,012455015	False	0,012455015
ENSMUSG00000024797	Vps51	142,9751711	118,627461	141,7757179	76,47299527	96,55145534	-0,012154171	False	0,012154171
ENSMUSG00000025086	Trub1	221,8047249	114,8414782	223,6710083	197,9301054	157,874677	0,012088171	False	0,012088171
ENSMUSG00000020190	Mknk2	1596,684884	1198,894553	1075,20591	1443,990087	1610,060755	0,012035513	False	0,012035513
ENSMUSG00000024068	Spast	372,5082837	203,1810768	281,7902467	375,6173591	199,6266577	0,011991231	False	0,011991231
ENSMUSG00000030470	Csrp3	322,2737641	143,8673463	294,11857	43,85951199	319,6636021	-0,011732261	False	0,011732261
ENSMUSG00000042988	Notum	563,3994581	17,66791973	567,983466	32,61348328	50,88522646	0,011690757	False	0,011690757
ENSMUSG00000034006	Pqlc1	751,1992774	527,5136032	757,3112879	709,624412	507,5475152	0,011690757	False	0,011690757
ENSMUSG00000059810	Rgs3	138,3381385	20,19190826	127,6862055	139,4507561	33,92348431	0,011556807	False	0,011556807
ENSMUSG00000022858	Tra2b	475,2958391	609,5432305	479,0434193	1084,117168	1076,418252	0,011330652	False	0,011330652
ENSMUSG00000032547	Ryk	513,9377773	449,2699587	467,5956906	481,3300291	517,9855104	0,01131804	False	0,01131804
ENSMUSG00000022443	Myh9	1187,08034	1196,370564	1135,086337	787,2220101	712,3931705	0,011246742	False	0,011246742
ENSMUSG00000005204	Senp3	394,9206078	517,4176491	391,8645618	486,9530434	545,3852477	-0,011207544	False	0,011207544
ENSMUSG00000014959	Gorasp2	1562,679978	2893,752852	1550,726951	1683,530499	4826,268018	-0,011077677	False	0,011077677
ENSMUSG00000007617	Homer1	139,8838161	138,8193693	126,805611	74,22378953	50,88522646	-0,01102018	False	0,01102018
ENSMUSG00000046111	Cep295	93,5134903	217,0630138	94,22361373	248,5372346	183,9696649	0,01091416	False	0,01091416
ENSMUSG00000035086	Becn1	540,987134	1037,359287	545,0880084	1262,929025	1474,366818	0,010894899	False	0,010894899
ENSMUSG00000022680	Pdxdc1	1268,22841	1077,743103	1277,74265	840,0783451	1101,208491	0,010782692	False	0,010782692
ENSMUSG00000015597	Zfp318	326,9107967	468,1998727	329,3423508	882,8132542	392,7295683	0,010691018	False	0,010691018
ENSMUSG00000022255	Mtdh	1060,334783	2720,859638	1068,161154	1927,569322	2673,431513	0,01060948	False	0,01060948
ENSMUSG00000000876	Pxmp4	1695,608246	4008,093789	1873,024546	2549,47471	1707,91696	0,010434945	False	0,010434945
ENSMUSG00000036693	Nop14	279,7676321	535,0855688	281,7902467	536,4355698	690,2124308	0,010392619	False	0,010392619
ENSMUSG00000002031	Ift46	372,5082837	456,8419243	369,8496988	975,0306897	480,1477779	-0,010333407	False	0,010333407
ENSMUSG00000003518	Dusp3	496,9353245	299,0926411	423,5659645	500,4482779	116,1226963	0,010162873	False	0,010162873
ENSMUSG00000020076	Ddx50	625,9993979	1328,879962	621,6997317	3387,303849	1959,733594	-0,009943313	False	0,009943313
ENSMUSG00000021431	Snrnp48	312,2268602	523,7276204	314,3722439	611,7839622	362,7203322	0,009879193	False	0,009879193
ENSMUSG00000019837	Gtf3c6	194,7553682	331,904492	294,9991645	193,4316939	200,9314071	-0,009838894	False	0,009838894
ENSMUSG00000042228	Lyn	371,7354449	292,7826697	222,7904138	305,8919811	369,2440792	-0,009701468	False	0,009701468
ENSMUSG00000022297	Fzd6	60,2814235	119,8894553	59,88042742	76,47299527	217,8931492	-0,009628966	False	0,009628966
ENSMUSG00000042229	Rabif	215,6220148	217,0630138	216,6262521	137,2015503	163,0936746	0,009609437	False	0,009609437
ENSMUSG00000019726	Lyst	348,550282	182,9891686	312,6110549	350,8760959	163,0936746	0,00959487	False	0,00959487
ENSMUSG00000050732	Vamp8	1244,270408	1982,592992	2087,009014	1594,686872	1252,559421	0,009578996	False	0,009578996
ENSMUSG00000007850	Hnrnph1	887,9917385	882,133992	1415,115395	942,4172064	1293,006652	-0,009548443	False	0,009548443
ENSMUSG00000025261	Huwe1	2214,183055	2199,656006	2502,649628	3371,559409	3778,554252	-0,009496576	False	0,009496576
ENSMUSG00000021520	Uqcrb	2456,081588	2144,128258	2471,82882	384,6141821	2300,273186	0,009220351	False	0,009220351
ENSMUSG00000020780	Srp68	959,8657434	1659,52246	966,0121894	1986,048671	3963,828667	0,009208764	False	0,009208764
ENSMUSG00000036002	Fam214b	258,1281468	446,7459702	259,7753837	438,5951199	542,7757489	0,009177263	False	0,009177263
ENSMUSG00000036098	Myrf	622,1352041	338,2144633	626,1027043	571,2982588	125,2559421	0,009171188	False	0,009171188
ENSMUSG00000021218	Gdi2	3576,697794	2794,055305	2423,396121	2016,412949	3554,137356	-0,009128788	False	0,009128788
ENSMUSG00000039849	Pcif1	338,5033781	396,2661996	336,387107	409,3554453	362,7203322	-0,00904782	False	0,00904782
ENSMUSG00000056579	Tug1	522,4390037	1590,112775	525,714929	668,0141057	891,1438378	0,009018096	False	0,009018096
ENSMUSG00000022387	Brd1	278,2219546	993,1894875	276,5066795	704,0013976	754,1451512	-0,008921936	False	0,008921936
ENSMUSG00000029028	Lrrc47	236,4886614	406,3621537	302,0439207	235,0420002	1008,571284	-0,008852436	False	0,008852436
ENSMUSG00000097589	Dleu2	346,2317657	189,2991399	278,2678686	344,1284787	165,7031734	-0,008790805	False	0,008790805
ENSMUSG00000033883	D3Ertd254e	108,9702656	109,7935012	108,3131261	111,3356843	138,303436	-0,008726436	False	0,008726436
ENSMUSG00000069520	Tmem19	779,0214729	542,6575344	783,7291236	515,0681152	233,550142	0,008692014	False	0,008692014
ENSMUSG00000073775	Kti12	125,1998796	259,9708188	184,0442549	125,9555216	348,3680889	0,008681194	False	0,008681194
ENSMUSG00000028719	Cmpk1	826,9374762	475,7718383	411,2376412	305,8919811	821,9921198	-0,00865369	False	0,00865369
ENSMUSG00000031960	Aars	2995,523045	4984,877351	4100,048089	3012,811093	4297,844512	0,008302285	False	0,008302285
ENSMUSG00000039307	Hexdc	72,6468437	175,417203	197,2531727	206,9269284	73,0659662	0,008299444	False	0,008299444
ENSMUSG00000048000	Gigyf2	579,6290721	3372,048679	582,9535728	2573,09137	1915,372114	0,008251033	False	0,008251033
ENSMUSG00000032563	Mrpl3	286,723181	160,2732718	97,74599182	139,4507561	288,3496166	0,008160556	False	0,008160556
ENSMUSG00000005262	Ufd1	473,7501616	918,7318257	476,4016358	525,189541	1544,823285	0,008051931	False	0,008051931
ENSMUSG00000040054	Baz2a	691,6906927	783,6984393	1024,131428	1361,894078	695,4314283	0,007781224	False	0,007781224
ENSMUSG00000042492	Tbc1d10b	142,9751711	213,277031	242,1634932	331,7578471	142,2176842	-0,007663763	False	0,007663763
ENSMUSG00000019122	Ccl9	1746,615604	786,2224278	1755,905475	951,4140294	1697,478965	0,007653048	False	0,007653048
ENSMUSG00000057789	Bak1	286,723181	285,2107041	221,0292247	97,84044983	281,8258696	-0,00763042	False	0,00763042
ENSMUSG00000018899	Irf1	584,2661047	176,6791973	587,3565454	303,6427753	96,55145534	0,007610938	False	0,007610938
ENSMUSG00000047215	Rpl9	1563,452817	1389,455687	1537,518033	1089,740183	1555,261281	-0,007578711	False	0,007578711
ENSMUSG00000037058	Paip2	874,8534795	829,1302329	732,6546414	611,7839622	879,4010932	0,007479912	False	0,007479912
ENSMUSG00000019066	Rab3d	203,2565946	51,74165491	204,2979288	84,34521537	23,48548914	0,007372417	False	0,007372417
ENSMUSG00000032777	Gtf3c1	723,377082	527,5136032	639,3116221	719,7458378	575,3944839	-0,00726035	False	0,00726035
ENSMUSG00000028656	Cap1	1387,245579	1379,359733	923,7436524	524,0649382	1380,424862	-0,007110843	False	0,007110843
ENSMUSG00000021036	Sptlc2	235,7158227	686,5248808	236,8799261	593,7903162	576,6992333	0,007107341	False	0,007107341
ENSMUSG00000021676	Iqgap2	4062,040537	3182,749539	1903,845354	1190,954441	4042,11363	-0,007094758	False	0,007094758
ENSMUSG00000030286	Emc3	698,6462416	694,0968464	449,9838002	468,9593975	701,9551753	0,006816773	False	0,006816773
ENSMUSG00000037787	Apopt1	201,7109171	104,7455241	200,7755508	84,34521537	122,6464433	-0,006705571	False	0,006705571
ENSMUSG00000031444	F10	6610,862777	2269,065691	6581,563449	1686,904307	2505,118841	-0,006408232	False	0,006408232
ENSMUSG00000032293	Ireb2	1034,058265	557,8014656	343,4318631	761,3561441	1038,58052	0,006295593	False	0,006295593
ENSMUSG00000021488	Nsd1	795,2510869	1733,980122	798,6992304	1641,920193	821,9921198	0,006241885	False	0,006241885
ENSMUSG00000029056	Pank4	171,5702053	364,7163429	170,835337	481,3300291	328,7968479	-0,006192613	False	0,006192613
ENSMUSG00000068134	Zfp120	154,5677526	155,2252947	75,73112879	131,578536	144,827183	0,00612431	False	0,00612431
ENSMUSG00000027742	Cog6	396,4662853	196,8711055	192,8502001	398,1094166	373,1583274	0,005966809	False	0,005966809
ENSMUSG00000019659	Ccdc12	154,5677526	220,8489966	213,103874	155,1951963	313,1398552	0,005844541	False	0,005844541
ENSMUSG00000003660	Snrnp200	976,8681962	661,2849955	590,8789235	980,6537041	842,8681101	0,005579851	False	0,005579851
ENSMUSG00000002393	Nr2f6	1057,243428	460,6279071	1053,191047	437,4705171	199,6266577	-0,005540429	False	0,005540429
ENSMUSG00000048277	Syngr2	1045,650846	769,8165023	1049,668669	345,2530816	713,6979199	0,005532808	False	0,005532808
ENSMUSG00000043336	Filip1l	124,4270408	218,325008	156,7458247	307,016584	123,9511927	-0,005527897	False	0,005527897
ENSMUSG00000025199	Chuk	1136,072981	1332,665945	1140,369905	2059,147858	2599,060798	0,005446354	False	0,005446354
ENSMUSG00000038642	Ctss	471,4316453	235,9929278	147,059285	137,2015503	469,7097827	-0,005278963	False	0,005278963

ENSMUSG00000022500	Litaf	769,7474078	1463,913349	1008,280726	766,9791584	2361,596408	-0,005197729	False	0,005197729
ENSMUSG00000042472	Zfp410	168,4788503	372,2883085	169,074148	527,4387468	657,5936958	0,005088587	False	0,005088587
ENSMUSG00000022040	Ephx2	7070,701841	2556,800383	3933,615725	7046,761594	2989,180867	-0,004893019	False	0,004893019
ENSMUSG00000074768	Bhmt	21960,98628	807,6763303	21888,938	1251,682996	3969,047664	-0,004740888	False	0,004740888
ENSMUSG00000024158	Hagh	2488,540816	344,5244347	2480,634765	431,8475027	502,3285177	-0,004590713	False	0,004590713
ENSMUSG00000071662	Polr2g	106,6517493	87,07760436	79,25350688	38,23649764	106,9894505	0,004560921	False	0,004560921
ENSMUSG00000041415	Dicer1	471,4316453	694,0968464	472,8792577	599,4133306	701,9551753	0,004423257	False	0,004423257
ENSMUSG00000060126	Tpt1	11600,30983	15868,3159	15846,2984	11635,14131	16908,24743	0,004325394	False	0,004325394
ENSMUSG00000021939	Ctsb	10492,83188	5295,327941	5582,088668	1936,566145	10461,48066	-0,004317038	False	0,004317038
ENSMUSG00000020910	Adprm	213,3034985	124,9374323	60,76102194	76,47299527	212,6741516	-0,004262929	False	0,004262929
ENSMUSG00000020868	Xylt2	250,3997591	165,3212489	194,6113891	249,6618375	106,9894505	-0,004257862	False	0,004257862
ENSMUSG00000017929	B4galt5	277,4491158	164,0592546	278,2678686	113,58489	95,24670595	0,004251126	False	0,004251126
ENSMUSG00000018697	Aatf	167,7060115	294,044664	168,1935535	178,8118566	317,0541034	0,004188007	False	0,004188007
ENSMUSG00000038174	Fam126b	196,3010458	196,8711055	149,7010685	159,6936078	157,874677	0,004183526	False	0,004183526
ENSMUSG00000021276	Cinp	127,5183959	108,5315069	89,82064113	104,5880671	127,8654409	0,003921003	False	0,003921003
ENSMUSG00000020386	Sar1b	2981,611947	1677,19038	2973,767697	950,2894265	2635,593781	-0,003800553	False	0,003800553
ENSMUSG00000021764	Ndufs4	519,3476486	442,9599874	444,700233	165,3166221	517,9855104	-0,003788852	False	0,003788852
ENSMUSG00000029048	Rer1	1165,440854	1132,008857	995,0718086	611,7839622	1162,531712	-0,003605719	False	0,003605719
ENSMUSG00000045316	Fahd1	626,7722366	92,12558143	625,2221098	107,9618757	23,48548914	-0,003572478	False	0,003572478
ENSMUSG00000021501	Caml	282,8589872	315,4985665	283,5514357	303,6427753	373,1583274	0,003527451	False	0,003527451
ENSMUSG00000052676	Zmat1	141,4294936	7,571965597	141,7757179	119,2079044	3,91424819	0,003527451	False	0,003527451
ENSMUSG00000031023	Akip1	156,1134301	156,487289	145,2980959	94,46664122	131,779689	0,003450822	False	0,003450822
ENSMUSG00000019849	Prep	462,9304189	239,7789106	455,2673673	143,9491676	461,8812864	-0,00327327	False	0,00327327
ENSMUSG00000021901	Bap1	340,0490556	437,9120103	340,7900796	506,0712922	631,4987079	0,003140454	False	0,003140454
ENSMUSG00000030763	Lcmt1	118,2443307	53,00375918	117,9996658	49,48252635	30,00923612	-0,002988241	False	0,002988241
ENSMUSG00000000827	Tpd52l2	307,5898276	241,0409048	308,2080823	223,7959715	182,6649155	0,002896903	False	0,002896903
ENSMUSG00000002847	Pla1a	731,8783084	733,2186686	335,5065124	319,3872155	711,0884211	0,002639732	False	0,002639732
ENSMUSG00000042870	Tom1	210,9849822	161,5352661	103,0295589	128,2047274	211,3694022	0,002626235	False	0,002626235
ENSMUSG00000024941	Scyl1	626,7722366	1183,750622	627,8638934	840,0783451	2044,542304	0,002510574	False	0,002510574
ENSMUSG00000020863	Luc7l3	948,273162	1239,278369	946,6391099	2873,360337	1111,646486	-0,002488178	False	0,002488178
ENSMUSG00000033684	Qsox1	4814,012653	7732,238869	4822,135596	6636,281545	8650,488499	0,002432286	False	0,002432286
ENSMUSG00000030603	Psmc4	1075,791558	3062,860084	1632,622242	1073,995742	4279,578021	-0,002410299	False	0,002410299
ENSMUSG00000023051	Tarbp2	131,3825897	186,7751514	144,4175014	131,578536	191,7981613	0,002150058	False	0,002150058
ENSMUSG00000020544	Cox11	76,51103752	100,9595413	76,61172331	103,4634642	127,8654409	0,001897287	False	0,001897287
ENSMUSG00000002111	Spi1	127,5183959	214,5390252	127,6862055	201,303914	468,4050333	0,001897287	False	0,001897287
ENSMUSG00000005936	Kctd20	387,9650589	400,0521824	387,4615892	590,4165076	570,1754863	-0,001873429	False	0,001873429
ENSMUSG00000026966	Ssna1	147,6122037	223,3729851	214,8650631	235,0420002	147,4366818	-0,001716492	False	0,001716492
ENSMUSG00000037415	Ranbp10	968,3669698	653,7130299	813,6693373	969,4076753	600,1847224	0,001549634	False	0,001549634
ENSMUSG00000001211	Agpat3	2780,673869	3090,623958	2783,55928	3974,346548	3618,070077	0,00149626	False	0,00149626
ENSMUSG00000022283	Pabpc1	3529,55463	8584,084998	3525,900462	4387,075802	10605,00309	-0,001494404	False	0,001494404
ENSMUSG00000013076	Amotl1	133,701106	82,0296273	103,0295589	133,8277417	48,27572767	0,00136581	False	0,00136581
ENSMUSG00000003623	Crot	7667,333366	2467,19879	4524,494648	7674,289996	1944,076601	0,001308375	False	0,001308375
ENSMUSG00000022964	Tmem50b	117,4714919	117,3654668	154,1040411	215,9237514	199,6266577	-0,001302708	False	0,001302708
ENSMUSG00000048234	Rnf149	512,3920997	140,0813635	77,49231784	103,4634642	512,7665128	0,001053815	False	0,001053815
ENSMUSG00000001383	Zmat2	425,0613195	355,8823831	425,3271536	271,0292921	375,7678262	0,000901982	False	0,000901982
ENSMUSG00000039183	Nubp2	318,4095703	487,1297867	385,7004001	318,2626127	677,1649368	-0,00066601	False	0,00066601
ENSMUSG00000009585	Apobec3	58,73574597	181,7271743	91,58183017	312,6395983	58,71372284	-0,000541044	False	0,000541044
ENSMUSG00000029624	Ptcd1	132,9282672	188,0371457	132,9697726	176,5626508	208,7599034	0,000450396	False	0,000450396
ENSMUSG00000004561	Mettl17	255,8096305	265,0187959	345,1930522	491,4514549	255,7308817	-0,00044419	False	0,00044419
ENSMUSG00000067713	Prkag1	468,3402903	782,436445	468,4762851	629,7776081	1118,170233	0,000418863	False	0,000418863
ENSMUSG00000041827	Oasl1	267,4022119	60,57572477	197,2531727	111,3356843	267,4736263	0,000385245	False	0,000385245
ENSMUSG00000031422	Morf4l2	1173,169242	2014,142849	1172,951902	1205,574278	2764,763971	-0,000267297	False	0,000267297
ENSMUSG00000071267	Zfp942	183,9356255	252,3988532	193,7307946	348,6268902	183,9696649	0,000266962	False	0,000266962
ENSMUSG00000028271	Gtf2b	189,3454969	422,7680792	189,327822	209,1761341	896,3628354	-0,000134678	False	0,000134678
ENSMUSG00000038525	Armc10	113,6072981	241,0409048	113,5966932	249,6618375	284,4353684	-0,000134678	False	0,000134678
ENSMUSG00000023094	Msrb2	107,424588	92,12558143	107,4325315	80,97140676	84,80871077	0,000106676	False	0,000106676
ENSMUSG00000023452	Pisd	100,4690392	97,17355849	66,92518359	98,9650527	100,4657035	-4,78991E-05	False	4,78991E-05
ENSMUSG00000039068	Zzz3	258,9009855	353,3583945	258,8947891	378,9911677	567,5659875	-3,45292E-05	False	3,45292E-05

1378

Ensembl id	gene name	02ShamLungA	04Ctrl1LungA	06Ctrl2LungA	08Ctrl3LungA	10Ctrl4LungA	log2FC	|log2FC| >= 1	abs log2FC
ENSMUSG00000002847	Pla1a	15,5652812	616,5604405	160,3442208	289,6394392	618,1276288	3,36476881	True	3,36476881
ENSMUSG00000022548	Apod	91,75323657	2417,262662	705,0752721	2318,238147	3426,39653	2,94194633	True	2,94194633
ENSMUSG00000022868	Ahsg	63,08035014	1830,473831	453,5764601	758,900236	788,9728852	2,846083195	True	2,846083195
ENSMUSG00000037379	Spon2	896,2325072	33,61310813	127,3967781	34,80163804	81,863352	-2,814544263	True	2,814544263
ENSMUSG00000027656	Wisp2	749,5911738	874,9011858	2129,503042	515,2887697	113,8968376	-2,718376276	True	2,718376276
ENSMUSG00000067149	Jchain	738,1220192	35,53385716	43,9299235	44,90533941	118,6425391	-2,637237949	True	2,637237949
ENSMUSG00000066108	Muc5b	1154,288485	11871,18942	196,5864076	1346,037549	2606,576585	-2,553768354	True	2,553768354
ENSMUSG00000007682	Dio2	25,39598512	3026,140106	4,39299235	138,0839187	3625,715996	-2,531324546	True	2,531324546
ENSMUSG00000023078	Cxcl13	176,1334452	1027,600734	35,1439388	2018,495006	1993,194657	-2,325321085	True	2,325321085
ENSMUSG00000071178	Serpina1b	40,96126633	686,6677803	203,1758962	415,3743895	323,8941318	2,310397035	True	2,310397035
ENSMUSG00000102918	Pcdhgc3	994,5395464	183,4315329	169,1302055	98,7917467	207,6244435	-2,260052438	True	2,260052438
ENSMUSG00000050064	Zfp697	398,962734	595,4322011	683,1103104	726,343865	85,42262817	-2,223563804	True	2,223563804
ENSMUSG00000061808	Ttr	44,23816763	724,1223865	204,2741443	386,1859189	303,7249002	2,207143074	True	2,207143074
ENSMUSG00000055866	Per2	369,4706223	1692,179901	1745,116211	2223,936934	2175,904168	2,195351393	True	2,195351393
ENSMUSG00000027485	Bpifb1	528,4003356	6762,957355	184,5056787	757,7776025	118,6425391	-2,155010004	True	2,155010004
ENSMUSG00000004187	Kifc2	411,2511139	245,8558766	948,8863461	673,5800911	100,8461583	-2,027863461	True	2,027863461
ENSMUSG00000006574	Slc4a1	371,9282982	93,15632824	58,20714863	60,6222082	46,27059026	-1,997298838	True	1,997298838
ENSMUSG00000032083	Apoa1	85,19943396	1507,787993	339,358659	999,1438019	563,5520608	1,993895074	True	1,993895074
ENSMUSG00000030359	Pzp	49,97274492	690,5092784	196,5864076	401,9027877	274,0642654	1,975950204	True	1,975950204
ENSMUSG00000003477	Inmt	24573,48289	11802,04245	62937,30315	51830,86538	6265,512491	-1,971597925	True	1,971597925
ENSMUSG00000022987	Zfp641	566,903926	554,1360968	1003,798752	1498,715703	147,1167485	-1,946142761	True	1,946142761
ENSMUSG00000073424	Cyp4f15	323,594004	416,8025408	384,3868306	632,0426522	84,23620278	-1,941672567	True	1,941672567
ENSMUSG00000058427	Cxcl2	12,2883799	315,9632164	3,294744262	50,51850684	5355,524216	-1,89905633	True	1,89905633
ENSMUSG00000002265	Peg3	177,7718959	2083,052329	643,5733792	2918,847062	810,3285422	1,856077387	True	1,856077387
ENSMUSG00000038357	Camp	15,5652812	1139,964553	4,39299235	318,8279098	800,8371391	-1,825055749	True	1,825055749
ENSMUSG00000017679	Ttpal	514,4735051	689,5489039	597,4469596	648,8821545	150,6760247	-1,771646905	True	1,771646905
ENSMUSG00000005681	Apoa2	78,64563135	1051,610097	267,9725333	615,2031499	297,7927732	1,768646601	True	1,768646601
ENSMUSG00000046417	Lrrc75a	222,0100635	147,8976758	373,4043497	309,8468419	66,43982191	-1,740504963	True	1,740504963
ENSMUSG00000032827	Ppp1r9a	1160,023062	650,1735486	2181,120702	1703,034997	352,3683412	-1,718997273	True	1,718997273
ENSMUSG00000059657	Stfa2l1	81,92253265	692,4300274	267,9725333	655,6179554	392,7068045	1,709752912	True	1,709752912
ENSMUSG00000041046	Ramp3	74,54950471	536,8493555	240,5163311	261,5736021	1129,476972	1,689864184	True	1,689864184
ENSMUSG00000029168	Dpysl5	254,7790766	194,9560271	388,7798229	482,7323987	79,49050121	-1,680392423	True	1,680392423
ENSMUSG00000022651	Retnlg	521,846533	4640,529671	1635,291402	6240,719544	2836,74311	1,647850241	True	1,647850241
ENSMUSG00000074971	Fibin	717,641386	496,5136258	1170,732461	1469,527232	232,5393767	-1,625788059	True	1,625788059
ENSMUSG00000031877	Ces2g	624,2496988	51,86022397	204,2741443	141,4518191	110,3375614	-1,611616612	True	1,611616612
ENSMUSG00000032648	Pygm	568,5423766	363,0215678	533,7485705	188,6024255	1372,694178	-1,591919655	True	1,591919655
ENSMUSG00000051177	Plcb1	389,9512554	66,26584174	131,7897705	84,19751139	37,96561252	-1,565055403	True	1,565055403
ENSMUSG00000040809	Chil3	1907,15656	8051,779958	5887,707997	7050,138287	5639,079885	1,5640365	True	1,5640365
ENSMUSG00000025511	Tspan4	414,5280152	2407,658916	1225,644866	1779,374074	2108,27792	1,563999502	True	1,563999502
ENSMUSG00000037071	Scd1	34944,0563	8551,174708	11857,7846	6689,772939	10470,20408	-1,559212591	True	1,559212591
ENSMUSG00000041757	Plekha6	1317,314325	369,7441894	451,3799639	409,7612221	298,9791986	-1,545185343	True	1,545185343
ENSMUSG00000029368	Alb	609,5036429	7726,212997	1773,670661	4720,673805	4644,855407	1,541031403	True	1,541031403
ENSMUSG00000023764	Sfi1	551,3386448	487,8702551	813,8018328	801,5603085	189,8280626	-1,538245443	True	1,538245443
ENSMUSG00000024440	Pcdh12	772,5294829	63,38471818	276,758518	97,66911322	43,89773948	-1,480962273	True	1,480962273
ENSMUSG00000028364	Tnc	339,9785105	118,1260657	121,9055377	74,09381003	60,50769495	-1,479679895	True	1,479679895
ENSMUSG00000044338	Aplnr	1709,723256	90,27520469	618,3136732	142,5744526	115,083263	-1,467352008	True	1,467352008
ENSMUSG00000001025	S100a6	2646,91703	10474,80487	9639,323463	10473,04778	7294,143305	1,462425571	True	1,462425571
ENSMUSG00000023034	Nr4a1	1509,832277	1544,282225	685,3068065	550,0904078	3567,581152	-1,456647642	True	1,456647642
ENSMUSG00000027952	Pmvk	334,2439332	6508,458108	903,8581759	3136,637958	5399,421956	1,435195043	True	1,435195043
ENSMUSG00000024299	Adamts10	3022,12223	2651,594044	5437,426281	4465,836004	1118,799144	-1,433610956	True	1,433610956
ENSMUSG00000005540	Fcer2a	847,0789876	51,86022397	316,2954492	15,71686879	64,06697113	-1,421223704	True	1,421223704
ENSMUSG00000028307	Aldob	92,5724619	866,2578152	247,1058197	571,420444	278,8099669	1,416473993	True	1,416473993
ENSMUSG00000031442	Mcf2l	1050,246869	1090,985452	1123,507793	3475,67327	395,0796553	-1,410513023	True	1,410513023
ENSMUSG00000079105	C7	1892,410504	2590,130075	716,057753	2226,182201	2415,562097	-1,402077217	True	1,402077217
ENSMUSG00000045672	Col27a1	451,3931549	159,42217	1192,697423	205,4419278	107,9647106	1,401771644	True	1,401771644
ENSMUSG00000002109	Ddb2	330,9670319	288,1123554	489,818647	603,9768151	125,7610915	-1,396001872	True	1,396001872
ENSMUSG00000056216	Cebpg	996,1779971	1199,507773	1057,612908	1601,997983	379,6561252	-1,391710262	True	1,391710262
ENSMUSG00000045777	Ifitm10	280,1750617	179,5900349	512,8818568	342,403213	106,7782852	-1,391710262	True	1,391710262
ENSMUSG00000049001	Ndnf	2447,02605	452,3363979	945,5916033	353,6295479	189,8280626	-1,37174033	True	1,37174033
ENSMUSG00000026073	Il1r2	81,10330733	2380,76843	207,5688885	1824,279414	2273,19105	1,355757569	True	1,355757569
ENSMUSG00000013033	Adgrl1	1661,388962	1289,782978	2915,848672	1947,769097	650,1611144	-1,353520697	True	1,353520697
ENSMUSG00000049690	Nckap5	1507,374601	146,9373012	593,0539672	243,6114663	139,9981962	-1,345802689	True	1,345802689
ENSMUSG00000057606	Colq	430,9125218	29,77161006	170,2284535	10,10370137	36,77918713	-1,339922819	True	1,339922819
ENSMUSG00000031613	Hpgd	9185,154361	7716,609252	10207,11772	18998,32647	3629,275272	-1,339622481	True	1,339622481
ENSMUSG00000037010	Apln	938,8322242	48,97910041	371,2078535	33,67900456	34,40633635	-1,338640128	True	1,338640128
ENSMUSG00000021091	Serpina3n	1843,256985	24650,89313	4657,670139	15706,76509	35096,83592	1,337351246	True	1,337351246
ENSMUSG00000031367	Ap1s2	747,9527231	953,6518963	1211,36764	1200,095196	298,9791986	-1,32290197	True	1,32290197
ENSMUSG00000061100	Retnla	363,736045	2329,868581	908,2511683	1460,546164	2733,524101	1,320199417	True	1,320199417
ENSMUSG00000017446	C1qtnf1	308,8479481	426,406286	124,1020339	308,7242084	450,8416487	-1,315369986	True	1,315369986
ENSMUSG00000030017	Reg3g	32,76901306	4593,471319	13,17897705	264,9415025	34,40633635	-1,31409383	True	1,31409383
ENSMUSG00000042474	Fcmr	1078,919755	48,0187259	437,1027388	62,86747517	13,0506793	-1,303543245	True	1,303543245
ENSMUSG00000026043	Col3a1	5013,658998	615,600066	2061,41166	1725,487667	1515,065225	-1,282231238	True	1,282231238
ENSMUSG00000094686	Ccl21a	1850,630013	320,765089	764,3806688	158,2913214	486,4344104	-1,275653295	True	1,275653295
ENSMUSG00000064057	Scgb3a1	831,5137064	17633,43652	932,4126262	2505,717939	344,0633635	-1,273065765	True	1,273065765
ENSMUSG00000003882	Il7r	252,3214006	523,4041123	185,6039268	453,543928	104,4054344	-1,273065765	True	1,273065765
ENSMUSG00000025743	Sdc3	2768,162378	754,8543711	1145,472755	983,4269331	872,0226626	-1,272985428	True	1,272985428
ENSMUSG00000057897	Camk2b	180,2295718	361,1008187	74,68086994	277,2904709	237,2850783	-1,271025108	True	1,271025108
ENSMUSG00000059824	Dbp	379,3013262	915,2369156	1222,350121	1185,50096	1257,610915	1,270800828	True	1,270800828
ENSMUSG00000063531	Sema3e	1522,120657	558,9379694	631,4926503	620,8163173	628,8054574	-1,269244876	True	1,269244876
ENSMUSG00000054855	Rnd1	430,9125218	625,2038112	179,0144382	316,5826428	1468,794634	-1,26731907	True	1,26731907
ENSMUSG00000026544	Dusp23	340,7977358	371,6649384	563,4012688	685,9290595	142,371047	-1,259259966	True	1,259259966
ENSMUSG00000002944	Cd36	15042,61545	4171,866906	6294,059789	4814,975018	3758,595639	-1,256992641	True	1,256992641
ENSMUSG00000021390	Ogn	5810,765241	628,0849347	2443,601995	964,3421638	1554,217263	-1,249718847	True	1,249718847
ENSMUSG00000045954	Cavin2	9260,523091	1649,923422	3917,450928	2402,435658	1153,20548	-1,241178485	True	1,241178485
ENSMUSG00000035258	Abi3bp	1486,893968	227,6087608	630,3944022	467,0155299	378,4696998	-1,237975142	True	1,237975142
ENSMUSG00000056069	Fam105a	397,3242834	450,4156489	499,7028798	641,0237201	168,4724056	-1,237804661	True	1,237804661
ENSMUSG00000029772	Ahcyl2	1356,637141	386,0705562	575,4819978	361,4879822	348,809065	-1,237192191	True	1,237192191
ENSMUSG00000022504	Ciita	799,5639187	55,70172204	339,358659	63,99010866	21,35565704	-1,236402545	True	1,236402545
ENSMUSG00000020836	Coro6	142,5452068	489,7910041	334,9656667	872,286218	348,809065	1,232593701	True	1,232593701
ENSMUSG00000034936	Arl4d	638,9957547	1967,807387	1501,305136	2355,285052	2428,612776	1,232338978	True	1,232338978
ENSMUSG00000024803	Ankrd1	213,8178102	420,6440389	91,15459126	284,0262718	677,4488984	-1,229994801	True	1,229994801
ENSMUSG00000043110	Lrrn4	425,9971698	6,722621626	118,6107934	181,8666246	34,40633635	-1,227963036	True	1,227963036
ENSMUSG00000004655	Aqp1	14085,76026	16721,08073	10089,60518	6084,67349	26002,8853	-1,21098568	True	1,21098568
ENSMUSG00000019370	Calm3	5909,891506	1206,230395	2555,623299	1084,463947	1488,963866	-1,209456448	True	1,209456448
ENSMUSG00000035283	Adrb1	1195,249751	338,0518303	517,2748492	317,7052763	339,3176619	-1,208309154	True	1,208309154
ENSMUSG00000031283	Chrdl1	339,9785105	41,29610427	148,2634918	108,8954481	67,6262473	-1,197280165	True	1,197280165
ENSMUSG00000079018	Ly6c1	4913,713509	31109,41176	11241,66742	32300,41064	22682,08063	1,19397039	True	1,19397039
ENSMUSG00000026971	Itgb6	947,0244775	1870,809561	414,039529	661,2311228	1204,221772	-1,193633205	True	1,193633205
ENSMUSG00000019577	Pdk4	97,48781386	1089,064703	42,83167541	1109,161883	1516,25165	-1,18654378	True	1,18654378
ENSMUSG00000001670	Tat	18,84218251	216,0842665	42,83167541	592,7504802	887,4461927	1,184712027	True	1,184712027
ENSMUSG00000052336	Cx3cr1	570,1808273	16,3263668	251,498812	59,49957472	3,559276174	-1,180867943	True	1,180867943
ENSMUSG00000022018	Rgcc	906,8824365	7569,67195	2053,723923	7547,464921	6470,764084	1,179254813	True	1,179254813
ENSMUSG00000025270	Alas2	2208,63148	941,1670276	886,2862065	987,917467	602,7040988	-1,160690293	True	1,160690293
ENSMUSG00000022996	Wnt10b	418,6241419	57,62247108	187,8004229	123,4896834	33,21991096	-1,156455201	True	1,156455201
ENSMUSG00000032053	Pou2af1	384,2166781	70,10733981	172,4249497	58,37694123	26,10135861	-1,155951599	True	1,155951599
ENSMUSG00000003032	Klf4	1749,865297	5423,234903	3883,405237	4829,569254	7519,56413	1,150078392	True	1,150078392
ENSMUSG00000057751	Megf6	1165,75764	912,355792	1425,526017	2379,982989	525,5864483	-1,149267901	True	1,149267901
ENSMUSG00000028150	Rorc	330,9670319	777,9033595	733,6297224	990,162734	1019,139411	1,148364574	True	1,148364574
ENSMUSG00000026494	Kif26b	338,3400599	113,3241931	152,6564842	94,30121276	98,47330747	-1,148185134	True	1,148185134

ENSMUSG00000021922	Itih4	1294,376016	1838,156827	584,2679825	1271,943739	1509,133098	-1,147554643	True	1,147554643
ENSMUSG00000051748	Wfdc21	86,01865929	750,0524985	189,9969191	663,4763898	529,1457245	1,143254475	True	1,143254475
ENSMUSG00000022376	Adcy8	552,1578701	133,492058	251,498812	172,8855567	196,9466149	-1,134529228	True	1,134529228
ENSMUSG00000021186	Fbln5	2596,12506	461,9401431	1183,911438	444,5628602	798,4642883	-1,132798717	True	1,132798717
ENSMUSG00000010051	Hyal1	277,7173857	161,342919	552,418788	537,7414394	126,9475169	-1,12938532	True	1,12938532
ENSMUSG00000032332	Col12a1	736,4835686	306,3594712	286,6427508	165,0271223	336,9448111	-1,128141029	True	1,128141029
ENSMUSG00000006717	Acot13	303,1133708	243,9351276	515,078353	425,4780909	139,9981962	-1,114449254	True	1,114449254
ENSMUSG00000070348	Ccnd1	4305,029091	1132,281557	1990,025534	635,4105526	1095,070636	-1,113236044	True	1,113236044
ENSMUSG00000025785	Exosc7	666,8494158	425,4459114	1082,872614	917,1915574	310,8434525	-1,101172826	True	1,101172826
ENSMUSG00000033763	Mtss1l	1319,772001	309,2405948	618,3136732	327,8089777	367,7918713	-1,093877902	True	1,093877902
ENSMUSG00000036036	Zfp57	420,2625925	501,3154984	826,9808098	849,8335483	196,9466149	-1,093486428	True	1,093486428
ENSMUSG00000020917	Acly	2836,15808	1206,230395	1308,013472	1055,275476	1329,982864	-1,09253029	True	1,09253029
ENSMUSG00000078921	Tgtp2	408,7934379	151,7391738	193,2916634	126,8575838	17,79638087	-1,080592622	True	1,080592622
ENSMUSG00000054641	Mmm1	465,3199855	212,2427685	195,4881596	220,0361631	186,2687864	-1,080482501	True	1,080482501
ENSMUSG00000042115	Klhdc8a	192,5179517	554,1360968	230,6320984	408,6385886	91,35475513	-1,075441249	True	1,075441249
ENSMUSG00000001496	Nkx2-1	3163,848211	3590,840323	4714,779039	5006,945344	1502,014545	-1,074781603	True	1,074781603
ENSMUSG00000045763	Basp1	339,9785105	567,5813401	161,4424689	263,818869	495,9258135	-1,074423417	True	1,074423417
ENSMUSG00000020520	Galnt10	327,6901306	65,30546722	155,9512284	84,19751139	39,15203791	-1,071237306	True	1,071237306
ENSMUSG00000007476	Lrrc8a	328,5093559	1267,694364	689,6997989	1060,888644	1671,673376	1,070034087	True	1,070034087
ENSMUSG00000016918	Sulf1	650,4649093	123,8883128	255,8918044	309,8468419	217,1158466	-1,069915966	True	1,069915966
ENSMUSG00000074896	Ifit3	1095,304262	523,4041123	2573,195269	1392,065522	804,3964153	-1,065334523	True	1,065334523
ENSMUSG00000040710	St8sia4	678,3185704	120,0468147	325,0814339	161,6592219	98,47330747	-1,061161827	True	1,061161827
ENSMUSG00000002489	Tiam1	620,9727975	297,7161006	291,0357432	231,262498	264,5728623	-1,060592826	True	1,060592826
ENSMUSG00000056313	Tcim	613,5997696	154,6202974	1277,262526	148,1876201	183,8959356	1,057685238	True	1,057685238
ENSMUSG00000001240	Ramp2	11511,75429	11944,17788	26279,97848	28430,69301	5538,233726	-1,055609859	True	1,055609859
ENSMUSG00000005125	Ndrg1	824,9599038	1486,659754	608,4294404	397,4122538	2472,510515	-1,053687643	True	1,053687643
ENSMUSG00000050931	Sgms2	195,794853	266,0237415	94,44933552	171,7629232	391,5203791	-1,051730289	True	1,051730289
ENSMUSG00000029198	Grpel1	412,0703392	529,1663594	198,7829038	314,3373759	949,140313	-1,051696938	True	1,051696938
ENSMUSG00000031779	Ccl22	213,8178102	242,974753	103,2353202	168,3950228	466,2651788	-1,05044538	True	1,05044538
ENSMUSG00000029669	Tspan12	914,2554644	334,2103322	441,4957311	423,2328239	205,2515927	-1,050197856	True	1,050197856
ENSMUSG00000035042	Ccl5	1102,67729	313,0820928	428,3167541	244,7340998	532,7050007	-1,049601903	True	1,049601903
ENSMUSG00000034765	Dusp5	236,7561194	175,7485368	151,5582361	114,5086155	606,2633749	-1,047945566	True	1,047945566
ENSMUSG00000033855	Ston1	412,0703392	138,2939306	110,9230568	199,8287604	78,30407582	-1,044126384	True	1,044126384
ENSMUSG00000051457	Spn	662,7532892	321,7254635	282,2497585	182,9892581	313,2163033	-1,0426418	True	1,0426418
ENSMUSG00000019889	Ptprk	1512,289953	1998,539372	736,9244667	1435,848228	2281,496027	-1,037146117	True	1,037146117
ENSMUSG00000007279	Scube2	720,099062	125,8090618	352,5376361	130,2254843	276,4371162	-1,030418103	True	1,030418103
ENSMUSG00000038801	Scgb1c1	283,451963	232,4106333	250,4005639	139,2065522	523,2135975	-1,025877143	True	1,025877143
ENSMUSG00000033124	Atg9a	435,8278737	556,0568459	214,158377	434,4591588	845,921304	-1,025080357	True	1,025080357
ENSMUSG00000037243	Zfp692	358,820693	176,7089113	644,6716273	429,9686248	186,2687864	-1,021888296	True	1,021888296
ENSMUSG00000026678	Rgs5	361,278369	246,8162511	330,5726743	731,9570324	79,49050121	1,018648083	True	1,018648083
ENSMUSG00000067279	Ppp1r3c	358,0014677	39,37535524	176,8179421	38,1695385	29,66063478	-1,017700827	True	1,017700827
ENSMUSG00000071637	Cebpd	897,0517326	11674,31264	1815,404089	6176,729436	18662,4714	1,017027619	True	1,017027619
ENSMUSG00000071042	Rasgrp3	1733,480791	364,9423168	856,6335082	235,7530319	342,8769381	-1,016921834	True	1,016921834
ENSMUSG00000032006	Pdgfd	355,5437917	49,93947493	175,719694	95,42384625	30,84706017	-1,016751373	True	1,016751373
ENSMUSG00000042606	Hirip3	201,5294303	211,2823939	265,7760372	291,8847062	99,65973287	-1,015907926	True	1,015907926
ENSMUSG00000012640	Zfp715	429,2740711	413,9214172	459,0677005	652,2500549	212,370145	-1,015318068	True	1,015318068
ENSMUSG00000055254	Ntrk2	365,3744956	121,0071893	735,8262186	21,33003622	188,6416372	1,00998915	True	1,00998915
ENSMUSG00000038508	Gdf15	18,02295718	214,1635175	36,24218688	67,35800911	2433,358477	1,007834265	True	1,007834265
ENSMUSG00000043099	Hic1	448,1162536	620,4019386	616,117177	223,4040636	945,5810368	-1,004217628	True	1,004217628
ENSMUSG00000032561	Acpp	160,568164	360,1404442	80,17211038	209,9324617	386,7746775	-1,002013521	True	1,002013521
ENSMUSG00000048572	Tmem252	154,8335867	2029,271356	309,7059607	801,5603085	3548,598345	1,000180693	True	1,000180693
ENSMUSG00000041538	H2-Ob	938,0129989	72,98846336	468,9519333	76,339077	67,6262473	-1,000167859	True	1,000167859
ENSMUSG00000061535	C1qtnf7	697,1607529	596,3925756	911,5459126	1129,369286	348,809065	-0,99905382	False	0,99905382
ENSMUSG00000001627	Ifrd1	774,9871589	2645,831797	1548,529803	3436,381098	2120,142174	0,998654839	False	0,998654839
ENSMUSG00000039109	F13a1	533,3156876	629,0453092	1005,995248	647,759521	266,945713	-0,998443385	False	0,998443385
ENSMUSG00000018339	Gpx3	37518,8815	95834,81277	92161,68475	74879,65347	77875,77626	0,996956939	False	0,996956939
ENSMUSG00000066361	Serpina3c	235,936894	1383,89968	118,6107934	359,2427153	3692,155818	-0,992165735	False	0,992165735
ENSMUSG00000060032	H2afj	901,9670845	2182,931279	1793,439127	2065,645613	2379,969335	0,991582087	False	0,991582087
ENSMUSG00000039114	Nrn1	431,7317471	54,74134752	217,4531213	153,8007875	21,35565704	-0,989430767	False	0,989430767
ENSMUSG00000023913	Pla2g7	340,7977358	1659,527167	675,4225738	1094,567648	1581,505047	0,986874647	False	0,986874647
ENSMUSG00000022893	Adamts1	944,5668015	1526,035109	477,737918	517,5340367	3533,174815	-0,983433442	False	0,983433442
ENSMUSG00000073940	Hbb-bt	944,5668015	478,2665099	356,9306284	382,8180185	277,6235416	-0,981838058	False	0,981838058
ENSMUSG00000033318	Gstt2	365,3744956	413,9214172	460,1659486	592,7504802	185,082361	-0,981208524	False	0,981208524
ENSMUSG00000026622	Nek2	208,0832329	933,4840314	105,4318164	365,9785162	713,0416601	-0,980850421	False	0,980850421
ENSMUSG00000041695	Kcnj2	235,936894	602,1548227	483,2291585	630,9200187	465,0787534	0,979073999	False	0,979073999
ENSMUSG00000026458	Ppfia4	213,8178102	284,2708573	115,3160492	108,8954481	850,6670055	-0,97343838	False	0,97343838
ENSMUSG00000025582	Nptx1	499,7274492	69,14696529	254,7935563	53,88640729	42,71131409	-0,971812673	False	0,971812673
ENSMUSG00000030895	Hpx	286,7288643	2462,400264	562,3030208	1723,2424	842,3620278	0,971660653	False	0,971660653
ENSMUSG00000023249	Parp3	1090,38891	962,295267	1621,014177	1533,517341	556,4335085	-0,970561587	False	0,970561587
ENSMUSG00000030474	Siglece	283,451963	464,8212667	295,4287355	529,883005	144,7438977	-0,969601733	False	0,969601733
ENSMUSG00000042082	Arsb	958,493632	332,2895832	476,6396699	376,0822176	491,180112	-0,9645167	False	0,9645167
ENSMUSG00000034981	Parm1	665,2109651	824,0013364	543,6328033	341,2805795	1612,352107	-0,962853629	False	0,962853629
ENSMUSG00000030790	Adm	190,0602758	370,7045639	97,74407978	166,1497558	1679,978354	-0,9593758	False	0,9593758
ENSMUSG00000028195	Cyr61	3049,975891	3150,988793	1143,276259	1187,746227	5921,449128	0,957152444	False	0,957152444
ENSMUSG00000014453	Blk	362,0975943	80,67145951	186,7021749	77,46171048	54,575568	-0,955639859	False	0,955639859
ENSMUSG00000024208	Uqcc2	264,6097805	315,9632164	420,6290175	258,2057016	136,43892	-0,955611147	False	0,955611147
ENSMUSG00000024673	Ms4a1	732,3874419	24,00936295	377,7973421	47,15060638	0	-0,954994502	False	0,954994502
ENSMUSG00000044258	Ctla2a	841,3444104	5404,987787	1627,603666	2706,669333	7852,949665	0,951981031	False	0,951981031
ENSMUSG00000056666	Retsat	376,0244249	145,0165522	194,3899115	107,7728146	143,5574723	-0,951873029	False	0,951873029
ENSMUSG00000029068	Ccnl2	1686,784947	5286,861721	3253,010835	6410,237201	6273,817469	0,947499575	False	0,947499575
ENSMUSG00000060572	Mfap2	1531,132135	1331,079082	2351,349155	2961,507134	794,9050121	-0,945744412	False	0,945744412
ENSMUSG00000045667	Smtnl2	403,0588607	892,1879272	773,1666535	806,0508424	828,1249231	0,939788878	False	0,939788878
ENSMUSG00000022122	Ednrb	1339,433409	1308,990468	1633,094906	698,2780278	1894,72135	-0,939749376	False	0,939749376
ENSMUSG00000021614	Vcan	145,8221081	638,6490544	353,6358841	279,5357378	465,0787534	0,938823276	False	0,938823276
ENSMUSG00000021775	Nr1d2	637,357304	1972,60926	1221,251873	2249,757504	1790,315915	0,938186492	False	0,938186492
ENSMUSG00000040447	Spns2	2918,899838	1087,143954	1527,66309	1101,303449	857,7855579	-0,934098299	False	0,934098299
ENSMUSG00000040557	Mettl27	242,4906967	242,974753	274,5620219	497,326634	126,9475169	-0,933697223	False	0,933697223
ENSMUSG00000026840	Lamc3	180,2295718	641,530178	437,1027388	951,9931955	344,0633635	0,932838533	False	0,932838533
ENSMUSG00000020893	Per1	828,2368051	7244,104989	1579,28075	5460,489272	18075,19084	0,931152443	False	0,931152443
ENSMUSG00000092274	Neat1	19125,63447	2891,687673	36438,77329	6707,735074	1437,947574	0,929966783	False	0,929966783
ENSMUSG00000054675	Tmem119	681,5954717	382,2290581	1298,129239	251,4699007	467,4516042	0,929446367	False	0,929446367
ENSMUSG00000027380	Acoxl	1105,954191	283,3104828	582,0714863	406,3933217	199,3194657	-0,926023378	False	0,926023378
ENSMUSG00000022272	Myo10	1495,905446	600,2340737	655,6541082	424,3554574	787,7864598	-0,925142462	False	0,925142462
ENSMUSG00000023007	Prpf40b	847,0789876	668,4206645	1148,767499	1192,236761	446,0959471	-0,925142462	False	0,925142462
ENSMUSG00000064267	Hvcn1	825,7791291	226,6483862	434,9062426	257,0830681	399,8253569	-0,925051539	False	0,925051539
ENSMUSG00000008398	Elk3	2995,907019	341,8933284	1578,182502	736,4475663	218,302272	-0,9247288	False	0,9247288
ENSMUSG00000071001	Hrct1	104,8608418	603,1151973	198,7829038	387,3085524	465,0787534	0,922717652	False	0,922717652
ENSMUSG00000018378	Cuedc1	817,5868759	860,4955681	1085,06911	1424,621893	431,8588424	-0,920812208	False	0,920812208
ENSMUSG00000042622	Maff	288,3673149	2120,506936	544,7310514	1229,283666	3259,11055	0,917636456	False	0,917636456
ENSMUSG00000039759	Thap3	439,104775	293,8746025	560,1065246	792,5792406	232,5393767	-0,91709019	False	0,91709019
ENSMUSG00000037762	Slc16a9	1980,88684	1631,676306	1543,038563	3738,369506	478,1294327	0,916262707	False	0,916262707
ENSMUSG00000058818	Pirb	1217,368835	1657,606418	1015,879481	2103,815151	645,4154128	-0,915466403	False	0,915466403
ENSMUSG00000017493	Igfbp4	3674,22559	3635,977925	1389,283831	2161,069459	6928,724285	0,915149541	False	0,915149541
ENSMUSG00000024787	Snx15	337,5208345	303,4783477	551,3205399	328,9316112	179,1502341	-0,913806631	False	0,913806631
ENSMUSG00000059108	Ifitm6	352,2668904	4490,711246	663,3418448	3536,295479	1506,760247	0,913083654	False	0,913083654
ENSMUSG00000064280	Ccdc146	303,9325961	76,82996143	161,4424689	151,5555205	66,43982191	-0,912731267	False	0,912731267
ENSMUSG00000040151	Hs2st1	2519,117879	603,1151973	1339,862667	749,9191681	722,5330633	-0,910833498	False	0,910833498
ENSMUSG00000050737	Ptges	554,6155461	799,0315989	516,1766011	295,2526066	666,7710699	-0,90953826	False	0,90953826
ENSMUSG00000055148	Klf2	3286,73201	8465,701376	7497,739693	6164,380467	13762,53454	0,90730208	False	0,90730208
ENSMUSG00000014773	Dll1	364,5552703	1769,970237	992,816271	683,6837925	2592,33948	0,907191667	False	0,907191667

ENSMUSG00000034641	Cd300ld	448,1162536	145,0165522	239,4180831	161,6592219	67,6262473	-0,904340933	False	0,904340933
ENSMUSG00000026580	Selp	113,0530951	930,6029079	60,40364481	188,6024255	1112,867017	-0,904292979	False	0,904292979
ENSMUSG00000046223	Plaur	676,6801197	7190,324016	1264,083549	2491,123704	15983,52287	0,901545911	False	0,901545911
ENSMUSG00000031431	Tsc22d3	1288,641439	7149,027911	2404,065063	6735,800911	7845,831112	0,899625048	False	0,899625048
ENSMUSG00000029155	Spata18	398,962734	183,4315329	215,2566251	154,923421	147,1167485	-0,890196353	False	0,890196353
ENSMUSG00000078670	Fam174b	3658,660308	944,0481511	1975,748309	922,8047249	1082,019957	-0,8889163	False	0,8889163
ENSMUSG00000037922	Bank1	355,5437917	20,16786488	192,1934153	32,55637107	3,559276174	-0,887468356	False	0,887468356
ENSMUSG00000030521	Mphosph10	176,9526705	345,7348265	327,2779301	352,5069144	383,2154014	0,887152783	False	0,887152783
ENSMUSG00000040855	Reps2	800,383144	134,4524325	433,8079945	188,6024255	145,9303231	-0,883634144	False	0,883634144
ENSMUSG00000028621	Cyb5rl	246,5868233	294,834977	281,1515104	403,0254212	134,0660692	-0,879151558	False	0,879151558
ENSMUSG00000009376	Met	949,4821535	427,3666605	517,2748492	499,5719009	481,6887088	-0,876209837	False	0,876209837
ENSMUSG00000005973	Rcn1	880,667226	243,9351276	450,2817158	480,4871317	282,3692431	-0,874099184	False	0,874099184
ENSMUSG00000034459	Ifit1	444,8393523	242,974753	666,6365891	607,3447155	387,9611029	-0,872478006	False	0,872478006
ENSMUSG00000043439	Epop	118,7876723	440,8119037	217,4531213	391,7990864	797,2778629	0,872319295	False	0,872319295
ENSMUSG00000028766	Alpl	103,2223911	3126,019056	188,898671	389,5538194	5611,792101	0,871856596	False	0,871856596
ENSMUSG00000043953	Ccrl2	552,1578701	630,0056838	461,2641967	301,9884075	1166,25616	-0,870587644	False	0,870587644
ENSMUSG00000027009	Itga4	1315,675874	720,2808884	706,1735202	434,4591588	558,8063593	-0,869172585	False	0,869172585
ENSMUSG00000038264	Sema7a	1369,744746	9007,352604	2498,514399	9335,820063	5423,150463	0,867163463	False	0,867163463
ENSMUSG00000026311	Asb1	489,0775199	233,3710079	264,6777891	194,2155929	268,1321384	-0,867119002	False	0,867119002
ENSMUSG00000014030	Pax5	634,8996281	102,7600734	348,1446437	55,00904078	80,67692661	-0,866841705	False	0,866841705
ENSMUSG00000024421	Lama3	1786,730437	660,7376683	672,1278295	662,3537563	979,9873732	-0,866486927	False	0,866486927
ENSMUSG00000028862	Map3k6	387,4935795	8785,50609	706,1735202	6834,592658	11566,46114	0,865850319	False	0,865850319
ENSMUSG00000039221	Rpl22l1	156,4720374	343,8140774	284,4462546	350,2616474	391,5203791	0,862251224	False	0,862251224
ENSMUSG00000078161	Erich3	475,1506894	890,2671781	261,3830448	610,712616	607,4498003	-0,862219562	False	0,862219562
ENSMUSG00000068735	Trp53i11	1011,743278	1309,950842	665,538341	556,8262087	2815,387453	-0,861544242	False	0,861544242
ENSMUSG00000029636	Wasf3	285,909639	157,5014209	132,8880186	148,1876201	96,10045669	-0,860194414	False	0,860194414
ENSMUSG00000040584	Abcb1a	634,8996281	231,4502588	348,1446437	350,2616474	196,9466149	-0,858095507	False	0,858095507
ENSMUSG00000061878	Sphk1	459,5854082	4997,788991	832,4720503	3123,166356	9184,118954	0,857068842	False	0,857068842
ENSMUSG00000024754	Tmem2	2109,505216	881,6238075	1165,241221	889,1257203	775,9222059	-0,856276011	False	0,856276011
ENSMUSG00000044033	Ccdc141	571,8192779	502,2758729	743,5139552	1070,992345	316,7755795	-0,852098138	False	0,852098138
ENSMUSG00000026691	Fmo3	1220,645737	1785,336229	1154,25874	1851,222617	676,262473	-0,85198935	False	0,85198935
ENSMUSG00000079293	Clec7a	728,2913153	142,1354287	404,1552962	160,5365884	195,7601896	-0,849605888	False	0,849605888
ENSMUSG00000022358	Fbxo32	391,5897061	729,8846336	217,4531213	772,3718378	938,4624845	-0,848638423	False	0,848638423
ENSMUSG00000032035	Ets1	3873,297344	1911,145291	1974,650061	1614,346952	2153,362085	-0,846971331	False	0,846971331
ENSMUSG00000021795	Sftpd	7973,520103	28544,25142	14334,33404	26743,37489	34839,38161	0,846186197	False	0,846186197
ENSMUSG00000046157	Tmem229b	550,5194194	114,2845676	306,4112164	99,91438019	103,219009	-0,845324346	False	0,845324346
ENSMUSG00000028717	Tal1	402,2396353	414,8817917	582,0714863	471,5060638	224,2343989	-0,843047633	False	0,843047633
ENSMUSG00000028969	Cdk5	670,1263171	508,03812	1049,925172	765,6360369	373,7239982	-0,842459858	False	0,842459858
ENSMUSG00000005043	Sgsh	621,7920228	116,2053167	347,0463956	191,970326	85,42262817	-0,841303563	False	0,841303563
ENSMUSG00000019256	Ahr	1729,384664	379,3479346	966,4583169	633,1652857	301,3520494	-0,839479385	False	0,839479385
ENSMUSG00000028780	Sema3c	7748,233138	1595,182074	4332,588705	2431,624129	1472,353911	-0,838638077	False	0,838638077
ENSMUSG00000042745	Id1	874,9326487	2813,897338	1563,905276	1870,307386	3477,412822	0,837909264	False	0,837909264
ENSMUSG00000017754	Pltp	16094,50077	3116,415311	9008,929061	3749,595841	6411,442814	-0,837140306	False	0,837140306
ENSMUSG00000060227	Casc4	2219,28141	656,8961703	1244,315083	795,947141	676,262473	-0,834740768	False	0,834740768
ENSMUSG00000024511	Rab27b	362,0975943	96,03745179	203,1758962	134,7160182	80,67692661	-0,833649334	False	0,833649334
ENSMUSG00000050953	Gja1	580,0115312	430,247784	477,737918	325,5637107	1379,81273	-0,833141686	False	0,833141686
ENSMUSG00000052212	Cd177	324,4132293	2903,212168	577,678494	2942,422365	1480,658888	0,832434134	False	0,832434134
ENSMUSG00000042099	Kank3	1984,982966	1168,775788	2910,357432	3530,682311	238,4715036	0,830820387	False	0,830820387
ENSMUSG00000001942	Siae	407,9742126	107,561946	229,5338503	169,5176563	53,38914261	-0,829771035	False	0,829771035
ENSMUSG00000026692	Fmo4	112,2338697	286,1916063	275,6602699	456,9118285	199,3194657	0,828574496	False	0,828574496
ENSMUSG00000062960	Kdr	5472,425181	1427,116534	3081,684133	1407,78239	1198,289645	-0,828461329	False	0,828461329
ENSMUSG00000026657	Frmd4a	1610,596992	557,9775949	908,2511683	798,192408	436,604544	-0,826432323	False	0,826432323
ENSMUSG00000040717	Il17rd	903,6055352	249,6973747	509,5871126	405,2706882	195,7601896	-0,826364316	False	0,826364316
ENSMUSG00000050315	Synpo2	521,846533	220,8861391	294,3304874	166,1497558	219,4886974	-0,826188607	False	0,826188607
ENSMUSG00000057969	Sema3b	1049,427643	338,0518303	591,9557191	451,2986611	385,5882522	-0,826041533	False	0,826041533
ENSMUSG00000020400	Tnip1	652,1033599	1167,815414	367,9131093	517,5340367	2977,927732	-0,825735571	False	0,825735571
ENSMUSG00000001665	Gstt3	794,6485667	844,1692013	959,8688284	1018,228571	448,4687979	-0,825309356	False	0,825309356
ENSMUSG00000029047	Pex10	262,9713298	118,1260657	465,6571891	245,8567333	60,50769495	0,824362732	False	0,824362732
ENSMUSG00000032688	Malt1	554,6155461	768,2996143	472,2466776	313,2147424	2413,189246	-0,824335937	False	0,824335937
ENSMUSG00000027995	Tlr2	323,594004	358,2196952	202,0776481	182,9892581	1218,458877	-0,822425914	False	0,822425914
ENSMUSG00000034255	Arhgap27	1634,354526	1549,084097	1958,17634	2350,794518	924,2253798	-0,822404356	False	0,822404356
ENSMUSG00000020427	Igfbp3	2094,75916	6999,209487	1185,009686	2033,089242	10703,92988	-0,821885531	False	0,821885531
ENSMUSG00000004815	Dgkq	326,8709053	502,2758729	473,3449257	701,6459283	185,082361	-0,820553561	False	0,820553561
ENSMUSG00000000204	Slfn4	489,0775199	1381,978931	863,2229967	2089,220916	1483,031739	0,819670146	False	0,819670146
ENSMUSG00000047842	Diras2	439,9240004	226,6483862	249,3023158	154,923421	147,1167485	-0,819358026	False	0,819358026
ENSMUSG00000052305	Hbb-bs	19448,40925	11023,17872	9980,878618	10931,68225	7524,309831	-0,819111848	False	0,819111848
ENSMUSG00000039697	Ncoa7	298,1980189	112,3638186	169,1302055	142,5744526	78,30407582	-0,818136335	False	0,818136335
ENSMUSG00000026825	Dnm1	371,9282982	82,59220854	655,6541082	202,0740273	85,42262817	0,817910399	False	0,817910399
ENSMUSG00000050373	Snx21	872,4749728	725,082761	1143,276259	1482,998834	495,9258135	-0,814989426	False	0,814989426
ENSMUSG00000026034	Clk1	2548,609991	5247,486366	5229,857392	8118,885365	4479,942277	0,813769526	False	0,813769526
ENSMUSG00000028194	Ddah1	1007,647152	226,6483862	573,2855016	284,0262718	52,20271722	-0,813664839	False	0,813664839
ENSMUSG00000049577	Zfpm1	515,2927304	609,8378189	293,2322393	425,4780909	1017,952986	-0,813348511	False	0,813348511
ENSMUSG00000039976	Tbc1d16	553,7963207	292,914228	315,1972011	301,9884075	230,1665259	-0,813100745	False	0,813100745
ENSMUSG00000078920	Ifi47	794,6485667	316,9235909	452,478212	293,0073396	436,604544	-0,812468645	False	0,812468645
ENSMUSG00000029263	Pigg	308,0287228	122,9279383	175,719694	175,1308237	68,81267269	-0,809788992	False	0,809788992
ENSMUSG00000056054	S100a8	2941,838148	16727,80335	5156,27477	17898,14566	13238,13452	0,809611269	False	0,809611269
ENSMUSG00000044468	Fam46c	887,2210286	294,834977	506,2923683	361,4879822	233,7258021	-0,809322823	False	0,809322823
ENSMUSG00000067780	Pi15	138,4490802	696,2715255	79,07386229	124,6123169	842,3620278	-0,80808267	False	0,80808267
ENSMUSG00000026180	Cxcr2	641,4534307	796,1504754	509,5871126	992,408001	366,6054459	-0,807116313	False	0,807116313
ENSMUSG00000029781	Fkbp9	2253,688873	566,6209656	1288,245007	636,5331861	765,2443774	-0,806881362	False	0,806881362
ENSMUSG00000027303	Ptpra	1354,99869	718,3601394	775,3631497	718,4854306	723,7194887	-0,805347381	False	0,805347381
ENSMUSG00000020883	Fbxl20	1470,509461	1285,94148	1213,564137	2569,708048	494,7393882	0,805288393	False	0,805288393
ENSMUSG00000020275	Rel	339,1592852	316,9235909	300,919976	194,2155929	568,2977624	-0,804303955	False	0,804303955
ENSMUSG00000005514	Por	6050,798262	24669,14024	3466,070964	14834,47887	33541,43224	-0,803824293	False	0,803824293
ENSMUSG00000022546	Gpt	455,4892816	453,2967725	549,1240437	693,7874939	261,0135861	-0,803292203	False	0,803292203
ENSMUSG00000058656	Samd12	666,8494158	22,08861391	382,1903344	50,51850684	10,67782852	-0,803069724	False	0,803069724
ENSMUSG00000027360	Hdc	2397,872531	3056,872091	1375,006605	2478,774735	3899,780261	-0,802316419	False	0,802316419
ENSMUSG00000018931	Natd1	611,1420936	350,536699	334,9656667	308,7242084	297,7927732	-0,801942359	False	0,801942359
ENSMUSG00000018500	Adora2b	212,9985849	255,4596218	273,4637738	122,3670499	409,31676	-0,799628714	False	0,799628714
ENSMUSG00000020889	Nr1d1	3068,818073	3968,267508	1763,786428	2433,869396	10807,14889	-0,799007242	False	0,799007242
ENSMUSG00000038393	Txnip	12920,00262	19916,24675	7436,2378	19082,52398	41607,93847	-0,796961552	False	0,796961552
ENSMUSG00000029066	Mrpl20	249,8637246	756,7751201	433,8079945	501,8171679	819,8199454	0,795915175	False	0,795915175
ENSMUSG00000037225	Fgf2	416,9856912	738,5280043	344,8498994	240,2435658	603,8905241	-0,795500085	False	0,795500085
ENSMUSG00000052544	St6galnac3	432,5509724	199,7578997	231,7303464	249,2246337	98,47330747	-0,795423478	False	0,795423478
ENSMUSG00000020524	Gria1	258,8752032	134,4524325	449,1834678	237,9982989	166,0995548	0,795048049	False	0,795048049
ENSMUSG00000037053	Azgp1	153,1951361	498,4343748	331,6709224	660,1084893	265,7592876	0,794749619	False	0,794749619
ENSMUSG00000028859	Csf3r	3572,641649	1394,4638	2061,41166	1737,836635	1097,443487	-0,793358576	False	0,793358576
ENSMUSG00000020467	Efemp1	1690,881074	401,4365485	977,4407978	803,8055754	663,2117937	-0,790693965	False	0,790693965
ENSMUSG00000040451	Sgms1	1139,542429	1595,182074	658,9488525	1302,254843	2299,292408	-0,790216249	False	0,790216249
ENSMUSG00000021327	Zkscan3	1128,073275	760,6166182	1950,488603	1011,49277	564,7384862	0,789974787	False	0,789974787
ENSMUSG00000054008	Ndst1	9987,175956	3726,25313	5777,883188	4432,157	3865,373925	-0,789535752	False	0,789535752
ENSMUSG00000043671	Dpy19l3	419,4433672	70,10733981	242,7128273	134,7160182	103,219009	-0,789225676	False	0,789225676
ENSMUSG00000062729	Ppox	758,6026524	802,873097	1086,167358	1083,341313	438,9773948	-0,789197766	False	0,789197766
ENSMUSG00000026414	Tnnt2	613,5997696	1060,253468	577,678494	371,5916836	328,6398334	0,789039355	False	0,789039355
ENSMUSG00000012428	Steap4	390,7704808	534,9286065	226,239106	376,0822176	870,8362372	-0,788473164	False	0,788473164
ENSMUSG00000022505	Emp2	20524,87133	6510,378857	11883,04431	9467,168181	6295,173126	-0,788468692	False	0,788468692
ENSMUSG00000036282	Naa30	339,9785105	405,2780466	365,7166131	459,1570955	196,9466149	-0,787638938	False	0,787638938
ENSMUSG00000000303	Cdh1	4844,898581	5711,347258	4153,574267	2809,951614	9000,223018	-0,785921178	False	0,785921178
ENSMUSG00000036053	Fmnl2	594,7575871	3013,655237	1024,665466	2778,517876	1414,219066	0,784779297	False	0,784779297
ENSMUSG00000062488	Ifit3b	258,8752032	46,09797686	445,8887235	90,9333123	56,94841878	0,784426934	False	0,784426934

ENSMUSG00000030562	Nox4	1366,467845	881,6238075	1320,094201	2353,039785	303,7249002	0,784074203	False	0,784074203
ENSMUSG00000037548	H2-DMb2	743,0373712	42,25647879	431,6114984	69,60327609	24,91493322	-0,783701474	False	0,783701474
ENSMUSG00000027298	Tyro3	390,7704808	360,1404442	272,3655257	248,1020002	672,7031968	0,783648626	False	0,783648626
ENSMUSG00000019710	Mrpl24	1135,446303	975,7405102	1685,810814	1328,075413	659,6525175	-0,783481313	False	0,783481313
ENSMUSG00000024548	Setbp1	416,1664659	242,0143785	238,319835	177,3760907	224,2343989	-0,782067956	False	0,782067956
ENSMUSG00000029313	Aff1	1952,213953	1762,28724	1348,648651	1296,641675	3356,397432	0,781802382	False	0,781802382
ENSMUSG00000003518	Dusp3	3538,234185	1233,120881	2058,116916	1747,940337	669,1439207	-0,781704599	False	0,781704599
ENSMUSG00000040010	Slc7a5	173,6757692	373,5856875	101,038824	254,8378012	647,7882636	-0,781486734	False	0,781486734
ENSMUSG00000024925	Rnaseh2c	236,7561194	154,6202974	406,3517923	214,4229957	99,65973287	0,779327543	False	0,779327543
ENSMUSG00000017144	Rnd3	208,0832329	255,4596218	196,5864076	121,2444164	462,7059026	-0,77924241	False	0,77924241
ENSMUSG00000030789	Itgax	862,6442688	156,5410464	502,997624	212,1777287	395,0796553	-0,778214168	False	0,778214168
ENSMUSG00000020385	Clk4	878,20955	1505,867244	1528,761338	2262,106473	1594,555726	0,777957461	False	0,777957461
ENSMUSG00000047261	Gap43	397,3242834	21,12823939	231,7303464	28,06583713	39,15203791	-0,777869984	False	0,777869984
ENSMUSG00000025508	Rplp2	3376,846796	6980,962371	5788,865669	6686,405038	10839,18237	0,777603952	False	0,777603952
ENSMUSG00000056962	Jmjd6	450,5739296	1064,094966	817,096577	772,3718378	1653,876995	0,777531721	False	0,777531721
ENSMUSG00000024810	Il33	1100,219614	457,1382705	642,4751311	236,8756654	239,657929	-0,77607901	False	0,77607901
ENSMUSG00000027194	Ttc17	699,6184289	958,4537689	408,5482885	617,4484169	1289,6444	-0,776061684	False	0,776061684
ENSMUSG00000022123	Scel	1335,337282	412,9610427	463,4606929	780,2302722	263,3864369	-0,775232307	False	0,775232307
ENSMUSG00000026874	Hc	7736,763984	2111,863565	4522,585624	2171,17316	2605,390159	-0,774582447	False	0,774582447
ENSMUSG00000058709	Egln2	1088,750459	1722,911885	636,9838907	1168,661458	3331,482499	-0,773344535	False	0,773344535
ENSMUSG00000065990	Aurkaip1	769,2525816	1498,184248	1314,602961	1433,602961	2026,414568	0,773097854	False	0,773097854
ENSMUSG00000024737	Slc15a3	480,8852667	451,3760234	266,8742852	330,0542447	821,0063707	0,771700691	False	0,771700691
ENSMUSG00000050370	Ch25h	72,09182874	861,4559426	123,0037858	428,8459914	1258,79734	0,770795068	False	0,770795068
ENSMUSG00000044017	Adgrd1	203,167881	119,0864402	165,8354612	433,3365253	231,3529513	-0,770663193	False	0,770663193
ENSMUSG00000032212	Sltm	824,1406785	1701,783646	1405,757552	2198,116364	2184,209145	0,77038527	False	0,77038527
ENSMUSG00000066152	Slc31a2	534,1349129	606,9566953	655,6541082	715,1175301	313,2163033	-0,770044878	False	0,770044878
ENSMUSG00000071715	Ncf4	262,1521045	767,3392398	446,9869716	552,3356747	551,6878069	0,769828654	False	0,769828654
ENSMUSG00000030811	Fbxl19	523,4849837	610,7981934	700,6822798	568,0525435	307,2841763	-0,768574677	False	0,768574677
ENSMUSG00000078201	Tmem203	190,8795011	378,3875601	325,0814339	377,204851	405,7574838	0,768138986	False	0,768138986
ENSMUSG00000031980	Agt	51,61119557	571,4228382	87,85984699	468,1381633	665,5846445	0,767519936	False	0,767519936
ENSMUSG00000079435	Rpl36a	1266,522355	2357,719442	2154,762748	2467,548401	2645,728623	0,766656487	False	0,766656487
ENSMUSG00000055003	Lrtm2	417,8049165	628,0849347	479,9344142	1200,095196	245,590056	-0,766577323	False	0,766577323
ENSMUSG00000033420	Antxr1	2417,533939	432,1685331	1421,133025	661,2311228	608,6362257	-0,766494539	False	0,766494539
ENSMUSG00000037960	Card19	714,3644847	1786,296603	1214,662385	1524,536273	2492,679747	0,76582311	False	0,76582311
ENSMUSG00000074115	Saa1	134,3529536	1283,060356	228,4356022	1256,22687	786,6000344	0,765759478	False	0,765759478
ENSMUSG00000016756	Cmah	619,3343469	168,0655406	364,618365	316,5826428	130,506793	-0,764331228	False	0,764331228
ENSMUSG00000038903	Ccdc68	276,078935	311,1613438	381,0920863	553,4583082	162,5402786	-0,764283542	False	0,764283542
ENSMUSG00000032010	Usp2	270,3443578	1332,039456	459,0677005	1187,746227	1207,781048	0,763908682	False	0,763908682
ENSMUSG00000040592	Cd79b	978,9742652	329,4084597	576,5802459	230,1398645	290,6742209	-0,763749526	False	0,763749526
ENSMUSG00000040181	Fmo1	7333,705123	5472,214003	8913,381477	12437,65638	1872,179267	0,762100501	False	0,762100501
ENSMUSG00000066687	Zbtb16	307,2094975	1321,475337	181,2109344	1699,667097	1880,484245	-0,761552807	False	0,761552807
ENSMUSG00000024887	Asah2	492,3544212	180,5504094	269,0707814	290,7620727	189,8280626	-0,759858119	False	0,759858119
ENSMUSG00000051853	Arf3	3075,371876	1557,727468	1816,502337	1304,50011	1741,672474	-0,759597651	False	0,759597651
ENSMUSG00000029718	Pcolce	2174,224017	749,092124	1286,04851	626,4294848	889,8190434	-0,75755553	False	0,75755553
ENSMUSG00000041096	Tspyl2	645,5495573	1754,604244	1090,560351	1774,88354	3000,469814	0,756469853	False	0,756469853
ENSMUSG00000026558	Uck2	434,1894231	581,0265833	390,9763191	257,0830681	1137,78195	-0,756089987	False	0,756089987
ENSMUSG00000020495	Smg8	246,5868233	193,9956526	160,3442208	132,4707513	416,4353123	0,7559967	False	0,7559967
ENSMUSG00000047084	Ngrn	346,5323131	346,695201	444,7904754	417,6196565	205,2515927	-0,755596469	False	0,755596469
ENSMUSG00000041609	Bicdl1	276,8981604	241,054004	172,4249497	175,1308237	467,4516042	0,75546154	False	0,75546154
ENSMUSG00000036093	Arl5a	1128,073275	545,4927262	632,5908984	587,1373128	669,1439207	-0,753472335	False	0,753472335
ENSMUSG00000051379	Flrt3	1196,068977	709,7167688	553,5170361	580,4015119	353,5547666	-0,752985293	False	0,752985293
ENSMUSG00000000805	Car4	3326,874051	860,4955681	5601,065246	2854,856953	834,0570501	0,751533984	False	0,751533984
ENSMUSG00000029763	Exoc4	1031,404686	418,7232898	612,8224328	580,4015119	433,0452678	-0,751069491	False	0,751069491
ENSMUSG00000006567	Atp7b	726,6528646	1131,321182	794,0333672	1499,838336	431,8588424	-0,750706497	False	0,750706497
ENSMUSG00000035638	Muc20	326,8709053	163,263668	194,3899115	131,3481178	131,6932184	-0,749767622	False	0,749767622
ENSMUSG00000023132	Gzma	489,0775199	53,780973	291,0357432	52,76377381	66,43982191	-0,748866807	False	0,748866807
ENSMUSG00000022346	Myc	129,4376016	837,4465796	217,4531213	312,0921089	1978,957553	0,748447637	False	0,748447637
ENSMUSG00000048234	Rnf149	287,5480896	298,6764751	171,3267016	195,3382264	907,6154243	-0,747053237	False	0,747053237
ENSMUSG00000028497	Hacd4	497,2697732	74,9092124	296,5269836	132,4707513	37,96561252	-0,745865345	False	0,745865345
ENSMUSG00000024983	Vti1a	677,499345	377,4271855	404,1552962	390,6764529	319,1484302	-0,745309798	False	0,745309798
ENSMUSG00000018001	Cyth3	3661,117984	1742,119376	1739,62497	2035,334509	2184,209145	-0,745173261	False	0,745173261
ENSMUSG00000033278	Ptprm	2429,822319	1067,936464	1038,942691	728,5891319	1449,811828	-0,744985156	False	0,744985156
ENSMUSG00000040532	Abhd11	425,9971698	1199,507773	713,8612568	960,9742634	1210,153899	0,74479986	False	0,74479986
ENSMUSG00000046618	Olfml2a	956,8551814	168,0655406	571,0890055	300,865774	143,5574723	-0,744584981	False	0,744584981
ENSMUSG00000047789	Slc38a9	512,8350544	606,9566953	549,1240437	977,8137656	306,0977509	-0,744502435	False	0,744502435
ENSMUSG00000020023	Tmcc3	2581,379004	5442,442393	1540,842067	2382,228256	10641,04933	-0,744422981	False	0,744422981
ENSMUSG00000074259	Gramd2	2089,843808	3019,417484	2760,995692	3823,689651	1248,119512	-0,743639038	False	0,743639038
ENSMUSG00000037300	Ttc13	534,1349129	648,2527996	714,9595049	828,5035121	319,1484302	-0,742976635	False	0,742976635
ENSMUSG00000029372	Ppbp	755,3257511	48,0187259	451,3799639	182,9892581	86,60905356	-0,742756592	False	0,742756592
ENSMUSG00000071984	Fndc1	343,2554118	171,9070387	205,3723923	106,6501811	182,7095103	-0,741040209	False	0,741040209
ENSMUSG00000028445	Enho	245,767598	605,0359463	410,7447847	497,326634	670,3303461	0,740947534	False	0,740947534
ENSMUSG00000024924	Vldlr	675,8608944	658,8169193	633,6891464	1047,417042	404,5710584	-0,740333223	False	0,740333223
ENSMUSG00000024302	Dtna	648,0072333	713,5582668	568,8925093	1014,860671	387,9611029	-0,740097902	False	0,740097902
ENSMUSG00000031765	Mt1	416,9856912	18881,9234	696,2892874	4491,656574	55083,35807	0,739688949	False	0,739688949
ENSMUSG00000001761	Smo	703,7145555	294,834977	421,7272656	379,450118	312,0298779	-0,738680056	False	0,738680056
ENSMUSG00000038894	Irs2	696,3415276	1561,568966	417,3342732	1069,869711	2508,103277	-0,738591658	False	0,738591658
ENSMUSG00000051506	Wdfy4	604,588291	219,9257646	362,4218688	190,8476925	212,370145	-0,738283027	False	0,738283027
ENSMUSG00000028657	Ppt1	2209,450706	922,9199117	1324,487193	838,6072135	913,5475513	-0,73825385	False	0,73825385
ENSMUSG00000029404	Arl6ip4	376,0244249	897,9501743	627,0996579	838,6072135	1058,291449	0,737868357	False	0,737868357
ENSMUSG00000040026	Saa3	145,0028828	8336,050816	241,6145792	8629,683601	19427,71578	0,736625928	False	0,736625928
ENSMUSG00000022307	Oxr1	1350,083338	767,3392398	808,3105923	751,0418016	810,3285422	-0,736469602	False	0,736469602
ENSMUSG00000021367	Edn1	832,3329318	1271,535862	990,6197749	499,5719009	1538,793732	-0,736468386	False	0,736468386
ENSMUSG00000003955	Fam162a	226,1061901	136,3731815	376,699094	174,0081902	112,7104122	0,736412074	False	0,736412074
ENSMUSG00000036158	Prickle1	2201,258452	266,984116	1322,290697	483,8550321	384,4018268	-0,735289165	False	0,735289165
ENSMUSG00000031075	Ano1	1401,694534	636,7283054	842,3562831	607,3447155	244,4036306	-0,734669512	False	0,734669512
ENSMUSG00000015126	Tsr3	186,7833745	423,5251624	310,8042087	392,9217198	532,7050007	0,734639993	False	0,734639993
ENSMUSG00000032238	Rora	669,3070918	1422,314661	1113,623561	1697,42183	1897,094201	0,734521433	False	0,734521433
ENSMUSG00000003617	Cp	6414,534307	11107,69167	3855,949035	9148,340271	10154,61492	-0,734258547	False	0,734258547
ENSMUSG00000006219	Fblim1	655,3802612	1400,226047	1090,560351	1090,077114	1122,35842	0,73402607	False	0,73402607
ENSMUSG00000028931	Kcnab2	310,4863988	169,0259152	186,7021749	120,1217829	145,9303231	-0,733791337	False	0,733791337
ENSMUSG00000031379	Pir	368,6513969	302,5179731	533,7485705	477,1192312	221,8615482	-0,732597575	False	0,732597575
ENSMUSG00000029625	Cpsf4	280,994287	605,0359463	466,7554372	533,2509055	798,4642883	0,732126029	False	0,732126029
ENSMUSG00000031340	Gabre	237,5753447	700,1130236	394,2710634	858,8146162	596,7719718	0,730802708	False	0,730802708
ENSMUSG00000032224	Fam81a	309,6671734	147,8976758	186,7021749	185,2345251	141,1846216	-0,729979723	False	0,729979723
ENSMUSG00000039646	Vasn	620,9727975	753,8939966	374,5025978	551,2130413	1203,035347	-0,729554345	False	0,729554345
ENSMUSG00000034880	Mrpl34	367,8321716	426,406286	676,5208219	500,6945344	221,8615482	-0,729388018	False	0,729388018
ENSMUSG00000008496	Pou2f2	1231,295666	1523,153985	979,637294	743,1833672	2044,210949	-0,728387113	False	0,728387113
ENSMUSG00000024283	Wac	1406,609886	1381,018557	869,8124852	1040,681241	2328,953043	0,72745929	False	0,72745929
ENSMUSG00000042677	Zc3h12a	274,4404844	635,7679309	165,8354612	253,7151677	3151,145839	-0,726740781	False	0,726740781
ENSMUSG00000021108	Prkch	1059,258347	1728,674132	640,278635	1568,318979	2072,685159	-0,726282722	False	0,726282722
ENSMUSG00000018425	Dhx40	719,2798367	1311,871591	434,9062426	844,2203809	2477,256217	-0,725848745	False	0,725848745
ENSMUSG00000020186	Csrp2	427,6356205	424,4855369	638,0821388	880,1446524	258,6407353	-0,725432515	False	0,725432515
ENSMUSG00000079003	Samd1	547,2425181	500,3551238	877,5002218	671,3348242	331,0126842	-0,725293824	False	0,725293824
ENSMUSG00000005615	Pcyt1a	1449,209603	847,0503248	877,5002218	831,8714126	809,1421168	-0,723794875	False	0,723794875
ENSMUSG00000022292	Rrm2b	500,5466745	191,1145291	303,1164721	245,8567333	81,863352	-0,723632348	False	0,723632348
ENSMUSG00000045680	Tcf21	1255,0532	760,6166182	2072,394141	510,7982358	1127,104122	0,72354989	False	0,72354989
ENSMUSG00000066880	Zfp617	271,9828084	349,5763245	309,7059607	383,940652	164,9131294	-0,721809202	False	0,721809202
ENSMUSG00000024906	Mus81	341,6169612	148,8580503	563,4012688	248,1020002	41,52488869	0,721783207	False	0,721783207
ENSMUSG00000044894	Uqcrq	648,8264586	1838,156827	1223,448369	1069,869711	1951,669769	0,721530558	False	0,721530558
ENSMUSG00000041134	Cyyr1	2388,041827	1449,205148	999,4057596	977,8137656	1289,6444	-0,72056627	False	0,72056627

ENSMUSG00000021366	Hivep1	1255,872426	1086,18358	597,4469596	757,7776025	2067,939457	0,719504029	False	0,719504029
ENSMUSG00000068329	Htra2	618,5151215	464,8212667	992,816271	926,1726253	376,096849	-0,717704647	False	0,717704647
ENSMUSG00000008193	Spib	539,8694902	25,93011198	328,3761781	56,13167426	10,67782852	-0,717261216	False	0,717261216
ENSMUSG00000020469	Myl7	287,5480896	472,5042628	194,3899115	48,27323987	39,15203791	0,716524092	False	0,716524092
ENSMUSG00000039953	Clstn1	4633,538447	2210,78214	2093,260855	1862,448952	2821,31958	-0,715744248	False	0,715744248
ENSMUSG00000029162	Khk	367,8321716	869,1389387	604,0364481	896,9841547	715,4145109	0,715587938	False	0,715587938
ENSMUSG00000017978	Cadps2	475,9699147	184,3919074	289,9374951	200,9513939	172,0316817	-0,715128469	False	0,715128469
ENSMUSG00000056185	Snx32	270,3443578	73,94883788	443,6922273	187,479792	15,42353009	0,714761033	False	0,714761033
ENSMUSG00000078794	Dact3	329,3285813	127,7298109	540,338059	187,479792	47,45701565	0,714334579	False	0,714334579
ENSMUSG00000023923	Tbc1d5	623,4304735	247,7766256	379,9938382	309,8468419	153,0488755	-0,714252651	False	0,714252651
ENSMUSG00000074211	Sdhaf1	289,1865403	604,0755718	474,4431738	573,665711	530,3321499	0,714234894	False	0,714234894
ENSMUSG00000032089	Il10ra	494,8120972	436,9704057	382,1903344	293,0073396	811,5149676	0,713736932	False	0,713736932
ENSMUSG00000022019	Tdrd3	451,3931549	435,0496566	632,5908984	636,5331861	275,2506908	-0,713638357	False	0,713638357
ENSMUSG00000032420	Nt5e	1188,695949	1828,553082	724,8437377	1161,925657	1531,67518	-0,713637825	False	0,713637825
ENSMUSG00000050335	Lgals3	2023,486557	7061,63383	3317,807472	4030,254212	6193,140542	0,713386907	False	0,713386907
ENSMUSG00000019487	Trip10	1143,638556	2322,185584	1874,709485	2472,038935	4011,304248	0,713035881	False	0,713035881
ENSMUSG00000049804	Armcx4	515,2927304	360,1404442	416,2360251	844,2203809	87,79547895	0,712227419	False	0,712227419
ENSMUSG00000038074	Fkbp14	203,167881	900,8312978	332,7691705	1048,539675	648,974689	0,711849438	False	0,711849438
ENSMUSG00000031697	Orc6	166,3027413	419,6836643	272,3655257	373,8369506	624,0597558	0,711732158	False	0,711732158
ENSMUSG00000027070	Lrp2	924,0861683	182,4711584	564,4995169	303,111041	213,5565704	-0,711055039	False	0,711055039
ENSMUSG00000049791	Fzd4	2324,142251	3545,70272	1420,034777	3102,958953	3147,586563	-0,710772111	False	0,710772111
ENSMUSG00000034673	Pbx2	1333,698832	1184,141781	1820,895329	1737,836635	815,0742438	-0,710429539	False	0,710429539
ENSMUSG00000039262	Prrc2b	4232,937262	2587,248951	2497,416151	2304,766545	2482,001919	-0,710240226	False	0,710240226
ENSMUSG00000052085	Dock8	1138,723204	533,968232	696,2892874	552,3356747	309,6570271	-0,709658373	False	0,709658373
ENSMUSG00000046711	Hmga1	393,2281567	644,4113015	240,5163311	331,1768781	1141,341226	-0,709231771	False	0,709231771
ENSMUSG00000059534	Uqcr10	725,8336393	1866,007688	1186,107934	1377,471286	2015,73674	0,708524473	False	0,708524473
ENSMUSG00000024892	Pcx	871,6557474	1106,351445	533,7485705	721,853331	1420,151193	-0,707598167	False	0,707598167
ENSMUSG00000028104	Polr3gl	728,2913153	613,679317	1243,216835	1316,849078	446,0959471	-0,7071616	False	0,7071616
ENSMUSG00000037855	Zfp365	381,7590022	119,0864402	233,9268426	145,9423531	84,23620278	-0,70660476	False	0,70660476
ENSMUSG00000039084	Chad	1598,308612	502,2758729	980,7355421	460,279729	832,8706247	-0,704609933	False	0,704609933
ENSMUSG00000024998	Plce1	592,2999111	212,2427685	363,5201169	360,3653488	281,1828177	-0,704292666	False	0,704292666
ENSMUSG00000026785	Pkn3	933,0976469	313,0820928	1518,877105	666,8442902	191,014488	0,702905174	False	0,702905174
ENSMUSG00000029193	Cckar	2172,585566	752,9336221	1335,469674	663,4763898	444,9095217	-0,702065782	False	0,702065782
ENSMUSG00000026496	Parp1	681,5954717	179,5900349	419,5307694	235,7530319	135,2524946	-0,700139125	False	0,700139125
ENSMUSG00000010607	Pigyl	251,5021752	424,4855369	408,5482885	539,9867064	653,7203906	0,69993573	False	0,69993573
ENSMUSG00000108414	Snhg1	116,3299964	374,546062	188,898671	371,5916836	522,0271722	0,6993894	False	0,6993894
ENSMUSG00000040693	Slco4c1	606,2267416	290,9934789	373,4043497	332,2995116	86,60905356	-0,69911876	False	0,69911876
ENSMUSG00000021025	Nfkbia	2322,503801	19271,83545	3770,285684	13441,29072	38109,16999	0,698992887	False	0,698992887
ENSMUSG00000060586	H2-Eb1	4127,257195	1028,561109	2163,548732	1203,463096	2542,509613	-0,698930115	False	0,698930115
ENSMUSG00000018845	Unc45b	444,020127	302,5179731	485,4256546	720,7306975	232,5393767	0,69883522	False	0,69883522
ENSMUSG00000055994	Nod2	146,6413335	490,7513787	309,7059607	237,9982989	255,0814591	0,698659452	False	0,698659452
ENSMUSG00000031146	Plp2	2297,107816	4214,123385	3719,766272	3971,877271	4544,009249	0,695393402	False	0,695393402
ENSMUSG00000029096	Htra3	445,6585776	474,4250119	652,3593639	551,2130413	275,2506908	-0,695192689	False	0,695192689
ENSMUSG00000020380	Rad50	490,7159706	254,4992473	230,6320984	303,111041	185,082361	-0,695041821	False	0,695041821
ENSMUSG00000024420	Zfp521	303,9325961	142,1354287	187,8004229	143,6970861	93,72760591	-0,694551097	False	0,694551097
ENSMUSG00000019738	Polr2i	312,9440747	751,9732475	506,2923683	573,665711	853,0398563	0,694065877	False	0,694065877
ENSMUSG00000026667	Uhmk1	1903,060434	680,9055332	1177,32195	743,1833672	629,9918828	-0,692812484	False	0,692812484
ENSMUSG00000043613	Mmp3	479,246816	1761,326866	296,5269836	983,4269331	2092,85439	-0,692605453	False	0,692605453
ENSMUSG00000025037	Maoa	966,6858853	249,6973747	598,5452076	294,1299731	220,6751228	-0,69158696	False	0,69158696
ENSMUSG00000024006	Stk38	1747,407622	1913,06604	2154,762748	2869,451188	1083,206382	-0,689908044	False	0,689908044
ENSMUSG00000021495	Fam193b	2376,572672	1638,398928	3832,885825	2692,075098	1341,847118	0,689548506	False	0,689548506
ENSMUSG00000039952	Dag1	7726,114055	4691,42952	3699,997807	4023,518411	4795,531431	-0,688052274	False	0,688052274
ENSMUSG00000037664	Cdkn1c	675,0416691	357,2593207	1087,265607	573,665711	298,9791986	0,687655952	False	0,687655952
ENSMUSG00000003585	Sec14l2	202,3486557	243,9351276	341,5551552	243,6114663	125,7610915	-0,68615762	False	0,68615762
ENSMUSG00000010067	Rassf1	629,1650508	1696,981773	1011,486489	1407,78239	2184,209145	0,68496661	False	0,68496661
ENSMUSG00000001435	Col18a1	876,5710994	360,1404442	526,0608339	462,5249959	545,75568	-0,683615874	False	0,683615874
ENSMUSG00000068663	Clec16a	697,1607529	434,0892821	414,039529	431,0912583	383,2154014	-0,683499552	False	0,683499552
ENSMUSG00000018921	Pelp1	565,2654753	540,6908536	327,2779301	471,5060638	907,6154243	0,683152543	False	0,683152543
ENSMUSG00000026489	Coq8a	243,309922	241,054004	151,5582361	369,3464166	251,5221829	-0,682922899	False	0,682922899
ENSMUSG00000006154	Eps8l1	938,0129989	343,8140774	1505,698128	565,8072766	283,5556685	0,682752737	False	0,682752737
ENSMUSG00000004612	Nkg7	361,278369	57,62247108	225,1408579	90,9333123	43,89773948	-0,682282981	False	0,682282981
ENSMUSG00000061132	Blnk	303,1133708	125,8090618	188,898671	80,82961094	111,5239868	-0,682244941	False	0,682244941
ENSMUSG00000032440	Tgfbr2	4527,85838	5195,626142	3144,284274	2822,300582	7506,51345	-0,681957188	False	0,681957188
ENSMUSG00000048794	Cfap100	353,9053411	405,2780466	438,2009869	647,759521	220,6751228	-0,681439534	False	0,681439534
ENSMUSG00000040234	Tm7sf3	670,9455424	271,7859886	418,4325213	360,3653488	333,3855349	-0,68120069	False	0,68120069
ENSMUSG00000024780	Cdc37l1	804,4792707	830,723958	986,2267825	1049,662309	501,8579405	-0,680776205	False	0,680776205
ENSMUSG00000033543	Gtf2a2	448,1162536	912,355792	718,2542492	848,7109148	979,9873732	0,680621567	False	0,680621567
ENSMUSG00000024446	Rpp21	256,4175272	530,1267339	488,7203989	410,8838556	962,1909923	0,680235766	False	0,680235766
ENSMUSG00000058230	Arhgap35	2336,430631	2103,220194	1571,593013	1725,487667	3743,172109	0,67995518	False	0,67995518
ENSMUSG00000027829	Ccnl1	1340,252634	2444,153148	2147,075011	2390,08669	4962,817412	0,679867624	False	0,679867624
ENSMUSG00000032334	Loxl1	1974,333037	3667,670284	1514,484113	1232,651567	4012,490673	-0,679600318	False	0,679600318
ENSMUSG00000063415	Cyp26b1	389,1320301	242,974753	414,039529	587,1373128	351,1819158	-0,679453321	False	0,679453321
ENSMUSG00000020413	Hus1	323,594004	186,3126565	202,0776481	161,6592219	175,5909579	-0,679275122	False	0,679275122
ENSMUSG00000035529	Prdm4	409,6126633	800,952348	255,8918044	534,373539	988,2923509	-0,678726375	False	0,678726375
ENSMUSG00000021607	Mrpl36	283,451963	249,6973747	453,5764601	267,1867695	115,083263	0,678241508	False	0,678241508
ENSMUSG00000033020	Polr2f	435,8278737	1065,05534	697,3875355	711,7496296	1194,730369	0,678202111	False	0,678202111
ENSMUSG00000032009	Sesn3	776,6256096	350,536699	485,4256546	298,6205071	359,4868936	-0,677968925	False	0,677968925
ENSMUSG00000021879	Dnah12	557,8924474	637,6886799	331,6709224	892,4936208	361,8597443	0,677854837	False	0,677854837
ENSMUSG00000041836	Ptpre	1069,908276	485,9495061	668,8330852	428,8459914	448,4687979	-0,677768999	False	0,677768999
ENSMUSG00000063146	Clip2	746,3142725	747,171375	683,1103104	467,0155299	1638,453465	-0,676312751	False	0,676312751
ENSMUSG00000071550	Cfap44	718,4606114	1020,878113	633,6891464	1233,7742	449,6552233	-0,676089838	False	0,676089838
ENSMUSG00000032515	Csrnp1	380,1205515	3247,026245	607,3311923	2154,333658	5877,551388	0,67602644	False	0,67602644
ENSMUSG00000037679	Inf2	841,3444104	1818,949337	1343,157411	1878,165821	2140,311406	0,674859987	False	0,674859987
ENSMUSG00000006095	Tbcb	383,3974528	771,1807379	611,7241847	681,4385255	868,4633864	0,674040562	False	0,674040562
ENSMUSG00000002346	Slc25a42	359,6399183	356,2989462	465,6571891	409,7612221	225,4208243	-0,673932365	False	0,673932365
ENSMUSG00000026914	Psmd14	398,962734	586,7888305	250,4005639	335,6674121	874,3955134	-0,672016183	False	0,672016183
ENSMUSG00000047878	A4galt	406,335762	507,0777455	676,5208219	765,6360369	255,0814591	-0,671714304	False	0,671714304
ENSMUSG00000057465	Saa2	304,7518215	2104,180569	485,4256546	1773,760907	885,0733419	0,671615513	False	0,671615513
ENSMUSG00000075273	Ttc30b	377,6628755	202,6390233	237,2215869	236,8756654	90,16832974	-0,670863674	False	0,670863674
ENSMUSG00000017309	Cd300lg	253,9598512	120,0468147	404,1552962	141,4518191	243,2172052	0,670309315	False	0,670309315
ENSMUSG00000024151	Msh2	370,2898476	201,6786488	232,8285945	198,7061269	125,7610915	-0,669386743	False	0,669386743
ENSMUSG00000041439	Mfsd6	1210,815033	761,5769927	606,2329443	696,0327609	708,2959586	-0,668916691	False	0,668916691
ENSMUSG00000048763	Hoxb3	305,5710468	280,4293592	356,9306284	426,6007244	192,2009134	-0,668892661	False	0,668892661
ENSMUSG00000032849	Abcc4	214,6370356	540,6908536	135,0845148	319,9505433	563,5520608	-0,668036732	False	0,668036732
ENSMUSG00000020570	Sypl	2483,071965	1152,449422	1562,807028	1430,23506	1447,438977	-0,667986426	False	0,667986426
ENSMUSG00000027937	Jtb	508,7389278	899,8709233	836,8650426	1026,087006	807,9556914	0,667350686	False	0,667350686
ENSMUSG00000054499	Dedd2	501,3658998	1926,511283	796,2298634	1294,396408	4272,317834	0,667321107	False	0,667321107
ENSMUSG00000025702	March8	1279,62996	805,7542205	727,0402339	746,5512677	677,4488984	-0,667314931	False	0,667314931
ENSMUSG00000026421	Csrp1	11506,83894	5619,151304	7246,240881	5201,160937	7171,94149	-0,667186893	False	0,667186893
ENSMUSG00000046229	Scand1	849,5366636	2166,604912	1348,648651	1591,894282	2897,250805	0,666766431	False	0,666766431
ENSMUSG00000060373	Hnrnpc	2758,331674	1978,371507	3456,186731	1738,959269	4064,69339	-0,665571803	False	0,665571803
ENSMUSG00000016024	Lbp	1421,355942	2279,929106	896,1704393	2529,293242	2939,96212	-0,665422841	False	0,665422841
ENSMUSG00000039270	Megf9	906,8824365	549,3342243	572,1872535	524,2698376	532,7050007	-0,66442818	False	0,66442818
ENSMUSG00000019467	Arhgef25	422,7202685	283,3104828	669,9313333	487,2229326	154,2353009	0,664309942	False	0,664309942
ENSMUSG00000041119	Pde9a	761,8795537	1449,205148	1206,974648	2119,53202	1250,492362	0,66376053	False	0,66376053
ENSMUSG00000002660	Clpp	437,4663244	775,022236	692,9945432	709,5043627	799,6507137	0,663672028	False	0,663672028
ENSMUSG00000016496	Cd274	384,2166781	323,6462125	277,8567661	217,7908961	608,6362257	0,66366006	False	0,66366006
ENSMUSG00000046201	Scaf8	856,9096916	671,301788	588,6609749	551,2130413	1356,084222	0,662231709	False	0,662231709
ENSMUSG00000063011	Msln	574,2769539	908,514294	941,1986109	1777,128807	1367,948476	0,661762546	False	0,661762546
ENSMUSG00000026875	Traf1	1293,556791	597,3529502	2046,036187	1720,997133	650,1611144	0,661488268	False	0,661488268

ENSMUSG00000028952	Zbtb48	186,7833745	464,8212667	295,4287355	373,8369506	1328,796438	0,661444113	False	0,661444113
ENSMUSG00000007097	Atp1a2	1745,769171	1319,554588	2760,995692	1593,016916	535,0778515	0,661325821	False	0,661325821
ENSMUSG00000032239	Rp9	627,5266001	1189,904028	991,7180229	1323,584879	1853,196461	0,66025336	False	0,66025336
ENSMUSG00000027695	Pld1	468,5968868	443,6930273	443,6922273	731,9570324	296,6063478	-0,659797895	False	0,659797895
ENSMUSG00000024914	Drap1	3221,193984	7110,612931	5087,085141	6595,471726	8761,751514	0,659243696	False	0,659243696
ENSMUSG00000030413	Pglyrp1	419,4433672	2309,700716	662,2435967	1779,374074	1611,165681	0,658885957	False	0,658885957
ENSMUSG00000009013	Dynll1	3624,252845	1866,007688	5720,774287	2074,626681	1605,233554	0,658526816	False	0,658526816
ENSMUSG00000015568	Lpl	2253,688873	17818,78881	3557,225555	13005,70893	13372,20058	0,658464093	False	0,658464093
ENSMUSG00000032549	Rab6b	1609,777767	235,2917569	1020,272473	697,1553943	86,60905356	-0,657907046	False	0,657907046
ENSMUSG00000041482	Piezo2	756,9642017	83,55258306	479,9344142	227,8945975	74,74479965	-0,657387807	False	0,657387807
ENSMUSG00000017466	Timp2	14160,30977	5962,965382	8979,276363	7097,288894	6280,936021	-0,657181738	False	0,657181738
ENSMUSG00000035877	Zhx3	547,2425181	816,3183402	347,0463956	932,9084262	821,0063707	-0,65705178	False	0,65705178
ENSMUSG00000037022	Mmaa	294,1018922	103,7204479	463,4606929	232,3851314	107,9647106	0,65613092	False	0,65613092
ENSMUSG00000043279	Trim56	1460,678757	830,723958	911,5459126	927,2952588	460,3330518	-0,655538242	False	0,655538242
ENSMUSG00000015536	Mocs2	407,9742126	826,8824599	642,4751311	758,900236	653,7203906	0,655162647	False	0,655162647
ENSMUSG00000004730	Adgre1	358,0014677	106,6015715	227,3373541	203,1966608	66,43982191	-0,655130747	False	0,655130747
ENSMUSG00000022595	Lypd2	752,8680751	1185,102155	725,9419858	407,5159551	529,1457245	0,654542435	False	0,654542435
ENSMUSG00000044641	Pard6b	1382,852351	2552,675469	878,5984699	1486,366734	4309,097021	-0,654371234	False	0,654371234
ENSMUSG00000089809	Rasgef1b	292,4634416	691,4696529	460,1659486	908,2104896	608,6362257	0,653897934	False	0,653897934
ENSMUSG00000033454	Zbtb1	322,7747787	337,0914558	342,6534033	429,9686248	205,2515927	-0,653134435	False	0,653134435
ENSMUSG00000046168	Kcnrg	226,1061901	355,3385716	116,4142973	257,0830681	169,6588309	0,652193811	False	0,652193811
ENSMUSG00000030967	Zranb1	892,1363806	2220,385885	1401,36456	2614,613387	3634,020973	0,65149614	False	0,65149614
ENSMUSG00000025795	Rassf3	2970,511034	790,3882283	1891,183207	945,2573946	749,8208473	-0,651422018	False	0,651422018
ENSMUSG00000073643	Wdfy1	391,5897061	1180,300283	615,018929	1438,093495	904,0561481	0,651287974	False	0,651287974
ENSMUSG00000039552	Rsph4a	588,2037845	374,546062	328,3761781	364,8558827	97,28688208	-0,651172913	False	0,651172913
ENSMUSG00000039735	Fnbp1l	2278,265633	1837,196453	2033,955458	4023,518411	1450,998254	-0,650890184	False	0,650890184
ENSMUSG00000040249	Lrp1	5674,773837	9650,803531	8908,988485	10876,07321	14485,0676	0,650698743	False	0,650698743
ENSMUSG00000020153	Ndufs7	822,5022278	1534,67848	1311,308216	1291,028508	1822,349401	0,650429366	False	0,650429366
ENSMUSG00000024601	Isoc1	935,5553229	239,133255	596,3487115	339,0353125	258,6407353	-0,649666783	False	0,649666783
ENSMUSG00000037253	Mex3c	601,3113897	577,1850853	438,2009869	444,5628602	943,208186	0,649463954	False	0,649463954
ENSMUSG00000043020	Wdr63	370,2898476	232,4106333	236,1233388	162,7818554	207,6244435	-0,649114348	False	0,649114348
ENSMUSG00000042870	Tom1	287,5480896	396,6346759	183,4074306	318,8279098	479,3158581	-0,648751163	False	0,648751163
ENSMUSG00000029592	Usp30	425,1779445	602,1548227	271,2672776	475,9965977	851,8534309	-0,648351732	False	0,648351732
ENSMUSG00000023960	Enpp5	1463,955659	611,7585679	934,6091224	752,1644351	742,7022949	-0,647436833	False	0,647436833
ENSMUSG00000045730	Adrb2	622,6112482	1508,748368	975,2443016	2458,567333	1254,051639	0,647432023	False	0,647432023
ENSMUSG00000075602	Ly6a	8355,279105	26294,09393	13079,03647	24955,01974	13976,09111	0,646496335	False	0,646496335
ENSMUSG00000025981	Coq10b	254,7790766	701,0733981	398,6640557	486,1002991	807,9556914	0,645926732	False	0,645926732
ENSMUSG00000023707	Ogfod2	254,7790766	482,108008	398,6640557	511,9208693	537,4507022	0,645926732	False	0,645926732
ENSMUSG00000019102	Aldh3a1	590,6614604	500,3551238	591,9557191	316,5826428	924,2253798	0,645913225	False	0,645913225
ENSMUSG00000029188	Slc34a2	24329,35375	7563,909703	15562,1754	15310,47547	3142,840861	-0,644654254	False	0,644654254
ENSMUSG00000042985	Upk3b	1630,2584	44,17722782	686,4050546	1042,926508	812,701393	-0,644463155	False	0,644463155
ENSMUSG00000029375	Cxcl15	18725,85251	4651,09379	11980,78839	7179,241138	6340,257291	-0,644308554	False	0,644308554
ENSMUSG00000024843	Chka	1321,410452	2831,184079	2064,706404	2336,200283	5945,177636	0,643857988	False	0,643857988
ENSMUSG00000022951	Rcan1	890,4979299	1025,679985	569,9907574	597,2410142	1529,302329	-0,643673732	False	0,643673732
ENSMUSG00000022361	Zhx1	854,4520156	524,3644868	542,5345552	500,6945344	546,9421054	-0,643611345	False	0,643611345
ENSMUSG00000041351	Rap1gap	906,8824365	2248,236746	1416,740033	3140,005858	4248,589326	0,643587608	False	0,643587608
ENSMUSG00000053907	Mat2a	3756,148122	4532,967725	4924,544424	4289,582547	2404,884268	-0,643286488	False	0,643286488
ENSMUSG00000027752	Exosc8	235,1176687	394,7139269	383,2885825	367,1011497	393,8932299	0,642794673	False	0,642794673
ENSMUSG00000033282	Rpgrip1l	351,4476651	193,9956526	225,1408579	195,3382264	92,54118052	-0,642481973	False	0,642481973
ENSMUSG00000031790	Mmp15	4274,717754	2215,584013	2524,872353	2739,225704	1878,111394	-0,642061022	False	0,642061022
ENSMUSG00000009076	Zmat5	389,9512554	356,2989462	608,4294404	394,0443533	180,3366595	0,641796167	False	0,641796167
ENSMUSG00000015837	Sqstm1	5178,323289	11695,44088	3320,003968	5651,336965	20442,10949	-0,641300071	False	0,641300071
ENSMUSG00000037411	Serpine1	321,136328	882,584182	559,0082765	500,6945344	1388,117708	0,64074483	False	0,64074483
ENSMUSG00000018906	P4ha2	775,8063842	1592,300951	1209,171144	1635,676988	1824,722252	0,640249903	False	0,640249903
ENSMUSG00000048310	Pskh1	823,3214532	478,2665099	528,25733	467,0155299	410,5031854	-0,640214938	False	0,640214938
ENSMUSG00000020628	Trappc12	480,8852667	199,7578997	308,6077126	200,9513939	205,2515927	-0,639918608	False	0,639918608
ENSMUSG00000029815	Malsu1	126,1607003	416,8025408	196,5864076	276,1678374	603,8905241	0,639901001	False	0,639901001
ENSMUSG00000028001	Fga	193,3371771	1185,102155	300,919976	1160,803024	475,7565819	0,638260798	False	0,638260798
ENSMUSG00000028871	Rspo1	436,647099	40,33572975	230,6320984	280,6583713	117,4561137	-0,637652664	False	0,637652664
ENSMUSG00000039601	Rcan2	507,9197025	326,5273361	235,0250907	270,5546699	206,4380181	-0,637396662	False	0,637396662
ENSMUSG00000070639	Lrrc8b	1567,17805	880,6634329	1008,191744	756,6549691	643,0425621	-0,63639905	False	0,63639905
ENSMUSG00000001741	Il16	666,8494158	406,2384211	429,4150022	408,6385886	316,7755795	-0,634988422	False	0,634988422
ENSMUSG00000027208	Fgf7	237,5753447	259,3011198	418,4325213	642,1463536	153,0488755	-0,634392678	False	0,634392678
ENSMUSG00000087370	Tmem170b	463,6815348	576,2247108	298,7234798	459,1570955	657,2796668	-0,634323637	False	0,634323637
ENSMUSG00000069678	Pcgf1	152,3759107	267,9444905	236,1233388	277,2904709	347,6226396	0,631905803	False	0,631905803
ENSMUSG00000024812	Tjp2	2379,030348	2696,731646	1535,350826	2170,050527	3739,612833	-0,631805328	False	0,631805328
ENSMUSG00000003873	Bax	442,3816763	881,6238075	685,3068065	749,9191681	1054,732173	0,631458386	False	0,631458386
ENSMUSG00000038871	Bpgm	1088,750459	624,2434367	702,8787759	643,268987	526,7728737	-0,63132553	False	0,63132553
ENSMUSG00000036745	Ttll7	907,7016618	1318,594213	781,9526382	1359,509151	586,0941433	-0,631085778	False	0,631085778
ENSMUSG00000079502	Cfap77	335,0631586	101,7996989	216,3548732	156,0460544	64,06697113	-0,631033449	False	0,631033449
ENSMUSG00000031304	Il2rg	930,6399709	406,2384211	1440,901491	480,4873117	335,7583857	0,63067665	False	0,63067665
ENSMUSG00000000628	Hk2	206,4447823	1084,262831	319,5901934	716,2401636	1930,314112	0,630467184	False	0,630467184
ENSMUSG00000036278	Macrod1	344,0746371	156,5410464	532,6503224	344,64848	256,2678845	0,630467184	False	0,630467184
ENSMUSG00000000531	Grasp	697,9799782	1080,421333	1122,409545	1416,763458	1233,882407	0,630336473	False	0,630336473
ENSMUSG00000030217	Art4	349,8092144	297,7161006	541,4363071	217,7908961	209,9972943	0,630223341	False	0,630223341
ENSMUSG00000030577	Cd22	861,8250435	95,07707728	556,8117803	114,5086155	61,69412035	-0,630205288	False	0,630205288
ENSMUSG00000030590	Fam98c	572,6385032	1070,817587	886,2862065	1092,322381	1121,171995	0,630147983	False	0,630147983
ENSMUSG00000009633	G0s2	541,5079408	529,1663594	837,9632907	358,1200818	279,9963923	0,62990455	False	0,62990455
ENSMUSG00000019997	Ctgf	1754,780649	2710,17689	1150,963996	2628,084989	1134,222674	-0,6295868	False	0,6295868
ENSMUSG00000034634	Ly6d	370,2898476	50,89984945	239,4180831	26,94320365	42,71131409	-0,629122873	False	0,629122873
ENSMUSG00000031380	Vegfd	791,3716654	347,6555755	1223,448369	852,0788153	247,9629068	0,628525903	False	0,628525903
ENSMUSG00000090291	Lrrc10b	446,477803	676,1036606	288,839247	410,8838556	729,6516156	-0,628321666	False	0,628321666
ENSMUSG00000023912	Slc25a27	199,8909797	169,0259152	310,8042087	350,2616474	129,3203676	-0,628263856	False	0,628263856
ENSMUSG00000055782	Abcd2	353,0861157	167,1051661	228,4356022	148,1876201	77,11765043	-0,628232576	False	0,628232576
ENSMUSG00000047547	Cltb	2185,693171	4756,734987	3378,211117	4288,459914	6562,118839	0,628168601	False	0,628168601
ENSMUSG00000037514	Pank2	584,9268831	674,1829116	641,3768831	736,4475663	378,4696998	-0,628078494	False	0,628078494
ENSMUSG00000008200	Fnbp4	1173,949893	4609,797686	1814,30584	5061,954385	4436,044538	0,628046842	False	0,628046842
ENSMUSG00000041794	Myrip	402,2396353	1888,096302	260,2847967	2540,519577	1666,927675	-0,627964201	False	0,627964201
ENSMUSG00000019528	Gyg	2793,558363	698,1922745	1807,716352	854,3240823	698,8045554	-0,627935639	False	0,627935639
ENSMUSG00000069515	Lyz1	5360,191312	2058,082592	3065,210412	1720,997133	3469,107844	-0,627719803	False	0,627719803
ENSMUSG00000026171	Rnf25	531,6772369	821,1202128	822,5878175	831,8714126	1159,137607	0,627042749	False	0,627042749
ENSMUSG00000024766	Lipo3	457,9469575	279,4689847	296,5269836	233,5077649	240,8443544	-0,627017111	False	0,627017111
ENSMUSG00000003134	Tbc1d8	841,3444104	7859,705055	1299,227487	3556,502881	9459,369644	0,626885657	False	0,626885657
ENSMUSG00000074604	Mgst2	232,6599927	243,9351276	255,8918044	349,1390139	150,6760247	-0,626773276	False	0,626773276
ENSMUSG00000035934	Pknox2	524,304209	209,3616449	809,4088404	434,4591588	132,8796438	0,626464479	False	0,626464479
ENSMUSG00000028228	Cpne3	1122,338697	519,5626142	727,0402339	606,222082	386,7746775	-0,626401006	False	0,626401006
ENSMUSG00000024921	Smarca2	4914,532734	1370,454437	3183,821205	2290,17231	976,428097	-0,626294933	False	0,626294933
ENSMUSG00000028410	Dnaja1	1520,482206	1188,943653	2346,956163	1065,379177	1282,525848	0,626261962	False	0,626261962
ENSMUSG00000004508	Gab2	783,1794122	1913,06604	507,3906164	1310,113277	2598,271607	-0,626246002	False	0,626246002
ENSMUSG00000050556	Kcnb1	330,9670319	230,4898843	254,7935563	214,4229957	641,8561367	-0,62622788	False	0,62622788
ENSMUSG00000041313	Slc7a1	391,5897061	468,6627648	260,2847967	253,7151677	751,0072727	-0,626133071	False	0,626133071
ENSMUSG00000052310	Slc39a1	1671,219666	2344,274198	1082,872614	1613,224318	3289,95761	-0,626037836	False	0,626037836
ENSMUSG00000028173	Wls	2216,823734	1183,181406	1436,508498	1219,179965	658,4660921	-0,625927534	False	0,625927534
ENSMUSG00000060188	Cxcl17	585,7461085	3685,9174	903,8581759	2127,390455	3467,921419	0,625820953	False	0,625820953
ENSMUSG00000000402	Egfl6	750,4103991	253,5388727	486,5239027	252,5925342	389,1475283	-0,625169135	False	0,625169135
ENSMUSG00000056234	Ncoa4	335,8823839	167,1051661	170,2284535	217,7908961	211,1837196	-0,625012483	False	0,625012483
ENSMUSG00000025534	Gusb	880,667226	369,7441894	571,0890055	382,8180185	374,9104236	-0,624881367	False	0,624881367
ENSMUSG00000036832	Lpar3	530,0387863	726,0431356	343,7516514	528,7603716	923,0389544	-0,62473129	False	0,62473129
ENSMUSG00000012609	Ttll5	407,9742126	207,4408959	264,6777891	236,8756654	239,657929	-0,624240831	False	0,624240831
ENSMUSG00000037536	Fbxo34	280,994287	334,2103322	182,3091825	198,7061269	581,3484417	-0,62415357	False	0,62415357

ENSMUSG00000019731	Slc35e1	1009,285602	595,4322011	628,197906	508,5529688	654,906816	-0,623972927	False	0,623972927
ENSMUSG00000028741	Mrto4	164,6642906	406,2384211	253,6953082	281,7810048	745,0751457	0,623569115	False	0,623569115
ENSMUSG00000031639	Tlr3	648,0072333	286,1916063	420,6290175	323,3184438	238,4715036	-0,623461538	False	0,623461538
ENSMUSG00000004897	Hdgf	4542,604436	8591,510437	6995,840317	8789,097556	9120,051983	0,622977677	False	0,622977677
ENSMUSG00000034041	Lyl1	436,647099	208,4012704	672,1278295	234,6303984	56,94841878	0,622267885	False	0,622267885
ENSMUSG00000025142	Aspscr1	1078,10053	2343,313824	1659,45286	2287,927043	2570,983823	0,622215936	False	0,622215936
ENSMUSG00000027257	Pacsin3	265,4290058	432,1685331	408,5482885	514,1661362	634,7375843	0,622180573	False	0,622180573
ENSMUSG00000079056	Kcnip3	66,35725145	664,5791664	102,1370721	756,6549691	569,4841878	0,622180573	False	0,622180573
ENSMUSG00000016495	Plgrkt	542,3271662	415,8421663	834,6685464	560,1941091	335,7583857	0,622039966	False	0,622039966
ENSMUSG00000058756	Thra	2582,198229	1297,465974	1678,123078	1399,923956	1350,152095	-0,621751227	False	0,621751227
ENSMUSG00000019944	Rhobtb1	270,3443578	329,4084597	175,719694	510,7982358	333,3855349	-0,621522355	False	0,621522355
ENSMUSG00000044217	Aqp5	5416,717859	6166,56478	8053,453225	6135,191997	3522,496987	-0,620820475	False	0,620820475
ENSMUSG00000020696	Rffl	290,0057656	412,0006682	328,3761781	369,3464166	188,6416372	-0,620433438	False	0,620433438
ENSMUSG00000052748	Swt1	480,0660414	766,3788653	738,0227147	1155,189856	777,1086313	0,620432333	False	0,620432333
ENSMUSG00000021990	Spata13	760,241103	630,9660583	436,0044907	598,3636476	1168,62901	0,620288076	False	0,620288076
ENSMUSG00000037262	Kin	190,8795011	363,9819423	293,2322393	420,987557	349,9954904	0,619381552	False	0,619381552
ENSMUSG00000029093	Sorcs2	1060,896798	1507,787993	690,798047	1245,000535	1962,347597	-0,618948411	False	0,618948411
ENSMUSG00000045761	Togaram2	925,724619	1503,946495	602,9382	1100,180816	1426,08332	-0,618572954	False	0,618572954
ENSMUSG00000052926	Rnaseh2a	220,3716128	186,3126565	305,3129683	257,0830681	143,5574723	-0,618309969	False	0,618309969
ENSMUSG00000048163	Selplg	2474,879711	1516,431364	1612,228192	1544,743676	854,2262817	-0,618302451	False	0,618302451
ENSMUSG00000018076	Med13l	819,2253265	1221,596387	533,7485705	1303,377476	1392,863409	-0,618100016	False	0,618100016
ENSMUSG00000038648	Creb3l2	2078,374653	861,4559426	1354,139892	1173,151992	881,5140657	-0,618078955	False	0,618078955
ENSMUSG00000025153	Fasn	6917,538657	3190,364149	2815,908096	2349,671885	4507,230061	-0,61801771	False	0,61801771
ENSMUSG00000053153	Spag16	606,2267416	246,8162511	337,1621628	395,1669868	215,9294212	-0,617395067	False	0,617395067
ENSMUSG00000063632	Sox11	635,7188534	151,7391738	975,2443016	340,157946	181,5230849	0,617374789	False	0,617374789
ENSMUSG00000035910	Dcdc2a	328,5093559	96,03745179	214,158377	139,2065522	68,81267269	-0,617256348	False	0,617256348
ENSMUSG00000026768	Itga8	2245,49662	247,7766256	1463,964701	452,4212946	312,0298779	-0,617153783	False	0,617153783
ENSMUSG00000043631	Ecm2	392,4089314	122,9279383	255,8918044	124,6123169	92,54118052	-0,616823936	False	0,616823936
ENSMUSG00000041577	Prelp	10183,79003	1765,168364	6641,106185	2522,557441	1466,421784	-0,616779109	False	0,616779109
ENSMUSG00000021823	Vcl	4141,184026	1889,056677	2700,592047	1454,932997	1759,468855	-0,616767593	False	0,616767593
ENSMUSG00000069662	Marcks	1794,103465	4710,63701	2750,013211	6125,088295	5722,129662	0,616175457	False	0,616175457
ENSMUSG00000027510	Rbm38	645,5495573	618,4811895	508,4888645	446,8081271	989,4787763	0,616140911	False	0,616140911
ENSMUSG00000058806	Col13a1	688,1492743	673,2225371	845,6510273	166,1497558	1054,732173	0,616083248	False	0,616083248
ENSMUSG00000015354	Pcolce2	3729,932912	688,5885294	2435,914258	881,2672859	1175,747563	-0,614686329	False	0,614686329
ENSMUSG00000040007	Bahd1	657,8379372	634,8075564	517,2748492	450,1760276	1007,275157	0,614653723	False	0,614653723
ENSMUSG00000051375	Pcdh1	4371,386342	1570,212337	2855,445027	2108,305685	1124,731271	-0,614375279	False	0,614375279
ENSMUSG00000028525	Pde4b	1037,958489	2285,691353	2279,963029	678,0706251	3301,821864	-0,614241295	False	0,614241295
ENSMUSG00000010358	Ifi35	996,9972224	805,7542205	1522,171849	1132,737187	651,3475398	-0,614161957	False	0,614161957
ENSMUSG00000024098	Twsg1	3364,558416	1324,35646	2198,692671	1371,858119	1504,387396	-0,613771209	False	0,613771209
ENSMUSG00000074733	Zfp950	684,0531477	311,1613438	1046,630427	544,4772403	177,9638087	0,613571781	False	0,613571781
ENSMUSG00000047721	Bola2	284,2711883	645,3716761	434,9062426	537,7414394	761,6851012	0,613436532	False	0,613436532
ENSMUSG00000037958	Nsrp1	377,6628755	728,9242591	577,678494	843,0977474	1564,895091	0,613167813	False	0,613167813
ENSMUSG00000047030	Spata2	337,5208345	414,8817917	220,7478656	225,6493305	758,125825	-0,612577071	False	0,612577071
ENSMUSG00000040563	Plppr2	339,1592852	221,8465136	604,0364481	429,9686248	288,3013701	-0,612401109	False	0,612401109
ENSMUSG00000022941	Ripply3	1039,596939	475,3853864	1589,164982	994,6532679	422,3674393	0,612244617	False	0,612244617
ENSMUSG00000049502	Dtx3l	725,014414	326,5273361	474,4431738	362,6106157	411,6896108	-0,611774378	False	0,611774378
ENSMUSG00000029430	Ran	1342,71031	1487,620128	1175,125454	879,0220189	2128,447152	-0,611176867	False	0,611176867
ENSMUSG00000056413	Adap1	205,625557	246,8162511	187,8004229	134,7160182	289,4877955	-0,610098184	False	0,610098184
ENSMUSG00000001774	Chordc1	833,9713824	452,3363979	1272,869533	514,1661362	370,1647221	0,61001477	False	0,61001477
ENSMUSG00000056267	Cep70	455,4892816	452,3363979	398,6640557	694,9101274	226,6072497	0,609409301	False	0,609409301
ENSMUSG00000036565	Ttyh3	3489,899891	1963,965889	2287,650766	1667,110726	1865,060715	-0,609318826	False	0,609318826
ENSMUSG00000031591	Asah1	2583,83668	1688,338403	1604,540456	1694,053929	1379,81273	-0,60903508	False	0,60903508
ENSMUSG00000039813	Tbc1d2	262,9713298	375,5064365	172,4249497	267,1867695	533,8914261	-0,608936974	False	0,608936974
ENSMUSG00000053040	Aph1c	244,1291473	256,4199963	291,0357432	327,8089777	160,1674278	-0,608063767	False	0,608063767
ENSMUSG00000024462	Gabbr1	933,0976469	954,6122708	1571,593013	1819,78888	612,1955019	-0,60803562	False	0,60803562
ENSMUSG00000012350	Ehf	602,130615	465,7816412	395,3693115	410,8838556	1259,983766	-0,606875579	False	0,606875579
ENSMUSG00000036968	Cnpy4	253,1406259	153,6599229	385,4850787	259,3283351	117,4561137	0,606735957	False	0,606735957
ENSMUSG00000016757	Ttll12	446,477803	125,8090618	293,2322393	149,3102535	123,3882407	-0,606544723	False	0,606544723
ENSMUSG00000019471	Cdc37	2514,202527	1460,729642	1396,971567	1651,393857	1544,725859	-0,606416625	False	0,606416625
ENSMUSG00000013236	Ptprs	972,4204626	317,8839654	1480,438422	483,8550321	270,5049892	0,606372326	False	0,606372326
ENSMUSG00000022048	Dpysl2	1830,149379	911,3954175	2786,255398	1724,365033	567,111337	0,606366098	False	0,606366098
ENSMUSG00000026082	Rev1	248,2252739	409,1195446	377,7973421	552,3356747	542,1964038	0,605962536	False	0,605962536
ENSMUSG00000053411	Cbx7	1747,407622	1572,133086	1724,249497	2726,876736	1148,459779	-0,605515856	False	0,605515856
ENSMUSG00000034664	Itga2b	350,6284398	230,4898843	533,7485705	623,0615843	281,1828177	-0,605239584	False	0,605239584
ENSMUSG00000038344	Txlng	471,0545628	438,8911547	691,8962951	539,9867064	309,6570271	-0,605222993	False	0,605222993
ENSMUSG00000024300	Myo1f	898,6901832	567,5813401	590,857471	539,9867064	304,9113256	-0,605013683	False	0,605013683
ENSMUSG00000044037	Als2cl	1969,417685	2995,408121	3150,873763	3913,50033	2996,910538	0,604983464	False	0,604983464
ENSMUSG00000047945	Marcksl1	406,335762	361,1008187	420,6290175	267,1867695	608,6362257	-0,604823773	False	0,604823773
ENSMUSG00000019817	Plagl1	691,4261756	239,133255	454,6747082	184,1118916	258,6407353	-0,604740467	False	0,604740467
ENSMUSG00000060961	Slc4a4	263,7905551	311,1613438	173,5231978	194,2155929	392,7068045	-0,604264365	False	0,604264365
ENSMUSG00000038459	Abhd17c	1365,648619	2335,630828	898,3669355	1075,482879	2597,085181	-0,604209591	False	0,604209591
ENSMUSG00000011148	Adssl1	232,6599927	640,5698035	353,6358841	601,7315481	501,8579405	0,604041523	False	0,604041523
ENSMUSG00000003379	Cd79a	1969,417685	279,4689847	1295,932743	205,4419278	144,7438977	-0,603778272	False	0,603778272
ENSMUSG00000004798	Ulk2	1413,163688	557,9775949	930,21613	728,5891319	520,8407468	-0,603290723	False	0,603290723
ENSMUSG00000063785	Utp14a	371,1090729	690,5092784	563,4012688	680,3158921	882,7004911	0,60231954	False	0,60231954
ENSMUSG00000020085	Aifm2	248,2252739	474,4250119	376,699094	538,8640729	660,8389429	0,601762547	False	0,601762547
ENSMUSG00000019820	Utrn	3262,974476	1338,762078	2150,369755	1658,129658	1248,119512	-0,60160295	False	0,60160295
ENSMUSG00000030102	Itpr1	1341,891085	651,1339232	881,8932142	884,6351864	690,4995777	-0,601113048	False	0,601113048
ENSMUSG00000052942	Glis3	349,8092144	491,7117532	230,6320984	270,5546699	596,7719718	-0,600974977	False	0,600974977
ENSMUSG00000030737	Slco2b1	369,4706223	289,0727299	281,1515104	560,1941091	161,3538532	0,600467157	False	0,600467157
ENSMUSG00000037649	H2-DMa	1416,44059	327,4877106	934,6091224	301,9884075	542,1964038	-0,599835067	False	0,599835067
ENSMUSG00000022947	Cbr3	211,3601342	340,9329539	139,4775071	279,5357378	403,384633	-0,599670804	False	0,599670804
ENSMUSG00000022037	Clu	4598,311758	5040,04547	3034,459466	3999,943108	11172,56791	-0,599664733	False	0,599664733
ENSMUSG00000018651	Tada2a	213,8178102	151,7391738	323,9831858	199,8287604	123,3882407	0,599536912	False	0,599536912
ENSMUSG00000031010	Usp9x	977,3358146	945,9689002	590,857471	825,1356117	1480,658888	0,599313048	False	0,599313048
ENSMUSG00000059772	Slx1b	373,5667489	230,4898843	551,3205399	565,8072766	96,10045669	0,598944684	False	0,598944684
ENSMUSG00000024194	Cuta	1344,348761	1129,400433	2036,151954	1269,698472	892,1918942	0,59893777	False	0,59893777
ENSMUSG00000020100	Slc29a3	445,6585776	132,5316835	294,3304874	212,1777287	116,2696883	-0,598501886	False	0,598501886
ENSMUSG00000029452	Tmem116	232,6599927	153,6599229	345,9481475	463,6476294	167,2859802	-0,598482218	False	0,598482218
ENSMUSG00000066677	Pydc3	576,7346299	84,51295758	381,0920863	237,9982989	72,37194887	0,597768001	False	0,597768001
ENSMUSG00000026972	Arrdc1	1612,235443	2108,022067	2286,552518	2516,944274	1065,410001	-0,597653714	False	0,597653714
ENSMUSG00000020682	Mmp28	706,1722315	106,6015715	466,7554372	276,1678374	88,98190434	-0,597353263	False	0,597353263
ENSMUSG00000033174	Mgll	3030,314483	1135,16268	4584,087517	2218,323767	1168,62901	0,597167065	False	0,597167065
ENSMUSG00000038774	Ascc3	428,4548458	195,9164017	283,3480066	179,6213576	249,1493322	-0,596568116	False	0,596568116
ENSMUSG00000056305	Usp39	679,1377957	754,8543711	449,1834678	637,6558196	1294,390102	-0,596399494	False	0,596399494
ENSMUSG00000006412	Pfdn2	350,6284398	530,1267339	533,7485705	596,1183807	1129,476972	0,596394277	False	0,596394277
ENSMUSG00000028082	Sh3d19	1673,677342	539,7304791	1107,034072	857,6919827	454,4009249	-0,596321801	False	0,596321801
ENSMUSG00000041782	Lad1	192,5179517	285,2312318	127,3967781	129,1028508	436,604544	-0,595664187	False	0,595664187
ENSMUSG00000073421	H2-Ab1	12060,63526	2967,55726	7983,165347	2826,791116	6698,557759	-0,595273101	False	0,595273101
ENSMUSG00000040152	Thbs1	1285,364537	2261,68199	1042,237435	850,9561818	2083,362987	-0,595020824	False	0,595020824
ENSMUSG00000020387	Jade2	1141,18088	1525,074734	755,5946841	1403,291857	1547,09871	-0,594843023	False	0,594843023
ENSMUSG00000066357	Wdr6	1852,268463	916,1972901	1226,743114	1078,850779	1184,05254	-0,594460042	False	0,594460042
ENSMUSG00000055639	Dach1	343,2554118	143,0958032	227,3373541	187,479792	111,5239868	-0,594447712	False	0,594447712
ENSMUSG00000060703	Cd302	354,7245664	942,1274021	235,0250907	510,7982358	1364,3892	-0,593884464	False	0,593884464
ENSMUSG00000047330	Kcne4	71,27260341	646,3320506	47,22466776	97,66911322	1473,540336	-0,593806977	False	0,593806977
ENSMUSG00000001918	Slc1a5	3043,422088	6671,721776	4591,775253	5297,707417	8816,327082	0,593357597	False	0,593357597
ENSMUSG00000029388	Eif2b1	254,7790766	570,4624637	384,3868306	486,1002991	696,4317047	0,593312106	False	0,593312106
ENSMUSG00000045629	Sh3tc2	515,2927304	100,8393244	341,5551552	341,2805795	59,32126956	-0,593273672	False	0,593273672
ENSMUSG00000049470	Aff4	1786,730437	1760,366491	1044,433931	1491,979902	2695,558489	0,593262222	False	0,593262222
ENSMUSG00000023043	Krt18	1613,873893	3269,114859	2433,717762	3786,642746	3955,542254	0,592634016	False	0,592634016

ENSMUSG00000005836	Gata6	1015,02018	745,2506259	644,6716273	715,1175301	1530,488755	0,592484035	False	0,592484035
ENSMUSG00000026335	Pam	4240,31029	1997,578997	2813,7116	1857,958418	2492,679747	-0,591695377	False	0,591695377
ENSMUSG00000028483	Snapc3	277,7173857	202,6390233	418,4325213	279,5357378	167,2859802	0,591377489	False	0,591377489
ENSMUSG00000021838	Samd4	784,8178628	477,3061354	512,8818568	520,9019372	423,5538647	-0,591346076	False	0,591346076
ENSMUSG00000038495	Otud7b	2356,911264	2182,931279	2197,594423	3924,726664	1564,895091	-0,5908315	False	0,5908315
ENSMUSG00000045934	Mtmr11	226,9254155	299,6368496	300,919976	425,4780909	150,6760247	-0,590768323	False	0,590768323
ENSMUSG00000052566	Hook2	258,8752032	401,4365485	389,878071	539,9867064	424,7402901	0,590766231	False	0,590766231
ENSMUSG00000027312	Atrn	929,0015203	572,3832127	617,2154251	568,0525435	569,4841878	-0,58990684	False	0,58990684
ENSMUSG00000029186	Pi4k2b	1009,285602	1756,524993	1679,221326	2574,198582	1518,624501	0,589430711	False	0,589430711
ENSMUSG00000006304	Arpc2	3661,117984	4095,997319	2433,717762	2596,651251	6099,412936	-0,589122399	False	0,589122399
ENSMUSG00000022372	Sla	404,6973113	444,6534018	269,0707814	381,695385	684,5674508	-0,588857528	False	0,588857528
ENSMUSG00000038156	Spon1	2859,915615	4457,098138	4532,469857	5352,716458	4300,792043	0,588629795	False	0,588629795
ENSMUSG00000002233	Rhoc	2085,747681	9730,514616	3134,400041	4264,88461	11393,24303	0,58762468	False	0,58762468
ENSMUSG00000032698	Lmo2	1037,958489	262,1822434	690,798047	395,1669868	288,3013701	-0,587412838	False	0,587412838
ENSMUSG00000039316	Rftn1	470,2353374	163,263668	313,0007049	199,8287604	243,2172052	-0,587217053	False	0,587217053
ENSMUSG00000024027	Glp1r	3044,241313	185,352282	2027,365969	565,8072766	59,32126956	-0,586476184	False	0,586476184
ENSMUSG00000057506	Bloc1s2	188,4218251	339,9725793	325,0814339	282,9036383	499,4850897	0,586344647	False	0,586344647
ENSMUSG00000029090	Adgra3	1214,911159	685,7074058	809,4088404	738,6928333	476,9430073	-0,585910309	False	0,585910309
ENSMUSG00000032514	Ttc21a	744,6758218	429,2874095	496,4081355	454,6665615	424,7402901	-0,585085757	False	0,585085757
ENSMUSG00000061046	Haghl	268,7059071	152,6995484	403,0570481	282,9036383	80,67692661	0,584956013	False	0,584956013
ENSMUSG00000011114	Tbrg1	1514,747629	2192,535024	1010,38824	1350,528083	4596,211966	-0,584167694	False	0,584167694
ENSMUSG00000031825	Crispld2	2417,533939	7955,742507	4586,284013	3623,86089	12205,94443	0,583991432	False	0,583991432
ENSMUSG00000027412	Lpin3	172,8565439	303,4783477	115,3160492	348,0163804	360,6733189	-0,583981906	False	0,583981906
ENSMUSG00000020300	Cpeb4	1052,704545	1051,610097	724,8437377	721,853331	1577,94577	0,583947043	False	0,583947043
ENSMUSG00000066196	Spag8	218,7331622	273,7067376	173,5231978	324,4410772	145,9303231	-0,58389227	False	0,58389227
ENSMUSG00000079654	Prrt4	404,6973113	65,30546722	606,2329443	553,4583082	28,47420939	0,58302899	False	0,58302899
ENSMUSG00000036572	Upf3b	510,3773784	873,9408113	764,3806688	1069,869711	928,9710814	0,582726907	False	0,582726907
ENSMUSG00000020642	Rnf144a	3271,166729	495,5532513	2184,415446	532,128272	391,5203791	-0,582558031	False	0,582558031
ENSMUSG00000039914	Coq10a	442,3816763	514,7607416	434,9062426	531,0056385	295,4199224	-0,582524508	False	0,582524508
ENSMUSG00000032051	Fdx1	302,2941455	1226,398259	452,478212	949,7479285	1223,204578	0,581895286	False	0,581895286
ENSMUSG00000039046	Usp6nl	856,9096916	821,1202128	544,7310514	603,9768151	1282,525848	0,581772828	False	0,581772828
ENSMUSG00000033938	Ndufb7	657,8379372	1456,888144	1094,953343	984,5495666	2007,431762	0,581731629	False	0,581731629
ENSMUSG00000036155	Mgat5	838,067509	478,2665099	560,1065246	427,7233579	522,0271722	-0,581365228	False	0,581365228
ENSMUSG00000062825	Actg1	8017,758271	7250,82761	7149,595049	4784,663914	11995,94713	0,581276242	False	0,581276242
ENSMUSG00000050912	Tmem123	3040,964412	1540,440727	2032,85721	1423,499259	1770,146684	-0,581020051	False	0,581020051
ENSMUSG00000045983	Eif4g1	3713,548405	3902,001666	2691,806062	2693,197731	5554,843682	0,58094793	False	0,58094793
ENSMUSG00000020393	Kremen1	957,6744067	1246,566124	640,278635	939,6442271	1843,705058	-0,580835377	False	0,580835377
ENSMUSG00000039640	Mrpl12	368,6513969	786,5467302	551,3205399	566,92991	931,3439321	0,580634127	False	0,580634127
ENSMUSG00000015647	Lama5	2534,68316	7146,146788	3790,05415	7010,846115	10521,22037	0,580413042	False	0,580413042
ENSMUSG00000024664	Fads3	2809,94287	3589,879948	3380,407613	3585,691352	1879,29782	-0,580347084	False	0,580347084
ENSMUSG00000055839	Elob	1854,726139	3899,120543	2773,076421	2942,422365	5086,205652	0,580281194	False	0,580281194
ENSMUSG00000025731	Mettl26	301,4749202	248,7370001	476,6396699	387,3085524	201,6923165	-0,579881863	False	0,579881863
ENSMUSG00000035671	Zswim4	444,8393523	819,1994638	297,6252317	513,0435028	1357,270648	-0,579787582	False	0,579787582
ENSMUSG00000052593	Adam17	896,2325072	510,9192435	599,6434557	572,5430775	574,2298894	-0,579768117	False	0,579768117
ENSMUSG00000029581	Fscn1	584,9268831	234,3313824	383,2885825	241,3661993	391,5203791	-0,579168893	False	0,579168893
ENSMUSG00000041203	Trir	1074,004403	1784,375854	1604,540456	1689,563395	1954,042619	0,579160257	False	0,579160257
ENSMUSG00000021466	Ptch1	1174,769118	399,5157995	786,3456306	600,6089146	410,5031854	-0,579141767	False	0,579141767
ENSMUSG00000028294	Cfap206	621,7920228	807,6749696	416,2360251	678,0706251	813,8878184	-0,579030273	False	0,579030273
ENSMUSG00000028430	Nol6	680,7762464	723,162012	537,0433147	455,789195	1219,645302	-0,578813975	False	0,578813975
ENSMUSG00000034926	Dhcr24	1554,070444	971,8990121	1011,486489	755,5323356	1040,495068	-0,578781774	False	0,578781774
ENSMUSG00000053436	Mapk14	1708,904031	1127,479684	1144,374507	1129,369286	1006,088732	-0,578512116	False	0,578512116
ENSMUSG00000033161	Atp1a1	7332,066673	12376,34641	4913,561943	7309,466622	13443,38611	-0,577450661	False	0,577450661
ENSMUSG00000071604	Fam189a2	3836,432204	1845,839823	2570,998773	2077,994581	2067,939457	-0,577436339	False	0,577436339
ENSMUSG00000027236	Eif3j1	427,6356205	825,9220854	638,0821388	689,2969599	1040,495068	0,577360121	False	0,577360121
ENSMUSG00000019461	Plscr3	2676,409142	1535,638854	3993,230046	2514,699007	1429,642596	0,577257509	False	0,577257509
ENSMUSG00000033453	Adamts15	703,7145555	3066,475836	1049,925172	3151,232193	2808,268901	0,577224251	False	0,577224251
ENSMUSG00000020120	Plek	1102,67729	460,0193941	739,1209628	538,8640729	739,1430188	-0,577085185	False	0,577085185
ENSMUSG00000038236	Hoxa7	216,2754862	145,0165522	208,6671366	538,8640729	417,6217377	-0,576652573	False	0,576652573
ENSMUSG00000030417	Pdcd5	437,4663244	947,8896492	652,3593639	826,2582451	1201,848921	0,576494954	False	0,576494954
ENSMUSG00000026511	Srp9	459,5854082	1366,612939	685,3068065	1000,266435	1860,315013	0,576417023	False	0,576417023
ENSMUSG00000023046	Igfbp6	2560,898371	3818,449083	5698,809326	6970,43131	9605,299968	0,576336786	False	0,576336786
ENSMUSG00000027569	Mrgbp	333,4247079	542,6116026	544,7310514	624,1842178	497,1122389	0,576210606	False	0,576210606
ENSMUSG00000024588	Fech	1090,38891	1354,12807	731,4332262	1149,576689	1309,813632	-0,576044724	False	0,576044724
ENSMUSG00000036398	Ppp1r11	908,5208871	725,082761	1354,139892	731,9570324	478,1294327	0,575785199	False	0,575785199
ENSMUSG00000073758	Sh3d21	395,6858327	1119,796688	589,7592229	1982,570735	1780,824512	0,575770664	False	0,575770664
ENSMUSG00000034771	Tle2	1079,73898	424,4855369	1608,933448	971,0779647	631,1783081	0,575422059	False	0,575422059
ENSMUSG00000001128	Cfp	529,2195609	1148,607923	788,5421268	849,8335483	1478,286038	0,575321444	False	0,575321444
ENSMUSG00000003809	Gcdh	325,2324546	314,0424674	392,0745672	405,2706882	218,302272	-0,575144084	False	0,575144084
ENSMUSG00000029518	Rab35	1580,285655	2168,525661	1060,907652	1381,96182	3091,82457	-0,574886285	False	0,574886285
ENSMUSG00000016319	Slc25a5	2138,178102	2528,666106	1171,830709	1415,640825	3184,36575	0,574624021	False	0,574624021
ENSMUSG00000042541	Sem1	613,5997696	1221,596387	913,7424087	1067,624444	1897,094201	0,574489574	False	0,574489574
ENSMUSG00000034863	Ano8	355,5437917	176,7089113	529,3555781	313,2147424	125,7610915	0,574209868	False	0,574209868
ENSMUSG00000035847	Ids	3058,987369	1914,026414	1715,463513	1573,932146	2054,888778	-0,573993841	False	0,573993841
ENSMUSG00000019917	Sept10	335,0631586	148,8580503	225,1408579	213,3003622	163,726704	-0,573605169	False	0,573605169
ENSMUSG00000029334	Prkg2	421,9010432	397,5950504	786,3456306	854,3240823	283,5556685	-0,573272657	False	0,573272657
ENSMUSG00000023959	Clic5	12985,54065	4843,168694	8729,974047	7014,214016	2687,253511	-0,572856812	False	0,572856812
ENSMUSG00000052713	Zfp608	1081,377431	572,3832127	727,0402339	620,8163173	548,1285308	-0,572763043	False	0,572763043
ENSMUSG00000001247	Lsr	1157,565386	994,9480006	923,6266415	791,4566071	1721,503243	0,57257521	False	0,57257521
ENSMUSG00000037999	Arap2	1015,839405	375,5064365	683,1103104	577,0336114	427,1131409	-0,572481871	False	0,572481871
ENSMUSG00000030259	Rassf8	888,040254	579,1058343	509,5871126	597,2410142	529,1457245	-0,572311831	False	0,572311831
ENSMUSG00000020801	Med31	180,2295718	300,5972241	267,9725333	286,2715387	418,8081631	0,572249389	False	0,572249389
ENSMUSG00000042473	Tbc1d8b	294,1018922	197,8371507	196,5864076	171,7629232	130,506793	-0,5720027	False	0,5720027
ENSMUSG00000028108	Ecm1	2668,216889	3966,346759	4299,641262	4426,543832	4425,366709	0,571934869	False	0,571934869
ENSMUSG00000028398	Dmac1	240,852246	230,4898843	358,0288765	190,8476925	163,726704	0,571927574	False	0,571927574
ENSMUSG00000021102	Glrx5	466,9584361	958,4537689	702,8787759	693,7874939	1390,490559	0,571199693	False	0,571199693
ENSMUSG00000025586	Cpeb1	398,962734	340,9329539	321,7866896	592,7504802	176,7773833	0,571170932	False	0,571170932
ENSMUSG00000015289	Lage3	311,3056241	253,5388727	462,3624448	348,0163804	112,7104122	0,570692579	False	0,570692579
ENSMUSG00000031517	Gpm6a	947,0244775	174,7881623	445,8887235	637,6558196	244,4036306	-0,570623788	False	0,570623788
ENSMUSG00000044456	Rin3	2049,701767	3384,359801	1380,497846	2998,554039	4401,638202	-0,570225375	False	0,570225375
ENSMUSG00000003382	Etv3	663,5725145	808,6353441	446,9869716	597,2410142	1491,336717	-0,570021349	False	0,570021349
ENSMUSG00000041598	Cdc42ep4	1956,31008	8996,788484	2903,767943	6158,7673	7883,796725	0,569791105	False	0,569791105
ENSMUSG00000030830	Itgal	1670,440441	778,863734	1125,70429	620,8163173	504,2307913	-0,569366101	False	0,569366101
ENSMUSG00000032038	St3gal4	607,045967	489,7910041	462,3624448	391,7990864	900,496872	0,5689155	False	0,5689155
ENSMUSG00000068323	Slc4a5	1439,378899	324,6065871	970,8513093	683,6837925	233,7258021	-0,568124152	False	0,568124152
ENSMUSG00000044626	Liph	802,0215947	873,9408113	671,0295814	1143,963521	541,0099784	-0,567985879	False	0,567985879
ENSMUSG00000042050	Wdr60	385,8551288	415,8421663	260,2847967	431,0912583	493,5529628	-0,567968236	False	0,567968236
ENSMUSG00000034177	Rnf43	384,2166781	177,6692858	259,1865486	178,4987242	193,3873388	-0,567929299	False	0,567929299
ENSMUSG00000032422	Snx14	515,2927304	258,3407453	347,0463956	312,0921089	347,6226396	-0,567870196	False	0,567870196
ENSMUSG00000030060	Hmces	232,6599927	397,5950504	344,8498994	413,1291226	441,3502455	0,567745394	False	0,567745394
ENSMUSG00000067931	Zfp948	186,7833745	750,0524985	276,758518	434,4591588	982,360224	0,567261674	False	0,567261674
ENSMUSG00000018449	Rpain	202,3486557	767,3392398	299,8217279	753,2870686	802,0235645	0,567261674	False	0,567261674
ENSMUSG00000072941	Sod3	9254,788514	4186,272524	13709,43088	3585,691352	6245,34326	0,566896753	False	0,566896753
ENSMUSG00000000903	Vpreb3	516,931181	319,8047145	721,5489934	765,6360369	172,0316817	0,566686509	False	0,566686509
ENSMUSG00000021676	Iqgap2	336,7016092	521,4833632	227,3373541	397,4122538	401,0117822	-0,566635862	False	0,566635862
ENSMUSG00000030816	Rnf40	1298,472143	2601,654569	1923,032401	2241,89907	4153,675295	0,566568008	False	0,566568008
ENSMUSG00000036594	H2-Aa	10373,85031	3555,306465	6011,810031	2812,196881	7005,841935	-0,566321114	False	0,566321114
ENSMUSG00000054986	Sec14l3	21265,45103	4747,131242	14361,79024	8561,202958	5228,576699	-0,566275857	False	0,566275857
ENSMUSG00000007721	Ccdc124	580,0115312	970,9386376	997,2092634	858,8146162	1466,421784	0,566265162	False	0,566265162
ENSMUSG00000053964	Lgals4	298,1980189	791,3486028	441,4957311	849,8335483	937,2760591	0,566128816	False	0,566128816
ENSMUSG00000055320	Tead1	2705,082028	1610,548067	1813,207592	1825,402047	1827,095103	-0,566120613	False	0,566120613

ENSMUSG00000022895	Ets2	2850,084911	2840,787824	2265,685804	1925,316427	7292,95688	-0,565909328	False	0,565909328
ENSMUSG00000050069	Grem2	939,6514495	130,6109344	634,7873945	269,4320365	92,54118052	-0,565852229	False	0,565852229
ENSMUSG00000031618	Nr3c2	305,5710468	169,0259152	206,4706404	186,3571586	141,1846216	-0,565571204	False	0,565571204
ENSMUSG00000032193	Ldlr	1008,466377	1149,568298	899,4651836	681,4385255	1440,320425	-0,565507566	False	0,565507566
ENSMUSG00000029722	Agfg2	391,5897061	629,0453092	264,6777891	543,3546069	320,3348556	-0,565105707	False	0,565105707
ENSMUSG00000068747	Sort1	6118,793964	1790,138101	4136,002297	2491,123704	2228,106885	-0,565010334	False	0,565010334
ENSMUSG00000037754	Ppp1r16b	3010,653075	448,4948999	2035,053706	894,7388877	485,247985	-0,565009604	False	0,565009604
ENSMUSG00000034911	Ushbp1	2057,074795	203,5993978	3042,147202	954,2384625	54,575568	0,564495712	False	0,564495712
ENSMUSG00000017286	Glod4	521,0273077	191,1145291	352,5376361	324,4410772	149,4895993	-0,563581704	False	0,563581704
ENSMUSG00000022636	Alcam	4713,822529	2496,973747	3190,410694	2962,629768	2636,237219	-0,563155293	False	0,563155293
ENSMUSG00000057182	Scn3a	499,7274492	24,00936295	338,2604109	66,23537563	21,35565704	-0,563007127	False	0,563007127
ENSMUSG00000085795	Zfp703	1729,384664	1825,671959	1170,732461	1204,58573	3024,198322	-0,562847376	False	0,562847376
ENSMUSG00000031749	St3gal2	1315,675874	581,9869579	890,6791989	681,4385255	313,2163033	-0,562826309	False	0,562826309
ENSMUSG00000029119	Man2b2	1701,531003	908,514294	1110,328816	1005,879603	1153,20548	-0,561183841	False	0,561183841
ENSMUSG00000030796	Tead2	440,7432257	239,133255	650,1628678	546,7225073	154,2353009	0,560862768	False	0,560862768
ENSMUSG00000020818	Mfsd11	321,9555533	294,834977	373,4043497	406,3933217	218,302272	-0,56053439	False	0,56053439
ENSMUSG00000002769	Gnmt	98,30703918	365,9026913	144,9687475	561,3167426	401,0117822	0,560375288	False	0,560375288
ENSMUSG00000001065	Zfp276	485,8006186	766,3788653	780,8543902	716,2401636	847,1077294	0,560079094	False	0,560079094
ENSMUSG00000006057	Atp5g1	1090,38891	1133,241931	1193,795671	739,8154668	1396,422686	-0,559605426	False	0,559605426
ENSMUSG00000074207	Adh1	1970,23691	1036,244105	2903,767943	1370,735485	599,1448226	0,559557047	False	0,559557047
ENSMUSG00000041143	Tmco4	267,0674565	321,7254635	181,2109344	252,5925342	351,1819158	-0,559534175	False	0,559534175
ENSMUSG00000037936	Scarb1	2529,767808	2492,171874	1550,726299	2210,465332	3727,748579	0,559299585	False	0,559299585
ENSMUSG00000064326	Siva1	337,5208345	503,2362474	509,5871126	497,326634	551,6878069	0,559217136	False	0,559217136
ENSMUSG00000002320	Tm9sf1	753,6873004	790,3882283	541,4363071	547,8451408	1110,494166	0,559163825	False	0,559163825
ENSMUSG00000032330	Cox7a2	794,6485667	1532,757731	1170,732461	1173,151992	1570,827218	0,55902255	False	0,55902255
ENSMUSG00000040560	Wdr7	352,2668904	239,133255	217,4531213	234,6303984	129,3203676	-0,55885411	False	0,55885411
ENSMUSG00000030022	Adamts9	453,0316056	1073,698711	313,0007049	307,601575	2343,190148	-0,558548813	False	0,558548813
ENSMUSG00000011658	Fuz	515,2927304	111,4034441	758,8894284	244,7340998	84,23620278	0,558497458	False	0,558497458
ENSMUSG00000096956	Snhg18	391,5897061	246,8162511	576,5802459	478,2418647	195,7601896	0,558178569	False	0,558178569
ENSMUSG00000032718	Mansc1	305,5710468	141,1750541	207,5688885	156,0460544	75,93122504	-0,557917631	False	0,557917631
ENSMUSG00000018547	Pip4k2b	732,3874419	342,8537029	497,5063836	371,5916836	160,1674278	-0,557892017	False	0,557892017
ENSMUSG00000110206	Flt3l	486,619844	206,4805214	716,057753	371,5916836	74,74479965	0,557280798	False	0,557280798
ENSMUSG00000038570	Saxo2	488,2582946	718,3601394	314,098953	556,8262087	230,1665259	0,557062747	False	0,557062747
ENSMUSG00000003812	Dnase2a	401,42041	351,4970736	546,9275475	623,0615843	272,87784	-0,556858731	False	0,556858731
ENSMUSG00000050105	Grrp1	362,0975943	946,9292747	532,6503224	1452,68773	1124,731271	0,556810142	False	0,556810142
ENSMUSG00000024621	Csf1r	3077,010326	1291,703727	2092,162607	1301,132209	1130,663398	-0,556534301	False	0,556534301
ENSMUSG00000027709	Mccc1	334,2439332	100,8393244	227,3373541	191,970326	154,2353009	-0,55606662	False	0,55606662
ENSMUSG00000021408	Ripk1	444,020127	730,8450081	302,018224	541,1093399	1321,677886	-0,555989468	False	0,555989468
ENSMUSG00000031489	Adrb3	254,7790766	97,95820083	374,5025978	149,3102535	119,8289645	0,555728924	False	0,555728924
ENSMUSG00000059248	Sept9	2348,719011	1121,717437	1597,950967	940,7668606	1587,437174	-0,555650987	False	0,555650987
ENSMUSG00000055485	Soga1	677,499345	460,9797686	358,0288765	321,0731768	416,4353123	-0,555516116	False	0,555516116
ENSMUSG00000036845	Lin37	349,8092144	553,1757223	513,9801049	614,0805164	562,3656355	0,555144225	False	0,555144225
ENSMUSG00000023961	Enpp4	905,2439858	450,4156489	616,117177	441,1949597	417,6217377	-0,555101929	False	0,555101929
ENSMUSG00000060791	Gmfg	453,0316056	1041,045977	665,538341	976,6911322	891,0054688	0,554910078	False	0,554910078
ENSMUSG00000022876	Samsn1	158,110488	374,546062	107,6283126	222,2814301	367,7918713	-0,554875429	False	0,554875429
ENSMUSG00000030512	Snrpa1	171,2180932	322,685838	251,498812	356,9974483	315,5891541	0,554716421	False	0,554716421
ENSMUSG00000001473	Tubb6	257,2367525	363,9819423	181,2109344	175,1308237	974,0552462	-0,554663756	False	0,554663756
ENSMUSG00000027247	Arhgap1	2737,851041	1864,086939	1645,175635	1429,112427	1863,87429	-0,554574808	False	0,554574808
ENSMUSG00000027339	Rassf2	740,5796952	1024,719611	868,7142371	1126,001386	504,2307913	-0,554570775	False	0,554570775
ENSMUSG00000029173	Sepsecs	360,4591437	265,0633669	529,3555781	404,1480547	181,5230849	0,554401387	False	0,554401387
ENSMUSG00000022257	Laptm4b	1101,038839	605,0359463	661,1453486	501,8171679	749,8208473	-0,554247518	False	0,554247518
ENSMUSG00000049517	Rps23	2881,215473	5771,850853	4230,451633	5880,354196	6670,08355	0,554134133	False	0,554134133
ENSMUSG00000037972	Snn	1473,786362	270,8256141	1003,798752	487,2229326	68,81267269	-0,554057352	False	0,554057352
ENSMUSG00000029169	Dhx15	1720,373186	2409,579665	1171,830709	1704,157631	3376,566664	-0,553957387	False	0,553957387
ENSMUSG00000063235	Ptpmt1	258,8752032	466,7420157	379,9938382	417,6196565	683,3810254	0,553719244	False	0,553719244
ENSMUSG00000022474	Pmm1	258,8752032	404,317672	379,9938382	596,1183807	498,2986643	0,553719244	False	0,553719244
ENSMUSG00000000374	Trappc10	951,1206041	1088,104329	647,9663716	835,239313	1436,761149	-0,553709348	False	0,553709348
ENSMUSG00000002825	Qtrt1	173,6757692	344,7744519	254,7935563	269,4320365	858,9719833	0,552932305	False	0,552932305
ENSMUSG00000039323	Igfbp2	5868,930239	2278,008357	2120,717057	1859,081052	4001,812845	-0,552443862	False	0,552443862
ENSMUSG00000037166	Ppp1r14a	1229,657215	438,8911547	1803,32336	597,2410142	314,4027287	0,552401915	False	0,552401915
ENSMUSG00000063849	Ppcdc	323,594004	374,546062	426,1202579	533,2509055	220,6751228	-0,552260875	False	0,552260875
ENSMUSG00000064284	Cdpf1	262,1521045	203,5993978	384,3868306	242,4888328	109,151136	0,552154782	False	0,552154782
ENSMUSG00000004864	Mapk13	218,7331622	1572,133086	320,6884415	1142,840888	1556,590113	0,552000392	False	0,552000392
ENSMUSG00000027429	Sec23b	1235,391792	1449,205148	1186,107934	843,0977474	1735,740347	-0,551196841	False	0,551196841
ENSMUSG00000048222	Mfap1b	281,8135123	427,3666605	412,9412809	468,1381633	589,6534195	0,551195862	False	0,551195862
ENSMUSG00000042406	Atf4	2517,479428	6607,376683	3687,917078	4765,579145	11632,90096	0,550826228	False	0,550826228
ENSMUSG00000040022	Rab11fip2	432,5509724	238,1728804	295,4287355	268,309403	232,5393767	-0,550059992	False	0,550059992
ENSMUSG00000020407	Upp1	267,8866818	315,0028419	196,5864076	182,9892581	409,31676	-0,549863897	False	0,549863897
ENSMUSG00000034607	Pof1b	295,7403429	90,27520469	202,0776481	152,678154	58,13484417	-0,549421306	False	0,549421306
ENSMUSG00000018398	Sept8	1734,300016	793,2693518	2538,05133	1170,906725	481,6887088	0,549367755	False	0,549367755
ENSMUSG00000015247	Nipsnap3b	325,2324546	355,3385716	375,6008459	222,2814301	378,4696998	-0,549083803	False	0,549083803
ENSMUSG00000038618	Rassf7	663,5725145	1179,339908	970,8513093	1155,189856	1259,983766	0,548996226	False	0,548996226
ENSMUSG00000041607	Mbp	996,1779971	1156,29092	680,9138142	788,0887066	2053,702352	-0,548931344	False	0,548931344
ENSMUSG00000020782	Llgl2	994,5395464	1336,841329	679,8155661	1070,992345	2375,223633	-0,548885342	False	0,548885342
ENSMUSG00000079363	Gbp4	1028,127785	289,0727299	702,8787759	272,7999369	162,5402786	-0,548671789	False	0,548671789
ENSMUSG00000025351	Cd63	1856,36459	4450,375516	2714,869272	3042,336745	6518,2211	0,548402647	False	0,548402647
ENSMUSG00000024253	Dync2li1	407,1549873	221,8465136	260,2847967	278,4131043	227,7936751	-0,548350957	False	0,548350957
ENSMUSG00000030956	Fam53b	1745,769171	1123,638186	1193,795671	927,2952588	830,4977739	-0,548306888	False	0,548306888
ENSMUSG00000020038	Cry1	289,1865403	798,0712244	422,8255137	557,9488422	752,1936981	0,548062027	False	0,548062027
ENSMUSG00000047139	Cd24a	10565,54904	13073,57831	7227,570663	9769,156589	15779,4577	-0,547785025	False	0,547785025
ENSMUSG00000028978	Nos3	453,0316056	1101,549572	662,2435967	693,7874939	2025,228143	0,547750286	False	0,547750286
ENSMUSG00000060548	Tnfrsf19	791,3716654	117,1656912	541,4363071	248,1020002	99,65973287	-0,547563778	False	0,547563778
ENSMUSG00000026110	Mgat4a	316,220976	426,406286	216,3548732	241,3661993	624,0597558	-0,547533453	False	0,547533453
ENSMUSG00000025784	Clec3b	584,1076578	118,1260657	399,7623038	95,42384625	276,4371162	-0,547091862	False	0,547091862
ENSMUSG00000031902	Nfatc3	1291,099115	761,5769927	879,696718	596,1183807	883,8869165	-0,546666047	False	0,546666047
ENSMUSG00000026857	Ntmt1	136,8106295	286,1916063	232,8285945	199,8287604	419,9945885	0,546583913	False	0,546583913
ENSMUSG00000022635	Zcrb1	670,9455424	1306,109344	979,637294	1324,707513	2078,617285	0,546052023	False	0,546052023
ENSMUSG00000038717	Atp5l	711,0875834	1383,89968	1037,844443	1158,557757	1895,907775	0,545491051	False	0,545491051
ENSMUSG00000031274	Col4a5	996,9972224	289,0727299	683,1103104	601,7315481	317,9620049	-0,545470918	False	0,545470918
ENSMUSG00000025006	Sorbs1	429,2740711	245,8558766	171,3267016	294,1299731	185,082361	-0,545445225	False	0,545445225
ENSMUSG00000032606	Nicn1	479,246816	74,9092124	328,3761781	160,5365884	27,287784	-0,545419376	False	0,545419376
ENSMUSG00000027499	Pkia	440,7432257	136,3731815	302,018224	202,0740273	112,7104122	-0,54530279	False	0,54530279
ENSMUSG00000024515	Smad4	1060,896798	1220,636012	727,0402339	965,4647973	1487,777441	-0,545177211	False	0,545177211
ENSMUSG00000044340	Phlpp1	1225,561088	595,4322011	840,1597869	680,3158921	817,4470946	-0,544706758	False	0,544706758
ENSMUSG00000042111	Ccdc115	343,2554118	597,3529502	518,3730973	500,6945344	825,7520723	0,544648241	False	0,544648241
ENSMUSG00000025290	Rps24	6932,284713	11827,97256	10110,47189	12267,01609	14881,33368	0,544447521	False	0,544447521
ENSMUSG00000022220	Adcy4	2535,502386	1829,513457	3697,80131	2919,969695	1443,879701	0,544396076	False	0,544396076
ENSMUSG00000035762	Tmem161b	167,9411919	352,4574481	244,9093235	301,9884075	392,7068045	0,544291565	False	0,544291565
ENSMUSG00000025151	Maged1	2003,005923	1693,140275	1790,144382	1240,510001	2920,979313	0,544285453	False	0,544285453
ENSMUSG00000020361	Hspa4	2122,612821	2069,607086	1563,905276	1652,51649	3095,383846	0,544277085	False	0,544277085
ENSMUSG00000019960	Dusp6	1194,430526	920,0387882	1741,821467	665,7216568	672,7031968	0,544273816	False	0,544273816
ENSMUSG00000031848	Lsm4	435,8278737	812,4768422	635,8856426	635,4105526	957,4452907	0,543930582	False	0,543930582
ENSMUSG00000029033	Acap3	480,8852667	701,0733981	767,6754131	840,8524804	754,5665488	0,543872768	False	0,543872768
ENSMUSG00000045319	Proser2	140,9067562	409,1195446	205,3723923	328,9316112	784,2271836	0,54350147	False	0,54350147
ENSMUSG00000026281	Dtymk	319,4978773	258,3407453	465,6571891	259,3283351	230,1665259	0,543461905	False	0,543461905
ENSMUSG00000037336	Mfsd2b	159,7489387	543,5719772	232,8285945	374,9595841	523,2135975	0,543461905	False	0,543461905
ENSMUSG00000027500	Stmn2	1043,693066	62,42434367	1521,073601	243,6114663	88,98190434	0,543392463	False	0,543392463
ENSMUSG00000038895	Zfp653	256,4175272	615,600066	373,4043497	528,7603716	879,1412149	0,542243854	False	0,542243854
ENSMUSG00000025607	Copg2	500,5466745	263,1426179	343,7516514	246,9793668	168,4724056	-0,542137958	False	0,542137958
ENSMUSG00000004788	Eif2b2	684,0531477	936,365155	470,0501814	657,8632224	1526,929479	-0,541293636	False	0,541293636

ENSMUSG00000039485	Tspyl4	517,7504064	209,3616449	355,8323803	304,2336745	158,9810024	-0,541058981	False	0,541058981
ENSMUSG00000040158	Tax1bp3	574,2769539	394,7139269	350,3411399	294,1299731	380,8425506	-0,540939243	False	0,540939243
ENSMUSG00000034460	Six4	223,6485141	261,2218689	153,7547322	356,9974483	232,5393767	-0,540602359	False	0,540602359
ENSMUSG00000025626	Phf6	282,6327377	160,3825445	194,3899115	117,876516	134,0660692	-0,539975237	False	0,539975237
ENSMUSG00000037035	Inhbb	462,8623095	3190,364149	318,4919454	736,4475663	5493,149562	-0,539326203	False	0,539326203
ENSMUSG00000041346	Wrap53	185,9641491	318,84434	270,1690295	304,2336745	341,6905127	0,538837783	False	0,538837783
ENSMUSG00000036887	C1qa	676,6801197	465,7816412	836,8650426	904,8425891	803,2099899	-0,538820227	False	0,538820227
ENSMUSG00000032554	Trf	6280,181353	4203,559265	4023,980992	3467,814836	4323,334126	-0,538661882	False	0,538661882
ENSMUSG00000027087	Itgav	665,2109651	795,1901008	457,9694525	527,6377381	1001,34303	-0,538560579	False	0,538560579
ENSMUSG00000040907	Atp1a3	331,7862572	141,1750541	228,4356022	147,0649866	106,7782852	-0,538466615	False	0,538466615
ENSMUSG00000034612	Chst11	500,5466745	421,6044134	481,0326623	344,64848	860,1584087	-0,53837895	False	0,53837895
ENSMUSG00000030554	Synm	1317,314325	163,263668	907,1529202	319,9505433	69,99909808	-0,538181955	False	0,538181955
ENSMUSG00000000290	Itgb2	1608,958541	1934,194279	1108,13232	1242,755268	2358,613678	-0,537996991	False	0,537996991
ENSMUSG00000028347	Tmeff1	286,7288643	80,67145951	416,2360251	191,970326	35,59276174	0,537714691	False	0,537714691
ENSMUSG00000023170	Gps2	1435,282772	3299,846844	2083,376622	2506,840573	6333,138739	0,537588668	False	0,537588668
ENSMUSG00000021903	Galnt15	529,2195609	2511,379364	364,618365	2872,819089	2884,200126	-0,537479159	False	0,537479159
ENSMUSG00000001416	Cct3	1011,743278	1174,538035	987,3250306	697,1553943	1477,099612	-0,537291094	False	0,537291094
ENSMUSG00000001467	Cyp51	423,5394938	614,6396915	423,9237617	328,9316112	293,0470716	0,537244432	False	0,537244432
ENSMUSG00000042750	Bex2	288,3673149	141,1750541	418,4325213	214,4229957	115,083263	0,537087333	False	0,537087333
ENSMUSG00000016427	Ndufa1	258,8752032	509,958869	375,6008459	387,3085524	494,7393882	0,536943531	False	0,536943531
ENSMUSG00000039963	Ccdc40	752,0488498	1071,777962	518,3730973	825,1356117	947,9538876	-0,536835529	False	0,536835529
ENSMUSG00000033965	Slc16a2	1194,430526	615,600066	823,6860656	727,4664984	748,6344219	-0,536156454	False	0,536156454
ENSMUSG00000097328	Tnfsf12	683,2339223	375,5064365	990,6197749	487,2229326	256,2678845	0,535951814	False	0,535951814
ENSMUSG00000028826	Tmem57	844,6213117	1265,773615	1224,546617	1408,905024	1583,877897	0,535871142	False	0,535871142
ENSMUSG00000029070	Mxra8	6478,433882	3864,54706	4306,230751	4056,074782	4469,264449	-0,535607681	False	0,535607681
ENSMUSG00000025583	Rptor	589,0230098	899,8709233	406,3517923	609,5899825	615,7547781	-0,535594735	False	0,535594735
ENSMUSG00000047434	Xxylt1	335,8823839	158,4617955	231,7303464	120,1217829	134,0660692	-0,535509146	False	0,535509146
ENSMUSG00000033059	Pygb	1343,529536	605,9963208	926,9213858	626,4294848	468,6380295	-0,535509146	False	0,535509146
ENSMUSG00000050896	Rtn4rl2	55,7073222	581,0265833	38,43868306	195,3382264	1592,182875	-0,53530806	False	0,53530806
ENSMUSG00000032265	Fam46a	444,8393523	811,5164677	644,6716273	739,8154668	1276,593721	0,53528007	False	0,53528007
ENSMUSG00000055044	Pdlim1	2122,612821	1876,571808	2321,696457	1465,036698	4447,908792	-0,534904434	False	0,534904434
ENSMUSG00000086290	Snhg12	142,5452068	290,0331044	206,4706404	397,4122538	408,1303346	0,53451712	False	0,53451712
ENSMUSG00000009555	Cdk9	1182,961372	2554,596218	1713,267016	2649,415025	3897,40741	0,534347051	False	0,534347051
ENSMUSG00000017670	Elmo2	1520,482206	1049,689348	1049,925172	876,776752	1023,885113	-0,534242423	False	0,534242423
ENSMUSG00000028164	Manba	425,9971698	232,4106333	254,7935563	236,8756654	294,233497	-0,533882346	False	0,533882346
ENSMUSG00000036087	Slain2	1801,476493	951,7311473	1244,315083	994,6532679	1047,61362	-0,533827978	False	0,533827978
ENSMUSG00000032841	Prr5l	483,3429427	87,39408113	333,8674186	138,0839187	10,67782852	-0,533771866	False	0,533771866
ENSMUSG00000020063	Sirt1	380,1205515	558,9379694	550,2222918	683,6837925	568,2977624	0,533557563	False	0,533557563
ENSMUSG00000053799	Exoc6	526,761885	363,9819423	363,5201169	335,6674121	279,9963923	-0,533284082	False	0,533284082
ENSMUSG00000009894	Snap47	583,2884325	221,8465136	403,0570481	262,6962355	129,3203676	-0,533225414	False	0,533225414
ENSMUSG00000106918	Mrpl33	227,7446408	412,0006682	329,4744262	395,1669868	404,5710584	0,532749382	False	0,532749382
ENSMUSG00000024697	Gna14	365,3744956	197,8371507	252,5970601	122,3670499	204,0651673	-0,532538084	False	0,532538084
ENSMUSG00000025986	Slc39a10	573,4577286	203,5993978	396,4675596	207,6871948	244,4036306	-0,53248432	False	0,53248432
ENSMUSG00000040605	Bace2	346,5323131	109,482695	210,8636328	108,8954481	239,657929	-0,532013214	False	0,532013214
ENSMUSG00000020034	Tcp11l2	746,3142725	1183,181406	516,1766011	1170,906725	1307,440781	-0,531918533	False	0,531918533
ENSMUSG00000028744	Pqlc2	198,252529	297,7161006	286,6427508	295,2526066	290,6742209	0,531914524	False	0,531914524
ENSMUSG00000025027	Xpnpep1	631,6227268	847,0503248	437,1027388	661,2311228	1089,138509	-0,531090665	False	0,531090665
ENSMUSG00000024981	Acsl5	2549,429216	1227,358634	1764,884676	1141,718254	1061,850725	-0,530600367	False	0,530600367
ENSMUSG00000020032	Nuak1	548,8809688	170,9466642	379,9938382	193,0929595	215,9294212	-0,530517293	False	0,530517293
ENSMUSG00000032068	Plet1	1374,660098	887,3860546	1227,841362	1001,389069	1984,88968	0,529983886	False	0,529983886
ENSMUSG00000022139	Mbnl2	3205,628703	3646,542045	2220,657633	4032,499479	3714,6979	-0,529620348	False	0,529620348
ENSMUSG00000002763	Pex6	834,7906077	331,3292087	1204,778152	734,2022994	282,3692431	0,529281239	False	0,529281239
ENSMUSG00000046402	Rbp1	781,5409615	480,187259	1127,900786	638,7784531	507,7900675	0,529246775	False	0,529246775
ENSMUSG00000030717	Nupr1	2904,153783	4190,114022	5200,204694	4615,146258	7203,974976	0,528871653	False	0,528871653
ENSMUSG00000001248	Gramd1a	1207,538131	2273,206484	1741,821467	2307,011812	3237,754893	0,528528011	False	0,528528011
ENSMUSG00000075705	Msrb1	1994,81367	4308,240087	2877,409989	3312,891415	4571,297033	0,528516804	False	0,528516804
ENSMUSG00000002129	Sf3a1	1241,945595	819,1994638	861,0265005	685,9290595	829,3113485	-0,528472429	False	0,528472429
ENSMUSG00000046010	Zfp830	167,1219666	241,054004	255,8918044	250,3472672	266,945713	0,528455019	False	0,528455019
ENSMUSG00000002108	Nr1h3	918,351591	427,3666605	1324,487193	597,2410142	162,5402786	0,528315394	False	0,528315394
ENSMUSG00000027953	Slc50a1	1178,04602	895,0690507	1698,989791	1563,828445	548,1285308	0,528281286	False	0,528281286
ENSMUSG00000020573	Pik3cg	290,8249909	197,8371507	144,9687475	151,5555205	201,6923165	-0,527995122	False	0,527995122
ENSMUSG00000052270	Fpr2	217,9139369	1369,494063	314,098953	1064,256544	554,0606577	0,527460666	False	0,527460666
ENSMUSG00000023939	Mrpl14	501,3658998	326,5273361	722,6472415	504,0624349	176,7773833	0,527427696	False	0,527427696
ENSMUSG00000022594	Lynx1	837,2482837	382,2290581	580,9732382	429,9686248	188,6416372	-0,527183804	False	0,527183804
ENSMUSG00000055862	Izumo4	415,3472406	220,8861391	598,5452076	475,9965977	183,8959356	0,527142247	False	0,527142247
ENSMUSG00000106864	Gtf3c2	907,7016618	1546,202974	1308,013472	1716,506599	1557,776539	0,527087296	False	0,527087296
ENSMUSG00000026895	Ndufa8	670,1263171	1082,342082	992,816271	965,4647973	1248,119512	0,526790591	False	0,526790591
ENSMUSG00000019947	Arid5b	1888,314378	1578,855707	1299,227487	1603,120617	2720,473422	0,526758761	False	0,526758761
ENSMUSG00000031167	Rbm3	1099,400388	5809,305459	1583,673742	4202,017135	7526,682682	0,526558258	False	0,526558258
ENSMUSG00000028066	Pmf1	229,3830914	327,4877106	159,2459727	235,7530319	404,5710584	-0,526502162	False	0,526502162
ENSMUSG00000001173	Ocrl	661,9340638	621,3623131	671,0295814	953,115829	308,4706017	0,525964035	False	0,525964035
ENSMUSG00000029840	Mtpn	3088,479481	1862,16619	2102,046839	1648,025956	2145,057107	-0,525880689	False	0,525880689
ENSMUSG00000023904	Hcfc1r1	2015,294303	1309,950842	2901,571447	1792,845676	1869,806417	0,525843916	False	0,525843916
ENSMUSG00000045826	Ptprcap	518,5696317	133,492058	360,2253727	124,6123169	136,43892	-0,52563792	False	0,52563792
ENSMUSG00000034810	Scn7a	4921,086537	618,4811895	3418,846296	1322,462246	513,7221944	-0,525467322	False	0,525467322
ENSMUSG00000026068	Il18rap	224,4677395	986,3046299	155,9512284	511,9208693	1068,969278	-0,525413199	False	0,525413199
ENSMUSG00000031951	Tmem231	335,8823839	233,3710079	211,9618809	199,8287604	175,5909579	-0,525330789	False	0,525330789
ENSMUSG00000060470	Adgrg3	189,2410504	359,1800697	272,3655257	294,1299731	304,9113256	0,525319034	False	0,525319034
ENSMUSG00000025855	Prkar1b	760,241103	356,2989462	528,25733	361,4879822	403,384633	-0,525216147	False	0,525216147
ENSMUSG00000031101	Sash3	602,130615	297,7161006	418,4325213	197,5834934	249,1493322	-0,525081488	False	0,525081488
ENSMUSG00000044786	Zfp36	3330,970178	5838,116695	2315,106968	3893,292927	12120,5218	-0,524863584	False	0,524863584
ENSMUSG00000040998	Npnt	7418,085332	5124,558428	4349,062426	3498,12594	5156,20475	-0,52473731	False	0,52473731
ENSMUSG00000006356	Crip2	16596,68589	11720,41062	23853,94846	17803,84444	11553,41046	0,523332904	False	0,523332904
ENSMUSG00000051682	Treml4	301,4749202	134,4524325	209,7653847	85,32014488	85,42262817	-0,523261363	False	0,523261363
ENSMUSG00000038563	Efl1	214,6370356	274,6671121	149,3617399	241,3661993	295,4199224	-0,523088396	False	0,523088396
ENSMUSG00000020850	Prpf8	4023,215579	3570,672458	2864,231012	2602,264419	5781,450932	0,523082557	False	0,523082557
ENSMUSG00000020109	Dnajb12	855,2712409	1293,624476	595,2504634	1000,266435	1797,434468	-0,522885191	False	0,522885191
ENSMUSG00000034390	Cmip	804,4792707	1505,867244	560,1065246	1005,879603	2496,239023	-0,522354013	False	0,522354013
ENSMUSG00000021831	Ero1l	591,4806858	306,3594712	411,8430328	354,7521813	307,2841763	-0,522236477	False	0,522236477
ENSMUSG00000014551	Mrps25	264,6097805	131,571309	379,9938382	143,6970861	39,15203791	0,522109638	False	0,522109638
ENSMUSG00000029321	Slc10a6	299,8364695	1133,241931	430,5132503	1222,547865	1980,143978	0,52188177	False	0,52188177
ENSMUSG00000055401	Fbxo6	651,2841346	1132,281557	940,1003628	1167,538825	934,9032083	0,521529926	False	0,521529926
ENSMUSG00000074622	Mafb	244,9483726	223,7672627	271,2672776	170,6402898	334,5719603	-0,521519386	False	0,521519386
ENSMUSG00000026628	Atf3	124,5222496	228,5691353	86,76159891	139,2065522	759,3122504	-0,521275001	False	0,521275001
ENSMUSG00000042737	Dpm3	325,2324546	466,7420157	517,2748492	483,8550321	563,5520608	0,521154114	False	0,521154114
ENSMUSG00000004558	Ndrg2	1257,510876	2265,523488	1804,421608	2282,313876	1817,603699	0,520965589	False	0,520965589
ENSMUSG00000032096	Arcn1	2662,482311	2820,619959	2199,790919	1855,713151	4026,727778	-0,520798219	False	0,520798219
ENSMUSG00000024165	Jpt2	511,1966038	356,2989462	277,8567661	202,0740273	329,8262588	-0,520790035	False	0,520790035
ENSMUSG00000030342	Cd9	3614,422141	7262,352105	5570,314299	5185,444068	8288,367783	0,52070253	False	0,52070253
ENSMUSG00000021096	Ppm1a	1449,209603	1994,697874	1010,38824	1618,837486	2911,48791	-0,520356517	False	0,520356517
ENSMUSG00000042349	Ikbke	352,2668904	1260,011368	505,1941202	756,6549691	1369,134902	0,520168966	False	0,520168966
ENSMUSG00000031015	Swap70	1379,57545	650,1735486	962,0653246	873,4088515	745,0751457	-0,520017599	False	0,520017599
ENSMUSG00000028184	Adgrl2	2026,763458	990,146128	1413,445289	928,4178923	855,4127071	-0,519961682	False	0,519961682
ENSMUSG00000031783	Polr2c	494,8120972	807,6749696	709,4682645	736,4475663	906,4289989	0,51985738	False	0,51985738
ENSMUSG00000040952	Rps19	2877,938572	6110,863058	4126,118064	5206,774105	8689,379566	0,519749304	False	0,519749304
ENSMUSG00000051343	Rab11fip5	643,9111067	598,3133247	449,1834678	612,9578829	971,6823954	-0,519556705	False	0,519556705
ENSMUSG00000026077	Npas2	338,3400599	52,82059849	189,9969191	42,66007244	236,0986529	-0,519084194	False	0,519084194
ENSMUSG00000001098	Kctd10	5273,353427	4735,606748	3100,354351	2800,970546	7556,343317	0,51896756	False	0,51896756
ENSMUSG00000071650	Ganab	2964,776457	1729,634507	2069,099397	1752,43087	1691,842608	-0,518920378	False	0,518920378
ENSMUSG00000040511	Pvr	643,0918813	2372,125059	921,4301453	1726,6103	2722,846273	0,518849921	False	0,518849921

ENSMUSG00000020159	Gabrp	2425,726192	1693,140275	1640,782643	902,5973221	998,9701794	-0,518715209	False	0,518715209
ENSMUSG00000027111	Itga6	2319,226899	749,092124	1618,817681	746,5512677	634,7375843	-0,51870346	False	0,51870346
ENSMUSG00000054843	Atml1	1024,850883	632,8868073	539,2398109	504,0624349	715,4145109	-0,518562726	False	0,518562726
ENSMUSG00000041445	Mmrn2	945,3860268	413,9214172	1354,139892	716,2401636	404,5710584	0,51840134	False	0,51840134
ENSMUSG00000064307	Lrrc51	466,9584361	332,2895832	668,8330852	510,7982358	139,9981962	0,518352073	False	0,518352073
ENSMUSG00000004565	Pnpla6	996,9972224	819,1994638	1375,006605	1136,105087	696,4317047	-0,517607603	False	0,517607603
ENSMUSG00000000093	Tbx2	5026,766604	4565,620458	7195,721469	3720,40737	4171,471676	0,517508639	False	0,517508639
ENSMUSG00000018846	Pank3	1001,912574	521,4833632	450,2817158	458,034462	699,9909808	-0,517348388	False	0,517348388
ENSMUSG00000017631	Abr	1574,551078	1088,104329	1100,444584	973,3232317	1034,562941	-0,516854063	False	0,516854063
ENSMUSG00000005800	Mmp8	444,8393523	1938,996152	635,8856426	3148,986926	825,7520723	0,515482915	False	0,515482915
ENSMUSG00000057278	Snrpg	247,4060486	461,9401431	353,6358841	370,4690501	484,0615596	0,515383903	False	0,515383903
ENSMUSG00000000711	Rab5b	2325,780702	1023,759236	1627,603666	1387,574988	762,8715266	-0,514965637	False	0,514965637
ENSMUSG00000038781	Stap2	757,783427	1147,647549	1228,93961	1462,791431	530,3321499	-0,514889378	False	0,514889378
ENSMUSG00000020232	Hmg20b	1196,068977	2226,148133	1708,874024	2461,935233	2353,867976	0,514745456	False	0,514745456
ENSMUSG00000028053	Ash1l	1400,056083	945,9689002	903,8581759	980,0590326	644,2289875	-0,514544064	False	0,514544064
ENSMUSG00000038550	Ciart	344,8938625	492,6721277	709,4682645	656,7405889	1001,34303	0,5144754	False	0,5144754
ENSMUSG00000038188	Scarf1	901,1478592	758,6958692	1287,146758	1022,719105	529,1457245	0,514340811	False	0,514340811
ENSMUSG00000022181	C6	626,7073748	370,7045639	461,2641967	461,4023624	894,564745	0,513393932	False	0,513393932
ENSMUSG00000044876	Zfp444	544,7848421	391,8328033	777,5596459	602,8541816	265,7592876	0,513266784	False	0,513266784
ENSMUSG00000023336	Wfdc1	525,1234343	172,8674132	749,0051956	120,1217829	281,1828177	0,512319147	False	0,512319147
ENSMUSG00000049792	Bag5	486,619844	418,7232898	355,8323803	368,2237832	694,0588539	0,51226285	False	0,51226285
ENSMUSG00000022426	Josd1	484,9813933	422,5647879	398,6640557	364,8558827	691,6860031	0,512187864	False	0,512187864
ENSMUSG00000047260	Emc6	1039,596939	1766,128738	1482,634918	1663,742825	2264,886072	0,512139103	False	0,512139103
ENSMUSG00000020288	Ahsa2	321,9555533	266,984116	459,0677005	306,4789415	167,2859802	0,511845394	False	0,511845394
ENSMUSG00000062906	Hdac10	391,5897061	274,6671121	527,159082	432,2138918	405,7574838	-0,511658664	False	0,511658664
ENSMUSG00000030786	Itgam	348,9899891	1902,50192	497,5063836	1567,196345	1537,607307	0,511529384	False	0,511529384
ENSMUSG00000001663	Gstt1	1776,080508	702,0337726	2531,461842	1357,263884	854,2262817	0,511273759	False	0,511273759
ENSMUSG00000015968	Cacna1d	579,1923059	274,6671121	374,5025978	406,3933217	214,7429958	-0,511165743	False	0,511165743
ENSMUSG00000030401	Rtn2	327,6901306	203,5993978	336,0639148	467,0155299	154,2353009	0,511138303	False	0,511138303
ENSMUSG00000059714	Flot1	1278,810735	1877,532183	1979,043054	2395,699858	1822,349401	0,510996835	False	0,510996835
ENSMUSG00000057719	Sh3rf2	799,5639187	247,7766256	561,2047727	340,157946	67,6262473	-0,510686092	False	0,510686092
ENSMUSG00000000266	Mid2	1372,202422	707,7960197	963,1635727	886,8804533	656,0932414	-0,510640583	False	0,510640583
ENSMUSG00000044328	Trp53i13	285,909639	200,7182742	379,9938382	267,1867695	298,9791986	-0,510387287	False	0,510387287
ENSMUSG00000018446	C1qbp	343,2554118	617,520815	488,7203989	502,9398014	976,428097	0,509726857	False	0,509726857
ENSMUSG00000050732	Vamp8	4534,412182	8559,818078	6455,502258	7970,697745	11878,49102	0,509613807	False	0,509613807
ENSMUSG00000042312	S100a13	546,4232928	777,9033595	929,117882	1095,690282	924,2253798	0,509571954	False	0,509571954
ENSMUSG00000037337	Map4k1	425,1779445	157,5014209	298,7234798	144,8197196	166,0995548	-0,509256122	False	0,509256122
ENSMUSG00000022008	Gpalpp1	656,1994866	715,4790159	486,5239027	471,5060638	933,7167829	0,50885055	False	0,50885055
ENSMUSG00000045624	Esf1	307,2094975	537,80973	437,1027388	560,1941091	1051,172897	0,5087496	False	0,5087496
ENSMUSG00000026395	Ptprc	2410,980136	1245,60575	1694,596799	1091,199748	857,7855579	-0,508677716	False	0,508677716
ENSMUSG00000053332	Gas5	668,4878665	1297,465974	951,0828437	1296,641675	1449,811828	0,508669637	False	0,508669637
ENSMUSG00000006589	Aprt	615,2382202	887,3860546	875,3037257	946,3800281	1095,070636	0,508638581	False	0,508638581
ENSMUSG00000022672	Prkdc	240,852246	111,4034441	342,6534033	162,7818554	73,55837426	0,508601639	False	0,508601639
ENSMUSG00000024175	Tekt4	280,1750617	398,5554249	208,6671366	278,4131043	189,8280626	0,508451818	False	0,508451818
ENSMUSG00000020173	Cobl	1100,219614	621,3623131	733,6297224	698,2780278	773,5493551	-0,508226279	False	0,508226279
ENSMUSG00000026072	Il1r1	935,5553229	759,6562437	988,4232787	657,8632224	1593,3693	-0,508035306	False	0,508035306
ENSMUSG00000035764	Fbxo45	269,5251324	215,123892	265,7760372	194,2155929	383,2154014	0,507735738	False	0,507735738
ENSMUSG00000043822	Adamtsl5	1250,957074	565,6605911	1778,063654	1138,350354	291,8606462	0,507274688	False	0,507274688
ENSMUSG00000029576	Radil	525,9426596	273,7067376	370,1096055	240,2435658	361,8597443	-0,506952941	False	0,506952941
ENSMUSG00000042363	Lgalsl	624,2496988	698,1922745	439,299235	654,4953219	804,3964153	-0,506919233	False	0,506919233
ENSMUSG00000021779	Thrb	343,2554118	188,2334055	241,6145792	225,6493305	81,863352	-0,506574956	False	0,506574956
ENSMUSG00000026594	Ralgps2	555,4347714	315,0028419	390,9763191	294,1299731	338,1312365	-0,506536268	False	0,506536268
ENSMUSG00000029071	Dvl1	1131,350176	1606,706569	1693,498551	1866,939486	1867,433566	0,506060933	False	0,506060933
ENSMUSG00000028643	Svbp	249,0444993	404,317672	374,5025978	353,6295479	498,2986643	0,505835279	False	0,505835279
ENSMUSG00000053113	Socs3	1161,661513	4503,196115	818,1948251	1765,902472	10687,31992	-0,505673437	False	0,505673437
ENSMUSG00000030203	Dusp16	887,2210286	1149,568298	624,9031617	738,6928333	2123,70145	-0,505660921	False	0,505660921
ENSMUSG00000025791	Pgm2	661,1148385	842,2484522	465,6571891	588,2599463	965,7502685	-0,505632644	False	0,505632644
ENSMUSG00000005481	Ddx39	204,8063316	423,5251624	298,7234798	290,7620727	527,9592991	0,505578778	False	0,505578778
ENSMUSG00000024665	Fads2	1074,004403	342,8537029	756,6929322	340,157946	266,945713	-0,505220033	False	0,505220033
ENSMUSG00000015672	Mrpl32	276,078935	391,8328033	400,8605519	432,2138918	408,1303346	0,505157368	False	0,505157368
ENSMUSG00000026380	Tfcp2l1	621,7920228	453,2967725	766,577165	882,3899194	242,0307798	0,504984201	False	0,504984201
ENSMUSG00000028639	Ybx1	10050,25631	14261,56159	16205,74878	19208,25893	21948,86974	0,504899666	False	0,504899666
ENSMUSG00000027184	Caprin1	3350,631586	1881,373681	2361,233388	1924,193794	1465,235358	-0,504892419	False	0,504892419
ENSMUSG00000025374	Nabp2	926,5438443	1509,708742	1383,79259	1314,603811	1847,264334	0,504696921	False	0,504696921
ENSMUSG00000024309	Pfdn6	371,1090729	629,0453092	587,5627268	526,5151046	743,8887203	0,504631647	False	0,504631647
ENSMUSG00000067925	Rtl8a	1417,259815	1052,570472	1870,316493	999,1438019	1829,467953	-0,504340022	False	0,504340022
ENSMUSG00000032085	Tagln	1800,657268	3395,884295	2580,883005	2553,991179	4695,871699	0,504229934	False	0,504229934
ENSMUSG00000025902	Sox17	1515,566854	608,8774444	2149,271507	1091,199748	510,1629182	0,50399025	False	0,50399025
ENSMUSG00000026600	Soat1	2010,378951	533,968232	1417,838281	709,5043627	450,8416487	-0,503774484	False	0,503774484
ENSMUSG00000038497	Tmco3	501,3658998	286,1916063	353,6358841	333,4221451	300,165624	-0,5035992	False	0,5035992
ENSMUSG00000066643	Wdr35	359,6399183	189,19378	253,6953082	229,017231	134,0660692	-0,503456323	False	0,503456323
ENSMUSG00000035597	Prpf39	856,0904662	1510,669117	1213,564137	1898,373224	1697,774735	0,503415192	False	0,503415192
ENSMUSG00000037361	Sf3b6	477,6083654	1007,432869	718,2542492	676,9479916	1288,457975	0,503216893	False	0,503216893
ENSMUSG00000020576	Nbas	511,1966038	276,5878612	273,4637738	215,5456292	360,6733189	-0,503185549	False	0,503185549
ENSMUSG00000001288	Rarg	2530,587034	1459,769267	3585,780005	2240,776437	1515,065225	0,502814885	False	0,502814885
ENSMUSG00000042426	Dhx29	356,363017	202,6390233	251,498812	162,7818554	223,0479736	-0,502796038	False	0,502796038
ENSMUSG00000034947	Tmem106a	586,5653338	236,2521314	414,039529	262,6962355	198,1330403	-0,502523299	False	0,502523299
ENSMUSG00000041881	Ndufa7	1142,000105	1854,483194	1626,505417	1617,714852	2374,037208	0,502394549	False	0,502394549
ENSMUSG00000030983	Bccip	376,8436502	657,8565448	533,7485705	568,0525435	709,482384	0,502194217	False	0,502194217
ENSMUSG00000037490	Slc2a12	240,0330207	339,9725793	207,5688885	253,7151677	135,2524946	0,502185503	False	0,502185503
ENSMUSG00000019969	Psen1	1450,848053	1385,820429	1004,897	1099,058182	2054,888778	0,502163875	False	0,502163875
ENSMUSG00000039105	Atp6v1g1	1204,26123	2382,689179	1705,57928	2136,371522	2733,524101	0,502113439	False	0,502113439
ENSMUSG00000097589	Dleu2	538,2310395	333,2499577	762,1841727	580,4015119	212,370145	0,501914057	False	0,501914057
ENSMUSG00000045039	Megf8	1376,298549	753,8939966	971,9495574	926,1726253	673,8896222	-0,501840108	False	0,501840108
ENSMUSG00000034903	Cobll1	718,4606114	703,9545216	507,3906164	877,8993855	785,413609	-0,501812226	False	0,501812226
ENSMUSG00000041625	Ggact	203,9871063	530,1267339	288,839247	642,1463536	405,7574838	0,501788822	False	0,501788822
ENSMUSG00000024899	Papss2	398,1435087	517,6418652	319,5901934	824,0129782	281,1828177	-0,501780098	False	0,501780098
ENSMUSG00000020846	Rflnb	883,124902	1223,517136	623,8049137	904,8425891	1306,254356	-0,501522579	False	0,501522579
ENSMUSG00000027508	Pag1	1081,377431	1008,393244	819,2930732	712,8722631	1530,488755	0,501122293	False	0,501122293
ENSMUSG00000021967	Mrpl57	367,8321716	746,2110004	520,5695934	556,8262087	843,5484532	0,501043379	False	0,501043379
ENSMUSG00000019054	Fis1	1611,416217	3311,371338	2279,963029	2757,18784	4224,860818	0,500681251	False	0,500681251
ENSMUSG00000024085	Man2a1	1758,876776	2268,404611	1243,216835	2082,485115	2417,934947	-0,500576468	False	0,500576468
ENSMUSG00000018678	Sp2	702,8953302	590,6303285	308,6077126	336,7900456	994,2244779	-0,500261753	False	0,500261753
ENSMUSG00000025492	Ifitm3	6448,122545	10128,10967	9252,740137	9120,274434	14199,13908	0,500198074	False	0,500198074
ENSMUSG00000032434	Cmtm6	1667,942765	1010,313993	1115,820057	862,1825167	1179,306839	-0,500130648	False	0,500130648
ENSMUSG00000034789	Rab24	491,5351959	490,7513787	673,2260776	548,9677743	347,6226396	-0,499772679	False	0,499772679
ENSMUSG00000028680	Plk3	209,7216836	998,7894987	296,5269836	554,5809417	1694,215459	0,499687362	False	0,499687362
ENSMUSG00000021952	Xpo4	371,1090729	339,9725793	262,4812929	345,7711135	561,1792101	-0,499628667	False	0,499628667
ENSMUSG00000001750	Tcirg1	1574,551078	2226,148133	2467,763452	2365,388753	2949,453523	0,499609037	False	0,499609037
ENSMUSG00000062190	Lancl2	254,7790766	360,1404442	188,898671	213,3003622	245,590056	0,499312822	False	0,499312822
ENSMUSG00000031245	Hmgn5	422,7202685	603,1151973	597,4469596	817,2771773	701,1774062	0,49910735	False	0,49910735
ENSMUSG00000038692	Hoxb4	410,4318886	580,0662088	881,8932142	875,6541185	648,974689	0,499074753	False	0,499074753
ENSMUSG00000042408	Zmym6	333,4247079	238,1728804	471,1484295	359,2427153	78,30407582	0,498820618	False	0,498820618
ENSMUSG00000046079	Lrrc8d	866,7403955	581,9869579	419,5307694	478,2418647	613,3819273	-0,498814285	False	0,498814285
ENSMUSG00000022620	Arsa	791,3716654	218,0050156	560,1065246	433,3365253	193,3873388	-0,498654177	False	0,498654177
ENSMUSG00000004356	Utp20	233,4792181	202,6390233	224,0426098	144,8197196	329,8262588	0,49841212	False	0,49841212
ENSMUSG00000063882	Uqcrh	1006,008701	1579,816082	1421,133025	1576,177413	1880,484245	0,498398821	False	0,498398821
ENSMUSG00000059555	Tor4a	1097,761938	508,03812	777,5596459	626,4294848	402,1982076	-0,497539971	False	0,497539971
ENSMUSG00000059586	Nsmce2	247,4060486	528,2059849	349,2428918	490,5908331	689,3131523	0,497349981	False	0,497349981
ENSMUSG00000029178	Klf3	2243,038944	2610,29794	1589,164982	1838,873649	4273,504259	-0,49718576	False	0,49718576

ENSMUSG00000024378	Stard4	575,0961792	321,7254635	407,4500404	287,3941722	275,2506908	-0,497180079	False	0,497180079
ENSMUSG00000079442	St6galnac4	657,0187119	352,4574481	465,6571891	386,1859189	430,672417	-0,496666207	False	0,496666207
ENSMUSG00000026792	Lrsam1	342,4361865	121,9675638	242,7128273	207,6871948	107,9647106	-0,496584808	False	0,496584808
ENSMUSG00000040618	Pck2	542,3271662	505,1569964	579,8749902	962,0968969	384,4018268	-0,496548247	False	0,496548247
ENSMUSG00000020205	Phlda1	1255,872426	1689,298777	1297,030991	890,2483538	2585,220928	-0,496410151	False	0,496410151
ENSMUSG00000070306	Ccdc153	1087,112008	926,7614098	1081,774366	1533,517341	627,619032	0,496343887	False	0,496343887
ENSMUSG00000042625	Safb2	1270,618481	2178,129407	1792,340879	2575,321215	2873,522298	0,496314136	False	0,496314136
ENSMUSG00000020935	Dcakd	721,7375127	561,819093	1018,075977	591,6278467	575,4163148	0,496299084	False	0,496299084
ENSMUSG00000020599	Rgs9	362,0975943	196,8767762	510,6853606	313,2147424	261,0135861	0,496056111	False	0,496056111
ENSMUSG00000028069	Gpatch4	204,8063316	361,1008187	288,839247	509,6756023	568,2977624	0,496006469	False	0,496006469
ENSMUSG00000030805	Stx4a	805,298496	549,3342243	1135,588522	980,0590326	462,7059026	0,495844629	False	0,495844629
ENSMUSG00000042523	Dnal1	628,3458255	347,6555755	419,5307694	445,6854936	351,1819158	-0,495532796	False	0,495532796
ENSMUSG00000024560	Cxxc1	1237,030243	2117,625812	1748,410955	1743,449803	4059,947689	0,495064055	False	0,495064055
ENSMUSG00000028948	Nol9	414,5280152	430,247784	446,9869716	294,1299731	567,111337	-0,495015799	False	0,495015799
ENSMUSG00000052852	Reep1	285,909639	183,4315329	173,5231978	169,5176563	202,8787419	-0,494941555	False	0,494941555
ENSMUSG00000035890	Rnf126	865,9211701	1545,242599	1315,701209	1220,302598	1664,554824	0,494931338	False	0,494931338
ENSMUSG00000027583	Zbtb46	593,9383617	315,9632164	836,8650426	541,1093399	219,4886974	0,494681767	False	0,494681767
ENSMUSG00000034647	Ankrd12	1416,44059	1995,658248	2039,446698	3107,449487	2625,559391	0,494594592	False	0,494594592
ENSMUSG00000020091	Eif4ebp2	4575,373449	2361,56094	3247,519594	2583,17965	2051,329501	-0,494551273	False	0,494551273
ENSMUSG00000022957	Itsn1	820,0445519	326,5273361	582,0714863	470,3834303	302,5384748	-0,494505945	False	0,494505945
ENSMUSG00000053310	Nrgn	387,4935795	212,2427685	545,8292994	395,1669868	323,8941318	0,494277433	False	0,494277433
ENSMUSG00000040857	Erf	703,7145555	772,1411124	499,7028798	598,3636476	1493,709568	-0,493919821	False	0,493919821
ENSMUSG00000055447	Cd47	3235,120814	6210,742007	4555,533067	6440,548305	8230,232939	0,493800291	False	0,493800291
ENSMUSG00000030846	Tial1	1008,466377	1723,87226	1420,034777	1607,611151	1961,161172	0,493763278	False	0,493763278
ENSMUSG00000018599	Mief2	235,936894	421,6044134	363,5201169	425,4780909	332,1991096	0,493647172	False	0,493647172
ENSMUSG00000064181	Rab3ip	438,2855497	636,7283054	650,1628678	782,4755392	616,9412035	0,493261888	False	0,493261888
ENSMUSG00000026766	Mmadhc	344,0746371	750,0524985	484,3274066	507,4303353	1149,646204	0,493261093	False	0,493261093
ENSMUSG00000025499	Hras	836,4290584	1428,076908	1177,32195	1245,000535	1494,895993	0,493193804	False	0,493193804
ENSMUSG00000063316	Rpl27	1527,855234	3298,886469	2150,369755	2857,10222	4628,245451	0,493076899	False	0,493076899
ENSMUSG00000052837	Junb	2004,644374	5450,125389	1424,427769	2189,135296	11890,35527	-0,492963857	False	0,492963857
ENSMUSG00000069919	Hba-a1	63321,20239	45018,5159	37412,91935	42053,85036	33135,67475	-0,492170154	False	0,492170154
ENSMUSG00000024074	Crim1	2355,272814	1092,906201	1674,828333	1410,027657	1148,459779	-0,491880949	False	0,491880949
ENSMUSG00000045466	Zfp956	151,5566854	386,0705562	213,060129	383,940652	446,0959471	0,491403146	False	0,491403146
ENSMUSG00000022820	Ndufb4	321,136328	616,5604405	454,6747082	451,2986611	488,8072612	0,490896622	False	0,490896622
ENSMUSG00000066877	Nck2	679,957021	462,9005176	457,9694525	483,8550321	427,1131409	-0,490868693	False	0,490868693
ENSMUSG00000013833	Med16	703,7145555	1187,983279	500,8011279	639,9010866	1626,589211	-0,490752542	False	0,490752542
ENSMUSG00000021708	Rasgrf2	770,0718069	127,7298109	548,0257956	264,9415025	67,6262473	-0,490749177	False	0,490749177
ENSMUSG00000049482	Ctu2	187,6025998	290,0331044	263,579541	270,5546699	443,7230963	0,490558572	False	0,490558572
ENSMUSG00000023980	Taf8	512,0158291	884,504931	719,3524973	807,1734759	1249,305937	0,490510481	False	0,490510481
ENSMUSG00000065954	Tacc1	3772,532629	7980,712244	5300,14527	8608,353565	8622,939744	0,490498524	False	0,490498524
ENSMUSG00000005413	Hmox1	631,6227268	1313,792341	887,3844546	923,9273584	3037,249002	0,490496197	False	0,490496197
ENSMUSG00000036867	Smad6	2805,027518	5054,451088	5150,78353	4728,53224	3940,118724	0,490224178	False	0,490224178
ENSMUSG00000022193	Psmb5	327,6901306	670,3414135	492,0151432	460,279729	746,2615711	0,490178684	False	0,490178684
ENSMUSG00000032475	Nck1	618,5151215	270,8256141	868,7142371	505,1850684	237,2850783	0,490072815	False	0,490072815
ENSMUSG00000046861	Hectd3	938,0129989	1059,293093	1060,907652	1284,292707	667,9574953	-0,489851614	False	0,489851614
ENSMUSG00000044674	Fzd1	891,3171553	421,6044134	548,0257956	288,5168057	634,7375843	-0,489778603	False	0,489778603
ENSMUSG00000032776	Mctp2	104,8608418	402,396923	74,68086994	123,4896834	683,3810254	-0,489665394	False	0,489665394
ENSMUSG00000039356	Exosc2	166,3027413	247,7766256	244,9093235	233,5077649	284,7420939	0,489658575	False	0,489658575
ENSMUSG00000047228	A2ml1	276,8981604	2087,854202	388,7798229	1049,662309	862,5312594	0,489597878	False	0,489597878
ENSMUSG00000062901	Klhl24	1726,926988	1860,245441	1230,037858	2309,257079	1725,062519	-0,48950437	False	0,48950437
ENSMUSG00000067786	Nnat	348,1707638	335,1707068	141,6740033	118,9991494	488,8072612	0,489470653	False	0,489470653
ENSMUSG00000026880	Stom	2292,192464	1602,86507	1221,251873	1198,972562	3217,585661	0,489250373	False	0,489250373
ENSMUSG00000022974	Paxbp1	867,5596208	1217,754889	1284,950262	1562,705811	1487,777441	0,489188963	False	0,489188963
ENSMUSG00000006411	Nectin4	272,8020337	307,3198457	194,3899115	285,1489053	480,5022835	-0,488901052	False	0,488901052
ENSMUSG00000036792	Mbd5	421,0818178	590,6303285	277,8567661	437,8270592	338,1312365	0,488154858	False	0,488154858
ENSMUSG00000037005	Xpnpep2	250,6829499	88,35445565	351,439388	188,6024255	183,8959356	0,487412017	False	0,487412017
ENSMUSG00000051483	Cbr1	660,2956132	725,082761	807,2123443	891,3709873	471,0108803	-0,487351675	False	0,487351675
ENSMUSG00000037426	Depdc5	420,2625925	224,7276372	299,8217279	294,1299731	126,9475169	-0,48718611	False	0,48718611
ENSMUSG00000068798	Rap1a	1763,792128	2904,172542	2472,156445	2626,962355	2517,59468	0,487089502	False	0,487089502
ENSMUSG00000032040	Dcps	355,5437917	153,6599229	253,6953082	141,4518191	153,0488755	-0,486930426	False	0,486930426
ENSMUSG00000037646	Vps13b	778,2640602	534,9286065	542,5345552	555,7035752	474,5701565	-0,485944214	False	0,485944214
ENSMUSG00000026436	Elk4	942,1091255	2564,199963	1318,995953	2125,145188	2797,591073	0,485474054	False	0,485474054
ENSMUSG00000027800	Tm4sf1	2874,661671	2053,280719	1556,21754	1576,177413	1208,967474	-0,485461288	False	0,485461288
ENSMUSG00000020277	Pfkl	838,8867344	609,8378189	1174,027205	801,5603085	533,8914261	0,484917902	False	0,484917902
ENSMUSG00000056708	Ier5	1107,592641	1197,587024	1349,746899	791,4566071	1963,534023	-0,484845214	False	0,484845214
ENSMUSG00000020422	Tns3	4340,25578	1551,965221	3101,452599	2093,71145	1754,723154	-0,48483599	False	0,48483599
ENSMUSG00000007603	Dus3l	541,5079408	1411,750541	757,7911803	1174,274626	1589,810024	0,484817855	False	0,484817855
ENSMUSG00000025089	Gfra1	384,2166781	553,1757223	274,5620219	546,7225073	366,6054459	-0,484788063	False	0,484788063
ENSMUSG00000043913	Ccdc60	252,3214006	151,7391738	152,6564842	169,5176563	180,3366595	-0,484569871	False	0,484569871
ENSMUSG00000022228	Zscan26	2028,401909	1450,165522	2837,873058	2358,652952	1219,645302	0,48446652	False	0,48446652
ENSMUSG00000075415	Fnbp1	1826,872478	813,4372167	1305,816976	876,776752	836,4299008	-0,48442323	False	0,48442323
ENSMUSG00000020122	Egfr	382,5782275	502,2758729	273,4637738	403,0254212	914,7339767	-0,484405043	False	0,484405043
ENSMUSG00000073650	Catip	314,5825254	285,2312318	354,7341322	440,0723262	148,3031739	0,484302118	False	0,484302118
ENSMUSG00000041650	Pcca	491,5351959	156,5410464	329,4744262	351,3842809	107,9647106	-0,484245069	False	0,484245069
ENSMUSG00000002332	Dhrs1	1151,830809	759,6562437	1611,129944	1223,670499	410,5031854	0,484144041	False	0,484144041
ENSMUSG00000042659	Arrdc4	814,3099746	579,1058343	560,1065246	343,5258465	1138,968376	0,484077711	False	0,484077711
ENSMUSG00000061028	Clasrp	775,8063842	1274,416985	1085,06911	1314,603811	1829,467953	0,484018381	False	0,484018381
ENSMUSG00000025608	Podxl	8819,779865	1618,231063	6308,337014	2885,168057	1833,027229	-0,483482912	False	0,483482912
ENSMUSG00000021125	Arg2	95,8493632	928,6821588	133,9862667	680,3158921	1601,674278	0,483244383	False	0,483244383
ENSMUSG00000029062	Cdk11b	1442,6558	2830,223704	2016,383489	3440,871632	4941,461755	0,483042914	False	0,483042914
ENSMUSG00000025978	Rftn2	437,4663244	174,7881623	313,0007049	279,5357378	167,2859802	-0,483006058	False	0,483006058
ENSMUSG00000037031	Tspan15	1210,815033	593,5114521	866,517741	832,9940461	568,2977624	-0,4826773	False	0,4826773
ENSMUSG00000027254	Map1a	314,5825254	189,19378	225,1408579	194,2155929	162,5402786	-0,482610637	False	0,482610637
ENSMUSG00000039607	Rbms3	1127,254049	712,5978923	1574,887757	1008,12487	588,4669941	0,482436319	False	0,482436319
ENSMUSG00000060860	Ube2s	504,6428011	914,2765411	744,6122033	705,0138287	1525,743053	0,482388983	False	0,482388983
ENSMUSG00000038274	Fau	1712,180932	2928,181905	2391,984334	3083,874184	4877,394783	0,482372776	False	0,482372776
ENSMUSG00000010406	Mrpl52	677,499345	1224,47751	974,1460535	946,3800281	1903,026328	0,482200076	False	0,482200076
ENSMUSG00000060803	Gstp1	1404,971435	2187,733152	1995,516775	1962,363332	2840,302387	0,482051383	False	0,482051383
ENSMUSG00000074272	Ceacam1	1819,49945	1979,331881	1128,999034	1124,878752	2541,323188	0,482038244	False	0,482038244
ENSMUSG00000026500	Cox20	254,7790766	434,0892821	355,8323803	395,1669868	421,1810139	0,481950997	False	0,481950997
ENSMUSG00000050854	Tmem125	276,078935	452,3363979	197,6846557	337,9126791	450,8416487	-0,48187992	False	0,48187992
ENSMUSG00000025647	Shisa5	7507,380892	4289,032597	4296,346518	3165,826428	5375,693448	0,481858811	False	0,481858811
ENSMUSG00000033751	Gadd45gip1	300,6556948	455,2175215	450,2817158	419,8649235	559,9927847	0,481812982	False	0,481812982
ENSMUSG00000028926	Cdk14	1595,031711	599,2736992	1142,178011	789,2113401	431,8588424	-0,481797591	False	0,481797591
ENSMUSG00000026858	Miga2	455,4892816	193,9956526	326,179682	159,4139549	78,30407582	-0,481750187	False	0,481750187
ENSMUSG00000043008	Klhl6	191,6987264	975,7405102	137,2810109	424,3554574	1377,439879	-0,48170867	False	0,48170867
ENSMUSG00000039000	Ube3c	585,7461085	316,9235909	419,5307694	258,2057016	236,0986529	-0,481498839	False	0,481498839
ENSMUSG00000064030	Pym1	395,6858327	824,9617109	552,418788	727,4664984	1203,035347	0,481406976	False	0,481406976
ENSMUSG00000053293	Pom121	1110,050317	1099,628823	795,1316153	841,9751139	2578,102375	-0,481359485	False	0,481359485
ENSMUSG00000015405	Ace2	360,4591437	247,7766256	250,4005639	258,2057016	227,7936751	-0,481314888	False	0,481314888
ENSMUSG00000032064	Dixdc1	714,3644847	570,4624637	654,5558601	996,8985349	344,0633635	0,480786316	False	0,480786316
ENSMUSG00000020027	Socs2	2144,731905	8535,808715	2992,726038	8379,336334	7324,990366	0,480662897	False	0,480662897
ENSMUSG00000031170	Slc38a5	543,9656168	108,5223205	389,878071	63,99010866	128,1339423	-0,480492452	False	0,480492452
ENSMUSG00000021981	Cab39l	775,8063842	556,0568459	538,1415628	534,373539	240,8443544	-0,48046427	False	0,48046427
ENSMUSG00000032624	Eml4	558,7116727	512,8399926	495,3098874	422,1101905	779,4814821	0,480410788	False	0,480410788
ENSMUSG00000037674	Rfx7	690,6069503	696,2715255	550,2222918	495,081367	1153,20548	-0,480199201	False	0,480199201
ENSMUSG00000032299	Commd4	505,4620265	835,5258306	705,0752721	749,9191681	868,4633864	0,480174573	False	0,480174573
ENSMUSG00000037339	Fam53a	508,7389278	980,5423828	709,4682645	884,6351864	1073,714979	0,479812661	False	0,479812661
ENSMUSG00000021993	Mipep	316,220976	226,6483862	221,8461137	226,771964	176,7773833	-0,47969078	False	0,47969078
ENSMUSG00000056851	Pcbp2	4769,529851	9664,248774	6649,892169	10927,71435	12116,96252	0,479483885	False	0,479483885

ENSMUSG00000093674	Rpl41	6817,593167	13008,27285	9505,337197	12023,40463	17299,26863	0,479475296	False	0,479475296
ENSMUSG00000032834	Pwp2	192,5179517	236,2521314	193,2916634	138,0839187	368,9782967	-0,479447667	False	0,479447667
ENSMUSG00000025139	Tollip	1492,628545	1452,086271	969,7530612	1141,718254	2080,990136	0,479414945	False	0,479414945
ENSMUSG00000032578	Cish	1028,94701	2953,151643	1434,312002	2692,075098	3985,202889	0,479190197	False	0,479190197
ENSMUSG00000047880	Cxcr5	361,278369	27,85086102	259,1865486	50,51850684	20,16923165	-0,479120032	False	0,479120032
ENSMUSG00000033295	Ptprf	5667,400809	4066,225709	3966,872092	3944,934067	3847,577544	-0,478996937	False	0,478996937
ENSMUSG00000036339	Tmem260	521,846533	188,2334055	374,5025978	215,5456292	107,9647106	-0,478649869	False	0,478649869
ENSMUSG00000017007	Rbpjl	1255,872426	628,0849347	1749,509203	807,1734759	288,3013701	0,478260334	False	0,478260334
ENSMUSG00000020267	Hint1	922,4477177	1799,741847	1284,950262	1431,357694	1787,943065	0,478173467	False	0,478173467
ENSMUSG00000026688	Mgst3	566,903926	491,7117532	789,6403749	584,8920458	384,4018268	0,478091497	False	0,478091497
ENSMUSG00000039199	Zdhhc1	2209,450706	2113,784314	2539,149578	2787,498944	1586,250748	-0,478066899	False	0,478066899
ENSMUSG00000001123	Lgals9	1842,437759	858,574819	1082,872614	727,4664984	1322,864311	-0,477950794	False	0,477950794
ENSMUSG00000006024	Napa	2590,390483	4547,373342	3607,744967	3901,151361	6602,457302	0,477927767	False	0,477927767
ENSMUSG00000036856	Wnt4	589,8422351	1591,340576	821,4895694	917,1915574	2517,59468	0,477913128	False	0,477913128
ENSMUSG00000038299	Wdr36	456,3085069	498,4343748	449,1834678	327,8089777	733,2108918	-0,477154184	False	0,477154184
ENSMUSG00000029022	Miip	578,3730805	907,5539194	805,0158481	1124,878752	1007,275157	0,477016779	False	0,477016779
ENSMUSG00000025868	Higd2a	1146,915457	1646,081924	1595,754471	1603,120617	1704,893287	0,47647964	False	0,47647964
ENSMUSG00000052557	Gan	714,3644847	1120,757062	993,9145191	1170,906725	2156,921361	0,476461422	False	0,476461422
ENSMUSG00000028889	Yrdc	194,9756277	659,7772938	271,2672776	363,7332492	1659,809122	0,476421233	False	0,476421233
ENSMUSG00000027684	Mecom	757,783427	439,8515292	544,7310514	508,5529688	325,0805572	-0,47624148	False	0,47624148
ENSMUSG00000027784	Ppm1l	485,8006186	300,5972241	349,2428918	306,4789415	205,2515927	-0,476133575	False	0,476133575
ENSMUSG00000079652	Fam71f2	109,7761938	341,8933284	152,6564842	492,8361	1413,032641	0,475723647	False	0,475723647
ENSMUSG00000055980	Irs1	395,6858327	264,1029924	550,2222918	317,7052763	198,1330403	0,475659179	False	0,475659179
ENSMUSG00000040883	Tmem205	1055,981446	488,8306296	1468,357693	926,1726253	476,9430073	0,475618966	False	0,475618966
ENSMUSG00000069769	Msi2	1293,556791	1206,230395	717,1560011	1170,906725	1798,620893	0,475547739	False	0,475547739
ENSMUSG00000074165	Zfp788	244,1291473	275,6274866	365,7166131	303,111041	175,5909579	-0,475425998	False	0,475425998
ENSMUSG00000025869	Nop16	168,7604173	239,133255	243,8110754	234,6303984	471,0108803	0,475413379	False	0,475413379
ENSMUSG00000003380	Rabac1	2942,657373	5532,717598	4090,974126	4700,466403	6534,831055	0,47532484	False	0,47532484
ENSMUSG00000018372	Cep95	247,4060486	246,8162511	352,5376361	326,6863442	177,9638087	-0,475296892	False	0,475296892
ENSMUSG00000062604	Srpk2	897,0517326	1115,95519	1029,058458	1298,886942	645,4154128	-0,474963157	False	0,474963157
ENSMUSG00000057530	Ece1	7288,64773	9164,854025	5244,134617	8873,295067	10408,50996	-0,474946455	False	0,474946455
ENSMUSG00000001909	Trmt1	602,9498403	1037,204479	837,9632907	1013,738037	1836,586506	0,474849055	False	0,474849055
ENSMUSG00000051550	Zfp579	1096,942712	789,4278537	1670,435341	1468,404599	932,5303575	-0,474608855	False	0,474608855
ENSMUSG00000043004	Gng2	778,2640602	360,1404442	560,1065246	508,5529688	291,8606462	-0,474558502	False	0,474558502
ENSMUSG00000033249	Hsf4	287,5480896	399,5157995	528,25733	520,9019372	479,3158581	0,474449305	False	0,474449305
ENSMUSG00000036864	Proser3	293,2826669	123,8883128	407,4500404	289,6394392	61,69412035	0,474331364	False	0,474331364
ENSMUSG00000047501	Cldn4	132,7145029	354,3781971	95,54758361	157,1686879	900,496872	-0,474034742	False	0,474034742
ENSMUSG00000038374	Rbm8a	703,7145555	1270,575487	977,4407978	1094,567648	1973,025426	0,474018969	False	0,474018969
ENSMUSG00000040435	Ppp1r15a	1180,503696	2966,596886	1639,684395	1749,06297	8775,988619	0,474015595	False	0,474015595
ENSMUSG00000086859	Snhg20	303,1133708	579,1058343	495,3098874	420,987557	692,8724285	0,473920099	False	0,473920099
ENSMUSG00000048218	Amigo2	723,3759633	798,0712244	1156,455236	2080,239848	520,8407468	-0,473903341	False	0,473903341
ENSMUSG00000039621	Prex1	1157,565386	635,7679309	833,5702984	697,1553943	775,9222059	-0,47371791	False	0,47371791
ENSMUSG00000032583	Mon1a	484,162168	672,2621626	719,3524973	687,051693	765,2443774	0,473533599	False	0,473533599
ENSMUSG00000038683	Pak1ip1	435,8278737	818,2390893	618,3136732	605,0994485	1313,372908	0,473413802	False	0,473413802
ENSMUSG00000034801	Sos2	956,0359561	1030,481858	688,6015508	951,9931955	2058,448054	-0,473395448	False	0,473395448
ENSMUSG00000025825	Iscu	1273,895383	2743,789998	1768,179421	1966,853866	4348,249059	0,473017873	False	0,473017873
ENSMUSG00000029403	Cdkl2	325,2324546	234,3313824	226,239106	233,5077649	130,506793	-0,472921052	False	0,472921052
ENSMUSG00000059851	Kmt5c	974,8781386	623,2830621	1353,041644	1106,916616	460,3330518	0,472912447	False	0,472912447
ENSMUSG00000024384	Iws1	509,5581531	472,5042628	383,2885825	367,1011497	707,1095332	0,472686907	False	0,472686907
ENSMUSG00000015542	Nat9	257,2367525	233,3710079	356,9306284	303,111041	121,0153899	0,472546924	False	0,472546924
ENSMUSG00000069729	Arid1b	1015,839405	1042,006352	688,6015508	888,0030868	1409,473365	0,472483872	False	0,472483872
ENSMUSG00000018774	Cd68	1197,707427	850,8918229	863,2229967	722,9759645	753,3801234	-0,472470329	False	0,472470329
ENSMUSG00000027227	Sord	856,0904662	1890,017051	1250,904572	2048,806111	1187,611817	0,472228189	False	0,472228189
ENSMUSG00000079057	Cyp4v3	732,3874419	541,6512281	786,3456306	1015,983304	368,9782967	0,472197735	False	0,472197735
ENSMUSG00000030770	Parva	3691,429321	2554,596218	2661,055116	2562,972247	2210,310504	-0,472181144	False	0,472181144
ENSMUSG00000056144	Trim34a	664,3917398	270,8256141	921,4301453	526,5151046	99,65973287	0,471840661	False	0,471840661
ENSMUSG00000047388	Atmin	849,5366636	681,8659077	630,3944022	506,3077018	1178,120414	0,471738885	False	0,471738885
ENSMUSG00000039512	Uhrf1bp1	398,962734	239,133255	287,7409989	267,1867695	161,3538532	-0,471483196	False	0,471483196
ENSMUSG00000069744	Psmb3	1055,162221	2533,467978	1462,866452	1632,309088	2938,775694	0,471333254	False	0,471333254
ENSMUSG00000057388	Mrpl18	240,0330207	421,6044134	332,7691705	386,1859189	443,7230963	0,471288894	False	0,471288894
ENSMUSG00000062609	Kcnj15	1903,060434	3164,434037	1372,810109	1987,061269	2912,674336	-0,471189295	False	0,471189295
ENSMUSG00000021809	Nudt13	282,6327377	266,0237415	326,179682	391,7990864	144,7438977	0,471185449	False	0,471185449
ENSMUSG00000059486	Kbtbd2	572,6385032	508,9984945	517,2748492	423,2328239	793,7185868	0,471002912	False	0,471002912
ENSMUSG00000025278	Flnb	1953,033178	900,8312978	1409,052296	1028,332272	1356,084222	-0,470991301	False	0,470991301
ENSMUSG00000029634	Rnf6	482,5237173	716,4393904	348,1446437	504,0624349	592,0262702	-0,470913029	False	0,470913029
ENSMUSG00000031029	Eif3f	3193,340323	3673,432531	2304,124487	3008,65774	5037,562211	-0,470847648	False	0,470847648
ENSMUSG00000038042	Ptpdc1	242,4906967	184,3919074	336,0639148	217,7908961	111,5239868	0,470806242	False	0,470806242
ENSMUSG00000030189	Ybx3	2174,224017	5020,83798	3012,494504	4759,965977	8477,009421	0,470458016	False	0,470458016
ENSMUSG00000014301	Pam16	189,2410504	313,0820928	313,0007049	299,7431406	262,2000115	0,470442675	False	0,470442675
ENSMUSG00000008384	Sertad1	293,2826669	1241,764252	406,3517923	635,4105526	1932,686962	0,470437448	False	0,470437448
ENSMUSG00000024287	Thoc1	466,9584361	724,1223865	646,8681235	661,2311228	793,7185868	0,470177479	False	0,470177479
ENSMUSG00000028861	Mrps15	444,020127	337,0914558	615,018929	394,0443533	220,6751228	0,47000574	False	0,47000574
ENSMUSG00000004609	Cd33	498,9082239	1376,216684	360,2253727	1238,264734	574,2298894	-0,469874649	False	0,469874649
ENSMUSG00000009073	Nf2	2141,455004	1309,950842	1546,333307	1259,59477	1214,899601	-0,469740043	False	0,469740043
ENSMUSG00000039958	Etfbkmt	297,3787935	630,9660583	411,8430328	605,0994485	214,7429958	-0,469690694	False	0,469690694
ENSMUSG00000008226	Scrn3	497,2697732	199,7578997	359,1271246	276,1678374	151,8624501	-0,469534116	False	0,469534116
ENSMUSG00000021536	Adcy2	348,1707638	132,5316835	251,498812	153,8007875	121,0153899	-0,469243478	False	0,469243478
ENSMUSG00000022353	Mtss1	1360,733267	519,5626142	982,9320382	683,6837925	863,7176848	-0,469220721	False	0,469220721
ENSMUSG00000026626	Ppp2r5a	3013,929976	4641,490045	4172,244484	5358,329625	5457,5568	0,4691778	False	0,4691778
ENSMUSG00000030814	Bcl7c	352,2668904	681,8659077	487,6221508	495,081367	875,5819387	0,469094781	False	0,469094781
ENSMUSG00000025403	Shmt2	528,4003356	350,5366699	731,4332262	434,4591588	332,1991096	0,469094781	False	0,469094781
ENSMUSG00000021752	Kctd6	226,9254155	620,4019386	314,098953	568,0525435	873,209088	0,469000935	False	0,469000935
ENSMUSG00000032289	Thsd4	662,7532892	410,0799192	478,8361661	426,6007244	469,8244549	-0,468939803	False	0,468939803
ENSMUSG00000052921	Arhgef15	3807,759318	654,9754212	2751,111459	1543,621042	237,2850783	-0,468927701	False	0,468927701
ENSMUSG00000018442	Derl2	987,1665185	896,9897997	1366,220621	953,115829	575,4163148	0,468825104	False	0,468825104
ENSMUSG00000042446	Zmym4	566,0847006	409,1195446	376,699094	391,7990864	328,6398334	-0,468495473	False	0,468495473
ENSMUSG00000009741	Ubp1	1631,89685	4120,967056	2257,998068	4050,461615	4686,380295	0,468494382	False	0,468494382
ENSMUSG00000025509	Pnpla2	2882,853924	14152,0789	2084,47487	10556,12266	29760,29451	-0,467813757	False	0,467813757
ENSMUSG00000041481	Serpina3g	124,5222496	419,6836643	90,05634317	616,3257834	189,8280626	-0,467503744	False	0,467503744
ENSMUSG00000041247	Lamp3	6179,416638	2695,771272	4469,869716	3783,274845	2372,850783	-0,467237867	False	0,467237867
ENSMUSG00000023092	Fhl1	10865,38551	7277,718097	15020,73909	9476,149249	7230,076334	0,46721644	False	0,46721644
ENSMUSG00000026181	Ppm1f	1964,502333	564,7002165	1421,133025	797,0697745	251,5221829	-0,467122277	False	0,467122277
ENSMUSG00000058301	Upf1	1345,167986	2076,329708	1859,334012	1882,656355	2957,7585	-0,466999611	False	0,466999611
ENSMUSG00000022394	L3mbtl2	361,278369	412,9610427	261,3830448	332,2995116	541,0099784	-0,466945318	False	0,466945318
ENSMUSG00000029148	Nrbp1	1799,018817	3553,385716	2486,43367	2703,301432	5736,366767	0,466867668	False	0,466867668
ENSMUSG00000029538	Srsf9	344,8938625	274,6671121	476,6396699	325,5637107	225,4208243	0,466746574	False	0,466746574
ENSMUSG00000028677	Rnf220	1256,691651	2733,225878	1736,330226	2221,691667	3795,374827	0,466410649	False	0,466410649
ENSMUSG00000032114	Slc37a4	240,852246	211,2823939	332,7691705	254,8378012	118,6425391	0,466373403	False	0,466373403
ENSMUSG00000033658	Ddx19b	297,3787935	343,8140774	215,2566251	242,4888328	478,1294327	-0,466244129	False	0,466244129
ENSMUSG00000074656	Eif2s2	915,8939151	2245,355623	1265,181797	2042,07031	4209,437288	0,466092293	False	0,466092293
ENSMUSG00000024740	Ddb1	4359,097963	4163,223535	2795,041382	3507,107008	6021,108861	0,465999575	False	0,465999575
ENSMUSG00000014313	Cox6c	1246,041722	2337,551577	1720,954753	1976,957568	2510,476128	0,465856791	False	0,465856791
ENSMUSG00000015478	Rnf5	734,0258926	563,739842	1013,682985	700,5232948	295,4199224	0,46570368	False	0,46570368
ENSMUSG00000072825	Cep170b	1393,50228	2073,448584	1009,289992	1423,499259	3744,358535	-0,46537461	False	0,46537461
ENSMUSG00000022148	Fyb	579,1923059	725,082761	419,5307694	559,0714757	786,6000344	-0,465265812	False	0,465265812
ENSMUSG00000039747	Orai2	415,3472406	461,9401431	315,1972011	300,865774	646,6018382	-0,465197972	False	0,465197972
ENSMUSG00000025145	Lrrc45	576,7346299	385,1101817	709,4682645	795,947141	371,3511475	0,464764973	False	0,464764973
ENSMUSG00000020132	Rab21	998,635673	1114,034441	1223,448369	1202,340463	723,7194887	-0,464527822	False	0,464527822
ENSMUSG00000028803	Nipal3	2057,89402	674,1829116	1491,420903	1271,943739	462,7059026	-0,46448122	False	0,46448122
ENSMUSG00000021520	Uqcrb	899,5094085	1622,072561	1241,020339	1347,160182	1726,248944	0,464316483	False	0,464316483

ENSMUSG00000047731	Wbp1l	1963,683108	2828,302955	1423,329521	1863,571586	3148,772988	-0,464292426	False	0,464292426
ENSMUSG00000031386	Hcfc1	1797,380366	1866,007688	1195,992167	1352,77335	2479,629068	0,464228573	False	0,464228573
ENSMUSG00000025357	Dgka	1493,44777	2060,003341	2214,068144	2902,007559	2254,208243	0,463999893	False	0,463999893
ENSMUSG00000023949	Tcte1	611,9613189	443,6930273	347,0463956	410,8838556	154,2353009	-0,463878584	False	0,463878584
ENSMUSG00000061136	Prpf40a	1014,200954	1042,966726	710,5665126	880,1446524	1398,795536	0,46384156	False	0,46384156
ENSMUSG00000041488	Stx3	905,2439858	1248,486873	1480,438422	2427,133595	1404,727663	0,463802061	False	0,463802061
ENSMUSG00000038279	Nop2	379,3013262	465,7816412	421,7272656	275,0452039	736,770168	-0,46367567	False	0,46367567
ENSMUSG00000019088	Dnase1l1	400,6011847	154,6202974	552,418788	336,7900456	117,4561137	0,463595699	False	0,463595699
ENSMUSG00000027698	Nceh1	1023,212433	582,9473324	708,3700164	742,0607337	466,2651788	-0,463496526	False	0,463496526
ENSMUSG00000027907	S100a11	8605,962055	22674,44237	11866,57058	14391,03865	25395,4355	0,463494675	False	0,463494675
ENSMUSG00000046470	Sox18	4332,063527	1336,841329	5972,273099	3810,218049	214,7429958	0,463225739	False	0,463225739
ENSMUSG00000017417	Plxdc1	437,4663244	72,02808884	317,3936973	117,876516	94,9140313	-0,462898485	False	0,462898485
ENSMUSG00000005054	Cstb	468,5968868	1129,400433	645,7698754	810,5413763	1117,612719	0,462672773	False	0,462672773
ENSMUSG00000034940	Synrg	1091,208135	729,8846336	791,836871	785,8434397	484,0615596	-0,462651153	False	0,462651153
ENSMUSG00000024142	Mlst8	381,7590022	328,4480851	526,0608339	352,5069144	118,6425391	0,462567466	False	0,462567466
ENSMUSG00000035126	Wdr78	544,7848421	96,03745179	395,3693115	202,0740273	33,21991096	-0,462485669	False	0,462485669
ENSMUSG00000027984	Hadh	1450,848053	1019,917738	1998,811519	1338,179114	905,2425735	0,462246003	False	0,462246003
ENSMUSG00000075284	Wipf1	1549,155092	988,225379	1088,363855	989,0401005	1124,731271	-0,461901242	False	0,461901242
ENSMUSG00000028062	Lamtor2	543,9656168	456,177896	749,0051956	534,373539	312,0298779	0,461460262	False	0,461460262
ENSMUSG00000040564	Apoc1	244,9483726	803,8334715	337,1621628	607,3447155	429,4859916	0,460964936	False	0,460964936
ENSMUSG00000031095	Cul4b	302,2941455	452,3363979	219,6496175	434,4591588	580,1620163	-0,460749052	False	0,460749052
ENSMUSG00000021427	Ssr1	3153,198282	3329,618454	3049,834939	2291,294943	4761,125095	-0,460652714	False	0,460652714
ENSMUSG00000022283	Pabpc1	7418,904557	10705,29475	5391,299861	9770,279222	12016,11636	-0,460573027	False	0,460573027
ENSMUSG00000062098	Btbd3	1113,327219	562,7794675	621,6084175	661,2311228	809,1421168	-0,460412655	False	0,460412655
ENSMUSG00000056870	Gulp1	339,9785105	66,26584174	247,1058197	187,479792	21,35565704	-0,46031457	False	0,46031457
ENSMUSG00000059119	Nap1l4	1246,860947	736,6072553	743,5139552	615,2031499	906,4289989	-0,460034659	False	0,460034659
ENSMUSG00000037075	Rnf139	522,6657583	552,2153478	379,9938382	398,5348873	839,989177	-0,459912622	False	0,459912622
ENSMUSG00000034164	Emid1	358,0014677	103,7204479	260,2847967	106,6501811	224,2343989	-0,459874456	False	0,459874456
ENSMUSG00000028475	Spaar	241,6714713	309,2405948	175,719694	279,5357378	231,3529513	-0,459771285	False	0,459771285
ENSMUSG00000036718	Micall2	231,0215421	299,6368496	168,0319574	177,3760907	624,0597558	-0,459291745	False	0,459291745
ENSMUSG00000022261	Sdc2	1470,509461	754,8543711	1069,693637	972,2005982	804,3964153	-0,459118402	False	0,459118402
ENSMUSG00000062981	Mrpl42	548,0617435	524,3644868	753,398188	397,4122538	489,9936866	0,45907413	False	0,45907413
ENSMUSG00000025728	Pigq	2036,594162	589,669954	1481,53667	925,0499918	526,7728737	-0,459064183	False	0,459064183
ENSMUSG00000051344	Plekhm3	623,4304735	350,536699	453,5764601	363,7332492	317,9620049	-0,458882907	False	0,458882907
ENSMUSG00000037896	Rcor1	668,4878665	452,3363979	410,7447847	360,3653488	486,4344104	-0,458656086	False	0,458656086
ENSMUSG00000029465	Arpc3	2124,251272	3939,456273	2919,143416	3211,854401	4774,175774	0,458590663	False	0,458590663
ENSMUSG00000035284	Vps13c	401,42041	107,561946	292,1339913	199,8287604	35,59276174	-0,458483739	False	0,458483739
ENSMUSG00000037643	Prkci	1700,711778	848,9710739	1237,725595	1140,595621	807,9556914	-0,458447164	False	0,458447164
ENSMUSG00000022559	Fbxl6	525,9426596	1020,878113	722,6472415	1054,152843	851,8534309	0,458386051	False	0,458386051
ENSMUSG00000054404	Slfn5	2791,919913	1180,300283	1950,488603	2031,966608	817,4470946	-0,458380864	False	0,458380864
ENSMUSG00000036905	C1qb	1019,935532	742,3695024	1103,739328	1232,651567	1497,268844	0,458268616	False	0,458268616
ENSMUSG00000038860	Garnl3	231,8407674	423,5251624	318,4919454	513,0435028	497,1122389	0,458122612	False	0,458122612
ENSMUSG00000019872	Smpdl3a	2647,736255	1742,119376	1927,425393	1631,186454	1626,589211	-0,458084403	False	0,458084403
ENSMUSG00000031216	Stard8	1805,57262	948,8500237	1758,295188	2479,897369	648,974689	0,457823969	False	0,457823969
ENSMUSG00000021660	Btf3	2120,97437	4417,722782	2912,553928	4031,376846	6742,455499	0,457557575	False	0,457557575
ENSMUSG00000001642	Akr1b3	1070,727502	670,3414135	779,7561421	508,5529688	762,8715266	-0,457496447	False	0,457496447
ENSMUSG00000040048	Ndufb10	600,4921644	941,1670276	852,2405158	847,5882814	824,5656469	0,457488936	False	0,457488936
ENSMUSG00000034354	Mtmr3	1911,252687	1642,240426	1277,262526	1628,941187	2624,372965	0,457454235	False	0,457454235
ENSMUSG00000034761	Map4k5	624,2496988	673,2225371	454,6747082	615,2031499	938,4624845	-0,457288465	False	0,457288465
ENSMUSG00000031723	Txnl4b	237,5753447	138,2939306	326,179682	167,2723893	149,4895993	0,457281795	False	0,457281795
ENSMUSG00000023075	Akirin1	688,9684996	869,1389387	551,3205399	501,8171679	1063,037151	-0,457276195	False	0,457276195
ENSMUSG00000026048	Ercc5	306,3902721	646,3320506	420,6290175	670,2121907	627,619032	0,457177887	False	0,457177887
ENSMUSG00000026961	Lrrc26	376,8436502	407,1987956	517,2748492	255,9604346	185,082361	0,456964963	False	0,456964963
ENSMUSG00000045092	S1pr1	4080,561351	7524,534348	2972,957573	4576,976719	5025,697957	-0,456868759	False	0,456868759
ENSMUSG00000020899	Pfas	240,852246	132,5316835	330,5726743	227,8945975	55,76199339	0,456819097	False	0,456819097
ENSMUSG00000035960	Apex1	297,3787935	340,9329539	266,8742852	216,6682627	469,8244549	-0,456813926	False	0,456813926
ENSMUSG00000033910	Gucy1a1	2692,793648	1618,231063	1960,372836	1962,363332	1210,153899	-0,456511497	False	0,456511497
ENSMUSG00000028713	Cyp4b1	9961,779971	8914,196275	11555,76638	14187,84199	7259,736969	-0,456486268	False	0,456486268
ENSMUSG00000029632	Ndufa4	772,5294829	1594,2217	1059,809404	1271,943739	1523,370202	0,456142936	False	0,456142936
ENSMUSG00000030921	Trim30a	748,7719485	349,5763245	545,8292994	458,034462	181,5230849	-0,456076549	False	0,456076549
ENSMUSG00000054387	Mdm4	2150,466482	1354,12807	2949,894363	2225,059568	759,3122504	0,456013647	False	0,456013647
ENSMUSG00000029505	Ep400	1826,872478	1553,88597	1389,283831	1357,263884	2505,730426	0,455855281	False	0,455855281
ENSMUSG00000063800	Prpf38a	217,0947115	297,7161006	304,2147202	310,9694754	519,6543214	0,455612663	False	0,455612663
ENSMUSG00000039737	Prkrip1	179,4103465	306,3594712	246,0075716	287,3941722	416,4353123	0,455439627	False	0,455439627
ENSMUSG00000028957	Per3	645,5495573	1459,769267	2082,278374	2625,839722	885,0733419	0,455269157	False	0,455269157
ENSMUSG00000058486	Wdr91	436,647099	523,4041123	318,4919454	692,6648604	748,6344219	-0,455210868	False	0,455210868
ENSMUSG00000030714	Sgf29	434,1894231	892,1879272	595,2504634	751,0418016	701,1774062	0,455172257	False	0,455172257
ENSMUSG00000024137	E4f1	363,736045	736,6072553	498,6046317	705,0138287	957,4452907	0,455004386	False	0,455004386
ENSMUSG00000036114	Rpp25l	196,6140784	342,8537029	382,1903344	269,4320365	386,7746775	0,454554775	False	0,454554775
ENSMUSG00000020460	Rps27a	2649,374706	5342,563443	3629,709929	4651,070529	6997,536958	0,454202358	False	0,454202358
ENSMUSG00000008763	Man1a2	899,5094085	1134,202306	656,7523563	981,1816661	1049,986471	-0,4537889	False	0,4537889
ENSMUSG00000066438	Plekhd1	677,499345	592,5510776	991,7180229	982,3042996	494,7393882	-0,453550789	False	0,453550789
ENSMUSG00000039308	Ndst2	613,5997696	928,6821588	448,0852197	711,7496296	1222,018153	-0,453524802	False	0,453524802
ENSMUSG00000031939	Taf1d	267,0674565	829,7635835	365,7166131	652,2500549	1360,829924	0,453521977	False	0,453521977
ENSMUSG00000031864	Ints10	306,3902721	223,7672627	211,9618809	187,479792	111,5239868	-0,453371508	False	0,453371508
ENSMUSG00000017652	Cd40	230,2023168	266,0237415	315,1972011	208,8098283	110,3375614	0,453352371	False	0,453352371
ENSMUSG00000027589	Pcmtd2	864,2827195	731,8053827	965,3600688	1183,255693	312,0298779	0,453186643	False	0,453186643
ENSMUSG00000034330	Plcg2	700,4376542	359,1800697	511,7836087	261,5736021	430,672417	-0,4527227	False	0,4527227
ENSMUSG00000039410	Prdm16	577,5538552	264,1029924	411,8430328	422,1101905	240,8443544	-0,452335821	False	0,452335821
ENSMUSG00000037101	Ttc29	344,0746371	196,8767762	251,498812	217,7908961	94,9140313	-0,452169965	False	0,452169965
ENSMUSG00000078954	Arhgap8	369,4706223	630,0056838	270,1690295	387,3085524	1070,155703	-0,451597353	False	0,451597353
ENSMUSG00000040383	Aqr	762,698779	657,8565448	545,8292994	559,0714757	1042,867919	0,451371154	False	0,451371154
ENSMUSG00000029992	Gfpt1	1143,638556	696,2715255	796,2298634	539,9867064	836,4299008	-0,451314622	False	0,451314622
ENSMUSG00000068917	Clk2	1000,274124	1367,573314	1496,912143	1402,169223	1427,269746	0,451222753	False	0,451222753
ENSMUSG00000044252	Osbpl1a	337,5208345	343,8140774	286,6427508	461,4023624	237,2850783	0,451048831	False	0,451048831
ENSMUSG00000005674	Tomm40l	541,5079408	231,4502588	740,2192109	325,5637107	103,219009	0,450970084	False	0,450970084
ENSMUSG00000041773	Enc1	313,7633001	609,8378189	229,5338503	342,403213	675,0760476	-0,450969685	False	0,450969685
ENSMUSG00000026921	Egfl7	3888,862625	5315,672957	8974,88337	7624,926632	6076,870854	0,450904074	False	0,450904074
ENSMUSG00000042535	Gtpbp1	939,6514495	894,1086762	687,5033027	727,4664984	1503,200971	-0,450759064	False	0,450759064
ENSMUSG00000020357	Flt4	1178,04602	1655,685669	862,1247486	1268,575838	2371,664357	-0,450427352	False	0,450427352
ENSMUSG00000039795	Zfand1	255,5983019	117,1656912	349,2428918	235,7530319	77,11765043	0,450352501	False	0,450352501
ENSMUSG00000039740	Alg2	431,7317471	385,1101817	589,7592229	426,6007244	293,0470716	0,449990891	False	0,449990891
ENSMUSG00000020440	Arf5	1520,482206	2816,778461	2110,832824	2076,871948	5295,016521	0,449883335	False	0,449883335
ENSMUSG00000026127	Imp4	716,0029354	705,8752707	794,0333672	524,2698376	1028,630814	-0,449655955	False	0,449655955
ENSMUSG00000003721	Insig2	478,4275907	559,898344	384,3868306	653,3726884	271,6914146	0,449605559	False	0,449605559
ENSMUSG00000045377	Tmem88	262,9713298	104,6808225	359,1271246	160,5365884	102,0325836	0,449589103	False	0,449589103
ENSMUSG00000035666	Gtf3c4	358,820693	286,1916063	275,6602699	226,771964	489,9936866	0,449500068	False	0,449500068
ENSMUSG00000066705	Fxyd6	460,4046335	492,6721277	337,1621628	574,7883444	962,1909923	-0,44945971	False	0,44945971
ENSMUSG00000059866	Tnip2	266,2482311	642,4905525	363,5201169	541,1093399	850,6670055	0,449263264	False	0,449263264
ENSMUSG00000037852	Cpe	397,3242834	325,5669616	291,0357432	474,8739643	648,974689	-0,449120623	False	0,449120623
ENSMUSG00000054469	Lclat1	865,1019448	332,2895832	633,6891464	344,64848	103,219009	-0,449094846	False	0,449094846
ENSMUSG00000021950	Anxa8	856,0904662	645,3716761	798,4263596	1168,661458	785,413609	0,449021901	False	0,449021901
ENSMUSG00000020074	Ccar1	1080,558206	2585,328202	1474,947181	2813,319514	4301,978469	0,448886505	False	0,448886505
ENSMUSG00000020089	Ppa1	165,483516	293,8746025	165,8354612	121,2444164	465,0787534	-0,448769206	False	0,448769206
ENSMUSG00000021709	Erbin	2505,191049	3078,00033	1836,270802	2970,488202	3270,974804	-0,448141795	False	0,448141795
ENSMUSG00000022089	Bin3	425,1779445	795,1901008	579,8749902	736,4475663	885,0733419	0,447675156	False	0,447675156
ENSMUSG00000074794	Arrdc3	1483,617066	1319,554588	1331,076682	2022,98554	1210,153899	0,447367239	False	0,447367239
ENSMUSG00000003299	Mrpl4	1640,089104	2310,66109	2432,619514	2236,285903	3448,938612	0,447330448	False	0,447330448
ENSMUSG00000021371	Mcur1	979,7934905	1034,323356	692,9945432	776,8623718	1335,914991	0,447278595	False	0,447278595
ENSMUSG00000035458	Tnni3	443,2009017	789,4278537	325,0814339	332,2995116	514,9086198	-0,447159654	False	0,447159654

ENSMUSG00000028293	Slc35a1	320,3171027	219,9257646	235,0250907	214,4229957	118,6425391	-0,446686046	False	0,446686046
ENSMUSG00000079560	Hoxa3	296,5595682	168,0655406	404,1552962	272,7999369	212,370145	0,446587834	False	0,446587834
ENSMUSG00000028214	Gem	299,8364695	269,8652395	291,0357432	220,0361631	520,8407468	-0,44643522	False	0,44643522
ENSMUSG00000045205	Dpy19l4	760,241103	390,8724288	557,9100284	496,2040005	338,1312365	-0,446424544	False	0,446424544
ENSMUSG00000021109	Hif1a	897,8709579	1056,41197	658,9488525	666,8442902	2408,443544	-0,446341628	False	0,446341628
ENSMUSG00000054717	Hmgb2	586,5653338	899,8709233	430,5132503	862,1825167	1427,269746	-0,446234169	False	0,446234169
ENSMUSG00000026786	Apbb1ip	691,4261756	632,8868073	479,9344142	510,7982358	942,0217607	0,446185165	False	0,446185165
ENSMUSG00000027660	Skil	711,9068088	1262,892491	969,7530612	1108,03925	1321,677886	0,445929025	False	0,445929025
ENSMUSG00000041702	Btbd7	1460,678757	958,4537689	1033,45145	983,4269331	1072,528554	-0,445622869	False	0,445622869
ENSMUSG00000025132	Arhgdia	16122,35443	15670,43101	12660,60395	11838,1701	24477,14225	-0,445616351	False	0,445616351
ENSMUSG00000024186	Rgs11	693,0646262	735,6468807	1127,900786	1222,547865	508,9764928	-0,445390859	False	0,445390859
ENSMUSG00000032878	Ccdc85a	514,4735051	331,3292087	463,4606929	700,5232948	148,3031739	0,445336244	False	0,445336244
ENSMUSG00000055407	Map6	1005,189476	1368,533688	839,0615388	868,9183176	898,1240212	0,445163477	False	0,445163477
ENSMUSG00000027452	Acss1	1418,07904	1443,4429	1127,900786	1929,806961	1154,391906	0,444518595	False	0,444518595
ENSMUSG00000020620	Abca8b	389,9512554	136,3731815	286,6427508	227,8945975	88,98190434	-0,444040002	False	0,444040002
ENSMUSG00000006464	Bbs1	350,6284398	334,2103322	265,7760372	279,5357378	476,9430073	0,443873861	False	0,443873861
ENSMUSG00000037738	Nek5	406,335762	736,6072553	298,7234798	716,2401636	593,2126956	-0,443861705	False	0,443861705
ENSMUSG00000042429	Adora1	228,5638661	533,0078575	168,0319574	658,9858558	898,1240212	-0,443861705	False	0,443861705
ENSMUSG00000026074	Map4k4	2362,645842	3886,635674	1737,428474	2578,689116	6418,561367	-0,4434498	False	0,4434498
ENSMUSG00000020641	Rsad2	366,193721	112,3638186	126,2985301	167,2723893	269,3185638	-0,443293377	False	0,443293377
ENSMUSG00000020096	Tbata	256,4175272	166,1447916	182,3091825	188,6024255	141,1846216	-0,44314665	False	0,44314665
ENSMUSG00000020142	Slc1a4	830,6944811	483,0683825	541,4363071	425,4780909	611,0090765	-0,443124158	False	0,443124158
ENSMUSG00000031161	Hdac6	290,8249909	519,5626142	395,3693115	451,2986611	465,0787534	0,443049647	False	0,443049647
ENSMUSG00000071176	Arhgef10	815,1291999	272,7463631	599,6434557	529,883005	277,6235416	-0,44292381	False	0,44292381
ENSMUSG00000025223	Ldb1	3264,629126	5671,971903	4436,922273	6824,488957	6577,542369	0,442647333	False	0,442647333
ENSMUSG00000009112	Bcl2l13	651,2841346	479,2268844	358,0288765	442,3175932	405,7574838	-0,442578238	False	0,442578238
ENSMUSG00000028583	Pdpn	2043,147964	1957,243268	1503,501632	3146,741659	2656,406451	-0,442467254	False	0,442467254
ENSMUSG00000020019	Ntn4	2079,193879	1181,260657	1186,107934	1530,14944	787,7864598	-0,442351733	False	0,442351733
ENSMUSG00000042719	Naa25	401,42041	453,2967725	295,4287355	367,1011497	638,2968605	-0,442303812	False	0,442303812
ENSMUSG00000061306	Slc38a10	1992,355994	1058,332719	898,3669355	1039,558607	1466,421784	-0,442175329	False	0,442175329
ENSMUSG00000019437	Tlcd1	198,252529	473,4646373	386,5833268	692,6648604	269,3185638	0,441974407	False	0,441974407
ENSMUSG00000001739	Cldn15	343,2554118	115,2449422	213,060129	239,1209324	252,7086083	-0,441807657	False	0,441807657
ENSMUSG00000050188	Lsm10	249,0444993	208,4012704	338,2604109	259,3283351	164,9131294	0,441730792	False	0,441730792
ENSMUSG00000027533	Fabp5	974,0589133	543,5719772	717,1560011	591,6278467	414,0624615	-0,441722054	False	0,441722054
ENSMUSG00000078566	Bnip3	693,0646262	1466,491889	941,1986109	2103,815151	1414,219066	0,441509306	False	0,441509306
ENSMUSG00000034509	Mad2l1bp	190,8795011	339,9725793	259,1865486	278,4131043	529,1457245	0,441328669	False	0,441328669
ENSMUSG00000002625	Akap8l	1682,688821	1081,381707	2284,356022	2063,400346	638,2968605	0,441019111	False	0,441019111
ENSMUSG00000079017	Ifi27l2a	454,6700562	110,4430696	617,2154251	224,526697	290,6742209	0,440954124	False	0,440954124
ENSMUSG00000021103	Mnat1	221,1908382	203,5993978	169,1302055	209,9324617	300,165624	0,440467134	False	0,440467134
ENSMUSG00000031762	Mt2	268,7059071	37417,15159	364,618365	6994,006613	120019,979	0,440359191	False	0,440359191
ENSMUSG00000073418	C4b	3169,582788	2176,208658	1770,375917	3529,559678	4300,792043	0,440309423	False	0,440309423
ENSMUSG00000070520	Nsmce3	249,0444993	362,0611933	369,0113574	337,9126791	532,7050007	0,440246938	False	0,440246938
ENSMUSG00000033705	Stard9	1692,519525	1103,470321	2296,436751	1847,854717	481,6887088	0,440224572	False	0,440224572
ENSMUSG00000056071	S100a9	3407,158133	18630,30527	4622,5262	18836,66725	17714,51752	0,440112589	False	0,440112589
ENSMUSG00000024966	Stip1	2456,037529	1666,249789	3332,084697	1389,820255	2045,397375	0,440092467	False	0,440092467
ENSMUSG00000042063	Zfp386	390,7704808	354,3781971	461,2641967	529,883005	141,1846216	0,439352368	False	0,439352368
ENSMUSG00000033685	Ucp2	8432,286286	19779,87357	11431,66434	24912,35967	15396,2423	0,439039707	False	0,439039707
ENSMUSG00000059854	Hydin	879,8480007	473,4646373	649,0646197	617,4484169	587,2805687	-0,438892193	False	0,438892193
ENSMUSG00000067288	Rps28	2211,089156	4941,126895	2997,119031	4086,385886	6062,633749	0,438819182	False	0,438819182
ENSMUSG00000040520	Manea	519,388857	383,1894327	352,5376361	377,204851	361,8597443	-0,438757287	False	0,438757287
ENSMUSG00000006205	Htra1	638,1765294	581,0265833	396,4675596	344,64848	864,9041102	0,438584643	False	0,438584643
ENSMUSG00000027551	Zfp64	287,5480896	420,6440389	236,1233388	212,1777287	1710,825414	-0,438530022	False	0,438530022
ENSMUSG00000029860	Zyx	6360,465435	5577,8552	5906,378214	5142,783996	8619,380467	0,438451836	False	0,438451836
ENSMUSG00000024011	Pi16	599,672939	361,1008187	442,5939792	360,3653488	341,6905127	-0,438192045	False	0,438192045
ENSMUSG00000029346	Srrd	154,0143614	260,2614944	208,6671366	231,262498	370,1647221	0,438138521	False	0,438138521
ENSMUSG00000051586	Mical3	579,1923059	784,6259812	840,1597869	908,2104896	926,5982306	0,43796267	False	0,43796267
ENSMUSG00000018800	Abca5	582,4692072	273,7067376	400,8605519	429,9686248	219,4886974	-0,437950393	False	0,437950393
ENSMUSG00000022204	Ngdn	312,1248494	585,8284559	422,8255172	446,8081271	1007,275157	0,437939213	False	0,437939213
ENSMUSG00000060261	Gtf2i	2399,510981	619,4415641	1771,474165	1187,746227	365,4190205	-0,43778999	False	0,43778999
ENSMUSG00000028959	Fastk	1024,850883	1869,849186	1388,185582	1534,639974	2035,905971	0,437786439	False	0,437786439
ENSMUSG00000036850	Mrpl41	237,5753447	775,9826105	321,7866896	533,2509055	977,6145224	0,437719529	False	0,437719529
ENSMUSG00000033032	Afap1l1	3539,872636	1954,362144	2494,121407	2613,490754	1725,062519	-0,437719398	False	0,437719398
ENSMUSG00000041801	Phlda3	722,556738	547,4134752	978,5390459	444,5628602	650,1611144	0,437518541	False	0,437518541
ENSMUSG00000029623	Pdap1	1149,373133	1597,102823	1556,21754	1776,006174	1841,332207	0,437196514	False	0,437196514
ENSMUSG00000021112	Mpp5	1112,507993	692,4300274	717,1560011	821,7677112	625,2461812	-0,437013151	False	0,437013151
ENSMUSG00000020022	Ndufa12	537,4118142	1088,104329	791,836871	727,4664984	1137,78195	0,436852773	False	0,436852773
ENSMUSG00000020474	Polm	508,7389278	947,8896492	688,6015508	822,8903447	1152,019055	0,436743939	False	0,436743939
ENSMUSG00000031480	Thsd1	764,3372297	564,7002165	739,1209628	875,6541185	933,7167829	-0,436724119	False	0,436724119
ENSMUSG00000028328	Tmod1	1241,12637	3598,523319	917,037153	3708,058402	3356,397432	-0,436597926	False	0,436597926
ENSMUSG00000067847	Romo1	329,3285813	482,108008	517,2748492	445,6854936	538,6371276	0,43649828	False	0,43649828
ENSMUSG00000044712	Slc38a6	346,5323131	190,1541546	468,9519333	331,1768781	144,7438977	0,436450171	False	0,436450171
ENSMUSG00000057963	Itpk1	625,0689241	872,0200623	845,6510273	1015,983304	991,8516271	0,436047153	False	0,436047153
ENSMUSG00000033055	Ankrd54	541,5079408	799,0315989	732,5314743	752,1644351	1014,39371	0,435908254	False	0,435908254
ENSMUSG00000026027	Stradb	272,8020337	238,1728804	369,0113574	310,9694754	109,151136	0,43581082	False	0,43581082
ENSMUSG00000038500	Prr3	367,0129463	575,2643362	496,4081355	525,3924711	1072,528554	0,435695805	False	0,435695805
ENSMUSG00000035399	Oser1	184,3256985	260,2614944	249,3023158	263,818869	487,6208358	0,435639061	False	0,435639061
ENSMUSG00000022668	Gtpbp8	276,8981604	206,4805214	374,5025978	276,1678374	85,42262817	0,435620257	False	0,435620257
ENSMUSG00000028848	Gpn2	277,7173857	546,4531007	375,6008459	382,8180185	911,1747005	0,435582816	False	0,435582816
ENSMUSG00000022469	Rapgef3	856,9096916	1872,73031	1158,651732	2711,159867	1380,999155	0,435231912	False	0,435231912
ENSMUSG00000022555	Dgat1	477,6083654	930,6029079	645,7698754	834,1166795	667,9574953	0,435192036	False	0,435192036
ENSMUSG00000038900	Rpl12	4404,974581	9595,101809	5955,799378	7230,882278	11864,25391	0,435161463	False	0,435161463
ENSMUSG00000038072	Galnt11	498,9082239	203,5993978	369,0113574	270,5546699	191,014488	-0,435109231	False	0,435109231
ENSMUSG00000025742	Prps2	822,5022278	205,5201468	608,4294404	310,9694754	153,0488755	-0,434929623	False	0,434929623
ENSMUSG00000041849	Card6	363,736045	263,1426179	269,0707814	261,5736021	209,9972943	-0,434906163	False	0,434906163
ENSMUSG00000043668	Tox3	292,4634416	134,4524325	216,3548732	180,7439911	183,8959356	-0,43485668	False	0,43485668
ENSMUSG00000062997	Rpl35	3701,260025	6995,367989	5002,520038	6391,152431	10206,81764	0,434638548	False	0,434638548
ENSMUSG00000034160	Ogt	3803,663191	2084,973078	5140,899297	3060,298881	1254,051639	0,434631249	False	0,434631249
ENSMUSG00000020653	Klf11	702,0761048	601,1944482	519,4713453	685,9290595	1086,765658	-0,434583256	False	0,434583256
ENSMUSG00000045302	Preb	1409,886787	2617,020561	1905,460432	2933,441297	2880,64085	0,43456033	False	0,43456033
ENSMUSG00000058503	Fam133b	403,878086	892,1879272	545,8292994	940,7668606	1480,658888	0,43452997	False	0,43452997
ENSMUSG00000020708	Psmc5	824,1406785	1327,237584	1113,623561	1160,803024	1257,610915	0,434299112	False	0,434299112
ENSMUSG00000020075	Ddx21	724,1951887	841,2880777	535,9450667	671,3348242	1318,11861	-0,434293457	False	0,434293457
ENSMUSG00000059713	Rcan3	431,7317471	190,1541546	319,5901934	227,8945975	116,2696883	-0,433912044	False	0,433912044
ENSMUSG00000019848	Popdc3	255,5983019	227,6087608	148,2634918	221,1587966	345,2497889	0,433762281	False	0,433762281
ENSMUSG00000037731	Themis2	657,0187119	484,9891316	456,8712044	400,7801542	887,4461927	0,43372519	False	0,43372519
ENSMUSG00000031740	Mmp2	1714,638608	1256,169869	930,21613	884,6351864	2315,902364	0,433669898	False	0,433669898
ENSMUSG00000031502	Col4a1	11533,87337	23296,76506	8541,075376	14704,25339	33117,87837	-0,433387458	False	0,433387458
ENSMUSG00000028609	Magoh	452,2123802	876,8219349	610,6259366	612,9578829	1397,609111	0,433288381	False	0,433288381
ENSMUSG00000069874	Irgm2	1037,958489	274,6671121	768,7736612	490,5908331	175,5909579	-0,433117933	False	0,433117933
ENSMUSG00000024646	Cyb5a	3688,15242	5167,775281	4979,456828	5923,014268	6452,967703	0,433090099	False	0,433090099
ENSMUSG00000031751	Amfr	2193,885424	2187,733152	1434,312002	1714,261332	2961,317777	0,43275113	False	0,43275113
ENSMUSG00000069808	Fam57a	412,0703392	234,3313824	305,3129683	172,8855567	99,65973287	-0,432601752	False	0,432601752
ENSMUSG00000040620	Dhx33	392,4089314	370,7045639	401,9588	290,7620727	522,0271722	-0,432518784	False	0,432518784
ENSMUSG00000044167	Foxo1	1639,269878	1967,807387	1214,662385	2011,759206	3508,259882	-0,432498017	False	0,432498017
ENSMUSG00000038119	Cdon	395,6858327	178,6296603	293,2322393	258,2057016	138,8117708	-0,432311683	False	0,432311683
ENSMUSG00000039770	Ypel5	878,20955	1832,39458	1185,009686	1692,931296	2977,927732	0,432261724	False	0,432261724
ENSMUSG00000018415	Gid4	1011,743278	562,7794675	750,1034437	690,4195934	541,0099784	-0,431681794	False	0,431681794
ENSMUSG00000042129	Rassf4	828,2368051	490,7513787	579,8749902	614,0805164	391,5203791	-0,431615485	False	0,431615485
ENSMUSG00000033361	Prrg3	1504,0977	1271,535862	2028,464217	1796,213576	904,0561481	0,431489572	False	0,431489572
ENSMUSG00000004952	Rasa4	414,5280152	247,7766256	559,0082765	446,8081271	297,7927732	0,431400036	False	0,431400036

ENSMUSG00000040818	Dennd6a	776,6256096	447,5345254	572,1872535	575,9109779	157,794577	-0,431373459	False	0,431373459
ENSMUSG00000026809	Spaca9	308,0287228	113,3241931	228,4356022	145,9423531	73,55837426	-0,431277369	False	0,431277369
ENSMUSG00000073002	Vamp5	561,1693487	396,6346759	756,6929322	742,0607337	215,9294212	0,431271759	False	0,431271759
ENSMUSG00000051695	Pcbp1	3273,624405	3100,088944	3106,943839	2428,256229	4971,122389	-0,430968143	False	0,430968143
ENSMUSG00000037369	Kdm6a	624,2496988	1011,274367	841,258035	1059,76601	1628,962062	0,430425159	False	0,430425159
ENSMUSG00000055240	Zfp101	244,9483726	249,6973747	228,4356022	330,0542447	61,69412035	0,430225445	False	0,430225445
ENSMUSG00000079215	Zfp664	1495,905446	1110,192943	864,3212448	1093,445015	994,2244779	-0,43020856	False	0,43020856
ENSMUSG00000072704	Smim10l1	1151,830809	810,5560931	1551,824548	1053,030209	529,1457245	0,430036636	False	0,430036636
ENSMUSG00000063410	Stk24	1266,522355	1095,787325	650,1628678	648,8821545	1706,079713	0,429812513	False	0,429812513
ENSMUSG00000038991	Txndc5	2519,937104	2804,293592	1779,161902	1944,401196	3394,363044	0,429753151	False	0,429753151
ENSMUSG00000026425	Srgap2	1468,87101	744,2902514	1090,560351	1063,133911	682,1946	-0,429638101	False	0,429638101
ENSMUSG00000063856	Gpx1	4366,47099	5971,608753	5881,118508	6126,210929	6870,589441	0,429622807	False	0,429622807
ENSMUSG00000039879	Heca	1430,36742	1624,953684	1062,005901	1482,998834	1898,280626	-0,429594	False	0,429594
ENSMUSG00000029575	Mmab	352,2668904	179,5900349	474,4431738	197,5834934	24,91493322	0,429566417	False	0,429566417
ENSMUSG00000019920	Lims1	2687,059071	1857,364318	1995,516775	1669,355993	1844,891483	-0,429265631	False	0,429265631
ENSMUSG00000024948	Map4k2	2716,551183	2000,460121	3302,431999	3657,539895	1154,391906	0,429097372	False	0,429097372
ENSMUSG00000068614	Actc1	1386,948478	509,958869	1030,156706	217,7908961	209,9972943	-0,429050381	False	0,429050381
ENSMUSG00000041763	Tpp2	788,0947641	919,0784137	585,3662306	730,8343989	1440,320425	-0,429029596	False	0,429029596
ENSMUSG00000051469	Zfp24	552,9770954	675,1432861	410,7447847	658,9858558	596,7719718	-0,428977467	False	0,428977467
ENSMUSG00000089917	Uckl1	506,2812518	488,8306296	728,138482	676,9479916	376,096849	-0,428834841	False	0,428834841
ENSMUSG00000047040	Prr15l	1439,378899	3049,189094	1937,309626	2579,811749	3612,665316	0,428608134	False	0,428608134
ENSMUSG00000057069	Ero1lb	339,9785105	189,19378	252,5970601	198,7061269	169,6588309	-0,428605711	False	0,428605711
ENSMUSG00000000751	Rpa1	755,3257511	1015,115865	561,2047727	885,7578199	1351,338521	-0,428571695	False	0,428571695
ENSMUSG00000075569	Rsph10b	455,4892816	288,1123554	454,6747082	612,9578829	205,2515927	0,428370843	False	0,428370843
ENSMUSG00000071645	Tut1	436,647099	643,450927	587,5627268	637,6558196	877,9547895	0,428275123	False	0,428275123
ENSMUSG00000042073	Abhd14b	1095,304262	565,6605911	1473,848933	1175,397259	329,8262588	0,428256971	False	0,428256971
ENSMUSG00000032244	Fem1b	918,351591	983,4235064	741,317459	682,561159	1394,049835	-0,428088275	False	0,428088275
ENSMUSG00000020695	Mrc2	1926,817968	366,8630659	1432,115506	336,7900456	454,4009249	-0,428072426	False	0,428072426
ENSMUSG00000027284	Cdan1	230,2023168	475,3853864	309,7059607	478,2418647	606,2633749	0,427996797	False	0,427996797
ENSMUSG00000040841	Six5	724,1951887	243,9351276	974,1460535	460,279729	123,3882407	0,4277595	False	0,4277595
ENSMUSG00000031995	St14	923,266943	1397,344924	686,4050546	953,115829	1595,742151	-0,427687656	False	0,427687656
ENSMUSG00000055436	Srsf11	2991,810893	6097,417814	4023,980992	7068,100423	8772,429343	0,427604503	False	0,427604503
ENSMUSG00000020473	Aebp1	2609,232665	1939,956526	2183,317198	3312,891415	3337,414626	-0,427601272	False	0,427601272
ENSMUSG00000027217	Tspan18	3561,172494	1050,649723	2647,876139	2241,89907	486,4344104	-0,42751668	False	0,42751668
ENSMUSG00000030134	Rasgef1a	2381,488024	1372,375186	3202,491423	2183,522129	1534,048031	0,427331413	False	0,427331413
ENSMUSG00000026469	Xpr1	1122,338697	795,1901008	834,6685464	824,0129782	819,8199454	-0,427232805	False	0,427232805
ENSMUSG00000039917	Rhbdd2	766,7949056	945,0085256	730,3349781	570,2978105	2298,105983	-0,427125258	False	0,427125258
ENSMUSG00000021704	Mtx3	305,5710468	176,7089113	227,3373541	153,8007875	137,6253454	-0,426673098	False	0,426673098
ENSMUSG00000024270	Slc39a6	625,0689241	420,6440389	453,5764601	364,8558827	465,0787534	-0,426540246	False	0,426540246
ENSMUSG00000006800	Sulf2	692,2454009	903,7124214	515,078353	588,2599463	1742,8589	-0,426491654	False	0,426491654
ENSMUSG00000049775	Tmsb4x	24965,0726	19557,06668	33550,38082	23046,54282	18637,55647	0,42641804	False	0,42641804
ENSMUSG00000018765	Fxr2	1698,254102	2595,892322	2282,159526	2852,611686	3606,733189	0,426347302	False	0,426347302
ENSMUSG00000028980	H6pd	4267,344726	5756,48486	3176,133469	5179,830901	8226,673663	-0,42606712	False	0,42606712
ENSMUSG00000047617	Paxx	371,9282982	623,2830621	499,7028798	601,7315481	529,1457245	0,426046012	False	0,426046012
ENSMUSG00000002210	Smg9	374,3859742	946,9292747	502,997624	573,665711	1068,969278	0,426025198	False	0,426025198
ENSMUSG00000070643	Sox13	2519,937104	1968,767762	3396,881334	2925,582863	1875,738544	-0,425928979	False	0,425928979
ENSMUSG00000071655	Ubxn1	1557,347346	3149,068044	2092,162607	3069,279949	4050,456286	0,42590423	False	0,42590423
ENSMUSG00000022805	Maats1	326,05168	239,133255	242,7128275	218,9135296	128,1339423	-0,425850297	False	0,425850297
ENSMUSG00000000708	Kat2b	1117,423345	1059,293093	1046,630427	1500,96097	395,0796553	0,425710596	False	0,425710596
ENSMUSG00000027809	Etfdh	488,2582946	656,8961703	363,5201169	478,2418647	709,482384	-0,425609349	False	0,425609349
ENSMUSG00000048232	Fbxo10	390,7704808	232,4106333	291,0357432	237,9982989	128,1339423	-0,425125142	False	0,425125142
ENSMUSG00000003038	Hmgn2	1002,7318	1449,205148	746,8086994	1122,633485	1611,165681	-0,425125142	False	0,425125142
ENSMUSG00000026928	Card9	235,936894	358,2196952	373,4043497	396,2896203	316,7755795	0,425060087	False	0,425060087
ENSMUSG00000062563	Cys1	399,7819593	299,6368496	842,3562831	821,7677112	297,7927732	-0,424904627	False	0,424904627
ENSMUSG00000017057	Il13ra1	1177,226794	1138,043804	755,5946841	794,8245076	1580,318621	0,424823177	False	0,424823177
ENSMUSG00000028333	Anp32b	1132,169401	1519,312487	1608,933448	2012,881839	1651,504145	0,424328791	False	0,424328791
ENSMUSG00000017723	Wfdc2	7352,547306	18322,98543	9866,660817	11721,41622	24207,82368	0,424317753	False	0,424317753
ENSMUSG00000068874	Selenbp1	10151,02102	26678,24373	13620,47278	30054,02103	27129,98942	0,424151936	False	0,424151936
ENSMUSG00000049354	Dcaf7	2096,397611	1386,780804	1521,073601	1086,709214	1562,52224	-0,424035645	False	0,424035645
ENSMUSG00000007872	Id3	1523,759107	2269,364986	2120,717057	2044,315577	3371,820962	0,423983076	False	0,423983076
ENSMUSG00000016253	Nelfcd	592,2999111	710,6771433	441,4957311	525,3924717	713,0416601	-0,423928381	False	0,423928381
ENSMUSG00000038059	Smim3	456,3085069	483,0683825	554,6152841	340,157946	1433,201873	-0,423804759	False	0,423804759
ENSMUSG00000032548	Slco2a1	11544,5233	7954,782132	11573,33835	15481,11576	6212,123349	0,423300851	False	0,423300851
ENSMUSG00000038195	Rilp	309,6671734	504,1966219	441,4957311	656,7405889	415,2488869	0,423257844	False	0,423257844
ENSMUSG00000028127	Abcd3	1468,051785	1885,215179	1264,083549	1698,544463	1095,070636	-0,422878928	False	0,422878928
ENSMUSG00000027459	Fam110a	765,9756803	524,3644868	1026,861962	597,2410142	537,4507022	0,422871765	False	0,422871765
ENSMUSG00000092417	Gpank1	262,1521045	533,968232	351,439388	505,1850684	949,140313	0,422871765	False	0,422871765
ENSMUSG00000020994	Pnn	1073,185178	1593,261325	1438,704995	1787,232509	1751,163877	0,422871765	False	0,422871765
ENSMUSG00000030849	Fgfr2	2617,424918	1952,441395	2892,785462	3109,694754	2642,169346	-0,422868909	False	0,422868909
ENSMUSG00000058099	Nfam1	1333,698832	1453,046646	886,2862065	1011,49277	1787,943065	0,422867874	False	0,422867874
ENSMUSG00000035783	Acta2	2859,09639	3937,535524	3870,22626	2133,003622	4227,233669	-0,422672843	False	0,422672843
ENSMUSG00000054920	Klhl5	4496,727817	4699,112516	5106,853606	6027,419182	1900,653477	0,42266484	False	0,42266484
ENSMUSG00000027955	Fam198b	975,6973639	727,9638846	663,3418448	715,1175301	387,9611029	-0,422566853	False	0,422566853
ENSMUSG00000001029	Icam2	2940,199697	1259,050993	3940,514138	1701,912364	845,921304	0,422469732	False	0,422469732
ENSMUSG00000039031	Arhgap18	863,4634942	546,4531007	518,3730973	644,3916205	367,7918713	-0,42219745	False	0,42219745
ENSMUSG00000039304	Tnfsf10	2776,354632	305,3990967	2072,394141	527,6377381	59,32126956	-0,421893449	False	0,421893449
ENSMUSG00000029802	Abcg2	329,3285813	264,1029924	249,3023158	441,1949597	173,2181071	0,421888586	False	0,421888586
ENSMUSG00000019055	Plod1	1005,189476	739,4883788	1346,452155	999,1438019	793,7185868	0,421695495	False	0,421695495
ENSMUSG00000022514	Il1rap	338,3400599	207,4408959	229,5338503	252,5925342	227,7936751	-0,421662006	False	0,421662006
ENSMUSG00000024083	Pja2	1613,054668	1204,309645	980,7355421	1129,369286	953,8860146	-0,421588956	False	0,421588956
ENSMUSG00000095567	Noc2l	925,7246219	751,012873	812,7035847	605,0994485	1239,814534	0,421469326	False	0,421469326
ENSMUSG00000025147	Mob2	797,925468	1519,312487	1068,595389	1306,745377	2521,153956	0,421389797	False	0,421389797
ENSMUSG00000027799	Nbea	598,0344884	446,5741508	354,7341322	365,9785162	329,8262588	-0,421328941	False	0,421328941
ENSMUSG00000003559	As3mt	301,4749202	147,8976758	225,1408579	177,3760907	200,5058911	-0,421210092	False	0,421210092
ENSMUSG00000061451	Tmem151a	390,7704808	70,10733981	232,8285945	428,8459914	523,2135975	0,421078546	False	0,421078546
ENSMUSG00000039377	Hlx	648,0072333	484,028757	866,517741	523,1472041	988,2923509	-0,420917154	False	0,420917154
ENSMUSG00000022565	Plec	4827,694849	3606,206315	6703,706326	4956,426837	5251,118782	-0,420852551	False	0,420852551
ENSMUSG00000029761	Cald1	2858,277164	1710,427016	2096,555599	2135,248889	1230,323131	-0,420741577	False	0,420741577
ENSMUSG00000033918	Parl	548,0617435	806,7145951	733,6297224	753,2870686	996,5973287	0,420713656	False	0,420713656
ENSMUSG00000027947	Il6ra	1151,011584	1691,219526	859,9282524	1679,459694	2360,986529	-0,420614153	False	0,420614153
ENSMUSG00000031451	Gas6	5035,778082	3762,747361	4267,792068	7395,909401	7686,85011	-0,42042828	False	0,42042828
ENSMUSG00000022940	Pigp	471,8737881	379,3479346	631,4926503	513,0435028	269,3185638	0,420364909	False	0,420364909
ENSMUSG00000001946	Esam	3879,031921	6603,535185	5190,320461	5728,798675	6437,544173	0,420126968	False	0,420126968
ENSMUSG00000020462	Cfap36	428,4548458	763,4977418	573,2855016	722,9759645	678,6353238	0,420110625	False	0,420110625
ENSMUSG00000044250	Pced1b	407,1549873	291,9538535	544,7310514	403,0254212	154,2353009	0,419966033	False	0,419966033
ENSMUSG00000053347	Zfp943	304,7518215	317,8839654	369,0113574	350,2616474	227,7936751	-0,419907154	False	0,419907154
ENSMUSG00000030029	Lrig1	376,0244249	511,8796181	281,1515104	352,5069144	830,4977739	-0,419478579	False	0,419478579
ENSMUSG00000021711	Trappc13	350,6284398	262,1822434	256,9900525	231,262498	226,6072497	-0,419373038	False	0,419373038
ENSMUSG00000020514	Mrpl22	203,167881	383,1894327	343,7516514	271,6773034	519,6543214	0,419221701	False	0,419221701
ENSMUSG00000044279	Crb3	640,6342054	1053,530846	856,6335082	937,3989602	1286,085124	0,419177281	False	0,419177281
ENSMUSG00000078866	Zfp970	274,4404844	373,5856875	504,0958721	434,4591588	205,2515927	-0,419099901	False	0,419099901
ENSMUSG00000038369	Ncoa6	1330,42193	1308,030093	995,0127672	1172,029359	2174,717742	-0,419096913	False	0,419096913
ENSMUSG00000030835	Nomo1	1286,183763	729,8846336	962,0653246	662,3537563	751,0072727	-0,41889002	False	0,41889002
ENSMUSG00000037239	Spred3	191,6987264	298,6764751	258,0883005	326,6863442	256,2678845	0,418811939	False	0,418811939
ENSMUSG00000032965	Ift57	367,0129463	460,9797686	274,5620219	362,6106157	672,7031968	-0,418698873	False	0,418698873
ENSMUSG00000028716	Pdzk1ip1	432,5509724	323,6462125	619,4119213	382,8180185	605,0769495	-0,418452531	False	0,418452531
ENSMUSG00000068270	Shroom4	795,4677921	298,6764751	595,2504634	344,64848	212,370145	-0,418306679	False	0,418306679
ENSMUSG00000047675	Rps8	5185,696317	8603,034932	6928,847184	8217,677112	10872,40229	0,418077616	False	0,418077616
ENSMUSG00000037119	Fam91a1	1335,337282	1411,750541	999,4057596	1227,038399	1739,299624	-0,418061749	False	0,418061749
ENSMUSG00000027091	Zc3h15	488,2582946	752,9336221	652,3593639	767,8813039	1362,016349	0,418022365	False	0,418022365

ENSMUSG00000042138	Msantd2	362,9168197	672,2621626	537,0433147	809,4187429	271,6914146	-0,417669944	False	0,417669944
ENSMUSG00000041189	Chrnb1	453,0316056	809,5957186	605,1346962	911,57839	614,5683527	0,417644603	False	0,417644603
ENSMUSG00000049106	Dcaf5	595,5768124	650,1735486	445,8887235	611,8352495	1070,155703	-0,417603872	False	0,417603872
ENSMUSG00000018830	Myh11	1283,726087	2132,03143	1714,365264	1733,346101	2476,069792	0,417337122	False	0,417337122
ENSMUSG00000022309	Angpt1	1038,777714	210,3220194	1387,087334	497,326634	147,1167485	0,417171658	False	0,417171658
ENSMUSG00000028081	Rps3a1	5379,033494	9785,255963	7181,444244	8882,276135	11129,8566	0,416927038	False	0,416927038
ENSMUSG00000036270	Edc4	991,2626451	1998,539372	1323,388945	1822,034147	3402,668022	0,416897864	False	0,416897864
ENSMUSG00000027287	Snap23	913,4362391	511,8796181	684,2085585	589,3825798	552,8742323	-0,416867877	False	0,416867877
ENSMUSG00000059325	Hopx	8034,142777	17249,28672	10725,49082	14031,79593	13158,64401	0,416827665	False	0,416827665
ENSMUSG00000038740	Mvb12b	855,2712409	500,3551238	535,9450667	431,0912583	640,6697113	-0,416801243	False	0,416801243
ENSMUSG00000061983	Rps12	3503,007496	7181,680645	4676,340356	6584,245391	9658,68911	0,416785861	False	0,416785861
ENSMUSG00000018909	Arrb1	6094,217204	1719,070387	4565,417299	3275,84451	696,4317047	-0,416694185	False	0,416694185
ENSMUSG00000007050	Lsm2	220,3716128	411,0402937	338,2604109	294,1299731	453,2144995	0,416515414	False	0,416515414
ENSMUSG00000037965	Zc3h7a	1488,532418	2674,643032	1986,73079	2843,630618	3554,530472	0,416505753	False	0,416505753
ENSMUSG00000037032	Apbb1	1550,793543	783,6656066	1161,946476	1064,256544	927,784656	-0,416463019	False	0,416463019
ENSMUSG00000021939	Ctsb	10351,73123	9991,736485	10508,0377	7756,27475	17266,04872	-0,416436251	False	0,416436251
ENSMUSG00000017188	Coa3	548,8809688	341,8933284	732,5314743	472,6286973	155,4217263	0,41639743	False	0,41639743
ENSMUSG00000033972	Zfp944	182,6872478	282,3501083	243,8110754	310,9694754	259,8271607	0,416387731	False	0,416387731
ENSMUSG00000027985	Lef1	180,2295718	351,4970736	240,5163311	252,5925342	344,0633635	0,416299111	False	0,416299111
ENSMUSG00000026748	Plxdc2	2401,968657	1315,71309	1800,028615	1534,639974	1314,559334	-0,416197484	False	0,416197484
ENSMUSG00000025158	Rfng	891,3171553	864,3370661	1401,36456	1002,511702	667,9574953	-0,416182573	False	0,416182573
ENSMUSG00000020437	Myo1g	854,4520156	786,5467302	679,8155661	528,7603716	1140,154801	0,416158335	False	0,416158335
ENSMUSG00000038515	Grtp1	373,5667489	186,3126565	429,4150022	498,4492674	69,99909808	0,416080627	False	0,416080627
ENSMUSG00000022186	Oxct1	1588,477908	611,7585679	1190,500927	863,3051502	608,6362257	-0,416076282	False	0,416076282
ENSMUSG00000022197	Pdzd2	7164,125481	3037,6646	5369,334899	3874,208158	2524,713233	-0,416047214	False	0,416047214
ENSMUSG00000021242	Npc2	13063,36706	17428,87675	17455,5551	18767,06397	24218,50151	0,415952798	False	0,415952798
ENSMUSG00000021738	Atxn7	823,3214532	999,7498732	617,2154251	982,3042996	717,7873617	-0,415681701	False	0,415681701
ENSMUSG00000019359	Gdpd2	478,4275907	1947,639522	638,0821388	947,5026615	2191,327698	0,415441559	False	0,415441559
ENSMUSG00000029111	Nelfa	607,8651923	563,739842	504,0958721	455,789195	927,784656	-0,415384685	False	0,415384685
ENSMUSG00000013974	Mcemp1	276,8981604	528,2059849	353,6358841	480,4871317	207,6244435	-0,415379168	False	0,415379168
ENSMUSG00000078485	Plekhn1	308,8479481	160,3825445	411,8430328	307,601575	66,43982191	0,415197837	False	0,415197837
ENSMUSG00000031818	Cox4i1	2272,531056	4419,643532	3030,066473	3530,682311	5611,792101	0,415049434	False	0,415049434
ENSMUSG00000026604	Ptpn14	1662,208188	1661,447916	1213,564137	1513,309938	2216,242631	0,415014747	False	0,415014747
ENSMUSG00000026480	Ncf2	675,0416691	1001,670622	506,2923683	832,9940461	1123,544846	-0,415005822	False	0,415005822
ENSMUSG00000026944	Abca2	1441,836575	921,9595372	1081,774366	984,5495666	908,8018497	-0,414508035	False	0,414508035
ENSMUSG00000003814	Calr	9473,521676	3820,369832	7107,861622	3321,872483	4948,580307	-0,414485236	False	0,414485236
ENSMUSG00000001632	Brpf1	706,1722315	2035,993978	941,1986109	1576,177413	3390,803768	0,4144791	False	0,4144791
ENSMUSG00000031622	Sin3b	835,6098331	1494,34275	1142,178011	1113,652417	1876,924969	0,414397648	False	0,414397648
ENSMUSG00000079547	H2-DMb1	1391,86383	292,914228	1044,433931	376,0822176	322,7077064	-0,414296842	False	0,414296842
ENSMUSG00000031786	Drc7	521,0273077	665,5395409	390,9763191	580,4015119	745,0751457	-0,41427776	False	0,41427776
ENSMUSG00000035722	Abca7	1096,123487	1563,489715	1460,669956	1974,712301	1545,912285	0,414219894	False	0,414219894
ENSMUSG00000028010	Gar1	148,2797841	249,6973747	229,5338503	197,5834934	461,5194772	0,414140506	False	0,414140506
ENSMUSG00000041895	Wipi1	513,6542797	946,9292747	385,4850787	944,1347611	1098,629912	-0,414122648	False	0,414122648
ENSMUSG00000022201	Zfr	1766,249804	1705,625144	1325,585442	1493,102535	2591,153055	-0,414059737	False	0,414059737
ENSMUSG00000037112	Sik2	307,2094975	292,914228	233,9268426	221,1587966	409,31676	0,413994923	False	0,413994923
ENSMUSG00000029675	Eln	7140,367946	22672,52162	5359,450667	8695,918977	19960,42078	-0,413913284	False	0,413913284
ENSMUSG00000028111	Ctsk	314,5825254	735,6468807	236,1233388	547,8451408	749,8208473	-0,413897887	False	0,413897887
ENSMUSG00000041144	Dnah7b	163,8450653	435,0496566	123,0037858	272,7999369	361,8597443	-0,413629503	False	0,413629503
ENSMUSG00000020547	Bzw2	253,9598512	412,0006682	338,2604109	346,8937469	569,4841878	0,4135339	False	0,4135339
ENSMUSG00000031027	Stk33	377,6628755	371,6649384	337,1621628	502,9398014	196,9466149	0,413286756	False	0,413286756
ENSMUSG00000057236	Rbbp4	1268,160805	1215,83414	780,8543902	922,8047249	1688,283332	0,412819347	False	0,412819347
ENSMUSG00000073491	Ifi213	353,9053411	71,06771433	471,1484295	161,6592219	58,13484417	0,4128181	False	0,4128181
ENSMUSG00000056749	Nfil3	182,6872478	569,5020891	137,2810109	177,3760907	1403,541238	-0,412243851	False	0,412243851
ENSMUSG00000031207	Msn	21815,15122	15185,44188	16394,64745	14486,46249	14473,20335	-0,412105596	False	0,412105596
ENSMUSG00000051359	Ncald	1323,868128	323,6462125	995,0127672	295,2526066	310,8434525	-0,411972478	False	0,411972478
ENSMUSG00000040687	Madd	924,9053937	440,8119037	695,1910393	507,4303353	297,7927732	-0,411896317	False	0,411896317
ENSMUSG00000020918	Kat2a	734,8451179	557,9775949	977,4407978	518,6566702	421,1810139	0,411569116	False	0,411569116
ENSMUSG00000000320	Alox12	418,6241419	109,482695	556,8117803	367,1011497	107,9647106	0,411534221	False	0,411534221
ENSMUSG00000045435	Tmem60	194,9756277	336,1310813	262,4812929	259,3283351	295,4199224	0,411486053	False	0,411486053
ENSMUSG00000020864	Ankrd40	3378,485247	1480,897507	2540,247826	2165,559993	1031,003665	-0,411407302	False	0,411407302
ENSMUSG00000027405	Nop56	516,1119557	883,5445565	686,4050546	694,9101274	955,07244	0,411376126	False	0,411376126
ENSMUSG00000062647	Rpl7a	5655,112429	8850,811557	7520,802903	8534,259755	11956,79509	0,411330982	False	0,411330982
ENSMUSG00000079465	Col4a3	3026,218356	2055,201468	1246,511579	2275,578075	1116,426293	-0,411283003	False	0,411283003
ENSMUSG00000028973	Abcb8	322,7747787	212,2427685	242,7128273	215,5456292	137,6253454	-0,411277495	False	0,411277495
ENSMUSG00000032002	Dcun1d5	442,3816763	657,8565448	663,3418448	588,2599463	1068,969278	0,411162179	False	0,411162179
ENSMUSG00000030815	Phkg2	909,3401125	2088,814577	1209,171144	1930,929595	3413,345851	0,411126557	False	0,411126557
ENSMUSG00000045045	Lrfn4	410,4318886	560,8587185	384,3868306	308,7242084	978,8009478	-0,41082421	False	0,41082421
ENSMUSG00000003423	Pih1d1	443,2009017	502,2758729	506,2923683	486,1002991	333,3855349	-0,410769307	False	0,410769307
ENSMUSG00000038060	Dlec1	455,4892816	160,3825445	342,6534033	254,8378012	143,5574723	-0,410667089	False	0,410667089
ENSMUSG00000024121	Atp6v0c	710,2683581	800,952348	642,4751311	534,373539	1074,901404	-0,410515645	False	0,410515645
ENSMUSG00000033813	Tcea1	507,9197025	575,2643362	382,1903344	522,0245706	733,2108918	-0,410309145	False	0,410309145
ENSMUSG00000030272	Camk1	934,7360976	602,1548227	1242,118587	1058,643377	500,6715151	0,410171902	False	0,410171902
ENSMUSG00000059183	Mtfmt	184,3256985	412,9610427	244,9093235	343,5258465	387,9611029	0,409990473	False	0,409990473
ENSMUSG00000029816	Gpnmb	339,9785105	557,9775949	255,8918044	561,3167426	724,9059141	-0,409909617	False	0,409909617
ENSMUSG00000027628	Aar2	565,2654753	613,679317	448,0852197	425,4780909	962,1909923	-0,409843739	False	0,409843739
ENSMUSG00000061315	Naca	2619,882594	3679,194778	3480,348189	4368,166891	5425,523314	0,409729486	False	0,409729486
ENSMUSG00000025188	Hps1	366,193721	196,8767762	275,6602699	186,3571586	234,9122275	-0,409715703	False	0,409715703
ENSMUSG00000039615	Stub1	1310,760522	1741,159001	2014,186992	1890,514789	1800,993744	0,409643827	False	0,409643827
ENSMUSG00000025241	Fyco1	1078,10053	647,2924251	811,6053366	801,5603085	416,4353123	-0,409641455	False	0,409641455
ENSMUSG00000014778	Fhod1	1709,723256	1817,028588	2171,236469	2401,313025	1287,27155	-0,409446401	False	0,409446401
ENSMUSG00000042286	Stab1	1807,21107	1249,447248	1360,72938	1351,650716	1096,257062	-0,409384839	False	0,409384839
ENSMUSG00000026927	Sdccag3	1144,457781	1818,949337	1519,975353	1659,252291	1918,449858	0,409383687	False	0,409383687
ENSMUSG00000038370	Pcp4l1	3948,666074	2973,319508	2918,045168	2774,027342	2588,780204	-0,409290868	False	0,409290868
ENSMUSG00000020794	Ube2g1	696,3415276	1063,134591	924,7248896	1079,973413	1461,676082	0,409229157	False	0,409229157
ENSMUSG00000027230	Creb3l1	856,9096916	781,7448576	482,1309104	562,4393761	1137,78195	0,409009025	False	0,409009025
ENSMUSG00000091956	C2cd4b	291,6442162	328,4480851	219,6496175	417,6196565	594,399121	-0,409005477	False	0,409005477
ENSMUSG00000046330	Rpl37a	2650,193931	4943,047644	3518,786872	4691,485335	6451,781278	0,4089802	False	0,4089802
ENSMUSG00000026361	Cdc73	620,1535722	593,5114521	488,7203989	525,3924711	823,3792215	0,408931519	False	0,408931519
ENSMUSG00000002768	Mea1	933,0976469	895,0690507	1238,823843	621,9389508	836,4299008	0,408871085	False	0,408871085
ENSMUSG00000022560	Slc52a2	253,1406259	177,6692858	336,0639148	157,1686879	111,5239868	0,408796579	False	0,408796579
ENSMUSG00000040123	Zmym5	833,1521571	1503,946495	1105,935824	1978,080201	1197,10322	0,408615769	False	0,408615769
ENSMUSG00000027935	Rab13	235,1176687	120,0468147	239,4180831	312,0921089	117,4561137	0,408588921	False	0,408588921
ENSMUSG00000037820	Tgm2	7957,954822	11899,04028	5995,336309	6404,624033	16701,31023	-0,408557025	False	0,408557025
ENSMUSG00000034462	Pkd2	1599,947063	2113,784314	1853,842772	2624,717089	1205,408198	-0,40850239	False	0,40850239
ENSMUSG00000060126	Tpt1	18531,69611	34901,93073	24596,36417	32101,70451	41688,6154	0,408450142	False	0,408450142
ENSMUSG00000027546	Atp9a	1200,984329	707,7960197	904,956424	806,0508424	836,4299008	-0,408297096	False	0,408297096
ENSMUSG00000034353	Ramp1	325,2324546	505,1569964	431,6114984	618,5710504	517,2814706	0,40826207	False	0,40826207
ENSMUSG00000026417	Pigr	1608,958541	4536,809223	1212,465889	2058,909812	3425,210105	-0,408182993	False	0,408182993
ENSMUSG00000021665	Hexb	849,5366636	370,7045639	640,278635	520,9019372	281,1828177	-0,407976341	False	0,407976341
ENSMUSG00000050730	Arhgap42	462,0430842	431,2081586	437,1027388	612,9578829	340,5040873	0,407760563	False	0,407760563
ENSMUSG00000030340	Scnn1a	9940,480112	26527,46493	7493,3467	16842,87018	39646,7773	-0,40770533	False	0,40770533
ENSMUSG00000044573	Acp1	453,0316056	615,600066	341,5551552	474,8739643	779,4814821	-0,407493135	False	0,407493135
ENSMUSG00000003746	Man1a	1092,02736	680,9055332	626,0014098	794,8245076	823,3792215	-0,407380056	False	0,407380056
ENSMUSG00000018433	Nol11	311,3056241	315,9632164	240,5163311	223,4040636	412,8760362	0,407377044	False	0,407377044
ENSMUSG00000015176	Nolc1	602,9498403	690,5092784	609,5276885	454,6665615	1530,488755	-0,407229084	False	0,407229084
ENSMUSG00000057147	Dph6	419,4433672	169,9862897	316,2954492	242,4888328	62,88054574	-0,407203234	False	0,407203234
ENSMUSG00000023832	Acat2	316,220976	396,6346759	456,8712044	654,4953219	238,4715036	-0,407116189	False	0,407116189
ENSMUSG00000005672	Kit	3464,503906	2094,576824	2612,7322	1866,939486	872,0226626	-0,407089524	False	0,407089524
ENSMUSG00000042790	Rnf214	488,2582946	318,84434	367,9131093	368,2237832	249,1493322	-0,407061741	False	0,407061741
ENSMUSG00000024529	Lox	885,582578	13490,38085	1174,027205	14807,53567	9200,728909	0,406767095	False	0,406767095

ENSMUSG00000047205	Dusp18	711,9068088	503,2362474	537,0433147	400,7801542	224,2343989	-0,406649947	False	0,406649947
ENSMUSG00000029309	Sparcl1	9238,404007	5144,726293	6661,972898	5322,405354	6970,249174	-0,406433408	False	0,406433408
ENSMUSG00000046886	Zfp474	286,7288643	406,2384211	216,3548732	327,8089777	335,7583857	-0,406287528	False	0,406287528
ENSMUSG00000048047	Zbtb33	371,9282982	248,7370001	274,5620219	280,6583713	239,657929	-0,406209428	False	0,406209428
ENSMUSG00000041939	Mvk	285,0904136	194,9560271	377,7973421	196,4608599	68,81267269	0,406193023	False	0,406193023
ENSMUSG00000024563	Smad2	703,7145555	613,679317	489,818647	468,1381633	932,5303575	0,406160338	False	0,406160338
ENSMUSG00000039405	Prss23	1538,505163	1176,458784	1447,490979	2038,702409	1136,595525	0,406121914	False	0,406121914
ENSMUSG00000020183	Cpm	3090,117932	1800,702221	1366,220621	1946,646463	4094,354025	0,405973954	False	0,405973954
ENSMUSG00000035329	Fbxo33	350,6284398	776,942985	464,558941	826,2582451	1429,642596	0,405918631	False	0,405918631
ENSMUSG00000026249	Serpine2	1708,904031	1672,97241	1680,319574	1289,905875	2444,036306	-0,405805585	False	0,405805585
ENSMUSG00000026750	Psmb7	804,4792707	1071,777962	607,3311923	717,3627971	1819,97655	-0,405571779	False	0,405571779
ENSMUSG00000025203	Scd2	10852,2779	2801,412469	8192,930732	5111,350258	2063,193755	-0,405546375	False	0,405546375
ENSMUSG00000030019	Fbxl14	700,4376542	544,5323517	613,9206809	516,4114032	927,784656	0,405533344	False	0,405533344
ENSMUSG00000061207	Stk19	407,9742126	1077,540209	540,338059	717,3627971	1891,162074	0,405384338	False	0,405384338
ENSMUSG00000042590	Ipo11	356,363017	194,9560271	269,0707814	181,8666246	193,3873388	-0,405361887	False	0,405361887
ENSMUSG00000031246	Sh3bgrl	3439,927146	1551,965221	2597,356727	1908,476925	1556,590113	-0,40533384	False	0,40533384
ENSMUSG00000006335	Tfpt	199,0717543	455,2175215	263,579541	360,3653488	584,9077179	0,404949857	False	0,404949857
ENSMUSG00000020744	Slc25a19	331,7862572	175,7485368	439,299235	220,0361631	119,8289645	0,404949857	False	0,404949857
ENSMUSG00000027878	Notch2	1880,122124	1864,086939	1420,034777	1634,554355	2795,218222	-0,404900114	False	0,404900114
ENSMUSG00000045409	Trim39	611,1420936	973,8197612	904,956424	1099,058182	809,1421168	0,404885265	False	0,404885265
ENSMUSG00000042831	Alkbh6	335,8823839	365,9026913	392,0745672	253,7151677	459,1466264	-0,404746363	False	0,404746363
ENSMUSG00000015533	Itga2	553,7963207	180,5504094	418,4325213	169,5176563	80,67692661	-0,404360484	False	0,404360484
ENSMUSG00000033545	Znrf1	1474,605588	2800,452094	1951,586851	2258,738572	2807,082476	0,404318539	False	0,404318539
ENSMUSG00000026791	Slc2a8	339,1592852	344,7744519	383,2885825	387,3085524	256,2678845	-0,404310299	False	0,404310299
ENSMUSG00000021728	Emb	2232,389015	2764,918237	1686,909062	2044,315577	3097,756697	-0,404206249	False	0,404206249
ENSMUSG00000020397	Med7	254,7790766	480,187259	337,1621628	371,5916836	494,7393882	0,40419584	False	0,40419584
ENSMUSG00000000776	Polr3d	222,8292888	255,4596218	233,9268426	168,3950228	368,9782967	-0,404089376	False	0,404089376
ENSMUSG00000042369	Rbm45	294,9211176	195,9164017	222,9443617	154,923421	209,9972943	-0,403645415	False	0,403645415
ENSMUSG00000025867	Cplx2	422,7202685	387,9913052	319,5901934	406,3933217	578,9755909	-0,403480148	False	0,403480148
ENSMUSG00000061119	Prcp	896,2325072	395,6743014	677,6190699	395,1669868	354,741192	-0,403398579	False	0,403398579
ENSMUSG00000037326	Capn15	851,9943396	1184,141781	1126,802538	1181,010426	1925,56841	0,403318967	False	0,403318967
ENSMUSG00000039994	Timeless	279,3558363	211,2823939	397,5658076	291,8847062	276,4371162	-0,402931408	False	0,402931408
ENSMUSG00000040289	Hey1	1429,548195	602,1548227	1890,048826	1509,942038	434,2316932	0,402891826	False	0,402891826
ENSMUSG00000002910	Arrdc2	294,9211176	1365,652565	389,878071	2053,296645	785,413609	0,402693883	False	0,402693883
ENSMUSG00000029552	Tes	471,8737881	522,4437378	377,7973421	356,9974483	860,1584087	-0,402487272	False	0,402487272
ENSMUSG00000025917	Cops5	553,7963207	1058,332719	783,0508863	731,9570324	1277,780146	0,402403492	False	0,402403492
ENSMUSG00000020262	Adarb1	759,4218777	1064,094966	1016,977729	1003,634336	1375,067028	0,402260267	False	0,402260267
ENSMUSG00000028953	Abcf2	859,3673675	2134,912553	1135,588522	1430,23506	2611,322286	0,402093272	False	0,402093272
ENSMUSG00000038127	Ccdc50	1398,417632	960,3745179	957,6723322	831,8714126	1058,291449	-0,402058286	False	0,402058286
ENSMUSG00000057322	Rpl38	2845,169559	5155,290412	3759,303203	5115,840792	6899,06365	0,401950646	False	0,401950646
ENSMUSG00000030301	Ccdc91	494,8120972	91,2355792	374,5025978	248,1020002	77,11765043	-0,401905046	False	0,401905046
ENSMUSG00000025314	Ptprj	1010,104828	1477,056009	866,517741	764,5134035	3030,130449	-0,401891322	False	0,401891322
ENSMUSG00000021294	Kif26a	666,0301905	135,412807	504,0958721	281,7810048	123,3882407	-0,401889434	False	0,401889434
ENSMUSG00000022443	Myh9	7041,241682	4224,687504	3613,236208	3159,090627	5329,422858	-0,401850555	False	0,401850555
ENSMUSG00000020156	Mum1	620,9727975	537,80973	820,3913213	691,5422269	421,1810139	0,401782159	False	0,401782159
ENSMUSG00000029817	Tra2a	1396,779182	1057,372344	2444,700243	1410,027657	1350,152095	-0,401620463	False	0,401620463
ENSMUSG00000025439	Clns1a	554,6155461	544,5323517	576,5802459	419,8649235	831,6841993	-0,401562789	False	0,401562789
ENSMUSG00000066319	Rtp3	443,2009017	42,25647879	585,3662306	125,7349503	13,0506793	0,401378704	False	0,401378704
ENSMUSG00000010362	Rdm1	274,4404844	100,8393244	362,4218688	182,9892581	60,50769495	0,401176694	False	0,401176694
ENSMUSG00000024130	Abca3	8034,142777	3974,029755	6084,294404	5566,01682	3554,530472	-0,401054137	False	0,401054137
ENSMUSG00000041235	Chd7	709,4491328	1495,303124	936,8056186	1440,338762	3416,905127	0,401050481	False	0,401050481
ENSMUSG00000036040	Adamtsl2	809,3946226	2928,181905	1156,455236	1068,747078	4638,92328	0,401005306	False	0,401005306
ENSMUSG00000031750	Il34	202,3486557	361,1008187	269,0707814	267,1867695	647,7882636	0,401005306	False	0,401005306
ENSMUSG00000026547	Tagln2	7768,713772	4953,611763	5777,883188	4087,50852	5883,483515	-0,401005154	False	0,401005154
ENSMUSG00000024870	Rab1b	2950,849626	2234,791503	1926,327145	1639,044888	2234,039012	-0,400990163	False	0,400990163
ENSMUSG00000033542	Arhgef5	1463,136433	1775,732484	1108,13232	1553,724744	2276,750326	-0,400934141	False	0,400934141
ENSMUSG00000027620	Rbm39	4171,495363	8474,344746	5507,714158	8538,750289	14712,86128	0,400889046	False	0,400889046
ENSMUSG00000040482	Dxo	528,4003356	709,7167688	766,577165	760,0228695	697,6181301	0,400806151	False	0,400806151
ENSMUSG00000048058	Ldlrad3	548,0617435	266,984116	415,137777	303,111041	215,9294212	-0,400748212	False	0,400748212
ENSMUSG00000043895	S1pr2	791,3716654	433,1289076	599,6434557	335,6674121	191,014488	-0,400250473	False	0,400250473
ENSMUSG00000086040	Wipf3	339,1592852	535,888981	256,9900525	586,0146793	296,6063478	-0,400250473	False	0,400250473
ENSMUSG00000041729	Coro2b	1835,064731	1684,496904	2158,057492	2695,442998	1390,490559	-0,400237006	False	0,400237006
ENSMUSG00000027893	Ahcyl1	1598,308612	2096,497573	1744,017963	2331,709749	1211,340324	-0,399941755	False	0,399941755
ENSMUSG00000034613	Ppm1h	488,2582946	302,5179731	370,1096055	246,9793668	231,3529513	-0,399691975	False	0,399691975
ENSMUSG00000038151	Prdm1	297,3787935	110,4430696	161,4424689	143,6970861	225,4208243	-0,399680973	False	0,399680973
ENSMUSG00000062328	Rpl17	6831,519998	12955,45225	9011,125557	12661,06045	17846,21074	0,39950071	False	0,39950071
ENSMUSG00000031538	Plat	499,7274492	5827,552575	378,8955902	1788,355142	15136,41514	-0,399341116	False	0,399341116
ENSMUSG00000025931	Paqr8	499,7274492	141,1750541	378,8955902	205,4419278	97,28688208	-0,399341116	False	0,399341116
ENSMUSG00000024012	Mtch1	4640,911475	4327,447578	5978,862588	5411,093399	3518,93771	-0,399268202	False	0,399268202
ENSMUSG00000045005	Fzd5	462,0430842	503,2362474	350,3411399	483,8550321	824,5656469	-0,399266974	False	0,399266974
ENSMUSG00000037197	Rbm17	705,3530061	1356,048819	930,21613	1269,698472	2020,482441	0,399220497	False	0,399220497
ENSMUSG00000039382	Wdr45	335,8823839	442,7326528	553,5170361	625,3068513	493,5529628	0,398479648	False	0,398479648
ENSMUSG00000037712	Fermt2	4322,232823	2632,386554	3055,326179	2490,00107	3279,279781	-0,398397792	False	0,398397792
ENSMUSG00000022817	Itgb5	1719,55396	770,2203634	1304,718728	894,7388877	785,413609	-0,398295566	False	0,398295566
ENSMUSG00000028911	Srsf4	1599,947063	3030,941979	2108,636328	3077,138383	4960,444561	0,398286126	False	0,398286126
ENSMUSG00000024781	Lipa	1500,820798	580,0662088	1138,883267	849,8335483	366,6054459	-0,398131845	False	0,398131845
ENSMUSG00000042557	Sin3a	1004,37025	1063,134591	762,1841727	976,6911322	1324,050737	-0,398079646	False	0,398079646
ENSMUSG00000024594	Prrc1	989,6241945	889,3068036	780,8543902	751,0418016	1594,555726	-0,397987564	False	0,397987564
ENSMUSG00000049232	Tigd2	239,2137953	473,4646373	315,1972011	529,883005	447,2823725	0,397954132	False	0,397954132
ENSMUSG00000045975	C2cd2	1127,254049	533,0078575	855,5352601	728,5891319	201,6923165	-0,397913472	False	0,397913472
ENSMUSG00000029009	Mthfr	593,1191364	543,5719772	607,3311923	781,3529057	393,8932299	0,397652383	False	0,397652383
ENSMUSG00000040842	Szrd1	1935,829447	2124,348434	2006,499256	1469,527232	3172,501496	-0,397599758	False	0,397599758
ENSMUSG00000056498	Tmem154	457,1277322	811,5164677	347,0463956	646,6368875	564,7384862	-0,397468799	False	0,397468799
ENSMUSG00000048264	Dip2c	481,704492	302,5179731	365,7166131	310,9694754	232,5393767	-0,397422213	False	0,397422213
ENSMUSG00000062866	Phactr2	2574,825201	1236,002005	1954,881596	1513,309938	981,1737986	-0,397393266	False	0,397393266
ENSMUSG00000030788	Rnf141	1728,565439	678,0244097	1312,406464	930,6631593	516,0950452	-0,397360616	False	0,397360616
ENSMUSG00000046756	Mrps7	419,4433672	747,171375	552,418788	555,7035752	1110,494166	0,397286354	False	0,397286354
ENSMUSG00000033540	Idua	557,073222	299,6368496	733,6297224	484,9776656	217,1158466	0,397185121	False	0,397185121
ENSMUSG00000030748	Il4ra	2705,901254	6508,458108	2054,822172	4294,073081	12453,90733	-0,397095646	False	0,397095646
ENSMUSG00000070877	Ldlrad1	283,451963	131,571309	215,2566251	207,6871948	55,76199339	-0,397046619	False	0,397046619
ENSMUSG00000001855	Nup214	1077,281304	1199,507773	818,1948251	838,6072135	2530,64536	-0,396878704	False	0,396878704
ENSMUSG00000036257	Pnpla8	861,8250435	1025,679985	654,5558601	847,5882814	1286,085124	-0,396878704	False	0,396878704
ENSMUSG00000022831	Hcls1	670,9455424	824,0013364	509,5871126	555,7035752	931,3439321	-0,396866882	False	0,396866882
ENSMUSG00000035021	Baz1a	457,9469575	979,5820083	602,9382	1028,332272	1829,467953	0,396829631	False	0,396829631
ENSMUSG00000071226	Cecr2	228,5638661	320,765089	300,919976	599,4862811	371,3511475	0,396782536	False	0,396782536
ENSMUSG00000015653	Steap2	229,3830914	216,0842665	186,7021749	301,9884075	74,74479965	0,39673412	False	0,39673412
ENSMUSG00000021273	Fdft1	916,7131404	1212,953016	1302,522232	1206,830997	1538,793732	0,396681398	False	0,396681398
ENSMUSG00000000489	Pdgfb	3117,152367	723,162012	2367,822876	846,4656479	501,8579405	-0,396667511	False	0,396667511
ENSMUSG00000038402	Foxf2	818,4061012	405,2780466	1077,381374	312,0921089	380,8425506	0,396640223	False	0,396640223
ENSMUSG00000050708	Ftl1	15911,81352	15991,1961	15290,90812	12087,39474	26944,90706	-0,396594947	False	0,396594947
ENSMUSG00000030468	Siglecg	552,1578701	75,86958692	419,5307694	106,6501811	83,04977739	-0,396304189	False	0,396304189
ENSMUSG00000032462	Pik3cb	697,9799782	484,9891316	501,899376	456,9118285	530,3321499	-0,396289442	False	0,396289442
ENSMUSG00000042804	Gpr153	503,0043505	214,1635175	382,1903344	195,3382264	183,8959356	-0,396279585	False	0,396279585
ENSMUSG00000023988	Bysl	331,7862572	335,1707068	365,7166131	153,8007875	436,604544	0,396073015	False	0,396073015
ENSMUSG00000016520	Lnx2	514,4735051	774,0618615	390,9763191	566,92991	1498,455269	-0,396015553	False	0,396015553
ENSMUSG00000042410	Agps	875,7518741	665,5395409	600,7417038	573,665711	596,7719718	-0,395997788	False	0,395997788
ENSMUSG00000025316	Banp	442,3816763	242,974753	582,0714863	285,1489053	163,726704	0,395904717	False	0,395904717
ENSMUSG00000026872	Zeb2	1706,446355	778,863734	1297,030991	989,0401005	883,8869165	-0,39578211	False	0,39578211
ENSMUSG00000057531	Dtnbp1	484,162168	1424,23541	636,9838907	1198,972562	2523,526807	0,395766534	False	0,395766534
ENSMUSG00000024533	Spire1	342,4361865	386,0705562	260,2847967	336,7900456	401,0117822	-0,395744119	False	0,395744119

ENSMUSG00000013997	Nit1	698,7992035	553,1757223	919,2336492	777,9850053	384,4018268	0,395553646	False	0,395553646
ENSMUSG00000028480	Glipr2	592,2999111	687,6281548	450,2817158	619,6936839	953,8860146	-0,395499973	False	0,395499973
ENSMUSG00000046027	Stard5	293,2826669	347,6555755	287,7409989	478,2418647	223,0479736	-0,394937769	False	0,394937769
ENSMUSG00000024096	Ralbp1	2726,381887	2602,614944	3935,022897	4645,457362	2073,871584	-0,394661092	False	0,394661092
ENSMUSG00000024622	Hmgxb3	594,7575871	629,0453092	431,6114984	470,3834303	781,8543328	0,394598074	False	0,394598074
ENSMUSG00000109564	Muc16	423,5394938	28,81123554	160,3442208	322,1958103	90,16832974	-0,394558769	False	0,394558769
ENSMUSG00000035247	Hectd1	1771,165156	3081,841828	1347,550403	2458,567333	4204,691587	-0,394359509	False	0,394359509
ENSMUSG00000049422	Chchd10	2786,185336	4386,030423	3715,37328	3662,030429	4029,100629	0,394352551	False	0,394352551
ENSMUSG00000022949	Clic6	903,6055352	1543,32185	687,5033027	1137,227721	1114,053442	-0,394326464	False	0,394326464
ENSMUSG00000037060	Cavin3	960,951308	817,2787148	1262,985301	1041,803874	577,7891655	0,394302612	False	0,394302612
ENSMUSG00000037606	Osbpl5	1798,199592	903,7124214	1368,417117	1160,803024	812,701393	-0,394045107	False	0,394045107
ENSMUSG00000036591	Arhgap21	1590,116359	1367,573314	1490,322655	1406,659757	2089,295114	0,393883948	False	0,393883948
ENSMUSG00000004996	Mri1	240,0330207	202,6390233	322,8849377	321,0731768	182,7095103	-0,393681157	False	0,393681157
ENSMUSG00000058600	Rpl30	3149,102155	6082,051822	4137,100545	5105,737091	7232,449185	0,393679462	False	0,393679462
ENSMUSG00000054517	Trim65	326,8709053	171,9070387	429,4150022	258,2057016	107,9647106	0,393651626	False	0,393651626
ENSMUSG00000018102	Hist1h2bc	869,1980715	661,6980429	1416,740033	727,4664984	979,9873732	-0,393511961	False	0,393511961
ENSMUSG00000033916	Chmp2a	1178,865245	2089,774951	1548,529803	1699,667097	2958,944926	0,393500335	False	0,393500335
ENSMUSG00000020143	Dock2	724,1951887	443,6930273	551,3205399	431,0912583	272,87784	-0,393487241	False	0,393487241
ENSMUSG00000037242	Clic4	11354,46303	15716,52899	8644,310696	11961,65979	16534,02425	-0,393436648	False	0,393436648
ENSMUSG00000075600	Zc3h3	314,5825254	417,7629153	439,299235	413,1291226	634,7375843	0,39315423	False	0,39315423
ENSMUSG00000024276	Zfp397	598,8537137	455,2175215	786,3456306	543,3546069	154,2353009	0,392959946	False	0,392959946
ENSMUSG00000033880	Lgals3bp	3754,509672	3050,149469	2752,209707	2697,688265	4929,597501	0,392845348	False	0,392845348
ENSMUSG00000055128	Cgrrf1	356,363017	546,4531007	467,8536852	550,0904078	558,8063593	0,392709794	False	0,392709794
ENSMUSG00000092607	Scnm1	486,619844	689,5489039	639,1803869	638,7784531	663,2117937	0,392520496	False	0,392520496
ENSMUSG00000033436	Armcx2	516,1119557	175,7485368	393,1728153	203,1966608	209,9972943	-0,392520477	False	0,392520477
ENSMUSG00000005397	Nid1	2153,743383	2829,26333	1640,782643	2003,900771	3737,239982	-0,392462229	False	0,392462229
ENSMUSG00000001910	Nacc1	2606,774989	2789,887975	2267,882301	1985,938635	4717,227356	-0,392445013	False	0,392445013
ENSMUSG00000010608	Rbm25	2815,677447	5671,971903	3695,604814	6484,331011	10555,62671	0,392328418	False	0,392328418
ENSMUSG00000029234	Tmem165	1004,37025	1373,335561	1082,872614	1388,697621	765,2443774	-0,392298757	False	0,392298757
ENSMUSG00000020424	Gatsl3	278,536611	212,2427685	341,5551552	351,3842809	268,1321384	-0,39215157	False	0,39215157
ENSMUSG00000022464	Slc38a4	270,3443578	3104,890816	354,7341322	1928,684328	2926,91144	0,391939905	False	0,391939905
ENSMUSG00000020814	Mxra7	1118,242571	302,5179731	1467,259445	459,1570955	169,6588309	0,391890821	False	0,391890821
ENSMUSG00000000085	Scmh1	890,4979299	905,6331704	678,717318	722,9759645	1488,963866	-0,391801433	False	0,391801433
ENSMUSG00000003526	Prodh	460,4046335	952,6915218	604,0364481	1215,812065	834,0570501	0,391733253	False	0,391733253
ENSMUSG00000017778	Cox7c	1062,535249	1771,890986	1393,676823	1427,989793	2141,497831	0,391385354	False	0,391385354
ENSMUSG00000000056	Narf	275,2597097	166,1447916	176,8179421	209,9324617	83,04977739	-0,390868188	False	0,390868188
ENSMUSG00000070462	Tlnrd1	1071,546727	936,365155	1116,918305	817,2771773	1685,910481	-0,390797411	False	0,390797411
ENSMUSG00000078427	Sarnp	303,1133708	459,0590196	397,5658076	397,4122538	612,1955019	0,390778864	False	0,390778864
ENSMUSG00000001768	Rin2	1988,259868	4874,861053	1516,680609	4392,864828	4284,182088	-0,390589024	False	0,390589024
ENSMUSG00000026618	Iars2	783,1794122	1019,917738	597,4469596	923,9273584	751,0072727	-0,390532202	False	0,390532202
ENSMUSG00000020149	Rab1	2728,839563	2418,223036	2268,980549	2048,806111	3577,072555	0,390491805	False	0,390491805
ENSMUSG00000047767	Atg16l2	294,1018922	123,8883128	385,4850787	281,7810048	71,18552348	0,390358951	False	0,390358951
ENSMUSG00000070934	Rraga	629,1650508	629,0453092	545,8292994	461,4023624	824,5656469	0,390195824	False	0,390195824
ENSMUSG00000032301	Psma4	948,6629281	1734,436379	1243,216835	1443,706662	1993,194657	0,390110469	False	0,390110469
ENSMUSG00000063801	Ap3s2	827,4175798	558,9379694	631,4926503	495,081367	480,5022835	-0,389849667	False	0,389849667
ENSMUSG00000050212	Eva1b	829,8752558	645,3716761	1087,265607	779,1076388	505,4172167	0,389738021	False	0,389738021
ENSMUSG00000020530	Ggnbp2	965,86666	2099,378696	1265,181797	1980,325468	3717,070751	0,389448763	False	0,389448763
ENSMUSG00000031565	Fgfr1	1180,503696	5561,528833	1546,333307	3426,277397	5041,121487	0,389448763	False	0,389448763
ENSMUSG00000004931	Apba3	453,8508309	594,4718266	595,2504634	617,4484169	677,4488984	0,38939024	False	0,38939024
ENSMUSG00000063931	Pepd	430,0932964	192,0749036	328,3761781	191,970326	157,794577	-0,389300174	False	0,389300174
ENSMUSG00000001119	Col6a1	3446,480949	2631,426179	4412,760815	3239,920238	3575,886129	-0,389279118	False	0,389279118
ENSMUSG00000020472	Zkscan17	683,2339223	1137,083429	521,6678415	876,776752	1563,708666	-0,389248106	False	0,389248106
ENSMUSG00000021994	Wnt5a	284,2711883	158,4617955	372,3061016	213,3003622	145,9303231	0,389221375	False	0,389221375
ENSMUSG00000020638	Cmpk2	512,0158291	207,4408959	390,9763191	190,8476925	239,657929	-0,389107184	False	0,389107184
ENSMUSG00000030557	Mef2a	2579,740553	2774,521982	1970,257069	2735,857804	2958,944926	-0,388842103	False	0,388842103
ENSMUSG00000029998	Pcyox1	1071,546727	481,1476335	752,2999399	818,3998107	352,3683412	-0,388817047	False	0,388817047
ENSMUSG00000038695	Josd2	666,8494158	230,4898843	873,1072295	316,5826428	174,4045325	0,388797831	False	0,388797831
ENSMUSG00000090035	Galnt4	481,704492	283,3104828	367,9131093	323,3184438	188,6416372	-0,388783295	False	0,388783295
ENSMUSG00000029053	Prkcz	1245,222496	787,5071047	951,0828437	940,7668606	597,9583972	-0,388760629	False	0,388760629
ENSMUSG00000002688	Prkd1	358,0014677	235,2917569	273,4637738	229,017231	212,370145	-0,388615773	False	0,388615773
ENSMUSG00000036989	Trim3	3475,97306	3309,450589	4137,100545	4029,131579	2655,220026	-0,388585486	False	0,388585486
ENSMUSG00000018008	Cyth4	1210,815033	1044,887476	941,1986109	918,3141909	1585,064323	0,388562895	False	0,388562895
ENSMUSG00000075590	Nrbp2	4446,755072	5929,352274	7106,763374	11037,73243	5820,60297	0,38841567	False	0,38841567
ENSMUSG00000004865	Srpk1	1085,473558	956,5330199	1225,644866	1365,122318	829,3113485	-0,388338842	False	0,388338842
ENSMUSG00000035367	Rmi1	276,8981604	211,2823939	362,4218688	293,0073396	177,9638087	0,388314543	False	0,388314543
ENSMUSG00000028015	Ctso	896,2325072	449,4552744	653,457612	684,806426	379,6561252	-0,388176816	False	0,388176816
ENSMUSG00000028029	Aimp1	274,4404844	540,6908536	359,1271246	463,6476294	562,3656355	0,388001305	False	0,388001305
ENSMUSG00000004961	Syt5	688,9684996	677,0640351	609,5276885	526,5151046	1035,749367	-0,387963103	False	0,387963103
ENSMUSG00000033082	Clec1a	1970,23691	193,0352781	1505,698128	1343,792282	79,49050121	-0,387936558	False	0,387936558
ENSMUSG00000029440	Psmd9	410,4318886	780,7844831	537,0433147	669,0895572	1118,799144	0,387895626	False	0,387895626
ENSMUSG00000092558	Med20	409,6126633	286,1916063	535,9450667	319,9505433	64,06697113	0,387824818	False	0,387824818
ENSMUSG00000040596	Pogk	449,7547043	306,3594712	343,7516514	275,0452039	264,5728623	-0,387771727	False	0,387771727
ENSMUSG00000028273	Pdlim5	1341,07186	973,8197612	1007,093496	955,3610959	1025,071538	-0,387661948	False	0,387661948
ENSMUSG00000025503	Taldo1	1834,245506	4365,862559	2399,672071	2922,214962	4674,516042	0,387650516	False	0,387650516
ENSMUSG00000004661	Arid3b	522,6657583	399,5157995	537,0433147	542,2319734	653,7203906	-0,387636089	False	0,387636089
ENSMUSG00000040661	Rad54l2	542,3271662	364,9423168	385,4850787	319,9505433	709,482384	0,387603424	False	0,387603424
ENSMUSG00000071337	Tia1	981,4319412	638,6490544	1283,852014	909,333123	275,2506908	0,387518786	False	0,387518786
ENSMUSG00000037204	Atg101	269,5251324	504,1966219	352,5376361	431,0912583	753,3801234	0,387357478	False	0,387357478
ENSMUSG00000030410	Dmwd	1340,252634	1958,203642	1752,803948	2103,815151	2620,813689	0,387159668	False	0,387159668
ENSMUSG00000034723	Tmx4	453,8508309	321,7254635	347,0463956	291,8847062	327,453408	-0,387089653	False	0,387089653
ENSMUSG00000074785	Plxnc1	471,0545628	127,7298109	360,2253727	168,3950228	86,60905356	-0,386994377	False	0,386994377
ENSMUSG00000075376	Rc3h2	899,5094085	1231,200132	1176,223702	1311,235911	1579,132196	0,38695219	False	0,38695219
ENSMUSG00000072115	Ang	563,6270247	426,406286	736,9244667	645,514254	342,8769381	0,386775966	False	0,386775966
ENSMUSG00000035517	Tdrd7	631,6227268	569,5020891	522,7660896	527,6377381	825,7520723	0,386645603	False	0,386645603
ENSMUSG00000037846	Rtkn2	3456,311653	218,9653901	4518,192632	2878,432256	49,82986643	0,386512471	False	0,386512471
ENSMUSG00000023921	Mut	614,4189949	356,2989462	470,0501814	456,9118285	173,2181071	-0,386408034	False	0,386408034
ENSMUSG00000032377	Plscr4	1113,327219	299,6368496	1455,178716	742,0607337	93,72760591	0,386318668	False	0,386318668
ENSMUSG00000020253	Ppm1m	652,9225852	397,5950504	853,3387639	751,0418016	163,726704	0,386206639	False	0,386206639
ENSMUSG00000064325	Hhip	353,0861157	50,89984945	270,1690295	96,54647973	64,06697113	-0,386157789	False	0,386157789
ENSMUSG00000027397	Slc20a1	749,5911738	1923,630159	979,637294	1579,545314	3032,5033	0,386143731	False	0,386143731
ENSMUSG00000027860	Vangl1	740,5796952	414,8817917	566,6960131	341,2805795	558,8063593	-0,386079942	False	0,386079942
ENSMUSG00000037211	Spry1	1895,687406	2643,911048	1450,785723	2345,181351	4068,252667	-0,385886633	False	0,385886633
ENSMUSG00000036103	Colec12	2061,990147	697,2319	1578,182502	1059,76601	1040,495068	-0,38577339	False	0,38577339
ENSMUSG00000027775	Mfsd1	1178,04602	799,0315989	901,6616798	761,145503	893,3783196	-0,385737784	False	0,385737784
ENSMUSG00000016382	Pls3	3215,459407	2313,542214	2461,173964	2108,305685	2219,801907	-0,385678241	False	0,385678241
ENSMUSG00000034674	Tdg	253,9598512	346,695201	194,3899115	273,9225704	474,5701565	-0,385647091	False	0,385647091
ENSMUSG00000047777	Phf13	259,6944285	311,1613438	198,7829038	261,5736021	453,2144995	-0,385621378	False	0,385621378
ENSMUSG00000063428	Ddo	351,4476651	192,0749036	269,0707814	216,6682627	105,5918598	-0,385324134	False	0,385324134
ENSMUSG00000029461	Fam168a	1266,522355	631,9264328	969,7530612	811,6640098	374,9104236	-0,385183212	False	0,385183212
ENSMUSG00000020869	Lrrc59	1522,939882	3113,534187	1166,339469	1476,263033	5771,959528	-0,384871239	False	0,384871239
ENSMUSG00000020781	Tsen54	190,8795011	263,1426179	260,2847967	249,2246337	323,8941318	0,384784496	False	0,384784496
ENSMUSG00000038055	Dexi	329,3285813	457,1382705	437,1027388	429,9686248	457,960201	0,384703665	False	0,384703665
ENSMUSG00000032076	Cadm1	2853,361812	1431,918406	2185,513694	1551,479477	1455,743955	-0,384690278	False	0,384690278
ENSMUSG00000033068	Entpd6	281,8135123	259,3011198	367,9131093	303,111041	193,3873388	0,384624295	False	0,384624295
ENSMUSG00000037805	Rpl10a	1878,483674	3078,960704	2452,387979	2871,696455	4608,07622	0,384618661	False	0,384618661
ENSMUSG00000028988	Ctnnbip1	187,6025998	332,2895832	244,9093235	305,356308	284,7420939	0,384567876	False	0,384567876
ENSMUSG00000035776	Cd99l2	906,0632111	328,4480851	694,0927912	516,4114032	385,5882522	-0,384483157	False	0,384483157
ENSMUSG00000054640	Slc8a1	378,4821009	154,6202974	289,9374951	158,2913214	141,1846216	-0,384483157	False	0,384483157
ENSMUSG00000021140	Pcnx	2208,63148	1804,543719	1747,312707	2882,92279	1842,518633	0,38437948	False	0,38437948
ENSMUSG00000018417	Myo1b	3692,248547	1414,631665	2829,087073	2320,483414	1698,96116	-0,384163092	False	0,384163092

ENSMUSG00000042834	Nrep	801,2023694	275,6274866	613,9206809	234,6303984	301,3520494	-0,384114417	False	0,384114417
ENSMUSG00000043496	Tril	566,0847006	67,22621626	433,8079945	106,6501811	67,6262473	-0,383961293	False	0,383961293
ENSMUSG00000041762	Gpr155	561,988574	897,9501743	646,8681235	1323,584879	430,672417	-0,383949872	False	0,383949872
ENSMUSG00000078671	Chd2	1017,477856	1568,291588	779,7561421	1453,810363	3075,214614	-0,383902479	False	0,383902479
ENSMUSG00000020048	Hsp90b1	7783,459827	3468,872759	5965,683611	3343,202519	3610,292466	-0,383724124	False	0,383724124
ENSMUSG00000030729	Pgm2l1	529,2195609	475,3853864	474,4431738	690,4195934	290,6742209	0,38360702	False	0,38360702
ENSMUSG00000032398	Snapc5	391,5897061	416,8025408	553,5170361	445,6854936	300,165624	-0,383584077	False	0,383584077
ENSMUSG00000071528	Usmg5	731,5682166	1606,706569	954,377588	1022,719105	1755,909579	0,383567766	False	0,383567766
ENSMUSG00000060716	Plekhh1	335,0631586	798,0712244	437,1027388	958,7289964	592,0262702	0,383539352	False	0,383539352
ENSMUSG00000060073	Psma3	1155,10771	2148,357797	1506,796376	1634,554355	2900,810082	0,383457084	False	0,383457084
ENSMUSG00000028949	Smarcd3	278,536611	233,3710079	354,7341322	325,5637107	213,5565704	-0,383248683	False	0,383248683
ENSMUSG00000003848	Nob1	211,3601342	371,6649384	275,6602699	334,5447786	790,1593106	0,383188063	False	0,383188063
ENSMUSG00000033590	Myo5c	2293,011689	1689,298777	2990,529542	2391,209324	1205,408198	0,38315726	False	0,38315726
ENSMUSG00000024580	Grpel2	452,2123802	803,8334715	589,7592229	864,4277836	971,6823954	0,383125586	False	0,383125586
ENSMUSG00000027472	Pdrg1	512,0158291	423,5251624	667,7348372	534,373539	314,4027287	0,383086899	False	0,383086899
ENSMUSG00000022610	Mapk12	811,8522986	1459,769267	1058,711156	1823,15678	1179,306839	0,383019854	False	0,383019854
ENSMUSG00000048490	Nrip1	386,6743541	595,4322011	296,5269836	418,74229	1198,289645	-0,382955688	False	0,382955688
ENSMUSG00000026271	Gpr35	262,9713298	201,6786488	163,638965	107,7728146	137,6253454	-0,382847162	False	0,382847162
ENSMUSG00000023084	Lrrc71	523,4849837	294,834977	559,0082765	682,561159	302,5384748	0,382810167	False	0,382810167
ENSMUSG00000061086	Myl4	507,1004771	388,9516798	237,2215869	68,4806426	109,151136	-0,382680694	False	0,382680694
ENSMUSG00000038168	P3h2	323,594004	192,0749036	248,2040678	161,6592219	181,5230849	-0,382658116	False	0,382658116
ENSMUSG00000038722	Bud31	532,4964622	939,2462785	694,0927912	708,3817292	1447,438977	0,382356605	False	0,382356605
ENSMUSG00000021143	Pacs2	1524,578333	1029,521483	1169,634213	1028,332272	720,1602125	-0,38235286	False	0,38235286
ENSMUSG00000022723	Crybg3	1087,931234	1245,60575	834,6685464	1123,756119	1509,133098	-0,382312058	False	0,382312058
ENSMUSG00000038187	Btbd10	229,3830914	224,7276372	202,0776481	162,7818554	298,9791986	0,382286063	False	0,382286063
ENSMUSG00000057123	Gja5	734,8451179	35,53385716	957,6723322	79,70697745	4,745701565	0,382091914	False	0,382091914
ENSMUSG00000030275	Etnk1	2201,258452	1379,097808	1689,105558	1275,311639	1265,915892	-0,382069053	False	0,382069053
ENSMUSG00000019866	Crybg1	333,4247079	462,9005176	255,8918044	267,1867695	564,7384862	-0,381827075	False	0,381827075
ENSMUSG00000052428	Tmco1	1107,592641	367,8234404	850,0440197	493,9587335	288,3013701	0,381817916	False	0,381817916
ENSMUSG00000076432	Ywhaq	2416,714713	1455,927769	1854,94102	1200,095196	1662,181973	-0,381673862	False	0,381673862
ENSMUSG00000059810	Rgs3	1309,122072	530,1267339	1004,897	743,1833672	469,8244549	-0,381551995	False	0,381551995
ENSMUSG00000029725	Ppp1r35	184,3256985	240,0936295	308,6077126	270,5546699	249,1493322	0,381339901	False	0,381339901
ENSMUSG00000022552	Sharpin	1061,716023	2000,460121	1382,694342	1698,544463	2325,393767	0,381084329	False	0,381084329
ENSMUSG00000039062	Anpep	2185,693171	1393,503426	1595,754471	1678,33706	1061,850725	-0,381058408	False	0,381058408
ENSMUSG00000032519	Slc25a38	474,3314641	477,3061354	657,8506044	612,9578829	364,2325951	-0,381035536	False	0,381035536
ENSMUSG00000079037	Prnp	2419,991615	2508,498241	1684,712566	1800,70411	3151,145839	0,380874477	False	0,380874477
ENSMUSG00000073468	Sft2d1	393,2281567	418,7232898	382,1903344	301,9884075	590,8398448	-0,380873459	False	0,380873459
ENSMUSG00000052013	Btla	543,9656168	417,7629153	286,6427508	339,0353125	209,9972943	-0,380831034	False	0,380831034
ENSMUSG00000056724	Nbeal2	1495,905446	1135,16268	1805,519856	1947,769097	1063,037151	0,380803672	False	0,380803672
ENSMUSG00000004951	Hspb1	2108,685991	8470,503248	2745,620219	3427,40003	19736,18638	0,380787806	False	0,380787806
ENSMUSG00000030516	Tjp1	6188,428117	2907,053666	4753,217722	4327,752086	2428,612776	-0,38066852	False	0,38066852
ENSMUSG00000069792	Wfdc17	624,2496988	415,8421663	371,2078535	534,373539	812,701393	0,380602147	False	0,380602147
ENSMUSG00000028383	Hsdl2	303,1133708	222,8068882	232,8285945	223,4040636	194,5737642	-0,380589241	False	0,380589241
ENSMUSG00000046434	Hnrnpa1	1740,034594	1969,728136	1336,567922	2108,305685	3446,565762	-0,380582833	False	0,380582833
ENSMUSG00000020611	Gna13	1531,132135	2076,329708	1176,223702	1353,895983	3848,763969	-0,380436324	False	0,380436324
ENSMUSG00000036019	Tmtc2	896,2325072	180,5504094	688,6015508	307,601575	96,10045669	-0,380203625	False	0,380203625
ENSMUSG00000029049	Morn1	201,5294303	237,2125059	154,8529803	285,1489053	250,3357576	-0,380091388	False	0,380091388
ENSMUSG00000035078	Mtmr9	531,6772369	357,2593207	408,5482885	324,4410772	339,3176619	-0,380044092	False	0,380044092
ENSMUSG00000020471	Pold2	464,5007601	335,1707068	356,9306284	236,8756654	332,1991096	-0,380037252	False	0,380037252
ENSMUSG00000033327	Tnxb	1649,919808	1223,517136	2147,075011	1265,207938	913,5475513	0,379976689	False	0,379976689
ENSMUSG00000029381	Shroom3	2651,013157	3330,578828	2037,250202	3485,776972	2926,91144	-0,379920656	False	0,379920656
ENSMUSG00000030353	Tead4	330,1478066	311,1613438	358,0288765	253,7151677	803,2099899	-0,379902292	False	0,379902292
ENSMUSG00000028433	Ubap2	1694,977201	1292,664101	1569,396517	1507,696771	2205,564802	0,379882282	False	0,379882282
ENSMUSG00000073684	Faap20	501,3658998	464,8212667	652,3593639	522,0245706	345,2497889	0,379803043	False	0,379803043
ENSMUSG00000032997	Chpf	927,3630696	1206,230395	712,7630087	735,3249328	1784,383788	-0,37971181	False	0,37971181
ENSMUSG00000030706	Mrpl48	437,4663244	683,7866568	660,0471005	569,175177	615,7547781	0,37970078	False	0,37970078
ENSMUSG00000052331	Ankrd44	1949,756277	1147,647549	1119,114801	1498,715703	857,7855579	-0,379567057	False	0,379567057
ENSMUSG00000029249	Rest	357,1822424	448,4948999	274,5620219	398,5348873	708,2959586	-0,379528275	False	0,379528275
ENSMUSG00000046574	Prr12	1104,31574	645,3716761	848,9457716	784,7208062	734,3973172	-0,379408413	False	0,379408413
ENSMUSG00000033777	Tlr13	345,7130878	676,1036606	265,7760372	415,3743895	797,2778629	-0,379364187	False	0,379364187
ENSMUSG00000021716	Srek1ip1	265,4290058	254,4992473	369,0113574	298,6205071	204,0651673	-0,379296092	False	0,379296092
ENSMUSG00000012848	Rps5	5065,270194	10933,86389	6588,390276	9480,639783	14642,86218	0,379286791	False	0,379286791
ENSMUSG00000035936	Aldh5a1	240,852246	245,8558766	154,8529803	174,0081902	313,2163033	0,379010931	False	0,379010931
ENSMUSG00000049521	Cdc42ep1	3713,548405	2464,321013	2544,640819	1832,137848	2855,725917	-0,378940863	False	0,378940863
ENSMUSG00000020697	Lig3	522,6657583	323,6462125	401,9588	392,9217198	204,0651673	-0,378841011	False	0,378841011
ENSMUSG00000050821	Fam131a	217,9139369	205,5201468	283,3480066	254,8378012	61,69412035	0,378816585	False	0,378816585
ENSMUSG00000030654	Arl6ip1	3593,941508	2092,656075	4114,037335	4672,400566	2270,818199	0,378597033	False	0,378597033
ENSMUSG00000018654	Ikzf1	614,4189949	812,4768422	526,0608339	472,6286973	855,4127071	-0,378515588	False	0,378515588
ENSMUSG00000030499	Kctd15	297,3787935	151,7391738	386,5833268	181,8666246	164,9131294	0,378477645	False	0,378477645
ENSMUSG00000084957	Bbip1	393,2281567	430,247784	452,478212	429,9686248	302,5384748	-0,378248002	False	0,378248002
ENSMUSG00000039543	Cfap70	376,8436502	196,8767762	289,9374951	221,1587966	168,4724056	-0,378224167	False	0,378224167
ENSMUSG00000047669	Msl3l2	326,8709053	98,91857535	251,498812	139,2065522	116,2696883	-0,378169384	False	0,378169384
ENSMUSG00000095440	Fignl2	290,8249909	223,7672627	505,1941202	326,6863442	287,1149447	-0,378152263	False	0,378152263
ENSMUSG00000090509	Sfta2	1335,337282	1735,396754	2019,678233	2003,900771	2108,27792	0,378061349	False	0,378061349
ENSMUSG00000024990	Rbp4	346,5323131	705,8752707	450,2817158	617,4484169	535,0778515	0,377838011	False	0,377838011
ENSMUSG00000057337	Chst3	746,3142725	236,2521314	574,3837497	161,6592219	174,4045325	-0,377768343	False	0,377768343
ENSMUSG00000014867	Surf4	3308,851094	4796,110343	2546,837315	2789,744211	7236,008461	-0,377623561	False	0,377623561
ENSMUSG00000028156	Eif4e	816,7676506	839,3673287	835,7667945	628,6747517	1218,458877	-0,377611903	False	0,377611903
ENSMUSG00000035530	Eif1	4163,303109	11419,81339	5408,871831	8027,952053	21587,00999	0,377599114	False	0,377599114
ENSMUSG00000026309	Ilkap	820,8637772	1147,647549	1066,398893	1444,829296	1271,848019	0,377532457	False	0,377532457
ENSMUSG00000037364	Srrt	1851,449238	3040,545724	2405,163311	3042,336745	4344,689783	0,377479862	False	0,377479862
ENSMUSG00000025040	Fundc1	544,7848421	294,834977	419,5307694	405,2706882	340,5040873	-0,376909938	False	0,376909938
ENSMUSG00000011171	Vipr2	379,3013262	203,5993978	292,1339913	205,4419278	236,0986529	-0,376714183	False	0,376714183
ENSMUSG00000039959	Hip1	2559,25992	867,2181897	1971,355317	1569,441612	633,5511589	-0,376538845	False	0,376538845
ENSMUSG00000021374	Nup153	1257,510876	1061,213842	846,7492754	868,9183176	1632,521338	0,376530971	False	0,376530971
ENSMUSG00000002329	Mdp1	388,3128048	290,0331044	504,0958721	295,2526066	196,9466149	0,376478859	False	0,376478859
ENSMUSG00000029502	Golga3	658,6571625	463,8608922	507,3906164	481,6097652	486,4344104	-0,376430885	False	0,376430885
ENSMUSG00000038855	Itpkb	4928,459564	2319,304461	3796,643638	2300,276011	3428,769381	-0,376412199	False	0,376412199
ENSMUSG00000067212	H2-T23	1959,586981	1421,354287	2543,54257	2119,53202	1687,096906	0,376289628	False	0,376289628
ENSMUSG00000025266	Gnl3l	489,8967453	377,4271855	337,1621628	294,1299731	367,7918713	-0,376279365	False	0,376279365
ENSMUSG00000027820	Mme	1787,549663	417,7629153	1377,203102	607,3447155	421,1810139	-0,376241989	False	0,376241989
ENSMUSG00000032322	Pstpip1	334,2439332	678,0244097	433,8079945	478,2418647	1162,696883	0,376155266	False	0,376155266
ENSMUSG00000026811	St6galnac6	668,4878665	202,6390233	515,078353	346,8937469	160,1674278	-0,376109465	False	0,376109465
ENSMUSG00000020483	Dynll2	2144,731905	903,7124214	1652,863372	935,1536932	791,345736	-0,375829846	False	0,375829846
ENSMUSG00000027435	Cd93	9795,477229	9375,176044	8951,820161	12710,45632	7578,885399	0,375828139	False	0,375828139
ENSMUSG00000034243	Golgb1	1628,619949	1653,76492	1580,378998	2112,796219	1138,968376	0,375503646	False	0,375503646
ENSMUSG00000054509	Parp4	786,4563135	945,9689002	606,2329443	783,5981727	1644,385592	-0,375494374	False	0,375494374
ENSMUSG00000025138	Sirt7	920,809267	1272,496236	1521,073601	1194,482028	1595,742151	0,37541089	False	0,37541089
ENSMUSG00000023087	Noct	230,2023168	764,4581163	300,919976	298,6205071	1351,338521	0,37541089	False	0,37541089
ENSMUSG00000021567	Nkd2	454,6700562	85,4733321	589,7592229	504,0624349	115,083263	0,375306081	False	0,375306081
ENSMUSG00000024620	Pdgfrb	2055,436344	3005,972241	1584,77199	2366,511387	3249,619147	-0,375169406	False	0,375169406
ENSMUSG00000000552	Zfp385a	423,5394938	320,765089	367,9131093	363,7332492	549,3149562	0,37513707	False	0,37513707
ENSMUSG00000053286	Trmt1l	653,7418106	491,7117532	847,8475235	792,5792406	212,370145	0,375083866	False	0,375083866
ENSMUSG00000026278	Bok	202,3486557	289,0727299	162,5407169	156,0460544	448,4687979	-0,374871384	False	0,374871384
ENSMUSG00000006134	Crkl	765,9756803	674,1829116	783,0508863	602,8541816	993,0380525	0,374550414	False	0,374550414
ENSMUSG00000034902	Pip5k1c	2737,851041	1927,471657	2077,885381	1859,081052	2111,837196	-0,374545336	False	0,374545336
ENSMUSG00000027014	Cwc22	260,5136538	329,4084597	200,9794	252,5925342	415,2488869	-0,374311352	False	0,374311352
ENSMUSG00000009563	Tor2a	461,2238588	560,8587185	355,8323803	483,8550321	537,4507022	-0,374269345	False	0,374269345
ENSMUSG00000018541	Cwc25	345,7130878	630,9660583	448,0852197	560,1941091	1067,782852	0,374197918	False	0,374197918
ENSMUSG00000040174	Alkbh3	246,5868233	497,4740003	319,5901934	353,6295479	612,1955019	0,374127431	False	0,374127431

ENSMUSG00000025377	Tepsin	326,05168	422,5647879	499,7028798	428,8459914	431,8588424	0,3740719	False	0,3740719
ENSMUSG00000004393	Ddx56	459,5854082	413,9214172	419,5307694	381,695385	595,5855464	0,37397575	False	0,37397575
ENSMUSG00000042851	Zc3h6	122,883799	486,9098806	159,2459727	452,4212946	520,8407468	0,373962164	False	0,373962164
ENSMUSG00000039159	Ube2h	2359,36894	2133,952179	1772,572413	1893,88269	3057,418233	0,373912882	False	0,373912882
ENSMUSG00000015023	Ddx19a	552,1578701	720,2808884	426,1202579	581,5241454	893,3783196	-0,373820174	False	0,373820174
ENSMUSG00000031370	Zrsr2	269,5251324	379,3479346	349,2428918	383,940652	594,399121	0,373810946	False	0,373810946
ENSMUSG00000005575	Ube2m	856,9096916	1647,042298	1110,328816	1357,263884	1913,704156	0,37377191	False	0,37377191
ENSMUSG00000039148	Sart1	905,2439858	1395,424175	1172,928957	1466,159332	2395,392865	0,373737042	False	0,373737042
ENSMUSG00000026803	Ttf1	293,2826669	263,1426179	379,9938382	309,8468419	202,8787419	0,373684215	False	0,373684215
ENSMUSG00000046280	She	1081,377431	303,4783477	834,6685464	452,4212946	325,0805572	-0,373594841	False	0,373594841
ENSMUSG00000036822	Topors	432,5509724	406,2384211	333,8674186	440,0723262	745,0751457	-0,373594841	False	0,373594841
ENSMUSG00000036580	Spg20	795,4677921	438,8911547	534,8468186	614,0805164	409,31676	-0,373375688	False	0,373375688
ENSMUSG00000005774	Rfx5	706,9914568	394,7139269	545,8292994	471,5060638	399,8253569	-0,373242943	False	0,373242943
ENSMUSG00000063049	Ing2	167,9411919	245,8558766	217,4531213	300,865774	412,8760362	0,372748285	False	0,372748285
ENSMUSG00000026581	Sell	1451,667279	631,9264328	1121,311297	847,5882814	276,4371162	-0,372523973	False	0,372523973
ENSMUSG00000002059	Rab34	381,7590022	899,8709233	494,2116393	854,3240823	1041,681494	0,372466811	False	0,372466811
ENSMUSG00000020680	Taf15	638,9957547	493,6325022	701,7805279	730,8343989	666,7710699	-0,372368957	False	0,372368957
ENSMUSG00000025337	Sbds	1162,480738	1753,64387	1504,59988	1921,948527	2448,782008	0,372173068	False	0,372173068
ENSMUSG00000014195	Dnajc7	1782,634311	1869,849186	2271,177045	2776,272609	1377,439879	-0,372021428	False	0,372021428
ENSMUSG00000055912	Tmem150a	689,7877249	533,0078575	951,0828437	926,1726253	556,4335085	-0,371995655	False	0,371995655
ENSMUSG00000021062	Rab15	466,1392108	465,7816412	360,2253727	472,6286973	580,1620163	-0,371861073	False	0,371861073
ENSMUSG00000031661	Nkd1	3628,348971	6856,113684	4695,010574	6743,659346	7651,257348	0,371815187	False	0,371815187
ENSMUSG00000020523	Fam114a2	917,5323657	756,7751201	1187,206183	1062,011277	622,8733304	0,371739555	False	0,371739555
ENSMUSG00000007039	Ddah2	1080,558206	592,5510776	1398,069815	693,7874939	625,2461812	0,371659619	False	0,371659619
ENSMUSG00000038812	Trmt112	467,7776615	764,4581163	657,8506044	605,0994485	1154,391906	0,371349302	False	0,371349302
ENSMUSG00000026113	Inpp4a	581,6499818	560,8587185	688,6015508	730,8343989	449,6552233	-0,37133202	False	0,37133202
ENSMUSG00000025982	Sf3b1	3897,054878	5822,750702	5040,958721	6663,952368	7387,870911	0,371313893	False	0,371313893
ENSMUSG00000056515	Rab31	1164,119189	1059,293093	591,9557191	742,0607337	1505,573822	0,371074671	False	0,371074671
ENSMUSG00000010097	Nxf1	2426,545417	3256,62999	3363,933892	3137,760591	3677,918713	0,370831417	False	0,370831417
ENSMUSG00000032077	Bud13	298,1980189	243,9351276	247,1058197	220,0361631	385,5882522	0,370790424	False	0,370790424
ENSMUSG00000052302	Tbc1d30	910,1593378	489,7910041	696,2892874	703,8911952	344,0633635	-0,370766694	False	0,370766694
ENSMUSG00000040274	Cdk6	915,8939151	435,0496566	708,3700164	491,7134665	417,6217377	-0,370677358	False	0,370677358
ENSMUSG00000009097	Tbx1	529,2195609	812,4768422	684,2085585	764,5134035	893,3783196	0,370569763	False	0,370569763
ENSMUSG00000044393	Dsg2	404,6973113	425,4459114	264,6777891	389,5538194	523,2135975	0,370556771	False	0,370556771
ENSMUSG00000019579	Mydgf	521,0273077	654,9754212	518,3730973	403,0254212	804,3964153	-0,370488147	False	0,370488147
ENSMUSG00000031231	Cox7b	664,3917398	1016,07624	876,4019738	858,8146162	968,1231193	0,370312608	False	0,370312608
ENSMUSG00000026003	Acadl	1211,634258	3316,17321	1566,101773	3401,57946	2986,23271	0,370223694	False	0,370223694
ENSMUSG00000007034	Slc44a4	434,1894231	516,6814906	261,3830448	335,6674121	561,1792101	0,370136981	False	0,370136981
ENSMUSG00000061024	Rrs1	159,7489387	328,4480851	206,4706404	295,2526066	650,1611144	0,370130303	False	0,370130303
ENSMUSG00000022521	Crebbp	956,0359561	1762,28724	1235,529098	1695,176563	3164,196518	0,369992205	False	0,369992205
ENSMUSG00000066363	Serpina3f	67,9957021	922,9199117	87,85984699	880,1446524	888,6326181	0,369760428	False	0,369760428
ENSMUSG00000020882	Cacnb1	430,0932964	246,8162511	555,7135322	297,4978736	192,2009134	0,369691727	False	0,369691727
ENSMUSG00000028494	Plin2	1606,500865	3995,157995	2075,688885	2626,962355	5080,273525	0,369668464	False	0,369668464
ENSMUSG00000029196	Tada2b	452,2123802	945,9689002	584,2679825	770,1265709	1249,305937	0,369629743	False	0,369629743
ENSMUSG00000024985	Tcf7l2	271,1635831	562,7794675	350,3411399	546,7225073	837,6163262	0,369596972	False	0,369596972
ENSMUSG00000033909	Usp36	790,5524401	1243,685001	1021,370721	1261,840037	2433,358477	0,369573538	False	0,369573538
ENSMUSG00000028960	Ube4b	1275,533833	1405,02792	987,3250306	1456,05563	1740,486049	-0,369504159	False	0,369504159
ENSMUSG00000045655	Fam216b	646,3687826	500,3551238	444,7904754	437,8270592	142,371047	-0,369405121	False	0,369405121
ENSMUSG00000028468	Rgp1	1089,569684	814,3975912	843,4545311	752,1644351	781,8543328	-0,369376265	False	0,369376265
ENSMUSG00000016503	Gtf3a	343,2554118	356,2989462	449,1834678	359,2427153	265,7592876	-0,369162355	False	0,369162355
ENSMUSG00000026930	Gpsm1	537,4118142	1260,971742	694,0927912	1213,566798	1634,894189	0,369100508	False	0,369100508
ENSMUSG00000040653	Ppp1r14c	1388,586928	528,2059849	1075,184878	678,0706251	304,9113256	-0,369032744	False	0,369032744
ENSMUSG00000033502	Cdc14a	855,2712409	1253,288746	662,2435967	1122,633485	1211,340324	-0,36902004	False	0,36902004
ENSMUSG00000020661	Dnmt3a	680,7762464	329,4084597	527,159082	444,5628602	322,7077064	-0,368942307	False	0,368942307
ENSMUSG00000057367	Birc2	557,8924474	1648,963047	720,4507453	1048,539675	2415,562097	0,368912781	False	0,368912781
ENSMUSG00000039318	Rab3gap2	653,7418106	838,4069542	506,2923683	798,192408	928,9710814	-0,368750231	False	0,368750231
ENSMUSG00000030738	Eif3c	2537,960062	3654,225041	1965,864076	2585,424917	5004,3423	-0,368505792	False	0,368505792
ENSMUSG00000022020	Naa16	194,1564024	250,6577492	341,5551552	296,3752401	284,7420939	0,368499555	False	0,368499555
ENSMUSG00000003190	Bcl2l12	308,0287228	184,3919074	397,5658076	200,9513939	327,453408	0,3681288	False	0,3681288
ENSMUSG00000022508	Bcl6	997,8164477	1009,353618	773,1666535	1001,389069	1268,288743	-0,367995035	False	0,367995035
ENSMUSG00000010517	Faf1	616,0574456	704,9148962	417,3342732	527,6377381	794,9050121	0,36771759	False	0,36771759
ENSMUSG00000004207	Psap	16698,26983	8292,833962	12941,75546	9113,538633	10485,62761	-0,367665305	False	0,367665305
ENSMUSG00000037887	Dusp8	786,4563135	789,4278537	909,3494164	609,5899825	1328,796438	-0,367527433	False	0,367527433
ENSMUSG00000001525	Tubb5	5419,99476	2256,880117	4201,897182	3076,01575	2562,678845	-0,367250596	False	0,367250596
ENSMUSG00000050549	Fam241a	259,6944285	400,476174	334,9656667	362,6106157	492,3665374	0,367198167	False	0,367198167
ENSMUSG00000030652	Coq7	172,8565439	479,2268844	222,9443617	236,8756654	476,9430073	0,367108493	False	0,367108493
ENSMUSG00000018189	Uchl5	194,1564024	386,0705562	250,4005639	438,9496927	363,0461697	0,36701853	False	0,36701853
ENSMUSG00000024193	Phf1	2709,99738	3494,802871	3963,577347	3867,472357	4395,706075	0,366919624	False	0,366919624
ENSMUSG00000023039	Krt7	5597,766656	9984,053488	7217,686431	9754,562353	9579,198609	0,366685118	False	0,366685118
ENSMUSG00000006736	Tspan31	1650,739033	1469,373012	2128,404793	1594,139549	1204,221772	0,366660497	False	0,366660497
ENSMUSG00000029571	Tmem106b	1550,793543	1696,981773	1208,072896	1999,410237	1255,238064	0,36656788	False	0,36656788
ENSMUSG00000030826	Bcat2	610,3228683	566,6209656	904,956424	901,4746887	473,3837311	-0,366562522	False	0,366562522
ENSMUSG00000040339	Fam102b	651,2841346	709,7167688	505,1941202	1035,068073	628,8054574	-0,366449235	False	0,366449235
ENSMUSG00000036615	Rfxap	179,4103465	243,9351276	250,4005639	231,262498	457,960201	0,366268242	False	0,366268242
ENSMUSG00000024293	Esco1	262,1521045	339,9725793	361,3236208	337,9126791	380,8425506	0,366246356	False	0,366246356
ENSMUSG00000042682	Selenok	716,0029354	1091,945827	1018,075977	922,8047249	1436,761149	0,366059889	False	0,366059889
ENSMUSG00000026021	Sumo1	646,3687826	855,6936955	885,1879585	832,9940461	1143,714077	0,365948661	False	0,365948661
ENSMUSG00000064215	Ifi27	994,5395464	480,187259	1281,655518	531,0056385	475,7565819	0,365907904	False	0,365907904
ENSMUSG00000032328	Tmem30a	3236,759265	1945,718773	2511,693376	2469,793668	1383,372006	-0,365889716	False	0,365889716
ENSMUSG00000028378	Ptgr1	302,2941455	274,6671121	305,3129683	234,6303984	438,9773948	0,365563099	False	0,365563099
ENSMUSG00000044533	Rps2	8804,214584	14713,89799	11342,70625	13132,56651	19743,30494	0,365498679	False	0,365498679
ENSMUSG00000037876	Jmjd1c	984,7088425	1825,671959	764,3806688	1306,745377	2217,429056	-0,36540592	False	0,36540592
ENSMUSG00000019777	Hdac2	754,5065257	675,1432861	971,9495574	776,8623718	468,6380295	0,365348063	False	0,365348063
ENSMUSG00000033863	Klf9	2386,403376	10010,94397	3073,996397	11921,24498	12656,78607	0,36527755	False	0,36527755
ENSMUSG00000003226	Ranbp2	1333,698832	1253,288746	1115,820057	1166,416191	1717,943967	0,365250059	False	0,365250059
ENSMUSG00000030785	Cox6a2	480,0660414	821,1202128	618,3136732	836,3619465	651,3475398	0,365106022	False	0,365106022
ENSMUSG00000037321	Tap1	1328,78348	1143,806051	1152,062244	1031,700173	2071,498733	-0,365082278	False	0,365082278
ENSMUSG00000036752	Tubb4b	6188,428117	7882,754043	5340,780449	4804,871317	11140,53442	-0,365075213	False	0,365075213
ENSMUSG00000039706	Ldb2	1767,888255	756,7751201	1372,810109	770,1265709	340,5040873	-0,364895005	False	0,364895005
ENSMUSG00000040242	Fgfr1op2	1720,373186	1602,86507	2003,204511	2071,25878	1335,914991	-0,364893344	False	0,364893344
ENSMUSG00000018412	Kansl1	1427,090519	1206,230395	978,5390459	1046,294408	1837,772931	0,364881677	False	0,364881677
ENSMUSG00000037348	Paqr7	1912,071912	811,5164677	2462,272212	1861,326319	290,6742209	0,364853482	False	0,364853482
ENSMUSG00000045679	Pqlc3	656,1994866	337,0914558	509,5871126	403,0254212	258,6407353	-0,364805673	False	0,364805673
ENSMUSG00000021932	Rnaseh2b	165,483516	386,0705562	213,060129	255,9604346	332,1991096	0,364573124	False	0,364573124
ENSMUSG00000023020	Cox14	661,9340638	510,9192435	852,2405158	525,3924711	537,4507022	0,364573124	False	0,364573124
ENSMUSG00000032812	Arap1	1716,277059	988,225379	1333,273178	963,2195303	829,3113485	-0,364310058	False	0,364310058
ENSMUSG00000004568	Arhgef18	1738,396143	968,0575141	1338,764419	1350,528083	358,3004682	-0,364233239	False	0,364233239
ENSMUSG00000024248	Cox7a2l	2039,871063	2120,506936	1584,77199	1917,457993	3033,689725	-0,364202678	False	0,364202678
ENSMUSG00000025232	Hexa	2024,305782	586,7888305	1572,691261	821,7677112	1017,952986	-0,364191753	False	0,364191753
ENSMUSG00000079084	Ccdc82	1189,515174	2321,22521	1530,957834	2657,27346	2270,818199	0,364060872	False	0,364060872
ENSMUSG00000052707	Tnrc6a	2192,246974	5229,23925	2821,399337	5742,270277	5018,579405	0,364000539	False	0,364000539
ENSMUSG00000090173	Fbxw10	20,48063316	522,4437378	26,3579541	102,1596472	1321,677886	0,363978076	False	0,363978076
ENSMUSG00000008301	Phax	430,0932964	577,1850853	553,5170361	678,0706251	997,783754	0,363978076	False	0,363978076
ENSMUSG00000038174	Fam126b	425,9971698	465,7816412	487,6221508	529,883005	331,0126842	-0,363957345	False	0,363957345
ENSMUSG00000026822	Lcn2	4480,343311	55886,11395	5765,802459	33369,15772	106765,2345	0,363912125	False	0,363912125
ENSMUSG00000014294	Ndufa2	429,2740711	756,7751201	552,418788	560,1941091	898,1240212	0,363863353	False	0,363863353
ENSMUSG00000038605	Samd10	395,6858327	444,6534018	307,5094645	319,9505433	511,3493436	-0,363724597	False	0,363724597
ENSMUSG00000028901	Gmeb1	353,9053411	323,6462125	397,5658076	275,0452039	516,0950452	-0,363694789	False	0,363694789
ENSMUSG00000041891	Lman1	1352,541014	898,9105488	862,1247486	665,7216568	1051,172897	-0,36367236	False	0,36367236
ENSMUSG00000040820	Hlcs	308,0287228	188,2334055	239,4180831	180,7439911	141,1846216	-0,363532762	False	0,363532762

ENSMUSG00000024070	Prkd3	1504,916925	1037,204479	1157,553484	1082,21868	1169,815436	-0,363402918	False	0,363402918
ENSMUSG00000034738	Nostrin	897,0517326	563,739842	697,3875355	422,1101905	463,892328	-0,363230608	False	0,363230608
ENSMUSG00000021596	Mctp1	267,0674565	168,0655406	265,7760372	343,5258465	202,8787419	0,363214463	False	0,363214463
ENSMUSG00000038791	Scgb3a2	13436,11458	9680,575141	10446,53581	3191,646999	9655,129834	-0,363091397	False	0,363091397
ENSMUSG00000070044	Fam149a	817,5868759	635,7679309	494,2116393	489,4681996	485,247985	-0,362871791	False	0,362871791
ENSMUSG00000030172	Erc1	460,4046335	266,0237415	358,0288765	276,1678374	325,0805572	-0,362826401	False	0,362826401
ENSMUSG00000062078	Qk	4920,267311	5333,920073	3826,296337	5903,929499	6085,175832	-0,362788086	False	0,362788086
ENSMUSG00000032489	Kif9	378,4821009	209,3616449	294,3304874	288,5168057	143,5574723	-0,362788086	False	0,362788086
ENSMUSG00000060450	Rnf14	1440,198124	1611,508441	1582,575494	1621,082753	1119,985569	-0,362787149	False	0,362787149
ENSMUSG00000005078	Jkamp	541,5079408	354,3781971	696,2892874	483,8550321	174,4045325	0,362704333	False	0,362704333
ENSMUSG00000031913	Vps4a	982,2511665	1576,934958	1283,852014	1262,962671	1967,093299	0,362648117	False	0,362648117
ENSMUSG00000032018	Sc5d	842,982861	559,898344	655,6541082	454,6665615	270,5049892	-0,362568381	False	0,362568381
ENSMUSG00000042744	Hectd4	1029,766235	800,952348	1324,487193	1283,170074	836,4299008	-0,362528554	False	0,362528554
ENSMUSG00000046879	Irgm1	642,272656	428,327035	508,4888645	431,0912583	825,7520723	0,362522808	False	0,362522808
ENSMUSG00000022371	Col14a1	408,7934379	112,3638186	177,9161902	68,4806426	317,9620049	-0,362517658	False	0,362517658
ENSMUSG00000026121	Sema4c	464,5007601	923,8802863	361,3236208	373,8369506	2224,547609	-0,362389387	False	0,362389387
ENSMUSG00000031967	Afg3l1	464,5007601	294,834977	361,3236208	266,064136	303,7249002	-0,362389387	False	0,362389387
ENSMUSG00000001504	Irx2	977,3358146	1601,904696	1256,395812	1482,998834	1320,49146	0,362364774	False	0,362364774
ENSMUSG00000026499	Acbd3	706,9914568	608,8774444	579,8749902	492,8361	908,8018497	0,362272989	False	0,362272989
ENSMUSG00000042485	Mustn1	561,1693487	674,1829116	675,4225738	715,1175301	436,604544	-0,362109065	False	0,362109065
ENSMUSG00000026698	Pigc	436,647099	338,0518303	561,2047727	453,543928	170,8452563	0,362059523	False	0,362059523
ENSMUSG00000054716	Zfp771	237,5753447	566,6209656	305,3129683	557,9488422	662,0253683	0,361903747	False	0,361903747
ENSMUSG00000024349	Tmem173	480,0660414	561,819093	348,1446437	361,4879822	616,9412035	0,361900115	False	0,361900115
ENSMUSG00000071076	Jund	4054,346141	7846,259812	5210,088927	5940,976404	17410,79262	0,361838732	False	0,361838732
ENSMUSG00000022575	Gsdmd	1003,551025	605,9963208	1289,343255	1008,12487	382,028976	0,361522424	False	0,361522424
ENSMUSG00000016252	Atp5e	578,3730805	702,0337726	493,1133913	450,1760276	1029,81724	-0,361511172	False	0,361511172
ENSMUSG00000026585	Kifap3	650,4649093	477,3061354	506,2923683	454,6665615	306,0977509	-0,361500492	False	0,361500492
ENSMUSG00000029561	Oasl2	1592,574035	522,4437378	2046,036187	1465,036698	628,8054574	0,36147122	False	0,36147122
ENSMUSG00000028329	Xpa	176,9526705	469,6231393	227,3373541	656,7405889	514,9086198	0,36147122	False	0,36147122
ENSMUSG00000037773	Pced1a	334,2439332	135,412807	429,4150022	232,3851314	77,11765043	0,36147122	False	0,36147122
ENSMUSG00000034930	Rtkn	862,6442688	680,9055332	1373,908357	1109,161883	671,5167715	-0,361342322	False	0,361342322
ENSMUSG00000029512	Ulk1	1391,044604	2233,831129	1082,872614	1957,872798	3798,934103	-0,361305143	False	0,361305143
ENSMUSG00000008036	Ap2s1	1037,139263	1771,890986	1332,17493	1453,810363	2001,499635	0,36117391	False	0,36117391
ENSMUSG00000033060	Lmo7	6285,096705	6288,532343	6250,129866	8072,857392	3953,169404	0,361144464	False	0,361144464
ENSMUSG00000022765	Snap29	664,3917398	397,5950504	401,9588	394,0443533	517,2814706	-0,361084623	False	0,361084623
ENSMUSG00000040265	Dnm3	349,8092144	145,9769267	272,3655257	184,1118916	162,5402786	-0,361024185	False	0,361024185
ENSMUSG00000039623	Ap5z1	408,7934379	258,3407453	524,9625858	336,7900456	328,6398334	0,360842567	False	0,360842567
ENSMUSG00000022297	Fzd6	802,84082	468,6627648	539,2398109	591,6278467	625,2461812	-0,360689631	False	0,360689631
ENSMUSG00000032497	Lrrfip2	951,1206041	2106,101318	1221,251873	1863,571586	3114,366652	0,36066058	False	0,36066058
ENSMUSG00000019302	Atp6v0a1	3790,555586	6485,40912	4923,446176	6509,028947	4866,716955	0,36053955	False	0,36053955
ENSMUSG00000037143	Cfap61	292,4634416	217,0446411	198,7829038	224,526697	227,7936751	-0,360528607	False	0,360528607
ENSMUSG00000041645	Ddx24	1394,321506	1306,109344	1086,167358	1101,303449	2588,780204	-0,360316846	False	0,360316846
ENSMUSG00000022913	Psmg1	154,0143614	208,4012704	197,6846557	205,4419278	456,7737756	0,360136009	False	0,360136009
ENSMUSG00000061436	Hipk2	756,9642017	797,1108499	589,7592229	905,9652226	1379,81273	-0,360098999	False	0,360098999
ENSMUSG00000078348	Sf3b5	465,3199855	848,0106993	717,1560011	597,2410142	1218,458877	0,360090091	False	0,360090091
ENSMUSG00000045932	Ifit2	348,1707638	200,7182742	271,2672776	217,7908961	255,0814591	-0,360080035	False	0,360080035
ENSMUSG00000062234	Gak	1871,929871	2778,36348	1458,47346	2186,890029	3526,056263	-0,360067254	False	0,360067254
ENSMUSG00000024833	Pola2	312,1248494	418,7232898	342,6534033	484,9776656	243,2172052	-0,359877931	False	0,359877931
ENSMUSG00000032946	Rasgrp2	1607,320091	962,295267	2062,509908	1692,931296	799,6507137	0,359743786	False	0,359743786
ENSMUSG00000029714	Gigyf1	1358,275591	2233,831129	1742,919715	2424,888328	3514,192009	0,359729886	False	0,359729886
ENSMUSG00000035901	Dennd5a	4023,215579	2559,39809	3135,49829	3079,38365	2425,0535	-0,359654312	False	0,359654312
ENSMUSG00000034032	Rpap1	448,1162536	275,6274866	349,2428918	212,1777287	250,3357576	-0,359642303	False	0,359642303
ENSMUSG00000006360	Crip1	2080,832329	941,1670276	2669,841101	2109,428319	2114,210047	0,359593161	False	0,359593161
ENSMUSG00000050379	Sept6	493,1736466	101,7996989	384,3868306	154,923421	107,9647106	-0,3595368	False	0,3595368
ENSMUSG00000038587	Akap12	2062,809372	6729,344247	1607,8352	1781,619341	12411,19602	-0,359490965	False	0,359490965
ENSMUSG00000038267	Slc22a23	1438,559673	727,9638846	1121,311297	861,0598832	977,6145224	-0,359438214	False	0,359438214
ENSMUSG00000061079	Zfp143	310,4863988	465,7816412	361,3236208	509,6756023	242,0307798	-0,35933954	False	0,35933954
ENSMUSG00000036078	Sigmar1	646,3687826	394,7139269	829,177306	452,4212946	122,2018153	0,359323109	False	0,359323109
ENSMUSG00000015656	Hspa8	20493,74077	14843,54855	26283,27323	14265,3037	16344,19619	0,358961608	False	0,358961608
ENSMUSG00000041264	Uspl1	546,4232928	1090,985452	700,6822798	828,5035121	1074,901404	0,358741427	False	0,358741427
ENSMUSG00000033917	Gde1	1882,5798	3769,469983	2413,949296	3214,099668	4413,502455	0,358684353	False	0,358684353
ENSMUSG00000036181	Hist1h1c	713,5452594	951,7311473	1293,736247	914,9462905	2317,088789	0,358682115	False	0,358682115
ENSMUSG00000029390	Tmed2	1184,599822	2179,089781	1518,877105	1546,988943	3147,586563	0,358605369	False	0,358605369
ENSMUSG00000033377	Palmd	1061,716023	496,5136258	828,0790579	593,8731137	406,9439092	-0,358557525	False	0,358557525
ENSMUSG00000070047	Fat1	1292,737565	1030,481858	1365,122373	941,8894941	1657,436272	0,358523972	False	0,358523972
ENSMUSG00000024120	Lrpprc	360,4591437	233,3710079	281,1515104	209,9324617	277,6235416	-0,358487949	False	0,358487949
ENSMUSG00000002997	Prkar2b	473,5122387	242,974753	364,618365	369,3464166	123,3882407	-0,358427134	False	0,358427134
ENSMUSG00000023827	Agpat4	688,9684996	777,9033595	678,717318	770,1265709	537,4507022	-0,358305594	False	0,358305594
ENSMUSG00000022013	Dnajc15	325,2324546	359,1800697	337,1621628	253,7151677	370,1647221	-0,35826146	False	0,35826146
ENSMUSG00000028532	Cachd1	384,2166781	311,1613438	345,9481475	299,7431406	662,0253683	-0,358193406	False	0,358193406
ENSMUSG00000051390	Zbtb22	819,2253265	1335,880954	639,1803869	955,3610959	1500,82812	-0,358037177	False	0,358037177
ENSMUSG00000060216	Arrb2	1042,873841	1998,539372	1336,567922	1728,855567	1727,43537	0,357968513	False	0,357968513
ENSMUSG00000032496	Ltf	209,7216836	5043,886968	163,638965	1302,254843	1829,467953	-0,357959715	False	0,357959715
ENSMUSG00000027522	Stx16	1228,83799	733,7261317	1574,887757	1004,756969	958,6317161	0,357954288	False	0,357954288
ENSMUSG00000019590	Cyb561	2107,04754	2204,059519	1644,077387	2172,295794	2646,915048	-0,357944657	False	0,357944657
ENSMUSG00000037475	Thoc2	1395,959956	1961,084766	1789,046134	2588,792817	2105,905069	0,357933033	False	0,357933033
ENSMUSG00000031065	Cdk16	2973,787935	4076,789829	3810,920863	4072,914284	3925,88162	0,357837879	False	0,357837879
ENSMUSG00000058624	Gda	1097,761938	2628,545056	856,6335082	2719,018301	2257,76752	-0,357815207	False	0,357815207
ENSMUSG00000039765	Cc2d2a	558,7116727	346,695201	436,0044907	423,2328239	234,9122275	-0,357760967	False	0,357760967
ENSMUSG00000024854	Pold4	294,9211176	269,8652395	371,2078535	407,5159551	230,1665259	-0,357651097	False	0,357651097
ENSMUSG00000045795	Whamm	407,1549873	1004,551746	521,6678415	922,8047249	1173,374712	0,357553426	False	0,357553426
ENSMUSG00000026434	Nucks1	904,4247605	1426,156159	1158,651732	1258,472137	1926,754835	0,357374591	False	0,357374591
ENSMUSG00000060038	Dhps	258,0559779	380,3083091	330,5726743	339,0353125	435,4181186	0,357283423	False	0,357283423
ENSMUSG00000042351	Grap2	407,1549873	317,8839654	292,1339913	260,4509686	180,3366595	-0,357077828	False	0,357077828
ENSMUSG00000038024	Dennd4c	412,0703392	321,7254635	294,3304874	317,7052763	259,8271607	-0,357060497	False	0,357060497
ENSMUSG00000018548	Trim37	571,0000526	348,61595	445,8887235	319,9505433	307,2841763	-0,356807164	False	0,356807164
ENSMUSG00000040711	Sh3pxd2b	496,4505479	1119,796688	387,6815749	526,5151046	1498,455269	-0,356777846	False	0,356777846
ENSMUSG00000039176	Polg	984,7088425	1260,971742	1409,052296	1468,404599	1268,288743	0,356766827	False	0,356766827
ENSMUSG00000033557	Fam20b	1234,572567	754,8543711	964,2618208	850,9561818	684,5674508	-0,356514807	False	0,356514807
ENSMUSG00000030724	Cd19	1075,642854	202,6390233	840,1597869	145,9423531	158,9810024	-0,356463499	False	0,356463499
ENSMUSG00000057841	Rpl32	3329,331727	4747,131242	2600,651471	3145,619026	7224,144207	-0,356359557	False	0,356359557
ENSMUSG00000073102	Drc1	425,9971698	276,5878612	332,7691705	263,818869	252,7086083	-0,356322065	False	0,356322065
ENSMUSG00000032059	Alg9	465,3199855	223,7672627	363,5201169	193,0929595	205,2515927	-0,356187948	False	0,356187948
ENSMUSG00000043284	Tmem11	453,0316056	954,6122708	579,8749902	645,514254	1774,892385	0,356130214	False	0,356130214
ENSMUSG00000047909	Ankrd16	453,0316056	983,4235064	579,8749902	1120,388218	711,8552348	0,356130214	False	0,356130214
ENSMUSG00000001134	Uxt	163,02584	467,7023902	208,6671366	349,1390139	492,3665374	0,356102752	False	0,356102752
ENSMUSG00000009030	Pdcl	553,7963207	343,8140774	432,7097464	326,6863442	317,9620049	-0,355955852	False	0,355955852
ENSMUSG00000031617	Tmem184c	455,4892816	585,8284559	490,9168951	505,1850684	355,9276174	-0,355833226	False	0,355833226
ENSMUSG00000055116	Amtl	534,9541382	275,6274866	204,2741443	125,7349503	684,5674508	0,355777484	False	0,355777484
ENSMUSG00000029185	Fam114a1	691,4261756	219,9257646	540,338059	316,5826428	396,2660807	-0,355712919	False	0,355712919
ENSMUSG00000020326	Ccng1	1543,420515	1206,230395	1163,044725	1047,417042	989,4787763	-0,355625694	False	0,355625694
ENSMUSG00000024232	Bambi	284,2711883	370,7045639	366,8148612	363,7332492	412,8760362	0,355612925	False	0,355612925
ENSMUSG00000049295	Zfp219	970,7820119	675,1432861	1242,118587	1072,114978	476,9430073	0,355583635	False	0,355583635
ENSMUSG00000061518	Cox5b	1023,212433	2365,402438	1309,11172	1513,309938	2942,33497	0,355482523	False	0,355482523
ENSMUSG00000070394	Tmem256	526,761885	824,0013364	886,2862065	729,7117654	673,8896222	0,355361349	False	0,355361349
ENSMUSG00000028403	Zdhhc21	393,2281567	345,7348265	264,6777891	273,9225704	503,0443659	0,355319016	False	0,355319016
ENSMUSG00000021250	Fos	1039,596939	442,7326528	159,2459727	277,2904709	812,701393	-0,355227019	False	0,355227019
ENSMUSG00000056501	Cebpb	1072,365952	3586,03845	1371,711861	1434,725594	8495,992227	0,355180144	False	0,355180144
ENSMUSG00000026466	Tor1aip1	1925,998743	2290,493225	1505,698128	2101,569884	3144,027287	-0,355174203	False	0,355174203
ENSMUSG00000024048	Myl12a	2272,531056	2368,283561	1563,905276	1848,97735	2906,742209	0,355103118	False	0,355103118

ENSMUSG00000066842	Hmcn1	1171,492217	127,7298109	915,9389049	325,5637107	144,7438977	-0,355024094	False	0,355024094
ENSMUSG00000037362	Nov	142,5452068	481,1476335	182,3091825	465,8928964	573,043464	0,3549677	False	0,3549677
ENSMUSG00000026289	Atg16l1	462,8623095	804,793846	591,9557191	600,6089146	1146,086928	0,35490617	False	0,35490617
ENSMUSG00000020911	Krt19	2741,127943	5302,227714	3505,607895	4226,715072	4497,738658	0,354894971	False	0,354894971
ENSMUSG00000028936	Rpl22	2242,219719	2948,34977	2867,525756	3061,421514	3546,225494	0,354878788	False	0,354878788
ENSMUSG00000034652	Cd300a	338,3400599	263,1426179	195,4881596	182,9892581	264,5728623	-0,354808915	False	0,354808915
ENSMUSG00000025484	Bet1l	306,3902721	570,4624637	416,2360251	391,7990864	1027,444389	0,354743541	False	0,354743541
ENSMUSG00000035151	Elmod2	255,5983019	152,6995484	199,8811519	171,7629232	132,8796438	-0,354735814	False	0,354735814
ENSMUSG00000035198	Tubg1	501,3658998	381,2686836	392,0745672	288,5168057	270,5049892	-0,354735814	False	0,354735814
ENSMUSG00000036281	Snapc4	530,8580116	806,7145951	678,717318	744,3060007	1448,625403	0,35448479	False	0,35448479
ENSMUSG00000026219	Trip12	2250,411972	2175,248283	1707,775776	2106,060418	2877,081574	0,354416994	False	0,354416994
ENSMUSG00000034620	Tmem5	299,0172442	472,5042628	233,9268426	324,4410772	629,9918828	-0,354171269	False	0,354171269
ENSMUSG00000000420	Galnt1	1367,28707	1682,576155	1069,693637	1439,216128	1411,846216	-0,354118513	False	0,354118513
ENSMUSG00000035851	Ythdc1	1267,34158	1762,28724	1619,915929	1778,251441	2841,488812	0,354113522	False	0,354113522
ENSMUSG00000019832	Rab32	249,8637246	192,0749036	195,4881596	152,678154	182,7095103	-0,354060237	False	0,354060237
ENSMUSG00000020903	Stx8	353,9053411	452,3363979	481,0326623	474,8739643	538,6371276	0,354032555	False	0,354032555
ENSMUSG00000001313	Rnd2	280,994287	426,406286	359,1271246	363,7332492	899,3104466	0,353953824	False	0,353953824
ENSMUSG00000026641	Usf1	709,4491328	906,5935449	908,2511683	946,3800281	1250,492362	0,353756642	False	0,353756642
ENSMUSG00000087687	Pet100	267,0674565	366,8630659	423,9237617	341,2805795	341,6905127	0,353754134	False	0,353754134
ENSMUSG00000036098	Myrf	1556,52812	547,4134752	862,1247486	1218,057331	545,75568	-0,353749601	False	0,353749601
ENSMUSG00000004947	Dtx2	420,2625925	1187,983279	537,0433147	593,8731137	1572,013643	0,353747404	False	0,353747404
ENSMUSG00000052384	Nrros	987,9857438	496,5136258	773,1666535	387,3085524	517,2814706	-0,353710809	False	0,353710809
ENSMUSG00000046364	Rpl27a	4016,661776	7930,772769	5132,113313	6523,623183	10665,96427	0,35355604	False	0,35355604
ENSMUSG00000055065	Ddx17	7508,200118	3937,535524	9593,197044	5980,268576	1950,483343	0,353544587	False	0,353544587
ENSMUSG00000040690	Col16a1	366,193721	166,1447916	467,8536852	130,2254843	277,6235416	0,353450364	False	0,353450364
ENSMUSG00000061244	Exoc5	748,7719485	1286,901854	956,5740841	1127,124019	1664,554824	0,353350318	False	0,353350318
ENSMUSG00000020271	Fbxw11	1076,462079	1026,64036	959,8688284	881,2672859	1375,067028	0,353204447	False	0,353204447
ENSMUSG00000027864	Ptgfrn	5840,257353	3634,057176	4572,006788	3873,085524	3173,687922	-0,353204398	False	0,353204398
ENSMUSG00000030409	Dmpk	951,9398294	745,2506259	2048,232683	857,6919827	931,3439321	-0,353144704	False	0,353144704
ENSMUSG00000036144	Meox2	312,1248494	84,51295758	398,6640557	127,9802173	56,94841878	0,353050315	False	0,353050315
ENSMUSG00000020736	Nt5c	363,736045	593,5114521	464,558941	617,4484169	478,1294327	0,352969751	False	0,352969751
ENSMUSG00000027509	Rae1	596,3960377	669,381039	555,7135322	467,0155299	959,8181415	-0,352800148	False	0,352800148
ENSMUSG00000071657	Bscl2	1282,087636	1069,857213	1191,599175	1000,266435	1637,26704	0,352794767	False	0,352794767
ENSMUSG00000022890	Atp5j	1207,538131	1946,679148	1541,940315	1805,194644	2113,023622	0,352678176	False	0,352678176
ENSMUSG00000034932	Mrpl54	324,4132293	702,9941471	429,4150022	414,2517561	813,8878184	0,352675161	False	0,352675161
ENSMUSG00000019699	Akt3	1586,020232	740,4487533	1242,118587	1138,350354	481,6887088	-0,352608259	False	0,352608259
ENSMUSG00000063457	Rps15	7114,971961	12557,8572	9084,708179	11174,69371	20043,47056	0,352582101	False	0,352582101
ENSMUSG00000061374	Fiz1	718,4606114	1001,670622	968,6548131	917,1915574	1410,65979	0,35231401	False	0,35231401
ENSMUSG00000030105	Arl8b	1497,543897	1457,848518	1337,66617	1173,151992	2190,141272	-0,352208354	False	0,352208354
ENSMUSG00000003644	Rps6ka1	1859,614991	1911,145291	1883,49547	2699,933532	1456,93038	-0,35209258	False	0,35209258
ENSMUSG00000054200	Ffar4	232,6599927	673,2225371	182,3091825	351,3842809	804,3964153	-0,351835924	False	0,351835924
ENSMUSG00000090115	Usp49	238,39457	357,2593207	360,2253727	304,2336745	580,1620163	0,351828473	False	0,351828473
ENSMUSG00000026854	Usp20	408,7934379	473,4646373	393,1728153	623,0615843	320,3348556	-0,351791252	False	0,351791252
ENSMUSG00000039741	Bahcc1	498,9082239	210,3220194	390,9763191	378,3274845	107,9647106	-0,351693222	False	0,351693222
ENSMUSG00000057375	Yipf1	512,8350544	335,1707068	401,9588	360,3653488	270,5049892	-0,351447244	False	0,351447244
ENSMUSG00000028699	Tspan1	1289,460664	850,8918229	644,6716273	446,8081271	1010,834433	-0,351221049	False	0,351221049
ENSMUSG00000038668	Lpar1	185,9641491	329,4084597	237,2215869	414,2517561	363,0461697	0,351210779	False	0,351210779
ENSMUSG00000030208	Emp1	3235,94004	4722,161505	2536,953082	2661,763994	7551,597615	-0,351088037	False	0,351088037
ENSMUSG00000023951	Vegfa	14759,98271	6446,994139	18822,87397	9027,095855	3822,662611	0,350795892	False	0,350795892
ENSMUSG00000021039	Snw1	1166,576865	1454,00702	914,8406568	1194,482028	1999,126784	-0,350688981	False	0,350688981
ENSMUSG00000045594	Glb1	404,6973113	179,5900349	317,3936973	268,309403	180,3366595	-0,350569784	False	0,350569784
ENSMUSG00000026918	Brd3	1320,591226	1498,184248	1519,975353	2007,268672	1035,749367	-0,350509028	False	0,350509028
ENSMUSG00000028337	Coro2a	515,2927304	850,8918229	404,1552962	871,1635845	947,9538876	-0,350482486	False	0,350482486
ENSMUSG00000040550	Otud6b	559,530898	438,8911547	389,878071	346,8937469	326,2669826	-0,350354606	False	0,350354606
ENSMUSG00000022757	Tfg	1218,188061	1135,16268	948,8863475	864,4277836	1553,030837	0,350349607	False	0,350349607
ENSMUSG00000027531	Impa1	566,0847006	395,6743014	721,5489934	490,5908331	169,6588309	0,350079425	False	0,350079425
ENSMUSG00000025169	Ogfod3	225,2869648	271,7859886	297,6252317	280,6583713	176,7773833	-0,349830131	False	0,349830131
ENSMUSG00000030603	Psmc4	1044,512291	2651,594044	1331,076682	1703,034997	3380,12594	0,349764216	False	0,349764216
ENSMUSG00000005682	Pan2	911,7977884	2422,064534	1161,946476	2165,559993	2286,241729	0,349757799	False	0,349757799
ENSMUSG00000040483	Xaf1	546,4232928	249,6973747	696,2892874	392,9217198	580,1620163	0,349667844	False	0,349667844
ENSMUSG00000021548	Ccnh	248,2252739	239,133255	316,2954492	231,262498	134,0660692	0,349622783	False	0,349622783
ENSMUSG00000019843	Fyn	1189,515174	976,7008847	1069,693637	910,4557565	1515,065225	0,349006228	False	0,349006228
ENSMUSG00000060152	Pop5	220,3716128	292,914228	289,9374951	280,6583713	304,9113256	0,348876697	False	0,348876697
ENSMUSG00000039206	Daglb	344,0746371	133,492058	270,1690295	187,479792	126,9475169	-0,348859248	False	0,348859248
ENSMUSG00000031508	Ankrd10	572,6385032	475,3853864	752,2999399	716,2401636	449,6552233	-0,348805451	False	0,348805451
ENSMUSG00000022696	Sidt1	195,794853	313,0820928	153,7547322	275,0452039	236,0986529	-0,348712026	False	0,348712026
ENSMUSG00000040407	Akap9	1050,246869	1300,347097	824,7843137	1301,132209	1110,494166	-0,348639684	False	0,348639684
ENSMUSG00000022562	Oplah	734,8451179	1216,794514	935,7073705	1427,989793	1392,863409	0,34861721	False	0,34861721
ENSMUSG00000014361	Mertk	1007,647152	879,7030584	1018,075977	1902,863757	791,345736	-0,348610492	False	0,348610492
ENSMUSG00000038671	Arfrp1	603,7690657	504,1966219	768,7736612	580,4015119	415,2488869	0,348562067	False	0,348562067
ENSMUSG00000005150	Wdr83	212,9985849	218,0050156	237,2215869	237,9982989	167,2859802	-0,348527304	False	0,348527304
ENSMUSG00000021286	Zfyve21	530,0387863	571,4228382	528,25733	777,9850053	416,4353123	-0,348005525	False	0,348005525
ENSMUSG00000005225	Plekha8	194,1564024	354,3781971	247,1058197	250,3472672	435,4181186	0,347909707	False	0,347909707
ENSMUSG00000020863	Luc7l3	2291,373238	3199,007519	3474,856949	4162,724963	2916,233612	0,347893819	False	0,347893819
ENSMUSG00000001285	Myg1	420,2625925	560,8587185	534,8468186	575,9109779	569,4841878	0,347834711	False	0,347834711
ENSMUSG00000026437	Cdk18	627,5266001	1253,288746	798,4263596	1176,519893	1218,458877	0,347482737	False	0,347482737
ENSMUSG00000068617	Efcab1	299,0172442	178,6296603	235,0250907	222,2814301	72,37194887	-0,347413903	False	0,347413903
ENSMUSG00000034574	Daam1	1114,965669	493,6325022	876,4019738	575,9109779	429,4859916	-0,347334651	False	0,347334651
ENSMUSG00000033715	Akr1c14	633,2611774	2627,584681	1275,066029	6395,642965	805,5828407	0,347232317	False	0,347232317
ENSMUSG00000027288	Zfp106	2191,427748	1719,070387	1418,936529	1642,412789	1722,689668	-0,347208282	False	0,347208282
ENSMUSG00000001576	Ergic1	1436,101997	782,7052321	1128,999034	688,1743265	951,5131638	-0,347113967	False	0,347113967
ENSMUSG00000009535	Rnmt	208,0832329	501,3154984	264,6777891	337,9126791	499,4850897	0,347076414	False	0,347076414
ENSMUSG00000024958	Gpr137	992,0818704	882,584182	1261,887052	1065,379177	694,0588539	0,347051698	False	0,347051698
ENSMUSG00000040752	Myh6	934,7360976	1188,943653	640,278635	96,54647973	69,99909808	0,34704933	False	0,34704933
ENSMUSG00000040848	Sft2d2	1980,88684	1108,272194	1427,722514	1296,641675	1557,776539	-0,346658172	False	0,346658172
ENSMUSG00000027030	Stk39	208,0832329	304,4387222	163,638965	195,3382264	313,2163033	-0,346644402	False	0,346644402
ENSMUSG00000026036	Nif3l1	308,8479481	290,0331044	273,4637738	174,0081902	392,7068045	0,34655585	False	0,34655585
ENSMUSG00000075227	Znhit2	253,9598512	435,0496566	322,8849377	390,6764529	494,7393882	0,346419704	False	0,346419704
ENSMUSG00000021998	Lcp1	6283,458255	3411,250288	4943,214641	3287,070845	2671,829981	-0,346109248	False	0,346109248
ENSMUSG00000000275	Trim25	1477,063264	1162,053167	1427,722514	1289,905875	2121,3286	-0,346055542	False	0,346055542
ENSMUSG00000021840	Mapk1ip1l	767,614131	773,1014869	671,0295814	603,9768151	1229,136705	-0,345888101	False	0,345888101
ENSMUSG00000024188	Luc7l	945,3860268	1489,540877	1201,483408	1454,932997	1828,281528	0,345841279	False	0,345841279
ENSMUSG00000070942	Il1rl2	188,4218251	278,5086102	148,2634918	287,3941722	219,4886974	-0,345802689	False	0,345802689
ENSMUSG00000049532	Sall2	315,4017507	98,91857535	248,2040678	167,2723893	98,47330747	-0,345663909	False	0,345663909
ENSMUSG00000051154	Commd3	832,3329318	1145,7268	1057,612908	1191,114128	1233,882407	0,345579066	False	0,345579066
ENSMUSG00000026301	Iqca	561,988574	261,2218689	407,4500404	442,3175932	236,0986529	-0,345458172	False	0,345458172
ENSMUSG00000044627	Swi5	1458,221081	1876,571808	1852,744523	1981,448101	2117,769323	0,345454497	False	0,345454497
ENSMUSG00000020260	Pofut2	1399,236858	1628,795182	1340,960915	1101,303449	2627,932242	-0,345428159	False	0,345428159
ENSMUSG00000039001	Rps21	2114,420568	4450,375516	2686,314822	4033,622112	4844,174872	0,345366027	False	0,345366027
ENSMUSG00000039047	Pigk	573,4577286	451,3760234	438,2009869	371,5916836	395,0796553	-0,345357358	False	0,345357358
ENSMUSG00000033960	Jcad	4887,498298	2030,231731	3847,16305	3080,506284	1509,133098	-0,345301225	False	0,345301225
ENSMUSG00000016510	Mtif3	230,2023168	236,2521314	181,2109344	258,2057016	252,7086083	-0,345232341	False	0,345232341
ENSMUSG00000039630	Hnrnpu	4584,384927	4080,631327	5822,911359	5406,602865	2967,249904	0,345012472	False	0,345012472
ENSMUSG00000010080	Epn3	1590,116359	3548,583844	2019,678233	2918,847062	4971,122389	0,344993126	False	0,344993126
ENSMUSG00000039450	Dcxr	1845,714661	3490,961373	2343,661419	2620,226555	5270,101588	0,344584627	False	0,344584627
ENSMUSG00000031858	Mau2	1070,727502	943,0877766	1056,51466	1359,509151	818,63352	0,344494498	False	0,344494498
ENSMUSG00000022221	Ripk3	196,6140784	249,6973747	154,8529803	174,0081902	638,2968605	-0,344467479	False	0,344467479
ENSMUSG00000036894	Rap2b	246,5868233	260,2614944	342,6534033	194,2155929	283,5556685	-0,344436675	False	0,344436675
ENSMUSG00000079555	Haus3	123,7030243	358,2196952	157,0494765	380,5727515	546,9421054	0,344338362	False	0,344338362
ENSMUSG00000073700	Klhl21	500,5466745	741,4091278	394,2710634	590,5052132	1225,577429	-0,344316771	False	0,344316771

ENSMUSG00000046546	Fam43a	793,8293414	876,8219349	769,8719093	625,3068513	1040,495068	-0,344264564	False	0,344264564
ENSMUSG00000045757	Zfp764	195,794853	219,9257646	240,5163311	264,9415025	154,2353009	-0,344209838	False	0,344209838
ENSMUSG00000006058	Snf8	524,304209	821,1202128	665,538341	678,0706251	882,7004911	0,344117653	False	0,344117653
ENSMUSG00000041362	Shtn1	570,1808273	308,2802203	449,1834678	408,6385886	275,2506908	-0,344114698	False	0,344114698
ENSMUSG00000019873	Reep3	1650,739033	1691,219526	1365,122373	1359,509151	2095,227241	0,343994661	False	0,343994661
ENSMUSG00000004268	Emg1	421,9010432	589,669954	610,6259366	535,4961725	978,8009478	0,343971608	False	0,343971608
ENSMUSG00000037443	Cep85	280,1750617	340,9329539	220,7478656	505,1850684	435,4181186	-0,343929059	False	0,343929059
ENSMUSG00000034842	Art3	280,1750617	363,0215678	220,7478656	316,5826428	355,9276174	-0,343929059	False	0,343929059
ENSMUSG00000031714	Gab1	1146,096232	933,4840314	635,8856426	760,0228695	1454,55753	0,343852173	False	0,343852173
ENSMUSG00000035944	Ttc38	743,0373712	645,3716761	617,2154251	943,0121276	554,0606577	0,343841552	False	0,343841552
ENSMUSG00000028367	Txn1	1827,691704	3408,369164	2319,499961	2426,010962	4290,114215	0,343791086	False	0,343791086
ENSMUSG00000051331	Cacna1c	566,0847006	487,8702551	718,2542492	373,8369506	494,7393882	0,34347669	False	0,34347669
ENSMUSG00000018770	Atp5g3	1281,268411	2429,74753	1794,537375	1625,573287	2792,845371	0,343375864	False	0,343375864
ENSMUSG00000051510	Mafg	1619,608471	2351,957194	2054,822172	2210,465332	3832,154014	0,343368452	False	0,343368452
ENSMUSG00000034729	Mrps10	253,9598512	401,4365485	347,0463956	322,1958103	370,1647221	0,343337296	False	0,343337296
ENSMUSG00000001930	Vwf	10034,69102	3847,260319	7909,582726	6135,191997	5410,099784	-0,343322704	False	0,343322704
ENSMUSG00000003575	Crtc1	544,7848421	311,1613438	429,4150022	354,7521813	245,590056	-0,343313968	False	0,343313968
ENSMUSG00000052861	Dnah6	1461,497983	723,162012	1152,062244	1041,803874	850,6670055	-0,343229173	False	0,343229173
ENSMUSG00000030137	Tuba8	274,4404844	374,546062	216,3548732	326,6863442	568,2977624	-0,343093701	False	0,343093701
ENSMUSG00000062778	Chia1	1369,744746	2550,75472	1737,428474	2278,945975	2676,575683	0,343046517	False	0,343046517
ENSMUSG00000069565	Dazap1	1412,344463	2423,024909	1791,242631	1958,995432	3867,746775	0,342868772	False	0,342868772
ENSMUSG00000036885	Arhgef26	1531,951361	696,2715255	906,0546721	1207,95363	583,7212925	-0,342805418	False	0,342805418
ENSMUSG00000036995	Asap3	303,1133708	167,1051661	384,3868306	222,2814301	126,9475169	0,342701416	False	0,342701416
ENSMUSG00000029472	Anapc5	2818,135123	2215,584013	3573,699276	3052,440446	1881,670671	0,342677451	False	0,342677451
ENSMUSG00000042632	Pla2g6	478,4275907	423,5251624	573,2855016	571,420444	377,2832744	-0,342652447	False	0,342652447
ENSMUSG00000022247	Brix1	348,1707638	609,8378189	441,4957311	460,279729	1063,037151	0,342604425	False	0,342604425
ENSMUSG00000058690	Ccser2	2205,354579	1449,205148	1700,088039	1410,027657	1739,299624	-0,34250415	False	0,34250415
ENSMUSG00000064138	Fam172a	421,9010432	324,6065871	332,7691705	277,2904709	221,8615482	-0,342382874	False	0,342382874
ENSMUSG00000058388	Phtf1	593,1191364	434,0892821	467,8536852	431,0912583	421,1810139	-0,342264502	False	0,342264502
ENSMUSG00000035969	Rusc2	3125,344621	2059,042966	2047,134435	2465,303134	2206,751228	-0,342250228	False	0,342250228
ENSMUSG00000057068	Fam47e	282,6327377	331,3292087	222,9443617	288,5168057	360,6733189	-0,34224487	False	0,34224487
ENSMUSG00000040370	Etfrf1	271,1635831	163,263668	343,7516514	249,2246337	93,72760591	0,342203205	False	0,342203205
ENSMUSG00000020463	Ppp4r3b	1019,116306	1139,964553	803,9176	1042,926508	1156,764756	-0,342199167	False	0,342199167
ENSMUSG00000025878	Uimc1	512,0158291	1230,239757	649,0646197	1161,925657	1334,728565	0,342173705	False	0,342173705
ENSMUSG00000106847	Peg13	3316,224122	1796,860723	2616,026944	2066,768246	2372,850783	-0,342164112	False	0,342164112
ENSMUSG00000032316	Clk3	1592,574035	2018,707237	2140,485522	2460,8126	2847,420939	0,342071257	False	0,342071257
ENSMUSG00000041361	Myzap	2216,004508	1036,244105	1657,256364	2808,82898	522,0271722	0,34200797	False	0,34200797
ENSMUSG00000006442	Srm	405,5165366	557,9775949	513,9801049	319,9505433	887,4461927	-0,341911831	False	0,341911831
ENSMUSG00000052539	Magi3	2044,786415	837,4465796	1613,32644	940,7668606	523,2135975	-0,341911774	False	0,341911774
ENSMUSG00000030691	Fchsd2	943,7475762	486,9098806	744,6122033	646,6368875	715,4145109	-0,341911774	False	0,341911774
ENSMUSG00000095304	Plac9	2207,812255	1742,119376	3624,218688	2182,399495	2076,244435	-0,34177401	False	0,34177401
ENSMUSG00000022564	Grina	4388,590074	5965,846505	5561,528315	7004,110314	8715,480924	0,341723873	False	0,341723873
ENSMUSG00000038013	Wipf2	956,8551814	1663,368665	1212,465889	1683,950228	1810,485147	0,341571663	False	0,341571663
ENSMUSG00000030530	Furin	2091,482259	3371,874932	1650,666875	2412,53936	5523,996622	-0,341476764	False	0,341476764
ENSMUSG00000028059	Arhgef2	3621,795169	2196,376523	2858,739772	2644,924491	2002,68606	-0,341325658	False	0,341325658
ENSMUSG00000016528	Mapkapk2	2578,102103	3737,777624	2035,053706	2161,069459	6354,494396	-0,341242533	False	0,341242533
ENSMUSG00000073802	Cdkn2b	457,1277322	579,1058343	296,5269836	324,4410772	543,3828292	0,341229687	False	0,341229687
ENSMUSG00000049488	Tmem67	328,5093559	190,1541546	259,1865486	259,3283351	189,8280626	-0,341154611	False	0,341154611
ENSMUSG00000028211	Trp53inp1	1105,954191	4140,174547	873,1072295	4523,090312	4999,596599	-0,341060877	False	0,341060877
ENSMUSG00000026640	Plxna2	723,3759633	2452,796519	571,0890055	1964,608599	3450,125038	-0,34103005	False	0,34103005
ENSMUSG00000026721	Rabgap1l	3021,303004	1766,128738	2385,394846	2135,248889	1888,789223	-0,340942788	False	0,340942788
ENSMUSG00000035095	Fam167a	591,4806858	749,092124	403,0570481	331,1768781	606,2633749	0,340812094	False	0,340812094
ENSMUSG00000025504	Eps8l2	2867,288643	3855,90369	3630,808177	4186,300266	4689,939572	0,340603567	False	0,340603567
ENSMUSG00000024387	Csnk2b	2407,703235	3471,753882	3048,736691	3220,835469	4670,956765	0,340553974	False	0,340553974
ENSMUSG00000033379	Atp6v0b	1635,173752	2163,723789	2598,454975	2195,871097	2070,312308	0,340404472	False	0,340404472
ENSMUSG00000075054	Yae1d1	454,6700562	193,9956526	359,1271246	335,6674121	88,98190434	-0,340325371	False	0,340325371
ENSMUSG00000036241	Ube2r2	2693,612874	3565,870585	3410,060311	3858,491289	5272,474439	0,340254734	False	0,340254734
ENSMUSG00000056174	Col8a2	387,4935795	246,8162511	254,7935563	288,5168057	306,0977509	-0,340179961	False	0,340179961
ENSMUSG00000068551	Zfp467	748,7719485	544,5323517	947,7880994	819,5224442	402,1982076	0,340038159	False	0,340038159
ENSMUSG00000025184	R3hcc1l	337,5208345	442,7326528	427,218506	623,0615843	615,7547781	0,339997582	False	0,339997582
ENSMUSG00000038065	Mturn	879,8480007	201,6786488	695,1910393	413,1291226	118,6425391	-0,339844824	False	0,339844824
ENSMUSG00000028517	Plpp3	4796,564287	2037,914727	3790,05415	2483,265269	2281,496027	-0,339782932	False	0,339782932
ENSMUSG00000004451	Ralb	1079,73898	1443,4429	1083,970862	853,2014488	1808,112296	-0,339724272	False	0,339724272
ENSMUSG00000091243	Vgll3	498,9082239	177,6692858	394,2710634	194,2155929	287,1149447	-0,339586619	False	0,339586619
ENSMUSG00000009772	Nuak2	261,3328792	824,0013364	330,5726743	544,4772403	643,0425621	0,339078828	False	0,339078828
ENSMUSG00000034321	Exosc1	308,8479481	497,4740003	432,7097464	390,6764529	536,2642768	0,339077557	False	0,339077557
ENSMUSG00000002103	Acp2	1241,945595	765,4184908	981,8337902	922,8047249	654,906816	-0,339051252	False	0,339051252
ENSMUSG00000021668	Polk	312,1248494	352,4574481	329,4744262	372,7143171	246,7764814	-0,338918313	False	0,338918313
ENSMUSG00000024844	Banf1	1274,714608	1204,309645	1612,228192	911,57839	1307,440781	0,338881672	False	0,338881672
ENSMUSG00000066829	Zfp810	303,1133708	357,2593207	304,2147202	391,7990864	239,657929	-0,338880821	False	0,338880821
ENSMUSG00000031483	Erlin2	1058,439122	800,952348	836,8650426	666,8442902	501,8579405	-0,338871403	False	0,338871403
ENSMUSG00000058594	Fbxo18	643,0918813	283,3104828	508,4888645	418,74229	135,2524946	-0,338808696	False	0,338808696
ENSMUSG00000028218	Fam92a	241,6714713	359,1800697	191,0951672	225,6493305	616,9412035	-0,338755884	False	0,338755884
ENSMUSG00000007950	Abhd8	355,5437917	363,9819423	543,6328033	425,4780909	281,1828177	-0,338518827	False	0,338518827
ENSMUSG00000024959	Bad	471,0545628	678,0244097	563,4012688	646,6368875	372,5375729	-0,33850824	False	0,33850824
ENSMUSG00000052446	Zfp961	244,9483726	208,4012704	309,7059607	244,7340998	74,74479965	0,338421443	False	0,338421443
ENSMUSG00000030471	Zdhhc13	277,7173857	354,3781971	219,6496175	272,7999369	514,9086198	-0,338413509	False	0,338413509
ENSMUSG00000026135	Zfp142	443,2009017	350,536699	583,1697344	412,0064891	503,0443659	-0,338395323	False	0,338395323
ENSMUSG00000048578	Mlec	3716,825306	2653,514793	2940,01013	2667,377161	2205,564802	-0,338249756	False	0,338249756
ENSMUSG00000028795	Ccdc28b	301,4749202	275,6274866	375,6008459	522,0245706	238,4715036	-0,338221108	False	0,338221108
ENSMUSG00000046873	Mbtps2	302,2941455	222,8068882	200,9794	239,1209324	198,1330403	-0,338212611	False	0,338212611
ENSMUSG00000020803	Txndc17	632,4419521	836,4862051	799,5246076	875,6541185	1002,529456	0,338209368	False	0,338209368
ENSMUSG00000024050	Wiz	1288,641439	1160,132418	1118,016553	1019,351205	1873,365693	-0,338199694	False	0,338199694
ENSMUSG00000009293	Ube2g2	558,7116727	856,65407	706,1735202	762,2681365	1236,255258	0,337918763	False	0,337918763
ENSMUSG00000051022	Hs3st1	695,5223022	550,2945988	848,9457716	699,4006613	1290,830826	-0,337892608	False	0,337892608
ENSMUSG00000032902	Slc16a1	367,8321716	349,5763245	291,0357432	387,3085524	638,2968605	-0,337851321	False	0,337851321
ENSMUSG00000115987	Vps28	1711,361707	2162,763414	2644,581395	2436,114663	2605,390159	0,337731142	False	0,337731142
ENSMUSG00000040850	Psme4	1061,716023	1526,035109	840,1597869	1151,821956	2313,529513	-0,337662301	False	0,337662301
ENSMUSG00000028842	Ago3	557,073222	441,7722782	703,977024	501,8171679	378,4696998	0,337661375	False	0,337661375
ENSMUSG00000052298	Cdc42se2	1871,110646	1122,677811	1310,209968	1139,472988	1480,658888	-0,33765556	False	0,33765556
ENSMUSG00000061455	Stx17	757,783427	866,2578152	531,5520743	957,6063629	473,3837311	0,33764715	False	0,33764715
ENSMUSG00000026177	Slc11a1	439,9240004	767,3392398	348,1446437	446,8081271	964,5638431	-0,337567483	False	0,337567483
ENSMUSG00000038893	Fam117a	898,6901832	590,6303285	1135,588522	824,0129782	199,3194657	0,337544425	False	0,337544425
ENSMUSG00000069539	Scyl2	611,9613189	845,1295758	484,3274066	689,2969599	1199,476071	-0,337457823	False	0,337457823
ENSMUSG00000038437	Mllt6	3000,822371	2375,006183	3177,231717	3224,20337	3371,820962	-0,337426654	False	0,337426654
ENSMUSG00000062006	Rpl34	2619,882594	4806,674462	3310,119735	4415,317497	6703,303461	0,337381243	False	0,337381243
ENSMUSG00000016933	Plcg1	2456,856754	2164,684163	2451,289731	3995,452574	1944,551216	-0,337376511	False	0,337376511
ENSMUSG00000049516	Spty2d1	525,9426596	482,108008	482,1309104	367,1011497	664,3982191	0,337142686	False	0,337142686
ENSMUSG00000039428	Tmem135	656,1994866	384,1498072	519,4713453	502,9398014	370,1647221	-0,337090294	False	0,337090294
ENSMUSG00000035891	Cerk	1040,416165	2080,171206	823,6860656	1370,735485	2035,905971	-0,336994232	False	0,336994232
ENSMUSG00000050106	Tmc8	226,1061901	415,8421663	179,0144382	254,8378012	683,3810254	-0,336924538	False	0,336924538
ENSMUSG00000018569	Cldn7	531,6772369	631,9264328	512,8818568	420,987557	1230,323131	-0,336773108	False	0,336773108
ENSMUSG00000055805	Fmnl1	1721,192411	2544,992473	1362,925876	1555,970011	5645,012012	-0,336701282	False	0,336701282
ENSMUSG00000006494	Pdk1	406,335762	405,2780466	418,4325213	513,0435028	231,3529513	0,336408819	False	0,336408819
ENSMUSG00000003184	Irf3	1386,948478	1530,836982	1932,916634	1888,269522	1098,629912	-0,336208718	False	0,336208718
ENSMUSG00000003402	Prkcsh	1001,912574	1176,458784	823,6860656	793,7018741	1554,217263	-0,336087509	False	0,336087509
ENSMUSG00000018286	Psmb6	1048,608418	2235,751878	1333,273178	1323,584879	2732,337676	0,335974683	False	0,335974683
ENSMUSG00000032531	Amotl2	2506,010274	1322,435711	1819,797081	1577,300047	1986,076105	-0,335471422	False	0,335471422
ENSMUSG00000036323	Srp72	1653,196709	2019,667611	1310,209968	1926,439061	2992,164837	-0,335460367	False	0,335460367
ENSMUSG00000030509	Asb7	264,6097805	262,1822434	209,7653847	294,1299731	306,0977509	-0,335089762	False	0,335089762

ENSMUSG00000048878	Hexim1	874,1134234	1344,524325	1102,64108	1192,236761	1299,135803	0,335070858	False	0,335070858
ENSMUSG00000026896	Ifih1	649,6456839	440,8119037	515,078353	469,2607968	288,3013701	-0,334861179	False	0,334861179
ENSMUSG00000035623	Rsf1	519,388857	451,3760234	411,8430328	601,7315481	627,619032	-0,33472048	False	0,33472048
ENSMUSG00000002881	Nab1	1078,919755	1245,60575	855,5352601	1038,435974	1351,338521	-0,334688348	False	0,334688348
ENSMUSG00000057110	Cntrl	799,5639187	1745,960874	1008,191744	1542,498409	1917,263432	0,33448477	False	0,33448477
ENSMUSG00000068290	Ddrgk1	1182,142146	1748,841997	1607,8352	1683,950228	1490,150291	0,334054321	False	0,334054321
ENSMUSG00000041859	Mcm3	264,6097805	374,546062	255,8918044	209,9324617	384,4018268	-0,33394112	False	0,33394112
ENSMUSG00000034035	Ccdc17	1538,505163	1220,636012	2230,541866	1926,439061	1344,219968	-0,333896226	False	0,333896226
ENSMUSG00000002227	Mov10	519,388857	337,0914558	654,5558601	444,5628602	423,5538647	0,333701255	False	0,333701255
ENSMUSG00000028469	Npr2	2089,024583	1705,625144	2254,703323	2632,575523	1620,657084	0,33364545	False	0,33364545
ENSMUSG00000025757	Hspa4l	1853,087689	964,216016	1470,554189	928,4178923	900,496872	-0,333571204	False	0,333571204
ENSMUSG00000053091	Lins1	204,8063316	229,5295098	255,8918044	216,6682627	162,5402786	-0,333463045	False	0,333463045
ENSMUSG00000042707	Dnali1	1086,292783	408,1591701	862,1247486	590,5052132	247,9629068	-0,333444452	False	0,333444452
ENSMUSG00000035451	Foxa1	1148,553908	1613,42919	946,6898514	911,57839	1870,992842	-0,333379942	False	0,333379942
ENSMUSG00000020590	Snx13	1101,858064	837,4465796	811,6053366	874,531485	613,3819273	-0,333356165	False	0,333356165
ENSMUSG00000021701	Plk2	1449,209603	772,1411124	1148,767499	569,175177	1825,908677	0,33334834	False	0,33334834
ENSMUSG00000027889	Ampd2	538,2310395	805,7542205	698,4857836	678,0706251	1015,580135	0,333209954	False	0,333209954
ENSMUSG00000047514	Tspyl1	938,0129989	745,2506259	1181,714942	936,2763267	870,8362372	0,333202244	False	0,333202244
ENSMUSG00000040713	Creg1	2161,116411	2393,253299	1715,463513	2500,104772	2516,408255	-0,333178346	False	0,333178346
ENSMUSG00000084128	Esrp2	900,3286339	1134,202306	1376,104854	1200,095196	1210,153899	0,333154386	False	0,333154386
ENSMUSG00000003352	Cacnb3	516,1119557	430,247784	650,1628678	588,2599463	306,0977509	0,333117112	False	0,333117112
ENSMUSG00000038872	Zfhx3	1164,938414	1467,452263	1843,958539	2339,568183	1631,334913	0,333059886	False	0,333059886
ENSMUSG00000021938	Pspc1	222,0100635	249,6973747	273,4637738	176,2534572	270,5049892	-0,332973518	False	0,332973518
ENSMUSG00000030495	Slc7a10	893,7748313	48,0187259	1125,70429	111,140715	18,98280626	0,332844573	False	0,332844573
ENSMUSG00000043411	Usp48	1244,403271	1620,151812	1567,200021	2148,720491	1650,317719	0,33273523	False	0,33273523
ENSMUSG00000002578	Ikzf4	212,9985849	227,6087608	169,1302055	181,8666246	427,1131409	-0,332709508	False	0,332709508
ENSMUSG00000053950	Adnp2	363,736045	260,2614944	288,839247	255,9604346	128,1339423	-0,332625112	False	0,332625112
ENSMUSG00000018659	Pnpo	484,9813933	509,958869	493,1133913	610,712616	230,1665259	0,33256425	False	0,33256425
ENSMUSG00000015850	Adamtsl4	2140,635778	4240,053497	3658,264379	2695,442998	4799,090708	0,332483053	False	0,332483053
ENSMUSG00000000216	Scnn1g	2523,214006	1731,555256	1515,582361	2003,900771	1061,850725	-0,332451502	False	0,332451502
ENSMUSG00000018363	Smurf2	2419,991615	1163,973916	1921,934153	1553,724744	1335,914991	-0,332443139	False	0,332443139
ENSMUSG00000039286	Fndc3b	1146,915457	1213,913391	769,8719093	985,6722	1443,879701	0,332191498	False	0,332191498
ENSMUSG00000033075	Senp1	648,0072333	514,7607416	397,5658076	486,1002991	473,3837311	-0,332107887	False	0,332107887
ENSMUSG00000040414	Slc25a28	873,2941981	2303,938469	1099,346336	2008,391305	2904,369358	0,332106301	False	0,332106301
ENSMUSG00000067924	Rtl8b	1046,150742	626,1641857	1316,799457	718,4854306	863,7176848	0,331944898	False	0,331944898
ENSMUSG00000038121	Fam210a	364,5552703	273,7067376	272,3655257	289,6394392	264,5728623	-0,331879496	False	0,331879496
ENSMUSG00000025103	Btbd1	1047,789193	1117,875939	832,4720503	1039,558607	1343,033543	-0,331874746	False	0,331874746
ENSMUSG00000039648	Kyat1	371,1090729	392,7931778	416,2360251	467,0155299	155,4217263	0,331627253	False	0,331627253
ENSMUSG00000059070	Rpl18	5377,395043	9976,370492	6766,306467	7656,360369	14524,21964	0,331461061	False	0,331461061
ENSMUSG00000034820	Cpsf7	1293,556791	2000,460121	1627,603666	1895,005323	2401,324992	0,331406041	False	0,331406041
ENSMUSG00000052062	Pard3b	491,5351959	332,2895832	350,3411399	390,6764529	370,1647221	-0,33132042	False	0,33132042
ENSMUSG00000002477	Snrpd1	330,1478066	532,0474829	421,7272656	415,3743895	841,1756024	0,331300207	False	0,331300207
ENSMUSG00000054752	Fsd1l	370,2898476	259,3011198	294,3304874	282,9036383	244,4036306	-0,331218007	False	0,331218007
ENSMUSG00000030465	Psd3	648,0072333	266,984116	515,078353	290,7620727	223,0479736	-0,331218007	False	0,331218007
ENSMUSG00000025630	Hprt	557,8924474	810,5560931	701,7805279	733,0796659	951,5131638	0,331032898	False	0,331032898
ENSMUSG00000031592	Pcm1	801,2023694	465,7816412	636,9838907	578,1562449	327,453408	-0,3309098	False	0,3309098
ENSMUSG00000008435	Rdh13	145,8221081	363,9819423	183,4074306	385,0632854	301,3520494	0,330842626	False	0,330842626
ENSMUSG00000024091	Vapa	1681,869595	3999,959867	2115,225816	3068,157315	6820,759574	0,33074584	False	0,33074584
ENSMUSG00000053846	Lipg	835,6098331	1039,125228	664,4400929	939,6442271	733,2108918	-0,33069034	False	0,33069034
ENSMUSG00000019842	Traf3ip2	479,246816	795,1901008	381,0920863	607,3447155	1477,099612	-0,330629197	False	0,330629197
ENSMUSG00000038102	Trappc11	580,0115312	705,8752707	461,2641967	536,6188059	695,2452793	-0,330488268	False	0,330488268
ENSMUSG00000036026	Tmem63b	2300,384717	2612,218689	1829,681314	2661,763994	2636,237219	-0,33028277	False	0,33028277
ENSMUSG00000015337	Endog	460,4046335	688,5885294	578,7767421	799,3150415	699,9909808	0,330104596	False	0,330104596
ENSMUSG00000024413	Npc1	2225,015987	1769,970237	2657,760372	2928,950763	1916,077007	-0,330090602	False	0,330090602
ENSMUSG00000034994	Eef2	29600,2495	30032,83192	23548,63549	30956,61836	36335,46403	-0,32996587	False	0,32996587
ENSMUSG00000054252	Fgfr3	2243,858169	1793,019225	3046,540195	2473,161568	1785,570214	-0,329596622	False	0,329596622
ENSMUSG00000043962	Thrap3	2201,258452	2396,134422	1751,705699	2321,606047	3821,476185	-0,329568133	False	0,329568133
ENSMUSG00000014226	Cacybp	650,4649093	517,6418652	992,816271	553,4583082	562,3656355	-0,329516927	False	0,329516927
ENSMUSG00000001962	Fam50a	463,6815348	389,9120543	528,25733	582,6467788	333,3855349	0,32948726	False	0,32948726
ENSMUSG00000031523	Dlc1	4790,010484	1893,858549	3812,019111	2055,541911	2695,558489	-0,329473462	False	0,329473462
ENSMUSG00000060938	Rpl26	3070,456524	4726,003003	3858,145531	4986,737941	7176,687192	0,329454388	False	0,329454388
ENSMUSG00000024867	Pip5k1b	284,2711883	522,4437378	226,239106	361,4879822	799,6507137	-0,329419561	False	0,329419561
ENSMUSG00000041126	H2afv	536,5925889	674,1829116	676,5208219	745,4286342	709,482384	0,329312931	False	0,329312931
ENSMUSG00000040938	Slc16a11	1544,239741	2198,297272	1843,958539	2935,686564	1229,136705	-0,329251364	False	0,329251364
ENSMUSG00000029641	Rasl11a	612,7805442	540,6908536	769,8719093	374,9595841	647,7882636	0,329247939	False	0,329247939
ENSMUSG00000025078	Nhlrc2	1861,279942	1232,160507	1481,53667	1119,265585	1480,658888	-0,329200722	False	0,329200722
ENSMUSG00000034610	Zcchc11	724,1951887	1061,213842	576,5802459	804,9282089	1978,957553	-0,328857182	False	0,328857182
ENSMUSG00000054770	Kctd18	259,6944285	465,7816412	326,179682	412,0064891	624,0597558	0,328851855	False	0,328851855
ENSMUSG00000052560	Cpne8	457,9469575	1032,402607	364,618365	647,759521	1843,705058	-0,328793277	False	0,328793277
ENSMUSG00000000001	Gnai3	1186,238273	1512,589866	944,4933552	1027,209639	2065,566606	-0,328781273	False	0,328781273
ENSMUSG00000022858	Tra2b	959,3128574	1343,563951	1204,778152	1216,934698	2091,667965	0,328694215	False	0,328694215
ENSMUSG00000011960	Ccnt1	789,7332148	1099,628823	991,7180229	1105,793983	1657,436272	0,328564606	False	0,328564606
ENSMUSG00000034156	Tspoap1	258,8752032	247,7766256	200,9794	258,2057016	325,0805572	0,328540491	False	0,328540491
ENSMUSG00000021182	Ccdc88c	1448,390377	1399,265673	1088,363855	1478,5083	1818,790125	0,328528579	False	0,328528579
ENSMUSG00000003484	Cyp4f18	375,2051996	345,7348265	471,1484295	363,7332492	258,6407353	0,328501814	False	0,328501814
ENSMUSG00000046792	Zfp787	297,3787935	531,0871084	373,4043497	526,5151046	562,3656355	0,328436963	False	0,328436963
ENSMUSG00000000627	Sema4f	425,9971698	74,9092124	534,8468186	141,4518191	59,32126956	0,328281914	False	0,328281914
ENSMUSG00000025491	Ifitm1	2257,785	3936,575149	2834,578314	3441,994266	4447,908792	0,328226018	False	0,328226018
ENSMUSG00000030400	Ercc2	595,5768124	794,2297263	880,7949661	747,6739012	1200,662496	0,328121588	False	0,328121588
ENSMUSG00000028919	Arhgef19	395,6858327	289,0727299	315,1972011	271,6773034	153,0488755	-0,328100688	False	0,328100688
ENSMUSG00000062157	Ifnlr1	452,2123802	508,03812	360,2253727	553,4583082	618,1276288	-0,328100688	False	0,328100688
ENSMUSG00000006931	P3h4	373,5667489	155,5806719	297,6252317	273,9225704	201,6923165	-0,327869208	False	0,327869208
ENSMUSG00000034412	Tbc1d10a	382,5782275	480,187259	562,3030208	579,2788784	800,8371391	0,327842352	False	0,327842352
ENSMUSG00000015750	Aph1a	1246,041722	924,8406608	992,816271	916,068924	766,4308028	-0,327753711	False	0,327753711
ENSMUSG00000027456	Sdcbp2	38,50359035	516,6814906	48,32291585	126,8575838	1084,392808	0,327714532	False	0,327714532
ENSMUSG00000042447	Mios	203,9871063	317,8839654	162,5407169	291,8847062	314,4027287	-0,327676802	False	0,327676802
ENSMUSG00000047875	Gpr157	147,4605588	438,8911547	117,5125454	282,9036383	602,7040988	-0,327514345	False	0,327514345
ENSMUSG00000096188	Cmtm4	1641,727554	1139,004178	1230,037858	1239,387368	1308,627207	-0,327160561	False	0,327160561
ENSMUSG00000028207	Asph	822,5022278	502,2758729	655,6541082	538,8640729	403,384633	-0,327084668	False	0,327084668
ENSMUSG00000031400	G6pdx	1287,822213	2945,468647	1615,522937	1789,477775	2143,870682	0,327067794	False	0,327067794
ENSMUSG00000023908	Pkmyt1	229,3830914	308,2802203	287,7409989	295,2526066	303,7249002	0,327011749	False	0,327011749
ENSMUSG00000032198	Dock6	2977,884062	2545,952847	3946,005378	4507,373443	2374,037208	-0,32694504	False	0,32694504
ENSMUSG00000001249	Hpn	216,2754862	533,968232	172,4249497	477,1192312	800,8371391	-0,326901606	False	0,326901606
ENSMUSG00000037716	Ccdc33	503,0043505	398,5554249	276,758518	630,9200187	168,4724056	0,326886249	False	0,326886249
ENSMUSG00000010660	Plcd1	241,6714713	363,0215678	303,1164721	451,2986611	574,2298894	0,326825077	False	0,326825077
ENSMUSG00000031059	Ndufb11	1423,813618	1904,422669	1785,75139	1786,109875	2553,187442	0,32677094	False	0,32677094
ENSMUSG00000031513	Leprotl1	907,7016618	704,9148962	723,7454896	621,9389508	663,2117937	-0,326735746	False	0,326735746
ENSMUSG00000027207	Galk2	349,8092144	247,7766256	278,9550142	270,5546699	202,8787419	-0,326535808	False	0,326535808
ENSMUSG00000032381	Fam96a	532,4964622	721,241263	667,7348372	736,4475663	694,0588539	0,32650337	False	0,32650337
ENSMUSG00000027613	Eif6	1012,562504	2938,746025	1269,574789	1708,648165	4240,284348	0,326334419	False	0,326334419
ENSMUSG00000025218	Poll	258,8752032	159,42217	206,4706404	134,7160182	185,082361	-0,326320132	False	0,326320132
ENSMUSG00000000555	Itga5	1341,07186	2779,323855	1681,417822	1915,212726	3986,389315	0,326291726	False	0,326291726
ENSMUSG00000021576	Pdcd6	593,9383617	1394,4638	744,6122033	965,4647973	2254,208243	0,326176043	False	0,326176043
ENSMUSG00000025821	Zfp282	371,9282982	323,6462125	330,5726743	358,1200818	466,2651788	0,326126172	False	0,326126172
ENSMUSG00000024830	Rps6kb2	649,6456839	814,3975912	1135,588522	1222,547865	826,9384977	0,326080206	False	0,326080206
ENSMUSG00000045180	Shroom2	297,3787935	546,4531007	237,2215869	612,9578829	695,2452793	-0,326066471	False	0,326066471
ENSMUSG00000052395	Rft1	346,5323131	266,0237415	169,1302055	216,6682627	276,4371162	-0,326038552	False	0,326038552
ENSMUSG00000056737	Capg	567,7231513	848,9710739	711,6647606	756,6549691	911,1747005	0,326010225	False	0,326010225
ENSMUSG00000033287	Kctd17	676,6801197	474,4250119	501,899376	419,8649235	539,823553	-0,32598608	False	0,32598608
ENSMUSG00000025393	Atp5b	6245,77389	7584,077568	4982,751573	5717,57234	7792,44197	-0,325937695	False	0,325937695

ENSMUSG00000037049	Smpd1	1753,142199	1309,950842	1166,339469	1301,132209	1398,795536	-0,325757922	False	0,325757922
ENSMUSG00000043923	Ccdc84	255,5983019	461,9401431	375,6008459	522,0245706	320,3348556	0,325702535	False	0,325702535
ENSMUSG00000022462	Slc38a2	1708,904031	5658,52666	2141,58377	4298,563615	5763,654551	0,325606731	False	0,325606731
ENSMUSG00000047832	Cdca4	278,536611	282,3501083	254,7935563	222,2814301	530,3321499	-0,325479542	False	0,325479542
ENSMUSG00000060538	Tmem219	344,8938625	314,0424674	446,9869716	391,7990864	275,2506908	-0,325406271	False	0,325406271
ENSMUSG00000020516	Rps6kb1	676,6801197	950,7707728	847,8475235	1076,605512	887,4461927	0,32533083	False	0,32533083
ENSMUSG00000041775	Mapk1ip1	774,1679336	969,9782631	1166,339469	1488,612001	1064,223576	0,325305866	False	0,325305866
ENSMUSG00000006373	Pgrmc1	3271,985954	3204,769766	2674,234093	2633,698156	4099,099727	0,325140534	False	0,325140534
ENSMUSG00000029056	Pank4	375,2051996	557,0172204	470,0501814	551,2130413	676,262473	0,325134963	False	0,325134963
ENSMUSG00000021013	Ttc8	287,5480896	145,0165522	229,5338503	189,725059	123,3882407	-0,325096324	False	0,325096324
ENSMUSG00000040188	Scamp2	1255,872426	1576,934958	1127,900786	1002,511702	2224,547609	-0,325070842	False	0,325070842
ENSMUSG00000037458	Azin1	1695,796426	1156,29092	1206,974648	969,9553313	1353,711371	-0,325042819	False	0,325042819
ENSMUSG00000028165	Cisd2	617,6958962	904,6727959	493,1133913	687,051693	811,5149676	-0,324977314	False	0,324977314
ENSMUSG00000018322	Tomm34	724,1951887	1476,095634	907,1529202	1140,595621	3643,512377	0,324967177	False	0,324967177
ENSMUSG00000021065	Fut8	743,0373712	729,8846336	744,6122033	912,7010235	593,2126956	-0,324885299	False	0,324885299
ENSMUSG00000039997	Ifi203	1586,020232	204,5597723	1266,280045	427,7233579	310,8434525	-0,324814675	False	0,324814675
ENSMUSG00000054723	Vmac	664,3917398	374,546062	530,4538262	442,3175932	218,302272	-0,324806961	False	0,324806961
ENSMUSG00000034892	Rps29	2252,869648	4563,699709	2821,399337	3255,637107	5691,282602	0,324647036	False	0,324647036
ENSMUSG00000045962	Wnk1	6252,327692	4628,044802	4423,743296	4345,714221	4992,478046	-0,32463731	False	0,32463731
ENSMUSG00000005625	Psmd4	1144,457781	2125,308808	1433,213754	1765,902472	3306,567565	0,324589551	False	0,324589551
ENSMUSG00000029076	Sdf4	2837,796531	2974,279882	2216,26464	2604,509686	3553,344047	0,32440623	False	0,32440623
ENSMUSG00000038069	Cdkn2aip	192,5179517	250,6577492	153,7547322	196,4608599	403,384633	-0,324362165	False	0,324362165
ENSMUSG00000020521	Rnft1	484,162168	460,9797686	521,6678415	606,222082	302,5384748	0,324356051	False	0,324356051
ENSMUSG00000029426	Scarb2	4223,925784	6265,483355	3373,818125	3699,077334	11769,33988	-0,324202283	False	0,324202283
ENSMUSG00000022200	Golph3	1726,926988	2037,914727	1379,399598	1587,403748	2936,402843	-0,324166638	False	0,324166638
ENSMUSG00000025810	Nrp1	2513,383302	704,9148962	2007,597504	1053,030209	628,8054574	-0,324160648	False	0,324160648
ENSMUSG00000025782	Taf3	532,4964622	799,9919734	666,6365891	812,7866433	1461,676082	0,324128563	False	0,324128563
ENSMUSG00000032621	Srek1	1089,569684	2410,54004	1364,024125	2566,340147	2290,987431	0,324110692	False	0,324110692
ENSMUSG00000028675	Pnrc2	1794,103465	1246,566124	1433,213754	1360,631784	1048,800046	-0,324009298	False	0,324009298
ENSMUSG00000091896	Ube2d2a	1306,664396	1973,569634	1702,284535	1635,676988	2859,285193	0,323999228	False	0,323999228
ENSMUSG00000060992	Copz1	2117,697469	2150,278546	2387,591342	1691,808662	2854,539491	-0,323930089	False	0,323930089
ENSMUSG00000051786	Tubgcp6	420,2625925	426,406286	500,8011279	527,6377381	335,7583857	-0,323867616	False	0,323867616
ENSMUSG00000026638	Irf6	478,4275907	382,2290581	382,1903344	339,0353125	354,741192	-0,323863131	False	0,323863131
ENSMUSG00000042203	Tbc1d22b	639,81498	629,0453092	522,7660896	481,6097652	800,8371391	0,32385411	False	0,32385411
ENSMUSG00000036528	Ppfibp2	933,9168723	636,7283054	870,9107333	1168,661458	815,0742438	0,323491019	False	0,323491019
ENSMUSG00000097195	Snhg5	122,883799	288,1123554	153,7547322	252,5925342	526,7728737	0,323336091	False	0,323336091
ENSMUSG00000027712	Anxa5	7023,218724	4786,506597	5613,145975	4202,017135	4659,092511	-0,323322787	False	0,323322787
ENSMUSG00000030047	Arhgap25	348,9899891	416,8025408	278,9550142	319,9505433	455,5873502	-0,323153169	False	0,323153169
ENSMUSG00000027285	Haus2	306,3902721	408,1591701	244,9093235	300,865774	659,6525175	-0,323122795	False	0,323122795
ENSMUSG00000042302	Ehbp1	235,1176687	181,5107839	277,8567661	294,1299731	93,72760591	0,323070849	False	0,323070849
ENSMUSG00000030763	Lcmt1	244,9483726	172,8674132	306,4112164	196,4608599	99,65973287	0,322991402	False	0,322991402
ENSMUSG00000034127	Tspan8	1748,226847	560,8587185	2186,611942	1497,593069	556,4335085	0,32280481	False	0,32280481
ENSMUSG00000031486	Adgra2	1045,331517	465,7816412	835,7667945	623,0615843	503,0443659	-0,322788206	False	0,322788206
ENSMUSG00000033216	Eefsec	305,5710468	269,8652395	382,1903344	268,309403	162,5402786	0,32278344	False	0,32278344
ENSMUSG00000041420	Meis3	206,4447823	266,984116	263,579541	258,2057016	314,4027287	0,322764902	False	0,322764902
ENSMUSG00000069135	Fgfr1op	280,994287	646,3320506	351,439388	538,8640729	578,9755909	0,322735093	False	0,322735093
ENSMUSG00000022280	Rnf19a	1100,219614	1794,939974	879,696718	1151,821956	3011,147643	-0,322713392	False	0,322713392
ENSMUSG00000041168	Lonp1	647,188008	1107,311819	809,4088404	998,0211684	1846,077909	0,322683731	False	0,322683731
ENSMUSG00000057722	Lepr	1197,707427	1965,886638	957,6723322	1091,199748	1860,315013	-0,322671507	False	0,322671507
ENSMUSG00000078763	Slfn1	322,7747787	489,7910041	258,0883005	428,8459914	279,9963923	-0,322663108	False	0,322663108
ENSMUSG00000034845	Plvap	13640,10169	7664,749028	15427,09088	17058,41581	10306,47737	0,322629272	False	0,322629272
ENSMUSG00000026479	Lamc2	2097,216836	3984,593875	1677,024829	2927,82813	4861,971253	-0,322571983	False	0,322571983
ENSMUSG00000000563	Atp5f1	1450,848053	2346,194947	1814,30584	1860,203685	2696,744914	0,322521239	False	0,322521239
ENSMUSG00000030246	Ldhb	2986,076315	2163,723789	2514,98812	2237,408536	3733,680706	0,322347521	False	0,322347521
ENSMUSG00000024235	Map3k8	218,7331622	617,520815	273,4637738	543,3546069	1010,834433	0,322177765	False	0,322177765
ENSMUSG00000035860	Cdhr3	1649,100582	1090,025078	1072,988381	1319,094345	1072,528554	-0,322131641	False	0,322131641
ENSMUSG00000035674	Ndufa3	1157,565386	1849,681322	1546,333307	1447,074562	2024,041717	0,322045573	False	0,322045573
ENSMUSG00000021266	Wars	2927,911317	2381,728804	2729,146497	2706,669333	3660,122332	0,322020012	False	0,322020012
ENSMUSG00000039662	Icmt	1062,535249	807,6749696	850,0440197	691,5422269	414,0624615	-0,321901243	False	0,321901243
ENSMUSG00000035863	Palm	992,9010958	551,2549733	1241,020339	896,9841547	506,6036421	0,321804838	False	0,321804838
ENSMUSG00000013275	Slc41a1	879,8480007	1058,332719	703,977024	847,5882814	1532,861606	-0,321725967	False	0,321725967
ENSMUSG00000013076	Amotl1	2279,904084	1295,545225	1824,190073	1452,68773	950,3267384	-0,321717071	False	0,321717071
ENSMUSG00000026083	Eif5b	2080,832329	3694,56077	2600,651471	4807,116584	6105,345063	0,321712349	False	0,321712349
ENSMUSG00000032733	Snx33	675,8608944	556,0568459	844,5527792	767,8813039	473,3837311	0,321461243	False	0,321461243
ENSMUSG00000028134	Ptbp2	482,5237173	1521,233236	602,9382	1260,717404	2346,749424	0,32141028	False	0,32141028
ENSMUSG00000031583	Wrn	439,104775	210,3220194	351,439388	303,111041	112,7104122	-0,321289331	False	0,321289331
ENSMUSG00000074793	Hspa12b	1396,779182	494,5928767	1745,116211	1174,274626	196,9466149	0,32121915	False	0,32121915
ENSMUSG00000004891	Nes	1789,188113	1162,053167	1432,115506	1307,86801	736,770168	-0,321157222	False	0,321157222
ENSMUSG00000024067	Dpy30	481,704492	389,9120543	674,3243257	511,9208693	385,5882522	-0,32108728	False	0,32108728
ENSMUSG00000006390	Elovl1	8984,444156	5009,313486	7192,426725	4232,328239	3715,884325	-0,320950633	False	0,320950633
ENSMUSG00000040356	Skiv2l	1248,499398	2248,236746	1559,512284	2106,060418	2420,307798	0,320899792	False	0,320899792
ENSMUSG00000042642	Flad1	353,9053411	210,3220194	283,3480066	225,6493305	126,9475169	-0,320788482	False	0,320788482
ENSMUSG00000001052	Sec24b	1345,167986	1187,022904	911,5459126	1123,756119	1679,978354	0,320656295	False	0,320656295
ENSMUSG00000029642	Polr1d	658,6571625	946,9292747	822,5878175	921,6820914	1694,215459	0,320641982	False	0,320641982
ENSMUSG00000029338	Antxr2	1342,71031	658,8169193	1075,184878	712,8722631	543,3828292	-0,320563325	False	0,320563325
ENSMUSG00000022982	Sod1	2942,657373	3830,933952	3674,738101	3787,765379	4800,277133	0,32052186	False	0,32052186
ENSMUSG00000020423	Btg2	2699,347451	2909,934789	2743,423722	3190,524365	2161,667063	-0,320466349	False	0,320466349
ENSMUSG00000035133	Arhgap5	1218,188061	851,8521974	974,1460535	975,5684987	478,1294327	-0,320421791	False	0,320421791
ENSMUSG00000058569	Tmed9	1241,945595	1651,844171	1309,11172	994,6532679	2449,968433	-0,320336375	False	0,320336375
ENSMUSG00000035413	Tmem98	644,730332	338,0518303	805,0158481	545,5998738	438,9773948	0,320321327	False	0,320321327
ENSMUSG00000018821	Avpi1	417,8049165	1265,773615	521,6678415	587,1373128	1488,963866	0,320302031	False	0,320302031
ENSMUSG00000008734	Gprc5b	702,0761048	964,216016	562,3030208	873,4088515	1553,030837	-0,320279629	False	0,320279629
ENSMUSG00000020743	Mif4gd	823,3214532	1431,918406	1027,96021	1357,263884	1674,046227	0,320256698	False	0,320256698
ENSMUSG00000031165	Was	426,8163951	341,8933284	258,0883005	226,771964	256,2678845	-0,320069324	False	0,320069324
ENSMUSG00000051674	Dcun1d4	344,0746371	275,6274866	198,7829038	245,8567333	200,5058911	-0,320001784	False	0,320001784
ENSMUSG00000026771	Spopl	339,9785105	312,1217183	289,9374951	424,3554574	73,55837426	0,319829673	False	0,319829673
ENSMUSG00000037997	Parp11	276,8981604	176,7089113	221,8461137	166,1497558	107,9647106	-0,319796189	False	0,319796189
ENSMUSG00000024873	Cnih2	23,75753447	436,9704057	29,65269836	139,2065522	1390,490559	0,319778272	False	0,319778272
ENSMUSG00000066456	Hmgn3	166,3027413	251,6181237	207,5688885	301,9884075	242,0307798	0,319778272	False	0,319778272
ENSMUSG00000039616	Mocos	240,852246	300,5972241	180,1126863	205,4419278	272,87784	0,319683309	False	0,319683309
ENSMUSG00000031511	Arhgef7	2025,944233	2348,115696	1661,649356	1623,32802	3391,990194	-0,319639913	False	0,319639913
ENSMUSG00000030314	Atg7	570,1808273	660,7376683	456,8712044	588,2599463	594,399121	-0,319632013	False	0,319632013
ENSMUSG00000022377	Asap1	1052,704545	774,0618615	822,5878175	782,4755392	843,5484532	-0,319557738	False	0,319557738
ENSMUSG00000050248	Evc2	293,2826669	181,5107839	235,0250907	227,8945975	196,9466149	-0,319477026	False	0,319477026
ENSMUSG00000038650	Rnh1	1551,612768	3722,411632	1936,211378	2565,217514	4406,383903	0,319467908	False	0,319467908
ENSMUSG00000034951	Cog7	444,8393523	555,0964714	340,4569071	412,0064891	454,4009249	0,319454102	False	0,319454102
ENSMUSG00000025135	Anapc11	1171,492217	896,9897997	1461,768204	1031,700173	875,5819387	0,319367189	False	0,319367189
ENSMUSG00000028003	Lrat	614,4189949	69,14696529	766,577165	112,2633485	61,69412035	0,319208205	False	0,319208205
ENSMUSG00000038642	Ctss	2334,792181	1856,403943	2098,752095	1544,743676	2912,674336	0,319050262	False	0,319050262
ENSMUSG00000055553	Kxd1	673,4032184	922,9199117	861,0265005	1026,087006	839,989177	0,318900123	False	0,318900123
ENSMUSG00000039753	Fbxl5	1389,406154	1806,464468	1733,035482	2338,44555	1843,705058	0,3188328	False	0,3188328
ENSMUSG00000038773	Kdm3b	1215,730385	1020,878113	1052,121668	1140,595621	1516,25165	0,318685901	False	0,318685901
ENSMUSG00000022889	Mrpl39	462,0430842	511,8796181	501,899376	370,4690501	478,1294327	-0,318674364	False	0,318674364
ENSMUSG00000038914	Dido1	880,667226	917,1576646	806,1140962	706,1364622	1574,386494	-0,318649963	False	0,318649963
ENSMUSG00000032702	Kank1	434,1894231	441,7722782	348,1446437	449,0533941	647,7882636	-0,318637755	False	0,318637755
ENSMUSG00000090553	Snrpe	300,6556948	526,2852358	423,9237617	374,9595841	737,9565934	0,318622814	False	0,318622814
ENSMUSG00000031628	Casp3	294,9211176	239,133255	203,1758962	184,1118916	367,7918713	0,318560466	False	0,318560466
ENSMUSG00000025280	Polr3a	281,8135123	491,7117532	351,439388	365,9785162	555,2470831	0,318535105	False	0,318535105
ENSMUSG00000024212	Mllt1	1164,119189	782,7052321	933,5108743	687,051693	639,4832859	-0,318500044	False	0,318500044
ENSMUSG00000028757	Ddost	2419,991615	1751,723121	1940,60437	1331,443314	1764,214557	-0,318496022	False	0,318496022

ENSMUSG00000025950	Idh1	1076,462079	1345,4847	863,2229967	1021,596472	1447,438977	-0,318492293	False	0,318492293
ENSMUSG00000017715	Pgs1	1161,661513	1990,856376	1448,589227	1515,555205	2159,294212	0,318458797	False	0,318458797
ENSMUSG00000021187	Tc2n	241,6714713	557,9775949	303,1164721	403,0254212	301,3520494	0,318402699	False	0,318402699
ENSMUSG00000053137	Mapk11	375,2051996	297,7161006	467,8536852	407,5159551	207,6244435	0,318377597	False	0,318377597
ENSMUSG00000035407	Kank4	933,9168723	48,97910041	749,0051956	191,970326	18,98280626	-0,318318415	False	0,318318415
ENSMUSG00000069094	Pde7a	822,5022278	769,2599889	924,7248896	1101,303449	659,6525175	-0,318313325	False	0,318313325
ENSMUSG00000033307	Mif	754,5065257	1141,885302	1075,184878	940,7668606	1370,321327	0,318303862	False	0,318303862
ENSMUSG00000061536	Sec22c	287,5480896	148,8580503	230,6320984	165,0271223	106,7782852	-0,318209938	False	0,318209938
ENSMUSG00000029647	Pan3	1264,064679	1962,04514	1575,986005	2246,389604	2391,833589	0,31818444	False	0,31818444
ENSMUSG00000078495	Zfp984	303,9325961	170,9466642	243,8110754	217,7908961	122,2018153	-0,317987746	False	0,317987746
ENSMUSG00000055675	Kbtbd11	820,0445519	1133,241931	657,8506044	843,0977474	791,345736	-0,317942301	False	0,317942301
ENSMUSG00000029621	Arpc1a	1306,664396	1628,795182	1658,354612	1758,044038	1760,655281	0,317916553	False	0,317916553
ENSMUSG00000003123	Lipe	1138,723204	1419,433538	1446,392731	2001,655504	1741,672474	0,317898194	False	0,317898194
ENSMUSG00000029191	Rfc1	632,4419521	451,3760234	507,3906164	489,4681996	373,7239982	-0,317836231	False	0,317836231
ENSMUSG00000024169	Ift140	532,4964622	346,695201	427,218506	369,3464166	315,5891541	-0,317797798	False	0,317797798
ENSMUSG00000027002	Nckap1	2558,440695	3031,902353	2052,625675	2697,688265	3028,944024	-0,317794236	False	0,317794236
ENSMUSG00000037455	Slc18b1	332,6054826	112,3638186	266,8742852	175,1308237	79,49050121	-0,317651649	False	0,317651649
ENSMUSG00000046768	Rhoj	2007,921275	3472,714257	1611,129944	2075,749314	4872,649082	-0,317629849	False	0,317629849
ENSMUSG00000060376	Bckdha	910,1593378	1428,076908	730,3349781	1280,924807	1760,655281	-0,317560806	False	0,317560806
ENSMUSG00000048497	Mmgt2	302,2941455	181,5107839	376,699094	211,0550952	71,18552348	0,317459525	False	0,317459525
ENSMUSG00000019659	Ccdc12	532,4964622	730,8450081	695,1910393	663,4763898	1035,749367	0,317273187	False	0,317273187
ENSMUSG00000024182	Axin1	747,1334978	1103,470321	599,6434557	693,7874939	1771,333109	-0,317261109	False	0,317261109
ENSMUSG00000062127	Cttnbp2nl	2125,070497	2775,482357	1705,57928	2349,671885	2765,557587	-0,317248885	False	0,317248885
ENSMUSG00000039611	Tmem246	502,1851252	308,2802203	403,0570481	321,0731768	336,9448111	-0,317235246	False	0,317235246
ENSMUSG00000040648	Ppip5k2	398,1435087	191,1145291	319,5901934	233,5077649	109,151136	-0,317065396	False	0,317065396
ENSMUSG00000039967	Zfp292	551,3386448	414,8817917	442,5939792	378,3274845	427,1131409	-0,316954901	False	0,316954901
ENSMUSG00000021094	Dhrs7	554,6155461	1017,036614	690,798047	792,5792406	899,3104466	0,316775948	False	0,316775948
ENSMUSG00000006818	Sod2	1155,10771	2740,908874	1438,704995	1960,118065	5413,65906	0,316743414	False	0,316743414
ENSMUSG00000071415	Rpl23	4064,176845	7143,265664	5061,825435	6879,497998	8675,142461	0,316694575	False	0,316694575
ENSMUSG00000002900	Lamb1	1241,945595	746,2110004	997,2092634	960,9742634	822,1927961	-0,316633786	False	0,316633786
ENSMUSG00000031781	Ciapin1	368,6513969	813,4372167	459,0677005	525,3924711	1071,342128	0,316449705	False	0,316449705
ENSMUSG00000050244	Heatr1	520,2080824	390,8724288	358,0288765	312,0921089	647,7882636	0,316433516	False	0,316433516
ENSMUSG00000025268	Maged2	508,7389278	211,2823939	408,5482885	213,3003622	245,590056	-0,316418882	False	0,316418882
ENSMUSG00000078317	F8a	170,3988679	231,4502588	226,239106	212,1777287	336,9448111	0,316357481	False	0,316357481
ENSMUSG00000075420	Smim6	253,1406259	234,3313824	315,1972011	182,9892581	239,657929	0,316315663	False	0,316315663
ENSMUSG00000025372	Baiap2	1585,201007	3318,093959	1973,551813	3109,694754	5201,288915	0,316128607	False	0,316128607
ENSMUSG00000015488	Cacfd1	659,4763879	671,301788	544,7310514	561,3167426	821,0063707	0,316072411	False	0,316072411
ENSMUSG00000005262	Ufd1	918,351591	1829,513457	1143,276259	1351,650716	2183,02272	0,316055555	False	0,316055555
ENSMUSG00000035437	Rabgap1	825,7791291	938,285904	663,3418448	947,5026615	896,9375958	-0,31600342	False	0,31600342
ENSMUSG00000018666	Cbx1	652,9225852	642,4905525	812,7035847	674,7027246	341,6905127	0,315817311	False	0,315817311
ENSMUSG00000074305	Peak1	1459,859532	895,0690507	1172,928957	1037,31334	612,1955019	-0,315713925	False	0,315713925
ENSMUSG00000041438	Utp4	343,2554118	672,2621626	427,218506	486,1002991	1407,100514	0,315691676	False	0,315691676
ENSMUSG00000055053	Nfic	4492,631691	2261,68199	3609,941463	3072,647849	1388,117708	-0,31558535	False	0,31558535
ENSMUSG00000020432	Tcn2	4000,277269	6189,613768	4978,35858	7783,217953	6148,056377	0,315570148	False	0,315570148
ENSMUSG00000049550	Clip1	1940,744799	2905,132917	1559,512284	3045,704645	3763,341341	-0,315515504	False	0,315515504
ENSMUSG00000035545	Leng8	6098,313331	8347,57531	7588,894284	8152,56437	9840,212195	0,31547942	False	0,31547942
ENSMUSG00000021929	Kpna3	564,44625	611,7585679	454,6747082	478,2418647	702,3638316	0,31538235	False	0,31538235
ENSMUSG00000025812	Pard3	926,5438443	1122,677811	744,6122033	882,3899194	1340,660692	-0,315369986	False	0,315369986
ENSMUSG00000026389	Steap3	512,8350544	104,6808225	638,0821388	154,923421	67,6262473	0,315247271	False	0,315247271
ENSMUSG00000032959	Pebp1	976,5165892	910,435043	882,9914623	720,7306975	1214,899601	0,315120636	False	0,315120636
ENSMUSG00000026696	Vamp4	474,3314641	381,2686836	564,4995169	594,9957472	390,3339537	-0,315087534	False	0,315087534
ENSMUSG00000022160	Mettl3	409,6126633	278,5086102	509,5871126	399,6575207	142,371047	0,315068475	False	0,315068475
ENSMUSG00000022837	Iqcb1	193,3371771	320,765089	240,5163311	378,3274845	660,8389429	0,315015775	False	0,315015775
ENSMUSG00000029335	Bmp3	1045,331517	383,1894327	1300,325736	377,204851	365,4190205	0,314912516	False	0,314912516
ENSMUSG00000039081	Zfp503	1664,665864	1143,806051	1308,013472	1300,009576	1338,287841	-0,314844178	False	0,314844178
ENSMUSG00000017478	Zc3h18	1080,558206	1158,211669	868,7142371	981,1816661	1589,810024	-0,3148232	False	0,3148232
ENSMUSG00000021243	Fcf1	374,3859742	936,365155	465,6571891	573,665711	1534,048031	0,314741864	False	0,314741864
ENSMUSG00000028688	Toe1	248,2252739	339,0122048	315,1972011	308,7242084	354,741192	0,314668599	False	0,314668599
ENSMUSG00000027411	Vps16	543,9656168	1378,137433	676,5208219	1185,50096	1932,686962	0,314618874	False	0,314618874
ENSMUSG00000025178	Pi4k2a	984,7088425	1250,407622	791,836871	969,9553313	1863,87429	-0,314493967	False	0,314493967
ENSMUSG00000026663	Atf6	1304,20672	1305,14897	768,7736612	941,8894941	1621,84351	0,314462064	False	0,314462064
ENSMUSG00000032202	Rab27a	1256,691651	1003,591371	997,2092634	1562,705811	645,4154128	0,314415503	False	0,314415503
ENSMUSG00000036450	Hif1an	1271,437707	781,7448576	1022,468969	756,6549691	778,2950567	-0,31440372	False	0,31440372
ENSMUSG00000033047	Eif3l	1560,624247	1772,85136	1255,297564	1384,207087	2406,070693	-0,31409383	False	0,31409383
ENSMUSG00000024677	Ms4a6b	335,8823839	189,19378	270,1690295	229,017231	54,575568	-0,31409383	False	0,31409383
ENSMUSG00000001366	Fbxo9	483,3429427	283,3104828	388,7798229	383,940652	232,5393767	-0,31409383	False	0,31409383
ENSMUSG00000038628	Polr3k	344,0746371	242,974753	276,758518	259,3283351	208,8108689	-0,31409383	False	0,31409383
ENSMUSG00000038679	Trps1	315,4017507	187,273031	253,6953082	186,3571586	204,0651673	-0,31409383	False	0,31409383
ENSMUSG00000022091	Sorbs3	6180,235863	9999,419481	7683,34362	14341,64277	11444,25932	0,314072378	False	0,314072378
ENSMUSG00000033845	Mrpl15	370,2898476	489,7910041	481,0326623	471,5060638	460,3330518	0,314023036	False	0,314023036
ENSMUSG00000028907	Utp11	439,104775	391,8328033	545,8292994	468,1381633	323,8941318	0,313884616	False	0,313884616
ENSMUSG00000030036	Mogs	641,4534307	417,7629153	797,3281115	337,9126791	373,7239982	0,313829005	False	0,313829005
ENSMUSG00000024777	Ppp2r5b	1002,7318	1365,652565	1407,954048	1246,123169	1519,810926	0,313510893	False	0,313510893
ENSMUSG00000033389	Arhgap44	1319,772001	1567,331213	1062,005901	1266,330571	1774,892385	-0,313496935	False	0,313496935
ENSMUSG00000034917	Tjp3	666,8494158	644,4113015	727,0402339	536,6188059	1224,391004	-0,313463402	False	0,313463402
ENSMUSG00000024645	Timm21	291,6442162	282,3501083	362,4218688	306,4789415	148,3031739	0,313460548	False	0,313460548
ENSMUSG00000032309	Fbxo22	616,8766709	849,9314484	766,577165	907,0878561	1312,186483	0,313448936	False	0,313448936
ENSMUSG00000040415	Dtx3	1501,640024	1866,007688	2385,394846	2487,755803	2612,508712	0,313415922	False	0,313415922
ENSMUSG00000021519	Mterf3	335,8823839	513,8003671	417,3342732	418,74229	589,6534195	0,313247273	False	0,313247273
ENSMUSG00000004056	Akt2	2243,858169	1977,411132	2048,232683	1806,317278	3157,077966	-0,312930165	False	0,312930165
ENSMUSG00000042812	Foxf1	2509,287175	5685,417146	3116,828072	4032,499479	6063,820175	0,312800987	False	0,312800987
ENSMUSG00000023272	Creld2	546,4232928	456,177896	678,717318	459,1570955	523,2135975	0,312791841	False	0,312791841
ENSMUSG00000026078	Pdcl3	695,5223022	894,1086762	914,8406568	1000,266435	863,7176848	0,312463053	False	0,312463053
ENSMUSG00000021573	Tppp	480,0660414	105,641197	386,5833268	326,6863442	64,06697113	-0,312453471	False	0,312453471
ENSMUSG00000059552	Trp53	1288,641439	864,3370661	1037,844443	932,9084262	748,6344219	-0,312260672	False	0,312260672
ENSMUSG00000075706	Gpx4	4006,831072	6083,012197	4975,063836	5870,250494	7177,873617	0,31225335	False	0,31225335
ENSMUSG00000033589	Reep4	610,3228683	967,0971396	796,2298634	757,7776025	1150,83263	0,312201851	False	0,312201851
ENSMUSG00000022500	Litaf	1299,291368	2210,78214	1046,630427	1329,198047	3813,171207	-0,311972887	False	0,311972887
ENSMUSG00000039715	Wdr34	361,278369	456,177896	291,0357432	401,9027877	349,9954904	-0,311914531	False	0,311914531
ENSMUSG00000021583	Erap1	1254,233975	617,520815	1010,38824	733,0796659	516,0950452	-0,311896752	False	0,311896752
ENSMUSG00000034320	Slc26a2	848,7174383	1053,530846	561,2047727	769,0039374	627,619032	0,31187633	False	0,31187633
ENSMUSG00000013150	Gfod2	309,6671734	269,8652395	384,3868306	239,1209324	224,2343989	0,311840452	False	0,311840452
ENSMUSG00000060969	Irx1	851,9943396	2151,23892	686,4050546	1039,558607	2358,613678	-0,311783669	False	0,311783669
ENSMUSG00000041736	Tspo	1311,579748	1627,834808	1881,298974	1758,044038	1901,839902	0,311648773	False	0,311648773
ENSMUSG00000030842	Lamtor1	1024,031658	1202,388896	911,5459126	825,1356117	1416,591917	-0,311557165	False	0,311557165
ENSMUSG00000056429	Tgoln1	3965,05058	2204,059519	2030,660714	1705,280264	3195,043579	-0,311503661	False	0,311503661
ENSMUSG00000032279	Idh3a	688,1492743	912,355792	596,3487115	554,5809417	787,7864598	-0,311323512	False	0,311323512
ENSMUSG00000034371	Tkfc	210,5409089	261,2218689	231,7303464	216,6682627	107,9647106	0,311175099	False	0,311175099
ENSMUSG00000029086	Prom1	671,7647678	1048,728974	541,4363071	545,5998738	933,7167829	-0,311164499	False	0,311164499
ENSMUSG00000006342	Susd2	671,7647678	208,4012704	541,4363071	279,5357378	322,7077064	0,311164499	False	0,311164499
ENSMUSG00000038615	Nfe2l1	4570,458097	2726,503256	3652,773139	2863,838021	3683,85084	-0,311124126	False	0,311124126
ENSMUSG00000037686	Aspg	54,88809688	583,9077069	68,09138142	554,5809417	583,7212925	0,310978885	False	0,310978885
ENSMUSG00000007338	Mrpl49	362,0975943	330,3688342	449,1834678	391,7990864	160,1674278	0,310926238	False	0,310926238
ENSMUSG00000025283	Sat1	1876,025998	4256,379863	2327,187697	3573,342384	5082,646376	0,310907754	False	0,310907754
ENSMUSG00000052273	Dnah3	810,2138479	843,2088267	609,5276885	1004,756969	622,8733304	0,310471936	False	0,310471936
ENSMUSG00000042508	Dmtf1	641,4534307	578,1454598	859,9282524	679,1932586	517,2814706	-0,310395016	False	0,310395016
ENSMUSG00000032220	Myo1e	1593,39326	701,0733981	1284,950262	772,3718378	717,7873617	-0,310389861	False	0,310389861
ENSMUSG00000049988	Lrrc25	209,7216836	460,9797686	169,1302055	180,7439911	698,8045554	-0,310341695	False	0,310341695
ENSMUSG00000019998	Stx7	1858,822266	1173,577661	1499,108639	1292,151142	1421,337619	-0,310283893	False	0,310283893
ENSMUSG00000026923	Notch1	2585,475131	2089,774951	2684,118326	2248,634871	3205,721407	0,310219626	False	0,310219626

ENSMUSG00000022871	Fetub	174,4949946	405,2780466	216,3548732	308,7242084	270,5049892	0,310213964	False	0,310213964
ENSMUSG00000027318	Adam33	250,6829499	111,4034441	310,8042087	303,111041	168,4724056	0,310142165	False	0,310142165
ENSMUSG00000006673	Qrich1	882,3056767	1103,470321	711,6647606	932,9084262	2015,73674	-0,310080767	False	0,310080767
ENSMUSG00000063576	Klhdc3	1078,10053	2232,870754	1336,567922	2008,391305	3195,043579	0,310041444	False	0,310041444
ENSMUSG00000024298	Zfp871	1260,787778	813,4372167	1016,977729	995,7759014	930,1575067	-0,310037368	False	0,310037368
ENSMUSG00000033149	Phldb2	3401,423556	2290,493225	2404,065063	2743,716238	2289,801005	-0,310007384	False	0,310007384
ENSMUSG00000021241	Isca2	517,7504064	699,1526491	668,8330852	812,7866433	641,8561367	0,309993192	False	0,309993192
ENSMUSG00000040651	Fam208a	944,5668015	892,1879272	1037,844443	1170,906725	236,0986529	0,309901419	False	0,309901419
ENSMUSG00000021298	Gpr132	272,8020337	269,8652395	240,5163311	174,0081902	338,1312365	0,309728899	False	0,309728899
ENSMUSG00000031722	Hp	16402,52949	22148,15713	14876,86859	13233,60352	35837,16537	-0,309712352	False	0,309712352
ENSMUSG00000069237	Fam8a1	1302,568269	1862,16619	1614,424689	1888,269522	1892,348499	0,309661154	False	0,309661154
ENSMUSG00000025083	Afap1l2	341,6169612	143,0958032	275,6602699	136,9612852	112,7104122	-0,309488255	False	0,309488255
ENSMUSG00000005320	Fgfr4	638,9957547	145,0165522	791,836871	126,8575838	462,7059026	0,3093969	False	0,3093969
ENSMUSG00000041777	Cir1	357,1822424	597,3529502	442,5939792	900,3520552	797,2778629	0,309323468	False	0,309323468
ENSMUSG00000029759	Pon3	995,3587717	485,9495061	1233,332602	669,0895572	554,0606577	0,309273379	False	0,309273379
ENSMUSG00000006299	Aamp	1914,529588	2442,232399	2372,215869	2458,567333	3520,124136	0,309245343	False	0,309245343
ENSMUSG00000007867	Ift43	430,9125218	540,6908536	555,7135322	646,6368875	533,8914261	0,309151359	False	0,309151359
ENSMUSG00000027677	Ttc14	1101,038839	445,6137763	1364,024125	719,608064	226,6072497	0,3090038	False	0,3090038
ENSMUSG00000028465	Tln1	9128,627814	6990,566116	11308,66056	8077,347926	7933,626591	0,30895814	False	0,30895814
ENSMUSG00000046245	Pilra	389,9512554	483,0683825	350,3411399	394,0443533	288,3013701	0,308933634	False	0,308933634
ENSMUSG00000041781	Cpsf2	410,4318886	331,3292087	277,8567661	275,0452039	206,4380181	-0,308877434	False	0,308877434
ENSMUSG00000051934	Spats2	288,3673149	402,396923	232,8285945	287,3941722	690,4995777	-0,308639399	False	0,308639399
ENSMUSG00000026864	Hspa5	7526,223075	5767,04898	4972,86734	3545,276546	9320,557874	0,308490258	False	0,308490258
ENSMUSG00000038371	Sbf2	1116,60412	1720,030762	901,6616798	1788,355142	1963,534023	-0,30845967	False	0,30845967
ENSMUSG00000041959	S100a10	1394,321506	2228,068882	1726,445993	2028,598708	2784,540393	0,308241945	False	0,308241945
ENSMUSG00000041570	Camsap2	944,5668015	755,8147456	679,8155661	757,7776025	762,8715266	-0,308212714	False	0,308212714
ENSMUSG00000020265	Sumo3	1114,965669	1722,911885	1380,497846	1659,252291	1513,878799	0,308189348	False	0,308189348
ENSMUSG00000023089	Ndufa5	330,9670319	561,819093	482,1309104	409,7612221	747,4479965	0,308095946	False	0,308095946
ENSMUSG00000083282	Ctsf	381,7590022	249,6973747	319,5901934	472,6286973	291,8606462	0,308045053	False	0,308045053
ENSMUSG00000030091	Nup210	1022,393208	620,4019386	825,8825617	758,900236	724,9059141	-0,307941603	False	0,307941603
ENSMUSG00000055900	Tmem69	245,767598	218,0050156	304,2147202	198,7061269	142,371047	0,30779524	False	0,30779524
ENSMUSG00000031996	Aplp2	14161,12899	7902,921908	11440,45033	7832,613827	9882,923509	-0,307792447	False	0,307792447
ENSMUSG00000058173	Smco4	267,0674565	163,263668	330,5726743	285,1489053	42,71131409	0,307763287	False	0,307763287
ENSMUSG00000038301	Snx10	303,1133708	452,3363979	244,9093235	388,4311859	596,7719718	-0,307609796	False	0,307609796
ENSMUSG00000017858	Ift52	666,0301905	444,6534018	538,1415628	518,6566702	294,233497	-0,307601838	False	0,307601838
ENSMUSG00000022623	Shank3	3571,003198	6604,49556	4419,350304	5717,57234	5783,823782	0,307504866	False	0,307504866
ENSMUSG00000042265	Trem1	95,8493632	341,8933284	118,6107934	181,8666246	732,0244664	0,307394547	False	0,307394547
ENSMUSG00000058715	Fcer1g	2626,436397	4034,53335	3631,906425	3250,02394	5431,455441	0,307343697	False	0,307343697
ENSMUSG00000003099	Ppp5c	1211,634258	1644,161175	1499,108639	1536,885241	1549,471561	0,307150663	False	0,307150663
ENSMUSG00000022175	Lrp10	3827,420726	4172,82728	3093,764862	3420,66423	5820,60297	-0,307008946	False	0,307008946
ENSMUSG00000005299	Letm1	771,7102576	1198,547398	623,8049137	900,3520552	1454,55753	-0,306964365	False	0,306964365
ENSMUSG00000031153	Gripap1	657,0187119	961,3348925	909,3494164	989,0401005	812,701393	0,306790908	False	0,306790908
ENSMUSG00000022442	Ttll1	321,9555533	117,1656912	260,2847967	168,3950228	83,04977739	-0,306770489	False	0,306770489
ENSMUSG00000020486	Sept4	3358,004613	1482,818256	4153,574267	2025,230807	997,783754	0,306749138	False	0,306749138
ENSMUSG00000032806	Slc10a3	337,5208345	246,8162511	247,1058197	160,5365884	272,87784	-0,30672132	False	0,30672132
ENSMUSG00000054079	Utp18	193,3371771	263,1426179	242,7128273	239,1209324	386,7746775	0,306621345	False	0,306621345
ENSMUSG00000032184	Lysmd2	634,8996281	405,2780466	785,2473825	446,8081271	245,590056	0,306618696	False	0,306618696
ENSMUSG00000032511	Scn5a	370,2898476	182,4711584	457,9694525	438,9496927	174,4045325	0,306596376	False	0,306596376
ENSMUSG00000039968	Rsbn1l	602,9498403	909,4746685	745,7104514	1040,681241	1359,643498	0,306577573	False	0,306577573
ENSMUSG00000022553	Maf1	1302,568269	1988,935627	1053,219916	1667,110726	2854,539491	-0,306552281	False	0,306552281
ENSMUSG00000027931	Npr1	3742,221292	1917,867912	4628,01744	3457,711135	1580,318621	0,306499428	False	0,306499428
ENSMUSG00000041506	Rrp9	235,1176687	467,7023902	365,7166131	290,7620727	734,3973172	0,306456136	False	0,306456136
ENSMUSG00000044452	Zfp507	398,1435087	331,3292087	302,018224	319,9505433	492,3665374	0,306444179	False	0,306444179
ENSMUSG00000043131	Mob1a	2516,660203	1679,695032	2035,053706	1456,05563	1739,299624	-0,30644357	False	0,30644357
ENSMUSG00000037295	Ldlrap1	1048,608418	1039,125228	821,4895694	950,870562	1296,762953	0,306438747	False	0,306438747
ENSMUSG00000044934	Zfp367	331,7862572	218,0050156	232,8285945	268,309403	212,370145	-0,306356515	False	0,306356515
ENSMUSG00000015961	Adss	1258,330102	1575,974584	1645,175635	1555,970011	1823,535826	0,306303816	False	0,306303816
ENSMUSG00000040599	Mis12	253,1406259	285,2312318	313,0007049	249,2246337	161,3538532	0,306226846	False	0,306226846
ENSMUSG00000029726	Mepce	1086,292783	1901,541546	1343,157411	1554,847377	2206,751228	0,306215393	False	0,306215393
ENSMUSG00000028772	Zcchc17	389,9512554	346,695201	482,1309104	319,9505433	353,5547666	0,306131131	False	0,306131131
ENSMUSG00000043384	Gprasp1	1053,52377	376,466811	1302,522232	583,7694123	276,4371162	0,306085132	False	0,306085132
ENSMUSG00000006906	Stambp	561,988574	599,2736992	454,6747082	577,0336114	640,6697113	-0,305706044	False	0,305706044
ENSMUSG00000021537	Cetn3	413,7087899	535,888981	628,197906	594,9957472	511,3493436	0,305693638	False	0,305693638
ENSMUSG00000020107	Anapc16	944,5668015	1768,049488	1210,269392	2193,62583	1167,442585	0,305626864	False	0,305626864
ENSMUSG00000027263	Tubgcp4	828,2368051	729,8846336	1023,567217	819,5224442	346,4362142	0,305490627	False	0,305490627
ENSMUSG00000018574	Acadvl	806,9369466	1235,04163	997,2092634	1380,839187	1276,593721	0,305440338	False	0,305440338
ENSMUSG00000028126	Pip5k1a	879,8480007	1491,461626	1087,265607	1338,179114	2482,001919	0,305378202	False	0,305378202
ENSMUSG00000000753	Serpinf1	569,3616019	493,6325022	396,4675596	316,5826428	703,550257	0,305308284	False	0,305308284
ENSMUSG00000030423	Pop4	271,9828084	549,3342243	336,0639148	486,1002991	968,1231193	0,305220176	False	0,305220176
ENSMUSG00000030123	Plxnd1	2719,008859	1018,957364	2200,889167	893,6162543	1329,982864	-0,304994356	False	0,304994356
ENSMUSG00000036820	Amdhd2	407,1549873	174,7881623	502,997624	412,0064891	169,6588309	0,304973511	False	0,304973511
ENSMUSG00000009876	Cox4i2	1005,189476	1473,214511	1698,989791	1241,632635	1746,418176	0,304770911	False	0,304770911
ENSMUSG00000028521	Slc35d1	187,6025998	313,0820928	231,7303464	266,064136	486,4344104	0,304767165	False	0,304767165
ENSMUSG00000040771	Oard1	291,6442162	213,203143	360,2253727	323,3184438	187,4552118	0,304690338	False	0,304690338
ENSMUSG00000045930	Clec14a	5938,564392	3269,114859	4808,130127	4639,844195	1479,472463	-0,30463827	False	0,30463827
ENSMUSG00000016344	Ppdpf	1035,500813	838,4069542	1698,989791	1091,199748	839,989177	-0,304606097	False	0,304606097
ENSMUSG00000042197	Zfp451	416,1664659	560,8587185	513,9801049	670,2121907	643,0425621	0,304551797	False	0,304551797
ENSMUSG00000011179	Odc1	1354,99869	2996,368496	1097,149839	1187,746227	4361,299738	-0,304530887	False	0,304530887
ENSMUSG00000025505	Tmem80	374,3859742	209,3616449	462,3624448	241,3661993	134,0660692	0,304497833	False	0,304497833
ENSMUSG00000017264	Exosc10	883,124902	918,1180391	752,2999399	715,1175301	1275,407296	-0,304437126	False	0,304437126
ENSMUSG00000003500	Impdh1	3791,374811	894,1086762	4681,831597	1883,778988	1404,727663	0,304351955	False	0,304351955
ENSMUSG00000044134	Fam109a	233,4792181	351,4970736	358,0288765	371,5916836	288,3013701	0,304283551	False	0,304283551
ENSMUSG00000030704	Rab6a	2017,751979	1971,648885	1628,701914	1822,034147	2491,493322	0,304261857	False	0,304261857
ENSMUSG00000042284	Itga1	5425,729338	1733,476005	4394,090598	2417,029894	1633,707764	-0,304252465	False	0,304252465
ENSMUSG00000031399	Fam3a	598,0344884	309,2405948	484,3274066	372,7143171	194,5737642	-0,304246044	False	0,304246044
ENSMUSG00000000581	C1d	217,9139369	328,4480851	269,0707814	282,9036383	484,0615596	0,304227268	False	0,304227268
ENSMUSG00000023286	Ube2j2	621,7920228	1258,090618	767,6754131	895,8615212	2664,711429	0,304064335	False	0,304064335
ENSMUSG00000001227	Sema6b	428,4548458	1908,264167	347,0463956	1385,329721	2757,252609	-0,304014623	False	0,304014623
ENSMUSG00000004561	Mettl17	385,8551288	476,3457609	524,9625858	560,1941091	714,2280855	0,303949868	False	0,303949868
ENSMUSG00000020806	Rhbdf2	1148,553908	2650,633669	1417,838281	1696,299196	5335,354984	0,303874416	False	0,303874416
ENSMUSG00000043885	Slc36a4	459,5854082	787,5071047	372,3061016	822,8903447	726,0923394	-0,303843733	False	0,303843733
ENSMUSG00000025885	Myo5b	1107,592641	1504,90687	897,2686874	1349,405449	2212,683355	-0,303815404	False	0,303815404
ENSMUSG00000047879	Usp14	720,9182874	685,7074058	621,6084175	527,6377381	889,8190434	0,303676229	False	0,303676229
ENSMUSG00000032473	Cldn18	12689,80031	9270,495222	10281,79859	9197,736145	9897,160614	-0,303576709	False	0,303576709
ENSMUSG00000048970	C1galt1c1	372,7475236	241,054004	302,018224	222,2814301	166,0995548	-0,303563162	False	0,303563162
ENSMUSG00000062312	Erbb2	763,5180043	944,0481511	942,296859	1234,896834	972,8688208	0,303519457	False	0,303519457
ENSMUSG00000008683	Rps15a	1753,961424	3181,720778	2164,64698	2851,489053	4177,403803	0,303514745	False	0,303514745
ENSMUSG00000004562	Arhgef40	608,6844176	495,5532513	751,2016918	469,2607968	498,2986643	0,303505879	False	0,303505879
ENSMUSG00000034006	Pqlc1	583,2884325	841,2880777	498,6046317	472,6286973	1182,866115	-0,303502233	False	0,303502233
ENSMUSG00000031696	Vps35	2168,489439	1398,305298	1757,19694	1567,196345	1239,814534	-0,303414525	False	0,303414525
ENSMUSG00000063200	Nol7	618,5151215	490,7513787	763,2824208	650,004788	382,028976	0,303408098	False	0,303408098
ENSMUSG00000031570	Plpp5	443,2009017	838,4069542	546,9275475	572,5430775	1041,681494	0,303388913	False	0,303388913
ENSMUSG00000026385	Dbi	762,698779	1389,661927	941,1986109	1183,255693	1925,56841	0,303385801	False	0,303385801
ENSMUSG00000031985	Gnpat	1276,353059	1369,494063	1189,402679	1575,05478	966,9366939	0,30337455	False	0,30337455
ENSMUSG00000038542	Pcid2	556,2539967	777,9033595	686,4050546	729,7117654	1345,406394	0,30331638	False	0,30331638
ENSMUSG00000034974	Dapk3	545,6040675	998,7894987	673,2260776	877,8993855	1217,272451	0,303236661	False	0,303236661
ENSMUSG00000020325	Fstl3	774,9871589	1550,044472	1054,318164	1216,934698	956,2588654	0,303228812	False	0,303228812
ENSMUSG00000018217	Pmp22	9194,985065	5108,232061	7452,711521	5490,800376	6222,801177	-0,303081814	False	0,303081814
ENSMUSG00000039201	Tbc1d25	308,8479481	300,5972241	349,2428918	350,2616474	250,3357576	-0,303032368	False	0,303032368
ENSMUSG00000030168	Adipor2	2652,651607	3840,537697	2150,369755	3949,424601	3004,029091	-0,302850456	False	0,302850456

ENSMUSG00000022682	Rrn3	555,4347714	706,8356452	450,2817158	528,7603716	709,482384	-0,302789599	False	0,302789599
ENSMUSG00000029014	Dnajc2	338,3400599	601,1944482	417,3342732	461,4023624	607,4498003	0,302729402	False	0,302729402
ENSMUSG00000037573	Tob1	1133,807852	1168,775788	919,2336492	969,9553313	1688,283332	-0,30267265	False	0,30267265
ENSMUSG00000040728	Esrp1	445,6585776	801,9127225	361,3236208	591,6278467	1090,324935	-0,302647303	False	0,302647303
ENSMUSG00000021493	Pdlim7	1002,7318	2589,1697	1236,627346	1288,783241	6482,628338	0,302475034	False	0,302475034
ENSMUSG00000047804	Akap10	300,6556948	177,6692858	243,8110754	198,7061269	183,8959356	-0,302348622	False	0,302348622
ENSMUSG00000069089	Cdk7	226,1061901	212,2427685	195,4881596	205,4419278	278,8099669	0,302281646	False	0,302281646
ENSMUSG00000075700	Selenot	1955,490854	1400,226047	1585,870238	1398,801323	1082,019957	-0,30225606	False	0,30225606
ENSMUSG00000051335	Gfod1	552,9770954	285,2312318	395,3693115	169,5176563	448,4687979	-0,302212111	False	0,302212111
ENSMUSG00000020738	Sumo2	351,4476651	487,8702551	449,1834678	433,3365253	530,3321499	0,302177974	False	0,302177974
ENSMUSG00000035845	Alg12	295,7403429	279,4689847	364,618365	286,2715387	174,4045325	0,302056169	False	0,302056169
ENSMUSG00000028093	Acp6	489,8967453	319,8047145	577,678494	603,9768151	87,79547895	0,302015463	False	0,302015463
ENSMUSG00000070923	Klhl9	1117,423345	710,6771433	870,9107333	689,2969599	906,4289989	-0,301909944	False	0,301909944
ENSMUSG00000030801	Kat8	213,8178102	297,7161006	263,579541	287,3941722	367,7918713	0,301856364	False	0,301856364
ENSMUSG00000030304	Ergic2	702,0761048	1028,561109	865,4194929	1050,784942	1258,79734	0,30177219	False	0,30177219
ENSMUSG00000024099	Ndufv2	996,9972224	1918,828287	1228,93961	1493,102535	1776,078811	0,301752633	False	0,301752633
ENSMUSG00000026743	Mllt10	1018,297081	946,9292747	944,4933552	1255,104237	736,770168	0,301648666	False	0,301648666
ENSMUSG00000031563	Wwc2	1855,545365	1401,186422	1312,406464	1505,451504	1098,629912	-0,301647039	False	0,301647039
ENSMUSG00000028409	Smu1	640,6342054	859,5351936	561,2047727	519,7793037	1008,461583	-0,30160164	False	0,30160164
ENSMUSG00000055531	Cpsf6	1310,760522	1204,309645	1615,522937	1300,009576	1064,223576	0,301597106	False	0,301597106
ENSMUSG00000047719	Ubiad1	280,1750617	288,1123554	205,3723923	126,8575838	345,2497889	0,301311985	False	0,301311985
ENSMUSG00000070697	Utp3	435,8278737	670,3414135	537,0433147	696,0327609	831,6841993	0,301279984	False	0,301279984
ENSMUSG00000054428	Atpif1	1744,13072	1415,592039	2237,131354	1983,693368	1866,24714	-0,301102618	False	0,301102618
ENSMUSG00000033964	Zbtb41	298,1980189	241,054004	194,3899115	193,0929595	242,0307798	-0,301080142	False	0,301080142
ENSMUSG00000054455	Vapb	1713,000158	1124,59856	1390,382079	1064,256544	913,5475513	-0,301043893	False	0,301043893
ENSMUSG00000049044	Rapgef4	384,2166781	652,0942977	473,3449257	830,7487791	822,1927961	0,300971711	False	0,300971711
ENSMUSG00000045128	Rpl18a	11114,43001	23553,18505	13691,85891	21057,23628	36700,88305	0,300884368	False	0,300884368
ENSMUSG00000090272	Mndal	1331,241156	333,2499577	1080,676118	481,6097652	434,2316932	-0,300837733	False	0,300837733
ENSMUSG00000031668	Eif2ak3	548,0617435	547,4134752	479,9344142	455,789195	675,0760476	0,300711598	False	0,300711598
ENSMUSG00000021671	Poc5	461,2238588	661,6980429	374,5025978	471,5060638	769,9900789	-0,300491418	False	0,300491418
ENSMUSG00000021750	Fam107a	171,2180932	747,171375	210,8636328	1362,877051	618,1276288	0,300475133	False	0,300475133
ENSMUSG00000037526	Atg14	361,278369	309,2405948	295,4287355	358,1200818	444,9095217	0,300401096	False	0,300401096
ENSMUSG00000040488	Ltbp4	11166,0412	8238,092615	7940,333652	6882,865898	13750,67028	0,300384161	False	0,300384161
ENSMUSG00000068250	Amn1	331,7862572	335,1707068	408,5482885	333,4221451	196,9466149	0,300252478	False	0,300252478
ENSMUSG00000020790	Ankfy1	1772,803607	1045,84785	1439,803243	1090,077114	1429,642596	-0,300161049	False	0,300161049
ENSMUSG00000024431	Nr3c1	2415,076263	1247,526499	1961,471084	1477,385667	1478,286038	-0,300132679	False	0,300132679
ENSMUSG00000053289	Ddx10	291,6442162	263,1426179	314,098953	236,8756654	364,2325951	-0,300079468	False	0,300079468
ENSMUSG00000020154	Ptprb	13427,92233	5842,918567	10906,70176	8214,309212	5945,177636	-0,300021208	False	0,300021208
ENSMUSG00000038957	Edc3	587,3845591	437,9307802	466,7554372	477,1192312	215,9294212	-0,299955503	False	0,299955503
ENSMUSG00000025613	Cct8	1205,080455	1327,237584	1344,255659	978,9363991	1841,332207	-0,299842432	False	0,299842432
ENSMUSG00000019878	Hsf2	229,3830914	299,6368496	284,4462546	360,3653488	282,3692431	0,299823903	False	0,299823903
ENSMUSG00000066595	Flvcr1	310,4863988	464,8212667	382,1903344	497,326634	491,180112	0,299761222	False	0,299761222
ENSMUSG00000027189	Trim44	2642,820903	1847,760573	2147,075011	2138,616789	1706,079713	-0,299706066	False	0,299706066
ENSMUSG00000042331	Specc1	1139,542429	542,6116026	925,8231377	543,3546069	665,5846445	-0,299646119	False	0,299646119
ENSMUSG00000020605	Hs1bp3	580,0115312	193,0352781	713,8612568	376,0822176	113,8968376	0,299562122	False	0,299562122
ENSMUSG00000022000	Zc3h13	736,4835686	556,0568459	598,5452076	687,051693	906,4289989	0,299540679	False	0,299540679
ENSMUSG00000038023	Atp6v0a2	1050,246869	1343,563951	853,3387639	901,4746887	1579,132196	-0,299537994	False	0,299537994
ENSMUSG00000010721	Lmbr1	249,0444993	187,273031	220,7478656	306,4789415	111,5239868	0,299384402	False	0,299384402
ENSMUSG00000020376	Rnf130	977,3358146	794,2297263	727,0402339	671,3348242	603,8905241	-0,299298001	False	0,299298001
ENSMUSG00000016831	Tox4	1177,226794	1216,794514	1015,879481	983,4269331	1448,625403	0,299292295	False	0,299292295
ENSMUSG00000035325	Sec31a	3200,713351	3433,338902	2810,416856	2601,141785	5547,72513	-0,299248437	False	0,299248437
ENSMUSG00000005312	Ubqln1	2236,485141	1523,153985	1765,982925	1419,008725	1817,603699	-0,299195497	False	0,299195497
ENSMUSG00000029060	Mib2	767,614131	546,4531007	944,4933552	815,0319103	487,6208358	0,299159377	False	0,299159377
ENSMUSG00000006782	Cnp	1067,4506	405,2780466	867,6159891	465,8928964	313,2163033	-0,299040761	False	0,299040761
ENSMUSG00000022223	Sdr39u1	367,8321716	390,8724288	412,9412809	420,987557	298,9791986	-0,299002555	False	0,299002555
ENSMUSG00000041638	Gcn1l1	979,7934905	722,2016375	902,7599279	803,8055754	1205,408198	0,298972169	False	0,298972169
ENSMUSG00000053559	Smagp	894,5940566	1100,589198	1327,781938	1435,848228	1363,202775	0,298970995	False	0,298970995
ENSMUSG00000022783	Spag6	385,0359035	215,123892	313,0007049	273,9225704	136,43892	-0,298827073	False	0,298827073
ENSMUSG00000005510	Ndufs3	575,9154046	1093,866576	708,3700164	726,343865	1359,643498	0,298646236	False	0,298646236
ENSMUSG00000068856	Sf3b4	785,6370881	903,7124214	642,4751311	638,7784531	1460,489657	-0,298547388	False	0,298547388
ENSMUSG00000039414	Heatr5b	476,78914	296,755726	387,6815749	354,7521813	125,7610915	-0,298479205	False	0,298479205
ENSMUSG00000020589	Fam49a	1034,681587	1272,496236	845,6510273	859,9372497	1150,83263	0,298474528	False	0,298474528
ENSMUSG00000032253	Phip	763,5180043	1046,808225	939,0021147	1289,905875	1576,759345	0,29846623	False	0,29846623
ENSMUSG00000021134	Srsf5	4878,48682	8017,206476	5999,729302	9619,846335	12356,62045	0,29846368	False	0,29846368
ENSMUSG00000035172	Plekhh3	416,1664659	548,3738497	511,7836087	568,0525435	578,9755909	0,298373223	False	0,298373223
ENSMUSG00000068220	Lgals1	1365,648619	391,8328033	1679,221326	471,5060638	403,384633	0,298206067	False	0,298206067
ENSMUSG00000068134	Zfp120	239,2137953	277,5482357	286,6427508	284,0262718	194,5737642	-0,297983398	False	0,297983398
ENSMUSG00000024856	Cdk2ap2	1304,20672	1753,64387	1191,599175	1060,888644	2046,5838	-0,297899327	False	0,297899327
ENSMUSG00000062352	Itgb1bp1	281,8135123	375,5064365	433,8079945	400,7801542	346,4362142	0,297848961	False	0,297848961
ENSMUSG00000042133	Ppig	1409,886787	2546,913222	1733,035482	2905,37546	3687,410116	0,297721873	False	0,297721873
ENSMUSG00000037892	Pcdh18	739,7604699	179,5900349	601,8399519	157,1686879	179,1502341	-0,29767833	False	0,29767833
ENSMUSG00000057596	Trim30d	333,4247079	143,0958032	271,2672776	166,1497558	60,50769495	-0,297645988	False	0,297645988
ENSMUSG00000032058	Ppp2r1b	413,7087899	246,8162511	508,4888654	394,0443533	199,3194657	0,29760057	False	0,29760057
ENSMUSG00000076435	Acsf2	1275,533833	900,8312978	1037,844443	1023,841739	947,9538876	-0,297510945	False	0,297510945
ENSMUSG00000035547	Capn5	754,5065257	315,0028419	613,9206809	497,326634	225,4208243	-0,297481109	False	0,297481109
ENSMUSG00000029270	Fam69a	329,3285813	165,1844171	267,9725333	181,8666246	123,3882407	-0,297442589	False	0,297442589
ENSMUSG00000095098	Ccdc85b	736,4835686	599,2736992	1001,602256	712,8722631	660,8389429	-0,29743828	False	0,29743828
ENSMUSG00000038533	Cbfa2t2	371,1090729	387,0309307	369,0113574	301,9884075	542,1964038	-0,297350103	False	0,297350103
ENSMUSG00000027981	Rnpc3	275,2597097	460,0193941	338,2604109	353,6295479	465,0787534	0,297340883	False	0,297340883
ENSMUSG00000039196	Orm1	137,6298549	1188,943653	169,1302055	858,8146162	752,1936981	0,297340883	False	0,297340883
ENSMUSG00000015093	Clic3	3077,010326	3780,994477	4309,525495	4475,939706	5191,797512	0,297236457	False	0,297236457
ENSMUSG00000026578	Ccdc181	742,2181458	1032,402607	604,0364481	948,625295	619,3140542	-0,297207667	False	0,297207667
ENSMUSG00000013622	Atraid	863,4634942	1581,736831	1060,907652	1540,253142	1347,779244	0,297091992	False	0,297091992
ENSMUSG00000018209	Stk4	2825,508151	1351,246947	2299,731495	1804,072011	1255,238064	-0,297044922	False	0,297044922
ENSMUSG00000035754	Wdr18	514,4735051	568,5417146	479,9344142	418,74229	818,63352	-0,297034154	False	0,297034154
ENSMUSG00000014329	Bicc1	820,0445519	667,46029	709,4682645	903,7199556	1267,102318	-0,297020284	False	0,297020284
ENSMUSG00000049115	Agtr1a	225,2869648	244,8955021	183,4074306	342,403213	195,7601896	-0,296711751	False	0,296711751
ENSMUSG00000034154	Ino80	515,2927304	626,1641857	419,5307694	437,8270592	656,0932414	-0,296615614	False	0,296615614
ENSMUSG00000031666	Rbl2	980,6127159	1114,034441	798,4263596	1316,849078	1095,070636	-0,296524118	False	0,296524118
ENSMUSG00000004789	Dlst	1787,549663	2691,929774	2195,397927	2246,389604	3879,611029	0,296499135	False	0,296499135
ENSMUSG00000031832	Taf1c	253,9598512	394,7139269	311,9024568	327,8089777	342,8769381	0,296494479	False	0,296494479
ENSMUSG00000027848	Olfml3	2558,440695	891,2275526	3142,087778	1374,103386	974,0552462	0,296458693	False	0,296458693
ENSMUSG00000032411	Tfdp2	1064,173699	1697,942148	866,517741	1892,760056	1875,738544	-0,296432461	False	0,296432461
ENSMUSG00000028868	Wasf2	3045,060539	3027,100481	1741,821467	2477,652102	3739,612833	0,296418003	False	0,296418003
ENSMUSG00000022125	Cln5	663,5725145	804,793846	540,338059	609,5899825	1076,08783	-0,296391828	False	0,296391828
ENSMUSG00000020519	Sap30l	302,2941455	751,9732475	371,2078535	606,222082	923,0389544	0,296274195	False	0,296274195
ENSMUSG00000024962	Vegfb	740,5796952	458,0986451	909,3494164	701,6459283	339,3176619	0,29617976	False	0,29617976
ENSMUSG00000033306	Lpp	1191,97285	860,4955681	970,8513093	946,3800281	751,0072727	-0,296029114	False	0,296029114
ENSMUSG00000052957	Gas1	519,388857	207,4408959	404,1552962	637,6558196	404,5710584	0,295962864	False	0,295962864
ENSMUSG00000031558	Slit2	517,7504064	189,19378	421,7272656	295,2526066	135,2524946	-0,295946483	False	0,295946483
ENSMUSG00000027109	Sp3	1142,000105	1123,638186	930,21613	1041,803874	1612,352107	-0,295924922	False	0,295924922
ENSMUSG00000025085	Ablim1	3222,013209	3344,024071	2624,812929	3017,638808	4503,670785	-0,295747803	False	0,295747803
ENSMUSG00000021360	Gcnt2	1790,007338	1636,478179	978,5390459	1081,096046	2197,259825	0,295739976	False	0,295739976
ENSMUSG00000028709	Mob3c	466,1392108	846,0899503	572,1872535	702,7685618	927,784656	0,295726485	False	0,295726485
ENSMUSG00000031600	Vps37a	829,0560305	622,3226876	675,4225738	636,5331861	291,8606462	-0,29567921	False	0,29567921
ENSMUSG00000030220	Arhgdib	2754,235548	3380,518303	2669,841101	2760,55574	1882,857096	0,295592511	False	0,295592511
ENSMUSG00000000732	Icosl	480,8852667	466,7420157	656,7523563	391,7990864	625,2461812	-0,295578692	False	0,295578692
ENSMUSG00000025362	Rps26	5450,306097	8674,102646	6689,4291	7702,388342	12496,61865	0,295545835	False	0,295545835
ENSMUSG00000040061	Plcb2	435,8278737	112,3638186	534,8468186	289,6394392	64,06697113	0,295367291	False	0,295367291
ENSMUSG00000060036	Rpl3	4848,994708	5835,235571	3951,496619	4914,889398	6903,809352	-0,295286506	False	0,295286506

ENSMUSG00000022009	Nufip1	154,8335867	283,3104828	189,9969191	225,6493305	614,5683527	0,295257568	False	0,295257568
ENSMUSG00000050608	Minos1	1135,446303	1712,347765	1438,704995	1393,188155	1955,229045	0,295130633	False	0,295130633
ENSMUSG00000031732	Phlpp2	280,994287	258,3407453	316,2954492	229,017231	399,8253569	-0,29508465	False	0,29508465
ENSMUSG00000037720	Tmem33	1251,776299	702,9941471	1020,272473	592,7504802	647,7882636	-0,295022277	False	0,295022277
ENSMUSG00000044783	Hjurp	1322,229677	756,7751201	1622,112425	1170,906725	765,2443774	0,294901012	False	0,294901012
ENSMUSG00000074064	Mlycd	337,5208345	558,9379694	414,039529	550,0904078	635,9240097	0,294791951	False	0,294791951
ENSMUSG00000034957	Cebpa	2477,337387	2019,667611	2784,058902	2497,859505	2683,694235	-0,294672482	False	0,294672482
ENSMUSG00000040703	Cyp2s1	7367,293362	8481,067368	6006,31879	7004,110314	11450,19145	-0,294653642	False	0,294653642
ENSMUSG00000033953	Ppp3r1	1033,043137	925,8010353	876,4019738	1018,228571	1267,102318	0,294632528	False	0,294632528
ENSMUSG00000035000	Dpp4	1790,007338	411,0402937	1459,571708	877,8993855	315,5891541	-0,29442041	False	0,29442041
ENSMUSG00000024357	Sil1	557,073222	454,257147	412,9412809	325,5637107	434,2316932	-0,294357756	False	0,294357756
ENSMUSG00000007989	Fzd3	257,2367525	127,7298109	209,7653847	111,140715	202,8787419	-0,294320156	False	0,294320156
ENSMUSG00000015468	Notch4	2789,462237	1556,767094	2220,657633	3420,66423	778,2950567	0,294289476	False	0,294289476
ENSMUSG00000068580	Zfyve19	335,8823839	305,3990967	411,8430328	287,3941722	336,9448111	0,29413845	False	0,29413845
ENSMUSG00000027936	Crtc2	1371,383197	2784,125727	1681,417822	2320,483414	3801,306954	0,29404652	False	0,29404652
ENSMUSG00000033862	Cdk10	489,0775199	228,5691353	599,6434557	396,2896203	170,8452563	0,294041784	False	0,294041784
ENSMUSG00000022747	St3gal6	1470,509461	1129,400433	954,377588	1145,086155	1199,476071	-0,29391169	False	0,29391169
ENSMUSG00000063511	Snrnp70	4725,291683	5805,463961	6648,793921	5792,788784	7739,052827	0,293854676	False	0,293854676
ENSMUSG00000037890	Wdr19	552,1578701	450,4156489	386,5833268	394,0443533	352,3683412	-0,293823862	False	0,293823862
ENSMUSG00000030754	Copb1	1291,099115	1541,401101	1053,219916	1103,548716	2102,345793	-0,29379305	False	0,29379305
ENSMUSG00000042572	Ube2q1	1952,213953	2601,654569	2393,082582	2415,90726	3363,515984	0,29375901	False	0,29375901
ENSMUSG00000034210	Efcab14	1165,75764	908,514294	951,0828437	905,9652226	679,8217492	-0,293624967	False	0,293624967
ENSMUSG00000030614	Tmem126b	258,0559779	171,9070387	316,2954492	203,1966608	52,20271722	0,293588748	False	0,293588748
ENSMUSG00000016128	Stard13	1580,285655	888,3464291	1289,343255	896,9841547	844,7348786	-0,29354897	False	0,29354897
ENSMUSG00000038593	Tctn1	531,6772369	706,8356452	433,8079945	764,5134035	817,4470946	-0,29349406	False	0,29349406
ENSMUSG00000033446	Lpar6	305,5710468	238,1728804	374,5025978	299,7431406	129,3203676	0,293467873	False	0,293467873
ENSMUSG00000069895	Atxn1l	932,2784216	777,9033595	645,7698754	696,0327609	1142,527652	0,293396302	False	0,293396302
ENSMUSG00000030269	Mtmr14	572,6385032	1126,51931	701,7805279	1083,341313	1539,980158	0,29339524	False	0,29339524
ENSMUSG00000009406	Elk1	367,8321716	231,4502588	262,4812929	203,1966608	300,165624	-0,293288903	False	0,293288903
ENSMUSG00000025077	Dclre1a	206,4447823	216,0842665	260,2847967	264,9415025	168,4724056	-0,293243647	False	0,293243647
ENSMUSG00000048997	Atxn7l2	152,3759107	321,7254635	186,7021749	251,4699007	359,4868936	0,29310389	False	0,29310389
ENSMUSG00000023055	Calcoco1	2699,347451	2835,025577	2203,085663	4153,743895	3591,309659	-0,293085095	False	0,293085095
ENSMUSG00000008730	Hipk1	3380,123697	5591,300443	2758,799196	4204,262402	7110,24737	-0,293035592	False	0,293035592
ENSMUSG00000021196	Pfkp	899,5094085	902,7520469	736,9244667	734,2022994	1644,385592	-0,292960739	False	0,292960739
ENSMUSG00000060671	Atp8b2	598,8537137	363,9819423	445,8887235	403,0254212	488,8072612	-0,292937912	False	0,292937912
ENSMUSG00000066150	Slc31a1	1237,849468	1566,370839	1010,38824	1529,026807	1340,660692	-0,29292613	False	0,29292613
ENSMUSG00000021282	Eif5	2892,684628	5573,053328	2361,233388	4520,845045	7934,813017	-0,292868397	False	0,292868397
ENSMUSG00000030276	Ttll3	456,3085069	764,4581163	559,0082765	764,5134035	906,4289989	0,292860093	False	0,292860093
ENSMUSG00000041936	Agrn	3259,697574	2617,020561	3883,405237	3993,207307	2276,750326	0,29280985	False	0,29280985
ENSMUSG00000028007	Snx7	269,5251324	300,5972241	281,1515104	220,0361631	489,9936866	-0,292679155	False	0,292679155
ENSMUSG00000000058	Cav2	4423,816763	1652,804545	3612,13796	3319,627216	944,3946114	-0,292438634	False	0,292438634
ENSMUSG00000037415	Ranbp10	752,0488498	920,9991627	1237,725595	1331,443314	1042,867919	0,292373469	False	0,292373469
ENSMUSG00000024712	Rfk	1635,992977	1904,422669	1375,006605	1335,933847	2765,557587	-0,292317985	False	0,292317985
ENSMUSG00000038481	Cdk19	1362,371718	407,1987956	1112,525313	507,4303353	198,1330403	-0,29228223	False	0,29228223
ENSMUSG00000029326	Enoph1	243,309922	264,1029924	294,3304874	198,7061269	365,4190205	-0,292158796	False	0,292158796
ENSMUSG00000001289	Pfdn5	964,2282093	1300,347097	1180,616694	1430,23506	1620,657084	0,292094104	False	0,292094104
ENSMUSG00000044229	Nxpe4	461,2238588	50,89984945	376,699094	90,9333123	9,49140313	-0,292054581	False	0,292054581
ENSMUSG00000089872	Rps6kc1	371,1090729	222,8068882	303,1164721	225,6493305	275,2506908	-0,291971019	False	0,291971019
ENSMUSG00000029545	Acads	332,6054826	241,054004	249,3023158	271,6773034	215,9294212	-0,291917905	False	0,291917905
ENSMUSG00000040724	Kcna2	292,4634416	88,35445565	358,0288765	163,9044888	74,74479965	0,291819655	False	0,291819655
ENSMUSG00000033581	Igf2bp2	209,7216836	414,8817917	171,3267016	323,3184438	692,8724285	-0,291726017	False	0,291726017
ENSMUSG00000040888	Gfer	188,4218251	403,3572975	230,6320984	232,3851314	927,784656	0,291627231	False	0,291627231
ENSMUSG00000014554	Dguok	520,2080824	216,0842665	425,0220098	294,1299731	58,13484417	-0,291551261	False	0,291551261
ENSMUSG00000037791	Phf12	1355,817915	1664,32904	1659,45286	1736,714002	2589,966629	0,291544208	False	0,291544208
ENSMUSG00000037025	Foxa2	873,2941981	713,5582668	682,0120623	653,3726884	513,7221944	-0,291436515	False	0,291436515
ENSMUSG00000032501	Trib1	860,1865929	1678,734657	702,8787759	861,0598832	2937,589269	-0,291373753	False	0,291373753
ENSMUSG00000015291	Gdi1	3901,97023	3042,466473	4775,182684	3677,747298	2988,605561	0,291353156	False	0,291353156
ENSMUSG00000033191	Tie1	5535,505531	2072,48821	6773,994203	3229,816537	2121,3286	0,291291675	False	0,291291675
ENSMUSG00000047866	Lonp2	956,8551814	524,3644868	781,9526382	552,3356747	721,3466379	-0,291219363	False	0,291219363
ENSMUSG00000002949	Timm44	534,9541382	781,7448576	654,5558601	843,0977474	1070,155703	0,291101104	False	0,291101104
ENSMUSG00000026112	Coa5	1429,548195	654,0150467	1168,535965	819,5224442	570,6706132	-0,290857121	False	0,290857121
ENSMUSG00000034826	Nup54	201,5294303	364,9423168	164,7372131	304,2336745	525,5864483	-0,29082405	False	0,29082405
ENSMUSG00000022234	Cct5	1464,774884	1406,948669	1261,887052	1173,151992	1791,502341	0,29049097	False	0,29049097
ENSMUSG00000041308	Sntb2	760,241103	793,2693518	621,6084175	737,5701998	1181,67969	-0,290450988	False	0,290450988
ENSMUSG00000038633	Degs1	1766,249804	1946,679148	1444,196235	1586,281115	2095,227241	-0,290422614	False	0,290422614
ENSMUSG00000038766	Gabpb2	934,7360976	682,8262823	764,3806688	658,9858558	474,5701565	-0,290267816	False	0,290267816
ENSMUSG00000035392	Dennd1a	867,5596208	597,3529502	709,4682645	696,0327609	532,7050007	-0,290224755	False	0,290224755
ENSMUSG00000030588	Yif1b	666,0301905	346,695201	544,7310514	307,601575	412,8760362	-0,290043467	False	0,290043467
ENSMUSG00000057551	Zfp317	307,2094975	253,5388727	375,6008459	353,6295479	130,506793	0,289977494	False	0,289977494
ENSMUSG00000046324	Ermp1	777,4448349	318,84434	635,8856426	371,5916836	328,6398334	-0,289972974	False	0,289972974
ENSMUSG00000038253	Hoxa5	1187,057498	970,9386376	1457,375212	1260,717404	1572,013643	-0,289937791	False	0,289937791
ENSMUSG00000071054	Safb	883,9441273	2014,865739	1080,676118	1831,015214	3713,511475	0,28990712	False	0,28990712
ENSMUSG00000041261	Car8	1174,769118	915,2369156	875,3037257	960,9742634	460,3330518	-0,289807548	False	0,289807548
ENSMUSG00000036598	Ccdc113	284,2711883	357,2593207	286,6427508	299,7431406	232,5393767	-0,289792852	False	0,289792852
ENSMUSG00000022439	Parvg	353,0861157	407,1987956	288,839247	342,403213	825,7520723	-0,289753305	False	0,289753305
ENSMUSG00000015714	Cers2	3579,195452	3450,625643	3925,138664	4660,051597	2928,097866	-0,289671555	False	0,289671555
ENSMUSG00000027223	Mapk8ip1	425,1779445	352,4574481	263,579541	345,7711135	519,6543214	0,289485488	False	0,289485488
ENSMUSG00000030122	Ptms	10178,87468	7059,713081	12439,85609	9417,772308	7893,288128	0,289391721	False	0,289391721
ENSMUSG00000032479	Map4	5551,070813	3454,467141	4542,35409	4364,798991	3370,634537	-0,289325924	False	0,289325924
ENSMUSG00000054435	Gimap4	2262,700352	537,80973	1851,646275	1009,247503	143,5574723	-0,289237019	False	0,289237019
ENSMUSG00000027905	Ddx20	277,7173857	468,6627648	339,358659	435,5817923	761,6851012	0,289193329	False	0,289193329
ENSMUSG00000028986	Klhl7	550,5194194	476,3457609	431,6114984	537,7414394	672,7031968	0,289176659	False	0,289176659
ENSMUSG00000027993	Trim2	534,9541382	577,1850853	494,2116393	642,1463536	437,7909694	-0,289173019	False	0,289173019
ENSMUSG00000029504	Ddx51	217,0947115	177,6692858	208,6671366	197,5834934	291,8606462	-0,289130282	False	0,289130282
ENSMUSG00000024866	Acy3	286,7288643	227,6087608	350,3411399	258,2057016	221,8615482	0,289073267	False	0,289073267
ENSMUSG00000074698	Csnk2a1	1452,486504	1355,088445	1832,976058	1541,375775	1188,798242	-0,289020871	False	0,289020871
ENSMUSG00000020224	Llph	194,1564024	242,974753	237,2215869	250,3472672	328,6398334	0,289016018	False	0,289016018
ENSMUSG00000040433	Zbtb38	679,1377957	829,7635835	564,4995169	737,5701998	563,5520608	0,288996018	False	0,288996018
ENSMUSG00000001986	Gria3	282,6327377	416,8025408	400,8605519	918,3141909	231,3529513	-0,288833081	False	0,288833081
ENSMUSG00000018362	Kpna2	370,2898476	417,7629153	382,1903344	303,111041	412,8760362	-0,288808591	False	0,288808591
ENSMUSG00000031703	Itfg1	1418,898266	1025,679985	1156,455236	883,5125529	1161,510458	-0,288769008	False	0,288769008
ENSMUSG00000031503	Col4a2	9430,102734	19958,50323	11519,52419	19683,1329	23211,22635	0,288735735	False	0,288735735
ENSMUSG00000024479	Mal2	6171,224385	4036,454099	5051,941202	3516,088076	4946,207456	-0,288718904	False	0,288718904
ENSMUSG00000029174	Tbc1d1	512,8350544	450,4156489	600,7417038	419,8649235	586,0941433	-0,288569613	False	0,288569613
ENSMUSG00000019986	Ahi1	244,1291473	185,352282	199,8811519	175,1308237	144,7438977	-0,288502116	False	0,288502116
ENSMUSG00000027750	Postn	4800,660414	2517,141612	2437,012506	1375,226019	3930,627321	-0,288473305	False	0,288473305
ENSMUSG00000112148	Lilrb4	395,6858327	308,2802203	323,9831858	315,4600094	289,4877955	-0,28843647	False	0,28843647
ENSMUSG00000050002	Idnk	515,2927304	411,0402937	629,2961541	610,712616	321,521281	0,288346886	False	0,288346886
ENSMUSG00000045038	Prkce	1195,249751	1863,126565	1459,571708	1716,506599	2804,709625	0,288232986	False	0,288232986
ENSMUSG00000037533	Rapgef6	830,6944811	896,9897997	654,5558601	728,5891319	1014,39371	0,288227829	False	0,288227829
ENSMUSG00000027566	Psma7	826,5983545	1531,797356	1009,289992	1189,991494	1995,567508	0,288082357	False	0,288082357
ENSMUSG00000063015	Ccni	3628,348971	3292,163847	3600,057231	4429,911733	3058,604659	0,287964733	False	0,287964733
ENSMUSG00000021911	Parg	380,9397768	397,5950504	316,2954492	293,0073396	465,0787534	0,287912095	False	0,287912095
ENSMUSG00000033386	Frrs1	293,2826669	213,203143	358,0288765	242,4888328	279,9963923	0,287784142	False	0,287784142
ENSMUSG00000023307	March5	833,9713824	1476,095634	1018,075977	1179,887793	2032,346695	0,287775447	False	0,287775447
ENSMUSG00000024597	Slc12a2	1282,906861	1981,25263	1566,101773	1844,486816	1812,857998	0,287761534	False	0,287761534
ENSMUSG00000040430	Pitpnc1	3951,942975	1511,629491	3237,635362	2128,513088	1477,099612	-0,287621618	False	0,287621618
ENSMUSG00000027999	Pla2g12a	329,3285813	1061,213842	401,9588	611,8352495	1509,133098	0,287519912	False	0,287519912
ENSMUSG00000029016	Clcn6	516,931181	546,4531007	533,7485705	630,9200187	336,9448111	0,2874849	False	0,2874849
ENSMUSG00000060477	Irak2	699,6184289	1058,332719	573,2855016	639,9010866	1600,487853	-0,287314498	False	0,287314498

ENSMUSG00000034120	Srsf2	3458,769329	4220,846006	4467,67322	4496,147108	4774,175774	0,287273394	False	0,287273394
ENSMUSG00000053617	Sh3pxd2a	2560,079145	2750,512619	2118,520561	1991,551803	3123,858055	0,287140488	False	0,287140488
ENSMUSG00000032563	Mrpl3	360,4591437	444,6534018	295,4287355	361,4879822	507,7900675	-0,287025586	False	0,287025586
ENSMUSG00000063894	Zkscan8	415,3472406	145,9769267	340,4569071	202,0740273	33,21991096	-0,286845767	False	0,286845767
ENSMUSG00000040170	Fmo2	16624,53955	26622,54201	28130,52651	47197,75699	13627,28204	-0,286816537	False	0,286816537
ENSMUSG00000027349	Fam98b	358,820693	422,5647879	366,8148612	294,1299731	665,5846445	-0,286809284	False	0,286809284
ENSMUSG00000014776	Nol3	336,7016092	123,8883128	410,7447847	118,9991494	199,3194657	0,286771641	False	0,286771641
ENSMUSG00000018199	Trove2	1092,846586	648,2527996	795,1316153	895,8615212	462,7059026	-0,28674324	False	0,28674324
ENSMUSG00000019763	Rmnd1	263,7905551	145,0165522	321,7866896	157,1686879	73,55837426	0,286711741	False	0,286711741
ENSMUSG00000063382	Bcl9l	3496,453694	2225,187758	2866,427508	2214,955866	2352,681551	-0,286638605	False	0,286638605
ENSMUSG00000046447	Camk2n1	1547,516642	2180,050156	2051,527427	2754,942573	1887,602797	0,286600291	False	0,286600291
ENSMUSG00000023909	Paqr4	690,6069503	475,3853864	842,3562831	514,1661362	303,7249002	0,286565711	False	0,286565711
ENSMUSG00000007589	Tinf2	508,7389278	975,7405102	620,5101694	747,6739012	957,4452907	0,286529364	False	0,286529364
ENSMUSG00000040928	S100pbp	532,4964622	757,7354946	510,6853606	859,9372497	436,604544	-0,286444795	False	0,286444795
ENSMUSG00000028402	Mpdz	521,0273077	362,0611933	427,218506	424,3554574	390,3339537	-0,286384846	False	0,286384846
ENSMUSG00000028943	Espn	420,2625925	147,8976758	315,1972011	344,64848	167,2859802	-0,286165396	False	0,286165396
ENSMUSG00000035559	Mpv17l2	480,0660414	1239,843503	585,3662306	1485,244101	1287,27155	0,286106633	False	0,286106633
ENSMUSG00000029110	Rnf4	1401,694534	1537,559603	1239,922091	1149,576689	2502,17115	-0,28606927	False	0,28606927
ENSMUSG00000026239	Pde6d	525,1234343	369,7441894	640,278635	504,0624349	147,1167485	0,286043291	False	0,286043291
ENSMUSG00000033596	Rfwd3	403,0588607	410,0799192	330,5726743	396,2896203	498,2986643	-0,286023064	False	0,286023064
ENSMUSG00000047963	Stbd1	333,4247079	220,8861391	273,4637738	216,6682627	198,1330403	-0,286011288	False	0,286011288
ENSMUSG00000037266	Rsrp1	5440,475394	7295,965213	7904,091485	9325,716362	4462,145897	-0,285995036	False	0,285995036
ENSMUSG00000028760	Eif4g3	3185,14807	4632,846675	2612,7322	5367,310693	5134,849093	-0,285801183	False	0,285801183
ENSMUSG00000028096	Gpr89	344,0746371	175,7485368	282,2497585	194,2155929	144,7438977	-0,285749204	False	0,285749204
ENSMUSG00000028789	Azin2	480,0660414	739,4883788	694,0927912	672,4574577	393,8932299	-0,285428266	False	0,285428266
ENSMUSG00000031504	Rab20	99,12626451	450,4156489	120,8072896	250,3472672	1299,135803	0,285368241	False	0,285368241
ENSMUSG00000049680	Urgcp	515,2927304	585,8284559	422,8255137	434,4591588	822,1927961	-0,285329807	False	0,285329807
ENSMUSG00000061887	Ssbp3	1612,235443	1261,932117	1964,765828	1412,272924	1321,677886	0,285294931	False	0,285294931
ENSMUSG00000047793	Sned1	1612,235443	1111,153317	1964,765828	1632,309088	632,3647335	0,285294931	False	0,285294931
ENSMUSG00000022816	Fstl1	1154,288485	1461,690016	1485,929662	1406,659757	1892,348499	0,285269578	False	0,285269578
ENSMUSG00000007415	Gatad1	1146,915457	941,1670276	923,6266415	926,1726253	711,8552348	-0,285236366	False	0,285236366
ENSMUSG00000022758	P2rx6	390,7704808	130,6109344	320,6884415	220,0361631	125,7610915	-0,285149133	False	0,285149133
ENSMUSG00000047090	Tmem198b	294,9211176	351,4970736	304,2147202	475,9965977	242,0307798	-0,285138598	False	0,285138598
ENSMUSG00000020694	Tlk2	646,3687826	1201,428522	530,4538262	901,4746887	1867,433566	-0,285130347	False	0,285130347
ENSMUSG00000051403	Ppp1r37	1764,611353	1641,280051	1480,438422	1448,197196	3152,332265	-0,28509241	False	0,28509241
ENSMUSG00000029713	Gnb2	3293,285813	5320,474829	4011,900263	4186,300266	5974,83827	0,284758017	False	0,284758017
ENSMUSG00000062373	Tmem65	715,1837101	570,4624637	549,1240437	587,1373128	392,7068045	-0,284615933	False	0,284615933
ENSMUSG00000000317	Bcl6b	734,0258926	511,8796181	893,9739432	356,9974483	202,8787419	0,284401827	False	0,284401827
ENSMUSG00000042348	Arl15	761,0603284	321,7254635	624,9031617	315,4600094	390,3339537	-0,284378179	False	0,284378179
ENSMUSG00000024052	Lpin2	2441,291473	4818,198956	2972,957573	7212,920143	4574,856309	0,284254321	False	0,284254321
ENSMUSG00000053175	Bcl3	742,2181458	2618,94131	903,8581759	1070,992345	4608,07622	0,284253145	False	0,284253145
ENSMUSG00000022706	Mrpl40	353,0861157	480,187259	431,6114984	429,9686248	438,9773948	0,284211297	False	0,284211297
ENSMUSG00000015605	Srf	1055,162221	641,530178	866,517741	489,4681996	819,8199454	-0,284163623	False	0,284163623
ENSMUSG00000038489	Polr2l	294,1018922	490,7513787	359,1271246	358,1200818	844,7348786	0,284127354	False	0,284127354
ENSMUSG00000038208	Pgap3	293,2826669	266,0237415	203,1758962	169,5176563	357,1140428	0,284093056	False	0,284093056
ENSMUSG00000028879	Stx12	1298,472143	1515,470989	1066,398893	1084,463947	1982,516829	-0,284067875	False	0,284067875
ENSMUSG00000056598	Drc3	521,846533	457,1382705	483,2291585	635,4105526	373,7239982	0,284063455	False	0,284063455
ENSMUSG00000026819	Slc25a25	399,7819593	1110,192943	328,3761781	351,3842809	1791,502341	-0,283863899	False	0,283863899
ENSMUSG00000027200	Sema6d	2147,189581	1299,386723	1763,786428	1436,970861	1487,777441	-0,283773696	False	0,283773696
ENSMUSG00000016028	Celsr1	760,241103	671,301788	460,1659486	554,5809417	925,4118052	0,283638475	False	0,283638475
ENSMUSG00000026933	Camsap1	338,3400599	415,8421663	411,8430328	554,5809417	447,2823725	0,283620579	False	0,283620579
ENSMUSG00000005779	Psmb4	943,7475762	2111,863565	1148,767499	1229,283666	2719,286997	0,283613899	False	0,283613899
ENSMUSG00000002984	Tomm40	507,1004771	957,4933944	617,2154251	698,2780278	896,9375958	0,283502486	False	0,283502486
ENSMUSG00000046718	Bst2	844,6213117	218,9653901	1027,96021	406,3933217	453,2144995	0,283407867	False	0,283407867
ENSMUSG00000021774	Ube2e1	646,3687826	577,1850853	442,5939792	496,2040005	786,6000344	0,283272727	False	0,283272727
ENSMUSG00000033237	Arid2	1181,322921	1933,233905	1437,606746	2313,747613	1668,1141	0,283265697	False	0,283265697
ENSMUSG00000030243	Recql	319,4978773	200,7182742	388,7798229	197,5834934	84,23620278	0,283147001	False	0,283147001
ENSMUSG00000067586	S1pr3	1235,391792	1175,49841	1288,245007	843,0977474	1503,200971	0,283069253	False	0,283069253
ENSMUSG00000024369	Nelfe	380,9397768	376,466811	463,4606929	337,9126791	345,2497889	0,282884047	False	0,282884047
ENSMUSG00000020448	Rnf185	938,8322242	1464,57114	1142,178011	1284,292707	1795,061617	0,282848249	False	0,282848249
ENSMUSG00000010045	Tmem115	847,0789876	990,146128	696,2892874	758,900236	1499,641695	-0,282809676	False	0,282809676
ENSMUSG00000022812	Gsk3b	1418,898266	1309,950842	1166,339469	1316,849078	2186,581996	-0,282783399	False	0,282783399
ENSMUSG00000019823	Mical1	904,4247605	1114,034441	743,5139552	1165,293558	1200,662496	-0,282640669	False	0,282640669
ENSMUSG00000039202	Abhd2	5823,872846	6571,842826	3681,327589	4004,433642	7084,146011	0,28261509	False	0,28261509
ENSMUSG00000031066	Usp11	414,5280152	240,0936295	316,2954492	257,0830681	504,2307913	0,282614613	False	0,282614613
ENSMUSG00000016946	Kctd5	278,536611	310,2009693	237,2215869	229,017231	357,1140428	-0,28241082	False	0,28241082
ENSMUSG00000001630	Stk38l	299,0172442	327,4877106	265,7760372	245,8567333	363,0461697	-0,28241082	False	0,28241082
ENSMUSG00000041961	Znrf3	294,9211176	193,9956526	220,7478656	242,4888328	170,8452563	-0,28241082	False	0,28241082
ENSMUSG00000034706	Dnaic2	512,8350544	286,1916063	421,7272656	337,9126791	156,6081516	0,282184582	False	0,282184582
ENSMUSG00000021268	Meg3	255,5983019	441,7722782	310,8042087	362,6106157	505,4172167	0,282127789	False	0,282127789
ENSMUSG00000020954	Strn3	815,9484252	967,0971396	671,0295814	954,2384625	1125,917696	-0,282101598	False	0,282101598
ENSMUSG00000009569	Mkl2	1243,584046	891,2275526	940,1003628	1022,719105	836,4299008	-0,282094057	False	0,282094057
ENSMUSG00000023809	Rps6ka2	634,0804027	521,4833632	445,8887235	335,6674121	487,6208358	-0,282044561	False	0,282044561
ENSMUSG00000074922	Fam122a	387,4935795	588,7095795	471,1484295	538,8640729	694,0588539	0,282009229	False	0,282009229
ENSMUSG00000025204	Ndufb8	422,7202685	775,9826105	513,9801049	547,8451408	807,9556914	0,282009229	False	0,282009229
ENSMUSG00000020919	Stat5b	977,3358146	604,0755718	801,7211038	803,8055754	361,8597443	-0,282007776	False	0,282007776
ENSMUSG00000022961	Son	8189,795589	13155,21015	9957,815409	15432,84252	21059,05069	0,282001828	False	0,282001828
ENSMUSG00000003166	Dgcr2	934,7360976	963,2556415	768,7736612	826,2582451	1366,762051	-0,2820002	False	0,2820002
ENSMUSG00000009905	Kdsr	484,162168	1038,164854	588,6609749	1108,03925	779,4814821	0,281946635	False	0,281946635
ENSMUSG00000024875	Yif1a	545,6040675	405,2780466	663,3418448	334,5447786	321,521281	0,281898137	False	0,281898137
ENSMUSG00000031154	Otud5	1531,132135	1969,728136	1861,530508	1934,297495	3069,282487	0,281890469	False	0,281890469
ENSMUSG00000017664	Slc35c2	822,5022278	426,406286	676,5208219	391,7990864	466,2651788	-0,281885249	False	0,281885249
ENSMUSG00000033009	Ogfod1	504,6428011	341,8933284	415,137777	374,9595841	232,5393767	-0,281672352	False	0,281672352
ENSMUSG00000036737	Oxsr1	1041,23539	1157,251294	1157,553484	1213,566798	856,5991325	-0,281604131	False	0,281604131
ENSMUSG00000024944	Arl2	1022,393208	928,6821588	1588,066734	1228,161033	841,1756024	-0,281471245	False	0,281471245
ENSMUSG00000028478	Clta	1604,043189	1949,560271	2149,271507	2229,550102	2835,556685	0,28143577	False	0,28143577
ENSMUSG00000029439	Sfswap	1105,134965	2579,565955	1343,157411	2463,057867	3183,179325	0,28140582	False	0,28140582
ENSMUSG00000004044	Cavin1	7481,165682	2787,967226	9092,395916	5276,377381	2270,818199	0,281397424	False	0,281397424
ENSMUSG00000020088	Sar1a	1839,160858	2104,180569	1920,835905	1513,309938	2949,453523	-0,281340174	False	0,281340174
ENSMUSG00000005251	Ripk4	356,363017	301,5575986	293,2322393	367,1011497	475,7565819	-0,281303894	False	0,281303894
ENSMUSG00000006395	Hyi	285,909639	235,2917569	470,0501814	403,0254212	251,5221829	-0,28110848	False	0,28110848
ENSMUSG00000029484	Anxa3	2722,28576	7363,191429	3307,923239	9475,026615	6104,158638	0,281107242	False	0,281107242
ENSMUSG00000032245	Cln6	831,5137064	636,7283054	1010,38824	893,6162543	595,5855464	0,281097803	False	0,281097803
ENSMUSG00000022472	Desi1	874,9326487	1063,134591	863,2229967	747,6739012	675,0760476	0,281080382	False	0,281080382
ENSMUSG00000027649	Ctnnbl1	341,6169612	628,0849347	281,1515104	431,0912583	654,906816	-0,281031809	False	0,281031809
ENSMUSG00000070473	Cldn3	2577,282877	6955,032259	3131,105297	4253,658276	11575,95254	0,280821133	False	0,280821133
ENSMUSG00000038582	Pptc7	815,1291999	1050,649723	671,0295814	978,9363991	1389,304133	-0,280652381	False	0,280652381
ENSMUSG00000058056	Palld	1169,853766	435,0496566	963,1635727	354,7521813	368,9782967	-0,280475467	False	0,280475467
ENSMUSG00000031546	Gins4	280,994287	356,2989462	347,0463956	326,6863442	231,3529513	-0,280445296	False	0,280445296
ENSMUSG00000022270	Fam134b	1388,586928	3341,142948	1143,276259	2896,394392	4045,710584	-0,28044344	False	0,28044344
ENSMUSG00000037993	Dhx38	728,2913153	944,0481511	599,6434557	669,0895572	1623,029935	-0,280410703	False	0,280410703
ENSMUSG00000026788	Zbtb43	322,7747787	455,2175215	265,7760372	368,2237832	824,5656469	-0,280316818	False	0,280316818
ENSMUSG00000028967	Errfi1	1575,370303	5015,075733	1913,148168	6346,247092	6509,916122	0,280257626	False	0,280257626
ENSMUSG00000028243	Ubxn2b	344,0746371	197,8371507	283,3480066	230,1398645	147,1167485	-0,280146498	False	0,280146498
ENSMUSG00000025150	Cbr2	107225,9453	151697,8777	131058,3373	168560,0499	130203,0681	0,28010941	False	0,28010941
ENSMUSG00000036391	Sec24a	319,4978773	274,6671121	288,839247	300,865774	387,9611029	0,280105668	False	0,280105668
ENSMUSG00000031534	Smim19	312,9440747	273,7067376	379,9938382	282,9036383	236,0986529	0,280071164	False	0,280071164
ENSMUSG00000028906	Epb41	3194,978773	3398,765419	2631,402417	5548,054684	3071,655338	-0,279974451	False	0,279974451
ENSMUSG00000026563	Tada1	303,9325961	271,7859886	369,0113574	328,9316112	244,4036306	0,279913811	False	0,279913811
ENSMUSG00000034327	Kctd9	262,1521045	348,61595	251,498812	359,2427153	215,9294212	-0,279844298	False	0,279844298

ENSMUSG00000049504	Proser1	658,6571625	773,1014869	542,5345552	561,3167426	1248,119512	-0,279812695	False	0,279812695
ENSMUSG00000024277	Mapre2	2479,795063	1624,953684	2042,741443	1682,827594	1701,334011	-0,279714289	False	0,279714289
ENSMUSG00000053581	Zfand2a	486,619844	808,6353441	400,8605519	405,2706882	1926,754835	-0,279694702	False	0,279694702
ENSMUSG00000042354	Gnl3	259,6944285	583,9077069	315,1972011	424,3554574	1208,967474	0,279439661	False	0,279439661
ENSMUSG00000038383	Pigu	714,3644847	367,8234404	588,6609749	358,1200818	149,4895993	-0,27922337	False	0,27922337
ENSMUSG00000038526	Car14	467,7776615	106,6015715	385,4850787	171,7629232	55,76199339	-0,27914795	False	0,27914795
ENSMUSG00000030357	Fkbp4	3321,958699	1415,592039	4030,570481	1527,904173	1129,476972	0,278949913	False	0,278949913
ENSMUSG00000008333	Snrpb2	474,3314641	655,9357957	575,4819978	619,6936839	1086,765658	0,278875228	False	0,278875228
ENSMUSG00000005469	Prkaca	1368,106295	1029,521483	1659,45286	1070,992345	823,3792215	0,278527322	False	0,278527322
ENSMUSG00000021978	Extl3	1413,163688	919,0784137	988,4232787	809,4187429	1165,069734	-0,278512276	False	0,278512276
ENSMUSG00000029267	Mtf2	344,0746371	475,3853864	417,3342732	621,9389508	442,5366709	0,278481855	False	0,278481855
ENSMUSG00000004994	Ccdc130	218,7331622	224,7276372	269,0707814	251,4699007	180,3366595	-0,278479262	False	0,278479262
ENSMUSG00000028163	Nfkb1	1839,980083	1447,284399	1405,757552	1296,641675	2231,666161	0,278431078	False	0,278431078
ENSMUSG00000021987	Mtmr6	1196,888202	1205,27002	1105,935824	1451,565096	778,2950567	0,278320872	False	0,278320872
ENSMUSG00000056612	Ppp1r14b	362,9168197	501,3154984	605,1346962	440,0723262	707,1095332	0,27810173	False	0,27810173
ENSMUSG00000016763	Scube1	489,0775199	183,4315329	593,0539672	325,5637107	118,6425391	0,27810024	False	0,27810024
ENSMUSG00000027944	Hax1	539,8694902	373,5856875	654,5558601	514,1661362	207,6244435	0,27790563	False	0,27790563
ENSMUSG00000023495	Pcbp4	697,1607529	845,1295758	885,1879585	948,625295	975,2416716	0,277681199	False	0,277681199
ENSMUSG00000005610	Eif4g2	9584,117095	11109,61242	7906,287981	7937,018741	12406,45032	-0,277645029	False	0,277645029
ENSMUSG00000054580	Pla2r1	425,9971698	117,1656912	351,439388	188,6024255	206,4380181	-0,277567954	False	0,277567954
ENSMUSG00000059436	Max	1495,905446	2202,13877	1813,207592	2126,267821	3363,515984	0,27752512	False	0,27752512
ENSMUSG00000026377	Nifk	312,9440747	346,695201	277,8567661	258,2057016	701,1774062	-0,277384003	False	0,277384003
ENSMUSG00000022629	Kif21a	624,2496988	662,6584174	515,078353	737,5701998	620,5004796	-0,27733131	False	0,27733131
ENSMUSG00000026004	Kansl1l	382,5782275	311,1613438	415,137777	463,6476294	215,9294212	0,277274006	False	0,277274006
ENSMUSG00000033487	Fndc3a	1047,789193	1014,155491	875,3037257	1269,698472	947,9538876	0,27713744	False	0,27713744
ENSMUSG00000025355	Mmp19	293,2826669	290,9934789	405,2535443	360,3653488	242,0307798	-0,277101279	False	0,277101279
ENSMUSG00000027574	Nkain4	358,0014677	32,65273361	433,8079945	246,9793668	58,13484417	0,277091138	False	0,277091138
ENSMUSG00000021782	Dlg5	573,4577286	550,2945988	473,3449257	651,1274214	708,2959586	-0,276795288	False	0,276795288
ENSMUSG00000029213	Commd8	430,9125218	546,4531007	601,8399519	550,0904078	522,0271722	0,276729881	False	0,276729881
ENSMUSG00000049764	Zfp280b	276,8981604	228,5691353	304,2147202	260,4509686	367,7918713	-0,276724866	False	0,276724866
ENSMUSG00000055067	Smyd3	240,0330207	264,1029924	230,6320984	290,7620727	111,5239868	0,276606208	False	0,276606208
ENSMUSG00000035278	Plekhj1	584,9268831	632,8868073	964,2618208	720,7306975	482,8751342	-0,276606123	False	0,276606123
ENSMUSG00000022561	Gpaa1	754,5065257	852,8125719	985,1285344	862,1825167	622,8733304	-0,276594577	False	0,276594577
ENSMUSG00000039234	Sec24d	761,0603284	551,2549733	501,899376	406,3933217	921,852529	0,276525159	False	0,276525159
ENSMUSG00000034872	Gipc3	371,1090729	48,97910041	306,4112164	165,0271223	42,71131409	-0,276374164	False	0,276374164
ENSMUSG00000005687	Bcas2	444,020127	582,9473324	691,8962951	537,7414394	799,6507137	0,276287579	False	0,276287579
ENSMUSG00000026094	Stk17b	766,7949056	889,3068036	729,23673	633,1652857	1205,408198	-0,276258593	False	0,276258593
ENSMUSG00000058392	Rrp1b	296,5595682	373,5856875	359,1271246	371,5916836	851,8534309	0,276172703	False	0,276172703
ENSMUSG00000022450	Ndufa6	812,6715239	1293,624476	984,0302863	1434,725594	1313,372908	0,276030376	False	0,276030376
ENSMUSG00000029238	Clock	1305,84517	1017,996989	928,0196339	1047,417042	1078,460681	-0,276010273	False	0,276010273
ENSMUSG00000062014	Gmfb	1640,908329	1178,379534	1355,23814	1166,416191	736,770168	-0,275948262	False	0,275948262
ENSMUSG00000013089	Etv5	1722,011636	888,3464291	1422,231273	1053,030209	419,9945885	-0,275938807	False	0,275938807
ENSMUSG00000041966	Dcaf17	302,2941455	284,2708573	350,3411399	365,9785162	144,7438977	0,275805923	False	0,275805923
ENSMUSG00000058440	Nrf1	405,5165366	711,6375178	490,9168951	550,0904078	969,3095447	0,275718071	False	0,275718071
ENSMUSG00000044562	Rasip1	3066,360397	6135,832795	3712,078535	3837,161253	5486,031009	0,275699963	False	0,275699963
ENSMUSG00000030852	Tacc2	1173,949893	1454,00702	969,7530612	1175,397259	2495,052598	-0,275681502	False	0,275681502
ENSMUSG00000049907	Rasl11b	280,994287	910,435043	361,3236208	340,157946	1034,562941	0,275663992	False	0,275663992
ENSMUSG00000022781	Pak2	1722,830862	1423,275036	1326,68369	1075,482879	837,6163262	-0,275566595	False	0,275566595
ENSMUSG00000041263	Rusc1	253,1406259	429,2874095	306,4112164	401,9027877	461,5194772	0,275530049	False	0,275530049
ENSMUSG00000031089	Slc6a14	866,7403955	2359,640191	716,057753	1422,376626	3126,230906	-0,275523993	False	0,275523993
ENSMUSG00000041741	Pde3a	1528,674459	1054,491221	1262,985301	1101,303449	934,9032083	-0,27544336	False	0,27544336
ENSMUSG00000007815	Rhoa	9773,358146	6618,901178	7881,028275	6174,484169	8074,811213	-0,27542583	False	0,27542583
ENSMUSG00000055809	Dnaaf3	412,0703392	543,5719772	340,4569071	454,6665615	471,0108803	-0,27541842	False	0,27541842
ENSMUSG00000032582	Rbm6	984,7088425	1191,824777	1217,957129	1334,811214	2070,312308	0,275403027	False	0,275403027
ENSMUSG00000039672	Kcne2	260,5136538	361,1008187	215,2566251	255,9604346	412,8760362	-0,275301347	False	0,275301347
ENSMUSG00000055024	Ep300	1889,952828	1973,569634	1561,70878	1917,457993	2374,037208	-0,275224774	False	0,275224774
ENSMUSG00000062421	Arf2	548,8809688	1056,41197	453,5764601	580,4015119	1678,791929	-0,275147549	False	0,275147549
ENSMUSG00000014668	Chfr	462,8623095	1150,528672	560,1065246	1288,783241	829,3113485	0,275118144	False	0,275118144
ENSMUSG00000037740	Mrps26	294,1018922	426,406286	377,7973421	355,8748148	639,4832859	0,275053771	False	0,275053771
ENSMUSG00000041120	Nbl1	4588,481054	5847,72044	4104,153103	3792,255913	7767,527037	-0,274960326	False	0,274960326
ENSMUSG00000038859	Baiap2l1	901,1478592	810,5560931	743,5139552	623,0615843	1090,324935	0,2749224	False	0,2749224
ENSMUSG00000030629	Zfand6	1194,430526	987,2650044	1436,508498	1214,689431	1166,25616	-0,274813648	False	0,274813648
ENSMUSG00000030753	Thap12	1099,400388	1065,05534	750,1034437	770,1265709	1329,982864	0,274690763	False	0,274690763
ENSMUSG00000031347	Cetn2	561,988574	235,2917569	464,558941	303,111041	233,7258021	-0,274679148	False	0,274679148
ENSMUSG00000067928	Zfp760	276,8981604	83,55258306	334,9656667	142,5744526	16,60995548	0,274657761	False	0,274657761
ENSMUSG00000027375	Mal	502,1851252	867,2181897	415,137777	528,7603716	951,5131638	-0,274629075	False	0,274629075
ENSMUSG00000040613	Apobec1	475,1506894	392,7931778	332,7691705	388,4311859	313,2163033	-0,27461525	False	0,27461525
ENSMUSG00000005533	Igf1r	1509,013051	1968,767762	1825,288321	2518,066907	2639,796496	0,274519085	False	0,274519085
ENSMUSG00000046275	Tusc5	185,9641491	318,84434	153,7547322	315,4600094	226,6072497	-0,274393706	False	0,274393706
ENSMUSG00000047123	Ticam1	377,6628755	284,2708573	325,0814339	324,4410772	456,7737756	0,274380852	False	0,274380852
ENSMUSG00000006169	Clint1	1588,477908	1721,951511	1313,504713	1484,121467	2457,086985	-0,274223649	False	0,274223649
ENSMUSG00000031948	Kars	968,324336	1022,798862	821,4895694	854,3240823	1171,001861	0,274181111	False	0,274181111
ENSMUSG00000059734	Ndufs8	675,0416691	816,3183402	741,317459	603,9768151	511,3493436	0,274155311	False	0,274155311
ENSMUSG00000039782	Cpeb2	237,5753447	225,6880117	269,0707814	196,4608599	405,7574838	-0,274143204	False	0,274143204
ENSMUSG00000057789	Bak1	1315,675874	1113,074066	820,3913213	799,3150415	1590,99645	0,274126501	False	0,274126501
ENSMUSG00000031149	Praf2	250,6829499	143,0958032	303,1164721	165,0271223	240,8443544	0,274008379	False	0,274008379
ENSMUSG00000036860	Mrpl55	208,9024583	309,2405948	252,5970601	255,9604346	437,7909694	0,274008379	False	0,274008379
ENSMUSG00000032846	Zswim6	493,9928719	654,9754212	408,5482885	471,5060638	882,7004911	-0,273983616	False	0,273983616
ENSMUSG00000048100	Taf13	178,5911212	284,2708573	264,6777891	262,6962355	215,9294212	0,273899472	False	0,273899472
ENSMUSG00000024610	Cd74	23430,66356	8458,018379	9517,417926	6305,832287	19379,07234	-0,273898301	False	0,273898301
ENSMUSG00000058006	Mdn1	317,0402014	215,123892	383,2885825	194,2155929	250,3357576	0,273765235	False	0,273765235
ENSMUSG00000029922	Mkrn1	1563,901148	2099,378696	1293,736247	1801,826744	2785,726819	-0,273605799	False	0,273605799
ENSMUSG00000003068	Stk11	2175,043242	3107,77194	2629,205921	3013,148274	6092,294384	0,273583056	False	0,273583056
ENSMUSG00000026159	Agfg1	1726,107763	1247,526499	1259,690556	1427,989793	1421,337619	-0,273536869	False	0,273536869
ENSMUSG00000034566	Atp5h	1373,840873	1974,530009	1660,551108	1747,940337	2350,3087	0,273447215	False	0,273447215
ENSMUSG00000074247	Dda1	989,6241945	1651,844171	1195,992167	1435,848228	2357,427252	0,273255264	False	0,273255264
ENSMUSG00000057666	Gapdh	7272,263224	14447,87425	8788,181196	9469,413448	18047,90305	0,273160196	False	0,273160196
ENSMUSG00000039021	Ttc16	512,8350544	415,8421663	535,9450667	619,6936839	214,7429958	0,273060384	False	0,273060384
ENSMUSG00000071847	Apcdd1	226,9254155	348,61595	187,8004229	227,8945975	285,9285193	-0,273017887	False	0,273017887
ENSMUSG00000051989	Smim11	281,8135123	378,3875601	551,3205399	391,7990864	340,5040873	0,272931329	False	0,272931329
ENSMUSG00000024695	Zfp91	1584,381782	2592,050824	1914,246416	2498,982138	2953,012799	0,272856539	False	0,272856539
ENSMUSG00000027933	Ints3	1064,173699	416,8025408	880,7949661	579,2788784	204,0651673	-0,272855524	False	0,272855524
ENSMUSG00000045216	Hs6st1	875,7518741	1927,471657	724,8437377	1042,926508	4218,928691	-0,272852159	False	0,272852159
ENSMUSG00000048109	Rbm15	132,7145029	275,6274866	160,3442208	212,1777287	596,7719718	0,272846321	False	0,272846321
ENSMUSG00000030649	Anapc15	200,710205	332,2895832	292,1339913	242,4888328	404,5710584	0,272804338	False	0,272804338
ENSMUSG00000029462	Vps29	772,5294829	801,9127225	881,8932142	857,6919827	639,4832859	-0,272683341	False	0,272683341
ENSMUSG00000003429	Rps11	3685,694744	6441,231892	4452,297746	5769,213481	7824,475455	0,272613487	False	0,272613487
ENSMUSG00000062270	Morf4l1	3222,013209	4189,153647	2667,644604	3384,739958	6267,885342	-0,272395932	False	0,272395932
ENSMUSG00000047844	Bex4	212,1793596	345,7348265	430,5132503	638,7784531	256,2678845	0,272368371	False	0,272368371
ENSMUSG00000026574	Dpt	1500,001573	1268,654738	1070,791885	941,8894941	1811,671572	0,272357428	False	0,272357428
ENSMUSG00000052299	Ltn1	640,6342054	436,0100311	530,4538262	508,5529688	372,5375729	-0,272273654	False	0,272273654
ENSMUSG00000044080	S100a1	1980,88684	2392,292924	2741,227226	2906,498093	2565,051696	0,272247584	False	0,272247584
ENSMUSG00000059005	Hnrnpa3	3596,399183	2978,12138	2759,897444	2862,715387	2578,102375	-0,272150607	False	0,272150607
ENSMUSG00000024222	Fkbp5	222,8292888	4413,881284	269,0707814	4324,384185	3910,45809	0,272046862	False	0,272046862
ENSMUSG00000025498	Irf7	611,1420936	506,117371	869,8124852	527,6377381	828,1249231	-0,272035862	False	0,272035862
ENSMUSG00000037926	Ssh2	2316,769223	3954,822265	1918,639409	3659,785162	4494,179382	-0,272030747	False	0,272030747
ENSMUSG00000061778	Mospd2	820,8637772	451,3760234	679,8155661	654,4953219	202,8787419	-0,271999429	False	0,271999429
ENSMUSG00000001054	Rmnd5b	654,5610359	894,1086762	810,5070885	790,3339736	1156,764756	0,2719347	False	0,2719347
ENSMUSG00000036985	Zdhhc9	966,6858853	609,8378189	800,6228557	629,7973852	503,0443659	-0,271924375	False	0,271924375
ENSMUSG00000017776	Crk	1703,169454	1923,630159	1740,723219	1865,816852	1410,65979	-0,271851886	False	0,271851886

ENSMUSG00000055368	Slc6a2	2297,107816	1235,04163	2773,076421	2598,896518	626,4326066	0,271668803	False	0,271668803
ENSMUSG00000024561	Mbd1	1045,331517	3534,178226	1261,887052	2457,444699	4048,083435	0,271622234	False	0,271622234
ENSMUSG00000046822	Slc39a3	791,3716654	586,7888305	655,6541082	452,4212946	424,7402901	-0,271420493	False	0,271420493
ENSMUSG00000030894	Tpp1	3411,25426	1969,728136	4117,33208	3311,768781	1959,974746	0,271407522	False	0,271407522
ENSMUSG00000036678	Aaas	339,1592852	503,2362474	430,5132503	489,4681996	409,31676	0,271254751	False	0,271254751
ENSMUSG00000015671	Psma2	820,8637772	1638,398928	990,6197749	1023,841739	2187,768421	0,271188595	False	0,271188595
ENSMUSG00000020811	Wscd1	883,9441273	193,0352781	732,5314743	387,3085524	109,151136	-0,271064434	False	0,271064434
ENSMUSG00000049299	Trappc1	737,3027939	930,6029079	909,3494164	763,39077	611,0090765	-0,271063412	False	0,271063412
ENSMUSG00000061479	Snrpa	1080,558206	1065,05534	1080,676118	749,9191681	1303,881505	0,271035978	False	0,271035978
ENSMUSG00000042350	Arel1	693,0646262	343,8140774	574,3837497	465,8928964	157,794577	-0,270974952	False	0,270974952
ENSMUSG00000056076	Eif3b	2573,186751	5915,90703	3104,747343	4626,372593	8054,641981	0,270919705	False	0,270919705
ENSMUSG00000024963	Dnajc4	319,4978773	508,03812	385,4850787	406,3933217	559,9927847	0,270868671	False	0,270868671
ENSMUSG00000032571	Pik3r4	637,357304	519,5626142	528,25733	469,2607968	482,8751342	-0,270861497	False	0,270861497
ENSMUSG00000004285	Atp6v1f	1767,888255	2487,370001	2512,791624	2133,003622	3401,481597	0,270859328	False	0,270859328
ENSMUSG00000072596	Ear2	869,1980715	290,9934789	720,4507453	474,8739643	230,1665259	-0,270785172	False	0,270785172
ENSMUSG00000020691	Mettl2	234,2984434	211,2823939	332,7691705	194,2155929	328,6398334	-0,270688335	False	0,270688335
ENSMUSG00000028914	Casp9	446,477803	244,8955021	370,1096055	314,3373759	137,6253454	-0,270635874	False	0,270635874
ENSMUSG00000062908	Acadm	760,241103	592,5510776	917,037153	856,5693492	441,3502455	0,270523157	False	0,270523157
ENSMUSG00000022043	Trim35	1895,687406	815,3579657	1571,593013	1035,068073	705,9231078	-0,270493428	False	0,270493428
ENSMUSG00000043067	Dpy19l1	435,8278737	338,0518303	361,3236208	284,0262718	277,6235416	-0,270466897	False	0,270466897
ENSMUSG00000020610	Amz2	604,588291	428,327035	729,23673	456,9118285	227,7936751	0,27043419	False	0,27043419
ENSMUSG00000020219	Timm13	355,5437917	509,958869	512,8818568	428,8459914	438,9773948	0,27043237	False	0,27043237
ENSMUSG00000020211	Sf3a2	1069,908276	1819,909711	1290,441503	1361,754418	2943,521396	0,270377625	False	0,270377625
ENSMUSG00000030655	Smg1	1762,153677	2125,308808	2248,113835	2347,426618	2426,239925	0,270332733	False	0,270332733
ENSMUSG00000071267	Zfp942	230,2023168	337,0914558	325,0814339	284,0262718	277,6235416	0,270227556	False	0,270227556
ENSMUSG00000055319	Sec23ip	740,5796952	602,1548227	594,1522153	614,0805164	448,4687979	-0,270227165	False	0,270227165
ENSMUSG00000024644	Cndp2	1305,025945	1994,697874	1573,789509	1597,50745	2591,153055	0,270164107	False	0,270164107
ENSMUSG00000004462	Tbccd1	238,39457	169,9862897	197,6846557	181,8666246	154,2353009	-0,270150482	False	0,270150482
ENSMUSG00000028763	Hspg2	3034,410609	1625,914059	2160,253988	1686,195495	2516,408255	-0,270050321	False	0,270050321
ENSMUSG00000023393	Slc17a9	154,8335867	400,476174	186,7021749	331,1768781	753,3801234	0,270020277	False	0,270020277
ENSMUSG00000033396	Spg11	448,9354789	246,8162511	372,3061016	340,157946	177,9638087	-0,270018855	False	0,270018855
ENSMUSG00000092341	Malat1	22227,22156	19880,7129	21558,60996	33827,19218	18434,67773	-0,269905425	False	0,269905425
ENSMUSG00000028138	Adh5	572,6385032	768,2996143	769,8719093	690,4195934	690,4995777	0,269848729	False	0,269848729
ENSMUSG00000029290	Zfp326	349,8092144	581,0265833	421,7272656	559,0714757	796,0914375	0,269742006	False	0,269742006
ENSMUSG00000027994	Mcub	253,1406259	143,0958032	208,6671366	129,1028508	209,9972943	-0,269568321	False	0,269568321
ENSMUSG00000074749	Kiz	516,931181	276,5878612	412,9412809	428,8459914	309,6570271	-0,269512592	False	0,269512592
ENSMUSG00000034485	Uaca	616,8766709	432,1685331	511,7836087	486,1002991	447,2823725	-0,269448145	False	0,269448145
ENSMUSG00000098112	Bin2	685,6915983	468,6627648	568,8925093	486,1002991	227,7936751	-0,26940376	False	0,26940376
ENSMUSG00000074797	Itpa	259,6944285	458,0986451	313,0007049	343,5258465	480,5022835	0,269350844	False	0,269350844
ENSMUSG00000044367	Slc16a13	252,3214006	209,3616449	198,7829038	139,2065522	128,1339423	-0,269265409	False	0,269265409
ENSMUSG00000027079	Clp1	162,2066147	271,7859886	195,4881596	251,4699007	391,5203791	0,269248576	False	0,269248576
ENSMUSG00000040562	Gstm2	5856,641859	3408,369164	4349,062426	4150,375995	4859,598403	-0,26923658	False	0,26923658
ENSMUSG00000022673	Mcm4	351,4476651	360,1404442	295,4287355	218,9135296	423,5538647	0,269235583	False	0,269235583
ENSMUSG00000002804	Nudt14	316,220976	230,4898843	381,0920863	284,0262718	163,726704	0,26920658	False	0,26920658
ENSMUSG00000002728	Naa20	462,0430842	740,4487533	605,1346962	556,8262087	1379,81273	0,269199733	False	0,269199733
ENSMUSG00000041974	Spidr	271,9828084	225,6880117	177,9161902	204,3192943	163,726704	-0,269185678	False	0,269185678
ENSMUSG00000050288	Fzd2	533,3156876	155,5806719	442,5939792	261,5736021	117,4561137	-0,26900594	False	0,26900594
ENSMUSG00000038271	Iffo1	497,2697732	760,6166182	671,0295814	992,408001	599,1448226	0,268876028	False	0,268876028
ENSMUSG00000024824	Rad9a	453,0316056	324,6065871	545,8292994	428,8459914	288,3013701	0,268838136	False	0,268838136
ENSMUSG00000039771	Polr2j	535,7733636	762,5373672	747,9069475	645,514254	1245,746661	0,268826095	False	0,268826095
ENSMUSG00000018411	Mapt	4504,100845	805,7542205	5426,4438	2293,54021	736,770168	0,268767914	False	0,268767914
ENSMUSG00000033809	Alg3	428,4548458	356,2989462	516,1766011	239,1209324	480,5022835	0,268721575	False	0,268721575
ENSMUSG00000052974	Cyp2f2	113804,3247	59703,60266	94465,80924	79082,79323	86134,48341	-0,26869122	False	0,26869122
ENSMUSG00000052684	Jun	3811,036219	3946,178894	3341,96893	3163,581161	6643,982191	-0,268624711	False	0,268624711
ENSMUSG00000019254	Ppp1r12c	1866,195294	2979,081755	2248,113835	2824,545849	4296,046342	0,26861512	False	0,26861512
ENSMUSG00000026974	Zmynd19	212,9985849	338,0518303	176,8179421	189,725059	537,4507022	-0,26857917	False	0,26857917
ENSMUSG00000036112	Metap2	1592,574035	1774,772109	1322,290697	1762,534572	2515,221829	-0,268321062	False	0,268321062
ENSMUSG00000074923	Pak6	126,9799256	1012,234742	105,4318164	446,8081271	1758,28243	-0,26829014	False	0,26829014
ENSMUSG00000027598	Itch	1056,800671	1240,803877	877,5002218	1053,030209	1316,932184	-0,268231893	False	0,268231893
ENSMUSG00000004665	Cnn2	6289,192832	2817,738836	5222,169656	3648,558827	2249,462542	-0,268225542	False	0,268225542
ENSMUSG00000047242	Taf9b	477,6083654	396,6346759	302,018224	304,2336745	281,1828177	-0,268017295	False	0,268017295
ENSMUSG00000061032	Rrp1	2003,005923	2641,990299	2411,7528	2853,734319	4160,793847	0,267915354	False	0,267915354
ENSMUSG00000020264	Slc36a2	619,3343469	103,7204479	745,7104514	284,0262718	72,37194887	0,267897105	False	0,267897105
ENSMUSG00000036585	Fgf1	5259,426596	3614,849686	4368,830892	3482,409071	3065,723211	-0,267658257	False	0,267658257
ENSMUSG00000026672	Optn	280,994287	643,450927	338,2604109	464,7702629	728,4651902	0,267593539	False	0,267593539
ENSMUSG00000027170	Eif3m	722,556738	999,7498732	869,8124852	1007,002236	1165,069734	0,267593539	False	0,267593539
ENSMUSG00000024286	Ccny	1581,10488	2167,565287	1313,504713	2137,494156	2670,643556	-0,267511693	False	0,267511693
ENSMUSG00000039865	Slc44a3	965,0474347	548,3738497	801,7211038	737,5701998	568,2977624	-0,267499405	False	0,267499405
ENSMUSG00000003541	Ier3	3493,176792	4566,580833	3626,415185	2902,007559	8714,294499	-0,267488383	False	0,267488383
ENSMUSG00000027601	Mtfr1	368,6513969	357,2593207	298,7234798	260,4509686	443,7230963	0,267402425	False	0,267402425
ENSMUSG00000033434	Gtpbp6	429,2740711	516,6814906	605,1346962	550,0904078	880,3276403	0,267376168	False	0,267376168
ENSMUSG00000026349	Ccnt2	1228,018764	1478,016383	1765,982925	1869,184753	1829,467953	0,267329655	False	0,267329655
ENSMUSG00000025939	Ube2w	328,5093559	280,4293592	395,3693115	291,8847062	332,1991096	0,267266434	False	0,267266434
ENSMUSG00000075254	Heg1	4771,168302	3675,35328	3964,675596	3702,445234	3294,703312	-0,267139754	False	0,267139754
ENSMUSG00000024451	Arap3	6027,040727	5008,353111	8041,372496	6342,879192	5025,697957	-0,267113532	False	0,267113532
ENSMUSG00000071253	Slc25a16	461,2238588	543,5719772	383,2885825	396,2896203	662,0253683	-0,267036121	False	0,267036121
ENSMUSG00000031931	Ankrd49	244,1291473	321,7254635	220,7478656	271,6773034	202,8787419	-0,267026849	False	0,267026849
ENSMUSG00000070493	Chchd2	1061,716023	1823,75121	1425,526017	1277,556906	2180,649869	0,266989614	False	0,266989614
ENSMUSG00000035539	Ccdc180	584,1076578	328,4480851	485,4256546	394,0443533	393,8932299	-0,266983942	False	0,266983942
ENSMUSG00000060063	Alox5ap	1487,713193	4178,589528	1790,144382	3810,218049	3855,882522	0,266979526	False	0,266979526
ENSMUSG00000037709	Fam13a	959,3128574	443,6930273	797,3281115	775,7397383	405,7574838	-0,266827858	False	0,266827858
ENSMUSG00000052160	Pld4	1368,925521	568,5417146	1137,785019	634,2879192	440,1638202	-0,266815966	False	0,266815966
ENSMUSG00000025512	Chid1	409,6126633	59,54322011	340,4569071	78,58434397	58,13484417	-0,266788115	False	0,266788115
ENSMUSG00000043909	Trp53bp1	661,9340638	385,1101817	550,2222918	364,8558827	316,7755795	-0,266672925	False	0,266672925
ENSMUSG00000006333	Rps9	5042,331885	7182,64102	6065,624187	7514,90855	8938,528898	0,266565035	False	0,266565035
ENSMUSG00000001674	Ddx18	464,5007601	477,3061354	481,0326623	386,1859189	746,2615711	-0,266385396	False	0,266385396
ENSMUSG00000089715	Cbx6	2945,115049	2966,596886	3586,878254	3896,660827	2448,782008	-0,266259643	False	0,266259643
ENSMUSG00000029859	Epha1	453,0316056	320,765089	376,699094	288,5168057	372,5375729	-0,266199139	False	0,266199139
ENSMUSG00000022035	Ccdc25	246,5868233	429,2874095	296,5269836	345,7711135	592,0262702	0,266067685	False	0,266067685
ENSMUSG00000024039	Cbs	190,8795011	256,4199963	229,5338503	546,7225073	265,7592876	0,266044752	False	0,266044752
ENSMUSG00000033781	Asb13	580,0115312	352,4574481	697,3875355	537,7414394	154,2353009	0,265878996	False	0,265878996
ENSMUSG00000058325	Dock1	1064,179699	742,3695024	810,5070885	716,2401636	885,0733419	-0,265864739	False	0,265864739
ENSMUSG00000028086	Fbxw7	436,647099	564,7002165	524,9625858	546,7225073	842,3620278	0,26574685	False	0,26574685
ENSMUSG00000045294	Insig1	936,3745482	778,863734	573,2855016	684,806426	620,5004796	-0,265714777	False	0,265714777
ENSMUSG00000012429	Mplkip	277,7173857	136,3731815	333,8674186	165,0271223	51,01629182	0,265657815	False	0,265657815
ENSMUSG00000056698	Elmod3	579,1923059	697,2319	696,2892874	755,5323356	1795,061617	0,26564439	False	0,26564439
ENSMUSG00000035268	Pkig	914,2554644	1453,046646	1389,283831	1099,058182	1632,521338	0,265598512	False	0,265598512
ENSMUSG00000031539	Ap3m2	479,246816	216,0842665	398,6640557	249,2246337	164,9131294	-0,265595312	False	0,265595312
ENSMUSG00000052889	Prkcb	390,7704808	227,6087608	325,0814339	230,1398645	265,7592876	-0,265520326	False	0,265520326
ENSMUSG00000030083	Abtb1	800,383144	1535,638854	962,0653246	1801,826744	1572,013643	0,265444072	False	0,265444072
ENSMUSG00000001555	Fkbp10	459,5854082	634,8075564	552,418788	751,0418016	678,6353238	0,265429394	False	0,265429394
ENSMUSG00000001036	Epn2	2375,753447	2239,593376	2220,657633	3156,845361	1976,584702	-0,265375343	False	0,265375343
ENSMUSG00000041671	Pyroxd1	190,0602758	259,3011198	228,4356022	287,3941722	325,0805572	0,265330488	False	0,265330488
ENSMUSG00000046432	Bex3	514,4735051	783,6656066	618,3136732	658,9858558	1039,308643	0,265242128	False	0,265242128
ENSMUSG00000040943	Tet2	545,6040675	657,8565448	655,6541082	854,3240823	686,9403015	0,265080519	False	0,265080519
ENSMUSG00000034707	Gns	3836,432204	4175,708404	3192,60719	3511,597542	5471,793904	-0,265030202	False	0,265030202
ENSMUSG00000036052	Dnajb5	658,6571625	1090,025078	824,7843137	791,4566071	943,208186	0,264982531	False	0,264982531
ENSMUSG00000032295	Man2c1	1105,954191	1656,646043	1328,880186	1772,638273	2147,429958	0,264919405	False	0,264919405
ENSMUSG00000025762	Larp1b	371,1090729	973,8197612	445,8887235	822,8903447	1038,122217	0,264840442	False	0,264840442
ENSMUSG00000026234	Ncl	2880,396248	4526,245103	2397,475575	3860,736556	5337,727835	-0,264751176	False	0,264751176

ENSMUSG00000029209	Gnpda2	239,2137953	226,6483862	251,498812	287,3941722	177,9638087	0,264730216	False	0,264730216
ENSMUSG00000025227	Mfsd13a	299,8364695	178,6296603	360,2253727	273,9225704	149,4895993	0,264723931	False	0,264723931
ENSMUSG00000009545	Kcnq1	538,2310395	430,247784	369,0113574	305,356308	646,6018382	0,264652016	False	0,264652016
ENSMUSG00000038143	Stox2	477,6083654	276,5878612	397,5658076	282,9036383	283,5556685	-0,264634421	False	0,264634421
ENSMUSG00000049892	Rasd1	63,08035014	1177,419159	75,77911803	290,7620727	3012,334068	0,264609681	False	0,264609681
ENSMUSG00000063802	Hspbp1	571,8192779	828,803209	788,5421268	475,9965977	1093,884211	-0,264607997	False	0,264607997
ENSMUSG00000024736	Tmem132a	330,9670319	99,87894987	397,5658076	115,631249	88,98190434	0,264506169	False	0,264506169
ENSMUSG00000048249	Crebrf	1448,390377	1494,34275	1205,8764	1955,627531	1287,27155	-0,264368456	False	0,264368456
ENSMUSG00000026213	Stk11ip	724,1951887	1029,521483	869,8124852	977,8137656	1201,848921	0,264325825	False	0,264325825
ENSMUSG00000021457	Syk	1622,066147	1260,971742	1264,083549	963,2195303	1948,110492	0,264242853	False	0,264242853
ENSMUSG00000029650	Slc46a3	456,3085069	446,5741508	548,0257956	514,1661362	181,5230849	0,264234253	False	0,264234253
ENSMUSG00000029020	Mfn2	1320,591226	1099,628823	1056,51466	996,8985349	990,6652017	-0,264167338	False	0,264167338
ENSMUSG00000030659	Nucb2	644,730332	781,7448576	774,2649016	849,8335483	887,4461927	0,264131386	False	0,264131386
ENSMUSG00000052928	Ctif	480,0660414	282,3501083	399,7623038	352,5069144	213,5565704	-0,26409045	False	0,26409045
ENSMUSG00000000355	Mcts1	290,8249909	194,9560271	349,2428918	314,3373759	112,7104122	0,264079506	False	0,264079506
ENSMUSG00000072501	Phf20l1	1531,951361	2716,899511	1839,565546	2932,318663	2296,919557	0,26399459	False	0,26399459
ENSMUSG00000063019	Manbal	582,4692072	955,5726453	734,7279705	699,4006613	1441,50685	0,263937378	False	0,263937378
ENSMUSG00000049164	Zfp518a	307,2094975	246,8162511	255,8918044	180,7439911	128,1339423	-0,263688876	False	0,263688876
ENSMUSG00000039115	Itga9	1470,509461	1326,277209	855,5352601	1271,943739	1765,400982	0,263679839	False	0,263679839
ENSMUSG00000073490	Ifi207	253,1406259	338,0518303	210,8636328	387,3085524	424,7402901	-0,263628763	False	0,263628763
ENSMUSG00000004383	Large1	409,6126633	491,7117532	354,7341322	335,6674121	296,6063478	0,263552522	False	0,263552522
ENSMUSG00000067203	H2-K2	285,909639	238,1728804	482,1309104	356,9974483	265,7592876	-0,263550109	False	0,263550109
ENSMUSG00000032727	Mier3	376,0244249	630,0056838	451,3799639	542,2319734	747,4479965	0,263516005	False	0,263516005
ENSMUSG00000026617	Bpnt1	465,3199855	352,4574481	387,6815749	300,865774	262,2000115	-0,263350982	False	0,263350982
ENSMUSG00000021240	Abcd4	258,0559779	122,9279383	309,7059607	220,0361631	61,69412035	0,263215099	False	0,263215099
ENSMUSG00000048277	Syngr2	2911,526811	3222,056508	2426,030025	2467,548401	3698,087945	-0,263178498	False	0,263178498
ENSMUSG00000070509	Rgma	580,8307565	484,028757	506,2923683	665,7216568	679,8217492	-0,263025087	False	0,263025087
ENSMUSG00000059981	Taok2	4223,925784	3177,87928	5068,414923	4656,683697	2827,251707	0,262950149	False	0,262950149
ENSMUSG00000028223	Decr1	643,0918813	269,8652395	535,9450667	339,0353125	226,6072497	-0,262939742	False	0,262939742
ENSMUSG00000038170	Pde4dip	491,5351959	362,0611933	409,6465366	334,5447786	306,0977509	-0,262915106	False	0,262915106
ENSMUSG00000028530	Jak1	3552,980241	3337,30145	2294,240255	2438,35993	4262,826431	0,262780649	False	0,262780649
ENSMUSG00000015846	Rxra	2224,196762	2058,082592	1853,842772	1987,061269	3040,808278	-0,262765529	False	0,262765529
ENSMUSG00000024395	Lims2	2525,671682	4200,678141	3334,281193	5150,64243	3030,130449	0,262712792	False	0,262712792
ENSMUSG00000028382	Ptbp3	2596,944285	2601,654569	1935,11313	2324,973948	3115,553077	0,262673233	False	0,262673233
ENSMUSG00000028832	Stmn1	880,667226	179,5900349	1056,51466	517,5340367	281,1828177	0,262643904	False	0,262643904
ENSMUSG00000000827	Tpd52l2	816,7676506	1248,486873	979,637294	1059,76601	1524,556628	0,26232197	False	0,26232197
ENSMUSG00000029482	Aacs	433,3701977	271,7859886	361,3236208	226,771964	163,726704	-0,262308374	False	0,262308374
ENSMUSG00000031974	Abcb10	335,8823839	202,6390233	280,0532623	241,3661993	157,794577	-0,262254898	False	0,262254898
ENSMUSG00000014873	Surf2	297,3787935	369,7441894	445,8887235	450,1760276	247,9629068	-0,262177449	False	0,262177449
ENSMUSG00000000605	Clcn4	1694,157975	1203,349271	2031,758962	1558,215278	1067,782852	0,26216085	False	0,26216085
ENSMUSG00000028024	Enpep	1406,609886	426,406286	1172,928957	580,4015119	503,0443659	-0,262106628	False	0,262106628
ENSMUSG00000030447	Cyfip1	1785,911212	1439,601402	1489,224407	1452,68773	1103,375614	-0,262099192	False	0,262099192
ENSMUSG00000029345	Tfip11	667,6686411	842,2484522	556,8117803	669,0895572	1168,62901	-0,261942547	False	0,261942547
ENSMUSG00000033014	Trim33	741,3989205	773,1014869	618,3136732	755,5323356	961,0045669	-0,261911105	False	0,261911105
ENSMUSG00000028603	Scp2	2304,480844	1724,832634	2763,192188	2313,747613	1613,538532	0,261894134	False	0,261894134
ENSMUSG00000031715	Smarca5	828,2368051	1074,659086	690,798047	876,776752	1261,170191	-0,26177931	False	0,26177931
ENSMUSG00000020668	Kif3c	448,9354789	167,1051661	374,5025978	252,5925342	79,49050121	-0,261532389	False	0,261532389
ENSMUSG00000015745	Plekho1	1459,859532	1749,802372	2565,507532	2012,881839	2167,59919	0,261362429	False	0,261362429
ENSMUSG00000020056	Washc3	280,994287	264,1029924	333,8674186	336,7900456	156,6081516	0,261308699	False	0,261308699
ENSMUSG00000026728	Vim	12321,96814	4149,778292	10280,70035	6422,586169	6919,232882	-0,261294162	False	0,261294162
ENSMUSG00000002897	Il17ra	1063,354474	1768,049488	887,3844546	1021,596472	3051,486106	-0,260991421	False	0,260991421
ENSMUSG00000057572	Zbtb8os	241,6714713	253,5388727	329,4744262	277,2904709	201,6923165	-0,260891051	False	0,260891051
ENSMUSG00000026158	Ogfrl1	601,3113897	814,3975912	720,4507453	1236,019467	1102,189188	0,260787516	False	0,260787516
ENSMUSG00000046410	Kcnk6	311,3056241	206,4805214	254,7935563	209,9324617	259,8271607	-0,260779392	False	0,260779392
ENSMUSG00000031774	Fam192a	593,1191364	796,1504754	807,2123443	710,6269962	723,7194887	0,260770577	False	0,260770577
ENSMUSG00000040940	Arhgef1	4773,625978	5917,82778	5718,577791	6411,359834	7319,058239	0,260570861	False	0,260570861
ENSMUSG00000034158	Lrrc58	1162,480738	2646,792171	1392,578575	2548,378012	3223,517788	0,260551921	False	0,260551921
ENSMUSG00000096210	H1f0	2583,83668	1391,582676	2156,959244	1398,801323	1383,372006	-0,260515966	False	0,260515966
ENSMUSG00000089876	Tmem102	176,9526705	262,1822434	211,9618809	258,2057016	281,1828177	0,2604413	False	0,2604413
ENSMUSG00000038213	Tapbpl	553,7963207	460,9797686	462,3624448	401,9027877	414,0624615	-0,260331249	False	0,260331249
ENSMUSG00000033004	Mycbp2	1246,041722	1492,422001	1546,333307	1494,225169	1787,943065	0,260303158	False	0,260303158
ENSMUSG00000073633	Fbxo36	373,5667489	412,9610427	311,9024568	417,6196565	434,2316932	-0,26027113	False	0,26027113
ENSMUSG00000001016	Ilf2	798,7446934	1190,864402	956,5740841	1066,501811	1492,523142	0,260142265	False	0,260142265
ENSMUSG00000024845	Tmem134	1190,334399	1600,944321	1425,526017	1466,159332	1679,978354	0,260127445	False	0,260127445
ENSMUSG00000030787	Lyve1	4975,974633	43353,22649	5959,094122	12940,59618	64691,03088	0,260113904	False	0,260113904
ENSMUSG00000078816	Prkcg	230,2023168	387,0309307	275,6602699	355,8748148	843,5484532	0,259988998	False	0,259988998
ENSMUSG00000024095	Hnrnpll	728,2913153	976,7008847	872,0089814	1141,718254	872,0226626	0,259827353	False	0,259827353
ENSMUSG00000060681	Slc9a6	788,9139895	515,7211161	658,9488525	505,1850684	522,0271722	-0,259701533	False	0,259701533
ENSMUSG00000009079	Ewsr1	2619,882594	3581,236577	3136,596538	3895,538194	5573,826488	0,259697805	False	0,259697805
ENSMUSG00000009995	Taz	865,1019448	578,1454598	1035,647946	921,6820914	313,2163033	0,259591606	False	0,259591606
ENSMUSG00000019143	Hars2	313,7633001	398,5554249	375,6008459	454,6665615	410,5031854	0,259523698	False	0,259523698
ENSMUSG00000021892	Sh3bp5	1761,334452	1110,192943	1499,108639	1011,49277	2108,27792	0,259396178	False	0,259396178
ENSMUSG00000075467	Dnlz	481,704492	361,1008187	576,5802459	388,4311859	319,1484302	0,259373032	False	0,259373032
ENSMUSG00000010057	Nprl2	298,1980189	251,6181237	356,9306284	326,6863442	143,5574723	0,259373032	False	0,259373032
ENSMUSG00000055737	Ghr	1595,850936	1055,451595	1333,273178	1083,341313	854,2262817	-0,259353491	False	0,259353491
ENSMUSG00000032449	Slc25a36	1957,129305	1209,111518	2342,56317	1682,827594	1283,712273	0,259348877	False	0,259348877
ENSMUSG00000034297	Med13	1123,157923	1120,757062	903,8581759	1101,303449	1344,219968	0,259208447	False	0,259208447
ENSMUSG00000027963	Extl2	231,0215421	193,0352781	185,6039268	171,7629232	192,2009134	-0,259162854	False	0,259162854
ENSMUSG00000005899	Smpd4	646,3687826	581,0265833	576,5802459	531,0056385	773,5493551	0,259135821	False	0,259135821
ENSMUSG00000073982	Rhog	2554,344568	3911,605412	3136,596538	3056,93098	6108,90434	0,259130829	False	0,259130829
ENSMUSG00000027394	Ttl	537,4118142	276,5878612	449,1834678	251,4699007	209,9972943	-0,258723207	False	0,258723207
ENSMUSG00000031924	Cyb5b	2991,810893	2297,215847	2500,710895	2246,389604	1627,775637	-0,258680709	False	0,258680709
ENSMUSG00000067219	Nipal1	139,2683055	375,5064365	116,4142973	147,0649866	745,0751457	-0,258598717	False	0,258598717
ENSMUSG00000027293	Ehd4	10387,77714	4121,927431	6336,891464	8683,770008	2977,927732	-0,258526774	False	0,258526774
ENSMUSG00000000561	Wdr77	201,5294303	222,8068882	304,2147202	191,970326	168,4724056	-0,258478229	False	0,258478229
ENSMUSG00000032026	Rexo2	1071,546727	990,146128	1281,655518	1188,868861	815,0742438	0,258313785	False	0,258313785
ENSMUSG00000022144	Gdnf	167,1219666	411,0402937	199,8811519	208,8098283	651,3475398	0,258241063	False	0,258241063
ENSMUSG00000031787	Katnb1	394,0473821	596,3925756	329,4744262	378,3274845	644,2289875	-0,258202629	False	0,258202629
ENSMUSG00000021891	Mettl6	311,3056241	566,6209656	372,3061016	417,6196565	638,2968605	0,258157619	False	0,258157619
ENSMUSG00000030759	Far1	1132,169401	529,1663594	946,6898514	657,8632224	232,5393767	-0,258126077	False	0,258126077
ENSMUSG00000021733	Slc4a7	248,2252739	311,1613438	207,5688885	285,1489053	510,1629182	-0,258059795	False	0,258059795
ENSMUSG00000040040	Ift88	559,530898	590,6303285	429,4150022	669,0895572	440,1638202	0,257981527	False	0,257981527
ENSMUSG00000031950	Gabarapl2	625,0689241	478,2665099	516,1766011	535,4961725	747,4479965	0,257957928	False	0,257957928
ENSMUSG00000000957	Mmp14	1128,073275	2253,038619	943,3951071	1969,099133	3097,756697	-0,257926758	False	0,257926758
ENSMUSG00000018263	Tbx5	899,5094085	1412,710916	752,2999399	954,2384625	2944,707821	-0,257830397	False	0,257830397
ENSMUSG00000044199	S1pr4	697,9799782	548,3738497	528,25733	583,7694123	417,6217377	-0,257787033	False	0,257787033
ENSMUSG00000062397	Zfp706	1867,833745	1231,200132	1502,403384	1048,539675	1562,52224	0,257489322	False	0,257489322
ENSMUSG00000048701	Ccdc6	832,3329318	1018,957364	696,2892874	916,068924	1595,742151	-0,257473892	False	0,257473892
ENSMUSG00000047766	Lrrc49	320,3171027	439,8515292	267,9725333	305,356308	417,6217377	-0,257415695	False	0,257415695
ENSMUSG00000005873	Reep5	2467,506684	2331,78933	2160,253988	2092,588816	2949,453523	0,257393683	False	0,257393683
ENSMUSG00000021488	Nsd1	2298,746266	2651,594044	2240,426098	3604,776121	1923,195559	-0,257341761	False	0,257341761
ENSMUSG00000002486	Tchp	279,3558363	305,3990967	298,7234798	339,0353125	233,7258021	-0,257286953	False	0,257286953
ENSMUSG00000039233	Tbce	527,5811103	895,0690507	630,3944022	884,6351864	1251,678788	0,256861813	False	0,256861813
ENSMUSG00000023118	Sympk	1725,288538	1647,042298	1444,196235	1491,979902	2524,713233	-0,256570872	False	0,256570872
ENSMUSG00000019822	Smpd2	466,1392108	681,8659077	556,8117803	832,9940461	765,2443774	0,256428859	False	0,256428859
ENSMUSG00000028982	Slc25a33	127,7991509	461,9401431	152,6564842	469,2607968	683,3810254	0,256410618	False	0,256410618
ENSMUSG00000029649	Pomp	1231,295666	1910,184916	1821,993577	1470,649866	2691,999213	0,256276577	False	0,256276577
ENSMUSG00000032842	Abcc10	262,1521045	475,3853864	358,0288765	546,7225073	219,4886974	-0,256257478	False	0,256257478
ENSMUSG00000029298	Gbp9	1071,546727	412,0006682	897,2686874	641,0237201	341,6905127	-0,256082793	False	0,256082793
ENSMUSG00000002980	Bcam	12451,40574	7513,009854	9784,292211	7577,776025	10426,30634	-0,256080475	False	0,256080475

ENSMUSG00000039756	Dnttip2	552,1578701	828,803209	462,3624448	573,665711	1456,93038	-0,256056593	False	0,256056593
ENSMUSG00000036879	Phkb	552,1578701	176,7089113	462,3624448	290,7620727	116,2696883	-0,256056593	False	0,256056593
ENSMUSG00000008892	Vdac3	1120,700247	1157,251294	1391,480327	1077,728146	938,4624845	-0,256029474	False	0,256029474
ENSMUSG00000032374	Plod2	903,6055352	566,6209656	756,6929322	669,0895572	544,5692546	-0,255985138	False	0,255985138
ENSMUSG00000050567	Maml1	617,6958962	744,2902514	517,2748492	541,1093399	1621,84351	-0,255965699	False	0,255965699
ENSMUSG00000050628	Ubald2	624,2496988	1282,099981	522,7660896	855,4467158	2102,345793	-0,25595766	False	0,25595766
ENSMUSG00000020307	Cdc34	637,357304	1262,892491	761,0859246	780,2302722	2676,575683	0,255956962	False	0,255956962
ENSMUSG00000022952	Runx1	942,9283508	1495,303124	789,6403749	935,1536932	3413,345851	-0,255952393	False	0,255952393
ENSMUSG00000021047	Nova1	188,4218251	304,4387222	270,1690295	413,1291226	157,794577	-0,255918459	False	0,255918459
ENSMUSG00000020029	Nudt4	2089,024583	2667,920411	1749,509203	2367,63402	2557,933144	-0,255879216	False	0,255879216
ENSMUSG00000012114	Med15	1087,112008	1541,401101	910,4476645	1255,104237	2124,887876	-0,255852599	False	0,255852599
ENSMUSG00000033021	Gmppa	986,3472931	1334,92058	1350,845148	1177,642526	1507,946672	0,25573406	False	0,25573406
ENSMUSG00000001552	Jup	11921,36695	7474,594873	14233,29521	9724,251249	5642,639161	0,255720035	False	0,255720035
ENSMUSG00000037563	Rps16	4926,821114	9203,269005	5882,216756	7322,938224	12531,02498	0,255702857	False	0,255702857
ENSMUSG00000024201	Kdm4b	475,9699147	634,8075564	398,6640557	556,8262087	578,9755909	-0,255696851	False	0,255696851
ENSMUSG00000017376	Nlk	525,1234343	439,8515292	414,039529	397,4122538	303,7249002	-0,255639951	False	0,255639951
ENSMUSG00000066894	Vsig10	548,0617435	553,1757223	459,0677005	647,759521	653,7203906	-0,255631504	False	0,255631504
ENSMUSG00000046020	Pofut1	548,0617435	155,5806719	459,0677005	273,9225704	110,3375614	-0,255631504	False	0,255631504
ENSMUSG00000020549	Elac2	335,0631586	406,2384211	306,4112164	280,6583713	519,6543214	-0,255617974	False	0,255617974
ENSMUSG00000030157	Clec2d	942,1091255	739,4883788	1124,606042	810,5413763	401,0117822	0,255453619	False	0,255453619
ENSMUSG00000006705	Pknox1	471,0545628	267,9444905	562,3030208	341,2805795	139,9981962	0,255453619	False	0,255453619
ENSMUSG00000006930	Hap1	362,0975943	332,2895832	338,2604109	432,2138918	272,87784	0,255366851	False	0,255366851
ENSMUSG00000030697	Ppp4c	1022,393208	1570,212337	1268,476541	1220,302598	3040,808278	0,255288781	False	0,255288781
ENSMUSG00000029098	Acox3	1200,165103	1145,7268	1228,93961	1432,480327	569,4841878	0,255282439	False	0,255282439
ENSMUSG00000024818	Slc25a45	448,1162536	240,0936295	534,8468186	280,6583713	97,28688208	0,255252704	False	0,255252704
ENSMUSG00000091586	Cyp4f17	196,6140784	312,1217183	164,7372131	371,5916836	366,6054459	-0,25520014	False	0,25520014
ENSMUSG00000038696	Mapkap1	727,47209	313,0820928	609,5276885	301,9884075	182,7095103	-0,25520014	False	0,25520014
ENSMUSG00000057265	Bbof1	209,7216836	571,4228382	175,719694	644,3916205	230,1665259	-0,25520014	False	0,25520014
ENSMUSG00000033697	Arhgap39	445,6585776	351,4970736	373,4043497	360,3653488	300,165624	-0,25520014	False	0,25520014
ENSMUSG00000030088	Aldh1l1	294,9211176	498,4343748	247,1058197	588,2599463	520,8407468	-0,25520014	False	0,25520014
ENSMUSG00000042507	Elmsan1	1540,143614	2010,063866	1290,441503	1349,405449	2720,473422	-0,25520014	False	0,25520014
ENSMUSG00000022451	Twf1	1111,688768	2209,821766	1326,68369	1683,950228	3287,584759	0,255071499	False	0,255071499
ENSMUSG00000022387	Brd1	1044,512291	1519,312487	1246,511579	1701,912364	2416,748522	0,255066815	False	0,255066815
ENSMUSG00000030201	Lrp6	1536,047487	989,1857535	1287,146758	1101,303449	900,496872	-0,255046261	False	0,255046261
ENSMUSG00000042594	Sh2b3	2200,439227	3580,276203	1843,958539	2648,292392	5056,545018	-0,25498531	False	0,25498531
ENSMUSG00000036676	Tmtc3	510,3773784	407,1987956	418,4325213	427,7233579	297,7927732	-0,254886391	False	0,254886391
ENSMUSG00000000759	Tubgcp3	874,1134234	1042,966726	1065,300645	1156,31249	1159,137607	0,254800734	False	0,254800734
ENSMUSG00000027455	Nsfl1c	1254,233975	1958,203642	1884,593718	1496,470436	3024,198322	0,254757272	False	0,254757272
ENSMUSG00000018583	G3bp1	1957,94853	1468,412638	2254,703323	1406,659757	2336,071595	0,25474165	False	0,25474165
ENSMUSG00000023990	Tfeb	580,8307565	506,117371	692,9945432	603,9768151	393,8932299	0,254726142	False	0,254726142
ENSMUSG00000028637	Ccdc30	352,2668904	277,5482357	267,9725333	295,2526066	240,8443544	-0,254719086	False	0,254719086
ENSMUSG00000067274	Rplp0	11197,99099	20168,82525	13359,08974	15941,39549	26883,21294	0,254581784	False	0,254581784
ENSMUSG00000020484	Xbp1	5016,9359	6511,339232	5154,078247	4205,385036	7125,6709	-0,254568609	False	0,254568609
ENSMUSG00000036545	Adamts2	686,5108236	1104,430696	575,4819978	791,4566071	1674,046227	-0,254511668	False	0,254511668
ENSMUSG00000020455	Trim11	771,7102576	928,6821588	973,0478055	920,5594579	1063,037151	0,254451624	False	0,254451624
ENSMUSG00000021338	Carmil1	911,7977884	1240,803877	764,3806688	1383,084454	1219,645302	-0,254422617	False	0,254422617
ENSMUSG00000018537	Pcgf2	797,925468	951,7311473	1174,027205	1240,510001	1038,122217	0,254300092	False	0,254300092
ENSMUSG00000028954	Nub1	1819,49945	2091,6957	1525,466593	2074,626681	3724,189303	-0,254291029	False	0,254291029
ENSMUSG00000022994	Adcy6	1027,308559	993,9876261	1083,970862	1315,726445	861,3448341	-0,254206738	False	0,254206738
ENSMUSG00000053835	H2-T24	482,5237173	32,65273361	575,4819978	131,3481178	9,49140313	0,254170942	False	0,254170942
ENSMUSG00000035953	Tmem55b	951,9398294	1135,16268	1161,946476	1229,283666	1241,000959	0,253956774	False	0,253956774
ENSMUSG00000025161	Slc16a3	519,388857	642,4905525	520,5695934	435,5817923	665,5846445	-0,253871413	False	0,253871413
ENSMUSG00000025486	Sirt3	259,6944285	151,7391738	211,9618809	217,7908961	123,3882407	-0,253871413	False	0,253871413
ENSMUSG00000014355	Anapc1	1496,724672	1135,16268	1169,634213	1074,360245	1255,238064	-0,25384785	False	0,25384785
ENSMUSG00000033706	Smyd5	345,7130878	182,4711584	289,9374951	133,5933847	250,3357576	-0,253833305	False	0,253833305
ENSMUSG00000030352	Tspan9	5424,910112	2065,765588	4550,041826	2537,151677	1694,215459	-0,253719426	False	0,253719426
ENSMUSG00000045896	Paip2b	495,6313226	462,9005176	313,0007049	452,4212946	590,8398448	0,253499757	False	0,253499757
ENSMUSG00000027074	Slc43a3	5974,610306	12383,06903	7122,138847	13503,03556	16639,61611	0,253465945	False	0,253465945
ENSMUSG00000026814	Eng	17672,32874	17284,82057	20609,72361	23724,61344	14825,57169	-0,253404424	False	0,253404424
ENSMUSG00000002372	Ranbp3	968,324336	1548,123723	1154,25874	1374,103386	2428,612776	0,253404398	False	0,253404398
ENSMUSG00000032324	Tspan3	887,2210286	957,4933944	810,5070885	744,3060007	1101,002763	-0,253397693	False	0,253397693
ENSMUSG00000044857	Lemd2	834,7906077	1688,338403	995,0127672	1298,886942	2714,541295	0,253300669	False	0,253300669
ENSMUSG00000055681	Cope	1415,621364	2509,458615	1701,186287	1687,318128	2616,067988	0,253296566	False	0,253296566
ENSMUSG00000019179	Mdh2	1753,961424	2765,878612	1471,652437	1740,081902	3292,330461	-0,25318003	False	0,25318003
ENSMUSG00000063888	Rpl7l1	670,9455424	811,5164677	826,9808098	831,8714126	799,6507137	0,253174296	False	0,253174296
ENSMUSG00000040363	Bcor	652,1033599	455,2175215	532,6503224	427,7233579	777,1086313	0,253015633	False	0,253015633
ENSMUSG00000002588	Pon1	1067,4506	699,1526491	842,3562831	502,9398014	895,7511704	-0,252999378	False	0,252999378
ENSMUSG00000041408	Wapl	965,86666	1227,358634	810,5070885	1069,869711	1564,895091	-0,252999232	False	0,252999232
ENSMUSG00000036890	Gtdc1	206,4447823	367,8234404	246,0075716	297,4978736	288,3013701	0,252946763	False	0,252946763
ENSMUSG00000006221	Hspb7	176,9526705	801,9127225	210,8636328	627,5521183	500,6715151	0,252946763	False	0,252946763
ENSMUSG00000004934	Pias4	663,5725145	872,9804368	790,7386229	871,1635845	1045,24077	0,252946763	False	0,252946763
ENSMUSG00000062115	Rai1	1046,969967	793,2693518	878,5984699	784,7208062	683,3810254	-0,252944167	False	0,252944167
ENSMUSG00000015340	Cybb	1464,774884	1229,279383	780,8543902	1114,775051	730,838041	-0,252866119	False	0,252866119
ENSMUSG00000045576	St7l	302,2941455	335,1707068	253,6953082	365,9785162	347,6226396	-0,2528562	False	0,2528562
ENSMUSG00000064294	Aox3	2688,697522	2902,251793	2497,416151	6690,895572	2256,581094	-0,252768836	False	0,252768836
ENSMUSG00000021193	Pitrm1	571,8192779	297,7161006	479,9344142	415,3743895	251,5221829	-0,252721992	False	0,252721992
ENSMUSG00000032372	Plscr2	534,9541382	204,5597723	255,8918044	449,0533941	137,6253454	-0,252528217	False	0,252528217
ENSMUSG00000030798	Cd37	1996,452121	769,2599889	1675,926581	910,4557565	485,247985	-0,252479524	False	0,252479524
ENSMUSG00000005102	Eif2ak4	286,7288643	605,9963208	341,5551552	552,3356747	907,6154243	0,252431423	False	0,252431423
ENSMUSG00000055239	Kcmf1	1087,931234	2397,094797	1295,932743	1877,043187	3179,620049	0,252403478	False	0,252403478
ENSMUSG00000035203	Epn1	4110,053463	5557,687335	5312,225999	4895,804629	7721,256446	0,252388827	False	0,252388827
ENSMUSG00000058355	Abce1	1374,660098	1645,121549	1594,656223	1154,067223	2682,50781	-0,252347677	False	0,252347677
ENSMUSG00000034560	Washc4	414,5280152	315,0028419	328,3761781	348,0163804	278,8099669	-0,252314395	False	0,252314395
ENSMUSG00000021188	Trip11	885,582578	1010,313993	743,5139552	820,6450777	1289,6444	-0,25226702	False	0,25226702
ENSMUSG00000022427	Tomm22	901,1478592	1120,757062	1149,865748	1073,237612	1388,117708	0,252133774	False	0,252133774
ENSMUSG00000020321	Mdh1	1209,995807	1487,620128	1128,999034	1015,983304	1456,93038	-0,252125354	False	0,252125354
ENSMUSG00000014418	Hps5	519,388857	384,1498072	567,7942612	618,5710504	200,5058911	0,252124253	False	0,252124253
ENSMUSG00000036002	Fam214b	895,4132819	1593,261325	1066,398893	1558,215278	1808,112296	0,252121564	False	0,252121564
ENSMUSG00000036959	Bcorl1	344,8938625	177,6692858	410,7447847	287,3941722	130,506793	0,252089801	False	0,252089801
ENSMUSG00000000078	Klf6	3103,225537	5865,007181	3695,604814	5113,595525	9010,900847	0,252041939	False	0,252041939
ENSMUSG00000028399	Ptprd	887,2210286	404,317672	1056,51466	673,5800911	503,0443659	0,251947321	False	0,251947321
ENSMUSG00000059923	Grb2	1546,697417	1655,685669	1533,15433	1298,886942	2661,152153	-0,251915126	False	0,251915126
ENSMUSG00000038619	Ensa	1276,353059	830,723958	1071,890133	892,4936208	828,1249231	-0,251870416	False	0,251870416
ENSMUSG00000004895	Prcc	512,8350544	765,4184908	610,6259366	698,2780278	1320,49146	0,25179399	False	0,25179399
ENSMUSG00000008976	Gabpa	667,6686411	595,4322011	602,9382	487,2229326	794,9050121	0,251650193	False	0,251650193
ENSMUSG00000013846	St3gal1	3585,749254	3898,160168	3300,235503	2724,631469	4268,758558	0,251541959	False	0,251541959
ENSMUSG00000028261	Ndufaf4	212,1793596	399,5157995	371,2078535	252,5925342	447,2823725	0,251527678	False	0,251527678
ENSMUSG00000048661	Lemd3	381,7590022	460,0193941	320,6884415	371,5916836	592,0262702	-0,251489821	False	0,251489821
ENSMUSG00000021279	Cdc42bpb	3170,402014	3545,70272	2663,251612	3589,059252	5031,630084	-0,251477054	False	0,251477054
ENSMUSG00000021969	Zdhhc20	1066,631375	1381,978931	1269,574789	1804,072011	1900,653477	0,251283714	False	0,251283714
ENSMUSG00000006676	Usp19	2227,473663	2317,383712	2200,889167	1871,43002	4091,981174	-0,251267273	False	0,251267273
ENSMUSG00000038039	Gcc2	821,6830025	679,9451587	542,5345552	690,4195934	648,974689	-0,251108516	False	0,251108516
ENSMUSG00000025894	Aasdhppt	240,852246	257,3803708	286,6427508	270,5546699	142,371047	0,251105416	False	0,251105416
ENSMUSG00000041028	Ghitm	2233,20824	1876,571808	1753,902196	1579,545314	1847,264334	-0,251018288	False	0,251018288
ENSMUSG00000024072	Yipf4	981,4319412	600,2340737	824,7843137	647,759521	683,3810254	-0,250871331	False	0,250871331
ENSMUSG00000021996	Esd	866,7403955	3807,884964	1031,254954	1761,411938	4691,125997	0,2507292	False	0,2507292
ENSMUSG00000057649	Brd9	833,9713824	992,066877	1136,68677	1212,444164	1001,34303	0,2504395	False	0,2504395
ENSMUSG00000018160	Med1	786,4563135	814,3975912	661,1453486	693,7874939	988,2923509	-0,250399154	False	0,250399154
ENSMUSG00000039713	Plekhg5	806,9369466	527,2456103	959,8688284	867,7956841	644,2289875	0,25038132	False	0,25038132
ENSMUSG00000041642	Kif21b	1025,670109	862,4163171	707,2717683	826,2582451	857,7855579	-0,250110405	False	0,250110405

ENSMUSG00000026123	Plekhb2	1718,734735	1079,460958	1445,294483	1233,7742	934,9032083	-0,249983424	False	0,249983424
ENSMUSG00000022354	Ndufb9	442,3816763	944,0481511	526,0608339	618,5710504	769,9900789	0,249938013	False	0,249938013
ENSMUSG00000061787	Rps17	2535,502386	4882,544049	3014,691	4118,942257	6498,051868	0,249738504	False	0,249738504
ENSMUSG00000017801	Mlx	513,6542797	649,2131741	619,4119213	610,712616	644,2289875	0,249695983	False	0,249695983
ENSMUSG00000042650	Alkbh5	1446,751927	1476,095634	1216,858881	1307,86801	2179,463444	-0,249655697	False	0,249655697
ENSMUSG00000048271	Rbm33	1385,310027	1900,581171	1165,241221	1783,864608	2510,476128	-0,249580239	False	0,249580239
ENSMUSG00000030615	Tmem126a	260,5136538	267,9444905	309,7059607	236,8756654	223,0479736	0,249540162	False	0,249540162
ENSMUSG00000036862	Dchs1	503,0043505	350,536699	308,6077126	413,1291226	597,9583972	0,249474235	False	0,249474235
ENSMUSG00000032867	Fbxw8	2210,269931	4107,521813	1859,334012	2876,186989	5942,804785	-0,24943661	False	0,24943661
ENSMUSG00000071078	Nr2c2ap	296,5595682	492,6721277	378,8955902	352,5069144	582,5348671	0,249329636	False	0,249329636
ENSMUSG00000000340	Dbt	321,136328	378,3875601	270,1690295	447,9307606	507,7900675	-0,249323574	False	0,249323574
ENSMUSG00000018750	Zbtb4	4688,426544	1615,349939	3944,90713	2799,847912	839,989177	-0,249112492	False	0,249112492
ENSMUSG00000005566	Trim28	1998,909797	2648,71292	2375,510613	2506,840573	3170,128645	0,249024282	False	0,249024282
ENSMUSG00000031590	Frg1	519,388857	687,6281548	617,2154251	684,806426	987,1059255	0,248959055	False	0,248959055
ENSMUSG00000032185	Carm1	1237,849468	1500,104997	1518,877105	2027,476074	1041,681494	-0,248921659	False	0,248921659
ENSMUSG00000042558	Adprhl2	353,0861157	476,3457609	419,5307694	444,5628602	756,9393996	0,248756534	False	0,248756534
ENSMUSG00000020946	Gosr2	1654,015934	1828,553082	1579,28075	1392,065522	1926,754835	-0,248746016	False	0,248746016
ENSMUSG00000026317	Cln8	433,3701977	367,8234404	462,3624448	355,8748148	514,9086198	0,248716477	False	0,248716477
ENSMUSG00000021916	Glt8d1	698,7992035	346,695201	830,2755541	473,7513308	320,3348556	0,248712258	False	0,248712258
ENSMUSG00000033159	Cnppd1	1238,668694	1976,450758	1471,652437	2181,276862	1968,279724	0,248646626	False	0,248646626
ENSMUSG00000058013	Sept11	1585,201007	642,4905525	1334,371426	715,1175301	618,1276288	-0,248505488	False	0,248505488
ENSMUSG00000007570	Fance	330,9670319	496,5136258	393,1728153	432,2138918	539,823553	0,248476059	False	0,248476059
ENSMUSG00000038539	Atf5	3301,478066	1466,491889	3921,84392	1999,410237	1417,778343	0,24842006	False	0,24842006
ENSMUSG00000026307	Scly	662,7532892	557,9775949	689,6997989	592,7504802	1251,678788	-0,248264732	False	0,248264732
ENSMUSG00000022090	Pdlim2	2895,961529	2460,479515	3198,098431	3439,748999	1365,575625	0,248260857	False	0,248260857
ENSMUSG00000025369	Smarcc2	3494,815243	2839,82745	2740,128978	2631,452889	2942,33497	-0,248254688	False	0,248254688
ENSMUSG00000038280	Ostm1	553,7963207	632,8868073	577,678494	593,8731137	466,2651788	-0,248204777	False	0,248204777
ENSMUSG00000040760	Appl1	700,4376542	433,1289076	589,7592229	461,4023624	427,1131409	-0,248130567	False	0,248130567
ENSMUSG00000037419	Endod1	898,6901832	424,4855369	756,6929322	403,0254212	614,5683527	-0,248115873	False	0,248115873
ENSMUSG00000026155	Smap1	1191,97285	2014,865739	1415,641785	1902,863757	2416,748522	0,248104875	False	0,248104875
ENSMUSG00000035765	Dym	729,1105406	850,8918229	613,9206809	967,7100643	659,6525175	-0,248085288	False	0,248085288
ENSMUSG00000015377	Dennd6b	245,767598	334,2103322	300,919976	345,7711135	291,8606462	0,247984972	False	0,247984972
ENSMUSG00000022721	Trmt2a	616,8766709	737,5676298	732,5314743	773,4944713	899,3104466	0,247908661	False	0,247908661
ENSMUSG00000079316	Rab9	573,4577286	680,9055332	701,7805279	921,6820914	843,5484532	0,247767513	False	0,247767513
ENSMUSG00000034675	Dbn1	284,2711883	1001,670622	239,4180831	255,9604346	3643,512377	-0,247735763	False	0,247735763
ENSMUSG00000024383	Map3k2	533,3156876	374,546062	449,1834678	386,1859189	427,1131409	-0,247684936	False	0,247684936
ENSMUSG00000049969	Plekhf2	555,4347714	548,3738497	467,8536852	493,9587335	715,4145109	-0,247560078	False	0,247560078
ENSMUSG00000023243	Kcnk5	135,9914042	492,6721277	161,4424689	212,1777287	596,7719718	0,247504678	False	0,247504678
ENSMUSG00000030218	Mgp	14569,10321	20920,7985	16693,37093	12272,62926	19577,20538	-0,247467714	False	0,247467714
ENSMUSG00000025616	Usp16	529,2195609	838,4069542	628,197906	912,7010235	1027,444389	0,247352747	False	0,247352747
ENSMUSG00000018849	Wwc1	4003,554171	2651,594044	2601,749719	2285,681776	3373,007387	-0,247245849	False	0,247245849
ENSMUSG00000036435	Exoc1	443,2009017	592,5510776	373,4043497	492,8361	733,2108918	-0,247222083	False	0,247222083
ENSMUSG00000072258	Taf1a	195,794853	249,6973747	282,2497585	232,3851314	443,7230963	0,247174924	False	0,247174924
ENSMUSG00000040774	Cept1	885,582578	836,4862051	1051,02342	871,1635845	571,8570386	0,247096071	False	0,247096071
ENSMUSG00000029221	Slc30a9	901,9670845	703,9545216	746,8086994	760,0228695	561,1792101	-0,247031955	False	0,247031955
ENSMUSG00000036661	Dennd3	1984,982966	7335,340568	1672,631837	5521,11148	15460,30927	-0,247006698	False	0,247006698
ENSMUSG00000040950	Mgl2	342,4361865	60,50359463	406,3517923	75,21644351	141,1846216	0,246894093	False	0,246894093
ENSMUSG00000040302	Rbm48	192,5179517	297,7161006	228,4356022	305,356308	514,9086198	0,246794536	False	0,246794536
ENSMUSG00000019428	Fkbp8	4816,225695	5881,333548	5714,184799	6184,58787	5770,773103	0,246644694	False	0,246644694
ENSMUSG00000048612	Myof	1728,565439	1269,615113	1204,778152	1402,169223	1456,93038	-0,246643282	False	0,246643282
ENSMUSG00000035711	Dok3	819,2253265	2225,187758	971,9495574	1297,764309	3587,750383	0,246621125	False	0,246621125
ENSMUSG00000046675	Tmem251	163,8450653	328,4480851	194,3899115	300,865774	380,8425506	0,246621125	False	0,246621125
ENSMUSG00000048758	Rpl29	4102,680435	7807,844831	4867,435523	7066,97779	11034,94256	0,246595084	False	0,246595084
ENSMUSG00000078451	Ppil6	299,0172442	341,8933284	251,498812	354,7521813	182,7095103	0,246582869	False	0,246582869
ENSMUSG00000041608	Entpd3	185,1449238	1183,181406	219,6496175	720,7306975	1880,484245	0,246548992	False	0,246548992
ENSMUSG00000025555	Farp1	1577,827979	2667,920411	1329,978434	1819,78888	4000,626419	-0,246537074	False	0,246537074
ENSMUSG00000024240	Epc1	683,2339223	1000,710248	843,4545311	810,5413763	1114,053442	0,246506228	False	0,246506228
ENSMUSG00000078234	Klhdc7a	416,9856912	351,4970736	330,5726743	282,9036383	149,4895993	-0,246485201	False	0,246485201
ENSMUSG00000022488	Nckap1l	1052,704545	887,3860546	836,8650426	762,2681365	778,2950567	-0,246466796	False	0,246466796
ENSMUSG00000047798	Cd300lf	715,1837101	1810,305966	602,9382	1541,375775	1127,104122	-0,24630374	False	0,24630374
ENSMUSG00000003420	Fcgrt	1180,503696	904,6727959	1400,266311	1231,528933	900,496872	0,246298676	False	0,246298676
ENSMUSG00000039988	Ankrd13c	732,3874419	799,9919734	673,2260776	617,4484169	950,3267384	-0,246288438	False	0,246288438
ENSMUSG00000022337	Emc2	513,6542797	546,4531007	643,5733792	623,0615843	433,0452678	-0,246279822	False	0,246279822
ENSMUSG00000038482	Tfdp1	351,4476651	416,8025408	281,1515104	321,0731768	331,0126842	0,246054202	False	0,246054202
ENSMUSG00000055204	Ankrd17	1609,777767	1625,914059	1357,434636	2034,211875	2695,558489	-0,245978805	False	0,245978805
ENSMUSG00000059291	Rpl11	3208,905604	6072,448077	3805,429623	5169,7272	7806,679074	0,245977989	False	0,245977989
ENSMUSG00000064262	Gimap8	908,5208871	387,9913052	1077,381374	903,7199556	74,74479965	0,245937441	False	0,245937441
ENSMUSG00000032560	Dnajc13	647,188008	521,4833632	527,159082	506,3077018	545,75568	-0,245929635	False	0,245929635
ENSMUSG00000090733	Rps27	5925,456787	11251,74785	7026,591263	9119,151801	14085,24224	0,245898605	False	0,245898605
ENSMUSG00000027875	Hmgcs2	885,582578	3033,823102	746,8086994	2784,131043	3278,093356	-0,245888107	False	0,245888107
ENSMUSG00000033767	Tmem131l	834,7906077	1445,363649	703,977024	1471,772499	1727,43537	-0,245886025	False	0,245886025
ENSMUSG00000035356	Nfkbiz	560,3501234	1016,07624	664,4400929	866,6730506	2446,409157	0,245810582	False	0,245810582
ENSMUSG00000032306	Mpi	316,220976	185,352282	347,0463956	374,9595841	106,7782852	0,24580203	False	0,24580203
ENSMUSG00000032788	Pdxk	787,2755388	613,679317	933,5108743	617,4484169	577,7891655	0,245798175	False	0,245798175
ENSMUSG00000042444	Mindy2	1354,179465	849,9314484	1142,178011	1014,860671	843,5484532	-0,245631432	False	0,245631432
ENSMUSG00000030281	Il17rc	651,2841346	549,3342243	719,3524973	684,806426	699,9909808	-0,245602906	False	0,245602906
ENSMUSG00000002996	Hbp1	1464,774884	2083,052329	1235,529098	2055,541911	3050,299681	-0,24554997	False	0,24554997
ENSMUSG00000070867	Trabd2b	528,4003356	431,2081586	530,4538262	520,9019372	626,4326066	0,245527926	False	0,245527926
ENSMUSG00000046111	Cep295	308,0287228	423,5251624	310,8042087	342,403213	259,8271607	-0,245512635	False	0,245512635
ENSMUSG00000048429	Timm29	554,6155461	702,0337726	467,8536852	509,6756023	1261,170191	-0,245430639	False	0,245430639
ENSMUSG00000071713	Csf2rb	969,9627866	818,2390893	666,6365891	632,0426522	638,2968605	-0,245406938	False	0,245406938
ENSMUSG00000040479	Dgkz	3440,746371	2087,854202	2902,669695	2736,980437	2301,665259	-0,245341138	False	0,245341138
ENSMUSG00000041133	Smc1a	1552,431994	1411,750541	1351,943396	1253,981603	1840,145782	0,245289995	False	0,245289995
ENSMUSG00000029833	Trim24	570,1808273	991,1065025	481,0326623	975,5684987	1303,881505	-0,245284672	False	0,245284672
ENSMUSG00000033460	Armcx1	307,2094975	79,71108499	259,1865486	206,5645613	18,98280626	-0,245231971	False	0,245231971
ENSMUSG00000052656	Rnf103	629,9842761	1005,51212	531,5520743	737,5701998	1159,137607	-0,245104786	False	0,245104786
ENSMUSG00000037894	H2afz	996,1779971	1379,097808	1180,616694	1344,914915	1799,807319	0,245065196	False	0,245065196
ENSMUSG00000029672	Fam3c	778,2640602	337,0914558	656,7523563	412,0064891	323,8941318	-0,244910264	False	0,244910264
ENSMUSG00000048486	Fitm2	549,7001941	751,012873	524,9625858	832,9940461	463,892328	-0,244855002	False	0,244855002
ENSMUSG00000039236	Isg20	892,1363806	746,2110004	799,5246076	630,9200187	1057,105024	0,244782539	False	0,244782539
ENSMUSG00000027342	Pcna	650,4649093	920,0387882	552,418788	548,9677743	959,8181415	-0,244749768	False	0,244749768
ENSMUSG00000066892	Fbxl12	497,2697732	419,6836643	651,2611158	523,1472041	526,7728737	-0,24472643	False	0,24472643
ENSMUSG00000070372	Capza1	770,0718069	628,0849347	590,857471	543,3546069	912,3611259	0,244611998	False	0,244611998
ENSMUSG00000002365	Snx9	1099,400388	1213,913391	928,0196339	994,6532679	1309,813632	-0,24448966	False	0,24448966
ENSMUSG00000041075	Fzd7	650,4649093	269,8652395	549,1240437	355,8748148	200,5058911	-0,244339148	False	0,244339148
ENSMUSG00000039982	Dtx4	755,3257511	773,1014869	710,5665126	637,6558196	1147,273353	-0,244321046	False	0,244321046
ENSMUSG00000027006	Dnajc10	1272,256932	965,1763905	1074,086629	1024,964372	686,9403015	-0,244279695	False	0,244279695
ENSMUSG00000026679	Enkur	247,4060486	190,1541546	281,1515104	293,0073396	98,47330747	0,244056032	False	0,244056032
ENSMUSG00000026956	Uap1l1	597,215263	306,3594712	707,2717683	551,2130413	230,1665259	0,244013638	False	0,244013638
ENSMUSG00000028854	Slc9a1	1173,949893	1038,164854	1097,149839	991,2853675	1574,386494	-0,243998492	False	0,243998492
ENSMUSG00000027188	Pamr1	373,5667489	237,2125059	255,8918044	315,4600094	224,2343989	-0,243908919	False	0,243908919
ENSMUSG00000036120	Rfxank	407,1549873	553,1757223	482,1309104	664,5990233	622,8733304	0,243846853	False	0,243846853
ENSMUSG00000029617	Ccz1	684,0531477	1074,659086	577,678494	875,6541185	1503,200971	-0,243841633	False	0,243841633
ENSMUSG00000062980	Cped1	1777,718959	723,162012	1501,305136	1288,783241	554,0606577	-0,243810035	False	0,243810035
ENSMUSG00000029101	Rgs12	2279,904084	2498,894496	2990,529542	3371,268356	1925,56841	-0,243688752	False	0,243688752
ENSMUSG00000022568	Scrib	1044,512291	1551,004846	1236,627346	1506,574137	1319,305035	0,243581345	False	0,243581345
ENSMUSG00000032091	Tmprss4	334,2439332	395,6743014	293,2322393	336,7900456	218,302272	0,243411992	False	0,243411992
ENSMUSG00000056204	Pgpep1	434,1894231	266,984116	513,9801049	387,3085524	186,2687864	0,243387935	False	0,243387935
ENSMUSG00000019734	Tmc4	1621,246921	1057,372344	1334,371426	1369,612852	1140,154801	-0,24333569	False	0,24333569
ENSMUSG00000003949	Hlf	245,767598	303,4783477	545,8292994	642,1463536	207,6244435	-0,243318422	False	0,243318422

ENSMUSG00000032000	Birc3	770,8910323	2613,179063	651,2611158	1560,460544	4408,756754	-0,243290854	False	0,243290854
ENSMUSG00000024589	Nedd4l	1761,334452	2534,428353	1488,126158	2210,465332	3250,805572	-0,243172044	False	0,243172044
ENSMUSG00000022579	Gpihbp1	2238,123592	3867,428184	2648,974387	5469,47034	4738,583013	0,243144188	False	0,243144188
ENSMUSG00000042826	Fgf11	377,6628755	74,9092124	446,9869716	163,9044888	42,71131409	0,243133808	False	0,243133808
ENSMUSG00000022604	Cep97	195,794853	311,1613438	231,7303464	301,9884075	261,0135861	0,243104145	False	0,243104145
ENSMUSG00000027465	Tbc1d20	1493,44777	1927,471657	1261,887052	1686,195495	2251,835393	-0,243063999	False	0,243063999
ENSMUSG00000054408	Spcs3	968,324336	815,3579657	818,1948251	587,1373128	488,8072612	-0,24304594	False	0,24304594
ENSMUSG00000070003	Ssbp4	1747,407622	2142,59555	2068,001149	2191,380563	2582,848077	0,243020799	False	0,243020799
ENSMUSG00000061981	Flot2	2278,265633	3187,483025	1925,228897	2566,340147	4737,396587	-0,242905984	False	0,242905984
ENSMUSG00000024965	Fermt3	824,9599038	900,8312978	729,23673	697,1553943	1038,122217	-0,242843735	False	0,242843735
ENSMUSG00000032221	Mns1	323,594004	113,3241931	273,4637738	186,3571586	93,72760591	-0,242835146	False	0,242835146
ENSMUSG00000051000	Fam160a1	318,678652	125,8090618	180,1126863	151,5555205	269,3185638	-0,242788701	False	0,242788701
ENSMUSG00000047714	Ppp1r2	2744,404844	7107,731807	2319,499961	3733,878972	10082,24297	-0,242679496	False	0,242679496
ENSMUSG00000020664	Dld	697,1607529	1191,824777	824,7843137	885,7578199	1377,439879	0,24252554	False	0,24252554
ENSMUSG00000037095	Lrg1	2410,160911	12172,74701	2037,250202	6697,631373	18558,06597	-0,242506295	False	0,242506295
ENSMUSG00000041957	Pkp2	745,4950471	599,2736992	881,8932142	852,0788153	506,6036421	0,242405207	False	0,242405207
ENSMUSG00000066324	Impad1	1332,879606	1479,937132	1126,802538	1205,708363	2448,782008	-0,242311755	False	0,242311755
ENSMUSG00000028385	Snx30	493,9928719	368,7838149	374,5025978	417,6196565	173,2181071	-0,242300606	False	0,242300606
ENSMUSG00000003062	Stard3nl	515,2927304	332,2895832	609,5276885	422,1101905	231,3529513	0,242299519	False	0,242299519
ENSMUSG00000040548	Tex2	614,4189949	367,8234404	519,4713453	422,1101905	237,2850783	-0,242178647	False	0,242178647
ENSMUSG00000025736	Jmjd8	933,9168723	205,5201468	789,6403749	270,5546699	228,9801005	-0,242098384	False	0,242098384
ENSMUSG00000044709	Gemin7	496,4505479	690,5092784	697,3875355	587,1373128	671,5167715	0,242047927	False	0,242047927
ENSMUSG00000011154	Cfap161	303,9325961	121,9675638	256,9900525	251,4699007	74,74479965	-0,242038892	False	0,242038892
ENSMUSG00000048537	Phldb1	1563,081923	1890,977426	2189,906686	2676,358229	1321,677886	-0,242022782	False	0,242022782
ENSMUSG00000072235	Tuba1a	5148,011952	3226,85838	3640,69241	2603,387052	4352,994761	-0,242007119	False	0,242007119
ENSMUSG00000021737	Psmd6	486,619844	920,0387882	575,4819978	648,8821545	1231,509556	0,241975645	False	0,241975645
ENSMUSG00000023074	Mospd1	212,9985849	312,1217183	180,1126863	263,818869	409,31676	-0,241944044	False	0,241944044
ENSMUSG00000043263	Ifi209	361,278369	114,2845676	427,218506	178,4987242	47,45701565	0,241863264	False	0,241863264
ENSMUSG00000024168	Tmem204	2884,492375	2375,006183	1666,042349	2377,737722	2439,290604	-0,241855806	False	0,241855806
ENSMUSG00000031701	Dnaja2	1418,07904	1673,932785	1199,286911	1511,064671	2476,069792	-0,241761104	False	0,241761104
ENSMUSG00000033530	Ttc7b	779,0832855	884,504931	658,9488525	753,2870686	1119,985569	-0,241611076	False	0,241611076
ENSMUSG00000022967	Ifnar1	1150,192358	872,0200623	886,2862065	800,437675	972,8688208	-0,241557964	False	0,241557964
ENSMUSG00000001924	Uba1	4386,132398	2939,706399	2804,925615	2336,200283	3709,952198	-0,241548763	False	0,241548763
ENSMUSG00000052155	Acvr2a	610,3228683	758,6958692	721,5489934	846,4656479	923,0389544	0,24152471	False	0,24152471
ENSMUSG00000079334	Nat6	1256,691651	1124,59856	2800,532623	2451,831532	1063,037151	-0,241438689	False	0,241438689
ENSMUSG00000018143	Mafk	834,7906077	1605,746194	706,1735202	1265,207938	2473,696941	-0,241391644	False	0,241391644
ENSMUSG00000047446	Arl4a	394,8666074	1345,4847	466,7554372	990,162734	1872,179267	0,241301459	False	0,241301459
ENSMUSG00000019861	Gopc	505,4620265	373,5856875	597,4469596	417,6196565	315,5891541	0,241207927	False	0,241207927
ENSMUSG00000027244	Atg13	1083,835107	1321,475337	917,037153	1310,113277	1961,161172	-0,241093194	False	0,241093194
ENSMUSG00000025792	Slc25a10	1169,034541	979,5820083	1381,596094	1057,520743	1191,171093	0,241018352	False	0,241018352
ENSMUSG00000021209	Ppp4r4	235,1176687	126,7694364	277,8567661	252,5925342	148,3031739	0,240958413	False	0,240958413
ENSMUSG00000004099	Dnmt1	724,1951887	541,6512281	612,8224328	596,1183807	508,9764928	-0,240909483	False	0,240909483
ENSMUSG00000026042	Col5a2	947,0244775	442,7326528	1119,114801	767,8813039	571,8570386	0,240884418	False	0,240884418
ENSMUSG00000006392	Med8	476,78914	987,2650044	563,4012688	671,3348242	1322,864311	0,240811437	False	0,240811437
ENSMUSG00000020700	Map3k3	1793,28424	1407,909043	1171,830709	1320,216979	2118,955749	0,240749283	False	0,240749283
ENSMUSG00000002017	Fam98a	545,6040675	390,8724288	644,6716273	546,7225073	403,384633	0,240710091	False	0,240710091
ENSMUSG00000031734	Irx3	1420,536716	1516,431364	1394,775071	1202,340463	2464,205538	-0,240590644	False	0,240590644
ENSMUSG00000031537	Ikbkb	1332,060381	2496,013372	1573,789509	2320,483414	3864,187499	0,240583117	False	0,240583117
ENSMUSG00000031652	N4bp1	1813,764873	1268,654738	1535,350826	1361,754418	1525,743053	-0,240419097	False	0,240419097
ENSMUSG00000005886	Ncoa2	1080,558206	793,2693518	766,577165	847,5882814	914,7339767	-0,240352643	False	0,240352643
ENSMUSG00000002957	Ap2a2	2021,848106	1765,168364	1588,066734	1703,034997	2388,274313	0,240293934	False	0,240293934
ENSMUSG00000018501	Ncor1	3410,435034	3209,571639	2887,294222	4342,346321	4322,1477	-0,240237652	False	0,240237652
ENSMUSG00000032011	Thy1	847,0789876	200,7182742	717,1560011	295,2526066	174,4045325	-0,240209524	False	0,240209524
ENSMUSG00000031256	Cstf2	663,5725145	561,819093	535,9450667	373,8369506	417,6217377	-0,240148476	False	0,240148476
ENSMUSG00000039458	Mtmr12	1074,004403	1460,729642	909,3494164	1815,298346	1922,009134	-0,240093248	False	0,240093248
ENSMUSG00000020170	Frs2	901,1478592	752,9336221	755,5946841	993,5306344	1064,223576	0,239965523	False	0,239965523
ENSMUSG00000009291	Pttg1ip	3906,885582	8612,638677	4613,740215	5981,391209	11820,35617	0,239917768	False	0,239917768
ENSMUSG00000028792	Ak2	488,2582946	1041,045977	576,5802459	753,2870686	1670,486951	0,239876857	False	0,239876857
ENSMUSG00000027593	Raly	1468,87101	1689,298777	1369,515365	1243,877902	2408,443544	-0,239862832	False	0,239862832
ENSMUSG00000028484	Psip1	852,8135649	722,2016375	917,037153	977,8137656	862,5312594	-0,239828695	False	0,239828695
ENSMUSG00000028706	Nsun4	539,0502649	476,3457609	555,7135322	636,5331861	196,9466149	0,239815926	False	0,239815926
ENSMUSG00000020949	Fkbp3	391,5897061	318,84434	462,3624448	389,5538194	242,0307798	0,23968138	False	0,23968138
ENSMUSG00000021375	Kif13a	1369,744746	1160,132418	1382,694342	1635,676988	1351,338521	-0,239617586	False	0,239617586
ENSMUSG00000019850	Tnfaip3	322,7747787	1605,746194	381,0920863	551,2130413	3349,27888	0,239611798	False	0,239611798
ENSMUSG00000028049	Scamp3	1304,20672	1980,292256	1539,743819	1715,383965	2880,64085	0,239517778	False	0,239517778
ENSMUSG00000032215	Rsl24d1	189,2410504	232,4106333	297,6252317	223,4040636	446,0959471	0,239430354	False	0,239430354
ENSMUSG00000061559	Wdr61	439,104775	475,3853864	518,3730973	389,5538194	370,1647221	0,239425624	False	0,239425624
ENSMUSG00000022433	Csnk1e	1878,483674	3438,140774	2217,362888	3457,711135	4547,568525	0,23927632	False	0,23927632
ENSMUSG00000057177	Gsk3a	2481,433514	4080,631327	2929,027649	3238,797605	5419,591187	0,239248011	False	0,239248011
ENSMUSG00000019854	Reps1	999,4548984	2151,23892	846,7492754	1282,04744	3910,45809	-0,239206618	False	0,239206618
ENSMUSG00000017639	Rab11fip4	409,6126633	443,6930273	347,0463956	401,9027877	537,4507022	-0,239131772	False	0,239131772
ENSMUSG00000038725	Pkhd1l1	304,7518215	14,40561777	93,35108743	258,2057016	99,65973287	-0,239113986	False	0,239113986
ENSMUSG00000024935	Slc1a1	394,0473821	304,4387222	317,3936973	246,9793668	465,0787534	0,239105917	False	0,239105917
ENSMUSG00000039989	Cbx4	821,6830025	710,6771433	969,7530612	749,9191681	688,1267269	0,239035502	False	0,239035502
ENSMUSG00000053898	Ech1	1078,10053	1179,339908	1318,995953	1344,914915	913,5475513	-0,238939981	False	0,238939981
ENSMUSG00000028270	Gbp2	1575,370303	960,3745179	1336,567922	738,6928333	1859,128588	0,238935574	False	0,238935574
ENSMUSG00000026471	Mr1	747,1334978	548,3738497	463,4606929	633,1652857	336,9448111	-0,238783887	False	0,238783887
ENSMUSG00000020163	Uqcr11	717,641386	983,4235064	846,7492754	863,3051502	1264,729467	0,238671755	False	0,238671755
ENSMUSG00000026761	Orc4	498,9082239	354,3781971	588,6609749	453,543928	367,7918713	0,238662537	False	0,238662537
ENSMUSG00000026966	Ssna1	380,1205515	448,4948999	539,2398109	508,5529688	580,1620163	0,238634552	False	0,238634552
ENSMUSG00000036707	Cab39	2354,453588	3219,175384	1995,516775	2573,075948	4297,232767	-0,238629878	False	0,238629878
ENSMUSG00000030411	Nova2	607,8651923	627,1245602	717,1560011	531,0056385	516,0950452	0,23853557	False	0,23853557
ENSMUSG00000048668	Rhno1	415,3472406	503,2362474	528,25733	551,2130413	489,9936866	0,238445191	False	0,238445191
ENSMUSG00000045248	Med26	188,4218251	274,6671121	272,3655257	222,2814301	563,5520608	0,238421344	False	0,238421344
ENSMUSG00000033170	Card10	1678,592694	1120,757062	1980,141302	1725,487667	1013,207284	0,238351177	False	0,238351177
ENSMUSG00000049807	Arhgap23	2015,294303	1618,231063	1460,669956	1671,60126	1708,452563	-0,238300347	False	0,238300347
ENSMUSG00000027901	Dennd2d	485,8006186	715,4790159	667,7348372	896,9841547	573,043464	0,238280241	False	0,238280241
ENSMUSG00000026568	Mpc2	417,8049165	575,2643362	599,6434557	492,8361	565,9249116	0,238278467	False	0,238278467
ENSMUSG00000014504	Srp19	293,2826669	563,739842	345,9481475	458,034462	818,63352	0,238264006	False	0,238264006
ENSMUSG00000063952	Brpf3	931,4591963	1413,67129	1239,922091	1584,035848	1098,629912	0,238141001	False	0,238141001
ENSMUSG00000019297	Nop9	287,5480896	370,7045639	243,8110754	319,9505433	455,5873502	-0,238039589	False	0,238039589
ENSMUSG00000036646	Man1b1	1545,058966	957,4933944	1310,209968	969,9553313	665,5846445	-0,237863868	False	0,237863868
ENSMUSG00000041229	Phf8	713,5452594	530,1267339	605,1346962	550,0904078	583,7212925	-0,237748635	False	0,237748635
ENSMUSG00000002015	Bcap31	2646,097805	2141,635175	3120,122816	2031,966608	2878,267999	0,237736431	False	0,237736431
ENSMUSG00000038416	Cdc16	590,6614604	533,0078575	524,9625858	564,6846431	696,4317047	0,237650401	False	0,237650401
ENSMUSG00000021476	Habp4	1046,969967	888,3464291	1234,43085	914,9462905	772,3629297	0,237625964	False	0,237625964
ENSMUSG00000030987	Stim1	1549,974318	2139,714426	1314,602961	2254,248038	1773,70596	-0,237617172	False	0,237617172
ENSMUSG00000025428	Atp5a1	5466,690604	5520,232729	3969,068588	4053,829515	6444,662725	-0,237437132	False	0,237437132
ENSMUSG00000018736	Ndel1	1283,726087	1306,109344	1121,311297	1088,954481	1958,788321	-0,237393753	False	0,237393753
ENSMUSG00000024789	Jak2	870,0172968	1017,996989	738,0227147	855,4467158	1375,067028	-0,237378863	False	0,237378863
ENSMUSG00000029676	Pot1a	249,8637246	158,4617955	211,9618809	182,9892581	148,3031739	-0,237336631	False	0,237336631
ENSMUSG00000034263	Ints14	498,9082239	718,3601394	455,7729563	423,2328239	875,5819387	-0,23732293	False	0,23732293
ENSMUSG00000048379	Socs4	445,6585776	525,3248613	410,7447847	427,7233579	340,5040873	0,237270989	False	0,237270989
ENSMUSG00000032870	Smap2	1566,358824	1589,419827	1328,880186	1392,065522	2194,886974	-0,237203711	False	0,237203711
ENSMUSG00000052917	Senp7	339,1592852	140,2146796	287,7409989	258,2057016	55,76199339	-0,237192191	False	0,237192191
ENSMUSG00000026674	Ddr2	1365,648619	771,1807379	1158,651732	791,4566071	631,1783081	-0,237139341	False	0,237139341
ENSMUSG00000030245	Golt1b	419,4433672	564,7002165	411,8430328	355,8748148	766,4308028	-0,237106195	False	0,237106195
ENSMUSG00000030930	Chst15	970,7820119	2485,449252	823,6860656	1315,726445	2718,100571	-0,237052794	False	0,237052794
ENSMUSG00000031557	Plekha2	2064,447823	839,3673287	1751,705699	1219,179965	575,4163148	-0,236995545	False	0,236995545
ENSMUSG00000032481	Smarcc1	761,0603284	554,1360968	645,7698754	590,5052132	555,2470831	-0,236990677	False	0,236990677

ENSMUSG00000000916	Nsun5	245,767598	369,7441894	403,0570481	289,6394392	347,6226396	0,236963339	False	0,236963339
ENSMUSG00000020532	Acaca	1028,94701	611,7585679	873,1072295	852,0788153	742,7022949	-0,236937934	False	0,236937934
ENSMUSG00000038241	Cep250	453,8508309	277,5482357	534,8468186	405,2706882	252,7086083	0,236907561	False	0,236907561
ENSMUSG00000026240	Cops7b	385,8551288	345,7348265	454,6747082	413,1291226	266,945713	0,236775473	False	0,236775473
ENSMUSG00000029463	Fam216a	406,335762	596,3925756	344,8498994	470,3834303	564,7384862	-0,236703797	False	0,236703797
ENSMUSG00000019782	Rwdd1	338,3400599	602,1548227	398,6640557	454,6665615	686,9403015	0,236699531	False	0,236699531
ENSMUSG00000020476	Dbnl	1585,201007	837,4465796	1345,353907	745,4286342	522,0271722	-0,236680053	False	0,236680053
ENSMUSG00000038235	F11r	3501,369046	3454,467141	3347,46017	2971,610835	4328,079827	-0,236673939	False	0,236673939
ENSMUSG00000059013	Sh2d3c	1779,357409	2722,661758	2096,555599	3148,986926	2473,696941	0,236664764	False	0,236664764
ENSMUSG00000034083	Ccdc174	290,8249909	405,2780466	342,6534033	444,5628602	595,5855464	0,236598769	False	0,236598769
ENSMUSG00000038335	Tsr1	236,7561194	350,536699	228,4356022	200,9513939	424,7402901	-0,236555131	False	0,236555131
ENSMUSG00000055720	Ubl7	1291,099115	2184,852028	1521,073601	1784,987242	2417,934947	0,236490206	False	0,236490206
ENSMUSG00000019715	Gle1	1085,473558	1592,300951	921,4301453	1661,497558	2772,676139	-0,236377879	False	0,236377879
ENSMUSG00000021661	Ankra2	285,909639	506,117371	349,2428918	336,7900456	430,672417	0,236290239	False	0,236290239
ENSMUSG00000058297	Spock2	12179,42293	7269,074726	7549,357353	10122,78614	10339,69728	-0,23625183	False	0,23625183
ENSMUSG00000024769	Cdc42bpg	3172,040464	1874,651059	2692,90431	2324,973948	2261,326796	-0,236248209	False	0,236248209
ENSMUSG00000009090	Ap1b1	974,0589133	1026,64036	826,9808098	836,3619465	1447,438977	-0,23615518	False	0,23615518
ENSMUSG00000038844	Kif16b	516,1119557	322,685838	438,2009869	396,2896203	342,8769381	-0,236091318	False	0,236091318
ENSMUSG00000030087	Klf15	519,388857	1772,85136	611,7241847	2382,228256	2179,463444	0,236066252	False	0,236066252
ENSMUSG00000005547	Cyp2a5	706,9914568	4017,246609	1185,009686	5057,463851	600,331248	-0,235934018	False	0,235934018
ENSMUSG00000020485	Supt4a	567,7231513	709,7167688	482,1309104	562,4393761	1092,697785	-0,235762648	False	0,235762648
ENSMUSG00000022453	Naga	1110,869543	1255,209495	943,3951071	1111,40715	1674,046227	-0,235755377	False	0,235755377
ENSMUSG00000041241	Mul1	362,0975943	453,2967725	307,5094645	312,0921089	565,9249116	-0,235747778	False	0,235747778
ENSMUSG00000031954	Cfdp1	734,0258926	644,4113015	864,3212448	660,1084893	732,0244664	0,235736668	False	0,235736668
ENSMUSG00000048696	Mex3d	302,2941455	255,4596218	241,6145792	230,1398645	355,9276174	0,235630841	False	0,235630841
ENSMUSG00000042178	Armc5	717,641386	993,9876261	609,5276885	684,806426	1715,571116	-0,235571334	False	0,235571334
ENSMUSG00000022350	Washc5	868,3788461	654,0150467	709,4682645	737,5701998	481,6887088	-0,235544215	False	0,235544215
ENSMUSG00000009647	Mcu	582,4692072	1233,120881	540,338059	719,608064	494,7393882	-0,23551302	False	0,23551302
ENSMUSG00000027706	Sec62	3563,63017	4194,915894	4732,351009	6169,993635	4342,316932	0,235294262	False	0,235294262
ENSMUSG00000042379	Esm1	223,6485141	449,4552744	189,9969191	534,373539	681,0081746	-0,235257149	False	0,235257149
ENSMUSG00000033768	Nrxn2	208,0832329	267,9444905	189,9969191	310,9694754	176,7773833	-0,23522701	False	0,23522701
ENSMUSG00000079477	Rab7	6548,88726	8319,724449	7708,603326	7776,482152	10000,37962	0,235219696	False	0,235219696
ENSMUSG00000027329	Spef1	576,7346299	580,0662088	745,7104514	760,0228695	489,9936866	-0,235144491	False	0,235144491
ENSMUSG00000049658	Bdp1	431,7317471	329,4084597	366,8148612	364,8558827	336,9448111	-0,235083095	False	0,235083095
ENSMUSG00000027253	Lrp4	1976,790713	1679,695032	2232,738362	2440,605197	1732,181071	-0,234960819	False	0,234960819
ENSMUSG00000034928	Rnf44	3348,17391	2224,227384	2411,7528	2079,117215	2845,048088	-0,234921428	False	0,234921428
ENSMUSG00000041375	Ccdc9	729,9297659	1032,402607	858,8300044	1191,114128	1679,978354	0,23461494	False	0,23461494
ENSMUSG00000043621	Ubxn10	1292,737565	1481,857881	736,9244667	1007,002236	1520,997352	0,234588213	False	0,234588213
ENSMUSG00000063870	Chd4	3585,749254	4181,470651	3047,638443	4111,083823	5584,504317	-0,234582848	False	0,234582848
ENSMUSG00000038705	Gmeb2	542,3271662	856,65407	638,0821388	880,1446524	1356,084222	0,234578711	False	0,234578711
ENSMUSG00000032402	Smad3	987,9857438	1211,032267	958,7705803	839,729847	1606,41998	-0,234564957	False	0,234564957
ENSMUSG00000023068	Nus1	841,3444104	1195,666275	786,3456306	715,1175301	2254,208243	-0,23451613	False	0,23451613
ENSMUSG00000036513	Commd2	341,6169612	506,117371	472,2466976	401,9027877	588,4669941	0,234466978	False	0,234466978
ENSMUSG00000024132	Eci1	427,6356205	290,9934789	363,5201169	281,7810048	228,9801005	-0,234346825	False	0,234346825
ENSMUSG00000028684	Urod	457,1277322	452,3363979	463,4606929	537,7414394	294,233497	0,234315308	False	0,234315308
ENSMUSG00000040652	Oaz2	4245,225642	2576,684832	3608,843215	2829,036383	2716,914146	-0,234304771	False	0,234304771
ENSMUSG00000063808	Gpatch1	414,5280152	612,7189424	487,6221508	664,5990233	537,4507022	0,234294056	False	0,234294056
ENSMUSG00000039678	Tbc1d13	1233,753342	702,0337726	1048,826923	795,947141	637,1104351	-0,234277367	False	0,234277367
ENSMUSG00000022912	Pros1	1300,929819	1000,710248	1105,935824	1015,983304	979,9873732	-0,234275465	False	0,234275465
ENSMUSG00000033228	Scaf11	1612,235443	2171,406785	1370,613613	2026,353441	3155,891541	-0,234240522	False	0,234240522
ENSMUSG00000006850	Tmco6	300,6556948	182,4711584	353,6358841	321,0731768	92,54118052	0,23415239	False	0,23415239
ENSMUSG00000031465	Angpt2	507,9197025	361,1008187	411,8430328	242,4888328	431,8588424	-0,234040608	False	0,234040608
ENSMUSG00000061904	Slc25a3	4366,47099	8178,549395	5135,408057	6643,744966	8847,174143	0,234011162	False	0,234011162
ENSMUSG00000030084	Plxna1	4204,264376	1460,729642	3574,797525	1890,514789	1080,833531	-0,233991862	False	0,233991862
ENSMUSG00000020228	Helb	176,9526705	307,3198457	150,459988	251,4699007	571,8570386	-0,233983655	False	0,233983655
ENSMUSG00000002204	Napsa	5780,453904	8063,304453	6798,155661	7934,773474	6801,776768	0,233960613	False	0,233960613
ENSMUSG00000091537	Tma7	798,7446934	816,3183402	810,5070885	679,1932586	1235,068832	-0,233912302	False	0,233912302
ENSMUSG00000040006	Ginm1	887,2210286	1353,167696	1043,335683	1220,302598	1944,551216	0,23383794	False	0,23383794
ENSMUSG00000028841	Cnksr1	513,6542797	1307,069719	604,0364481	838,6072135	2305,224535	0,23383794	False	0,23383794
ENSMUSG00000057286	St6galnac2	573,4577286	325,5669616	674,3243257	524,2698376	272,87784	0,233755498	False	0,233755498
ENSMUSG00000022681	Ntan1	786,4563135	689,5489039	924,7248896	734,2022994	685,7538761	0,233657592	False	0,233657592
ENSMUSG00000034292	Traf3ip1	835,6098331	710,6771433	690,798047	670,2121907	523,2135975	-0,233635169	False	0,233635169
ENSMUSG00000032127	Vps11	793,0101161	1599,983947	932,4126262	1178,76516	2269,631773	0,233629271	False	0,233629271
ENSMUSG00000045664	Cdc42ep2	729,9297659	671,301788	808,3105923	620,8163173	832,8706247	-0,233591177	False	0,233591177
ENSMUSG00000018427	Ypel2	356,363017	235,2917569	303,1164721	257,0830681	259,8271607	-0,233475369	False	0,233475369
ENSMUSG00000034064	Poglut1	636,5380787	305,3990967	541,4363071	418,74229	319,1484302	-0,233455187	False	0,233455187
ENSMUSG00000026341	Actr3	3484,165314	3929,852527	3646,18365	2963,752401	4146,556742	-0,233388151	False	0,233388151
ENSMUSG00000028234	Rps20	3452,215526	6293,334216	4058,026683	5557,035752	8430,73883	0,233255815	False	0,233255815
ENSMUSG00000007833	Aldh16a1	326,05168	456,177896	432,7097464	542,2319734	383,2154014	0,233054891	False	0,233054891
ENSMUSG00000028701	Lurap1	264,6097805	154,6202974	225,1408579	209,9324617	189,8280626	-0,233038491	False	0,233038491
ENSMUSG00000034088	Hdlbp	4332,882752	5930,312648	3686,818829	4584,835154	9426,149734	-0,232950674	False	0,232950674
ENSMUSG00000049436	Upk1b	805,298496	354,3781971	534,8468186	946,3800281	554,0606577	0,232895989	False	0,232895989
ENSMUSG00000009863	Sdhb	865,9211701	1617,270688	736,9244667	1057,520743	1656,249846	-0,232718941	False	0,232718941
ENSMUSG00000056973	Ces1d	7066,637667	8430,167518	6014,006527	7979,678813	9055,985011	0,232697505	False	0,232697505
ENSMUSG00000029599	Ddx54	877,3903247	1567,331213	746,8086994	990,162734	2712,168444	-0,232480064	False	0,232480064
ENSMUSG00000042729	Wdr74	304,7518215	587,749205	358,0288765	428,8459914	1073,714979	0,232441108	False	0,232441108
ENSMUSG00000040111	Gramd1b	603,7690657	816,3183402	513,9801049	794,8245076	800,8371391	-0,232284325	False	0,232284325
ENSMUSG00000028238	Atp6v0d2	419,4433672	397,5950504	539,2398109	554,5809417	357,1140428	-0,232091167	False	0,232091167
ENSMUSG00000044224	Dnajc21	277,7173857	415,8421663	326,179682	436,7044258	621,686905	0,232049422	False	0,232049422
ENSMUSG00000031485	Plpbp	760,241103	1174,538035	892,8756951	1113,652417	1129,476972	0,232002312	False	0,232002312
ENSMUSG00000045598	Zfp553	285,909639	246,8162511	265,7760372	245,8567333	335,7583857	0,231864173	False	0,231864173
ENSMUSG00000003528	Slc25a1	1148,553908	897,9501743	1348,648651	815,0319103	816,2606692	0,231695976	False	0,231695976
ENSMUSG00000063694	Cycs	291,6442162	441,7722782	374,5025978	342,403213	397,4525061	0,231486776	False	0,231486776
ENSMUSG00000032120	C2cd2l	725,8336393	901,7916723	879,696718	852,0788153	936,0896337	0,231347961	False	0,231347961
ENSMUSG00000028159	Dapp1	242,4906967	218,9653901	242,7128273	206,5645613	370,1647221	-0,231336635	False	0,231336635
ENSMUSG00000030214	Plbd1	974,0589133	829,7635835	612,8224658	518,6566702	531,5185753	-0,23130869	False	0,23130869
ENSMUSG00000035805	Mlc1	734,8451179	348,61595	626,0014098	610,712616	250,3357576	-0,231274301	False	0,231274301
ENSMUSG00000034312	Iqsec1	2572,367525	2033,112854	1988,927286	2191,380563	1745,231751	-0,231256732	False	0,231256732
ENSMUSG00000026490	Cdc42bpa	2442,929924	2005,261993	1963,66758	2867,205921	2165,226339	0,231033042	False	0,231033042
ENSMUSG00000041453	Rpl21	5887,772422	10463,28037	6910,176966	9470,536082	13581,01145	0,231000749	False	0,231000749
ENSMUSG00000020745	Pafah1b1	2661,663086	3004,051492	2267,882301	2378,860355	4379,096119	-0,230982197	False	0,230982197
ENSMUSG00000033350	Chst2	466,9584361	217,0446411	548,0257956	205,4419278	168,4724056	0,230949661	False	0,230949661
ENSMUSG00000058835	Abi1	1868,65297	2618,94131	1592,459727	2122,899921	3117,925928	-0,230741782	False	0,230741782
ENSMUSG00000028343	Erp44	910,9785631	867,2181897	877,5002218	776,8623718	1068,969278	0,23073138	False	0,23073138
ENSMUSG00000043241	Upf2	679,1377957	989,1857535	578,7767421	1054,152843	1269,475169	-0,230697375	False	0,230697375
ENSMUSG00000049755	Zfp672	420,2625925	334,2103322	493,1133913	351,3842809	186,2687864	0,230628384	False	0,230628384
ENSMUSG00000063060	Sox7	2134,081976	484,028757	2504,005639	504,0624349	450,8416487	0,230622217	False	0,230622217
ENSMUSG00000020263	Appl2	1922,721841	3231,660253	2255,801572	4181,809733	3501,14133	0,230490104	False	0,230490104
ENSMUSG00000027523	Gnas	13522,95247	15865,38704	17088,74024	16522,91964	20173,97735	0,230472547	False	0,230472547
ENSMUSG00000047735	Samd9l	1363,190943	581,9869579	1161,946476	685,9290595	276,4371162	-0,230444041	False	0,230444041
ENSMUSG00000029536	Gatc	537,4118142	530,1267339	630,3944022	522,0245706	401,0117822	0,230226687	False	0,230226687
ENSMUSG00000032352	Lrrc1	533,3156876	273,7067376	454,6747082	359,2427153	259,8271607	-0,230155011	False	0,230155011
ENSMUSG00000050994	Adgb	362,0975943	267,9444905	252,5970601	308,7242084	281,1828177	-0,230059976	False	0,230059976
ENSMUSG00000022718	Dgcr8	466,1392108	624,2434367	487,6221508	667,9669237	397,4525061	-0,2299784	False	0,2299784
ENSMUSG00000038910	Plcl2	222,8292888	262,1822434	189,9969191	191,970326	319,1484302	-0,229962849	False	0,229962849
ENSMUSG00000091811	Inafm1	575,9154046	931,5632824	675,4225738	879,0220189	1676,419078	0,229933486	False	0,229933486
ENSMUSG00000054065	Pkp3	249,8637246	348,61595	213,060129	281,7810048	393,8932299	-0,229880826	False	0,229880826
ENSMUSG00000028600	Podn	767,614131	213,203143	654,5558601	483,8550321	603,8905241	-0,229864953	False	0,229864953
ENSMUSG00000027447	Cst3	8807,491486	8008,563105	8355,471449	7510,418016	11825,10187	-0,229837967	False	0,229837967
ENSMUSG00000019929	Dcn	1841,618534	2984,844002	1570,494765	4927,238367	3527,242688	-0,229755124	False	0,229755124

ENSMUSG00000022452	Smdt1	982,2511665	1312,831966	1253,101068	1151,821956	1553,030837	0,229753847	False	0,229753847
ENSMUSG00000041084	Ostc	1052,704545	797,1108499	1234,43085	647,759521	743,8887203	0,229745441	False	0,229745441
ENSMUSG00000011096	Akt1s1	947,8437028	1095,787325	808,3105923	967,7100643	1535,234456	-0,229739428	False	0,229739428
ENSMUSG00000034525	Ice1	706,9914568	1082,342082	602,9382	1041,803874	1837,772931	-0,229682646	False	0,229682646
ENSMUSG00000025786	Zdhhc3	3294,924263	2810,055839	2680,823581	2758,310473	2612,508712	-0,229646505	False	0,229646505
ENSMUSG00000020922	Lsm12	635,7188534	581,0265833	646,8681235	542,2319734	842,3620278	-0,229478689	False	0,229478689
ENSMUSG00000020720	Psmd12	587,3845591	1143,806051	688,6015508	856,5693492	1385,744857	0,229364089	False	0,229364089
ENSMUSG00000052031	Tagap1	285,909639	335,1707068	414,039529	395,1669868	436,604544	0,229336807	False	0,229336807
ENSMUSG00000027276	Jag1	560,3501234	656,8961703	741,317459	815,0319103	876,7683641	0,229336807	False	0,229336807
ENSMUSG00000033735	Spr	516,931181	605,9963208	678,717318	619,6936839	608,6362257	0,229336807	False	0,229336807
ENSMUSG00000046637	Ttc34	247,4060486	168,0655406	199,8811519	211,0550952	98,47330747	-0,229261113	False	0,229261113
ENSMUSG00000021236	Entpd5	964,2282093	2048,478847	822,5878175	1790,600409	1428,456171	-0,229204932	False	0,229204932
ENSMUSG00000030255	Sspn	531,6772369	152,6995484	453,5764601	310,9694754	77,11765043	-0,229204932	False	0,229204932
ENSMUSG00000032312	Csk	1730,20389	1104,430696	1476,045429	1024,964372	1184,05254	-0,229204932	False	0,229204932
ENSMUSG00000020101	Vsir	2506,010274	1761,326866	1588,066734	1499,838336	2137,938555	-0,229171939	False	0,229171939
ENSMUSG00000039929	Urb1	192,5179517	348,61595	263,579541	225,6493305	569,4841878	0,229089519	False	0,229089519
ENSMUSG00000037697	Ddhd1	846,2597623	1036,244105	1066,398893	1210,198897	991,8516271	0,22902375	False	0,22902375
ENSMUSG00000022199	Slc22a17	527,5811103	224,7276372	618,3136732	499,5719009	221,8615482	-0,228945999	False	0,228945999
ENSMUSG00000006519	Cyba	1346,806437	2062,884465	1658,354612	1578,42268	2101,159368	0,22894107	False	0,22894107
ENSMUSG00000045752	Tssc4	371,1090729	776,942985	434,9062426	508,5529688	964,5638431	0,228861145	False	0,228861145
ENSMUSG00000004937	Sgta	1837,522407	2153,159669	2423,833529	2245,26697	2253,021818	0,228693465	False	0,228693465
ENSMUSG00000036553	Sh3tc1	936,3745482	1580,776457	1097,149839	1724,365033	1987,26253	0,228602944	False	0,228602944
ENSMUSG00000055172	C1ra	1045,331517	626,1641857	709,4682645	1224,793132	1119,985569	0,228577547	False	0,228577547
ENSMUSG00000003813	Rad23a	1219,826511	594,4718266	1041,139187	726,343865	596,7719718	-0,228513025	False	0,228513025
ENSMUSG00000024325	Ring1	519,388857	327,4877106	608,4294404	432,2138918	269,3185638	0,228274901	False	0,228274901
ENSMUSG00000031642	Sh3rf1	498,9082239	259,3011198	354,7341322	288,5168057	425,9267155	-0,228169227	False	0,228169227
ENSMUSG00000014856	Tmem208	421,0818178	411,0402937	602,9382	478,2418647	359,4868936	-0,228161409	False	0,228161409
ENSMUSG00000030873	Scnn1b	3401,423556	2657,356291	3422,14104	3984,226239	2669,45713	0,228160905	False	0,228160905
ENSMUSG00000055491	Pprc1	575,9154046	691,4696529	691,8962951	491,7134665	1509,133098	-0,228039045	False	0,228039045
ENSMUSG00000074030	Exoc8	453,8508309	370,7045639	531,5520743	491,7134665	163,726704	0,227992836	False	0,227992836
ENSMUSG00000028923	Necap2	1413,163688	1955,322519	1655,059868	1831,015214	2452,341284	0,22795482	False	0,22795482
ENSMUSG00000021910	Nisch	7425,45836	6340,392567	8156,688545	8555,589791	7517,191279	-0,227907915	False	0,227907915
ENSMUSG00000067235	H2-Q10	239,2137953	418,7232898	204,2741443	254,8378012	441,3502455	-0,227793983	False	0,227793983
ENSMUSG00000003345	Csnk1g2	3159,752084	3536,098975	2698,395551	3369,023089	4861,971253	-0,227709524	False	0,227709524
ENSMUSG00000066800	Rnasel	565,2654753	264,1029924	367,9131093	482,7323987	252,7086083	-0,227704928	False	0,227704928
ENSMUSG00000021890	Eaf1	842,982861	699,1526491	776,4613978	413,1291226	987,1059255	0,227701608	False	0,227701608
ENSMUSG00000026245	Farsb	507,9197025	381,2686836	433,8079945	316,5826428	365,4190205	-0,227543798	False	0,227543798
ENSMUSG00000001380	Hars	629,9842761	893,1483017	741,317459	737,5701998	1007,275157	0,227464547	False	0,227464547
ENSMUSG00000004633	Chn2	245,767598	312,1217183	270,1690295	209,9324617	329,8262588	-0,227369456	False	0,227369456
ENSMUSG00000033467	Crlf2	602,9498403	1115,95519	515,078353	547,8451408	1636,080615	-0,227246079	False	0,227246079
ENSMUSG00000054814	Usp46	946,2052521	353,4178226	808,3105923	491,7134665	342,8769381	-0,227243416	False	0,227243416
ENSMUSG00000036478	Btg1	2292,192464	3858,784813	1958,17634	2432,746763	7135,162303	-0,227217495	False	0,227217495
ENSMUSG00000000600	Krit1	671,7647678	1017,996989	786,3456306	904,8425891	1032,19009	0,227207442	False	0,227207442
ENSMUSG00000060743	H3f3a	3820,047698	4076,789829	3597,860734	3263,495542	4902,309717	-0,227172584	False	0,227172584
ENSMUSG00000009640	Dmap1	309,6671734	485,9495061	362,4218688	434,4591588	603,8905241	0,226951555	False	0,226951555
ENSMUSG00000029434	Vps33a	746,3142725	637,6886799	534,8468186	559,0714757	533,8914261	-0,226931005	False	0,226931005
ENSMUSG00000021947	Cryl1	289,1865403	125,8090618	247,1058197	179,6213576	125,7610915	-0,226871417	False	0,226871417
ENSMUSG00000024542	Cep192	354,7245664	391,8328033	336,0639148	303,111041	435,4181186	-0,226852843	False	0,226852843
ENSMUSG00000062867	Impdh2	598,8537137	746,2110004	511,7836087	602,8541816	807,9556914	-0,226669687	False	0,226669687
ENSMUSG00000052144	Ppp4r2	566,903926	823,0409619	663,3418448	781,3529057	998,9701794	0,226648276	False	0,226648276
ENSMUSG00000027669	Gnb4	562,8077993	125,8090618	481,0326623	278,4131043	68,81267269	-0,226507465	False	0,226507465
ENSMUSG00000034748	Sirt6	317,8594267	317,8839654	385,4850787	271,6773034	387,9611029	-0,22649483	False	0,22649483
ENSMUSG00000031145	Prickle3	407,9742126	477,3061354	553,5170361	674,7027246	494,7393882	0,226436917	False	0,226436917
ENSMUSG00000004100	Ppan	274,4404844	541,6512281	382,1903344	321,0731768	1235,068832	0,226408826	False	0,226408826
ENSMUSG00000017837	Nkiras2	754,5065257	635,7679309	484,3274066	499,5719009	882,7004911	0,226390622	False	0,226390622
ENSMUSG00000028452	Vcp	3619,337493	4502,23574	3093,764862	3259,005008	5418,404762	-0,22636209	False	0,22636209
ENSMUSG00000044221	Grsf1	827,4175798	1048,728974	707,2717683	967,7100643	1302,69508	-0,226350935	False	0,226350935
ENSMUSG00000030800	Prss8	2102,951413	2739,9485	2460,075716	2954,771333	2688,439937	0,226287201	False	0,226287201
ENSMUSG00000040936	Ulk4	335,8823839	224,7276372	264,6777891	392,9217198	220,6751228	0,226285787	False	0,226285787
ENSMUSG00000064373	Selenop	18108,15662	16219,76523	17860,80865	21181,8486	14438,79701	0,226188813	False	0,226188813
ENSMUSG00000048285	Frmd6	1024,031658	663,6187919	1141,079763	1197,849929	526,7728737	0,226186856	False	0,226186856
ENSMUSG00000037465	Klf10	829,0560305	708,7563942	984,0302863	973,3232317	791,345736	-0,226179762	False	0,226179762
ENSMUSG00000034342	Cbl	1368,106295	862,4163171	1169,634213	884,6351864	836,4299008	-0,226122908	False	0,226122908
ENSMUSG00000030120	Mlf2	4351,724935	5472,214003	6108,455862	5090,020222	6174,157736	0,226084019	False	0,226084019
ENSMUSG00000038697	Taf5l	321,136328	582,9473324	274,5620219	370,4690501	1000,156605	-0,226053795	False	0,226053795
ENSMUSG00000017999	Ddx27	425,1779445	704,9148962	363,5201169	480,4871317	1124,731271	-0,226031557	False	0,226031557
ENSMUSG00000019433	Gipc1	1085,473558	898,9105488	1035,647946	803,8055754	1269,475169	0,225907594	False	0,225907594
ENSMUSG00000048445	Ccdc57	192,5179517	450,4156489	225,1408579	450,1760276	601,5176734	0,225834918	False	0,225834918
ENSMUSG00000034751	Mast4	748,7719485	470,5835138	640,278635	424,3554574	554,0606577	-0,225826517	False	0,225826517
ENSMUSG00000027406	Idh3b	1264,064679	1762,28724	1478,241926	1560,460544	1665,741249	0,225812113	False	0,225812113
ENSMUSG00000018334	Ksr1	445,6585776	539,7304791	381,0920863	409,7612221	727,2787648	-0,225799224	False	0,225799224
ENSMUSG00000044308	Ubr3	1309,941297	1760,366491	1120,213049	1470,649866	1652,69057	-0,225729022	False	0,225729022
ENSMUSG00000002524	Puf60	1689,224623	1781,494731	1535,350826	1444,829296	2655,220026	-0,225477505	False	0,225477505
ENSMUSG00000063171	Rps4l	314,5825254	477,3061354	269,0707814	316,5826428	493,5529628	-0,225452797	False	0,225452797
ENSMUSG00000019877	Serinc1	3192,521098	2846,550071	1975,748309	2368,756654	3732,494281	0,225443896	False	0,225443896
ENSMUSG00000026836	Acvr1	440,7432257	540,6908536	529,3555781	515,2887697	703,550257	0,225442755	False	0,225442755
ENSMUSG00000022841	Ap2m1	1957,94853	1674,893159	1557,315788	1512,187305	1537,607307	-0,225273771	False	0,225273771
ENSMUSG00000018882	Mrpl45	339,1592852	437,9307802	396,4675596	442,3175932	410,5031854	0,225239834	False	0,225239834
ENSMUSG00000031403	Dkc1	283,451963	263,1426179	364,618365	242,4888328	314,4027287	-0,225185951	False	0,225185951
ENSMUSG00000019841	Rev3l	498,0889985	621,3623131	426,1202579	582,6467788	611,0090765	-0,225142907	False	0,225142907
ENSMUSG00000038181	Chpf2	729,1105406	1178,379534	852,2405158	875,6541185	1333,54214	0,225123081	False	0,225123081
ENSMUSG00000039068	Zzz3	799,5639187	897,9501743	684,2085585	770,1265709	1067,782852	-0,22477722	False	0,22477722
ENSMUSG00000037736	Limch1	11388,87049	4733,685999	9745,853528	9047,303258	3619,783869	-0,224764227	False	0,224764227
ENSMUSG00000032249	Anp32a	3931,462342	1612,468816	4593,97175	2744,838871	1356,084222	0,22467595	False	0,22467595
ENSMUSG00000031960	Aars	1237,849468	2232,870754	1446,392731	1755,798771	2474,883366	0,22462345	False	0,22462345
ENSMUSG00000032553	Srprb	1128,073275	1159,172043	1118,016553	855,4467158	1318,11861	0,224619414	False	0,224619414
ENSMUSG00000041012	Cmtm8	899,5094085	581,9869579	769,8719093	602,8541816	760,4986758	-0,22451994	False	0,22451994
ENSMUSG00000021684	Pde8b	861,0058182	256,4199963	736,9244667	501,8171679	187,4552118	-0,224506233	False	0,224506233
ENSMUSG00000066037	Hnrnpr	1044,512291	894,1086762	1082,872614	1038,435974	1220,831728	-0,224307367	False	0,224307367
ENSMUSG00000028669	Pithd1	446,477803	759,6562437	382,1903344	592,7504802	740,3294441	-0,224297159	False	0,224297159
ENSMUSG00000040331	Nsmce4a	598,8537137	993,0272515	699,5840317	880,1446524	1217,272451	0,224293731	False	0,224293731
ENSMUSG00000025584	Pde8a	566,903926	981,5027573	662,2435967	919,4368244	1033,376516	0,224257729	False	0,224257729
ENSMUSG00000037580	Gch1	197,4333037	607,9170699	230,6320984	281,7810048	994,2244779	0,224227946	False	0,224227946
ENSMUSG00000045917	Tmem268	368,6513969	379,3479346	425,0220098	408,6385886	315,5891541	-0,224209599	False	0,224209599
ENSMUSG00000074746	Pdzd8	897,8709579	920,9991627	532,6503224	690,4195934	1048,800046	0,224159633	False	0,224159633
ENSMUSG00000034248	Slc25a37	1558,985796	2212,702889	1820,895329	2121,777287	2117,769323	0,22404021	False	0,22404021
ENSMUSG00000040035	Disp2	906,8824365	314,0424674	776,4613978	140,3291857	245,590056	-0,224001337	False	0,224001337
ENSMUSG00000003119	Cdk12	605,4075163	587,749205	518,3730973	574,7883444	893,3783196	-0,22391574	False	0,22391574
ENSMUSG00000079562	Maea	1325,506578	1298,426348	1246,511579	1134,982454	1852,010036	-0,223873836	False	0,223873836
ENSMUSG00000073436	Eme2	184,3256985	287,1519809	215,2566251	343,5258465	231,3529513	0,223800418	False	0,223800418
ENSMUSG00000020986	Sec23a	394,8666074	219,9257646	322,8849377	273,9225704	338,1312365	-0,223782071	False	0,223782071
ENSMUSG00000027679	Dnajc19	376,8436502	463,8608922	492,0151432	434,4591588	322,7077064	-0,223738053	False	0,223738053
ENSMUSG00000042759	Apobr	421,0818178	491,7117532	374,5025978	425,4780909	276,4371162	0,223712258	False	0,223712258
ENSMUSG00000049571	Cfap46	578,3730805	264,1029924	495,3098874	405,2706882	230,1665259	-0,223668985	False	0,223668985
ENSMUSG00000024539	Ptpn2	253,9598512	664,5791664	296,5269836	378,3274845	1333,54214	0,223562956	False	0,223562956
ENSMUSG00000060568	Fam78b	253,9598512	92,19595372	296,5269836	171,7629232	97,28688208	0,223562956	False	0,223562956
ENSMUSG00000032458	Copb2	2082,47078	2768,759735	1783,554894	2006,146038	3903,339537	-0,223540633	False	0,223540633
ENSMUSG00000034616	Ssh3	1034,681587	1986,054503	1208,072896	1649,14859	2869,963021	0,223520649	False	0,223520649
ENSMUSG00000026773	Pfkfb3	926,5438443	3883,754551	1081,774366	2274,455441	5013,833703	0,223468465	False	0,223468465
ENSMUSG00000034022	Cpsf1	1123,157923	1754,604244	1311,308216	1619,960119	3033,689725	0,22344603	False	0,22344603

ENSMUSG00000030265	Kras	1477,063264	2337,551577	1724,249497	2012,881839	2253,021818	0,223236927	False	0,223236927
ENSMUSG00000033364	Usp37	303,9325961	261,2218689	282,2497585	220,0361631	354,741192	0,223015454	False	0,223015454
ENSMUSG00000027176	Cstf3	398,962734	384,1498072	465,6571891	359,2427153	351,1819158	0,223014257	False	0,223014257
ENSMUSG00000036644	Tbc1d9b	2190,608523	1231,200132	1876,905981	1463,914065	1369,134902	-0,222975304	False	0,222975304
ENSMUSG00000076437	Selenoh	185,1449238	216,0842665	277,8567661	259,3283351	262,2000115	0,222939036	False	0,222939036
ENSMUSG00000026975	Dph7	225,2869648	193,0352781	289,9374951	245,8567333	234,9122275	-0,22289931	False	0,22289931
ENSMUSG00000028180	Zranb2	961,7705334	589,669954	1122,409545	670,2121907	332,1991096	0,222834552	False	0,222834552
ENSMUSG00000026303	Mlph	588,2037845	401,4365485	431,6114984	504,0624349	219,4886974	-0,222713625	False	0,222713625
ENSMUSG00000058446	Znrf2	817,5868759	634,8075564	700,6822798	505,1850684	671,5167715	-0,222611627	False	0,222611627
ENSMUSG00000029413	Naaa	1396,779182	852,8125719	1197,090415	734,2022994	975,2416716	-0,22257184	False	0,22257184
ENSMUSG00000047843	Bri3	2017,751979	2745,710747	2714,869272	2354,162419	4337,57123	0,222465008	False	0,222465008
ENSMUSG00000005804	Bloc1s6	349,8092144	238,1728804	299,8217279	237,9982989	187,4552118	-0,222463354	False	0,222463354
ENSMUSG00000042810	Krba1	507,1004771	515,7211161	562,3030208	591,6278467	316,7755795	0,222418327	False	0,222418327
ENSMUSG00000042548	Asxl1	1110,869543	1898,660422	952,1810918	1630,063821	3597,241786	-0,222381515	False	0,222381515
ENSMUSG00000064120	Mocs1	661,1148385	1955,322519	566,6960131	1488,612001	1894,72135	-0,222325843	False	0,222325843
ENSMUSG00000024030	Abcg1	1463,136433	607,9170699	1254,199316	854,3240823	773,5493551	-0,222297665	False	0,222297665
ENSMUSG00000024190	Dusp1	3603,772211	10164,6039	3089,37187	6393,397698	12395,77249	-0,222194284	False	0,222194284
ENSMUSG00000046311	Zfp62	443,2009017	339,0122048	379,9938382	323,3184438	242,0307798	-0,221984792	False	0,221984792
ENSMUSG00000007892	Rplp1	10606,5103	18174,12738	12370,66646	14611,07481	26732,53692	0,22197316	False	0,22197316
ENSMUSG00000037280	Galnt6	320,3171027	373,5856875	255,8918044	308,7242084	282,3692431	0,221938355	False	0,221938355
ENSMUSG00000017314	Mpp2	453,8508309	392,7931778	766,577165	539,9867064	389,1475283	-0,221901005	False	0,221901005
ENSMUSG00000039043	Arpin	434,1894231	181,5107839	372,3061016	191,970326	151,8624501	-0,221835322	False	0,221835322
ENSMUSG00000041278	Ttc1	403,0588607	524,3644868	470,0501814	526,5151046	686,9403015	0,221824246	False	0,221824246
ENSMUSG00000021357	Exoc2	629,9842761	734,6865062	475,5414219	495,081367	629,9918828	0,221812957	False	0,221812957
ENSMUSG00000003234	Abcf3	687,330049	649,2131741	798,4263596	801,5603085	261,0135861	0,221808038	False	0,221808038
ENSMUSG00000030034	Ino80b	396,505058	661,6980429	462,3624448	584,8920458	796,0914375	0,22168495	False	0,22168495
ENSMUSG00000036918	Ttc7	1407,429111	919,0784137	1206,974648	820,6450777	974,0552462	-0,221666886	False	0,221666886
ENSMUSG00000014353	Tmem87b	710,2683581	1756,524993	828,0790579	1477,385667	2304,03811	0,221404295	False	0,221404295
ENSMUSG00000002963	Pnkp	638,9957547	614,6396915	710,5665126	708,3817292	548,1285308	-0,221292116	False	0,221292116
ENSMUSG00000033669	Zfp7	235,1176687	351,4970736	309,7059607	330,0542447	201,6923165	-0,221226834	False	0,221226834
ENSMUSG00000025134	Alyref	316,220976	387,9913052	271,2672776	303,111041	811,5149676	-0,221218041	False	0,221218041
ENSMUSG00000014496	Ankrd28	430,0932964	544,5323517	369,0113574	490,5908331	646,6018382	-0,220984425	False	0,220984425
ENSMUSG00000035614	Togaram1	532,4964622	593,5114521	456,8712044	544,4772403	634,7375843	-0,220984425	False	0,220984425
ENSMUSG00000005087	Cd44	2378,211123	2784,125727	2040,544946	2053,296645	5064,849995	-0,220922306	False	0,220922306
ENSMUSG00000031834	Pik3r2	796,2870174	575,2643362	928,0196339	826,2582451	474,5701565	0,220866792	False	0,220866792
ENSMUSG00000039427	Alg1	561,988574	673,2225371	710,5665126	656,7405889	654,906816	0,220748847	False	0,220748847
ENSMUSG00000000148	Brat1	388,3128048	354,3781971	452,478212	369,3464166	234,9122275	0,220629043	False	0,220629043
ENSMUSG00000025474	Tubgcp2	391,5897061	453,2967725	336,0639148	447,9307606	524,4000229	-0,220607201	False	0,220607201
ENSMUSG00000028691	Prdx1	4078,922901	4120,967056	3925,138664	2968,242935	4752,820117	0,220595562	False	0,220595562
ENSMUSG00000038141	Tmem181a	350,6284398	433,1289076	300,919976	337,9126791	365,4190205	-0,220563139	False	0,220563139
ENSMUSG00000005813	Metap1	398,962734	519,5626142	377,7973421	342,403213	512,535769	-0,220557755	False	0,220557755
ENSMUSG00000024382	Ercc3	511,1966038	523,4041123	445,8887235	425,4780909	595,5855464	0,220430492	False	0,220430492
ENSMUSG00000033943	Mga	1033,862362	742,3695024	887,3844546	886,8804533	494,7393882	-0,220412948	False	0,220412948
ENSMUSG00000027378	Nphp1	392,4089314	457,1382705	288,839247	318,8279098	387,9611029	0,220272725	False	0,220272725
ENSMUSG00000023886	Smoc2	207,2640076	261,2218689	177,9161902	444,5628602	893,3783196	-0,220271807	False	0,220271807
ENSMUSG00000040782	Cop1	783,9986375	913,3161666	663,3418448	702,7685618	821,0063707	0,220263223	False	0,220263223
ENSMUSG00000034998	Foxn2	428,4548458	339,0122048	360,2253727	340,157946	367,7918713	-0,220253574	False	0,220253574
ENSMUSG00000025495	Ptdss2	427,6356205	324,6065871	356,9306284	367,1011497	256,2678845	-0,220204397	False	0,220204397
ENSMUSG00000002580	Mien1	506,2812518	307,3198457	589,7592229	300,865774	204,0651673	0,220187015	False	0,220187015
ENSMUSG00000037652	Phc3	1132,169401	690,5092784	971,9495574	876,776752	939,6489099	-0,220136491	False	0,220136491
ENSMUSG00000026826	Nr4a2	166,3027413	279,4689847	142,7722514	230,1398645	419,9945885	-0,22009634	False	0,22009634
ENSMUSG00000037640	Zfp60	280,994287	85,4733321	327,2779301	153,8007875	21,35565704	0,219975519	False	0,219975519
ENSMUSG00000006941	Eif1b	485,8006186	605,9963208	759,9876765	565,8072766	812,701393	0,219946403	False	0,219946403
ENSMUSG00000002602	Axl	1773,622832	1431,918406	1407,954048	2065,645613	1592,182875	0,219893516	False	0,219893516
ENSMUSG00000014907	Naf1	320,3171027	304,4387222	282,2497585	275,0452039	427,1131409	-0,219832084	False	0,219832084
ENSMUSG00000014426	Map3k4	501,3658998	206,4805214	430,5132503	252,5925342	187,4552118	-0,219806234	False	0,219806234
ENSMUSG00000016995	Matn4	903,6055352	477,3061354	584,2679825	456,9118285	775,9222059	-0,219781093	False	0,219781093
ENSMUSG00000027742	Cog6	573,4577286	515,7211161	667,7348372	561,3167426	474,5701565	0,219588166	False	0,219588166
ENSMUSG00000030284	Creld1	838,8867344	459,0590196	720,4507453	664,5990233	429,4859916	-0,219576231	False	0,219576231
ENSMUSG00000035770	Dync1li2	1185,419047	693,3904019	1018,075977	989,0401005	493,5529628	-0,219551913	False	0,219551913
ENSMUSG00000078812	Eif5a	4711,364853	7598,483186	6211,691182	5485,187209	10356,30724	0,2193958	False	0,2193958
ENSMUSG00000026837	Col5a1	818,4061012	702,9941471	836,8650426	873,4088515	894,564745	-0,219304222	False	0,219304222
ENSMUSG00000004896	Rmad1	425,9971698	529,1663594	607,3311923	535,4961725	495,9258135	0,219280476	False	0,219280476
ENSMUSG00000028191	Bcl10	421,9010432	598,3133247	362,4218688	367,1011497	1007,275157	-0,219234643	False	0,219234643
ENSMUSG00000030978	Rrm1	392,4089314	337,0914558	246,0075716	300,865774	336,9448111	-0,219217818	False	0,219217818
ENSMUSG00000021751	Acox2	290,0057656	421,6044134	280,0532623	490,5908331	249,1493322	-0,219070876	False	0,219070876
ENSMUSG00000032186	Tmod2	962,5897587	507,0777455	826,9808098	713,9948966	144,7438977	-0,219067222	False	0,219067222
ENSMUSG00000050144	Slc25a44	659,4763879	709,7167688	526,0608339	608,467349	767,6172281	0,219066086	False	0,219066086
ENSMUSG00000033902	Mapkbp1	832,3329318	978,6216338	968,6548131	1239,387368	1204,221772	0,218821923	False	0,218821923
ENSMUSG00000028633	Ctps	341,6169612	852,8125719	397,5658076	568,0525435	1611,165681	0,218814078	False	0,218814078
ENSMUSG00000075225	Ccdc162	516,1119557	527,2456103	440,3974831	600,6089146	244,4036306	0,218741837	False	0,218741837
ENSMUSG00000027423	Snx5	1234,572567	919,0784137	1060,907652	886,8804533	914,7339767	-0,218712558	False	0,218712558
ENSMUSG00000039461	Tcta	1067,4506	602,1548227	1242,118587	705,0138287	284,7420939	0,21863361	False	0,21863361
ENSMUSG00000054277	Arfgap3	746,3142725	1028,561109	641,3768831	990,162734	1091,51136	-0,21861092	False	0,21861092
ENSMUSG00000007987	Ift22	458,7661829	138,2939306	394,2710634	187,479792	85,42262817	-0,218571219	False	0,218571219
ENSMUSG00000041915	Ammecr1l	629,1650508	585,8284559	454,6747082	542,2319734	732,0244664	0,218453335	False	0,218453335
ENSMUSG00000053536	Cstf2t	533,3156876	488,8306296	537,0433147	370,4690501	620,5004796	0,21844256	False	0,21844256
ENSMUSG00000057409	Zfp53	185,9641491	289,0727299	216,3548732	239,1209324	240,8443544	0,218375097	False	0,218375097
ENSMUSG00000024426	Atat1	570,1808273	242,0143785	663,3418448	378,3274845	132,8796438	0,218333008	False	0,218333008
ENSMUSG00000039523	Cep104	791,3716654	785,5863557	568,8925093	650,004788	920,6661036	0,21832262	False	0,21832262
ENSMUSG00000032050	Rdx	3338,343206	1822,790835	2869,722252	2693,197731	2261,326796	-0,21822117	False	0,21822117
ENSMUSG00000032135	Mcam	4180,506841	4163,223535	3448,498994	3431,890564	4863,157679	0,218215505	False	0,218215505
ENSMUSG00000030707	Coro1a	2934,46512	4144,016045	2780,764157	2522,557441	3822,662611	-0,218210438	False	0,218210438
ENSMUSG00000074766	Ism1	1102,67729	1387,741178	1282,753766	1516,677839	947,9538876	-0,218121845	False	0,218121845
ENSMUSG00000024613	Tcof1	721,7375127	448,4948999	620,5101694	379,450118	442,5366709	-0,218019387	False	0,218019387
ENSMUSG00000043252	Tmem64	495,6313226	381,2686836	426,1202579	328,9316112	379,6561252	-0,218006725	False	0,218006725
ENSMUSG00000010054	Tusc2	727,47209	846,0899503	963,1635727	881,2672859	934,9032083	0,21791915	False	0,21791915
ENSMUSG00000020982	Nemf	890,4979299	1368,533688	1035,647946	1416,763458	1996,753933	0,2178495	False	0,2178495
ENSMUSG00000049305	Ccdc71	915,0746897	880,6634329	821,4895694	785,8434397	1064,223576	0,217839861	False	0,217839861
ENSMUSG00000032246	Calml4	280,994287	148,8580503	241,6145792	196,4608599	185,082361	-0,217833288	False	0,217833288
ENSMUSG00000042105	Inpp5f	327,6901306	218,0050156	381,0920863	322,1958103	187,4552118	0,217807427	False	0,217807427
ENSMUSG00000061360	Phf5a	488,2582946	690,5092784	567,7942612	619,6936839	834,0570501	0,217723714	False	0,217723714
ENSMUSG00000032042	Srpr	2328,238378	3426,61628	2002,106263	2237,408536	4252,148602	-0,217720229	False	0,217720229
ENSMUSG00000034037	Fgd5	1615,512344	783,6656066	1389,283831	1040,681241	747,4479965	-0,217650402	False	0,217650402
ENSMUSG00000069495	Epc2	761,0603284	1104,430696	654,5558601	985,6722	1940,99194	-0,217494501	False	0,217494501
ENSMUSG00000028572	Hook1	185,1449238	266,0237415	159,2459727	211,0550952	353,5547666	-0,217398108	False	0,217398108
ENSMUSG00000002981	Clptm1	2945,115049	2985,804376	2497,416151	2365,388753	3424,023679	0,217368686	False	0,217368686
ENSMUSG00000020577	Tspan13	5742,769539	840,3277032	4939,919897	1972,467034	671,5167715	-0,217259019	False	0,217259019
ENSMUSG00000031691	Tnpo2	888,040254	824,9617109	1032,353202	1003,634336	678,6353238	0,21723967	False	0,21723967
ENSMUSG00000020231	Dip2a	484,162168	352,4574481	389,878071	416,497023	219,4886974	-0,217184169	False	0,217184169
ENSMUSG00000025858	Get4	530,0387863	1188,943653	616,117177	828,5035121	1849,637185	0,217106823	False	0,217106823
ENSMUSG00000037935	Smarce1	1051,885319	1114,034441	904,956424	917,1915574	1824,722252	-0,217057195	False	0,217057195
ENSMUSG00000022615	Tymp	329,3285813	130,6109344	283,3480066	198,7061269	147,1167485	-0,216952671	False	0,216952671
ENSMUSG00000014498	Ankrd52	944,5668015	727,9638846	699,5840317	722,9759645	812,701393	-0,216927463	False	0,216927463
ENSMUSG00000026924	Sec16a	1418,07904	1496,263499	1220,153625	1384,207087	1754,723154	-0,216875143	False	0,216875143
ENSMUSG00000048833	Slc39a9	1500,001573	1290,743352	1220,153625	1145,086155	968,1231193	-0,216761846	False	0,216761846
ENSMUSG00000038700	Hoxb5	447,2970283	458,0986451	506,2923683	519,7793037	294,233497	0,216666017	False	0,216666017
ENSMUSG00000090019	Gimap1	436,647099	159,42217	507,3906164	372,7143171	51,01629182	0,216629083	False	0,216629083
ENSMUSG00000032741	Tpcn1	1794,103465	847,0503248	1544,136811	1139,472988	685,7538761	-0,216462511	False	0,216462511
ENSMUSG00000021500	Ddx46	1271,437707	1687,378028	1477,143678	1942,155929	1752,350303	0,21634938	False	0,21634938

ENSMUSG00000044502	Bod1	517,7504064	487,8702551	459,0677005	445,6854936	761,6851012	-0,216230777	False	0,216230777
ENSMUSG00000038291	Snx25	1123,977148	698,1922745	909,3494164	1305,622743	268,1321384	0,21612539	False	0,21612539
ENSMUSG00000037885	Stk35	568,5423766	582,9473324	574,3837497	489,4681996	919,4796782	-0,216052753	False	0,216052753
ENSMUSG00000006699	Cdc42	8321,690867	8150,698534	8369,748674	7164,646903	9950,549756	-0,215981091	False	0,215981091
ENSMUSG00000027080	Med19	380,1205515	319,8047145	441,4957311	369,3464166	266,945713	0,215942461	False	0,215942461
ENSMUSG00000028654	Mycl	380,1205515	342,8537029	327,2779301	475,9965977	442,5366709	-0,21594071	False	0,21594071
ENSMUSG00000032405	Pias1	1533,589811	1320,514962	1582,575494	1731,100834	1814,044423	-0,215812009	False	0,215812009
ENSMUSG00000024399	Ltb	437,4663244	175,7485368	376,699094	139,2065522	111,5239868	-0,215759401	False	0,215759401
ENSMUSG00000001441	Npepps	1372,202422	1799,741847	1593,557975	2364,26612	2340,817297	0,215758188	False	0,215758188
ENSMUSG00000078695	Cisd3	484,9813933	568,5417146	699,5840317	699,4006613	417,6217377	-0,21573259	False	0,21573259
ENSMUSG00000032308	Ulk3	242,4906967	297,7161006	306,4112164	240,2435658	208,8108689	-0,21573259	False	0,21573259
ENSMUSG00000028140	Mrpl9	312,1248494	470,5835138	362,4218688	435,5817923	685,7538761	0,215546791	False	0,215546791
ENSMUSG00000034254	Agpat1	2068,54395	1039,125228	2401,868567	1429,112427	535,0778515	0,215541597	False	0,215541597
ENSMUSG00000026409	Pfkfb2	246,5868233	279,4689847	289,9374951	287,3941722	212,370145	-0,215514743	False	0,215514743
ENSMUSG00000021420	Fars2	321,136328	276,5878612	249,3023158	236,8756654	269,3185638	-0,215448036	False	0,215448036
ENSMUSG00000038467	Chmp4b	2262,700352	4556,016713	2627,009425	3489,144872	8755,819387	0,215375835	False	0,215375835
ENSMUSG00000026946	Nmi	286,7288643	220,8861391	210,8636328	246,9793668	163,726704	-0,215296624	False	0,215296624
ENSMUSG00000074088	Snrnp40	337,5208345	498,4343748	404,1552962	391,7990864	748,6344219	0,215137474	False	0,215137474
ENSMUSG00000026355	Mcm6	326,05168	331,3292087	265,7760372	327,8089777	378,4696998	0,21507715	False	0,21507715
ENSMUSG00000024908	Ppp6r3	1898,145082	2214,623638	1635,291402	2047,683477	3020,639046	-0,215042526	False	0,215042526
ENSMUSG00000030750	Nsmce1	398,962734	492,6721277	343,7516514	348,0163804	424,7402901	-0,214887351	False	0,214887351
ENSMUSG00000072568	Fam84b	2798,473715	1886,175553	2106,439832	2411,416726	1878,111394	-0,214759207	False	0,214759207
ENSMUSG00000028522	Mier1	1261,607003	1477,056009	1087,265607	1662,620192	1596,928577	-0,214558156	False	0,214558156
ENSMUSG00000014959	Gorasp2	2367,561194	3600,444068	2040,544946	2106,060418	5315,185753	-0,214447226	False	0,214447226
ENSMUSG00000011752	Pgam1	288,3673149	260,2614944	164,7372131	160,5365884	334,5719603	0,21440889	False	0,21440889
ENSMUSG00000022109	Med4	225,2869648	445,6137763	261,3830448	361,4879822	607,4498003	0,21440172	False	0,21440172
ENSMUSG00000084883	Ccdc85c	598,8537137	375,5064365	516,1766011	484,9776656	423,5538647	-0,214338885	False	0,214338885
ENSMUSG00000053199	Arhgap20	392,4089314	86,43370661	338,2604109	204,3192943	71,18552348	-0,21422354	False	0,21422354
ENSMUSG00000038594	Cep85l	413,7087899	824,9617109	479,9344142	777,9850053	1503,200971	0,214221656	False	0,214221656
ENSMUSG00000001281	Itgb7	738,9412445	204,5597723	636,9838907	203,1966608	270,5049892	-0,214202769	False	0,214202769
ENSMUSG00000011884	Gltp	1735,119242	862,4163171	1495,813895	1121,510852	962,1909923	-0,214104121	False	0,214104121
ENSMUSG00000020777	Acox1	1723,650087	2915,697036	1485,929662	3086,119451	2809,455326	-0,214101101	False	0,214101101
ENSMUSG00000059811	Atl2	547,2425181	974,7801357	634,7873945	837,48458	1038,122217	0,214093155	False	0,214093155
ENSMUSG00000025340	Rabgef1	577,5538552	783,6656066	669,9313333	827,3808786	1212,52675	0,21405775	False	0,21405775
ENSMUSG00000037400	Atp11b	984,7088425	1031,442232	848,9457716	1111,40715	1397,609111	-0,214024812	False	0,214024812
ENSMUSG00000059278	Naa38	376,8436502	287,1519809	437,1027388	300,865774	150,6760247	0,214006334	False	0,214006334
ENSMUSG00000025899	Alkbh8	231,8407674	294,834977	199,8811519	249,2246337	214,7429958	-0,213991838	False	0,213991838
ENSMUSG00000020018	Snrpf	291,6442162	477,3061354	338,2604109	420,987557	583,7212925	0,213924874	False	0,213924874
ENSMUSG00000009281	Rarres2	1373,021647	569,5020891	1592,459727	1063,133911	1116,426293	0,213902516	False	0,213902516
ENSMUSG00000074925	Ptar1	639,81498	859,5351936	752,2999399	742,0607337	994,2244779	0,213882498	False	0,213882498
ENSMUSG00000027187	Cat	5713,277427	3316,17321	4904,775958	4926,115733	2167,59919	-0,213868064	False	0,213868064
ENSMUSG00000023051	Tarbp2	309,6671734	397,5950504	359,1271246	436,7044258	401,0117822	0,213776166	False	0,213776166
ENSMUSG00000018166	Erbb3	879,8480007	1542,361476	1020,272473	2147,597857	1795,061617	0,213628273	False	0,213628273
ENSMUSG00000030884	Uqcrc2	1323,048902	1669,130912	1141,079763	1369,612852	1738,113198	-0,213466746	False	0,213466746
ENSMUSG00000034401	Spata6	634,0804027	947,8896492	546,9275475	790,3339736	907,6154243	-0,213316059	False	0,213316059
ENSMUSG00000063160	Numbl	642,272656	1201,428522	744,6122033	974,4458652	2187,768421	0,213303384	False	0,213303384
ENSMUSG00000030942	Thumpd1	367,0129463	403,3572975	389,878071	316,5826428	704,7366824	-0,213248795	False	0,213248795
ENSMUSG00000003031	Cdkn1b	1441,017349	1095,787325	1670,435341	1641,290155	1042,867919	0,213136435	False	0,213136435
ENSMUSG00000030990	Pgap2	641,4534307	289,0727299	743,5139552	542,2319734	201,6923165	0,213015291	False	0,213015291
ENSMUSG00000009630	Ppp2cb	1506,555376	2434,549403	1746,214459	2018,495006	3179,620049	0,21297705	False	0,21297705
ENSMUSG00000001493	Meox1	330,9670319	137,3335561	285,5445027	194,2155929	88,98190434	-0,212971905	False	0,212971905
ENSMUSG00000030116	Mfap5	411,2511139	89,31483017	476,6396699	158,2913214	90,16832974	0,212879443	False	0,212879443
ENSMUSG00000022124	Fbxl3	1089,569684	1312,831966	940,1003628	1561,583178	1460,489657	-0,212871779	False	0,212871779
ENSMUSG00000043832	Clec4a3	308,0287228	128,6901854	265,7760372	150,432887	41,52488869	-0,21285385	False	0,21285385
ENSMUSG00000036863	Syde2	175,3142199	248,7370001	203,1758962	345,7711135	230,1665259	0,212786239	False	0,212786239
ENSMUSG00000051790	Nlgn2	1147,734682	1330,118707	1852,744523	1915,212726	1500,82812	0,212765828	False	0,212765828
ENSMUSG00000044345	Marveld1	3820,047698	3156,75104	4427,03804	3163,581161	2220,988332	0,212751118	False	0,212751118
ENSMUSG00000046836	Brox	782,3601868	906,5935449	608,4294404	667,9669237	826,9384977	0,212622934	False	0,212622934
ENSMUSG00000029344	Tpst2	1159,203837	1751,723121	1343,157411	1776,006174	1905,399178	0,212494115	False	0,212494115
ENSMUSG00000027863	Cd2	549,7001941	147,8976758	474,4431738	208,8098283	144,7438977	-0,212409689	False	0,212409689
ENSMUSG00000034227	Foxj1	2904,973008	5292,623968	2507,300384	3141,128492	6528,898928	-0,212389912	False	0,212389912
ENSMUSG00000031295	Phka2	328,5093559	603,1151973	422,8255137	1298,886942	283,5556685	-0,212302462	False	0,212302462
ENSMUSG00000049878	Rlf	455,4892816	733,7261317	393,1728153	566,92991	1402,354812	-0,212253531	False	0,212253531
ENSMUSG00000034543	Morc2a	633,2611774	1235,04163	733,6297224	1251,736336	1779,638087	0,212251453	False	0,212251453
ENSMUSG00000001995	Sipa1l2	1232,934116	1513,55024	1272,869533	1064,256544	1951,669769	-0,212249748	False	0,212249748
ENSMUSG00000015335	Zdhhc12	367,8321716	518,6022397	426,1202579	468,1381633	589,6534195	0,212212972	False	0,212212972
ENSMUSG00000028708	Mknk1	1037,139263	730,8450081	1201,483408	864,4277836	867,276961	0,212207098	False	0,212207098
ENSMUSG00000042359	Osbpl6	1702,350229	889,3068036	1299,227487	1469,527232	455,5873502	-0,212175782	False	0,212175782
ENSMUSG00000021131	Erh	206,4447823	307,3198457	308,6077126	239,1209324	530,3321499	0,211984471	False	0,211984471
ENSMUSG00000042148	Cox10	398,1435087	633,8471818	343,7516514	465,8928964	1022,698687	-0,211921892	False	0,211921892
ENSMUSG00000040761	Spen	1500,820798	1454,967395	1295,932743	1479,630934	1913,704156	-0,211760879	False	0,211760879
ENSMUSG00000022529	Zfp263	396,505058	511,8796181	537,0433147	650,004788	459,1466264	0,211615676	False	0,211615676
ENSMUSG00000003762	Coq8b	793,8293414	1453,046646	919,2336492	1173,151992	1010,834433	0,211602722	False	0,211602722
ENSMUSG00000034290	Nek9	2126,708948	1567,331213	1655,059868	1636,799621	1836,586506	-0,211595755	False	0,211595755
ENSMUSG00000019961	Tmpo	1611,416217	1180,300283	1865,923501	1498,715703	1236,255258	0,21156066	False	0,21156066
ENSMUSG00000035150	Eif2s3x	314,5825254	343,8140774	329,4744262	271,6773034	493,5529628	-0,211544489	False	0,211544489
ENSMUSG00000021037	Ahsa1	1064,173699	956,5330199	1232,234354	771,2492044	1128,290547	0,21154301	False	0,21154301
ENSMUSG00000025041	Nt5c2	670,1263171	1406,948669	578,7767421	962,0968969	1656,249846	-0,211426117	False	0,211426117
ENSMUSG00000059975	Zfp74	234,2984434	205,5201468	271,2672776	213,3003622	105,5918598	0,211367659	False	0,211367659
ENSMUSG00000066026	Dhrs3	3172,040464	2739,9485	3617,6292	3346,57042	3702,833646	-0,211262399	False	0,211262399
ENSMUSG00000037907	Ankrd13b	265,4290058	336,1310813	421,7272656	365,9785162	307,2841763	0,211247441	False	0,211247441
ENSMUSG00000052512	Nav2	2037,413387	1323,396086	1819,797081	2358,652952	1104,562039	0,211224429	False	0,211224429
ENSMUSG00000020766	Galk1	425,9971698	331,3292087	493,1133913	459,1570955	195,7601896	0,211075586	False	0,211075586
ENSMUSG00000059089	Fcgr4	185,9641491	405,2780466	215,2566251	383,940652	367,7918713	0,211033121	False	0,211033121
ENSMUSG00000033272	Slc35a4	1884,218251	2219,425511	1850,548027	1627,818554	3257,924124	-0,211026186	False	0,211026186
ENSMUSG00000066258	Trim12a	638,1765294	421,6044134	722,6472415	738,6928333	252,7086083	0,21101903	False	0,21101903
ENSMUSG00000037742	Eef1a1	58213,33248	65256,48812	50294,27116	62739,49496	77761,87942	-0,210955531	False	0,210955531
ENSMUSG00000002767	Mrpl2	299,8364695	606,9566953	347,0463956	475,9965977	773,5493551	0,210952675	False	0,210952675
ENSMUSG00000029415	Sdad1	279,3558363	395,6743014	247,1058197	241,3661993	663,2117937	-0,210880305	False	0,210880305
ENSMUSG00000020580	Rock2	2528,948583	2229,989631	2476,549437	2410,294093	2926,91144	0,210841389	False	0,210841389
ENSMUSG00000025246	Tbl1x	2009,559726	1070,817587	1736,330226	1370,735485	1332,355714	-0,210838102	False	0,210838102
ENSMUSG00000039990	Edrf1	269,5251324	366,8630659	311,9024568	322,1958103	472,1973057	0,21067511	False	0,21067511
ENSMUSG00000020572	Nampt	1028,94701	3262,392237	1190,500927	3472,30537	2527,086083	0,210400057	False	0,210400057
ENSMUSG00000020099	Unc5b	448,1162536	415,8421663	366,8148612	301,9884075	518,467896	0,210381603	False	0,210381603
ENSMUSG00000022489	Pde1b	249,0444993	785,5863557	215,2566251	434,4591588	894,564745	-0,210345905	False	0,210345905
ENSMUSG00000025026	Add3	2739,489492	1713,30814	2367,822876	1779,374074	998,9701794	-0,210345905	False	0,210345905
ENSMUSG00000028102	Pex11b	239,2137953	284,2708573	276,758518	293,0073396	306,0977509	0,210327129	False	0,210327129
ENSMUSG00000043881	Kbtbd7	239,2137953	153,6599229	276,758518	179,6213576	131,6932184	0,210327129	False	0,210327129
ENSMUSG00000023806	Rsph3b	299,8364695	270,8256141	230,6320984	346,8937469	244,4036306	0,210317964	False	0,210317964
ENSMUSG00000027858	Tspan2	3677,502491	1398,305298	4254,613091	3596,917687	1236,255258	0,210301622	False	0,210301622
ENSMUSG00000039145	Camk1d	355,5437917	307,3198457	264,6777891	156,0460544	251,5221829	-0,21028633	False	0,21028633
ENSMUSG00000027433	Xrn2	936,3745482	955,5726453	809,4088404	1082,21868	1167,442585	-0,210217114	False	0,210217114
ENSMUSG00000044337	Ackr3	738,9412445	2782,204978	795,1316153	638,7784531	6751,946902	-0,210144006	False	0,210144006
ENSMUSG00000025898	Cwf19l2	328,5093559	516,6814906	379,9938382	612,9578829	724,9059141	0,210041566	False	0,210041566
ENSMUSG00000022136	Dnajc3	2343,803659	2652,554419	2026,267721	2373,247188	3135,722309	-0,210026917	False	0,210026917
ENSMUSG00000020821	Kif1c	3539,872636	2176,208658	2711,574528	3060,298881	2928,097866	-0,210024894	False	0,210024894
ENSMUSG00000004264	Phb2	1367,28707	2056,161843	1581,477246	1737,836635	3032,5033	0,209956622	False	0,209956622
ENSMUSG00000029500	Pgam5	250,6829499	369,7441894	289,9374951	290,7620727	384,4018268	0,209878041	False	0,209878041
ENSMUSG00000010110	Stx5a	1462,317208	2122,427685	1691,302055	2214,955866	2967,249904	0,209878041	False	0,209878041
ENSMUSG00000054520	Sh3bp2	384,2166781	191,1145291	228,4356022	207,6871948	332,1991096	-0,209871939	False	0,209871939

ENSMUSG00000029166	Mapre3	2194,70465	1864,086939	2538,05133	2504,595306	1649,131294	0,209694443	False	0,209694443
ENSMUSG00000037808	Fam76b	554,6155461	940,2066531	641,3768831	1002,511702	1074,901404	0,2096843	False	0,2096843
ENSMUSG00000002325	Irf9	1156,746161	900,8312978	1337,66617	1041,803874	978,8009478	0,209645809	False	0,209645809
ENSMUSG00000025324	Atp10a	316,220976	177,6692858	273,4637738	231,262498	141,1846216	-0,209583341	False	0,209583341
ENSMUSG00000041842	Fhdc1	1290,279889	1536,599229	1115,820057	1956,750165	1975,398276	-0,209579662	False	0,209579662
ENSMUSG00000016758	Bik	249,8637246	216,0842665	341,5551552	284,0262718	317,9620049	-0,209547434	False	0,209547434
ENSMUSG00000043991	Pura	1096,123487	1322,435711	1583,673742	2052,174011	947,9538876	-0,209521551	False	0,209521551
ENSMUSG00000034109	Golim4	1064,173699	655,9357957	920,3318973	650,004788	526,7728737	-0,209507517	False	0,209507517
ENSMUSG00000030126	Tmcc1	839,7059597	970,9386376	1118,016553	1045,171775	1289,6444	0,209495895	False	0,209495895
ENSMUSG00000045251	Zfp688	203,167881	248,7370001	256,9900525	331,1768781	234,9122275	0,209449466	False	0,209449466
ENSMUSG00000025817	Nudt5	351,4476651	121,9675638	406,3517923	196,4608599	28,47420939	0,209419388	False	0,209419388
ENSMUSG00000038637	Lrrc56	475,1506894	549,3342243	466,7554372	536,6188059	279,9963923	0,209299054	False	0,209299054
ENSMUSG00000010095	Slc3a2	2726,381887	5656,605911	3151,972011	3989,839407	8010,744242	0,209267068	False	0,209267068
ENSMUSG00000020057	Dram1	4798,202738	5113,994308	4357,848411	5546,932051	3857,068947	0,209195936	False	0,209195936
ENSMUSG00000020844	Nxn	428,4548458	338,0518303	495,3098874	432,2138918	297,7927732	0,209188252	False	0,209188252
ENSMUSG00000015461	Atf6b	1510,651502	800,952348	1306,915224	815,0319103	523,2135975	-0,209005318	False	0,209005318
ENSMUSG00000033096	Apmap	873,2941981	979,5820083	867,6159891	755,5323356	1211,340324	-0,208974254	False	0,208974254
ENSMUSG00000061175	Fnip2	409,6126633	354,3781971	443,6922273	437,8270592	443,7230963	-0,208970471	False	0,208970471
ENSMUSG00000035585	Tsen34	631,6227268	546,4531007	779,7561421	599,4862811	628,8054574	-0,208965401	False	0,208965401
ENSMUSG00000037750	Fam222b	628,3458255	383,1894327	543,6328033	443,4402267	498,2986643	-0,208926288	False	0,208926288
ENSMUSG00000056770	Setd3	1716,277059	1469,373012	1398,069815	1983,693368	1681,164779	0,20890657	False	0,20890657
ENSMUSG00000027630	Tbl1xr1	690,6069503	861,4559426	842,3562831	798,192408	997,783754	0,208871703	False	0,208871703
ENSMUSG00000001323	Srr	706,9914568	166,1447916	611,7241847	319,9505433	80,67692661	-0,208811467	False	0,208811467
ENSMUSG00000044534	Ackr2	912,6170138	1474,174885	1194,893919	1645,780689	1054,732173	0,208795248	False	0,208795248
ENSMUSG00000015759	Cnih1	1134,627077	1037,204479	1311,308216	949,7479285	1046,427195	0,208788625	False	0,208788625
ENSMUSG00000025967	Eef1b2	1716,277059	2270,32536	1983,436046	2732,489903	3408,600149	0,208719414	False	0,208719414
ENSMUSG00000046694	Fam46b	267,0674565	1073,698711	308,6077126	1007,002236	593,2126956	0,208569931	False	0,208569931
ENSMUSG00000038975	Rabggtb	267,0674565	395,6743014	308,6077126	391,7990864	522,0271722	0,208569931	False	0,208569931
ENSMUSG00000032280	Tle3	1201,803554	1549,084097	1040,040939	1121,510852	3179,620049	-0,208560776	False	0,208560776
ENSMUSG00000038456	Dennd2a	403,0588607	293,8746025	296,5269836	327,8089777	348,809065	-0,208553004	False	0,208553004
ENSMUSG00000062202	Btbd9	582,4692072	753,8939966	504,0958721	665,7216568	934,9032083	-0,208483642	False	0,208483642
ENSMUSG00000028917	Plekhm2	646,3687826	1384,860055	746,8086994	1124,878752	2479,629068	0,208381211	False	0,208381211
ENSMUSG00000024456	Diaph1	3373,569895	2971,398758	3475,955197	3009,780374	3897,40741	0,208238697	False	0,208238697
ENSMUSG00000046916	Myct1	1552,431994	243,9351276	1793,439127	533,2509055	306,0977509	0,208198707	False	0,208198707
ENSMUSG00000008575	Nfib	5682,96609	4221,806381	6447,814521	6565,160622	4192,827333	0,208186201	False	0,208186201
ENSMUSG00000031778	Cx3cl1	1404,15221	998,7894987	1622,112425	600,6089146	1031,003665	0,208174481	False	0,208174481
ENSMUSG00000020528	Prpsap2	339,9785105	224,7276372	294,3304874	193,0929595	175,5909579	-0,208006571	False	0,208006571
ENSMUSG00000053838	Nudcd3	1334,518057	931,5632824	1155,356988	910,4557565	825,7520723	-0,207980135	False	0,207980135
ENSMUSG00000041164	Zmiz2	2731,297239	2367,323187	2231,640114	2092,588816	3154,705115	0,20791883	False	0,20791883
ENSMUSG00000027782	Kpna4	1364,829394	1547,163348	1181,714942	1251,736336	1710,825414	-0,207838558	False	0,207838558
ENSMUSG00000025035	Arl3	509,5581531	341,8933284	342,6534033	441,1949597	359,4868936	-0,207830493	False	0,207830493
ENSMUSG00000041515	Irf8	1151,011584	712,5978923	814,9000809	381,695385	996,5973287	-0,207819741	False	0,207819741
ENSMUSG00000025510	Cd151	4903,882805	3489,040624	5663,665387	4612,900991	3331,482499	0,207811531	False	0,207811531
ENSMUSG00000053453	Thoc7	402,2396353	533,968232	464,558941	537,7414394	754,5665488	0,207806403	False	0,207806403
ENSMUSG00000032252	Glce	282,6327377	188,2334055	214,158377	244,7340998	123,3882407	-0,207713452	False	0,207713452
ENSMUSG00000036256	Igfbp7	7956,316371	11792,43871	9187,943499	11408,20148	15322,68393	0,207641341	False	0,207641341
ENSMUSG00000031402	Mpp1	830,6944811	505,1569964	719,3524973	627,5521183	265,7592876	-0,207619076	False	0,207619076
ENSMUSG00000001349	Cnn1	353,9053411	702,9941471	387,6815749	306,4789451	576,6027402	-0,207575587	False	0,207575587
ENSMUSG00000032540	Abhd5	806,9369466	882,584182	914,8406568	914,9462905	698,8045554	-0,207566934	False	0,207566934
ENSMUSG00000034520	Gjc1	277,7173857	335,1707068	320,6884415	415,3743895	422,3674393	0,20755486	False	0,20755486
ENSMUSG00000031822	Gse1	1637,631428	1890,977426	2052,625675	2434,992029	2250,648967	0,207521446	False	0,207521446
ENSMUSG00000048458	Fam212b	336,7016092	255,4596218	388,7798229	191,970326	156,6081516	0,207482731	False	0,207482731
ENSMUSG00000027602	Map1lc3a	1459,040307	2050,399596	1684,712566	1695,176563	2389,460738	0,207482731	False	0,207482731
ENSMUSG00000005698	Ctcf	1417,259815	1217,754889	1636,38965	1347,160182	1365,575625	0,207412057	False	0,207412057
ENSMUSG00000037012	Hk1	1491,80932	1757,485368	1411,248792	1292,151142	2178,277018	-0,207288314	False	0,207288314
ENSMUSG00000001300	Efnb2	2437,195346	1900,581171	2813,7116	2575,321215	2205,564802	0,207252572	False	0,207252572
ENSMUSG00000031176	Dynlt3	1382,033126	1184,141781	1191,599175	1050,784942	1197,10322	-0,207244643	False	0,207244643
ENSMUSG00000029578	Wipi2	891,3171553	1120,757062	772,0684055	1174,274626	1152,019055	-0,207210198	False	0,207210198
ENSMUSG00000006731	B4galnt1	2597,76351	3626,37418	2250,310331	2641,556591	4419,434582	-0,207146128	False	0,207146128
ENSMUSG00000036632	Alg5	265,4290058	421,6044134	306,4112164	325,5637107	401,0117822	0,207143074	False	0,207143074
ENSMUSG00000042978	Sbk1	546,4232928	301,5575986	473,3449257	244,7340998	342,8769381	-0,207127127	False	0,207127127
ENSMUSG00000039480	Nt5dc1	300,6556948	337,0914558	285,5445027	260,4509686	382,028976	-0,207100483	False	0,207100483
ENSMUSG00000075232	Amd1	636,5380787	497,4740003	734,7279705	400,7801542	448,4687979	0,206963377	False	0,206963377
ENSMUSG00000016194	Hsd11b1	2134,901201	3023,258982	3122,319313	2357,530319	1849,637185	-0,206926999	False	0,206926999
ENSMUSG00000022300	Dcaf13	368,6513969	617,520815	432,7097464	425,4780909	858,9719833	0,206827621	False	0,206827621
ENSMUSG00000019124	Scrn1	389,1320301	210,3220194	337,1621628	253,7151677	122,2018153	-0,206817093	False	0,206817093
ENSMUSG00000054309	Cpsf3	970,7820119	802,873097	789,6403749	785,8434397	841,1756024	-0,20674037	False	0,20674037
ENSMUSG00000043535	Setx	755,3257511	602,1548227	575,4819978	654,4953219	354,741192	-0,206716093	False	0,206716093
ENSMUSG00000018672	Copz2	788,9139895	450,4156489	910,4476645	538,8640729	539,823553	0,206708068	False	0,206708068
ENSMUSG00000021559	Dapk1	2247,135071	2785,086102	1947,193859	3416,173696	3864,187499	-0,206690321	False	0,206690321
ENSMUSG00000039703	Nploc4	915,0746897	1423,275036	792,9351191	1047,417042	2394,20644	-0,206686679	False	0,206686679
ENSMUSG00000024913	Lrp5	2666,578438	2670,801534	2310,713976	3296,051913	2636,237219	-0,206651074	False	0,206651074
ENSMUSG00000034201	Gas2l1	1595,031711	1474,174885	1840,663795	1679,459694	1243,37381	0,20664103	False	0,20664103
ENSMUSG00000017737	Mmp9	917,5323657	2992,526998	795,1316153	2469,793668	2225,734034	-0,206565365	False	0,206565365
ENSMUSG00000001017	Chtop	1818,680225	1772,85136	1908,755176	1576,177413	2600,644458	-0,206461966	False	0,206461966
ENSMUSG00000020905	Usp43	270,3443578	571,4228382	311,9024568	420,987557	1210,153899	0,20629667	False	0,20629667
ENSMUSG00000052751	Repin1	352,2668904	440,8119037	470,0501814	406,3933217	500,6715151	0,206207812	False	0,206207812
ENSMUSG00000042903	Foxo4	687,330049	569,5020891	792,9351191	765,6360369	594,399121	0,206199791	False	0,206199791
ENSMUSG00000042305	Tmem183a	793,0101161	1372,375186	914,8406568	1423,499259	1515,065225	0,206181213	False	0,206181213
ENSMUSG00000054459	Vsnl1	276,078935	96,99782631	318,4919454	191,970326	49,82986643	0,206176073	False	0,206176073
ENSMUSG00000024454	Hdac3	756,9642017	884,504931	882,9914623	980,0590326	873,209088	0,206102071	False	0,206102071
ENSMUSG00000041329	Atp1b2	274,4404844	217,0446411	306,4112164	316,5826428	208,8108689	0,206088842	False	0,206088842
ENSMUSG00000037104	Socs5	634,0804027	342,8537029	731,4332262	495,081367	129,3203676	0,206060376	False	0,206060376
ENSMUSG00000024978	Gpam	1555,708895	1873,69684	1348,648651	1915,212726	2148,616384	-0,20605758	False	0,20605758
ENSMUSG00000031878	Nae1	510,3773784	588,7095795	590,857471	685,9290595	765,2443774	0,205991718	False	0,205991718
ENSMUSG00000062761	Zfp512	455,4892816	405,2780466	475,5414219	525,3924711	138,8117708	0,205978421	False	0,205978421
ENSMUSG00000029771	Irf5	780,7217362	758,6958692	686,4050546	453,543928	900,496872	0,205912828	False	0,205912828
ENSMUSG00000026577	Blzf1	343,2554118	503,2362474	297,6252317	398,5348873	555,2470831	-0,205785628	False	0,205785628
ENSMUSG00000021771	Vdac2	1957,94853	3206,690515	2257,998068	2582,057016	3364,70241	0,205701411	False	0,205701411
ENSMUSG00000028063	Lmna	1969,417685	1779,573982	2271,177045	1846,732083	1948,110492	0,205671054	False	0,205671054
ENSMUSG00000024986	Hhex	398,1435087	340,9329539	331,6709224	313,2147424	345,2497889	-0,205648004	False	0,205648004
ENSMUSG00000026035	Ppil3	269,5251324	184,3919074	310,8042087	263,818869	71,18552348	0,205586234	False	0,205586234
ENSMUSG00000041132	N4bp2l1	303,9325961	517,6418652	263,579541	514,1661362	361,8597443	-0,205513016	False	0,205513016
ENSMUSG00000035297	Cops4	557,8924474	566,6209656	591,9557191	483,8550321	838,8027516	-0,205412154	False	0,205412154
ENSMUSG00000025025	Mxi1	842,982861	575,2643362	971,9495574	868,9183176	513,7221944	0,205378143	False	0,205378143
ENSMUSG00000038047	Haus6	163,8450653	283,3104828	188,898671	259,3283351	296,6063478	0,20528033	False	0,20528033
ENSMUSG00000058704	Memo1	286,7288643	493,6325022	330,5726743	420,987557	497,1122389	0,20528033	False	0,20528033
ENSMUSG00000058135	Gstm1	14166,86357	9312,7517	12288,29785	11998,70669	11502,39417	-0,205235302	False	0,205235302
ENSMUSG00000019087	Atp6ap1	3673,406364	3286,4016	4234,844625	3205,1186	3342,160327	0,205190538	False	0,205190538
ENSMUSG00000007670	Khsrp	1622,885372	1712,347765	1487,02791	1407,78239	2786,913244	-0,205136758	False	0,205136758
ENSMUSG00000045503	Sys1	298,1980189	580,0662088	343,7516514	374,9595841	758,125825	0,205095971	False	0,205095971
ENSMUSG00000109511	Nup62	471,0545628	523,4041123	472,2466776	408,6385886	1039,308643	-0,205068731	False	0,205068731
ENSMUSG00000053198	Prx	3689,790871	3905,843164	2343,661419	4026,886312	4253,335028	0,205055448	False	0,205055448
ENSMUSG00000018899	Irf1	1640,908329	1673,932785	1780,26015	1423,499259	2256,581094	-0,205052901	False	0,205052901
ENSMUSG00000049382	Krt8	2284,819436	5281,099474	2633,598914	2893,026491	7003,469085	0,204955489	False	0,204955489
ENSMUSG00000029401	Rilpl2	175,3142199	270,8256141	237,2215869	202,0740273	453,2144995	0,204940884	False	0,204940884
ENSMUSG00000004567	Mcoln1	992,9010958	933,4840314	1144,374507	1077,728146	733,2108918	0,204837343	False	0,204837343
ENSMUSG00000006784	Ttc25	283,451963	231,4502588	286,6427508	326,6863442	125,7610915	0,20480189	False	0,20480189
ENSMUSG00000025208	Mrpl43	502,1851252	712,5978923	578,7767421	650,004788	931,3439321	0,204787653	False	0,204787653
ENSMUSG00000022544	Eef2kmt	321,136328	265,0633669	370,1096055	333,4221451	149,4895993	0,204766702	False	0,204766702

ENSMUSG00000008348	Ubc	1564,720374	2799,49172	1803,32336	2204,852165	5175,187557	0,204753253	False	0,204753253
ENSMUSG00000057113	Npm1	2357,73049	2875,361307	2717,065768	3207,363867	3777,578446	0,204650673	False	0,204650673
ENSMUSG00000038212	Mfsd14b	875,7518741	478,2665099	759,9876765	543,3546069	440,1638202	-0,204546145	False	0,204546145
ENSMUSG00000027357	Crls1	224,4677395	220,8861391	206,4706404	177,3760907	258,6407353	0,204441398	False	0,204441398
ENSMUSG00000061613	U2af1	600,4921644	1173,577661	691,8962951	899,2294217	1906,585604	0,204410395	False	0,204410395
ENSMUSG00000007038	Neu1	600,4921644	1229,279383	691,8962951	955,3610959	1511,505948	0,204410395	False	0,204410395
ENSMUSG00000041268	Dmxl2	539,0502649	533,0078575	467,8536852	663,4763898	500,6715151	-0,204362389	False	0,204362389
ENSMUSG00000061740	Cyp2d22	1294,376016	1735,396754	1669,337093	2660,64136	1491,336717	0,204349248	False	0,204349248
ENSMUSG00000073616	Cops9	440,7432257	580,0662088	665,538341	516,4114032	507,7900675	0,204293781	False	0,204293781
ENSMUSG00000022003	Slc25a30	247,4060486	301,5575986	275,6602699	354,7521813	214,7429958	-0,204269696	False	0,204269696
ENSMUSG00000056091	St3gal5	569,3616019	655,9357957	387,6815749	560,1941091	556,4335085	0,204209408	False	0,204209408
ENSMUSG00000007029	Vars	1310,760522	2366,362812	1137,785019	1731,100834	3174,874347	-0,204176137	False	0,204176137
ENSMUSG00000031684	Slc10a7	427,6356205	224,7276372	371,2078535	268,309403	129,3203676	-0,204154796	False	0,204154796
ENSMUSG00000035139	Secisbp2	378,4821009	597,3529502	436,0044907	506,3077018	793,7185868	0,20411792	False	0,20411792
ENSMUSG00000029135	Fosl2	927,3630696	2156,040793	805,0158481	1459,423531	5972,46542	-0,20411709	False	0,20411709
ENSMUSG00000035992	Fnip1	585,7461085	620,4019386	508,4888645	827,3808786	707,1095332	-0,204059284	False	0,204059284
ENSMUSG00000038965	Ube2l3	2702,624352	2282,810229	2221,755881	1926,439061	3113,180227	0,204028099	False	0,204028099
ENSMUSG00000026176	Ctdsp1	5267,61885	4463,820759	6067,820683	5398,744431	4067,066241	0,204027492	False	0,204027492
ENSMUSG00000019849	Prep	1873,568322	966,136765	1626,505417	976,6911322	1055,918598	-0,204012961	False	0,204012961
ENSMUSG00000027361	Gabpb1	625,8881495	642,4905525	620,5101694	543,3546069	1405,914089	-0,204010816	False	0,204010816
ENSMUSG00000059689	Zfp637	208,0832329	252,5784982	318,4919454	276,1678374	239,657929	0,203815953	False	0,203815953
ENSMUSG00000032602	Slc25a20	374,3859742	644,4113015	325,0814339	635,4105526	716,6009363	-0,203725225	False	0,203725225
ENSMUSG00000021598	Med10	424,3587191	778,863734	488,7203989	581,5241454	1465,235358	0,203725003	False	0,203725003
ENSMUSG00000005103	Wdr1	2153,743383	1747,881623	1870,316493	1372,980752	1682,351205	-0,203563942	False	0,203563942
ENSMUSG00000003363	Pld3	1865,376069	1013,195116	1619,915929	1248,368436	1312,186483	-0,203547572	False	0,203547572
ENSMUSG00000030879	Mrpl17	472,6930134	455,2175215	583,1697344	458,034462	410,5031854	-0,203510124	False	0,203510124
ENSMUSG00000027164	Traf6	756,1449764	1222,556761	656,7523563	736,4475663	2211,496929	-0,203313399	False	0,203313399
ENSMUSG00000019188	H13	2842,711883	5820,829953	3272,779301	3415,051062	8481,755122	0,203248433	False	0,203248433
ENSMUSG00000021097	Clmn	416,1664659	250,6577492	300,919976	361,4879822	158,9810024	-0,203213034	False	0,203213034
ENSMUSG00000015002	Efr3a	1212,453483	1401,186422	1053,219916	1314,603811	1321,677886	-0,203122691	False	0,203122691
ENSMUSG00000022097	Sftpc	244507,6294	281452,1581	343578,1282	366553,3045	293179,9513	0,203010228	False	0,203010228
ENSMUSG00000038406	Scaf1	1948,117827	2885,925426	1692,400303	2486,63317	4746,88799	-0,203010089	False	0,203010089
ENSMUSG00000061665	Cd2ap	1892,410504	1908,264167	1644,077387	2817,810048	1690,656183	-0,202946866	False	0,202946866
ENSMUSG00000024238	Zeb1	2011,198177	906,5935449	1747,312707	1571,686879	690,4995777	-0,202917424	False	0,202917424
ENSMUSG00000040093	Bmf	752,0488498	846,0899503	653,457612	1088,954481	1168,62901	-0,202732721	False	0,202732721
ENSMUSG00000029911	Ssbp1	275,2597097	447,5345254	464,558941	484,9776656	316,7755795	0,202667666	False	0,202667666
ENSMUSG00000030876	Mettl9	702,0761048	739,4883788	563,4012688	690,4195934	807,9556914	0,202648751	False	0,202648751
ENSMUSG00000079481	Nhsl2	563,6270247	343,8140774	489,818647	378,3274845	251,5221829	-0,20249309	False	0,20249309
ENSMUSG00000014606	Slc25a11	1129,711725	724,1223865	981,8337902	736,4475663	449,6552233	-0,202403956	False	0,202403956
ENSMUSG00000069917	Hba-a2	25148,57907	21857,16365	18794,31952	21792,56122	16758,25865	-0,20237069	False	0,20237069
ENSMUSG00000042207	Kdm5b	598,8537137	520,5229887	531,5520743	546,7225073	803,2099899	-0,202241747	False	0,202241747
ENSMUSG00000002985	Apoe	8824,695217	7670,511275	11593,10681	10387,72764	10676,6421	-0,202223709	False	0,202223709
ENSMUSG00000030602	Pak4	1028,127785	702,0337726	1182,81319	783,5981727	664,3982191	0,20220265	False	0,20220265
ENSMUSG00000024220	Zfp523	617,6958962	515,7211161	605,1346962	710,6269962	319,1484302	0,202195751	False	0,202195751
ENSMUSG00000025287	Acot9	462,0430842	1136,123055	531,5520743	633,1652857	977,6145224	0,202183649	False	0,202183649
ENSMUSG00000053841	Txlna	1269,799256	1591,340576	1460,669956	1490,857268	1755,909579	0,202029795	False	0,202029795
ENSMUSG00000032216	Nedd4	8161,941928	4183,3914	7095,780893	6640,377065	2853,353066	-0,201950984	False	0,201950984
ENSMUSG00000038205	Prkab2	294,1018922	238,1728804	338,2604109	285,1489053	253,8950337	0,201818271	False	0,201818271
ENSMUSG00000000631	Myo18a	2438,833797	2120,506936	2572,097021	2793,112111	3331,482499	-0,201782242	False	0,201782242
ENSMUSG00000001175	Calm1	11670,684	9810,225701	8253,334377	7174,750604	10147,49637	-0,201765294	False	0,201765294
ENSMUSG00000025979	Mob4	667,6686411	863,3766916	833,5702984	767,8813039	1033,376516	0,20175104	False	0,20175104
ENSMUSG00000031728	Zfp821	311,3056241	407,1987956	358,0288765	428,8459914	710,6688094	0,201744311	False	0,201744311
ENSMUSG00000031490	Eif4ebp1	841,3444104	1736,357128	967,556565	1385,329721	2847,420939	0,201649507	False	0,201649507
ENSMUSG00000055371	Stam2	525,1234343	567,5813401	381,0920863	462,5249959	603,8905241	0,201630456	False	0,201630456
ENSMUSG00000033373	Fntb	367,0129463	458,0986451	363,5201169	426,6007244	319,1484302	-0,201603403	False	0,201603403
ENSMUSG00000041130	Zfp598	1445,932701	3102,970067	1662,747604	2240,776437	4388,587522	0,201568786	False	0,201568786
ENSMUSG00000060429	Sntb1	774,1679336	322,685838	673,2260776	388,4311859	482,8751342	-0,201555491	False	0,201555491
ENSMUSG00000058546	Rpl23a	3570,183973	4675,103153	4105,251351	4686,994801	6097,040086	0,201472137	False	0,201472137
ENSMUSG00000071068	Treml2	321,9555533	450,4156489	426,1202579	438,9496927	279,9963923	-0,201453296	False	0,201453296
ENSMUSG00000021040	Slirp	242,4906967	330,3688342	344,8498994	284,0262718	278,8099669	0,201352738	False	0,201352738
ENSMUSG00000046556	Zfp319	851,1751143	668,4206645	638,0821388	674,7027246	740,3294441	-0,201288563	False	0,201288563
ENSMUSG00000021114	Atp6v1d	1058,439122	1288,822603	1216,858881	1255,104237	1306,254356	0,201223576	False	0,201223576
ENSMUSG00000000743	Chmp1a	1282,906861	1526,995484	1119,114801	1115,897684	2052,515927	-0,201211681	False	0,201211681
ENSMUSG00000025940	Tmem70	290,0057656	271,7859886	226,239106	218,9135296	333,3855349	0,201109927	False	0,201109927
ENSMUSG00000022400	Rbx1	1063,354474	1578,855707	1222,350121	1308,990644	1702,520436	0,201034974	False	0,201034974
ENSMUSG00000025130	P4hb	7649,926099	9464,490874	7681,147123	6654,971301	10576,98236	-0,201013366	False	0,201013366
ENSMUSG00000030494	Rhpn2	311,3056241	270,8256141	342,6534033	433,3365253	425,9267155	-0,200967449	False	0,200967449
ENSMUSG00000068739	Sars	1228,83799	1722,911885	1412,34704	1808,562545	2013,363889	0,200799907	False	0,200799907
ENSMUSG00000039831	Arhgap29	3993,723467	2115,705063	3474,856949	3155,722727	2495,052598	-0,200780854	False	0,200780854
ENSMUSG00000028412	Slc44a1	1657,292836	931,5632824	1441,999739	1040,681241	690,4995777	-0,200757639	False	0,200757639
ENSMUSG00000051451	Crebzf	734,8451179	350,536699	844,5527792	516,4114032	167,2859802	0,200747378	False	0,200747378
ENSMUSG00000005803	Sqor	821,6830025	551,2549733	714,9595049	605,0994485	402,1982076	-0,200720393	False	0,200720393
ENSMUSG00000030970	Ctbp2	849,5366636	600,2340737	976,3425497	821,7677112	669,1439207	0,200711195	False	0,200711195
ENSMUSG00000073599	Ecscr	1538,505163	680,9055332	1338,764419	826,2582451	555,2470831	-0,200627172	False	0,200627172
ENSMUSG00000033793	Atp6v1h	637,357304	853,7729464	554,6152841	614,0805164	1159,137607	-0,200615003	False	0,200615003
ENSMUSG00000075701	Selenos	842,982861	1597,102823	968,6548131	1155,189856	2279,123177	0,200479343	False	0,200479343
ENSMUSG00000014592	Camta1	344,0746371	684,7470313	395,3693115	647,759521	772,3629297	0,200479343	False	0,200479343
ENSMUSG00000039850	Endov	469,4161121	196,8767762	408,5482885	252,5925342	96,10045669	-0,200360753	False	0,200360753
ENSMUSG00000008668	Rps18	3112,237016	5863,086432	3575,895773	4546,665615	8383,281815	0,200352752	False	0,200352752
ENSMUSG00000025231	Sufu	486,619844	350,536699	359,1271246	288,5168057	423,5538647	-0,2002497	False	0,2002497
ENSMUSG00000059409	Ppp2r5d	1195,249751	946,9292747	973,0478055	1032,822806	1040,495068	-0,200041979	False	0,200041979
ENSMUSG00000042524	Sun2	2542,056188	3029,02123	2212,969896	2384,473523	2982,673434	-0,200012093	False	0,200012093
ENSMUSG00000004460	Dnajb11	1026,489334	1159,172043	1032,353202	893,6162543	1678,791929	-0,199991304	False	0,199991304
ENSMUSG00000018983	E2f2	822,5022278	562,7794675	716,057753	566,92991	370,1647221	-0,199943635	False	0,199943635
ENSMUSG00000020962	Gtf2a1	617,6958962	534,9286065	709,4682645	653,3726884	382,028976	0,199841406	False	0,199841406
ENSMUSG00000021846	Peli2	413,7087899	674,1829116	360,2253727	682,561159	923,0389544	-0,199715808	False	0,199715808
ENSMUSG00000019368	Sec14l4	1531,951361	5345,444567	1759,393436	5399,867064	3998,253569	0,199707642	False	0,199707642
ENSMUSG00000016637	Ift27	393,2281567	339,0122048	325,0814339	342,403213	238,4715036	-0,199670389	False	0,199670389
ENSMUSG00000045078	Rnf216	942,1091255	1273,456611	820,3913213	977,8137656	1651,504145	-0,199581948	False	0,199581948
ENSMUSG00000031835	Mbtps1	2247,954296	1412,710916	1604,540456	1407,78239	1957,601896	-0,1995253	False	0,1995253
ENSMUSG00000032570	Atp2c1	1436,101997	891,2275526	1239,922091	1250,613703	608,6362257	-0,199521989	False	0,199521989
ENSMUSG00000016921	Srsf6	3083,564129	3981,712751	4045,945954	3540,786012	4316,215573	0,199470807	False	0,199470807
ENSMUSG00000001105	Ift20	846,2597623	1104,430696	1141,079763	1143,963521	971,6823954	0,199384259	False	0,199384259
ENSMUSG00000040327	Cul9	481,704492	327,4877106	419,5307694	363,7332492	395,0796553	-0,199371752	False	0,199371752
ENSMUSG00000030619	Eed	369,4706223	394,7139269	321,7866896	394,0443533	518,467896	-0,199355004	False	0,199355004
ENSMUSG00000031636	Pdlim3	257,2367525	167,1051661	224,0426098	176,2534572	100,8461583	-0,199323642	False	0,199323642
ENSMUSG00000024269	Tpgs2	502,1851252	242,0143785	576,5802459	263,818869	233,7258021	0,199302113	False	0,199302113
ENSMUSG00000004837	Grap	1494,266996	2474,885133	1739,62497	3147,864293	1715,571116	0,199250979	False	0,199250979
ENSMUSG00000001700	Gramd3	290,0057656	638,6490544	252,5970601	337,9126791	596,7719718	-0,199243735	False	0,199243735
ENSMUSG00000041530	Ago1	1854,726139	550,2945988	1615,522937	939,6442271	312,0298779	-0,199204947	False	0,199204947
ENSMUSG00000031229	Atrx	1871,110646	2051,35997	1629,800162	2776,272609	1855,569312	-0,199199794	False	0,199199794
ENSMUSG00000034430	Zxdc	277,7173857	380,3083091	330,5726743	318,8279098	397,4525061	0,199160431	False	0,199160431
ENSMUSG00000022533	Atp13a3	2004,644374	2835,985951	1746,214459	2404,680925	3676,732288	-0,199115571	False	0,199115571
ENSMUSG00000022808	Snx4	1296,014467	1221,596387	997,2092634	998,0211684	1487,777441	0,199076905	False	0,199076905
ENSMUSG00000006476	Nsmf	1416,44059	1625,914059	1726,445993	1701,912364	1687,096906	0,198980911	False	0,198980911
ENSMUSG00000036833	Pnpla7	308,8479481	657,8565448	269,0707814	804,9282089	714,2280855	-0,19891101	False	0,19891101
ENSMUSG00000006288	Ttc5	430,9125218	494,5928767	496,4081355	517,5340367	505,4172167	0,19884644	False	0,19884644
ENSMUSG00000060935	Tmem263	239,2137953	174,7881623	274,5620219	252,5925342	98,47330747	0,19883149	False	0,19883149
ENSMUSG00000028221	Tmem55a	603,7690657	407,1987956	526,0608339	415,3743895	325,0805572	-0,198767199	False	0,198767199
ENSMUSG00000024014	Pim1	917,5323657	2083,052329	799,5246076	1341,547015	4035,032756	-0,198616612	False	0,198616612

ENSMUSG00000020614	Fam20a	343,2554118	295,7953515	323,9831858	317,7052763	393,8932299	0,198522154	False	0,198522154
ENSMUSG00000031155	Pim2	295,7403429	250,6577492	339,358659	277,2904709	237,2850783	0,198479766	False	0,198479766
ENSMUSG00000000171	Sdhd	657,8379372	734,6865062	573,2855016	609,5899825	901,6832974	-0,198478416	False	0,198478416
ENSMUSG00000031232	Magt1	1432,825096	538,7701046	1248,708075	626,4294848	544,5692546	-0,19842627	False	0,19842627
ENSMUSG00000053965	Pde5a	3549,70334	676,1036606	3093,764862	992,408001	488,8072612	-0,198334908	False	0,198334908
ENSMUSG00000042688	Mapk6	859,3673675	1219,675638	749,0051956	874,531485	2102,345793	-0,198299269	False	0,198299269
ENSMUSG00000028479	Gne	880,667226	824,0013364	767,6754131	1001,389069	991,8516271	-0,198100534	False	0,198100534
ENSMUSG00000033728	Lrrc14	329,3285813	294,834977	377,7973421	237,9982989	312,0298779	0,198084828	False	0,198084828
ENSMUSG00000048644	Ctxn1	940,4706749	1008,393244	930,21613	1093,445015	819,8199454	-0,19807587	False	0,19807587
ENSMUSG00000029468	P2rx7	534,1349129	306,3594712	465,6571891	444,5628602	239,657929	-0,197935935	False	0,197935935
ENSMUSG00000060090	Rp2	501,3658998	276,5878612	437,1027388	390,6764529	121,0153899	-0,197891458	False	0,197891458
ENSMUSG00000024940	Ltbp3	3324,416375	1981,25263	2715,96752	2593,283351	2898,437231	-0,197825845	False	0,197825845
ENSMUSG00000025159	Mms19	1020,754757	1373,335561	1170,732461	1548,111576	1924,381985	0,197775134	False	0,197775134
ENSMUSG00000032555	Topbp1	536,5925889	870,0993132	467,8536852	760,0228695	971,6823954	-0,197769712	False	0,197769712
ENSMUSG00000042712	Tceal9	1286,183763	1475,13526	1534,252578	1784,987242	1942,178365	0,197750464	False	0,197750464
ENSMUSG00000034744	Nagk	493,1736466	252,5784982	565,597765	432,2138918	137,6253454	0,19768071	False	0,19768071
ENSMUSG00000020758	Itgb4	662,7532892	1143,806051	759,9876765	871,1635845	1810,485147	0,1975041	False	0,1975041
ENSMUSG00000033955	Tnks1bp1	2981,160963	4764,417983	2668,742852	2600,019152	12476,44941	-0,197352023	False	0,197352023
ENSMUSG00000036402	Gng12	1547,516642	1772,85136	1349,746899	1645,780689	2505,730426	-0,197266021	False	0,197266021
ENSMUSG00000064127	Med14	459,5854082	294,834977	400,8605519	275,0452039	212,370145	-0,197232542	False	0,197232542
ENSMUSG00000034793	G6pc3	825,7791291	704,9148962	946,6898514	620,8163173	934,9032083	0,1971359	False	0,1971359
ENSMUSG00000024661	Fth1	28645,85199	57364,13033	24987,34048	43335,8978	106843,5386	-0,19712889	False	0,19712889
ENSMUSG00000038518	Jarid2	444,020127	370,7045639	432,7097464	373,8369506	508,9764928	0,196973953	False	0,196973953
ENSMUSG00000055652	Klhl25	299,8364695	327,4877106	282,2497585	261,5736021	422,3674393	-0,196958919	False	0,196958919
ENSMUSG00000030125	Lrrc23	870,0172968	317,8839654	997,2092634	666,8442902	268,1321384	0,196852201	False	0,196852201
ENSMUSG00000026238	Ptma	9675,051106	6598,733313	11089,01094	7419,484704	8200,572304	0,196789506	False	0,196789506
ENSMUSG00000026005	Rpe	367,0129463	157,5014209	420,6290175	245,8567333	83,04977739	0,196717424	False	0,196717424
ENSMUSG00000042520	Ubap2l	4307,486767	4010,523987	4075,598652	4003,311008	4936,716053	0,196705303	False	0,196705303
ENSMUSG00000075470	Deaf1	826,5983545	721,241263	451,3799639	504,0624349	692,8724285	-0,196704554	False	0,196704554
ENSMUSG00000079036	Alkbh1	208,9024583	296,755726	239,4180831	280,6583713	648,974689	0,196702653	False	0,196702653
ENSMUSG00000039477	Tnrc18	2299,565492	1184,141781	2006,499256	1489,734635	1474,726761	-0,196680665	False	0,196680665
ENSMUSG00000032369	Plscr1	1072,365952	1942,83765	935,7073705	1030,577539	3924,695194	-0,196667997	False	0,196667997
ENSMUSG00000041025	Iffo2	392,4089314	381,2686836	437,1027388	342,403213	597,9583972	-0,196661639	False	0,196661639
ENSMUSG00000032612	Usp4	1954,671629	1827,592708	1439,803243	1487,489368	2239,971139	0,196553878	False	0,196553878
ENSMUSG00000003309	Ap1m2	447,2970283	471,5438883	557,9100284	553,4583082	390,3339537	-0,196524211	False	0,196524211
ENSMUSG00000029145	Eif2b4	445,6585776	697,2319	510,6853606	616,3257834	696,4317047	0,196495829	False	0,196495829
ENSMUSG00000021385	Ippk	190,0602758	287,1519809	233,9268426	217,7908961	423,5538647	0,196486622	False	0,196486622
ENSMUSG00000053644	Aldh7a1	765,156455	261,2218689	667,7348372	426,6007244	364,2325951	-0,196479462	False	0,196479462
ENSMUSG00000066798	Zbtb6	295,7403429	190,1541546	258,0883005	189,725059	188,6416372	-0,196466316	False	0,196466316
ENSMUSG00000018287	Spag7	484,9813933	575,2643362	606,2329443	555,7035752	732,0244664	0,196386124	False	0,196386124
ENSMUSG00000048307	Ankrd46	1196,888202	1371,414812	1072,988381	1099,058182	1036,935792	0,196376608	False	0,196376608
ENSMUSG00000056458	Mok	191,6987264	408,1591701	219,6496175	388,4311859	376,096849	0,196363235	False	0,196363235
ENSMUSG00000019984	Med23	436,647099	474,4250119	381,0920863	420,987557	525,5864483	-0,196328106	False	0,196328106
ENSMUSG00000042496	Prdm10	236,7561194	157,5014209	271,2672776	216,6682627	162,5402786	0,196313314	False	0,196313314
ENSMUSG00000067148	Polr1c	377,6628755	553,1757223	432,7097464	523,1472041	713,0416601	0,196300644	False	0,196300644
ENSMUSG00000021687	Scamp1	892,1363806	874,9011858	778,657894	781,3529057	1305,06793	-0,196274657	False	0,196274657
ENSMUSG00000014349	Ube2z	1881,760575	1920,749036	1921,934153	1642,412789	2395,392865	-0,196266313	False	0,196266313
ENSMUSG00000030351	Tspan11	1132,169401	3340,182573	1297,030991	2913,233894	1925,56841	0,196123114	False	0,196123114
ENSMUSG00000035885	Cox8a	2896,780755	4222,766755	3881,208741	3318,504582	5369,761321	0,196082769	False	0,196082769
ENSMUSG00000026889	Rbm18	528,4003356	602,1548227	461,2641967	556,8262087	805,5828407	-0,196038068	False	0,196038068
ENSMUSG00000027787	Nmd3	246,5868233	424,4855369	215,2566251	254,8378012	638,2968605	-0,196038068	False	0,196038068
ENSMUSG00000032288	Imp3	674,2224437	860,4955681	588,6609749	767,8813039	1157,951182	-0,195787665	False	0,195787665
ENSMUSG00000032046	Abhd12	1264,064679	950,7707728	1103,739328	929,5405258	1048,800046	-0,195670796	False	0,195670796
ENSMUSG00000028799	Zfp362	1062,535249	751,012873	678,717318	786,9660732	927,784656	-0,19564881	False	0,19564881
ENSMUSG00000038576	Susd4	303,1133708	96,99782631	264,6777891	161,6592219	93,72760591	-0,19562036	False	0,19562036
ENSMUSG00000021281	Tnfaip2	761,0603284	1366,612939	731,4332262	664,5990233	2773,862565	-0,195526646	False	0,195526646
ENSMUSG00000003970	Rpl8	5275,811103	10175,16802	6041,462729	7690,039374	13373,38701	0,195504979	False	0,195504979
ENSMUSG00000026888	Grb14	824,9599038	505,1569964	720,4507453	610,712616	456,7737756	-0,195424199	False	0,195424199
ENSMUSG00000025971	Maip1	340,7977358	547,4134752	297,6252317	392,9217198	965,7502685	-0,195418912	False	0,195418912
ENSMUSG00000022262	Dnah5	865,1019448	463,8608922	549,1240437	755,5323356	466,2651788	-0,19537665	False	0,19537665
ENSMUSG00000045176	Borcs6	376,0244249	667,46029	430,5132503	577,0336114	938,4624845	0,195231265	False	0,195231265
ENSMUSG00000022407	Adsl	398,962734	352,4574481	293,2322393	279,5357378	456,7737756	0,19522583	False	0,19522583
ENSMUSG00000021703	Serinc5	1199,345878	915,2369156	695,1910393	826,2582451	1047,61362	-0,195141054	False	0,195141054
ENSMUSG00000084989	Crocc2	235,1176687	289,0727299	205,3723923	436,7044258	246,7764814	-0,195140702	False	0,195140702
ENSMUSG00000020069	Hnrnph3	716,8221607	626,1641857	1057,612908	947,5026615	681,0081746	-0,195074245	False	0,195074245
ENSMUSG00000056938	Acbd4	619,3343469	640,5698035	795,1316153	1113,652417	541,0099784	-0,195063253	False	0,195063253
ENSMUSG00000002948	Map2k7	1222,284187	1732,51563	1399,168063	1640,167522	1633,707764	0,194989507	False	0,194989507
ENSMUSG00000024997	Prdx3	723,3759633	631,9264328	608,4294404	534,373539	471,0108803	-0,194989047	False	0,194989047
ENSMUSG00000026856	Dolpp1	465,3199855	419,6836643	532,6503224	381,695385	290,6742209	0,194965582	False	0,194965582
ENSMUSG00000036990	Otud4	938,8322242	820,1598383	956,5740841	890,2483538	1150,83263	-0,194962262	False	0,194962262
ENSMUSG00000041571	Selenow	3376,027571	2949,310145	4106,349599	3281,457677	3518,93771	-0,194949144	False	0,194949144
ENSMUSG00000049339	Retreg2	1309,941297	822,0805874	1144,374507	991,2853675	715,4145109	-0,194942897	False	0,194942897
ENSMUSG00000023971	Rrp36	244,1291473	251,6181237	262,4812929	213,3003622	344,0633635	-0,194758138	False	0,194758138
ENSMUSG00000021835	Bmp4	1344,348761	1864,086939	1538,64557	1761,411938	2324,207341	0,194753482	False	0,194753482
ENSMUSG00000031060	Rbm10	856,9096916	622,3226876	980,7355421	801,5603085	708,2959586	0,194720993	False	0,194720993
ENSMUSG00000063904	Dpp3	901,9670845	1110,192943	830,2755541	788,0887066	933,7167829	-0,194716759	False	0,194716759
ENSMUSG00000055932	Fto	1522,120657	1112,113692	1329,978434	1177,642526	1154,391906	-0,194679872	False	0,194679872
ENSMUSG00000066551	Hmgb1	2547,790766	3564,910211	2915,848672	3220,835469	5090,951354	0,194669045	False	0,194669045
ENSMUSG00000028693	Nasp	201,5294303	327,4877106	230,6320984	399,6575207	612,1955019	0,194602777	False	0,194602777
ENSMUSG00000042210	Abhd14a	268,7059071	96,99782631	307,5094645	196,4608599	96,10045669	0,194602777	False	0,194602777
ENSMUSG00000022438	Parvb	3748,775094	3199,007519	3806,527871	3511,597542	4290,114215	0,194596782	False	0,194596782
ENSMUSG00000023110	Prmt5	678,3185704	872,0200623	595,2504634	592,7504802	854,2262817	-0,194538064	False	0,194538064
ENSMUSG00000046668	Cxxc5	384,2166781	243,9351276	315,1972011	225,6493305	335,7583857	-0,194496713	False	0,194496713
ENSMUSG00000026222	Sp100	2252,050423	1172,617286	1968,060573	1632,309088	1105,748465	-0,194464505	False	0,194464505
ENSMUSG00000038780	Smurf1	997,8164477	1518,352113	872,0089814	1018,228571	2010,991038	-0,194431456	False	0,194431456
ENSMUSG00000020329	Polrmt	358,0014677	635,7679309	409,6465366	587,1373128	482,8751342	0,194414115	False	0,194414115
ENSMUSG00000023008	Fmnl3	1577,008754	905,6331704	1378,30135	1069,869711	1182,866115	-0,194299317	False	0,194299317
ENSMUSG00000024941	Scyl1	1694,977201	1822,790835	1481,53667	1708,648165	2511,662553	-0,194171532	False	0,194171532
ENSMUSG00000000902	Smarcb1	1000,274124	897,9501743	1144,374507	1009,247503	616,9412035	0,194163842	False	0,194163842
ENSMUSG00000036501	Fam13b	456,3085069	371,6649384	305,3129683	389,5538194	522,0271722	0,194115353	False	0,194115353
ENSMUSG00000024054	Smchd1	770,8910323	488,8306296	539,2398109	434,4591588	673,8896222	-0,194014637	False	0,194014637
ENSMUSG00000037278	Tmem97	338,3400599	387,0309307	345,9481475	319,9505433	281,1828177	0,193974864	False	0,193974864
ENSMUSG00000062949	Atp11c	506,2812518	767,3392398	442,5939792	711,7496296	503,0443659	-0,193955235	False	0,193955235
ENSMUSG00000020289	Nprl3	516,931181	582,9473324	517,2748492	612,9578829	452,0280741	-0,193559851	False	0,193559851
ENSMUSG00000032452	Clstn2	303,9325961	121,9675638	265,7760372	187,479792	224,2343989	-0,193540375	False	0,193540375
ENSMUSG00000030560	Ctsc	3249,047645	2348,115696	2841,167802	2387,841423	2044,210949	-0,193532859	False	0,193532859
ENSMUSG00000027502	Rtf2	936,3745482	1337,801703	1070,791885	1303,377476	1676,419078	0,193520485	False	0,193520485
ENSMUSG00000024654	Asrgl1	242,4906967	251,6181237	192,1934153	277,2904709	145,9303231	0,19346864	False	0,19346864
ENSMUSG00000029373	Pf4	154,8335867	135,412807	256,9900525	400,7801542	257,4543099	-0,193354265	False	0,193354265
ENSMUSG00000030483	Cyp2b10	5172,588712	8341,813063	6633,418448	13219,00929	4523,840017	-0,193338573	False	0,193338573
ENSMUSG00000038147	Cd84	435,0086484	365,9026913	417,3342732	497,326634	326,2669826	0,193149612	False	0,193149612
ENSMUSG00000067851	Arfgef1	1384,490802	1211,032267	873,1072295	1132,737187	1049,986471	-0,193118164	False	0,193118164
ENSMUSG00000018965	Ywhah	2214,366058	1709,466642	2531,461842	2021,862907	1937,432664	0,193077003	False	0,193077003
ENSMUSG00000027133	Nop10	867,5596208	828,803209	1072,988381	758,900236	838,8027516	-0,193052663	False	0,193052663
ENSMUSG00000015143	Actn1	2809,123645	8784,545716	3211,277408	6057,730286	10267,32534	0,193027172	False	0,193027172
ENSMUSG00000020689	Itgb3	675,0416691	417,7629153	430,5132503	590,5052132	135,2524946	-0,193026763	False	0,193026763
ENSMUSG00000026478	Lamc1	2749,320196	2166,604912	2573,195269	2136,371522	3142,840861	0,192994285	False	0,192994285
ENSMUSG00000025326	Ube3a	1032,223911	1000,710248	1124,606042	1179,887793	710,6688094	0,19289371	False	0,19289371
ENSMUSG00000054693	Adam10	1358,275591	947,8896492	1188,304431	1020,473838	812,701393	-0,192871743	False	0,192871743
ENSMUSG00000018648	Dusp14	787,2755388	899,8709233	741,317459	688,1743265	594,399121	0,192849424	False	0,192849424

ENSMUSG00000036452	Arhgap26	407,9742126	649,2131741	356,9306284	491,7134665	569,4841878	-0,19283426	False	0,19283426
ENSMUSG00000028249	Sdcbp	3589,026156	3875,11118	3337,575938	3140,005858	4912,987545	-0,192825186	False	0,192825186
ENSMUSG00000028057	Rit1	744,6758218	1047,768599	851,1422677	1042,926508	1596,928577	0,192787781	False	0,192787781
ENSMUSG00000023027	Atf1	448,1162536	617,520815	392,0745672	513,0435028	1071,342128	-0,192744994	False	0,192744994
ENSMUSG00000021748	Pdhb	905,2439858	1660,487542	1034,549698	1343,792282	1969,466149	0,19262436	False	0,19262436
ENSMUSG00000021133	Susd6	2585,475131	3192,284898	2262,39106	2955,893967	4688,753146	-0,192581103	False	0,192581103
ENSMUSG00000024319	Vps52	1019,935532	1015,115865	1033,45145	892,4936208	1350,152095	-0,192564202	False	0,192564202
ENSMUSG00000049957	Ccdc137	424,3587191	444,6534018	600,7417038	379,450118	371,3511475	-0,192500284	False	0,192500284
ENSMUSG00000023009	Nckap5l	444,020127	638,6490544	507,3906164	575,9109779	829,3113485	0,192471765	False	0,192471765
ENSMUSG00000024538	Ppic	865,9211701	854,733321	898,3669355	757,7776025	1179,306839	-0,192461195	False	0,192461195
ENSMUSG00000021870	Slmap	1292,737565	1131,321182	939,0021147	1120,388218	1067,782852	-0,192420859	False	0,192420859
ENSMUSG00000006418	Rnf114	1970,23691	1306,109344	1724,249497	1468,404599	1695,401884	-0,19240057	False	0,19240057
ENSMUSG00000021824	Ap3m1	986,3472931	601,1944482	863,2229967	694,9101274	346,4362142	-0,19236241	False	0,19236241
ENSMUSG00000057766	Ankrd29	280,994287	205,5201468	299,8217279	321,0731768	121,0153899	0,192361345	False	0,192361345
ENSMUSG00000025366	Esyt1	2347,899786	1547,163348	2054,822172	1538,007875	1545,912285	-0,192357286	False	0,192357286
ENSMUSG00000022092	Ppp3cc	290,8249909	332,2895832	242,7128273	297,4978736	200,5058911	0,19228982	False	0,19228982
ENSMUSG00000021807	Rtraf	1109,231092	1732,51563	1267,378293	1822,034147	1913,704156	0,19228725	False	0,19228725
ENSMUSG00000026223	Itm2c	3018,845328	2357,719442	2085,573118	1688,440762	3448,938612	0,192155609	False	0,192155609
ENSMUSG00000021712	Trim23	238,39457	265,0633669	208,6671366	325,5637107	312,0298779	-0,19214797	False	0,19214797
ENSMUSG00000030701	Plekhb1	2654,290058	1501,065372	3032,262969	2611,245487	2026,414568	0,192068839	False	0,192068839
ENSMUSG00000039474	Wfs1	527,5811103	484,028757	477,737918	374,9595841	602,7040988	0,192056965	False	0,192056965
ENSMUSG00000036533	Cdc42ep3	559,530898	489,7910041	635,8856426	621,9389508	523,2135975	-0,192051524	False	0,192051524
ENSMUSG00000031201	Brcc3	358,820693	436,0100311	314,098953	429,9686248	454,4009249	-0,192043958	False	0,192043958
ENSMUSG00000042724	Map3k9	179,4103465	303,4783477	157,0494765	253,7151677	298,9791986	-0,192043958	False	0,192043958
ENSMUSG00000021024	Psma6	998,635673	1746,921248	874,2054776	1141,718254	2523,526807	-0,191986025	False	0,191986025
ENSMUSG00000047193	Dync2h1	352,2668904	402,396923	287,7409989	364,8558827	339,3176619	0,191950391	False	0,191950391
ENSMUSG00000015305	Sash1	2066,086274	3220,135759	1808,8146	3254,514474	5253,491632	-0,191855956	False	0,191855956
ENSMUSG00000022108	Itm2b	20929,56864	13380,89816	16766,95355	18323,62375	14438,79701	-0,191837734	False	0,191837734
ENSMUSG00000040374	Pex2	333,4247079	355,3385716	303,1164721	267,1867695	380,8425506	0,19183366	False	0,19183366
ENSMUSG00000061414	Cracr2a	752,8680751	403,3572975	859,9282524	839,729847	459,1466264	0,191819211	False	0,191819211
ENSMUSG00000052214	Opa3	1032,223911	1219,675638	969,7530612	903,7199556	1047,61362	-0,191808271	False	0,191808271
ENSMUSG00000017002	Slpi	409,6126633	819,1994638	467,8536852	1834,383115	2044,210949	0,1917971	False	0,1917971
ENSMUSG00000030064	Frmd4b	1191,153625	1166,855039	1096,051591	1042,926508	1567,267942	-0,191721993	False	0,191721993
ENSMUSG00000036966	Spryd3	906,8824365	890,2671781	778,657894	756,6549691	1035,749367	0,191687494	False	0,191687494
ENSMUSG00000063704	Mapk15	1067,4506	3479,436878	1219,055377	2823,423215	3906,898813	0,191594357	False	0,191594357
ENSMUSG00000031422	Morf4l2	1676,954243	2091,6957	1896,674447	1468,404599	2460,646261	-0,191593787	False	0,191593787
ENSMUSG00000029304	Spp1	1711,361707	1954,362144	3741,731234	2426,010962	2325,393767	0,191553109	False	0,191553109
ENSMUSG00000078974	Sec61g	442,3816763	904,6727959	505,1941202	514,1661362	1016,76656	0,191546219	False	0,191546219
ENSMUSG00000062937	Mtap	344,8938625	259,3011198	302,018224	213,3003622	272,87784	-0,19151685	False	0,19151685
ENSMUSG00000036529	Sbf1	1564,720374	2873,440558	1786,849638	2611,245487	4124,01466	0,191513377	False	0,191513377
ENSMUSG00000079559	Colca2	271,9828084	238,1728804	210,8636328	176,2534572	201,6923165	-0,191506314	False	0,191506314
ENSMUSG00000036986	Pml	1400,875308	719,3205139	1226,743114	902,5973221	732,0244664	-0,191495375	False	0,191495375
ENSMUSG00000022100	Xpo7	1181,322921	784,6259812	1034,549698	883,5125529	775,9222059	-0,191400435	False	0,191400435
ENSMUSG00000028458	Tesk1	1752,322973	2243,434874	1590,263231	1534,639974	3053,858957	-0,191368465	False	0,191368465
ENSMUSG00000039463	Slc9a8	817,5868759	877,7823094	716,057753	766,7586704	1035,749367	-0,191296086	False	0,191296086
ENSMUSG00000022797	Tfrc	751,2296244	597,3529502	857,7317563	564,6846431	338,1312365	0,191272579	False	0,191272579
ENSMUSG00000046808	Atp10d	342,4361865	733,7261317	390,9763191	843,0977474	1417,778343	0,191246064	False	0,191246064
ENSMUSG00000030350	Prmt8	187,6025998	832,644707	214,158377	755,5323356	454,4009249	0,19099829	False	0,19099829
ENSMUSG00000009927	Rps25	3062,264271	5317,593706	3495,723662	4077,404818	7354,651	0,190992353	False	0,190992353
ENSMUSG00000020077	Srgn	3193,340323	6793,68934	3645,085402	4141,394927	14190,8341	0,190886303	False	0,190886303
ENSMUSG00000034573	Ptpn13	1246,041722	936,365155	1091,658599	1060,888644	1000,156605	-0,190830632	False	0,190830632
ENSMUSG00000030271	Ogg1	230,2023168	256,4199963	274,5620219	287,3941722	201,6923165	-0,190746227	False	0,190746227
ENSMUSG00000030222	Rerg	270,3443578	407,1987956	263,579541	236,8756654	354,741192	-0,190668251	False	0,190668251
ENSMUSG00000055762	Eef1d	1463,136433	1282,099981	1583,673742	1445,951929	1880,484245	-0,190555531	False	0,190555531
ENSMUSG00000051579	Tceal8	710,2683581	810,5560931	923,6266415	891,3709873	823,3792215	0,190547813	False	0,190547813
ENSMUSG00000029152	Ociad1	2179,139369	2714,018388	1988,927286	1909,559558	3174,874347	-0,19048833	False	0,19048833
ENSMUSG00000028703	Lrrc41	813,4907493	1027,600734	746,8086994	712,8722631	1749,977452	-0,190482351	False	0,190482351
ENSMUSG00000031505	Naxd	1170,672992	1335,880954	1359,631132	1633,431721	1800,993744	0,190453311	False	0,190453311
ENSMUSG00000033186	Mzt1	421,0818178	443,6930273	369,0113574	420,987557	519,6543214	-0,190435362	False	0,190435362
ENSMUSG00000078652	Psme3	1629,439174	1780,534356	1506,796376	1427,989793	2339,630872	-0,190389831	False	0,190389831
ENSMUSG00000042423	Fbrs	2047,244091	2837,9067	2335,973682	2541,642211	4945,021031	0,190340896	False	0,190340896
ENSMUSG00000027867	Spag17	396,505058	283,3104828	316,2954492	452,4212946	320,3348556	0,190327566	False	0,190327566
ENSMUSG00000044018	Mrpl50	302,2941455	303,4783477	297,6252317	264,9415025	361,8597443	-0,190279182	False	0,190279182
ENSMUSG00000003154	Foxj2	1209,176582	1089,064703	1068,595389	1059,76601	1822,349401	-0,190279182	False	0,190279182
ENSMUSG00000026701	Prdx6	21066,37927	30403,53649	24036,25764	25844,14546	51679,50362	0,190269915	False	0,190269915
ENSMUSG00000090958	Lrrc32	1927,637193	3886,635674	2327,187697	2199,238998	6845,674508	0,190170852	False	0,190170852
ENSMUSG00000002983	Relb	555,4347714	1723,87226	633,6891464	916,068924	3074,028189	0,19015781	False	0,19015781
ENSMUSG00000035513	Ntng2	362,0975943	202,6390233	317,3936673	262,6962355	201,6923165	-0,190105112	False	0,190105112
ENSMUSG00000048911	Rnf24	280,1750617	198,7975252	151,5582361	157,1686879	245,590056	-0,190076401	False	0,190076401
ENSMUSG00000019979	Apaf1	322,7747787	412,0006682	383,2885825	368,2237832	495,9258135	0,19005496	False	0,19005496
ENSMUSG00000028073	Pear1	961,7705334	2472,964384	1097,149839	2381,105622	4241,470774	0,189995939	False	0,189995939
ENSMUSG00000040270	Bach2	279,3558363	244,8955021	177,9161902	229,017231	214,7429958	-0,189937685	False	0,189937685
ENSMUSG00000020674	Pxdn	2594,486609	1548,123723	2274,471789	1644,658056	1360,829924	-0,189917549	False	0,189917549
ENSMUSG00000053477	Tcf4	3466,142357	3038,624975	4637,901673	4875,597226	4000,626419	-0,189912279	False	0,189912279
ENSMUSG00000029686	Cul1	1384,490802	2632,386554	1579,28075	2408,048826	3509,446307	0,189912194	False	0,189912194
ENSMUSG00000049811	Fam161a	439,9240004	305,3990967	416,2360251	501,8171679	77,11765043	0,189907518	False	0,189907518
ENSMUSG00000052534	Pbx1	2166,850989	1899,620796	2131,699538	2506,840573	2565,051696	-0,189888489	False	0,189888489
ENSMUSG00000031986	Sprtn	521,846533	753,8939966	595,2504634	712,8722631	1365,575625	0,189871244	False	0,189871244
ENSMUSG00000055945	Prr18	610,3228683	848,0106993	544,7310514	599,4862811	535,0778515	-0,189823835	False	0,189823835
ENSMUSG00000022131	Gpr180	303,1133708	345,7348265	310,8042087	322,1958103	201,6923165	0,189808443	False	0,189808443
ENSMUSG00000038250	Usp38	430,9125218	509,958869	377,7973421	445,6854936	885,0733419	-0,18978247	False	0,18978247
ENSMUSG00000031748	Gnao1	1185,419047	1224,47751	1169,634213	1564,951078	1039,308643	-0,18977299	False	0,18977299
ENSMUSG00000062683	Atp5g2	843,8020863	796,1504754	813,8018328	739,8154668	1129,476972	-0,189739193	False	0,189739193
ENSMUSG00000074918	Inafm2	680,7762464	1123,638186	776,4613978	815,0319103	1080,833531	0,189733503	False	0,189733503
ENSMUSG00000041697	Cox6a1	2481,433514	3902,001666	2857,641523	2830,159016	5003,155875	0,189709315	False	0,189709315
ENSMUSG00000027341	Tmem230	339,9785105	189,19378	387,6815749	230,1398645	124,5746661	0,189428612	False	0,189428612
ENSMUSG00000115338	Pnp	690,6069503	787,5071047	893,9739432	938,5215937	885,0733419	0,189428086	False	0,189428086
ENSMUSG00000024146	Cript	838,8867344	1355,088445	956,5740841	1213,566798	1598,115002	0,189400673	False	0,189400673
ENSMUSG00000032423	Syncrip	1079,73898	1376,216684	1231,136106	1317,971712	1551,844412	0,189307672	False	0,189307672
ENSMUSG00000047824	Pygo2	1024,850883	898,9105488	1054,318164	905,9652226	1249,305937	-0,189164547	False	0,189164547
ENSMUSG00000032860	P2ry2	195,794853	523,4041123	182,3091825	171,7629232	918,2932528	-0,188924191	False	0,188924191
ENSMUSG00000049076	Acap2	1336,975733	1608,627318	1172,928957	1482,998834	2171,158466	-0,188857646	False	0,188857646
ENSMUSG00000022892	App	13386,96106	9196,546384	8729,974047	8473,637547	11744,42495	-0,18885242	False	0,18885242
ENSMUSG00000079184	Mphosph8	590,6614604	825,9220854	673,2260776	1094,567648	911,1747005	0,188759579	False	0,188759579
ENSMUSG00000032855	Pkd1	3215,459407	2479,687005	3664,853868	3454,343234	3216,399236	0,188730797	False	0,188730797
ENSMUSG00000050234	Gja4	1657,292836	841,2880777	1454,080468	633,1652857	883,8869165	-0,188721433	False	0,188721433
ENSMUSG00000036104	Rab3gap1	808,5753973	1057,372344	709,4682645	885,7578199	1008,461583	-0,188644155	False	0,188644155
ENSMUSG00000019278	Dpep1	1729,384664	1600,944321	2429,324769	3450,975334	1517,438075	-0,188621158	False	0,188621158
ENSMUSG00000074582	Arfgef2	1262,426228	2206,940642	1438,704995	1770,393006	2194,886974	0,188571715	False	0,188571715
ENSMUSG00000007613	Tgfbr1	744,6758218	971,8990121	653,457612	825,1356117	1103,375614	-0,188518861	False	0,188518861
ENSMUSG00000067158	Col4a4	2563,356047	1649,923422	2100,948591	2921,092329	790,1593106	0,188474078	False	0,188474078
ENSMUSG00000024896	Minpp1	247,4060486	223,7672627	364,618365	245,8567333	217,1158466	-0,188415744	False	0,188415744
ENSMUSG00000033257	Ttll4	688,1492743	1054,491221	784,1491344	1003,634336	1138,968376	0,188406511	False	0,188406511
ENSMUSG00000035179	Ppp1r32	196,6140784	270,8256141	224,0426098	270,5546699	266,945713	0,188406511	False	0,188406511
ENSMUSG00000062963	Ufc1	660,2956132	1292,664101	752,2999399	920,5594579	1333,54214	0,188195914	False	0,188195914
ENSMUSG00000041231	Ublcp1	564,44625	768,2996143	745,7104514	707,2590957	643,0425621	0,188078025	False	0,188078025
ENSMUSG00000043015	Nemp2	299,0172442	117,1656912	262,4812929	239,1209324	34,40633635	-0,188014081	False	0,188014081
ENSMUSG00000006576	Slc4a3	425,1779445	140,2146796	484,3274066	315,4600094	104,4054344	0,187915882	False	0,187915882
ENSMUSG00000027177	Hipk3	2632,170974	4634,767424	2310,713976	3583,446085	6088,735108	-0,187914512	False	0,187914512
ENSMUSG00000022246	Rai14	1749,046072	2475,845507	1992,222031	2184,644762	3365,888835	0,187810151	False	0,187810151

ENSMUSG00000018507	Trpv2	376,0244249	332,2895832	328,3761781	304,2336745	428,2995662	0,18779384	False	0,18779384
ENSMUSG00000029782	Tmem209	510,3773784	425,4459114	448,0852197	414,2517561	279,9963923	-0,187791246	False	0,187791246
ENSMUSG00000027714	Exosc9	232,6599927	384,1498072	204,2741443	259,3283351	516,0950452	-0,187716544	False	0,187716544
ENSMUSG00000033499	Larp4b	1829,330154	3342,103322	2083,376622	3390,353125	2508,103277	0,187608192	False	0,187608192
ENSMUSG00000037553	Zdhhc18	2129,985849	2461,439889	1870,316493	1985,938635	2611,322286	-0,187561424	False	0,187561424
ENSMUSG00000023022	Lima1	1852,268463	1120,757062	1626,505417	1445,951929	1039,308643	-0,187517587	False	0,187517587
ENSMUSG00000020719	Ddx5	14785,37869	12983,30311	19916,72907	14122,72924	16739,27585	-0,187513726	False	0,187513726
ENSMUSG00000017405	Nek8	211,3601342	267,9444905	185,6039268	225,6493305	215,9294212	-0,187476055	False	0,187476055
ENSMUSG00000026193	Fn1	4193,614447	2966,596886	2134,994282	1987,061269	3682,664414	-0,187444292	False	0,187444292
ENSMUSG00000027132	Katnbl1	212,1793596	260,2614944	241,6145792	270,5546699	243,2172052	0,18742319	False	0,18742319
ENSMUSG00000022475	Hdac7	5427,367788	3147,147295	4766,396699	4459,100203	3238,941318	-0,187353647	False	0,187353647
ENSMUSG00000031918	Mtmr2	1040,416165	1256,169869	913,7424087	1057,520743	1663,368399	-0,187301299	False	0,187301299
ENSMUSG00000026821	Ralgds	1537,685938	1496,263499	1662,747604	1350,528083	3394,363044	-0,187237233	False	0,187237233
ENSMUSG00000027665	Pik3ca	1650,739033	1879,452932	2007,597504	2414,784627	1898,280626	0,187200724	False	0,187200724
ENSMUSG00000040525	Cblc	213,8178102	276,5878612	187,8004229	259,3283351	308,4706017	-0,187181717	False	0,187181717
ENSMUSG00000060675	Pla2g16	691,4261756	467,7023902	607,3311923	505,1850684	493,5529628	-0,187091754	False	0,187091754
ENSMUSG00000029231	Pdgfra	2863,192516	6797,530838	2514,98812	2781,885776	6279,749596	-0,187073093	False	0,187073093
ENSMUSG00000050947	Amigo1	548,8809688	279,4689847	482,1309104	410,8838556	231,3529513	-0,187068391	False	0,187068391
ENSMUSG00000058317	Ube2e2	317,8594267	197,8371507	154,8529803	184,1118916	361,8597443	0,187041747	False	0,187041747
ENSMUSG00000061898	Rbak	190,0602758	702,0337726	216,3548732	684,806426	570,6706132	0,186942589	False	0,186942589
ENSMUSG00000064068	Mtx1	507,9197025	764,4581163	613,9206809	578,1562449	1044,054344	0,186858991	False	0,186858991
ENSMUSG00000042167	Papd4	603,7690657	887,3860546	530,4538262	719,608064	1268,288743	-0,186769666	False	0,186769666
ENSMUSG00000028538	St3gal3	435,0086484	213,203143	382,1903344	240,2435658	129,3203676	-0,18675279	False	0,18675279
ENSMUSG00000032413	Rasa2	290,0057656	367,8234404	254,7935563	372,7143171	422,3674393	-0,18675279	False	0,18675279
ENSMUSG00000050989	Selenon	1595,031711	944,0481511	1401,36456	962,0968969	1269,475169	-0,18675279	False	0,18675279
ENSMUSG00000030583	Sipa1l3	2396,23408	1476,095634	2105,341584	1436,970861	1566,081516	-0,186714523	False	0,186714523
ENSMUSG00000038902	Pogz	759,4218777	683,7866568	864,3212448	691,5422269	659,6525175	0,186666062	False	0,186666062
ENSMUSG00000029723	Tsc22d4	1412,344463	1751,723121	1241,020339	1420,131359	2888,945828	-0,186565237	False	0,186565237
ENSMUSG00000004266	Ptpn6	1239,487919	892,1879272	828,0790579	617,4484169	1089,138509	-0,186556772	False	0,186556772
ENSMUSG00000028833	Ncdn	643,9111067	794,2297263	571,0890055	565,8072766	1480,658888	-0,186550804	False	0,186550804
ENSMUSG00000040209	Zfp704	1462,317208	659,7772938	1284,950262	1139,472988	485,247985	-0,18654378	False	0,18654378
ENSMUSG00000032757	Bet1	389,9512554	259,3011198	342,6534033	303,111041	112,7104122	-0,18654378	False	0,18654378
ENSMUSG00000031578	Mak16	298,1980189	584,8680814	339,358659	454,6665615	641,8561367	0,186540152	False	0,186540152
ENSMUSG00000059326	Csf2ra	1395,959956	1811,266341	1916,442913	1601,997983	1588,623599	0,186519782	False	0,186519782
ENSMUSG00000035245	Eogt	1454,124955	1108,272194	1569,396517	1654,761757	1176,933988	0,186472274	False	0,186472274
ENSMUSG00000058070	Eml1	1125,615599	1016,07624	1208,072896	1280,924807	559,9927847	0,186471562	False	0,186471562
ENSMUSG00000024587	Nars	1287,822213	1442,482526	1266,280045	1457,178264	1131,849823	-0,18625089	False	0,18625089
ENSMUSG00000023963	Cyp39a1	400,6011847	239,133255	354,7341322	455,789195	189,8280626	0,186200037	False	0,186200037
ENSMUSG00000038803	Ost4	950,3013788	878,7426839	1268,476541	835,239313	1076,08783	-0,186195504	False	0,186195504
ENSMUSG00000029534	St7	205,625557	393,7535524	205,3723923	180,7439911	487,6208358	-0,186071901	False	0,186071901
ENSMUSG00000034867	Ankrd27	714,3644847	328,4480851	812,7035847	641,0237201	83,04977739	0,1860689	False	0,1860689
ENSMUSG00000020230	Prmt2	873,2941981	478,2665099	767,6754131	451,2986611	342,8769381	-0,185971313	False	0,185971313
ENSMUSG00000020961	Ston2	585,7461085	372,625313	481,0326623	287,3941722	514,9086198	-0,185959043	False	0,185959043
ENSMUSG00000045394	Epcam	749,5911738	1847,760573	658,9488525	1009,247503	2570,983823	-0,185937478	False	0,185937478
ENSMUSG00000039230	Tbcd	649,6456839	854,733321	571,0890055	623,0615843	723,7194887	-0,185937478	False	0,185937478
ENSMUSG00000022761	Lztr1	1006,008701	809,5957186	1144,374507	1017,105938	657,2796668	0,185916482	False	0,185916482
ENSMUSG00000005378	Bud23	250,6829499	417,7629153	329,4744262	285,1489053	543,3828292	0,185851619	False	0,185851619
ENSMUSG00000070730	Rmdn3	567,7231513	474,4250119	645,7698754	534,373539	569,4841878	0,185832567	False	0,185832567
ENSMUSG00000020392	Cdkn2aipnl	632,4419521	621,3623131	719,3524973	636,5331861	494,7393882	0,185765824	False	0,185765824
ENSMUSG00000034586	Hid1	956,8551814	2031,192105	841,258035	1186,623594	3421,650828	-0,185752212	False	0,185752212
ENSMUSG00000073139	Tmem185a	515,2927304	481,1476335	342,6534033	352,5069144	586,0941433	0,185740181	False	0,185740181
ENSMUSG00000030881	Arfip2	857,7289169	1045,84785	1134,490274	975,5684987	1025,071538	0,185721414	False	0,185721414
ENSMUSG00000028393	Alad	670,1263171	467,7023902	762,1841727	682,561159	382,028976	0,185706584	False	0,185706584
ENSMUSG00000025413	Ttc4	491,5351959	459,0590196	588,6609749	432,2138918	612,1955019	-0,18554928	False	0,18554928
ENSMUSG00000018379	Srsf1	2467,506684	2110,90319	2806,023863	2256,493305	1096,257062	0,185473286	False	0,185473286
ENSMUSG00000018604	Tbx3	2532,225484	2088,814577	2879,606485	2318,238147	2277,936751	0,185465796	False	0,185465796
ENSMUSG00000034343	Ube2f	777,4448349	1209,111518	884,0897104	937,3989602	1652,69057	0,185452461	False	0,185452461
ENSMUSG00000050213	Snip1	274,4404844	264,1029924	292,1339913	241,3661993	352,3683412	-0,185269661	False	0,185269661
ENSMUSG00000040043	Rbms2	1002,7318	577,1850853	881,8932142	724,098598	442,5366709	-0,185259901	False	0,185259901
ENSMUSG00000026643	Nmt2	707,8106821	804,793846	897,2686874	955,3610959	1171,001861	0,185255739	False	0,185255739
ENSMUSG00000090213	Tmem189	1186,238273	2086,893827	1348,648651	1872,552653	3246,05987	0,185120723	False	0,185120723
ENSMUSG00000022299	Slc25a32	331,7862572	320,765089	304,2147202	377,204851	247,9629068	0,1850941	False	0,1850941
ENSMUSG00000071547	Nt5dc2	668,4878665	572,3832127	759,9876765	584,8920458	481,6887088	0,18507465	False	0,18507465
ENSMUSG00000024735	Prpf19	1217,368835	1070,817587	1052,121668	830,7487791	1015,580135	-0,185053599	False	0,185053599
ENSMUSG00000097336	Fendrr	5882,037845	6329,828448	7103,468629	12362,43994	5174,001131	-0,185035697	False	0,185035697
ENSMUSG00000019432	Ddx39b	2716,551183	4972,819254	3088,273622	3401,57946	8697,684543	0,185024352	False	0,185024352
ENSMUSG00000029864	Gstk1	802,84082	564,7002165	912,6441606	898,1067882	778,2950567	0,184938492	False	0,184938492
ENSMUSG00000011306	Sugp1	705,3530061	706,8356452	620,5101694	926,1726253	812,701393	-0,184890605	False	0,184890605
ENSMUSG00000017897	Eya2	1120,700247	980,5423828	710,5665126	801,5603085	985,9195001	-0,184858691	False	0,184858691
ENSMUSG00000025239	Limd1	1544,239741	1895,779298	1358,532884	1784,987242	2150,989234	-0,184847259	False	0,184847259
ENSMUSG00000025059	Gk	209,7216836	259,3011198	184,5056787	190,8476925	298,9791986	-0,184810813	False	0,184810813
ENSMUSG00000071359	Tbpl1	366,193721	481,1476335	416,2360251	438,9496927	646,6018382	0,184794782	False	0,184794782
ENSMUSG00000021843	Ktn1	998,635673	999,7498732	878,5984699	1211,321531	1257,610915	-0,184754456	False	0,184754456
ENSMUSG00000036686	Cc2d1a	565,2654753	858,574819	642,4751311	743,1833672	863,7176848	0,184712027	False	0,184712027
ENSMUSG00000029073	Cptp	263,7905551	290,0331044	299,8217279	246,9793668	149,4895993	0,184712027	False	0,184712027
ENSMUSG00000049037	Clec4a1	397,3242834	349,5763245	306,4112164	277,2904709	98,47330747	-0,18470949	False	0,18470949
ENSMUSG00000041560	Nop53	2133,26275	3573,553581	1876,905981	3180,420664	4975,868091	-0,184705287	False	0,184705287
ENSMUSG00000038806	Sde2	288,3673149	312,1217183	305,3129683	253,7151677	570,6706132	-0,184697882	False	0,184697882
ENSMUSG00000031536	Polb	364,5552703	320,765089	520,5695934	322,1958103	418,8081631	-0,184620426	False	0,184620426
ENSMUSG00000021474	Sfxn1	476,78914	516,6814906	419,5307694	531,0056385	602,7040988	-0,18457475	False	0,18457475
ENSMUSG00000033294	Noc4l	285,0904136	564,7002165	323,9831858	359,2427153	791,345736	0,184499413	False	0,184499413
ENSMUSG00000032469	Dbr1	159,7489387	279,4689847	140,5757552	277,2904709	581,3484417	-0,184458549	False	0,184458549
ENSMUSG00000059498	Fcgr3	752,8680751	1438,641028	855,5352601	1297,764309	1628,962062	0,184430231	False	0,184430231
ENSMUSG00000034403	Pja1	752,8680751	637,6886799	855,5352601	772,3718378	462,7059026	0,184430231	False	0,184430231
ENSMUSG00000039456	Morc3	471,8737881	518,6022397	535,9450667	565,8072766	415,2488869	-0,184424733	False	0,184424733
ENSMUSG00000041341	Atg2b	479,246816	451,3760234	408,5482885	478,2418647	544,5692546	0,184346686	False	0,184346686
ENSMUSG00000042198	Chchd7	201,5294303	311,1613438	289,9374951	324,4410772	228,9801005	0,184231689	False	0,184231689
ENSMUSG00000009687	Fxyd5	4467,235706	6123,347926	5401,184094	5075,425987	8601,584087	0,184146537	False	0,184146537
ENSMUSG00000039069	Mtg2	290,0057656	114,2845676	329,4744262	163,9044888	84,23620278	0,184084905	False	0,184084905
ENSMUSG00000069763	Tmem100	8348,725303	8680,825268	11556,86462	8908,096705	7348,718873	-0,184063179	False	0,184063179
ENSMUSG00000028524	Sgip1	410,4318886	245,8558766	361,3236208	352,5069144	237,2850783	-0,183851255	False	0,183851255
ENSMUSG00000049103	Ccr2	1259,968552	379,3479346	1109,230568	556,8262087	91,35475513	-0,183828446	False	0,183828446
ENSMUSG00000026288	Inpp5d	1425,452068	2036,954353	1618,817681	2202,606898	2485,561195	0,183520983	False	0,183520983
ENSMUSG00000026028	Trak2	1572,093402	1785,336229	1242,118587	1695,176563	1520,997352	0,183508866	False	0,183508866
ENSMUSG00000020827	Mink1	3385,858275	2857,114191	3098,157855	3845,019687	3331,482499	0,183469399	False	0,183469399
ENSMUSG00000030432	Rpl28	5078,377799	7417,932777	5766,900707	6825,61159	10525,96607	0,183428456	False	0,183428456
ENSMUSG00000002455	Prpf6	761,0603284	622,3226876	611,7241847	670,2121907	569,4841878	-0,183392891	False	0,183392891
ENSMUSG00000024042	Sik1	1456,582631	4436,930273	1653,96162	2827,913749	8234,978641	0,18333821	False	0,18333821
ENSMUSG00000034413	Neurl1b	251,5021752	151,7391738	285,5445027	197,5834934	88,98190434	0,183144732	False	0,183144732
ENSMUSG00000026565	Pou2f1	367,8321716	514,7607416	323,9831858	418,74229	832,8706247	-0,183128726	False	0,183128726
ENSMUSG00000021962	Dcp1a	525,1234343	560,8587185	466,7554372	462,5249959	781,8543328	-0,183125245	False	0,183125245
ENSMUSG00000039168	Dap	1273,895383	1227,358634	1099,346336	841,9751139	1446,252552	0,183072702	False	0,183072702
ENSMUSG00000034271	Jdp2	374,3859742	708,7563942	425,0220098	662,3537563	727,2787648	0,183011166	False	0,183011166
ENSMUSG00000040859	Bsdc1	1142,000105	1278,258483	1005,995248	1227,038399	2060,820905	-0,182939293	False	0,182939293
ENSMUSG00000037363	Letm2	248,2252739	201,6786488	221,8461137	281,7810048	151,8624501	0,182924345	False	0,182924345
ENSMUSG00000035596	Mboat7	1144,457781	1085,223205	986,2267825	1020,473838	1299,135803	0,182888006	False	0,182888006
ENSMUSG00000026087	Mrpl30	438,2855497	637,6886799	497,5063836	642,1463536	866,0905356	0,182843923	False	0,182843923
ENSMUSG00000031024	St5	2212,727607	1019,917738	1929,62189	1554,847377	2511,662553	0,182816786	False	0,182816786
ENSMUSG00000036944	Tmem71	408,7934379	360,1404442	242,7128273	305,356308	180,3366595	-0,182812413	False	0,182812413
ENSMUSG00000025789	St8sia2	502,1851252	339,9725793	569,9907574	301,9884075	419,9945885	0,182719229	False	0,182719229

ENSMUSG00000061755	Bod1l	1051,885319	926,7614098	1141,079763	1303,377476	1008,461583	-0,182707548	False	0,182707548
ENSMUSG00000033430	Terf2ip	411,2511139	509,958869	466,7554372	507,4303353	531,5185753	0,182647242	False	0,182647242
ENSMUSG00000028514	Usp24	1101,858064	1120,757062	970,8513093	1051,907576	1351,338521	-0,182616133	False	0,182616133
ENSMUSG00000022241	Tars	576,7346299	884,504931	654,5558601	736,4475663	1059,477874	0,182608666	False	0,182608666
ENSMUSG00000039844	Rapgef1	2206,99303	1732,51563	1591,361479	1351,650716	2504,544001	0,182465885	False	0,182465885
ENSMUSG00000016664	Pacsin2	2360,188166	2616,060187	2611,633952	2805,46108	2079,803711	-0,182454508	False	0,182454508
ENSMUSG00000040033	Stat2	996,9972224	1131,321182	1234,43085	1136,105087	1386,931282	0,182347178	False	0,182347178
ENSMUSG00000024338	Psmb8	992,0818704	736,6072553	1125,70429	721,853331	735,5837426	0,182296809	False	0,182296809
ENSMUSG00000028556	Dock7	796,2870174	482,108008	701,7805279	635,4105526	377,2832744	-0,182268618	False	0,182268618
ENSMUSG00000036199	Ndufa13	1024,031658	1202,388896	1556,21754	1161,925657	1271,848019	0,182257447	False	0,182257447
ENSMUSG00000002014	Ssr4	879,8480007	1383,89968	998,3075115	1120,388218	1169,815436	0,182229971	False	0,182229971
ENSMUSG00000014769	Psmb1	1509,013051	2409,579665	1712,168768	1758,044038	2720,473422	0,182219631	False	0,182219631
ENSMUSG00000028414	Fktn	533,3156876	289,0727299	470,0501814	426,6007244	128,1339423	-0,182174982	False	0,182174982
ENSMUSG00000050619	Zscan29	343,2554118	418,7232898	388,7798229	433,3365253	302,5384748	-0,18216384	False	0,18216384
ENSMUSG00000030061	Uba3	705,3530061	594,4718266	487,6221508	420,987557	621,686905	-0,182157269	False	0,182157269
ENSMUSG00000028032	Papss1	684,0531477	440,8119037	602,9382	358,1200818	376,096849	-0,182098284	False	0,182098284
ENSMUSG00000032744	Heyl	675,0416691	1877,532183	959,8688284	594,9957472	1229,136705	-0,182097203	False	0,182097203
ENSMUSG00000021905	Dph3	516,931181	1107,311819	586,4644787	679,1932586	1359,643498	0,182071501	False	0,182071501
ENSMUSG00000038462	Uqcrfs1	559,530898	1312,831966	634,7873945	917,1915574	1515,065225	0,182055679	False	0,182055679
ENSMUSG00000020902	Ntn1	1798,199592	1386,780804	1107,034072	1095,690282	1585,064323	-0,182011775	False	0,182011775
ENSMUSG00000059878	Zfp422	602,9498403	441,7722782	519,4713453	481,6097652	531,5185753	-0,181917877	False	0,181917877
ENSMUSG00000029034	Ints11	395,6858327	411,0402937	549,1240437	428,8459914	348,809065	-0,181917877	False	0,181917877
ENSMUSG00000070319	Eif3g	979,7934905	1631,676306	1111,427064	1317,971712	2457,086985	0,181863665	False	0,181863665
ENSMUSG00000022235	Cmbl	272,8020337	235,2917569	240,5163311	204,3192943	45,08416487	-0,181719543	False	0,181719543
ENSMUSG00000022023	Wbp4	638,9957547	778,863734	563,4012688	758,900236	1047,61362	-0,181643534	False	0,181643534
ENSMUSG00000021254	Gpatch2l	422,7202685	325,5669616	361,3236208	372,7143171	281,1828177	-0,18163305	False	0,18163305
ENSMUSG00000024002	Brd4	2297,927041	4288,072223	2606,142711	4130,168592	11727,81499	0,181583094	False	0,181583094
ENSMUSG00000026482	Rgl1	1512,289953	917,1576646	1085,06911	856,5693492	1333,54214	-0,181471361	False	0,181471361
ENSMUSG00000046865	Fbl	365,3744956	511,8796181	343,7516514	322,1958103	907,6154243	-0,181438199	False	0,181438199
ENSMUSG00000035401	Emsy	1227,199539	1391,582676	1522,171849	1900,618491	2040,651673	0,181356778	False	0,181356778
ENSMUSG00000032396	Dis3l	371,1090729	227,6087608	327,2779301	277,2904709	181,5230849	-0,181326955	False	0,181326955
ENSMUSG00000002395	Use1	775,8063842	1084,262831	879,696718	1174,274626	1314,559334	0,181309582	False	0,181309582
ENSMUSG00000032314	Etfa	544,7848421	588,7095795	520,5695934	702,7685618	480,5022835	-0,181143276	False	0,181143276
ENSMUSG00000038400	Pmepa1	1802,295718	3760,826612	2311,812224	1589,649015	1942,178365	-0,181127484	False	0,181127484
ENSMUSG00000037795	N4bp2	286,7288643	302,5179731	219,6496175	229,017231	325,0805572	0,181110128	False	0,181110128
ENSMUSG00000031826	Usp10	817,5868759	1149,568298	926,9213858	960,9742634	1290,830826	0,181074948	False	0,181074948
ENSMUSG00000028161	Ppp3ca	4289,46381	2974,279882	4863,042531	4512,986611	4250,962177	0,181061889	False	0,181061889
ENSMUSG00000032177	Pde4a	620,9727975	253,5388727	703,977024	564,6846431	170,8452563	0,180998273	False	0,180998273
ENSMUSG00000028581	Laptm5	4851,452384	3298,886469	3740,632986	2648,292392	4279,436386	-0,180995911	False	0,180995911
ENSMUSG00000001120	Pcbp3	589,0230098	384,1498072	667,7348372	442,3175932	390,3339537	0,180951318	False	0,180951318
ENSMUSG00000048416	Mlf1	248,2252739	218,9653901	217,4531213	156,0460544	212,370145	-0,180947162	False	0,180947162
ENSMUSG00000000276	Dgke	644,730332	605,0359463	521,6678415	730,8343989	600,331248	-0,180848682	False	0,180848682
ENSMUSG00000033788	Dysf	312,9440747	523,4041123	354,7341322	520,9019372	639,4832859	0,180833291	False	0,180833291
ENSMUSG00000054263	Lifr	1490,170869	970,9386376	1689,105558	1655,884391	618,1276288	0,180781725	False	0,180781725
ENSMUSG00000023942	Slc29a1	1458,221081	968,0575141	1652,863372	1264,085304	1175,747563	0,18075801	False	0,18075801
ENSMUSG00000028416	Bag1	2391,318728	2828,302955	2710,47628	2711,159867	3399,108746	0,180739948	False	0,180739948
ENSMUSG00000035202	Lars2	1212,453483	1723,87226	1069,693637	2161,069459	2325,393767	-0,180731734	False	0,180731734
ENSMUSG00000020079	Supv3l1	175,3142199	237,2125059	204,2741443	198,7061269	332,1991096	0,180693338	False	0,180693338
ENSMUSG00000031644	Nek1	448,1162536	247,7766256	395,3693115	334,5447786	174,4045325	-0,180672162	False	0,180672162
ENSMUSG00000046688	Tifa	160,568164	430,247784	141,6740033	285,1489053	909,9882751	-0,180610824	False	0,180610824
ENSMUSG00000021792	Fam213a	648,8264586	558,9379694	551,3205399	735,3249328	498,2986643	0,180549251	False	0,180549251
ENSMUSG00000031353	Rbbp7	1145,277006	1197,587024	866,517741	962,0968969	1297,949378	0,180537534	False	0,180537534
ENSMUSG00000062515	Fabp4	79,46485667	808,6353441	90,05634317	400,7801542	461,5194772	0,180510927	False	0,180510927
ENSMUSG00000052151	Plpp2	430,9125218	597,3529502	493,1133913	488,3455661	518,467896	0,180507376	False	0,180507376
ENSMUSG00000043059	Zfp513	513,6542797	768,2996143	582,0714863	687,051693	977,6145224	0,180398681	False	0,180398681
ENSMUSG00000023072	Cep89	326,05168	211,2823939	287,7409989	200,9513939	161,3538532	-0,180329854	False	0,180329854
ENSMUSG00000029405	G3bp2	1582,743331	1318,594213	1396,971567	1294,396408	1268,288743	-0,180124659	False	0,180124659
ENSMUSG00000032232	Cgnl1	2549,429216	1396,384549	2250,310331	1514,432572	1669,300525	-0,180050311	False	0,180050311
ENSMUSG00000025190	Got1	364,5552703	720,2808884	321,7866896	381,695385	1197,10322	-0,180032907	False	0,180032907
ENSMUSG00000020706	Ftsj3	364,5552703	465,7816412	321,7866896	363,7332492	673,8896222	-0,180032907	False	0,180032907
ENSMUSG00000068876	Cgn	1336,156508	1782,455105	1179,518446	1485,244101	1684,724056	-0,179891024	False	0,179891024
ENSMUSG00000031660	Brd7	1061,716023	1493,382375	1469,455941	2146,475224	937,2760591	-0,179852003	False	0,179852003
ENSMUSG00000021901	Bap1	837,2482837	1023,759236	739,1209628	898,1067882	1633,707764	-0,179845022	False	0,179845022
ENSMUSG00000019189	Rnf145	1746,588396	993,0272515	1541,940315	1095,690282	1348,96567	-0,179792738	False	0,179792738
ENSMUSG00000025409	Mbd6	2359,36894	2083,052329	3159,659748	2732,489903	2478,442642	-0,179701952	False	0,179701952
ENSMUSG00000022840	Adcy5	440,7432257	259,3011198	288,839247	319,9505433	389,1475283	-0,179621201	False	0,179621201
ENSMUSG00000026365	Cfh	2646,91703	3208,611264	2337,07193	5188,811969	3438,260784	-0,179610835	False	0,179610835
ENSMUSG00000060657	Marf1	1572,912627	888,3464291	1781,358398	1340,424381	621,686905	0,179539273	False	0,179539273
ENSMUSG00000031432	Prps1	376,8436502	487,8702551	332,7691705	424,3554574	447,2823725	-0,179444303	False	0,179444303
ENSMUSG00000024953	Prdx5	1835,883957	3413,171037	2078,983629	2662,886627	4686,380295	0,179403527	False	0,179403527
ENSMUSG00000003662	Ciao1	580,0155312	596,3925756	563,4012688	656,7405889	245,590056	0,179242039	False	0,179242039
ENSMUSG00000002280	Narfl	326,8709053	465,7816412	370,1096055	397,4122538	657,2796668	0,17923161	False	0,17923161
ENSMUSG00000036898	Zfp157	225,2869648	198,7975252	218,5513694	165,0271223	255,0814591	0,179194198	False	0,179194198
ENSMUSG00000026848	Tor1b	739,7604699	779,8241086	714,9595049	653,3726884	815,0742438	-0,17915206	False	0,17915206
ENSMUSG00000011158	Brf1	548,0617435	594,4718266	779,7561421	938,5215937	484,0615596	-0,179147902	False	0,179147902
ENSMUSG00000039449	Prpf18	662,7532892	828,803209	585,3662306	937,3989602	1287,27155	-0,179132405	False	0,179132405
ENSMUSG00000032652	Crebl2	765,156455	446,5741508	623,8049137	675,8253581	198,1330403	-0,179104288	False	0,179104288
ENSMUSG00000055917	Zfp277	344,0746371	246,8162511	284,4462546	389,5538194	195,7601896	0,179101109	False	0,179101109
ENSMUSG00000059791	Nrm	346,5323131	157,5014209	259,1865486	185,2345251	306,0977509	-0,178997441	False	0,178997441
ENSMUSG00000035242	Oaz1	3048,33744	4163,223535	3596,762486	3450,975334	4562,992055	0,178981549	False	0,178981549
ENSMUSG00000035640	Cbarp	606,2267416	630,0056838	616,117177	535,4961725	767,6172281	-0,17898123	False	0,17898123
ENSMUSG00000039452	Snx22	294,9211176	74,9092124	333,8674186	147,0649866	106,7782852	0,178946182	False	0,178946182
ENSMUSG00000031792	Usb1	479,246816	590,6303285	542,5345552	551,2130413	715,4145109	0,178946182	False	0,178946182
ENSMUSG00000074102	Rbm15b	753,6873004	1058,332719	958,7705803	853,2014488	1556,590113	0,178920332	False	0,178920332
ENSMUSG00000047454	Gphn	425,1779445	467,7023902	375,6008459	497,326634	411,6896108	-0,178866449	False	0,178866449
ENSMUSG00000016356	Col20a1	141,7259815	266,0237415	125,200282	150,432887	813,8878184	-0,178866449	False	0,178866449
ENSMUSG00000010047	Hyal2	1537,685938	2803,333218	1941,702619	2805,46108	1740,486049	0,178729377	False	0,178729377
ENSMUSG00000020677	Ddx52	547,2425181	816,3183402	619,4119213	827,3808786	1122,35842	0,178718825	False	0,178718825
ENSMUSG00000030917	Tmem159	784,8178628	693,3904019	946,6898514	980,0590326	812,701393	-0,178690012	False	0,178690012
ENSMUSG00000026922	Agpat2	562,8077993	1157,251294	636,9838907	782,4755392	905,2425735	0,178614566	False	0,178614566
ENSMUSG00000090862	Rps13	2577,282877	4072,948331	2916,94692	3374,636257	5903,652747	0,178608241	False	0,178608241
ENSMUSG00000026339	Ccdc93	324,4132293	228,5691353	286,6427508	263,818869	137,6253454	-0,178578859	False	0,178578859
ENSMUSG00000049401	Ogfr	752,0488498	1467,452263	851,1422677	1044,049141	2387,087887	0,178573921	False	0,178573921
ENSMUSG00000022800	Fyttd1	653,7418106	725,082761	577,678494	650,004788	1027,444389	-0,178454182	False	0,178454182
ENSMUSG00000058396	Gpr182	2272,531056	2008,143117	1848,351531	1799,581477	1114,053442	-0,178437919	False	0,178437919
ENSMUSG00000031007	Atp6ap2	909,3401125	1120,757062	1029,058458	1035,068073	1305,06793	0,178433041	False	0,178433041
ENSMUSG00000029406	Pitpnm2	3521,030453	3054,951342	3984,444061	2723,508835	3075,214614	0,178380738	False	0,178380738
ENSMUSG00000074578	Zfas1	215,4562609	375,5064365	243,8110754	447,9307606	409,31676	0,178368642	False	0,178368642
ENSMUSG00000040549	Ckap5	832,3329318	543,5719772	674,3243257	534,373539	735,5837426	-0,178271125	False	0,178271125
ENSMUSG00000007880	Arid1a	3032,772159	2680,40528	3214,572152	3141,128492	4217,742266	0,178185964	False	0,178185964
ENSMUSG00000024778	Fas	516,931181	1522,193611	456,8712044	1567,196345	1940,99194	-0,178184712	False	0,178184712
ENSMUSG00000060802	B2m	20808,32329	12288,95233	13132,85063	11913,38655	18390,77999	-0,178178051	False	0,178178051
ENSMUSG00000046897	Zfp740	1780,176635	2571,882959	2014,186992	2689,829831	2747,761206	0,178177229	False	0,178177229
ENSMUSG00000017774	Myo1c	7507,380892	6635,227544	5582,395028	5477,328775	5887,042791	-0,17816374	False	0,17816374
ENSMUSG00000060600	Eno3	283,451963	421,6044134	320,6884415	556,8262087	424,7402901	0,178068096	False	0,178068096
ENSMUSG00000030213	Atf7ip	1912,071912	1007,432869	1690,203807	1311,235911	1065,410001	-0,177939565	False	0,177939565
ENSMUSG00000032609	Klhdc8b	865,1019448	443,6930273	978,5390459	607,3447155	425,9267155	0,177759267	False	0,177759267
ENSMUSG00000020003	Pex7	349,8092144	395,6743014	358,0288765	318,8279098	255,0814591	0,177745075	False	0,177745075
ENSMUSG00000067336	Bmpr2	8662,488603	5748,801864	7416,469334	7658,605636	3408,600149	-0,177699797	False	0,177699797
ENSMUSG00000034488	Edil3	267,0674565	209,3616449	236,1233388	118,9991494	217,1158466	-0,17766354	False	0,17766354

ENSMUSG00000064246	Chil1	16872,76483	12482,94798	13072,44698	16653,14512	19083,65242	0,177640917	False	0,177640917
ENSMUSG00000024339	Tap2	1456,582631	1287,862229	1506,796376	1346,037549	1688,283332	-0,17760928	False	0,17760928
ENSMUSG00000005142	Man2b1	1847,353111	1524,11436	1610,031696	1642,412789	2089,295114	0,177556632	False	0,177556632
ENSMUSG00000003269	Cyth2	995,3587717	1944,758399	1125,70429	1525,658906	2597,085181	0,177539361	False	0,177539361
ENSMUSG00000048000	Gigyf2	848,7174383	721,241263	730,3349781	959,8516299	581,3484417	0,177527097	False	0,177527097
ENSMUSG00000032601	Prkar2a	1966,140784	2149,318171	1738,526722	2112,796219	2041,838098	-0,177501386	False	0,177501386
ENSMUSG00000051185	Fam174a	389,1320301	410,0799192	415,137777	440,0723262	256,2678845	0,177480915	False	0,177480915
ENSMUSG00000031887	Tradd	233,4792181	206,4805214	354,7341322	250,3472672	263,3864369	-0,177288452	False	0,177288452
ENSMUSG00000026979	Psd4	625,8881495	435,0496566	553,5170361	413,1291226	384,4018268	-0,17727714	False	0,17727714
ENSMUSG00000026637	Traf5	298,1980189	143,0958032	337,1621628	174,0081902	187,4552118	0,17717197	False	0,17717197
ENSMUSG00000034247	Plekhm1	1561,443472	1381,018557	1804,421608	1597,50745	1659,809122	-0,177147636	False	0,177147636
ENSMUSG00000042502	Cd2bp2	917,5323657	936,365155	811,6053366	812,7866433	1282,525848	-0,176980698	False	0,176980698
ENSMUSG00000020477	Mrps24	439,9240004	691,4696529	562,3030208	497,326634	804,3964153	0,176939385	False	0,176939385
ENSMUSG00000004980	Hnrnpa2b1	5038,235758	3414,131411	5695,514581	4404,091163	2512,848979	0,17690756	False	0,17690756
ENSMUSG00000029126	Nsg1	596,3960377	691,4696529	576,5802459	527,6377381	689,3131523	-0,176722919	False	0,176722919
ENSMUSG00000006732	Mettl1	233,4792181	327,4877106	252,5970601	206,5645613	541,0099784	-0,176701378	False	0,176701378
ENSMUSG00000028149	Rap1gds1	1180,503696	609,8378189	1044,433931	734,2022994	582,5348671	-0,176681325	False	0,176681325
ENSMUSG00000057672	Pkn1	5289,737933	4008,603238	5978,862588	5384,150195	4074,184794	0,176674804	False	0,176674804
ENSMUSG00000053754	Chd8	1885,037476	2974,279882	2130,60129	2933,441297	4831,124193	0,176667433	False	0,176667433
ENSMUSG00000060002	Chpt1	1739,215368	1549,084097	1634,193154	1965,731233	1331,169289	0,176629487	False	0,176629487
ENSMUSG00000045095	Magi1	621,7920228	474,4250119	512,8818568	702,7685618	631,1783081	0,176617546	False	0,176617546
ENSMUSG00000029422	Rsrc2	929,0015203	749,092124	1049,925172	966,5874308	877,9547895	0,176533648	False	0,176533648
ENSMUSG00000023010	Tmbim6	16611,43195	16310,04044	15277,72914	14698,64022	22181,40911	-0,176493746	False	0,176493746
ENSMUSG00000029432	Nipsnap2	1133,807852	1163,013541	891,777447	1032,822806	1281,339423	0,176476526	False	0,176476526
ENSMUSG00000000804	Usp32	785,6370881	780,7844831	695,1910393	947,5026615	740,3294441	-0,176453551	False	0,176453551
ENSMUSG00000038332	Sesn1	539,8694902	1263,852866	477,737918	1531,272074	2802,336774	-0,1763913	False	0,1763913
ENSMUSG00000025404	R3hdm2	2954,945753	2176,208658	2614,928696	2367,63402	2256,581094	-0,176360038	False	0,176360038
ENSMUSG00000021770	Samd8	401,42041	723,162012	453,5764601	669,0895572	974,0552462	0,176231797	False	0,176231797
ENSMUSG00000029227	Fip1l1	969,1435613	1080,421333	857,7317563	881,2672859	1819,97655	-0,176183856	False	0,176183856
ENSMUSG00000018920	Cxcl16	1354,99869	2969,478009	1530,957834	2071,25878	3835,71329	0,176143091	False	0,176143091
ENSMUSG00000114797	Nupl1	384,2166781	434,0892821	464,558941	495,081367	481,6887088	0,176071657	False	0,176071657
ENSMUSG00000000876	Pxmp4	828,2368051	520,5229887	935,7073705	848,7109148	261,0135861	0,176014103	False	0,176014103
ENSMUSG00000029752	Asns	342,4361865	406,2384211	336,0639148	303,111041	592,0262702	-0,175988761	False	0,175988761
ENSMUSG00000025234	Arih1	1418,07904	1526,995484	1255,297564	1315,726445	2658,779302	-0,175908557	False	0,175908557
ENSMUSG00000036526	Card11	301,4749202	91,2355792	266,8742852	143,6970861	93,72760591	-0,175877688	False	0,175877688
ENSMUSG00000078622	Ccdc47	1382,033126	873,9408113	1223,448369	1127,124019	419,9945885	-0,175838976	False	0,175838976
ENSMUSG00000028224	Nbn	276,078935	218,9653901	192,1934153	166,1497558	244,4036306	-0,175815097	False	0,175815097
ENSMUSG00000041297	Cdk13	1138,723204	1559,648217	1008,191744	1377,471286	2379,969335	-0,17564706	False	0,17564706
ENSMUSG00000031584	Gsr	965,0474347	2579,565955	854,437012	1442,584029	4221,301542	-0,175625714	False	0,175625714
ENSMUSG00000036427	Gpi1	5590,393628	9584,537689	6313,828255	8676,834207	11307,8204	0,175565149	False	0,175565149
ENSMUSG00000000958	Slc7a7	484,9813933	161,342919	429,4150022	215,5456292	149,4895993	-0,175556804	False	0,175556804
ENSMUSG00000044477	Zfand3	2396,23408	2263,602739	2242,622595	2121,777287	3407,413724	-0,175495616	False	0,175495616
ENSMUSG00000056215	Lrguk	333,4247079	174,7881623	231,7303464	295,2526066	118,6425391	-0,17541122	False	0,17541122
ENSMUSG00000026887	Mrrf	371,1090729	287,1519809	295,4287355	252,5925342	328,6398334	-0,175335917	False	0,175335917
ENSMUSG00000024429	Gnl1	819,2253265	956,5330199	968,6548131	925,0499918	1328,796438	0,175271017	False	0,175271017
ENSMUSG00000052142	Rasal3	368,6513969	238,1728804	416,2360251	233,5077649	99,65973287	0,175144612	False	0,175144612
ENSMUSG00000020282	Rhbdf1	1053,52377	1354,12807	1189,402679	1414,518191	2339,630872	0,175014365	False	0,175014365
ENSMUSG00000021277	Traf3	769,2525816	761,5769927	594,1522153	586,0146793	868,4633864	0,175007647	False	0,175007647
ENSMUSG00000040725	Hnrnpul1	3607,868338	3349,786319	3144,284274	3197,260166	4072,998368	0,174944545	False	0,174944545
ENSMUSG00000020993	Trappc6b	669,3070918	1017,996989	755,5946841	1018,228571	761,6851012	0,174944251	False	0,174944251
ENSMUSG00000022428	Cby1	404,6973113	282,3501083	456,8712044	278,4131043	193,3873388	0,174944251	False	0,174944251
ENSMUSG00000022403	St13	1748,226847	892,1879272	1973,551813	1042,926508	1029,81724	0,174901997	False	0,174901997
ENSMUSG00000008429	Herpud2	1074,823628	1232,160507	952,1810918	1139,472988	1522,183777	-0,174792057	False	0,174792057
ENSMUSG00000042015	Wdr41	514,4735051	400,476174	455,7729563	365,9785162	244,4036306	-0,174781458	False	0,174781458
ENSMUSG00000033276	Stk36	440,7432257	387,0309307	497,5063836	452,4212946	294,233497	0,174776642	False	0,174776642
ENSMUSG00000028467	Gba2	447,2970283	544,5323517	524,9625858	634,2879192	396,2660807	-0,174763692	False	0,174763692
ENSMUSG00000022231	Sema5a	445,6585776	699,1526491	712,7630087	1164,170924	503,0443659	0,17474677	False	0,17474677
ENSMUSG00000055670	Zzef1	1222,284187	1194,7059	1082,872614	1293,273775	1499,641695	-0,174716219	False	0,174716219
ENSMUSG00000001150	Mcm3ap	640,6342054	567,5813401	621,6084175	588,2599463	972,8688208	-0,17467367	False	0,17467367
ENSMUSG00000015755	Tab2	2138,178102	2605,496067	1894,477951	2296,908111	3736,053557	-0,17458168	False	0,17458168
ENSMUSG00000038658	Ric1	774,9871589	686,6677803	753,398188	780,2302722	907,6154243	-0,174560135	False	0,174560135
ENSMUSG00000049493	Pls1	318,678652	315,9632164	377,7973421	303,111041	282,3692431	-0,174519425	False	0,174519425
ENSMUSG00000025487	Psmd13	865,1019448	1492,422001	976,3425497	1177,642526	1859,128588	0,174517254	False	0,174517254
ENSMUSG00000032437	Stt3b	2198,800776	4904,632663	1948,292107	5930,872703	4625,872601	-0,174506897	False	0,174506897
ENSMUSG00000062859	Tcp11	336,7016092	497,4740003	379,9938382	626,4294848	433,0452678	0,174505409	False	0,174505409
ENSMUSG00000071172	Srsf3	2109,505216	2477,766256	2518,282864	1869,184753	3822,662611	-0,17449548	False	0,17449548
ENSMUSG00000049858	Suox	305,5710468	1299,386723	344,8498994	612,9578829	418,8081631	0,174460693	False	0,174460693
ENSMUSG00000047694	Yipf6	1440,198124	1006,472495	1276,164278	994,6532679	666,7710699	-0,174453237	False	0,174453237
ENSMUSG00000037826	Ppm1k	278,536611	214,1635175	236,1233388	314,3373759	106,7782852	0,174446855	False	0,174446855
ENSMUSG00000033488	Cryzl2	290,0057656	130,6109344	327,2779301	191,970326	130,506793	0,174434735	False	0,174434735
ENSMUSG00000024187	Fam234a	1635,992977	1527,955858	1886,790214	1866,939486	1449,811828	-0,174300891	False	0,174300891
ENSMUSG00000023079	Gtf2ird1	389,1320301	485,9495061	344,8498994	459,1570955	641,8561367	-0,17429119	False	0,17429119
ENSMUSG00000038866	Zcchc2	533,3156876	501,3154984	572,1872535	472,6286973	599,1448226	-0,174282536	False	0,174282536
ENSMUSG00000056602	Fry	823,3214532	1082,342082	832,4720503	1728,855567	729,6516156	-0,174248029	False	0,174248029
ENSMUSG00000017491	Rarb	195,794853	257,3803708	173,5231978	188,6024255	270,5049892	-0,174214295	False	0,174214295
ENSMUSG00000057335	Cep170	490,7159706	278,5086102	434,9062426	381,695385	228,9801005	-0,174183808	False	0,174183808
ENSMUSG00000024758	Rtn3	4742,495415	3142,345423	5350,664682	3821,444384	2203,191952	0,174071741	False	0,174071741
ENSMUSG00000015120	Ube2i	1984,982966	2953,151643	1759,393436	2647,169758	4731,46446	-0,174048492	False	0,174048492
ENSMUSG00000040297	Suco	597,215263	717,3997649	529,3555781	636,5331861	1066,596427	-0,174013903	False	0,174013903
ENSMUSG00000025809	Itgb1	9238,404007	6672,682151	8062,23921	6180,097336	8188,70805	-0,174007786	False	0,174007786
ENSMUSG00000025580	Eif4a3	946,2052581	1524,11436	1067,497141	1200,095196	2454,714135	0,174007132	False	0,174007132
ENSMUSG00000028476	Reck	1060,077573	578,1454598	758,8894284	939,6442271	486,4344104	-0,173983315	False	0,173983315
ENSMUSG00000038506	Dcun1d2	382,5782275	214,1635175	431,6114984	319,9505433	85,42262817	0,173978527	False	0,173978527
ENSMUSG00000041712	Ubr7	524,304209	618,4811895	494,2116393	464,7702629	901,6832974	-0,173886363	False	0,173886363
ENSMUSG00000024097	Srsf7	1323,868128	1493,382375	1881,298974	1516,677839	1859,128588	0,173824189	False	0,173824189
ENSMUSG00000024140	Epas1	44725,6067	39460,82857	47928,64478	50451,14883	32638,56251	0,173786069	False	0,173786069
ENSMUSG00000029322	Plac8	701,2568795	318,84434	412,9412809	260,4509686	621,686905	-0,173754828	False	0,173754828
ENSMUSG00000032290	Ptpn9	1092,846586	1006,472495	1015,879481	821,7677112	1232,695982	0,173726144	False	0,173726144
ENSMUSG00000037486	Asxl2	614,4189949	381,2686836	544,7310514	497,326634	230,1665259	-0,17367871	False	0,17367871
ENSMUSG00000002107	Celf2	2050,520992	1817,988962	2222,854129	2955,893967	2300,478834	-0,173647073	False	0,173647073
ENSMUSG00000029201	Ugdh	392,4089314	493,6325022	442,5939792	492,8361	603,8905241	0,173625947	False	0,173625947
ENSMUSG00000024376	Epb41l4a	768,4333563	699,1526491	850,0440197	866,6730506	568,2977624	0,173567699	False	0,173567699
ENSMUSG00000039599	Fam149b	621,7920228	385,1101817	551,3205399	433,3365253	316,7755795	-0,173540757	False	0,173540757
ENSMUSG00000025092	Hspa12a	403,878086	175,7485368	344,8498994	358,1200818	221,8615482	-0,173476448	False	0,173476448
ENSMUSG00000049580	Tsku	837,2482837	2475,845507	742,4157071	2015,127106	2041,838098	-0,17342828	False	0,17342828
ENSMUSG00000032525	Nktr	2089,024583	2867,678311	2355,742148	2561,849613	5137,221944	0,173352165	False	0,173352165
ENSMUSG00000022285	Ywhaz	6008,198545	7343,023564	6856,36281	5328,018521	11841,71183	0,173333391	False	0,173333391
ENSMUSG00000062580	Timm17a	541,5079408	958,4537689	610,6259366	748,7965347	1331,169289	0,173306376	False	0,173306376
ENSMUSG00000029003	Mad2l2	314,5825254	824,9617109	354,7341322	689,2969599	861,3448341	0,173299619	False	0,173299619
ENSMUSG00000074884	Serf2	2391,318728	1246,566124	2120,717057	1352,77335	1316,932184	-0,173254282	False	0,173254282
ENSMUSG00000042570	Mier2	343,2554118	387,0309307	448,0852197	462,5249959	525,5864483	0,173166402	False	0,173166402
ENSMUSG00000033417	Cacul1	750,4103991	691,4696529	665,538341	688,1743265	972,8688208	-0,17315804	False	0,17315804
ENSMUSG00000018567	Gabarap	5475,702083	4856,613937	5881,118508	5481,819308	6269,071767	-0,173093148	False	0,173093148
ENSMUSG00000027804	Ppid	646,3687826	573,3435872	865,4194929	651,1274214	580,1620163	-0,172957562	False	0,172957562
ENSMUSG00000047554	Tmem41b	491,5351959	436,0100311	374,5025978	401,9027877	275,2506908	-0,172933396	False	0,172933396
ENSMUSG00000021248	Tmed10	3838,070655	5123,598053	3404,569071	3865,22709	6789,912514	-0,172909068	False	0,172909068
ENSMUSG00000042303	Sgsm3	540,6887155	1300,347097	609,5276885	1413,395558	1200,662496	0,172893512	False	0,172893512
ENSMUSG00000024668	Sdhaf2	504,6428011	460,9797686	568,8925093	486,1002991	378,4696998	0,172893512	False	0,172893512
ENSMUSG00000035007	Rundc1	416,9856912	643,450927	470,0501814	572,5430775	891,0054688	0,172816905	False	0,172816905
ENSMUSG00000030094	Xpc	826,5983545	767,3392398	623,8049137	931,7857928	670,3303461	0,172811842	False	0,172811842

ENSMUSG00000022906	Parp9	617,6958962	559,898344	696,2892874	645,514254	546,9421054	0,172790081	False	0,172790081
ENSMUSG00000025816	Sec61a2	404,6973113	456,177896	472,2466776	564,6846431	478,1294327	0,172753279	False	0,172753279
ENSMUSG00000003200	Sh3gl1	708,6299075	782,7052321	710,5665126	628,6747517	1205,408198	-0,172718531	False	0,172718531
ENSMUSG00000029478	Ncor2	2792,739138	3513,049987	2477,647685	2557,359079	4274,690685	-0,172709767	False	0,172709767
ENSMUSG00000036893	Ehmt1	702,0761048	864,3370661	759,9876765	1013,738037	622,8733304	-0,172688625	False	0,172688625
ENSMUSG00000040455	Usp45	364,5552703	346,695201	363,5201169	410,8838556	257,4543099	0,172593087	False	0,172593087
ENSMUSG00000035311	Gnptab	829,0560305	728,9242591	701,7805279	709,5043627	735,5837426	-0,172580013	False	0,172580013
ENSMUSG00000008475	Arpc5	3080,287228	5493,342243	3471,562204	3869,717624	6145,683527	0,172520138	False	0,172520138
ENSMUSG00000021238	Aldh6a1	1485,255517	1771,890986	1317,897705	2689,829831	1518,624501	-0,172472755	False	0,172472755
ENSMUSG00000032115	Hyou1	1989,079093	1616,310314	2241,524346	1200,095196	2021,668867	0,172379525	False	0,172379525
ENSMUSG00000030538	Cib1	645,5495573	902,7520469	810,5070885	727,4664984	895,7511704	0,172352958	False	0,172352958
ENSMUSG00000068040	Tm9sf4	1336,975733	931,5632824	940,1003628	800,437675	1186,425391	-0,172351901	False	0,172351901
ENSMUSG00000026781	Acbd5	1000,274124	830,723958	944,4933552	1127,124019	741,5158695	0,172250843	False	0,172250843
ENSMUSG00000021477	Ctsl	1566,358824	5309,91071	1764,884676	4263,761977	5495,522412	0,17215917	False	0,17215917
ENSMUSG00000038068	Rnf144b	649,6456839	1157,251294	576,5802459	1092,322381	1757,096004	-0,172131679	False	0,172131679
ENSMUSG00000036023	Parp2	364,5552703	414,8817917	410,7447847	428,8459914	457,960201	0,172104699	False	0,172104699
ENSMUSG00000020716	Nf1	806,1177213	1553,88597	908,2511683	1634,554355	1754,723154	0,172100778	False	0,172100778
ENSMUSG00000017418	Arl5b	596,3960377	461,9401431	529,3555781	472,6286973	460,3330518	-0,172033539	False	0,172033539
ENSMUSG00000033565	Rbfox2	1089,569684	967,0971396	1212,465889	994,6532679	1284,898699	-0,172025755	False	0,172025755
ENSMUSG00000074221	Zfp568	251,5021752	304,4387222	283,3480066	297,4978736	374,9104236	0,172004175	False	0,172004175
ENSMUSG00000015749	Anp32e	1298,472143	1177,419159	1462,866452	1196,727295	1261,170191	0,171983007	False	0,171983007
ENSMUSG00000028899	Taf12	355,5437917	396,6346759	476,6396699	389,5538194	315,5891541	-0,17197964	False	0,17197964
ENSMUSG00000037824	Tspan14	1944,840925	1014,155491	2191,004934	1261,840037	930,1575067	0,171940576	False	0,171940576
ENSMUSG00000061143	Maml3	462,0430842	520,5229887	399,7623038	482,7323987	428,2995662	0,171934496	False	0,171934496
ENSMUSG00000022885	St6gal1	906,8824365	567,5813401	805,0158481	507,4303353	544,5692546	-0,171898354	False	0,171898354
ENSMUSG00000028945	Rheb	1133,807852	1498,184248	1277,262526	1284,292707	1759,468855	0,171878918	False	0,171878918
ENSMUSG00000026670	Uap1	639,81498	905,6331704	721,5489934	720,7306975	1329,982864	0,171805524	False	0,171805524
ENSMUSG00000047821	Trim16	1651,558258	3117,375685	1466,161197	1861,326319	2618,440839	-0,171784132	False	0,171784132
ENSMUSG00000027671	Actl6a	469,4161121	630,9660583	585,3662306	528,7603716	723,7194887	0,171746695	False	0,171746695
ENSMUSG00000049562	Ap5b1	246,5868233	169,9862897	197,6846557	218,9135296	132,8796438	-0,171734588	False	0,171734588
ENSMUSG00000048677	Tpcn2	361,278369	559,898344	502,997624	619,6936839	406,9439092	0,171719077	False	0,171719077
ENSMUSG00000056536	Pign	434,1894231	537,80973	385,4850787	450,1760276	596,7719718	-0,171649565	False	0,171649565
ENSMUSG00000028245	Nsmaf	837,2482837	838,4069542	727,0402339	943,0121276	677,4488984	0,171620811	False	0,171620811
ENSMUSG00000002871	Tpra1	1363,190943	862,4163171	1535,350826	1307,86801	659,6525175	0,171580692	False	0,171580692
ENSMUSG00000095677	Dynlt1f	312,9440747	352,4574481	307,5094645	286,2715387	284,7420939	0,171544232	False	0,171544232
ENSMUSG00000037600	Kdf1	344,8938625	463,8608922	467,8536852	388,4311859	584,9077179	0,171506579	False	0,171506579
ENSMUSG00000032123	Dpagt1	911,7977884	533,0078575	1026,861962	611,8352495	294,233497	0,171456442	False	0,171456442
ENSMUSG00000109324	Prmt1	548,0617435	863,3766916	617,2154251	676,9479916	759,3122504	0,171435684	False	0,171435684
ENSMUSG00000034101	Ctnnd1	1178,04602	872,9804368	1326,68369	1311,235911	793,7185868	0,171428544	False	0,171428544
ENSMUSG00000031812	Map1lc3b	3348,993135	7815,527827	2974,055821	5549,177318	13572,70648	-0,171295693	False	0,171295693
ENSMUSG00000009739	Pou6f1	679,957021	480,187259	621,6084175	765,6360369	317,9620049	0,171215177	False	0,171215177
ENSMUSG00000046207	Pik3r6	227,7446408	256,4199963	293,2322393	271,6773034	335,7583857	0,171091666	False	0,171091666
ENSMUSG00000015804	Med28	670,1263171	516,6814906	754,4964361	488,3455661	581,3484417	0,17108102	False	0,17108102
ENSMUSG00000034111	Tmed8	395,6858327	174,7881623	351,439388	218,9135296	75,93122504	-0,171079519	False	0,171079519
ENSMUSG00000017969	Ptgis	936,3745482	831,6843325	1264,083549	1479,630934	1340,660692	-0,171049666	False	0,171049666
ENSMUSG00000028080	Lrba	701,2568795	556,0568459	490,9168951	568,0525435	622,8733304	-0,171004217	False	0,171004217
ENSMUSG00000021904	Sema3g	3271,985954	1005,51212	3683,524085	2386,71879	269,3185638	0,170920121	False	0,170920121
ENSMUSG00000028271	Gtf2b	490,7159706	808,6353441	552,418788	665,7216568	704,7366824	0,170874162	False	0,170874162
ENSMUSG00000035900	Gramd4	527,5811103	468,6627648	461,2641967	401,9027877	360,6733189	-0,170842733	False	0,170842733
ENSMUSG00000030595	Nfkbib	612,7805442	2114,744688	689,6997989	1314,603811	4029,100629	0,170598054	False	0,170598054
ENSMUSG00000023277	Twf2	679,1377957	469,6231393	764,3806688	488,3455661	316,7755795	0,170586969	False	0,170586969
ENSMUSG00000059439	Bcas3	873,2941981	861,4559426	1000,504008	1031,700173	775,9222059	-0,170555741	False	0,170555741
ENSMUSG00000029427	Zcchc8	717,641386	807,6749696	932,4126262	1087,831847	949,140313	0,170511738	False	0,170511738
ENSMUSG00000021789	Sftpa1	37208,39511	41874,24973	53533,00477	56895,06503	51843,23032	0,170435177	False	0,170435177
ENSMUSG00000022971	Ifnar2	885,582578	762,5373672	756,6929322	854,3240823	996,5973287	0,170383867	False	0,170383867
ENSMUSG00000019804	Snx3	3042,602863	1959,164017	2674,234093	1826,52468	2703,863467	-0,170283734	False	0,170283734
ENSMUSG00000069601	Ank3	1376,298549	1005,51212	987,3250306	1053,030209	1223,204578	-0,170127744	False	0,170127744
ENSMUSG00000005583	Mef2c	683,2339223	220,8861391	607,3311923	310,9694762	250,3357576	-0,169896139	False	0,169896139
ENSMUSG00000026516	Nvl	585,7461085	442,7326528	658,9488525	553,4583082	351,1819158	0,169891023	False	0,169891023
ENSMUSG00000049932	H2afx	361,278369	430,247784	434,9062426	406,3933217	742,7022949	0,169765816	False	0,169765816
ENSMUSG00000020752	Recql5	427,6356205	298,6764751	481,0326623	359,2427153	250,3357576	0,169752828	False	0,169752828
ENSMUSG00000007564	Ppp2r1a	3529,222707	6349,035938	4007,507271	3969,632004	11144,0937	0,169654799	False	0,169654799
ENSMUSG00000028041	Adam15	3832,336078	4893,108169	4486,343437	3407,192628	7600,241056	-0,169640571	False	0,169640571
ENSMUSG00000021275	Tecpr2	324,4132293	313,0820928	331,6709224	364,8558827	202,8787419	0,169494062	False	0,169494062
ENSMUSG00000040731	Eif4h	4420,539862	7392,002665	3930,629905	4913,766765	7710,578618	-0,16946204	False	0,16946204
ENSMUSG00000039275	Foxk2	725,014414	615,600066	644,6716273	579,2788784	564,7384862	-0,169445187	False	0,169445187
ENSMUSG00000028423	Nfx1	869,1980715	1013,195116	977,4407978	1141,718254	1162,696883	0,16932435	False	0,16932435
ENSMUSG00000022237	Ankrd33b	1600,766288	1405,02792	1800,028615	1376,348653	1459,303231	0,169257152	False	0,169257152
ENSMUSG00000037857	Nufip2	431,7317471	498,4343748	433,8079945	383,940652	677,4488984	-0,169251862	False	0,169251862
ENSMUSG00000024472	Dcp2	501,3658998	315,9632164	445,8887235	324,4410772	312,0298779	-0,169180161	False	0,169180161
ENSMUSG00000031447	Lamp1	11456,04697	8028,73097	8144,607816	7492,455881	10189,02126	-0,169093837	False	0,169093837
ENSMUSG00000024911	Fibp	752,8680751	1035,28373	935,7073705	846,4656479	1229,136705	0,169054437	False	0,169054437
ENSMUSG00000021731	Mrps30	348,1707638	493,6325022	309,7059607	361,4879822	682,1946	-0,168895914	False	0,168895914
ENSMUSG00000017210	Med24	1054,342995	1409,829792	937,9038667	960,9742634	1662,181973	-0,168832314	False	0,168832314
ENSMUSG00000025373	Rnf41	537,4118142	377,4271855	604,0364481	462,5249959	236,0986529	0,168607568	False	0,168607568
ENSMUSG00000000439	Mkrn2	431,7317471	511,8796181	497,5063836	565,8072766	485,247985	0,168587038	False	0,168587038
ENSMUSG00000040681	Hmgn1	1672,038891	1359,890317	1459,571708	1460,546164	1487,777441	-0,168449678	False	0,168449678
ENSMUSG00000039347	Atp6v0e2	657,8379372	424,4855369	585,3662306	462,5249959	288,3013701	-0,16839269	False	0,16839269
ENSMUSG00000098557	Kctd12	1292,737565	1142,845676	1045,532179	1452,68773	722,5330633	0,168295187	False	0,168295187
ENSMUSG00000027822	Slc33a1	448,9354789	399,5157995	392,0745672	322,1958103	360,6733189	-0,168255558	False	0,168255558
ENSMUSG00000022051	Bnip3l	3007,376174	3324,816581	2676,430589	3674,379397	3524,869837	-0,168195099	False	0,168195099
ENSMUSG00000020709	Adap2	194,9756277	504,1966219	173,5231978	577,0336114	308,4706017	-0,168165251	False	0,168165251
ENSMUSG00000037174	Elf2	1087,112008	1565,410464	967,556565	1249,491069	2519,967531	-0,168082682	False	0,168082682
ENSMUSG00000067150	Xpo5	668,4876665	783,6656066	602,9382	594,9957472	1078,460681	-0,168022018	False	0,168022018
ENSMUSG00000049154	Fam183b	403,878086	368,7838149	538,1415628	488,3455661	359,4868936	-0,167980696	False	0,167980696
ENSMUSG00000028089	Chd1l	335,8823839	399,5157995	378,8955902	450,1760276	298,9791986	-0,167911019	False	0,167911019
ENSMUSG00000029156	Sgcb	413,7087899	403,3572975	405,2535443	464,7702629	277,6235416	0,167902154	False	0,167902154
ENSMUSG00000034435	Tmem30b	412,0703392	601,1944482	366,8148612	434,4591588	746,2615711	-0,167838533	False	0,167838533
ENSMUSG00000040250	Ints13	414,5280152	621,3623131	369,0113574	460,279729	917,1068274	-0,167804387	False	0,167804387
ENSMUSG00000037236	Matr3	1028,127785	915,2369156	1159,74998	1152,944589	1150,83263	-0,167802438	False	0,167802438
ENSMUSG00000031533	Mrps31	207,2640076	273,7067376	232,8285945	237,9982989	386,7746775	0,167798644	False	0,167798644
ENSMUSG00000038145	Snrk	2314,311547	2252,078245	2060,313412	2411,416726	2608,949435	-0,167719275	False	0,167719275
ENSMUSG00000027843	Ptpn22	212,1793596	997,8291241	188,898671	417,6196565	1017,952986	-0,167671768	False	0,167671768
ENSMUSG00000000168	Dlat	625,8881495	702,9941471	684,2085585	680,3158921	379,6561252	0,167607817	False	0,167607817
ENSMUSG00000031441	Atp11a	9253,969289	7813,607078	8239,057152	8239,007148	7533,801234	-0,167593061	False	0,167593061
ENSMUSG00000038175	Mylip	758,6026524	1539,480352	675,4225738	1699,667097	2037,092397	-0,167554018	False	0,167554018
ENSMUSG00000034024	Cct2	1590,935584	2948,34977	1786,849638	2207,097432	4248,589326	0,167542815	False	0,167542815
ENSMUSG00000036636	Clcn7	852,8135649	970,9386376	959,8688284	1271,943739	759,3122504	-0,167537102	False	0,167537102
ENSMUSG00000026655	Fam107b	1095,304262	861,4559426	583,1697344	482,7323987	975,2416716	-0,16750001	False	0,16750001
ENSMUSG00000025171	Ubtd1	308,0287228	573,3435872	345,9481475	351,3842809	853,0398563	0,167490931	False	0,167490931
ENSMUSG00000022150	Dab2	1499,182348	1334,92058	1588,066734	1545,866309	2783,353968	-0,167421958	False	0,167421958
ENSMUSG00000028268	Gbp3	466,9584361	299,6368496	427,218506	269,4320365	524,4000229	0,167373607	False	0,167373607
ENSMUSG00000028857	Tmem222	580,8307565	799,9919734	721,5489934	652,2500549	1206,594623	0,167307311	False	0,167307311
ENSMUSG00000035941	Ibtk	571,0000526	984,3838809	508,4888645	745,4286342	1015,580135	-0,167274698	False	0,167274698
ENSMUSG00000021262	Evl	688,1492743	440,8119037	612,8224328	455,789195	543,3828292	-0,167252441	False	0,167252441
ENSMUSG00000037902	Sirpa	1759,696001	1292,664101	1567,200021	1240,510001	1155,578331	-0,167136894	False	0,167136894
ENSMUSG00000040990	Sh3kbp1	711,0875834	372,625313	798,4263596	486,1002991	219,4886974	0,167132086	False	0,167132086
ENSMUSG00000028128	F3	615,2382202	5126,479177	690,798047	1403,291857	5437,387568	0,167118874	False	0,167118874
ENSMUSG00000023011	Faim2	680,7762464	56,66209656	764,3806688	108,8954481	37,96561252	0,167110594	False	0,167110594
ENSMUSG00000023861	Mpc1	453,8508309	994,9480006	509,5871126	753,2870686	926,5982306	0,167110594	False	0,167110594

ENSMUSG00000021048	Mthfd1	1193,611301	537,80973	1063,104149	666,8442902	281,1828177	-0,16705016	False	0,16705016
ENSMUSG00000035919	Bbs9	361,278369	507,0777455	321,7866896	475,9965977	359,4868936	-0,167006227	False	0,167006227
ENSMUSG00000022425	Enpp2	1327,964254	1441,522151	1182,81319	1783,864608	1325,237162	-0,166994076	False	0,166994076
ENSMUSG00000054733	Msra	363,736045	672,2621626	323,9831858	511,9208693	635,9240097	-0,166972957	False	0,166972957
ENSMUSG00000056763	Cspp1	615,2382202	766,3788653	548,0257956	819,5224442	688,1267269	-0,166901328	False	0,166901328
ENSMUSG00000071647	Eml3	742,2181458	858,574819	661,1453486	1014,860671	1325,237162	-0,166875799	False	0,166875799
ENSMUSG00000093803	Ppp2r3d	1010,104828	1482,818256	1507,894624	1133,85982	2255,394669	0,166737268	False	0,166737268
ENSMUSG00000045969	Ing1	572,6385032	600,2340737	556,8117803	699,4006613	510,1629182	-0,166666639	False	0,166666639
ENSMUSG00000018848	Rars	489,0775199	711,6375178	566,6960131	548,9677743	1203,035347	0,166658308	False	0,166658308
ENSMUSG00000034854	Mfsd12	347,3515385	193,0352781	389,878071	252,5925342	118,6425391	0,166626524	False	0,166626524
ENSMUSG00000027108	Ola1	346,5323131	388,9516798	332,7691705	296,3752401	344,0633635	0,166601052	False	0,166601052
ENSMUSG00000035232	Pdk3	389,1320301	346,695201	439,299235	419,8649235	386,7746775	-0,166591869	False	0,166591869
ENSMUSG00000025068	Gsto1	2138,178102	4582,9072	2353,545651	1905,109024	2533,01821	-0,166508467	False	0,166508467
ENSMUSG00000001891	Ugp2	1044,512291	888,3464291	906,0546721	930,6631593	609,8226511	-0,166498466	False	0,166498466
ENSMUSG00000041112	Elmo1	895,4132819	692,4300274	1004,897	950,870562	587,2805687	0,166422012	False	0,166422012
ENSMUSG00000033991	Ttc37	273,6212591	198,7975252	243,8110754	186,3571586	168,4724056	-0,166416662	False	0,166416662
ENSMUSG00000023883	Phf10	538,2310395	791,3486028	604,0364481	733,0796659	863,7176848	0,166410013	False	0,166410013
ENSMUSG00000027573	Gid8	621,7920228	510,9192435	509,5871126	395,1669868	554,0606577	-0,166388179	False	0,166388179
ENSMUSG00000036371	Serbp1	3188,424971	4505,116864	3578,092269	4134,659126	5750,603871	0,166346658	False	0,166346658
ENSMUSG00000022570	Tsta3	417,8049165	586,7888305	372,3061016	435,5817923	780,6679074	-0,166340209	False	0,166340209
ENSMUSG00000031657	Heatr3	296,5595682	329,4084597	332,7691705	280,6583713	242,0307798	0,166199861	False	0,166199861
ENSMUSG00000039849	Pcif1	1441,836575	2101,299445	1284,950262	1744,572436	2904,369358	-0,166195134	False	0,166195134
ENSMUSG00000028788	Ptp4a2	5149,650403	7142,30529	4589,578757	6752,640414	8424,806703	-0,166112749	False	0,166112749
ENSMUSG00000046062	Ppp1r15b	1316,4951	2061,92409	1477,143678	1703,034997	3069,282487	0,166108008	False	0,166108008
ENSMUSG00000018593	Sparc	29449,51204	23791,35793	25556,23299	24782,13419	26247,28893	-0,166075314	False	0,166075314
ENSMUSG00000035933	Cog5	745,4950471	199,7578997	664,4400929	336,7900456	98,47330747	-0,166059638	False	0,166059638
ENSMUSG00000021111	Papola	2576,463652	1880,413306	2296,436751	2040,947676	684,5674508	-0,165995191	False	0,165995191
ENSMUSG00000024213	Nudt3	841,3444104	806,7145951	1141,079763	749,9191681	885,0733419	-0,165961399	False	0,165961399
ENSMUSG00000028955	Vamp3	1668,76199	1544,282225	1649,568627	1423,499259	1872,179267	0,165940382	False	0,165940382
ENSMUSG00000022770	Dlg1	1638,450653	1145,7268	1428,820762	1259,59477	1460,489657	-0,165880082	False	0,165880082
ENSMUSG00000019857	Asf1a	195,794853	397,5950504	219,6496175	321,0731768	525,5864483	0,165861146	False	0,165861146
ENSMUSG00000031667	Aktip	727,47209	537,80973	815,998329	672,4574577	380,8425506	0,165674299	False	0,165674299
ENSMUSG00000019027	Dnah1	492,3544212	370,7045639	416,2360251	438,9496927	399,8253569	-0,165641609	False	0,165641609
ENSMUSG00000039473	Ubn1	1501,640024	1745,960874	1338,764419	1673,846526	2472,510515	-0,165636895	False	0,165636895
ENSMUSG00000052920	Prkg1	276,078935	197,8371507	206,4706404	271,6773034	309,6570271	0,165590372	False	0,165590372
ENSMUSG00000057193	Slc44a2	9273,630696	7018,416977	8217,09219	8268,195619	5551,284406	-0,165561753	False	0,165561753
ENSMUSG00000028106	Rprd2	684,872373	858,574819	610,6259366	794,8245076	1214,899601	-0,165546295	False	0,165546295
ENSMUSG00000048376	F2r	3969,965932	4135,372674	2853,248531	2951,403433	4452,654493	0,165539042	False	0,165539042
ENSMUSG00000034187	Nsf	834,7906077	1403,107171	767,6754131	744,3060007	1137,78195	-0,165518502	False	0,165518502
ENSMUSG00000031389	Arhgap4	832,3329318	448,4948999	933,5108743	428,8459914	270,5049892	0,165506109	False	0,165506109
ENSMUSG00000020009	Ifngr1	2113,601342	2726,503256	1884,593718	2216,0785	4152,488869	-0,165449748	False	0,165449748
ENSMUSG00000032565	Nudt16	208,9024583	305,3990967	218,5513694	381,695385	186,2687864	-0,165443531	False	0,165443531
ENSMUSG00000032766	Gng11	885,582578	248,7370001	789,6403749	413,1291226	281,1828177	-0,165431083	False	0,165431083
ENSMUSG00000051397	Tacstd2	731,5682166	1205,27002	652,3593639	781,3529057	2226,920459	-0,16532548	False	0,16532548
ENSMUSG00000054027	Nt5dc3	345,7130878	228,5691353	387,6815749	304,2336745	252,7086083	0,165296949	False	0,165296949
ENSMUSG00000034118	Tpst1	555,4347714	323,6462125	495,3098874	309,8468419	406,9439092	-0,165286075	False	0,165286075
ENSMUSG00000026000	Lancl1	525,9426596	497,4740003	589,7592229	502,9398014	462,7059026	0,165220555	False	0,165220555
ENSMUSG00000039110	Mycbpap	389,1320301	152,6995484	347,0463956	304,2336745	104,4054344	-0,16513119	False	0,16513119
ENSMUSG00000027852	Nras	1655,654385	1856,403943	1466,161197	1604,24325	1678,791929	0,165109123	False	0,165109123
ENSMUSG00000052911	Lamb2	2777,993082	2477,766256	3070,701652	2903,130193	3469,107844	-0,165002912	False	0,165002912
ENSMUSG00000031930	Wwp2	1105,954191	1026,64036	808,3105923	892,4936208	1239,814534	0,164832692	False	0,164832692
ENSMUSG00000061118	Dnajc30	562,8077993	661,6980429	738,0227147	630,9200187	1090,324935	0,164814806	False	0,164814806
ENSMUSG00000036438	Calm2	3751,23277	9191,744511	4205,191927	5381,904928	11664,93445	0,16480686	False	0,16480686
ENSMUSG00000022711	Pmm2	895,4132819	1336,841329	1280,55727	1286,537974	1003,715881	0,164725324	False	0,164725324
ENSMUSG00000037523	Mavs	689,7877249	342,8537029	773,1666535	581,5241454	168,4724056	0,16462696	False	0,16462696
ENSMUSG00000027646	Src	1418,07904	1874,651059	1376,104854	1265,207938	2328,953043	-0,164563435	False	0,164563435
ENSMUSG00000017404	Rpl19	1958,767756	3100,088944	2195,397927	2721,263568	4452,654493	0,164536107	False	0,164536107
ENSMUSG00000062232	Rapgef2	572,6385032	510,9192435	526,0608339	600,6089146	760,4986758	-0,164529403	False	0,164529403
ENSMUSG00000023944	Hsp90ab1	25508,21899	15892,27752	28589,59421	13394,14011	23567,15397	0,164527971	False	0,164527971
ENSMUSG00000025722	Wdr73	351,4476651	386,0705562	294,3304874	313,2147424	393,8932299	0,164494751	False	0,164494751
ENSMUSG00000028645	Slc2a1	749,5911738	1343,563951	668,8330852	995,7759014	1870,992842	-0,164457751	False	0,164457751
ENSMUSG00000047407	Tgif1	507,1004771	806,7145951	452,478212	617,4484169	1231,509556	-0,164423307	False	0,164423307
ENSMUSG00000021578	Ccdc127	589,0230098	640,5698035	722,6472415	699,4006613	525,5864483	-0,164395915	False	0,164395915
ENSMUSG00000022536	Glyr1	2317,588449	4098,878443	2068,001149	4095,366954	5525,183047	-0,164387412	False	0,164387412
ENSMUSG00000022265	Ank	421,9010432	376,466811	369,0113574	300,865774	317,9620049	-0,164381971	False	0,164381971
ENSMUSG00000003531	Dgcr6	405,5165366	454,257147	584,2679825	568,0525435	361,8597443	-0,164330126	False	0,164330126
ENSMUSG00000005374	Tbl2	479,246816	357,2593207	537,0433147	358,1200818	366,6054459	0,164269605	False	0,164269605
ENSMUSG00000037784	Dzip1l	543,9656168	777,9033595	485,4256546	642,1463536	631,1783081	-0,164265107	False	0,164265107
ENSMUSG00000034893	Cog3	429,2740711	630,0056838	481,0326623	542,2319734	906,4289989	0,164235823	False	0,164235823
ENSMUSG00000037405	Icam1	9588,213222	23615,6094	10744,16104	14041,89963	36288,00702	0,164218937	False	0,164218937
ENSMUSG00000026879	Gsn	36969,18131	37905,02185	41759,78528	32991,95286	46140,08347	-0,164208918	False	0,164208918
ENSMUSG00000020250	Txnrd1	1119,881021	4037,414473	999,4057596	1973,589667	9779,7045	-0,164203028	False	0,164203028
ENSMUSG00000030681	Mvp	1302,568269	2215,584013	1459,571708	1765,902472	2990,978411	0,164186104	False	0,164186104
ENSMUSG00000015957	Wnt11	299,0172442	871,0596878	266,8742852	646,6368875	1276,593721	-0,164068386	False	0,164068386
ENSMUSG00000071064	Zfp827	313,7633001	138,2939306	280,0532623	221,1587966	201,6923165	-0,16397538	False	0,16397538
ENSMUSG00000004768	Rab23	316,220976	403,3572975	282,2497585	313,2147424	363,0461697	-0,163960723	False	0,163960723
ENSMUSG00000015668	Pdzd11	403,878086	287,1519809	452,478212	314,3373759	161,3538532	0,163928455	False	0,163928455
ENSMUSG00000047371	Zfp768	498,9082239	558,9379694	384,3868306	394,0443533	537,4507022	0,163913732	False	0,163913732
ENSMUSG00000024404	Riok3	1365,648619	3057,832465	1219,055377	1952,259631	4718,413781	-0,163822663	False	0,163822663
ENSMUSG00000025155	Dus1l	421,9010432	588,7095795	500,8011279	472,6286973	732,0244664	0,163802576	False	0,163802576
ENSMUSG00000026915	Strbp	701,2568795	507,0777455	626,0014098	552,3356747	335,7583857	-0,163777113	False	0,163777113
ENSMUSG00000040253	Gbp7	782,3601868	361,1008187	876,4019738	661,2311228	160,1674278	0,163759778	False	0,163759778
ENSMUSG00000026202	Tuba4a	1076,462079	1654,725294	960,9670765	985,6722	2638,61007	-0,163738589	False	0,163738589
ENSMUSG00000031994	Adamts8	467,7776615	829,7635835	697,3875355	417,6196565	1338,287841	-0,16363335	False	0,16363335
ENSMUSG00000028518	Prkaa2	367,8321716	298,6764751	362,4218688	412,0064891	265,7592876	0,163619393	False	0,163619393
ENSMUSG00000036180	Gatad2a	2007,10205	2571,882959	2248,113835	2562,972247	4281,809237	0,163601118	False	0,163601118
ENSMUSG00000008958	Vps72	1103,496515	1236,002005	1385,989086	1307,86801	1477,099612	0,16359901	False	0,16359901
ENSMUSG00000006315	Tmem147	690,6069503	892,1879272	878,5984699	773,4944713	788,9728852	0,163526126	False	0,163526126
ENSMUSG00000041977	Arhgef11	1408,248336	1041,045977	1239,922091	1257,349503	1184,05254	-0,163516038	False	0,163516038
ENSMUSG00000027185	Nat10	343,2554118	563,739842	389,878071	306,4789415	1423,71047	-0,163494518	False	0,163494518
ENSMUSG00000021518	Ptdss1	888,040254	613,679317	792,9351191	510,7982358	533,8914261	-0,16342225	False	0,16342225
ENSMUSG00000069520	Tmem19	448,9354789	564,7002165	400,8605519	546,7225073	409,31676	-0,163407665	False	0,163407665
ENSMUSG00000031820	Babam1	1199,345878	1072,738337	1343,157411	1220,302598	939,6489099	0,163380615	False	0,163380615
ENSMUSG00000040969	Arhgef38	468,5968868	142,1354287	418,4325213	266,064136	78,30407582	-0,163352385	False	0,163352385
ENSMUSG00000028617	Lrrc42	228,5638661	265,0633669	289,9374951	255,9604346	304,9113256	0,163323477	False	0,163323477
ENSMUSG00000030407	Qpctl	343,2554118	510,9192435	384,3868306	420,987557	551,6878069	0,163276443	False	0,163276443
ENSMUSG00000012017	Scarf2	3004,918498	2129,150306	2620,419937	2302,521278	2683,694235	0,163105553	False	0,163105553
ENSMUSG00000024927	Rela	2935,284345	4553,13559	2621,518185	2760,55574	7459,056435	-0,163097713	False	0,163097713
ENSMUSG00000029767	Calu	3390,773627	2779,323855	2293,142007	2034,211875	3796,561252	0,163078813	False	0,163078813
ENSMUSG00000028793	Rnf19b	603,7690657	1416,552414	539,2398109	623,0615843	6298,732402	-0,16306983	False	0,16306983
ENSMUSG00000049625	Tifab	611,1420936	218,9653901	545,8292994	198,7061269	67,6262473	-0,163058014	False	0,163058014
ENSMUSG00000021494	Ddx41	620,9727975	812,4768422	554,6152841	678,0706251	1315,745759	-0,163042696	False	0,163042696
ENSMUSG00000031683	Lsm6	298,1980189	306,3594712	333,8674186	217,7908961	296,6063478	0,163004638	False	0,163004638
ENSMUSG00000000149	Gna12	1678,592694	1018,957364	995,0127672	760,0228695	1879,29782	0,162941508	False	0,162941508
ENSMUSG00000021832	Psmc6	685,6915983	1208,151144	767,6754131	992,408001	1404,727663	0,162936597	False	0,162936597
ENSMUSG00000020810	Cygb	719,2798367	733,7261317	642,4751311	774,6171048	1360,829924	-0,16291255	False	0,16291255
ENSMUSG00000034522	Zfp395	738,9412445	408,1591701	660,0471005	502,9398014	467,4516042	-0,162890678	False	0,162890678
ENSMUSG00000002496	Tsc2	2712,455056	1664,32904	3036,655962	2250,880138	2296,919557	0,162884234	False	0,162884234
ENSMUSG00000026305	Lrrfip1	1659,750512	2019,667611	1482,634918	1581,790581	2673,016406	-0,162803002	False	0,162803002
ENSMUSG00000079487	Med12	982,2511665	1317,633839	877,5002218	1631,186454	1293,203676	-0,162692486	False	0,162692486

ENSMUSG00000019338	Zfp687	720,099062	799,0315989	590,857471	806,0508424	652,5339652	0,162675453	False	0,162675453
ENSMUSG00000026393	Nek7	2136,539652	4112,323686	1908,755176	3829,302818	3974,525061	-0,162644023	False	0,162644023
ENSMUSG00000045980	Tmem104	669,3070918	460,9797686	445,8887235	380,5727515	597,9583972	-0,162623188	False	0,162623188
ENSMUSG00000020190	Mknk2	4586,023378	7433,298769	5133,211561	9651,280073	10663,59142	0,162618012	False	0,162618012
ENSMUSG00000027519	Rab22a	1178,865245	1323,396086	1053,219916	1158,557757	2168,785615	-0,162592107	False	0,162592107
ENSMUSG00000035498	Cdcp1	557,8924474	664,5791664	515,078353	498,4492674	1061,850725	-0,162540347	False	0,162540347
ENSMUSG00000037353	Letmd1	483,3429427	566,6209656	589,7592229	652,2500549	431,8588424	-0,162487347	False	0,162487347
ENSMUSG00000052997	Uba2	915,8939151	893,1483017	826,9808098	772,3718378	1025,071538	0,162472186	False	0,162472186
ENSMUSG00000052397	Ezr	3965,05058	6081,091448	3542,94833	5100,123923	9465,301771	-0,162388844	False	0,162388844
ENSMUSG00000024065	Ehd3	355,5437917	374,546062	418,4325213	317,7052763	340,5040873	-0,162338215	False	0,162338215
ENSMUSG00000020775	Mrpl38	720,9182874	806,7145951	921,4301453	837,48458	825,7520723	0,162222611	False	0,162222611
ENSMUSG00000026447	Pik3c2b	302,2941455	201,6786488	338,2604109	208,8098283	205,2515927	0,162181299	False	0,162181299
ENSMUSG00000001424	Snd1	1608,958541	1218,715263	1393,676823	1099,058182	1437,947574	-0,162116074	False	0,162116074
ENSMUSG00000037351	Actr1b	1669,581215	1988,935627	1868,119997	2019,61764	2217,429056	0,162100854	False	0,162100854
ENSMUSG00000073411	H2-D1	21755,34777	22503,4957	19443,38414	19452,99303	34606,84224	-0,162090736	False	0,162090736
ENSMUSG00000020273	Papolg	314,5825254	198,7975252	281,1515104	190,8476925	131,6932184	-0,162090736	False	0,162090736
ENSMUSG00000030850	Ate1	678,3185704	596,3925756	606,2329443	599,4862811	440,1638202	-0,162090736	False	0,162090736
ENSMUSG00000021866	Anxa11	3163,028986	3821,330207	2826,890577	3325,240383	4973,49524	-0,162090736	False	0,162090736
ENSMUSG00000027082	Tfpi	550,5194194	291,9538535	492,0151432	443,4402267	228,9801005	-0,162090736	False	0,162090736
ENSMUSG00000020021	Fgd6	592,2999111	369,7441894	529,3555781	472,6286973	277,6235416	-0,162090736	False	0,162090736
ENSMUSG00000037103	Dcaf15	376,0244249	706,8356452	336,0639148	504,0624349	917,1068274	-0,162090736	False	0,162090736
ENSMUSG00000015027	Galns	255,5983019	327,4877106	228,4356022	277,2904709	301,3520494	-0,162090736	False	0,162090736
ENSMUSG00000035476	Tab3	238,39457	300,5972241	213,060129	248,1020002	383,2154014	-0,162090736	False	0,162090736
ENSMUSG00000027282	Mtch2	1203,442005	1400,226047	1346,452155	1491,979902	1522,183777	0,161996348	False	0,161996348
ENSMUSG00000029469	Ift81	379,3013262	216,0842665	311,9024568	339,0353125	81,863352	-0,161908869	False	0,161908869
ENSMUSG00000000142	Axin2	513,6542797	518,6022397	615,018929	483,8550321	459,1466264	-0,16184272	False	0,16184272
ENSMUSG00000027315	Spint1	691,4261756	1172,617286	776,4613978	773,4944713	1749,977452	0,161815759	False	0,161815759
ENSMUSG00000022206	Npr3	1944,0217	3020,377859	2058,116916	1737,836635	2315,902364	-0,161751855	False	0,161751855
ENSMUSG00000024474	Ik	1657,292836	2120,506936	1481,53667	1881,533721	3185,552176	-0,161734207	False	0,161734207
ENSMUSG00000028849	Map7d1	4253,417895	4096,957694	4034,963473	3802,359615	5291,457245	-0,161727622	False	0,161727622
ENSMUSG00000018796	Acsl1	1558,985796	2326,027082	1393,676823	1870,307386	1853,196461	-0,161711728	False	0,161711728
ENSMUSG00000002885	Adgre5	13079,75156	5930,312648	14630,86102	10127,27667	5376,879873	0,161679535	False	0,161679535
ENSMUSG00000034300	Fam53c	622,6112482	603,1151973	634,7873945	524,2698376	696,4317047	0,161650241	False	0,161650241
ENSMUSG00000047417	Rexo1	1141,18088	1888,096302	1020,272473	1628,941187	3208,094258	-0,161572992	False	0,161572992
ENSMUSG00000034055	Phka1	449,7547043	1760,366491	502,997624	916,068924	1102,189188	0,161413214	False	0,161413214
ENSMUSG00000026280	Atg4b	868,3788461	811,5164677	776,4613978	791,4566071	1225,577429	-0,16141038	False	0,16141038
ENSMUSG00000024678	Ms4a4d	765,9756803	1844,879449	856,6335082	2018,495006	1767,773833	0,161379524	False	0,161379524
ENSMUSG00000026520	Pycr2	442,3816763	391,8328033	370,1096055	272,7999369	494,7393882	0,161377133	False	0,161377133
ENSMUSG00000024841	Eif1ad	427,6356205	674,1829116	492,0151432	478,2418647	1136,595525	0,161358398	False	0,161358398
ENSMUSG00000062300	Nectin2	1471,328686	1326,277209	1315,701209	1371,858119	2461,832687	-0,161287677	False	0,161287677
ENSMUSG00000017764	Zswim1	303,9325961	271,7859886	204,2741443	230,1398645	252,7086083	-0,161280327	False	0,161280327
ENSMUSG00000031816	Mthfsd	513,6542797	221,8465136	574,3837497	285,1489053	83,04977739	0,161217268	False	0,161217268
ENSMUSG00000020454	Eif4enif1	1338,614184	2288,572476	1197,090415	2109,428319	3730,12143	-0,161208084	False	0,161208084
ENSMUSG00000031378	Abcd1	1688,423398	1020,878113	1887,888462	1451,565096	696,4317047	0,161096805	False	0,161096805
ENSMUSG00000041696	Rasl12	938,8322242	1710,427016	1351,943396	1049,662309	1351,338521	0,160986001	False	0,160986001
ENSMUSG00000003051	Elf3	434,1894231	1019,917738	485,4256546	533,2509055	2150,989234	0,160925775	False	0,160925775
ENSMUSG00000059456	Ptk2b	1013,381729	590,6303285	818,1948251	561,3167426	906,4289989	-0,1609118	False	0,1609118
ENSMUSG00000040212	Emp3	1351,721789	1837,196453	2211,871648	1511,064671	2368,105081	0,16076716	False	0,16076716
ENSMUSG00000042705	Commd10	440,7432257	225,6880117	394,2710634	234,6303984	220,6751228	-0,160750564	False	0,160750564
ENSMUSG00000047412	Zbtb44	506,2812518	738,5280043	631,4926503	818,3998107	565,9249116	0,160671585	False	0,160671585
ENSMUSG00000091337	Eid1	1226,380314	467,7023902	1097,149839	720,7306975	384,4018268	-0,160645874	False	0,160645874
ENSMUSG00000023169	Slc38a1	657,0187119	556,0568459	385,4850787	450,1760276	734,3973172	0,160626329	False	0,160626329
ENSMUSG00000003948	Mmd	929,8207456	463,8608922	688,6015508	831,8714126	532,7050007	-0,160592075	False	0,160592075
ENSMUSG00000041629	Fam104a	699,6184289	1301,307472	781,9526382	934,0310597	2619,627264	0,160512936	False	0,160512936
ENSMUSG00000040322	Slc25a24	731,5682166	618,4811895	654,5558601	593,8731137	561,1792101	-0,16047608	False	0,16047608
ENSMUSG00000070426	Rnf121	354,7245664	261,2218689	317,3936973	253,7151677	134,0660692	-0,160425768	False	0,160425768
ENSMUSG00000006050	Sra1	408,7934379	712,5978923	456,8712044	636,5331861	823,3792215	0,160415477	False	0,160415477
ENSMUSG00000030795	Fus	2727,201112	3665,749535	2440,30725	3237,674971	5293,830096	-0,160358289	False	0,160358289
ENSMUSG00000032375	Aph1b	335,0631586	223,7672627	299,8217279	193,0929595	173,2181071	-0,160328127	False	0,160328127
ENSMUSG00000020841	Cpd	3557,895593	2302,017719	3183,821205	2699,933532	2282,682453	-0,160264855	False	0,160264855
ENSMUSG00000031575	Ash2l	571,0000526	408,1591701	638,0821388	527,6377381	334,5719603	0,160251272	False	0,160251272
ENSMUSG00000020817	Rabep1	635,7188534	711,6375178	568,8925093	739,8154668	640,6697113	-0,16023279	False	0,16023279
ENSMUSG00000031791	Tmem38a	315,4017507	239,133255	282,2497585	251,4699007	137,6253454	-0,160218322	False	0,160218322
ENSMUSG00000024608	Rps14	5648,558626	10581,40644	6311,631758	8386,072135	14299,98524	0,160130262	False	0,160130262
ENSMUSG00000022637	Cblb	874,9326487	1622,072561	783,0508863	1551,479477	2778,608266	-0,160065901	False	0,160065901
ENSMUSG00000022421	Nptxr	288,3673149	309,2405948	258,0883005	337,9126791	463,892328	-0,160042907	False	0,160042907
ENSMUSG00000020415	Pttg1	833,9713824	962,295267	1053,219916	931,7857928	968,1231193	0,160000455	False	0,160000455
ENSMUSG00000006281	Tep1	554,6155461	235,2917569	496,4081355	298,6205071	310,8434525	-0,159961297	False	0,159961297
ENSMUSG00000001419	Mef2d	2586,294356	3805,964215	2889,490718	2918,847062	5360,269918	0,159928752	False	0,159928752
ENSMUSG00000001687	Ubl3	2381,488024	2904,172542	2131,699538	2323,851314	3224,704213	-0,159859189	False	0,159859189
ENSMUSG00000007739	Cct4	1327,145029	1187,983279	1134,490274	1110,284517	1048,800046	-0,159811506	False	0,159811506
ENSMUSG00000024423	Impact	675,8608944	605,0359463	780,8543902	855,4467158	664,3982191	-0,159705484	False	0,159705484
ENSMUSG00000019802	Sec63	1965,321558	2287,612102	1759,393436	2017,372373	2315,902364	-0,159687245	False	0,159687245
ENSMUSG00000030861	Acadsb	1200,984329	1110,192943	1220,153625	1341,547015	484,0615596	0,159680289	False	0,159680289
ENSMUSG00000025470	Zfp511	307,2094975	313,0820928	445,8887235	275,0452039	309,6570271	-0,159554072	False	0,159554072
ENSMUSG00000042211	Fbxo38	685,6915983	943,0877766	613,9206809	852,0788153	1019,139411	-0,159507575	False	0,159507575
ENSMUSG00000029635	Cdk8	643,0918813	1181,260657	718,2542492	1352,77335	1293,203676	0,159469746	False	0,159469746
ENSMUSG00000024926	Kat5	873,2941981	1086,18358	781,9526382	835,239313	1619,470659	-0,159386527	False	0,159386527
ENSMUSG00000016940	Kctd2	622,6112482	695,311151	724,8437377	820,6450777	771,1765043	0,159327086	False	0,159327086
ENSMUSG00000023411	Nfatc4	553,7963207	645,3716761	731,4332262	529,883005	495,9258135	-0,159231148	False	0,159231148
ENSMUSG00000057342	Sphk2	1426,271293	1005,51212	1277,262526	1023,841739	1064,223576	-0,159193342	False	0,159193342
ENSMUSG00000080316	Spaca6	571,0000526	1058,332719	928,0196339	1269,698472	511,3493436	-0,15918163	False	0,15918163
ENSMUSG00000054611	Kdm2a	2218,462184	2348,115696	1986,73079	2237,408536	3343,346753	-0,159163566	False	0,159163566
ENSMUSG00000015889	Lta4h	960,1320827	764,4581163	858,8300044	859,9372497	740,3294441	-0,159001498	False	0,159001498
ENSMUSG00000031548	Sfrp1	293,2826669	170,9466642	163,638965	299,7431406	327,453408	0,158997836	False	0,158997836
ENSMUSG00000002342	Tmem161a	648,0072333	702,0337726	753,398188	580,4015119	660,8389429	-0,15895864	False	0,15895864
ENSMUSG00000002799	Jag2	793,8293414	429,2874095	886,2862065	572,5430775	558,8063593	0,158943772	False	0,158943772
ENSMUSG00000057176	Ccdc189	345,7130878	583,9077069	364,618365	643,268987	309,6570271	-0,158904035	False	0,158904035
ENSMUSG00000032217	Rnf111	710,2683581	865,2974407	792,9351191	898,1067882	951,5131638	0,158838608	False	0,158838608
ENSMUSG00000022614	Lmf2	1650,739033	1302,267846	1842,860291	1380,839187	1273,034445	0,158834642	False	0,158834642
ENSMUSG00000042190	Cmklr1	1582,743331	905,6331704	1306,915224	510,7982358	1417,778343	-0,158795319	False	0,158795319
ENSMUSG00000027968	Larp7	338,3400599	357,2593207	398,6640557	303,111041	431,8588424	-0,1586276	False	0,1586276
ENSMUSG00000046949	Nqo2	508,7389278	169,0259152	455,7729563	257,0830681	124,5746661	-0,158610167	False	0,158610167
ENSMUSG00000049288	Lix1l	824,9599038	1236,962379	739,1209628	803,8055754	1186,425391	-0,158513509	False	0,158513509
ENSMUSG00000040746	Rnf167	2500,275697	3085,683326	2790,64839	3056,93098	3811,984782	0,158513178	False	0,158513178
ENSMUSG00000024782	Ak3	3394,050528	1802,62297	3041,048954	2625,839722	963,3774177	-0,158439002	False	0,158439002
ENSMUSG00000025733	Rhot2	243,309922	218,0050156	385,4850787	293,0073396	220,6751228	-0,158433826	False	0,158433826
ENSMUSG00000047284	Neurl4	979,7934905	920,0387882	1154,25874	877,8993855	988,2923509	-0,158422102	False	0,158422102
ENSMUSG00000025217	Btrc	469,4161121	289,0727299	420,6290175	322,1958103	158,9810024	-0,158318982	False	0,158318982
ENSMUSG00000031921	Terf2	754,5065257	676,1036606	853,3387639	836,3619465	816,2606692	-0,15828892	False	0,15828892
ENSMUSG00000036138	Acaa1a	1300,110593	924,8406608	1450,785723	1143,963521	1264,729467	0,158200103	False	0,158200103
ENSMUSG00000058793	Cds2	3640,637351	4062,384211	2903,767943	3566,606583	3766,900617	0,158135654	False	0,158135654
ENSMUSG00000032786	Alas1	6269,531424	6994,407614	6261,112346	6303,58702	4770,616498	0,157844253	False	0,157844253
ENSMUSG00000048706	Lurap1l	702,8953302	441,7722782	784,1491344	615,2031499	633,5511589	0,157818191	False	0,157818191
ENSMUSG00000026107	Nabp1	346,5323131	114,2845676	386,5833268	235,7530319	16,60995548	0,15778953	False	0,15778953
ENSMUSG00000068227	Il2rb	457,9469575	257,3803708	208,6671366	158,2913214	410,5031854	-0,157787089	False	0,157787089
ENSMUSG00000038608	Dock10	888,040254	740,4487533	855,5352601	654,4953219	990,6652017	0,157772503	False	0,157772503
ENSMUSG00000021302	Ggps1	757,783427	689,5489039	665,538341	845,3430144	669,1439207	0,157751274	False	0,157751274
ENSMUSG00000073409	H2-Q6	303,1133708	262,1822434	216,3548732	188,6024255	338,1312365	0,157725805	False	0,157725805
ENSMUSG00000002699	Lcp2	435,0086484	441,7722782	358,0288765	312,0921089	485,247985	0,157678139	False	0,157678139

ENSMUSG00000031540	Kat6a	2129,166624	1216,794514	1908,755176	1457,178264	1399,981962	-0,157656787	False	0,157656787
ENSMUSG00000040669	Phc1	557,073222	621,3623131	439,299235	489,4681996	588,4669941	0,157567773	False	0,157567773
ENSMUSG00000054072	Iigp1	723,3759633	445,6137763	382,1903344	383,940652	806,7692661	0,157410465	False	0,157410465
ENSMUSG00000029310	Nudt9	580,0115312	801,9127225	646,8681235	870,0409511	707,1095332	0,157390038	False	0,157390038
ENSMUSG00000070738	Dgkd	1221,464962	1027,600734	1148,767499	1152,944589	1362,016349	0,157131541	False	0,157131541
ENSMUSG00000024660	Incenp	237,5753447	241,054004	213,060129	216,6682627	459,1466264	-0,157124483	False	0,157124483
ENSMUSG00000028964	Park7	692,2454009	719,3205139	685,3068065	620,8163173	764,057952	-0,157117086	False	0,157117086
ENSMUSG00000039191	Rbpj	748,7719485	648,2527996	630,3944022	643,268987	671,5167715	-0,157102956	False	0,157102956
ENSMUSG00000026491	Ahctf1	681,5954717	1123,638186	759,9876765	880,1446524	1723,876093	0,157060274	False	0,157060274
ENSMUSG00000028274	Rngtt	258,0559779	231,4502588	229,5338503	163,9044888	191,014488	-0,156981875	False	0,156981875
ENSMUSG00000026987	Baz2b	1217,368835	1367,573314	1379,399598	1880,411088	1357,270648	0,156942093	False	0,156942093
ENSMUSG00000030870	Ubfd1	526,761885	483,0683825	584,2679825	373,8369506	587,2805687	0,156898944	False	0,156898944
ENSMUSG00000022992	Kansl2	321,136328	366,8630659	358,0288765	403,0254212	462,7059026	0,156890075	False	0,156890075
ENSMUSG00000020900	Myh10	2311,034646	577,1850853	2576,490013	876,776752	510,1629182	0,156868112	False	0,156868112
ENSMUSG00000032637	Atxn2l	5049,704913	5650,843664	5629,619696	5880,354196	7975,15148	0,156838381	False	0,156838381
ENSMUSG00000018012	Rac3	404,6973113	363,0215678	388,7798229	432,2138918	696,4317047	-0,156788	False	0,156788
ENSMUSG00000024855	Pacs1	1554,070444	1163,013541	1336,567922	1196,727295	1394,049835	-0,156769765	False	0,156769765
ENSMUSG00000053819	Camk2d	886,4018033	933,4840314	795,1316153	899,2294217	1464,048933	-0,156767132	False	0,156767132
ENSMUSG00000033769	Exoc6b	919,1708164	625,2038112	668,8330852	602,8541816	824,5656469	-0,156698636	False	0,156698636
ENSMUSG00000029622	Arpc1b	3253,962997	5628,75505	2919,143416	4182,932366	6499,238293	-0,156652754	False	0,156652754
ENSMUSG00000028005	Gucy1b1	1694,157975	1476,095634	1519,975353	1330,32068	855,4127071	-0,156520478	False	0,156520478
ENSMUSG00000014748	Tex261	2214,366058	1634,55743	1986,73079	1468,404599	1588,623599	-0,156497339	False	0,156497339
ENSMUSG00000031755	Bbs2	368,6513969	403,3572975	361,3236208	410,8838556	295,4199224	0,156473422	False	0,156473422
ENSMUSG00000040410	Fbxl4	293,2826669	263,1426179	213,060129	259,3283351	177,9638087	-0,156446887	False	0,156446887
ENSMUSG00000074748	Atxn7l3b	2218,462184	1936,115028	2241,524346	1778,251441	2472,510515	0,156416695	False	0,156416695
ENSMUSG00000028651	Ppie	271,9828084	351,4970736	358,0288765	303,111041	501,8579405	0,15633094	False	0,15633094
ENSMUSG00000021497	Txndc15	706,1722315	533,0078575	758,8894284	786,9660732	385,5882522	0,156281349	False	0,156281349
ENSMUSG00000022965	Ifngr2	2432,279994	2084,012704	2710,47628	2090,34355	2219,801907	0,156237066	False	0,156237066
ENSMUSG00000061950	Ppp4r1	1006,008701	1655,685669	902,7599279	1374,103386	1852,010036	-0,156228497	False	0,156228497
ENSMUSG00000025323	Sp4	299,8364695	338,0518303	269,0707814	369,3464166	399,8253569	-0,156190135	False	0,156190135
ENSMUSG00000078350	Smim1	582,4692072	818,2390893	649,0646197	1210,198897	672,7031968	0,156180335	False	0,156180335
ENSMUSG00000053897	Slc39a8	3392,412077	6348,075564	3044,343698	7591,247627	9214,966014	-0,156180181	False	0,156180181
ENSMUSG00000056214	Pard6g	541,5079408	485,9495061	701,7805279	550,0904078	523,2135975	-0,15617608	False	0,15617608
ENSMUSG00000002031	Ift46	996,1779971	1479,937132	893,9739432	1397,678689	1948,110492	-0,156170765	False	0,156170765
ENSMUSG00000044827	Tlr1	320,3171027	118,1260657	356,9306284	178,4987242	56,94841878	0,156142875	False	0,156142875
ENSMUSG00000004317	Clcn5	198,252529	331,3292087	177,9161902	239,1209324	298,9791986	-0,15614147	False	0,15614147
ENSMUSG00000030739	Myh14	5999,187066	5383,859548	7068,324691	10493,25519	5836,0265	-0,156126244	False	0,156126244
ENSMUSG00000007817	Zmiz1	3947,846849	5558,64771	3542,94833	4130,168592	8393,959643	-0,156115598	False	0,156115598
ENSMUSG00000059142	Zfp945	366,193721	512,8399926	462,3624448	733,0796659	328,6398334	-0,156099698	False	0,156099698
ENSMUSG00000035697	Arhgap45	2454,399078	3509,208489	2734,637738	2925,582863	3809,611931	0,155979883	False	0,155979883
ENSMUSG00000046731	Kctd11	567,7231513	298,6764751	509,5871126	321,0731768	123,3882407	-0,155858782	False	0,155858782
ENSMUSG00000028977	Casz1	2448,664501	1813,18709	2368,921125	2727,999369	2694,372064	0,155848191	False	0,155848191
ENSMUSG00000068923	Syt11	407,1549873	290,0331044	453,5764601	391,7990864	290,6742209	0,155767694	False	0,155767694
ENSMUSG00000029063	Nadk	2510,925626	2253,998994	1984,534294	2249,757504	1872,179267	-0,155732425	False	0,155732425
ENSMUSG00000075000	Nrbf2	169,5796426	339,9725793	188,898671	243,6114663	608,6362257	0,155649562	False	0,155649562
ENSMUSG00000033365	Ipo13	1004,37025	1139,964553	1126,802538	1073,237612	901,6832974	-0,155598499	False	0,155598499
ENSMUSG00000049624	Slc17a5	362,0975943	430,247784	325,0814339	574,7883444	397,4525061	-0,155577429	False	0,155577429
ENSMUSG00000021669	Col4a3bp	1167,39609	1565,410464	1482,634918	1966,853866	1300,322229	0,155575036	False	0,155575036
ENSMUSG00000054874	Pcnx3	1319,772001	1228,319008	1248,708075	1236,019467	1469,98106	0,15550885	False	0,15550885
ENSMUSG00000026608	Kctd3	382,5782275	650,1735486	426,1202579	486,1002991	779,4814821	0,155505867	False	0,155505867
ENSMUSG00000066129	Kndc1	691,4261756	518,6022397	520,5695934	620,8163173	465,0787534	-0,155408744	False	0,155408744
ENSMUSG00000074916	Chst14	372,7475236	234,3313824	415,137777	253,7151677	307,2841763	0,155391454	False	0,155391454
ENSMUSG00000031979	Cog2	347,3515385	713,5582668	311,9024568	469,2607968	1086,765658	-0,15530157	False	0,15530157
ENSMUSG00000018900	Slc22a5	411,2511139	720,2808884	457,9694525	894,7388877	665,5846445	0,155231784	False	0,155231784
ENSMUSG00000021767	Kat6b	556,2539967	619,4415641	463,4606929	554,5809417	507,7900675	0,155224393	False	0,155224393
ENSMUSG00000030095	Tmem43	1790,007338	2557,477341	1993,320279	2490,00107	2387,087887	0,155208033	False	0,155208033
ENSMUSG00000026126	Ptpn18	616,8766709	785,5863557	781,9526382	783,5981727	686,9403015	0,15520264	False	0,15520264
ENSMUSG00000000346	Dazap2	6928,188587	12247,65623	6221,575415	8597,12723	17758,41526	-0,155198258	False	0,155198258
ENSMUSG00000033478	Fam160b1	1148,553908	1031,442232	1165,241221	1221,425232	1303,881505	-0,155155549	False	0,155155549
ENSMUSG00000028132	Tmem56	253,1406259	203,5993978	227,3373541	200,9513939	208,8108689	-0,155104306	False	0,155104306
ENSMUSG00000020755	Sap30bp	590,6614604	873,9408113	530,4538262	609,5899825	1397,609111	-0,155104306	False	0,155104306
ENSMUSG00000020078	Vps26a	1092,846586	1487,620128	1216,858881	1527,904173	1452,184679	0,155070979	False	0,155070979
ENSMUSG00000024354	Slc23a1	340,7977358	259,3011198	274,5620219	379,450118	154,2353009	0,154994493	False	0,154994493
ENSMUSG00000021662	Arhgef28	1080,558206	682,8262823	591,9557191	717,3627971	970,49597	-0,15498266	False	0,15498266
ENSMUSG00000024663	Rab3il1	241,6714713	105,641197	269,0707814	129,1028508	227,7936751	0,154938559	False	0,154938559
ENSMUSG00000037287	Tbcel	1231,295666	1764,207989	1105,935824	1980,325468	2070,312308	-0,154909561	False	0,154909561
ENSMUSG00000022964	Tmem50b	3361,281515	2062,884465	3019,083992	2331,709749	1955,229045	-0,154900483	False	0,154900483
ENSMUSG00000029265	Dr1	611,1420936	633,8471818	591,9557191	548,9677743	872,0226626	-0,154786391	False	0,154786391
ENSMUSG00000021417	Eci2	563,6270247	1523,153985	506,2923683	1509,942038	1672,859802	-0,15477005	False	0,15477005
ENSMUSG00000033033	Calhm2	402,2396353	63,38471818	361,3236208	108,8954481	30,84706017	-0,154763676	False	0,154763676
ENSMUSG00000040118	Cacna2d1	479,246816	452,3363979	430,5132503	436,7044258	690,4995777	-0,154711206	False	0,154711206
ENSMUSG00000028630	Dyrk2	1498,363122	987,2650044	1149,865748	1346,037549	1263,543042	-0,154668642	False	0,154668642
ENSMUSG00000034724	Cnot6l	1104,31574	910,435043	945,5916033	1229,283666	752,1936981	0,154665148	False	0,154665148
ENSMUSG00000029128	Rab28	666,0301905	720,2808884	729,23673	598,3636476	675,0760476	-0,154565045	False	0,154565045
ENSMUSG00000002105	Slc39a13	1078,919755	1191,824777	1469,455941	1538,007875	969,3095447	-0,154558205	False	0,154558205
ENSMUSG00000067713	Prkag1	1240,307144	1792,05885	1380,497846	1585,158481	2429,799201	0,154491209	False	0,154491209
ENSMUSG00000024290	Rock1	1442,6558	1748,841997	1605,638704	2168,927893	2096,413666	0,154420167	False	0,154420167
ENSMUSG00000030802	Bckdk	1137,903979	647,2924251	1022,468969	838,6072135	333,3855349	-0,154321762	False	0,154321762
ENSMUSG00000031819	Emc8	523,4849837	660,7376683	589,7592229	470,3834303	784,2271836	-0,154310913	False	0,154310913
ENSMUSG00000004880	Lbr	897,0517326	1299,386723	998,3075115	1113,652417	1351,338521	0,154293094	False	0,154293094
ENSMUSG00000003228	Grk5	1495,086221	1099,628823	1663,845852	1221,425232	975,2416716	0,154293094	False	0,154293094
ENSMUSG00000053641	Dennd4a	827,4175798	1780,534356	743,5139552	945,2573946	2580,475226	-0,154255789	False	0,154255789
ENSMUSG00000071659	Hnrnpul2	1851,449238	1404,067545	1627,603666	1663,742825	1354,897797	-0,15422255	False	0,15422255
ENSMUSG00000025907	Rb1cc1	1041,23539	1359,890317	935,7073705	1268,575838	1448,625403	-0,15416693	False	0,15416693
ENSMUSG00000003438	Timm50	326,05168	423,5251624	318,4919454	293,0073396	411,6896108	-0,154163849	False	0,154163849
ENSMUSG00000024079	Eif2ak2	444,8393523	290,0331044	399,7623038	371,5916836	368,9782967	-0,154141983	False	0,154141983
ENSMUSG00000070565	Rasal2	740,5796952	302,5179731	665,538341	647,759521	270,5049892	-0,154133214	False	0,154133214
ENSMUSG00000013584	Aldh1a2	528,4003356	42,25647879	246,0075716	474,8739643	224,2343989	-0,154086723	False	0,154086723
ENSMUSG00000006611	Hfe	805,298496	417,7629153	723,7454896	505,1850684	204,0651673	-0,154041187	False	0,154041187
ENSMUSG00000027778	Ift80	579,1923059	226,6483862	520,5695934	370,4690501	207,6244435	-0,153951391	False	0,153951391
ENSMUSG00000040865	Ino80d	794,6485667	931,5632824	884,0897104	901,4746887	1383,372006	0,153875801	False	0,153875801
ENSMUSG00000030431	Tmem238	304,7518215	463,8608922	373,4043497	273,9225704	469,8244549	-0,153866699	False	0,153866699
ENSMUSG00000038811	Gngt2	674,2224437	574,3039617	750,1034437	636,5331861	567,111337	0,153864912	False	0,153864912
ENSMUSG00000000838	Fmr1	592,2999111	661,6980429	658,9488525	674,7027246	665,5846445	0,153838618	False	0,153838618
ENSMUSG00000037669	Ldah	548,8809688	432,1685331	610,6259366	400,7801542	237,2850783	0,153795552	False	0,153795552
ENSMUSG00000022477	Aco2	3226,928561	3500,565118	2908,160935	2900,884926	4277,063535	-0,153668572	False	0,153668572
ENSMUSG00000068823	Csde1	4767,8914	3891,437547	3782,366413	4286,214647	3827,408312	-0,153647276	False	0,153647276
ENSMUSG00000025921	Rdh10	385,0359035	264,1029924	343,7516514	258,2057016	428,2995662	0,153627237	False	0,153627237
ENSMUSG00000006932	Ctnnb1	9787,284976	8798,951333	6068,918931	5149,519797	6953,639218	-0,153577115	False	0,153577115
ENSMUSG00000042675	Ypel3	2277,446408	2047,518472	3570,404532	3382,494691	2209,124079	-0,153540638	False	0,153540638
ENSMUSG00000022983	Scaf4	918,351591	1085,223205	1021,370721	1050,784942	1744,045325	0,153388108	False	0,153388108
ENSMUSG00000036550	Cnot1	2100,493737	1656,646043	1340,960915	1459,423531	2336,071595	0,153356006	False	0,153356006
ENSMUSG00000024732	Ccdc86	182,6872478	354,3781971	203,1758962	246,9793668	848,2941547	0,153353325	False	0,153353325
ENSMUSG00000003344	Btbd2	1142,000105	532,0474829	1026,861962	802,6829419	335,7583857	-0,153320526	False	0,153320526
ENSMUSG00000041992	Rapgef5	2397,872531	4558,897837	2666,546356	4263,761977	3540,293368	0,15321744	False	0,15321744
ENSMUSG00000073725	Lmbrd1	1411,525238	1531,797356	1022,468969	1333,68858	1569,640793	0,153179517	False	0,153179517
ENSMUSG00000010936	Vac14	726,6528646	1008,393244	653,457612	678,0706251	1251,678788	-0,153172671	False	0,153172671
ENSMUSG00000025533	Asl	283,451963	432,1685331	315,1972011	518,6566702	319,1484302	0,153150464	False	0,153150464
ENSMUSG00000032231	Anxa2	6081,928824	5940,876768	6703,706326	5469,47034	7438,887203	-0,153127803	False	0,153127803
ENSMUSG00000026816	Gtf3c5	489,8967453	473,4646373	544,7310514	417,6196565	428,2995662	0,153066401	False	0,153066401

ENSMUSG00000019927	Ube2d1	835,6098331	514,7607416	929,117882	560,1941091	638,2968605	0,153032181	False	0,153032181
ENSMUSG00000020828	Pld2	849,5366636	915,2369156	945,5916033	1049,662309	764,057952	-0,152994144	False	0,152994144
ENSMUSG00000032536	Trak1	2524,033231	2748,59187	2270,078797	2707,791966	3140,468011	-0,152988529	False	0,152988529
ENSMUSG00000017485	Top2b	1502,459249	1256,169869	1182,81319	1196,727295	1351,338521	-0,152936735	False	0,152936735
ENSMUSG00000025364	Pa2g4	1094,485036	1849,681322	1216,858881	1371,858119	2267,258923	0,152909638	False	0,152909638
ENSMUSG00000040396	Abhd13	503,0043505	644,4113015	452,478212	474,8739643	1134,222674	-0,152722554	False	0,152722554
ENSMUSG00000026817	Ak1	625,0689241	280,4293592	562,3030208	343,5258465	187,4552118	-0,152667482	False	0,152667482
ENSMUSG00000022793	B4galt4	887,2210286	534,9286065	986,2267825	813,9092768	373,7239982	0,152625872	False	0,152625872
ENSMUSG00000047394	Odf3b	1103,496515	1226,398259	1266,280045	1436,970861	1322,864311	0,152345481	False	0,152345481
ENSMUSG00000008035	Mid1ip1	3256,420673	1271,535862	2930,125897	1683,950228	1575,57292	-0,152324429	False	0,152324429
ENSMUSG00000030685	Kctd13	268,7059071	358,2196952	371,2078535	298,6205071	325,0805572	0,152285205	False	0,152285205
ENSMUSG00000040732	Erg	1434,463547	870,0993132	1465,062949	1594,139549	326,2669826	0,15226662	False	0,15226662
ENSMUSG00000004070	Hmox2	713,5452594	1196,626649	792,9351191	982,3042996	1435,574723	0,152197883	False	0,152197883
ENSMUSG00000021156	Zmynd11	3485,803764	3809,805713	3805,429623	4350,204755	3136,908734	-0,152147798	False	0,152147798
[truncated: 572,275 more chars]
